# Transcriptome data-based screening of potential host of genetic transformation for a blue-hued *Bougainvillea* transgene

**DOI:** 10.1590/1678-4685-GMB-2023-0099

**Published:** 2024-03-04

**Authors:** Rong Sun, Shan Liu, Jia Long, Jinglei Gao, Yi Diao

**Affiliations:** 1 Panzhihua University, Faculty of Biological and Chemical Engineering, Department of Biological Engineering, Panzhihua, Sichuan, P.R. China.

**Keywords:** Bougainvillea, transcriptome, blue colour, transgene host, delphinidin

## Abstract

*Bougainvillea* is a popular ornamental plant. Although *Bougainvillea* is abundant in germplasm resources, cultivars and flower colors, there is no rare blue colour varieties, due to the absence of delphinidin-based anthocyanins. This study analyzed the *Bougainvillea* leaf and bract transcriptome to select hosts of genetic transformation that would be suitable for the accumulation of delphinidin. A total of 36 gigabyte (GB) of raw data was obtained by transcriptome sequencing, with 4,058 significantly differentially expressed genes, including 1,854 upregulated and 2,204 downregulated genes. Annotation of these genes was performed using Gene Ontology and Kyoto Encyclopedia of Genes and Genomes databases. Through annotation, two *CHS* genes, one *F3H* gene, one *DFR* gene, and one *F3'H* gene involved in the delphinidin biosynthesis pathway were identified. The expression levels of these genes and total flavonoid content in the bracts of six *Bougainvillea* varieties were examined through quantitative real-time PCR and spectrophotometry, respectively. Through the comprehensive evaluation based on membership function method, the suitable host order for a blue-hued *Bougainvillea* transgene is *Singapore White*>*Elizabeth Angu*s>*Ratana Yellow*>*China Beauty*>*Orange King*>*Brilliant Variegata*. Thus, *Singapore White* variety was the most appropriate transgene host for blue-hued *Bougainvillea.* The results of this study provide a reference for the directed breeding of blue-hued *Bougainvillea*.

## Introduction

*Bougainvillea* is an evergreen vine-like shrub in the *Bougainvillea* genus of the Nyctaginaceae family, Caryophyllales (Centrospermae) order (Xu et al., 2008). *Bougainvillea* is a popular and widely used ornamental plant that has the characteristics of rich flower colours, long flowering periods, high eurytopicity and excellent vitality (Wu and Tang, 2010). At present, there are approximately 500 ornamental varieties of *Bougainvillea* worldwide (Sindhu et al., 2020), including those with monochrome single bract, monochrome double bract, double colored single bract, single colored spotted leaf and so on. Among them, the monochrome single bract is most widely cultivated, including those with red, orange, pink-purple, and white bracts (Zeng et al., 2018). As an ornamental plant, development of novel coloured flowers is an important breeding goal for *Bougainvillea*. So far, blue *Bougainvillea* lines have not been bred by the traditional breeding methods. Introducing heterologous anthocyanins biosynthetic genes into non-blue-flower plants through molecular biology and plant transformation technology may achieve this goal. However, screening for a suitable blue transgene host is a prerequisite for the breeding of blue *Bougainvillea*.

Previous research indicated that the generation of blue flower colour would require a combination of multiple conditions, the accumulation of delphinidin-based anthocyanins, as well as accumulation of flavone co-pigments and an appropriate vacuolar pH (An, 1973; Dai and Hong, 2016; Noda, 2018). Anthocyanins are flavonoids that are synthesized from phenylalanine through catalysis by a series of enzymes. And different types of anthocyanins stably exist in various organs and present different colors. At present, blue flower breeding is mainly carried out under a strategy of establishing delphinidin biosynthesis pathway. In the delphinidin biosynthesis pathway, flavonoid-3′5′-hydroxylase (F3′5′H) is believed to be an essential enzyme for blue flower breeding. For most plants, F3′5′H deficiency is the main cause of the failure to form blue flowers. Therefore, the *F3′5′H* gene that encodes the F3′5′H enzyme is also referred to as the “blue gene” (Meng and Dai, 2004). For example, violet flower carnation varieties have been successfully created by expressing heterologous *F3’5’H* (Mol et al., 1999; Tanaka et al., 1998). Introduction of the *Medicago sativa F3′5′H* gene into the dahlia leads to the production of delphinidin derivatives in dahlia, resulting in purple flowers (Nakano et al., 2016; Noda, 2018). Subsequently, various blue dahlias have been cultivated using genetically modified dahlias as hybrid parents (Noda, 2018). Similarly, expression of the viola *F3’5’H* gene in rose cultivars resulted in the accumulation of a high percentage of delphinidin (up to 95%) and a novel bluish flower color (Katsumoto et al., 2007).

The previously report indicated that betalain is the main pigment in *Bougainvillea* bracts, which can not coexist with anthocyanins in the same plant (Stafford, 1994). The key to determining whether an exogenous *F3′5′H* gene can be introduced into *Bougainvillea* is first knowing whether there is a presynthetic pathway of delphinidin in the plant. With the development of high-throughput sequencing technology, the cost of transcriptome sequencing has decreased and sequencing efficiency has improved. Therefore it is widely used to exploit novel genes in species with less-studied genomes.

In this study, we performed transcriptome sequencing of the bracts and leaves of the *Bougainvillea* cultivar *Singapore White*. Based on the differential gene expression results and gene annotation information, the key enzyme genes of the delphinidin synthesis pathway were discovered. The expression of each gene in the bracts of different colors *Bougainvillea* was determined by Quantitative real-time PCR (RT-qPCR) and the total flavonoids content in these bracts was detected by spectrophotometry. Finally, membership function analysis was used to evaluate the suitability of different varieties as hosts of blue *Bougainvillea* genetic transformation.

## Material and Methods 

### Plant material

Cuttings of different color monochrome single bract *Bougainvillea* cultivars (*Bougainvillea glabra* ‘Singapore White’, *Bougainvillea* **×** *buttiana ‘* China Beauty’, *Bougainvillea* **×** *buttiana* ‘Brilliant Variegata’, *Bougainvillea* **×** *buttiana* ‘Orange King’, *Bougainvillea* **×s** *pectoglabra* ‘Ratana Yellow’ and *Bougainvillea glabra* ‘Elizabeth Angus’) with similar growth statuses were selected and planted in the nursery of Panzhihua University. The leaves and bracts of *Singapore White* at blooming stage were collected for transcriptome sequencing. The bracts of above cultivars at blooming stage were collected for gene expression analysis and flavonoids content measurements.

### Transcriptome sequencing

After the samples were collected, they were quickly frozen in liquid nitrogen and sent to Tiangen Biotech (Beijing) Co., Ltd. on dry ice for transcriptome sequencing and database construction. After extracting of total RNA, first the RNA concentration was determined by a Qubit® 2.0 Flurometer (Life Technologies, CA, USA), and the integrity of the RNA was analyzed using an Agilent 2100 RNA Nano 6000 Assay Kit (Agilent Technologies, CA, USA). Then, random hexamers were used to synthesize first-strand cDNA, and double-stranded cDNA were purified by AMPure XP Beads (Beckman Coulter, USA). Finally, an Illumina HiSeq X Ten high-throughput sequencing platform was used for library sequencing. The sequencing data were filtered for quality using trim_galore (Bolger et al., 2014) to retain bases with a quality value greater than 20. The filtered data were then screened for length to remove reads with a length less than 50 bp or only one end. Each of the samples was represented by twice replicate containers.

### Gene expression quantification

The fragments per kilobase of transcript sequence per million mapped reads (FPKM) values were calculated to assess gene expression levels. The differentially expressed genes (DEGs) were assessed by DESeq2 software (Anders and Huber, 2010). The false discovery rate (FDR) was used to determine the threshold *P*-value in multiple tests. In two samples, the genes with a FDR of < 0.05 were defined as DEGs. The differentially expressed genes were then subjected to Gene Ontology (GO) and Kyoto Encyclopedia of Genes and Genomes (KEGG) enrichment analyzes. Genes related to anthocyanin biosynthesis pathway were screened based on the gene annotation information.

### RT-qPCR validation

Total RNA from the bracts of six cultivars was extracted according to the instructions from the RNAprep pure Plant Kit (Tiangen, Beijing). The same amount of RNAs from samples was used for reverse transcription into the single stranded cDNA according to the PrimeScript RT Reagent Kit with gDNA Eraser (TaKaRa). F1-ATPase alpha subunit (ATP1) was used as an internal control according to previous report (Bai, 2014). The specific primers were designed according to the sequences obtained by transcriptome screening (Table 1). A 25 μL reaction system with TB Green^®^ Premix Ex *Taq*™ (Tli RNase H Plus,TaKaRa) was used for quantification on a CFX96 Real-Time PCR Instrument (Bio-Rad). The reaction system consisted of 12.5 μL of TB Green, 5 pmol of each upstream and downstream primer, 120 ng of template, and sufficient RNase-Free ddH_2_O to increase the volume to 25 μL. The PCR procedure was as follows: 95 °C for 60 s followed by 40 cycles at 95 °C for 10 s, 60 °C for 30 s, and 72 °C for 30 s. The 2^−ΔΔCt^ method (Livak and Schmittgen, 2001) was used to calculate differences among gene expression. Each experiment was performed on three biological replicates.

**Table 1 -:** Quantitative RT-PCR primers used in this study.

Primer	Sequence (5’→3’)
ATP1F	GTAGCGATTGGACAGAAACG
ATP1R	GAAAGATGGTGAACTATGCCTG
CHS1qF	GTTCCCAGATTTCTACTTCCGTGT
CHS1qR	GCCGCTTTCTAATGTTGGTTCT
CHS2qF	GCGGAGAACAACAAGGGAGC
CHS2qR	TCGTTCAATGGACAAGTCAGGAT
F3HqF	ACGTCCGAAAGTGGGTTACAAT
F3HqR	TGCTTCCACCATTTCCCGTC
DFRqF	AAGGCTCTGATGTGATGTGGTATG
DFRqR	ACTATCGTTGAGGGTTGGTTGC
F3’HqF	TGACTGGGAGTTGGCTGATGG
F3’HqR	TGGAAGCCTGAGTCGTGGGT

### Measurement of total flavonoid contents

The standard curve of total flavonoid content was drawn using rutin as the standard. A total of 0.5 g of freeze-dried *Bougainvillea* powder (filtered through a 100-mesh sieve) was ultrasonically extracted with 25 mL of 60% ethanol for 40 min, soaked for 24 h, and centrifuged. Two millilitres of each extract was diluted with 60% ethanol to 25 mL. Then, 2 mL of the diluted solution was transferred into a 25-mL volumetric flask, mixed evenly with 1.0 mL of 5% NaNO_2_ and 1.0 mL of 10% Al(NO)_3_, and allowed to stand for 5 min. The solution was then mixed with 5 mL of 4% NaOH solution, and filled with 60% ethanol to volume, followed by incubation at room temperature for 5 min. The absorbance at 510 nm was measured by a UV-visible spectrometer, with blank reagent as a reference. Three measurements were taken in parallel, and the flavonoid content was calculated according to the following formula:

$$W=\frac{C\times V_{1}\times V_{2}}{M\times V_{3}\times 1000}\times 100\%$$

(1) 

C is the instrument detection concentration calculated by the standard curve (mg/mL); V_1_ is the volume of the extract (mL); V_2_ is the constant volume (mL) during the measurement; V_3_ is the aspirated measurement volume (mL); and M is the sample weight (g).

### Data analysis

The suitability of different varieties as hosts of blue *Bougainvillea* genetic transformation is evaluate by membership function method. The calculation formula is as follows: 

Membership function value:

$$U(X_{ij})=\left(X_{ij}-X_{jmin}\right)/(X_{jmax}-(X_{jmin})$$

(2) 

Anti membership function value:

$$U\left(X_{ij}\right)=1-\left(X_{ij}-X_{jmin}\right)/(X_{jmax}-(X_{jmin})$$

(3) 

U(X_ij_) is the membership function value of index j of category i. X_ij_ is the measured value of index j of category i. X_jmin_ is the minimum value of index j of all categories. X_jmax_ is the maximum value of index j of all categories. i is a variety. j is an index.

## Results 

###  Overview of the Bougainvillea transcriptome 

Approximately 9 GB of high-quality nucleotide sequence data was obtained for each sample with GC content >42% and Base quality score > 20 ratio >97.7% (Table 2). The results showed that the quality of the sequencing met the standards, with sufficient data resources for further data analysis.

**Table 2 -:** Statistics of the *Bougainvillea* transcriptome data.

Sample	Raw Reads	Bases	GC (%)	Q20	Q30	Avg. quality
WL1	58,835,634	8.825 GB	42.85%	97.91%	94.05%	35.99
WL2	59,456,782	8.919 GB	43.15%	97.85%	93.93%	35.965
WF1	56,448,268	8.467 GB	43.06%	97.74%	93.67%	35.925
WF2	66,465,624	9.970 GB	42.95%	97.83%	93.92%	35.97

WL1, biological replicate 1 of the leaf sample; WL2, biological replicate 2 of the leaf sample; WF1, biological replicate 1 of the bract sample; WF2, biological replicate 2 of the bract sample.

### Analysis of differentially expressed genes

**Figure 1 -:**
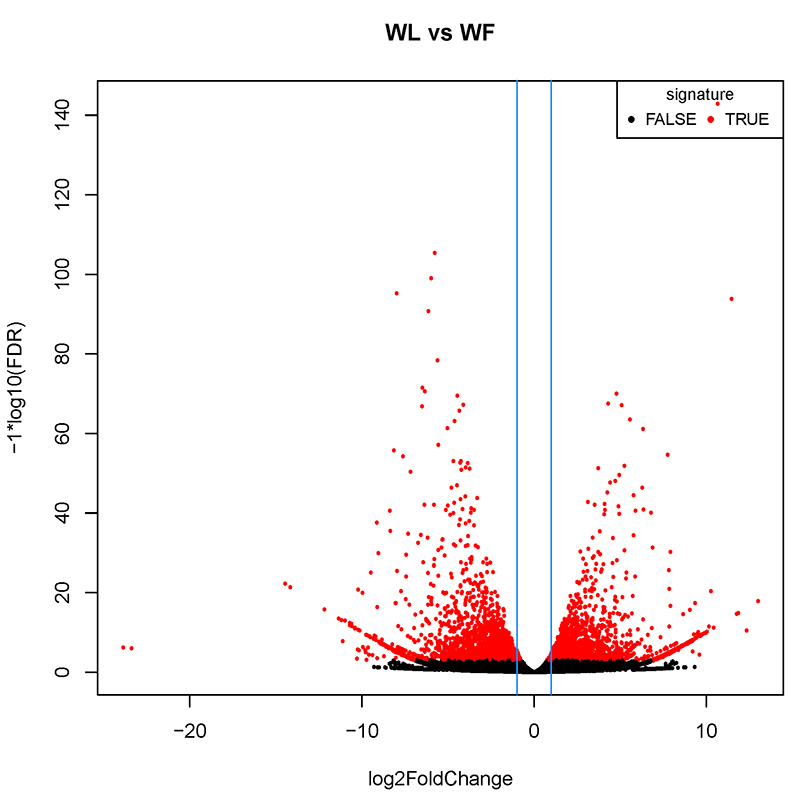
Differentially expressed genes (DEGs) volcano map. The abscissa represents the logarithmic value of the fold difference in the expression of a certain gene in two samples; the ordinate represents the negative logarithm of the statistically significant change in gene expression. The red color represents the upregulated and the black color shows the downregulated genes.

The main ornamental part of *Bougainvillea* is bracts, which are specialized leaves. In order to identify the genes involved in anthocyanin synthesis, the DEGs between leaves and bracts were assessed by DESeq2. A total of 5,063 DEGs were identified in comparisons of leaf *vs* bract. This included 2,363 upregulated and 2,700 downregulated genes in the leaf sample in comparison to the bracts. Furthermore, of these DEGs, 4,058 showed significant differences, including 1,854 upregulated and 2,204 downregulated genes (Figure 1).

### GO and KEGG annotations of DEGs

**Figure 2 -:**
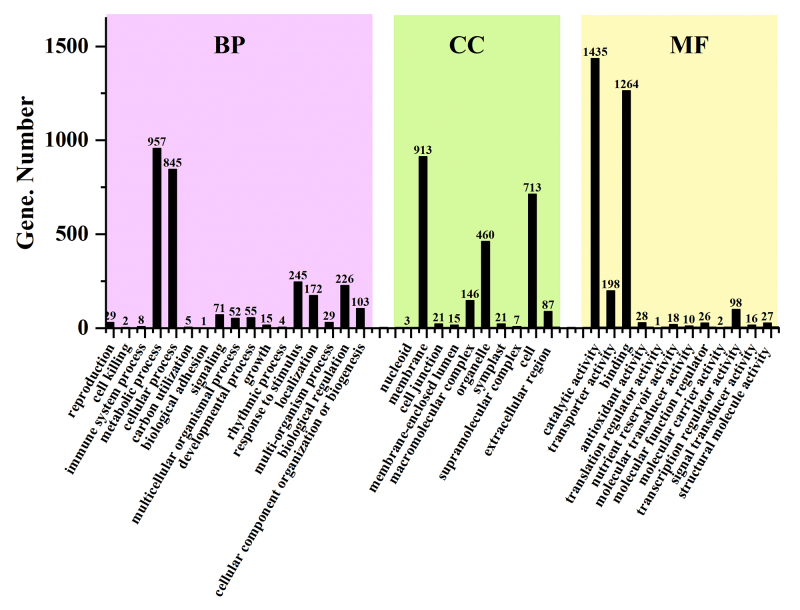
Gene ontology of differential expressed genes. BP: biological process, CC: cellular component, MF: molecular function.

In order to obtain comprehensive gene function information, we used the GO, KEGG, Transcription factor, Nucleotide Sequence Database, Non-Redundant Protein Sequence Database and Universal Protein databases to annotate the function of the DEGs (Table S1). A total of 4,058 genes had annotation information, among which 2,638 genes could be assigned the GO terms. The genes were categorized into three subcategories: biological process (17 GO terms), cellular component (10 GO terms) and molecular function (12 GO terms) (Figure 2). A total of 2,819 genes were categorized in the biological process category, 2,386 genes in the cellular component category, and 3,123 genes in the molecular function category. In the biological process category, the “metabolic process” subcategory had the maximum number of genes, and the “biological adhesion” subcategory had the fewest genes. In the cellular component category, the subcategory “membrane” was the most-enriched component and the “nucleoid” was the fewest. In the molecular function category, the “catalytic activity” subcategory had the maximum number of genes, the presence of 1,435 genes in this subcategory suggests the possibility of their participation in catalysis. These results indicated that a large number of DEGs are involved in the metabolic process and have catalytic activities, which was conducive to our subsequent screening of functional genes.

**Figure 3 -:**
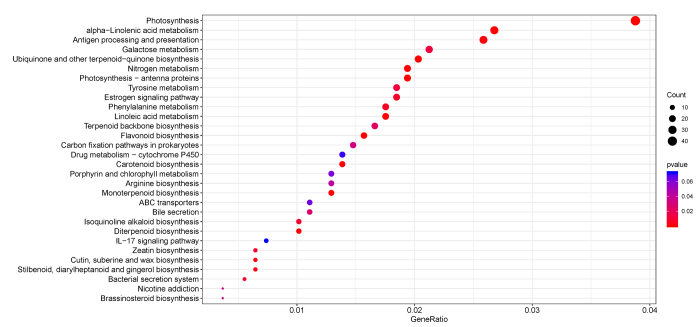
Number of DEGs belonging to the top 30 pathways. GeneRatio: the ratio of the number of DEGs enriched in the pathway to the total number of DEGs.

KEGG is a database for biological systems that integrates genomic, chemical and systemic functional information (Ogata et al., 1999). In this study, 1,083 DEGs were annotated using the KEGG database, involved in 231 metabolic pathways (Table S2). The metabolic pathway with the most annotated genes was ko00195, which is related to photosynthesis, consistent with the functional differences between the bracts and leaves. The bracts mainly synthesize pigment to display different colors, while the leaves carry out photosynthesis with chlorophyll. Moreover, 19 and 17 DEGs were mapped into ko00360 (Phenylalanine metabolism) and ko00941 (Flavonoid biosynthesis) respectively, which we were very interested in for their potential roles in anthocyanin synthesis (Figure 3).

### Identification of genes involved in anthocyanin biosynthesis pathway

**Figure 4 -:**
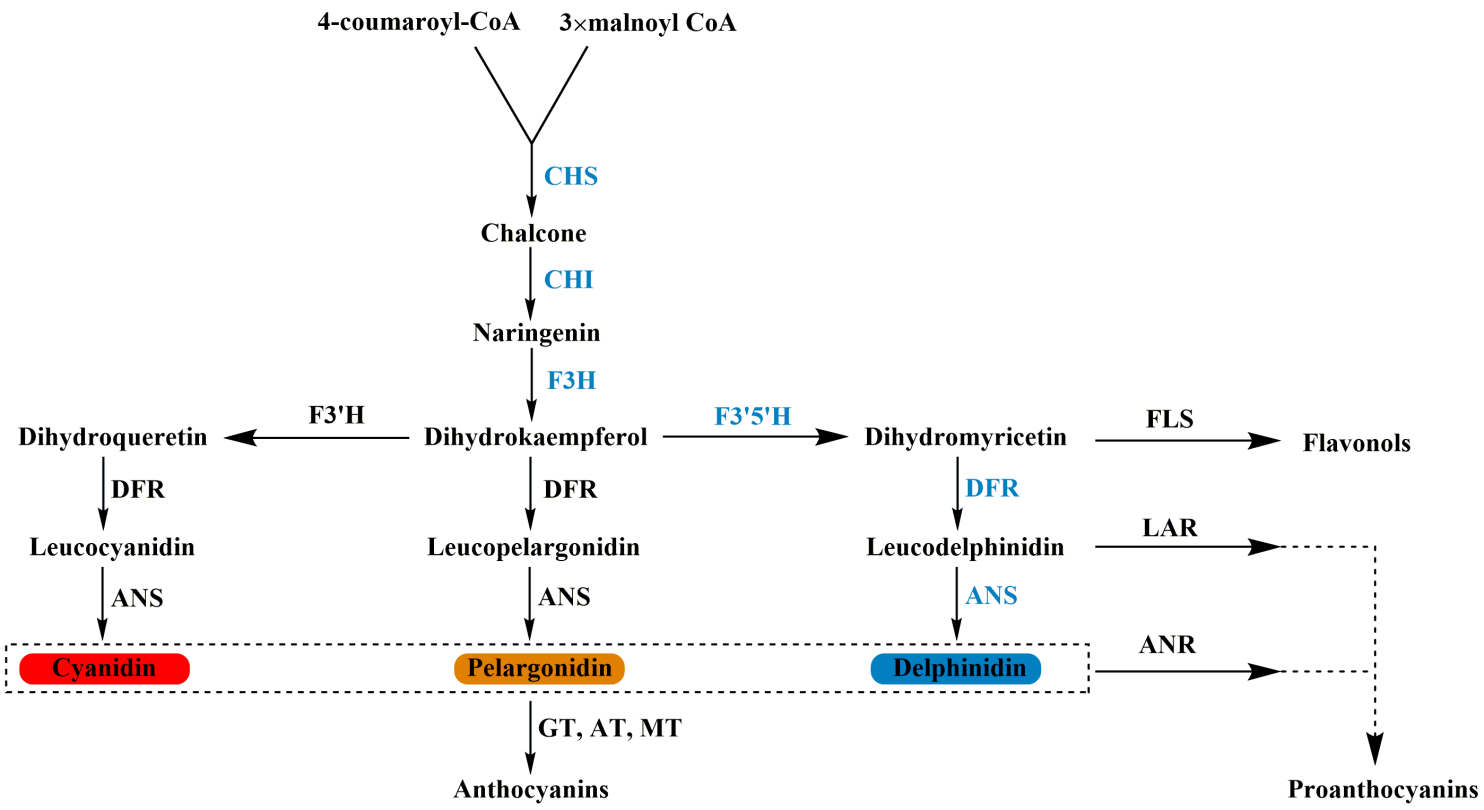
The biosynthetic pathway of anthocyanins. CHS: chalcone synthase; CHI: chalcone isomerase; F3H: flavanone 3-hydroxylase; F3′5′H: flavonoid-3′5′-hydroxylase; F3′H: flavonoid-3′-hydroxylase; FLS: flavonol synthase; DFR: dihydroflavonol 4-reductase; ANS: anthocyanin synthase; ANR: anthocyanidin reductase; GT: glycosyl transferases; AT: acyl transferase; MT: methyl transferase. The key enzymes involved in delphinidin biosynthesis pathway were highlighted blue.

According to the above gene annotation results, a total of 36 DEGs that might be involved in anthocyanin biosynthesis pathway were obtained, including transcription factors and key enzyme genes. As previously reported that chalcone synthase (CHS), chalcone isomerase (CHI), flavanone 3-hydroxylase (F3H), flavonoid 3’,5’-hydroxylase (F3′5′H), dihydroflavonol 4-reductase (DFR) and anthocyanin synthase (ANS) are the key enzymes involved in delphinidin biosynthesis pathway (Figure 4, Nielsen et al., 2002). Therefore, we focused on these genes and obtained five sequences predicted as candidate *CHS* genes, three sequences predicted as candidate *F3H* genes, and one sequence predicted as candidate *DFR* gene (Table 3). We did not find any *CHI*, *F3′5′H*, or *ANS* sequences but found a flavonoid-3′-hydroxylase (*F3′H*) sequence. Therefore, we speculated that *Bougainvillea* can synthesize cyanidin, a competitor of delphinidin, which is referred to as the “red gene” and mainly makes flowers appear orange or red. The absence of *F3′5′H* and existence of competitive pathways made it impossible for *Bougainvillea* to synthesize delphinidin. After BLAST alignment and sequence analysis, we obtained the full-length sequences of two *CHS* genes, one *F3H* gene, one *DFR* gene, and one *F3′H* gene. 

**Table 3 -:** The DEGs corresponding to key enzyme genes involved in delphinidin biosynthesis pathway and their annotation.

Gene ID	NR annotation	NT annotation
DN122252_c2_g1_i3	XP_021747699.1:chalcone synthase like [*Chenopodium quinoa*]	gi|478686564|gb|KC261503.1|:*Tulipa fosteriana* CHS1 mRNA, complete cds
DN122252_c2_g1_i6	XP_021747699.1:chalcone synthase like [*Chenopodium quinoa*]	gi|306415504|gb|HQ161731.1|:*Lilium hybrid* cultivar Siberia chalcone synthase mRNA, complete cds
DN127560_c3_g3_i1	CAA10511.1 chalcone synthase [Catharanthus roseus]	gi|77994621|gb|DQ205352.1|:*Rheum palmatum* chalcone synthase 1 (CHS1) mRNA, complete cds
DN127560_c3_g17_i1	XP_021837196.1:chalcone synthase like [Spinacia oleracea]	gi|215513675|gb|FJ384161.1|:*Cardamine maritima* isolate M3 chalcone synthase gene, exon 2 and partial cds
DN127560_c3_g3_i2	BAB40787.2:chalcone synthase [*Lilium hybrid division I*]	gi|507310951|gb|KC820130.1|:*Apium graveolens* chalcone synthase protein (CHS) mRNA, partial cds
DN122915_c2_g2_i5	XP_010679937.1:PREDICTED: naringenin, 2 oxoglutarate 3 dioxygenase [*Beta vulgaris subsp*. vulgaris]	gi|134039063|gb|EF468104.1|:*Dimocarpus longan* flavanone-3-hydroxylase (f3h) mRNA, complete cds
DN119162_c3_g1_i2	XP_002275563.1:PREDICTED: flavanone 3dioxygenase [*Vitis vinifera*] DN122915_c2_g2_i1	gi|339715869|gb|HM543570.1|:P*runus persica* flavanone 3-hydroxylase (F3H) mRNA, complete cds
DN122915_c2_g2_i1	XP_010679937.1:PREDICTED: naringenin, 2 oxoglutarate 3 dioxygenase [*Beta vulgaris subsp.* vulgaris]	gi|134039063|gb|EF468104.1|:*Dimocarpus longan* flavanone-3-hydroxylase (f3h) mRNA, complete cds
DN156182_c0_g1_i1	BAD67186.1:dihydroflavonol 4 reductase [*Phytolacca americana*]	gi|54888725|dbj|AB128768.1|:Phytolacca americana dfr mRNA for dihydroflavonol 4-reductase, complete cds
DN115083_c0_g1_i2	AMQ23620.1:flavonoid 3’hydroxylase [Silene littorea]	gi|325551318|gb|HQ290518.1|:Camellia nitidissima flavonoid-3’-hydroxylase (F3’H) mRNA, complete cds

### Gene expression analysis through RT-qPCR

**Figure 5 -:**
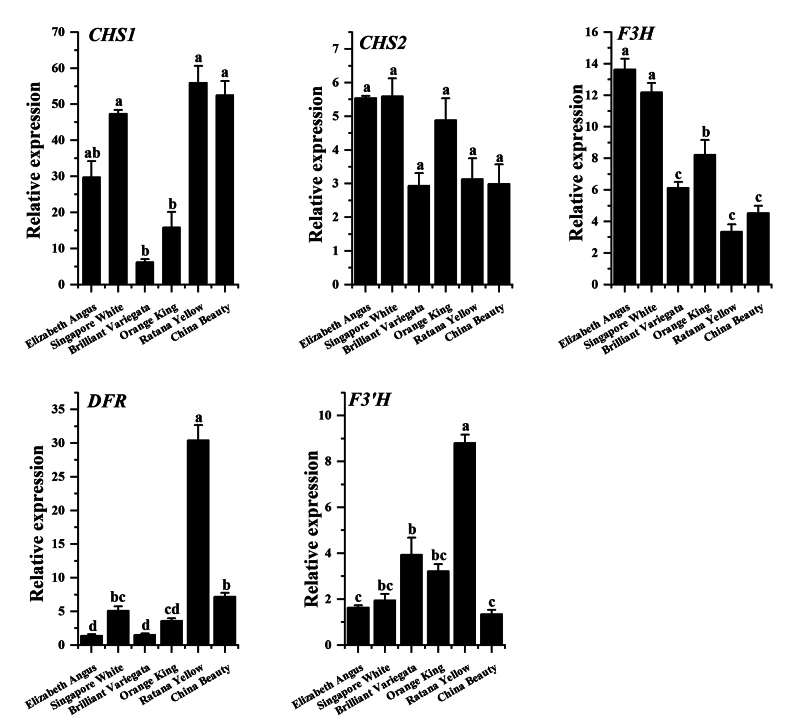
Expression analysis of the genes involved in anthocyanin biosynthesis pathway. The quantitative real-time PCR assay was used to examine genes relative transcription levels in the bracts of single-colour, single-petal *Bougainvillea*. Error bars were obtained from three measurements. Small letter(s) above the bars indicate significant differences (P<0.05) among the samples.

To determine the suitable host of genetic transformation for a blue *Bougainvillea*, we examined the gene expression in the bracts of single-colour, single-petal *Bougainvillea* through RT-qPCR. The results (Figure 5) showed that the transcript level of *CHS1* was highest in *Ratana Yellow*, followed by *China Beauty* and *Singapore White*. The transcript level of *CHS2* was not high in any colour *Bougainvillea*, though its expression was higher in *Singapore White* and *Elizabeth Angus* than in others. The transcript level of *F3H* in *Elizabeth Angus* was 4.06-fold higher than in *Ratana Yellow*, and the level in *Singapore White* was 3.63-fold higher than in *Ratana Yellow*. The *DFR* gene had the highest expression in *Ratana Yellow*. The overall transcript level of *F3′H* was low, though that in *Ratana Yellow* was significantly higher than other colours. The catalysed products of the CHS and F3H provide substrates for the catalysis by F3′5′H while the F3′H and F3′5′H are competitive relationship. Therefore, based on the above results, we speculated that the *Singapore White* and *Elizabeth Angus* were more suitable as blue *Bougainvillea* transgene recipients.

### Analysis of total flavonoids concentrations in bracts

**Figure 6 -:**
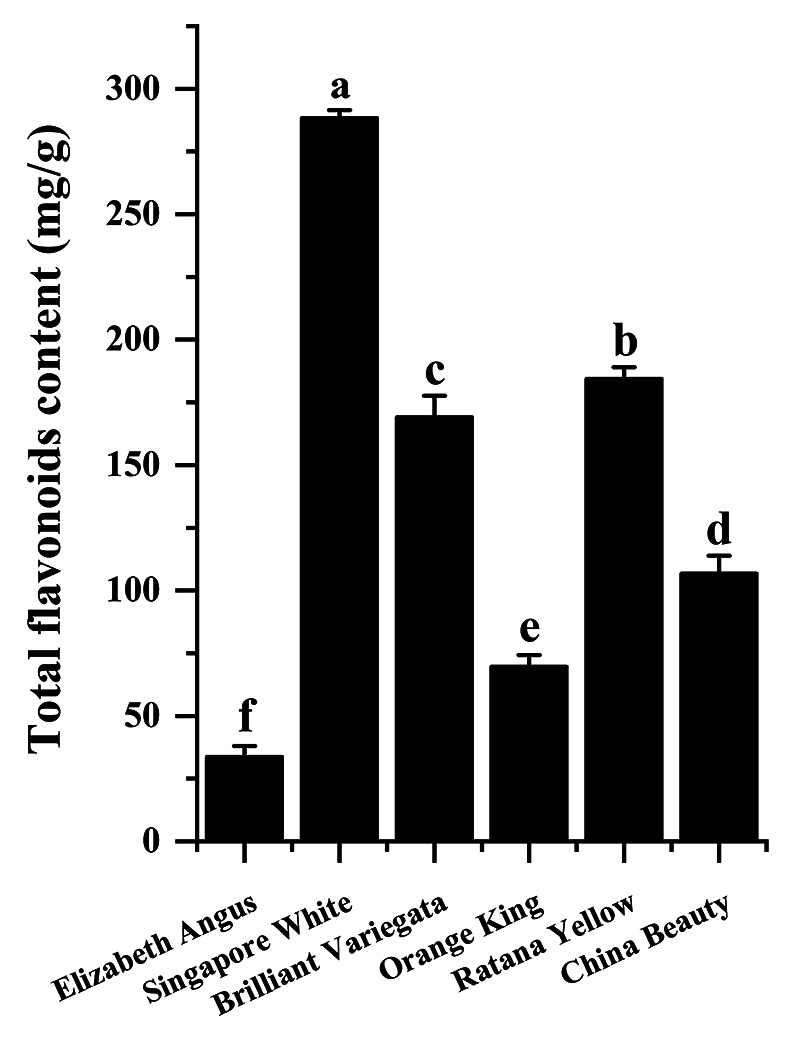
Total flavonoids concentrations in the bracts of *Bougainvillea*. Error bars were obtained from three measurements.

The level of total flavonoids can indicate the amounts of precursor substances. Samples with high flavonoid content are more suitable as blue *Bougainvillea* transgene recipients. Therefore, we examined the flavonoid content in the same samples of the above. The results showed that the highest flavonoid content was in *Singapore White* (288.42±3.14 mg/g), followed by *Ratana Yellow* (184.49±4.47 mg/g), and the lowest was in *Elizabeth Angus* (33.71±4.24 mg/g) (Figure 6). 

### Comprehensive evaluation

The comprehensive membership value of each variety was calculated by membership function formula. According to the average value of the membership function, the variety suitability ranking is obtained (Table 4). The results indicate that the highest average value is in *Singapore White*, followed by *Elizabeth Angus.* The lowest average value is in *Brilliant Variegata*. In summary, the suitable host order for a blue *Bougainvillea* transgene is *Singapore White*>*Elizabeth Angu*s>*Ratana Yellow*>*China Beauty*>*Orange King*> *Brilliant Variegata*. 

**Table 4 -:** The membership function values of the suitability indexes of different *Bougainvillea* cultivars.

Cultivars	Expression level					Flavonoid content	Average value of membership function	Precedence
	*CHS1*	*CHS2*	*F3H*	*DFR*	*F3’H*			
*Elizabeth Angus*	0.473	0.979	1.000	0.000	0.962	0.000	0.569	2
*Singapore White*	0.827	1.000	0.859	0.128	0.921	1.000	0.789	1
*Brilliant Variegata*	0.000	0.000	0.270	0.003	0.653	0.532	0.243	6
*Baolao Cheng*	0.194	0.735	0.476	0.076	0.749	0.141	0.395	5
*Ratana Yellow*	1.000	0.076	0.000	1.000	0.000	0.592	0.445	3
*China Beauty*	0.930	0.022	0.115	0.199	1.000	0.287	0.426	4

## Discussion

As an economy and society develop, the pursuit for novel varieties of flowers with different colors, fragrances, and shapes has become more and more intense. Among them, blue flowers are very popular. Previous study has suggested that the formation of blue flowers requires a special anthocyanin, delphinidin, as well as an appropriate colour rendering environment (An, 1973). However, it is difficult to breed blue flowers with traditional breeding methods. The development of plant genetic engineering technologies has provided tremendous potential for improving and modifying flower traits, breaking boundaries separating species, and providing technical capabilities for directional flower breeding. For example, Courtney-Gutterson et al. (1994) introduced the anti-sense and sense *CHS* gene into pink chrysanthemum (Moneymaker) to make it bloom with fully white and very pale pink flowers. Brugliera et al. (2000) introduced the *F3′5′H* and *difF* genes of petunia together into *Dianthus caryophyllus* that did not have intrinsic F3′5′H activity and obtained blue *D. caryophyllus* plants, since the cytochrome *b*_*5*_ encoded by the *difF* gene could maximally activate F3′5′H activity. Therefore, in this study we focused on these genes to select hosts of genetic transformation that would be suitable for the accumulation of delphinidin.

Betalains are secondary metabolites of *Bougainvillea*, which are responsible for its bract color. The previous study reported that betalain and anthocyanin derivatives have never been found in the same plant (Stafford, 1994). The lack of delphinidin-based anthocyanin leads to no blue *Bougainvillea*. However, Grotewold (2006) reported plants that accumulate the betalain could also synthesize flavone, flavonoids, and even proanthocyanidins, and the lack of some important key enzymes may be the reason why they cannot synthesize anthocyanin. Our results confirm that *Bougainvillea* can synthesize flavonoids and contain key genes in the anthocyanin synthesis pathway, but it lacks the most critical *F3′5′H* gene for the synthesis of delphinidin and *ANS* for the last step. The use of molecular breeding based on genetic engineering methods can overcome this situation. Introducing the exogenous *F3′5′H* gene into the plants may make them able to synthesize delphinidin, thereby achieving the directional cultivation of flower colors.

Here, we only discussed the possibility of blue *Bougainvillea* and found the most appropriate transgene host for blue *Bougainvillea* was *Singapore White*. We speculated that the high betalain levels in other colors may block the synthesis of anthocyanin. A previous study showed that the synthetic precursors of the betalain and anthocyanin are both related to phenylalanine, and thus have a certain competitive relationship (Wang et al., 2006). In addition to delphinidin, the generation of blue flowers also requires a suitable pH environment and appropriate amounts of flavone co-pigment. Zhang et al. (2001) found that flower color tends to red at low pH, white at high pH, and blue at pH close to 7. Harborne and Williams (2000) reported that delphinidin glycosides require less flavone co-pigment to be present to shift the spectrum to blue, when delphinidin glycosides alone are present in plants, in most cases the flowers appear red-purple. Therefore, further studies are needed to verify the functions of key enzyme genes, adjust the pH, examine the concentrations of flavone co-pigment, investigate how to inhibit the betalain synthesis pathway and redirect the metabolic flow to the anthocyanin synthesis pathway, in order to finally achieve the goal of blue *Bougainvillea* cultivar.

The present study revealed that *Bougainvillea* has the potential to synthesize delphinidin. *Singapore White* is the most appropriate host of blue *Bougainvillea* genetic transformation. This study provides a new direction for the cultivation of new colors of *Bougainvillea* and lays the foundation for the breeding of blue *Bougainvillea* by genetic engineering. 

## Supplementary material

The following online material is available for this article:

Table S1 - All annotation information of differentially expressed genes (DEGs).

Table S2 - The pathways of DEGs annotated in the Kyoto Encyclopedia of Genes and Genomes data library.
