# Supplementary material for: Transcriptome data-based screening of potential host of genetic transformation for a blue-hued Bougainvillea transgene
Source: Genet Mol Biol. 2024 Mar 4;47(1):e20230099. doi: 10.1590/1678-4685-GMB-2023-0099 (PMC10941975; doi:10.1590/1678-4685-GMB-2023-0099)
Supplement: Table S2 - [file 1415-4757-GMB-47-1-e20230099-s2.pdf]

Genetics and Molecular Biology

https://doi.org/10.1590/1678-4685-GMB-2023-0099

Supplementary Material to "Transcriptome data-based screening of potential host of genetic transformation for a blue-hued *Bougainvillea* transgene"

Table S2 - The pathways of DEGs annotated in the Kyoto Encyclopedia of Genes and Genomes (KEGG) data library.

Pathway ID	Description	Gene Ratio	PathwayClass	KEGG link	TargetGene	GeneList	geneUp	geneDown
ko00195	Photosynthesis	42/1083	Metabolism; Energy metabolism	KEGG link	42	DN105065_c0_g1_i1;DN105718_c0_g1_i3;DN107577_c0_g2_i1;DN112747_c0_g1_i1;DN112838_c1_g1_i1;DN112838_c1_g1_i4;DN113031_c0_g1_i2;DN114005_c0_g1_i1;DN115222_c1_g2_i2;DN115897_c0_g1_i1;DN117369_c1_g1_i3;DN117369_c1_g3_i1;DN118160_c5_g1_i1;DN118160_c5_g1_i2;DN118355_c2_g8_i3;DN118524_c3_g11_i1;DN118892_c0_g1_i1;DN119643_c2_g3_i1;DN119886_c5_g1_i2;DN119963_c6_g1_i	DN105065_c0_g1_i1;DN105718_c0_g1_i3;DN107577_c0_g2_i1;DN112747_c0_g1_i1;DN112838_c1_g1_i1;DN112838_c1_g1_i4;DN113031_c0_g1_i2;DN114005_c0_g1_i1;DN115222_c1_g2_i2;DN115897_c0_g1_i1;DN117369_c1_g1_i3;DN117369_c1_g3_i1;DN118160_c5_g1_i1;DN118160_c5_g1_i2;DN118355_c2_g8_i3;DN118524_c3_g11_i1;DN118892_c0_g1_i1;DN119643_c2_g3_i1;DN119886_c5_g1_i2;DN119963_c6_g1_i	DN122548_c5_g1_i1;

Pathway ID	Description	Gene Ratio	PathwayClass	KEGG link	TargetGene	GeneList	geneUp	geneDown
						2;DN120248_c4_g1_i1;DN120417_c1_g1_i1;DN120417_c1_g1_i2;DN121346_c7_g7_i1;DN121667_c3_g5_i1;DN122042_c3_g1_i1;DN122208_c1_g1_i1;DN122548_c5_g1_i1;DN123048_c1_g7_i3;DN123350_c5_g4_i2;DN123350_c5_g4_i4;DN123400_c0_g2_i1;DN123400_c0_g2_i3;DN123400_c0_g2_i4;DN123468_c5_g1_i1;DN123468_c5_g2_i2;DN123468_c5_g4_i1;DN123468_c5_g6_i1;DN123468_c5_g6_i2;DN124975_c3_g3_i4;DN128504_c1_g4_i4;DN129073_c2_g1_i2;	2;DN120248_c4_g1_i1;DN120417_c1_g1_i1;DN120417_c1_g1_i2;DN121346_c7_g7_i1;DN121667_c3_g5_i1;DN122042_c3_g1_i1;DN122208_c1_g1_i1;DN123048_c1_g7_i3;DN123350_c5_g4_i2;DN123350_c5_g4_i4;DN123400_c0_g2_i1;DN123400_c0_g2_i3;DN123400_c0_g2_i4;DN123468_c5_g1_i1;DN123468_c5_g2_i2;DN123468_c5_g4_i1;DN123468_c5_g6_i1;DN123468_c5_g6_i2;DN124975_c3_g3_i4;DN128504_c1_g4_i4;DN129073_c2_g1_i2;	

Pathway ID	Description	Gene Ratio	PathwayClass	KEGG link	TargetGene	GeneList	geneUp	geneDown
ko00592	alpha-Linolenic acid metabolism	29/1083	Metabolism; Lipid metabolism	KEGG link	29	DN105742_c1_g1_i2;DN116058_c3_g1_i1;DN116058_c3_g3_i1;DN116058_c3_g3_i2;DN116058_c3_g3_i3;DN118477_c0_g3_i3;DN118477_c0_g4_i1;DN119207_c3_g2_i1;DN119207_c3_g2_i9;DN120170_c2_g1_i1;DN120170_c2_g1_i4;DN120597_c2_g1_i2;DN120597_c2_g1_i3;DN122154_c0_g1_i2;DN124134_c3_g5_i5;DN124134_c3_g5_i6;DN124134_c3_g8_i1;DN124134_c3_g8_i2;DN124134_c3_g8_i6;DN126023_c1_g2_i6;DN127115_c2_g2_i1;DN128682_c2_g1_i1;DN128682_c2_g3_i1;DN128682_c2_g3_i2;DN128682_c2_g5_i12;DN128682_c2_g5_i14;DN128682_c2_g5_i4;DN128682_c2_g5_i7;DN128682_c2_g5_i9;	DN105742_c1_g1_i2;DN116058_c3_g1_i1;DN116058_c3_g3_i1;DN116058_c3_g3_i2;DN116058_c3_g3_i3;DN118477_c0_g4_i1;DN119207_c3_g2_i1;DN119207_c3_g2_i9;DN120170_c2_g1_i1;DN122154_c0_g1_i2;DN128682_c2_g1_i1;DN128682_c2_g3_i1;DN128682_c2_g3_i2;DN128682_c2_g5_i12;DN128682_c2_g5_i4;DN128682_c2_g5_i7;DN128682_c2_g5_i9;	DN118477_c0_g3_i3;DN120170_c2_g1_i4;DN120597_c2_g1_i2;DN120597_c2_g1_i3;DN124134_c3_g5_i5;DN124134_c3_g5_i6;DN124134_c3_g8_i1;DN124134_c3_g8_i2;DN124134_c3_g8_i6;DN126023_c1_g2_i6;DN127115_c2_g2_i1;DN128682_c2_g5_i14;

Pathway ID	Description	Gene Ratio	PathwayClass	KEGG link	TargetGene	GeneList	geneUp	geneDown
ko04612	Antigen processing and presentation	28/1083	Organismal Systems; Immune system	KEGG link	28	DN114565_c0_g4_i2;DN115464_c1_g2_i2;DN116862_c1_g1_i1;DN117680_c0_g1_i2;DN117925_c0_g1_i4;DN118165_c1_g1_i3;DN118868_c0_g6_i1;DN118987_c1_g3_i3;DN119004_c0_g7_i1;DN119658_c0_g2_i4;DN120287_c5_g4_i1;DN120646_c0_g2_i3;DN121311_c2_g1_i2;DN121311_c2_g1_i5;DN121920_c1_g1_i1;DN121920_c1_g1_i5;DN123480_c2_g1_i1;DN125018_c1_g2_i1;DN125018_c1_g3_i3;DN125807_c2_g10_i1;DN125807_c2_g1_i1;DN126821_c2_g3_i1;DN126821_c2_g3_i2;DN126821_c3_g7_i1;DN126830_c1_g3_i2;DN127764_c0_g3_i2;DN79076_c0_g1_i1;DN92669_c0_g1_i1;	DN115464_c1_g2_i2;DN116862_c1_g1_i1;DN117680_c0_g1_i2;DN117925_c0_g1_i4;DN118868_c0_g6_i1;DN118987_c1_g3_i3;DN120287_c5_g4_i1;DN120646_c0_g2_i3;DN121311_c2_g1_i2;DN121311_c2_g1_i5;DN121920_c1_g1_i1;DN121920_c1_g1_i5;DN123480_c2_g1_i1;DN125018_c1_g2_i1;DN125018_c1_g3_i3;DN125807_c2_g10_i1;DN125807_c2_g1_i1;DN126821_c3_g7_i1;DN126830_c1_g3_i2;DN127764_c0_g3_i2;	DN114565_c0_g4_i2;DN118165_c1_g1_i3;DN119004_c0_g7_i1;DN119658_c0_g2_i4;DN126821_c2_g3_i1;DN126821_c2_g3_i2;DN79076_c0_g1_i1;DN92669_c0_g1_i1;

Pathway ID	Description	Gene Ratio	PathwayClass	KEGG link	TargetGene	GeneList	geneUp	geneDown
ko00052	Galactose metabolism	23/1083	Metabolism; Carbohydrate metabolism	KEGG link	23	DN105642_c0_g1_i1;DN108492_c0_g1_i1;DN110505_c0_g1_i1;DN111224_c1_g1_i1;DN113697_c0_g1_i5;DN113697_c0_g1_i7;DN114577_c0_g1_i1;DN116561_c5_g1_i1;DN118842_c0_g1_i11;DN123080_c2_g3_i2;DN123314_c4_g1_i1;DN123314_c4_g1_i2;DN123348_c0_g2_i12;DN123921_c3_g2_i1;DN125573_c1_g1_i2;DN125573_c1_g1_i3;DN126602_c3_g2_i1;DN126736_c0_g7_i1;DN127577_c6_g2_i1;DN127793_c1_g4_i2;DN127819_c3_g2_i8;DN127819_c3_g5_i2;DN128231_c3_g2_i2;	DN123080_c2_g3_i2;DN127577_c6_g2_i1;	DN105642_c0_g1_i1;DN108492_c0_g1_i1;DN110505_c0_g1_i1;DN111224_c1_g1_i1;DN113697_c0_g1_i5;DN113697_c0_g1_i7;DN114577_c0_g1_i1;DN116561_c5_g1_i1;DN118842_c0_g1_i11;DN123314_c4_g1_i1;DN123314_c4_g1_i2;DN123348_c0_g2_i12;DN123921_c3_g2_i1;DN125573_c1_g1_i2;DN125573_c1_g1_i3;DN126602_c3_g2_i1;DN126736_c0_g7_i1;DN127793_c1_g4_i2;DN127819_c3_g2_i8;DN127819_c3_g5_i2;DN128231_c3_g2_i2;
ko00130	Ubiquinone and other terpenoid-quinone biosynthesis	22/1083	Metabolism; Metabolism of cofactors and vitamins	KEGG link	22	DN112977_c0_g1_i2;DN115984_c0_g1_i6;DN118288_c0_g5_i3;DN118288_c0_g5_i4;DN120213_c0_g1_i1;DN121774_c3_g1_i1;DN121774_c3_g3_i1;DN121774_c3_g3_i3;DN122023_c0_g3_i2;DN122037_c2_g1_i3;DN122320_c2_g2_i10;DN123784_c4_g1_i4;DN123784_c4_g1_i5;DN123784_c4_g1_i6;DN123874_c6_g1_i4;DN123921_c2_g2_i1;DN128011_	DN118288_c0_g5_i3;DN118288_c0_g5_i4;DN122023_c0_g3_i2;DN122037_c2_g1_i3;DN122320_c2_g2_i10;DN123784_c4_g1_i4;DN123784_c4_g1_i5;DN123784_c4_g1_i6;DN123874_c6_g1_i4;DN128066_c1_g1_i18;DN129144_c6_g2_i1;	DN112977_c0_g1_i2;DN115984_c0_g1_i6;DN120213_c0_g1_i1;DN121774_c3_g1_i1;DN121774_c3_g3_i1;DN121774_c3_g3_i3;DN123921_c2_g2_i1;DN128011_c3_g2_i1;DN128011_c3_g2_i4;DN128011_c3_g4_i2;DN128687_c2_g3_i7;

Pathway ID	Description	Gene Ratio	PathwayClass	KEGG link	TargetGene	GeneList	geneUp	geneDown
						c3_g2_i1;DN128011_c3_g2_i4;DN128011_c3_g4_i2;DN128066_c1_g1_i18;DN128687_c2_g3_i7;DN129144_c6_g2_i1;		
ko00196	Photosynthesis - antenna proteins	21/1083	Metabolism; Energy metabolism	KEGG link	21	DN105589_c0_g1_i1;DN110976_c0_g1_i1;DN112645_c1_g2_i1;DN113941_c0_g3_i1;DN119874_c4_g6_i2;DN120715_c3_g1_i2;DN121312_c3_g1_i3;DN122884_c1_g6_i1;DN125241_c4_g3_i1;DN126362_c3_g1_i5;DN126362_c3_g1_i7;DN126362_c3_g2_i1;DN126519_c0_g7_i1;DN126617_c0_g2_i2;DN126617_c0_g2_i5;DN128351_c5_g5_i1;DN128351_c5_g5_i2;DN128493_c1_g1_i1;DN128543_c1_g10_i1;DN128955_c0_g2_i2;DN240231_c0_g1_i1;	DN105589_c0_g1_i1;DN110976_c0_g1_i1;DN112645_c1_g2_i1;DN113941_c0_g3_i1;DN119874_c4_g6_i2;DN120715_c3_g1_i2;DN121312_c3_g1_i3;DN122884_c1_g6_i1;DN125241_c4_g3_i1;DN126362_c3_g1_i5;DN126362_c3_g1_i7;DN126362_c3_g2_i1;DN126519_c0_g7_i1;DN126617_c0_g2_i2;DN126617_c0_g2_i5;DN128351_c5_g5_i1;DN128351_c5_g5_i2;DN128493_c1_g1_i1;DN128543_c1_g10_i1;DN128955_c0_g2_i2;DN240231_c0_g1_i1;	;
ko00910	Nitrogen metabolism	21/1083	Metabolism; Energy metabolism	KEGG link	21	DN106864_c0_g1_i1;DN113011_c0_g1_i2;DN114384_c1_g1_i1;DN114384_c1_g1_i3;DN115512_c1_g2_i6;DN117349_c0_g4_i1;DN117349_c0_g7_i3;DN117349_c0_g7_i8;DN117500_c0_g8_i5;DN119632_c0_g1_i1;DN121962_c0_g1_i3;DN122908_c1	DN106864_c0_g1_i1;DN113011_c0_g1_i2;DN114384_c1_g1_i1;DN114384_c1_g1_i3;DN115512_c1_g2_i6;DN117500_c0_g8_i5;DN121962_c0_g1_i3;DN122908_c1_g1_i2;DN122908_c1_g2_i8;DN126086_c0_g1_i4;DN126838_c0_g1_i1;DN126838_c0	DN117349_c0_g4_i1;DN117349_c0_g7_i3;DN117349_c0_g7_i8;DN119632_c0_g1_i1;DN123245_c2_g1_i1;

Pathway ID	Description	Gene Ratio	PathwayClass	KEGG link	TargetGene	GeneList	geneUp	geneDown
						_g1_i2;DN122908_c1_g2_i8;DN123245_c2_g1_i1;DN126086_c0_g1_i4;DN126838_c0_g1_i1;DN126838_c0_g1_i2;DN127759_c2_g1_i5;DN127759_c2_g1_i6;DN127759_c2_g1_i8;DN128285_c3_g1_i3;	_g1_i2;DN127759_c2_g1_i5;DN127759_c2_g1_i6;DN127759_c2_g1_i8;DN128285_c3_g1_i3;	
ko04915	Estrogen signaling pathway	20/1083	Organismal Systems; Endocrine system	KEGG link	20	DN114565_c0_g4_i2;DN115464_c1_g2_i2;DN118868_c0_g6_i1;DN120287_c5_g4_i1;DN121311_c2_g1_i2;DN121311_c2_g1_i5;DN121920_c1_g1_i1;DN121920_c1_g1_i5;DN125807_c2_g10_i1;DN125807_c2_g1_i1;DN126218_c3_g4_i4;DN126218_c3_g4_i8;DN126821_c2_g3_i1;DN126821_c2_g3_i2;DN126821_c3_g7_i1;DN126830_c1_g3_i2;DN127764_c0_g3_i2;DN128341_c3_g5_i2;DN128767_c7_g2_i1;DN92669_c0_g1_i1;	DN115464_c1_g2_i2;DN118868_c0_g6_i1;DN120287_c5_g4_i1;DN121311_c2_g1_i2;DN121311_c2_g1_i5;DN121920_c1_g1_i1;DN121920_c1_g1_i5;DN125807_c2_g10_i1;DN125807_c2_g1_i1;DN126218_c3_g4_i4;DN126218_c3_g4_i8;DN126821_c3_g7_i1;DN126830_c1_g3_i2;DN127764_c0_g3_i2;DN128341_c3_g5_i2;	DN114565_c0_g4_i2;DN126821_c2_g3_i1;DN126821_c2_g3_i2;DN128767_c7_g2_i1;DN92669_c0_g1_i1;
ko00350	Tyrosine metabolism	20/1083	Metabolism; Amino acid metabolism	KEGG link	20	DN114201_c1_g8_i4;DN114201_c1_g8_i7;DN117599_c0_g1_i2;DN118246_c0_g2_i1;DN119009_c0_g1_i2;DN119057_c0_g6_i15;DN119057_c0_g6_i21;DN119057_c0_g6_i29;DN	DN114201_c1_g8_i4;DN114201_c1_g8_i7;DN119057_c0_g6_i15;DN119057_c0_g6_i21;DN119057_c0_g6_i29;DN123784_c4_g1_i4;DN123784_c4_g1_i5;DN123784_c4_g1_i6;DN	DN117599_c0_g1_i2;DN118246_c0_g2_i1;DN119009_c0_g1_i2;DN124134_c3_g5_i5;DN124134_c3_g5_i6;DN124134_c3_g8_i1;DN124134_c3_g8_i2;DN124134_c3_g8_i6;DN124

Pathway ID	Description	Gene Ratio	PathwayClass	KEGG link	TargetGene	GeneList	geneUp	geneDown
						123784_c4_g1_i4;DN123784_c4_g1_i5;DN123784_c4_g1_i6;DN124134_c3_g5_i5;DN124134_c3_g5_i6;DN124134_c3_g8_i1;DN124134_c3_g8_i2;DN124134_c3_g8_i6;DN124216_c1_g1_i1;DN124867_c3_g1_i11;DN125946_c2_g1_i5;DN128952_c2_g1_i2;	125946_c2_g1_i5;	216_c1_g1_i1;DN124867_c3_g1_i11;DN128952_c2_g1_i2;
ko04217	Necroptosis	19/1083	Cellular Processes; Cell growth and death	KEGG link	19	DN105361_c0_g2_i1;DN105361_c0_g2_i2;DN114384_c1_g1_i1;DN114384_c1_g1_i3;DN117349_c0_g4_i1;DN117349_c0_g7_i3;DN117349_c0_g7_i8;DN119632_c0_g1_i1;DN121311_c2_g1_i2;DN121311_c2_g1_i5;DN121920_c1_g1_i1;DN121920_c1_g1_i5;DN127616_c4_g1_i1;DN127616_c4_g1_i10;DN127616_c4_g1_i2;DN127616_c4_g1_i5;DN127764_c0_g3_i2;DN79193_c0_g1_i1;DN92669_c0_g1_i1;	DN114384_c1_g1_i1;DN114384_c1_g1_i3;DN121311_c2_g1_i2;DN121311_c2_g1_i5;DN121920_c1_g1_i1;DN121920_c1_g1_i5;DN127616_c4_g1_i1;DN127616_c4_g1_i10;DN127616_c4_g1_i2;DN127616_c4_g1_i5;DN127764_c0_g3_i2;	DN105361_c0_g2_i1;DN105361_c0_g2_i2;DN117349_c0_g4_i1;DN117349_c0_g7_i3;DN117349_c0_g7_i8;DN119632_c0_g1_i1;DN79193_c0_g1_i1;DN92669_c0_g1_i1;
ko00591	Linoleic acid metabolism	19/1083	Metabolism; Lipid metabolism	KEGG link	19	DN105742_c1_g1_i2;DN116058_c3_g1_i1;DN116058_c3_g3_i1;DN116058_c3_g3_i2;DN116058_c3_g3_i3;DN118477_c0_g3_i3;DN118477_c0	DN105742_c1_g1_i2;DN116058_c3_g1_i1;DN116058_c3_g3_i1;DN116058_c3_g3_i2;DN116058_c3_g3_i3;DN118477_c0_g4_i1;DN120170_c2	DN118477_c0_g3_i3;DN120170_c2_g1_i4;DN127115_c2_g2_i1;DN128682_c2_g5_i14;

Pathway ID	Description	Gene Ratio	PathwayClass	KEGG link	TargetGene	GeneList	geneUp	geneDown
						_g4_i1;DN120170_c2_g1_i1;DN120170_c2_g1_i4;DN122154_c0_g1_i2;DN127115_c2_g2_i1;DN128682_c2_g1_i1;DN128682_c2_g3_i1;DN128682_c2_g3_i2;DN128682_c2_g5_i12;DN128682_c2_g5_i14;DN128682_c2_g5_i4;DN128682_c2_g5_i7;DN128682_c2_g5_i9;	_g1_i1;DN122154_c0_g1_i2;DN128682_c2_g1_i1;DN128682_c2_g3_i1;DN128682_c2_g3_i2;DN128682_c2_g5_i12;DN128682_c2_g5_i4;DN128682_c2_g5_i7;DN128682_c2_g5_i9;	
ko00360	Phenylalanine metabolism	19/1083	Metabolism; Amino acid metabolism	KEGG link	19	DN108568_c0_g1_i1;DN108568_c0_g2_i1;DN108568_c0_g2_i3;DN115984_c0_g1_i6;DN118246_c0_g2_i1;DN119009_c0_g1_i2;DN120213_c0_g1_i1;DN123784_c4_g1_i4;DN123784_c4_g1_i5;DN123784_c4_g1_i6;DN123921_c2_g2_i1;DN124867_c3_g1_i11;DN125074_c5_g2_i10;DN125074_c5_g2_i3;DN125946_c2_g1_i5;DN126775_c2_g1_i1;DN127959_c3_g1_i5;DN128687_c2_g3_i7;DN128952_c2_g1_i2;	DN123784_c4_g1_i4;DN123784_c4_g1_i5;DN123784_c4_g1_i6;DN125946_c2_g1_i5;	DN108568_c0_g1_i1;DN108568_c0_g2_i1;DN108568_c0_g2_i3;DN115984_c0_g1_i6;DN118246_c0_g2_i1;DN119009_c0_g1_i2;DN120213_c0_g1_i1;DN123921_c2_g2_i1;DN124867_c3_g1_i11;DN125074_c5_g2_i10;DN125074_c5_g2_i3;DN126775_c2_g1_i1;DN127959_c3_g1_i5;DN128687_c2_g3_i7;DN128952_c2_g1_i2;

Pathway ID	Description	Gene Ratio	PathwayClass	KEGG link	TargetGene	GeneList	geneUp	geneDown
ko00900	Terpenoid backbone biosynthesis	18/1083	Metabolism; Metabolism of terpenoids and polyketides	KEGG link	18	DN117724_c0_g1_i1;DN119871_c0_g2_i3;DN121051_c2_g4_i14;DN121857_c1_g1_i3;DN121857_c1_g1_i5;DN122448_c1_g1_i4;DN122448_c1_g1_i9;DN122471_c0_g1_i10;DN122471_c0_g1_i11;DN122471_c0_g1_i2;DN122593_c1_g6_i2;DN122593_c1_g6_i3;DN122593_c1_g6_i6;DN127052_c4_g2_i6;DN127248_c0_g1_i5;DN128162_c1_g10_i3;DN128162_c1_g10_i4;DN128162_c1_g9_i1;	DN117724_c0_g1_i1;DN119871_c0_g2_i3;DN121051_c2_g4_i14;DN122448_c1_g1_i4;DN122448_c1_g1_i9;DN122471_c0_g1_i10;DN122471_c0_g1_i11;DN122471_c0_g1_i2;DN122593_c1_g6_i2;DN122593_c1_g6_i3;DN122593_c1_g6_i6;DN127248_c0_g1_i5;DN128162_c1_g10_i3;DN128162_c1_g10_i4;DN128162_c1_g9_i1;	DN121857_c1_g1_i3;DN121857_c1_g1_i5;DN127052_c4_g2_i6;
ko00941	Flavonoid biosynthesis	17/1083	Metabolism; Biosynthesis of other secondary metabolites	KEGG link	17	DN115083_c0_g1_i2;DN115984_c0_g1_i6;DN117404_c0_g1_i1;DN119162_c3_g1_i2;DN121234_c2_g1_i1;DN122252_c2_g1_i3;DN122252_c2_g1_i6;DN122915_c2_g2_i1;DN122915_c2_g2_i2;DN122915_c2_g2_i5;DN123921_c2_g2_i1;DN125074_c5_g2_i10;DN125074_c5_g2_i3;DN127560_c3_g17_i1;DN127560_c3_g3_i1;DN127560_c3_g3_i2;DN127959_c3_g1_i5;	DN117404_c0_g1_i1;	DN115083_c0_g1_i2;DN115984_c0_g1_i6;DN119162_c3_g1_i2;DN121234_c2_g1_i1;DN122252_c2_g1_i3;DN122252_c2_g1_i6;DN122915_c2_g2_i1;DN122915_c2_g2_i2;DN122915_c2_g2_i5;DN123921_c2_g2_i1;DN125074_c5_g2_i10;DN125074_c5_g2_i3;DN127560_c3_g17_i1;DN127560_c3_g3_i1;DN127560_c3_g3_i2;DN127959_c3_g1_i5;

Pathway ID	Description	Gene Ratio	PathwayClass	KEGG link	TargetGene	GeneList	geneUp	geneDown
ko00720	Carbon fixation pathways in prokaryotes	16/1083	Metabolism; Energy metabolism	KEGG link	16	DN101592_c0_g2_i2;DN106303_c0_g1_i1;DN115040_c3_g4_i1;DN115465_c0_g3_i1;DN120286_c3_g2_i2;DN122084_c0_g4_i3;DN122084_c0_g4_i4;DN124509_c1_g1_i1;DN127098_c2_g1_i1;DN127098_c2_g1_i11;DN127098_c2_g1_i13;DN127098_c2_g1_i16;DN127098_c2_g1_i19;DN127098_c2_g1_i3;DN127426_c2_g1_i1;DN84165_c0_g2_i1;	DN120286_c3_g2_i2;DN127098_c2_g1_i1;DN127098_c2_g1_i11;DN127098_c2_g1_i13;DN127098_c2_g1_i16;DN127098_c2_g1_i19;DN127098_c2_g1_i3;	DN101592_c0_g2_i2;DN106303_c0_g1_i1;DN115040_c3_g4_i1;DN115465_c0_g3_i1;DN122084_c0_g4_i3;DN122084_c0_g4_i4;DN124509_c1_g1_i1;DN127426_c2_g1_i1;DN84165_c0_g2_i1;
ko00562	Inositol phosphate metabolism	16/1083	Metabolism; Carbohydrate metabolism	KEGG link	16	DN102195_c0_g2_i1;DN110344_c0_g1_i1;DN112958_c0_g1_i1;DN113227_c0_g1_i4;DN117469_c1_g2_i7;DN118676_c0_g1_i2;DN118676_c1_g2_i1;DN122358_c2_g1_i3;DN122358_c2_g1_i9;DN126097_c4_g1_i17;DN126097_c4_g1_i6;DN126097_c4_g2_i4;DN127939_c1_g1_i1;DN128395_c1_g1_i1;DN128599_c1_g2_i10;DN128870_c4_g2_i3;	DN117469_c1_g2_i7;DN118676_c0_g1_i2;DN118676_c1_g2_i1;DN122358_c2_g1_i3;DN122358_c2_g1_i9;DN128599_c1_g2_i10;	DN102195_c0_g2_i1;DN110344_c0_g1_i1;DN112958_c0_g1_i1;DN113227_c0_g1_i4;DN126097_c4_g1_i17;DN126097_c4_g1_i6;DN126097_c4_g2_i4;DN127939_c1_g1_i1;DN128395_c1_g1_i1;DN128870_c4_g2_i3;

Pathway ID	Description	Gene Ratio	PathwayClass	KEGG link	TargetGene	GeneList	geneUp	geneDown
ko01524	Platinum drug resistance	16/1083	Human Diseases; Drug resistance: Antineoplastic	KEGG link	16	DN107141_c0_g12_i1;DN117491_c1_g1_i6;DN119031_c8_g3_i1;DN119031_c8_g4_i1;DN119031_c8_g4_i2;DN119031_c8_g6_i1;DN119709_c4_g4_i1;DN123920_c0_g1_i1;DN125291_c4_g4_i1;DN126107_c1_g4_i3;DN127489_c0_g2_i3;DN127489_c0_g2_i7;DN127828_c1_g15_i2;DN128061_c0_g1_i3;DN128775_c1_g1_i17;DN128775_c1_g1_i4;	DN107141_c0_g12_i1;DN119031_c8_g3_i1;DN119031_c8_g4_i1;DN119031_c8_g4_i2;DN119031_c8_g6_i1;DN123920_c0_g1_i1;DN125291_c4_g4_i1;DN128775_c1_g1_i17;	DN117491_c1_g1_i6;DN119709_c4_g4_i1;DN126107_c1_g4_i3;DN127489_c0_g2_i3;DN127489_c0_g2_i7;DN127828_c1_g15_i2;DN128061_c0_g1_i3;DN128775_c1_g1_i4;
ko00250	Alanine, aspartate and glutamate metabolism	16/1083	Metabolism; Amino acid metabolism	KEGG link	16	DN114384_c1_g1_i1;DN114384_c1_g1_i3;DN115512_c1_g2_i6;DN116103_c3_g4_i2;DN116103_c3_g4_i5;DN117349_c0_g4_i1;DN117349_c0_g7_i3;DN117349_c0_g7_i8;DN118157_c4_g11_i6;DN118157_c4_g11_i7;DN118157_c4_g12_i2;DN119632_c0_g1_i1;DN123990_c5_g4_i4;DN127574_c1_g5_i4;DN127574_c1_g5_i5;DN127574_c1_g5_i6;	DN114384_c1_g1_i1;DN114384_c1_g1_i3;DN115512_c1_g2_i6;DN116103_c3_g4_i2;DN116103_c3_g4_i5;DN123990_c5_g4_i4;DN127574_c1_g5_i4;DN127574_c1_g5_i5;DN127574_c1_g5_i6;	DN117349_c0_g4_i1;DN117349_c0_g7_i3;DN117349_c0_g7_i8;DN118157_c4_g11_i6;DN118157_c4_g11_i7;DN118157_c4_g12_i2;DN119632_c0_g1_i1;

Pathway ID	Description	Gene Ratio	PathwayClass	KEGG link	TargetGene	GeneList	geneUp	geneDown
ko00982	Drug metabolism - cytochrome P450	15/1083	Metabolism; Xenobiotics biodegradation and metabolism	KEGG link	15	DN107141_c0_g12_i1;DN117491_c1_g1_i6;DN119031_c8_g3_i1;DN119031_c8_g4_i1;DN119031_c8_g4_i2;DN119031_c8_g6_i1;DN119057_c0_g6_i15;DN119057_c0_g6_i21;DN119057_c0_g6_i29;DN119709_c4_g4_i1;DN123478_c1_g1_i8;DN125291_c4_g4_i1;DN127828_c1_g15_i2;DN128775_c1_g1_i17;DN128775_c1_g1_i4;	DN107141_c0_g12_i1;DN119031_c8_g3_i1;DN119031_c8_g4_i1;DN119031_c8_g4_i2;DN119031_c8_g6_i1;DN119057_c0_g6_i15;DN119057_c0_g6_i21;DN119057_c0_g6_i29;DN125291_c4_g4_i1;DN128775_c1_g1_i17;	DN117491_c1_g1_i6;DN119709_c4_g4_i1;DN123478_c1_g1_i8;DN127828_c1_g15_i2;DN128775_c1_g1_i4;
ko02024	Quorum sensing	15/1083	Cellular Processes; Cellular community - prokaryotes	KEGG link	15	DN110344_c0_g1_i1;DN112958_c0_g1_i1;DN117616_c0_g1_i2;DN117616_c0_g1_i3;DN120559_c0_g3_i3;DN122030_c2_g2_i1;DN123568_c0_g1_i1;DN125273_c0_g5_i1;DN127497_c4_g1_i10;DN127497_c4_g1_i12;DN127497_c4_g1_i8;DN128213_c1_g1_i10;DN128213_c1_g1_i11;DN128213_c1_g1_i5;DN128395_c1_g1_i1;	DN120559_c0_g3_i3;DN122030_c2_g2_i1;DN127497_c4_g1_i12;DN128213_c1_g1_i10;DN128213_c1_g1_i11;DN128213_c1_g1_i5;	DN110344_c0_g1_i1;DN112958_c0_g1_i1;DN117616_c0_g1_i2;DN117616_c0_g1_i3;DN123568_c0_g1_i1;DN125273_c0_g5_i1;DN127497_c4_g1_i10;DN127497_c4_g1_i8;DN128395_c1_g1_i1;

Pathway ID	Description	Gene Ratio	PathwayClass	KEGG link	TargetGene	GeneList	geneUp	geneDown
ko00906	Carotenoid biosynthesis	15/1083	Metabolism; Metabolism of terpenoids and polyketides	KEGG link	15	DN116125_c1_g1_i1;DN118440_c1_g1_i1;DN123254_c7_g3_i6;DN124420_c0_g3_i1;DN124560_c0_g1_i6;DN124560_c0_g1_i8;DN124746_c1_g3_i2;DN124746_c1_g3_i3;DN124746_c1_g4_i1;DN125283_c0_g1_i10;DN126294_c1_g2_i11;DN126929_c1_g1_i1;DN126929_c1_g1_i2;DN127260_c1_g5_i3;DN128227_c2_g3_i5;	DN116125_c1_g1_i1;DN123254_c7_g3_i6;DN124420_c0_g3_i1;DN124560_c0_g1_i6;DN124560_c0_g1_i8;DN125283_c0_g1_i10;DN127260_c1_g5_i3;	DN118440_c1_g1_i1;DN124746_c1_g3_i2;DN124746_c1_g3_i3;DN124746_c1_g4_i1;DN126294_c1_g2_i11;DN126929_c1_g1_i1;DN126929_c1_g1_i2;DN128227_c2_g3_i5;
ko04931	Insulin resistance	15/1083	Human Diseases; Endocrine and metabolic diseases	KEGG link	15	DN115243_c1_g1_i4;DN115243_c1_g1_i5;DN117551_c6_g6_i6;DN118441_c4_g4_i1;DN118441_c4_g4_i3;DN118441_c4_g4_i4;DN119061_c2_g1_i2;DN119061_c2_g1_i4;DN119061_c2_g1_i5;DN119812_c4_g1_i3;DN121348_c2_g1_i2;DN124625_c0_g2_i9;DN124879_c2_g5_i3;DN125188_c0_g3_i1;DN126926_c1_g5_i5;	DN117551_c6_g6_i6;DN124625_c0_g2_i9;	DN115243_c1_g1_i4;DN115243_c1_g1_i5;DN118441_c4_g4_i1;DN118441_c4_g4_i3;DN118441_c4_g4_i4;DN119061_c2_g1_i2;DN119061_c2_g1_i4;DN119061_c2_g1_i5;DN119812_c4_g1_i3;DN121348_c2_g1_i2;DN124879_c2_g5_i3;DN125188_c0_g3_i1;DN126926_c1_g5_i5;

Pathway ID	Description	Gene Ratio	PathwayClass	KEGG link	TargetGene	GeneList	geneUp	geneDown
ko00980	Metabolism of xenobiotics by cytochrome P450	14/1083	Metabolism; Xenobiotics biodegradation and metabolism	KEGG link	14	DN107141_c0_g12_i1;DN117491_c1_g1_i6;DN119031_c8_g3_i1;DN119031_c8_g4_i1;DN119031_c8_g4_i2;DN119031_c8_g6_i1;DN119057_c0_g6_i15;DN119057_c0_g6_i21;DN119057_c0_g6_i29;DN119709_c4_g4_i1;DN125291_c4_g4_i1;DN127828_c1_g15_i2;DN128775_c1_g1_i17;DN128775_c1_g1_i4;	DN107141_c0_g12_i1;DN119031_c8_g3_i1;DN119031_c8_g4_i1;DN119031_c8_g4_i2;DN119031_c8_g6_i1;DN119057_c0_g6_i15;DN119057_c0_g6_i21;DN119057_c0_g6_i29;DN125291_c4_g4_i1;DN128775_c1_g1_i17;	DN117491_c1_g1_i6;DN119709_c4_g4_i1;DN127828_c1_g15_i2;DN128775_c1_g1_i4;
ko05204	Chemical carcinogenesis	14/1083	Human Diseases; Cancers: Overview	KEGG link	14	DN107141_c0_g12_i1;DN117491_c1_g1_i6;DN119031_c8_g3_i1;DN119031_c8_g4_i1;DN119031_c8_g4_i2;DN119031_c8_g6_i1;DN119057_c0_g6_i15;DN119057_c0_g6_i21;DN119057_c0_g6_i29;DN119709_c4_g4_i1;DN125291_c4_g4_i1;DN127828_c1_g15_i2;DN128775_c1_g1_i17;DN128775_c1_g1_i4;	DN107141_c0_g12_i1;DN119031_c8_g3_i1;DN119031_c8_g4_i1;DN119031_c8_g4_i2;DN119031_c8_g6_i1;DN119057_c0_g6_i15;DN119057_c0_g6_i21;DN119057_c0_g6_i29;DN125291_c4_g4_i1;DN128775_c1_g1_i17;	DN117491_c1_g1_i6;DN119709_c4_g4_i1;DN127828_c1_g15_i2;DN128775_c1_g1_i4;
ko00860	Porphyrin and chlorophyll metab	14/1083	Metabolism; Metabolism of cofactors and	KEGG link	14	DN113964_c0_g2_i2;DN114711_c1_g2_i2;DN117055_c0_g3_i1;DN117055_c0_g3_i2;DN121858_c2_g2_i1;DN122006_c5_g2_i5;DN122006_c5_g3_i2;DN122861_c2_g1_i4;DN124515_c1_g2_i13;DN124515_c1_g2_i	DN113964_c0_g2_i2;DN114711_c1_g2_i2;DN117055_c0_g3_i1;DN117055_c0_g3_i2;DN121858_c2_g2_i1;DN122006_c5_g2_i5;DN122006_c5_g3_i2;DN122861_c2_g1_i4;DN124515_c1_g2_i13;DN124515_c1_g2_i	DN126720_c2_g1_i1;DN127128_c5_g4_i1;DN127128_c5_g4_i2;

Pathway ID	Description	Gene Ratio	PathwayClass	KEGG link	TargetGene	GeneList	geneUp	geneDown
	olism		vitamins			3;DN126142_c1_g4_i8;DN126720_c2_g1_i1;DN127128_c5_g4_i1;DN127128_c5_g4_i2;	3;DN126142_c1_g4_i8;	
ko00220	Arginine biosynthesis	14/1083	Metabolism; Amino acid metabolism	KEGG link	14	DN114384_c1_g1_i1;DN114384_c1_g1_i3;DN117349_c0_g4_i1;DN117349_c0_g7_i3;DN117349_c0_g7_i8;DN118150_c2_g7_i2;DN118157_c4_g11_i6;DN118157_c4_g11_i7;DN118157_c4_g12_i2;DN119632_c0_g1_i1;DN123444_c0_g4_i1;DN127574_c1_g5_i4;DN127574_c1_g5_i5;DN127574_c1_g5_i6;	DN114384_c1_g1_i1;DN114384_c1_g1_i3;DN127574_c1_g5_i4;DN127574_c1_g5_i5;DN127574_c1_g5_i6;	DN117349_c0_g4_i1;DN117349_c0_g7_i3;DN117349_c0_g7_i8;DN118150_c2_g7_i2;DN118157_c4_g11_i6;DN118157_c4_g11_i7;DN118157_c4_g12_i2;DN119632_c0_g1_i1;DN123444_c0_g4_i1;
ko04010	MAPK signaling pathway	14/1083	Environmental Information Processing; Signal transduction	KEGG link	14	DN114565_c0_g4_i2;DN115464_c1_g2_i2;DN118868_c0_g6_i1;DN120287_c5_g4_i1;DN120346_c0_g1_i7;DN123214_c4_g2_i3;DN123214_c4_g2_i5;DN123214_c4_g2_i9;DN125807_c2_g10_i1;DN125807_c2_g1_i1;DN126821_c2_g3_i1;DN126821_c2_g3_i2;DN126821_c3_g7_i1;DN126830_c1_g3_i2;	DN115464_c1_g2_i2;DN118868_c0_g6_i1;DN120287_c5_g4_i1;DN120346_c0_g1_i7;DN123214_c4_g2_i3;DN123214_c4_g2_i5;DN123214_c4_g2_i9;DN125807_c2_g10_i1;DN125807_c2_g1_i1;DN126821_c3_g7_i1;DN126830_c1_g3_i2;	DN114565_c0_g4_i2;DN126821_c2_g3_i1;DN126821_c2_g3_i2;

Pathway ID	Description	Gene Ratio	PathwayClass	KEGG link	TargetGene	GeneList	geneUp	geneDown
ko00902	Monoterpenoid biosynthesis	14/1083	Metabolism; Metabolism of terpenoids and polyketides	KEGG link	14	DN102099_c0_g1_i1;DN119920_c2_g5_i1;DN119920_c2_g5_i3;DN119920_c2_g5_i4;DN119920_c2_g5_i8;DN124472_c0_g2_i4;DN127223_c0_g1_i1;DN128865_c7_g6_i2;DN129195_c56_g4_i5;DN129195_c59_g1_i2;DN129195_c59_g1_i3;DN129195_c59_g4_i2;DN129195_c59_g4_i4;DN82637_c0_g1_i1;	DN119920_c2_g5_i1;DN119920_c2_g5_i3;DN119920_c2_g5_i4;DN119920_c2_g5_i8;DN128865_c7_g6_i2;DN129195_c56_g4_i5;	DN102099_c0_g1_i1;DN124472_c0_g2_i4;DN127223_c0_g1_i1;DN129195_c59_g1_i2;DN129195_c59_g1_i3;DN129195_c59_g4_i2;DN129195_c59_g4_i4;DN82637_c0_g1_i1;
ko00400	Phenylalanine, tyrosine and tryptophan biosynthesis	13/1083	Metabolism; Amino acid metabolism	KEGG link	13	DN113088_c2_g1_i2;DN115175_c0_g3_i1;DN117053_c0_g1_i4;DN117616_c0_g1_i2;DN117616_c0_g1_i3;DN118660_c1_g8_i4;DN118981_c0_g8_i1;DN121583_c0_g1_i5;DN123568_c0_g1_i1;DN123784_c4_g1_i4;DN123784_c4_g1_i5;DN123784_c4_g1_i6;DN125710_c2_g3_i6;	DN121583_c0_g1_i5;DN123784_c4_g1_i4;DN123784_c4_g1_i5;DN123784_c4_g1_i6;	DN113088_c2_g1_i2;DN115175_c0_g3_i1;DN117053_c0_g1_i4;DN117616_c0_g1_i2;DN117616_c0_g1_i3;DN118660_c1_g8_i4;DN118981_c0_g8_i1;DN123568_c0_g1_i1;DN125710_c2_g3_i6;

Pathway ID	Description	Gene Ratio	PathwayClass	KEGG link	TargetGene	GeneList	geneUp	geneDown
ko05205	Proteoglycans in cancer	13/1083	Human Diseases; Cancers: Overview	KEGG link	13	DN106153_c0_g1_i1;DN107831_c0_g3_i1;DN109405_c0_g1_i1;DN115243_c1_g1_i4;DN115243_c1_g1_i5;DN117557_c0_g1_i17;DN119004_c0_g7_i1;DN120133_c0_g2_i3;DN124625_c0_g2_i9;DN125310_c4_g3_i1;DN126049_c0_g1_i1;DN131056_c0_g1_i1;DN13234_c0_g1_i1;	DN117557_c0_g1_i17;DN124625_c0_g2_i9;DN125310_c4_g3_i1;	DN106153_c0_g1_i1;DN107831_c0_g3_i1;DN109405_c0_g1_i1;DN115243_c1_g1_i4;DN115243_c1_g1_i5;DN119004_c0_g7_i1;DN120133_c0_g2_i3;DN126049_c0_g1_i1;DN131056_c0_g1_i1;DN13234_c0_g1_i1;
ko00640	Propanoate metabolism	13/1083	Metabolism; Carbohydrate metabolism	KEGG link	13	DN101592_c0_g2_i2;DN115465_c0_g3_i1;DN117396_c6_g1_i2;DN118978_c1_g1_i5;DN119133_c3_g6_i10;DN119133_c3_g6_i9;DN120286_c3_g2_i2;DN122084_c0_g4_i3;DN122084_c0_g4_i4;DN124032_c4_g5_i14;DN124032_c4_g5_i4;DN127426_c2_g1_i1;DN127595_c2_g1_i1;	DN118978_c1_g1_i5;DN119133_c3_g6_i10;DN119133_c3_g6_i9;DN120286_c3_g2_i2;DN124032_c4_g5_i14;DN124032_c4_g5_i4;	DN101592_c0_g2_i2;DN115465_c0_g3_i1;DN117396_c6_g1_i2;DN122084_c0_g4_i3;DN122084_c0_g4_i4;DN127426_c2_g1_i1;DN127595_c2_g1_i1;
ko04390	Hippo signaling pathway	13/1083	Environmental Information Processing; Signal transduction	KEGG link	13	DN107826_c1_g1_i6;DN115243_c1_g1_i4;DN115243_c1_g1_i5;DN121802_c0_g3_i3;DN121802_c0_g3_i4;DN122415_c1_g1_i1;DN123205_c4_g2_i7;DN124625_c0_g2_i9;DN125626_c0_g3_i12;DN126049_c0_g1_i1;DN128018_c4_g5_i1;DN131056_c0_g1_i1;DN13234_c0_g1_i1;	DN121802_c0_g3_i3;DN123205_c4_g2_i7;DN124625_c0_g2_i9;DN125626_c0_g3_i12;DN128018_c4_g5_i1;	DN107826_c1_g1_i6;DN115243_c1_g1_i4;DN115243_c1_g1_i5;DN121802_c0_g3_i4;DN122415_c1_g1_i1;DN126049_c0_g1_i1;DN131056_c0_g1_i1;DN13234_c0_g1_i1;

Pathway ID	Description	Gene Ratio	PathwayClass	KEGG link	TargetGene	GeneList	geneUp	geneDown
ko00460	Cyano amino acid metabolism	12/1083	Metabolism; Metabolism of other amino acids	KEGG link	12	DN115960_c0_g1_i1;DN119000_c2_g2_i3;DN119000_c2_g2_i4;DN119461_c1_g1_i1;DN119461_c1_g1_i2;DN121962_c0_g1_i3;DN122398_c0_g1_i1;DN123021_c1_g1_i1;DN126066_c2_g1_i10;DN126604_c3_g2_i2;DN128845_c1_g1_i2;DN128845_c1_g1_i5;	DN119000_c2_g2_i3;DN119000_c2_g2_i4;DN119461_c1_g1_i2;DN121962_c0_g1_i3;DN126066_c2_g1_i10;DN126604_c3_g2_i2;	DN115960_c0_g1_i1;DN119461_c1_g1_i1;DN122398_c0_g1_i1;DN123021_c1_g1_i1;DN128845_c1_g1_i2;DN128845_c1_g1_i5;
ko00040	Pentose and glucuronate interconversions	12/1083	Metabolism; Carbohydrate metabolism	KEGG link	12	DN105642_c0_g1_i1;DN111918_c0_g1_i2;DN118493_c0_g2_i1;DN122733_c3_g2_i12;DN122733_c3_g2_i14;DN122733_c3_g2_i8;DN124568_c0_g1_i2;DN125273_c0_g5_i1;DN126719_c1_g1_i1;DN126736_c0_g7_i1;DN127561_c0_g3_i3;DN127561_c0_g8_i9;	DN122733_c3_g2_i12;DN122733_c3_g2_i14;DN122733_c3_g2_i8;DN127561_c0_g3_i3;DN127561_c0_g8_i9;	DN105642_c0_g1_i1;DN111918_c0_g1_i2;DN118493_c0_g2_i1;DN124568_c0_g1_i2;DN125273_c0_g5_i1;DN126719_c1_g1_i1;DN126736_c0_g7_i1;
ko04070	Phosphatidylinositol signaling system	12/1083	Environmental Information Processing; Signal transduction	KEGG link	12	DN113227_c0_g1_i4;DN119056_c0_g3_i5;DN119776_c1_g6_i1;DN125127_c0_g2_i5;DN126097_c4_g1_i17;DN126097_c4_g1_i6;DN126097_c4_g2_i4;DN127939_c1_g1_i1;DN128244_c6_g1_i3;DN128599_c1_g2_i10;DN128767_c7_g2_i1;DN128870_c4_g2_i3;	DN119056_c0_g3_i5;DN119776_c1_g6_i1;DN128599_c1_g2_i10;	DN113227_c0_g1_i4;DN125127_c0_g2_i5;DN126097_c4_g1_i17;DN126097_c4_g1_i6;DN126097_c4_g2_i4;DN127939_c1_g1_i1;DN128244_c6_g1_i3;DN128767_c7_g2_i1;DN128870_c4_g2_i3;

Pathway ID	Description	Gene Ratio	PathwayClass	KEGG link	TargetGene	GeneList	geneUp	geneDown
ko02010	ABC transporters	12/1083	Environmental Information Processing; Membrane transport	KEGG link	12	DN113690_c0_g1_i1;DN123920_c0_g1_i1;DN124392_c3_g1_i5;DN124392_c3_g1_i9;DN125656_c1_g1_i4;DN126107_c1_g4_i3;DN127139_c0_g2_i1;DN127139_c0_g2_i2;DN127849_c1_g4_i3;DN127849_c1_g4_i4;DN127849_c1_g5_i2;DN128061_c0_g1_i3;	DN123920_c0_g1_i1;DN127139_c0_g2_i1;DN127139_c0_g2_i2;	DN113690_c0_g1_i1;DN124392_c3_g1_i5;DN124392_c3_g1_i9;DN125656_c1_g1_i4;DN126107_c1_g4_i3;DN127849_c1_g4_i3;DN127849_c1_g4_i4;DN127849_c1_g5_i2;DN128061_c0_g1_i3;
ko04976	Bile secretion	12/1083	Organismal Systems; Digestive system	KEGG link	12	DN113690_c0_g1_i1;DN121857_c1_g1_i3;DN121857_c1_g1_i5;DN123920_c0_g1_i1;DN125656_c1_g1_i4;DN126107_c1_g4_i3;DN127139_c0_g2_i1;DN127139_c0_g2_i2;DN127849_c1_g4_i3;DN127849_c1_g4_i4;DN127849_c1_g5_i2;DN128061_c0_g1_i3;	DN123920_c0_g1_i1;DN127139_c0_g2_i1;DN127139_c0_g2_i2;	DN113690_c0_g1_i1;DN121857_c1_g1_i3;DN121857_c1_g1_i5;DN125656_c1_g1_i4;DN126107_c1_g4_i3;DN127849_c1_g4_i3;DN127849_c1_g4_i4;DN127849_c1_g5_i2;DN128061_c0_g1_i3;
ko04910	Insulin signaling pathway	12/1083	Organismal Systems; Endocrine system	KEGG link	12	DN109405_c0_g1_i1;DN115243_c1_g1_i4;DN115243_c1_g1_i5;DN117551_c6_g6_i6;DN122236_c3_g6_i5;DN122236_c3_g6_i7;DN122236_c3_g6_i8;DN123921_c3_g2_i1;DN124625_c0_g2_i9;DN124994_c1_g1_i2;DN127595_c2_g1_i1;DN128767_c7_g2_i1;	DN117551_c6_g6_i6;DN122236_c3_g6_i5;DN122236_c3_g6_i7;DN122236_c3_g6_i8;DN124625_c0_g2_i9;DN124994_c1_g1_i2;	DN109405_c0_g1_i1;DN115243_c1_g1_i4;DN115243_c1_g1_i5;DN123921_c3_g2_i1;DN127595_c2_g1_i1;DN128767_c7_g2_i1;

Pathway ID	Description	Gene Ratio	PathwayClass	KEGG link	TargetGene	GeneList	geneUp	geneDown
ko04919	Thyroid hormone signaling pathway	11/1083	Organismal Systems; Endocrine system	KEGG link	11	DN110344_c0_g1_i1;DN112958_c0_g1_i1;DN126049_c0_g1_i1;DN126097_c4_g1_i17;DN126097_c4_g1_i6;DN126097_c4_g2_i4;DN126428_c0_g3_i6;DN127939_c1_g1_i1;DN128395_c1_g1_i1;DN131056_c0_g1_i1;DN13234_c0_g1_i1;	;	DN110344_c0_g1_i1;DN112958_c0_g1_i1;DN126049_c0_g1_i1;DN126097_c4_g1_i17;DN126097_c4_g1_i6;DN126097_c4_g2_i4;DN126428_c0_g3_i6;DN127939_c1_g1_i1;DN128395_c1_g1_i1;DN131056_c0_g1_i1;DN13234_c0_g1_i1;
ko00061	Fatty acid biosynthesis	11/1083	Metabolism; Lipid metabolism	KEGG link	11	DN115465_c0_g3_i1;DN117874_c0_g1_i6;DN117874_c0_g1_i8;DN119487_c1_g2_i10;DN119487_c1_g2_i8;DN120286_c3_g2_i2;DN123630_c1_g1_i9;DN127497_c4_g1_i10;DN127497_c4_g1_i12;DN127497_c4_g1_i8;DN127595_c2_g1_i1;	DN119487_c1_g2_i10;DN120286_c3_g2_i2;DN123630_c1_g1_i9;DN127497_c4_g1_i12;	DN115465_c0_g3_i1;DN117874_c0_g1_i6;DN117874_c0_g1_i8;DN119487_c1_g2_i8;DN127497_c4_g1_i10;DN127497_c4_g1_i8;DN127595_c2_g1_i1;
ko05200	Pathways in cancer	11/1083	Human Diseases; Cancers: Overview	KEGG link	11	DN111348_c0_g1_i3;DN120304_c1_g1_i1;DN121311_c2_g1_i2;DN121311_c2_g1_i5;DN121920_c1_g1_i1;DN121920_c1_g1_i5;DN127489_c0_g2_i3;DN127489_c0_g2_i7;DN127764_c0_g3_i2;DN128341_c3_g5_i2;DN92669_c0_g1_i1;	DN111348_c0_g1_i3;DN120304_c1_g1_i1;DN121311_c2_g1_i2;DN121311_c2_g1_i5;DN121920_c1_g1_i1;DN121920_c1_g1_i5;DN127764_c0_g3_i2;DN128341_c3_g5_i2;	DN127489_c0_g2_i3;DN127489_c0_g2_i7;DN92669_c0_g1_i1;

Pathway ID	Description	Gene Ratio	PathwayClass	KEGG link	TargetGene	GeneList	geneUp	geneDown
ko00904	Diterpenoid biosynthesis	11/1083	Metabolism; Metabolism of terpenoids and polyketides	KEGG link	11	DN113764_c0_g1_i1;DN115687_c1_g2_i1;DN116525_c1_g1_i10;DN116525_c1_g1_i12;DN116525_c1_g1_i2;DN125485_c0_g1_i3;DN125485_c0_g3_i1;DN125485_c0_g3_i2;DN125485_c0_g3_i3;DN125485_c0_g3_i4;DN128863_c1_g1_i4;	DN113764_c0_g1_i1;	DN115687_c1_g2_i1;DN116525_c1_g1_i10;DN116525_c1_g1_i12;DN116525_c1_g1_i2;DN125485_c0_g1_i3;DN125485_c0_g3_i1;DN125485_c0_g3_i2;DN125485_c0_g3_i3;DN125485_c0_g3_i4;DN128863_c1_g1_i4;
ko00330	Arginine and proline metabolism	11/1083	Metabolism; Amino acid metabolism	KEGG link	11	DN100430_c0_g1_i1;DN108548_c0_g1_i3;DN120099_c1_g1_i4;DN121980_c1_g1_i1;DN121980_c1_g1_i6;DN122131_c1_g3_i1;DN123388_c2_g4_i10;DN123471_c2_g3_i13;DN127018_c1_g2_i2;DN127776_c4_g4_i2;DN81897_c0_g1_i1;	;	DN100430_c0_g1_i1;DN108548_c0_g1_i3;DN120099_c1_g1_i4;DN121980_c1_g1_i1;DN121980_c1_g1_i6;DN122131_c1_g3_i1;DN123388_c2_g4_i10;DN123471_c2_g3_i13;DN127018_c1_g2_i2;DN127776_c4_g4_i2;DN81897_c0_g1_i1;
ko00950	Isoquinoline alkaloid biosynthesis	11/1083	Metabolism; Biosynthesis of other secondary metabolites	KEGG link	11	DN114201_c1_g8_i4;DN114201_c1_g8_i7;DN117599_c0_g1_i2;DN118246_c0_g2_i1;DN119009_c0_g1_i2;DN123784_c4_g1_i4;DN123784_c4_g1_i5;DN123784_c4_g1_i6;DN124216_c1_g1_i1;DN125946_c2_g1_i5;DN128952_c2_g1_i2;	DN114201_c1_g8_i4;DN114201_c1_g8_i7;DN123784_c4_g1_i4;DN123784_c4_g1_i5;DN123784_c4_g1_i6;DN125946_c2_g1_i5;	DN117599_c0_g1_i2;DN118246_c0_g2_i1;DN119009_c0_g1_i2;DN124216_c1_g1_i1;DN128952_c2_g1_i2;

Pathway ID	Description	Gene Ratio	PathwayClass	KEGG link	TargetGene	GeneList	geneUp	geneDown
ko04020	Calcium signaling pathway	10/1083	Environmental Information Processing; Signal transduction	KEGG link	10	DN120033_c1_g1_i1;DN120033_c1_g1_i3;DN120033_c1_g1_i5;DN120033_c1_g1_i6;DN120346_c0_g1_i7;DN126097_c4_g1_i17;DN126097_c4_g1_i6;DN126097_c4_g2_i4;DN127939_c1_g1_i1;DN128767_c7_g2_i1;	DN120033_c1_g1_i1;DN120346_c0_g1_i7;	DN120033_c1_g1_i3;DN120033_c1_g1_i5;DN120033_c1_g1_i6;DN126097_c4_g1_i17;DN126097_c4_g1_i6;DN126097_c4_g2_i4;DN127939_c1_g1_i1;DN128767_c7_g2_i1;
ko04391	Hippo signaling pathway - fly	10/1083	Environmental Information Processing; Signal transduction	KEGG link	10	DN107826_c1_g1_i6;DN121802_c0_g3_i3;DN121802_c0_g3_i4;DN122415_c1_g1_i1;DN123205_c4_g2_i7;DN125626_c0_g3_i12;DN126049_c0_g1_i1;DN128018_c4_g5_i1;DN131056_c0_g1_i1;DN13234_c0_g1_i1;	DN121802_c0_g3_i3;DN123205_c4_g2_i7;DN125626_c0_g3_i12;DN128018_c4_g5_i1;	DN107826_c1_g1_i6;DN121802_c0_g3_i4;DN122415_c1_g1_i1;DN126049_c0_g1_i1;DN131056_c0_g1_i1;DN13234_c0_g1_i1;
ko04727	GABAergic synapse	10/1083	Organismal Systems; Nervous system	KEGG link	10	DN114384_c1_g1_i1;DN114384_c1_g1_i3;DN117349_c0_g4_i1;DN117349_c0_g7_i3;DN117349_c0_g7_i8;DN118851_c4_g2_i14;DN118851_c4_g2_i9;DN119632_c0_g1_i1;DN120376_c0_g9_i4;DN120376_c0_g9_i6;	DN114384_c1_g1_i1;DN114384_c1_g1_i3;DN118851_c4_g2_i14;DN118851_c4_g2_i9;	DN117349_c0_g4_i1;DN117349_c0_g7_i3;DN117349_c0_g7_i8;DN119632_c0_g1_i1;DN120376_c0_g9_i4;DN120376_c0_g9_i6;

Pathway ID	Description	Gene Ratio	PathwayClass	KEGG link	TargetGene	GeneList	geneUp	geneDown
ko04921	Oxytocin signaling pathway	10/1083	Organismal Systems; Endocrine system	KEGG link	10	DN104121_c0_g2_i1;DN115243_c1_g1_i4;DN115243_c1_g1_i5;DN117551_c6_g6_i6;DN120346_c0_g1_i7;DN124625_c0_g2_i9;DN126049_c0_g1_i1;DN128767_c7_g2_i1;DN131056_c0_g1_i1;DN13234_c0_g1_i1;	DN117551_c6_g6_i6;DN120346_c0_g1_i7;DN124625_c0_g2_i9;	DN104121_c0_g2_i1;DN115243_c1_g1_i4;DN115243_c1_g1_i5;DN126049_c0_g1_i1;DN128767_c7_g2_i1;DN131056_c0_g1_i1;DN13234_c0_g1_i1;
ko00410	beta-Alanine metabolism	10/1083	Metabolism; Metabolism of other amino acids	KEGG link	10	DN118246_c0_g2_i1;DN119009_c0_g1_i2;DN120099_c1_g1_i4;DN121980_c1_g1_i1;DN121980_c1_g1_i6;DN122131_c1_g3_i1;DN123388_c2_g4_i10;DN123471_c2_g3_i13;DN125946_c2_g1_i5;DN128952_c2_g1_i2;	DN125946_c2_g1_i5;	DN118246_c0_g2_i1;DN119009_c0_g1_i2;DN120099_c1_g1_i4;DN121980_c1_g1_i1;DN121980_c1_g1_i6;DN122131_c1_g3_i1;DN123388_c2_g4_i10;DN123471_c2_g3_i13;DN128952_c2_g1_i2;
ko00053	Ascorbate and aldarate metabolism	10/1083	Metabolism; Carbohydrate metabolism	KEGG link	10	DN110662_c0_g1_i1;DN114192_c0_g1_i12;DN114192_c0_g1_i13;DN118493_c0_g2_i1;DN118676_c0_g1_i2;DN118676_c1_g2_i1;DN121635_c0_g1_i4;DN121980_c1_g1_i1;DN121980_c1_g1_i6;DN123388_c2_g4_i10;	DN110662_c0_g1_i1;DN114192_c0_g1_i12;DN114192_c0_g1_i13;DN118676_c0_g1_i2;DN118676_c1_g2_i1;DN121635_c0_g1_i4;	DN118493_c0_g2_i1;DN121980_c1_g1_i1;DN121980_c1_g1_i6;DN123388_c2_g4_i10;

Pathway ID	Description	Gene Ratio	PathwayClass	KEGG link	TargetGene	GeneList	geneUp	geneDown
ko02020	Two-component system	9/1083	Environmental Information Processing; Signal transduction	KEGG link	9	DN114384_c1_g1_i1;DN114384_c1_g1_i3;DN115634_c0_g2_i1;DN117349_c0_g4_i1;DN117349_c0_g7_i3;DN117349_c0_g7_i8;DN119632_c0_g1_i1;DN127489_c0_g2_i3;DN127489_c0_g2_i7;	DN114384_c1_g1_i1;DN114384_c1_g1_i3;	DN115634_c0_g2_i1;DN117349_c0_g4_i1;DN117349_c0_g7_i3;DN117349_c0_g7_i8;DN119632_c0_g1_i1;DN127489_c0_g2_i3;DN127489_c0_g2_i7;
ko05206	MicroRNAs in cancer	9/1083	Human Diseases; Cancers: Overview	KEGG link	9	DN106153_c0_g1_i1;DN113690_c0_g1_i1;DN117299_c2_g2_i18;DN125656_c1_g1_i4;DN127139_c0_g2_i1;DN127139_c0_g2_i2;DN127849_c1_g4_i3;DN127849_c1_g4_i4;DN127849_c1_g5_i2;	DN127139_c0_g2_i1;DN127139_c0_g2_i2;	DN106153_c0_g1_i1;DN113690_c0_g1_i1;DN117299_c2_g2_i18;DN125656_c1_g1_i4;DN127849_c1_g4_i3;DN127849_c1_g4_i4;DN127849_c1_g5_i2;
ko05110	Vibrio cholerae infection	9/1083	Human Diseases; Infectious diseases: Bacterial	KEGG link	9	DN112748_c0_g1_i1;DN118354_c1_g1_i1;DN118669_c1_g3_i1;DN126049_c0_g1_i1;DN127182_c3_g1_i4;DN127818_c2_g2_i4;DN131056_c0_g1_i1;DN13234_c0_g1_i1;DN49078_c0_g1_i1;	;	DN112748_c0_g1_i1;DN118354_c1_g1_i1;DN118669_c1_g3_i1;DN126049_c0_g1_i1;DN127182_c3_g1_i4;DN127818_c2_g2_i4;DN131056_c0_g1_i1;DN13234_c0_g1_i1;DN49078_c0_g1_i1;

Pathway ID	Description	Gene Ratio	PathwayClass	KEGG link	TargetGene	GeneList	geneUp	geneDown
ko05034	Alcoholism	9/1083	Human Diseases; Substance dependence	KEGG link	9	DN108183_c0_g1_i1;DN115243_c1_g1_i4;DN115243_c1_g1_i5;DN124625_c0_g2_i9;DN126198_c0_g1_i14;DN128767_c7_g2_i1;DN79193_c0_g1_i1;DN90169_c0_g1_i1;DN91198_c0_g1_i1;	DN108183_c0_g1_i1;DN124625_c0_g2_i9;	DN115243_c1_g1_i4;DN115243_c1_g1_i5;DN126198_c0_g1_i14;DN128767_c7_g2_i1;DN79193_c0_g1_i1;DN90169_c0_g1_i1;DN91198_c0_g1_i1;
ko04724	Glutamatergic synapse	9/1083	Organismal Systems; Nervous system	KEGG link	9	DN114384_c1_g1_i1;DN114384_c1_g1_i3;DN117349_c0_g4_i1;DN117349_c0_g7_i3;DN117349_c0_g7_i8;DN118017_c1_g1_i2;DN119632_c0_g1_i1;DN120346_c0_g1_i7;DN126155_c2_g6_i1;	DN114384_c1_g1_i1;DN114384_c1_g1_i3;DN118017_c1_g1_i2;DN120346_c0_g1_i7;	DN117349_c0_g4_i1;DN117349_c0_g7_i3;DN117349_c0_g7_i8;DN119632_c0_g1_i1;DN126155_c2_g6_i1;
ko04810	Regulation of actin cytoskeleton	9/1083	Cellular Processes; Cell motility	KEGG link	9	DN113227_c0_g1_i4;DN115243_c1_g1_i4;DN115243_c1_g1_i5;DN116020_c4_g2_i1;DN124625_c0_g2_i9;DN126049_c0_g1_i1;DN128870_c4_g2_i3;DN131056_c0_g1_i1;DN13234_c0_g1_i1;	DN124625_c0_g2_i9;	DN113227_c0_g1_i4;DN115243_c1_g1_i4;DN115243_c1_g1_i5;DN116020_c4_g2_i1;DN126049_c0_g1_i1;DN128870_c4_g2_i3;DN131056_c0_g1_i1;DN13234_c0_g1_i1;
ko00960	Tropane, piperidine and pyridi	9/1083	Metabolism; Biosynthesis of other secondary	KEGG link	9	DN118246_c0_g2_i1;DN119009_c0_g1_i2;DN123784_c4_g1_i4;DN123784_c4_g1_i5;DN123784_c4_g1_i6;DN125946_c2_g1_i5;DN126422_c3_g2_i5;DN128952_c2_g1_i2;DN129116_c5_g1_i1;	DN123784_c4_g1_i4;DN123784_c4_g1_i5;DN123784_c4_g1_i6;DN125946_c2_g1_i5;DN126422_c3_g2_i5;	DN118246_c0_g2_i1;DN119009_c0_g1_i2;DN128952_c2_g1_i2;DN129116_c5_g1_i1;

Pathway ID	Description	Gene Ratio	PathwayClass	KEGG link	TargetGene	GeneList	geneUp	geneDown
	ne alkaloid biosynthesis		metabolites					
ko04210	Apoptosis	9/1083	Cellular Processes; Cell growth and death	KEGG link	9	DN114856_c0_g2_i1;DN119004_c0_g7_i1;DN120026_c3_g1_i3;DN122612_c1_g1_i4;DN126049_c0_g1_i1;DN127489_c0_g2_i3;DN127489_c0_g2_i7;DN131056_c0_g1_i1;DN13234_c0_g1_i1;	DN122612_c1_g1_i4;	DN114856_c0_g2_i1;DN119004_c0_g7_i1;DN120026_c3_g1_i3;DN126049_c0_g1_i1;DN127489_c0_g2_i3;DN127489_c0_g2_i7;DN131056_c0_g1_i1;DN13234_c0_g1_i1;
ko03060	Protein export	9/1083	Genetic Information Processing; Folding, sorting and degradation	KEGG link	9	DN103751_c0_g1_i1;DN112748_c0_g1_i1;DN116859_c1_g1_i19;DN120559_c0_g3_i3;DN122030_c2_g2_i1;DN123880_c0_g1_i1;DN128213_c1_g1_i10;DN128213_c1_g1_i11;DN128213_c1_g1_i5;	DN116859_c1_g1_i19;DN120559_c0_g3_i3;DN122030_c2_g2_i1;DN123880_c0_g1_i1;DN128213_c1_g1_i10;DN128213_c1_g1_i11;DN128213_c1_g1_i5;	DN103751_c0_g1_i1;DN112748_c0_g1_i1;

Pathway ID	Description	Gene Ratio	PathwayClass	KEGG link	TargetGene	GeneList	geneUp	geneDown
ko04261	Adrenergic signaling in cardiomyocytes	8/1083	Organismal Systems; Circulatory system	KEGG link	8	DN115243_c1_g1_i4;DN115243_c1_g1_i5;DN118058_c0_g3_i1;DN118058_c0_g4_i1;DN123205_c4_g2_i7;DN124625_c0_g2_i9;DN125626_c0_g3_i12;DN128767_c7_g2_i1;	DN123205_c4_g2_i7;DN124625_c0_g2_i9;DN125626_c0_g3_i12;	DN115243_c1_g1_i4;DN115243_c1_g1_i5;DN118058_c0_g3_i1;DN118058_c0_g4_i1;DN128767_c7_g2_i1;
ko04530	Tight junction	8/1083	Cellular Processes; Cellular community - eukaryotes	KEGG link	8	DN114856_c0_g2_i1;DN117551_c6_g6_i6;DN123205_c4_g2_i7;DN123480_c2_g1_i1;DN125626_c0_g3_i12;DN126049_c0_g1_i1;DN131056_c0_g1_i1;DN13234_c0_g1_i1;	DN117551_c6_g6_i6;DN123205_c4_g2_i7;DN123480_c2_g1_i1;DN125626_c0_g3_i12;	DN114856_c0_g2_i1;DN126049_c0_g1_i1;DN131056_c0_g1_i1;DN13234_c0_g1_i1;
ko04723	Retrograde endocannabinoid signaling	8/1083	Organismal Systems; Nervous system	KEGG link	8	DN104612_c0_g2_i1;DN118105_c1_g3_i3;DN118105_c1_g3_i4;DN118851_c4_g2_i14;DN118851_c4_g2_i9;DN120376_c0_g9_i4;DN120376_c0_g9_i6;DN122063_c1_g1_i9;	DN118851_c4_g2_i14;DN118851_c4_g2_i9;DN122063_c1_g1_i9;	DN104612_c0_g2_i1;DN118105_c1_g3_i3;DN118105_c1_g3_i4;DN120376_c0_g9_i4;DN120376_c0_g9_i6;

Pathway ID	Description	Gene Ratio	PathwayClass	KEGG link	TargetGene	GeneList	geneUp	geneDown
ko04657	IL-17 signaling pathway	8/1083	Organismal Systems; Immune system	KEGG link	8	DN121311_c2_g1_i2;DN121311_c2_g1_i5;DN121920_c1_g1_i1;DN121920_c1_g1_i5;DN122911_c1_g1_i1;DN127764_c0_g3_i2;DN128341_c3_g5_i2;DN92669_c0_g1_i1;	DN121311_c2_g1_i2;DN121311_c2_g1_i5;DN121920_c1_g1_i1;DN121920_c1_g1_i5;DN122911_c1_g1_i1;DN127764_c0_g3_i2;DN128341_c3_g5_i2;	DN92669_c0_g1_i1;
ko04024	cAMP signaling pathway	8/1083	Environmental Information Processing; Signal transduction	KEGG link	8	DN115243_c1_g1_i4;DN115243_c1_g1_i5;DN118017_c1_g1_i2;DN119207_c3_g2_i1;DN119207_c3_g2_i9;DN124625_c0_g2_i9;DN126155_c2_g6_i1;DN128767_c7_g2_i1;	DN118017_c1_g1_i2;DN119207_c3_g2_i1;DN119207_c3_g2_i9;DN124625_c0_g2_i9;	DN115243_c1_g1_i4;DN115243_c1_g1_i5;DN126155_c2_g6_i1;DN128767_c7_g2_i1;
ko00730	Thiamine metabolism	7/1083	Metabolism; Metabolism of cofactors and vitamins	KEGG link	7	DN115194_c0_g10_i1;DN115194_c0_g10_i2;DN120547_c0_g1_i1;DN120547_c0_g1_i7;DN122471_c0_g1_i10;DN122471_c0_g1_i11;DN122471_c0_g1_i2;	DN115194_c0_g10_i1;DN115194_c0_g10_i2;DN120547_c0_g1_i1;DN120547_c0_g1_i7;DN122471_c0_g1_i10;DN122471_c0_g1_i11;DN122471_c0_g1_i2;	;
ko04961	Endocrine and other	7/1083	Organismal Systems; Excretory	KEGG link	7	DN111858_c0_g1_i2;DN113019_c1_g1_i4;DN113019_c1_g1_i5;DN118948_c1_g4_i1;DN122213_c0_g2_i1;DN128828_c1_g1_i3;DN128828_c1	DN111858_c0_g1_i2;DN113019_c1_g1_i4;DN113019_c1_g1_i5;DN122213_c0_g2_i1;DN128828_c1_g1_i7;	DN118948_c1_g4_i1;DN128828_c1_g1_i3;

Pathway ID	Description	Gene Ratio	PathwayClass	KEGG link	TargetGene	GeneList	geneUp	geneDown
	factor-regulated calcium reabsorption		system			_g1_i7;		
ko05130	Pathogenic Escherichia coli infection	7/1083	Human Diseases; Infectious diseases: Bacterial	KEGG link	7	DN114856_c0_g2_i1;DN116315_c2_g1_i2;DN120827_c1_g3_i1;DN123945_c0_g2_i1;DN126049_c0_g1_i1;DN131056_c0_g1_i1;DN13234_c0_g1_i1;	DN120827_c1_g3_i1;DN123945_c0_g2_i1;	DN114856_c0_g2_i1;DN116315_c2_g1_i2;DN126049_c0_g1_i1;DN131056_c0_g1_i1;DN13234_c0_g1_i1;
ko00945	Stilbenoid, diarylheptanoid and gingerol biosynthesis	7/1083	Metabolism; Biosynthesis of other secondary metabolites	KEGG link	7	DN115984_c0_g1_i6;DN117404_c0_g1_i1;DN117839_c2_g3_i3;DN123921_c2_g2_i1;DN125074_c5_g2_i10;DN125074_c5_g2_i3;DN127959_c3_g1_i5;	DN117404_c0_g1_i1;	DN115984_c0_g1_i6;DN117839_c2_g3_i3;DN123921_c2_g2_i1;DN125074_c5_g2_i10;DN125074_c5_g2_i3;DN127959_c3_g1_i5;

Pathway ID	Description	Gene Ratio	PathwayClass	KEGG link	TargetGene	GeneList	geneUp	geneDown
ko04150	mTOR signaling pathway	7/1083	Environmental Information Processing; Signal transduction	KEGG link	7	DN109405_c0_g1_i1;DN117551_c6_g6_i6;DN118669_c1_g3_i1;DN120304_c1_g1_i1;DN126236_c2_g2_i14;DN126236_c2_g2_i8;DN127818_c2_g2_i4;	DN117551_c6_g6_i6;DN120304_c1_g1_i1;	DN109405_c0_g1_i1;DN118669_c1_g3_i1;DN126236_c2_g2_i14;DN126236_c2_g2_i8;DN127818_c2_g2_i4;
ko05416	Viral myocarditis	7/1083	Human Diseases; Cardiovascular diseases	KEGG link	7	DN118058_c0_g3_i1;DN118058_c0_g4_i1;DN126049_c0_g1_i1;DN127489_c0_g2_i3;DN127489_c0_g2_i7;DN131056_c0_g1_i1;DN13234_c0_g1_i1;	;	DN118058_c0_g3_i1;DN118058_c0_g4_i1;DN126049_c0_g1_i1;DN127489_c0_g2_i3;DN127489_c0_g2_i7;DN131056_c0_g1_i1;DN13234_c0_g1_i1;
ko00565	Ether lipid metabolism	7/1083	Metabolism; Lipid metabolism	KEGG link	7	DN110344_c0_g1_i1;DN112958_c0_g1_i1;DN118017_c1_g1_i2;DN122154_c0_g1_i2;DN126155_c2_g6_i1;DN127115_c2_g2_i1;DN128395_c1_g1_i1;	DN118017_c1_g1_i2;DN122154_c0_g1_i2;	DN110344_c0_g1_i1;DN112958_c0_g1_i1;DN126155_c2_g6_i1;DN127115_c2_g2_i1;DN128395_c1_g1_i1;
ko05215	Prostate cancer	7/1083	Human Diseases; Cancers: Specific types	KEGG link	7	DN121311_c2_g1_i2;DN121311_c2_g1_i5;DN121920_c1_g1_i1;DN121920_c1_g1_i5;DN127764_c0_g3_i2;DN128341_c3_g5_i2;DN92669_c0_g1_i1;	DN121311_c2_g1_i2;DN121311_c2_g1_i5;DN121920_c1_g1_i1;DN121920_c1_g1_i5;DN127764_c0_g3_i2;DN128341_c3_g5_i2;	DN92669_c0_g1_i1;

Pathway ID	Description	Gene Ratio	PathwayClass	KEGG link	TargetGene	GeneList	geneUp	geneDown
ko00073	Cutin, suberine and wax biosynthesis	7/1083	Metabolism; Lipid metabolism	KEGG link	7	DN116486_c0_g1_i3;DN119070_c1_g1_i1;DN119070_c1_g1_i2;DN127148_c1_g10_i4;DN127998_c1_g1_i1;DN127998_c1_g3_i2;DN77198_c0_g1_i2;	DN116486_c0_g1_i3;DN119070_c1_g1_i1;DN119070_c1_g1_i2;DN127148_c1_g10_i4;	DN127998_c1_g1_i1;DN127998_c1_g3_i2;DN77198_c0_g1_i2;
ko05231	Choline metabolism in cancer	7/1083	Human Diseases; Cancers: Overview	KEGG link	7	DN113227_c0_g1_i4;DN118017_c1_g1_i2;DN119056_c0_g3_i5;DN119776_c1_g6_i1;DN125127_c0_g2_i5;DN126155_c2_g6_i1;DN128244_c6_g1_i3;	DN118017_c1_g1_i2;DN119056_c0_g3_i5;DN119776_c1_g6_i1;	DN113227_c0_g1_i4;DN125127_c0_g2_i5;DN126155_c2_g6_i1;DN128244_c6_g1_i3;
ko04659	Th17 cell differentiation	7/1083	Organismal Systems; Immune system	KEGG link	7	DN120346_c0_g1_i7;DN121311_c2_g1_i2;DN121311_c2_g1_i5;DN121920_c1_g1_i1;DN121920_c1_g1_i5;DN127764_c0_g3_i2;DN92669_c0_g1_i1;	DN120346_c0_g1_i7;DN121311_c2_g1_i2;DN121311_c2_g1_i5;DN121920_c1_g1_i1;DN121920_c1_g1_i5;DN127764_c0_g3_i2;	DN92669_c0_g1_i1;
ko04072	Phospholipase D signaling pathway	7/1083	Environmental Information Processing; Signal transducti	KEGG link	7	DN113227_c0_g1_i4;DN118017_c1_g1_i2;DN119056_c0_g3_i5;DN119776_c1_g6_i1;DN125127_c0_g2_i5;DN126155_c2_g6_i1;DN128244_c6_g1_i3;	DN118017_c1_g1_i2;DN119056_c0_g3_i5;DN119776_c1_g6_i1;	DN113227_c0_g1_i4;DN125127_c0_g2_i5;DN126155_c2_g6_i1;DN128244_c6_g1_i3;

Pathway ID	Description	Gene Ratio	PathwayClass	KEGG link	TargetGene	GeneList	geneUp	geneDown
			on					
ko05323	Rheumatoid arthritis	7/1083	Human Diseases; Immune diseases	KEGG link	7	DN118354_c1_g1_i1;DN118669_c1_g3_i1;DN119004_c0_g7_i1;DN119880_c0_g6_i1;DN127182_c3_g1_i4;DN127818_c2_g2_i4;DN49078_c0_g1_i1;	;	DN118354_c1_g1_i1;DN118669_c1_g3_i1;DN119004_c0_g7_i1;DN119880_c0_g6_i1;DN127182_c3_g1_i4;DN127818_c2_g2_i4;DN49078_c0_g1_i1;
ko00908	Zeatin biosynthesis	7/1083	Metabolism; Metabolism of terpenoids and polyketides	KEGG link	7	DN116583_c1_g3_i1;DN116785_c0_g1_i1;DN119092_c4_g1_i1;DN121693_c0_g5_i2;DN124391_c2_g10_i1;DN125951_c0_g4_i3;DN126602_c2_g2_i1;	;	DN116583_c1_g3_i1;DN116785_c0_g1_i1;DN119092_c4_g1_i1;DN121693_c0_g5_i2;DN124391_c2_g10_i1;DN125951_c0_g4_i3;DN126602_c2_g2_i1;
ko04712	Circadian rhythm - plant	7/1083	Organismal Systems; Environmental adaptation	KEGG link	7	DN122252_c2_g1_i3;DN122252_c2_g1_i6;DN125822_c1_g1_i7;DN125998_c0_g2_i2;DN127560_c3_g17_i1;DN127560_c3_g3_i1;DN127560_c3_g3_i2;	DN125822_c1_g1_i7;DN125998_c0_g2_i2;	DN122252_c2_g1_i3;DN122252_c2_g1_i6;DN127560_c3_g17_i1;DN127560_c3_g3_i1;DN127560_c3_g3_i2;

Pathway ID	Description	Gene Ratio	PathwayClass	KEGG link	TargetGene	GeneList	geneUp	geneDown
ko04611	Platelet activation	6/1083	Organismal Systems; Immune system	KEGG link	6	DN115243_c1_g1_i4;DN115243_c1_g1_i5;DN124625_c0_g2_i9;DN126049_c0_g1_i1;DN131056_c0_g1_i1;DN13234_c0_g1_i1;	DN124625_c0_g2_i9;	DN115243_c1_g1_i4;DN115243_c1_g1_i5;DN126049_c0_g1_i1;DN131056_c0_g1_i1;DN13234_c0_g1_i1;
ko00380	Tryptophan metabolism	6/1083	Metabolism; Amino acid metabolism	KEGG link	6	DN121980_c1_g1_i1;DN121980_c1_g1_i6;DN122576_c1_g3_i3;DN123388_c2_g4_i10;DN124716_c1_g3_i5;DN124836_c1_g2_i1;	DN124716_c1_g3_i5;DN124836_c1_g2_i1;	DN121980_c1_g1_i1;DN121980_c1_g1_i6;DN122576_c1_g3_i3;DN123388_c2_g4_i10;
ko04940	Type I diabetes mellitus	6/1083	Human Diseases; Endocrine and metabolic diseases	KEGG link	6	DN126700_c3_g2_i3;DN126841_c0_g1_i3;DN126841_c0_g2_i2;DN126841_c0_g2_i3;DN127046_c4_g1_i2;DN127046_c4_g1_i3;	DN126700_c3_g2_i3;DN126841_c0_g1_i3;DN126841_c0_g2_i2;DN126841_c0_g2_i3;DN127046_c4_g1_i2;DN127046_c4_g1_i3;	;
ko01040	Biosynthesis of unsaturated fatty acids	6/1083	Metabolism; Lipid metabolism	KEGG link	6	DN119207_c3_g2_i1;DN119207_c3_g2_i9;DN119487_c1_g2_i10;DN119487_c1_g2_i8;DN120560_c1_g2_i4;DN123630_c1_g1_i9;	DN119207_c3_g2_i1;DN119207_c3_g2_i9;DN119487_c1_g2_i10;DN120560_c1_g2_i4;DN123630_c1_g1_i9;	DN119487_c1_g2_i8;

Pathway ID	Description	Gene Ratio	PathwayClass	KEGG link	TargetGene	GeneList	geneUp	geneDown
ko03070	Bacterial secretion system	6/1083	Environmental Information Processing; Membrane transport	KEGG link	6	DN120559_c0_g3_i3;DN122030_c2_g2_i1;DN123880_c0_g1_i1;DN128213_c1_g1_i10;DN128213_c1_g1_i11;DN128213_c1_g1_i5;	DN120559_c0_g3_i3;DN122030_c2_g2_i1;DN123880_c0_g1_i1;DN128213_c1_g1_i10;DN128213_c1_g1_i11;DN128213_c1_g1_i5;	;
ko04920	Adipocytokine signaling pathway	6/1083	Organismal Systems; Endocrine system	KEGG link	6	DN117551_c6_g6_i6;DN127497_c4_g1_i10;DN127497_c4_g1_i12;DN127497_c4_g1_i8;DN127501_c0_g1_i2;DN127501_c0_g1_i3;	DN117551_c6_g6_i6;DN127497_c4_g1_i12;	DN127497_c4_g1_i10;DN127497_c4_g1_i8;DN127501_c0_g1_i2;DN127501_c0_g1_i3;
ko04728	Dopaminergic synapse	6/1083	Organismal Systems; Nervous system	KEGG link	6	DN115243_c1_g1_i4;DN115243_c1_g1_i5;DN123205_c4_g2_i7;DN124625_c0_g2_i9;DN125626_c0_g3_i12;DN128767_c7_g2_i1;	DN123205_c4_g2_i7;DN124625_c0_g2_i9;DN125626_c0_g3_i12;	DN115243_c1_g1_i4;DN115243_c1_g1_i5;DN128767_c7_g2_i1;
ko00450	Selenocompound metab	6/1083	Metabolism; Metabolism of other	KEGG link	6	DN114093_c5_g1_i2;DN123754_c4_g1_i6;DN126749_c1_g1_i2;DN126749_c1_g1_i3;DN126749_c1_g1_i6;DN128295_c0_g3_i7;	DN128295_c0_g3_i7;	DN114093_c5_g1_i2;DN123754_c4_g1_i6;DN126749_c1_g1_i2;DN126749_c1_g1_i3;DN126749_c1_g1_i6;

Pathway ID	Description	Gene Ratio	PathwayClass	KEGG link	TargetGene	GeneList	geneUp	geneDown
	olism		amino acids					
ko00625	Chloroalkane and chloroalkene degradation	6/1083	Metabolism; Xenobiotics biodegradation and metabolism	KEGG link	6	DN119057_c0_g6_i15;DN119057_c0_g6_i21;DN119057_c0_g6_i29;DN121980_c1_g1_i1;DN121980_c1_g1_i6;DN123388_c2_g4_i10;	DN119057_c0_g6_i15;DN119057_c0_g6_i21;DN119057_c0_g6_i29;	DN121980_c1_g1_i1;DN121980_c1_g1_i6;DN123388_c2_g4_i10;
ko04914	Progesterone-mediated oocyte maturation	6/1083	Organismal Systems; Endocrine system	KEGG link	6	DN121311_c2_g1_i2;DN121311_c2_g1_i5;DN121920_c1_g1_i1;DN121920_c1_g1_i5;DN127764_c0_g3_i2;DN92669_c0_g1_i1;	DN121311_c2_g1_i2;DN121311_c2_g1_i5;DN121920_c1_g1_i1;DN121920_c1_g1_i5;DN127764_c0_g3_i2;	DN92669_c0_g1_i1;
ko01523	Antifolate resistance	6/1083	Human Diseases; Drug resistance: Antineopl	KEGG link	6	DN123920_c0_g1_i1;DN124509_c1_g1_i1;DN126107_c1_g4_i3;DN128061_c0_g1_i3;DN128241_c5_g1_i15;DN128241_c5_g1_i9;	DN123920_c0_g1_i1;DN128241_c5_g1_i15;DN128241_c5_g1_i9;	DN124509_c1_g1_i1;DN126107_c1_g4_i3;DN128061_c0_g1_i3;

Pathway ID	Description	Gene Ratio	PathwayClass	KEGG link	TargetGene	GeneList	geneUp	geneDown
			astic					
ko04068	FoxO signaling pathway	6/1083	Environmental Information Processing; Signal transduction	KEGG link	6	DN117551_c6_g6_i6;DN120304_c1_g1_i1;DN121802_c0_g3_i3;DN121802_c0_g3_i4;DN124836_c1_g2_i1;DN124924_c1_g1_i1;	DN117551_c6_g6_i6;DN120304_c1_g1_i1;DN121802_c0_g3_i3;DN124836_c1_g2_i1;DN124924_c1_g1_i1;	DN121802_c0_g3_i4;
ko05100	Bacterial invasion of epithelial cells	6/1083	Human Diseases; Infectious diseases: Bacterial	KEGG link	6	DN113019_c1_g1_i4;DN113019_c1_g1_i5;DN122213_c0_g2_i1;DN126049_c0_g1_i1;DN131056_c0_g1_i1;DN13234_c0_g1_i1;	DN113019_c1_g1_i4;DN113019_c1_g1_i5;DN122213_c0_g2_i1;	DN126049_c0_g1_i1;DN131056_c0_g1_i1;DN13234_c0_g1_i1;
ko04071	Sphingolipid signaling pathw	6/1083	Environmental Information Processing; Signal	KEGG link	6	DN118017_c1_g1_i2;DN122899_c1_g3_i2;DN122899_c1_g3_i5;DN123205_c4_g2_i7;DN125626_c0_g3_i12;DN126155_c2_g6_i1;	DN118017_c1_g1_i2;DN123205_c4_g2_i7;DN125626_c0_g3_i12;	DN122899_c1_g3_i2;DN122899_c1_g3_i5;DN126155_c2_g6_i1;

Pathway ID	Description	Gene Ratio	PathwayClass	KEGG link	TargetGene	GeneList	geneUp	geneDown
	ay		transduction					
ko04510	Focal adhesion	6/1083	Cellular Processes; Cellular community - eukaryotes	KEGG link	6	DN115243_c1_g1_i4;DN115243_c1_g1_i5;DN124625_c0_g2_i9;DN126049_c0_g1_i1;DN131056_c0_g1_i1;DN13234_c0_g1_i1;	DN124625_c0_g2_i9;	DN115243_c1_g1_i4;DN115243_c1_g1_i5;DN126049_c0_g1_i1;DN131056_c0_g1_i1;DN13234_c0_g1_i1;
ko04310	Wnt signaling pathway	6/1083	Environmental Information Processing; Signal transduction	KEGG link	6	DN120346_c0_g1_i7;DN121764_c2_g1_i6;DN121802_c0_g3_i3;DN121802_c0_g3_i4;DN122392_c2_g2_i1;DN123359_c1_g2_i6;	DN120346_c0_g1_i7;DN121764_c2_g1_i6;DN121802_c0_g3_i3;	DN121802_c0_g3_i4;DN122392_c2_g2_i1;DN123359_c1_g2_i6;
ko04136	Autophagy - other	5/1083	Cellular Processes; Transport and catabolis	KEGG link	5	DN123205_c4_g2_i7;DN124918_c3_g3_i7;DN125626_c0_g3_i12;DN127630_c1_g7_i1;DN127630_c1_g7_i7;	DN123205_c4_g2_i7;DN125626_c0_g3_i12;DN127630_c1_g7_i1;	DN124918_c3_g3_i7;DN127630_c1_g7_i7;

Pathway ID	Description	Gene Ratio	PathwayClass	KEGG link	TargetGene	GeneList	geneUp	geneDown
			m					
ko00100	Steroid biosynthesis	5/1083	Metabolism; Lipid metabolism	KEGG link	5	DN122154_c0_g1_i2;DN125071_c0_g1_i13;DN125071_c0_g1_i5;DN125668_c0_g2_i10;DN128244_c4_g1_i1;	DN122154_c0_g1_i2;DN125071_c0_g1_i13;DN125071_c0_g1_i5;DN128244_c4_g1_i1;	DN125668_c0_g2_i10;
ko00521	Streptomycin biosynthesis	5/1083	Metabolism; Biosynthesis of other secondary metabolites	KEGG link	5	DN102195_c0_g2_i1;DN122358_c2_g1_i3;DN122358_c2_g1_i9;DN123080_c2_g3_i2;DN123921_c3_g2_i1;	DN122358_c2_g1_i3;DN122358_c2_g1_i9;DN123080_c2_g3_i2;	DN102195_c0_g2_i1;DN123921_c3_g2_i1;
ko00290	Valine, leucine and isoleucine biosynthesis	5/1083	Metabolism; Amino acid metabolism	KEGG link	5	DN116773_c0_g1_i5;DN122597_c0_g2_i3;DN122597_c0_g3_i1;DN126982_c2_g1_i2;DN128195_c1_g1_i3;	DN116773_c0_g1_i5;DN122597_c0_g2_i3;DN122597_c0_g3_i1;DN126982_c2_g1_i2;	DN128195_c1_g1_i3;

Pathway ID	Description	Gene Ratio	PathwayClass	KEGG link	TargetGene	GeneList	geneUp	geneDown
ko00830	Retinol metabolism	5/1083	Metabolism; Metabolism of cofactors and vitamins	KEGG link	5	DN106927_c0_g1_i1;DN119057_c0_g6_i15;DN119057_c0_g6_i21;DN119057_c0_g6_i29;DN124052_c1_g4_i6;	DN119057_c0_g6_i15;DN119057_c0_g6_i21;DN119057_c0_g6_i29;	DN106927_c0_g1_i1;DN124052_c1_g4_i6;
ko04922	Glucagon signaling pathway	5/1083	Organismal Systems; Endocrine system	KEGG link	5	DN117551_c6_g6_i6;DN120346_c0_g1_i7;DN127571_c3_g2_i5;DN127595_c2_g1_i1;DN128767_c7_g2_i1;	DN117551_c6_g6_i6;DN120346_c0_g1_i7;	DN127571_c3_g2_i5;DN127595_c2_g1_i1;DN128767_c7_g2_i1;
ko04113	Meiosis - yeast	5/1083	Cellular Processes; Cell growth and death	KEGG link	5	DN115243_c1_g1_i4;DN115243_c1_g1_i5;DN123205_c4_g2_i7;DN124625_c0_g2_i9;DN125626_c0_g3_i12;	DN123205_c4_g2_i7;DN124625_c0_g2_i9;DN125626_c0_g3_i12;	DN115243_c1_g1_i4;DN115243_c1_g1_i5;
ko04720	Long-term potentiation	5/1083	Organismal Systems; Nervous system	KEGG link	5	DN115243_c1_g1_i4;DN115243_c1_g1_i5;DN120346_c0_g1_i7;DN124625_c0_g2_i9;DN128767_c7_g2_i1;	DN120346_c0_g1_i7;DN124625_c0_g2_i9;	DN115243_c1_g1_i4;DN115243_c1_g1_i5;DN128767_c7_g2_i1;

Pathway ID	Description	Gene Ratio	PathwayClass	KEGG link	TargetGene	GeneList	geneUp	geneDown
ko04270	Vascular smooth muscle contraction	5/1083	Organismal Systems; Circulatory system	KEGG link	5	DN115243_c1_g1_i4;DN115243_c1_g1_i5;DN124625_c0_g2_i9;DN127115_c2_g2_i1;DN128767_c7_g2_i1;	DN124625_c0_g2_i9;	DN115243_c1_g1_i4;DN115243_c1_g1_i5;DN127115_c2_g2_i1;DN128767_c7_g2_i1;
ko00600	Sphingolipid metabolism	5/1083	Metabolism; Lipid metabolism	KEGG link	5	DN118842_c0_g1_i11;DN122031_c1_g2_i2;DN122337_c3_g3_i3;DN122899_c1_g3_i2;DN122899_c1_g3_i5;	;	DN118842_c0_g1_i11;DN122031_c1_g2_i2;DN122337_c3_g3_i3;DN122899_c1_g3_i2;DN122899_c1_g3_i5;
ko03320	PPAR signaling pathway	5/1083	Organismal Systems; Endocrine system	KEGG link	5	DN119207_c3_g2_i1;DN119207_c3_g2_i9;DN127497_c4_g1_i10;DN127497_c4_g1_i12;DN127497_c4_g1_i8;	DN119207_c3_g2_i1;DN119207_c3_g2_i9;DN127497_c4_g1_i12;	DN127497_c4_g1_i10;DN127497_c4_g1_i8;
ko04022	cGMP-PKG signaling pathway	5/1083	Environmental Information Processing; Signal	KEGG link	5	DN115243_c1_g1_i4;DN115243_c1_g1_i5;DN120346_c0_g1_i7;DN124625_c0_g2_i9;DN128767_c7_g2_i1;	DN120346_c0_g1_i7;DN124625_c0_g2_i9;	DN115243_c1_g1_i4;DN115243_c1_g1_i5;DN128767_c7_g2_i1;

Pathway ID	Description	Gene Ratio	PathwayClass	KEGG link	TargetGene	GeneList	geneUp	geneDown
			transduction					
ko00062	Fatty acid elongation	5/1083	Metabolism; Lipid metabolism	KEGG link	5	DN119650_c5_g1_i2;DN120331_c0_g1_i1;DN120560_c1_g2_i4;DN127698_c3_g1_i1;DN127698_c3_g1_i2;	DN119650_c5_g1_i2;DN120560_c1_g2_i4;DN127698_c3_g1_i1;DN127698_c3_g1_i2;	DN120331_c0_g1_i1;
ko05120	Epithelial cell signaling in Helicobacter pylori infection	5/1083	Human Diseases; Infectious diseases: Bacterial	KEGG link	5	DN118354_c1_g1_i1;DN118669_c1_g3_i1;DN127182_c3_g1_i4;DN127818_c2_g2_i4;DN49078_c0_g1_i1;	;	DN118354_c1_g1_i1;DN118669_c1_g3_i1;DN127182_c3_g1_i4;DN127818_c2_g2_i4;DN49078_c0_g1_i1;
ko04015	Rap1 signaling pathway	5/1083	Environmental Information Processing; Signal	KEGG link	5	DN116020_c4_g2_i1;DN126049_c0_g1_i1;DN128767_c7_g2_i1;DN131056_c0_g1_i1;DN13234_c0_g1_i1;	;	DN116020_c4_g2_i1;DN126049_c0_g1_i1;DN128767_c7_g2_i1;DN131056_c0_g1_i1;DN13234_c0_g1_i1;

Pathway ID	Description	Gene Ratio	PathwayClass	KEGG link	TargetGene	GeneList	geneUp	geneDown
			transduction					
ko04966	Collecting duct acid secretion	5/1083	Organismal Systems; Excretory system	KEGG link	5	DN118354_c1_g1_i1;DN118669_c1_g3_i1;DN127182_c3_g1_i4;DN127818_c2_g2_i4;DN49078_c0_g1_i1;	;	DN118354_c1_g1_i1;DN118669_c1_g3_i1;DN127182_c3_g1_i4;DN127818_c2_g2_i4;DN49078_c0_g1_i1;
ko04260	Cardiac muscle contraction	5/1083	Organismal Systems; Circulatory system	KEGG link	5	DN109738_c0_g1_i1;DN115634_c0_g2_i1;DN118058_c0_g3_i1;DN118058_c0_g4_i1;DN119966_c0_g1_i2;	;	DN109738_c0_g1_i1;DN115634_c0_g2_i1;DN118058_c0_g3_i1;DN118058_c0_g4_i1;DN119966_c0_g1_i2;
ko04137	Mitophagy - animal	5/1083	Cellular Processes; Transport and catabolism	KEGG link	5	DN127616_c4_g1_i1;DN127616_c4_g1_i10;DN127616_c4_g1_i2;DN127616_c4_g1_i5;DN128963_c1_g1_i5;	DN127616_c4_g1_i1;DN127616_c4_g1_i10;DN127616_c4_g1_i2;DN127616_c4_g1_i5;DN128963_c1_g1_i5;	;

Pathway ID	Description	Gene Ratio	PathwayClass	KEGG link	TargetGene	GeneList	geneUp	geneDown
ko04111	Cell cycle - yeast	5/1083	Cellular Processes; Cell growth and death	KEGG link	5	DN109259_c0_g1_i2;DN111348_c0_g1_i3;DN122415_c1_g1_i1;DN123205_c4_g2_i7;DN125626_c0_g3_i12;	DN109259_c0_g1_i2;DN111348_c0_g1_i3;DN123205_c4_g2_i7;DN125626_c0_g3_i12;	DN122415_c1_g1_i1;
ko05031	Amphetamine addiction	5/1083	Human Diseases; Substance dependence	KEGG link	5	DN115243_c1_g1_i4;DN115243_c1_g1_i5;DN120346_c0_g1_i7;DN124625_c0_g2_i9;DN128767_c7_g2_i1;	DN120346_c0_g1_i7;DN124625_c0_g2_i9;	DN115243_c1_g1_i4;DN115243_c1_g1_i5;DN128767_c7_g2_i1;
ko04013	MAPK signaling pathway - fly	5/1083	Environmental Information Processing; Signal transduction	KEGG link	5	DN107826_c1_g1_i6;DN116020_c4_g2_i1;DN123205_c4_g2_i7;DN125626_c0_g3_i12;DN132218_c0_g1_i1;	DN123205_c4_g2_i7;DN125626_c0_g3_i12;	DN107826_c1_g1_i6;DN116020_c4_g2_i1;DN132218_c0_g1_i1;
ko04745	Phototransduction - fly	4/1083	Organismal Systems; Sensory system	KEGG link	4	DN126049_c0_g1_i1;DN128767_c7_g2_i1;DN131056_c0_g1_i1;DN13234_c0_g1_i1;	;	DN126049_c0_g1_i1;DN128767_c7_g2_i1;DN131056_c0_g1_i1;DN13234_c0_g1_i1;

Pathway ID	Description	Gene Ratio	PathwayClass	KEGG link	TargetGene	GeneList	geneUp	geneDown
ko05033	Nicotine addiction	4/1083	Human Diseases; Substance dependence	KEGG link	4	DN118851_c4_g2_i14;DN118851_c4_g2_i9;DN120376_c0_g9_i4;DN120376_c0_g9_i6;	DN118851_c4_g2_i14;DN118851_c4_g2_i9;	DN120376_c0_g9_i4;DN120376_c0_g9_i6;
ko04115	p53 signaling pathway	4/1083	Cellular Processes; Cell growth and death	KEGG link	4	DN125732_c2_g2_i8;DN125822_c1_g1_i7;DN127489_c0_g2_i3;DN127489_c0_g2_i7;	DN125732_c2_g2_i8;DN125822_c1_g1_i7;	DN127489_c0_g2_i3;DN127489_c0_g2_i7;
ko00983	Drug metabolism - other enzymes	4/1083	Metabolism; Xenobiotics biodegradation and metabolism	KEGG link	4	DN119346_c0_g1_i3;DN120199_c0_g4_i1;DN122614_c3_g3_i6;DN127816_c0_g2_i20;	DN120199_c0_g4_i1;DN122614_c3_g3_i6;DN127816_c0_g2_i20;	DN119346_c0_g1_i3;
ko03440	Homologous recombination	4/1083	Genetic Information Processing; Replicatio	KEGG link	4	DN107177_c0_g1_i2;DN123446_c1_g1_i1;DN124862_c0_g1_i13;DN125968_c2_g5_i14;	DN107177_c0_g1_i2;DN124862_c0_g1_i13;	DN123446_c1_g1_i1;DN125968_c2_g5_i14;

Pathway ID	Description	Gene Ratio	PathwayClass	KEGG link	TargetGene	GeneList	geneUp	geneDown
			n and repair					
ko04710	Circadian rhythm	4/1083	Organismal Systems; Environmental adaptation	KEGG link	4	DN117551_c6_g6_i6;DN117915_c3_g2_i1;DN121802_c0_g3_i3;DN121802_c0_g3_i4;	DN117551_c6_g6_i6;DN121802_c0_g3_i3;	DN117915_c3_g2_i1;DN121802_c0_g3_i4;
ko05131	Shigellosis	4/1083	Human Diseases; Infectious diseases: Bacterial	KEGG link	4	DN116020_c4_g2_i1;DN126049_c0_g1_i1;DN131056_c0_g1_i1;DN13234_c0_g1_i1;	;	DN116020_c4_g2_i1;DN126049_c0_g1_i1;DN131056_c0_g1_i1;DN13234_c0_g1_i1;
ko04933	AGE-RAGE signaling pathway in diabetic compl	4/1083	Human Diseases; Endocrine and metabolic diseases	KEGG link	4	DN126097_c4_g1_i17;DN126097_c4_g1_i6;DN126097_c4_g2_i4;DN127939_c1_g1_i1;	;	DN126097_c4_g1_i17;DN126097_c4_g1_i6;DN126097_c4_g2_i4;DN127939_c1_g1_i1;

Pathway ID	Description	Gene Ratio	PathwayClass	KEGG link	TargetGene	GeneList	geneUp	geneDown
	ications							
ko05322	Systemic lupus erythematosus	4/1083	Human Diseases; Immune diseases	KEGG link	4	DN108183_c0_g1_i1;DN79193_c0_g1_i1;DN90169_c0_g1_i1;DN91198_c0_g1_i1;	DN108183_c0_g1_i1;	DN79193_c0_g1_i1;DN90169_c0_g1_i1;DN91198_c0_g1_i1;
ko05410	Hypertrophic cardiomyopathy (HCM)	4/1083	Human Diseases; Cardiovascular diseases	KEGG link	4	DN117551_c6_g6_i6;DN126049_c0_g1_i1;DN131056_c0_g1_i1;DN13234_c0_g1_i1;	DN117551_c6_g6_i6;	DN126049_c0_g1_i1;DN131056_c0_g1_i1;DN13234_c0_g1_i1;
ko04211	Longevity regulating pathway	4/1083	Organismal Systems; Aging	KEGG link	4	DN117551_c6_g6_i6;DN124836_c1_g2_i1;DN127501_c0_g1_i2;DN127501_c0_g1_i3;	DN117551_c6_g6_i6;DN124836_c1_g2_i1;	DN127501_c0_g1_i2;DN127501_c0_g1_i3;

Pathway ID	Description	Gene Ratio	PathwayClass	KEGG link	TargetGene	GeneList	geneUp	geneDown
ko05222	Small cell lung cancer	4/1083	Human Diseases; Cancers: Specific types	KEGG link	4	DN111348_c0_g1_i3;DN120304_c1_g1_i1;DN127489_c0_g2_i3;DN127489_c0_g2_i7;	DN111348_c0_g1_i3;DN120304_c1_g1_i1;	DN127489_c0_g2_i3;DN127489_c0_g2_i7;
ko05032	Morphine addiction	4/1083	Human Diseases; Substance dependence	KEGG link	4	DN118851_c4_g2_i14;DN118851_c4_g2_i9;DN120376_c0_g9_i4;DN120376_c0_g9_i6;	DN118851_c4_g2_i14;DN118851_c4_g2_i9;	DN120376_c0_g9_i4;DN120376_c0_g9_i6;
ko04014	Ras signaling pathway	4/1083	Environmental Information Processing; Signal transduction	KEGG link	4	DN118017_c1_g1_i2;DN126155_c2_g6_i1;DN127115_c2_g2_i1;DN128767_c7_g2_i1;	DN118017_c1_g1_i2;	DN126155_c2_g6_i1;DN127115_c2_g2_i1;DN128767_c7_g2_i1;
ko04750	Inflammatory mediator regula	4/1083	Organismal Systems; Sensory system	KEGG link	4	DN115243_c1_g1_i4;DN115243_c1_g1_i5;DN124625_c0_g2_i9;DN128767_c7_g2_i1;	DN124625_c0_g2_i9;	DN115243_c1_g1_i4;DN115243_c1_g1_i5;DN128767_c7_g2_i1;

Pathway ID	Description	Gene Ratio	PathwayClass	KEGG link	TargetGene	GeneList	geneUp	geneDown
	tion of TRP channels							
ko00905	Brassinosteroid biosynthesis	4/1083	Metabolism; Metabolism of terpenoids and polyketides	KEGG link	4	DN116413_c0_g1_i2;DN118083_c2_g4_i2;DN122425_c0_g1_i1;DN122425_c0_g1_i4;	DN118083_c2_g4_i2;	DN116413_c0_g1_i2;DN122425_c0_g1_i1;DN122425_c0_g1_i4;
ko04668	TNF signaling pathway	4/1083	Environmental Information Processing; Signal transduction	KEGG link	4	DN127616_c4_g1_i1;DN127616_c4_g1_i10;DN127616_c4_g1_i2;DN127616_c4_g1_i5;	DN127616_c4_g1_i1;DN127616_c4_g1_i10;DN127616_c4_g1_i2;DN127616_c4_g1_i5;	;
ko05014	Amyotrophic lateral	4/1083	Human Diseases; Neurodegenerative	KEGG link	4	DN120346_c0_g1_i7;DN124836_c1_g2_i1;DN127489_c0_g2_i3;DN127489_c0_g2_i7;	DN120346_c0_g1_i7;DN124836_c1_g2_i1;	DN127489_c0_g2_i3;DN127489_c0_g2_i7;

Pathway ID	Description	Gene Ratio	PathwayClass	KEGG link	TargetGene	GeneList	geneUp	geneDown
	sclerosis (ALS)		diseases					
ko00310	Lysine degradation	4/1083	Metabolism; Amino acid metabolism	KEGG link	4	DN121980_c1_g1_i1;DN121980_c1_g1_i6;DN123388_c2_g4_i10;DN124716_c1_g3_i5;	DN124716_c1_g3_i5;	DN121980_c1_g1_i1;DN121980_c1_g1_i6;DN123388_c2_g4_i10;
ko05132	Salmonella infection	4/1083	Human Diseases; Infectious diseases: Bacterial	KEGG link	4	DN116020_c4_g2_i1;DN126049_c0_g1_i1;DN131056_c0_g1_i1;DN13234_c0_g1_i1;	;	DN116020_c4_g2_i1;DN126049_c0_g1_i1;DN131056_c0_g1_i1;DN13234_c0_g1_i1;
ko03410	Base excision repair	4/1083	Genetic Information Processing; Replication and repair	KEGG link	4	DN105361_c0_g2_i1;DN105361_c0_g2_i2;DN114741_c0_g1_i1;DN114741_c0_g1_i6;	;	DN105361_c0_g2_i1;DN105361_c0_g2_i2;DN114741_c0_g1_i1;DN114741_c0_g1_i6;
ko00340	Histidine metab	4/1083	Metabolism; Amino acid	KEGG link	4	DN109982_c0_g1_i6;DN121980_c1_g1_i1;DN121980_c1_g1_i6;DN123388_c2_g4_i10;	DN109982_c0_g1_i6;	DN121980_c1_g1_i1;DN121980_c1_g1_i6;DN123388_c2_g4_i10;

Pathway ID	Description	Gene Ratio	PathwayClass	KEGG link	TargetGene	GeneList	geneUp	geneDown
	olism		metabolism					
ko00920	Sulfur metabolism	4/1083	Metabolism; Energy metabolism	KEGG link	4	DN123754_c4_g1_i6;DN123873_c0_g2_i4;DN127489_c0_g2_i3;DN127489_c0_g2_i7;	;	DN123754_c4_g1_i6;DN123873_c0_g2_i4;DN127489_c0_g2_i3;DN127489_c0_g2_i7;
ko04670	Leukocyte transendothelial migration	3/1083	Organismal Systems; Immune system	KEGG link	3	DN126049_c0_g1_i1;DN131056_c0_g1_i1;DN13234_c0_g1_i1;	;	DN126049_c0_g1_i1;DN131056_c0_g1_i1;DN13234_c0_g1_i1;
ko04011	MAPK signaling pathway - yeast	3/1083	Environmental Information Processing; Signal transduction	KEGG link	3	DN113227_c0_g1_i4;DN124836_c1_g2_i1;DN128018_c4_g5_i1;	DN124836_c1_g2_i1;DN128018_c4_g5_i1;	DN113227_c0_g1_i4;
ko05412	Arrhythmogenic	3/1083	Human Diseases; Cardiovas	KEGG link	3	DN126049_c0_g1_i1;DN131056_c0_g1_i1;DN13234_c0_g1_i1;	;	DN126049_c0_g1_i1;DN131056_c0_g1_i1;DN13234_c0_g1_i1;

Pathway ID	Description	Gene Ratio	PathwayClass	KEGG link	TargetGene	GeneList	geneUp	geneDown
	right ventricular cardiomyopathy (ARVC)		cular diseases					
ko00909	Sesquiterpenoid and triterpenoid biosynthesis	3/1083	Metabolism; Metabolism of terpenoids and polyketides	KEGG link	3	DN117978_c0_g4_i2;DN117978_c0_g4_i4;DN125668_c0_g2_i10;	DN117978_c0_g4_i2;	DN117978_c0_g4_i4;DN125668_c0_g2_i10;
ko04666	Fc gamma R-mediated phagocytosis	3/1083	Organismal Systems; Immune system	KEGG link	3	DN113227_c0_g1_i4;DN118017_c1_g1_i2;DN126155_c2_g6_i1;	DN118017_c1_g1_i2;	DN113227_c0_g1_i4;DN126155_c2_g6_i1;

Pathway ID	Description	Gene Ratio	PathwayClass	KEGG link	TargetGene	GeneList	geneUp	geneDown
ko04066	HIF-1 signaling pathway	3/1083	Environmental Information Processing; Signal transduction	KEGG link	3	DN109405_c0_g1_i1;DN123859_c0_g4_i17;DN123921_c3_g2_i1;	DN123859_c0_g4_i17;	DN109405_c0_g1_i1;DN123921_c3_g2_i1;
ko00965	Betalain biosynthesis	3/1083	Metabolism; Biosynthesis of other secondary metabolites	KEGG link	3	DN116739_c1_g2_i1;DN127565_c1_g1_i1;DN75078_c0_g1_i1;	DN116739_c1_g2_i1;DN75078_c0_g1_i1;	DN127565_c1_g1_i1;
ko04340	Hedgehog signaling pathway	3/1083	Environmental Information Processing; Signal transduction	KEGG link	3	DN110707_c0_g2_i3;DN121802_c0_g3_i3;DN121802_c0_g3_i4;	DN121802_c0_g3_i3;	DN110707_c0_g2_i3;DN121802_c0_g3_i4;

Pathway ID	Description	Gene Ratio	PathwayClass	KEGG link	TargetGene	GeneList	geneUp	geneDown
ko00760	Nicotinate and nicotinamide metabolism	3/1083	Metabolism; Metabolism of cofactors and vitamins	KEGG link	3	DN116902_c0_g1_i1;DN126525_c1_g3_i3;DN126525_c1_g8_i1;	DN116902_c0_g1_i1;DN126525_c1_g3_i3;DN126525_c1_g8_i1;	;
ko04520	Adherens junction	3/1083	Cellular Processes; Cellular community - eukaryotes	KEGG link	3	DN126049_c0_g1_i1;DN131056_c0_g1_i1;DN13234_c0_g1_i1;	;	DN126049_c0_g1_i1;DN131056_c0_g1_i1;DN13234_c0_g1_i1;

Pathway ID	Description	Gene Ratio	PathwayClass	KEGG link	TargetGene	GeneList	geneUp	geneDown
ko05167	Kaposi's sarcoma-associated herpesvirus infection	3/1083	Human Diseases; Infectious diseases: Viral	KEGG link	3	DN120346_c0_g1_i7;DN127489_c0_g2_i3;DN127489_c0_g2_i7;	DN120346_c0_g1_i7;	DN127489_c0_g2_i3;DN127489_c0_g2_i7;
ko00981	Insect hormone biosynthesis	3/1083	Metabolism; Metabolism of terpenoids and polyketides	KEGG link	3	DN121980_c1_g1_i1;DN121980_c1_g1_i6;DN123388_c2_g4_i10;	;	DN121980_c1_g1_i1;DN121980_c1_g1_i6;DN123388_c2_g4_i10;
ko04962	Vasopressin-regulated water reabso	3/1083	Organismal Systems; Excretory system	KEGG link	3	DN111858_c0_g1_i2;DN115552_c2_g1_i1;DN118948_c1_g4_i1;	DN111858_c0_g1_i2;DN115552_c2_g1_i1;	DN118948_c1_g4_i1;

Pathway ID	Description	Gene Ratio	PathwayClass	KEGG link	TargetGene	GeneList	geneUp	geneDown
	rption							
ko05414	Dilated cardiomyopathy	3/1083	Human Diseases; Cardiovascular diseases	KEGG link	3	DN126049_c0_g1_i1;DN131056_c0_g1_i1;DN13234_c0_g1_i1;	;	DN126049_c0_g1_i1;DN131056_c0_g1_i1;DN13234_c0_g1_i1;
ko04216	Ferroptosis	3/1083	Cellular Processes; Cell growth and death	KEGG link	3	DN127497_c4_g1_i10;DN127497_c4_g1_i12;DN127497_c4_g1_i8;	DN127497_c4_g1_i12;	DN127497_c4_g1_i10;DN127497_c4_g1_i8;
ko04918	Thyroid hormone synthesis	3/1083	Organismal Systems; Endocrine system	KEGG link	3	DN103751_c0_g1_i1;DN126805_c1_g4_i2;DN128341_c3_g5_i2;	DN128341_c3_g5_i2;	DN103751_c0_g1_i1;DN126805_c1_g4_i2;
ko04371	Apelin signaling pathway	3/1083	Environmental Information Processin	KEGG link	3	DN109405_c0_g1_i1;DN117551_c6_g6_i6;DN128767_c7_g2_i1;	DN117551_c6_g6_i6;	DN109405_c0_g1_i1;DN128767_c7_g2_i1;

Pathway ID	Description	Gene Ratio	PathwayClass	KEGG link	TargetGene	GeneList	geneUp	geneDown
			g; Signal transduction					
ko04972	Pancreatic secretion	3/1083	Organismal Systems; Digestive system	KEGG link	3	DN111858_c0_g1_i2;DN118948_c1_g4_i1;DN127115_c2_g2_i1;	DN111858_c0_g1_i2;	DN118948_c1_g4_i1;DN127115_c2_g2_i1;
ko05161	Hepatitis B	3/1083	Human Diseases; Infectious diseases: Viral	KEGG link	3	DN107826_c1_g1_i6;DN127489_c0_g2_i3;DN127489_c0_g2_i7;	;	DN107826_c1_g1_i6;DN127489_c0_g2_i3;DN127489_c0_g2_i7;
ko00401	Novobiocin biosynthesis	3/1083	Metabolism; Biosynthesis of other secondary metabolites	KEGG link	3	DN123784_c4_g1_i4;DN123784_c4_g1_i5;DN123784_c4_g1_i6;	DN123784_c4_g1_i4;DN123784_c4_g1_i5;DN123784_c4_g1_i6;	;

Pathway ID	Description	Gene Ratio	PathwayClass	KEGG link	TargetGene	GeneList	geneUp	geneDown
ko00903	Limonene and pinene degradation	3/1083	Metabolism; Metabolism of terpenoids and polyketides	KEGG link	3	DN121980_c1_g1_i1;DN121980_c1_g1_i6;DN123388_c2_g4_i10;	;	DN121980_c1_g1_i1;DN121980_c1_g1_i6;DN123388_c2_g4_i10;
ko04540	Gap junction	3/1083	Cellular Processes; Cellular community - eukaryotes	KEGG link	3	DN114856_c0_g2_i1;DN116315_c2_g1_i2;DN120827_c1_g3_i1;	DN120827_c1_g3_i1;	DN114856_c0_g2_i1;DN116315_c2_g1_i2;
ko00770	Pantothenate and CoA biosynthesis	3/1083	Metabolism; Metabolism of cofactors and vitamins	KEGG link	3	DN116773_c0_g1_i5;DN126982_c2_g1_i2;DN128195_c1_g1_i3;	DN116773_c0_g1_i5;DN126982_c2_g1_i2;	DN128195_c1_g1_i3;

Pathway ID	Description	Gene Ratio	PathwayClass	KEGG link	TargetGene	GeneList	geneUp	geneDown
ko04912	GnRH signaling pathway	3/1083	Organismal Systems; Endocrine system	KEGG link	3	DN118017_c1_g1_i2;DN126155_c2_g6_i1;DN128767_c7_g2_i1;	DN118017_c1_g1_i2;	DN126155_c2_g6_i1;DN128767_c7_g2_i1;
ko00511	Other glycan degradation	3/1083	Metabolism; Glycan biosynthesis and metabolism	KEGG link	3	DN123937_c0_g3_i2;DN123937_c0_g3_i3;DN127624_c2_g2_i3;	DN123937_c0_g3_i2;DN123937_c0_g3_i3;DN127624_c2_g2_i3;	;
ko00626	Naphthalene degradation	3/1083	Metabolism; Xenobiotics biodegradation and metabolism	KEGG link	3	DN119057_c0_g6_i15;DN119057_c0_g6_i21;DN119057_c0_g6_i29;	DN119057_c0_g6_i15;DN119057_c0_g6_i21;DN119057_c0_g6_i29;	;
ko05230	Central carbon metabolism	3/1083	Human Diseases; Cancers: Overview	KEGG link	3	DN123921_c3_g2_i1;DN127571_c3_g2_i5;DN127577_c6_g2_i1;	DN127577_c6_g2_i1;	DN123921_c3_g2_i1;DN127571_c3_g2_i5;

Pathway ID	Description	Gene Ratio	PathwayClass	KEGG link	TargetGene	GeneList	geneUp	geneDown
	in cancer							
ko04341	Hedgehog signaling pathway - fly	3/1083	Environmental Information Processing; Signal transduction	KEGG link	3	DN110707_c0_g2_i3;DN121802_c0_g3_i3;DN121802_c0_g3_i4;	DN121802_c0_g3_i3;	DN110707_c0_g2_i3;DN121802_c0_g3_i4;
ko04392	Hippo signaling pathway -multiple species	3/1083	Environmental Information Processing; Signal transduction	KEGG link	3	DN121802_c0_g3_i3;DN121802_c0_g3_i4;DN122415_c1_g1_i1;	DN121802_c0_g3_i3;	DN121802_c0_g3_i4;DN122415_c1_g1_i1;
ko04970	Salivary secretion	2/1083	Organismal Systems; Digestive	KEGG link	2	DN124251_c0_g11_i1;DN128767_c7_g2_i1;	;	DN124251_c0_g11_i1;DN128767_c7_g2_i1;

Pathway ID	Description	Gene Ratio	PathwayClass	KEGG link	TargetGene	GeneList	geneUp	geneDown
			system					
ko00790	Folate biosynthesis	2/1083	Metabolism; Metabolism of cofactors and vitamins	[KEGG link]()	2	DN128241_c5_g1_i15;DN128241_c5_g1_i9;	DN128241_c5_g1_i15;DN128241_c5_g1_i9;	;
ko00514	Other types of O-glycan biosynthesis	2/1083	Metabolism; Glycan biosynthesis and metabolism	[KEGG link]()	2	DN123911_c0_g2_i4;DN123911_c0_g2_i7;	DN123911_c0_g2_i4;DN123911_c0_g2_i7;	;
ko04930	Type II diabetes mellitus	2/1083	Human Diseases; Endocrine and metabolic diseases	[KEGG link]()	2	DN123921_c3_g2_i1;DN127571_c3_g2_i5;	;	DN123921_c3_g2_i1;DN127571_c3_g2_i5;

Pathway ID	Description	Gene Ratio	PathwayClass	KEGG link	TargetGene	GeneList	geneUp	geneDown
ko00510	N-Glycan biosynthesis	2/1083	Metabolism; Glycan biosynthesis and metabolism	KEGG link	2	DN125872_c1_g2_i1;DN125872_c1_g2_i4;	DN125872_c1_g2_i1;DN125872_c1_g2_i4;	;
ko00780	Biotin metabolism	2/1083	Metabolism; Metabolism of cofactors and vitamins	KEGG link	2	DN119487_c1_g2_i10;DN119487_c1_g2_i8;	DN119487_c1_g2_i10;	DN119487_c1_g2_i8;
ko04350	TGF-beta signaling pathway	2/1083	Environmental Information Processing; Signal transduction	KEGG link	2	DN123205_c4_g2_i7;DN125626_c0_g3_i12;	DN123205_c4_g2_i7;DN125626_c0_g3_i12;	;

Pathway ID	Description	Gene Ratio	PathwayClass	KEGG link	TargetGene	GeneList	geneUp	geneDown
ko03450	Non-homologous end-joining	2/1083	Genetic Information Processing; Replication and repair	KEGG link	2	DN125968_c2_g5_i14;DN128690_c2_g1_i3;	DN128690_c2_g1_i3;	DN125968_c2_g5_i14;
ko04973	Carbohydrate digestion and absorption	2/1083	Organismal Systems; Digestive system	KEGG link	2	DN115973_c1_g1_i9;DN123921_c3_g2_i1;	DN115973_c1_g1_i9;	DN123921_c3_g2_i1;
ko05160	Hepatitis C	2/1083	Human Diseases; Infectious diseases: Viral	KEGG link	2	DN123205_c4_g2_i7;DN125626_c0_g3_i12;	DN123205_c4_g2_i7;DN125626_c0_g3_i12;	;
ko04924	Renin secretion	2/1083	Organismal Systems; Endocrine	KEGG link	2	DN120346_c0_g1_i7;DN128767_c7_g2_i1;	DN120346_c0_g1_i7;	DN128767_c7_g2_i1;

Pathway ID	Description	Gene Ratio	PathwayClass	KEGG link	TargetGene	GeneList	geneUp	geneDown
			system					
ko04975	Fat digestion and absorption	2/1083	Organismal Systems; Digestive system	KEGG link	2	DN124052_c1_g4_i6;DN127115_c2_g2_i1;	;	DN124052_c1_g4_i6;DN127115_c2_g2_i1;
ko02026	Biofilm formation - Escherichia coli	2/1083	Cellular Processes; Cellular community - prokaryotes	KEGG link	2	DN119965_c2_g2_i1;DN126429_c5_g2_i9;	DN126429_c5_g2_i9;	DN119965_c2_g2_i1;
ko04380	Osteoclast differentiation	2/1083	Organismal Systems; Development	KEGG link	2	DN119880_c0_g6_i1;DN120346_c0_g1_i7;	DN120346_c0_g1_i7;	DN119880_c0_g6_i1;
ko00563	Glycosylphosphatidylino	2/1083	Metabolism; Glycan biosynthesis and	KEGG link	2	DN118702_c3_g2_i2;DN123280_c0_g1_i3;	DN118702_c3_g2_i2;DN123280_c0_g1_i3;	;

Pathway ID	Description	Gene Ratio	PathwayClass	[KEGG link]	TargetGene	GeneList	geneUp	geneDown
	sitol (GPI)-anchor biosynthesis		metabolism					
ko05202	Transcriptional misregulation in cancer	2/1083	Human Diseases; Cancers: Overview	KEGG link	2	DN117557_c0_g1_i17;DN69058_c0_g1_i1;	DN117557_c0_g1_i17;	DN69058_c0_g1_i1;
ko05020	Prion diseases	2/1083	Human Diseases; Neurodegenerative diseases	KEGG link	2	DN103751_c0_g1_i1;DN121330_c5_g6_i4;	DN121330_c5_g6_i4;	DN103751_c0_g1_i1;
ko04214	Apoptosis - fly	2/1083	Cellular Processes; Cell growth and death	KEGG link	2	DN127489_c0_g2_i3;DN127489_c0_g2_i7;	;	DN127489_c0_g2_i3;DN127489_c0_g2_i7;

Pathway ID	Description	Gene Ratio	PathwayClass	KEGG link	TargetGene	GeneList	geneUp	geneDown
ko05210	Colorectal cancer	2/1083	Human Diseases; Cancers: Specific types	KEGG link	2	DN127489_c0_g2_i3;DN127489_c0_g2_i7;	;	DN127489_c0_g2_i3;DN127489_c0_g2_i7;
ko00740	Riboflavin metabolism	2/1083	Metabolism; Metabolism of cofactors and vitamins	KEGG link	2	DN119817_c2_g1_i6;DN119880_c0_g6_i1;	DN119817_c2_g1_i6;	DN119880_c0_g6_i1;
ko04923	Regulation of lipolysis in adipocytes	2/1083	Organismal Systems; Endocrine system	KEGG link	2	DN118105_c1_g3_i3;DN118105_c1_g3_i4;	;	DN118105_c1_g3_i3;DN118105_c1_g3_i4;
ko00590	Arachidonic acid metabolism	2/1083	Metabolism; Lipid metabolism	KEGG link	2	DN122154_c0_g1_i2;DN127115_c2_g2_i1;	DN122154_c0_g1_i2;	DN127115_c2_g2_i1;

Pathway ID	Description	Gene Ratio	PathwayClass	KEGG link	TargetGene	GeneList	geneUp	geneDown
ko04711	Circadian rhythm - fly	2/1083	Organismal Systems; Environmental adaptation	KEGG link	2	DN121802_c0_g3_i3;DN121802_c0_g3_i4;	DN121802_c0_g3_i3;	DN121802_c0_g3_i4;
ko00531	Glycosaminoglycan degradation	2/1083	Metabolism; Glycan biosynthesis and metabolism	KEGG link	2	DN120133_c0_g2_i3;DN125310_c4_g3_i1;	DN125310_c4_g3_i1;	DN120133_c0_g2_i3;
ko04730	Long-term depression	2/1083	Organismal Systems; Nervous system	KEGG link	2	DN123205_c4_g2_i7;DN125626_c0_g3_i12;	DN123205_c4_g2_i7;DN125626_c0_g3_i12;	;
ko04215	Apoptosis - multiple species	2/1083	Cellular Processes; Cell growth and death	KEGG link	2	DN127489_c0_g2_i3;DN127489_c0_g2_i7;	;	DN127489_c0_g2_i3;DN127489_c0_g2_i7;

Pathway ID	Description	Gene Ratio	PathwayClass	KEGG link	TargetGene	GeneList	geneUp	geneDown
ko04139	Mitophagy - yeast	2/1083	Cellular Processes; Transport and catabolism	KEGG link	2	DN113227_c0_g1_i4;DN118671_c4_g1_i8;	DN118671_c4_g1_i8;	DN113227_c0_g1_i4;
ko00670	One carbon pool by folate	1/1083	Metabolism; Metabolism of cofactors and vitamins	KEGG link	1	DN124509_c1_g1_i1;	;	DN124509_c1_g1_i1;
ko04650	Natural killer cell mediated cytotoxicity	1/1083	Organismal Systems; Immune system	KEGG link	1	DN120346_c0_g1_i7;	DN120346_c0_g1_i7;	;
ko00232	Caffeine metabolism	1/1083	Metabolism; Biosynthesis of	KEGG link	1	DN127816_c0_g2_i20;	DN127816_c0_g2_i20;	;

Pathway ID	Description	Gene Ratio	PathwayClass	KEGG link	TargetGene	GeneList	geneUp	geneDown
			other secondary metabolites					
ko00901	Indole alkaloid biosynthesis	1/1083	Metabolism; Biosynthesis of other secondary metabolites	KEGG link	1	DN127270_c4_g5_i1;	DN127270_c4_g5_i1;	;
ko00261	Monobactam biosynthesis	1/1083	Metabolism; Biosynthesis of other secondary metabolites	KEGG link	1	DN123754_c4_g1_i6;	;	DN123754_c4_g1_i6;
ko01051	Biosynthesis of ansam	1/1083	Metabolism; Metabolism of	KEGG link	1	DN125313_c0_g5_i3;	DN125313_c0_g5_i3;	;

Pathway ID	Description	Gene Ratio	PathwayClass	KEGG link	TargetGene	GeneList	geneUp	geneDown
	ycins		terpenoids and polyketides					
ko00254	Aflatoxin biosynthesis	1/1083	Metabolism; Biosynthesis of other secondary metabolites	KEGG link	1	DN127595_c2_g1_i1;	;	DN127595_c2_g1_i1;
ko04658	Th1 and Th2 cell differentiation	1/1083	Organismal Systems; Immune system	KEGG link	1	DN120346_c0_g1_i7;	DN120346_c0_g1_i7;	;
ko03050	Proteasome	1/1083	Genetic Information Processing;	KEGG link	1	DN125245_c0_g2_i2;	;	DN125245_c0_g2_i2;

Pathway ID	Description	Gene Ratio	PathwayClass	KEGG link	TargetGene	GeneList	geneUp	geneDown
			Folding, sorting and degradation					
ko00603	Glycosphingolipid biosynthesis - globo and isoglobo series	1/1083	Metabolism; Glycan biosynthesis and metabolism	KEGG link	1	DN118842_c0_g1_i11;	;	DN118842_c0_g1_i11;
ko05146	Amoebiasis	1/1083	Human Diseases; Infectious diseases: Parasitic	KEGG link	1	DN191328_c0_g1_i1;	;	DN191328_c0_g1_i1;
ko00660	C5-Branched	1/1083	Metabolism; Carbohydr	KEGG link	1	DN126982_c2_g1_i2;	DN126982_c2_g1_i2;	;

Pathway ID	Description	Gene Ratio	PathwayClass	KEGG link	TargetGene	GeneList	geneUp	geneDown
	dibasic acid metabolism		ate metabolism					
ko01521	EGFR tyrosine kinase inhibitor resistance	1/1083	Human Diseases; Drug resistance: Antineoplastic	KEGG link	1	DN109405_c0_g1_i1;	;	DN109405_c0_g1_i1;
ko04112	Cell cycle - Caulobacter	1/1083	Cellular Processes; Cell growth and death	KEGG link	1	DN117560_c0_g1_i2;	DN117560_c0_g1_i2;	;
ko00515	Mannose type O-glycan biosynthesis	1/1083	Metabolism; Glycan biosynthesis and metabolism	KEGG link	1	DN118291_c0_g1_i1;	;	DN118291_c0_g1_i1;

Pathway ID	Description	Gene Ratio	PathwayClass	KEGG link	TargetGene	GeneList	geneUp	geneDown
ko04916	Melanogenesis	1/1083	Organismal Systems; Endocrine system	KEGG link	1	DN128767_c7_g2_i1;	;	DN128767_c7_g2_i1;
ko03030	DNA replication	1/1083	Genetic Information Processing; Replication and repair	KEGG link	1	DN111603_c0_g1_i1;	;	DN111603_c0_g1_i1;
ko03460	Fanconi anemia pathway	1/1083	Genetic Information Processing; Replication and repair	KEGG link	1	DN124862_c0_g1_i13;	DN124862_c0_g1_i13;	;

Pathway ID	Description	Gene Ratio	PathwayClass	KEGG link	TargetGene	GeneList	geneUp	geneDown
ko00524	Neomycin, kanamycin and gentamicin biosynthesis	1/1083	Metabolism; Biosynthesis of other secondary metabolites	KEGG link	1	DN123921_c3_g2_i1;	;	DN123921_c3_g2_i1;
ko04360	Axon guidance	1/1083	Organismal Systems; Development	KEGG link	1	DN120346_c0_g1_i7;	DN120346_c0_g1_i7;	;
ko00944	Flavone and flavonol biosynthesis	1/1083	Metabolism; Biosynthesis of other secondary metabolites	KEGG link	1	DN115083_c0_g1_i2;	;	DN115083_c0_g1_i2;
ko00650	Butanoate	1/1083	Metabolism;	KEGG link	1	DN126982_c2_g1_i2;	DN126982_c2_g1_i2;	;

Pathway ID	Description	Gene Ratio	PathwayClass	KEGG link	TargetGene	GeneList	geneUp	geneDown
	metabolism		Carbohydrate metabolism					
ko04660	T cell receptor signaling pathway	1/1083	Organismal Systems; Immune system	KEGG link	1	DN120346_c0_g1_i7;	DN120346_c0_g1_i7;	;
ko04740	Olfactory transduction	1/1083	Organismal Systems; Sensory system	KEGG link	1	DN128767_c7_g2_i1;	;	DN128767_c7_g2_i1;
ko04130	SNARE interactions in vesicular transp	1/1083	Genetic Information Processing; Folding, sorting and	KEGG link	1	DN125588_c4_g2_i5;	DN125588_c4_g2_i5;	;

Pathway ID	Description	Gene Ratio	PathwayClass	KEGG link	TargetGene	GeneList	geneUp	geneDown
	ort		degradation					
ko04744	Phototransduction	1/1083	Organismal Systems; Sensory system	KEGG link	1	DN128767_c7_g2_i1;	;	DN128767_c7_g2_i1;
ko03022	Basal transcription factors	1/1083	Genetic Information Processing; Transcription	KEGG link	1	DN119865_c0_g2_i2;	;	DN119865_c0_g2_i2;
ko04370	VEGF signaling pathway	1/1083	Environmental Information Processing; Signal transduction	KEGG link	1	DN120346_c0_g1_i7;	DN120346_c0_g1_i7;	;

Pathway ID	Description	Gene Ratio	PathwayClass	KEGG link	TargetGene	GeneList	geneUp	geneDown
ko00300	Lysine biosynthesis	1/1083	Metabolism; Amino acid metabolism	KEGG link	1	DN124655_c0_g2_i2;	DN124655_c0_g2_i2;	;
ko04713	Circadian entrainment	1/1083	Organismal Systems; Environmental adaptation	KEGG link	1	DN128767_c7_g2_i1;	;	DN128767_c7_g2_i1;
ko04971	Gastric acid secretion	1/1083	Organismal Systems; Digestive system	KEGG link	1	DN128767_c7_g2_i1;	;	DN128767_c7_g2_i1;
ko00120	Primary bile acid biosynthesis	1/1083	Metabolism; Lipid metabolism	KEGG link	1	DN43497_c0_g1_i1;	;	DN43497_c0_g1_i1;
ko04925	Aldosterone synthe	1/1083	Organismal Systems;	KEGG link	1	DN128767_c7_g2_i1;	;	DN128767_c7_g2_i1;

Pathway ID	Description	Gene Ratio	PathwayClass	KEGG link	TargetGene	GeneList	geneUp	geneDown
	sis and secretion		Endocrine system					
ko04662	B cell receptor signaling pathway	1/1083	Organismal Systems; Immune system	KEGG link	1	DN120346_c0_g1_i7;	DN120346_c0_g1_i7;	;
ko05214	Glioma	1/1083	Human Diseases; Cancers: Specific types	KEGG link	1	DN128767_c7_g2_i1;	;	DN128767_c7_g2_i1;
ko04622	RIG-I-like receptor signaling pathway	1/1083	Organismal Systems; Immune system	KEGG link	1	DN115170_c0_g1_i1;	;	DN115170_c0_g1_i1;
