# Supplementary material for: Transcriptome data-based screening of potential host of genetic transformation for a blue-hued Bougainvillea transgene
Source: Genet Mol Biol. 2024 Mar 4;47(1):e20230099. doi: 10.1590/1678-4685-GMB-2023-0099 (PMC10941975; doi:10.1590/1678-4685-GMB-2023-0099)
Supplement: Table S1 - [file 1415-4757-GMB-47-1-e20230099-s1.pdf]

Genetics and Molecular Biology

https://doi.org/10.1590/1678-4685-GMB-2023-0099

Supplementary Material to "Transcriptome data-based screening of potential host of genetic transformation for a blue-hued *Bougainvillea* transgene"

Table S1 - All annotation information of DEGs.

geneid	trend	GO	Pathway	TF	NR	NT	Uniprot
DN127706_c1_g2_i1	UP	[GO:0005737:cytoplasm][GO:0005524:ATP binding][GO:0016485:protein processing][GO:0009408:response to heat]	[ko04213:Longevity regulating pathway - multiple species]	Achn116231:Actinidia chinensis|ARF|ARF family protein	XP_021770041.1:chaperone protein ClpB3, chloroplasticlike [Chenopodium quinoa]	gi|359488961|ref|XM_002284207.2|:PREDICTED: Vitis vinifera chaperone protein ClpB3, chloroplastic-like (LOC100252611), mRNA	A0A0J8CU40|A0A0J8CU40_BETVU:Uncharacterized protein;OS=Beta vulgaris subsp. vulgaris.;|GO:0005737,|GO:0005524,|GO:0016485,GO:0009408,
DN120278_c2_g4_i2	UP	-	-	-	XP_021857368.1:uncharacterized protein LOC110796612 [Spinacia oleracea]	-	-

geneid	trend	GO	Pathway	TF	NR	NT	Uniprot
DN123216_c2_g7_i8	UP	[GO:0005737:cytoplasm][GO:0005634:nucleus][GO:0000977:RNA polymerase II regulatory region sequence-specific DNA binding][GO:0003700:DNA-binding transcription factor activity][GO:0048438:floral whorl development][GO:0010077:maintenance of inflorescence meristem identity][GO:0000165:MAPK cascade][GO:0045944:positive regulation of transcription by RNA polymerase II][GO:0000060:obsolete protein import into nucleus, translocation][GO:0048510:regulation of timing of transition from vegetative to reproductive phase][GO:0006351:transcription, DNA-templated]	-	Bv1_007680_jyqa.t1:Beta vulgaris|MIKC_MADS|MIKC_MADS family protein	XP_021740351.1:MADSbox protein AGL24like isoform X1 [Chenopodium quinoa]	gi|55792843|gb|AY643733.1|:Solanum tuberosum MADS16 (MADS16) mRNA, complete cds	A0A0J8D685|A0A0J8D685_BETVU:Uncharacterized protein;OS=Beta vulgaris subsp. vulgaris.;|GO:0005737,GO:0005634,|GO:0000977,GO:0003700,|GO:0048438,GO:0010077,GO:0000165,GO:0045944,GO:0000060,GO:0048510,GO:0006351,

geneid	trend	GO	Pathway	TF	NR	NT	Uniprot
DN123216_c2_g7_i1	UP	[GO:0005737:cytoplasm][GO:0005634:nucleus][GO:0000977:RNA polymerase II regulatory region sequence-specific DNA binding][GO:0003700:DNA-binding transcription factor activity][GO:0048438:floral whorl development][GO:0010077:maintenance of inflorescence meristem identity][GO:0000165:MAPK cascade][GO:0045944:positive regulation of transcription by RNA polymerase II][GO:0000060:obsolete protein import into nucleus, translocation][GO:0048510:regulation of timing of transition from vegetative to reproductive phase][GO:0006351:transcription, DNA-templated]	-	Bv1_007680_jyqa.t1:Beta vulgaris|MIKC_MADS|MIKC_MADS family protein	XP_021740351.1:MADSbox protein AGL24like isoform X1 [Chenopodium quinoa]	gi|55792843|gb|AY643733.1|:Solanum tuberosum MADS16 (MADS16) mRNA, complete cds	A0A0J8D685|A0A0J8D685_BETVU:Uncharacterized protein;OS=Beta vulgaris subsp. vulgaris.;|GO:0005737,GO:0005634,|GO:0000977,GO:0003700,|GO:0048438,GO:0010077,GO:0000165,GO:0045944,GO:0000060,GO:0048510,GO:0006351,
DN120278_c2_g6_i2	UP	-	-	-	XP_021857368.1:uncharacterized protein LOC110796612 [Spinacia oleracea]	-	-
DN120278_c	UP	-	-	-	-	-	-

geneid	trend	GO	Pathway	TF	NR	NT	Uniprot
2_g3_i3							
DN119432_c1_g2_i7	UP	[GO:0005634:nucleus][GO:0000977:RNA polymerase II regulatory region sequence-specific DNA binding][GO:0003700:DNA-binding transcription factor activity][GO:0000165:MAPK cascade][GO:0045944:positive regulation of transcription by RNA polymerase II][GO:0006351:transcription, DNA-templated]	-	Sp_023090_ietn.t2:Spinacia oleracea|MIKC_MADS|MIKC_MADS family protein	XP_021844484.1:agamouslike MADSbox protein AGL19 [Spinacia oleracea] >KNA23649.1 hypothetical protein SOVF_023090 isoform B [Spinacia oleracea]	gi|449519945|ref|XM_004166947.1|:PREDICTED: Cucumis sativus agamous-like MADS-box protein AGL19-like (LOC101223959), mRNA	A0A0K9RXL2|A0A0K9RXL2_SPIOL: Uncharacterized protein;OS=Spinacia oleracea;|GO:0005634,|GO:0000977,GO:0003700,|GO:0000165,GO:0045944,GO:0006351,
DN3604_c0_g1_i1	UP	-	[ko04141:Protein processing in endoplasmic reticulum]	WALNUT_00030998-RA:Juglans regia|NF-YB|NF-YB family protein	XP_002308192.2:18.2 kDa class I heat shock family protein [Populus trichocarpa] >EEE91715.2 18.2 kDa class I heat shock family protein [Populus trichocarpa]	gi|470143875|ref|XM_004307545.1|:PREDICTED: Fragaria vesca subsp. vesca 17.5 kDa class I heat shock protein-like (LOC101312485), mRNA	B9HC70|B9HC70_POPTR:18.2 kDa class I heat shock family protein;OS=Populus trichocarpa;

geneid	trend	GO	Pathway	TF	NR	NT	Uniprot
DN128910_c2_g1_i3	UP	[GO:0005737:cytoplasm][GO:0005634:nucleus][GO:0000977:RNA polymerase II regulatory region sequence-specific DNA binding][GO:0003700:DNA-binding transcription factor activity][GO:0048438:floral whorl development][GO:0010077:maintenance of inflorescence meristem identity][GO:0000165:MAPK cascade][GO:0045944:positive regulation of transcription by RNA polymerase II][GO:0000060:obsolete protein import into nucleus, translocation][GO:0048510:regulation of timing of transition from vegetative to reproductive phase][GO:0006351:transcription, DNA-templated]	-	Bv1_007680_jyqa.t1:Beta vulgaris|MIKC_MADS|MIKC_MADS family protein	XP_010675143.1:PREDICTED: MADSbox protein JOINTLESS [Beta vulgaris subsp. vulgaris] >KMT19713.1 hypothetical protein BVRB_1g007680 [Beta vulgaris subsp. vulgaris]	-	A0A0J8D685|A0A0J8D685_BETVU:Uncharacterized protein;OS=Beta vulgaris subsp. vulgaris.;|GO:0005737,GO:0005634,|GO:0000977,GO:0003700,|GO:0048438,GO:0010077,GO:0000165,GO:0045944,GO:0000060,GO:0048510,GO:0006351,
DN125985_c1_g1_i5	UP	[GO:0016787:hydrolase activity]	-	329122:Arabidopsis lyrata|C3H|C3H family protein	XP_021771392.1:nudix hydrolase 10like [Chenopodium quinoa]	gi|478164044|gb|GAHK01012559.1|:TSA: Jatropha curcas Unigene10_JC-CK_1A transcribed RNA sequence	A0A0J8CGU6|A0A0J8CGU6_BETVU: Uncharacterized protein;OS=Beta vulgaris subsp. vulgaris.;|GO:00167

geneid	trend	GO	Pathway	TF	NR	NT	Uniprot
							87,
DN126394_c3_g1_i1	UP	-	-	-	-	-	-
DN120852_c2_g1_i9	UP	-	-	-	XP_010695939.1:PREDICTED: putative hydrolase C777.06c isoform X1 [Beta vulgaris subsp. vulgaris]	-	A0A0J8BBF9|A0A0J8BBF9_BETVU:Uncharacterized protein;OS=Beta vulgaris subsp. vulgaris.;
DN121117_c0_g5_i3	UP	[GO:0005622:intracellular][GO:0008270:zinc ion binding]	-	Sp_021810_twdn.t2:Spinacia oleracea|CO-like|CO-like family protein	ACB36911.2:CONSTANSlike protein 1 [Oxybasis rubra]	gi|334847619|gb|EU395770.2|:Chenopodium rubrum CONSTANS-like protein 1 (COL1) mRNA, complete cds, alternatively spliced	B4YA13|B4YA13_OXYRB:CONSTANS-like protein 1;OS=Oxybasis rubra;|GO:0005622,|GO:0008270,

geneid	trend	GO	Pathway	TF	NR	NT	Uniprot
DN121777_c4_g3_i1	UP	-	-	Bv6_145140_chao.t1:Beta vulgaris|Nin-like|Nin-like family protein	XP_010682023.1:PREDICTED: protein NLP9 [Beta vulgaris subsp. vulgaris] >KMT07949.1 hypothetical protein BVRB_6g145140 [Beta vulgaris subsp. vulgaris]	-	A0A0J8C6U7|A0A0J8C6U7_BETVU:Uncharacterized protein;OS=Beta vulgaris subsp. vulgaris.;
DN125271_c2_g4_i3	UP	-	-	-	XP_021864826.1:uncharacterized protein LOC110803610 [Spinacia oleracea] >KNA03155.1 hypothetical protein SOVF_211870 [Spinacia oleracea]	-	A0A0K9Q7F5|A0A0K9Q7F5_SPIOL:Uncharacterized protein;OS=Spinacia oleracea;
DN113561_c3_g4_i1	UP	-	-	-	-	-	-
DN129129_c11_g7_i2	UP	[GO:0005524:ATP binding][GO:0019538:protein metabolic process]	[ko04213:Longevity regulating pathway - multiple species]	Achn116231:Actinidia chinensis|ARF|ARF family protein	XP_021761516.1:chaperone protein ClpB1 [Chenopodium quinoa]	gi|332189094|gb|CP002684.1|:Arabidopsis thaliana chromosome 1, complete sequence	A0A0J8E3S6|A0A0J8E3S6_BETVU:Uncharacterized protein;OS=Beta vulgaris subsp.

geneid	trend	GO	Pathway	TF	NR	NT	Uniprot
							vulgaris.;|GO:0005524,|GO:0019538,
DN125839_c0_g6_i3	UP	-	-	-	-	-	-
DN106395_c0_g1_i2	UP	[GO:0043565:sequence-specific DNA binding][GO:0003700:DNA-binding transcription factor activity]	-	Sp_071390_ugwc.t2:Spinacia oleracea|bZIP|bZIP family protein	XP_021728696.1:uncharacterized protein LOC110695771 [Chenopodium quinoa]	-	A0A0K9RFU7|A0A0K9RFU7_SPIOL:Uncharacterized protein;OS=Spinacia oleracea;|GO:0043565,GO:0003700,
DN116240_c0_g3_i3	UP	[GO:0016788:hydrolase activity, acting on ester bonds]	-	-	XP_021734889.1:GDSL esterase/lipase 7like [Chenopodium quinoa]	-	A0A0J8D321|A0A0J8D321_BETVU:Uncharacterized protein;OS=Beta vulgaris subsp. vulgaris.;|GO:0016788,

geneid	trend	GO	Pathway	TF	NR	NT	Uniprot
DN120199_c0_g4_i1	UP	[GO:0005524:ATP binding][GO:0004849:uridine kinase activity][GO:0044211:CTP salvage][GO:0044206:UMP salvage]	[ko00240:Pyrimidine metabolism][ko00983:Drug metabolism - other enzymes]	-	XP_015893752.1:PREDICTED: uridine kinaselike protein 1, chloroplastic isoform X2 [Ziziphus jujuba]	gi|521452270|gb|GAHZ01068214.1|:TSA: Salicornia europaea Unigene82313_SeCKS transcribed RNA sequence	A0A0J8CUM5|A0A0J8CUM5_BETVU: Uridine kinase;OS=Beta vulgaris subsp. vulgaris.;|GO:0005524,GO:0004849,|GO:0044211,GO:0044206,
DN124932_c0_g2_i4	UP	-	[ko04141:Protein processing in endoplasmic reticulum]	WALNUT_00030998-RA:Juglans regia|NF-YB|NF-YB family protein	XP_021743706.1:22.0 kDa class IV heat shock proteinlike [Chenopodium quinoa]	-	A0A0K9RYH1|A0A0K9RYH1_SPIOL: Uncharacterized protein;OS=Spinacia oleracea;
DN119429_c2_g1_i4	UP	[GO:0003755:peptidyl-prolyl cis-trans isomerase activity][GO:0006457:protein folding]	[ko03040:Spliceosome]	-	XP_021752890.1:peptidylprolyl cistrans isomerase CYP22like [Chenopodium quinoa]	-	B9GX77|B9GX77_POPTR:Peptidyl-prolyl cis-trans isomerase;OS=Populus trichocarpa;|GO:0003755,|GO:0006457,

geneid	trend	GO	Pathway	TF	NR	NT	Uniprot
DN119211_c0_g1_i1	UP	-	-	-	XP_010695281.1:PREDICTED: uncharacterized protein LOC104907951 [Beta vulgaris subsp. vulgaris] >KMS97765.1 hypothetical protein BVRB_5g123980 [Beta vulgaris subsp. vulgaris]	-	A0A0J8E3R6|A0A0J8E3R6_BETVU:Uncharacterized protein;OS=Beta vulgaris subsp. vulgaris.;
DN124480_c0_g1_i11	UP	[GO:0005524:ATP binding]	[ko04146:Peroxisome]	EMT26392:Aegilops tauschii|FAR1|FAR1 family protein	KNA13845.1:hypothetical protein SOVF_112890 isoform B [Spinacia oleracea]	gi|460389762|ref|XM_004240470.1|:PREDICTED: Solanum lycopersicum peroxisome biogenesis protein 6-like, transcript variant 2 (LOC101250222), mRNA	A0A0K9R2W0|A0A0K9R2W0_SPIOL: Uncharacterized protein;OS=Spinacia oleracea;|GO:0005524,

geneid	trend	GO	Pathway	TF	NR	NT	Uniprot
DN120351_c0_g2_i7	UP	[GO:0009941:chloroplast envelope][GO:0009570:chloroplast stroma][GO:0009579:thylakoid][GO:0019538:protein metabolic process]	-	-	XP_021725262.1:ATPdependent Clp protease ATPbinding subunit CLPT1, chloroplasticlike isoform X1 [Chenopodium quinoa] >XP_021722766.1 ATPdependent Clp protease ATPbinding subunit CLPT1, chloroplasticlike isoform X1 [Chenopodium quinoa]	-	A0A061G188|A0A061G188_THECC:Double Clp-N motif protein;OS=Theobroma cacao;|GO:0009941,GO:0009570,GO:0009579,|GO:0019538,

geneid	trend	GO	Pathway	TF	NR	NT	Uniprot
DN116486_c0_g1_i3	UP	[GO:0018472:1-hydroxy-2-naphthaldehyde dehydrogenase activity][GO:0034525:1-naphthaldehyde dehydrogenase activity][GO:0044104:2,5-dioxovalerate dehydrogenase (NAD+) activity][GO:0018474:2-carboxybenzaldehyde dehydrogenase activity][GO:0034520:2-naphthaldehyde dehydrogenase activity][GO:0004028:3-chloroallyl aldehyde dehydrogenase activity][GO:0034538:3-methylsalicylaldehyde dehydrogenase activity][GO:0034530:4-hydroxymethylsalicyaldehyde dehydrogenase activity][GO:0019115:benzaldehyde dehydrogenase activity][GO:0018473:cis-2-methyl-5-isopropylhexa-2,5-dienal dehydrogenase activity][GO:0034822:citronellal dehydrogenase activity][GO:0080019:fatty-acyl-CoA reductase (alcohol-forming)	[ko00073:Cutin, suberine and wax biosynthesis][ko04146:Peroxisome][ko04212:Longevity regulating pathway - worm]	-	KNA07365.1:hypothetical protein SOVF_172600 [Spinacia oleracea]	-	A0A0K9QJC3|A0A0K9QJC3_SPIOL:Fatty acyl-CoA reductase;OS=Spinacia oleracea;|GO:0018472,GO:0034525,GO:0044104,GO:0018474,GO:0034520,GO:0004028,GO:0034538,GO:0034530,GO:0019115,GO:0018473,GO:0034822,GO:0080019,GO:0034832,GO:0043796,GO:0043878,GO:0052814,GO:0043870,GO:0043745,GO:0034602,GO:0034601,GO:0034604,GO:0018475,|GO:0006629,

geneid	trend	GO	Pathway	TF	NR	NT	Uniprot
		activity][GO:0034832:geranial dehydrogenase activity][GO:0043796:glyceraldehyde dehydrogenase (NADP) activity][GO:0043878:glyceraldehyde -3-phosphate dehydrogenase (NAD+) (non-phosphorylating) activity][GO:0052814:medium-chain-aldehyde dehydrogenase activity][GO:0043870:N-acetyl-gam ma-aminoadipyl-phosphate reductase activity][GO:0043745:N2-acetyl-L-a minoadipate semialdehyde dehydrogenase activity][GO:0034602:oxoglutarate dehydrogenase (NAD+) activity][GO:0034601:oxoglutarate dehydrogenase [NAD(P)+] activity][GO:0034604:pyruvate dehydrogenase (NAD+) activity][GO:0018475:trans-2-methyl -5-isopropylhexa-2,5-dienal dehydrogenase activity][GO:0006629:lipid metabolic process]					

geneid	trend	GO	Pathway	TF	NR	NT	Uniprot
DN128682_c2_g5_i7	UP	[GO:0046872:metal ion binding][GO:0016702:oxidoreductase activity, acting on single donors with incorporation of molecular oxygen, incorporation of two atoms of oxygen][GO:0006633:fatty acid biosynthetic process][GO:0031408:oxylipin biosynthetic process]	[ko00591:Linoleic acid metabolism][ko00592:alpha-Linolenic acid metabolism]	Lus10031505:Linum usitatissimum|NF-YA|NF-YA family protein	XP_021772004.1:linoleate 13Slipoxygenase 21, chloroplasticlike [Chenopodium quinoa]	-	A0A0K9S108|A0A0K9S108_SPIOL:Lipoxygenase;OS=Spinacia oleracea;|GO:0046872,GO:0016702,|GO:0006633,GO:0031408,
DN119204_c1_g7_i5	UP	[GO:0016791:phosphatase activity][GO:0005975:carbohydrate metabolic process]	[ko00710:Carbon fixation in photosynthetic organisms]	-	XP_021769134.1:sedoheptulose1,7bisphosphatase, chloroplastic [Chenopodium quinoa]	gi|2529375|gb|L76556.1|SPIS17B:Spinacia oleracea chloroplast sedoheptulose-1,7-bisphosphatase mRNA, complete cds	A0A0J8D260|A0A0J8D260_BETVU:Uncharacterized protein;OS=Beta vulgaris subsp. vulgaris.;|GO:0016791,|GO:0005975,
DN123027_c1_g3_i1	UP	-	-	-	XP_009335445.1:PREDICTED: uncharacterized protein LOC103928158 [Pyrus x bretschneideri]	-	M5WPQ5|M5WPQ5_PRUPE:Uncharacterized protein;OS=Prunus persica;

geneid	trend	GO	Pathway	TF	NR	NT	Uniprot
DN127439_c0_g1_i5	UP	[GO:0008270:zinc ion binding]	-	-	XP_010695111.1:PREDICTED: uncharacterized protein LOC104907813 [Beta vulgaris subsp. vulgaris] >KMT18815.1 hypothetical protein BVRB_2g029230 [Beta vulgaris subsp. vulgaris]	gi|359487301|ref|XM_002274402.2|:PREDICTED: Vitis vinifera uncharacterized LOC100249974 (LOC100249974), mRNA	A0A0J8CYI0|A0A0J8CYI0_BETVU:Uncharacterized protein;OS=Beta vulgaris subsp. vulgaris.;|GO:0008270,
DN115131_c0_g1_i3	UP	[GO:0009535:chloroplast thylakoid membrane][GO:0031977:thylakoid lumen][GO:0006979:response to oxidative stress]	-	-	XP_010674669.1:PREDICTED: chloroplastic lipocalin [Beta vulgaris subsp. vulgaris] >KMT13688.1 hypothetical protein BVRB_4g080700 [Beta vulgaris subsp. vulgaris]	gi|521517872|gb|GAHZ01015104.1|:TSA: Salicornia europaea Unigene15792_SeCKS transcribed RNA sequence	A0A0J8CNS8|A0A0J8CNS8_BETVU:Uncharacterized protein;OS=Beta vulgaris subsp. vulgaris.;|GO:0009535,GO:0031977,|GO:0006979,
DN111802_c0_g1_i1	UP	-	-	-	-	-	-

geneid	trend	GO	Pathway	TF	NR	NT	Uniprot
DN122356_c0_g1_i1	UP	[GO:0005634:nucleus][GO:0004842:ubiquitin-protein transferase activity][GO:0008270:zinc ion binding][GO:0031347:regulation of defense response]	-	Zosma25g00280.1:Zostera marina|B3|B3 family protein	KNA13187.1:hypothetical protein SOVF_119030, partial [Spinacia oleracea]	-	A0A0K9R319|A0A0K9R319_SPIOL:Uncharacterized protein;OS=Spinacia oleracea;|GO:0005634,|GO:0004842,GO:0008270,|GO:0031347,
DN126707_c4_g4_i2	UP	-	-	-	-	-	-
DN124932_c0_g2_i11	UP	-	[ko04141:Protein processing in endoplasmic reticulum]	WALNUT_00030998-RA:Juglans regia|NF-YB|NF-YB family protein	XP_021743706.1:22.0 kDa class IV heat shock proteinlike [Chenopodium quinoa]	-	A0A0K9RYH1|A0A0K9RYH1_SPIOL: Uncharacterized protein;OS=Spinacia oleracea;
DN124851_c2_g14_i1	UP	[GO:0016021:integral component of membrane][GO:0005524:ATP binding][GO:0004674:protein serine/threonine kinase activity]	-	-	KNA13056.1:hypothetical protein SOVF_120350 [Spinacia oleracea]	-	A0A0K9R0L3|A0A0K9R0L3_SPIOL:Uncharacterized protein;OS=Spinacia oleracea;|GO:0016021,|GO:0005524,GO:0004674,
DN129093_c9_g17_i1	UP	-	-	-	-	-	-

geneid	trend	GO	Pathway	TF	NR	NT	Uniprot
DN117689_c1_g1_i2	UP	[GO:0016021:integral component of membrane][GO:0016787:hydrolase activity][GO:0006629:lipid metabolic process]	-	-	XP_021768782.1:uncharacterized protein LOC110733070 [Chenopodium quinoa]	gi|521735544|gb|GAIA01009137.1|:TSA: Salicornia europaea Unigene9496_Se200S transcribed RNA sequence	A0A0J8C8K8|A0A0J8C8K8_BETVU:Uncharacterized protein;OS=Beta vulgaris subsp. vulgaris.;|GO:0016021,|GO:0016787,|GO:0006629,

geneid	trend	GO	Pathway	TF	NR	NT	Uniprot
DN128069_c 2_g4_i2	UP	[GO:0016021:integral component of membrane][GO:0005215:transporter activity][GO:0009926:auxin polar transport][GO:0051301:cell division][GO:0052544:defense response by callose deposition in cell wall][GO:0042742:defense response to bacterium][GO:0050832:defense response to fungus][GO:0001736:establishment of planar polarity][GO:0010150:leaf senescence][GO:0031348:negative regulation of defense response][GO:0009789:positive regulation of abscisic acid-activated signaling pathway][GO:0010119:regulation of stomatal movement][GO:0009408:response to heat][GO:0002237:response to molecule of bacterial origin][GO:0009651:response to salt stress][GO:0048765:root hair cell differentiation][GO:0001944:vasculat ure development]	[ko04016:MAPK signaling pathway - plant][ko04075:Plan t hormone signal transduction]	-	XP_021866106.1:et hyleneinsensitive protein 2 isoform X1 [Spinacia oleracea] >XP_021866107.1 ethyleneinsensitive protein 2 isoform X1 [Spinacia oleracea]	gi|312986088|gb|HQ 441183.1|:Dianthus caryophyllus ethylene insensitive (EIN2) mRNA, complete cds	A0A0J8B2A2|A0A0 J8B2A2_BETVU:U ncharacterized protein;OS=Beta vulgaris subsp. vulgaris.;|GO:00160 21,|GO:0005215,|G O:0009926,GO:0051 301,GO:0052544,G O:0042742,GO:0050 832,GO:0001736,G O:0010150,GO:0031 348,GO:0009789,G O:0010119,GO:0009 408,GO:0002237,G O:0009651,GO:0048 765,GO:0001944,

geneid	trend	GO	Pathway	TF	NR	NT	Uniprot
DN122566_c3_g1_i4	UP	[GO:0005524:ATP binding][GO:0004674:protein serine/threonine kinase activity]	[ko04064:NF-kappa B signaling pathway][ko04620:Toll-like receptor signaling pathway][ko04621:NOD-like receptor signaling pathway][ko04624:Toll and Imd signaling pathway][ko04722:Neurotrophin signaling pathway][ko05133:Pertussis][ko05140:Leishmaniasis][ko05142:Chagas disease (American trypanosomiasis)][ko05145:Toxoplasmosis][ko05152:Tuberculosis][ko05162:Measles][ko05164:Influenza A]	EMT28879:Aegilops tauschii|ERF|ERF family protein	XP_010675411.1:PREDICTED: putative serine/threonineprotein kinase isoform X1 [Beta vulgaris subsp. vulgaris]	gi|502089246|ref|XM_004488795.1|:PREDICTED: Cicer arietinum probable leucine-rich repeat receptor-like serine/threonine-protein kinase At3g14840-like (LOC101506238), transcript variant X3, mRNA	A0A067K8F1|A0A067K8F1_JATCU:Uncharacterized protein;OS=Jatropha curcas;|GO:0005524,GO:0004674,

geneid	trend	GO	Pathway	TF	NR	NT	Uniprot
DN125274_c1_g8_i2	UP	[GO:0000287:magnesium ion binding][GO:0010333:terpene synthase activity][GO:0008152:metabolic process]	-	Cotton_A_17926_BGI-A2_v1.0:Gossypium arboreum|GRAS|GRAS family protein	XP_010694277.1:PREDICTED: probable sesquiterpene synthase [Beta vulgaris subsp. vulgaris] >KMS98542.1 hypothetical protein BVRB_4g092310 [Beta vulgaris subsp. vulgaris]	-	A0A0J8BEZ1|A0A0J8BEZ1_BETVU:Uncharacterized protein;OS=Beta vulgaris subsp. vulgaris.;|GO:0000287,GO:0010333,|GO:0008152,
DN126027_c2_g2_i2	UP	[GO:0050660:flavin adenine dinucleotide binding][GO:0016614:oxidoreductase activity, acting on CH-OH group of donors]	-	CCG025416.1:Populus euphratica|G2-like|G2-like family protein	XP_021844452.1:protein HOTHEADlike [Spinacia oleracea] >KNA23641.1 hypothetical protein SOVF_023020 [Spinacia oleracea]	-	A0A0K9RVV0|A0A0K9RVV0_SPIOL: Uncharacterized protein;OS=Spinacia oleracea;|GO:0050660,GO:0016614,
DN123216_c2_g7_i7	UP	[GO:0005737:cytoplasm][GO:0005634:nucleus][GO:0000977:RNA polymerase II regulatory region sequence-specific DNA binding][GO:0003700:DNA-binding	-	Bv1_007680_jyqa.t1:Beta vulgaris|MIKC_MADS|MIKC_MADS family protein	XP_021740351.1:MADSbox protein AGL24like isoform X1 [Chenopodium quinoa]	gi|55792843|gb|AY643733.1|:Solanum tuberosum MADS16 (MADS16) mRNA, complete cds	A0A0J8D685|A0A0J8D685_BETVU:Uncharacterized protein;OS=Beta vulgaris subsp.

geneid	trend	GO	Pathway	TF	NR	NT	Uniprot
		transcription factor activity][GO:0048438:floral whorl development][GO:0010077:maintenance of inflorescence meristem identity][GO:0000165:MAPK cascade][GO:0045944:positive regulation of transcription by RNA polymerase II][GO:0000060:obsolete protein import into nucleus, translocation][GO:0048510:regulation of timing of transition from vegetative to reproductive phase][GO:0006351:transcription, DNA-templated]					vulgaris.;|GO:0005737,GO:0005634,|GO:0000977,GO:0003700,|GO:0048438,GO:0010077,GO:0000165,GO:0045944,GO:0000060,GO:0048510,GO:0006351,
DN125805_c2_g5_i2	UP	[GO:0016021:integral component of membrane]	-	Aradu.QK1RA:Arachis duranensis|C2H2|C2H2 family protein	KNA19156.1:hypothetical protein SOVF_064200 [Spinacia oleracea]	gi|521445153|gb|GAHZ01075331.1|:TSA: Salicornia europaea Unigene92155_SeCKS transcribed RNA sequence	A0A0K9RI29|A0A0K9RI29_SPIOL:Uncharacterized protein;OS=Spinacia oleracea;|GO:0016021,

geneid	trend	GO	Pathway	TF	NR	NT	Uniprot
DN121315_c0_g2_i1	UP	[GO:0009570:chloroplast stroma][GO:0009534:chloroplast thylakoid][GO:0044238:primary metabolic process]	-	-	XP_021763081.1:ribosomebinding factor PSRP1, chloroplastic [Chenopodium quinoa]	gi|521742540|gb|GAIA01004898.1|:TSA: Salicornia europaea Unigene5184_Se200S transcribed RNA sequence	A0A0J8B8J2|A0A0J8B8J2_BETVU:Uncharacterized protein;OS=Beta vulgaris subsp. vulgaris.;|GO:0009570,GO:0009534,|GO:0044238,
DN127497_c4_g1_i12	UP	[GO:0003824:catalytic activity][GO:0008152:metabolic process]	[ko00061:Fatty acid biosynthesis][ko00071:Fatty acid degradation][ko02024:Quorum sensing][ko03320:PPAR signaling pathway][ko04146:Peroxisome][ko04216:Ferroptosis][ko04920:Adipocytokine signaling pathway]	TRIUR3_12546-P1: Triticum urartu|WRKY|WRKY family protein	XP_021836797.1:long chain acylCoA synthetase 1like [Spinacia oleracea]	gi|521696965|gb|GAIA01019096.1|:TSA: Salicornia europaea Unigene20056_Se200S transcribed RNA sequence	A0A0J8C8V6|A0A0J8C8V6_BETVU:Uncharacterized protein;OS=Beta vulgaris subsp. vulgaris.;|GO:0003824,|GO:0008152,

geneid	trend	GO	Pathway	TF	NR	NT	Uniprot
DN125473_c0_g1_i16	UP	[GO:0008270:zinc ion binding]	-	-	XP_010694486.1:PREDICTED: DNAdependent metalloprotease WSS1 [Beta vulgaris subsp. vulgaris] >XP_010694487.1 PREDICTED: DNAdependent metalloprotease WSS1 [Beta vulgaris subsp. vulgaris] >XP_019108113.1 PREDICTED: DNAdependent metalloprotease WSS1 [Beta vulgaris subsp. vulgaris] >KMS98332.1 hypothetical protein BVRB_4g093460 [Beta vulgaris subsp. vulgaris]	gi|521492891|gb|GAHZ01037995.1|:TSA: Salicornia europaea Unigene42861_SeCKS transcribed RNA sequence	A0A0J8BEE3|A0A0J8BEE3_BETVU:Uncharacterized protein;OS=Beta vulgaris subsp. vulgaris.;|GO:0008270,

geneid	trend	GO	Pathway	TF	NR	NT	Uniprot
DN126612_c1_g3_i14	UP	[GO:0016021:integral component of membrane]	-	-	XP_010688396.1:PREDICTED: uncharacterized protein LOC104902347 [Beta vulgaris subsp. vulgaris]	gi|359484327|ref|XM_002280143.2|:PREDICTED: Vitis vinifera uncharacterized LOC100252538 (LOC100252538), mRNA	A0A0J8BPR9|A0A0J8BPR9_BETVU:Uncharacterized protein;OS=Beta vulgaris subsp. vulgaris.;|GO:0016021,
DN112872_c0_g1_i1	UP	[GO:0016021:integral component of membrane][GO:0005886:plasma membrane][GO:0005524:ATP binding][GO:0000287:magnesium ion binding][GO:0004012:phospholipid-translocating ATPase activity]	-	-	XP_021759697.1:phospholipidtransporting ATPase 1like [Chenopodium quinoa]	gi|521450299|gb|GAHZ01070185.1|:TSA: Salicornia europaea Unigene85626_SeCKS transcribed RNA sequence	A0A0J8B7L3|A0A0J8B7L3_BETVU:Phospholipid-transporting ATPase;OS=Beta vulgaris subsp. vulgaris.;|GO:0016021,GO:0005886,|GO:0005524,GO:0000287,GO:0004012,
DN110360_c0_g1_i1	UP	[GO:0009061:anaerobic respiration][GO:0006979:response to oxidative stress]	-	RrC4426_p1:Raphanus raphanistrum|Trihelix|Trihelix family protein	XP_010683892.1:PREDICTED: uncharacterized protein LOC104898501 [Beta vulgaris subsp. vulgaris] >KMT06360.1	-	A0A0J8BXU2|A0A0J8BXU2_BETVU: Uncharacterized protein;OS=Beta vulgaris subsp. vulgaris.;|GO:0009061,GO:0006979,

geneid	trend	GO	Pathway	TF	NR	NT	Uniprot
					hypothetical protein BVRB_7g160420 [Beta vulgaris subsp. vulgaris]		
DN125271_c2_g4_i1	UP	-	-	-	XP_021715431.1:uncharacterized protein LOC110683384 [Chenopodium quinoa]	-	A0A0J8BYU2|A0A0J8BYU2_BETVU: Uncharacterized protein;OS=Beta vulgaris subsp. vulgaris.;
DN121180_c2_g1_i2	UP	[GO:0048046:apoplast][GO:0005618:cell wall][GO:0004553:hydrolase activity, hydrolyzing O-glycosyl compounds][GO:0016762:xyloglucan:xyloglucosyl transferase activity][GO:0071555:cell wall organization][GO:0006073:cellular glucan metabolic process]	-	Lus10007348:Linum usitatissimum|MYB_related|MYB_related family protein	AAS77347.1:sadtomato protein, partial [Capsicum annuum]	gi|359485717|ref|XM_002281538.2|:PREDICTED: Vitis vinifera xyloglucan endotransglucosylase/hydrolase protein 9-like (LOC100248328), mRNA	Q6R5L6|Q6R5L6_CAPAN:Xyloglucan endotransglucosylase/hydrolase;OS=Capsicum annuum;|GO:0048046,GO:0005618,|GO:0004553,GO:0016762,|GO:0071555,GO:0006073,
DN119739_c1_g6_i8	UP	[GO:0016021:integral component of membrane][GO:0070469:respirasome][GO:0009916:alternative oxidase activity][GO:0046872:metal ion binding]	-	-	XP_010677502.1:PREDICTED: ubiquinol oxidase 2, mitochondriallike [Beta vulgaris subsp.	gi|242384781|emb|FP093028.1|:Phyllostachys edulis cDNA clone: bphyem122a05, full	A0A0J8CD04|A0A0J8CD04_BETVU:Ubiquinol oxidase;OS=Beta vulgaris subsp.

geneid	trend	GO	Pathway	TF	NR	NT	Uniprot
					vulgaris]	insert sequence	vulgaris.;|GO:0016021,GO:0070469,|GO:0009916,GO:0046872,
DN119237_c1_g1_i1	UP	[GO:0016021:integral component of membrane][GO:0005524:ATP binding][GO:0004674:protein serine/threonine kinase activity]	[ko04064:NF-kappa B signaling pathway][ko04620:Toll-like receptor signaling pathway][ko04624:Toll and Imd signaling pathway][ko04722:Neurotrophin signaling pathway][ko05133:Pertussis][ko05140:Leishmaniasis][ko05142:Chagas disease (American trypanosomiasis)][ko05145:Toxoplasmosis][ko05152:Tuberculosis][ko05162:Measles][ko05169:Epst	Aco009877.1:Ananas comosus|WRKY|WRKY family protein	XP_010690948.1:PREDICTED: putative receptorlike protein kinase At3g47110 isoform X1 [Beta vulgaris subsp. vulgaris] >XP_019107571.1 PREDICTED: putative receptorlike protein kinase At3g47110 isoform X1 [Beta vulgaris subsp. vulgaris] >XP_019107572.1 PREDICTED: putative receptorlike protein kinase At3g47110 isoform X1 [Beta vulgaris subsp. vulgaris]	-	A0A0J8BK63|A0A0J8BK63_BETVU:Uncharacterized protein;OS=Beta vulgaris subsp. vulgaris.;|GO:0016021,|GO:0005524,GO:0004674,

geneid	trend	GO	Pathway	TF	NR	NT	Uniprot
			ein-Barr virus infection]		>XP_019107573.1 PREDICTED: putative receptorlike protein kinase At3g47110 isoform X1 [Beta vulgaris subsp. vulgaris] >XP_019107574.1 PREDICTED: putative receptorlike protein kinase At3g47110 isoform X1 [Beta vulgaris subsp. vulgaris]		
DN125073_c4_g1_i7	UP	[GO:0016021:integral component of membrane]	-	Achn185971:Actinidia chinensis|SBP|SBP family protein	XP_021770841.1:sugar transporter ERD6like 16 [Chenopodium quinoa]	-	A0A0J8B4N7|A0A0J8B4N7_BETVU:Uncharacterized protein;OS=Beta vulgaris subsp. vulgaris.;|GO:0016021,|GO:0022891,

geneid	trend	GO	Pathway	TF	NR	NT	Uniprot
DN124560_c0_g1_i6	UP	[GO:0016021:integral component of membrane][GO:0005506:iron ion binding][GO:0016491:oxidoreductase activity][GO:0006633:fatty acid biosynthetic process]	[ko00906:Carotenoid biosynthesis]	-	XP_021726730.1:betacarotene hydroxylase 2, chloroplasticlike [Chenopodium quinoa]	gi|350538546|ref|NM_001247419.1|:Solanum lycopersicum beta-carotene hydroxylase (CrtR-b1), mRNA >gi|5870597|emb|Y14809.1| Lycopersicon esculentum mRNA for beta-carotene hydroxylase (CrtR-b1 gene)	A0A0K9RTT3|A0A0K9RTT3_SPIOL:Uncharacterized protein;OS=Spinacia oleracea;|GO:0016021,|GO:0005506,GO:0016491,|GO:0006633,
DN124716_c1_g3_i5	UP	[GO:0004591:oxoglutarate dehydrogenase (succinyl-transferring) activity][GO:0030976:thiamine pyrophosphate binding][GO:0006099:tricarboxylic acid cycle]	[ko00020:Citrate cycle (TCA cycle)][ko00310:Lysine degradation][ko00380:Tryptophan metabolism]	-	XP_021771281.1:2oxoglutarate dehydrogenase, mitochondriallike [Chenopodium quinoa]	gi|512395007|gb|GAJZ01006163.1|:TSA: Prunus avium Pa_17809 transcribed RNA sequence	A0A059BCG3|A0A059BCG3_EUCGR: Uncharacterized protein;OS=Eucalyptus grandis;|GO:0004591,GO:0030976,|GO:0006099,

geneid	trend	GO	Pathway	TF	NR	NT	Uniprot
DN127788_c0_g1_i3	UP	-	-	-	XP_021835084.1:uncharacterized protein LOC110774804 isoform X1 [Spinacia oleracea] >KNA25020.1 hypothetical protein SOVF_010290 isoform B [Spinacia oleracea]	-	A0A0K9RZU0|A0A0K9RZU0_SPIOL: Uncharacterized protein;OS=Spinacia oleracea;
DN119569_c1_g5_i7	UP	-	-	-	-	-	-
DN118328_c0_g1_i5	UP	[GO:0003677:DNA binding][GO:0003887:DNA-directed DNA polymerase activity][GO:0006260:DNA replication]	-	-	XP_021776129.1:uncharacterized protein LOC110739953 [Chenopodium quinoa]	-	A0A0J8EER8|A0A0J8EER8_BETVU:Uncharacterized protein;OS=Beta vulgaris subsp. vulgaris.;|GO:0003677,GO:0003887,|GO:0006260,

geneid	trend	GO	Pathway	TF	NR	NT	Uniprot
DN122817_c2_g2_i4	UP	[GO:0009330:DNA topoisomerase complex (ATP-hydrolyzing)][GO:0005634:nucleus][GO:0005524:ATP binding][GO:0003677:DNA binding][GO:0003918:DNA topoisomerase type II (ATP-hydrolyzing) activity][GO:0006265:DNA topological change]	-	-	EEF48847.1:Type II DNA topoisomerase VI subunit B, putative [Ricinus communis]	gi|521527106|gb|GAHZ01009834.1|:TSA: Salicornia europaea Unigene10381_SeCKS transcribed RNA sequence	B9RI75|B9RI75_RICCO:DNA topoisomerase 6 subunit B;OS=Ricinus communis;|GO:0009330,GO:0005634,|GO:0005524,GO:0003677,GO:0003918,|GO:0006265,
DN129093_c7_g3_i1	UP	[GO:0005524:ATP binding][GO:0004672:protein kinase activity]	-	Aco009877.1:Ananas comosus|WRKY|WRKY family protein	KDP44790.1:hypothetical protein JCGZ_01290 [Jatropha curcas]	gi|255581222|ref|XM_002531378.1|:Ricinus communis receptor-kinase, putative, mRNA	A0A067LJH4|A0A067LJH4_JATCU:Uncharacterized protein;OS=Jatropha curcas;|GO:0005524,GO:0004672,

geneid	trend	GO	Pathway	TF	NR	NT	Uniprot
DN127002_c2_g4_i5	UP	[GO:0008270:zinc ion binding]	-	Sp_131580_yrmf.t1:Spinacia oleracea|C3H|C3H family protein	XP_021860995.1:putative RINGtype E3 ubiquitin transferase C3H69 [Spinacia oleracea] >XP_021860996.1 putative RINGtype E3 ubiquitin transferase C3H69 [Spinacia oleracea] >KNA11798.1 hypothetical protein SOVF_131580 [Spinacia oleracea]	gi|359487282|ref|XM_002279846.2|:PREDICTED: Vitis vinifera E3 ubiquitin-protein ligase makorin-like (LOC100243143), mRNA	A0A0K9QYM6|A0A0K9QYM6_SPIOL:Uncharacterized protein;OS=Spinacia oleracea;|GO:0008270,
DN96730_c0_g1_i1	UP	-	-	KN538740.1_FGP057:Oryza longistaminata|C3H|C3H family protein	XP_021723995.1:uncharacterized protein LOC110691378 [Chenopodium quinoa]	-	A0A0K9RA55|A0A0K9RA55_SPIOL:Uncharacterized protein;OS=Spinacia oleracea;
DN103687_c0_g1_i1	UP	[GO:0043565:sequence-specific DNA binding][GO:0003700:DNA-binding transcription factor activity]	-	GSVIVT01010152001:Vitis vinifera|bZIP|bZIP family protein	XP_002269495.1:PREDICTED: basic leucine zipper 43 [Vitis vinifera] >CBI27427.3 unnamed protein	-	A5BFE4|A5BFE4_VITVI:Putative uncharacterized protein;OS=Vitis vinifera;|GO:0043565,GO:0003700,

geneid	trend	GO	Pathway	TF	NR	NT	Uniprot
					product, partial [Vitis vinifera]		
DN128682_c2_g3_i1	UP	[GO:0046872:metal ion binding][GO:0016702:oxidoreductase activity, acting on single donors with incorporation of molecular oxygen, incorporation of two atoms of oxygen][GO:0006633:fatty acid biosynthetic process][GO:0031408:oxylipin biosynthetic process]	[ko00591:Linoleic acid metabolism][ko00592:alpha-Linolenic acid metabolism]	-	XP_021772004.1:linoleate 13Slipoxygenase 21, chloroplasticlike [Chenopodium quinoa]	gi|521567668|gb|GAIA01074515.1|:TSA: Salicornia europaea Unigene90436_Se200S transcribed RNA sequence	W9QY99|W9QY99_9ROSA:Lipoxygenase;OS=Morus notabilis.;|GO:0046872,GO:0016702,|GO:0006633,GO:0031408,
DN114876_c0_g1_i4	UP	[GO:0005634:nucleus][GO:0003677:DNA binding][GO:0006355:regulation of transcription, DNA-templated][GO:0006351:transcription, DNA-templated]	-	Neem_6492_f_1:Azadirachta indica|NAC|NAC family protein	XP_010682089.1:PREDICTED: NAC domaincontaining protein 35 [Beta vulgaris subsp. vulgaris] >KMT07858.1 hypothetical protein BVRB_6g145550 [Beta vulgaris subsp. vulgaris]	gi|449478298|ref|XM_004155229.1|:PREDICTED: Cucumis sativus protein FEZ-like (LOC101216775), mRNA	A0A0K9RMX9|A0A0K9RMX9_SPIOL:Uncharacterized protein;OS=Spinacia oleracea;|GO:0005634,|GO:0003677,|GO:0006355,GO:0006351,

geneid	trend	GO	Pathway	TF	NR	NT	Uniprot
DN116755_c2_g3_i1	UP	-	-	-	XP_010669376.1:PREDICTED: uncharacterized protein LOC104886611 [Beta vulgaris subsp. vulgaris]	-	A0A0J8CW27|A0A0J8CW27_BETVU: Uncharacterized protein;OS=Beta vulgaris subsp. vulgaris.;
DN114643_c1_g1_i7	UP	[GO:0005634:nucleus][GO:0000977:RNA polymerase II regulatory region sequence-specific DNA binding][GO:0003700:DNA-binding transcription factor activity][GO:0000165:MAPK cascade][GO:0045944:positive regulation of transcription by RNA polymerase II][GO:0006351:transcription, DNA-templated]	-	Sp_023090_ietn.t2:Spinacia oleracea|MIKC_MADS|MIKC_MADS family protein	XP_021754069.1:MADSbox protein SOC1like isoform X2 [Chenopodium quinoa]	gi|285818378|gb|AY830920.2|:Pisum sativum SUPPRESSOR OF OVEREXPRESSION OF CONSTANS 1 a (SOC1a) mRNA, complete cds	A0A0K9RXL2|A0A0K9RXL2_SPIOL: Uncharacterized protein;OS=Spinacia oleracea;|GO:0005634,|GO:0000977,GO:0003700,|GO:0000165,GO:0045944,GO:0006351,
DN128827_c3_g1_i13	UP	-	-	-	XP_021854764.1:uncharacterized protein LOC110794125 isoform X1 [Spinacia oleracea] >KNA15739.1 hypothetical protein	gi|225459553|ref|XM_002285817.1|:PREDICTED: Vitis vinifera long chain acyl-CoA synthetase 9, chloroplastic-like (LOC100265978),	A0A0K9R9S2|A0A0K9R9S2_SPIOL:Uncharacterized protein;OS=Spinacia oleracea;

geneid	trend	GO	Pathway	TF	NR	NT	Uniprot
					SOVF_095500 isoform A [Spinacia oleracea]	mRNA	
DN120439_c3_g1_i4	UP	-	-	-	XP_010102559.1:hypothetical protein L484_018031 [Morus notabilis] >EXB93645.1 hypothetical protein L484_018031 [Morus notabilis]	-	W9RII0|W9RII0_9ROSA:Uncharacterized protein;OS=Morus notabilis.;
DN125916_c1_g4_i7	UP	[GO:0016021:integral component of membrane]	-	-	XP_010691117.1:PREDICTED: probable leucinerich repeat receptorlike protein kinase At1g35710 isoform X1 [Beta vulgaris subsp. vulgaris]	-	A0A0J8ECA8|A0A0J8ECA8_BETVU:Uncharacterized protein;OS=Beta vulgaris subsp. vulgaris.;|GO:0016021,

geneid	trend	GO	Pathway	TF	NR	NT	Uniprot
DN120438_c2_g1_i2	UP	[GO:0005737:cytoplasm][GO:0005524:ATP binding][GO:0016485:protein processing][GO:0009408:response to heat]	[ko04213:Longevity regulating pathway - multiple species]	Achn116231:Actinidia chinensis|ARF|ARF family protein	XP_010670181.1:PREDICTED: chaperone protein ClpB3, chloroplastic [Beta vulgaris subsp. vulgaris] >KMT17275.1 hypothetical protein BVRB_2g039750 [Beta vulgaris subsp. vulgaris]	gi|521452409|gb|GAHZ01068075.1|:TSA: Salicornia europaea Unigene82161_SeCKS transcribed RNA sequence	A0A0J8CU40|A0A0J8CU40_BETVU:Uncharacterized protein;OS=Beta vulgaris subsp. vulgaris.;|GO:0005737,|GO:0005524,|GO:0016485,GO:0009408,
DN117742_c2_g2_i3	UP	[GO:0009507:chloroplast][GO:0005739:mitochondrion][GO:0005525:GTP binding]	-	mrna03235.1-v1.0-hybrid:Fragaria vesca|ARR-B|ARR-B family protein	XP_010672868.1:PREDICTED: short integuments 2, mitochondrial [Beta vulgaris subsp. vulgaris]	gi|521605377|gb|GAIA01061875.1|:TSA: Salicornia europaea Unigene73785_Se200S transcribed RNA sequence	A0A0J8CSY7|A0A0J8CSY7_BETVU:Uncharacterized protein;OS=Beta vulgaris subsp. vulgaris.;|GO:0009507,GO:0005739,|GO:0005525,
DN127880_c1_g4_i1	UP	[GO:0003677:DNA binding]	-	AHYPO_015113-RA:Amaranthus hypochondriacus|ZF-HD|ZF-HD family protein	XP_021746683.1:zincfinger homeodomain protein 8like [Chenopodium quinoa]	-	A0A0J8C4D5|A0A0J8C4D5_BETVU:Uncharacterized protein;OS=Beta vulgaris subsp. vulgaris.;|GO:00036

geneid	trend	GO	Pathway	TF	NR	NT	Uniprot
							77,
DN123363_c0_g3_i5	UP	-	-	-	-	-	-
DN113430_c0_g1_i1	UP	-	-	AHYPO_011234-RA:Amaranthus hypochondriacus|Nin-like|Nin-like family protein	XP_021720213.1:protein NLP9like [Chenopodium quinoa] >XP_021720219.1 protein NLP9like [Chenopodium quinoa] >XP_021720226.1 protein NLP9like [Chenopodium quinoa]	-	A0A061EIU7|A0A061EIU7_THECC:Plant regulator RWP-RK family protein, putative isoform 3;OS=Theobroma cacao;
DN113491_c0_g2_i1	UP	-	-	-	XP_010690149.1:PREDICTED: protein GOS9like [Beta vulgaris subsp. vulgaris]	-	A0A0K9QBJ0|A0A0K9QBJ0_SPIOL:Uncharacterized protein;OS=Spinacia oleracea;

geneid	trend	GO	Pathway	TF	NR	NT	Uniprot
DN126651_c3_g3_i10	UP	[GO:0008168:methyltransferase activity]	-	-	XP_010673295.1:PREDICTED: salicylate carboxymethyltransferaselike [Beta vulgaris subsp. vulgaris]	-	A0A0J8CSG5|A0A0J8CSG5_BETVU:Uncharacterized protein;OS=Beta vulgaris subsp. vulgaris.;|GO:0008168,
DN126009_c1_g1_i9	UP	[GO:0016020:membrane][GO:0020037:heme binding][GO:0005506:iron ion binding][GO:0016709:oxidoreductase activity, acting on paired donors, with incorporation or reduction of molecular oxygen, NAD(P)H as one donor, and incorporation of one atom of oxygen][GO:0044550:secondary metabolite biosynthetic process]	-	Sme2.5_00297.1_g00019.1:Solanum melongena|MYB|MYB family protein	XP_021863527.1:cytochrome P450 71A26like isoform X1 [Spinacia oleracea]	-	D7SHG6|D7SHG6_VITVI:Putative uncharacterized protein;OS=Vitis vinifera;GN=VIT_17s0000g09510 {ECO:0000313|EMBL:CBI14925.3};|GO:0016020,|GO:0020037,GO:0005506,GO:0016709,|GO:0044550,
DN126399_c3_g2_i5	UP	[GO:0005524:ATP binding][GO:0003777:microtubule motor activity][GO:0007018:microtubule-based movement]	-	-	XP_021680924.1:kinesinlike protein KIN12F [Hevea brasiliensis]	-	Unreviewed|A0A0D3QSP8_TOBAC:Kinesin-related protein;OS=Nicotiana tabacum;|GO:00055

geneid	trend	GO	Pathway	TF	NR	NT	Uniprot
							24,GO:0003777,|GO:0007018,
DN126604_c3_g2_i2	UP	[GO:0004553:hydrolase activity, hydrolyzing O-glycosyl compounds][GO:0005975:carbohydrate metabolic process]	[ko00460:Cyanoamino acid metabolism][ko00500:Starch and sucrose metabolism][ko00940:Phenylpropanoid biosynthesis]	Lus10009218:Linum usitatissimum|C2H2|C2H2 family protein	XP_021719663.1:betaglucosidase 46like isoform X1 [Chenopodium quinoa]	gi|224078655|ref|XM_002305558.1|:Populus trichocarpa predicted protein, mRNA	A0A0K9QUL6|A0A0K9QUL6_SPIOL: Uncharacterized protein;OS=Spinacia oleracea;|GO:0004553,|GO:0005975,
DN113100_c2_g1_i2	UP	-	-	-	-	-	-
DN118803_c0_g2_i1	UP	-	-	-	-	-	-
DN122353_c2_g1_i4	UP	-	-	-	XP_021769483.1:peptideN4(Nacetylbetaglucosaminyl)asparagine amidase A [Chenopodium quinoa]	-	A0A0K9QVL7|A0A0K9QVL7_SPIOL: Uncharacterized protein;OS=Spinacia oleracea;

geneid	trend	GO	Pathway	TF	NR	NT	Uniprot
DN128491_c1_g1_i4	UP	[GO:0005524:ATP binding][GO:0003676:nucleic acid binding]	[ko03040:Spliceosome]	-	XP_010670756.1:PREDICTED: DExHbox ATPdependent RNA helicase DExH12like [Beta vulgaris subsp. vulgaris]	gi|478166734|gb|GAHK01009869.1|:TSA: Jatropha curcas CL3885.Contig1_JC-CK_1A transcribed RNA sequence	A0A0J8CXP5|A0A0J8CXP5_BETVU:Uncharacterized protein;OS=Beta vulgaris subsp. vulgaris.;|GO:0005524,GO:0003676,
DN123784_c2_g1_i1	UP	-	[ko04141:Protein processing in endoplasmic reticulum]	WALNUT_00030998-RA:Juglans regia|NF-YB|NF-YB family protein	XP_021732682.1:18.1 kDa class I heat shock proteinlike [Chenopodium quinoa]	-	V4W3A2|V4W3A2_9ROSI:Uncharacterized protein;OS=Citrus clementina.;
DN122822_c1_g1_i7	UP	[GO:0016593:Cdc73/Paf1 complex][GO:0003677:DNA binding][GO:0016570:histone modification][GO:0006368:transcription elongation from RNA polymerase II promoter]	-	-	XP_010673218.1:PREDICTED: protein RTF1 homolog [Beta vulgaris subsp. vulgaris] >KMT14913.1 hypothetical protein BVRB_3g064270 [Beta vulgaris subsp. vulgaris]	gi|242083647|ref|XM_002442204.1|:Sorghum bicolor hypothetical protein, mRNA	A0A0J8CSG2|A0A0J8CSG2_BETVU:Uncharacterized protein;OS=Beta vulgaris subsp. vulgaris.;|GO:0016593,|GO:0003677,|GO:0016570,GO:0006368,

geneid	trend	GO	Pathway	TF	NR	NT	Uniprot
DN115026_c3_g2_i4	UP	[GO:0043565:sequence-specific DNA binding][GO:0003700:DNA-binding transcription factor activity]	-	Sp_190610_kzew.t1:Spinacia oleracea|WRKY|WRKY family protein	XP_021867482.1:probable WRKY transcription factor 70 [Spinacia oleracea] >KNA05388.1 hypothetical protein SOVF_190610 [Spinacia oleracea]	-	A0A0K9QFD5|A0A0K9QFD5_SPIOL:Uncharacterized protein;OS=Spinacia oleracea;|GO:0043565,GO:0003700,
DN102792_c1_g1_i1	UP	[GO:0003676:nucleic acid binding][GO:0015074:DNA integration]	-	PK03054.1:Cannabis sativa|HD-ZIP|HD-ZIP family protein	XP_006605035.1:PREDICTED: uncharacterized protein LOC102664868 [Glycine max]	-	A5BFR8|A5BFR8_VITVI:Putative uncharacterized protein;OS=Vitis vinifera;|GO:0003676,|GO:0015074,
DN113208_c4_g2_i1	UP	-	-	-	-	-	-
DN118851_c4_g2_i9	UP	[GO:0016021:integral component of membrane][GO:0005774:vacuolar membrane]	[ko04721:Synaptic vesicle cycle][ko04723:Retrograde endocannabinoid signaling][ko04727:GABAergic	-	XP_021745828.1:amino acid transporter AVT1Alike [Chenopodium quinoa]	-	A0A0J8CSZ0|A0A0J8CSZ0_BETVU:Uncharacterized protein;OS=Beta vulgaris subsp. vulgaris.;|GO:0016021,GO:0005774,

geneid	trend	GO	Pathway	TF	NR	NT	Uniprot
			synapse][ko05032: Morphine addiction][ko05033: Nicotine addiction]				
DN127197_c 1_g1_i4	UP	[GO:0016021:integral component of membrane][GO:0015297:antiporter activity]	-	Zpz_sc01010.1.g001 10.1.am.mk:Zoysia pacifica|MYB|MYB family protein	XP_021743366.1:pr otein DETOXIFICATION 24like isoform X1 [Chenopodium quinoa]	-	A0A0J8CB22|A0A0 J8CB22_BETVU:U ncharacterized protein;OS=Beta vulgaris subsp. vulgaris.;|GO:00160 21,|GO:0015297,GO :0015238,
DN127925_c 2_g1_i4	UP	[GO:0016021:integral component of membrane][GO:0005524:ATP binding][GO:0005388:calcium-transp orting ATPase activity][GO:0046872:metal ion binding]	-	Lus10040166:Linum usitatissimum|GRAS |GRAS family protein	XP_010687432.1:P REDICTED: calciumtransporting ATPase 10, plasma membranetype isoform X1 [Beta vulgaris subsp. vulgaris]	gi|349727145|emb|F Q382954.1|:Vitis vinifera clone SS0ABG37YK12	A0A0J8EL09|A0A0 J8EL09_BETVU:Ca lcium-transporting ATPase;OS=Beta vulgaris subsp. vulgaris.;|GO:00160 21,|GO:0005524,GO :0005388,GO:00468 72,

geneid	trend	GO	Pathway	TF	NR	NT	Uniprot
DN127603_c1_g1_i3	UP	[GO:0016021:integral component of membrane][GO:0055114:oxidation-reduction process]	-	678320713:Utricularia gibba|M-type_MADS|M-type_MADS family protein	XP_021845163.1:cytochrome b561 and DOMON domaincontaining protein At3g61750 [Spinacia oleracea] >KNA25964.1 hypothetical protein SOVF_001860 [Spinacia oleracea]	-	A0A0K9S3Y6|A0A0K9S3Y6_SPIOL:Uncharacterized protein;OS=Spinacia oleracea;|GO:0016021,|GO:0055114,
DN123481_c4_g1_i1	UP	[GO:0043531:ADP binding][GO:0006952:defense response]	-	XP_016498898.1:Nicotiana tabacum|bHLH|bHLH family protein	KNA23061.1:hypothetical protein SOVF_028320 [Spinacia oleracea]	-	A0A0K9RU44|A0A0K9RU44_SPIOL:Uncharacterized protein;OS=Spinacia oleracea;|GO:0043531,|GO:0006952,
DN118358_c5_g1_i5	UP	-	-	-	-	-	-

geneid	trend	GO	Pathway	TF	NR	NT	Uniprot
DN120779_c0_g1_i11	UP	[GO:0005829:cytosol][GO:0005774:vacuolar membrane][GO:0003924:GTPase activity]	-	Achn091111:Actinidia chinensis|G2-like|G2-like family protein	XP_010680443.1:PREDICTED: protein misato homolog 1 [Beta vulgaris subsp. vulgaris] >KMT09360.1 hypothetical protein BVRB_6g134540 [Beta vulgaris subsp. vulgaris]	gi|521625412|gb|GAIA01053160.1|:TSA: Salicornia europaea Unigene62303_Se200S transcribed RNA sequence	A0A0J8C6I6|A0A0J8C6I6_BETVU:Uncharacterized protein;OS=Beta vulgaris subsp. vulgaris.;|GO:0005829,GO:0005774,|GO:0003924,
DN123998_c4_g1_i1	UP	[GO:0046872:metal ion binding][GO:0016491:oxidoreductase activity]	[ko00270:Cysteine and methionine metabolism]	676709864:Sisymbrium irio|STAT|STAT family protein	XP_010686763.1:PREDICTED: 1aminocyclopropane 1carboxylate oxidase [Beta vulgaris subsp. vulgaris] >KMT04076.1 hypothetical protein BVRB_8g185300 [Beta vulgaris subsp. vulgaris]	gi|121488646|emb|AJ890086.1|:Prunus domestica subsp. insititia mRNA for 1-aminocyclopropane-1-carboxylate oxidase 1 (aco1 gene)	A0A0J8EM69|A0A0J8EM69_BETVU:Uncharacterized protein;OS=Beta vulgaris subsp. vulgaris.;|GO:0046872,GO:0016491,
DN122970_c0_g6_i3	UP	-	-	Zmw_sc01201.1.g00120.1:Zoysia matrella|C2H2|C2H2 family protein	XP_021758255.1:methylesterase 17like [Chenopodium quinoa]	-	M5X1P8|M5X1P8_PRUPE:Uncharacterized protein;OS=Prunus

geneid	trend	GO	Pathway	TF	NR	NT	Uniprot
							persica;
DN120359_c0_g2_i9	UP	[GO:0005524:ATP binding][GO:0016887:ATPase activity]	-	-	XP_021739774.1:ATPase ASNA1 homolog [Chenopodium quinoa]	gi|359486630|ref|XM_002271717.2|:PREDICTED: Vitis vinifera putative arsenical pump-driving ATPase-like (LOC100266785), mRNA	A0A0K9QIW8|A0A0K9QIW8_SPIOL: Uncharacterized protein;OS=Spinacia oleracea;|GO:0005524,GO:0016887,
DN125311_c4_g1_i11	UP	-	-	KN538758.1_FGP044:Oryza longistaminata|ARF|ARF family protein	XP_021720300.1:uncharacterized protein LOC110687953 isoform X1 [Chenopodium quinoa]	gi|359486270|ref|XM_002268123.2|:PREDICTED: Vitis vinifera uncharacterized LOC100262534 (LOC100262534), mRNA	A0A0J8C6R1|A0A0J8C6R1_BETVU:Uncharacterized protein;OS=Beta vulgaris subsp. vulgaris.;

geneid	trend	GO	Pathway	TF	NR	NT	Uniprot
DN117551_c6_g6_i6	UP	[GO:0005524:ATP binding][GO:0004674:protein serine/threonine kinase activity][GO:0007165:signal transduction]	[ko04068:FoxO signaling pathway][ko04140:Autophagy - animal][ko04150:mTOR signaling pathway][ko04151:PI3K-Akt signaling pathway][ko04152:AMPK signaling pathway][ko04211:Longevity regulating pathway][ko04213:Longevity regulating pathway - multiple species][ko04371:Apelin signaling pathway][ko04530:Tight junction][ko04710:Circadian rhythm][ko04910:Insulin signaling pathway][ko04920:Adipocytokine	Csa11g091210.1:Camelina sativa|bHLH|bHLH family protein	XP_021851427.1:CBLinteracting serine/threonineprotein kinase 1 [Spinacia oleracea] >KNA19803.1 hypothetical protein SOVF_058140 [Spinacia oleracea]	gi|357450322|ref|XM_003595390.1|:Medicago truncatula CBL-interacting protein kinase (MTR_2g045470) mRNA, complete cds	A0A0K9RJX8|A0A0K9RJX8_SPIOL:Non-specific serine/threonine protein kinase;OS=Spinacia oleracea;|GO:0005524,GO:0004674,|GO:0007165,

geneid	trend	GO	Pathway	TF	NR	NT	Uniprot
			signaling pathway][ko04921: Oxytocin signaling pathway][ko04922: Glucagon signaling pathway][ko04931:Insulin resistance][ko04932: Non-alcoholic fatty liver disease (NAFLD)][ko05410: Hypertrophic cardiomyopathy (HCM)][ko05418:Fluid shear stress and atherosclerosis]				
DN119941_c0_g1_i2	UP	[GO:0003676:nucleic acid binding][GO:0008270:zinc ion binding]	-	-	XP_015079427.1:PREDICTED: uncharacterized protein LOC107023294 [Solanum pennellii]	-	K4C139|K4C139_SOLLC:Uncharacterized protein;OS=Solanum lycopersicum;|GO:0003676,GO:0008270,

geneid	trend	GO	Pathway	TF	NR	NT	Uniprot
DN117469_c1_g2_i7	UP	[GO:0048046:apoplast][GO:0005618:cell wall][GO:0009570:chloroplast stroma][GO:0005829:cytosol][GO:0005739:mitochondrion][GO:0005886:plasma membrane][GO:0009506:plasmodesma][GO:0005774:vacuolar membrane][GO:0005507:copper ion binding][GO:0004807:triose-phosphate isomerase activity][GO:0044262:cellular carbohydrate metabolic process][GO:0006096:glycolytic process][GO:0009735:response to cytokinin]	[ko00010:Glycolysis / Gluconeogenesis][ko00051:Fructose and mannose metabolism][ko00562:Inositol phosphate metabolism][ko00710:Carbon fixation in photosynthetic organisms]	LPERR01G31650.1: Leersia perrieri|MYB|MYB family protein	XP_010690624.1:PREDICTED: triosephosphate isomerase, cytosolic [Beta vulgaris subsp. vulgaris] >KMT18318.1 hypothetical protein BVRB_2g024780 [Beta vulgaris subsp. vulgaris]	gi|521706646|gb|GAIA01014587.1|:TSA: Salicornia europaea Unigene15247_Se200S transcribed RNA sequence	A0A0J8D2A4|A0A0J8D2A4_BETVU:Uncharacterized protein;OS=Beta vulgaris subsp. vulgaris.;|GO:0048046,GO:0005618,GO:0009570,GO:0005829,GO:0005739,GO:0005886,GO:0009506,GO:0005774,|GO:0005507,GO:0004807,|GO:0044262,GO:0006096,GO:0009735,
DN115512_c1_g2_i6	UP	-	[ko00250:Alanine, aspartate and glutamate metabolism][ko00910:Nitrogen metabolism]	-	OAO96246.1:GLT1 [Arabidopsis thaliana]	gi|521549321|gb|GAIA01082847.1|:TSA: Salicornia europaea Unigene101125_Se200S transcribed RNA sequence	A0A178UQD5|A0A178UQD5_ARATH: GLT1;OS=Arabidopsis thaliana;GN=AXX17_At5g52410 {ECO:0000313|EMBL:OAO96246.1};

geneid	trend	GO	Pathway	TF	NR	NT	Uniprot
DN118488_c0_g8_i2	UP	[GO:0016020:membrane][GO:0020037:heme binding][GO:0005506:iron ion binding][GO:0016709:oxidoreductase activity, acting on paired donors, with incorporation or reduction of molecular oxygen, NAD(P)H as one donor, and incorporation of one atom of oxygen][GO:0044550:secondary metabolite biosynthetic process]	-	676766070:Sisymbrium irio|bHLH|bHLH family protein	XP_010671012.1:PREDICTED: cytochrome P450 83B1like [Beta vulgaris subsp. vulgaris]	gi|521509070|gb|GAHZ01021850.1|:TSA: Salicornia europaea Unigene23481_SeCKS transcribed RNA sequence	A5CBQ0|A5CBQ0_VITVI:Putative uncharacterized protein;OS=Vitis vinifera;GN=VIT_18s0001g13820{ECO:0000313|EMBL:CCB46013.1};|GO:0016020,|GO:0020037,GO:0005506,GO:0016709,|GO:0044550,
DN121806_c0_g8_i1	UP	[GO:0008234:cysteine-type peptidase activity]	-	FANhyb_rscf00000253.1.g00010.1:Fragaria x ananassa|BBR-BPC|BBR-BPC family protein	XP_021765962.1:zingipain2like [Chenopodium quinoa]	-	V5M3B7|V5M3B7_MIRJA:RCR3-like cysteine protease;OS=Mirabilis jalapa;|GO:0008234,

geneid	trend	GO	Pathway	TF	NR	NT	Uniprot
DN125814_c0_g1_i13	UP	[GO:0005634:nucleus][GO:0016818:hydrolase activity, acting on acid anhydrides, in phosphorus-containing anhydrides][GO:0003676:nucleic acid binding][GO:0008081:phosphoric diester hydrolase activity][GO:0008270:zinc ion binding][GO:0006281:DNA repair]	-	ONIVA05G13280.1:Oryza nivara|NAC|NAC family protein	XP_021865613.1:uncharacterized protein LOC110804338 isoform X3 [Spinacia oleracea]	gi|255539986|ref|XM_002511012.1|:Ricinus communis tyrosyl-DNA phosphodiesterase, putative, mRNA	A0A0K9QN35|A0A0K9QN35_SPIOL:Uncharacterized protein;OS=Spinacia oleracea;|GO:0005634,|GO:0016818,GO:0003676,GO:0008081,GO:0008270,|GO:0006281,
DN122449_c3_g2_i33	UP	-	-	-	XP_011089142.1:IQ domaincontaining protein IQM2 [Sesamum indicum]	-	K4ASE6|K4ASE6_SOLLC:Uncharacterized protein;OS=Solanum lycopersicum;
DN122250_c1_g1_i9	UP	[GO:0016021:integral component of membrane][GO:0005886:plasma membrane][GO:0046715:active borate transmembrane transporter activity][GO:0015168:glycerol transmembrane transporter activity][GO:0015204:urea transmembrane transporter activity]	-	ONIVA01G08700.1:Oryza nivara|BES1|BES1 family protein	XP_021740679.1:aquaporin NIP61like [Chenopodium quinoa]	gi|521632925|gb|GAIA01049970.1|:TSA: Salicornia europaea Unigene58423_Se200S transcribed RNA sequence	A0A0J8BI22|A0A0J8BI22_BETVU:Uncharacterized protein;OS=Beta vulgaris subsp. vulgaris.;|GO:0016021,GO:0005886,|GO:0046715,GO:0015168,GO:0015204,

geneid	trend	GO	Pathway	TF	NR	NT	Uniprot
DN120765_c2_g2_i8	UP	[GO:0005634:nucleus][GO:0003677:DNA binding][GO:0003700:DNA-binding transcription factor activity][GO:0007275:multicellular organism development][GO:0006351:transcription, DNA-templated]	-	Dca22443.1:Dianthus caryophyllus|AP2|AP2 family protein	XP_021744270.1:ethyleneresponsive transcription factor RAP27like isoform X5 [Chenopodium quinoa]	gi|224068643|ref|XM_002326128.1|:Populus trichocarpa AP2 domain-containing transcription factor (RAP6), mRNA	A0A0J8BEG5|A0A0J8BEG5_BETVU:Uncharacterized protein;OS=Beta vulgaris subsp. vulgaris.;|GO:0005634,|GO:0003677,GO:0003700,|GO:0007275,GO:0006351,
DN123587_c1_g1_i11	UP	[GO:0020037:heme binding][GO:0046872:metal ion binding][GO:0019825:oxygen binding]	-	-	XP_021850850.1:nonsymbiotic hemoglobin 1like [Spinacia oleracea] >KNA03363.1 hypothetical protein SOVF_209930 [Spinacia oleracea]	gi|521750151|gb|GAIA01001073.1|:TSA: Salicornia europaea Unigene1095_Se200S transcribed RNA sequence	A0A0K9Q9J4|A0A0K9Q9J4_SPIOL:Uncharacterized protein;OS=Spinacia oleracea;|GO:0020037,GO:0046872,GO:0019825,
DN125872_c1_g2_i1	UP	[GO:0016020:membrane][GO:0003830:beta-1,4-mannosylglycoprotein 4-beta-N-acetylglucosaminyltransferase activity][GO:0006487:protein N-linked glycosylation]	[ko00510:N-Glycan biosynthesis]	Do024028.1:Dichanthelium oligosanthes|NAC|NAC family protein	XP_021852768.1:beta1,4mannosylglycoprotein 4betaNacetylglucosaminyltransferase [Spinacia oleracea] >KNA11801.1 hypothetical protein	gi|502151496|ref|XM_004508413.1|:PREDICTED: Cicer arietinum uncharacterized LOC101501638 (LOC101501638), mRNA	A0A0K9QZ45|A0A0K9QZ45_SPIOL:Uncharacterized protein;OS=Spinacia oleracea;|GO:0016020,|GO:0003830,|GO:0006487,

geneid	trend	GO	Pathway	TF	NR	NT	Uniprot
					SOVF_131810 [Spinacia oleracea]		
DN124180_c1_g9_i2	UP	[GO:0016021:integral component of membrane][GO:0005524:ATP binding][GO:0004674:protein serine/threonine kinase activity]	-	-	XP_019107575.1:PREDICTED: probable LRR receptorlike serine/threonineprotein kinase At3g47570 isoform X2 [Beta vulgaris subsp. vulgaris]	-	A0A0K9RTZ9|A0A0K9RTZ9_SPIOL:Uncharacterized protein;OS=Spinacia oleracea;|GO:0016021,|GO:0005524,GO:0004674,
DN110971_c0_g1_i1	UP	-	-	-	-	-	-
DN118350_c4_g1_i1	UP	[GO:0016868:intramolecular transferase activity, phosphotransferases][GO:0005975:carbohydrate metabolic process]	-	-	XP_021733113.1:uncharacterized protein LOC110699924 [Chenopodium quinoa]	-	A0A0D2NPE6|A0A0D2NPE6_GOSRA: Uncharacterized protein;OS=Gossypium raimondii;|GO:0016868,|GO:0005975,

geneid	trend	GO	Pathway	TF	NR	NT	Uniprot
DN96880_c0_g3_i1	UP	[GO:0046872:metal ion binding][GO:0003676:nucleic acid binding]	-	SapurV1A.1699s0060.1.p:Salix purpurea|C2H2|C2H2 family protein	XP_002283623.2:PREDICTED: zinc finger protein 6 [Vitis vinifera] >CBI26997.3 unnamed protein product, partial [Vitis vinifera]	gi|332189094|gb|CP002684.1|:Arabidopsis thaliana chromosome 1, complete sequence	A5BMV3|A5BMV3_VITVI:Putative uncharacterized protein;OS=Vitis vinifera;GN=VIT_01s0011g03780 {ECO:0000313|EMBL:CCB50897.1};|GO:0046872,GO:0003676,
DN109360_c0_g1_i1	UP	[GO:0008270:zinc ion binding]	-	-	XP_021853438.1:uncharacterized protein LOC110792926 [Spinacia oleracea]	-	A0A151RJK5|A0A151RJK5_CAJCA:Sporozoite surface protein 2;OS=Cajanus cajan;|GO:0008270,
DN116634_c1_g1_i2	UP	[GO:0016021:integral component of membrane][GO:0016811:hydrolase activity, acting on carbon-nitrogen (but not peptide) bonds, in linear amides][GO:0006672:ceramide metabolic process]	-	-	XP_007042235.1:PREDICTED: uncharacterized protein LOC18607807 [Theobroma cacao] >EOX98066.1 Senescence associated gene 18	gi|255576757|ref|XM_002529220.1|:Ricinus communis conserved hypothetical protein, mRNA	A0A061E151|A0A061E151_THECC:Senescence associated gene 18 isoform 1;OS=Theobroma cacao;|GO:0016021,|GO:0016811,|GO:0006672,

geneid	trend	GO	Pathway	TF	NR	NT	Uniprot
					isoform 1 [Theobroma cacao]		
DN128828_c1_g1_i7	UP	[GO:0030131:clathrin adaptor complex][GO:0008565:obsolete protein transporter activity][GO:0006886:intracellular protein transport][GO:0016192:vesicle-mediated transport]	[ko04144:Endocytosis][ko04721:Synaptic vesicle cycle][ko04961:Endocrine and other factor-regulated calcium reabsorption][ko05016:Huntington's disease]	FANhyb_rscf00000217.1.g00006.1:Fragaria x ananassa|WRKY|WRKY family protein	XP_010689981.1:PREDICTED: AP2 complex subunit alpha1like [Beta vulgaris subsp. vulgaris]	gi|255573013|ref|XM_002527391.1|:Ricinus communis AP-2 complex subunit alpha, putative, mRNA	A0A0J8BPD0|A0A0J8BPD0_BETVU:Uncharacterized protein;OS=Beta vulgaris subsp. vulgaris.;|GO:0030131,|GO:0008565,|GO:0006886,GO:0016192,
DN126173_c0_g1_i6	UP	[GO:0005524:ATP binding][GO:0004672:protein kinase activity]	-	ONIVA10G07200.3:Oryza nivara|CAMTA|CAMTA family protein	XP_021743743.1:serine/threonineprotein kinase CTR1like [Chenopodium quinoa]	gi|224063386|ref|XM_002301087.1|:Populus trichocarpa predicted protein, mRNA	A0A0J8D8M4|A0A0J8D8M4_BETVU:Uncharacterized protein;OS=Beta vulgaris subsp. vulgaris.;|GO:0005524,GO:0004672,

geneid	trend	GO	Pathway	TF	NR	NT	Uniprot
DN119057_c0_g6_i15	UP	[GO:0016491:oxidoreductase activity][GO:0008270:zinc ion binding]	[ko00010:Glycolysis / Gluconeogenesis][ko00071:Fatty acid degradation][ko00350:Tyrosine metabolism][ko00625:Chloroalkane and chloroalkene degradation][ko00626:Naphthalene degradation][ko00680:Methane metabolism][ko00830:Retinol metabolism][ko00980:Metabolism of xenobiotics by cytochrome P450][ko00982:Drug metabolism - cytochrome P450][ko05204:Chemical carcinogenesis]	-	OAY23266.1:hypothetical protein MANES_18G064700 [Manihot esculenta]	-	A0A151RDF6|A0A151RDF6_CAJCA: Alcohol dehydrogenase-like 2;OS=Cajanus cajan;|GO:0016491, GO:0008270,

geneid	trend	GO	Pathway	TF	NR	NT	Uniprot
DN123911_c0_g2_i4	UP	[GO:0016021:integral component of membrane]	[ko00514:Other types of O-glycan biosynthesis]	-	XP_021774450.1:hydroxyproline Oarabinosyltransferase 3like isoform X2 [Chenopodium quinoa]	gi|521504412|gb|GAHZ01026508.1|:TSA: Salicornia europaea Unigene29164_SeCKS transcribed RNA sequence	A0A0J8CTP8|A0A0J8CTP8_BETVU:Uncharacterized protein;OS=Beta vulgaris subsp. vulgaris.;|GO:0016021,
DN119553_c0_g2_i3	UP	[GO:0005759:mitochondrial matrix][GO:0000774:adenyl-nucleotide exchange factor activity][GO:0006457:protein folding]	-	-	KNA10526.1:hypothetical protein SOVF_143620 [Spinacia oleracea]	gi|521689119|gb|GAIA01023207.1|:TSA: Salicornia europaea Unigene24810_Se200S transcribed RNA sequence	A0A0K9QVI0|A0A0K9QVI0_SPIOL:GrpE protein homolog;OS=Spinacia oleracea;|GO:0005759,|GO:0000774,|GO:0006457,
DN124159_c1_g1_i2	UP	-	-	-	KYP59155.1:hypothetical protein KK1_014586 [Cajanus cajan]	-	A0A151UHV7|A0A151UHV7_CAJCA:Uncharacterized protein;OS=Cajanus cajan;
DN118358_c5_g1_i1	UP	-	-	-	-	-	-
DN128089_c4_g1_i1	UP	-	-	-	-	-	-

geneid	trend	GO	Pathway	TF	NR	NT	Uniprot
DN122589_c1_g2_i12	UP	[GO:0050660:flavin adenine dinucleotide binding][GO:0017150:tRNA dihydrouridine synthase activity]	-	Dca41227.1:Dianthus caryophyllus|C3H|C3H family protein	XP_021747545.1:tRNAdihydrouridine(20) synthase [NAD(P)+]like [Chenopodium quinoa] >XP_021747550.1 tRNAdihydrouridine (20) synthase [NAD(P)+]like [Chenopodium quinoa]	gi|521737218|gb|GAIA01008217.1|:TSA: Salicornia europaea Unigene8547_Se200S transcribed RNA sequence	V4SKP9|V4SKP9_9ROSI:tRNA-dihydrouridine synthase;OS=Citrus clementina.;|GO:0050660,GO:0017150,
DN118660_c0_g1_i7	UP	[GO:0016021:integral component of membrane]	-	MDP0000270267:Malus domestica|MYB|MYB family protein	XP_021837557.1:transmembrane protein 205 [Spinacia oleracea] >KNA26048.1 hypothetical protein SOVF_001140 [Spinacia oleracea]	gi|470129096|ref|XM_004300416.1|:PREDICTED: Fragaria vesca subsp. vesca transmembrane protein 205-like (LOC101303039), mRNA	A0A0K9S2U8|A0A0K9S2U8_SPIOL:Uncharacterized protein;OS=Spinacia oleracea;|GO:0016021,

geneid	trend	GO	Pathway	TF	NR	NT	Uniprot
DN128603_c0_g1_i14	UP	[GO:0016021:integral component of membrane]	-	Oropetium_20150105_17443A:Oropetium thomaeum|bZIP|bZIP family protein	XP_021727048.1:FT interacting protein 1like [Chenopodium quinoa]	gi|359481881|ref|XM_002277934.2|:PREDICTED: Vitis vinifera multiple C2 and transmembrane domain-containing protein 2-like (LOC100262254), mRNA	A0A0K9QH78|A0A0K9QH78_SPIOL:Uncharacterized protein;OS=Spinacia oleracea;|GO:0016021,
DN120018_c2_g7_i7	UP	[GO:0008081:phosphoric diester hydrolase activity][GO:0006629:lipid metabolic process]	-	Kaladp0020s0147.1.p:Kalanchoe laxiflora|bZIP|bZIP family protein	XP_010690604.1:PREDICTED: PIPLC X domaincontaining protein At5g67130 isoform X1 [Beta vulgaris subsp. vulgaris] >KMT01614.1 hypothetical protein BVRB_9g216080 [Beta vulgaris subsp. vulgaris]	gi|470119924|ref|XM_004296012.1|:PREDICTED: Fragaria vesca subsp. vesca PI-PLC X domain-containing protein At5g67130-like (LOC101293574), mRNA	A0A0J8BNU4|A0A0J8BNU4_BETVU: Uncharacterized protein;OS=Beta vulgaris subsp. vulgaris.;|GO:0008081,|GO:0006629,

geneid	trend	GO	Pathway	TF	NR	NT	Uniprot
DN110641_c0_g2_i3	UP	[GO:0046872:metal ion binding]	-	evm.model.supercontig_1307.1:Carica papaya|C2H2|C2H2 family protein	KMS98817.1:hypothetical protein BVRB_3g068910 [Beta vulgaris subsp. vulgaris]	-	A0A0J8BFM8|A0A0J8BFM8_BETVU: Uncharacterized protein;OS=Beta vulgaris subsp. vulgaris.;|GO:0046872,
DN128394_c4_g4_i14	UP	-	-	Rsa1.0_04342.1_g00002.1:Raphanus sativus|LBD|LBD family protein	XP_011013113.1:PREDICTED: LOW QUALITY PROTEIN: uncharacterized protein LOC105117228 [Populus euphratica]	-	F4NCG3|F4NCG3_BETVU:Uncharacterized protein;OS=Beta vulgaris subsp. vulgaris.;
DN122744_c1_g1_i1	UP	-	-	-	XP_021743698.1:uncharacterized protein LOC110709766 [Chenopodium quinoa]	gi|521483751|gb|GAHZ01047135.1|:TSA: Salicornia europaea Unigene55534_SeCKS transcribed RNA sequence	A0A0J8D663|A0A0J8D663_BETVU:Uncharacterized protein;OS=Beta vulgaris subsp. vulgaris.;

geneid	trend	GO	Pathway	TF	NR	NT	Uniprot
DN123747_c0_g1_i1	UP	-	-	-	XP_021750846.1:uncharacterized protein LOC110716518 [Chenopodium quinoa]	gi|521741977|gb|GAIA01005270.1|:TSA: Salicornia europaea Unigene5559_Se200S transcribed RNA sequence	A0A0J8BM31|A0A0J8BM31_BETVU: Uncharacterized protein;OS=Beta vulgaris subsp. vulgaris.;
DN120848_c0_g1_i10	UP	[GO:0016021:integral component of membrane][GO:0015031:protein transport]	-	-	XP_021721565.1:preprotein translocase subunit SCY2, chloroplasticlike [Chenopodium quinoa]	gi|521542055|gb|GAHZ01002247.1|:TSA: Salicornia europaea Unigene2305_SeCKS transcribed RNA sequence	A0A0J8CMM8|A0A0J8CMM8_BETVU: Uncharacterized protein;OS=Beta vulgaris subsp. vulgaris.;|GO:0016021,|GO:0015031,
DN124671_c3_g1_i5	UP	[GO:0016021:integral component of membrane]	-	-	XP_010680367.1:PREDICTED: uncharacterized protein LOC104895527 [Beta vulgaris subsp. vulgaris] >KMT09292.1 hypothetical protein BVRB_6g133910 [Beta vulgaris subsp. vulgaris]	-	A0A0J8C6V9|A0A0J8C6V9_BETVU:Uncharacterized protein;OS=Beta vulgaris subsp. vulgaris.;|GO:0016021,

geneid	trend	GO	Pathway	TF	NR	NT	Uniprot
DN125822_c 1_g1_i7	UP	[GO:0061630:ubiquitin protein ligase activity][GO:0008270:zinc ion binding]	[ko04115:p53 signaling pathway][ko04120: Ubiquitin mediated proteolysis][ko04712:Circadian rhythm - plant]	OMERI02G32430.2: Oryza meridionalis|GeBP|GeBP family protein	XP_021837564.1:E3 ubiquitinprotein ligase COP1like [Spinacia oleracea] >KNA04927.1 hypothetical protein SOVF_195150 [Spinacia oleracea]	-	A0A0K9QCC6|A0A0K9QCC6_SPIOL: Uncharacterized protein;OS=Spinacia oleracea;|GO:0061630,GO:0008270,
DN125836_c 1_g4_i6	UP	[GO:0046872:metal ion binding][GO:0008233:peptidase activity]	-	-	XP_021731733.1:presequence protease 1, chloroplastic/mitochondriallike [Chenopodium quinoa]	gi|449495594|ref|XM_004159841.1|:PREDICTED: Cucumis sativus presequence protease 1, chloroplastic/mitochondrial-like (LOC101228559), mRNA	A0A023EB19|A0A023EB19_SILLA:Presequence protease 1;OS=Silene latifolia;|GO:0046872,GO:0008233,
DN126093_c 1_g4_i13	UP	-	-	-	XP_021858301.1:mediator of RNA polymerase II transcription subunit 16 isoform X1 [Spinacia oleracea] >KNA05569.1 hypothetical protein	gi|478155242|gb|GAHK01021361.1|:TSA: Jatropha curcas Unigene8819_JC-CK_1A transcribed RNA sequence	A0A0K9QE69|A0A0K9QE69_SPIOL:Uncharacterized protein;OS=Spinacia oleracea;

geneid	trend	GO	Pathway	TF	NR	NT	Uniprot
					SOVF_189130 [Spinacia oleracea]		
DN125224_c1_g5_i1	UP	-	-	Rsa1.0_04342.1_g00002.1:Raphanus sativus|LBD|LBD family protein	XP_021836103.1:uncharacterized protein LOC110775811 [Spinacia oleracea]	-	F4NCG3|F4NCG3_BETVU:Uncharacterized protein;OS=Beta vulgaris subsp. vulgaris.;
DN128207_c2_g2_i2	UP	[GO:0005829:cytosol][GO:0030904:retromer complex][GO:0008565:obsolete protein transporter activity][GO:0042147:retrograde transport, endosome to Golgi]	[ko04144:Endocytosis]	EMT28005:Aegilops tauschii|MYB_related|MYB_related family protein	XP_010679886.1:PREDICTED: vacuolar protein sortingassociated protein 35A [Beta vulgaris subsp. vulgaris] >KMT09562.1 hypothetical protein BVRB_6g130220 [Beta vulgaris subsp.	gi|470102701|ref|XM_004287742.1|:PREDICTED: Fragaria vesca subsp. vesca vacuolar protein sorting-associated protein 35A-like (LOC101293426), mRNA	A0A0J8C7M2|A0A0J8C7M2_BETVU: Vacuolar protein sorting-associated protein 35;OS=Beta vulgaris subsp. vulgaris.;|GO:0005829,GO:0030904,|GO:0008565,|GO:0042147,

geneid	trend	GO	Pathway	TF	NR	NT	Uniprot
					vulgaris]		
DN121522_c1_g1_i1	UP	[GO:0016021:integral component of membrane][GO:0005524:ATP binding][GO:0004222:metalloendopeptidase activity][GO:0008270:zinc ion binding][GO:0051301:cell division]	-	Achn040601:Actinidia chinensis|WRKY|WRKY family protein	XP_009598017.1:PREDICTED: ATPdependent zinc metalloprotease FTSH 10, mitochondriallike [Nicotiana tomentosiformis] >XP_016470439.1 PREDICTED: ATPdependent zinc metalloprotease FTSH 10, mitochondriallike [Nicotiana tabacum]	gi|224104402|ref|XM_002313390.1|:Populus trichocarpa predicted protein, mRNA	A0A061ERL6|A0A061ERL6_THECC:Cell division protease ftsH isoform 1;OS=Theobroma cacao;|GO:0016021,|GO:0005524,GO:0004222,GO:0008270,|GO:0051301,

geneid	trend	GO	Pathway	TF	NR	NT	Uniprot
DN112718_c0_g1_i1	UP	-	[ko04141:Protein processing in endoplasmic reticulum]	WALNUT_00030998-RA:Juglans regia|NF-YB|NF-YB family protein	XP_021732378.1:18.3 kDa class I heat shock protein [Chenopodium quinoa]	gi|509069|emb|X64561.1|:H.vulgare hsp18 mRNA for 18 kd heat shock protein	A0A0J8CXG7|A0A0J8CXG7_BETVU: Uncharacterized protein;OS=Beta vulgaris subsp. vulgaris.;
DN119092_c3_g2_i5	UP	[GO:0004491:methylmalonate-semialdehyde dehydrogenase (acylating) activity]	-	-	XP_015080794.1:PREDICTED: uncharacterized protein LOC107024343 [Solanum pennellii]	-	A0A151RHK1|A0A151RHK1_CAJCA: Retrovirus-related Pol polyprotein from transposon TNT 1-94;OS=Cajanus cajan;|GO:0004491,
DN107177_c0_g1_i2	UP	[GO:0005524:ATP binding]	[ko03440:Homologous recombination]	ORUFI07G12350.1: Oryza rufipogon|MYB_related|MYB_related family protein	XP_021753144.1:protein CHROMATIN REMODELING 35like [Chenopodium quinoa]	-	A0A0K9QB09|A0A0K9QB09_SPIOL:Uncharacterized protein;OS=Spinacia oleracea;|GO:0005524,

geneid	trend	GO	Pathway	TF	NR	NT	Uniprot
DN122406_c0_g1_i2	UP	[GO:0005829:cytosol][GO:0005634:nucleus]	-	-	XP_010689282.1:PREDICTED: nitrilespecifier protein 5 [Beta vulgaris subsp. vulgaris] >KMT02267.1 hypothetical protein BVRB_9g206540 [Beta vulgaris subsp. vulgaris]	-	A0A0J8BM28|A0A0J8BM28_BETVU: Uncharacterized protein;OS=Beta vulgaris subsp. vulgaris.;|GO:0005829,GO:0005634,
DN117977_c3_g7_i5	UP	[GO:0003676:nucleic acid binding]	-	Rsa1.0_00145.1_g00025.1:Raphanus sativus|MYB|MYB family protein	XP_010690177.1:PREDICTED: uncharacterized protein LOC104903764 [Beta vulgaris subsp. vulgaris]	-	F4NCI7|F4NCI7_BETVU:Uncharacterized protein;OS=Beta vulgaris subsp. vulgaris.;|GO:0003676,
DN123795_c0_g1_i4	UP	[GO:0016021:integral component of membrane]	-	Seita.5G004700.1.p:Setaria italica|bHLH|bHLH family protein	XP_021856469.1:WPP domaininteracting tailanchored protein 2like isoform X2 [Spinacia oleracea] >KNA15985.1	-	A0A0K9R917|A0A0K9R917_SPIOL:Uncharacterized protein;OS=Spinacia oleracea;|GO:0016021,

geneid	trend	GO	Pathway	TF	NR	NT	Uniprot
					hypothetical protein SOVF_093410 isoform A [Spinacia oleracea]		
DN119336_c3_g3_i3	UP	-	-	-	-	-	-

geneid	trend	GO	Pathway	TF	NR	NT	Uniprot
DN121920_c1_g1_i5	UP	[GO:0005524:ATP binding][GO:0006457:protein folding][GO:0006950:response to stress]	[ko04141:Protein processing in endoplasmic reticulum][ko04151:PI3K-Akt signaling pathway][ko04217:Necroptosis][ko04612:Antigen processing and presentation][ko04621:NOD-like receptor signaling pathway][ko04626:Plant-pathogen interaction][ko04657:IL-17 signaling pathway][ko04659:Th17 cell differentiation][ko04914:Progesterone-mediated oocyte maturation][ko04915:Estrogen signaling pathway][ko05200:Pathways in	Do001279.1:Dichanthelium oligosanthes|bZIP|bZIP family protein	XP_019168655.1:PREDICTED: heat shock protein 83like [Ipomoea nil]	gi|359474126|ref|XM_003631357.1|:PREDICTED: Vitis vinifera heat shock protein 83-like, transcript variant 2 (LOC100265661), mRNA	A0A067G9H4|A0A067G9H4_CITSI:Uncharacterized protein;OS=Citrus sinensis;|GO:0005524,|GO:0006457,GO:0006950,

geneid	trend	GO	Pathway	TF	NR	NT	Uniprot
			cancer][ko05215:Prostate cancer][ko05418:Fluid shear stress and atherosclerosis]				

geneid	trend	GO	Pathway	TF	NR	NT	Uniprot
DN125648_c0_g2_i8	UP	[GO:0005743:mitochondrial inner membrane][GO:0005759:mitochondrial matrix][GO:0005525:GTP binding][GO:0003924:GTPase activity][GO:0043022:ribosome binding][GO:0045727:positive regulation of translation][GO:0006412:translation]	-	KZV46390.1:Dorcoceras hygrometricum|LBD|LBD family protein	XP_021742076.1:translation factor GUF1 homolog, chloroplastic isoform X1 [Chenopodium quinoa]	gi|460381798|ref|XM_004236581.1|:PREDICTED: Solanum lycopersicum translation factor GUF1 homolog, chloroplastic-like (LOC101249451), mRNA	A0A0D2RXT4|A0A0D2RXT4_GOSRA: Translation factor GUF1 homolog, mitochondrial;OS=Gossypium raimondii;|GO:0005743,GO:0005759,|GO:0005525,GO:0003924,GO:0043022,|GO:0045727,GO:0006412,
DN125018_c1_g3_i3	UP	[GO:0008233:peptidase activity]	[ko04142:Lysosome][ko04612:Antigen processing and presentation]	-	XP_021739062.1:vacuolarprocessing enzyme alphaisozymelike [Chenopodium quinoa]	gi|356506931|ref|XM_003522179.1|:PREDICTED: Glycine max vacuolar-processing enzyme-like (LOC100807010), mRNA	A0A0K9QZ96|A0A0K9QZ96_SPIOL:Uncharacterized protein;OS=Spinacia oleracea;|GO:0008233,
DN125297_c10_g2_i2	UP	-	-	-	-	-	-

geneid	trend	GO	Pathway	TF	NR	NT	Uniprot
DN128276_c1_g2_i6	UP	-	-	Dca61174.1:Dianthus caryophyllus|bHLH|bHLH family protein	XP_010694661.1:PREDICTED: protein SIEVE ELEMENT OCCLUSION Blike [Beta vulgaris subsp. vulgaris]	gi|225452267|ref|XM_002271914.1|:PREDICTED: Vitis vinifera uncharacterized LOC100261056 (LOC100261056), mRNA	A0A0J8BDR1|A0A0J8BDR1_BETVU: Uncharacterized protein;OS=Beta vulgaris subsp. vulgaris.;
DN121111_c0_g1_i5	UP	-	-	Dca61174.1:Dianthus caryophyllus|bHLH|bHLH family protein	XP_021746786.1:protein SIEVE ELEMENT OCCLUSION Clike [Chenopodium quinoa]	-	A0A0K9RPQ8|A0A0K9RPQ8_SPIOL:Uncharacterized protein;OS=Spinacia oleracea;
DN127086_c2_g2_i4	UP	-	-	-	XP_021855412.1:uncharacterized protein LOC110794765 isoform X2 [Spinacia oleracea] >KNA13119.1 hypothetical protein SOVF_119600 isoform A [Spinacia oleracea]	-	A0A0K9R2H1|A0A0K9R2H1_SPIOL:Uncharacterized protein;OS=Spinacia oleracea;

geneid	trend	GO	Pathway	TF	NR	NT	Uniprot
DN124825_c3_g2_i4	UP	[GO:0005615:extracellular space][GO:0005794:Golgi apparatus][GO:0005773:vacuole][GO:0030598:rRNA N-glycosylase activity][GO:0006952:defense response][GO:0017148:negative regulation of translation]	-	-	P85208.1:RecName: Full=Dioicin2; AltName: Full=Ribosomeinactivating protein; AltName: Full=rRNA Nglycosidase	-	P85208|RIPD2_PHYDI:Dioicin-2;OS=Phytolacca dioica;|GO:0005615,GO:0005794,GO:0005773,|GO:0030598,|GO:0006952,GO:0017148,
DN118479_c0_g1_i13	UP	[GO:0016021:integral component of membrane][GO:0015297:antiporter activity]	-	TRIUR3_11943-P1: Triticum urartu|ERF|ERF family protein	XP_021722645.1:protein DETOXIFICATION 33like [Chenopodium quinoa]	gi|512397657|gb|GAJZ01003513.1|:TSA: Prunus avium Pa_07596 transcribed RNA sequence	A0A0K9R9I6|A0A0K9R9I6_SPIOL:Protein DETOXIFICATION;OS=Spinacia oleracea;|GO:0016021,|GO:0015297,GO:0015238,
DN118358_c5_g1_i4	UP	-	-	-	-	-	-

geneid	trend	GO	Pathway	TF	NR	NT	Uniprot
DN126558_c0_g1_i28	UP	[GO:0003677:DNA binding][GO:0003721:telomerase RNA reverse transcriptase activity][GO:0050000:chromosome localization][GO:0007000:nucleolus organization][GO:0007004:telomere maintenance via telomerase]	[ko05166:HTLV-I infection]	-	XP_021856298.1:telomerase reverse transcriptase isoform X1 [Spinacia oleracea]	-	A0A0J8BE57|A0A0J8BE57_BETVU:Uncharacterized protein;OS=Beta vulgaris subsp. vulgaris.;|GO:0003677,GO:0003721,|GO:0050000,GO:0007000,GO:0007004,
DN121210_c0_g1_i7	UP	[GO:0016021:integral component of membrane][GO:0005524:ATP binding][GO:0004674:protein serine/threonine kinase activity]	-	-	XP_021752725.1:probable LRR receptorlike serine/threonineprotein kinase At5g10290 isoform X1 [Chenopodium quinoa] >XP_021752726.1 probable LRR receptorlike serine/threonineprotein kinase At5g10290 isoform X1 [Chenopodium quinoa]	-	A0A0J8C4P4|A0A0J8C4P4_BETVU:Uncharacterized protein;OS=Beta vulgaris subsp. vulgaris.;|GO:0016021,|GO:0005524,GO:0004674,

geneid	trend	GO	Pathway	TF	NR	NT	Uniprot
DN121802_c0_g3_i3	UP	[GO:0005524:ATP binding][GO:0004674:protein serine/threonine kinase activity]	[ko04068:FoxO signaling pathway][ko04310: Wnt signaling pathway][ko04340: Hedgehog signaling pathway][ko04341: Hedgehog signaling pathway - fly][ko04390:Hippo signaling pathway][ko04391: Hippo signaling pathway - fly][ko04392:Hippo signaling pathway -multiple species][ko04710:Circadian rhythm][ko04711:Circadian rhythm - fly]	-	XP_010688272.1:PREDICTED: casein kinase 1like protein 4 [Beta vulgaris subsp. vulgaris] >XP_010688273.1 PREDICTED: casein kinase 1like protein 4 [Beta vulgaris subsp. vulgaris] >KMT03143.1 hypothetical protein BVRB_8g196990 [Beta vulgaris subsp. vulgaris]	-	A0A0J8BTB6|A0A0J8BTB6_BETVU:Uncharacterized protein;OS=Beta vulgaris subsp. vulgaris.;|GO:0005524,GO:0004674,

geneid	trend	GO	Pathway	TF	NR	NT	Uniprot
DN120838_c0_g1_i9	UP	[GO:0016021:integral component of membrane][GO:0046872:metal ion binding]	-	AHYPO_001353-RA:Amaranthus hypochondriacus|C2H2|C2H2 family protein	XP_021771000.1:uncharacterized protein LOC110735150 isoform X1 [Chenopodium quinoa]	gi|512399619|gb|GAJZ01001551.1|:TSA: Prunus avium Pa_02994 transcribed RNA sequence	A0A0K9QQL8|A0A0K9QQL8_SPIOL: Uncharacterized protein;OS=Spinacia oleracea;|GO:0016021,|GO:0046872,
DN122908_c1_g1_i2	UP	[GO:0004089:carbonate dehydratase activity][GO:0008270:zinc ion binding][GO:0015976:carbon utilization]	[ko00910:Nitrogen metabolism]	-	AEV42276.1:chloroplast beta carbonic anhydrase 1 [Mesembryanthemum nodiflorum]	-	G9FZ35|G9FZ35_MESNO:Carbonic anhydrase;OS=Mesembryanthemum nodiflorum;|GO:0004089,GO:0008270,|GO:0015976,
DN120690_c4_g2_i4	UP	-	-	-	-	-	-
DN118954_c0_g4_i5	UP	[GO:0009507:chloroplast][GO:0004851:uroporphyrin-III C-methyltransferase activity][GO:0055114:oxidation-reduction process][GO:0019354:siroheme biosynthetic process]	-	-	XP_010667452.1:PREDICTED: uncharacterized protein LOC104884494 [Beta vulgaris subsp. vulgaris] >KMS95363.1 hypothetical protein	gi|521742619|gb|GAIA01004858.1|:TSA: Salicornia europaea Unigene5144_Se200S transcribed RNA sequence	A0A0J8B653|A0A0J8B653_BETVU:Uncharacterized protein;OS=Beta vulgaris subsp. vulgaris.;|GO:0009507,|GO:0004851,|GO:0055114,GO:0019354,

geneid	trend	GO	Pathway	TF	NR	NT	Uniprot
					BVRB_008870 [Beta vulgaris subsp. vulgaris]		
DN123937_c0_g3_i3	UP	[GO:0005737:cytoplasm][GO:0033925:mannosyl-glycoprotein endo-beta-N-acetylglucosaminidase activity]	[ko00511:Other glycan degradation]	KN539694.1_FGP007:Oryza longistaminata|LBD|LBD family protein	XP_021855810.1:cytosolic endobetaNacetylglucosaminidase 1like isoform X1 [Spinacia oleracea]	gi|521660662|gb|GAIA01036916.1|:TSA: Salicornia europaea Unigene40983_Se200S transcribed RNA sequence	A0A0J8BEV7|A0A0J8BEV7_BETVU:Uncharacterized protein;OS=Beta vulgaris subsp. vulgaris.;|GO:0005737,|GO:0033925,
DN127229_c1_g2_i5	UP	-	-	-	-	-	-
DN110296_c1_g1_i1	UP	[GO:0046872:metal ion binding]	-	AHYPO_015489-RA:Amaranthus hypochondriacus|C2H2|C2H2 family protein	XP_021726017.1:zinc finger protein 7like [Chenopodium quinoa]	gi|521704416|gb|GAIA01015716.1|:TSA: Salicornia europaea Unigene16403_Se200S transcribed RNA sequence	A0A0K9R7R1|A0A0K9R7R1_SPIOL:Uncharacterized protein;OS=Spinacia oleracea;|GO:0046872,

geneid	trend	GO	Pathway	TF	NR	NT	Uniprot
DN124309_c0_g1_i2	UP	-	-	-	KNA03916.1:hypothetical protein SOVF_204650 [Spinacia oleracea]	-	A0A0K9QBJ0|A0A0K9QBJ0_SPIOL:Uncharacterized protein;OS=Spinacia oleracea;
DN120528_c1_g2_i8	UP	-	[ko04141:Protein processing in endoplasmic reticulum]	Ote100209250011:Ocimum tenuiflorum|MYB_related|MYB_related family protein	XP_010666147.1:PREDICTED: small heat shock protein, chloroplastic [Beta vulgaris subsp. vulgaris] >KMS96310.1 hypothetical protein BVRB_000230 [Beta vulgaris subsp. vulgaris]	gi|224120951|ref|XM_002318424.1|:Populus trichocarpa predicted protein, mRNA	A0A0J8DZL2|A0A0J8DZL2_BETVU:Uncharacterized protein;OS=Beta vulgaris subsp. vulgaris.;

geneid	trend	GO	Pathway	TF	NR	NT	Uniprot
DN123216_c2_g7_i9	UP	[GO:0005737:cytoplasm][GO:0005634:nucleus][GO:0000977:RNA polymerase II regulatory region sequence-specific DNA binding][GO:0003700:DNA-binding transcription factor activity][GO:0048438:floral whorl development][GO:0010077:maintenance of inflorescence meristem identity][GO:0000165:MAPK cascade][GO:0045944:positive regulation of transcription by RNA polymerase II][GO:0000060:obsolete protein import into nucleus, translocation][GO:0048510:regulation of timing of transition from vegetative to reproductive phase][GO:0006351:transcription, DNA-templated]	-	Bv1_007680_jyqa.t1:Beta vulgaris|MIKC_MADS|MIKC_MADS family protein	XP_021740351.1:MADSbox protein AGL24like isoform X1 [Chenopodium quinoa]	gi|55792843|gb|AY643733.1|:Solanum tuberosum MADS16 (MADS16) mRNA, complete cds	A0A0J8D685|A0A0J8D685_BETVU:Uncharacterized protein;OS=Beta vulgaris subsp. vulgaris.;|GO:0005737,GO:0005634,|GO:0000977,GO:0003700,|GO:0048438,GO:0010077,GO:0000165,GO:0045944,GO:0000060,GO:0048510,GO:0006351,

geneid	trend	GO	Pathway	TF	NR	NT	Uniprot
DN124189_c0_g3_i6	UP	[GO:0005509:calcium ion binding][GO:0005544:calcium-dependent phospholipid binding]	-	-	XP_010680554.1:PREDICTED: annexin D2 [Beta vulgaris subsp. vulgaris] >KMT08792.1 hypothetical protein BVRB_6g135160 [Beta vulgaris subsp. vulgaris]	gi|521512199|gb|GAHZ01018721.1|:TSA: Salicornia europaea Unigene19664_SeCKS transcribed RNA sequence	A0A0J8C5G7|A0A0J8C5G7_BETVU:Annexin;OS=Beta vulgaris subsp. vulgaris.;|GO:0005509,GO:0005544,
DN126628_c0_g3_i20	UP	-	-	-	-	-	-
DN128467_c2_g5_i1	UP	-	-	-	-	-	-

geneid	trend	GO	Pathway	TF	NR	NT	Uniprot
DN90335_c0_g1_i3	UP	[GO:0016021:integral component of membrane][GO:0020037:heme binding][GO:0005506:iron ion binding][GO:0004497:monooxygenase activity][GO:0016705:oxidoreductase activity, acting on paired donors, with incorporation or reduction of molecular oxygen]	-	Zmw_sc00276.1.g00230.1:Zoysia matrella|NAC|NAC family protein	XP_021845103.1:betaamyrin 28oxidaselike [Spinacia oleracea]	-	A0A068VC91|A0A068VC91_COFCA:Uncharacterized protein;OS=Coffea canephora;|GO:0016021,|GO:0020037,GO:0005506,GO:0004497,GO:0016705,
DN124497_c3_g5_i14	UP	[GO:0016773:phosphotransferase activity, alcohol group as acceptor][GO:0005975:carbohydrate metabolic process]	-	Spipo16G0011000:Spirodela polyrhiza|SRS|SRS family protein	XP_021738697.1:uncharacterized protein LOC110705155 isoform X3 [Chenopodium quinoa]	-	A0A0K9S018|A0A0K9S018_SPIOL:Uncharacterized protein;OS=Spinacia oleracea;|GO:0016773,|GO:0005975,
DN125648_c0_g2_i15	UP	[GO:0009507:chloroplast][GO:0005525:GTP binding][GO:0003924:GTPase activity][GO:0045727:positive regulation of translation][GO:0006412:translation]	-	KZV46390.1:Dorcoceras hygrometricum|LBD|LBD family protein	XP_021718707.1:translation factor GUF1 homolog, chloroplasticlike [Chenopodium quinoa]	gi|460381798|ref|XM_004236581.1|:PREDICTED: Solanum lycopersicum translation factor GUF1 homolog, chloroplastic-like	A0A0J8DX36|A0A0J8DX36_BETVU:Translation factor GUF1 homolog, chloroplastic;OS=Beta vulgaris subsp. vulgaris.;|GO:00095

geneid	trend	GO	Pathway	TF	NR	NT	Uniprot
						(LOC101249451), mRNA	07,|GO:0005525,GO:0003924,|GO:0045727,GO:0006412,
DN128467_c2_g5_i3	UP	-	-	-	-	-	-
DN123363_c0_g3_i4	UP	-	-	-	XP_021771347.1:glutaminylpeptide cyclotransferaselike isoform X1 [Chenopodium quinoa]	-	A0A0K9RG25|A0A0K9RG25_SPIOL:Uncharacterized protein;OS=Spinacia oleracea;

geneid	trend	GO	Pathway	TF	NR	NT	Uniprot
DN117439_c2_g1_i1	UP	[GO:0009570:chloroplast stroma][GO:0009534:chloroplast thylakoid][GO:0019898:extrinsic component of membrane][GO:0009654:photosystem II oxygen evolving complex][GO:0031977:thylakoid lumen][GO:0005509:calcium ion binding][GO:0015979:photosynthesis]	-	-	XP_021745859.1:psbP domaincontaining protein 6, chloroplasticlike [Chenopodium quinoa]	gi|356571887|ref|XM_003554055.1|:PREDICTED: Glycine max psbP domain-containing protein 6, chloroplastic-like (LOC100795124), mRNA	A0A0J8CNY4|A0A0J8CNY4_BETVU: Uncharacterized protein;OS=Beta vulgaris subsp. vulgaris.;|GO:0009570,GO:0009534,GO:0019898,GO:0009654,GO:0031977,|GO:0005509,|GO:0015979,
DN125439_c0_g1_i3	UP	[GO:0071949:FAD binding][GO:0016491:oxidoreductase activity]	-	-	KNA16154.1:hypothetical protein SOVF_091900 [Spinacia oleracea]	gi|225425868|ref|XM_002266226.1|:PREDICTED: Vitis vinifera 2,4-dichlorophenol 6-monooxygenase-like (LOC100251202), mRNA	A0A0K9RAX3|A0A0K9RAX3_SPIOL: Uncharacterized protein;OS=Spinacia oleracea;|GO:0071949,GO:0016491,
DN117997_c2_g1_i10	UP	-	[ko04075:Plant hormone signal transduction]	-	XP_021716556.1:protein TIFY 10Alike [Chenopodium quinoa]	-	A0A0K9REE4|A0A0K9REE4_SPIOL:Uncharacterized protein;OS=Spinacia

geneid	trend	GO	Pathway	TF	NR	NT	Uniprot
							oleracea;
DN122311_c0_g2_i1	UP	-	-	-	-	-	-
DN115319_c0_g1_i2	UP	[GO:0003676:nucleic acid binding][GO:0008270:zinc ion binding]	-	-	XP_019071462.1:PREDICTED: uncharacterized protein LOC101255439 [Solanum lycopersicum]	-	K4C139|K4C139_SOLLC:Uncharacterized protein;OS=Solanum lycopersicum;|GO:0003676,GO:0008270,
DN119487_c1_g2_i10	UP	[GO:0016491:oxidoreductase activity]	[ko00061:Fatty acid biosynthesis][ko00780:Biotin metabolism][ko01040:Biosynthesis of unsaturated fatty acids]	-	XP_010672066.1:PREDICTED: shortchain type dehydrogenase/reductase [Beta vulgaris subsp. vulgaris] >KMT15639.1 hypothetical protein BVRB_3g056300 [Beta vulgaris subsp. vulgaris]	-	A0A0J8CUB0|A0A0J8CUB0_BETVU: Uncharacterized protein;OS=Beta vulgaris subsp. vulgaris.;|GO:0016491,

geneid	trend	GO	Pathway	TF	NR	NT	Uniprot
DN105520_c0_g1_i1	UP	[GO:0006952:defense response][GO:0009607:response to biotic stimulus]	-	-	XP_010693433.1:PREDICTED: kirolalike [Beta vulgaris subsp. vulgaris]	-	A0A0J8D1B6|A0A0J8D1B6_BETVU:Uncharacterized protein;OS=Beta vulgaris subsp. vulgaris.;|GO:0006952,GO:0009607,
DN127865_c2_g3_i1	UP	[GO:0003676:nucleic acid binding][GO:0008270:zinc ion binding][GO:0015074:DNA integration]	-	Rsa1.0_02878.1_g00005.1:Raphanus sativus|LBD|LBD family protein	KYP44533.1:Retrovirusrelated Pol polyprotein from transposon TNT 194 [Cajanus cajan]	-	A0A151TPU3|A0A151TPU3_CAJCA:Retrovirus-related Pol polyprotein from transposon TNT 1-94;OS=Cajanus cajan;|GO:0003676,GO:0008270,|GO:0015074,
DN127344_c1_g5_i4	UP	[GO:0043248:proteasome assembly]	-	Kalax.0006s0195.1.p:Kalanchoe marnieriana|MYB_related|MYB_related family protein	XP_019104401.1:PREDICTED: proteasomeassociated protein ECM29 homolog [Beta vulgaris subsp. vulgaris] >KMT14635.1 hypothetical protein	gi|521483266|gb|GAHZ01047620.1|:TSA: Salicornia europaea Unigene56086_SeCKS transcribed RNA sequence	A0A0J8CLM0|A0A0J8CLM0_BETVU: Uncharacterized protein;OS=Beta vulgaris subsp. vulgaris.;|GO:0043248,

geneid	trend	GO	Pathway	TF	NR	NT	Uniprot
					BVRB_4g074030 [Beta vulgaris subsp. vulgaris]		
DN120634_c0_g1_i5	UP	[GO:0046872:metal ion binding][GO:0016491:oxidoreductase activity]	-	676709864:Sisymbrium irio|STAT|STAT family protein	XP_006492604.1:PREDICTED: probable 2oxoglutarate/Fe(II) dependent dioxygenase [Citrus sinensis]	-	A0A061E128|A0A061E128_THECC:2-oxoglutarate and Fe(II)-dependent oxygenase superfamily protein, putative;OS=Theobroma cacao;|GO:0046872, GO:0016491,

geneid	trend	GO	Pathway	TF	NR	NT	Uniprot
DN120033_c1_g1_i1	UP	[GO:0016021:integral component of membrane]	[ko04020:Calcium signaling pathway][ko04218:Cellular senescence][ko04621:NOD-like receptor signaling pathway]	Zmw_sc01544.1.g00110.1:Zoysia matrella|GRAS|GRAS family protein	XP_010680913.1:PREDICTED: calcium uniporter protein 2, mitochondrial isoform X2 [Beta vulgaris subsp. vulgaris] >KMT08501.1 hypothetical protein BVRB_6g137880 isoform B [Beta vulgaris subsp. vulgaris]	gi|356562328|ref|XM_003549376.1|:PREDICTED: Glycine max uncharacterized protein LOC100808882 (LOC100808882), mRNA	A0A0J8EZ60|A0A0J8EZ60_BETVU:Uncharacterized protein;OS=Beta vulgaris subsp. vulgaris.;|GO:0016021,
DN123280_c0_g1_i3	UP	[GO:0006506:GPI anchor biosynthetic process]	[ko00563:Glycosylphosphatidylinositol (GPI)-anchor biosynthesis]	-	XP_021847747.1:phosphatidylinositol Nacetylglucosaminyltransferase subunit A [Spinacia oleracea] >KNA14441.1 hypothetical protein SOVF_106800 [Spinacia oleracea]	gi|359478862|ref|XM_002279532.2|:PREDICTED: Vitis vinifera phosphatidylinositol N-acetylglucosaminyltransferase subunit A-like (LOC100256927), mRNA	A0A0K9R4L6|A0A0K9R4L6_SPIOL:Uncharacterized protein;OS=Spinacia oleracea;|GO:0006506,

geneid	trend	GO	Pathway	TF	NR	NT	Uniprot
DN122966_c4_g2_i16	UP	[GO:0003824:catalytic activity][GO:0030170:pyridoxal phosphate binding]	-	-	XP_010675591.1:PREDICTED: uncharacterized protein LOC104891581 [Beta vulgaris subsp. vulgaris] >XP_019104672.1 PREDICTED: uncharacterized protein LOC104891581 [Beta vulgaris subsp. vulgaris] >XP_019104674.1 PREDICTED: uncharacterized protein LOC104891581 [Beta vulgaris subsp. vulgaris] >XP_019104675.1 PREDICTED: uncharacterized protein LOC104891581 [Beta vulgaris subsp. vulgaris] >XP_019104676.1 PREDICTED: uncharacterized	gi|449464769|ref|XM_004150054.1|:PREDICTED: Cucumis sativus uncharacterized LOC101215148 (LOC101215148), mRNA	A0A0J8CHJ7|A0A0J8CHJ7_BETVU:Uncharacterized protein;OS=Beta vulgaris subsp. vulgaris.;|GO:0003824,GO:0030170,

geneid	trend	GO	Pathway	TF	NR	NT	Uniprot
DN114039_c0_g2_i1	UP	-	-	-	-	-	-
DN119204_c1_g5_i7	UP	[GO:0015035:protein disulfide oxidoreductase activity]	-	KZV45610.1:Dorcoceras hygrometricum|MIKC_MADS|MIKC_MADS family protein	XP_021739390.1:uncharacterized protein LOC110705771 [Chenopodium quinoa]	-	A0A0K9RP58|A0A0K9RP58_SPIOL:Uncharacterized protein;OS=Spinacia oleracea;|GO:0015035,
DN125906_c1_g2_i7	UP	-	-	-	XP_016549168.1:PREDICTED: protein ACCELERATED CELL DEATH 6like isoform X1 [Capsicum annuum]	-	-
DN125466_c6_g1_i11	UP	[GO:0016021:integral component of membrane][GO:0009536:plastid][GO:0005315:inorganic phosphate transmembrane transporter activity]	-	-	XP_021744802.1:probable anion transporter 3, chloroplastic isoform X1 [Chenopodium quinoa]	-	A0A0J8CJK8|A0A0J8CJK8_BETVU:Uncharacterized protein;OS=Beta vulgaris subsp. vulgaris.;|GO:0016021,GO:0009536,|GO:0005315,

geneid	trend	GO	Pathway	TF	NR	NT	Uniprot
DN119461_c 1_g1_i2	UP	[GO:0004553:hydrolase activity, hydrolyzing O-glycosyl compounds][GO:0005975:carbohydrate metabolic process]	[ko00460:Cyanoamino acid metabolism][ko00500:Starch and sucrose metabolism][ko00940:Phenylpropanoid biosynthesis]	676737120:Sisymbrium irio|NF-YC|NF-YC family protein	XP_021751155.1:betaglucosidase 12like [Chenopodium quinoa]	gi|359478416|ref|XM_003632070.1|:PREDICTED: Vitis vinifera beta-glucosidase 12-like (LOC100250160), mRNA	A0A0J8CL01|A0A0J8CL01_BETVU:Uncharacterized protein;OS=Beta vulgaris subsp. vulgaris.;|GO:0004553,|GO:0005975,
DN127624_c 2_g2_i3	UP	[GO:0004560:alpha-L-fucosidase activity][GO:0005975:carbohydrate metabolic process]	[ko00511:Other glycan degradation]	-	XP_021842270.1:alphaLfucosidase 1like [Spinacia oleracea]	-	A0A0K9RR78|A0A0K9RR78_SPIOL:Uncharacterized protein;OS=Spinacia oleracea;|GO:0004560,|GO:0005975,
DN124185_c 2_g2_i2	UP	[GO:0016021:integral component of membrane][GO:0016760:cellulose synthase (UDP-forming) activity][GO:0030244:cellulose biosynthetic process]	-	FANhyb_rscf00001203.1.g00002.1:Fragaria x ananassa|LBD|LBD family protein	XP_010671117.1:PREDICTED: cellulose synthaselike protein D3 [Beta vulgaris subsp. vulgaris] >KMT16700.1 hypothetical protein BVRB_3g049840 [Beta vulgaris subsp. vulgaris]	gi|521526525|gb|GAHZ01010124.1|:TSA: Salicornia europaea Unigene10721_SeCKS transcribed RNA sequence	A0A0J8CSE6|A0A0J8CSE6_BETVU:Uncharacterized protein;OS=Beta vulgaris subsp. vulgaris.;|GO:0016021,|GO:0016760,|GO:0030244,

geneid	trend	GO	Pathway	TF	NR	NT	Uniprot
DN119570_c3_g4_i1	UP	[GO:0008080:N-acetyltransferase activity]	-	-	XP_021748681.1:uncharacterized protein LOC110714476 isoform X1 [Chenopodium quinoa]	-	A0A0D2QZP8|A0A0D2QZP8_GOSRA: Uncharacterized protein;OS=Gossypium raimondii;|GO:0008080,
DN127271_c2_g1_i1	UP	[GO:0005524:ATP binding][GO:0003883:CTP synthase activity][GO:0044210:'de novo' CTP biosynthetic process][GO:0006541:glutamine metabolic process]	[ko00240:Pyrimidine metabolism]	-	XP_021770131.1:CTP synthaselike [Chenopodium quinoa]	gi|32987484|dbj|AK102275.1|:Oryza sativa Japonica Group cDNA clone:J033089D14, full insert sequence	A0A0J8C8A9|A0A0J8C8A9_BETVU:CTP synthase;OS=Beta vulgaris subsp. vulgaris.;|GO:0005524,GO:0003883,|GO:0044210,GO:0006541,
DN120439_c3_g1_i1	UP	-	-	-	XP_010102559.1:hypothetical protein L484_018031 [Morus notabilis] >EXB93645.1 hypothetical protein L484_018031 [Morus notabilis]	gi|388508431|gb|BT142488.1|:Lotus japonicus clone JCVI-FLLj-7P10 unknown mRNA	W9RII0|W9RII0_9ROSA:Uncharacterized protein;OS=Morus notabilis.;

geneid	trend	GO	Pathway	TF	NR	NT	Uniprot
DN124723_c0_g2_i12	UP	-	-	-	XP_021856855.1:DNA repair RAD52like protein 1, mitochondrial [Spinacia oleracea] >KNA05357.1 hypothetical protein SOVF_191090 [Spinacia oleracea]	gi|224127741|ref|XM_002329130.1|:Populus trichocarpa predicted protein, mRNA	A0A0K9QDK3|A0A0K9QDK3_SPIOL: Uncharacterized protein;OS=Spinacia oleracea;
DN124181_c2_g2_i4	UP	[GO:0016021:integral component of membrane]	-	-	XP_021841915.1:uncharacterized protein LOC110782127 isoform X1 [Spinacia oleracea]	gi|359484136|ref|XM_002267935.2|:PREDICTED: Vitis vinifera uncharacterized LOC100243889 (LOC100243889), mRNA	A0A0K9RFS0|A0A0K9RFS0_SPIOL:Uncharacterized protein;OS=Spinacia oleracea;|GO:0016021,
DN126027_c2_g1_i4	UP	[GO:0050660:flavin adenine dinucleotide binding][GO:0016614:oxidoreductase activity, acting on CH-OH group of donors]	-	CCG025416.1:Populus euphratica|G2-like|G2-like family protein	XP_006440517.1:hypothetical protein CICLE_v10019427mg [Citrus clementina] >ESR53757.1 hypothetical protein CICLE_v10019427	gi|521516688|gb|GAHZ01015539.1|:TSA: Salicornia europaea Unigene16244_SeCKS transcribed RNA sequence	V4TK39|V4TK39_9ROSI:Uncharacterized protein;OS=Citrus clementina.;|GO:0050660,GO:0016614,

geneid	trend	GO	Pathway	TF	NR	NT	Uniprot
					mg [Citrus clementina]		
DN128531_c1_g2_i5	UP	-	-	-	XP_002524155.1:PREDICTED: uncharacterized protein LOC8274802 [Ricinus communis] >EEF38218.1 Interalphatrypsin inhibitor heavy chain H3 precursor, putative [Ricinus communis]	-	B9SDT6|B9SDT6_RICCO:Inter-alpha-trypsin inhibitor heavy chain H3, putative;OS=Ricinus communis;
DN116652_c2_g3_i1	UP	-	-	-	-	-	-

geneid	trend	GO	Pathway	TF	NR	NT	Uniprot
DN121485_c1_g1_i13	UP	[GO:0016021:integral component of membrane][GO:0004553:hydrolase activity, hydrolyzing O-glycosyl compounds][GO:0005975:carbohydrate metabolic process]	[ko00051:Fructose and mannose metabolism]	678452482:Utricularia gibba|WRKY|WRKY family protein	KNA09186.1:hypothetical protein SOVF_155690 [Spinacia oleracea]	gi|521736912|gb|GAIA01008452.1|:TSA: Salicornia europaea Unigene8788_Se200S transcribed RNA sequence	A0A0K9QRQ8|A0A0K9QRQ8_SPIOL: Uncharacterized protein;OS=Spinacia oleracea;|GO:0016021,|GO:0004553,|GO:0005975,
DN122808_c1_g2_i2	UP	[GO:0016021:integral component of membrane][GO:0005215:transporter activity][GO:0006814:sodium ion transport][GO:0055085:transmembrane transport]	-	Zmw_sc06488.1.g00020.1:Zoysia matrella|C3H|C3H family protein	XP_010690536.1:PREDICTED: dicarboxylate transporter 2, chloroplastic [Beta vulgaris subsp. vulgaris] >KMT01551.1 hypothetical protein BVRB_9g215520 [Beta vulgaris subsp. vulgaris]	gi|521577449|gb|GAIA01070380.1|:TSA: Salicornia europaea Unigene84693_Se200S transcribed RNA sequence	A0A0J8EF09|A0A0J8EF09_BETVU:Uncharacterized protein;OS=Beta vulgaris subsp. vulgaris.;|GO:0016021,|GO:0005215,|GO:0006814,GO:0055085,
DN113793_c0_g1_i1	UP	[GO:0016021:integral component of membrane][GO:0005524:ATP binding][GO:0004672:protein kinase activity]	-	Aco009877.1:Ananas comosus|WRKY|WRKY family protein	XP_010673059.1:PREDICTED: putative receptorlike protein kinase At3g47110 [Beta vulgaris subsp.	-	A0A0J8CT25|A0A0J8CT25_BETVU:Uncharacterized protein;OS=Beta vulgaris subsp. vulgaris.;|GO:00160

geneid	trend	GO	Pathway	TF	NR	NT	Uniprot
					vulgaris]		21,|GO:0005524,GO:0004672,
DN126810_c0_g1_i2	UP	[GO:0016021:integral component of membrane][GO:0004970:ionotropic glutamate receptor activity]	-	mrna09106.1-v1.0-hybrid:Fragaria vesca|C2H2|C2H2 family protein	XP_010689636.1:PREDICTED: glutamate receptor 3.4like [Beta vulgaris subsp. vulgaris]	gi|502083264|ref|XM_004487350.1|:PREDICTED: Cicer arietinum glutamate receptor 3.4-like (LOC101505478), transcript variant X5, mRNA	A0A0J8BPY7|A0A0J8BPY7_BETVU:Glutamate receptor;OS=Beta vulgaris subsp. vulgaris.;|GO:0016021,|GO:0004970,
DN105982_c1_g1_i1	UP	[GO:0016021:integral component of membrane][GO:0016491:oxidoreductase activity]	-	-	XP_012078863.1:ferric reduction oxidase 7, chloroplastic [Jatropha curcas] >KDP32466.1 hypothetical protein JCGZ_13391 [Jatropha curcas]	-	A0A067K893|A0A067K893_JATCU:Uncharacterized protein;OS=Jatropha curcas;|GO:0016021,|GO:0016491,

geneid	trend	GO	Pathway	TF	NR	NT	Uniprot
DN127616_c4_g1_i2	UP	-	[ko04137:Mitophagy - animal][ko04217:Necroptosis][ko04668:TNF signaling pathway]	-	KDO75962.1:hypothetical protein CISIN_1g022998mg [Citrus sinensis]	gi|521666413|gb|GAIA01034331.1|:TSA: Salicornia europaea Unigene38004_Se200S transcribed RNA sequence	A0A067GKI2|A0A067GKI2_CITSI:Uncharacterized protein;OS=Citrus sinensis;
DN124788_c0_g7_i16	UP	-	-	-	-	-	-
DN121188_c2_g3_i2	UP	[GO:0016021:integral component of membrane][GO:0055085:transmembrane transport]	-	BGIOSGA009723-PA:Oryza sativa subsp. indica|FAR1|FAR1 family protein	XP_021858240.1:probable metalnicotianamine transporter YSL7 [Spinacia oleracea] >KNA25526.1 hypothetical protein SOVF_005950 isoform A [Spinacia oleracea]	-	A0A0K9S199|A0A0K9S199_SPIOL:Uncharacterized protein;OS=Spinacia oleracea;|GO:0016021,|GO:0055085,
DN127809_c0_g1_i5	UP	[GO:0005622:intracellular][GO:0003746:translation elongation factor activity]	-	-	XP_021741470.1:uncharacterized protein LOC110707738 isoform X2 [Chenopodium quinoa]	gi|521512876|gb|GAHZ01018044.1|:TSA: Salicornia europaea Unigene18985_SeCKS transcribed RNA sequence	A0A0K9QMT7|A0A0K9QMT7_SPIOL:Uncharacterized protein;OS=Spinacia oleracea;|GO:0005622,|GO:0003746,

geneid	trend	GO	Pathway	TF	NR	NT	Uniprot
DN125535_c0_g1_i1	UP	-	-	-	XP_010667142.1:PREDICTED: uncharacterized protein LOC104884226 isoform X1 [Beta vulgaris subsp. vulgaris] >XP_010667143.1 PREDICTED: uncharacterized protein LOC104884226 isoform X1 [Beta vulgaris subsp. vulgaris] >XP_010667145.1 PREDICTED: uncharacterized protein LOC104884226 isoform X1 [Beta vulgaris subsp. vulgaris]	-	A0A0J8B395|A0A0J8B395_BETVU:Uncharacterized protein;OS=Beta vulgaris subsp. vulgaris.;

geneid	trend	GO	Pathway	TF	NR	NT	Uniprot
DN126404_c6_g2_i2	UP	[GO:0016021:integral component of membrane]	-	-	XP_021764363.1:BI1like protein [Chenopodium quinoa]	-	B9SFF7|B9SFF7_RICCO:Transmembrane BAX inhibitor motif-containing protein, putative;OS=Ricinus communis;|GO:0016021,
DN126951_c6_g1_i6	UP	[GO:0016021:integral component of membrane][GO:0008289:lipid binding]	-	-	XP_010696554.1:PREDICTED: synaptotagmin5 [Beta vulgaris subsp. vulgaris] >KMT18972.1 hypothetical protein BVRB_2g030700 [Beta vulgaris subsp. vulgaris]	gi|460398614|ref|XM_004244804.1|:PREDICTED: Solanum lycopersicum synaptotagmin-5-like (LOC101264538), mRNA	A0A0J8CZG6|A0A0J8CZG6_BETVU:Uncharacterized protein;OS=Beta vulgaris subsp. vulgaris.;|GO:0016021,|GO:0008289,
DN126472_c1_g2_i9	UP	[GO:0005096:GTPase activator activity][GO:0007165:signal transduction]	-	-	XP_021734234.1:rho GTPaseactivating protein 7like [Chenopodium quinoa]	gi|224105982|ref|XM_002313966.1|:Populus trichocarpa predicted protein, mRNA	A0A0K9S053|A0A0K9S053_SPIOL:Uncharacterized protein;OS=Spinacia oleracea;|GO:0005096,|GO:0007165,

geneid	trend	GO	Pathway	TF	NR	NT	Uniprot
DN124103_c0_g2_i8	UP	[GO:0016021:integral component of membrane][GO:0005215:transporter activity]	-	-	XP_021866383.1:metal transporter Nramp6like isoform X2 [Spinacia oleracea] >KNA10318.1 hypothetical protein SOVF_145490 [Spinacia oleracea]	-	A0A0K9QUD1|A0A0K9QUD1_SPIOL: Uncharacterized protein;OS=Spinacia oleracea;|GO:0016021,|GO:0005215,
DN124862_c0_g1_i13	UP	[GO:0005694:chromosome][GO:0003677:DNA binding][GO:0003917:DNA topoisomerase type I activity][GO:0008270:zinc ion binding][GO:0006265:DNA topological change]	[ko03440:Homologous recombination][ko03460:Fanconi anemia pathway]	MDP0000277077:Malus domestica|NF-YB|NF-YB family protein	XP_010674423.1:PREDICTED: DNA topoisomerase 3alpha [Beta vulgaris subsp. vulgaris] >KMT14051.1 hypothetical protein BVRB_4g078790 [Beta vulgaris subsp. vulgaris]	gi|449437639|ref|XM_004136551.1|:PREDICTED: Cucumis sativus topoisomerase (DNA) III alpha (TOP3A), mRNA	A0A0J8FDZ6|A0A0J8FDZ6_BETVU:DNA topoisomerase;OS=Beta vulgaris subsp. vulgaris.;|GO:0005694,|GO:0003677,GO:0003917,GO:0008270,|GO:0006265,

geneid	trend	GO	Pathway	TF	NR	NT	Uniprot
DN123012_c1_g4_i6	UP	[GO:0005525:GTP binding]	-	-	XP_010672952.1:PREDICTED: GTPbinding protein BRASSINAZOLE INSENSITIVE PALE GREEN 2, chloroplastic [Beta vulgaris subsp. vulgaris] >KMT15111.1 hypothetical protein BVRB_3g062300 [Beta vulgaris subsp. vulgaris]	gi|521634747|gb|GAIA01049048.1|:TSA: Salicornia europaea Unigene57393_Se200S transcribed RNA sequence	A0A0J8FGW4|A0A0J8FGW4_BETVU: Uncharacterized protein;OS=Beta vulgaris subsp. vulgaris.;|GO:0005525,
DN124056_c0_g2_i1	UP	-	-	-	-	-	-
DN114731_c5_g2_i1	UP	-	-	-	-	-	-

geneid	trend	GO	Pathway	TF	NR	NT	Uniprot
DN119875_c3_g3_i3	UP	[GO:0005524:ATP binding][GO:0004674:protein serine/threonine kinase activity]	-	mrna28322.1-v1.0-hybrid:Fragaria vesca|HD-ZIP|HD-ZIP family protein	XP_021848695.1:probable serine/threonineprotein kinase PIX13 [Spinacia oleracea] >KNA11255.1 hypothetical protein SOVF_136890 [Spinacia oleracea]	-	A0A0K9QVH7|A0A0K9QVH7_SPIOL: Uncharacterized protein;OS=Spinacia oleracea;|GO:0005524,GO:0004674,
DN123310_c1_g1_i21	UP	-	-	-	-	-	-
DN105666_c1_g1_i3	UP	[GO:0003993:acid phosphatase activity]	-	GSMUA_Achr8P25970_001:Musa acuminata|MYB|MYB family protein	XP_021860433.1:acid phosphatase 1like [Spinacia oleracea] >KNA20723.1 hypothetical protein SOVF_049830 [Spinacia oleracea]	-	A0A0K9RML3|A0A0K9RML3_SPIOL: Uncharacterized protein;OS=Spinacia oleracea;|GO:0003993,
DN129178_c13_g6_i7	UP	-	-	-	-	-	-

geneid	trend	GO	Pathway	TF	NR	NT	Uniprot
DN124924_c1_g1_i1	UP	[GO:0036459:thiol-dependent ubiquitinyl hydrolase activity][GO:0016579:protein deubiquitination][GO:0006511:ubiquitin-dependent protein catabolic process]	[ko04068:FoxO signaling pathway][ko05168: Herpes simplex infection][ko05169: Epstein-Barr virus infection][ko05203: Viral carcinogenesis]	-	XP_002309275.1:UBIQUITINSPECIFIC PROTEASE 12 family protein [Populus trichocarpa] >EEE92798.1 UBIQUITINSPECIFIC PROTEASE 12 family protein [Populus trichocarpa]	gi|359481112|ref|XM_002267519.2|:PREDICTED: Vitis vinifera ubiquitin carboxyl-terminal hydrolase 12-like (LOC100244518), mRNA	B9H9P5|B9H9P5_POPTR:UBIQUITIN-SPECIFIC PROTEASE 12 family protein;OS=Populus trichocarpa;|GO:0036459,|GO:0016579, GO:0006511,
DN117668_c0_g4_i3	UP	[GO:0008810:cellulase activity][GO:0030245:cellulose catabolic process]	[ko00500:Starch and sucrose metabolism]	mrna31843.1-v1.0-hybrid:Fragaria vesca|WRKY|WRKY family protein	XP_010677073.1:PREDICTED: endoglucanase 9 [Beta vulgaris subsp. vulgaris]	-	A0A068UTD0|A0A068UTD0_COFCA: Endoglucanase;OS=Coffea canephora;|GO:0008810,|GO:0030245,
DN122180_c0_g2_i6	UP	-	-	Achn177831:Actinidia chinensis|Nin-like|Nin-like family protein	XP_021676251.1:protein ABIL2like [Hevea brasiliensis]	-	B9RSD0|B9RSD0_RICCO:Protein ABIL2, putative;OS=Ricinus communis;

geneid	trend	GO	Pathway	TF	NR	NT	Uniprot
DN125796_c5_g8_i6	UP	-	-	-	XP_021863503.1:(+)menthofuran synthaselike [Spinacia oleracea]	-	-
DN116019_c0_g2_i3	UP	-	-	-	-	-	-
DN127680_c0_g2_i3	UP	[GO:0016021:integral component of membrane][GO:0005524:ATP binding][GO:0042626:ATPase activity, coupled to transmembrane movement of substances]	-	RrC2480_p5:Raphanus raphanistrum|HSF|HSF family protein	XP_021853038.1:putative ABC transporter C family member 15 [Spinacia oleracea]	gi|255588744|ref|XM_002534659.1|:Ricinus communis multidrug resistance-associated protein 2, 6 (mrp2, 6), abc-transoprter, putative, mRNA	A0A0J8BIE8|A0A0J8BIE8_BETVU:Uncharacterized protein;OS=Beta vulgaris subsp. vulgaris.;|GO:0016021,|GO:0005524,GO:0042626,
DN125492_c5_g1_i2	UP	-	-	-	XP_021291835.1:UPF0613 protein PB24D3.06c [Herrania umbratica]	gi|388514472|gb|BT145504.1|:Lotus japonicus clone JCVI-FLLj-3J23 unknown mRNA	A0A0J8BFE2|A0A0J8BFE2_BETVU:Uncharacterized protein;OS=Beta vulgaris subsp. vulgaris.;

geneid	trend	GO	Pathway	TF	NR	NT	Uniprot
DN126051_c1_g4_i3	UP	[GO:0005634:nucleus][GO:0003677:DNA binding][GO:0006355:regulation of transcription, DNA-templated][GO:0006351:transcription, DNA-templated]	-	AHYPO_015098-RA:Amaranthus hypochondriacus|G2-like|G2-like family protein	XP_021739925.1:myb family transcription factor PHL8like isoform X1 [Chenopodium quinoa]	-	A0A0J8EV67|A0A0J8EV67_BETVU:Uncharacterized protein;OS=Beta vulgaris subsp. vulgaris.;|GO:0005634,|GO:0003677,|GO:0006355,GO:0006351,
DN114833_c1_g1_i3	UP	-	-	Lus10036329:Linum usitatissimum|GATA|GATA family protein	XP_010684624.1:PREDICTED: uncharacterized protein LOC104899184 [Beta vulgaris subsp. vulgaris] >KMT05854.1 hypothetical protein BVRB_7g165170 [Beta vulgaris subsp. vulgaris]	-	A0A0J8C0L3|A0A0J8C0L3_BETVU:Uncharacterized protein;OS=Beta vulgaris subsp. vulgaris.;

geneid	trend	GO	Pathway	TF	NR	NT	Uniprot
DN127630_c1_g7_i1	UP	[GO:0005737:cytoplasm][GO:0008234:cysteine-type peptidase activity][GO:0006914:autophagy][GO:0015031:protein transport]	[ko04136:Autophagy - other][ko04138:Autophagy - yeast][ko04140:Autophagy - animal]	-	XP_021855598.1:cysteine protease ATG4like isoform X2 [Spinacia oleracea] >KNA05567.1 hypothetical protein SOVF_189160 isoform A [Spinacia oleracea]	-	A0A0K9QE75|A0A0K9QE75_SPIOL:Uncharacterized protein;OS=Spinacia oleracea;|GO:0005737,|GO:0008234,|GO:0006914,GO:0015031,
DN125567_c0_g1_i1	UP	-	-	-	-	gi|332656411|gb|CP002687.1|:Arabidopsis thaliana chromosome 4, complete genome	-
DN126538_c0_g2_i4	UP	[GO:0003676:nucleic acid binding][GO:0004523:RNA-DNA hybrid ribonuclease activity]	-	-	XP_021757487.1:uncharacterized protein LOC110722525 [Chenopodium quinoa]	-	F4NCH2|F4NCH2_BETVU:Uncharacterized protein;OS=Beta vulgaris subsp. vulgaris.;|GO:0003676,GO:0004523,

geneid	trend	GO	Pathway	TF	NR	NT	Uniprot
DN116058_c3_g1_i1	UP	[GO:0046872:metal ion binding][GO:0016702:oxidoreductase activity, acting on single donors with incorporation of molecular oxygen, incorporation of two atoms of oxygen][GO:0006633:fatty acid biosynthetic process][GO:0031408:oxylipin biosynthetic process]	[ko00591:Linoleic acid metabolism][ko00592:alpha-Linolenic acid metabolism]	Tp57577_TGAC_v2_mRNA8161:Trifolium pratense|MYB_related|MYB_related family protein	ACQ76787.1:lipoxygenase [Camellia sinensis]	-	C4NZX3|C4NZX3_CAMSI:Lipoxygenase;OS=Camellia sinensis;|GO:0046872,GO:0016702,|GO:0006633,GO:0031408,
DN126876_c0_g1_i3	UP	[GO:0030598:rRNA N-glycosylase activity][GO:0051607:defense response to virus][GO:0017148:negative regulation of translation]	-	-	Q40772.1:RecName: Full=Antiviral protein 2; AltName: Full=PAPII; AltName: Full=Ribosomeinactivating protein; AltName: Full=rRNA Nglycosidase; Flags: Precursor >CAA55342.1 pokeweed antiviral protein II [Phytolacca	-	Q40772|RIP2_PHYAM:Antiviral protein 2;OS=Phytolacca americana;|GO:0030598,|GO:0051607,GO:0017148,

geneid	trend	GO	Pathway	TF	NR	NT	Uniprot
					americana] >prf||2014245A antiviral protein II		
DN128320_c1_g1_i4	UP	-	-	-	XP_021715314.1:random slug protein 5like [Chenopodium quinoa]	-	A0A0K9RRN1|A0A0K9RRN1_SPIOL: Uncharacterized protein;OS=Spinacia oleracea;
DN124560_c0_g1_i8	UP	[GO:0016021:integral component of membrane][GO:0005506:iron ion binding][GO:0016491:oxidoreductase activity][GO:0006633:fatty acid biosynthetic process]	[ko00906:Carotenoid biosynthesis]	-	XP_021726730.1:betacarotene hydroxylase 2, chloroplasticlike [Chenopodium quinoa]	-	A0A0K9RTT3|A0A0K9RTT3_SPIOL:Uncharacterized protein;OS=Spinacia oleracea;|GO:0016021,|GO:0005506,GO:0016491,|GO:0006633,

geneid	trend	GO	Pathway	TF	NR	NT	Uniprot
DN120505_c5_g4_i1	UP	-	[ko04141:Protein processing in endoplasmic reticulum]	-	XP_010678070.1:PREDICTED: hsp70binding protein 1 [Beta vulgaris subsp. vulgaris] >KMT11049.1 hypothetical protein BVRB_5g111390 [Beta vulgaris subsp. vulgaris]	-	A0A0J8CG16|A0A0J8CG16_BETVU:Uncharacterized protein;OS=Beta vulgaris subsp. vulgaris.;
DN83037_c0_g1_i1	UP	-	-	-	-	-	-
DN126290_c2_g5_i21	UP	[GO:0016779:nucleotidyltransferase activity][GO:0003723:RNA binding][GO:0006396:RNA processing]	-	-	KNA18888.1:hypothetical protein SOVF_066910 [Spinacia oleracea]	gi|47104840|gb|BT013425.1|:Lycopersicon esculentum clone 132065F, mRNA sequence	A0A0K9RHB1|A0A0K9RHB1_SPIOL: Uncharacterized protein;OS=Spinacia oleracea;|GO:0016779,GO:0003723,|GO:0006396,
DN121295_c2_g2_i11	UP	[GO:0043565:sequence-specific DNA binding][GO:0003700:DNA-binding transcription factor activity]	-	Sp_110710_zzug.t1: Spinacia oleracea|WRKY|WRKY family protein	XP_021748208.1:probable WRKY transcription factor 41 [Chenopodium	-	A0A0K9R5K5|A0A0K9R5K5_SPIOL:Uncharacterized protein;OS=Spinacia

geneid	trend	GO	Pathway	TF	NR	NT	Uniprot
					quinoa]		oleracea;|GO:0043565,GO:0003700,
DN121921_c2_g1_i9	UP	[GO:0016592:mediator complex][GO:0009631:cold acclimation][GO:0010150:leaf senescence][GO:0006355:regulation of transcription, DNA-templated][GO:0048364:root development]	-	-	XP_003631633.1:PREDICTED: mediator of RNA polymerase II transcription subunit 32 [Vitis vinifera]	gi|502130934|ref|XM_004500763.1|:PREDICTED: Cicer arietinum mediator of RNA polymerase II transcription subunit 32-like (LOC101497203), mRNA	F6HQK7|F6HQK7_VITVI:Putative uncharacterized protein;OS=Vitis vinifera;GN=VIT_03s0063g02280{ECO:0000313|EMBL:CCB56964.1};|GO:0016592,|GO:0009631,GO:0010150,GO:0006355,GO:0048364,
DN108328_c0_g2_i1	UP	[GO:0016021:integral component of membrane]	-	-	XP_010696182.1:PREDICTED: ankyrin1like [Beta vulgaris subsp. vulgaris]	-	A0A0J8BAZ9|A0A0J8BAZ9_BETVU:Uncharacterized protein;OS=Beta vulgaris subsp. vulgaris.;|GO:0016021,

geneid	trend	GO	Pathway	TF	NR	NT	Uniprot
DN121434_c1_g1_i7	UP	[GO:0009941:chloroplast envelope][GO:0003824:catalytic activity]	-	XP_010558909.1:Tarenaya hassleriana|M-type_MADS|M-type_MADS family protein	XP_010679754.1:PREDICTED: pheophytinase, chloroplastic [Beta vulgaris subsp. vulgaris] >KMT09433.1 hypothetical protein BVRB_6g129050 [Beta vulgaris subsp. vulgaris]	gi|388507221|gb|BT141883.1|:Lotus japonicus clone JCVI-FLLj-9O11 unknown mRNA	A0A0J8CBT2|A0A0J8CBT2_BETVU:Uncharacterized protein;OS=Beta vulgaris subsp. vulgaris.;|GO:0009941,|GO:0003824,
DN116401_c3_g1_i3	UP	-	-	-	XP_010685303.1:PREDICTED: uncharacterized protein LOC104899744 [Beta vulgaris subsp. vulgaris] >KMT04821.1 hypothetical protein BVRB_7g169920 [Beta vulgaris subsp. vulgaris]	-	A0A0J8EPA9|A0A0J8EPA9_BETVU:Uncharacterized protein;OS=Beta vulgaris subsp. vulgaris.;

geneid	trend	GO	Pathway	TF	NR	NT	Uniprot
DN128783_c1_g3_i8	UP	-	[ko03013:RNA transport]	-	XP_010690435.1:PREDICTED: uncharacterized protein LOC104903976 [Beta vulgaris subsp. vulgaris] >KMT01450.1 hypothetical protein BVRB_9g214570 [Beta vulgaris subsp. vulgaris]	gi|521509898|gb|GAHZ01021022.1|:TSA: Salicornia europaea Unigene22230_SeCKS transcribed RNA sequence	A0A0J8BJ33|A0A0J8BJ33_BETVU:Uncharacterized protein;OS=Beta vulgaris subsp. vulgaris.;
DN193768_c0_g1_i1	UP	-	-	-	XP_021734036.1:uncharacterized protein LOC110700741 [Chenopodium quinoa]	-	-
DN126868_c0_g4_i1	UP	[GO:0050660:flavin adenine dinucleotide binding][GO:0017150:tRNA dihydrouridine synthase activity]	-	Thecc1EG000515t1:Theobroma cacao|B3|B3 family protein	XP_021735039.1:tRNAdihydrouridine(16/17) synthase [NAD(P)(+)]like [Chenopodium quinoa]	gi|521734314|gb|GAIA01009755.1|:TSA: Salicornia europaea Unigene10191_Se200S transcribed RNA sequence	A0A0J8CBY9|A0A0J8CBY9_BETVU: Uncharacterized protein;OS=Beta vulgaris subsp. vulgaris.;|GO:0050660,GO:0017150,

geneid	trend	GO	Pathway	TF	NR	NT	Uniprot
DN118105_c1_g2_i7	UP	[GO:0016021:integral component of membrane]	-	-	XP_021734094.1:WAT1related protein At3g02690, chloroplasticlike [Chenopodium quinoa]	gi|521551022|gb|GAIA01081971.1|:TSA: Salicornia europaea Unigene100081_Se200S transcribed RNA sequence	A0A0K9RJ91|A0A0K9RJ91_SPIOL:Uncharacterized protein;OS=Spinacia oleracea;|GO:0016021,
DN125543_c3_g1_i11	UP	-	-	-	-	-	-
DN119512_c4_g1_i3	UP	[GO:0048046:apoplast][GO:0009941:chloroplast envelope][GO:0009570:chloroplast stroma][GO:0009535:chloroplast thylakoid membrane][GO:0010319:stromule][GO:0051287:NAD binding][GO:0050661:NADP binding][GO:0016620:oxidoreductase activity, acting on the aldehyde or oxo group of donors, NAD or NADP as acceptor][GO:0006006:glucose metabolic process]	[ko00710:Carbon fixation in photosynthetic organisms]	MDP0000275412:Malus domestica|Trihelix|Trihelix family protein	XP_010686856.1:PREDICTED: glyceraldehyde3phosphate dehydrogenase B, chloroplastic [Beta vulgaris subsp. vulgaris] >KMT19244.1 hypothetical protein BVRB_1g014260 [Beta vulgaris subsp. vulgaris]	gi|512395725|gb|GAJZ01005445.1|:TSA: Prunus avium Pa_14655 transcribed RNA sequence	A0A0J8D4K0|A0A0J8D4K0_BETVU:Glyceraldehyde-3-phosphate dehydrogenase;OS=Beta vulgaris subsp. vulgaris.;|GO:0048046,GO:0009941,GO:0009570,GO:0009535,GO:0010319,|GO:0051287,GO:0050661,GO:0016620,|GO:0006006,

geneid	trend	GO	Pathway	TF	NR	NT	Uniprot
DN119608_c2_g1_i5	UP	[GO:0005524:ATP binding][GO:0008026:ATP-dependent helicase activity][GO:0003676:nucleic acid binding]	[ko03040:Spliceosome]	KXZ49867.1:Gonium pectorale|C2H2|C2H2 family protein	XP_021744633.1:probable premRNAsplicing factor ATPdependent RNA helicase DEAH4 [Chenopodium quinoa]	-	A0A0J8C196|A0A0J8C196_BETVU:Uncharacterized protein;OS=Beta vulgaris subsp. vulgaris.;|GO:0005524,GO:0008026,GO:0003676,
DN120009_c1_g3_i8	UP	[GO:0016272:prefoldin complex][GO:0006457:protein folding]	-	-	XP_012067711.1:protein UXT homolog [Jatropha curcas] >KDP41248.1 hypothetical protein JCGZ_15655 [Jatropha curcas]	gi|521670625|gb|GAIA01032212.1|:TSA: Salicornia europaea Unigene35350_Se200S transcribed RNA sequence	A0A067L233|A0A067L233_JATCU:Uncharacterized protein;OS=Jatropha curcas;|GO:0016272,|GO:0006457,
DN126166_c1_g4_i9	UP	-	-	-	XP_021846853.1:methyltransferaselike protein 22 isoform X2 [Spinacia oleracea]	gi|521524196|gb|GAHZ01011836.1|:TSA: Salicornia europaea Unigene12456_SeCKS transcribed RNA sequence	A0A0J8ECQ1|A0A0J8ECQ1_BETVU:Uncharacterized protein;OS=Beta vulgaris subsp. vulgaris.;

geneid	trend	GO	Pathway	TF	NR	NT	Uniprot
DN127980_c3_g2_i5	UP	[GO:0016021:integral component of membrane][GO:0016760:cellulose synthase (UDP-forming) activity][GO:0030244:cellulose biosynthetic process]	-	-	KMS99540.1:hypothetical protein BVRB_1g023090 isoform B [Beta vulgaris subsp. vulgaris]	-	A0A0J8E8W8|A0A0J8E8W8_BETVU: Uncharacterized protein;OS=Beta vulgaris subsp. vulgaris.;|GO:0016021,|GO:0016760,|GO:0030244,
DN112133_c1_g1_i2	UP	[GO:0004725:protein tyrosine phosphatase activity][GO:0008138:protein tyrosine/serine/threonine phosphatase activity]	-	-	KNA04167.1:hypothetical protein SOVF_202180 [Spinacia oleracea]	-	A0A0K9QA68|A0A0K9QA68_SPIOL:Uncharacterized protein;OS=Spinacia oleracea;|GO:0004725,GO:0008138,
DN128501_c0_g1_i15	UP	-	-	-	XP_021847150.1:protein SWEETIE [Spinacia oleracea]	gi|521708661|gb|GAIA01013788.1|:TSA: Salicornia europaea Unigene14432_Se200S transcribed RNA sequence	A0A0J8CY60|A0A0J8CY60_BETVU:Uncharacterized protein;OS=Beta vulgaris subsp. vulgaris.;

geneid	trend	GO	Pathway	TF	NR	NT	Uniprot
DN119000_c2_g2_i3	UP	[GO:0016810:hydrolase activity, acting on carbon-nitrogen (but not peptide) bonds][GO:0006807:nitrogen compound metabolic process]	[ko00460:Cyanoamino acid metabolism]	WALNUT_00024380-RA:Juglans regia|TCP|TCP family protein	XP_014518652.1:PREDICTED: bifunctional nitrilase/nitrile hydratase NIT4A [Vigna radiata var. radiata]	gi|359479486|ref|XM_002272801.2|:PREDICTED: Vitis vinifera bifunctional nitrilase/nitrile hydratase NIT4B-like (LOC100250098), mRNA	A0A0L9V641|A0A0L9V641_PHAAN:Uncharacterized protein;OS=Phaseolus angularis;|GO:0016810,|GO:0006807,
DN121392_c0_g1_i2	UP	-	-	-	XP_021746660.1:uncharacterized protein LOC110712505 [Chenopodium quinoa]	-	A0A0J7YLV9|A0A0J7YLV9_BETVU:Uncharacterized protein;OS=Beta vulgaris subsp. vulgaris.;
DN121619_c1_g6_i6	UP	-	-	-	XP_021734917.1:uncharacterized protein LOC110701594 [Chenopodium quinoa]	-	A0A0J8B8X6|A0A0J8B8X6_BETVU:Uncharacterized protein;OS=Beta vulgaris subsp. vulgaris.;

geneid	trend	GO	Pathway	TF	NR	NT	Uniprot
DN117632_c0_g2_i3	UP	[GO:0009507:chloroplast]	-	-	XP_021762789.1:uncharacterized protein LOC110727536 [Chenopodium quinoa]	gi|224129343|ref|XM_002320527.1|:Populus trichocarpa predicted protein, mRNA	A0A0J8C8V3|A0A0J8C8V3_BETVU:Uncharacterized protein;OS=Beta vulgaris subsp. vulgaris.;|GO:0009507,
DN118962_c3_g3_i1	UP	-	-	-	-	-	-
DN121530_c4_g1_i1	UP	-	[ko04141:Protein processing in endoplasmic reticulum]	WALNUT_00030998-RA:Juglans regia|NF-YB|NF-YB family protein	XP_021716168.1:17.4 kDa class I heat shock proteinlike [Chenopodium quinoa]	-	D2T0E1|D2T0E1_9ERIC:Cytosolic class I small heat shock protein type 1;OS=Rhododendron ellipticum.;
DN120766_c1_g1_i1	UP	[GO:0016756:glutathione gamma-glutamylcysteinyltransferase activity][GO:0046872:metal ion binding][GO:0046938:phytochelatin biosynthetic process][GO:0010038:response to metal ion]	-	-	XP_021722235.1:glutathione gammaglutamylcysteinyltransferase 1like isoform X1 [Chenopodium quinoa]	gi|224129599|ref|XM_002320590.1|:Populus trichocarpa predicted protein, mRNA	A0A0J8BXF0|A0A0J8BXF0_BETVU:Uncharacterized protein;OS=Beta vulgaris subsp. vulgaris.;|GO:0016756,GO:0046872,|GO:0046938,GO:0010038,

geneid	trend	GO	Pathway	TF	NR	NT	Uniprot
DN117689_c1_g1_i6	UP	[GO:0016021:integral component of membrane][GO:0016787:hydrolase activity][GO:0006629:lipid metabolic process]	-	-	XP_021768782.1:uncharacterized protein LOC110733070 [Chenopodium quinoa]	gi|521735544|gb|GAIA01009137.1|:TSA: Salicornia europaea Unigene9496_Se200S transcribed RNA sequence	A0A0J8C8K8|A0A0J8C8K8_BETVU:Uncharacterized protein;OS=Beta vulgaris subsp. vulgaris.;|GO:0016021,|GO:0016787,|GO:0006629,
DN116972_c0_g3_i6	UP	-	-	Rsa1.0_00424.1_g00007.1:Raphanus sativus|C2H2|C2H2 family protein	XP_010684619.1:PREDICTED: uncharacterized protein LOC104899180 [Beta vulgaris subsp. vulgaris]	-	A0A0V0IYZ4|A0A0V0IYZ4_SOLCH:Putative ovule protein;OS=Solanum chacoense;

geneid	trend	GO	Pathway	TF	NR	NT	Uniprot
DN127260_c1_g5_i3	UP	[GO:0009507:chloroplast][GO:0046422:violaxanthin de-epoxidase activity]	[ko00906:Carotenoid biosynthesis]	-	XP_010674199.1:PREDICTED: violaxanthin deepoxidase, chloroplastic [Beta vulgaris subsp. vulgaris] >XP_010674200.1 PREDICTED: violaxanthin deepoxidase, chloroplastic [Beta vulgaris subsp. vulgaris] >XP_010674201.1 PREDICTED: violaxanthin deepoxidase, chloroplastic [Beta vulgaris subsp. vulgaris] >KMT13852.1 hypothetical protein BVRB_4g076960 [Beta vulgaris subsp.	gi|17221328|emb|AJ250433.2|:Spinacia oleracea mRNA for violaxanthin de-epoxidase (svde1 gene)	A0A0J8CJY2|A0A0J8CJY2_BETVU:Uncharacterized protein;OS=Beta vulgaris subsp. vulgaris.;|GO:0009507,|GO:0046422,

geneid	trend	GO	Pathway	TF	NR	NT	Uniprot
					vulgaris]		
DN121573_c2_g2_i1	UP	[GO:0003676:nucleic acid binding]	-	-	CCA66153.1:hypothetical protein [Beta vulgaris subsp. vulgaris]	-	F4NCK1|F4NCK1_BETVU:Uncharacterized protein;OS=Beta vulgaris subsp. vulgaris.;|GO:00036

geneid	trend	GO	Pathway	TF	NR	NT	Uniprot
							76,
DN126009_c1_g1_i6	UP	[GO:0016020:membrane][GO:0020037:heme binding][GO:0005506:iron ion binding][GO:0016709:oxidoreductase activity, acting on paired donors, with incorporation or reduction of molecular oxygen, NAD(P)H as one donor, and incorporation of one atom of oxygen][GO:0044550:secondary metabolite biosynthetic process]	-	Sme2.5_00297.1_g00019.1:Solanum melongena|MYB|MYB family protein	XP_021863527.1:cytochrome P450 71A26like isoform X1 [Spinacia oleracea]	-	D7SHG6|D7SHG6_VITVI:Putative uncharacterized protein;OS=Vitis vinifera;GN=VIT_17s0000g09510 {ECO:0000313|EMBL:CBI14925.3};|GO:0016020,|GO:0020037,GO:0005506,GO:0016709,|GO:0044550,
DN120278_c2_g6_i1	UP	-	-	-	-	-	-

geneid	trend	GO	Pathway	TF	NR	NT	Uniprot
DN126009_c1_g1_i1	UP	[GO:0016021:integral component of membrane][GO:0020037:heme binding][GO:0005506:iron ion binding][GO:0004497:monooxygenase activity][GO:0016705:oxidoreductase activity, acting on paired donors, with incorporation or reduction of molecular oxygen]	-	OPUNC10G09700.4:Oryza punctata|E2F/DP|E2F/DP family protein	XP_021863527.1:cytochrome P450 71A26like isoform X1 [Spinacia oleracea]	-	A5B9L0|A5B9L0_VITVI:Putative uncharacterized protein;OS=Vitis vinifera;|GO:0016021,|GO:0020037,GO:0005506,GO:0004497,GO:0016705,
DN121525_c2_g1_i1	UP	[GO:0003676:nucleic acid binding]	-	-	CCA66140.1:hypothetical protein [Beta vulgaris subsp. vulgaris]	-	F4NCJ8|F4NCJ8_BETVU:Uncharacterized protein;OS=Beta vulgaris subsp. vulgaris.;|GO:0003676,
DN114224_c0_g1_i4	UP	[GO:0004375:glycine dehydrogenase (decarboxylating) activity][GO:0006546:glycine catabolic process]	[ko00260:Glycine, serine and threonine metabolism][ko00630:Glyoxylate and dicarboxylate metabolism]	mrna02686.1-v1.0-hybrid:Fragaria vesca|GATA|GATA family protein	XP_012077290.1:glycine dehydrogenase (decarboxylating), mitochondrial [Jatropha curcas] >KDP34101.1 hypothetical protein	gi|470109507|ref|XM_004290991.1|:PREDICTED: Fragaria vesca subsp. vesca glycine dehydrogenase [decarboxylating],	A0A067KD00|A0A067KD00_JATCU: Uncharacterized protein;OS=Jatropha curcas;|GO:0004375,|GO:0006546,

geneid	trend	GO	Pathway	TF	NR	NT	Uniprot
					JCGZ_07672 [Jatropha curcas]	mitochondrial-like (LOC101308733), mRNA	
DN123291_c 3_g14_i3	UP	-	-	-	XP_003565108.1:P REDICTED: protein PHLOEM PROTEIN 2LIKE A1like [Brachypodium distachyon] >XP_003566130.1 PREDICTED: protein PHLOEM PROTEIN 2LIKE A1like [Brachypodium distachyon] >KQK02231.1 hypothetical protein BRADI_2g00232 [Brachypodium	-	I1HB14|I1HB14_BR ADI:Uncharacterize d protein;OS=Brachyp odium distachyon;

geneid	trend	GO	Pathway	TF	NR	NT	Uniprot
					distachyon] >KQK02236.1 hypothetical protein BRADI_2g00250 [Brachypodium distachyon]		
DN119920_c2_g5_i1	UP	-	[ko00902:Monoterpenoid biosynthesis]	-	XP_021760280.1:(+)neomenthol dehydrogenaselike [Chenopodium quinoa] >XP_021717522.1 (+)neomenthol dehydrogenaselike	-	A0A0K9R8D1|A0A0K9R8D1_SPIOL:Uncharacterized protein;OS=Spinacia oleracea;

geneid	trend	GO	Pathway	TF	NR	NT	Uniprot
					[Chenopodium quinoa]		
DN128247_c0_g1_i3	UP	-	[ko04016:MAPK signaling pathway - plant][ko04075:Plant hormone signal transduction]	PSME_00044429-RA:Pseudotsuga menziesii|MYB_related|MYB_related family protein	XP_010677239.1:PREDICTED: abscisic acid receptor PYR1 [Beta vulgaris subsp. vulgaris] >KMT11653.1 hypothetical protein BVRB_5g105930 [Beta vulgaris subsp. vulgaris]	-	A0A0J8CHW7|A0A0J8CHW7_BETVU: Uncharacterized protein;OS=Beta vulgaris subsp. vulgaris.;
DN122940_c3_g5_i4	UP	[GO:0008146:sulfotransferase activity]	-	676777512:Sisymbrium irio|ERF|ERF family protein	XP_010672565.1:PREDICTED: cytosolic sulfotransferase 5like [Beta vulgaris subsp. vulgaris]	-	A0A0K9RVR2|A0A0K9RVR2_SPIOL:Sulfotransferase;OS=Spinacia oleracea;|GO:0008146,
DN126037_c	UP	-	-	-	-	-	-

geneid	trend	GO	Pathway	TF	NR	NT	Uniprot
3_g4_i8							
DN126892_c 2_g1_i15	UP	-	-	-	XP_010671966.1:P REDICTED: uncharacterized protein LOC104888641 [Beta vulgaris subsp. vulgaris] >XP_010671968.1 PREDICTED: uncharacterized protein LOC104888641 [Beta vulgaris subsp. vulgaris] >KMT20615.1 hypothetical protein BVRB_1g005940 [Beta vulgaris subsp. vulgaris]	gi|521671339|gb|GA IA01031858.1|:TSA: Salicornia europaea Unigene34959_Se20 0S transcribed RNA sequence	A0A0J8D387|A0A0 J8D387_BETVU:Un characterized protein;OS=Beta vulgaris subsp. vulgaris.;

geneid	trend	GO	Pathway	TF	NR	NT	Uniprot
DN121968_c2_g4_i2	UP	[GO:0016021:integral component of membrane][GO:0005524:ATP binding][GO:0004672:protein kinase activity]	[ko04064:NF-kappa B signaling pathway][ko04620:Toll-like receptor signaling pathway][ko04621:NOD-like receptor signaling pathway][ko04624:Toll and Imd signaling pathway][ko04722:Neurotrophin signaling pathway][ko05133:Pertussis][ko05140:Leishmaniasis][ko05142:Chagas disease (American trypanosomiasis)][ko05145:Toxoplasmosis][ko05152:Tuberculosis][ko05162:Measles][ko05164:Influenza A]	312791:Arabidopsis lyrata|bHLH|bHLH family protein	XP_021862646.1:LEAF RUST 10 DISEASERESISTANCE LOCUS RECEPTORLIKE PROTEIN KINASElike 1.3 isoform X2 [Spinacia oleracea]	gi|521667582|gb|GAIA01033743.1|:TSA: Salicornia europaea Unigene37040_Se200S transcribed RNA sequence	A0A0K9QW24|A0A0K9QW24_SPIOL: Uncharacterized protein;OS=Spinacia oleracea;|GO:0016021,|GO:0005524,GO:0004672,

geneid	trend	GO	Pathway	TF	NR	NT	Uniprot
DN122471_c0_g1_i11	UP	[GO:0008661:1-deoxy-D-xylulose-5-phosphate synthase activity][GO:0016114:terpenoid biosynthetic process]	[ko00730:Thiamine metabolism][ko00900:Terpenoid backbone biosynthesis]	-	AAQ84169.1:1deoxyDxylulose 5phosphate synthase [Pueraria montana var. lobata]	gi|35187001|gb|AY315652.1|:Pueraria montana var. lobata 1-deoxy-D-xylulose 5-phosphate synthase (Dxs) mRNA, complete cds	Q6EJC9|Q6EJC9_PUEML:1-deoxy-D-xylulose 5-phosphate synthase;OS=Pueraria montana var. lobata;|GO:0008661,|GO:0016114,
DN115332_c0_g1_i1	UP	[GO:0003924:GTPase activity][GO:0016151:nickel cation binding][GO:0006807:nitrogen compound metabolic process]	-	-	XP_019190146.1:PREDICTED: urease accessory protein G [Ipomoea nil]	gi|478161251|gb|GAHK01015352.1|:TSA: Jatropha curcas Unigene2806_JC-CK_1A transcribed RNA sequence	Q9AR64|Q9AR64_SOLTU:Urease accessory protein G;OS=Solanum tuberosum;|GO:0003924,GO:0016151,|GO:0006807,
DN121051_c2_g4_i14	UP	[GO:0016740:transferase activity][GO:0008299:isoprenoid biosynthetic process]	[ko00900:Terpenoid backbone biosynthesis]	889097:Arabidopsis lyrata|STAT|STAT family protein	XP_021846071.1:solanesyldiphosphate synthase 1, mitochondriallike [Spinacia oleracea]	-	A0A0K9QK47|A0A0K9QK47_SPIOL:Uncharacterized protein;OS=Spinacia oleracea;|GO:0016740,|GO:0008299,

geneid	trend	GO	Pathway	TF	NR	NT	Uniprot
DN127654_c3_g4_i4	UP	[GO:0016021:integral component of membrane]	-	mrna12442.1-v1.0-hybrid:Fragaria vesca|NAC|NAC family protein	XP_019103735.1:PREDICTED: uncharacterized protein LOC104887465 [Beta vulgaris subsp. vulgaris]	gi|359482029|ref|XM_002275830.2|:PREDICTED: Vitis vinifera IAA-amino acid hydrolase ILR1-like (LOC100266557), mRNA	A0A0J8CYE6|A0A0J8CYE6_BETVU:Uncharacterized protein;OS=Beta vulgaris subsp. vulgaris.;|GO:0016021,
DN126637_c0_g4_i1	UP	-	-	LOC_Os11g12360.1:Oryza sativa subsp. japonica|M-type_MADS|M-type_MADS family protein	XP_010668412.1:PREDICTED: uncharacterized protein LOC104885415 [Beta vulgaris subsp. vulgaris]	gi|352962224|gb|AC245990.4|:Solanum lycopersicum strain Heinz 1706 chromosome 1 clone sle-60e12 map 1, complete sequence	Q7G3C6|Q7G3C6_ORYSJ:Transposon protein, putative, CACTA, En/Spm sub-class;OS=Oryza sativa subsp. japonica;GN=LOC_Os10g21980 {ECO:0000313|EMBL:AAP53328.2};
DN113800_c0_g1_i1	UP	[GO:0016491:oxidoreductase activity]	-	EcC042108.10:Eucalyptus camaldulensis|C2H2|C2H2 family protein	XP_021685965.1:nonfunctional NADPHdependent codeinone reductase 2like [Hevea brasiliensis]	gi|356577290|ref|XM_003556713.1|:PREDICTED: Glycine max NAD(P)H-dependent 6'-deoxychalcone synthase-like	A0A059D6C3|A0A059D6C3_EUCGR:Uncharacterized protein;OS=Eucalyptus grandis;|GO:0016491,

geneid	trend	GO	Pathway	TF	NR	NT	Uniprot
						(LOC100790074), mRNA	
DN124540_c0_g1_i2	UP	[GO:0006950:response to stress]	-	Bv3_057240_hnxc.t1:Beta vulgaris|HSF|HSF family protein	XP_010673093.1:PREDICTED: putative disease resistance RPP13like protein 1 [Beta vulgaris subsp. vulgaris] >KMT15250.1 hypothetical protein BVRB_3g063590 [Beta vulgaris subsp. vulgaris]	-	A0A0J8CNA5|A0A0J8CNA5_BETVU: Uncharacterized protein;OS=Beta vulgaris subsp. vulgaris.;|GO:0006950,
DN127739_c2_g1_i1	UP	-	-	-	-	-	-

geneid	trend	GO	Pathway	TF	NR	NT	Uniprot
DN125626_c0_g3_i12	UP	[GO:0004721:phosphoprotein phosphatase activity]	[ko03015:mRNA surveillance pathway][ko04013:MAPK signaling pathway - fly][ko04071:Sphingolipid signaling pathway][ko04111:Cell cycle - yeast][ko04113:Meiosis - yeast][ko04114:Oocyte meiosis][ko04136:Autophagy - other][ko04138:Autophagy - yeast][ko04140:Autophagy - animal][ko04151:PI3K-Akt signaling pathway][ko04152:AMPK signaling pathway][ko04261:Adrenergic signaling in cardiomyocytes][ko04350:TGF-beta signaling pathway][ko04390:Hippo signaling	Achn144111:Actinidia chinensis|bHLH|bHLH family protein	XP_021858142.1:serine/threonineprotein phosphatase PP2A catalytic subunit [Spinacia oleracea] >KNA24326.1 hypothetical protein SOVF_016820 [Spinacia oleracea]	gi|521577617|gb|GAIA01070295.1|:TSA: Salicornia europaea Unigene84601_Se200S transcribed RNA sequence	A0A0K9RXS4|A0A0K9RXS4_SPIOL:Serine/threonine-protein phosphatase;OS=Spinacia oleracea;|GO:0004721,

geneid	trend	GO	Pathway	TF	NR	NT	Uniprot
DN115382_c0_g2_i3	UP	-	-	-	XP_010674352.1:PREDICTED: uncharacterized protein LOC104890530 [Beta vulgaris subsp. vulgaris] >KMT13990.1 hypothetical protein BVRB_4g078210 [Beta vulgaris subsp. vulgaris]	-	A0A0J8CJX8|A0A0J8CJX8_BETVU:Uncharacterized protein;OS=Beta vulgaris subsp. vulgaris.;
DN105490_c0_g1_i1	UP	-	-	-	-	-	-
DN120690_c4_g2_i3	UP	-	-	-	-	-	-
DN117408_c8_g1_i18	UP	[GO:0003755:peptidyl-prolyl cis-trans isomerase activity][GO:0006457:protein folding]	-	-	KNA03368.1:hypothetical protein SOVF_209880 [Spinacia oleracea]	-	A0A0K9Q9J9|A0A0K9Q9J9_SPIOL:Uncharacterized protein;OS=Spinacia oleracea;|GO:0003755,|GO:0006457,

geneid	trend	GO	Pathway	TF	NR	NT	Uniprot
DN121814_c3_g1_i17	UP	[GO:0003839:gamma-glutamylcyclotransferase activity][GO:0006751:glutathione catabolic process]	-	-	XP_010999365.1:PREDICTED: cation transport regulatorlike protein 2 [Populus euphratica]	-	A0A0K9RY99|A0A0K9RY99_SPIOL:Gamma-glutamylcyclotransferase;OS=Spinacia oleracea;|GO:0003839,|GO:0006751,
DN74344_c0_g1_i1	UP	[GO:0003676:nucleic acid binding][GO:0008270:zinc ion binding]	-	-	KNA19102.1:hypothetical protein SOVF_064640 [Spinacia oleracea]	-	A0A0K9RJT4|A0A0K9RJT4_SPIOL:Uncharacterized protein;OS=Spinacia oleracea;|GO:0003676,GO:0008270,
DN118358_c5_g1_i10	UP	-	-	-	-	-	-
DN123338_c1_g6_i3	UP	-	-	-	-	-	-
DN117274_c4_g3_i19	UP	[GO:0016021:integral component of membrane][GO:0055085:transmembrane transport]	-	Jcr4S00206.70:Jatropha curcas|FAR1|FAR1 family protein	XP_021727617.1:protein PINLIKES 1like [Chenopodium quinoa]	-	A0A0K9QZF3|A0A0K9QZF3_SPIOL:Uncharacterized protein;OS=Spinacia oleracea;|GO:0016021,|GO:0055085,

geneid	trend	GO	Pathway	TF	NR	NT	Uniprot
DN119650_c4_g1_i2	UP	-	[ko04016:MAPK signaling pathway - plant][ko04075:Plant hormone signal transduction]	PSME_00044429-RA:Pseudotsuga menziesii|MYB_related|MYB_related family protein	KNA04718.1:hypothetical protein SOVF_197070 [Spinacia oleracea]	-	A0A0K9QDG5|A0A0K9QDG5_SPIOL: Uncharacterized protein;OS=Spinacia oleracea;
DN105688_c0_g1_i2	UP	-	-	-	XP_021740690.1:receptorlike protein 12 [Chenopodium quinoa]	-	-
DN114005_c0_g1_i1	UP	[GO:0009654:photosystem II oxygen evolving complex][GO:0015979:photosynthesis]	[ko00195:Photosynthesis]	-	XP_021763320.1:photosystem II reaction center Psb28 proteinlike [Chenopodium quinoa]	gi|521675160|gb|GAIA01029719.1|:TSA: Salicornia europaea Unigene32594_Se200S transcribed RNA sequence	A0A0K9RZ56|A0A0K9RZ56_SPIOL:Photosystem II reaction center Psb28 protein;OS=Spinacia oleracea;|GO:0009654,|GO:0015979,
DN115574_c1_g2_i5	UP	-	-	-	XP_010679616.1:PREDICTED: UPF0553 protein isoform X1 [Beta vulgaris subsp. vulgaris] >KMT09826.1 hypothetical protein	gi|255568566|ref|XM_002525211.1|:Ricinus communis conserved hypothetical protein, mRNA	A0A0J8F2D3|A0A0J8F2D3_BETVU:Uncharacterized protein;OS=Beta vulgaris subsp. vulgaris.;

geneid	trend	GO	Pathway	TF	NR	NT	Uniprot
					BVRB_6g128020 [Beta vulgaris subsp. vulgaris]		
DN119207_c3_g2_i1	UP	[GO:0005777:peroxisome][GO:0003995:acyl-CoA dehydrogenase activity][GO:0003997:acyl-CoA oxidase activity][GO:0050660:flavin adenine dinucleotide binding][GO:0006635:fatty acid beta-oxidation]	[ko00071:Fatty acid degradation][ko00592:alpha-Linolenic acid metabolism][ko01040:Biosynthesis of unsaturated fatty acids][ko03320:PPAR signaling pathway][ko04024:cAMP signaling pathway][ko04146:Peroxisome]	-	XP_021722500.1:acylcoenzyme A oxidase 3, peroxisomallike isoform X1 [Chenopodium quinoa]	gi|521576490|gb|GAIA01070862.1|:TSA: Salicornia europaea Unigene85211_Se200S transcribed RNA sequence	A0A0J8EFZ4|A0A0J8EFZ4_BETVU:Acyl-coenzyme A oxidase;OS=Beta vulgaris subsp. vulgaris.;|GO:0005777,|GO:0003995,GO:0003997,GO:0050660,|GO:0006635,

geneid	trend	GO	Pathway	TF	NR	NT	Uniprot
DN117557_c0_g1_i17	UP	[GO:0005524:ATP binding][GO:0003676:nucleic acid binding][GO:0004672:protein kinase activity][GO:0006306:DNA methylation]	[ko03040:Spliceosome][ko05202:Transcriptional misregulation in cancer][ko05205:Proteoglycans in cancer]	-	XP_010108792.1:Shaggyrelated protein kinase epsilon [Morus notabilis] >EXC20307.1 Shaggyrelated protein kinase epsilon [Morus notabilis]	gi|147811543|emb|AM450058.2|:Vitis vinifera contig VV78X108547.35, whole genome shotgun sequence	W9SBA1|W9SBA1_9ROSA:Shaggy-related protein kinase epsilon;OS=Morus notabilis.;|GO:0005524,GO:0003676,GO:0004672,|GO:0006306,
DN113207_c0_g1_i4	UP	[GO:0016021:integral component of membrane][GO:0055114:oxidation-reduction process]	-	PDK_30s976561g001:Phoenix dactylifera|bZIP|bZIP family protein	XP_010683561.1:PREDICTED: probable transmembrane ascorbate ferrireductase 3 [Beta vulgaris subsp. vulgaris] >KMT06600.1 hypothetical protein BVRB_7g157680 [Beta vulgaris subsp. vulgaris]	-	A0A0J8BYL6|A0A0J8BYL6_BETVU:Uncharacterized protein;OS=Beta vulgaris subsp. vulgaris.;|GO:0016021,|GO:0055114,
DN127464_c5_g5_i3	UP	-	-	-	-	-	-

geneid	trend	GO	Pathway	TF	NR	NT	Uniprot
DN128338_c2_g2_i5	UP	-	-	-	-	-	-
DN124450_c1_g4_i1	UP	[GO:0008270:zinc ion binding][GO:0006355:regulation of transcription, DNA-templated]	-	-	XP_010670017.1:PREDICTED: protein strawberry notch homolog 1 [Beta vulgaris subsp. vulgaris] >KMT17383.1 hypothetical protein BVRB_2g038380 [Beta vulgaris subsp. vulgaris]	gi|334184014|ref|NM_106583.5|:Arabidopsis thaliana RING/FYVE/PHD zinc finger domain-containing protein mRNA, complete cds	A0A0J8CZQ9|A0A0J8CZQ9_BETVU:Uncharacterized protein;OS=Beta vulgaris subsp. vulgaris.;|GO:0008270,|GO:0006355,
DN117668_c0_g4_i2	UP	[GO:0008810:cellulase activity][GO:0030245:cellulose catabolic process]	[ko00500:Starch and sucrose metabolism]	mrna31843.1-v1.0-hybrid:Fragaria vesca|WRKY|WRKY family protein	XP_010677073.1:PREDICTED: endoglucanase 9 [Beta vulgaris subsp. vulgaris]	-	A0A068UTD0|A0A068UTD0_COFCA:Endoglucanase;OS=Coffea canephora;|GO:0008810,|GO:0030245,
DN119271_c0_g1_i4	UP	[GO:0005524:ATP binding][GO:0004672:protein kinase activity]	-	-	XP_021733680.1:probable serine/threonineprotein kinase DDB_G0280111	gi|521689601|gb|GAIA01022800.1|:TSA: Salicornia europaea Unigene24368_Se20 0S transcribed RNA	A0A0K9R370|A0A0K9R370_SPIOL:Uncharacterized protein;OS=Spinacia oleracea;|GO:00055

geneid	trend	GO	Pathway	TF	NR	NT	Uniprot
					[Chenopodium quinoa]	sequence	24,GO:0004672,
DN122384_c 2_g2_i4	UP	[GO:0016021:integral component of membrane][GO:0005524:ATP binding][GO:0004674:protein serine/threonine kinase activity]	[ko04064:NF-kappa B signaling pathway][ko04620:T oll-like receptor signaling pathway][ko04621: NOD-like receptor signaling pathway][ko04624:T oll and Imd signaling pathway][ko04722: Neurotrophin signaling pathway][ko05133:P ertussis][ko05140:L eishmaniasis][ko051 42:Chagas disease (American trypanosomiasis)][k	FANhyb_rscf00000 217.1.g00006.1:Frag aria x ananassa|WRKY|W RKY family protein	XP_021732936.1:pr obable serine/threonineprot ein kinase PBL7 isoform X2 [Chenopodium quinoa]	gi|521539453|gb|GA HZ01003552.1|:TSA : Salicornia europaea Unigene3824_SeCK S transcribed RNA sequence	A0A0J8BLC2|A0A0 J8BLC2_BETVU:U ncharacterized protein;OS=Beta vulgaris subsp. vulgaris.;|GO:00160 21,|GO:0005524,GO :0004674,

geneid	trend	GO	Pathway	TF	NR	NT	Uniprot
			o05145:Toxoplasmosis][ko05152:Tuberculosis][ko05162:Measles][ko05164:Influenza A]				
DN108264_c0_g1_i6	UP	-	-	-	XP_021774712.1:ultravioletB receptor UVR8like [Chenopodium quinoa]	gi|521692390|gb|GAIA01021386.1|:TSA: Salicornia europaea Unigene22485_Se200S transcribed RNA sequence	A0A0J8BCL1|A0A0J8BCL1_BETVU:Uncharacterized protein;OS=Beta vulgaris subsp. vulgaris.;
DN123124_c3_g3_i1	UP	-	-	-	-	-	-

geneid	trend	GO	Pathway	TF	NR	NT	Uniprot
DN125891_c0_g2_i13	UP	[GO:0032039:integrator complex][GO:0016180:snRNA processing]	-	-	KNA11326.1:hypothetical protein SOVF_136250 isoform A [Spinacia oleracea]	-	A0A0K9QXS9|A0A0K9QXS9_SPIOL: Uncharacterized protein;OS=Spinacia oleracea;|GO:0032039,|GO:0016180,
DN118524_c3_g11_i2	UP	[GO:0009706:chloroplast inner membrane][GO:0016021:integral component of membrane][GO:0015078:proton transmembrane transporter activity]	-	-	YP_009221819.1:chloroplast envelope membrane protein (chloroplast) [Mesembryanthemum crystallinum] >AIS35695.1 chloroplast envelope membrane protein (chloroplast) [Mesembryanthemum crystallinum]	gi|148607972|gb|EF534108.1|:Beta vulgaris chloroplast sequence	A0A0G2R7U9|A0A0G2R7U9_MESCR: envelope membrane protein, chloroplastic;OS=Mesembryanthemum crystallinum;|GO:0009706,GO:0016021,|GO:0015078,
DN112361_c0_g1_i1	UP	[GO:0043565:sequence-specific DNA binding][GO:0003700:DNA-binding transcription factor activity][GO:0008270:zinc ion binding]	-	Bv_007620_enci.t1: Beta vulgaris|GATA|GATA family protein	XP_021749841.1:putative GATA transcription factor 22 [Chenopodium quinoa]	-	A0A0J8B3F7|A0A0J8B3F7_BETVU:Uncharacterized protein;OS=Beta vulgaris subsp. vulgaris.;|GO:0043565,GO:0003700,GO:

geneid	trend	GO	Pathway	TF	NR	NT	Uniprot
							0008270,
DN128582_c0_g4_i8	UP	[GO:0016779:nucleotidyltransferase activity][GO:0003723:RNA binding][GO:0006396:RNA processing]	-	-	XP_021864119.1:uncharacterized protein LOC110802966 [Spinacia oleracea] >KNA18357.1 hypothetical protein SOVF_071460 [Spinacia oleracea]	-	A0A0K9RHN5|A0A0K9RHN5_SPIOL: Uncharacterized protein;OS=Spinacia oleracea;|GO:0016779,GO:0003723,|GO:0006396,
DN98874_c0_g1_i1	UP	-	-	-	-	-	-
DN111711_c1_g1_i1	UP	-	-	-	XP_021769373.1:gibberellinregulated protein 1like [Chenopodium quinoa]	gi|521738633|gb|GAIA01007484.1|:TSA: Salicornia europaea Unigene7799_Se200S transcribed RNA sequence	A0A0J8BJK8|A0A0J8BJK8_BETVU:Uncharacterized protein;OS=Beta vulgaris subsp. vulgaris.;

geneid	trend	GO	Pathway	TF	NR	NT	Uniprot
DN119211_c0_g1_i3	UP	-	-	-	XP_010695281.1:PREDICTED: uncharacterized protein LOC104907951 [Beta vulgaris subsp. vulgaris] >KMS97765.1 hypothetical protein BVRB_5g123980 [Beta vulgaris subsp. vulgaris]	-	A0A0J8E3R6|A0A0J8E3R6_BETVU:Uncharacterized protein;OS=Beta vulgaris subsp. vulgaris.;
DN126696_c1_g1_i2	UP	-	-	-	XP_021748223.1:BAG family molecular chaperone regulator 6like [Chenopodium quinoa]	-	A0A0K9QPD3|A0A0K9QPD3_SPIOL: Uncharacterized protein;OS=Spinacia oleracea;
DN121223_c6_g14_i2	UP	-	-	-	-	-	-

geneid	trend	GO	Pathway	TF	NR	NT	Uniprot
DN127772_c2_g6_i7	UP	[GO:0016021:integral component of membrane][GO:0005886:plasma membrane][GO:0016760:cellulose synthase (UDP-forming) activity][GO:0046872:metal ion binding][GO:0071555:cell wall organization][GO:0030244:cellulose biosynthetic process]	-	FANhyb_rscf00001203.1.g00002.1:Fragaria x ananassa|LBD|LBD family protein	XP_019158892.1:PREDICTED: cellulose synthase A catalytic subunit 3 [UDPforming] [Ipomoea nil] >XP_019158893.1 PREDICTED: cellulose synthase A catalytic subunit 3 [UDPforming] [Ipomoea nil]	gi|225450118|ref|XM_002278961.1|:PREDICTED: Vitis vinifera cellulose synthase A catalytic subunit 3 [UDP-forming]-like (LOC100256811), mRNA	A0A061DWE0|A0A061DWE0_THECC: Cellulose synthase;OS=Theobroma cacao;|GO:0016021, GO:0005886,|GO:0016760,GO:0046872,|GO:0071555,GO:0030244,
DN127908_c0_g2_i11	UP	[GO:0009941:chloroplast envelope][GO:0009533:chloroplast stromal thylakoid][GO:0009535:chloroplast thylakoid membrane][GO:0004252:serine-type endopeptidase activity][GO:0009658:chloroplast organization][GO:0010206:photosystem II repair][GO:0030163:protein catabolic process]	-	-	XP_010684757.1:PREDICTED: protease Dolike 2, chloroplastic isoform X1 [Beta vulgaris subsp. vulgaris] >KMT05819.1 hypothetical protein BVRB_7g165930 [Beta vulgaris subsp. vulgaris]	gi|359479762|ref|XM_002270211.2|:PREDICTED: Vitis vinifera protease Do-like 2, chloroplastic-like (LOC100251195), mRNA	A0A0J8C0I0|A0A0J8C0I0_BETVU:Uncharacterized protein;OS=Beta vulgaris subsp. vulgaris.;|GO:0009941,GO:0009533,GO:0009535,|GO:0004252,|GO:0009658,GO:0010206,GO:0030163,

geneid	trend	GO	Pathway	TF	NR	NT	Uniprot
DN118954_c0_g4_i8	UP	[GO:0009507:chloroplast][GO:0004851:uroporphyrin-III C-methyltransferase activity][GO:0055114:oxidation-reduction process][GO:0019354:siroheme biosynthetic process]	-	-	XP_010667452.1:PREDICTED: uncharacterized protein LOC104884494 [Beta vulgaris subsp. vulgaris] >KMS95363.1 hypothetical protein BVRB_008870 [Beta vulgaris subsp. vulgaris]	gi|521742619|gb|GAIA01004858.1|:TSA: Salicornia europaea Unigene5144_Se200S transcribed RNA sequence	A0A0J8B653|A0A0J8B653_BETVU:Uncharacterized protein;OS=Beta vulgaris subsp. vulgaris.;|GO:0009507,|GO:0004851,|GO:0055114,GO:0019354,
DN122778_c2_g3_i7	UP	-	-	-	GAU32968.1:hypothetical protein TSUD_358350 [Trifolium subterraneum]	-	A0A0K9QAR1|A0A0K9QAR1_SPIOL: Uncharacterized protein;OS=Spinacia oleracea;

geneid	trend	GO	Pathway	TF	NR	NT	Uniprot
DN127531_c1_g2_i13	UP	-	-	-	XP_010683787.1:PREDICTED: BTB/POZ domaincontaining protein FBL11 isoform X3 [Beta vulgaris subsp. vulgaris] >KMT06841.1 hypothetical protein BVRB_7g159950 isoform B [Beta vulgaris subsp. vulgaris]	-	A0A0J8EUH4|A0A0J8EUH4_BETVU: Uncharacterized protein;OS=Beta vulgaris subsp. vulgaris.;
DN123274_c1_g2_i8	UP	-	-	-	-	-	-
DN124496_c2_g8_i5	UP	-	-	DCAR_025729:Daucus carota|B3|B3 family protein	XP_010692733.1:PREDICTED: protein PLANT CADMIUM RESISTANCE 2like [Beta vulgaris subsp. vulgaris]	gi|521525561|gb|GAHZ01010883.1|:TSA: Salicornia europaea Unigene11498_SeCKS transcribed RNA sequence	A0A0K9RJW7|A0A0K9RJW7_SPIOL: Uncharacterized protein;OS=Spinacia oleracea;

geneid	trend	GO	Pathway	TF	NR	NT	Uniprot
DN123696_c5_g3_i5	UP	[GO:0000932:P-body][GO:0010494:cytoplasmic stress granule][GO:0005524:ATP binding][GO:0004004:ATP-dependent RNA helicase activity][GO:0003676:nucleic acid binding][GO:0033962:cytoplasmic mRNA processing body assembly][GO:0006417:regulation of translation][GO:0010501:RNA secondary structure unwinding]	[ko03018:RNA degradation]	Manes.01G259600.1.p:Manihot esculenta|MYB|MYB family protein	OVA14083.1:Helicase [Macleaya cordata]	gi|359474224|ref|XM_002271321.2|:PREDICTED: Vitis vinifera DEAD-box ATP-dependent RNA helicase 8-like (LOC100241111), mRNA	F6HUE2|F6HUE2_VITVI:Putative uncharacterized protein;OS=Vitis vinifera;GN=VIT_02s0025g03790 {ECO:0000313|EMBL:CCB58365.1};|GO:0000932,GO:0010494,|GO:0005524,GO:0004004,GO:0003676,|GO:0033962,GO:0006417,GO:0010501,
DN107458_c0_g3_i1	UP	-	-	-	XP_021621596.1:uncharacterized protein LOC110621636 [Manihot esculenta] >OAY43480.1 hypothetical protein MANES_08G073900 [Manihot esculenta]	-	B9S1D0|B9S1D0_RICCO:Inter-alpha-trypsin inhibitor heavy chain H3, putative;OS=Ricinus communis;

geneid	trend	GO	Pathway	TF	NR	NT	Uniprot
DN118078_c2_g1_i5	UP	-	-	-	XP_021767403.1:transcription factor GTE1like isoform X2 [Chenopodium quinoa]	-	A0A072U971|A0A072U971_MEDTR:Global transcription factor group protein;OS=Medicago truncatula;GN=MTR_6g033375 {ECO:0000313|EMBL:KEH25663.1};
DN122005_c0_g1_i2	UP	-	-	-	XP_010682948.1:PREDICTED: Fbox/kelchrepeat protein At3g23880like [Beta vulgaris subsp. vulgaris]	-	-

geneid	trend	GO	Pathway	TF	NR	NT	Uniprot
DN114643_c1_g1_i6	UP	[GO:0005634:nucleus][GO:0000977:RNA polymerase II regulatory region sequence-specific DNA binding][GO:0003700:DNA-binding transcription factor activity][GO:0000165:MAPK cascade][GO:0045944:positive regulation of transcription by RNA polymerase II][GO:0006351:transcription, DNA-templated]	-	Dca23368.1:Dianthus caryophyllus|MIKC_MADS|MIKC_MADS family protein	XP_021735939.1:MADSbox protein SOC1like [Chenopodium quinoa]	-	A0A0K9RW27|A0A0K9RW27_SPIOL: Uncharacterized protein;OS=Spinacia oleracea;|GO:0005634,|GO:0000977,GO:0003700,|GO:0000165,GO:0045944,GO:0006351,
DN125971_c6_g6_i3	UP	-	-	-	-	gi|502119898|ref|XM_004496716.1|:PREDICTED: Cicer arietinum polyubiquitin-like (LOC101505770), mRNA	-

geneid	trend	GO	Pathway	TF	NR	NT	Uniprot
DN128612_c0_g2_i3	UP	-	-	-	XP_010692549.1:PREDICTED: BTB/POZ domaincontaining protein At2g30600 [Beta vulgaris subsp. vulgaris] >XP_010692550.1 PREDICTED: BTB/POZ domaincontaining protein At2g30600 [Beta vulgaris subsp. vulgaris] >XP_019107760.1 PREDICTED: BTB/POZ domaincontaining protein At2g30600 [Beta vulgaris subsp. vulgaris] >KMS99745.1 hypothetical protein BVRB_1g021120 isoform A [Beta	gi|521491267|gb|GAHZ01039619.1|:TSA: Salicornia europaea Unigene44893_SeCKS transcribed RNA sequence	A0A0J8E9H1|A0A0J8E9H1_BETVU:Uncharacterized protein;OS=Beta vulgaris subsp. vulgaris.;

geneid	trend	GO	Pathway	TF	NR	NT	Uniprot
					vulgaris subsp. vulgaris]		

geneid	trend	GO	Pathway	TF	NR	NT	Uniprot
DN124655_c0_g2_i2	UP	[GO:0010285:L,L-diaminopimelate aminotransferase activity][GO:0030170:pyridoxal phosphate binding][GO:0009089:lysine biosynthetic process via diaminopimelate]	[ko00300:Lysine biosynthesis]	-	XP_021848454.1:LLdiaminopimelate aminotransferase, chloroplasticlike [Spinacia oleracea]	gi|359475514|ref|XM_002269117.2|:PREDICTED: Vitis vinifera LL-diaminopimelate aminotransferase, chloroplastic-like (LOC100242846), mRNA	A0A0K9RJC1|A0A0K9RJC1_SPIOL:Uncharacterized protein;OS=Spinacia oleracea;|GO:0010285,GO:0030170,|GO:0009089,
DN108838_c0_g1_i1	UP	-	-	-	-	-	-
DN118012_c0_g3_i5	UP	[GO:0003725:double-stranded RNA binding]	-	-	XP_010680139.1:PREDICTED: uncharacterized protein LOC104895357 [Beta vulgaris subsp. vulgaris] >KMT09098.1 hypothetical protein BVRB_6g132060 [Beta vulgaris subsp. vulgaris]	-	A0A0J8CAS3|A0A0J8CAS3_BETVU:Uncharacterized protein;OS=Beta vulgaris subsp. vulgaris.;|GO:0003725,
DN128338_c	UP	-	-	-	-	-	-

geneid	trend	GO	Pathway	TF	NR	NT	Uniprot
2_g2_i3							
DN128086_c 2_g1_i7	UP	[GO:0032502:developmental process]	-	-	OMP06881.1:Transposase, Tc1like protein [Corchorus olitorius]	-	K7LU34|K7LU34_SOYBN:Uncharacterized protein;OS=Glycine max;|GO:0032502,
DN122566_c 3_g1_i2	UP	[GO:0005524:ATP binding][GO:0004674:protein serine/threonine kinase activity]	[ko04064:NF-kappa B signaling pathway][ko04620:Toll-like receptor signaling pathway][ko04621:NOD-like receptor signaling pathway][ko04624:Toll and Imd signaling pathway][ko04722:Neurotrophin signaling pathway][ko05133:Pertussis][ko05140:Leishmaniasis][ko05142:Chagas disease	EMT28879:Aegilops tauschii|ERF|ERF family protein	XP_010675411.1:PREDICTED: putative serine/threonineprotein kinase isoform X1 [Beta vulgaris subsp. vulgaris]	gi|502089246|ref|XM_004488795.1|:PREDICTED: Cicer arietinum probable leucine-rich repeat receptor-like serine/threonine-protein kinase At3g14840-like (LOC101506238), transcript variant X3, mRNA	A0A0D2QZZ2|A0A0D2QZZ2_GOSRA:Uncharacterized protein;OS=Gossypium raimondii;|GO:0005524,GO:0004674,

geneid	trend	GO	Pathway	TF	NR	NT	Uniprot
			(American trypanosomiasis)][ko05145:Toxoplasmosis][ko05152:Tuberculosis][ko05162:Measles][ko05164:Influenza A]				
DN116240_c0_g3_i1	UP	[GO:0016788:hydrolase activity, acting on ester bonds]	-	-	XP_021736818.1:GDSL esterase/lipase 7like [Chenopodium quinoa]	-	A0A0J8D321|A0A0J8D321_BETVU:Uncharacterized protein;OS=Beta vulgaris subsp. vulgaris.;|GO:0016788,

geneid	trend	GO	Pathway	TF	NR	NT	Uniprot
DN121373_c0_g1_i2	UP	[GO:0009507:chloroplast][GO:0008967:phosphoglycolate phosphatase activity]	[ko00630:Glyoxylate and dicarboxylate metabolism]	PK12308.1:Cannabis sativa|MYB_related|MYB_related family protein	XP_021716855.1:phosphoglycolate phosphatase 2like [Chenopodium quinoa]	gi|359495615|ref|XM_003634990.1|:PREDICTED: Vitis vinifera 4-nitrophenylphosphatase-like, transcript variant 2 (LOC100243036), mRNA	A0A0J8BWG4|A0A0J8BWG4_BETVU: Phosphoglycolate phosphatase;OS=Beta vulgaris subsp. vulgaris.;|GO:0009507,|GO:0008967,
DN105535_c0_g1_i2	UP	-	-	-	XP_021857319.1:osmotinlike protein [Spinacia oleracea] >KNA11472.1 hypothetical protein SOVF_134970 [Spinacia oleracea]	-	A0A0K9QW13|A0A0K9QW13_SPIOL: Uncharacterized protein;OS=Spinacia oleracea;
DN124089_c1_g3_i7	UP	[GO:0016758:transferase activity, transferring hexosyl groups][GO:0008152:metabolic process]	-	PH01002606G0040: Phyllostachys heterocycla|HSF|HSF family protein	XP_021719267.1:UDPglycosyltransferase 79B6like [Chenopodium quinoa]	gi|343457674|gb|JF346162.1|:Brassica rapa subsp. campestris UDP-glucose:flavonoid 3-O-glucosyltransferase 1 (UF3GT1) mRNA, complete	A0A0K9RSC5|A0A0K9RSC5_SPIOL:Uncharacterized protein;OS=Spinacia oleracea;|GO:0016758,|GO:0008152,

geneid	trend	GO	Pathway	TF	NR	NT	Uniprot
						cds	
DN118039_c3_g2_i1	UP	-	-	-	-	-	-
DN120346_c0_g1_i7	UP	[GO:0005509:calcium ion binding]	[ko04010:MAPK signaling pathway][ko04020:Calcium signaling pathway][ko04022:cGMP-PKG signaling pathway][ko04114:Oocyte meiosis][ko04218:Cellular senescence][ko04310:Wnt signaling pathway][ko04360:Axon guidance][ko04370:VEGF signaling	-	XP_011095878.1:calcineurin Blike protein 3 isoform X2 [Sesamum indicum]	gi|84873756|gb|DQ337188.1|:Populus euphratica calcineurin B-like protein (CBL4) mRNA, complete cds	A0A061GHY2|A0A061GHY2_THECC:Calcineurin B-like protein 2 isoform 1;OS=Theobroma cacao;|GO:0005509,

geneid	trend	GO	Pathway	TF	NR	NT	Uniprot
			pathway][ko04380:Osteoclast differentiation][ko04650:Natural killer cell mediated cytotoxicity][ko04658:Th1 and Th2 cell differentiation][ko04659:Th17 cell differentiation][ko04660:T cell receptor signaling pathway][ko04662:B cell receptor signaling pathway][ko04720:Long-term potentiation][ko04724:Glutamatergic synapse][ko04921:Oxytocin signaling pathway][ko04922:Glucagon signaling pathway][ko04924:Renin				

geneid	trend	GO	Pathway	TF	NR	NT	Uniprot
			secretion][ko05010:Alzheimer's disease][ko05014:Amyotrophic lateral sclerosis (ALS)][ko05031:Amphetamine addiction][ko05152:Tuberculosis][ko05166:HTLV-I infection][ko05167:Kaposi's sarcoma-associated herpesvirus infection]				
DN100549_c0_g1_i1	UP	-	-	-	-	-	-
DN123506_c1_g3_i1	UP	-	-	-	-	-	-
DN106639_c0_g2_i1	UP	[GO:0009733:response to auxin]	[ko04075:Plant hormone signal transduction]	-	XP_010696118.1:PREDICTED: uncharacterized protein LOC104908675	-	A0A0J8BB19|A0A0J8BB19_BETVU:Uncharacterized protein;OS=Beta vulgaris subsp.

geneid	trend	GO	Pathway	TF	NR	NT	Uniprot
					[Beta vulgaris subsp. vulgaris] >KMS97198.1 hypothetical protein BVRB_7g177800 [Beta vulgaris subsp. vulgaris]		vulgaris.;|GO:0009733,
DN118486_c0_g1_i1	UP	-	-	-	-	-	-
DN156182_c0_g1_i1	UP	[GO:0003824:catalytic activity][GO:0050662:coenzyme binding]	[ko00941:Flavonoid biosynthesis]	676768160:Sisymbrium irio|GeBP|GeBP family protein	BAD67186.1:dihydroflavonol 4reductase [Phytolacca americana]	gi|54888725|dbj|AB128768.1|:Phytolacca americana dfr mRNA for dihydroflavonol 4-reductase, complete cds	Q5W9T9|Q5W9T9_PHYAM:Dihydroflavonol 4-reductase;OS=Phytolacca americana;|GO:0003824,GO:0050662,
DN97652_c0_g2_i1	UP	-	-	LOC_Os11g12360.1:Oryza sativa subsp. japonica|M-type_MADS|M-type_MADS family protein	BAT90125.1:hypothetical protein VIGAN_06130700 [Vigna angularis var. angularis]	-	A0A0S3SBF2|A0A0S3SBF2_PHAAN:Uncharacterized protein;OS=Vigna angularis var. angularis.;
DN126870_c4_g4_i1	UP	-	-	-	-	-	-

geneid	trend	GO	Pathway	TF	NR	NT	Uniprot
DN125455_c3_g2_i8	UP	[GO:0005739:mitochondrion][GO:0050897:cobalt ion binding][GO:0016874:ligase activity][GO:0008270:zinc ion binding][GO:0006552:leucine catabolic process]	[ko00280:Valine, leucine and isoleucine degradation]	CCG019154.1:Populus euphratica|bZIP|bZIP family protein	XP_021854838.1:methylcrotonoylCoA carboxylase beta chain, mitochondrial [Spinacia oleracea]	gi|359475366|ref|XM_002282231.2|:PREDICTED: Vitis vinifera methylcrotonoyl-CoA carboxylase beta chain, mitochondrial-like (LOC100253805), mRNA	A0A0J8BGX7|A0A0J8BGX7_BETVU: Uncharacterized protein;OS=Beta vulgaris subsp. vulgaris.;|GO:0005739,|GO:0050897,GO:0016874,GO:0008270,|GO:0006552,
DN112995_c0_g1_i3	UP	-	-	KN540176.1_FGP005:Oryza longistaminata|NAC|NAC family protein	XP_021841787.1:chaperone protein dnaJ 10 [Spinacia oleracea] >KNA17874.1 hypothetical protein SOVF_075920 [Spinacia oleracea]	-	A0A0K9RFX2|A0A0K9RFX2_SPIOL:Uncharacterized protein;OS=Spinacia oleracea;

geneid	trend	GO	Pathway	TF	NR	NT	Uniprot
DN126446_c0_g3_i4	UP	[GO:0005829:cytosol]	-	-	XP_010686761.1:PREDICTED: HSPinteracting protein [Beta vulgaris subsp. vulgaris] >KMT04074.1 hypothetical protein BVRB_8g185280 [Beta vulgaris subsp. vulgaris]	-	A0A0J8BVR4|A0A0J8BVR4_BETVU: Uncharacterized protein;OS=Beta vulgaris subsp. vulgaris.;|GO:0005829,
DN119096_c0_g1_i4	UP	-	-	-	-	-	-
DN127531_c1_g2_i2	UP	-	-	-	XP_010683787.1:PREDICTED: BTB/POZ domaincontaining protein FBL11 isoform X3 [Beta vulgaris subsp. vulgaris] >KMT06841.1 hypothetical protein BVRB_7g159950 isoform B [Beta	-	A0A0J8EUH4|A0A0J8EUH4_BETVU: Uncharacterized protein;OS=Beta vulgaris subsp. vulgaris.;

geneid	trend	GO	Pathway	TF	NR	NT	Uniprot
					vulgaris subsp. vulgaris]		
DN121605_c0_g3_i2	UP	[GO:0003676:nucleic acid binding][GO:0015074:DNA integration]	-	Rsa1.0_01791.1_g00001.1:Raphanus sativus|GRAS|GRAS family protein	GAU34891.1:hypothetical protein TSUD_144220 [Trifolium subterraneum]	-	A0A151RKD3|A0A151RKD3_CAJCA: Retrovirus-related Pol polyprotein from transposon TNT 1-94;OS=Cajanus cajan;|GO:0003676,|GO:0015074,
DN120629_c2_g1_i3	UP	-	-	-	-	-	-

geneid	trend	GO	Pathway	TF	NR	NT	Uniprot
DN120765_c2_g2_i2	UP	[GO:0005634:nucleus][GO:0003677:DNA binding][GO:0003700:DNA-binding transcription factor activity][GO:0007275:multicellular organism development][GO:0006351:transcription, DNA-templated]	-	cra_locus_16828_iso_9:Catharanthus roseus|AP2|AP2 family protein	EYU39564.1:hypothetical protein MIMGU_mgv1a021246mg [Erythranthe guttata]	gi|224068643|ref|XM_002326128.1|:Populus trichocarpa AP2 domain-containing transcription factor (RAP6), mRNA	A0A022RHV3|A0A022RHV3_ERYGU: Uncharacterized protein;OS=Erythranthe guttata;|GO:0005634,|GO:0003677,GO:0003700,|GO:0007275,GO:0006351,
DN120257_c3_g5_i1	UP	-	-	-	-	-	-
DN109982_c0_g1_i6	UP	[GO:0005737:cytoplasm][GO:0003879:ATP phosphoribosyltransferase activity][GO:0000287:magnesium ion binding][GO:0000105:histidine biosynthetic process]	[ko00340:Histidine metabolism]	-	XP_021727328.1:ATP phosphoribosyltransferase 1, chloroplasticlike [Chenopodium quinoa]	gi|521697630|gb|GAIA01018766.1|:TSA: Salicornia europaea Unigene19724_Se200S transcribed RNA sequence	A0A0K9RQV9|A0A0K9RQV9_SPIOL: Uncharacterized protein;OS=Spinacia oleracea;|GO:0005737,|GO:0003879,GO:0000287,|GO:0000105,

geneid	trend	GO	Pathway	TF	NR	NT	Uniprot
DN127296_c1_g2_i5	UP	[GO:0005096:GTPase activator activity][GO:0007165:signal transduction]	-	-	KNA20911.1:hypothetical protein SOVF_047780 isoform A [Spinacia oleracea]	gi|460385692|ref|XM_004238488.1|:PREDICTED: Solanum lycopersicum uncharacterized LOC101245542 (LOC101245542), mRNA	A0A0K9RPY8|A0A0K9RPY8_SPIOL:Uncharacterized protein;OS=Spinacia oleracea;|GO:0005096,|GO:0007165,
DN105142_c0_g1_i1	UP	[GO:0046872:metal ion binding][GO:0003950:NAD+ ADP-ribosyltransferase activity]	-	Sp_163300_egro.t1: Spinacia oleracea|C2H2|C2H2 family protein	XP_021718660.1:uncharacterized protein LOC110686370 [Chenopodium quinoa]	gi|521697893|gb|GAIA01018508.1|:TSA: Salicornia europaea Unigene19463_Se200S transcribed RNA sequence	A0A0K9QM51|A0A0K9QM51_SPIOL: Uncharacterized protein;OS=Spinacia oleracea;|GO:0046872,GO:0003950,
DN115438_c0_g8_i4	UP	[GO:0046872:metal ion binding][GO:0030001:metal ion transport]	-	-	XP_021723836.1:uncharacterized protein LOC110691233 [Chenopodium quinoa]	-	A0A0K9Q7F0|A0A0K9Q7F0_SPIOL:Uncharacterized protein;OS=Spinacia oleracea;|GO:0046872,|GO:0030001,

geneid	trend	GO	Pathway	TF	NR	NT	Uniprot
DN124645_c2_g3_i4	UP	[GO:0005622:intracellular][GO:0006886:intracellular protein transport][GO:0016192:vesicle-mediated transport]	[ko04138:Autophagy - yeast]	-	XP_010678074.1:PREDICTED: vacuolar protein sortingassociated protein 18 homolog isoform X2 [Beta vulgaris subsp. vulgaris] >KMT11055.1 hypothetical protein BVRB_5g111440 isoform A [Beta vulgaris subsp. vulgaris]	gi|225447638|ref|XM_002271348.1|:PREDICTED: Vitis vinifera vacuolar protein sorting-associated protein 18 homolog-like (LOC100254609), mRNA	A0A0J8F584|A0A0J8F584_BETVU:Uncharacterized protein;OS=Beta vulgaris subsp. vulgaris.;|GO:0005622,|GO:0006886,GO:0016192,
DN127384_c2_g4_i1	UP	[GO:0016021:integral component of membrane]	-	Bv5_101120_supk.t1:Beta vulgaris|ERF|ERF family protein	XP_021770245.1:hexose carrier protein HEX6like [Chenopodium quinoa]	gi|521512606|gb|GAHZ01018314.1|:TSA: Salicornia europaea Unigene19256_SeCKS transcribed RNA sequence	A0A0J8C995|A0A0J8C995_BETVU:Uncharacterized protein;OS=Beta vulgaris subsp. vulgaris.;|GO:0016021,|GO:0022891,

geneid	trend	GO	Pathway	TF	NR	NT	Uniprot
DN127023_c1_g1_i11	UP	[GO:0005737:cytoplasm][GO:0003999:adenine phosphoribosyltransferase activity][GO:0006168:adenine salvage][GO:0009116:nucleoside metabolic process]	[ko00230:Purine metabolism]	MDP0000573178:Malus domestica|bHLH|bHLH family protein	XP_021769363.1:adenine phosphoribosyltransferase 5like [Chenopodium quinoa]	gi|521698930|gb|GAIA01017969.1|:TSA: Salicornia europaea Unigene18923_Se200S transcribed RNA sequence	A0A0K9QSX0|A0A0K9QSX0_SPIOL: Uncharacterized protein;OS=Spinacia oleracea;|GO:0005737,|GO:0003999,|GO:0006168,GO:0009116,
DN121365_c0_g3_i6	UP	[GO:0003677:DNA binding]	-	AHYPO_007595-RA:Amaranthus hypochondriacus|Trihelix|Trihelix family protein	GAV85429.1:Myb_DNAbind_4 domaincontaining protein [Cephalotus follicularis]	gi|224062887|ref|XM_002300884.1|:Populus trichocarpa predicted protein, mRNA	A0A0J8D164|A0A0J8D164_BETVU:Uncharacterized protein;OS=Beta vulgaris subsp. vulgaris.;|GO:0003677,
DN129023_c3_g4_i2	UP	[GO:0008234:cysteine-type peptidase activity]	-	LOC_Os11g12360.1:Oryza sativa subsp. japonica|M-type_MADS|M-type_MADS family protein	XP_014521995.1:PREDICTED: uncharacterized protein LOC106778540 [Vigna radiata var. radiata]	gi|197724756|emb|CU570969.16|:M.truncatula DNA sequence from clone MTH2-75D5 on chromosome 3, complete sequence	A5AM06|A5AM06_VITVI:Putative uncharacterized protein;OS=Vitis vinifera;|GO:0008234,

geneid	trend	GO	Pathway	TF	NR	NT	Uniprot
DN116058_c3_g3_i3	UP	[GO:0046872:metal ion binding][GO:0016702:oxidoreductase activity, acting on single donors with incorporation of molecular oxygen, incorporation of two atoms of oxygen][GO:0006633:fatty acid biosynthetic process][GO:0031408:oxylipin biosynthetic process]	[ko00591:Linoleic acid metabolism][ko00592:alpha-Linolenic acid metabolism]	Tp57577_TGAC_v2_mRNA8161:Trifolium pratense|MYB_related|MYB_related family protein	XP_010673574.1:PREDICTED: linoleate 13Slipoxygenase 21, chloroplastic [Beta vulgaris subsp. vulgaris] >KMT14429.1 hypothetical protein BVRB_4g072070 [Beta vulgaris subsp. vulgaris]	-	A0A0J8CQY6|A0A0J8CQY6_BETVU: Lipoxygenase;OS=Beta vulgaris subsp. vulgaris.;|GO:0046872,GO:0016702,|GO:0006633,GO:0031408,
DN122051_c0_g1_i6	UP	[GO:0016021:integral component of membrane]	-	Achn355811:Actinidia chinensis|WRKY|WRKY family protein	XP_010683099.1:PREDICTED: uncharacterized protein LOC104897842 [Beta vulgaris subsp. vulgaris]	gi|356577286|ref|XM_003556711.1|:PREDICTED: Glycine max uncharacterized protein LOC100789016 (LOC100789016), mRNA	A0A0J8EV03|A0A0J8EV03_BETVU:Uncharacterized protein;OS=Beta vulgaris subsp. vulgaris.;|GO:0016021,

geneid	trend	GO	Pathway	TF	NR	NT	Uniprot
DN117056_c0_g1_i4	UP	[GO:0004553:hydrolase activity, hydrolyzing O-glycosyl compounds][GO:0005975:carbohydrate metabolic process]	[ko00051:Fructose and mannose metabolism]	Zmw_sc05871.1.g00020.1:Zoysia matrella|bZIP|bZIP family protein	XP_021865831.1:mannan endo1,4betamannosidase 7 [Spinacia oleracea]	-	A0A0J8F1I2|A0A0J8F1I2_BETVU:Uncharacterized protein;OS=Beta vulgaris subsp. vulgaris.;|GO:0004553,|GO:0005975,
DN127215_c1_g4_i16	UP	[GO:0016021:integral component of membrane][GO:0008324:cation transmembrane transporter activity][GO:0006813:potassium ion transport]	-	Do016940.1:Dichanthelium oligosanthes|FAR1|FAR1 family protein	XP_010681888.1:PREDICTED: probable ion channel SYM8 isoform X1 [Beta vulgaris subsp. vulgaris] >KMT08062.1 hypothetical protein BVRB_6g144160 isoform A [Beta vulgaris subsp. vulgaris]	gi|255571019|ref|XM_002526415.1|:Ricinus communis conserved hypothetical protein, mRNA	A0A0J8C3I0|A0A0J8C3I0_BETVU:Uncharacterized protein;OS=Beta vulgaris subsp. vulgaris.;|GO:0016021,|GO:0008324,|GO:0006813,
DN118404_c3_g8_i4	UP	-	-	-	KNA23776.1:hypothetical protein SOVF_022060 isoform A, partial [Spinacia oleracea]	gi|521559327|gb|GAIA01077522.1|:TSA: Salicornia europaea Unigene94321_Se20 0S transcribed RNA sequence	A0A0K9RW55|A0A0K9RW55_SPIOL: Uncharacterized protein;OS=Spinacia oleracea;

geneid	trend	GO	Pathway	TF	NR	NT	Uniprot
DN127734_c0_g5_i1	UP	-	-	-	-	-	-
DN119890_c0_g4_i4	UP	[GO:0016021:integral component of membrane][GO:0015079:potassium ion transmembrane transporter activity]	-	mrna09346.1-v1.0-hybrid:Fragaria vesca|NF-X1|NF-X1 family protein	ASM90213.1:K+ uptake permease 2 [Sesuvium portulacastrum]	gi|14091470|gb|AF367865.1|:Mesembryanthemum crystallinum potassium transporter HAK2p (HAK2) mRNA, complete cds	Q94KC6|Q94KC6_MESCR:Potassium transporter;OS=Mesembryanthemum crystallinum;|GO:0016021,|GO:0015079,
DN124849_c1_g6_i3	UP	-	-	-	-	-	-
DN125755_c1_g1_i1	UP	[GO:0004518:nuclease activity]	-	-	XP_021723440.1:uncharacterized protein LOC110690861 isoform X2 [Chenopodium quinoa]	gi|521536990|gb|GAHZ01005109.1|:TSA: Salicornia europaea Unigene5398_SeCKS transcribed RNA sequence	A0A0J8EJZ0|A0A0J8EJZ0_BETVU:Uncharacterized protein;OS=Beta vulgaris subsp. vulgaris.;|GO:0004518,
DN114450_c2_g9_i11	UP	-	-	-	-	-	-

geneid	trend	GO	Pathway	TF	NR	NT	Uniprot
DN121593_c0_g1_i25	UP	[GO:0016874:ligase activity]	-	-	XP_021619187.1:ultravioletB receptor UVR8like isoform X3 [Manihot esculenta] >OAY43577.1 hypothetical protein MANES_08G080400 [Manihot esculenta]	-	A0A151TZY2|A0A151TZY2_CAJCA:Putative E3 ubiquitin-protein ligase HERC2;OS=Cajanus cajan;|GO:0016874,
DN105666_c1_g1_i2	UP	[GO:0003993:acid phosphatase activity]	-	GSMUA_Achr8P25970_001:Musa acuminata|MYB|MYB family protein	XP_021860433.1:acid phosphatase 1like [Spinacia oleracea] >KNA20723.1 hypothetical protein SOVF_049830 [Spinacia oleracea]	-	A0A0K9RML3|A0A0K9RML3_SPIOL:Uncharacterized protein;OS=Spinacia oleracea;|GO:0003993,
DN122940_c3_g12_i2	UP	[GO:0003676:nucleic acid binding]	-	-	EEF46711.1:oligoribonuclease, mitochondrial, putative [Ricinus communis]	-	B9RPJ0|B9RPJ0_RICCO:Oligoribonuclease, mitochondrial, putative;OS=Ricinus communis;|GO:0003676,

geneid	trend	GO	Pathway	TF	NR	NT	Uniprot
DN118371_c0_g7_i2	UP	-	-	-	XP_021727969.1:Fbox/kelchrepeat protein At3g27150like [Chenopodium quinoa] >XP_021727970.1 Fbox/kelchrepeat protein At3g27150like [Chenopodium quinoa]	-	A0A0J8F0M5|A0A0J8F0M5_BETVU:Uncharacterized protein;OS=Beta vulgaris subsp. vulgaris.;
DN128599_c1_g2_i10	UP	[GO:0052866:phosphatidylinositol phosphate phosphatase activity]	[ko00562:Inositol phosphate metabolism][ko04070:Phosphatidylinositol signaling system]	-	XP_010676764.1:PREDICTED: phosphoinositide phosphatase SAC8 isoform X2 [Beta vulgaris subsp. vulgaris]	gi|359481160|ref|XM_003632536.1|:PREDICTED: Vitis vinifera phosphatidylinositide phosphatase SAC1-like, transcript variant 2 (LOC100267852), mRNA	A0A0J8CEV1|A0A0J8CEV1_BETVU:Uncharacterized protein;OS=Beta vulgaris subsp. vulgaris.;|GO:0052866,

geneid	trend	GO	Pathway	TF	NR	NT	Uniprot
DN127531_c1_g2_i3	UP	-	-	-	XP_010683787.1:PREDICTED: BTB/POZ domaincontaining protein FBL11 isoform X3 [Beta vulgaris subsp. vulgaris] >KMT06841.1 hypothetical protein BVRB_7g159950 isoform B [Beta vulgaris subsp. vulgaris]	-	A0A0J8EUH4|A0A0J8EUH4_BETVU: Uncharacterized protein;OS=Beta vulgaris subsp. vulgaris.;
DN118639_c1_g1_i1	UP	-	-	-	XP_021716270.1:LOW QUALITY PROTEIN: BURP domain protein RD22like [Chenopodium quinoa]	-	A0A0K9R0Z0|A0A0K9R0Z0_SPIOL:Uncharacterized protein;OS=Spinacia oleracea;
DN121263_c6_g2_i3	UP	[GO:0008080:N-acetyltransferase activity]	-	-	XP_021838620.1:uncharacterized protein LOC110778370 [Spinacia oleracea]	-	A0A0K9Q771|A0A0K9Q771_SPIOL:Uncharacterized protein;OS=Spinacia

geneid	trend	GO	Pathway	TF	NR	NT	Uniprot
							oleracea;|GO:0008080,
DN121420_c2_g1_i11	UP	[GO:0015030:Cajal body][GO:0003690:double-stranded DNA binding][GO:0003725:double-stranded RNA binding][GO:0008270:zinc ion binding][GO:0008380:RNA splicing][GO:0080188:RNA-directed DNA methylation][GO:0009845:seed germination]	-	-	XP_010693851.1:PREDICTED: zinc finger protein ZOP1 [Beta vulgaris subsp. vulgaris] >XP_010693852.1 PREDICTED: zinc finger protein ZOP1 [Beta vulgaris subsp. vulgaris] >KMS98811.1 hypothetical protein BVRB_3g068860 isoform A [Beta vulgaris subsp. vulgaris]	-	A0A0J8BD44|A0A0J8BD44_BETVU:Uncharacterized protein;OS=Beta vulgaris subsp. vulgaris.;|GO:0015030,|GO:0003690,GO:0003725,GO:0008270,|GO:0008380,GO:0080188,GO:0009845,
DN112165_c0_g1_i3	UP	-	-	Sp_026020_gcng.t1:Spinacia oleracea|bHLH|bHLH family protein	XP_021732497.1:transcription factor bHLH51like [Chenopodium quinoa]	-	A0A0K9RWK7|A0A0K9RWK7_SPIOL:Uncharacterized protein;OS=Spinacia oleracea;

geneid	trend	GO	Pathway	TF	NR	NT	Uniprot
DN113764_c0_g1_i1	UP	[GO:0046872:metal ion binding][GO:0016491:oxidoreductase activity]	[ko00904:Diterpenoid biosynthesis]	Zpz_sc00083.1.g00290.1.am.mk:Zoysia pacifica|Dof|Dof family protein	CAD19319.1:GA20 oxidase [Beta vulgaris]	gi|21322507|emb|AJ422049.1|:Beta vulgaris mRNA for GA20 oxidase (20ox gene)	Q8L6W4|Q8L6W4_BETVU:GA20 oxidase;OS=Beta vulgaris;|GO:0046872,GO:0016491,
DN126222_c1_g4_i18	UP	-	-	-	KNA04691.1:hypothetical protein SOVF_197320 isoform A, partial [Spinacia oleracea]	gi|521670653|gb|GAIA01032198.1|:TSA: Salicornia europaea Unigene35333_Se200S transcribed RNA sequence	A0A0K9QDU4|A0A0K9QDU4_SPIOL: Uncharacterized protein;OS=Spinacia oleracea;
DN114201_c1_g8_i4	UP	[GO:0016831:carboxy-lyase activity][GO:0030170:pyridoxal phosphate binding][GO:0006520:cellular amino acid metabolic process]	[ko00350:Tyrosine metabolism][ko00950:Isoquinoline alkaloid biosynthesis]	-	XP_021722165.1:tyrosine decarboxylase 1like [Chenopodium quinoa]	gi|521666069|gb|GAIA01034503.1|:TSA: Salicornia europaea Unigene38193_Se200S transcribed RNA sequence	A0A0K9RYU8|A0A0K9RYU8_SPIOL: Uncharacterized protein;OS=Spinacia oleracea;|GO:0016831,GO:0030170,|GO:0006520,
DN122254_c1_g1_i11	UP	[GO:0016021:integral component of membrane]	-	-	XP_021738044.1:uncharacterized protein LOC110704552 [Chenopodium quinoa] >XP_021738045.1 uncharacterized	gi|502113728|ref|XM_004494683.1|:PREDICTED: Cicer arietinum uncharacterized LOC101512623 (LOC101512623),	A0A0J8CIH1|A0A0J8CIH1_BETVU:Uncharacterized protein;OS=Beta vulgaris subsp. vulgaris.;|GO:0016021,

geneid	trend	GO	Pathway	TF	NR	NT	Uniprot
					protein LOC110704552 [Chenopodium quinoa]	transcript variant X4, mRNA	
DN128675_c2_g3_i1	UP	-	-	-	XP_010677875.1:PREDICTED: uncharacterized protein LOC104893474 [Beta vulgaris subsp. vulgaris]	-	A0A0J8DVH8|A0A0J8DVH8_BETVU: Uncharacterized protein;OS=Beta vulgaris subsp. vulgaris.;
DN120929_c1_g3_i5	UP	[GO:0009570:chloroplast stroma][GO:0030785:[ribulose-bisphosphate carboxylase]-lysine N-methyltransferase activity][GO:0016279:protein-lysine N-methyltransferase activity][GO:0018023:peptidyl-lysine trimethylation]	-	Zpz_sc00801.1.g00050.1.am.mk:Zoysia pacifica|NAC|NAC family protein	XP_021739961.1:fructosebisphosphate aldolaselysine Nmethyltransferase, chloroplasticlike [Chenopodium quinoa]	gi|3403235|gb|AF071544.1|AF071544:Spinacia oleracea ribulose-1,5-bisphosphate carboxylase/oxygenase small subunit N-methyltransferase I (rbcL) mRNA, chloroplast gene encoding chloroplast protein, complete	A0A0J8EV42|A0A0J8EV42_BETVU:Uncharacterized protein;OS=Beta vulgaris subsp. vulgaris.;|GO:0009570,|GO:0030785,GO:0016279,|GO:0018023,

geneid	trend	GO	Pathway	TF	NR	NT	Uniprot
						cds	
DN123874_c6_g1_i4	UP	[GO:0031305:integral component of mitochondrial inner membrane][GO:0002083:4-hydroxybenzoate decaprenyltransferase activity][GO:0047293:4-hydroxybenzoate nonaprenyltransferase activity][GO:0008299:isoprenoid biosynthetic process][GO:0006744:ubiquinone biosynthetic process]	[ko00130:Ubiquinone and other terpenoid-quinone biosynthesis]	-	XP_010684755.1:PREDICTED: 4hydroxybenzoate polyprenyltransferase, mitochondrial isoform X1 [Beta vulgaris subsp. vulgaris] >KMT05818.1 hypothetical protein BVRB_7g165920 [Beta vulgaris subsp. vulgaris]	gi|521738271|gb|GAIA01007665.1|:TSA: Salicornia europaea Unigene7984_Se200S transcribed RNA sequence	A0A0J8C0V6|A0A0J8C0V6_BETVU:4-hydroxybenzoate polyprenyltransferase, mitochondrial;OS=Beta vulgaris subsp. vulgaris.;|GO:0031305,|GO:0002083,GO:0047293,|GO:0008299,GO:0006744,

geneid	trend	GO	Pathway	TF	NR	NT	Uniprot
DN117659_c0_g1_i9	UP	-	-	-	XP_021846310.1:protein SMG9like [Spinacia oleracea] >KNA18498.1 hypothetical protein SOVF_070220 [Spinacia oleracea]	-	A0A0K9RG82|A0A0K9RG82_SPIOL:Uncharacterized protein;OS=Spinacia oleracea;
DN128200_c3_g2_i8	UP	[GO:0003856:3-dehydroquinate synthase activity][GO:0016491:oxidoreductase activity][GO:0009073:aromatic amino acid family biosynthetic process]	-	-	XP_010693330.1:PREDICTED: uncharacterized protein LOC104906292 isoform X1 [Beta vulgaris subsp. vulgaris]	gi|115451012|ref|NM_001055642.1|:Oryza sativa Japonica Group Os03g0170300 (Os03g0170300) mRNA, complete cds	A0A0J8BGQ6|A0A0J8BGQ6_BETVU:Uncharacterized protein;OS=Beta vulgaris subsp. vulgaris.;|GO:0003856,GO:0016491,|GO:0009073,
DN128140_c1_g2_i3	UP	-	-	-	OMP05307.1:Transposase, Tc1like protein [Corchorus olitorius]	-	A0A0A9EXU2|A0A0A9EXU2_ARUDO:Uncharacterized protein;OS=Arundo donax;

geneid	trend	GO	Pathway	TF	NR	NT	Uniprot
DN115753_c0_g1_i4	UP	[GO:0016021:integral component of membrane][GO:0019915:lipid storage]	-	Sopen07g030510.1: Solanum pennellii|NAC|NAC family protein	XP_010671871.1:PREDICTED: seipin2 [Beta vulgaris subsp. vulgaris]	-	A0A0J8CS03|A0A0J8CS03_BETVU:Uncharacterized protein;OS=Beta vulgaris subsp. vulgaris.;|GO:0016021,|GO:0019915,
DN119207_c3_g2_i9	UP	[GO:0005777:peroxisome][GO:0003995:acyl-CoA dehydrogenase activity][GO:0003997:acyl-CoA oxidase activity][GO:0050660:flavin adenine dinucleotide binding][GO:0006635:fatty acid beta-oxidation]	[ko00071:Fatty acid degradation][ko00592:alpha-Linolenic acid metabolism][ko01040:Biosynthesis of unsaturated fatty acids][ko03320:PPAR signaling pathway][ko04024:cAMP signaling pathway][ko04146:Peroxisome]	-	XP_021722500.1:acylcoenzyme A oxidase 3, peroxisomallike isoform X1 [Chenopodium quinoa]	gi|521576490|gb|GAIA01070862.1|:TSA: Salicornia europaea Unigene85211_Se200S transcribed RNA sequence	A0A0J8EFZ4|A0A0J8EFZ4_BETVU:Acyl-coenzyme A oxidase;OS=Beta vulgaris subsp. vulgaris.;|GO:0005777,|GO:0003995,GO:0003997,GO:0050660,|GO:0006635,
DN124180_c1_g7_i1	UP	-	-	-	-	-	-

geneid	trend	GO	Pathway	TF	NR	NT	Uniprot
DN120966_c0_g12_i3	UP	[GO:0003677:DNA binding]	-	Kalax.0274s0026.1.p:Kalanchoe marnieriana|MYB_related|MYB_related family protein	XP_021716220.1:transcription factor MYB48like isoform X2 [Chenopodium quinoa]	gi|388492991|gb|BT134767.1|:Medicago truncatula clone JCVI-FLMt-16D24 unknown mRNA	A0A059AGB7|A0A059AGB7_EUCGR: Uncharacterized protein;OS=Eucalyptus grandis;|GO:0003677,

geneid	trend	GO	Pathway	TF	NR	NT	Uniprot
DN128354_c1_g2_i10	UP	[GO:0003677:DNA binding][GO:0004518:nuclease activity]	-	-	XP_010680737.1:PREDICTED: uncharacterized protein LOC104895820 isoform X1 [Beta vulgaris subsp. vulgaris] >XP_010680738.1 PREDICTED: uncharacterized protein LOC104895820 isoform X1 [Beta vulgaris subsp. vulgaris] >XP_010680739.1 PREDICTED: uncharacterized protein LOC104895820 isoform X1 [Beta vulgaris subsp. vulgaris] >XP_010680741.1 PREDICTED: uncharacterized protein LOC104895820 isoform X2 [Beta vulgaris subsp.	gi|255566109|ref|XM_002523997.1|:Ricinus communis conserved hypothetical protein, mRNA	A0A0J8C9R2|A0A0J8C9R2_BETVU:Uncharacterized protein;OS=Beta vulgaris subsp. vulgaris.;|GO:0003677,GO:0004518,

geneid	trend	GO	Pathway	TF	NR	NT	Uniprot
DN110300_c0_g6_i5	UP	-	-	-	-	-	-
DN119817_c2_g1_i6	UP	[GO:0008686:3,4-dihydroxy-2-butanone-4-phosphate synthase activity][GO:0003935:GTP cyclohydrolase II activity][GO:0009231:riboflavin biosynthetic process]	[ko00740:Riboflavin metabolism]	Rsa1.0_03182.1_g00005.1:Raphanus sativus|C3H|C3H family protein	KDO60655.1:hypothetical protein CISIN_1g008849mg [Citrus sinensis]	gi|478163090|gb|GAHK01013513.1|:TSA: Jatropha curcas Unigene965_JC-CK_1A transcribed RNA sequence	A0A067F3I0|A0A067F3I0_CITSI:Uncharacterized protein;OS=Citrus sinensis;|GO:0008686,GO:0003935,|GO:0009231,
DN121725_c2_g1_i11	UP	-	-	-	XP_021754861.1:general transcription factor 3C polypeptide 6like [Chenopodium quinoa]	-	A0A0J8CK95|A0A0J8CK95_BETVU:Uncharacterized protein;OS=Beta vulgaris subsp. vulgaris.;
DN124104_c1_g3_i18	UP	[GO:0006511:ubiquitin-dependent protein catabolic process]	-	MDP0000304904:Malus domestica|TCP|TCP family protein	XP_021772924.1:cullin1like [Chenopodium quinoa]	-	A0A0J8CNX7|A0A0J8CNX7_BETVU:Uncharacterized protein;OS=Beta vulgaris subsp. vulgaris.;|GO:0006511,

geneid	trend	GO	Pathway	TF	NR	NT	Uniprot
DN118951_c1_g1_i9	UP	[GO:0016021:integral component of membrane]	-	GRMZM2G048403_P01:Zea mays|MYB_related|MYB_related family protein	XP_010688396.1:PREDICTED: uncharacterized protein LOC104902347 [Beta vulgaris subsp. vulgaris]	gi|147775340|emb|AM465187.2|:Vitis vinifera contig VV78X069854.3, whole genome shotgun sequence	A0A0J8BPR9|A0A0J8BPR9_BETVU:Uncharacterized protein;OS=Beta vulgaris subsp. vulgaris.;|GO:0016021,
DN128143_c0_g7_i2	UP	[GO:0003824:catalytic activity][GO:0050662:coenzyme binding]	-	MDP0000265073:Malus domestica|NAC|NAC family protein	XP_021729150.1:uncharacterized protein LOC110696170 [Chenopodium quinoa]	gi|147815392|emb|AM454817.2|:Vitis vinifera contig VV78X149673.4, whole genome shotgun sequence	A0A0J8CLD0|A0A0J8CLD0_BETVU:Uncharacterized protein;OS=Beta vulgaris subsp. vulgaris.;|GO:0003824,GO:0050662,
DN120771_c0_g1_i1	UP	[GO:0046872:metal ion binding][GO:0030001:metal ion transport]	-	678301159:Utricularia gibba|MIKC_MADS|MIKC_MADS family protein	KHG04831.1:superoxide dismutase 1 copper chaperone [Gossypium arboreum]	-	A0A0B0N118|A0A0B0N118_GOSAR:Superoxide dismutase 1 copper chaperone;OS=Gossypium arboreum;|GO:0046872,|GO:0030001,

geneid	trend	GO	Pathway	TF	NR	NT	Uniprot
DN117968_c1_g1_i11	UP	[GO:0016787:hydrolase activity][GO:0016042:lipid catabolic process]	-	-	XP_021770987.1:uncharacterized protein LOC110735137 [Chenopodium quinoa]	gi|502137572|ref|XM_004503071.1|:PREDICTED: Cicer arietinum uncharacterized LOC101501707 (LOC101501707), mRNA	A0A0J8CZ81|A0A0J8CZ81_BETVU:Uncharacterized protein;OS=Beta vulgaris subsp. vulgaris.;|GO:0016787,|GO:0016042,

geneid	trend	GO	Pathway	TF	NR	NT	Uniprot
DN122183_c0_g1_i2	UP	[GO:0005634:nucleus][GO:0003677:DNA binding][GO:0009738:abscisic acid-activated signaling pathway][GO:0050832:defense response to fungus][GO:0002229:defense response to oomycetes][GO:0009682:induced systemic resistance][GO:0009867:jasmonic acid mediated signaling pathway][GO:0009787:regulation of abscisic acid-activated signaling pathway][GO:2000071:regulation of defense response by callose deposition][GO:2000022:regulation of jasmonic acid mediated signaling pathway][GO:0009414:response to water deprivation][GO:0010118:stomatal movement]	-	Bv2_042870_fzxy.t1:Beta vulgaris|HB-other|HB-other family protein	XP_010670653.1:PREDICTED: protein OVEREXPRESSOR OF CATIONIC PEROXIDASE 3 [Beta vulgaris subsp. vulgaris] >KMT16800.1 hypothetical protein BVRB_2g042870 [Beta vulgaris subsp. vulgaris]	-	A0A0J8CSQ8|A0A0J8CSQ8_BETVU:Uncharacterized protein;OS=Beta vulgaris subsp. vulgaris.;|GO:0005634,|GO:0003677,|GO:0009738,GO:0050832,GO:0002229,GO:0009682,GO:0009867,GO:0009787,GO:2000071,GO:2000022,GO:0009414,GO:0010118,

geneid	trend	GO	Pathway	TF	NR	NT	Uniprot
DN123623_c1_g1_i4	UP	[GO:0003723:RNA binding]	-	-	XP_021730294.1:pumilio homolog 24like [Chenopodium quinoa]	-	A0A0J8CX64|A0A0J8CX64_BETVU:Uncharacterized protein;OS=Beta vulgaris subsp. vulgaris.;|GO:0003723,

geneid	trend	GO	Pathway	TF	NR	NT	Uniprot
DN126692_c9_g1_i5	UP	[GO:0016817:hydrolase activity, acting on acid anhydrides]	-	-	XP_010682137.1:PREDICTED: DExHbox ATPdependent RNA helicase DExH18, mitochondrial [Beta vulgaris subsp. vulgaris] >XP_010682138.1 PREDICTED: DExHbox ATPdependent RNA helicase DExH18, mitochondrial [Beta vulgaris subsp. vulgaris] >XP_010682139.1 PREDICTED: DExHbox ATPdependent RNA helicase DExH18, mitochondrial [Beta vulgaris subsp. vulgaris] >KMT07902.1	gi|521621512|gb|GAIA01055152.1|:TSA: Salicornia europaea Unigene64855_Se200S transcribed RNA sequence	A0A0J8C309|A0A0J8C309_BETVU:Uncharacterized protein;OS=Beta vulgaris subsp. vulgaris.;|GO:0016817,

geneid	trend	GO	Pathway	TF	NR	NT	Uniprot
					hypothetical protein BVRB_6g145980 [Beta vulgaris subsp. vulgaris]		

geneid	trend	GO	Pathway	TF	NR	NT	Uniprot
DN112718_c0_g1_i2	UP	-	[ko04141:Protein processing in endoplasmic reticulum]	WALNUT_00030998-RA:Juglans regia|NF-YB|NF-YB family protein	XP_021732378.1:18.3 kDa class I heat shock protein [Chenopodium quinoa]	-	A0A0J8CXG7|A0A0J8CXG7_BETVU: Uncharacterized protein;OS=Beta vulgaris subsp. vulgaris.;
DN123937_c0_g3_i2	UP	[GO:0005737:cytoplasm][GO:0033925:mannosyl-glycoprotein endo-beta-N-acetylglucosaminidase activity]	[ko00511:Other glycan degradation]	KN539694.1_FGP007:Oryza longistaminata|LBD|LBD family protein	XP_021855810.1:cytosolic endobetaNacetylglucosaminidase 1like isoform X1 [Spinacia oleracea]	gi|521660662|gb|GAIA01036916.1|:TSA: Salicornia europaea Unigene40983_Se200S transcribed RNA sequence	A0A0J8BEV7|A0A0J8BEV7_BETVU:Uncharacterized protein;OS=Beta vulgaris subsp. vulgaris.;|GO:0005737,|GO:0033925,
DN123363_c0_g3_i8	UP	-	-	-	XP_021844212.1:glutaminylpeptide cyclotransferase [Spinacia oleracea] >KNA17787.1 hypothetical protein SOVF_076840 [Spinacia oleracea]	gi|21403363|gb|AY084653.1|:Arabidopsis thaliana clone 114197 mRNA, complete sequence	A0A0K9RG25|A0A0K9RG25_SPIOL:Uncharacterized protein;OS=Spinacia oleracea;
DN116130_c3_g8_i16	UP	-	-	-	-	-	-

geneid	trend	GO	Pathway	TF	NR	NT	Uniprot
DN119742_c0_g1_i2	UP	[GO:0005829:cytosol][GO:0005634:nucleus][GO:0070694:deoxyribonucleoside 5'-monophosphate N-glycosidase activity][GO:0043733:DNA-3-methyl base glycosylase activity][GO:0070635:nicotinamide riboside hydrolase activity][GO:0070636:nicotinic acid riboside hydrolase activity][GO:0000701:purine-specific mismatch base pair DNA N-glycosylase activity][GO:0017065:single-strand selective uracil DNA N-glycosylase activity][GO:0009691:cytokinin biosynthetic process]	-	Vang05g09490.1:Vigna angularis|WRKY|WRKY family protein	XP_010690268.1:PREDICTED: cytokinin riboside 5'monophosphate phosphoribohydrolase LOG5 [Beta vulgaris subsp. vulgaris]	-	A0A0J8BIL4|A0A0J8BIL4_BETVU:Cytokinin riboside 5'-monophosphate phosphoribohydrolase;OS=Beta vulgaris subsp. vulgaris.;|GO:0005829,GO:0005634,|GO:0070694,GO:0043733,GO:0070635,GO:0070636,GO:0000701,GO:0017065,|GO:0009691,

geneid	trend	GO	Pathway	TF	NR	NT	Uniprot
DN106395_c0_g1_i1	UP	[GO:0043565:sequence-specific DNA binding][GO:0003700:DNA-binding transcription factor activity]	-	Bv7_159880_nyaj.t1:Beta vulgaris|bZIP|bZIP family protein	XP_021728696.1:uncharacterized protein LOC110695771 [Chenopodium quinoa]	-	A0A0J8C452|A0A0J8C452_BETVU:Uncharacterized protein;OS=Beta vulgaris subsp. vulgaris.;|GO:0043565,GO:0003700,

geneid	trend	GO	Pathway	TF	NR	NT	Uniprot
DN119237_c1_g7_i2	UP	[GO:0016021:integral component of membrane][GO:0005524:ATP binding][GO:0004674:protein serine/threonine kinase activity]	[ko04064:NF-kappa B signaling pathway][ko04620:Toll-like receptor signaling pathway][ko04624:Toll and Imd signaling pathway][ko04722: Neurotrophin signaling pathway][ko05133:Pertussis][ko05140:Leishmaniasis][ko05142:Chagas disease (American trypanosomiasis)][ko05145:Toxoplasmosis][ko05152:Tuberculosis][ko05162:Measles][ko05169:Epstein-Barr virus infection]	Aco009877.1:Ananas comosus|WRKY|WRKY family protein	XP_010690948.1:PREDICTED: putative receptorlike protein kinase At3g47110 isoform X1 [Beta vulgaris subsp. vulgaris] >XP_019107571.1 PREDICTED: putative receptorlike protein kinase At3g47110 isoform X1 [Beta vulgaris subsp. vulgaris] >XP_019107572.1 PREDICTED: putative receptorlike protein kinase At3g47110 isoform X1 [Beta vulgaris subsp. vulgaris] >XP_019107573.1 PREDICTED: putative receptorlike protein kinase At3g47110 isoform X1 [Beta vulgaris subsp. vulgaris] >XP_019107574.1 PREDICTED: putative receptorlike	-	A0A0J8BK63|A0A0J8BK63_BETVU:Uncharacterized protein;OS=Beta vulgaris subsp. vulgaris.;|GO:0016021,|GO:0005524,GO:0004674,

geneid	trend	GO	Pathway	TF	NR	NT	Uniprot
DN120439_c3_g1_i3	UP	-	-	-	XP_010102559.1:hypothetical protein L484_018031 [Morus notabilis] >EXB93645.1 hypothetical protein L484_018031 [Morus notabilis]	gi|388508431|gb|BT142488.1|:Lotus japonicus clone JCVI-FLLj-7P10 unknown mRNA	W9RII0|W9RII0_9ROSA:Uncharacterized protein;OS=Morus notabilis.;
DN128748_c1_g6_i1	UP	-	-	-	-	-	-
DN108672_c1_g1_i1	UP	-	-	Rsa1.0_04342.1_g00002.1:Raphanus sativus|LBD|LBD family protein	XP_021836103.1:uncharacterized protein LOC110775811 [Spinacia oleracea]	-	F4NCG3|F4NCG3_BETVU:Uncharacterized protein;OS=Beta vulgaris subsp. vulgaris.;
DN116301_c0_g2_i10	UP	[GO:0005524:ATP binding]	-	-	AAZ41846.1:Rubisco activase 2 [Mesembryanthemum crystallinum]	gi|71834883|gb|DQ087187.1|:Mesembryanthemum crystallinum Rubisco activase 2 mRNA, complete cds	Q45YI8|Q45YI8_MESCR:Rubisco activase 2;OS=Mesembryanthemum crystallinum;|GO:0005524,

geneid	trend	GO	Pathway	TF	NR	NT	Uniprot
DN119942_c0_g1_i5	UP	[GO:0016787:hydrolase activity][GO:0008152:metabolic process]	-	-	KOM38701.1:hypothetical protein LR48_Vigan03g208300 [Vigna angularis]	gi|502108257|ref|XM_004493371.1|:PREDICTED: Cicer arietinum haloacid dehalogenase-like hydrolase domain-containing protein 3-like (LOC101512920), mRNA	A0A0L9U7G3|A0A0L9U7G3_PHAAN: Uncharacterized protein;OS=Phaseolus angularis;|GO:0016787,|GO:0008152,
DN108838_c0_g2_i1	UP	-	-	-	-	-	-
DN115893_c4_g1_i2	UP	[GO:0016021:integral component of membrane]	-	-	XP_010667529.1:PREDICTED: uncharacterized protein LOC104884553 [Beta vulgaris subsp. vulgaris] >KMS95320.1 hypothetical protein BVRB_009370 [Beta vulgaris subsp. vulgaris]	-	A0A0J8DWT3|A0A0J8DWT3_BETVU: Uncharacterized protein;OS=Beta vulgaris subsp. vulgaris.;|GO:0016021,

geneid	trend	GO	Pathway	TF	NR	NT	Uniprot
DN120518_c1_g7_i4	UP	-	-	-	-	-	-
DN124150_c2_g2_i2	UP	[GO:0016788:hydrolase activity, acting on ester bonds]	-	-	XP_021720400.1:GDSL esterase/lipase 2like [Chenopodium quinoa]	gi|31616512|gb|AY299194.1|:Chenopodium rubrum GDSL-lipase mRNA, complete cds	A0A0J8BXN2|A0A0J8BXN2_BETVU: Uncharacterized protein;OS=Beta vulgaris subsp. vulgaris.;|GO:0016788,
DN123372_c3_g2_i1	UP	[GO:0016021:integral component of membrane][GO:0009678:hydrogen-translocating pyrophosphatase activity][GO:0004427:inorganic diphosphatase activity]	[ko00190:Oxidative phosphorylation]	XP_016511461.1:Nicotiana tabacum|B3|B3 family protein	OMO86263.1:Pyrophosphateenergised proton pump [Corchorus capsularis]	gi|359487543|ref|XM_002282322.2|:PREDICTED: Vitis vinifera H+-pyrophosphatase (LOC100233104), mRNA	A0A061F2U2|A0A061F2U2_THECC:Inorganic H pyrophosphatase family protein isoform 1;OS=Theobroma cacao;|GO:0016021,|GO:0009678,GO:0004427,|GO:0015992,

geneid	trend	GO	Pathway	TF	NR	NT	Uniprot
DN126830_c1_g3_i2	UP	[GO:0005524:ATP binding]	[ko03040:Spliceosome][ko04010:MAPK signaling pathway][ko04141:Protein processing in endoplasmic reticulum][ko04144:Endocytosis][ko04213:Longevity regulating pathway - multiple species][ko04612:Antigen processing and presentation][ko04915:Estrogen signaling pathway][ko05134:Legionellosis][ko05145:Toxoplasmosis][ko05162:Measles][ko05164:Influenza A][ko05169:Epstein-Barr virus infection]	FANhyb_rscf00000233.1.g00002.1:Fragaria x ananassa|GATA|GATA family protein	XP_021748678.1:heat shock 70 kDa proteinlike [Chenopodium quinoa]	gi|502150024|ref|XM_004507696.1|:PREDICTED: Cicer arietinum heat shock 70 kDa protein-like (LOC101492688), mRNA	A0A059AKI8|A0A059AKI8_EUCGR:Uncharacterized protein;OS=Eucalyptus grandis;|GO:0005524,

geneid	trend	GO	Pathway	TF	NR	NT	Uniprot
DN127991_c1_g1_i3	UP	[GO:0030598:rRNA N-glycosylase activity][GO:0006952:defense response][GO:0017148:negative regulation of translation]	-	-	AAL35962.1:bouganin [Bougainvillea spectabilis]	gi|17149253|gb|AF445416.1|AF445416: Bougainvillea spectabilis bouganin mRNA, complete cds	Q8W4U4|Q8W4U4_9CARY:rRNA N-glycosidase;OS=Bougainvillea spectabilis.;|GO:0030598,|GO:0006952, GO:0017148,
DN106864_c0_g1_i1	UP	[GO:0004089:carbonate dehydratase activity][GO:0008270:zinc ion binding][GO:0015976:carbon utilization]	[ko00910:Nitrogen metabolism]	Jcr4S00223.20:Jatropha curcas|bHLH|bHLH family protein	AEV42276.1:chloroplast beta carbonic anhydrase 1 [Mesembryanthemum nodiflorum]	gi|359372871|gb|JN228098.1|:Mesembryanthemum nodiflorum chloroplast beta carbonic anhydrase 1 (CA1) mRNA, complete cds; nuclear gene for chloroplast product	G9FZ35|G9FZ35_MESNO:Carbonic anhydrase;OS=Mesembryanthemum nodiflorum;|GO:0004089,GO:0008270,|GO:0015976,

geneid	trend	GO	Pathway	TF	NR	NT	Uniprot
DN128682_c2_g5_i9	UP	[GO:0046872:metal ion binding][GO:0016702:oxidoreductase activity, acting on single donors with incorporation of molecular oxygen, incorporation of two atoms of oxygen][GO:0006633:fatty acid biosynthetic process][GO:0031408:oxylipin biosynthetic process]	[ko00591:Linoleic acid metabolism][ko00592:alpha-Linolenic acid metabolism]	Lus10031505:Linum usitatissimum|NF-YA|NF-YA family protein	XP_021772004.1:linoleate 13Slipoxygenase 21, chloroplasticlike [Chenopodium quinoa]	-	W9QY99|W9QY99_9ROSA:Lipoxygenase;OS=Morus notabilis.;|GO:0046872,GO:0016702,|GO:0006633,GO:0031408,
DN121530_c4_g4_i1	UP	-	[ko04141:Protein processing in endoplasmic reticulum]	WALNUT_00030998-RA:Juglans regia|NF-YB|NF-YB family protein	XP_008465538.1:PREDICTED: 17.6 kDa class I heat shock protein 3like [Cucumis melo]	-	A0A0J8BZ31|A0A0J8BZ31_BETVU:Uncharacterized protein;OS=Beta vulgaris subsp. vulgaris.;
DN124851_c2_g5_i1	UP	[GO:0016021:integral component of membrane][GO:0005524:ATP binding][GO:0004674:protein serine/threonine kinase activity]	-	-	XP_021850836.1:probable LRR receptorlike serine/threonineprotein kinase At3g47570 [Spinacia oleracea]	-	A0A0K9R0L3|A0A0K9R0L3_SPIOL:Uncharacterized protein;OS=Spinacia oleracea;|GO:0016021,|GO:0005524,GO:0004674,

geneid	trend	GO	Pathway	TF	NR	NT	Uniprot
DN127497_c3_g1_i2	UP	[GO:0006457:protein folding]	-	-	XP_017632208.1:PREDICTED: peptidylprolyl cistrans isomerase FKBP13, chloroplasticlike isoform X1 [Gossypium arboreum]	-	A0A0F7GZF0|A0A0F7GZF0_9ASPA:FK506 binding protein 13;OS=Cypripedium formosanum.;|GO:0006457,
DN106838_c0_g11_i1	UP	[GO:0016021:integral component of membrane][GO:0005524:ATP binding][GO:0004674:protein serine/threonine kinase activity]	-	Aco009877.1:Ananas comosus|WRKY|WRKY family protein	XP_011469009.1:PREDICTED: putative receptorlike protein kinase At3g47110 isoform X1 [Fragaria vesca subsp. vesca]	gi|157283494|gb|EF652944.1|:Prunus cerasus var. caproniana isolate FIRIgl.62 kinase-like protein gene, partial cds	A0A0J8BGG3|A0A0J8BGG3_BETVU: Uncharacterized protein;OS=Beta vulgaris subsp. vulgaris.;|GO:0016021,|GO:0005524,GO:0004674,
DN117474_c0_g3_i6	UP	[GO:0016021:integral component of membrane][GO:0005524:ATP binding][GO:0004672:protein kinase activity]	-	SMil_00011498-RA_Salv:Salvia miltiorrhiza|bHLH|bHLH family protein	XP_021864481.1:probable LRR receptorlike serine/threonineprotein kinase At1g56140 isoform X1 [Spinacia oleracea]	gi|521493522|gb|GAHZ01037398.1|:TSA: Salicornia europaea Unigene42165_SeCKS transcribed RNA sequence	A0A0J8C0I1|A0A0J8C0I1_BETVU:Uncharacterized protein;OS=Beta vulgaris subsp. vulgaris.;|GO:0016021,|GO:0005524,GO:0004672,

geneid	trend	GO	Pathway	TF	NR	NT	Uniprot
DN119941_c0_g1_i1	UP	[GO:0003676:nucleic acid binding][GO:0008270:zinc ion binding]	-	PK03054.1:Cannabis sativa|HD-ZIP|HD-ZIP family protein	XP_015079427.1:PREDICTED: uncharacterized protein LOC107023294 [Solanum pennellii]	-	K4C139|K4C139_SOLLC:Uncharacterized protein;OS=Solanum lycopersicum;|GO:0003676,GO:0008270,
DN124309_c0_g1_i7	UP	-	-	-	KNA14922.1:hypothetical protein SOVF_102940 [Spinacia oleracea]	-	A0A0K9R7Z8|A0A0K9R7Z8_SPIOL:Uncharacterized protein;OS=Spinacia oleracea;
DN125872_c1_g2_i4	UP	[GO:0016020:membrane][GO:0003830:beta-1,4-mannosylglycoprotein 4-beta-N-acetylglucosaminyltransferase activity][GO:0006487:protein N-linked glycosylation]	[ko00510:N-Glycan biosynthesis]	Do024028.1:Dichanthelium oligosanthes|NAC|NAC family protein	XP_021852768.1:beta1,4mannosylglycoprotein 4betaNacetylglucosaminyltransferase [Spinacia oleracea] >KNA11801.1 hypothetical protein SOVF_131810 [Spinacia oleracea]	gi|502151496|ref|XM_004508413.1|:PREDICTED: Cicer arietinum uncharacterized LOC101501638 (LOC101501638), mRNA	A0A0K9QZ45|A0A0K9QZ45_SPIOL:Uncharacterized protein;OS=Spinacia oleracea;|GO:0016020,|GO:0003830,|GO:0006487,
DN169068_c	UP	-	-	-	-	-	-

geneid	trend	GO	Pathway	TF	NR	NT	Uniprot
0_g1_i1							
DN113474_c0_g1_i1	UP	[GO:0005524:ATP binding][GO:0019538:protein metabolic process]	[ko04213:Longevity regulating pathway - multiple species]	Achn116231:Actinidia chinensis|ARF|ARF family protein	XP_021761516.1:chaperone protein ClpB1 [Chenopodium quinoa]	-	A0A0K9R0K3|A0A0K9R0K3_SPIOL:Uncharacterized protein;OS=Spinacia oleracea;|GO:0005524,|GO:0019538,
DN113554_c0_g1_i1	UP	-	-	Sevir.9G255600.1.p:Setaria viridis|C2H2|C2H2 family protein	XP_021760049.1:protein trichome birefringencelike 19 [Chenopodium quinoa]	gi|521457484|gb|GAHZ01063000.1|:TSA: Salicornia europaea Unigene75813_SeCKS transcribed RNA sequence	A0A0K9QYV1|A0A0K9QYV1_SPIOL: Uncharacterized protein;OS=Spinacia oleracea;
DN75078_c0_g1_i1	UP	[GO:0016758:transferase activity, transferring hexosyl groups][GO:0008152:metabolic process]	[ko00965:Betalain biosynthesis]	-	BAG71126.1:glucosyltransferase [Phytolacca americana] >BAH05015.1 glucosyltransferase [Phytolacca americana]	-	B5MGN8|B5MGN8_PHYAM:Glycosyltransferase;OS=Phytolacca americana;|GO:0016758,|GO:0008152,

geneid	trend	GO	Pathway	TF	NR	NT	Uniprot
DN118477_c0_g4_i1	UP	[GO:0046872:metal ion binding][GO:0016702:oxidoreductase activity, acting on single donors with incorporation of molecular oxygen, incorporation of two atoms of oxygen][GO:0006633:fatty acid biosynthetic process][GO:0031408:oxylipin biosynthetic process]	[ko00591:Linoleic acid metabolism][ko00592:alpha-Linolenic acid metabolism]	-	KNA25435.1:hypothetical protein SOVF_005490 [Spinacia oleracea]	-	A0A0K9S108|A0A0K9S108_SPIOL:Lipoxygenase;OS=Spinacia oleracea;|GO:0046872,GO:0016702,|GO:0006633,GO:0031408,
DN127633_c6_g2_i3	UP	-	-	-	-	-	-
DN121979_c5_g7_i2	UP	-	-	-	-	-	-
DN106651_c0_g1_i1	UP	[GO:0005524:ATP binding][GO:0019538:protein metabolic process]	[ko04213:Longevity regulating pathway - multiple species]	Achn116231:Actinidia chinensis|ARF|ARF family protein	OWM90377.1:hypothetical protein CDL15_Pgr014679 [Punica granatum]	gi|351725606|ref|NM_001251193.1|:Glycine max heat shock protein (SB100), mRNA >gi|530206|gb|L35272.1|SOYSB100HS Glycine max heat shock protein (SB100) mRNA,	A0A061FWY3|A0A061FWY3_THECC:Heat shock protein 101;OS=Theobroma cacao;|GO:0005524,|GO:0019538,

geneid	trend	GO	Pathway	TF	NR	NT	Uniprot
						complete cds	
DN127412_c 3_g12_i3	UP	-	-	-	-	-	-
DN124180_c 1_g8_i1	UP	[GO:0016021:integral component of membrane][GO:0005524:ATP binding][GO:0004672:protein kinase activity]	-	-	XP_021731641.1:probable LRR receptorlike serine/threonineprotein kinase At3g47570 [Chenopodium quinoa]	-	A0A067KV63|A0A067KV63_JATCU: Uncharacterized protein;OS=Jatropha curcas;|GO:0016021,|GO:0005524,GO:0004672,
DN101637_c 0_g1_i1	UP	-	-	-	-	-	-
DN124576_c 0_g2_i5	UP	[GO:0008168:methyltransferase activity]	-	EMT20798:Aegilops tauschii|MYB_related|MYB_related family protein	XP_021738647.1:salicylate carboxymethyltransferaselike [Chenopodium	-	A0A0K9RIG8|A0A0K9RIG8_SPIOL:Uncharacterized protein;OS=Spinacia oleracea;|GO:00081

geneid	trend	GO	Pathway	TF	NR	NT	Uniprot
					quinoa]		68,
DN118165_c2_g6_i1	UP	[GO:0016021:integral component of membrane][GO:0008270:zinc ion binding]	-	XP_016459247.1:Nicotiana tabacum|C2H2|C2H2 family protein	XP_021768768.1:uncharacterized protein LOC110733056 isoform X1 [Chenopodium quinoa]	-	A0A0J8FGV2|A0A0J8FGV2_BETVU:Uncharacterized protein;OS=Beta vulgaris subsp. vulgaris.;|GO:0016021,|GO:0008270,
DN109715_c1_g1_i7	UP	[GO:0005840:ribosome][GO:0003735:structural constituent of ribosome][GO:0006412:translation]	[ko03010:Ribosome]	Araip.FQ8DT:Arachis ipaensis|SBP|SBP family protein	XP_010689247.1:PREDICTED: 50S ribosomal protein L9, chloroplastic [Beta vulgaris subsp. vulgaris] >KMT02272.1 hypothetical protein BVRB_9g206220 [Beta vulgaris subsp. vulgaris]	gi|521699774|gb|GAIA01017541.1|:TSA: Salicornia europaea Unigene18461_Se200S transcribed RNA sequence	A0A0J8BM33|A0A0J8BM33_BETVU: Uncharacterized protein;OS=Beta vulgaris subsp. vulgaris.;|GO:0005840,|GO:0003735,|GO:0006412,

geneid	trend	GO	Pathway	TF	NR	NT	Uniprot
DN109415_c0_g1_i1	UP	[GO:0006952:defense response][GO:0009607:response to biotic stimulus]	-	-	XP_021739486.1:kirolalike [Chenopodium quinoa]	-	A5AKK0|A5AKK0_VITVI:Putative uncharacterized protein;OS=Vitis vinifera;GN=VIT_01s0011g05120 {ECO:0000313|EMBL:CBI26885.3};|GO:0006952,GO:0009607,
DN65409_c0_g1_i1	UP	-	-	-	-	-	-
DN128394_c4_g4_i6	UP	-	-	Rsa1.0_04342.1_g00002.1:Raphanus sativus|LBD|LBD family protein	XP_020256684.1:uncharacterized protein LOC109833427 [Asparagus officinalis]	-	F4NCG3|F4NCG3_BETVU:Uncharacterized protein;OS=Beta vulgaris subsp. vulgaris.;

geneid	trend	GO	Pathway	TF	NR	NT	Uniprot
DN114643_c1_g1_i12	UP	[GO:0005634:nucleus][GO:0000977:RNA polymerase II regulatory region sequence-specific DNA binding][GO:0003700:DNA-binding transcription factor activity][GO:0000165:MAPK cascade][GO:0045944:positive regulation of transcription by RNA polymerase II][GO:0006351:transcription, DNA-templated]	-	Sp_023090_ietn.t2:Spinacia oleracea|MIKC_MADS|MIKC_MADS family protein	XP_021735939.1:MADSbox protein SOC1like [Chenopodium quinoa]	gi|285818378|gb|AY830920.2|:Pisum sativum SUPPRESSOR OF OVEREXPRESSION OF CONSTANS 1 a (SOC1a) mRNA, complete cds	A0A0K9RXL2|A0A0K9RXL2_SPIOL:Uncharacterized protein;OS=Spinacia oleracea;|GO:0005634,|GO:0000977,GO:0003700,|GO:0000165,GO:0045944,GO:0006351,
DN116058_c3_g3_i2	UP	[GO:0046872:metal ion binding][GO:0016702:oxidoreductase activity, acting on single donors with incorporation of molecular oxygen, incorporation of two atoms of oxygen][GO:0006633:fatty acid biosynthetic process][GO:0031408:oxylipin biosynthetic process]	[ko00591:Linoleic acid metabolism][ko00592:alpha-Linolenic acid metabolism]	Tp57577_TGAC_v2_mRNA8161:Trifolium pratense|MYB_related|MYB_related family protein	XP_021772004.1:linoleate 13Slipoxygenase 21, chloroplasticlike [Chenopodium quinoa]	-	A0A0K9S108|A0A0K9S108_SPIOL:Lipoxygenase;OS=Spinacia oleracea;|GO:0046872,GO:0016702,|GO:0006633,GO:0031408,

geneid	trend	GO	Pathway	TF	NR	NT	Uniprot
DN119835_c3_g1_i1	UP	[GO:0030060:L-malate dehydrogenase activity][GO:0005975:carbohydrate metabolic process][GO:0006108:malate metabolic process]	[ko00020:Citrate cycle (TCA cycle)][ko00270:Cysteine and methionine metabolism][ko00620:Pyruvate metabolism][ko00630:Glyoxylate and dicarboxylate metabolism][ko00710:Carbon fixation in photosynthetic organisms]	-	XP_021733853.1:malate dehydrogenase, chloroplasticlike [Chenopodium quinoa]	gi|521750824|gb|GAIA01000736.1|:TSA: Salicornia europaea Unigene750_Se200S transcribed RNA sequence	A0A0K9RQF2|A0A0K9RQF2_SPIOL:Uncharacterized protein;OS=Spinacia oleracea;|GO:0030060,|GO:0005975,GO:0006108,
DN128703_c3_g2_i1	UP	-	-	-	-	-	-
DN120634_c0_g1_i1	UP	[GO:0050589:leucocyanidin oxygenase activity][GO:0046872:metal ion binding]	-	676709864:Sisymbrium irio|STAT|STAT family protein	XP_021635709.1:protein DMR6LIKE OXYGENASE 2like [Hevea brasiliensis]	-	B9S9D4|B9S9D4_RICCO:Leucoanthocyanidin dioxygenase, putative;OS=Ricinus communis;|GO:0050589,GO:0046872,

geneid	trend	GO	Pathway	TF	NR	NT	Uniprot
DN114643_c1_g1_i8	UP	[GO:0005634:nucleus][GO:0000977:RNA polymerase II regulatory region sequence-specific DNA binding][GO:0003700:DNA-binding transcription factor activity][GO:0000165:MAPK cascade][GO:0045944:positive regulation of transcription by RNA polymerase II][GO:0006351:transcription, DNA-templated]	-	Sp_023090_ietn.t2:Spinacia oleracea|MIKC_MADS|MIKC_MADS family protein	XP_021844484.1:agamouslike MADSbox protein AGL19 [Spinacia oleracea] >KNA23649.1 hypothetical protein SOVF_023090 isoform B [Spinacia oleracea]	gi|285818378|gb|AY830920.2|:Pisum sativum SUPPRESSOR OF OVEREXPRESSION OF CONSTANS 1 a (SOC1a) mRNA, complete cds	A0A0K9RXL2|A0A0K9RXL2_SPIOL: Uncharacterized protein;OS=Spinacia oleracea;|GO:0005634,|GO:0000977,GO:0003700,|GO:0000165,GO:0045944,GO:0006351,

geneid	trend	GO	Pathway	TF	NR	NT	Uniprot
DN119057_c0_g6_i29	UP	[GO:0016491:oxidoreductase activity][GO:0008270:zinc ion binding]	[ko00010:Glycolysis / Gluconeogenesis][ko00071:Fatty acid degradation][ko00350:Tyrosine metabolism][ko00625:Chloroalkane and chloroalkene degradation][ko00626:Naphthalene degradation][ko00680:Methane metabolism][ko00830:Retinol metabolism][ko00980:Metabolism of xenobiotics by cytochrome P450][ko00982:Drug metabolism - cytochrome P450][ko05204:Chemical carcinogenesis]	-	GAV83260.1:ADH_zinc_N domaincontaining protein/ADH_N domaincontaining protein [Cephalotus follicularis]	-	E0CRP3|E0CRP3_VITVI:Putative uncharacterized protein;OS=Vitis vinifera;GN=VIT_18s0001g06360 {ECO:0000313|EMBL:CBI19197.3};|GO:0016491,GO:0008270,

geneid	trend	GO	Pathway	TF	NR	NT	Uniprot
DN98518_c1_g1_i1	UP	-	-	-	-	-	-
DN121920_c1_g1_i1	UP	[GO:0005524:ATP binding][GO:0006457:protein folding][GO:0006950:response to stress]	[ko04141:Protein processing in endoplasmic reticulum][ko04151:PI3K-Akt signaling pathway][ko04217:Necroptosis][ko04612:Antigen processing and presentation][ko04621:NOD-like receptor signaling pathway][ko04626:Plant-pathogen interaction][ko04657:IL-17 signaling pathway][ko04659:Th17 cell differentiation][ko04914:Progesterone-mediated oocyte maturation][ko04915:Estrogen signaling	Do001279.1:Dichanthelium oligosanthes|bZIP|bZIP family protein	XP_019168655.1:PREDICTED: heat shock protein 83like [Ipomoea nil]	gi|225426163|ref|XM_002278858.1|:PREDICTED: Vitis vinifera heat shock protein 83-like, transcript variant 1 (LOC100265661), mRNA	A0A067G9H4|A0A067G9H4_CITSI:Uncharacterized protein;OS=Citrus sinensis;|GO:0005524,|GO:0006457,GO:0006950,

geneid	trend	GO	Pathway	TF	NR	NT	Uniprot
			pathway][ko05200:Pathways in cancer][ko05215:Prostate cancer][ko05418:Fluid shear stress and atherosclerosis]				

geneid	trend	GO	Pathway	TF	NR	NT	Uniprot
DN124231_c1_g3_i11	UP	[GO:0016021:integral component of membrane]	-	-	XP_021857934.1:sulfite exporter TauE/SafE family protein 4 [Spinacia oleracea] >KNA13303.1 hypothetical protein SOVF_117870 [Spinacia oleracea]	gi|521746996|gb|GAIA01002365.1|:TSA: Salicornia europaea Unigene2418_Se200S transcribed RNA sequence	A0A0K9R1B8|A0A0K9R1B8_SPIOL:Uncharacterized protein;OS=Spinacia oleracea;|GO:0016021,
DN121488_c0_g7_i1	UP	[GO:0005634:nucleus][GO:0003677:DNA binding][GO:0006355:regulation of transcription, DNA-templated][GO:0006351:transcription, DNA-templated]	-	WALNUT_00009408-RA:Juglans regia|G2-like|G2-like family protein	XP_010689788.1:PREDICTED: probable transcription factor GLK1 isoform X1 [Beta vulgaris subsp. vulgaris]	gi|224095574|ref|XM_002310377.1|:Populus trichocarpa predicted protein, mRNA	A0A068V513|A0A068V513_COFCA:Uncharacterized protein;OS=Coffea canephora;|GO:0005634,|GO:0003677,|GO:0006355,GO:0006351,

geneid	trend	GO	Pathway	TF	NR	NT	Uniprot
DN120171_c0_g17_i1	UP	[GO:0009536:plastid][GO:0004497:monooxygenase activity][GO:0016984:ribulose-bisphosphate carboxylase activity][GO:0015977:carbon fixation][GO:0009853:photorespiration][GO:0015979:photosynthesis]	[ko00630:Glyoxylate and dicarboxylate metabolism][ko00710:Carbon fixation in photosynthetic organisms]	mrna16933.1-v1.0-hybrid:Fragaria vesca|M-type_MADS|M-type_MADS family protein	XP_020997029.1:ribulose bisphosphate carboxylase small chain, chloroplastic isoform X2 [Arachis duranensis]	gi|521548633|gb|GAHZ01000024.1|:TSA: Salicornia europaea Unigene24_SeCKS transcribed RNA sequence	F4HRR5|F4HRR5_ARATH:Ribulose bisphosphate carboxylase small chain;OS=Arabidopsis thaliana;|GO:0009536,|GO:0004497,GO:0016984,|GO:0015977,GO:0009853,GO:0015979,
DN122803_c0_g8_i1	UP	[GO:0030598:rRNA N-glycosylase activity][GO:0006952:defense response][GO:0017148:negative regulation of translation]	-	-	AAL35962.1:bouganin [Bougainvillea spectabilis]	gi|17149253|gb|AF445416.1|AF445416: Bougainvillea spectabilis bouganin mRNA, complete cds	Q8W4U4|Q8W4U4_9CARY:rRNA N-glycosidase;OS=Bougainvillea spectabilis.;|GO:0030598,|GO:0006952,GO:0017148,

geneid	trend	GO	Pathway	TF	NR	NT	Uniprot
DN113019_c1_g1_i5	UP	[GO:0030132:clathrin coat of coated pit][GO:0030130:clathrin coat of trans-Golgi network vesicle][GO:0005198:structural molecule activity][GO:0006886:intracellular protein transport][GO:0016192:vesicle-mediated transport]	[ko04142:Lysosome][ko04144:Endocytosis][ko04721:Synaptic vesicle cycle][ko04961:Endocrine and other factor-regulated calcium reabsorption][ko05016:Huntington's disease][ko05100:Bacterial invasion of epithelial cells]	GRMZM2G395549_P01:Zea mays|bHLH|bHLH family protein	XP_021859505.1:clathrin heavy chain 1like [Spinacia oleracea] >KNA23309.1 hypothetical protein SOVF_026060 [Spinacia oleracea]	gi|224112466|ref|XM_002316165.1|:Populus trichocarpa predicted protein, mRNA	A0A0K9RWL2|A0A0K9RWL2_SPIOL:Clathrin heavy chain;OS=Spinacia oleracea;|GO:0030132,GO:0030130,|GO:0005198,|GO:0006886,GO:0016192,
DN114172_c0_g1_i1	UP	[GO:0005737:cytoplasm][GO:0005634:nucleus][GO:0008565:obsolete protein transporter activity][GO:0006606:protein import into nucleus]	-	MDP0000309902:Malus domestica|GATA|GATA family protein	KYP57914.1:Importin subunit alpha1 [Cajanus cajan]	-	A0A151SSU3|A0A151SSU3_CAJCA:Importin subunit alpha-1;OS=Cajanus cajan;|GO:0005737,GO:0005634,|GO:0008565,|GO:0006606,
DN114298_c3_g1_i1	UP	[GO:0031390:Ctf18 RFC-like complex][GO:0007064:mitotic sister chromatid cohesion]	-	OPUNC09G03970.3:Oryza punctata|G2-like|G2-like family protein	XP_021764880.1:putative uncharacterized protein DDB_G0287975	-	A0A0K9RWS2|A0A0K9RWS2_SPIOL:Uncharacterized protein;OS=Spinacia oleracea;|GO:00313

geneid	trend	GO	Pathway	TF	NR	NT	Uniprot
					[Chenopodium quinoa]		90,|GO:0007064,
DN128337_c2_g1_i4	UP	[GO:0016021:integral component of membrane][GO:0009506:plasmodesma]	-	-	XP_010667283.1:PREDICTED: uncharacterized protein LOC104884344 isoform X1 [Beta vulgaris subsp. vulgaris]	-	A0A0J8B3C4|A0A0J8B3C4_BETVU:Uncharacterized protein;OS=Beta vulgaris subsp. vulgaris.;|GO:0016021,GO:0009506,
DN117762_c0_g5_i1	UP	[GO:0016021:integral component of membrane]	-	-	KMT08510.1:hypothetical protein BVRB_6g137950 isoform A [Beta vulgaris subsp. vulgaris]	-	A0A0J8C413|A0A0J8C413_BETVU:Uncharacterized protein;OS=Beta vulgaris subsp. vulgaris.;|GO:0016021,

geneid	trend	GO	Pathway	TF	NR	NT	Uniprot
DN123894_c0_g1_i8	UP	-	-	-	XP_021717427.1:uncharacterized protein LOC110685243 [Chenopodium quinoa] >XP_021717428.1 uncharacterized protein LOC110685243 [Chenopodium quinoa] >XP_021717429.1 uncharacterized protein LOC110685243 [Chenopodium quinoa]	-	A0A0J8B5U4|A0A0J8B5U4_BETVU:Uncharacterized protein;OS=Beta vulgaris subsp. vulgaris.;
DN115642_c3_g1_i5	UP	-	-	-	XP_021769921.1:uncharacterized protein LOC110734179 [Chenopodium quinoa]	gi|521561643|gb|GAIA01076279.1|:TSA: Salicornia europaea Unigene92362_Se200S transcribed RNA sequence	A0A0J8FN88|A0A0J8FN88_BETVU:Uncharacterized protein;OS=Beta vulgaris subsp. vulgaris.;
DN119877_c7_g4_i1	UP	-	-	-	-	-	-

geneid	trend	GO	Pathway	TF	NR	NT	Uniprot
DN126519_c0_g7_i1	UP	[GO:0009535:chloroplast thylakoid membrane][GO:0016021:integral component of membrane][GO:0009522:photosystem I][GO:0009523:photosystem II][GO:0016168:chlorophyll binding][GO:0046872:metal ion binding][GO:0009765:photosynthesis, light harvesting][GO:0018298:protein-chromophore linkage]	[ko00196:Photosynthesis - antenna proteins]	Bradi3g42627.3.p:Brachypodium distachyon|ERF|ERF family protein	XP_021849032.1:photosystem I chlorophyll a/bbinding protein 5, chloroplastic [Spinacia oleracea]	gi|478160826|gb|GAHK01015777.1|:TSA: Jatropha curcas Unigene3231_JC-CK_1A transcribed RNA sequence	A0A0K9RPR5|A0A0K9RPR5_SPIOL:Chlorophyll a-b binding protein, chloroplastic;OS=Spinacia oleracea;|GO:0009535,GO:0016021,GO:0009522,GO:0009523,|GO:0016168,GO:0046872,|GO:0009765,GO:0018298,
DN123363_c0_g3_i6	UP	-	-	-	XP_010671626.1:PREDICTED: glutaminylpeptide cyclotransferase isoform X1 [Beta vulgaris subsp. vulgaris]	-	A0A0J8FJU8|A0A0J8FJU8_BETVU:Uncharacterized protein;OS=Beta vulgaris subsp. vulgaris.;

geneid	trend	GO	Pathway	TF	NR	NT	Uniprot
DN116859_c1_g1_i19	UP	[GO:0016021:integral component of membrane][GO:0008233:peptidase activity][GO:0006465:signal peptide processing]	[ko03060:Protein export]	-	XP_010691888.1:PREDICTED: signal peptidase complex catalytic subunit SEC11A isoform X2 [Beta vulgaris subsp. vulgaris] >KMT00301.1 hypothetical protein BVRB_1g016550 isoform B [Beta vulgaris subsp. vulgaris]	-	A0A0J8EB87|A0A0J8EB87_BETVU:Signal peptidase I;OS=Beta vulgaris subsp. vulgaris.;|GO:0016021,|GO:0008233,|GO:0006465,
DN128832_c2_g2_i1	UP	-	-	-	-	-	-

geneid	trend	GO	Pathway	TF	NR	NT	Uniprot
DN127035_c6_g1_i1	UP	[GO:0004866:endopeptidase inhibitor activity]	-	-	ANC73390.1:aplha amylase inhibitor [Amaranthus hypochondriacus] >ANC73391.1 alpha amylase inhibitor [Achyranthes aspera] >ANC73392.1 alpha amylase inhibitor [Celosia argentea] >ANC73393.1 alpha amylase inhibitor [Amaranthus paniculatus]	-	A0A0J8CNK7|A0A0J8CNK7_BETVU: Uncharacterized protein;OS=Beta vulgaris subsp. vulgaris.;|GO:0004866,
DN126318_c1_g1_i1	UP	[GO:0004451:isocitrate lyase activity][GO:0019752:carboxylic acid metabolic process]	[ko00630:Glyoxylate and dicarboxylate metabolism]	-	XP_010687663.1:PREDICTED: isocitrate lyase [Beta vulgaris subsp. vulgaris] >KMT03505.1 hypothetical protein BVRB_8g191840 [Beta vulgaris subsp. vulgaris]	gi|255730958|ref|XM_002550358.1|:Candida tropicalis MYA-3404 isocitrate lyase, mRNA	A0A0J8BQ05|A0A0J8BQ05_BETVU:Isocitrate lyase;OS=Beta vulgaris subsp. vulgaris.;|GO:0004451,|GO:0019752,

geneid	trend	GO	Pathway	TF	NR	NT	Uniprot
DN115194_c0_g10_i1	UP	[GO:0009570:chloroplast stroma][GO:0005829:cytosol][GO:0046872:metal ion binding][GO:0016491:oxidoreductase activity][GO:0006950:response to stress][GO:0009228:thiamine biosynthetic process][GO:0052837:thiazole biosynthetic process]	[ko00730:Thiamine metabolism]	-	OWM81248.1:hypothetical protein CDL15_Pgr007286 [Punica granatum]	-	Unreviewed|Q84QE4_TOBAC:Thiamine thiazole synthase, chloroplastic;OS=Nicotiana tabacum;|GO:0009570,GO:0005829,|GO:0046872,GO:0016491,|GO:0006950,GO:0009228,GO:0052837,
DN126027_c2_g1_i8	UP	[GO:0050660:flavin adenine dinucleotide binding][GO:0016614:oxidoreductase activity, acting on CH-OH group of donors]	-	CCG025416.1:Populus euphratica|G2-like|G2-like family protein	XP_010676566.1:PREDICTED: protein HOTHEAD isoform X1 [Beta vulgaris subsp. vulgaris]	gi|521516688|gb|GAHZ01015539.1|:TSA: Salicornia europaea Unigene16244_SeCKS transcribed RNA sequence	A0A0J8CJ91|A0A0J8CJ91_BETVU:Uncharacterized protein;OS=Beta vulgaris subsp. vulgaris.;|GO:0050660,GO:0016614,
DN124826_c1_g6_i3	UP	-	-	mrna13309.1-v1.0-hybrid:Fragaria vesca|FAR1|FAR1 family protein	XP_021765163.1:protein IQDOMAIN 14like [Chenopodium quinoa]	-	A0A0K9RC71|A0A0K9RC71_SPIOL:Uncharacterized protein;OS=Spinacia oleracea;

geneid	trend	GO	Pathway	TF	NR	NT	Uniprot
DN9666_c0_g1_i1	UP	[GO:0006952:defense response][GO:0009607:response to biotic stimulus]	-	-	XP_021745404.1:MLPlike protein 43 [Chenopodium quinoa] >XP_021745405.1 MLPlike protein 43 [Chenopodium quinoa] >XP_021749487.1 MLPlike protein 43 [Chenopodium quinoa]	-	A0A0K9S0I6|A0A0K9S0I6_SPIOL:Uncharacterized protein;OS=Spinacia oleracea;|GO:0006952,GO:0009607,
DN123413_c1_g2_i3	UP	[GO:0016021:integral component of membrane][GO:0016844:strictosidine synthase activity][GO:0009058:biosynthetic process]	-	KN538684.1_FGP011:Oryza longistaminata|MYB_related|MYB_related family protein	XP_010672984.1:PREDICTED: protein STRICTOSIDINE SYNTHASELIKE 10 [Beta vulgaris subsp. vulgaris] >KMT15141.1 hypothetical protein BVRB_3g062580 [Beta vulgaris subsp. vulgaris]	-	A0A0J8FGY4|A0A0J8FGY4_BETVU:Uncharacterized protein;OS=Beta vulgaris subsp. vulgaris.;|GO:0016021,|GO:0016844,|GO:0009058,

geneid	trend	GO	Pathway	TF	NR	NT	Uniprot
DN114679_c5_g1_i5	UP	-	-	-	XP_021734517.1:receptor like protein 30like [Chenopodium quinoa]	-	A0A0K9RX07|A0A0K9RX07_SPIOL:Uncharacterized protein;OS=Spinacia oleracea;
DN121488_c0_g6_i1	UP	[GO:0003676:nucleic acid binding][GO:0015074:DNA integration]	-	XP_016649571.1:Prunus mume|G2-like|G2-like family protein	AAT38758.1:Putative gagpol polyprotein, identical [Solanum demissum]	-	Q6L3N8|Q6L3N8_SOLDE:Putative gag-pol polyprotein, identical;OS=Solanum demissum;|GO:0003676,|GO:0015074,
DN124482_c2_g3_i4	UP	-	[ko04141:Protein processing in endoplasmic reticulum]	-	OMO66971.1:hypothetical protein CCACVL1_20870 [Corchorus capsularis]	gi|521705427|gb|GAIA01015221.1|:TSA: Salicornia europaea Unigene15895_Se200S transcribed RNA sequence	A0A0K9R4N4|A0A0K9R4N4_SPIOL:Uncharacterized protein;OS=Spinacia oleracea;

geneid	trend	GO	Pathway	TF	NR	NT	Uniprot
DN127450_c0_g1_i4	UP	[GO:0016161:beta-amylase activity][GO:0000272:polysaccharide catabolic process]	[ko00500:Starch and sucrose metabolism]	Spipo1G0030700:Spirodela polyrhiza|BES1|BES1 family protein	XP_010695452.1:PREDICTED: betaamylase 3, chloroplastic [Beta vulgaris subsp. vulgaris] >KMS97658.1 hypothetical protein BVRB_5g124880 [Beta vulgaris subsp. vulgaris]	gi|255552939|ref|XM_002517467.1|:Ricinus communis Beta-amylase, putative, mRNA	A0A0J8BC68|A0A0J8BC68_BETVU:Beta-amylase;OS=Beta vulgaris subsp. vulgaris.;|GO:0016161,|GO:0000272,
DN128682_c2_g5_i12	UP	[GO:0046872:metal ion binding][GO:0016702:oxidoreductase activity, acting on single donors with incorporation of molecular oxygen, incorporation of two atoms of oxygen][GO:0006633:fatty acid biosynthetic process][GO:0031408:oxylipin biosynthetic process]	[ko00591:Linoleic acid metabolism][ko00592:alpha-Linolenic acid metabolism]	Lus10031505:Linum usitatissimum|NF-YA|NF-YA family protein	XP_021772004.1:linoleate 13Slipoxygenase 21, chloroplasticlike [Chenopodium quinoa]	gi|521542694|gb|GAHZ01002082.1|:TSA: Salicornia europaea Unigene2137_SeCKS transcribed RNA sequence	M5W1M2|M5W1M2_PRUPE:Lipoxygenase;OS=Prunus persica;|GO:0046872,GO:0016702,|GO:0006633,GO:0031408,

geneid	trend	GO	Pathway	TF	NR	NT	Uniprot
DN119333_c6_g3_i5	UP	[GO:0009507:chloroplast]	-	-	XP_010674978.1:PREDICTED: uncharacterized protein LOC104891035 [Beta vulgaris subsp. vulgaris]	gi|521736370|gb|GAIA01008725.1|:TSA: Salicornia europaea Unigene9074_Se200S transcribed RNA sequence	A0A0J8CNE4|A0A0J8CNE4_BETVU:Uncharacterized protein;OS=Beta vulgaris subsp. vulgaris.;|GO:0009507,
DN120771_c0_g1_i7	UP	[GO:0046872:metal ion binding][GO:0030001:metal ion transport]	-	678301159:Utricularia gibba|MIKC_MADS|MIKC_MADS family protein	XP_010686628.1:PREDICTED: heavy metalassociated isoprenylated plant protein 7 [Beta vulgaris subsp. vulgaris] >KMT04370.1 hypothetical protein BVRB_8g184430 [Beta vulgaris subsp. vulgaris]	-	A0A0J8BS74|A0A0J8BS74_BETVU:Uncharacterized protein;OS=Beta vulgaris subsp. vulgaris.;|GO:0046872,|GO:0030001,

geneid	trend	GO	Pathway	TF	NR	NT	Uniprot
DN109575_c0_g1_i2	UP	-	-	-	XP_010695811.1:PREDICTED: uncharacterized protein LOC104908389 [Beta vulgaris subsp. vulgaris] >KMT18893.1 hypothetical protein BVRB_2g029940 [Beta vulgaris subsp. vulgaris]	-	A0A0J8D3Y5|A0A0J8D3Y5_BETVU:Uncharacterized protein;OS=Beta vulgaris subsp. vulgaris.;
DN112432_c0_g2_i2	UP	-	-	-	-	-	-
DN119835_c3_g1_i2	UP	[GO:0030060:L-malate dehydrogenase activity][GO:0005975:carbohydrate metabolic process][GO:0006108:malate metabolic process]	[ko00020:Citrate cycle (TCA cycle)][ko00270:Cysteine and methionine metabolism][ko00620:Pyruvate metabolism][ko00630:Glyoxylate and dicarboxylate metabolism][ko00710:Carbon fixation in	-	XP_021733853.1:malate dehydrogenase, chloroplasticlike [Chenopodium quinoa]	gi|521750824|gb|GAIA01000736.1|:TSA: Salicornia europaea Unigene750_Se200S transcribed RNA sequence	A0A0K9RQF2|A0A0K9RQF2_SPIOL:Uncharacterized protein;OS=Spinacia oleracea;|GO:0030060,|GO:0005975,GO:0006108,

geneid	trend	GO	Pathway	TF	NR	NT	Uniprot
			photosynthetic organisms]				
DN110971_c0_g1_i3	UP	-	-	-	-	-	-

geneid	trend	GO	Pathway	TF	NR	NT	Uniprot
DN121635_c0_g1_i4	UP	[GO:0005829:cytosol][GO:0004130:cytochrome-c peroxidase activity][GO:0020037:heme binding][GO:0034599:cellular response to oxidative stress][GO:0042744:hydrogen peroxide catabolic process][GO:0000302:response to reactive oxygen species][GO:0009845:seed germination][GO:0010431:seed maturation]	[ko00053:Ascorbate and aldarate metabolism][ko00480:Glutathione metabolism]	-	XP_021738555.1:putative Lascorbate peroxidase 6 [Chenopodium quinoa]	gi|357466208|ref|XM_003603341.1|:Medicago truncatula hypothetical protein (MTR_3g107060) mRNA, complete cds	D7SUU6|D7SUU6_VITVI:Putative uncharacterized protein;OS=Vitis vinifera;GN=VIT_04s0008g05490 {ECO:0000313|EMBL:CBI21045.3};|GO:0005829,|GO:0004130,GO:0020037,|GO:0034599,GO:0042744,GO:0000302,GO:0009845,GO:0010431,

geneid	trend	GO	Pathway	TF	NR	NT	Uniprot
DN120287_c5_g4_i1	UP	[GO:0005618:cell wall][GO:0009507:chloroplast][GO:0005886:plasma membrane][GO:0005524:ATP binding]	[ko03040:Spliceosome][ko04010:MAPK signaling pathway][ko04141:Protein processing in endoplasmic reticulum][ko04144:Endocytosis][ko04213:Longevity regulating pathway - multiple species][ko04612:Antigen processing and presentation][ko04915:Estrogen signaling pathway][ko05134:Legionellosis][ko05145:Toxoplasmosis][ko05162:Measles][ko05164:Influenza A][ko05169:Epstein-Barr virus infection]	FANhyb_rscf00000233.1.g00002.1:Fragaria x ananassa|GATA|GATA family protein	XP_009615698.1:PREDICTED: heat shock 70 kDa protein [Nicotiana tomentosiformis] >XP_016474934.1 PREDICTED: heat shock 70 kDa protein [Nicotiana tabacum]	gi|357480002|ref|XM_003610239.1|:Medicago truncatula Heat-shock protein (MTR_4g130540) mRNA, complete cds	K4BLR5|K4BLR5_SOLLC:Uncharacterized protein;OS=Solanum lycopersicum;|GO:0005618,GO:0009507,GO:0005886,|GO:0005524,

geneid	trend	GO	Pathway	TF	NR	NT	Uniprot
DN80569_c0_g2_i1	UP	-	-	-	-	-	-
DN127740_c4_g3_i1	UP	[GO:0004185:serine-type carboxypeptidase activity]	-	KZV23174.1:Dorcoceras hygrometricum|C3H|C3H family protein	KMT14734.1:hypothetical protein BVRB_4g075000 [Beta vulgaris subsp. vulgaris]	gi|521449580|gb|GAHZ01070904.1|:TSA: Salicornia europaea Unigene86491_SeCKS transcribed RNA sequence	A0A0J8CRS9|A0A0J8CRS9_BETVU:Carboxypeptidase;OS=Beta vulgaris subsp. vulgaris.;|GO:0004185,
DN120278_c2_g4_i3	UP	-	-	-	XP_021857368.1:uncharacterized protein LOC110796612 [Spinacia oleracea]	-	-
DN122442_c3_g1_i5	UP	-	[ko04075:Plant hormone signal transduction]	-	XP_010686279.1:PREDICTED: protein TRANSPORT INHIBITOR RESPONSE 1 [Beta vulgaris subsp. vulgaris] >KMT04489.1 hypothetical protein BVRB_8g181550 [Beta vulgaris subsp.	-	A0A0J8BWU9|A0A0J8BWU9_BETVU:Uncharacterized protein;OS=Beta vulgaris subsp. vulgaris.;

geneid	trend	GO	Pathway	TF	NR	NT	Uniprot
					vulgaris]		
DN120084_c 2_g1_i2	UP	[GO:0005634:nucleus][GO:0003677: DNA binding][GO:0006355:regulation of transcription, DNA-templated][GO:0006351:transc ription, DNA-templated]	-	SapurV1A.3982s001 0.1.p:Salix purpurea|NAC|NAC family protein	XP_011025924.1:P REDICTED: NAC domaincontaining protein 7like isoform X2 [Populus euphratica]	gi|73759950|dbj|AB 217775.1|:Zinnia elegans Ze567 mRNA for NAC-domain protein Ze567, complete cds	R4N7K6|R4N7K6_J ATCU:NAC transcription factor 017;OS=Jatropha curcas;|GO:0005634 ,|GO:0003677,|GO:0 006355,GO:0006351 ,
DN117762_c 0_g7_i7	UP	[GO:0016021:integral component of membrane]	-	676708970:Sisymbri um irio|ERF|ERF family protein	XP_010680925.1:P REDICTED: uncharacterized protein LOC104895967 [Beta vulgaris subsp. vulgaris]	-	A0A0J8C413|A0A0 J8C413_BETVU:Un characterized protein;OS=Beta vulgaris subsp. vulgaris.;|GO:00160 21,

geneid	trend	GO	Pathway	TF	NR	NT	Uniprot
DN115034_c0_g4_i1	UP	-	-	-	XP_015383344.1:PREDICTED: receptorlike protein 12 isoform X2 [Citrus sinensis]	-	A0A067DCS8|A0A067DCS8_CITSI:Uncharacterized protein;OS=Citrus sinensis;
DN88909_c0_g2_i1	UP	-	-	-	-	-	-
DN118998_c0_g1_i1	UP	[GO:0016021:integral component of membrane][GO:0005524:ATP binding][GO:0005388:calcium-transporting ATPase activity][GO:0046872:metal ion binding]	-	-	XP_021837180.1:calciumtransporting ATPase 2, plasma membranetype [Spinacia oleracea] >KNA10150.1 hypothetical protein SOVF_147120 [Spinacia oleracea]	gi|4884965|gb|AF145478.1|AF145478:Mesembryanthemum crystallinum calcium ATPase mRNA, partial cds	A0A0K9QSD7|A0A0K9QSD7_SPIOL:Calcium-transporting ATPase;OS=Spinacia oleracea;|GO:0016021,|GO:0005524,GO:0005388,GO:0046872,
DN120325_c0_g1_i2	UP	[GO:0046872:metal ion binding][GO:0016491:oxidoreductase activity]	-	Zpz_sc00083.1.g00290.1.am.mk:Zoysia pacifica|Dof|Dof family protein	KMT00547.1:hypothetical protein BVRB_9g218460 [Beta vulgaris subsp. vulgaris]	-	A0A0J8BHV9|A0A0J8BHV9_BETVU: Uncharacterized protein;OS=Beta vulgaris subsp. vulgaris.;|GO:0046872,GO:0016491,

geneid	trend	GO	Pathway	TF	NR	NT	Uniprot
DN122429_c0_g1_i2	UP	[GO:0016021:integral component of membrane][GO:0005524:ATP binding][GO:0004672:protein kinase activity]	[ko04064:NF-kappa B signaling pathway][ko04620:Toll-like receptor signaling pathway][ko04621:NOD-like receptor signaling pathway][ko04624:Toll and Imd signaling pathway][ko04722:Neurotrophin signaling pathway][ko05133:Pertussis][ko05140:Leishmaniasis][ko05142:Chagas disease (American trypanosomiasis)][ko05145:Toxoplasmosis][ko05152:Tuberculosis][ko05162:Measles][ko05164:Influenza A]	Do000506.1:Dichanthelium oligosanthes|HD-ZIP|HD-ZIP family protein	XP_021861471.1:LEAF RUST 10 DISEASERESISTANCE LOCUS RECEPTORLIKE PROTEIN KINASElike 2.1 [Spinacia oleracea]	-	A0A0K9RJ85|A0A0K9RJ85_SPIOL:Uncharacterized protein;OS=Spinacia oleracea;|GO:0016021,|GO:0005524,GO:0004672,

geneid	trend	GO	Pathway	TF	NR	NT	Uniprot
DN124731_c2_g4_i2	UP	[GO:0003676:nucleic acid binding][GO:0008270:zinc ion binding][GO:0015074:DNA integration]	-	Sopen12g015990.1: Solanum pennellii|TCP|TCP family protein	AAT39297.2:Gagpol protein, putative [Solanum demissum]	-	Q6L3M7|Q6L3M7_SOLDE:Gag-pol protein, putative;OS=Solanum demissum;|GO:0003676,GO:0008270,|GO:0015074,
DN120170_c2_g1_i1	UP	[GO:0046872:metal ion binding][GO:0016702:oxidoreductase activity, acting on single donors with incorporation of molecular oxygen, incorporation of two atoms of oxygen][GO:0006633:fatty acid biosynthetic process][GO:0031408:oxylipin biosynthetic process]	[ko00591:Linoleic acid metabolism][ko00592:alpha-Linolenic acid metabolism]	Lus10031505:Linum usitatissimum|NF-YA|NF-YA family protein	XP_021772004.1:linoleate 13Slipoxygenase 21, chloroplasticlike [Chenopodium quinoa]	-	A0A0J8FF20|A0A0J8FF20_BETVU:Lipoxygenase;OS=Beta vulgaris subsp. vulgaris.;|GO:0046872,GO:0016702,|GO:0006633,GO:0031408,

geneid	trend	GO	Pathway	TF	NR	NT	Uniprot
DN122045_c3_g2_i7	UP	[GO:0016020:membrane][GO:0020037:heme binding][GO:0005506:iron ion binding][GO:0016709:oxidoreductase activity, acting on paired donors, with incorporation or reduction of molecular oxygen, NAD(P)H as one donor, and incorporation of one atom of oxygen][GO:0044550:secondary metabolite biosynthetic process]	-	676766070:Sisymbrium irio|bHLH|bHLH family protein	XP_021851209.1:cytochrome P450 83B1like [Spinacia oleracea]	-	A5CBQ0|A5CBQ0_VITVI:Putative uncharacterized protein;OS=Vitis vinifera;GN=VIT_18s0001g13820 {ECO:0000313|EMBL:CCB46013.1};|GO:0016020,|GO:0020037,GO:0005506,GO:0016709,|GO:0044550,
DN120344_c3_g1_i1	UP	-	[ko04141:Protein processing in endoplasmic reticulum]	WALNUT_00030998-RA:Juglans regia|NF-YB|NF-YB family protein	XP_021720644.1:22.7 kDa class IV heat shock proteinlike [Chenopodium quinoa]	-	A0A0J8C6M4|A0A0J8C6M4_BETVU: Uncharacterized protein;OS=Beta vulgaris subsp. vulgaris.;
DN128271_c3_g1_i3	UP	-	-	-	KNA11917.1:hypothetical protein SOVF_130610 [Spinacia oleracea]	-	A0A0K9QXC2|A0A0K9QXC2_SPIOL: Uncharacterized protein;OS=Spinacia oleracea;

geneid	trend	GO	Pathway	TF	NR	NT	Uniprot
DN118441_c3_g1_i3	UP	[GO:0003824:catalytic activity]	-	-	XP_010669338.1:PREDICTED: thermospermine synthase ACAULIS5 isoform X2 [Beta vulgaris subsp. vulgaris] >KMT17872.1 hypothetical protein BVRB_2g034040 isoform A [Beta vulgaris subsp. vulgaris]	gi|380863127|gb|JF815490.1|:Dimocarpus longan clone Unigene66981 spermidine synthase mRNA, complete cds	A0A0J8CW98|A0A0J8CW98_BETVU: Uncharacterized protein;OS=Beta vulgaris subsp. vulgaris.;|GO:0003824,
DN117982_c4_g2_i2	UP	[GO:0046872:metal ion binding]	-	AHYPO_019266-RA:Amaranthus hypochondriacus|C3H|C3H family protein	XP_021852534.1:zinc finger CCCH domaincontaining protein 20like [Spinacia oleracea] >KNA18312.1 hypothetical protein SOVF_072110 [Spinacia oleracea]	gi|332656411|gb|CP002687.1|:Arabidopsis thaliana chromosome 4, complete genome	A0A0K9RHJ2|A0A0K9RHJ2_SPIOL:Uncharacterized protein;OS=Spinacia oleracea;|GO:0046872,

geneid	trend	GO	Pathway	TF	NR	NT	Uniprot
DN118429_c6_g1_i4	UP	[GO:0005634:nucleus][GO:0000989:obsolete transcription factor activity, transcription factor binding][GO:0006355:regulation of transcription, DNA-templated][GO:0006351:transcription, DNA-templated]	-	orange1.1g011736m:Citrus sinensis|GRAS|GRAS family protein	KDO83224.1:hypothetical protein CISIN_1g011736mg [Citrus sinensis]	-	A0A067GUE9|A0A067GUE9_CITSI:Uncharacterized protein;OS=Citrus sinensis;|GO:0005634,|GO:0000989,|GO:0006355,GO:0006351,
DN123877_c2_g4_i1	UP	[GO:0046872:metal ion binding]	-	Sp_072110_mwiu.t1:Spinacia oleracea|C3H|C3H family protein	XP_021852534.1:zinc finger CCCH domaincontaining protein 20like [Spinacia oleracea] >KNA18312.1 hypothetical protein SOVF_072110 [Spinacia oleracea]	-	A0A0K9RHJ2|A0A0K9RHJ2_SPIOL:Uncharacterized protein;OS=Spinacia oleracea;|GO:0046872,
DN126798_c1_g1_i1	UP	-	-	cra_locus_5362_iso_4:Catharanthus roseus|MYB_related|MYB_related family protein	CBI36582.3:unnamed protein product, partial [Vitis vinifera]	gi|359484808|ref|XM_003633119.1|:PREDICTED: Vitis vinifera uncharacterized LOC100257033 (LOC100257033),	F6HYM3|F6HYM3_VITVI:Putative uncharacterized protein;OS=Vitis vinifera;GN=VIT_11s0037g00840 {ECO:0000313|EM

geneid	trend	GO	Pathway	TF	NR	NT	Uniprot
						mRNA	BL:CCB59787.1};
DN120936_c3_g9_i3	UP	[GO:0005634:nucleus][GO:0003677:DNA binding][GO:0006355:regulation of transcription, DNA-templated][GO:0006351:transcription, DNA-templated]	-	Sp_054400_ncjx.t1: Spinacia oleracea|NAC|NAC family protein	XP_021720834.1:NAC domaincontaining protein 35like [Chenopodium quinoa]	gi|32982298|dbj|AK072275.1|:Oryza sativa Japonica Group cDNA clone:J023012F04, full insert sequence	A0A0K9RMX9|A0A0K9RMX9_SPIOL:Uncharacterized protein;OS=Spinacia oleracea;|GO:0005634,|GO:0003677,|GO:0006355,GO:0006351,
DN117925_c0_g1_i4	UP	[GO:0003700:DNA-binding transcription factor activity]	[ko04612:Antigen processing and presentation][ko05152:Tuberculosis]	Sp_083180_dggx.t1: Spinacia oleracea|NF-YA|NF-YA family protein	XP_021859872.1:nuclear transcription factor Y subunit A10like isoform X2 [Spinacia oleracea] >KNA17105.1 hypothetical protein SOVF_083180 isoform A [Spinacia oleracea]	-	A0A0K9RCB1|A0A0K9RCB1_SPIOL: Uncharacterized protein;OS=Spinacia oleracea;|GO:0003700,

geneid	trend	GO	Pathway	TF	NR	NT	Uniprot
DN128533_c0_g1_i2	UP	-	-	Bv6_145140_chao.t1:Beta vulgaris|Nin-like|Nin-like family protein	XP_010682023.1:PREDICTED: protein NLP9 [Beta vulgaris subsp. vulgaris] >KMT07949.1 hypothetical protein BVRB_6g145140 [Beta vulgaris subsp. vulgaris]	-	A0A0J8C6U7|A0A0J8C6U7_BETVU:Uncharacterized protein;OS=Beta vulgaris subsp. vulgaris.;
DN80842_c0_g1_i2	UP	-	-	-	-	-	-
DN119031_c8_g4_i1	UP	-	[ko00480:Glutathione metabolism][ko00980:Metabolism of xenobiotics by cytochrome P450][ko00982:Drug metabolism - cytochrome P450][ko01524:Platinum drug resistance][ko05204:Chemical carcinogenesis][ko0	-	XP_021769783.1:probable glutathione Stransferase parC [Chenopodium quinoa]	-	M5X2D7|M5X2D7_PRUPE:Uncharacterized protein;OS=Prunus persica;

geneid	trend	GO	Pathway	TF	NR	NT	Uniprot
			5418:Fluid shear stress and atherosclerosis]				
DN123696_c 5_g4_i1	UP	-	-	-	-	-	-
DN115056_c 0_g1_i2	UP	-	-	-	XP_010690973.1:P REDICTED: uncharacterized protein LOC104904428 [Beta vulgaris subsp. vulgaris] >KMT00526.1 hypothetical protein BVRB_9g218230 [Beta vulgaris subsp.	-	A0A0J8EBX7|A0A0 J8EBX7_BETVU:U ncharacterized protein;OS=Beta vulgaris subsp. vulgaris.;

geneid	trend	GO	Pathway	TF	NR	NT	Uniprot
					vulgaris]		
DN128727_c3_g2_i2	UP	[GO:0004553:hydrolase activity, hydrolyzing O-glycosyl compounds][GO:0005975:carbohydrate metabolic process]	-	Itr_sc004833.1_g00001.1:Ipomoea trifida|FAR1|FAR1 family protein	XP_010692567.1:PREDICTED: glucan endo1,3betaglucosidase [Beta vulgaris subsp. vulgaris] >KMS99763.1 hypothetical protein BVRB_1g021260 [Beta vulgaris subsp. vulgaris]	gi|109809726|dbj|AB242265.2|:Sesbania rostrata SrGLU3 mRNA for beta-1,3-glucanase, complete cds	A0A0J8BIE7|A0A0J8BIE7_BETVU:Uncharacterized protein;OS=Beta vulgaris subsp. vulgaris.;|GO:0004553,|GO:0005975,

geneid	trend	GO	Pathway	TF	NR	NT	Uniprot
DN116058_c3_g3_i1	UP	[GO:0046872:metal ion binding][GO:0016702:oxidoreductase activity, acting on single donors with incorporation of molecular oxygen, incorporation of two atoms of oxygen][GO:0006633:fatty acid biosynthetic process][GO:0031408:oxylipin biosynthetic process]	[ko00591:Linoleic acid metabolism][ko00592:alpha-Linolenic acid metabolism]	Tp57577_TGAC_v2_mRNA8161:Trifolium pratense|MYB_related|MYB_related family protein	XP_021772004.1:linoleate 13Slipoxygenase 21, chloroplasticlike [Chenopodium quinoa]	-	A0A0K9S108|A0A0K9S108_SPIOL:Lipoxygenase;OS=Spinacia oleracea;|GO:0046872,GO:0016702,|GO:0006633,GO:0031408,
DN124150_c2_g2_i4	UP	[GO:0016788:hydrolase activity, acting on ester bonds]	-	-	XP_021720400.1:GDSL esterase/lipase 2like [Chenopodium quinoa]	gi|31616512|gb|AY299194.1|:Chenopodium rubrum GDSL-lipase mRNA, complete cds	A0A0J8BXN2|A0A0J8BXN2_BETVU: Uncharacterized protein;OS=Beta vulgaris subsp. vulgaris.;|GO:0016788,

geneid	trend	GO	Pathway	TF	NR	NT	Uniprot
DN111712_c0_g1_i1	UP	[GO:0005524:ATP binding][GO:0004672:protein kinase activity]	[ko04064:NF-kappa B signaling pathway][ko04620:Toll-like receptor signaling pathway][ko04621:NOD-like receptor signaling pathway][ko04624:Toll and Imd signaling pathway][ko04722:Neurotrophin signaling pathway][ko05133:Pertussis][ko05140:Leishmaniasis][ko05142:Chagas disease (American trypanosomiasis)][ko05145:Toxoplasmosis][ko05152:Tuberculosis][ko05162:Measles][ko05164:Influenza A]	XP_016443488.1:Nicotiana tabacum|bHLH|bHLH family protein	XP_009588466.1:PREDICTED: rust resistance kinase Lr10like isoform X1 [Nicotiana tomentosiformis]	gi|514775065|ref|XM_004968083.1|:PREDICTED: Setaria italica probable receptor-like protein kinase At1g67000-like (LOC101753271), transcript variant X2, mRNA	M1B3R3|M1B3R3_SOLTU:Uncharacterized protein;OS=Solanum tuberosum;|GO:0005524,GO:0004672,

geneid	trend	GO	Pathway	TF	NR	NT	Uniprot
DN114192_c0_g1_i13	UP	[GO:0020037:heme binding][GO:0004601:peroxidase activity][GO:0006979:response to oxidative stress]	[ko00053:Ascorbate and aldarate metabolism][ko00480:Glutathione metabolism]	-	XP_021743586.1:thylakoid lumenal 29 kDa protein, chloroplasticlike [Chenopodium quinoa]	-	A0A0K9QG33|A0A0K9QG33_SPIOL:Uncharacterized protein;OS=Spinacia oleracea;|GO:0020037,GO:0004601,|GO:0006979,
DN123046_c0_g1_i3	UP	-	-	Dca61174.1:Dianthus caryophyllus|bHLH|bHLH family protein	XP_010695991.1:PREDICTED: protein SIEVE ELEMENT OCCLUSION B [Beta vulgaris subsp. vulgaris] >KMS97262.1 hypothetical protein BVRB_7g177090 isoform A [Beta vulgaris subsp. vulgaris]	gi|521507504|gb|GAHZ01023416.1|:TSA: Salicornia europaea Unigene25200_SeCKS transcribed RNA sequence	A0A0J8BBC2|A0A0J8BBC2_BETVU:Uncharacterized protein;OS=Beta vulgaris subsp. vulgaris.;
DN126024_c8_g13_i6	UP	-	-	-	-	-	-
DN108605_c0_g2_i1	UP	-	-	-	-	-	-

geneid	trend	GO	Pathway	TF	NR	NT	Uniprot
DN125254_c0_g3_i1	UP	[GO:0009941:chloroplast envelope][GO:0009535:chloroplast thylakoid membrane][GO:0005524:ATP binding][GO:0004176:ATP-dependent peptidase activity][GO:0004222:metalloendopeptidase activity][GO:0008237:metallopeptidase activity][GO:0010205:photoinhibition][GO:0010206:photosystem II repair][GO:0006508:proteolysis][GO:0010304:PSII associated light-harvesting complex II catabolic process][GO:0010027:thylakoid membrane organization]	-	-	XP_021742006.1:ATPdependent zinc metalloprotease FTSH 6, chloroplasticlike [Chenopodium quinoa]	-	F6H5S6|F6H5S6_VITVI:Putative uncharacterized protein;OS=Vitis vinifera;GN=VIT_14s0108g00590 {ECO:0000313|EMBL:CCB47547.1};|GO:0009941,GO:0009535,|GO:0005524,GO:0004176,GO:0004222,GO:0008237,|GO:0010205,GO:0010206,GO:0006508,GO:0010304,GO:0010027,

geneid	trend	GO	Pathway	TF	NR	NT	Uniprot
DN111881_c0_g1_i1	UP	-	-	-	XP_002274217.1:PREDICTED: uncharacterized protein LOC100258720 [Vitis vinifera] >CAN68427.1 hypothetical protein VITISV_031997 [Vitis vinifera] >CBI28296.3 unnamed protein product, partial [Vitis vinifera]	-	T1DFA9|T1DFA9_SILLA:Uncharacterized protein;OS=Silene latifolia;
DN120141_c6_g2_i19	UP	[GO:0003676:nucleic acid binding][GO:0000166:nucleotide binding]	[ko03040:Spliceosome]	Csa08g011330.1:Camelina sativa|ZF-HD|ZF-HD family protein	KZV21167.1:serine/argininerich splicing factor 33 [Dorcoceras hygrometricum]	-	A0A0D2TA21|A0A0D2TA21_GOSRA: Uncharacterized protein;OS=Gossypium raimondii;|GO:0003676,GO:0000166,

geneid	trend	GO	Pathway	TF	NR	NT	Uniprot
DN117163_c0_g3_i2	UP	-	-	-	XP_010694569.1:PREDICTED: hemebinding protein 2 [Beta vulgaris subsp. vulgaris] >KMS98270.1 hypothetical protein BVRB_4g094190 [Beta vulgaris subsp. vulgaris]	-	A0A0J8E522|A0A0J8E522_BETVU:Uncharacterized protein;OS=Beta vulgaris subsp. vulgaris.;
DN125678_c1_g1_i2	UP	[GO:0016021:integral component of membrane][GO:0005215:transporter activity]	-	Gh_D11G2978:Gossypium hirsutum|STAT|STAT family protein	XP_021765723.1:probable purine permease 11 [Chenopodium quinoa]	gi|147854852|emb|AM444518.2|:Vitis vinifera contig VV78X022735.4, whole genome shotgun sequence	A0A0J8F705|A0A0J8F705_BETVU:Uncharacterized protein;OS=Beta vulgaris subsp. vulgaris.;|GO:0016021,|GO:0005215,
DN127229_c1_g2_i3	UP	-	-	-	-	-	-
DN114143_c3_g13_i16	UP	-	-	-	XP_019242573.1:PREDICTED: uncharacterized protein LOC109222707	-	F4NCG1|F4NCG1_BETVU:Uncharacterized protein;OS=Beta vulgaris subsp.

geneid	trend	GO	Pathway	TF	NR	NT	Uniprot
					[Nicotiana attenuata]		vulgaris.;
DN112702_c0_g3_i1	UP	-	-	Rsa1.0_00424.1_g00007.1:Raphanus sativus|C2H2|C2H2 family protein	XP_010675525.1:PREDICTED: uncharacterized protein LOC104891516 [Beta vulgaris subsp. vulgaris]	-	A0A0J8D6A8|A0A0J8D6A8_BETVU:Uncharacterized protein;OS=Beta vulgaris subsp. vulgaris.;
DN119035_c0_g2_i1	UP	[GO:0016021:integral component of membrane][GO:0016491:oxidoreductase activity]	-	-	XP_021721026.1:ferric reduction oxidase 7, chloroplasticlike [Chenopodium quinoa]	gi|521541520|gb|GAHZ01002514.1|:TSA: Salicornia europaea Unigene2598_SeCKS transcribed RNA sequence	A0A0J8C6F4|A0A0J8C6F4_BETVU:Uncharacterized protein;OS=Beta vulgaris subsp. vulgaris.;|GO:0016021,|GO:0016491,
DN113806_c1_g1_i3	UP	[GO:0016021:integral component of membrane]	-	-	XP_021865200.1:uncharacterized protein LOC110803957 [Spinacia oleracea] >KNA15410.1 hypothetical protein SOVF_098010	gi|521711847|gb|GAIA01012166.1|:TSA: Salicornia europaea Unigene12786_Se200S transcribed RNA sequence	A0A0K9R7C7|A0A0K9R7C7_SPIOL:Uncharacterized protein;OS=Spinacia oleracea;|GO:0016021,

geneid	trend	GO	Pathway	TF	NR	NT	Uniprot
					[Spinacia oleracea]		
DN128623_c7_g1_i1	UP	[GO:0008146:sulfotransferase activity]	-	676777512:Sisymbrium irio|ERF|ERF family protein	XP_010672565.1:PREDICTED: cytosolic sulfotransferase 5like [Beta vulgaris subsp. vulgaris]	-	A0A0K9R9P6|A0A0K9R9P6_SPIOL:Sulfotransferase;OS=Spinacia oleracea;|GO:0008146,
DN127185_c1_g1_i2	UP	[GO:0046872:metal ion binding][GO:0016491:oxidoreductase activity]	[ko00270:Cysteine and methionine metabolism]	-	KJB57824.1:hypothetical protein B456_009G182300 [Gossypium raimondii]	gi|385049712|gb|JF426641.1|:Silene dioica isolate dio_Sd449_P1 autosomal locus C1E4 genomic sequence	A0A0D2TLY8|A0A0D2TLY8_GOSRA:Uncharacterized protein;OS=Gossypium raimondii;|GO:0046872,GO:0016491,
DN124826_c1_g6_i2	UP	-	-	mrna13309.1-v1.0-hybrid:Fragaria vesca|FAR1|FAR1 family protein	XP_021765163.1:protein IQDOMAIN 14like [Chenopodium quinoa]	-	A0A0K9RC71|A0A0K9RC71_SPIOL:Uncharacterized protein;OS=Spinacia oleracea;

geneid	trend	GO	Pathway	TF	NR	NT	Uniprot
DN42390_c0_g1_i1	UP	-	-	-	-	-	-
DN125463_c1_g1_i2	UP	-	-	-	-	-	-
DN120279_c5_g1_i4	UP	-	-	-	-	-	-
DN125319_c2_g3_i3	UP	[GO:0043565:sequence-specific DNA binding][GO:0003700:DNA-binding transcription factor activity]	-	Bv8_181200_pssw.t2:Beta vulgaris|WRKY|WRKY family protein	XP_010686241.1:PREDICTED: probable WRKY transcription factor 65 isoform X2 [Beta vulgaris subsp. vulgaris] >KMT04448.1 hypothetical protein BVRB_8g181200 isoform B [Beta vulgaris subsp. vulgaris]	gi|406856213|gb|JX416195.1|:Tamarix hispida WRKY transcription factor 6 (WRKY6) gene, complete cds	A0A0J8BWV5|A0A0J8BWV5_BETVU: Uncharacterized protein;OS=Beta vulgaris subsp. vulgaris.;|GO:0043565,GO:0003700,
DN121032_c3_g1_i2	UP	-	-	-	XP_010685049.1:PREDICTED: uncharacterized protein LOC104899534	-	-

geneid	trend	GO	Pathway	TF	NR	NT	Uniprot
					[Beta vulgaris subsp. vulgaris]		
DN126651_c3_g3_i6	UP	[GO:0008168:methyltransferase activity]	-	-	XP_021852224.1:salicylate carboxymethyltransferaselike [Spinacia oleracea]	-	A0A0J8CSG5|A0A0J8CSG5_BETVU:Uncharacterized protein;OS=Beta vulgaris subsp. vulgaris.;|GO:0008168,
DN117014_c0_g1_i5	UP	[GO:0003824:catalytic activity][GO:0050662:coenzyme binding]	-	-	XP_010255955.1:PREDICTED: chloroplast stemloop binding protein of 41 kDa a, chloroplastic [Nelumbo nucifera]	gi|225436923|ref|XM_002275030.1|:PREDICTED: Vitis vinifera uncharacterized LOC100266662 (LOC100266662), mRNA	A5AWW0|A5AWW0_VITVI:Putative uncharacterized protein;OS=Vitis vinifera;GN=VIT_07s0104g00510 {ECO:0000313|EMBL:CCB56816.1};|GO:0003824,GO:0050662,

geneid	trend	GO	Pathway	TF	NR	NT	Uniprot
DN118025_c0_g1_i1	UP	[GO:0016788:hydrolase activity, acting on ester bonds]	-	Sopen08g002450.1: Solanum pennellii|B3|B3 family protein	XP_006449476.1:hypothetical protein CICLE_v10015707mg [Citrus clementina] >ESR62716.1 hypothetical protein CICLE_v10015707mg [Citrus clementina]	-	V4UK90|V4UK90_9ROSI:Uncharacterized protein;OS=Citrus clementina.;|GO:0016788,
DN95397_c0_g2_i1	UP	-	-	Itr_sc001764.1_g00003.1:Ipomoea trifida|MYB_related|MYB_related family protein	XP_021682122.1:uncharacterized protein LOC110666060 [Hevea brasiliensis]	-	A0A151TZI1|A0A151TZI1_CAJCA:Retrovirus-related Pol polyprotein from transposon TNT 1-94;OS=Cajanus cajan;
DN115321_c0_g1_i4	UP	[GO:0016021:integral component of membrane]	-	-	XP_010695745.1:PREDICTED: uncharacterized protein LOC104908341 [Beta vulgaris subsp. vulgaris]	-	A0A0J8BBY1|A0A0J8BBY1_BETVU: Uncharacterized protein;OS=Beta vulgaris subsp. vulgaris.;|GO:0016021,

geneid	trend	GO	Pathway	TF	NR	NT	Uniprot
DN120528_c1_g2_i6	UP	-	[ko04141:Protein processing in endoplasmic reticulum]	Ote100209250011:Ocimum tenuiflorum|MYB_related|MYB_related family protein	AEM05963.1:chloroplast low molecular weight heat shock protein HSP26.04m [Chenopodium album]	gi|224120951|ref|XM_002318424.1|:Populus trichocarpa predicted protein, mRNA	G0X8X5|G0X8X5_CHEAL:Chloroplast low molecular weight heat shock protein HSP26.04m;OS=Chenopodium album;
DN128682_c2_g3_i2	UP	[GO:0046872:metal ion binding][GO:0016702:oxidoreductase activity, acting on single donors with incorporation of molecular oxygen, incorporation of two atoms of oxygen][GO:0006633:fatty acid biosynthetic process][GO:0031408:oxylipin biosynthetic process]	[ko00591:Linoleic acid metabolism][ko00592:alpha-Linolenic acid metabolism]	-	XP_021772004.1:linoleate 13Slipoxygenase 21, chloroplasticlike [Chenopodium quinoa]	gi|160958637|emb|CU232254.1|:Populus EST from mild drought-stressed leaves	W9QY99|W9QY99_9ROSA:Lipoxygenase;OS=Morus notabilis.;|GO:0046872,GO:0016702,|GO:0006633,GO:0031408,
DN117808_c1_g3_i3	UP	-	-	Dca33329.1:Dianthus caryophyllus|bHLH|bHLH family protein	XP_010678431.1:PREDICTED: transcription factor PIF7 [Beta vulgaris subsp. vulgaris] >KMT10741.1 hypothetical protein BVRB_5g113990	-	A0A0J8F4J9|A0A0J8F4J9_BETVU:Uncharacterized protein;OS=Beta vulgaris subsp. vulgaris.;

geneid	trend	GO	Pathway	TF	NR	NT	Uniprot
					[Beta vulgaris subsp. vulgaris]		
DN116297_c1_g1_i2	UP	-	-	-	-	-	-
DN128682_c2_g5_i4	UP	[GO:0046872:metal ion binding][GO:0016702:oxidoreductase activity, acting on single donors with incorporation of molecular oxygen, incorporation of two atoms of oxygen][GO:0006633:fatty acid biosynthetic process][GO:0031408:oxylipin biosynthetic process]	[ko00591:Linoleic acid metabolism][ko00592:alpha-Linolenic acid metabolism]	Lus10031505:Linum usitatissimum|NF-YA|NF-YA family protein	XP_021772004.1:linoleate 13Slipoxygenase 21, chloroplasticlike [Chenopodium quinoa]	-	A0A0K9S108|A0A0K9S108_SPIOL:Lipoxygenase;OS=Spinacia oleracea;|GO:0046872,GO:0016702,|GO:0006633,GO:0031408,
DN118229_c2_g1_i1	UP	-	[ko04141:Protein processing in endoplasmic reticulum]	Ote100209250011:Ocimum tenuiflorum|MYB_related|MYB_related family protein	XP_015894503.1:PREDICTED: small heat shock protein, chloroplasticlike [Ziziphus jujuba]	-	M1AVE4|M1AVE4_SOLTU:Uncharacterized protein;OS=Solanum tuberosum;

geneid	trend	GO	Pathway	TF	NR	NT	Uniprot
DN123363_c0_g3_i3	UP	-	-	-	XP_021771347.1:glutaminylpeptide cyclotransferaselike isoform X1 [Chenopodium quinoa]	gi|227202639|dbj|AK318678.1|:Arabidopsis thaliana AT4G25720 mRNA, complete cds, clone: RAFL07-93-C21	A0A0K9RG25|A0A0K9RG25_SPIOL:Uncharacterized protein;OS=Spinacia oleracea;
DN122429_c0_g1_i5	UP	[GO:0016021:integral component of membrane][GO:0005524:ATP binding][GO:0004672:protein kinase activity]	[ko04064:NF-kappa B signaling pathway][ko04620:Toll-like receptor signaling pathway][ko04621: NOD-like receptor signaling pathway][ko04624:Toll and Imd signaling pathway][ko04722: Neurotrophin signaling pathway][ko05133:Pertussis][ko05140:Leishmaniasis][ko05142:Chagas disease (American	Do000506.1:Dichanthelium oligosanthes|HD-ZIP|HD-ZIP family protein	XP_021861471.1:LEAF RUST 10 DISEASERESISTANCE LOCUS RECEPTORLIKE PROTEIN KINASElike 2.1 [Spinacia oleracea]	-	A0A0K9RJ85|A0A0K9RJ85_SPIOL:Uncharacterized protein;OS=Spinacia oleracea;|GO:0016021,|GO:0005524,GO:0004672,

geneid	trend	GO	Pathway	TF	NR	NT	Uniprot
			trypanosomiasis)][ko05145:Toxoplasmosis][ko05152:Tuberculosis][ko05162:Measles][ko05164:Influenza A]				
DN105742_c1_g1_i2	UP	[GO:0046872:metal ion binding][GO:0016702:oxidoreductase activity, acting on single donors with incorporation of molecular oxygen, incorporation of two atoms of oxygen]	[ko00591:Linoleic acid metabolism][ko00592:alpha-Linolenic acid metabolism]	-	XP_006445969.1:hypothetical protein CICLE_v10014209mg [Citrus clementina] >ESR59209.1 hypothetical protein CICLE_v10014209mg [Citrus	-	A0A067FC45|A0A067FC45_CITSI:Uncharacterized protein;OS=Citrus sinensis;|GO:0046872,GO:0016702,

geneid	trend	GO	Pathway	TF	NR	NT	Uniprot
					clementina]		
DN127148_c1_g10_i4	UP	[GO:0016021:integral component of membrane][GO:0005506:iron ion binding][GO:0016491:oxidoreductase activity][GO:0006633:fatty acid biosynthetic process]	[ko00073:Cutin, suberine and wax biosynthesis]	-	XP_021756466.1:protein ECERIFERUM 1like isoform X1 [Chenopodium quinoa]	gi|478168577|gb|GAHK01008026.1|:TSA: Jatropha curcas CL3027.Contig1_JC-CK_1A transcribed RNA sequence	A0A0J8BIM5|A0A0J8BIM5_BETVU:Uncharacterized protein;OS=Beta vulgaris subsp. vulgaris.;|GO:0016021,|GO:0005506,GO:0016491,|GO:0006633,

geneid	trend	GO	Pathway	TF	NR	NT	Uniprot
DN123205_c4_g2_i7	UP	[GO:0004721:phosphoprotein phosphatase activity]	[ko03015:mRNA surveillance pathway][ko04013:MAPK signaling pathway - fly][ko04071:Sphingolipid signaling pathway][ko04111:Cell cycle - yeast][ko04113:Meiosis - yeast][ko04114:Oocyte meiosis][ko04136:Autophagy - other][ko04138:Autophagy - yeast][ko04140:Autophagy - animal][ko04151:PI3K-Akt signaling pathway][ko04152:AMPK signaling pathway][ko04261:Adrenergic signaling in cardiomyocytes][ko04350:TGF-beta signaling pathway][ko04390:Hippo signaling	Achn144111:Actinidia chinensis|bHLH|bHLH family protein	XP_004146760.1:PREDICTED: serine/threonineprotein phosphatase PP2A catalytic subunit [Cucumis sativus] >XP_008464795.1 PREDICTED: serine/threonineprotein phosphatase PP2A catalytic subunit [Cucumis melo] >KGN47785.1 hypothetical protein Csa_6G401550 [Cucumis sativus]	gi|357486392|ref|XM_003613436.1|:Medicago truncatula Serine/threonine protein phosphatase (MTR_5g037200) mRNA, complete cds	A0A0A0KDJ6|A0A0A0KDJ6_CUCSA: Serine/threonine-protein phosphatase;OS=Cucumis sativus;|GO:0004721,

geneid	trend	GO	Pathway	TF	NR	NT	Uniprot
DN102146_c0_g1_i1	UP	-	-	Zmw_sc01994.1.g00160.1:Zoysia matrella|ZF-HD|ZF-HD family protein	XP_011099948.1:protein RSI1 [Sesamum indicum]	-	A0A068TZD5|A0A068TZD5_COFCA: Uncharacterized protein;OS=Coffea canephora;
DN120040_c0_g3_i2	UP	-	-	Sp_155820_caso.t1: Spinacia oleracea|TCP|TCP family protein	XP_021860188.1:transcription factor TCP14 [Spinacia oleracea] >KNA09198.1 hypothetical protein SOVF_155820 [Spinacia oleracea]	gi|513910483|gb|GAKM01131363.1|:TSA: Triticum turgidum UCW_Tt-k51_contig_14640 transcribed RNA sequence	A0A0K9QR91|A0A0K9QR91_SPIOL:Uncharacterized protein;OS=Spinacia oleracea;
DN127064_c0_g1_i2	UP	-	-	Dca41372.1:Dianthus caryophyllus|C2H2|C2H2 family protein	KRH55323.1:hypothetical protein GLYMA_06G246200 [Glycine max]	-	A0A0R0JT58|A0A0R0JT58_SOYBN:Uncharacterized protein;OS=Glycine max;
DN127311_c2_g4_i2	UP	[GO:0016491:oxidoreductase activity][GO:0009699:phenylpropanoid biosynthetic process]	-	-	XP_010274481.1:PREDICTED: isoeugenol synthase 1like [Nelumbo nucifera]	-	Q15GI3|IGS1_PETHY:Isoeugenol synthase 1;OS=Petunia hybrida;|GO:0016491,|GO:0009699,

geneid	trend	GO	Pathway	TF	NR	NT	Uniprot
DN122740_c0_g4_i1	UP	-	-	RrC887_p5:Raphanus raphanistrum|G2-like|G2-like family protein	BAW35437.1:Cterminal peptidase [Nepenthes alata]	-	A0A061GC60|A0A061GC60_THECC:Uncharacterized protein;OS=Theobroma cacao;
DN127905_c3_g4_i2	UP	-	-	-	-	-	-
DN126094_c0_g2_i2	UP	-	-	-	XP_021753335.1:filamentlike plant protein 7 [Chenopodium quinoa] >XP_021753336.1 filamentlike plant protein 7 [Chenopodium quinoa]	-	A0A0J8BJK5|A0A0J8BJK5_BETVU:Uncharacterized protein;OS=Beta vulgaris subsp. vulgaris.;
DN120898_c0_g5_i5	UP	[GO:0004190:aspartic-type endopeptidase activity][GO:0006629:lipid metabolic process]	-	PH01000046G0930:Phyllostachys heterocycla|WRKY|WRKY family protein	XP_010694065.1:PREDICTED: cyprosin [Beta vulgaris subsp. vulgaris] >KMS98621.1 hypothetical protein	-	A0A0J8BCL6|A0A0J8BCL6_BETVU:Uncharacterized protein;OS=Beta vulgaris subsp. vulgaris.;|GO:0004190,|GO:0006629,

geneid	trend	GO	Pathway	TF	NR	NT	Uniprot
					BVRB_3g070190 [Beta vulgaris subsp. vulgaris]		
DN124320_c3_g1_i1	UP	-	-	-	XP_010688351.1:PREDICTED: uncharacterized protein LOC104902318 [Beta vulgaris subsp. vulgaris] >KMT03219.1 hypothetical protein BVRB_8g197670 [Beta vulgaris subsp. vulgaris]	-	A0A0J8BT98|A0A0J8BT98_BETVU:Uncharacterized protein;OS=Beta vulgaris subsp. vulgaris.;
DN126024_c8_g13_i3	UP	-	-	-	-	-	-
DN124172_c4_g8_i1	UP	-	-	-	-	-	-

geneid	trend	GO	Pathway	TF	NR	NT	Uniprot
DN127098_c2_g1_i11	UP	[GO:0009507:chloroplast][GO:0005524:ATP binding][GO:0016301:kinase activity][GO:0046872:metal ion binding][GO:0050242:pyruvate, phosphate dikinase activity][GO:0015979:photosynthesis][GO:0006090:pyruvate metabolic process]	[ko00620:Pyruvate metabolism][ko00710:Carbon fixation in photosynthetic organisms][ko00720:Carbon fixation pathways in prokaryotes]	-	Q42910.1:RecName: Full=Pyruvate, phosphate dikinase, chloroplastic; AltName: Full=Pyruvate, orthophosphate dikinase; Flags: Precursor >CAA57872.1 pyruvate,orthophosphate dikinase [Mesembryanthemum crystallinum]	gi|559470|emb|X78347.1|:M.crystallinum PPDK mRNA	Q42910|PPDK_MESCR:Pyruvate, phosphate dikinase, chloroplastic;OS=Mesembryanthemum crystallinum;|GO:0009507,|GO:0005524,GO:0016301,GO:0046872,GO:0050242,|GO:0015979,GO:0006090,
DN111858_c0_g1_i2	UP	[GO:0005622:intracellular][GO:0005886:plasma membrane][GO:0005525:GTP binding][GO:0007264:small GTPase mediated signal transduction]	[ko04144:Endocytosis][ko04961:Endocrine and other factor-regulated calcium reabsorption][ko04962:Vasopressin-regulated water reabsorption][ko04972:Pancreatic secretion]	CCG001517.1:Populus euphratica|bHLH|bHLH family protein	XP_010666947.1:PREDICTED: rasrelated protein RABA5e [Beta vulgaris subsp. vulgaris] >KMS95723.1 hypothetical protein BVRB_005650 [Beta vulgaris subsp. vulgaris]	gi|521522937|gb|GAHZ01012785.1|:TSA: Salicornia europaea Unigene13424_SeCKS transcribed RNA sequence	A0A0J8B6Y7|A0A0J8B6Y7_BETVU:Uncharacterized protein;OS=Beta vulgaris subsp. vulgaris.;|GO:0005622,GO:0005886,|GO:0005525,|GO:0007264,

geneid	trend	GO	Pathway	TF	NR	NT	Uniprot
DN113011_c0_g1_i2	UP	[GO:0004089:carbonate dehydratase activity][GO:0008270:zinc ion binding][GO:0006730:one-carbon metabolic process]	[ko00910:Nitrogen metabolism]	Gh_D13G2412:Gossypium hirsutum|GATA|GATA family protein	XP_008802055.1:PREDICTED: alpha carbonic anhydrase 7like [Phoenix dactylifera]	-	I1LL36|I1LL36_SOYBN:Uncharacterized protein;OS=Glycine max;|GO:0004089,GO:0008270,|GO:0006730,
DN113820_c3_g1_i1	UP	-	-	-	XP_021742161.1:uncharacterized protein LOC110708354 [Chenopodium quinoa]	-	A0A0K9RX66|A0A0K9RX66_SPIOL:Uncharacterized protein;OS=Spinacia oleracea;
DN128931_c5_g2_i2	UP	[GO:0016021:integral component of membrane][GO:0005524:ATP binding][GO:0004672:protein kinase activity]	-	-	XP_021756550.1:putative receptorlike protein kinase At4g00960 isoform X1 [Chenopodium quinoa]	-	A0A0L9U167|A0A0L9U167_PHAAN:Uncharacterized protein;OS=Phaseolus angularis;|GO:0016021,|GO:0005524,GO:0004672,

geneid	trend	GO	Pathway	TF	NR	NT	Uniprot
DN128682_c2_g1_i1	UP	[GO:0046872:metal ion binding][GO:0016702:oxidoreductase activity, acting on single donors with incorporation of molecular oxygen, incorporation of two atoms of oxygen][GO:0006633:fatty acid biosynthetic process][GO:0031408:oxylipin biosynthetic process]	[ko00591:Linoleic acid metabolism][ko00592:alpha-Linolenic acid metabolism]	Aan016747:Artemisia annua|ERF|ERF family protein	XP_021772004.1:linoleate 13Slipoxygenase 21, chloroplasticlike [Chenopodium quinoa]	gi|326418055|gb|JF304313.1|:Capsicum annuum cultivar TL 1791 putative 13-lipoxygenase (LOX) mRNA, complete cds	A0A0J8FF20|A0A0J8FF20_BETVU:Lipoxygenase;OS=Beta vulgaris subsp. vulgaris.;|GO:0046872,GO:0016702,|GO:0006633,GO:0031408,
DN126986_c3_g3_i3	UP	[GO:0016021:integral component of membrane]	-	-	XP_010672073.1:PREDICTED: protein Oglucosyltransferase 1 isoform X1 [Beta vulgaris subsp. vulgaris] >XP_019103926.1 PREDICTED: protein Oglucosyltransferase 1 isoform X1 [Beta vulgaris subsp. vulgaris] >KMT20626.1 hypothetical protein	gi|224127623|ref|XM_002320084.1|:Populus trichocarpa predicted protein, mRNA	A0A0J8FYA5|A0A0J8FYA5_BETVU:Uncharacterized protein;OS=Beta vulgaris subsp. vulgaris.;|GO:0016021,

geneid	trend	GO	Pathway	TF	NR	NT	Uniprot
					BVRB_1g006040 [Beta vulgaris subsp. vulgaris]		
DN126651_c3_g3_i4	UP	[GO:0008168:methyltransferase activity]	-	-	XP_021733667.1:salicylate carboxymethyltransferaselike [Chenopodium quinoa]	-	A0A0K9RDN0|A0A0K9RDN0_SPIOL: Uncharacterized protein;OS=Spinacia oleracea;|GO:0008168,

geneid	trend	GO	Pathway	TF	NR	NT	Uniprot
DN121311_c2_g1_i5	UP	[GO:0005524:ATP binding][GO:0006457:protein folding][GO:0006950:response to stress]	[ko04141:Protein processing in endoplasmic reticulum][ko04151:PI3K-Akt signaling pathway][ko04217:Necroptosis][ko04612:Antigen processing and presentation][ko04621:NOD-like receptor signaling pathway][ko04626:Plant-pathogen interaction][ko04657:IL-17 signaling pathway][ko04659:Th17 cell differentiation][ko04914:Progesterone-mediated oocyte maturation][ko04915:Estrogen signaling pathway][ko05200:Pathways in	Do001279.1:Dichanthelium oligosanthes|bZIP|bZIP family protein	XP_011076104.1:heat shock protein 83 [Sesamum indicum]	gi|356530817|ref|XM_003533929.1|:PREDICTED: Glycine max heat shock protein 83-like (LOC100811234), mRNA	A0A0J8CQY4|A0A0J8CQY4_BETVU: Uncharacterized protein;OS=Beta vulgaris subsp. vulgaris.;|GO:0005524,|GO:0006457,GO:0006950,

geneid	trend	GO	Pathway	TF	NR	NT	Uniprot
			cancer][ko05215:Prostate cancer][ko05418:Fluid shear stress and atherosclerosis]				

geneid	trend	GO	Pathway	TF	NR	NT	Uniprot
DN128531_c1_g2_i2	UP	-	-	-	XP_002526246.1:PREDICTED: uncharacterized protein sll0103 [Ricinus communis] >EEF36143.1 protein binding protein, putative [Ricinus communis]	-	B9SJS7|B9SJS7_RICCO:Protein binding protein, putative;OS=Ricinus communis;
DN118415_c2_g2_i2	UP	-	-	Rsa1.0_02166.1_g00002.1:Raphanus sativus|ERF|ERF family protein	XP_010691138.1:PREDICTED: protein LURPonerelated 12 [Beta vulgaris subsp. vulgaris] >KMT00666.1 hypothetical protein BVRB_9g219560 [Beta vulgaris subsp. vulgaris]	-	A0A0J8ECC3|A0A0J8ECC3_BETVU:Uncharacterized protein;OS=Beta vulgaris subsp. vulgaris.;

geneid	trend	GO	Pathway	TF	NR	NT	Uniprot
DN113031_c0_g1_i2	UP	[GO:0019898:extrinsic component of membrane][GO:0009654:photosystem II oxygen evolving complex][GO:0005509:calcium ion binding][GO:0015979:photosynthesis]	[ko00195:Photosynthesis]	-	XP_010683142.1:PREDICTED: photosynthetic NDH subunit of lumenal location 2, chloroplastic [Beta vulgaris subsp. vulgaris]	-	A0A0K9RS47|A0A0K9RS47_SPIOL:Uncharacterized protein;OS=Spinacia oleracea;|GO:0019898,GO:0009654,|GO:0005509,|GO:0015979,
DN128522_c4_g1_i1	UP	[GO:0016021:integral component of membrane][GO:0016491:oxidoreductase activity]	-	-	KNA10876.1:hypothetical protein SOVF_140320 [Spinacia oleracea]	gi|521541520|gb|GAHZ01002514.1|:TSA: Salicornia europaea Unigene2598_SeCKS transcribed RNA sequence	A0A0K9QWG6|A0A0K9QWG6_SPIOL:Uncharacterized protein;OS=Spinacia oleracea;|GO:0016021,|GO:0016491,
DN127195_c2_g3_i9	UP	[GO:0046872:metal ion binding]	-	-	XP_020267927.1:uncharacterized protein LOC109843404 [Asparagus officinalis] >ONK68537.1 uncharacterized protein A4U43_C05F12970 [Asparagus officinalis]	-	A0A0K9Q741|A0A0K9Q741_SPIOL:Uncharacterized protein;OS=Spinacia oleracea;|GO:0046872,

geneid	trend	GO	Pathway	TF	NR	NT	Uniprot
DN118188_c1_g1_i3	UP	[GO:0046872:metal ion binding][GO:0030001:metal ion transport]	-	-	XP_021754236.1:heavy metalassociated isoprenylated plant protein 39like [Chenopodium quinoa]	-	A0A0K9RME2|A0A0K9RME2_SPIOL: Uncharacterized protein;OS=Spinacia oleracea;|GO:0046872,|GO:0030001,
DN124071_c2_g3_i2	UP	[GO:0003676:nucleic acid binding][GO:0008270:zinc ion binding][GO:0015074:DNA integration]	-	Sme2.5_09905.1_g00002.1:Solanum melongena|bHLH|bHLH family protein	XP_021844840.1:uncharacterized protein LOC110784696 [Spinacia oleracea]	-	A5B4M9|A5B4M9_VITVI:Putative uncharacterized protein;OS=Vitis vinifera;|GO:0003676,GO:0008270,|GO:0015074,
DN118553_c4_g1_i3	UP	-	-	-	XP_010687040.1:PREDICTED: NADH dehydrogenase [ubiquinone] 1 alpha subcomplex assembly factor 3 [Beta vulgaris subsp. vulgaris] >KMT03951.1 hypothetical protein BVRB_8g187600 [Beta vulgaris subsp.	-	A0A0J8ELS5|A0A0J8ELS5_BETVU:Uncharacterized protein;OS=Beta vulgaris subsp. vulgaris.;

geneid	trend	GO	Pathway	TF	NR	NT	Uniprot
					vulgaris]		
DN126024_c8_g13_i5	UP	-	-	-	-	-	-
DN127098_c2_g1_i19	UP	[GO:0009507:chloroplast][GO:0005524:ATP binding][GO:0016301:kinase activity][GO:0046872:metal ion binding][GO:0050242:pyruvate, phosphate dikinase activity][GO:0015979:photosynthesis][GO:0006090:pyruvate metabolic process]	[ko00620:Pyruvate metabolism][ko00710:Carbon fixation in photosynthetic organisms][ko00720:Carbon fixation pathways in prokaryotes]	-	Q42910.1:RecName: Full=Pyruvate, phosphate dikinase, chloroplastic; AltName: Full=Pyruvate, orthophosphate dikinase; Flags: Precursor >CAA57872.1 pyruvate,orthophosphate dikinase [Mesembryanthemu	gi|559470|emb|X78347.1|:M.crystallinum PPDK mRNA	Q42910|PPDK_MESCR:Pyruvate, phosphate dikinase, chloroplastic;OS=Mesembryanthemum crystallinum;|GO:0009507,|GO:0005524,GO:0016301,GO:0046872,GO:0050242,|GO:0015979,GO:0006090,

geneid	trend	GO	Pathway	TF	NR	NT	Uniprot
					m crystallinum]		
DN118206_c 1_g1_i1	UP	-	-	-	KMT08561.1:hypothetical protein BVRB_6g138410 [Beta vulgaris subsp. vulgaris]	-	A0A0K9RWY3|A0A0K9RWY3_SPIOL:Uncharacterized protein;OS=Spinacia oleracea;
DN121695_c 1_g1_i5	UP	-	-	Sp_143900_sark.t1: Spinacia oleracea|bHLH|bHLH family protein	XP_021836627.1:transcription factor bHLH130like [Spinacia oleracea] >XP_021840406.1 transcription factor bHLH130like [Spinacia oleracea] >KNA10511.1	-	A0A0K9QVG3|A0A0K9QVG3_SPIOL: Uncharacterized protein;OS=Spinacia oleracea;

geneid	trend	GO	Pathway	TF	NR	NT	Uniprot
					hypothetical protein SOVF_143900 [Spinacia oleracea]		
DN118429_c6_g1_i2	UP	[GO:0005634:nucleus][GO:0000989:obsolete transcription factor activity, transcription factor binding][GO:0006355:regulation of transcription, DNA-templated][GO:0006351:transcription, DNA-templated]	-	orange1.1g011736m:Citrus sinensis|GRAS|GRAS family protein	KDO83224.1:hypothetical protein CISIN_1g011736mg [Citrus sinensis]	-	A0A067GUE9|A0A067GUE9_CITSI:Uncharacterized protein;OS=Citrus sinensis;|GO:0005634,|GO:0000989,|GO:0006355,GO:0006351,

geneid	trend	GO	Pathway	TF	NR	NT	Uniprot
DN115893_c4_g1_i5	UP	[GO:0016021:integral component of membrane]	-	-	XP_010667529.1:PREDICTED: uncharacterized protein LOC104884553 [Beta vulgaris subsp. vulgaris] >KMS95320.1 hypothetical protein BVRB_009370 [Beta vulgaris subsp. vulgaris]	-	A0A0J8DWT3|A0A0J8DWT3_BETVU: Uncharacterized protein;OS=Beta vulgaris subsp. vulgaris.;|GO:0016021,
DN122191_c7_g1_i3	UP	-	-	-	XP_015899497.1:PREDICTED: uncharacterized protein LOC107432813 [Ziziphus jujuba]	-	A0A0J8BT98|A0A0J8BT98_BETVU:Uncharacterized protein;OS=Beta vulgaris subsp. vulgaris.;
DN114897_c0_g5_i1	UP	-	-	-	-	-	-

geneid	trend	GO	Pathway	TF	NR	NT	Uniprot
DN120948_c4_g2_i1	UP	-	-	-	XP_010677359.1:PREDICTED: rhodaneselike domaincontaining protein 10 [Beta vulgaris subsp. vulgaris] >KMT11356.1 hypothetical protein BVRB_5g106890 [Beta vulgaris subsp. vulgaris]	-	A0A0J8F5W5|A0A0J8F5W5_BETVU:Uncharacterized protein;OS=Beta vulgaris subsp. vulgaris.;
DN127497_c3_g1_i1	UP	[GO:0006457:protein folding]	-	-	XP_009777144.1:PREDICTED: peptidylprolyl cistrans isomerase FKBP13, chloroplastic [Nicotiana sylvestris] >XP_016509102.1 PREDICTED: peptidylprolyl cistrans isomerase FKBP13,	gi|460397889|ref|XM_004244450.1|:PREDICTED: Solanum lycopersicum peptidyl-prolyl cis-trans isomerase FKBP13, chloroplastic-like, transcript variant 1 (LOC101268705), mRNA	A0A0F7H0E7|A0A0F7H0E7_9ROSI:FK506 binding protein 13;OS=Erodium gruinum.;|GO:0006457,

geneid	trend	GO	Pathway	TF	NR	NT	Uniprot
					chloroplasticlike [Nicotiana tabacum]		
DN105666_c 1_g1_i1	UP	[GO:0003993:acid phosphatase activity]	-	GSMUA_Achr8P25 970_001:Musa acuminata|MYB|MY B family protein	XP_021860433.1:aci d phosphatase 1like [Spinacia oleracea] >KNA20723.1 hypothetical protein SOVF_049830 [Spinacia oleracea]	-	A0A0K9RML3|A0A 0K9RML3_SPIOL: Uncharacterized protein;OS=Spinacia oleracea;|GO:00039 93,
DN122016_c 5_g5_i1	UP	-	-	-	-	-	-
DN123338_c 1_g3_i1	UP	-	-	-	-	-	-
DN126563_c 0_g2_i1	UP	-	-	-	OWM65075.1:hypot hetical protein CDL15_Pgr028793 [Punica granatum]	-	A0A151S6I0|A0A15 1S6I0_CAJCA:Unc haracterized protein;OS=Cajanus

geneid	trend	GO	Pathway	TF	NR	NT	Uniprot
							cajan;
DN118165_c2_g6_i4	UP	[GO:0016021:integral component of membrane][GO:0008270:zinc ion binding]	-	XP_016459247.1:Nicotiana tabacum|C2H2|C2H2 family protein	XP_021768768.1:uncharacterized protein LOC110733056 isoform X1 [Chenopodium quinoa]	-	A0A0J8FGV2|A0A0J8FGV2_BETVU:Uncharacterized protein;OS=Beta vulgaris subsp. vulgaris.;|GO:0016021,|GO:0008270,
DN128218_c2_g1_i6	UP	[GO:0005788:endoplasmic reticulum lumen][GO:0009506:plasmodesma][GO:0016491:oxidoreductase activity][GO:0009860:pollen tube growth][GO:0009408:response to heat]	-	Achn036321:Actinidia chinensis|G2-like|G2-like family protein	XP_010676784.2:PREDICTED: dnaJ protein ERDJ3A [Beta vulgaris subsp. vulgaris]	-	A0A0J8CFP2|A0A0J8CFP2_BETVU:Uncharacterized protein;OS=Beta vulgaris subsp. vulgaris.;|GO:0005788,GO:0009506,|GO:0016491,|GO:0009860,GO:0009408,
DN128498_c3_g6_i3	UP	[GO:0005524:ATP binding][GO:0004672:protein kinase activity]	-	-	KNA03362.1:hypothetical protein SOVF_209960 [Spinacia oleracea]	-	A0A0K9Q7W1|A0A0K9Q7W1_SPIOL:Uncharacterized protein;OS=Spinacia oleracea;|GO:00055

geneid	trend	GO	Pathway	TF	NR	NT	Uniprot
							24,GO:0004672,
DN122808_c1_g2_i4	UP	[GO:0016021:integral component of membrane][GO:0005215:transporter activity][GO:0006814:sodium ion transport][GO:0055085:transmembrane transport]	-	Zmw_sc06488.1.g00020.1:Zoysia matrella|C3H|C3H family protein	XP_010690536.1:PREDICTED: dicarboxylate transporter 2, chloroplastic [Beta vulgaris subsp. vulgaris] >KMT01551.1 hypothetical protein BVRB_9g215520 [Beta vulgaris subsp. vulgaris]	gi|521577449|gb|GAIA01070380.1|:TSA: Salicornia europaea Unigene84693_Se200S transcribed RNA sequence	A0A0J8EF09|A0A0J8EF09_BETVU:Uncharacterized protein;OS=Beta vulgaris subsp. vulgaris.;|GO:0016021,|GO:0005215,|GO:0006814,GO:0055085,
DN120279_c5_g1_i1	UP	-	-	-	-	-	-

geneid	trend	GO	Pathway	TF	NR	NT	Uniprot
DN123920_c0_g1_i1	UP	[GO:0016021:integral component of membrane][GO:0005524:ATP binding][GO:0042626:ATPase activity, coupled to transmembrane movement of substances]	[ko01523:Antifolate resistance][ko01524:Platinum drug resistance][ko02010:ABC transporters][ko04976:Bile secretion]	RrC2480_p5:Raphanus raphanistrum|HSF|HSF family protein	XP_021652403.1:ABC transporter C family member 10 [Hevea brasiliensis]	gi|255545089|ref|XM_002513560.1|:Ricinus communis multidrug resistance-associated protein 1, 3 (mrp1, 3), abc-transoprter, putative, mRNA	A0A0K9S0L2|A0A0K9S0L2_SPIOL:Uncharacterized protein;OS=Spinacia oleracea;|GO:0016021,|GO:0005524,GO:0042626,
DN115999_c0_g1_i1	UP	[GO:0016021:integral component of membrane][GO:0005524:ATP binding][GO:0004672:protein kinase activity]	-	XP_016443488.1:Nicotiana tabacum|bHLH|bHLH family protein	XP_021749488.1:serine/threonineprotein kinaselike protein CCR1 [Chenopodium quinoa]	gi|521509820|gb|GAHZ01021100.1|:TSA: Salicornia europaea Unigene22328_SeCKS transcribed RNA sequence	A0A0J8DV52|A0A0J8DV52_BETVU:Uncharacterized protein;OS=Beta vulgaris subsp. vulgaris.;|GO:0016021,|GO:0005524,GO:0004672,
DN111711_c1_g1_i6	UP	-	-	-	XP_021769373.1:gibberellinregulated protein 1like [Chenopodium quinoa]	gi|521738633|gb|GAIA01007484.1|:TSA: Salicornia europaea Unigene7799_Se200S transcribed RNA sequence	A0A0J8BFU2|A0A0J8BFU2_BETVU:Uncharacterized protein;OS=Beta vulgaris subsp. vulgaris.;

geneid	trend	GO	Pathway	TF	NR	NT	Uniprot
DN120286_c3_g2_i2	UP	[GO:0009317:acetyl-CoA carboxylase complex][GO:0009570:chloroplast stroma][GO:0003989:acetyl-CoA carboxylase activity][GO:0005524:ATP binding][GO:0016740:transferase activity][GO:0008270:zinc ion binding][GO:0006633:fatty acid biosynthetic process][GO:2001295:malonyl-CoA biosynthetic process]	[ko00061:Fatty acid biosynthesis][ko00620:Pyruvate metabolism][ko00640:Propanoate metabolism][ko00720:Carbon fixation pathways in prokaryotes]	GSVIVT01010035001:Vitis vinifera|C3H|C3H family protein	YP_009221816.1:acetylCoA carboxylase carboxyltransferase beta subunit (chloroplast) [Mesembryanthemum crystallinum] >AIS35692.1 acetylCoA carboxylase carboxyltransferase beta subunit (chloroplast) [Mesembryanthemum crystallinum]	gi|7636084|emb|AJ400848.1|:Spinacia oleracea complete chloroplast genome	A0A0G2R7Y3|A0A0G2R7Y3_MESCR: Acetyl-coenzyme A carboxylase carboxyl transferase subunit beta, chloroplastic;OS=Mesembryanthemum crystallinum;|GO:0009317,GO:0009570,|GO:0003989,GO:0005524,GO:0016740,GO:0008270,|GO:0006633,GO:2001295,
DN120283_c0_g3_i2	UP	[GO:0005737:cytoplasm][GO:0005524:ATP binding][GO:0046914:transition metal ion binding][GO:1901671:positive regulation of superoxide dismutase activity][GO:0006457:protein folding]	-	LPERR06G05720.2:Leersia perrieri|ARF|ARF family protein	KHN07881.1:20 kDa chaperonin, chloroplastic [Glycine soja]	-	A0A0B2PEQ2|A0A0B2PEQ2_GLYSO: 20 kDa chaperonin, chloroplastic;OS=Glycine soja;|GO:0005737,|GO:0005524,GO:0046914,|GO:1901671,GO:0006457,

geneid	trend	GO	Pathway	TF	NR	NT	Uniprot
DN123338_c1_g5_i11	UP	[GO:0003676:nucleic acid binding][GO:0008270:zinc ion binding][GO:0015074:DNA integration]	-	Rsa1.0_02878.1_g00005.1:Raphanus sativus|LBD|LBD family protein	KYP71150.1:Retrovirusrelated Pol polyprotein from transposon TNT 194 [Cajanus cajan]	-	A0A151TVS9|A0A151TVS9_CAJCA:Retrovirus-related Pol polyprotein from transposon TNT 1-94;OS=Cajanus cajan;|GO:0003676,GO:0008270,|GO:0015074,
DN128302_c8_g3_i2	UP	[GO:0008270:zinc ion binding]	-	KZV46403.1:Dorcoceras hygrometricum|C2H2|C2H2 family protein	XP_002274294.2:PREDICTED: glucan endo1,3betaglucosidase 13 [Vitis vinifera]	-	A0A0D3GMB3|A0A0D3GMB3_9ORYZ:Uncharacterized protein;OS=Oryza barthii.;|GO:0008270,
DN122646_c5_g2_i8	UP	[GO:0009055:electron transfer activity][GO:0051536:iron-sulfur cluster binding]	-	-	XP_021757901.1:photosynthetic NDH subunit of subcomplex B 3, chloroplasticlike [Chenopodium quinoa]	gi|521543980|gb|GAHZ01001439.1|:TSA: Salicornia europaea Unigene1468_SeCKS transcribed RNA sequence	A0A0J8CTG9|A0A0J8CTG9_BETVU:Uncharacterized protein;OS=Beta vulgaris subsp. vulgaris.;|GO:0009055,GO:0051536,

geneid	trend	GO	Pathway	TF	NR	NT	Uniprot
DN114356_c1_g1_i1	UP	[GO:0009941:chloroplast envelope][GO:0030093:chloroplast photosystem I][GO:0009508:plastid chromosome][GO:0009773:photosynthetic electron transport in photosystem I][GO:0009737:response to abscisic acid]	-	-	XP_010688395.1:PREDICTED: protein CURVATURE THYLAKOID 1B, chloroplastic [Beta vulgaris subsp. vulgaris] >KMT20202.1 hypothetical protein BVRB_1g002080 [Beta vulgaris subsp. vulgaris]	-	A0A0J8D349|A0A0J8D349_BETVU:Uncharacterized protein;OS=Beta vulgaris subsp. vulgaris.;|GO:0009941,GO:0030093,GO:0009508,|GO:0009773,GO:0009737,
DN119783_c3_g8_i1	UP	-	[ko04141:Protein processing in endoplasmic reticulum]	Jcr4S16451.20:Jatropha curcas|MYB_related|MYB_related family protein	XP_021731829.1:26.5 kDa heat shock protein, mitochondriallike [Chenopodium quinoa]	-	A0A0J8BI61|A0A0J8BI61_BETVU:Uncharacterized protein;OS=Beta vulgaris subsp. vulgaris.;
DN121864_c3_g6_i3	UP	-	-	-	-	-	-

geneid	trend	GO	Pathway	TF	NR	NT	Uniprot
DN126086_c0_g1_i4	UP	[GO:0020037:heme binding][GO:0051536:iron-sulfur cluster binding][GO:0016491:oxidoreductase activity]	[ko00910:Nitrogen metabolism]	-	XP_021754875.1:ferredoxinnitrite reductase, chloroplasticlike [Chenopodium quinoa]	gi|21257|emb|X07568.1|:Spinach mRNA for nitrite reductase NiR (E.C.1.7.7.1.)	T1RUX3|T1RUX3_SILVU:Ferredoxin--nitrite reductase;OS=Silene vulgaris;|GO:0020037,GO:0051536,GO:0016491,
DN128019_c1_g7_i1	UP	[GO:0046872:metal ion binding][GO:0016491:oxidoreductase activity]	-	Achn004841:Actinidia chinensis|NAC|NAC family protein	XP_010672757.1:PREDICTED: 1aminocyclopropane 1carboxylate oxidase homolog 1 [Beta vulgaris subsp. vulgaris] >KMT15414.1 hypothetical protein BVRB_3g061180 [Beta vulgaris subsp. vulgaris]	-	A0A0J8CTN1|A0A0J8CTN1_BETVU:Uncharacterized protein;OS=Beta vulgaris subsp. vulgaris.;|GO:0046872,GO:0016491,
DN127195_c2_g3_i12	UP	[GO:0046872:metal ion binding]	-	-	XP_020267927.1:uncharacterized protein LOC109843404 [Asparagus officinalis] >ONK68537.1	-	A0A0K9Q741|A0A0K9Q741_SPIOL:Uncharacterized protein;OS=Spinacia oleracea;|GO:0046872,

geneid	trend	GO	Pathway	TF	NR	NT	Uniprot
					uncharacterized protein A4U43_C05F12970 [Asparagus officinalis]		
DN124360_c1_g4_i4	UP	[GO:0016021:integral component of membrane][GO:0003841:1-acylglycerol-3-phosphate O-acyltransferase activity][GO:0008654:phospholipid biosynthetic process]	-	-	XP_002317596.1:hypothetical protein POPTR_0011s14220g [Populus trichocarpa] >EEE98208.1 hypothetical protein POPTR_0011s14220g [Populus trichocarpa]	gi|356521609|ref|XM_003529399.1|:PREDICTED: Glycine max DNA-damage-repair/toleration protein DRT111, chloroplastic-like (LOC100805487), mRNA	B9I0E6|B9I0E6_POPTR:1-acyl-sn-glycerol-3-phosphate acyltransferase;OS=Populus trichocarpa;|GO:0016021,|GO:0003841,|GO:0008654,
DN120734_c2_g1_i2	UP	[GO:0003677:DNA binding]	-	Thecc1EG017336t1:Theobroma cacao|bHLH|bHLH family protein	XP_007032008.2:PREDICTED: transcription factor ICE1 [Theobroma cacao]	gi|521448215|gb|GAHZ01072269.1|:TSA: Salicornia europaea Unigene87998_SeCKS transcribed RNA sequence	A0A061EKV1|A0A061EKV1_THECC:Basic helix-loop-helix DNA-binding superfamily protein, putative isoform 1;OS=Theobroma cacao;|GO:0003677,

geneid	trend	GO	Pathway	TF	NR	NT	Uniprot
DN117208_c0_g2_i3	UP	[GO:0048046:apoplast][GO:0009941:chloroplast envelope][GO:0016020:membrane][GO:0005777:peroxisome][GO:0009506:plasmodesma][GO:0010287:plastoglobule][GO:0005840:ribosome][GO:0010319:stromule][GO:0005773:vacuole][GO:0003824:catalytic activity][GO:0050662:coenzyme binding][GO:0010297:heteropolysaccharide binding][GO:0003723:RNA binding][GO:0009658:chloroplast organization][GO:0007623:circadian rhythm][GO:0005996:monosaccharide metabolic process][GO:0032544:plastid translation][GO:0045893:positive regulation of transcription, DNA-templated][GO:0045727:positive regulation of translation][GO:0006364:rRNA processing]	-	-	XP_010674555.1:PREDICTED: chloroplast stemloop binding protein of 41 kDa b, chloroplastic [Beta vulgaris subsp. vulgaris] >KMT14184.1 hypothetical protein BVRB_4g080010 [Beta vulgaris subsp. vulgaris]	gi|521650915|gb|GAIA01041588.1|:TSA: Salicornia europaea Unigene46723_Se200S transcribed RNA sequence	A0A0J8CQA7|A0A0J8CQA7_BETVU: Uncharacterized protein;OS=Beta vulgaris subsp. vulgaris.;|GO:0048046,GO:0009941,GO:0016020,GO:0005777,GO:0009506,GO:0010287,GO:0005840,GO:0010319,GO:0005773,|GO:0003824,GO:0050662,GO:0010297,GO:0003723,|GO:0009658,GO:0007623,GO:0005996,GO:0032544,GO:0045893,GO:0045727,GO:0006364,

geneid	trend	GO	Pathway	TF	NR	NT	Uniprot
DN106235_c1_g1_i1	UP	-	-	-	-	-	-
DN122831_c0_g1_i2	UP	-	-	-	KNA12742.1:hypothetical protein SOVF_123180 [Spinacia oleracea]	-	A0A0K9R1S1|A0A0K9R1S1_SPIOL:Uncharacterized protein;OS=Spinacia oleracea;
DN127229_c1_g2_i1	UP	-	-	-	-	-	-
DN123875_c1_g1_i2	UP	[GO:0004857:enzyme inhibitor activity]	-	-	CAN70288.1:hypothetical protein VITISV_015784 [Vitis vinifera]	-	A5BLC4|A5BLC4_VITVI:Putative uncharacterized protein;OS=Vitis vinifera;|GO:0004857,
DN129118_c8_g5_i1	UP	[GO:0016021:integral component of membrane]	-	-	XP_021771418.1:receptorlike protein 12 [Chenopodium quinoa]	-	A0A0J8BF03|A0A0J8BF03_BETVU:Uncharacterized protein;OS=Beta vulgaris subsp. vulgaris.;|GO:0016021,

geneid	trend	GO	Pathway	TF	NR	NT	Uniprot
DN121341_c0_g1_i4	UP	-	-	-	XP_021716988.1:uncharacterized protein LOC110684854 isoform X2 [Chenopodium quinoa] >XP_021716989.1 uncharacterized protein LOC110684854 isoform X2 [Chenopodium quinoa] >XP_021716990.1 uncharacterized protein LOC110684854 isoform X2 [Chenopodium quinoa]	gi|297823304|ref|XM_002879489.1|:Arabidopsis lyrata subsp. lyrata hypothetical protein, mRNA	-
DN122123_c0_g2_i6	UP	[GO:0016021:integral component of membrane]	-	Medtr0036s0260.1:Medicago truncatula|MYB_related|MYB_related family protein	XP_021776168.1:probable zinc metallopeptidase EGY3, chloroplastic [Chenopodium	gi|521663387|gb|GAIA01035561.1|:TSA: Salicornia europaea Unigene39377_Se200S transcribed RNA	A0A0J8BLU0|A0A0J8BLU0_BETVU:Uncharacterized protein;OS=Beta vulgaris subsp.

geneid	trend	GO	Pathway	TF	NR	NT	Uniprot
					quinoa]	sequence	vulgaris.;|GO:0016021,
DN112255_c0_g1_i2	UP	-	[ko04141:Protein processing in endoplasmic reticulum]	-	XP_021738936.1:17.3 kDa class II heat shock proteinlike [Chenopodium quinoa]	-	A0A0J8FQ33|A0A0J8FQ33_BETVU:Uncharacterized protein;OS=Beta vulgaris subsp. vulgaris.;
DN120547_c0_g1_i1	UP	[GO:0009570:chloroplast stroma][GO:0080041:ADP-ribose pyrophosphohydrolase activity][GO:0051536:iron-sulfur cluster binding][GO:0009228:thiamine biosynthetic process]	[ko00730:Thiamine metabolism]	-	XP_010685602.1:PREDICTED: phosphomethylpyrimidine synthase, chloroplastic isoform X2 [Beta vulgaris subsp. vulgaris] >XP_019106532.1 PREDICTED: phosphomethylpyrimidine synthase, chloroplastic isoform X1 [Beta	gi|521697686|gb|GAIA01018715.1|:TSA: Salicornia europaea Unigene19672_Se200S transcribed RNA sequence	A0A0J8BYS7|A0A0J8BYS7_BETVU:Uncharacterized protein;OS=Beta vulgaris subsp. vulgaris.;|GO:0009570,|GO:0080041,GO:0051536,|GO:0009228,

geneid	trend	GO	Pathway	TF	NR	NT	Uniprot
					vulgaris subsp. vulgaris] >KMT05103.1 hypothetical protein BVRB_7g172570 [Beta vulgaris subsp. vulgaris]		
DN128241_c5_g1_i9	UP	[GO:0005524:ATP binding][GO:0004326:tetrahydrofolylpolyglutamate synthase activity]	[ko00790:Folate biosynthesis][ko01523:Antifolate resistance]	-	XP_021853507.1:folylpolyglutamate synthase isoform X1 [Spinacia oleracea] >KNA20868.1 hypothetical protein SOVF_048480 isoform A [Spinacia oleracea]	gi|261597519|gb|GU011931.1|:Cordia alliodora microsatellite C5_seg109 sequence	A0A0K9RN08|A0A0K9RN08_SPIOL:Uncharacterized protein;OS=Spinacia oleracea;|GO:0005524,GO:0004326,

geneid	trend	GO	Pathway	TF	NR	NT	Uniprot
DN117762_c0_g1_i1	UP	[GO:0016021:integral component of membrane]	-	-	KMT08510.1:hypothetical protein BVRB_6g137950 isoform A [Beta vulgaris subsp. vulgaris]	-	A0A0J8C413|A0A0J8C413_BETVU:Uncharacterized protein;OS=Beta vulgaris subsp. vulgaris.;|GO:0016021,
DN121117_c0_g5_i4	UP	[GO:0005622:intracellular][GO:0008270:zinc ion binding]	-	Sp_021810_twdn.t2:Spinacia oleracea|CO-like|CO-like family protein	XP_021854091.1:zinc finger protein CONSTANSLIKE 2 isoform X1 [Spinacia oleracea] >KNA23804.1 hypothetical protein SOVF_021810 isoform B [Spinacia oleracea]	gi|334847619|gb|EU395770.2|:Chenopodium rubrum CONSTANS-like protein 1 (COL1) mRNA, complete cds, alternatively spliced	A0A0K9RY14|A0A0K9RY14_SPIOL:Uncharacterized protein;OS=Spinacia oleracea;|GO:0005622,|GO:0008270,
DN126024_c8_g3_i1	UP	-	-	-	-	-	-
DN112255_c0_g1_i4	UP	-	[ko04141:Protein processing in endoplasmic reticulum]	-	XP_021738936.1:17.3 kDa class II heat shock proteinlike [Chenopodium quinoa]	-	A0A0J8FQ33|A0A0J8FQ33_BETVU:Uncharacterized protein;OS=Beta vulgaris subsp.

geneid	trend	GO	Pathway	TF	NR	NT	Uniprot
							vulgaris.;
DN127985_c0_g7_i1	UP	[GO:0016021:integral component of membrane][GO:0015299:solute:proton antiporter activity][GO:0006813:potassium ion transport]	-	-	XP_010671923.1:PREDICTED: K(+) efflux antiporter 2, chloroplastic [Beta vulgaris subsp. vulgaris] >KMT20607.1 hypothetical protein BVRB_1g005870 [Beta vulgaris subsp. vulgaris]	gi|224130219|ref|XM_002320746.1|:Populus trichocarpa predicted protein, mRNA	A0A0J8D465|A0A0J8D465_BETVU:Uncharacterized protein;OS=Beta vulgaris subsp. vulgaris.;|GO:0016021,|GO:0015299,|GO:0006813,
DN187712_c0_g2_i1	UP	-	-	-	XP_021739636.1:protein ACCELERATED CELL DEATH 6like [Chenopodium quinoa]	-	A0A0J8CD44|A0A0J8CD44_BETVU:Uncharacterized protein;OS=Beta vulgaris subsp. vulgaris.;

geneid	trend	GO	Pathway	TF	NR	NT	Uniprot
DN125985_c 1_g1_i7	UP	[GO:0016787:hydrolase activity]	-	329122:Arabidopsis lyrata|C3H|C3H family protein	XP_021771392.1:nudix hydrolase 10like [Chenopodium quinoa]	gi|478164044|gb|GAHK01012559.1|:TSA: Jatropha curcas Unigene10_JC-CK_1A transcribed RNA sequence	A0A0J8CGU6|A0A0J8CGU6_BETVU: Uncharacterized protein;OS=Beta vulgaris subsp. vulgaris.;|GO:0016787,
DN118938_c 0_g4_i1	UP	[GO:0005737:cytoplasm][GO:0003677:DNA binding][GO:0004842:ubiquitin-protein transferase activity][GO:0008270:zinc ion binding]	-	Csa15g002520.1:Camelina sativa|C3H|C3H family protein	XP_010667008.1:PREDICTED: E3 ubiquitinprotein ligase ORTHRUS 2 [Beta vulgaris subsp. vulgaris] >KMS95667.1 hypothetical protein BVRB_006020 [Beta vulgaris subsp. vulgaris]	-	A0A0J8B7A2|A0A0J8B7A2_BETVU:Uncharacterized protein;OS=Beta vulgaris subsp. vulgaris.;|GO:0005737,|GO:0003677,GO:0004842,GO:0008270,

geneid	trend	GO	Pathway	TF	NR	NT	Uniprot
DN119942_c0_g1_i3	UP	[GO:0016787:hydrolase activity][GO:0008152:metabolic process]	-	MDP0000298233:Malus domestica|C3H|C3H family protein	XP_021861602.1:haloacid dehalogenaselike hydrolase domaincontaining protein 3 [Spinacia oleracea] >KNA06903.1 hypothetical protein SOVF_176790 [Spinacia oleracea]	gi|502108257|ref|XM_004493371.1|:PREDICTED: Cicer arietinum haloacid dehalogenase-like hydrolase domain-containing protein 3-like (LOC101512920), mRNA	A0A0K9QJM7|A0A0K9QJM7_SPIOL: Uncharacterized protein;OS=Spinacia oleracea;|GO:0016787,|GO:0008152,
DN129129_c11_g7_i1	UP	[GO:0005524:ATP binding][GO:0019538:protein metabolic process]	[ko04213:Longevity regulating pathway - multiple species]	-	XP_021761516.1:chaperone protein ClpB1 [Chenopodium quinoa]	-	A0A0J8E3S6|A0A0J8E3S6_BETVU:Uncharacterized protein;OS=Beta vulgaris subsp. vulgaris.;|GO:0005524,|GO:0019538,
DN114935_c6_g4_i2	UP	[GO:0003676:nucleic acid binding]	-	LOC_Os12g06010.1:Oryza sativa subsp. japonica|bZIP|bZIP family protein	XP_021848094.1:uncharacterized protein LOC110787762 [Spinacia oleracea]	-	F4NCI7|F4NCI7_BETVU:Uncharacterized protein;OS=Beta vulgaris subsp. vulgaris.;|GO:0003676,

geneid	trend	GO	Pathway	TF	NR	NT	Uniprot
DN116167_c2_g2_i2	UP	-	-	-	-	-	-
DN112673_c0_g1_i1	UP	[GO:0008080:N-acetyltransferase activity]	-	-	XP_010670040.1:PREDICTED: probable acetyltransferase NATA1like [Beta vulgaris subsp. vulgaris] >KMT17407.1 hypothetical protein BVRB_2g038620 [Beta vulgaris subsp. vulgaris]	-	A0A0J8CUX5|A0A0J8CUX5_BETVU: Uncharacterized protein;OS=Beta vulgaris subsp. vulgaris.;|GO:0008080,
DN121109_c2_g2_i5	UP	[GO:0046872:metal ion binding][GO:0016491:oxidoreductase activity]	-	Zpz_sc00083.1.g00290.1.am.mk:Zoysia pacifica|Dof|Dof family protein	XP_010672757.1:PREDICTED: 1aminocyclopropane 1carboxylate oxidase homolog 1 [Beta vulgaris subsp. vulgaris] >KMT15414.1 hypothetical protein BVRB_3g061180 [Beta vulgaris subsp.	-	A0A0J8CTN1|A0A0J8CTN1_BETVU:Uncharacterized protein;OS=Beta vulgaris subsp. vulgaris.;|GO:0046872,GO:0016491,

geneid	trend	GO	Pathway	TF	NR	NT	Uniprot
					vulgaris]		
DN115321_c0_g1_i5	UP	[GO:0016021:integral component of membrane]	-	-	XP_010695745.1:PREDICTED: uncharacterized protein LOC104908341 [Beta vulgaris subsp. vulgaris]	-	A0A0J8BBY1|A0A0J8BBY1_BETVU: Uncharacterized protein;OS=Beta vulgaris subsp. vulgaris.;|GO:0016021,

geneid	trend	GO	Pathway	TF	NR	NT	Uniprot
DN126751_c0_g7_i5	UP	[GO:0004445:inositol-polyphosphate 5-phosphatase activity][GO:0046855:inositol phosphate dephosphorylation][GO:0032957:inositol trisphosphate metabolic process][GO:0046856:phosphatidylinositol dephosphorylation][GO:0009737:response to abscisic acid][GO:0009845:seed germination]	-	Eucgr.E02489.1.p:Eucalyptus grandis|M-type_MADS|M-type_MADS family protein	XP_002868018.1:type I inositol polyphosphate 5phosphatase 2 [Arabidopsis lyrata subsp. lyrata] >EFH44277.1 hypothetical protein ARALYDRAFT_914893 [Arabidopsis lyrata subsp. lyrata]	-	D7MCX8|D7MCX8_ARALL:Putative uncharacterized protein;OS=Arabidopsis lyrata subsp. lyrata;|GO:0004445,|GO:0046855,GO:0032957,GO:0046856,GO:0009737,GO:0009845,
DN109700_c0_g1_i1	UP	-	-	-	-	-	-
DN119620_c4_g1_i5	UP	[GO:0071949:FAD binding][GO:0016491:oxidoreductase activity]	-	-	XP_021753040.1:uncharacterized protein LOC110718509 [Chenopodium quinoa]	-	A0A0K9RCI1|A0A0K9RCI1_SPIOL:Uncharacterized protein;OS=Spinacia oleracea;|GO:0071949,GO:0016491,
DN124826_c1_g6_i1	UP	-	-	mrna13309.1-v1.0-hybrid:Fragaria vesca|FAR1|FAR1 family protein	XP_021765163.1:protein IQDOMAIN 14like [Chenopodium quinoa]	-	A0A0K9RC71|A0A0K9RC71_SPIOL:Uncharacterized protein;OS=Spinacia oleracea;

geneid	trend	GO	Pathway	TF	NR	NT	Uniprot
DN128162_c1_g10_i4	UP	[GO:0016765:transferase activity, transferring alkyl or aryl (other than methyl) groups]	[ko00900:Terpenoid backbone biosynthesis]	-	XP_010685566.1:PREDICTED: dehydrodolichyl diphosphate synthase 2 [Beta vulgaris subsp. vulgaris] >KMT05070.1 hypothetical protein BVRB_7g172260 [Beta vulgaris subsp. vulgaris]	-	A0A0J8BU46|A0A0J8BU46_BETVU:Alkyl transferase;OS=Beta vulgaris subsp. vulgaris.;|GO:0016765,
DN128610_c3_g2_i2	UP	-	-	Tp57577_TGAC_v2_mRNA39490:Trifolium pratense|NAC|NAC family protein	XP_010686531.1:PREDICTED: stemspecific protein TSJT1 [Beta vulgaris subsp. vulgaris] >KMT04278.1 hypothetical protein BVRB_8g183540 [Beta vulgaris subsp. vulgaris]	gi|478162451|gb|GAHK01014152.1|:TSA: Jatropha curcas Unigene1606_JC-CK_1A transcribed RNA sequence	A0A0J8BWG5|A0A0J8BWG5_BETVU: Uncharacterized protein;OS=Beta vulgaris subsp. vulgaris.;

geneid	trend	GO	Pathway	TF	NR	NT	Uniprot
DN127829_c7_g2_i1	UP	[GO:0003676:nucleic acid binding][GO:0008270:zinc ion binding][GO:0015074:DNA integration]	-	XP_010556248.1:Tarenaya hassleriana|ERF|ERF family protein	XP_016752567.1:PREDICTED: uncharacterized protein LOC107960800 [Gossypium hirsutum]	-	A0A178VTT7|A0A178VTT7_ARATH: Uncharacterized protein;OS=Arabidopsis thaliana;GN=AXX17_At2g08830{ECO:0000313|EMBL:OAP08483.1};|GO:0003676,GO:0008270,|GO:0015074,
DN118407_c6_g16_i1	UP	-	-	-	-	-	-
DN115552_c2_g1_i1	UP	[GO:0030286:dynein complex][GO:0007017:microtubule-based process]	[ko04962:Vasopressin-regulated water reabsorption]	-	XP_004252346.1:PREDICTED: dynein light chain LC6, flagellar outer arm [Solanum lycopersicum] >XP_015068296.1 PREDICTED: dynein light chain LC6, flagellar outer armlike [Solanum pennellii]	gi|357454934|ref|XM_003597700.1|:Medicago truncatula Dynein light chain (MTR_2g101850) mRNA, complete cds	K4DFB2|K4DFB2_SOLLC:Uncharacterized protein;OS=Solanum lycopersicum;|GO:0030286,|GO:0007017,

geneid	trend	GO	Pathway	TF	NR	NT	Uniprot
					>XP_015061091.1 PREDICTED: dynein light chain LC6, flagellar outer arm [Solanum pennellii]		
DN117543_c2_g1_i6	UP	-	-	-	XP_010673722.1:PREDICTED: uncharacterized protein At5g02240 [Beta vulgaris subsp. vulgaris] >KMT14575.1 hypothetical protein BVRB_4g073450 [Beta vulgaris subsp. vulgaris]	gi|224054521|ref|XM_002298266.1|:Populus trichocarpa predicted protein, mRNA	A0A0J8CLG9|A0A0J8CLG9_BETVU:Uncharacterized protein;OS=Beta vulgaris subsp. vulgaris.;

geneid	trend	GO	Pathway	TF	NR	NT	Uniprot
DN127764_c0_g3_i2	UP	[GO:0005737:cytoplasm][GO:0005524:ATP binding][GO:0006457:protein folding][GO:0006950:response to stress]	[ko04141:Protein processing in endoplasmic reticulum][ko04151:PI3K-Akt signaling pathway][ko04217:Necroptosis][ko04612:Antigen processing and presentation][ko04621:NOD-like receptor signaling pathway][ko04626:Plant-pathogen interaction][ko04657:IL-17 signaling pathway][ko04659:Th17 cell differentiation][ko04914:Progesterone-mediated oocyte maturation][ko04915:Estrogen signaling pathway][ko05200:Pathways in	OMERI06G27590.3:Oryza meridionalis|bZIP|bZIP family protein	XP_011084460.1:heat shock protein 83like [Sesamum indicum]	gi|288310311|gb|FJ969430.1|:Glycine max HSP90-1 mRNA, complete cds	P51819|HSP83_IPONI:Heat shock protein 83;OS=Ipomoea nil;|GO:0005737,|GO:0005524,|GO:0006457,GO:0006950,

geneid	trend	GO	Pathway	TF	NR	NT	Uniprot
			cancer][ko05215:Prostate cancer][ko05418:Fluid shear stress and atherosclerosis]				

geneid	trend	GO	Pathway	TF	NR	NT	Uniprot
DN122442_c2_g1_i1	UP	[GO:0016021:integral component of membrane]	-	-	XP_004249871.1:PREDICTED: uncharacterized protein LOC101263658 [Solanum lycopersicum] >XP_015058128.1 PREDICTED: uncharacterized protein LOC107004444 [Solanum pennellii]	gi|470110624|ref|XM_004291529.1|:PREDICTED: Fragaria vesca subsp. vesca aquaporin PIP2-2-like (LOC101300022), mRNA	K4D4F2|K4D4F2_SOLLC:Uncharacterized protein;OS=Solanum lycopersicum;|GO:0016021,
DN124320_c3_g1_i5	UP	-	-	-	XP_010688351.1:PREDICTED: uncharacterized protein LOC104902318 [Beta vulgaris subsp. vulgaris] >KMT03219.1 hypothetical protein BVRB_8g197670 [Beta vulgaris subsp. vulgaris]	-	A0A0J8BT98|A0A0J8BT98_BETVU:Uncharacterized protein;OS=Beta vulgaris subsp. vulgaris.;

geneid	trend	GO	Pathway	TF	NR	NT	Uniprot
DN127811_c0_g1_i2	UP	-	-	-	-	-	-
DN112072_c0_g1_i2	UP	[GO:0016021:integral component of membrane][GO:0005215:transporter activity]	-	GSMUA_Achr10P22520_001:Musa acuminata|TALE|TALE family protein	XP_021749960.1:protein NRT1/ PTR FAMILY 5.9like [Chenopodium quinoa]	gi|521742496|gb|GAIA01004920.1|:TSA: Salicornia europaea Unigene5207_Se200S transcribed RNA sequence	A0A0J8BBF4|A0A0J8BBF4_BETVU:Uncharacterized protein;OS=Beta vulgaris subsp. vulgaris.;|GO:0016021,|GO:0005215,
DN128497_c4_g1_i3	UP	-	-	-	-	-	-
DN122045_c3_g2_i9	UP	[GO:0016020:membrane][GO:0020037:heme binding][GO:0005506:iron ion binding][GO:0016709:oxidoreductase activity, acting on paired donors, with incorporation or reduction of molecular oxygen, NAD(P)H as one donor, and incorporation of one atom of oxygen][GO:0044550:secondary metabolite biosynthetic process]	-	676766070:Sisymbrium irio|bHLH|bHLH family protein	XP_021759822.1:cytochrome P450 83B1like isoform X1 [Chenopodium quinoa]	-	A5CBQ0|A5CBQ0_VITVI:Putative uncharacterized protein;OS=Vitis vinifera;GN=VIT_18s0001g13820 {ECO:0000313|EMBL:CCB46013.1};|GO:0016020,|GO:0020037,GO:0005506,GO:0016709,|GO:0044550,

geneid	trend	GO	Pathway	TF	NR	NT	Uniprot
DN109925_c0_g1_i2	UP	-	-	evm.model.supercontig_19.98:Carica papaya|bHLH|bHLH family protein	XP_021732588.1:uncharacterized protein LOC110699372 [Chenopodium quinoa]	-	A0A0J8FT38|A0A0J8FT38_BETVU:Uncharacterized protein;OS=Beta vulgaris subsp. vulgaris.;
DN124032_c4_g5_i14	UP	[GO:0003824:catalytic activity][GO:0008152:metabolic process]	[ko00280:Valine, leucine and isoleucine degradation][ko00640:Propanoate metabolism]	-	OMO57826.1:hypothetical protein COLO4_35079 [Corchorus olitorius]	gi|521508439|gb|GAHZ01022481.1|:TSA: Salicornia europaea Unigene24172_SeCKS transcribed RNA sequence	G7JTF7|G7JTF7_MEDTR:Branched chain alpha-keto acid dehydrogenase E1 beta subunit;OS=Medicago truncatula;GN=MTR_4g021470 {ECO:0000313|EMBL:AES87123.1};|GO:0003824,|GO:0008152,

geneid	trend	GO	Pathway	TF	NR	NT	Uniprot
DN128247_c0_g1_i1	UP	-	[ko04016:MAPK signaling pathway - plant][ko04075:Plant hormone signal transduction]	PSME_00044429-RA:Pseudotsuga menziesii|MYB_related|MYB_related family protein	XP_010677239.1:PREDICTED: abscisic acid receptor PYR1 [Beta vulgaris subsp. vulgaris] >KMT11653.1 hypothetical protein BVRB_5g105930 [Beta vulgaris subsp. vulgaris]	-	A0A0J8CHW7|A0A0J8CHW7_BETVU: Uncharacterized protein;OS=Beta vulgaris subsp. vulgaris.;
DN118676_c0_g1_i2	UP	[GO:0005737:cytoplasm][GO:0050113:inositol oxygenase activity][GO:0005506:iron ion binding][GO:0019310:inositol catabolic process]	[ko00053:Ascorbate and aldarate metabolism][ko00562:Inositol phosphate metabolism]	-	XP_021725011.1:inositol oxygenase 1like [Chenopodium quinoa]	gi|470123220|ref|XM_004297579.1|:PREDICTED: Fragaria vesca subsp. vesca inositol oxygenase 1-like (LOC101306622), mRNA	A0A0K9QFB1|A0A0K9QFB1_SPIOL:Uncharacterized protein;OS=Spinacia oleracea;|GO:0005737,|GO:0050113,GO:0005506,|GO:0019310,
DN118229_c2_g1_i2	UP	-	[ko04141:Protein processing in endoplasmic reticulum]	Ote100209250011:Ocimum tenuiflorum|MYB_related|MYB_related family protein	XP_021744250.1:small heat shock protein, chloroplasticlike [Chenopodium quinoa]	-	M1AVE4|M1AVE4_SOLTU:Uncharacterized protein;OS=Solanum tuberosum;

geneid	trend	GO	Pathway	TF	NR	NT	Uniprot
DN120278_c2_g4_i1	UP	-	-	-	XP_021857368.1:uncharacterized protein LOC110796612 [Spinacia oleracea]	-	-
DN127603_c1_g1_i1	UP	[GO:0016021:integral component of membrane][GO:0055114:oxidation-reduction process]	-	678320713:Utricularia gibba|M-type_MADS|M-type_MADS family protein	XP_021845163.1:cytochrome b561 and DOMON domaincontaining protein At3g61750 [Spinacia oleracea] >KNA25964.1 hypothetical protein SOVF_001860 [Spinacia oleracea]	-	A0A0K9S3Y6|A0A0K9S3Y6_SPIOL:Uncharacterized protein;OS=Spinacia oleracea;|GO:0016021,|GO:0055114,
DN123186_c1_g1_i1	UP	-	-	-	-	-	-
DN119942_c0_g1_i6	UP	[GO:0016787:hydrolase activity][GO:0008152:metabolic process]	-	MDP0000298233:Malus domestica|C3H|C3H family protein	XP_021861602.1:haloacid dehalogenaselike hydrolase domaincontaining protein 3 [Spinacia oleracea] >KNA06903.1	gi|357509516|ref|XM_003624999.1|:Medicago truncatula Phosphoglycolate phosphatase (MTR_7g090370) mRNA, complete cds	A0A0K9QJM7|A0A0K9QJM7_SPIOL: Uncharacterized protein;OS=Spinacia oleracea;|GO:00167 87,|GO:0008152,

geneid	trend	GO	Pathway	TF	NR	NT	Uniprot
					hypothetical protein SOVF_176790 [Spinacia oleracea]		
DN124633_c 1_g2_i11	UP	[GO:0004553:hydrolase activity, hydrolyzing O-glycosyl compounds][GO:0005975:carbohydra te metabolic process]	[ko00500:Starch and sucrose metabolism]	-	XP_010675793.1:P REDICTED: isoamylase 3, chloroplastic isoform X1 [Beta vulgaris subsp. vulgaris]	gi|521671411|gb|GA IA01031822.1|:TSA: Salicornia europaea Unigene34912_Se20 0S transcribed RNA sequence	A0A0J8FVX6|A0A0 J8FVX6_BETVU:U ncharacterized protein;OS=Beta vulgaris subsp. vulgaris.;|GO:00045 53,|GO:0005975,
DN126511_c 3_g4_i2	UP	[GO:0003676:nucleic acid binding][GO:0015074:DNA integration]	-	PK03054.1:Cannabi s sativa|HD-ZIP|HD-Z IP family protein	KYP53274.1:Retrov irusrelated Pol polyprotein from transposon TNT 194 [Cajanus cajan]	-	A0A151SEK6|A0A1 51SEK6_CAJCA:Re trovirus-related Pol polyprotein from transposon TNT 1-94;OS=Cajanus cajan;|GO:0003676,| GO:0015074,
DN123904_c 0_g2_i2	UP	-	-	-	-	-	-

geneid	trend	GO	Pathway	TF	NR	NT	Uniprot
DN127967_c2_g2_i4	UP	-	-	-	-	-	-
DN122269_c3_g2_i3	UP	[GO:0016021:integral component of membrane][GO:0008643:carbohydrate transport]	-	-	XP_010692309.1:PREDICTED: bidirectional sugar transporter SWEET6blike [Beta vulgaris subsp. vulgaris]	-	A0A0K9R724|A0A0K9R724_SPIOL:Bidirectional sugar transporter SWEET;OS=Spinacia oleracea;|GO:0016021,|GO:0008643,
DN127571_c3_g1_i6	UP	-	-	-	-	-	-
DN123693_c0_g3_i2	UP	[GO:0009507:chloroplast][GO:0005524:ATP binding][GO:0008974:phosphoribulokinase activity][GO:0019253:reductive pentose-phosphate cycle]	[ko00710:Carbon fixation in photosynthetic organisms]	Niben101Scf12623g00009.1:Nicotiana benthamiana|BBR-BPC|BBR-BPC family protein	P27774.1:RecName: Full=Phosphoribulokinase, chloroplastic; Short=PRK; Short=PRKase; AltName: Full=Phosphopentokinase; Flags: Precursor >AAA33034.1 phosphoribulokinase	gi|167265|gb|M73707.1|CIPPRK:Mesembryanthemum crystallinum phosphoribulokinase mRNA, complete CDS	P27774|KPPR_MESCR:Phosphoribulokinase, chloroplastic;OS=Mesembryanthemum crystallinum;|GO:0009507,|GO:0005524,GO:0008974,|GO:0019253,

geneid	trend	GO	Pathway	TF	NR	NT	Uniprot
					[Mesembryanthemum crystallinum]		
DN128509_c1_g5_i4	UP	[GO:0016021:integral component of membrane][GO:0005524:ATP binding][GO:0005509:calcium ion binding][GO:0004672:protein kinase activity]	-	XP_004487156.1:Cicer arietinum|Trihelix|Trihelix family protein	XP_020232980.1:uncharacterized protein LOC109813230 [Cajanus cajan]	-	A0A151U7C0|A0A151U7C0_CAJCA:Wall-associated receptor kinase-like 4;OS=Cajanus cajan;|GO:0016021,|GO:0005524,GO:0005509,GO:0004672,
DN110887_c0_g1_i2	UP	-	-	-	-	-	-

geneid	trend	GO	Pathway	TF	NR	NT	Uniprot
DN118017_c1_g1_i2	UP	[GO:0016020:membrane][GO:0005509:calcium ion binding][GO:0070290:N-acylphosphatidylethanolamine-specific phospholipase D activity][GO:0004630:phospholipase D activity][GO:0016042:lipid catabolic process][GO:0046470:phosphatidylcholine metabolic process]	[ko00564:Glycerophospholipid metabolism][ko00565:Ether lipid metabolism][ko04014:Ras signaling pathway][ko04024:cAMP signaling pathway][ko04071:Sphingolipid signaling pathway][ko04072:Phospholipase D signaling pathway][ko04144:Endocytosis][ko04666:Fc gamma R-mediated phagocytosis][ko04724:Glutamatergic synapse][ko04912:GnRH signaling pathway][ko05231:Choline metabolism in cancer]	-	XP_021754507.1:phospholipase D alpha 4like [Chenopodium quinoa]	-	A0A0K9QC38|A0A0K9QC38_SPIOL:Phospholipase D;OS=Spinacia oleracea;|GO:0016020,|GO:0005509,GO:0070290,GO:0004630,|GO:0016042,GO:0046470,

geneid	trend	GO	Pathway	TF	NR	NT	Uniprot
DN109960_c0_g1_i2	UP	-	-	-	-	-	-
DN127312_c4_g10_i2	UP	[GO:0005524:ATP binding]	-	-	AEI69347.1:chloroplast rubisco activase [Ophiopogon japonicus]	gi|21950711|gb|AY117142.1|:Chenopodium quinoa rubisco activase (rca) mRNA, complete cds	F8UAA7|F8UAA7_9ASPA:Chloroplast rubisco activase;OS=Ophiopogon japonicus.;|GO:0005524,
DN121695_c1_g1_i2	UP	-	-	Sp_143900_sark.t1: Spinacia oleracea|bHLH|bHLH family protein	XP_021836627.1:transcription factor bHLH130like [Spinacia oleracea] >XP_021840406.1 transcription factor bHLH130like [Spinacia oleracea] >KNA10511.1 hypothetical protein SOVF_143900 [Spinacia oleracea]	gi|521493425|gb|GAHZ01037495.1|:TSA: Salicornia europaea Unigene42272_SeCKS transcribed RNA sequence	A0A0K9QVG3|A0A0K9QVG3_SPIOL: Uncharacterized protein;OS=Spinacia oleracea;

geneid	trend	GO	Pathway	TF	NR	NT	Uniprot
DN111104_c0_g4_i2	UP	[GO:0008168:methyltransferase activity]	-	EMT20798:Aegilops tauschii|MYB_related|MYB_related family protein	XP_010113410.1:Salicylate Omethyltransferase [Morus notabilis] >EXC35437.1 Salicylate Omethyltransferase [Morus notabilis]	-	W9SN62|W9SN62_9ROSA:Salicylate O-methyltransferase;OS=Morus notabilis.;|GO:0008168,
DN118702_c3_g2_i2	UP	[GO:0016021:integral component of membrane][GO:0006506:GPI anchor biosynthetic process]	[ko00563:Glycosylphosphatidylinositol (GPI)-anchor biosynthesis]	-	XP_010676542.1:PREDICTED: phosphatidylinositol glycan anchor biosynthesis class U protein [Beta vulgaris subsp. vulgaris] >KMT12147.1 hypothetical protein BVRB_5g100990 isoform A [Beta vulgaris subsp. vulgaris]	gi|224058596|ref|XM_002299522.1|:Populus trichocarpa predicted protein, mRNA	A0A0K9QJ93|A0A0K9QJ93_SPIOL:Uncharacterized protein;OS=Spinacia oleracea;|GO:0016021,|GO:0006506,

geneid	trend	GO	Pathway	TF	NR	NT	Uniprot
DN124589_c2_g2_i5	UP	-	-	-	XP_010682796.1:PREDICTED: Fbox/kelchrepeat protein At1g67480 [Beta vulgaris subsp. vulgaris] >XP_010682797.1 PREDICTED: Fbox/kelchrepeat protein At1g67480 [Beta vulgaris subsp. vulgaris] >KMT07483.1 hypothetical protein BVRB_6g151000 [Beta vulgaris subsp. vulgaris]	-	A0A0J8C608|A0A0J8C608_BETVU:Uncharacterized protein;OS=Beta vulgaris subsp. vulgaris.;
DN126792_c1_g2_i1	UP	-	-	-	XP_006846942.1:snakin2 [Amborella trichopoda] >ERN08523.1 hypothetical protein AMTR_s00017p00002980 [Amborella trichopoda]	-	W1PKF9|W1PKF9_AMBTC:Uncharacterized protein;OS=Amborella trichopoda.;

geneid	trend	GO	Pathway	TF	NR	NT	Uniprot
DN126024_c8_g13_i1	UP	-	-	-	-	-	-
DN127619_c2_g1_i3	UP	[GO:0003676:nucleic acid binding]	-	-	CCA66153.1:hypothetical protein [Beta vulgaris subsp. vulgaris]	-	F4NCK1|F4NCK1_BETVU:Uncharacterized protein;OS=Beta vulgaris subsp. vulgaris.;|GO:0003676,
DN119172_c2_g1_i1	UP	-	-	-	-	-	-
DN121965_c1_g2_i6	UP	[GO:0005737:cytoplasm][GO:0005634:nucleus][GO:0010309:acireductone dioxygenase [iron(II)-requiring] activity][GO:0005506:iron ion binding][GO:0019509:L-methionine salvage from methylthioadenosine]	[ko00270:Cysteine and methionine metabolism]	LPERR03G04120.1:Leersia perrieri|HSF|HSF family protein	XP_021725170.1:1,2dihydroxy3keto5methylthiopentene dioxygenase 1like [Chenopodium quinoa]	-	A0A0H2UIA1|A0A0H2UIA1_SOLLC:1,2-dihydroxy-3-keto-5-methylthiopentene dioxygenase;OS=Solanum lycopersicum;|GO:0005737,GO:0005634,|GO:0010309,GO:0005506,|GO:0019509,
DN123328_c2_g3_i1	UP	-	-	-	-	-	-

geneid	trend	GO	Pathway	TF	NR	NT	Uniprot
DN124130_c4_g3_i2	UP	[GO:0005524:ATP binding][GO:0004672:protein kinase activity]	-	Achn158001:Actinidia chinensis|bHLH|bHLH family protein	XP_019104247.1:PREDICTED: wallassociated receptor kinaselike 22 [Beta vulgaris subsp. vulgaris] >KMT14786.1 hypothetical protein BVRB_4g075510 [Beta vulgaris subsp. vulgaris]	-	A0A0J8FG34|A0A0J8FG34_BETVU:Uncharacterized protein;OS=Beta vulgaris subsp. vulgaris.;|GO:0005524,GO:0004672,
DN116987_c0_g1_i1	UP	-	-	-	-	-	-
DN113557_c0_g1_i2	UP	[GO:0016021:integral component of membrane][GO:0008168:methyltransferase activity]	-	CCG010610.1:Populus euphratica|NAC|NAC family protein	XP_010693483.1:PREDICTED: probable methyltransferase PMT15 [Beta vulgaris subsp. vulgaris]>KMS99034.1 hypothetical protein BVRB_3g066400 [Beta vulgaris subsp. vulgaris]	gi|460377166|ref|XM_004234318.1|:PREDICTED: Solanum lycopersicum probable methyltransferase PMT15-like (LOC101267776), mRNA	A0A0J8BCH3|A0A0J8BCH3_BETVU: Uncharacterized protein;OS=Beta vulgaris subsp. vulgaris.;|GO:0016021,|GO:0008168,

geneid	trend	GO	Pathway	TF	NR	NT	Uniprot
DN114714_c0_g1_i1	UP	-	-	-	XP_010675712.1:PREDICTED: polygalacturonase inhibitor 1like [Beta vulgaris subsp. vulgaris]	-	A0A0J8CL12|A0A0J8CL12_BETVU:Uncharacterized protein;OS=Beta vulgaris subsp. vulgaris.;
DN173955_c0_g1_i1	UP	-	-	-	GAV83756.1:zfRVT domaincontaining protein, partial [Cephalotus follicularis]	-	-
DN111298_c0_g1_i1	UP	[GO:0016021:integral component of membrane][GO:0005634:nucleus][GO:0003677:DNA binding][GO:0006355:regulation of transcription, DNA-templated]	-	XP_009781429.1:Nicotiana sylvestris|S1Fa-like|S1Fa-like family protein	XP_009781429.1:PREDICTED: DNAbinding protein S1FAlike [Nicotiana sylvestris] >XP_016432997.1 PREDICTED: DNAbinding protein S1FAlike [Nicotiana tabacum]	-	A0A0B0N225|A0A0B0N225_GOSAR:DNA-binding S1FA2;OS=Gossypium arboreum;|GO:0016021,GO:0005634,|GO:0003677,|GO:0006355,
DN122740_c0_g4_i3	UP	-	-	RrC887_p5:Raphanus raphanistrum|G2-like|G2-like family	BAW35437.1:Cterminal peptidase [Nepenthes alata]	-	A0A061GC60|A0A061GC60_THECC:Uncharacterized protein;OS=Theobro

geneid	trend	GO	Pathway	TF	NR	NT	Uniprot
				protein			ma cacao;
DN128908_c5_g1_i12	UP	[GO:0005634:nucleus][GO:0000977:RNA polymerase II regulatory region sequence-specific DNA binding][GO:0003700:DNA-binding transcription factor activity][GO:0000165:MAPK cascade][GO:0045944:positive regulation of transcription by RNA polymerase II][GO:0006351:transcription, DNA-templated]	-	Bv9_214370_scjj.t1:Beta vulgaris|MIKC_MADS|MIKC_MADS family protein	XP_021775635.1:MADSbox protein AGL24like [Chenopodium quinoa] >XP_021775643.1 MADSbox protein AGL24like [Chenopodium quinoa] >XP_021775649.1 MADSbox protein AGL24like [Chenopodium quinoa] >XP_021775653.1 MADSbox protein AGL24like [Chenopodium quinoa] >XP_021775660.1	gi|2735765|gb|AF008652.1|AF008652:Solanum tuberosum MADS transcriptional factor (Stmads11) mRNA, complete cds	A0A0J8BN34|A0A0J8BN34_BETVU:Uncharacterized protein;OS=Beta vulgaris subsp. vulgaris.;|GO:0005634,|GO:0000977,GO:0003700,|GO:0000165,GO:0045944,GO:0006351,

geneid	trend	GO	Pathway	TF	NR	NT	Uniprot
					MADSbox protein AGL24like [Chenopodium quinoa]		
DN110662_c0_g1_i1	UP	[GO:0020037:heme binding][GO:0004601:peroxidase activity][GO:0006979:response to oxidative stress]	[ko00053:Ascorbate and aldarate metabolism][ko00480:Glutathione metabolism]	PH01001490G0340:Phyllostachys heterocycla|MYB_related|MYB_related family protein	XP_010696372.1:PREDICTED: Lascorbate peroxidase 2, cytosoliclike [Beta vulgaris subsp. vulgaris]	gi|154199606|gb|DQ246218.2|:Litchi chinensis putative ascorbate peroxidase mRNA, complete cds	V4SNA9|V4SNA9_9ROSI:Uncharacterized protein;OS=Citrus clementina.;|GO:0020037,GO:0004601,|GO:0006979,

geneid	trend	GO	Pathway	TF	NR	NT	Uniprot
DN125032_c0_g3_i8	UP	[GO:0016021:integral component of membrane][GO:0055085:transmembrane transport]	-	-	XP_012083252.1:mitochondrial adenine nucleotide transporter ADNT1 [Jatropha curcas] >KDP28516.1 hypothetical protein JCGZ_14287 [Jatropha curcas]	gi|521735486|gb|GAIA01009166.1|:TSA: Salicornia europaea Unigene9525_Se200S transcribed RNA sequence	A0A067JX18|A0A067JX18_JATCU:Uncharacterized protein;OS=Jatropha curcas;|GO:0016021,|GO:0055085,
DN115998_c0_g1_i2	UP	-	-	-	-	-	-
DN128531_c1_g2_i3	UP	-	-	-	XP_002524159.1:PREDICTED: uncharacterized protein sll0103 [Ricinus communis] >EEF38222.1 protein binding protein, putative [Ricinus communis]	-	B9SDU0|B9SDU0_RICCO:Protein binding protein, putative;OS=Ricinus communis;
DN113820_c3_g1_i3	UP	-	-	-	-	-	-

geneid	trend	GO	Pathway	TF	NR	NT	Uniprot
DN117139_c4_g1_i2	UP	[GO:0016021:integral component of membrane]	-	-	XP_021765661.1:uncharacterized protein LOC110730191 [Chenopodium quinoa]	gi|224138099|ref|XM_002326482.1|:Populus trichocarpa predicted protein, mRNA	A0A0J8F6U0|A0A0J8F6U0_BETVU:Uncharacterized protein;OS=Beta vulgaris subsp. vulgaris.;|GO:0016021,
DN127937_c0_g4_i3	UP	[GO:0016021:integral component of membrane][GO:0005524:ATP binding][GO:0004672:protein kinase activity][GO:0048544:recognition of pollen]	-	Kaladp0839s0004.1.p:Kalanchoe laxiflora|ERF|ERF family protein	XP_021733575.1:LOW QUALITY PROTEIN: Gtype lectin Sreceptorlike serine/threonineprotein kinase At4g03230 [Chenopodium quinoa]	-	A0A0J8CA67|A0A0J8CA67_BETVU:Uncharacterized protein;OS=Beta vulgaris subsp. vulgaris.;|GO:0016021,|GO:0005524,GO:0004672,|GO:0048544,
DN119570_c3_g10_i1	UP	[GO:0008080:N-acetyltransferase activity]	-	-	XP_021760396.1:uncharacterized protein LOC110725217 isoform X2 [Chenopodium quinoa]	-	A0A0J8D733|A0A0J8D733_BETVU:Uncharacterized protein;OS=Beta vulgaris subsp. vulgaris.;|GO:0008080,

geneid	trend	GO	Pathway	TF	NR	NT	Uniprot
DN117369_c1_g3_i1	UP	[GO:0009522:photosystem I][GO:0015979:photosynthesis]	[ko00195:Photosynthesis]	MDP0000319772:Malus domestica|MYB_related|MYB_related family protein	XP_021728060.1:photosystem I reaction center subunit N, chloroplasticlike [Chenopodium quinoa]	gi|255571378|ref|XM_002526592.1|:Ricinus communis Photosystem I reaction center subunit N, chloroplast precursor, putative, mRNA	A0A061DJ27|A0A061DJ27_THECC:Photosystem I reaction center subunit PSI;OS=Theobroma cacao;|GO:0009522,|GO:0015979,
DN129073_c1_g1_i1	UP	-	-	AHYPO_004439-RA:Amaranthus hypochondriacus|bHLH|bHLH family protein	XP_021767200.1:transcription factor MYC2like [Chenopodium quinoa]	-	M0RY21|M0RY21_MUSAM:Uncharacterized protein;OS=Musa acuminata subsp. malaccensis;
DN122602_c2_g1_i19	UP	[GO:0016021:integral component of membrane][GO:0032472:Golgi calcium ion transport]	-	-	XP_021723857.1:GDT1like protein 5 [Chenopodium quinoa]	gi|521742316|gb|GAIA01005012.1|:TSA: Salicornia europaea Unigene5299_Se200S transcribed RNA sequence	A0A0J8B7Y4|A0A0J8B7Y4_BETVU:Uncharacterized protein;OS=Beta vulgaris subsp. vulgaris.;|GO:0016021,|GO:0032472,

geneid	trend	GO	Pathway	TF	NR	NT	Uniprot
DN125575_c0_g2_i4	UP	[GO:0016021:integral component of membrane][GO:0005524:ATP binding][GO:0000287:magnesium ion binding][GO:0004012:phospholipid-translocating ATPase activity]	-	-	EOY22253.1:ATPase E1E2 type family protein / haloacid dehalogenaselike hydrolase family protein isoform 3 [Theobroma cacao]	gi|255541871|ref|XM_002511954.1|:Ricinus communis Phospholipid-transporting ATPase, putative, mRNA	A0A061FYX9|A0A061FYX9_THECC: Phospholipid-transporting ATPase;OS=Theobroma cacao;|GO:0016021,|GO:0005524,GO:0000287,GO:0004012,
DN123532_c0_g5_i1	UP	[GO:0005525:GTP binding]	-	-	XP_010692167.1:PREDICTED: GTPbinding protein At2g22870 [Beta vulgaris subsp. vulgaris] >KMT18465.1 hypothetical protein BVRB_2g026080 [Beta vulgaris subsp. vulgaris]	-	A0A0J8CXJ9|A0A0J8CXJ9_BETVU:Uncharacterized protein;OS=Beta vulgaris subsp. vulgaris.;|GO:0005525,

geneid	trend	GO	Pathway	TF	NR	NT	Uniprot
DN125857_c5_g2_i1	UP	-	-	-	XP_010687999.1:PREDICTED: uncharacterized protein LOC104902039 isoform X1 [Beta vulgaris subsp. vulgaris]	-	A0A0J8BNC4|A0A0J8BNC4_BETVU: Uncharacterized protein;OS=Beta vulgaris subsp. vulgaris.;
DN226613_c0_g1_i1	UP	[GO:0016021:integral component of membrane][GO:0005524:ATP binding][GO:0004672:protein kinase activity]	-	Kaladp0839s0004.1.p:Kalanchoe laxiflora|ERF|ERF family protein	XP_021726249.1:cysteinerich repeat secretory protein 38like [Chenopodium quinoa]	-	A5BPV0|A5BPV0_VITVI:Putative uncharacterized protein;OS=Vitis vinifera;|GO:0016021,|GO:0005524,GO:0004672,

geneid	trend	GO	Pathway	TF	NR	NT	Uniprot
DN120547_c0_g1_i7	UP	[GO:0009570:chloroplast stroma][GO:0080041:ADP-ribose pyrophosphohydrolase activity][GO:0051536:iron-sulfur cluster binding][GO:0009228:thiamine biosynthetic process]	[ko00730:Thiamine metabolism]	-	XP_010685602.1:PREDICTED: phosphomethylpyrimidine synthase, chloroplastic isoform X2 [Beta vulgaris subsp. vulgaris] >XP_019106532.1 PREDICTED: phosphomethylpyrimidine synthase, chloroplastic isoform X1 [Beta vulgaris subsp. vulgaris] >KMT05103.1 hypothetical protein BVRB_7g172570 [Beta vulgaris subsp. vulgaris]	gi|521697686|gb|GAIA01018715.1|:TSA: Salicornia europaea Unigene19672_Se200S transcribed RNA sequence	A0A0J8BYS7|A0A0J8BYS7_BETVU:Uncharacterized protein;OS=Beta vulgaris subsp. vulgaris.;|GO:0009570,|GO:0080041,GO:0051536,|GO:0009228,

geneid	trend	GO	Pathway	TF	NR	NT	Uniprot
DN122547_c0_g3_i1	UP	[GO:0016021:integral component of membrane][GO:0020037:heme binding][GO:0005506:iron ion binding][GO:0016705:oxidoreductase activity, acting on paired donors, with incorporation or reduction of molecular oxygen]	-	Sme2.5_00297.1_g00019.1:Solanum melongena|MYB|MYB family protein	XP_010676211.1:PREDICTED: 7ethoxycoumarin Odeethylaselike [Beta vulgaris subsp. vulgaris]	gi|502175960|ref|XM_004515716.1|:PREDICTED: Cicer arietinum geraniol 8-hydroxylase-like (LOC101501165), mRNA	A0A0J8F9N7|A0A0J8F9N7_BETVU:Uncharacterized protein;OS=Beta vulgaris subsp. vulgaris.;|GO:0016021,|GO:0020037,GO:0005506,GO:0016705,
DN112584_c0_g2_i3	UP	-	-	-	KMS98548.1:hypothetical protein BVRB_4g092370 [Beta vulgaris subsp. vulgaris]	-	A0A0J8BEV9|A0A0J8BEV9_BETVU:Uncharacterized protein;OS=Beta vulgaris subsp. vulgaris.;

geneid	trend	GO	Pathway	TF	NR	NT	Uniprot
DN125000_c0_g1_i6	UP	[GO:0016020:membrane][GO:0020037:heme binding][GO:0005506:iron ion binding][GO:0016709:oxidoreductase activity, acting on paired donors, with incorporation or reduction of molecular oxygen, NAD(P)H as one donor, and incorporation of one atom of oxygen][GO:0098542:defense response to other organism][GO:0042343:indole glucosinolate metabolic process][GO:0044550:secondary metabolite biosynthetic process]	-	Cc10_g03650:Coffea canephora|G2-like|G2-like family protein	XP_010679549.1:PREDICTED: isoflavone 2'hydroxylaselike isoform X1 [Beta vulgaris subsp. vulgaris] >XP_010679550.1 PREDICTED: isoflavone 2'hydroxylaselike isoform X2 [Beta vulgaris subsp. vulgaris]	-	F6HSX8|F6HSX8_VITVI:Putative uncharacterized protein;OS=Vitis vinifera;GN=VIT_07s0129g00860 {ECO:0000313|EMBL:CCB57788.1};|GO:0016020,|GO:0020037,GO:0005506,GO:0016709,|GO:0098542,GO:0042343,GO:0044550,
DN110665_c0_g1_i2	UP	-	-	OPUNC06G18860.1:Oryza punctata|G2-like|G2-like family protein	XP_021727289.1:protein C2DOMAIN ABARELATED 7like [Chenopodium quinoa]	-	A0A0K9RM00|A0A0K9RM00_SPIOL: Uncharacterized protein;OS=Spinacia oleracea;

geneid	trend	GO	Pathway	TF	NR	NT	Uniprot
DN127706_c1_g1_i1	UP	[GO:0005737:cytoplasm][GO:0005524:ATP binding][GO:0016485:protein processing][GO:0009408:response to heat]	[ko04213:Longevity regulating pathway - multiple species]	-	XP_021770041.1:chaperone protein ClpB3, chloroplasticlike [Chenopodium quinoa]	gi|297811680|ref|XM_002873678.1|:Arabidopsis lyrata subsp. lyrata APG6/CLPB-P/CLPB3, mRNA	A0A0J8CU40|A0A0J8CU40_BETVU:Uncharacterized protein;OS=Beta vulgaris subsp. vulgaris.;|GO:0005737,|GO:0005524,|GO:0016485,GO:0009408,
DN119942_c0_g1_i1	UP	[GO:0016787:hydrolase activity][GO:0008152:metabolic process]	-	-	XP_021861602.1:haloacid dehalogenaselike hydrolase domaincontaining protein 3 [Spinacia oleracea] >KNA06903.1 hypothetical protein SOVF_176790 [Spinacia oleracea]	gi|224089986|ref|XM_002308858.1|:Populus trichocarpa predicted protein, mRNA	A0A0K9QJM7|A0A0K9QJM7_SPIOL:Uncharacterized protein;OS=Spinacia oleracea;|GO:0016787,|GO:0008152,

geneid	trend	GO	Pathway	TF	NR	NT	Uniprot
DN125073_c4_g1_i5	UP	[GO:0016021:integral component of membrane]	-	MDP0000321461:Malus domestica|G2-like|G2-like family protein	XP_021770841.1:sugar transporter ERD6like 16 [Chenopodium quinoa]	-	A0A0J8B4N7|A0A0J8B4N7_BETVU:Uncharacterized protein;OS=Beta vulgaris subsp. vulgaris.;|GO:0016021,|GO:0022891,
DN115998_c1_g1_i1	UP	[GO:0005615:extracellular space][GO:0005794:Golgi apparatus][GO:0005773:vacuole][GO:0030598:rRNA N-glycosylase activity][GO:0006952:defense response][GO:0017148:negative regulation of translation]	-	-	P85208.1:RecName: Full=Dioicin2; AltName: Full=Ribosomeinactivating protein; AltName: Full=rRNA Nglycosidase	-	P85208|RIPD2_PHYDI:Dioicin-2;OS=Phytolacca dioica;|GO:0005615,GO:0005794,GO:0005773,|GO:0030598,|GO:0006952,GO:0017148,
DN125797_c3_g3_i2	UP	[GO:0016758:transferase activity, transferring hexosyl groups][GO:0008152:metabolic process]	-	Tp57577_TGAC_v2_mRNA33215:Trifolium pratense|bHLH|bHLH family protein	XP_021841626.1:anthocyanidin 3Oglucosyltransferase 2like [Spinacia oleracea]	gi|521750879|gb|GAIA01000709.1|:TSA: Salicornia europaea Unigene723_Se200S transcribed RNA sequence	A0A0K9RCZ4|A0A0K9RCZ4_SPIOL:Glycosyltransferase;OS=Spinacia oleracea;|GO:0016758,|GO:0008152,

geneid	trend	GO	Pathway	TF	NR	NT	Uniprot
DN122593_c1_g6_i2	UP	[GO:0016765:transferase activity, transferring alkyl or aryl (other than methyl) groups]	[ko00900:Terpenoid backbone biosynthesis]	-	XP_010685566.1:PREDICTED: dehydrodolichyl diphosphate synthase 2 [Beta vulgaris subsp. vulgaris] >KMT05070.1 hypothetical protein BVRB_7g172260 [Beta vulgaris subsp. vulgaris]	-	A0A0J8BU46|A0A0J8BU46_BETVU:Alkyl transferase;OS=Beta vulgaris subsp. vulgaris.;|GO:0016765,
DN120971_c6_g1_i1	UP	-	-	-	-	-	-
DN126683_c6_g2_i4	UP	-	-	-	KNA13792.1:hypothetical protein SOVF_113620 [Spinacia oleracea]	-	A0A0K9R4Q3|A0A0K9R4Q3_SPIOL:Uncharacterized protein;OS=Spinacia oleracea;

geneid	trend	GO	Pathway	TF	NR	NT	Uniprot
DN125071_c0_g1_i13	UP	[GO:0008168:methyltransferase activity][GO:0006694:steroid biosynthetic process]	[ko00100:Steroid biosynthesis]	-	XP_021736207.1:cycloartenolC24methyltransferaselike [Chenopodium quinoa] >XP_021736215.1 cycloartenolC24methyltransferaselike [Chenopodium quinoa]	gi|359487306|ref|XM_003633516.1|:PREDICTED: Vitis vinifera cycloartenol-C-24-methyltransferase-like, transcript variant 2 (LOC100263590), mRNA	A0A0K9RAF8|A0A0K9RAF8_SPIOL: Methyltransferase;OS=Spinacia oleracea;|GO:0008168,|GO:0006694,
DN118117_c3_g3_i8	UP	[GO:0005886:plasma membrane]	-	-	XP_010668142.1:PREDICTED: uncharacterized protein At1g76660 isoform X2 [Beta vulgaris subsp. vulgaris] >KMS94867.1 hypothetical protein BVRB_014550 [Beta vulgaris subsp. vulgaris]	-	A0A0J8B4U7|A0A0J8B4U7_BETVU:Uncharacterized protein;OS=Beta vulgaris subsp. vulgaris.;|GO:0005886,

geneid	trend	GO	Pathway	TF	NR	NT	Uniprot
DN117762_c0_g7_i2	UP	[GO:0016021:integral component of membrane]	-	676708970:Sisymbrium irio|ERF|ERF family protein	XP_010680925.1:PREDICTED: uncharacterized protein LOC104895967 [Beta vulgaris subsp. vulgaris]	-	A0A0J8C413|A0A0J8C413_BETVU:Uncharacterized protein;OS=Beta vulgaris subsp. vulgaris.;|GO:0016021,
DN113527_c0_g1_i1	UP	-	[ko04141:Protein processing in endoplasmic reticulum]	WALNUT_00030998-RA:Juglans regia|NF-YB|NF-YB family protein	XP_021727631.1:18.1 kDa class I heat shock proteinlike [Chenopodium quinoa]	-	A0A0K9QNA4|A0A0K9QNA4_SPIOL: Uncharacterized protein;OS=Spinacia oleracea;
DN123467_c0_g1_i1	UP	[GO:0009507:chloroplast][GO:0051301:cell division][GO:0010020:chloroplast fission][GO:0032955:regulation of division septum assembly]	-	-	XP_010683082.1:PREDICTED: cell division topological specificity factor homolog, chloroplastic [Beta vulgaris subsp. vulgaris] >KMT06977.1 hypothetical protein BVRB_6g153110 [Beta vulgaris subsp. vulgaris]	gi|147788810|emb|AM443377.2|:Vitis vinifera contig VV78X080730.12, whole genome shotgun sequence	A0A0J8C0H1|A0A0J8C0H1_BETVU:Uncharacterized protein;OS=Beta vulgaris subsp. vulgaris.;|GO:0009507,|GO:0051301,GO:0010020,GO:0032955,

geneid	trend	GO	Pathway	TF	NR	NT	Uniprot
DN124231_c1_g4_i1	UP	[GO:0005576:extracellular region]	[ko04016:MAPK signaling pathway - plant][ko04075:Plant hormone signal transduction][ko04626:Plant-pathogen interaction]	Dca23924.1:Dianthus caryophyllus|FAR1|FAR1 family protein	XP_021776183.1:pathogenesisrelated protein PRB13like [Chenopodium quinoa]	-	A0A0J8BHU2|A0A0J8BHU2_BETVU: Uncharacterized protein;OS=Beta vulgaris subsp. vulgaris.;|GO:0005576,
DN117680_c0_g1_i2	UP	[GO:0003700:DNA-binding transcription factor activity]	[ko04612:Antigen processing and presentation][ko05152:Tuberculosis]	AHYPO_014525-RA:Amaranthus hypochondriacus|NF-YA|NF-YA family protein	XP_021715040.1:nuclear transcription factor Y subunit A7like [Chenopodium quinoa] >XP_021715041.1 nuclear transcription factor Y subunit A7like [Chenopodium quinoa]	-	A0A0K9RF32|A0A0K9RF32_SPIOL:Uncharacterized protein;OS=Spinacia oleracea;|GO:0003700,

geneid	trend	GO	Pathway	TF	NR	NT	Uniprot
DN118751_c0_g4_i1	UP	[GO:0016021:integral component of membrane]	-	-	XP_021772126.1:lysine histidine transporter 2like isoform X2 [Chenopodium quinoa] >XP_021772127.1 lysine histidine transporter 2like isoform X2 [Chenopodium quinoa]	gi|460371953|ref|XM_004231751.1|:PREDICTED: Solanum lycopersicum lysine histidine transporter 1-like (LOC101248631), mRNA	A0A0K9RSW6|A0A0K9RSW6_SPIOL: Uncharacterized protein;OS=Spinacia oleracea;|GO:0016021,
DN124584_c0_g1_i1	UP	[GO:0008236:serine-type peptidase activity]	-	-	OUZ99806.1:Peptidase S9 [Macleaya cordata]	gi|225458518|ref|XM_002284218.1|:PREDICTED: Vitis vinifera uncharacterized LOC100267853 (LOC100267853), mRNA	A0A061FC18|A0A061FC18_THECC:Acylamino-acid-releasing enzyme, putative isoform 1;OS=Theobroma cacao;|GO:0008236,

geneid	trend	GO	Pathway	TF	NR	NT	Uniprot
DN120438_c2_g1_i3	UP	[GO:0005737:cytoplasm][GO:0005524:ATP binding][GO:0016485:protein processing][GO:0009408:response to heat]	[ko04213:Longevity regulating pathway - multiple species]	Achn116231:Actinidia chinensis|ARF|ARF family protein	XP_010670181.1:PREDICTED: chaperone protein ClpB3, chloroplastic [Beta vulgaris subsp. vulgaris] >KMT17275.1 hypothetical protein BVRB_2g039750 [Beta vulgaris subsp. vulgaris]	gi|521452409|gb|GAHZ01068075.1|:TSA: Salicornia europaea Unigene82161_SeCKS transcribed RNA sequence	A0A0J8CU40|A0A0J8CU40_BETVU:Uncharacterized protein;OS=Beta vulgaris subsp. vulgaris.;|GO:0005737,|GO:0005524,|GO:0016485,GO:0009408,
DN118676_c1_g2_i1	UP	[GO:0005737:cytoplasm][GO:0050113:inositol oxygenase activity][GO:0005506:iron ion binding][GO:0019310:inositol catabolic process]	[ko00053:Ascorbate and aldarate metabolism][ko00562:Inositol phosphate metabolism]	-	XP_010688397.1:PREDICTED: inositol oxygenase 4 [Beta vulgaris subsp. vulgaris] >KMT03253.1 hypothetical protein BVRB_8g197960 [Beta vulgaris subsp. vulgaris]	gi|521663731|gb|GAIA01035390.1|:TSA: Salicornia europaea Unigene39183_Se200S transcribed RNA sequence	A0A0J8BTL0|A0A0J8BTL0_BETVU:Uncharacterized protein;OS=Beta vulgaris subsp. vulgaris.;|GO:0005737,|GO:0050113,GO:0005506,|GO:0019310,

geneid	trend	GO	Pathway	TF	NR	NT	Uniprot
DN117386_c1_g2_i4	UP	[GO:0016021:integral component of membrane]	-	-	XP_010676033.1:PREDICTED: uncharacterized protein LOC104891943 [Beta vulgaris subsp. vulgaris] >KMT12854.1 hypothetical protein BVRB_4g089460 [Beta vulgaris subsp. vulgaris]	gi|255557553|ref|XM_002519761.1|:Ricinus communis conserved hypothetical protein, mRNA	A0A0J8CLC6|A0A0J8CLC6_BETVU:Uncharacterized protein;OS=Beta vulgaris subsp. vulgaris.;|GO:0016021,
DN128162_c1_g10_i3	UP	[GO:0016765:transferase activity, transferring alkyl or aryl (other than methyl) groups]	[ko00900:Terpenoid backbone biosynthesis]	-	XP_010685566.1:PREDICTED: dehydrodolichyl diphosphate synthase 2 [Beta vulgaris subsp. vulgaris] >KMT05070.1 hypothetical protein BVRB_7g172260 [Beta vulgaris subsp. vulgaris]	-	A0A0J8BU46|A0A0J8BU46_BETVU:Alkyl transferase;OS=Beta vulgaris subsp. vulgaris.;|GO:0016765,

geneid	trend	GO	Pathway	TF	NR	NT	Uniprot
DN124778_c3_g1_i1	UP	-	-	-	-	-	-
DN117389_c1_g1_i2	UP	[GO:0016021:integral component of membrane][GO:0022857:transmembrane transporter activity]	-	GSVIVT01019717001:Vitis vinifera|ERF|ERF family protein	XP_021748182.1:WAT1related protein At4g08300like [Chenopodium quinoa]	-	A0A0K9RW05|A0A0K9RW05_SPIOL: WAT1-related protein;OS=Spinacia oleracea;|GO:0016021,|GO:0022857,
DN117808_c1_g1_i1	UP	-	-	AHYPO_008088-RA:Amaranthus hypochondriacus|bHLH|bHLH family protein	XP_010678431.1:PREDICTED: transcription factor PIF7 [Beta vulgaris subsp. vulgaris] >KMT10741.1 hypothetical protein BVRB_5g113990 [Beta vulgaris subsp. vulgaris]	-	A0A0J8F4J9|A0A0J8F4J9_BETVU:Uncharacterized protein;OS=Beta vulgaris subsp. vulgaris.;
DN126852_c4_g11_i3	UP	[GO:0016747:transferase activity, transferring acyl groups other than amino-acyl groups]	-	-	XP_021852926.1:benzyl alcohol Obenzoyltransferase like [Spinacia oleracea] >KNA08666.1	-	A0A0K9QQ73|A0A0K9QQ73_SPIOL:Uncharacterized protein;OS=Spinacia oleracea;|GO:0016747,

geneid	trend	GO	Pathway	TF	NR	NT	Uniprot
					hypothetical protein SOVF_160600 [Spinacia oleracea]		
DN127271_c2_g1_i8	UP	[GO:0005524:ATP binding][GO:0003883:CTP synthase activity][GO:0044210:'de novo' CTP biosynthetic process][GO:0006541:glutamine metabolic process]	[ko00240:Pyrimidine metabolism]	-	XP_021770131.1:CTP synthaselike [Chenopodium quinoa]	gi|521681627|gb|GAIA01026511.1|:TSA: Salicornia europaea Unigene28600_Se200S transcribed RNA sequence	A0A0J8C8A9|A0A0J8C8A9_BETVU:CTP synthase;OS=Beta vulgaris subsp. vulgaris.;|GO:0005524,GO:0003883,|GO:0044210,GO:0006541,
DN128931_c5_g2_i1	UP	[GO:0016021:integral component of membrane][GO:0005524:ATP binding][GO:0004672:protein kinase activity]	-	SMil_00011498-RA_Salv:Salvia miltiorrhiza|bHLH|bHLH family protein	XP_021756550.1:putative receptorlike protein kinase At4g00960 isoform X1 [Chenopodium quinoa]	-	A0A0L9U167|A0A0L9U167_PHAAN:Uncharacterized protein;OS=Phaseolus angularis;|GO:0016021,|GO:0005524,GO:0004672,

geneid	trend	GO	Pathway	TF	NR	NT	Uniprot
DN117808_c1_g1_i3	UP	-	-	AHYPO_008088-RA:Amaranthus hypochondriacus|bHLH|bHLH family protein	XP_010678431.1:PREDICTED: transcription factor PIF7 [Beta vulgaris subsp. vulgaris] >KMT10741.1 hypothetical protein BVRB_5g113990 [Beta vulgaris subsp. vulgaris]	-	A0A0J8F4J9|A0A0J8F4J9_BETVU:Uncharacterized protein;OS=Beta vulgaris subsp. vulgaris.;
DN109114_c0_g1_i1	UP	-	-	-	XP_021836130.1:uncharacterized protein LOC110775844 [Spinacia oleracea] >KNA23402.1 hypothetical protein SOVF_025130 [Spinacia oleracea]	-	A0A0K9RX23|A0A0K9RX23_SPIOL:Uncharacterized protein;OS=Spinacia oleracea;

geneid	trend	GO	Pathway	TF	NR	NT	Uniprot
DN126651_c3_g5_i5	UP	[GO:0009507:chloroplast][GO:0015035:protein disulfide oxidoreductase activity][GO:0045454:cell redox homeostasis][GO:0006662:glycerol ether metabolic process]	-	AA81G00050:Aethionema arabicum|HRT-like|HRT-like family protein	XP_010678488.1:PREDICTED: thioredoxinlike 12, chloroplastic [Beta vulgaris subsp. vulgaris] >KMT10788.1 hypothetical protein BVRB_5g114440 [Beta vulgaris subsp. vulgaris]	-	A0A0J8CFJ7|A0A0J8CFJ7_BETVU:Uncharacterized protein;OS=Beta vulgaris subsp. vulgaris.;|GO:0009507,|GO:0015035,|GO:0045454,GO:0006662,
DN120171_c0_g10_i1	UP	[GO:0009507:chloroplast][GO:0004497:monooxygenase activity][GO:0016984:ribulose-bisphosphate carboxylase activity][GO:0009853:photorespiration][GO:0019253:reductive pentose-phosphate cycle]	[ko00630:Glyoxylate and dicarboxylate metabolism][ko00710:Carbon fixation in photosynthetic organisms]	-	XP_021769982.1:ribulose bisphosphate carboxylase small chain 3, chloroplasticlike [Chenopodium quinoa]	gi|21570|emb|X69763.1|:S.tuberosum rbcS3 gene for ribulose-(1,5)-bisphosphate carboxylase/oxygenase small subunit	P04715|RBS2_PETHY:Ribulose bisphosphate carboxylase small chain SSU11A, chloroplastic;OS=Petunia hybrida;|GO:0009507,|GO:0004497,GO:0016984,|GO:0009853,GO:0019253,
DN67149_c0_g1_i1	UP	-	-	-	-	-	-

geneid	trend	GO	Pathway	TF	NR	NT	Uniprot
DN124499_c4_g3_i1	UP	[GO:0016021:integral component of membrane][GO:0016655:oxidoreductase activity, acting on NAD(P)H, quinone or similar compound as acceptor]	-	Zpz_sc00801.1.g00070.1.am.mk:Zoysia pacifica|bHLH|bHLH family protein	XP_010665936.1:PREDICTED: NAD(P)Hquinone oxidoreductase subunit L, chloroplastic [Beta vulgaris subsp. vulgaris]	gi|297838890|ref|XM_002887281.1|:Arabidopsis lyrata subsp. lyrata hypothetical protein, mRNA	A0A0J8B9A8|A0A0J8B9A8_BETVU:Uncharacterized protein;OS=Beta vulgaris subsp. vulgaris.;|GO:0016021,|GO:0016655,
DN116431_c0_g1_i2	UP	[GO:0016021:integral component of membrane]	-	-	XP_021725674.1:uncharacterized protein LOC110692909 [Chenopodium quinoa]	-	A0A0J8B4Y6|A0A0J8B4Y6_BETVU:Uncharacterized protein;OS=Beta vulgaris subsp. vulgaris.;|GO:0016021,
DN104544_c0_g1_i1	UP	[GO:0016021:integral component of membrane]	-	-	KVH95778.1:hypothetical protein Ccrd_002160 [Cynara cardunculus var. scolymus]	gi|478165971|gb|GAHK01010632.1|:TSA: Jatropha curcas CL4280.Contig2_JC-CK_1A transcribed RNA sequence	A0A103XRZ7|A0A103XRZ7_CYNCS:Uncharacterized protein;OS=Cynara cardunculus var. scolymus;|GO:0016021,

geneid	trend	GO	Pathway	TF	NR	NT	Uniprot
DN126696_c1_g1_i5	UP	-	-	-	XP_021748223.1:BAG family molecular chaperone regulator 6like [Chenopodium quinoa]	-	A0A0K9QPD3|A0A0K9QPD3_SPIOL: Uncharacterized protein;OS=Spinacia oleracea;
DN118211_c4_g2_i1	UP	[GO:0016021:integral component of membrane][GO:0022857:transmembrane transporter activity]	-	NNU_016725-RA:Nelumbo nucifera|GRAS|GRAS family protein	XP_021760541.1:WAT1related protein At4g08300like isoform X1 [Chenopodium quinoa]	-	A0A0J8EWU9|A0A0J8EWU9_BETVU: WAT1-related protein;OS=Beta vulgaris subsp. vulgaris.;|GO:0016021,|GO:0022857,
DN119031_c8_g3_i1	UP	[GO:0004364:glutathione transferase activity]	[ko00480:Glutathione metabolism][ko00980:Metabolism of xenobiotics by cytochrome P450][ko00982:Drug metabolism - cytochrome P450][ko01524:Platinum drug resistance][ko05204:	-	XP_008226256.1:PREDICTED: probable glutathione Stransferase parC [Prunus mume]	-	O49821|O49821_CARPA:Glutathione transferase;OS=Carica papaya;|GO:0004364,

geneid	trend	GO	Pathway	TF	NR	NT	Uniprot
			Chemical carcinogenesis][ko05418:Fluid shear stress and atherosclerosis]				
DN106982_c0_g1_i1	UP	-	-	-	-	-	-
DN128623_c7_g1_i3	UP	[GO:0008146:sulfotransferase activity]	-	676777512:Sisymbrium irio|ERF|ERF family protein	XP_021839049.1:cytosolic sulfotransferase 5like [Spinacia oleracea]	-	A0A0K9R9P6|A0A0K9R9P6_SPIOL:Sulfotransferase;OS=Spinacia oleracea;|GO:0008146,
DN126024_c8_g9_i1	UP	-	-	-	-	-	-

geneid	trend	GO	Pathway	TF	NR	NT	Uniprot
DN127253_c0_g3_i1	UP	[GO:0009733:response to auxin]	-	Rsa1.0_03425.1_g00001.1:Raphanus sativus|ZF-HD|ZF-HD family protein	XP_021716071.1:auxinresponsive protein SAUR41like [Chenopodium quinoa]	-	A0A0J8EP37|A0A0J8EP37_BETVU:Uncharacterized protein;OS=Beta vulgaris subsp. vulgaris.;|GO:0009733,
DN116167_c5_g10_i1	UP	-	-	-	-	-	-
DN115546_c0_g1_i5	UP	-	-	-	-	-	-
DN126831_c2_g4_i1	UP	[GO:0016021:integral component of membrane][GO:0005524:ATP binding][GO:0004672:protein kinase activity]	-	Lsa000376:Lactuca sativa|Dof|Dof family protein	XP_010693989.1:PREDICTED: receptorlike protein kinase FERONIA isoform X1 [Beta vulgaris subsp. vulgaris] >XP_010693992.1 PREDICTED: receptorlike protein kinase FERONIA isoform X2 [Beta vulgaris subsp.	gi|356568797|ref|XM_003552547.1|:PREDICTED: Glycine max receptor-like protein kinase FERONIA-like (LOC100780062), mRNA	A0A0J8D2Y2|A0A0J8D2Y2_BETVU:Uncharacterized protein;OS=Beta vulgaris subsp. vulgaris.;|GO:0016021,|GO:0005524,GO:0004672,

geneid	trend	GO	Pathway	TF	NR	NT	Uniprot
					vulgaris]		
DN127884_c2_g1_i1	UP	[GO:0003676:nucleic acid binding]	-	-	KMT04760.1:hypothetical protein BVRB_7g169390 [Beta vulgaris subsp. vulgaris]	-	A0A0J8BT86|A0A0J8BT86_BETVU:Uncharacterized protein;OS=Beta vulgaris subsp. vulgaris.;|GO:0003676,

geneid	trend	GO	Pathway	TF	NR	NT	Uniprot
DN128718_c4_g7_i1	UP	[GO:0005576:extracellular region][GO:0008061:chitin binding][GO:0004568:chitinase activity][GO:0005975:carbohydrate metabolic process][GO:0006032:chitin catabolic process]	[ko00520:Amino sugar and nucleotide sugar metabolism]	-	OIV99861.1:hypothetical protein TanjilG_26199 [Lupinus angustifolius]	-	F6HCL5|F6HCL5_VITVI:Putative uncharacterized protein;OS=Vitis vinifera;GN=VIT_11s0149g00380 {ECO:0000313|EMBL:CCB49960.1};|GO:0005576,|GO:0008061,GO:0004568,|GO:0005975,GO:0006032,
DN128396_c4_g11_i1	UP	-	-	Tp57577_TGAC_v2_mRNA39490:Trifolium pratense|NAC|NAC family protein	ACL81159.1:putative auxin/aluminumresponsive protein [Mirabilis jalapa]	gi|521538450|gb|GAHZ01004063.1|:TSA: Salicornia europaea Unigene4352_SeCKS transcribed RNA sequence	B8YIB1|B8YIB1_MIRJA:Putative auxin/aluminum-responsive protein;OS=Mirabilis jalapa;
DN114101_c0_g2_i1	UP	[GO:0016021:integral component of membrane][GO:0005886:plasma membrane]	-	-	XP_021867311.1:CASPlike protein 1E1 [Spinacia oleracea]	-	A0A0J8B8K3|A0A0J8B8K3_BETVU:CASP-like protein;OS=Beta vulgaris subsp. vulgaris.;|GO:0016021,GO:0005886,

geneid	trend	GO	Pathway	TF	NR	NT	Uniprot
DN127577_c6_g2_i1	UP	[GO:0005737:cytoplasm][GO:0003872:6-phosphofructokinase activity][GO:0005524:ATP binding][GO:0046872:metal ion binding][GO:0006002:fructose 6-phosphate metabolic process]	[ko00010:Glycolysis / Gluconeogenesis][ko00030:Pentose phosphate pathway][ko00051:Fructose and mannose metabolism][ko00052:Galactose metabolism][ko00680:Methane metabolism][ko03018:RNA degradation][ko04152:AMPK signaling pathway][ko05230:Central carbon metabolism in cancer]	Lj5g3v1697360.1:Lotus japonicus|ERF|ERF family protein	XP_009344489.1:PREDICTED: ATPdependent 6phosphofructokinase 6like [Pyrus x bretschneideri]	gi|521472743|gb|GAHZ01058142.1|:TSA: Salicornia europaea Unigene69403_SeCKS transcribed RNA sequence	A0A0J8BL20|A0A0J8BL20_BETVU:ATP-dependent 6-phosphofructokinase;OS=Beta vulgaris subsp. vulgaris.;|GO:0005737,|GO:0003872,GO:0005524,GO:0046872,|GO:0006002,
DN121629_c0_g2_i3	UP	[GO:0005622:intracellular][GO:0008270:zinc ion binding]	-	AHYPO_012079-RA:Amaranthus hypochondriacus|DBB|DBB family protein	XP_021753490.1:Bbox zinc finger protein 19like isoform X2 [Chenopodium quinoa]	-	A0A0J8BJT3|A0A0J8BJT3_BETVU:Uncharacterized protein;OS=Beta vulgaris subsp. vulgaris.;|GO:00056

geneid	trend	GO	Pathway	TF	NR	NT	Uniprot
							22,|GO:0008270,
DN126024_c8_g8_i1	UP	-	-	-	-	-	-
DN122970_c0_g6_i4	UP	[GO:0016787:hydrolase activity]	-	Zjn_sc00009.1.g01550.1.am.mk:Zoysia japonica|C2H2|C2H2 family protein	XP_010244542.1:PREDICTED: methylesterase 17like [Nelumbo nucifera]	-	B9N1T8|B9N1T8_POPTR:Hydrolase family protein;OS=Populus trichocarpa;|GO:0016787,
DN127036_c2_g1_i3	UP	-	-	-	-	-	-
DN117820_c2_g2_i1	UP	-	[ko04075:Plant hormone signal transduction]	-	XP_021729590.1:BRI1 kinase inhibitor 1like [Chenopodium quinoa]	gi|478161144|gb|GAHK01015459.1|:TSA: Jatropha curcas Unigene2913_JC-CK_1A transcribed RNA sequence	A0A0K9RGD0|A0A0K9RGD0_SPIOL: Uncharacterized protein;OS=Spinacia oleracea;

geneid	trend	GO	Pathway	TF	NR	NT	Uniprot
DN119920_c2_g5_i8	UP	-	[ko00902:Monoterpenoid biosynthesis]	Niben101Scf02581g01014.1:Nicotiana benthamiana|MYB_related|MYB_related family protein	XP_021760280.1:(+)neomenthol dehydrogenaselike [Chenopodium quinoa] >XP_021717522.1 (+)neomenthol dehydrogenaselike [Chenopodium quinoa]	gi|255541519|ref|XM_002511778.1|:Ricinus communis carbonyl reductase, putative, mRNA	A0A0K9R8D1|A0A0K9R8D1_SPIOL:Uncharacterized protein;OS=Spinacia oleracea;
DN121515_c0_g3_i2	UP	[GO:0016021:integral component of membrane]	-	Dca41434.1:Dianthus caryophyllus|HD-ZIP|HD-ZIP family protein	XP_021835340.1:uncharacterized membrane protein At1g16860 [Spinacia oleracea] >KNA25592.1 hypothetical protein SOVF_005190 [Spinacia oleracea]	gi|521484083|gb|GAHZ01046803.1|:TSA: Salicornia europaea Unigene55159_SeCKS transcribed RNA sequence	A0A0K9S381|A0A0K9S381_SPIOL:Uncharacterized protein;OS=Spinacia oleracea;|GO:0016021,

geneid	trend	GO	Pathway	TF	NR	NT	Uniprot
DN115936_c2_g2_i5	UP	[GO:0009507:chloroplast][GO:0047714:galactolipase activity][GO:0008970:phospholipase A1 activity][GO:0004806:triglyceride lipase activity][GO:0006629:lipid metabolic process]	-	-	XP_010673678.1:PREDICTED: phospholipase A1Igamma3, chloroplastic [Beta vulgaris subsp. vulgaris]	gi|224104168|ref|XM_002313309.1|:Populus trichocarpa predicted protein, mRNA	A0A0J8CRA1|A0A0J8CRA1_BETVU: Uncharacterized protein;OS=Beta vulgaris subsp. vulgaris.;|GO:0009507,|GO:0047714,GO:0008970,GO:0004806,|GO:0006629,
DN126232_c0_g4_i3	UP	[GO:0004553:hydrolase activity, hydrolyzing O-glycosyl compounds][GO:0005975:carbohydrate metabolic process]	[ko00520:Amino sugar and nucleotide sugar metabolism]	-	XP_021858327.1:acidic endochitinase SE2 [Spinacia oleracea] >KNA08634.1 hypothetical protein SOVF_160890 [Spinacia oleracea]	-	A0A0K9QN10|A0A0K9QN10_SPIOL:Uncharacterized protein;OS=Spinacia oleracea;|GO:0004553,|GO:0005975,
DN127197_c1_g1_i1	UP	[GO:0016021:integral component of membrane][GO:0015297:antiporter activity]	-	Zpz_sc01010.1.g00110.1.am.mk:Zoysia pacifica|MYB|MYB family protein	XP_021743366.1:protein DETOXIFICATION 24like isoform X1 [Chenopodium quinoa]	-	A0A0J8CB22|A0A0J8CB22_BETVU:Uncharacterized protein;OS=Beta vulgaris subsp. vulgaris.;|GO:0016021,|GO:0015297,GO:0015238,

geneid	trend	GO	Pathway	TF	NR	NT	Uniprot
DN127312_c4_g20_i1	UP	[GO:0005524:ATP binding]	-	-	XP_014504832.1:PREDICTED: ribulose bisphosphate carboxylase/oxygenase activase, chloroplasticlike [Vigna radiata var. radiata]	gi|21950711|gb|AY117142.1|:Chenopodium quinoa rubisco activase (rca) mRNA, complete cds	A0A0L9UWT4|A0A0L9UWT4_PHAAN:Uncharacterized protein;OS=Phaseolus angularis;|GO:0005524,
DN125839_c0_g4_i7	UP	-	-	-	-	-	-
DN122406_c0_g1_i4	UP	[GO:0005829:cytosol][GO:0005634:nucleus]	-	-	XP_010689282.1:PREDICTED: nitrilespecifier protein 5 [Beta vulgaris subsp. vulgaris] >KMT02267.1 hypothetical protein BVRB_9g206540 [Beta vulgaris subsp. vulgaris]	-	A0A0J8BM28|A0A0J8BM28_BETVU:Uncharacterized protein;OS=Beta vulgaris subsp. vulgaris.;|GO:0005829,GO:0005634,

geneid	trend	GO	Pathway	TF	NR	NT	Uniprot
DN128775_c1_g1_i17	UP	-	[ko00480:Glutathione metabolism][ko00980:Metabolism of xenobiotics by cytochrome P450][ko00982:Drug metabolism - cytochrome P450][ko01524:Platinum drug resistance][ko05204:Chemical carcinogenesis][ko05418:Fluid shear stress and atherosclerosis]	-	XP_021638097.1:glutathione Stransferase L3like isoform X2 [Hevea brasiliensis]	gi|509467285|gb|GAJL01014896.1|:TSA: Triticum aestivum TA_RNA_121579 transcribed RNA sequence	M5WH15|M5WH15_PRUPE:Uncharacterized protein;OS=Prunus persica;

geneid	trend	GO	Pathway	TF	NR	NT	Uniprot
DN125946_c2_g1_i5	UP	[GO:0016021:integral component of membrane][GO:0005507:copper ion binding][GO:0008131:primary amine oxidase activity][GO:0048038:quinone binding][GO:0009308:amine metabolic process]	[ko00260:Glycine, serine and threonine metabolism][ko00350:Tyrosine metabolism][ko00360:Phenylalanine metabolism][ko00410:beta-Alanine metabolism][ko00950:Isoquinoline alkaloid biosynthesis][ko00960:Tropane, piperidine and pyridine alkaloid biosynthesis]	-	XP_010676213.1:PREDICTED: primary amine oxidase [Beta vulgaris subsp. vulgaris] >KMT12584.1 hypothetical protein BVRB_5g098280 [Beta vulgaris subsp. vulgaris]	gi|460400388|ref|XM_004245669.1|:PREDICTED: Solanum lycopersicum copper-containing amine oxidase (cuAO), mRNA	A0A0J8CKH2|A0A0J8CKH2_BETVU:Amine oxidase;OS=Beta vulgaris subsp. vulgaris.;|GO:0016021,|GO:0005507,GO:0008131,GO:0048038,|GO:0009308,
DN124774_c1_g1_i4	UP	-	[ko04141:Protein processing in endoplasmic reticulum]	WALNUT_00030998-RA:Juglans regia|NF-YB|NF-YB family protein	XP_002533590.1:PREDICTED: 18.1 kDa class I heat shock protein [Ricinus communis] >EEF28795.1 heatshock protein, putative [Ricinus communis]	-	B9T5S1|B9T5S1_RICCO:Heat-shock protein, putative;OS=Ricinus communis;

geneid	trend	GO	Pathway	TF	NR	NT	Uniprot
DN127023_c1_g1_i6	UP	[GO:0005737:cytoplasm][GO:0003999:adenine phosphoribosyltransferase activity][GO:0006168:adenine salvage][GO:0009116:nucleoside metabolic process]	[ko00230:Purine metabolism]	Oropetium_20150105_18128A:Oropetium thomaeum|bHLH|bHLH family protein	XP_021769363.1:adenine phosphoribosyltransferase 5like [Chenopodium quinoa]	gi|521698930|gb|GAIA01017969.1|:TSA: Salicornia europaea Unigene18923_Se200S transcribed RNA sequence	A0A0K9QSX0|A0A0K9QSX0_SPIOL: Uncharacterized protein;OS=Spinacia oleracea;|GO:0005737,|GO:0003999,|GO:0006168,GO:0009116,
DN112645_c1_g2_i1	UP	[GO:0009535:chloroplast thylakoid membrane][GO:0016021:integral component of membrane][GO:0009522:photosystem I][GO:0009523:photosystem II][GO:0016168:chlorophyll binding][GO:0046872:metal ion binding][GO:0009765:photosynthesis, light harvesting][GO:0018298:protein-chromophore linkage]	[ko00196:Photosynthesis - antenna proteins]	-	XP_010677168.1:PREDICTED: chlorophyll ab binding protein CP29.3, chloroplastic [Beta vulgaris subsp. vulgaris] >KMT11857.1 hypothetical protein BVRB_5g105530 [Beta vulgaris subsp. vulgaris]	gi|521491958|gb|GAHZ01038928.1|:TSA: Salicornia europaea Unigene44095_SeCKS transcribed RNA sequence	A0A0J8CEA0|A0A0J8CEA0_BETVU:Chlorophyll a-b binding protein, chloroplastic;OS=Beta vulgaris subsp. vulgaris.;|GO:0009535,GO:0016021,GO:0009522,GO:0009523,|GO:0016168,GO:0046872,|GO:0009765,GO:0018298,

geneid	trend	GO	Pathway	TF	NR	NT	Uniprot
DN121311_c2_g1_i2	UP	[GO:0005524:ATP binding][GO:0006457:protein folding][GO:0006950:response to stress]	[ko04141:Protein processing in endoplasmic reticulum][ko04151:PI3K-Akt signaling pathway][ko04217:Necroptosis][ko04612:Antigen processing and presentation][ko04621:NOD-like receptor signaling pathway][ko04626:Plant-pathogen interaction][ko04657:IL-17 signaling pathway][ko04659:Th17 cell differentiation][ko04914:Progesterone-mediated oocyte maturation][ko04915:Estrogen signaling pathway][ko05200:Pathways in	Do001279.1:Dichanthelium oligosanthes|bZIP|bZIP family protein	XP_011084460.1:heat shock protein 83like [Sesamum indicum]	gi|225464588|ref|XM_002273986.1|:PREDICTED: Vitis vinifera heat shock protein 83-like (LOC100257736), mRNA	A0A151U689|A0A151U689_CAJCA:Heat shock protein 83;OS=Cajanus cajan;|GO:0005524,|GO:0006457,GO:0006950,

geneid	trend	GO	Pathway	TF	NR	NT	Uniprot
			cancer][ko05215:Prostate cancer][ko05418:Fluid shear stress and atherosclerosis]				
DN129113_c3_g5_i2	UP	-	-	-	-	-	-

geneid	trend	GO	Pathway	TF	NR	NT	Uniprot
DN123098_c2_g1_i1	UP	-	-	-	XP_021772971.1:protein FANTASTIC FOUR 1like [Chenopodium quinoa]	-	A0A0K9QD42|A0A0K9QD42_SPIOL:Uncharacterized protein;OS=Spinacia oleracea;
DN105065_c0_g1_i1	UP	[GO:0010207:photosystem II assembly]	[ko00195:Photosynthesis]	-	XP_016169226.1:photosystem II repair protein PSB27H1, chloroplastic [Arachis ipaensis]	-	A0A162A634|A0A162A634_DAUCA:Uncharacterized protein;OS=Daucus carota subsp. sativus.;|GO:0010207,
DN128003_c0_g1_i1	UP	[GO:0043531:ADP binding][GO:0006952:defense response]	[ko04626:Plant-pathogen interaction]	MDP0000578301:Malus domestica|WRKY|WRKY family protein	XP_021725257.1:LOW QUALITY PROTEIN: probable disease resistance protein At1g12280 [Chenopodium quinoa]	-	A0A0J8CHJ0|A0A0J8CHJ0_BETVU:Uncharacterized protein;OS=Beta vulgaris subsp. vulgaris.;|GO:0043531,|GO:0006952,

geneid	trend	GO	Pathway	TF	NR	NT	Uniprot
DN125907_c2_g5_i1	UP	[GO:0016021:integral component of membrane][GO:0005886:plasma membrane][GO:0008515:sucrose transmembrane transporter activity]	-	Bv9_221290_hwtg.t1:Beta vulgaris|bHLH|bHLH family protein	XP_010678287.1:PREDICTED: bidirectional sugar transporter SWEET12 [Beta vulgaris subsp. vulgaris] >KMT10957.1 hypothetical protein BVRB_5g112900 [Beta vulgaris subsp. vulgaris]	gi|255549907|ref|XM_002515959.1|:Ricinus communis transcription factor, putative, mRNA	A0A0J8CBG8|A0A0J8CBG8_BETVU: Bidirectional sugar transporter SWEET;OS=Beta vulgaris subsp. vulgaris.;|GO:0016021,GO:0005886,|GO:0008515,
DN122746_c3_g1_i1	UP	[GO:0051537:2 iron, 2 sulfur cluster binding][GO:0010277:chlorophyllide a oxygenase [overall] activity]	-	-	XP_010673485.1:PREDICTED: protochlorophyllide dependent translocon component 52, chloroplastic [Beta vulgaris subsp. vulgaris] >KMT14327.1 hypothetical protein BVRB_4g071110 [Beta vulgaris subsp. vulgaris]	gi|521513233|gb|GAHZ01017687.1|:TSA: Salicornia europaea Unigene18627_SeCKS transcribed RNA sequence	A0A0J8CLF4|A0A0J8CLF4_BETVU:Uncharacterized protein;OS=Beta vulgaris subsp. vulgaris.;|GO:0051537,GO:0010277,

geneid	trend	GO	Pathway	TF	NR	NT	Uniprot
DN114772_c0_g1_i3	UP	[GO:0005829:cytosol][GO:0005634:nucleus][GO:0070694:deoxyribonucleoside 5'-monophosphate N-glycosidase activity][GO:0043733:DNA-3-methyl base glycosylase activity][GO:0016799:hydrolase activity, hydrolyzing N-glycosyl compounds][GO:0070635:nicotinamide riboside hydrolase activity][GO:0070636:nicotinic acid riboside hydrolase activity][GO:0000701:purine-specific mismatch base pair DNA N-glycosylase activity][GO:0017065:single-strand selective uracil DNA N-glycosylase activity][GO:0009691:cytokinin biosynthetic process]	-	Vang05g09490.1:Vigna angularis|WRKY|WRKY family protein	XP_019156593.1:PREDICTED: cytokinin riboside 5'monophosphate phosphoribohydrolase LOG1like [Ipomoea nil]	gi|521515004|gb|GAHZ01016427.1|:TSA: Salicornia europaea Unigene17294_SeCKS transcribed RNA sequence	E0CPM6|E0CPM6_VITVI:Cytokinin riboside 5'-monophosphate phosphoribohydrolase;OS=Vitis vinifera;GN=VIT_18s0001g14030{ECO:0000313|EMBL:CBI19842.3};|GO:0005829,GO:0005634,|GO:0070694,GO:0043733,GO:0016799,GO:0070635,GO:0070636,GO:0000701,GO:0017065,|GO:0009691,

geneid	trend	GO	Pathway	TF	NR	NT	Uniprot
DN120025_c0_g5_i6	UP	-	-	-	XP_010682446.1:PREDICTED: uncharacterized protein LOC104897290 [Beta vulgaris subsp. vulgaris] >XP_010682449.1 PREDICTED: uncharacterized protein LOC104897290 [Beta vulgaris subsp. vulgaris] >XP_010682451.1 PREDICTED: uncharacterized protein LOC104897290 [Beta vulgaris subsp. vulgaris] >XP_019105947.1 PREDICTED: uncharacterized protein	-	A0A0J8C6P1|A0A0J8C6P1_BETVU:Uncharacterized protein;OS=Beta vulgaris subsp. vulgaris.;

geneid	trend	GO	Pathway	TF	NR	NT	Uniprot
					LOC104897290 [Beta vulgaris subsp. vulgaris] >KMT07713.1 hypothetical protein BVRB_6g148340 [Beta vulgaris subsp. vulgaris]		

geneid	trend	GO	Pathway	TF	NR	NT	Uniprot
DN125374_c2_g1_i1	UP	-	-	-	KNA16179.1:hypothetical protein SOVF_091520 [Spinacia oleracea]	gi|356503512|ref|XM_003520504.1|:PREDICTED: Glycine max uncharacterized protein LOC100794761 (LOC100794761), mRNA	A0A0K9RAZ8|A0A0K9RAZ8_SPIOL: Uncharacterized protein;OS=Spinacia oleracea;
DN126109_c4_g1_i3	UP	[GO:0016787:hydrolase activity][GO:0008152:metabolic process]	-	-	XP_021762045.1:CBBYlike protein [Chenopodium quinoa]	gi|460188620|gb|GABL01070893.1|:TSA: Camelina sativa comp60680_c1_seq1 transcribed RNA sequence	A0A0K9QH67|A0A0K9QH67_SPIOL:Uncharacterized protein;OS=Spinacia oleracea;|GO:0016787,|GO:0008152,
DN118188_c1_g1_i1	UP	[GO:0046872:metal ion binding][GO:0030001:metal ion transport]	-	-	XP_021754236.1:heavy metalassociated isoprenylated plant protein 39like [Chenopodium quinoa]	-	A0A0K9RME2|A0A0K9RME2_SPIOL: Uncharacterized protein;OS=Spinacia oleracea;|GO:0046872,|GO:0030001,

geneid	trend	GO	Pathway	TF	NR	NT	Uniprot
DN127759_c2_g1_i8	UP	[GO:0004089:carbonate dehydratase activity][GO:0008270:zinc ion binding][GO:0006730:one-carbon metabolic process]	[ko00910:Nitrogen metabolism]	Sopen10g033340.1: Solanum pennellii|MYB|MYB family protein	XP_021774553.1:alpha carbonic anhydrase 7like [Chenopodium quinoa] >XP_021775735.1 alpha carbonic anhydrase 7like [Chenopodium quinoa]	-	A0A0J8B744|A0A0J8B744_BETVU:Uncharacterized protein;OS=Beta vulgaris subsp. vulgaris.;|GO:0004089,GO:0008270,|GO:0006730,
DN89745_c0_g1_i1	UP	[GO:0005634:nucleus][GO:0043565:sequence-specific DNA binding][GO:0006355:regulation of transcription, DNA-templated]	-	Cc06_g04990:Coffea canephora|HD-ZIP|HD-ZIP family protein	GAV63810.1:Homeobox domaincontaining protein/HALZ domaincontaining protein [Cephalotus follicularis]	-	A0A068V448|A0A068V448_COFCA:Uncharacterized protein;OS=Coffea canephora;|GO:0005634,|GO:0043565,|GO:0006355,
DN124790_c1_g3_i6	UP	[GO:0031011:Ino80 complex][GO:0006338:chromatin remodeling]	-	-	XP_021721805.1:actinrelated protein 9like isoform X2 [Chenopodium quinoa]	gi|521493760|gb|GAHZ01037160.1|:TSA: Salicornia europaea Unigene41903_SeCKS transcribed RNA sequence	A0A0K9RVQ4|A0A0K9RVQ4_SPIOL: Uncharacterized protein;OS=Spinacia oleracea;|GO:0031011,|GO:0006338,
DN125651_c	UP	-	-	-	-	-	-

geneid	trend	GO	Pathway	TF	NR	NT	Uniprot
7_g4_i1							
DN128494_c1_g5_i2	UP	-	-	cra_locus_5362_iso_4:Catharanthus roseus|MYB_related|MYB_related family protein	XP_021750054.1:protein TSSlike [Chenopodium quinoa]	gi|521658724|gb|GAIA01037973.1|:TSA: Salicornia europaea Unigene42502_Se200S transcribed RNA sequence	A0A0J8BU18|A0A0J8BU18_BETVU:Uncharacterized protein;OS=Beta vulgaris subsp. vulgaris.;
DN120349_c1_g2_i1	UP	-	-	EMT27370:Aegilops tauschii|GATA|GATA family protein	XP_021865165.1:pentatricopeptide repeatcontaining protein At1g02150 [Spinacia oleracea] >KNA14339.1 hypothetical protein SOVF_108240 [Spinacia oleracea]	gi|521444504|gb|GAHZ01075980.1|:TSA: Salicornia europaea Unigene92908_SeCKS transcribed RNA sequence	A0A0K9R5X2|A0A0K9R5X2_SPIOL:Uncharacterized protein;OS=Spinacia oleracea;
DN124231_c1_g3_i3	UP	[GO:0016021:integral component of membrane]	-	Zmw_sc06582.1.g00020.1:Zoysia matrella|C2H2|C2H2 family protein	XP_021857934.1:sulfite exporter TauE/SafE family protein 4 [Spinacia oleracea] >KNA13303.1 hypothetical protein SOVF_117870	gi|521746996|gb|GAIA01002365.1|:TSA: Salicornia europaea Unigene2418_Se200S transcribed RNA sequence	A0A0K9R1B8|A0A0K9R1B8_SPIOL:Uncharacterized protein;OS=Spinacia oleracea;|GO:0016021,

geneid	trend	GO	Pathway	TF	NR	NT	Uniprot
					[Spinacia oleracea]		
DN122123_c0_g2_i11	UP	[GO:0016021:integral component of membrane]	-	Medtr0036s0260.1:Medicago truncatula|MYB_related|MYB_related family protein	XP_021776168.1:probable zinc metallopeptidase EGY3, chloroplastic [Chenopodium quinoa]	gi|521663387|gb|GAIA01035561.1|:TSA: Salicornia europaea Unigene39377_Se200S transcribed RNA sequence	A0A0J8BLU0|A0A0J8BLU0_BETVU:Uncharacterized protein;OS=Beta vulgaris subsp. vulgaris.;|GO:0016021,
DN119512_c4_g1_i6	UP	[GO:0051287:NAD binding][GO:0050661:NADP binding][GO:0016620:oxidoreductase activity, acting on the aldehyde or oxo group of donors, NAD or NADP as acceptor][GO:0006006:glucose metabolic process]	[ko00710:Carbon fixation in photosynthetic organisms]	MDP0000275412:Malus domestica|Trihelix|Trihelix family protein	XP_012464265.1:PREDICTED: glyceraldehyde3phosphate dehydrogenase B, chloroplastic [Gossypium raimondii] >KJB14201.1 hypothetical protein B456_002G114000 [Gossypium	gi|224086077|ref|XM_002307770.1|:Populus trichocarpa predicted protein, mRNA	A0A0D2QBD6|A0A0D2QBD6_GOSRA:Glyceraldehyde-3-phosphate dehydrogenase;OS=Gossypium raimondii;|GO:0051287,GO:0050661,GO:0016620,|GO:0006006,

geneid	trend	GO	Pathway	TF	NR	NT	Uniprot
					raimondii]		
DN124922_c1_g5_i5	UP	[GO:0016021:integral component of membrane][GO:0005524:ATP binding][GO:0004672:protein kinase activity]	[ko04064:NF-kappa B signaling pathway][ko04620:Toll-like receptor signaling pathway][ko04621:NOD-like receptor signaling pathway][ko04624:Toll and Imd signaling pathway][ko04722:Neurotrophin signaling pathway][ko05133:Pertussis][ko05140:L	312791:Arabidopsis lyrata|bHLH|bHLH family protein	XP_010672642.1:PREDICTED: LEAF RUST 10 DISEASERESISTANCE LOCUS RECEPTORLIKE PROTEIN KINASElike 1.2 [Beta vulgaris subsp. vulgaris] >KMT15302.1 hypothetical protein BVRB_3g060110 [Beta vulgaris subsp. vulgaris]	-	A0A0J8CP48|A0A0J8CP48_BETVU:Uncharacterized protein;OS=Beta vulgaris subsp. vulgaris.;|GO:0016021,|GO:0005524,GO:0004672,

geneid	trend	GO	Pathway	TF	NR	NT	Uniprot
			eishmaniasis][ko05142:Chagas disease (American trypanosomiasis)][ko05145:Toxoplasmosis][ko05152:Tuberculosis][ko05162:Measles][ko05164:Influenza A]				

geneid	trend	GO	Pathway	TF	NR	NT	Uniprot
DN120503_c2_g2_i10	UP	[GO:0005634:nucleus][GO:0003677:DNA binding][GO:0003700:DNA-binding transcription factor activity][GO:0007275:multicellular organism development][GO:0006351:transcription, DNA-templated]	-	Sp_192560_dzus.t1:Spinacia oleracea|AP2|AP2 family protein	XP_010672391.1:PREDICTED: AP2like ethyleneresponsive transcription factor At2g41710 [Beta vulgaris subsp. vulgaris] >XP_010672863.1 PREDICTED: AP2like ethyleneresponsive transcription factor At2g41710 [Beta vulgaris subsp. vulgaris]	gi|521556079|gb|GAIA01079519.1|:TSA: Salicornia europaea Unigene96590_Se200S transcribed RNA sequence	A0A0K9QD46|A0A0K9QD46_SPIOL:Uncharacterized protein;OS=Spinacia oleracea;|GO:0005634,|GO:0003677,GO:0003700,|GO:0007275,GO:0006351,
DN115206_c1_g1_i2	UP	-	-	-	XP_021858559.1:uncharacterized protein LOC110797753 [Spinacia oleracea] >KNA16276.1 hypothetical protein SOVF_090600 [Spinacia oleracea]	-	A0A0K9R9U1|A0A0K9R9U1_SPIOL:Uncharacterized protein;OS=Spinacia oleracea;

geneid	trend	GO	Pathway	TF	NR	NT	Uniprot
DN127616_c4_g1_i1	UP	-	[ko04137:Mitophagy - animal][ko04217:Necroptosis][ko04668:TNF signaling pathway]	-	XP_019106672.1:PREDICTED: probable 2carboxyDarabinitol 1phosphatase [Beta vulgaris subsp. vulgaris] >KMT04244.1 hypothetical protein BVRB_8g183200 [Beta vulgaris subsp. vulgaris]	gi|521666413|gb|GAIA01034331.1|:TSA: Salicornia europaea Unigene38004_Se200S transcribed RNA sequence	A0A0J8BW62|A0A0J8BW62_BETVU: Uncharacterized protein;OS=Beta vulgaris subsp. vulgaris.;
DN122908_c1_g2_i8	UP	[GO:0004089:carbonate dehydratase activity][GO:0008270:zinc ion binding][GO:0015976:carbon utilization]	[ko00910:Nitrogen metabolism]	Jcr4S00223.20:Jatropha curcas|bHLH|bHLH family protein	AEV42276.1:chloroplast beta carbonic anhydrase 1 [Mesembryanthemum nodiflorum]	gi|359372871|gb|JN228098.1|:Mesembryanthemum nodiflorum chloroplast beta carbonic anhydrase 1 (CA1) mRNA, complete cds; nuclear gene for chloroplast product	G9FZ35|G9FZ35_MESNO:Carbonic anhydrase;OS=Mesembryanthemum nodiflorum;|GO:0004089,GO:0008270,|GO:0015976,

geneid	trend	GO	Pathway	TF	NR	NT	Uniprot
DN120869_c5_g1_i1	UP	[GO:0005634:nucleus][GO:0003677:DNA binding][GO:0003700:DNA-binding transcription factor activity][GO:0006351:transcription, DNA-templated]	-	Bv6_142620_qrgc.t1:Beta vulgaris|RAV|RAV family protein	XP_010681667.1:PREDICTED: AP2/ERF and B3 domaincontaining transcription factor RAV1 [Beta vulgaris subsp. vulgaris] >KMT08208.1 hypothetical protein BVRB_6g142620 [Beta vulgaris subsp. vulgaris]	gi|356576288|ref|XM_003556217.1|:PREDICTED: Glycine max AP2/ERF and B3 domain-containing transcription repressor TEM1-like (LOC100789009), mRNA	A0A0J8C877|A0A0J8C877_BETVU:Uncharacterized protein;OS=Beta vulgaris subsp. vulgaris.;|GO:0005634,|GO:0003677,GO:0003700,|GO:0006351,
DN127818_c1_g1_i1	UP	-	-	-	XP_021766820.1:plastidlipidassociated protein, chloroplasticlike [Chenopodium quinoa]	-	A0A0K9QBN7|A0A0K9QBN7_SPIOL:Uncharacterized protein;OS=Spinacia oleracea;
DN122945_c4_g3_i1	UP	[GO:0016021:integral component of membrane][GO:0005524:ATP binding][GO:0004674:protein serine/threonine kinase activity]	-	Sme2.5_00580.1_g00006.1:Solanum melongena|CO-like|CO-like family protein	XP_021746530.1:rust resistance kinase Lr10like isoform X2 [Chenopodium quinoa]	-	A0A0J8C2K3|A0A0J8C2K3_BETVU:Uncharacterized protein;OS=Beta vulgaris subsp. vulgaris.;|GO:00160

geneid	trend	GO	Pathway	TF	NR	NT	Uniprot
							21,|GO:0005524,GO:0004674,
DN124231_c1_g3_i4	UP	[GO:0016021:integral component of membrane]	-	Zmw_sc06582.1.g00020.1:Zoysia matrella|C2H2|C2H2 family protein	XP_021624293.1:sulfite exporter TauE/SafE family protein 4like isoform X1 [Manihot esculenta]	gi|357440250|ref|XM_003590355.1|:Medicago truncatula hypothetical protein (MTR_1g061710) mRNA, complete cds	A0A0K9R1B8|A0A0K9R1B8_SPIOL:Uncharacterized protein;OS=Spinacia oleracea;|GO:0016021,
DN126792_c1_g4_i1	UP	-	-	-	-	-	-
DN123251_c0_g2_i2	UP	[GO:0005634:nucleus][GO:0043565:sequence-specific DNA binding][GO:0003700:DNA-binding transcription factor activity]	-	Bv6_154690_swdf.t1:Beta vulgaris|HD-ZIP|HD-ZIP family protein	XP_010683269.1:PREDICTED: homeoboxleucine zipper protein HAT5 isoform X1 [Beta vulgaris subsp. vulgaris] >KMT07141.1 hypothetical protein	-	A0A0J8EVD7|A0A0J8EVD7_BETVU:Uncharacterized protein;OS=Beta vulgaris subsp. vulgaris.;|GO:0005634,|GO:0043565,GO:0003700,

geneid	trend	GO	Pathway	TF	NR	NT	Uniprot
					BVRB_6g154690 [Beta vulgaris subsp. vulgaris]		
DN113843_c0_g1_i2	UP	-	-	-	XP_010665890.1:PREDICTED: uncharacterized protein LOC104883135 [Beta vulgaris subsp. vulgaris] >KMS96479.1 hypothetical protein BVRB_9g224720 [Beta vulgaris subsp. vulgaris]	-	A0A0J8B5G6|A0A0J8B5G6_BETVU:Uncharacterized protein;OS=Beta vulgaris subsp. vulgaris.;

geneid	trend	GO	Pathway	TF	NR	NT	Uniprot
DN124231_c0_g2_i1	UP	-	-	-	XP_021854875.1:EG45like domain containing protein [Spinacia oleracea] >KNA16851.1 hypothetical protein SOVF_085510 [Spinacia oleracea]	-	A0A0K9RBG4|A0A0K9RBG4_SPIOL: Uncharacterized protein;OS=Spinacia oleracea;
DN122236_c3_g6_i5	UP	[GO:0016021:integral component of membrane][GO:0042132:fructose 1,6-bisphosphate 1-phosphatase activity][GO:0006001:fructose catabolic process]	[ko00010:Glycolysis / Gluconeogenesis][ko00030:Pentose phosphate pathway][ko00051:Fructose and mannose metabolism][ko00680:Methane metabolism][ko00710:Carbon fixation in photosynthetic organisms][ko04152:AMPK signaling pathway][ko04910:Insulin signaling pathway]	-	EOY16677.1:Inositol monophosphatase family protein isoform 3, partial [Theobroma cacao]	gi|11139717|gb|AF317553.1|AF317553: Beta vulgaris cytosolic fructose-1,6-bisphosphatase mRNA, complete cds	A0A061FJ67|A0A061FJ67_THECC:Inositol monophosphatase family protein isoform 3;OS=Theobroma cacao;|GO:0016021,|GO:0042132,|GO:0006001,

geneid	trend	GO	Pathway	TF	NR	NT	Uniprot
DN123721_c0_g1_i3	UP	[GO:0005789:endoplasmic reticulum membrane][GO:0016021:integral component of membrane][GO:0004579:dolichyl-diphosphooligosaccharide-protein glycotransferase activity][GO:0006486:protein glycosylation]	-	Sme2.5_01445.1_g00001.1:Solanum melongena|MYB|MYB family protein	XP_021771025.1:protein WVD2like 7 [Chenopodium quinoa]	-	A0A0K9QF52|A0A0K9QF52_SPIOL:Dolichyl-diphosphooligosaccharide--protein glycosyltransferase subunit 1;OS=Spinacia oleracea;|GO:0005789,GO:0016021,|GO:0004579,|GO:0006486,
DN127846_c1_g3_i7	UP	[GO:0005634:nucleus][GO:0009734:auxin-activated signaling pathway][GO:0006355:regulation of transcription, DNA-templated][GO:0006351:transcription, DNA-templated]	[ko04075:Plant hormone signal transduction]	-	XP_010687936.1:PREDICTED: auxinresponsive protein IAA29 [Beta vulgaris subsp. vulgaris] >KMT02809.1 hypothetical protein BVRB_8g193910 [Beta vulgaris subsp. vulgaris]	-	A0A0J8BS29|A0A0J8BS29_BETVU:Auxin-responsive protein;OS=Beta vulgaris subsp. vulgaris.;|GO:0005634,|GO:0009734,GO:0006355,GO:0006351,

geneid	trend	GO	Pathway	TF	NR	NT	Uniprot
DN127312_c4_g10_i3	UP	[GO:0005524:ATP binding]	-	-	XP_002524206.1:PREDICTED: ribulose bisphosphate carboxylase/oxygenase activase 2, chloroplastic isoform X1 [Ricinus communis] >EEF38130.1 Ribulose bisphosphate carboxylase/oxygenase activase 1, chloroplast precursor, putative [Ricinus communis]	gi|21950711|gb|AY117142.1|:Chenopodium quinoa rubisco activase (rca) mRNA, complete cds	B9SDY7|B9SDY7_RICCO:Ribulose bisphosphate carboxylase/oxygenase activase 1, chloroplast, putative;OS=Ricinus communis;|GO:0005524,
DN123287_c0_g4_i1	UP	[GO:0016021:integral component of membrane][GO:0015297:antiporter activity]	-	-	XP_021864580.1:protein DETOXIFICATION 27like [Spinacia oleracea] >KNA04351.1 hypothetical protein SOVF_199750	-	A0A0K9QCU0|A0A0K9QCU0_SPIOL:Protein DETOXIFICATION;OS=Spinacia oleracea;|GO:0016021,|GO:0015297,GO:0015238,

geneid	trend	GO	Pathway	TF	NR	NT	Uniprot
					[Spinacia oleracea]		
DN125839_c0_g4_i5	UP	-	-	-	-	-	-
DN125797_c3_g4_i1	UP	[GO:0016758:transferase activity, transferring hexosyl groups][GO:0008152:metabolic process]	-	Tp57577_TGAC_v2_mRNA33215:Trifolium pratense|bHLH|bHLH family protein	XP_021725455.1:anthocyanidin 3Oglucosyltransferase 2like [Chenopodium quinoa]	gi|219566993|dbj|AB368370.1|:Phytolacca americana PaGT1 mRNA for glucosyltransferase, complete cds	B5MGN8|B5MGN8_PHYAM:Glycosyltransferase;OS=Phytolacca americana;|GO:0016758,|GO:0008152,

geneid	trend	GO	Pathway	TF	NR	NT	Uniprot
DN119070_c1_g1_i2	UP	[GO:0016021:integral component of membrane][GO:0020037:heme binding][GO:0005506:iron ion binding][GO:0004497:monooxygenase activity][GO:0016705:oxidoreductase activity, acting on paired donors, with incorporation or reduction of molecular oxygen]	[ko00073:Cutin, suberine and wax biosynthesis]	Sme2.5_00297.1_g00019.1:Solanum melongena|MYB|MYB family protein	XP_019105929.1:PREDICTED: cytochrome P450 86B1 [Beta vulgaris subsp. vulgaris] >KMT07595.1 hypothetical protein BVRB_6g147270 [Beta vulgaris subsp. vulgaris]	gi|521663462|gb|GAIA01035524.1|:TSA: Salicornia europaea Unigene39337_Se200S transcribed RNA sequence	A0A0J8C1F4|A0A0J8C1F4_BETVU:Uncharacterized protein;OS=Beta vulgaris subsp. vulgaris.;|GO:0016021,|GO:0020037,GO:0005506,GO:0004497,GO:0016705,
DN128498_c3_g6_i2	UP	[GO:0005524:ATP binding][GO:0004672:protein kinase activity]	-	AA10G00404:Aethionema arabicum|WRKY|WRKY family protein	XP_021734778.1:wallassociated receptor kinaselike 22 [Chenopodium quinoa]	-	A0A0K9Q7W1|A0A0K9Q7W1_SPIOL: Uncharacterized protein;OS=Spinacia oleracea;|GO:0005524,GO:0004672,
DN127175_c0_g3_i1	UP	-	-	-	XP_010676513.1:PREDICTED: protein P21like [Beta vulgaris subsp. vulgaris]	gi|46949199|gb|AY589699.1|:Mirabilis jalapa clone FS-87 thaumatin-like protein mRNA, partial cds	A0A0J8CEC0|A0A0J8CEC0_BETVU:Uncharacterized protein;OS=Beta vulgaris subsp. vulgaris.;

geneid	trend	GO	Pathway	TF	NR	NT	Uniprot
DN127312_c4_g20_i2	UP	[GO:0005524:ATP binding]	-	-	XP_014504832.1:PREDICTED: ribulose bisphosphate carboxylase/oxygenase activase, chloroplasticlike [Vigna radiata var. radiata]	gi|21950711|gb|AY117142.1|:Chenopodium quinoa rubisco activase (rca) mRNA, complete cds	A0A0L9UWT4|A0A0L9UWT4_PHAAN:Uncharacterized protein;OS=Phaseolus angularis;|GO:0005524,
DN115926_c2_g1_i2	UP	[GO:0016491:oxidoreductase activity][GO:0008270:zinc ion binding][GO:0042558:pteridine-containing compound metabolic process]	-	Ote100259360061:Ocimum tenuiflorum|HB-other|HB-other family protein	XP_021842915.1:2methylenefuran3one reductaselike [Spinacia oleracea]	gi|460385949|ref|XM_004238615.1|:PREDICTED: Solanum lycopersicum quinone oxidoreductase-like protein At1g23740, chloroplastic-like (LOC101250690), mRNA	A0A0K9RVS6|A0A0K9RVS6_SPIOL:Uncharacterized protein;OS=Spinacia oleracea;|GO:0016491,GO:0008270,|GO:0042558,

geneid	trend	GO	Pathway	TF	NR	NT	Uniprot
DN115940_c0_g1_i1	UP	[GO:0009570:chloroplast stroma][GO:0009579:thylakoid][GO:0005524:ATP binding][GO:0046863:ribulose-1,5-bisphosphate carboxylase/oxygenase activator activity]	-	GSBRNA2T00086041001:Brassica napus|ARF|ARF family protein	GAV76554.1:AAA domaincontaining protein [Cephalotus follicularis]	gi|502096954|ref|XM_004490816.1|:PREDICTED: Cicer arietinum ribulose bisphosphate carboxylase/oxygenase activase 1, chloroplastic-like (LOC101494120), mRNA	D7THJ7|D7THJ7_VITVI:Putative uncharacterized protein;OS=Vitis vinifera;GN=VIT_08s0007g00840 {ECO:0000313|EMBL:CBI30513.3};|GO:0009570,GO:0009579,|GO:0005524,GO:0046863,
DN125274_c1_g8_i9	UP	[GO:0000287:magnesium ion binding][GO:0010333:terpene synthase activity][GO:0008152:metabolic process]	-	Cotton_A_17926_BGI-A2_v1.0:Gossypium arboreum|GRAS|GRAS family protein	XP_010694277.1:PREDICTED: probable sesquiterpene synthase [Beta vulgaris subsp. vulgaris] >KMS98542.1 hypothetical protein BVRB_4g092310 [Beta vulgaris subsp. vulgaris]	-	A0A0J8BEZ1|A0A0J8BEZ1_BETVU:Uncharacterized protein;OS=Beta vulgaris subsp. vulgaris.;|GO:0000287,GO:0010333,|GO:0008152,

geneid	trend	GO	Pathway	TF	NR	NT	Uniprot
DN127405_c1_g1_i12	UP	-	-	-	XP_010676284.1:PREDICTED: uncharacterized protein LOC104892145 isoform X1 [Beta vulgaris subsp. vulgaris]	gi|521497374|gb|GAHZ01033546.1|:TSA: Salicornia europaea Unigene37373_SeCKS transcribed RNA sequence	A0A0K9RL19|A0A0K9RL19_SPIOL:Uncharacterized protein;OS=Spinacia oleracea;
DN108505_c0_g1_i1	UP	-	-	-	XP_014492609.1:PREDICTED: putative lipidtransfer protein DIR1 [Vigna radiata var. radiata]	-	A0A0S3RVP9|A0A0S3RVP9_PHAAN:Uncharacterized protein;OS=Vigna angularis var. angularis.;
DN124482_c2_g3_i3	UP	-	[ko04141:Protein processing in endoplasmic reticulum]	-	GAU26721.1:hypothetical protein TSUD_314980 [Trifolium subterraneum]	gi|521705427|gb|GAIA01015221.1|:TSA: Salicornia europaea Unigene15895_Se200S transcribed RNA sequence	M5XMI8|M5XMI8_PRUPE:Uncharacterized protein;OS=Prunus persica;
DN110125_c0_g1_i2	UP	-	-	-	XP_021739054.1:uncharacterized protein LOC110705478 [Chenopodium quinoa]	-	A0A0J8D3V7|A0A0J8D3V7_BETVU:Uncharacterized protein;OS=Beta vulgaris subsp.

geneid	trend	GO	Pathway	TF	NR	NT	Uniprot
							vulgaris.;
DN129144_c 7_g10_i7	UP	-	-	LOC_Os11g12360.1 :Oryza sativa subsp. japonica|M-type_M ADS|M-type_MAD S family protein	XP_010684970.1:P REDICTED: uncharacterized protein LOC104899469 [Beta vulgaris subsp. vulgaris]	-	A2I5F1|A2I5F1_BE TVU:Transposon-lik e protein;OS=Beta vulgaris;
DN127253_c 1_g3_i7	UP	-	-	-	XP_020245250.1:un characterized protein LOC109823384 [Asparagus officinalis]	-	-
DN114192_c 0_g1_i12	UP	[GO:0020037:heme binding][GO:0004601:peroxidase activity][GO:0006979:response to oxidative stress]	[ko00053:Ascorbate and aldarate metabolism][ko0048 0:Glutathione metabolism]	-	XP_021743586.1:th ylakoid lumenal 29 kDa protein, chloroplasticlike [Chenopodium quinoa]	gi|512399694|gb|GA JZ01001476.1|:TSA: Prunus avium Pa_02853 transcribed RNA sequence	A0A0K9QG33|A0A 0K9QG33_SPIOL:U ncharacterized protein;OS=Spinacia oleracea;|GO:00200 37,GO:0004601,|GO :0006979,

geneid	trend	GO	Pathway	TF	NR	NT	Uniprot
DN122803_c0_g10_i10	UP	[GO:0030598:rRNA N-glycosylase activity][GO:0006952:defense response][GO:0017148:negative regulation of translation]	-	-	ABI64066.1:ribosome inactivating protein [Bougainvillea spectabilis]	gi|114325705|gb|DQ989495.1|:Bougainvillea spectabilis ribosome inactivating protein (RIP) gene, complete cds	Q09EH2|Q09EH2_9CARY:rRNA N-glycosidase;OS= Bougainvillea spectabilis.;|GO:0030598,|GO:0006952, GO:0017148,
DN114044_c4_g4_i1	UP	[GO:0005960:glycine cleavage complex][GO:0005739:mitochondrion][GO:0019464:glycine decarboxylation via glycine cleavage system]	[ko00260:Glycine, serine and threonine metabolism][ko00630:Glyoxylate and dicarboxylate metabolism]	Pbr004711.1:Pyrus bretschneideri|NAC|NAC family protein	P93255.1:RecName: Full=Glycine cleavage system H protein, mitochondrial; Flags: Precursor >AAB38501.1 glycine cleavage system protein H precursor [Mesembryanthemum crystallinum]	gi|1724105|gb|U79768.1|MCU79768:Mesembryanthemum crystallinum glycine cleavage system protein H precursor, mRNA, complete cds	P93255|GCSH_MESCR:Glycine cleavage system H protein, mitochondrial;OS= Mesembryanthemum crystallinum;|GO:0005960,GO:0005739,|GO:0019464,

geneid	trend	GO	Pathway	TF	NR	NT	Uniprot
DN116124_c2_g4_i1	UP	[GO:0005576:extracellular region]	[ko04016:MAPK signaling pathway - plant][ko04075:Plant hormone signal transduction][ko04626:Plant-pathogen interaction]	-	XP_015613013.1:PREDICTED: pathogenesisrelated protein PRB12 [Oryza sativa Japonica Group] >AAM93438.1 putative type1 pathogenesisrelated protein [Oryza sativa Japonica Group] >AAP52566.1 Pathogenesisrelated protein PRB12 precursor, putative, expressed [Oryza sativa Japonica Group] >BAF26192.1 Os10g0191300 [Oryza sativa Japonica Group] >EAY77921.1 hypothetical protein OsI_32961 [Oryza	-	M0UNM7|M0UNM7_HORVD:Uncharacterized protein;OS=Hordeum vulgare var. distichum;|GO:0005576,

geneid	trend	GO	Pathway	TF	NR	NT	Uniprot
					sativa Indica Group] >BAH01584.1 unnamed protein product [Oryza sativa Japonica Group] >BAT10193.1 Os10g0191300 [Oryza sativa Japonica Group]		

geneid	trend	GO	Pathway	TF	NR	NT	Uniprot
DN127555_c3_g1_i8	UP	[GO:0005618:cell wall][GO:0005576:extracellular region][GO:0016020:membrane][GO:0009664:plant-type cell wall organization]	-	676732396:Sisymbrium irio|bHLH|bHLH family protein	XP_010690765.1:PREDICTED: expansinA4 [Beta vulgaris subsp. vulgaris] >KMT18338.1 hypothetical protein BVRB_2g024950 [Beta vulgaris subsp. vulgaris]	gi|356501064|ref|XM_003519301.1|:PREDICTED: Glycine max expansin-A4-like (LOC100803242), mRNA	A0A0J8D2C1|A0A0J8D2C1_BETVU:Uncharacterized protein;OS=Beta vulgaris subsp. vulgaris.;|GO:0005618,GO:0005576,GO:0016020,|GO:0009664,
DN115459_c0_g4_i4	UP	[GO:0009507:chloroplast][GO:0005840:ribosome][GO:0000166:nucleotide binding][GO:0019843:rRNA binding]	[ko03013:RNA transport][ko03015:mRNA surveillance pathway][ko03018:RNA degradation]	CCG030364.1:Populus euphratica|NF-YA|NF-YA family protein	XP_015936795.1:30S ribosomal protein 2, chloroplastic [Arachis duranensis]	gi|7578880|gb|AF240462.1|:Spinacia oleracea plastid-specific ribosomal protein 2 precursor (Psrp-2) mRNA, complete cds; nuclear gene for chloroplast product	P82277|RRP2_SPIOL:30S ribosomal protein 2, chloroplastic;OS=Spinacia oleracea;|GO:0009507,GO:0005840,|GO:0000166,GO:0019843,
DN125110_c4_g1_i5	UP	-	-	-	-	-	A0A0V0HD32|A0A0V0HD32_SOLCH:Putative ovule protein;OS=Solanum chacoense;

geneid	trend	GO	Pathway	TF	NR	NT	Uniprot
DN114151_c1_g1_i4	UP	[GO:0016021:integral component of membrane][GO:0005385:zinc ion transmembrane transporter activity]	-	Achn296391:Actinidia chinensis|NF-YC|NF-YC family protein	XP_010691362.1:PREDICTED: zinc transporter 1 [Beta vulgaris subsp. vulgaris] >KMT00842.1 hypothetical protein BVRB_9g221170 [Beta vulgaris subsp. vulgaris]	-	A0A0J8BIM2|A0A0J8BIM2_BETVU:Uncharacterized protein;OS=Beta vulgaris subsp. vulgaris.;|GO:0016021,|GO:0005385,
DN120862_c2_g2_i5	UP	[GO:0005737:cytoplasm][GO:0046914:transition metal ion binding][GO:0046916:cellular transition metal ion homeostasis][GO:0030001:metal ion transport]	-	Zjn_sc00020.1.g02760.1.am.mk:Zoysia japonica|MYB_related|MYB_related family protein	XP_002284075.1:PREDICTED: heavy metalassociated isoprenylated plant protein 7 [Vitis vinifera]	gi|470123002|ref|XM_004297478.1|:PREDICTED: Fragaria vesca subsp. vesca uncharacterized LOC101297429 (LOC101297429), mRNA	F6GSX0|F6GSX0_VITVI:Putative uncharacterized protein;OS=Vitis vinifera;GN=VIT_17s0000g01110 {ECO:0000313|EMBL:CCB43339.1};|GO:0005737,|GO:0046914,|GO:0046916, GO:0030001,

geneid	trend	GO	Pathway	TF	NR	NT	Uniprot
DN124231_c1_g3_i13	UP	[GO:0016021:integral component of membrane]	-	Zmw_sc06582.1.g00020.1:Zoysia matrella|C2H2|C2H2 family protein	XP_021857934.1:sulfite exporter TauE/SafE family protein 4 [Spinacia oleracea] >KNA13303.1 hypothetical protein SOVF_117870 [Spinacia oleracea]	gi|521746996|gb|GAIA01002365.1|:TSA: Salicornia europaea Unigene2418_Se200S transcribed RNA sequence	A0A0K9R1B8|A0A0K9R1B8_SPIOL:Uncharacterized protein;OS=Spinacia oleracea;|GO:0016021,
DN125383_c0_g2_i4	UP	-	-	-	XP_021722303.1:SAC3 family protein Alike isoform X2 [Chenopodium quinoa]	gi|521751171|gb|GAIA01000562.1|:TSA: Salicornia europaea Unigene573_Se200S transcribed RNA sequence	A0A0K9S253|A0A0K9S253_SPIOL:Uncharacterized protein;OS=Spinacia oleracea;
DN118685_c2_g1_i3	UP	[GO:0046872:metal ion binding][GO:0003676:nucleic acid binding][GO:0003700:DNA-binding transcription factor activity]	-	Bv7_162330_mjoz.t1:Beta vulgaris|C2H2|C2H2 family protein	XP_010684114.1:PREDICTED: zinc finger protein JACKDAW [Beta vulgaris subsp. vulgaris] >KMT06310.1 hypothetical protein BVRB_7g162330 [Beta vulgaris subsp.	gi|255561736|ref|XM_002521832.1|:Ricinus communis nucleic acid binding protein, putative, mRNA	A0A0J8BXP1|A0A0J8BXP1_BETVU:Uncharacterized protein;OS=Beta vulgaris subsp. vulgaris.;|GO:0046872,GO:0003676,GO:0003700,

geneid	trend	GO	Pathway	TF	NR	NT	Uniprot
					vulgaris]		
DN105589_c0_g1_i1	UP	[GO:0009535:chloroplast thylakoid membrane][GO:0016021:integral component of membrane][GO:0009522:photosystem I][GO:0009523:photosystem II][GO:0016168:chlorophyll binding][GO:0046872:metal ion binding][GO:0009765:photosynthesis, light harvesting][GO:0018298:protein-chromophore linkage]	[ko00196:Photosynthesis - antenna proteins]	-	XP_021750115.1:photosystem I chlorophyll a/bbinding protein 6, chloroplasticlike [Chenopodium quinoa]	gi|521512273|gb|GAHZ01018647.1|:TSA: Salicornia europaea Unigene19590_SeCKS transcribed RNA sequence	A0A0K9RHL0|A0A0K9RHL0_SPIOL:Chlorophyll a-b binding protein, chloroplastic;OS=Spinacia oleracea;|GO:0009535,GO:0016021,GO:0009522,GO:0009523,|GO:0016168,GO:0046872,|GO:0009765,GO:0018298,

geneid	trend	GO	Pathway	TF	NR	NT	Uniprot
DN119180_c0_g4_i2	UP	[GO:0005634:nucleus][GO:0000977:RNA polymerase II regulatory region sequence-specific DNA binding][GO:0003700:DNA-binding transcription factor activity][GO:0000165:MAPK cascade][GO:0045944:positive regulation of transcription by RNA polymerase II][GO:0006351:transcription, DNA-templated]	-	Bv9_214370_scjj.t1:Beta vulgaris|MIKC_MADS|MIKC_MADS family protein	XP_010690400.1:PREDICTED: MADSbox protein AGL24 [Beta vulgaris subsp. vulgaris] >XP_010690401.1 PREDICTED: MADSbox protein AGL24 [Beta vulgaris subsp. vulgaris] >XP_010690402.1 PREDICTED: MADSbox protein AGL24 [Beta vulgaris subsp. vulgaris] >KMT01428.1 hypothetical protein BVRB_9g214370 [Beta vulgaris subsp. vulgaris]	-	A0A0J8BN34|A0A0J8BN34_BETVU:Uncharacterized protein;OS=Beta vulgaris subsp. vulgaris.;|GO:0005634,|GO:0000977,GO:0003700,|GO:0000165,GO:0045944,GO:0006351,

geneid	trend	GO	Pathway	TF	NR	NT	Uniprot
DN123911_c0_g2_i7	UP	[GO:0016021:integral component of membrane]	[ko00514:Other types of O-glycan biosynthesis]	-	XP_021774450.1:hydroxyproline Oarabinosyltransferase 3like isoform X2 [Chenopodium quinoa]	gi|521504412|gb|GAHZ01026508.1|:TSA: Salicornia europaea Unigene29164_SeCKS transcribed RNA sequence	A0A0J8CTP8|A0A0J8CTP8_BETVU:Uncharacterized protein;OS=Beta vulgaris subsp. vulgaris.;|GO:0016021,
DN107837_c1_g1_i1	UP	[GO:0046872:metal ion binding][GO:0003676:nucleic acid binding]	-	Bv2_034570_jzgp.t1:Beta vulgaris|C2H2|C2H2 family protein	XP_010669389.1:PREDICTED: protein indeterminatedomain 7 [Beta vulgaris subsp. vulgaris] >XP_010669390.1 PREDICTED: protein indeterminatedomain 7 [Beta vulgaris subsp. vulgaris] >XP_010669391.1 PREDICTED: protein indeterminatedomain 7 [Beta vulgaris subsp. vulgaris] >KMT17934.1	gi|449532570|ref|XM_004173206.1|:PREDICTED: Cucumis sativus zinc finger protein NUTCRACKER-like (LOC101215280), mRNA	A0A0J8D0S8|A0A0J8D0S8_BETVU:Uncharacterized protein;OS=Beta vulgaris subsp. vulgaris.;|GO:0046872,GO:0003676,

geneid	trend	GO	Pathway	TF	NR	NT	Uniprot
					hypothetical protein BVRB_2g034570 [Beta vulgaris subsp. vulgaris]		
DN125857_c5_g6_i1	UP	-	-	-	-	-	-

geneid	trend	GO	Pathway	TF	NR	NT	Uniprot
DN124890_c1_g3_i1	UP	[GO:0004332:fructose-bisphosphate aldolase activity][GO:0006096:glycolytic process]	[ko00010:Glycolysis / Gluconeogenesis][ko000030:Pentose phosphate pathway][ko00051:Fructose and mannose metabolism][ko00680:Methane metabolism][ko00710:Carbon fixation in photosynthetic organisms]	FANhyb_rscf00000068.1.g00012.1:Fragaria x ananassa|FAR1|FAR1 family protein	XP_021907901.1:fructosebisphosphate aldolase 1, chloroplastic [Carica papaya] >AER26531.1 fructose 1,6 bisphosphate aldolase class 1 [Carica papaya]	gi|255581399|ref|XM_002531462.1|:Ricinus communis fructose-bisphosphate aldolase, putative, mRNA	G8FMI9|G8FMI9_CARPA:Fructose-bisphosphate aldolase;OS=Carica papaya;|GO:0004332,|GO:0006096,
DN128494_c1_g3_i1	UP	-	-	cra_locus_5362_iso_4:Catharanthus roseus|MYB_related|MYB_related family protein	XP_010666903.1:PREDICTED: protein TSS [Beta vulgaris subsp. vulgaris] >KMT20309.1 hypothetical protein BVRB_1g003100 [Beta vulgaris subsp. vulgaris]	gi|255564712|ref|XM_002523305.1|:Ricinus communis eukaryotic translation initiation factor 3 subunit, putative, mRNA	A0A0J8D7F3|A0A0J8D7F3_BETVU:Uncharacterized protein;OS=Beta vulgaris subsp. vulgaris.;

geneid	trend	GO	Pathway	TF	NR	NT	Uniprot
DN116039_c0_g1_i3	UP	[GO:0003824:catalytic activity]	-	Ote100129680091:Ocimum tenuiflorum|bHLH|bHLH family protein	XP_009596104.1:PREDICTED: uncharacterized protein ycf23like [Nicotiana tomentosiformis]	gi|521676530|gb|GAIA01029094.1|:TSA: Salicornia europaea Unigene31903_Se200S transcribed RNA sequence	A0A061GD15|A0A061GD15_THECC: Uncharacterized protein isoform 1;OS=Theobroma cacao;|GO:0003824,
DN122216_c3_g2_i2	UP	[GO:0016021:integral component of membrane][GO:0005524:ATP binding][GO:0004672:protein kinase activity]	[ko04064:NF-kappa B signaling pathway][ko04620:Toll-like receptor signaling pathway][ko04624:Toll and Imd signaling pathway][ko04722: Neurotrophin signaling pathway][ko05133:Pertussis][ko05140:Leishmaniasis][ko05142:Chagas disease (American trypanosomiasis)][ko05145:Toxoplasmosis][ko05152:Tuberc	-	XP_010694005.1:PREDICTED: putative receptorlike protein kinase At5g39000 [Beta vulgaris subsp. vulgaris] >KMT18668.1 hypothetical protein BVRB_2g027970 [Beta vulgaris subsp. vulgaris]	-	A0A0J8D3A9|A0A0J8D3A9_BETVU:Uncharacterized protein;OS=Beta vulgaris subsp. vulgaris.;|GO:0016021,|GO:0005524,GO:0004672,

geneid	trend	GO	Pathway	TF	NR	NT	Uniprot
			ulosis][ko05162:Measles][ko05169:Epstein-Barr virus infection]				
DN120000_c0_g5_i4	UP	[GO:0043565:sequence-specific DNA binding][GO:0003700:DNA-binding transcription factor activity][GO:0006351:transcription, DNA-templated]	-	Sp_166700_qmuh.t1:Spinacia oleracea|bZIP|bZIP family protein	XP_021847086.1:basic leucine zipper 23like [Spinacia oleracea] >KNA07994.1 hypothetical protein SOVF_166700 [Spinacia oleracea]	gi|292789741|dbj|AK339299.1|:Lotus japonicus cDNA, clone: LjFL3-069-AH02, HTC	A0A0K9QL71|A0A0K9QL71_SPIOL:Uncharacterized protein;OS=Spinacia oleracea;|GO:0043565,GO:0003700,|GO:0006351,

geneid	trend	GO	Pathway	TF	NR	NT	Uniprot
DN123048_c1_g7_i3	UP	[GO:0019898:extrinsic component of membrane][GO:0009654:photosystem II oxygen evolving complex][GO:0005509:calcium ion binding][GO:0015979:photosynthesis]	[ko00195:Photosynthesis]	Araip.Q7CA9:Arachis ipaensis|bHLH|bHLH family protein	XP_021739618.1:photosynthetic NDH subunit of lumenal location 1, chloroplasticlike [Chenopodium quinoa]	-	A0A0K9RYL4|A0A0K9RYL4_SPIOL: Uncharacterized protein;OS=Spinacia oleracea;|GO:0019898,GO:0009654,|GO:0005509,|GO:0015979,
DN118181_c1_g4_i1	UP	-	-	-	P42764.1:RecName: Full=Jasmonateinduced protein homolog >AAA86977.1 jasmonateinduced protein homolog; similar to barley jasmonateinduced protein, SwissProt Accession Number P32024 [Atriplex canescens]	-	P42764|JIPH_ATRCA:Jasmonate-induced protein homolog;OS=Atriplex canescens;

geneid	trend	GO	Pathway	TF	NR	NT	Uniprot
DN125632_c0_g1_i6	UP	[GO:0016021:integral component of membrane][GO:0005524:ATP binding][GO:0016887:ATPase activity]	-	MDP0000265619:Malus domestica|Nin-like|Nin-like family protein	XP_021714209.1:ABC transporter G family member 11like isoform X1 [Chenopodium quinoa]	gi|359497154|ref|XM_003635390.1|:PREDICTED: Vitis vinifera ABC transporter G family member 11-like (LOC100852871), mRNA	A0A0K9R4W0|A0A0K9R4W0_SPIOL: Uncharacterized protein;OS=Spinacia oleracea;|GO:0016021,|GO:0005524,GO:0016887,
DN116177_c0_g1_i5	UP	[GO:0000079:regulation of cyclin-dependent protein serine/threonine kinase activity]	-	Gh_A08G1256:Gossypium hirsutum|WRKY|WRKY family protein	XP_021764130.1:cyclinU11like isoform X2 [Chenopodium quinoa]	-	A0A067F9I5|A0A067F9I5_CITSI:Uncharacterized protein;OS=Citrus sinensis;|GO:0000079,
DN123384_c0_g2_i5	UP	[GO:0016021:integral component of membrane][GO:0016760:cellulose synthase (UDP-forming) activity][GO:0071555:cell wall organization][GO:0030244:cellulose biosynthetic process]	-	Sme2.5_02298.1_g00001.1:Solanum melongena|ARF|ARF family protein	XP_010675676.1:PREDICTED: cellulose synthaselike protein G2 [Beta vulgaris subsp. vulgaris] >KMT12691.1 hypothetical protein BVRB_4g087890 [Beta vulgaris subsp. vulgaris]	-	A0A0J8F9Z4|A0A0J8F9Z4_BETVU:Uncharacterized protein;OS=Beta vulgaris subsp. vulgaris.;|GO:0016021,|GO:0016760,|GO:0071555,GO:0030244,

geneid	trend	GO	Pathway	TF	NR	NT	Uniprot
DN108904_c0_g1_i1	UP	[GO:0009535:chloroplast thylakoid membrane][GO:0009512:cytochrome b6f complex][GO:0016021:integral component of membrane]	-	PGSC0003DMP400044805:Solanum tuberosum|B3|B3 family protein	XP_010684011.1:PREDICTED: uncharacterized protein LOC104898612 [Beta vulgaris subsp. vulgaris] >KMT19285.1 hypothetical protein BVRB_1g012920 [Beta vulgaris subsp. vulgaris]	-	A0A0J8CZJ8|A0A0J8CZJ8_BETVU:Uncharacterized protein;OS=Beta vulgaris subsp. vulgaris.;|GO:0009535,GO:0009512,GO:0016021,
DN116401_c3_g1_i1	UP	-	-	-	XP_010685303.1:PREDICTED: uncharacterized protein LOC104899744 [Beta vulgaris subsp. vulgaris] >KMT04821.1 hypothetical protein BVRB_7g169920 [Beta vulgaris subsp. vulgaris]	-	A0A0J8EPA9|A0A0J8EPA9_BETVU:Uncharacterized protein;OS=Beta vulgaris subsp. vulgaris.;

geneid	trend	GO	Pathway	TF	NR	NT	Uniprot
DN127616_c4_g1_i5	UP	-	[ko04137:Mitophagy - animal][ko04217:Necroptosis][ko04668:TNF signaling pathway]	-	XP_019106672.1:PREDICTED: probable 2carboxyDarabinitol 1phosphatase [Beta vulgaris subsp. vulgaris] >KMT04244.1 hypothetical protein BVRB_8g183200 [Beta vulgaris subsp. vulgaris]	gi|521666413|gb|GAIA01034331.1|:TSA: Salicornia europaea Unigene38004_Se200S transcribed RNA sequence	A0A0J8BW62|A0A0J8BW62_BETVU: Uncharacterized protein;OS=Beta vulgaris subsp. vulgaris.;
DN128770_c2_g3_i2	UP	[GO:0016021:integral component of membrane]	-	-	XP_021752782.1:receptorlike protein 12 [Chenopodium quinoa]	-	A0A0K9R8A7|A0A0K9R8A7_SPIOL:Uncharacterized protein;OS=Spinacia oleracea;|GO:0016021,

geneid	trend	GO	Pathway	TF	NR	NT	Uniprot
DN122316_c0_g1_i3	UP	[GO:0004553:hydrolase activity, hydrolyzing O-glycosyl compounds][GO:0005975:carbohydrate metabolic process]	[ko00520:Amino sugar and nucleotide sugar metabolism]	-	XP_010687347.1:PREDICTED: probable betaDxylosidase 5 [Beta vulgaris subsp. vulgaris] >KMT03815.1 hypothetical protein BVRB_8g189590 [Beta vulgaris subsp. vulgaris]	gi|297834873|ref|XM_002885273.1|:Arabidopsis lyrata subsp. lyrata beta-1,4-xylosidase, mRNA	A0A0J8BQN3|A0A0J8BQN3_BETVU: Uncharacterized protein;OS=Beta vulgaris subsp. vulgaris.;|GO:0004553,|GO:0005975,
DN119031_c8_g6_i1	UP	-	[ko00480:Glutathione metabolism][ko00980:Metabolism of xenobiotics by cytochrome P450][ko00982:Drug metabolism - cytochrome P450][ko01524:Platinum drug resistance][ko05204:Chemical carcinogenesis][ko0	-	XP_008364312.1:PREDICTED: probable glutathione Stransferase [Malus domestica]	-	M5X2D7|M5X2D7_PRUPE:Uncharacterized protein;OS=Prunus persica;

geneid	trend	GO	Pathway	TF	NR	NT	Uniprot
			5418:Fluid shear stress and atherosclerosis]				
DN128375_c2_g5_i2	UP	[GO:0030246:carbohydrate binding][GO:0004553:hydrolase activity, hydrolyzing O-glycosyl compounds][GO:0005975:carbohydrate metabolic process]	-	Phvul.002G126900.1:Phaseolus vulgaris|FAR1|FAR1 family protein	KNA15567.1:hypothetical protein SOVF_097260 [Spinacia oleracea]	gi|521498910|gb|GAHZ01032010.1|:TSA: Salicornia europaea Unigene35284_SeCKS transcribed RNA sequence	A0A0K9R9X2|A0A0K9R9X2_SPIOL:Uncharacterized protein;OS=Spinacia oleracea;|GO:0030246,GO:0004553,|GO:0005975,

geneid	trend	GO	Pathway	TF	NR	NT	Uniprot
DN124774_c1_g1_i1	UP	-	[ko04141:Protein processing in endoplasmic reticulum]	WALNUT_00030998-RA:Juglans regia|NF-YB|NF-YB family protein	XP_002533590.1:PREDICTED: 18.1 kDa class I heat shock protein [Ricinus communis] >EEF28795.1 heatshock protein, putative [Ricinus communis]	-	B9T5S1|B9T5S1_RICCO:Heat-shock protein, putative;OS=Ricinus communis;
DN122593_c1_g6_i6	UP	[GO:0016765:transferase activity, transferring alkyl or aryl (other than methyl) groups]	[ko00900:Terpenoid backbone biosynthesis]	-	XP_010685566.1:PREDICTED: dehydrodolichyl diphosphate synthase 2 [Beta vulgaris subsp. vulgaris] >KMT05070.1 hypothetical protein BVRB_7g172260 [Beta vulgaris subsp. vulgaris]	-	A0A0J8BU46|A0A0J8BU46_BETVU:Alkyl transferase;OS=Beta vulgaris subsp. vulgaris.;|GO:0016765,

geneid	trend	GO	Pathway	TF	NR	NT	Uniprot
DN128162_c1_g9_i1	UP	[GO:0016765:transferase activity, transferring alkyl or aryl (other than methyl) groups]	[ko00900:Terpenoid backbone biosynthesis]	-	XP_010685566.1:PREDICTED: dehydrodolichyl diphosphate synthase 2 [Beta vulgaris subsp. vulgaris] >KMT05070.1 hypothetical protein BVRB_7g172260 [Beta vulgaris subsp. vulgaris]	-	A0A0J8BU46|A0A0J8BU46_BETVU:Alkyl transferase;OS=Beta vulgaris subsp. vulgaris.;|GO:0016765,
DN119070_c1_g1_i1	UP	[GO:0016021:integral component of membrane][GO:0020037:heme binding][GO:0005506:iron ion binding][GO:0004497:monooxygenase activity][GO:0016705:oxidoreductase activity, acting on paired donors, with incorporation or reduction of molecular oxygen]	[ko00073:Cutin, suberine and wax biosynthesis]	Sme2.5_00297.1_g00019.1:Solanum melongena|MYB|MYB family protein	XP_019105929.1:PREDICTED: cytochrome P450 86B1 [Beta vulgaris subsp. vulgaris] >KMT07595.1 hypothetical protein BVRB_6g147270 [Beta vulgaris subsp. vulgaris]	gi|521663462|gb|GAIA01035524.1|:TSA: Salicornia europaea Unigene39337_Se200S transcribed RNA sequence	A0A0J8C1F4|A0A0J8C1F4_BETVU:Uncharacterized protein;OS=Beta vulgaris subsp. vulgaris.;|GO:0016021,|GO:0020037,GO:0005506,GO:0004497,GO:0016705,

geneid	trend	GO	Pathway	TF	NR	NT	Uniprot
DN114166_c2_g13_i1	UP	[GO:0016021:integral component of membrane]	-	-	XP_010693808.1:PREDICTED: uncharacterized protein LOC104906713 isoform X1 [Beta vulgaris subsp. vulgaris] >KMS98760.1 hypothetical protein BVRB_3g068390 isoform A [Beta vulgaris subsp. vulgaris]	-	A0A0J8E6D8|A0A0J8E6D8_BETVU:Uncharacterized protein;OS=Beta vulgaris subsp. vulgaris.;|GO:0016021,
DN125802_c0_g1_i8	UP	[GO:0005737:cytoplasm][GO:0046914:transition metal ion binding][GO:0046916:cellular transition metal ion homeostasis][GO:0030001:metal ion transport]	-	-	XP_021605141.1:heavy metalassociated isoprenylated plant protein 39like [Manihot esculenta] >OAY56450.1 hypothetical protein MANES_02G017600 [Manihot esculenta]	-	B9GWV4|B9GWV4_POPTR:Uncharacterized protein;OS=Populus trichocarpa;|GO:0005737,|GO:0046914,|GO:0046916,GO:0030001,

geneid	trend	GO	Pathway	TF	NR	NT	Uniprot
DN116739_c 1_g2_i1	UP	[GO:0016758:transferase activity, transferring hexosyl groups][GO:0008152:metabolic process]	[ko00965:Betalain biosynthesis]	AA31G00555:Aethi onema arabicum|CO-like|C O-like family protein	BAD91803.1:cyclo DOPA 5Oglucosyltransfera se [Mirabilis jalapa]	gi|62086400|dbj|AB 182643.1|:Mirabilis jalapa cDOPA5GT mRNA for cyclo-DOPA 5-O-glucosyltransfer ase, complete cds	Q59J81|Q59J81_MI RJA:Cyclo-DOPA 5-O-glucosyltransfer ase;OS=Mirabilis jalapa;|GO:0016758, |GO:0008152,
DN125161_c 10_g4_i6	UP	[GO:0046872:metal ion binding]	-	462924824:Eragrosti s tef|GRAS|GRAS family protein	XP_021844933.1:pr otein yippeelike At5g53940 [Spinacia oleracea]	-	E0CSG5|E0CSG5_ VITVI:Protein yippee-like;OS=Viti s vinifera;GN=VIT_1 9s0014g02250 {ECO:0000313|EM BL:CBI20264.3};|G O:0046872,
DN240231_c 0_g1_i1	UP	[GO:0016020:membrane][GO:00097 65:photosynthesis, light harvesting]	[ko00196:Photosynt hesis - antenna proteins]	Bradi3g42627.3.p:B rachypodium distachyon|ERF|ERF family protein	XP_009416547.1:P REDICTED: chlorophyll ab binding protein of LHCII type 1 [Musa acuminata subsp. malaccensis] >ABF69952.1 chlorophyll AB	gi|359754902|gb|HQ 731870.1|:Oxytropis maydelliana isolate om_Aiken_sn_20g chloroplast photosystem II light harvesting complex protein type I (lhcbI) gene, partial cds;	A0A199VHI1|A0A1 99VHI1_ANACO:C hlorophyll a-b binding protein, chloroplastic;OS=A nanas comosus;|GO:00160 20,|GO:0009765,

geneid	trend	GO	Pathway	TF	NR	NT	Uniprot
					binding protein (CAB), putative [Musa acuminata]	nuclear gene for chloroplast product	
DN117839_c1_g1_i2	UP	[GO:0016021:integral component of membrane][GO:0005524:ATP binding][GO:0005509:calcium ion binding][GO:0030247:polysaccharide binding][GO:0004672:protein kinase activity]	[ko04064:NF-kappa B signaling pathway][ko04620:Toll-like receptor signaling pathway][ko04621: NOD-like receptor signaling pathway][ko04624:Toll and Imd signaling pathway][ko04722: Neurotrophin signaling pathway][ko05133:Pertussis][ko05140:L	AA10G00404:Aethionema arabicum|WRKY|WRKY family protein	XP_010672297.1:PREDICTED: wallassociated receptor kinase 3like [Beta vulgaris subsp. vulgaris]	gi|357119507|ref|XM_003561433.1|:PREDICTED: Brachypodium distachyon wall-associated receptor kinase-like 17-like (LOC100841907), mRNA	A0A0J8CUX4|A0A0J8CUX4_BETVU: Uncharacterized protein;OS=Beta vulgaris subsp. vulgaris.;|GO:0016021,|GO:0005524,GO:0005509,GO:0030247,GO:0004672,

geneid	trend	GO	Pathway	TF	NR	NT	Uniprot
			eishmaniasis][ko05142:Chagas disease (American trypanosomiasis)][ko05145:Toxoplasmosis][ko05152:Tuberculosis][ko05162:Measles][ko05164:Influenza A]				
DN115973_c1_g1_i9	UP	[GO:0004556:alpha-amylase activity][GO:0005509:calcium ion binding][GO:0005975:carbohydrate metabolic process]	[ko00500:Starch and sucrose metabolism][ko04973:Carbohydrate digestion and absorption]	CCG032957.1:Populus euphratica|ERF|ERF family protein	XP_021756521.1:probable alphaamylase 2 isoform X1 [Chenopodium quinoa] >XP_021756522.1 probable alphaamylase 2 isoform X1	gi|521506657|gb|GAHZ01024263.1|:TSA: Salicornia europaea Unigene26201_SeCKS transcribed RNA sequence	A0A067KX57|A0A067KX57_JATCU: Alpha-amylase;OS=Jatropha curcas;|GO:0004556,GO:0005509,|GO:0005975,

geneid	trend	GO	Pathway	TF	NR	NT	Uniprot
					[Chenopodium quinoa]		
DN127211_c3_g2_i10	UP	[GO:0016021:integral component of membrane]	-	CCG026078.1:Populus euphratica|CO-like|CO-like family protein	XP_010675760.1:PREDICTED: uncharacterized protein LOC104891722 [Beta vulgaris subsp. vulgaris]	-	A0A0J8FA99|A0A0J8FA99_BETVU:Uncharacterized protein;OS=Beta vulgaris subsp. vulgaris.;|GO:0016021,
DN128140_c1_g2_i4	UP	[GO:0032502:developmental process]	-	-	OMP05307.1:Transposase, Tc1like protein [Corchorus olitorius]	-	K7LU34|K7LU34_SOYBN:Uncharacterized protein;OS=Glycine max;|GO:0032502,
DN117274_c4_g3_i5	UP	[GO:0016021:integral component of membrane][GO:0055085:transmembrane transport]	-	Jcr4S00206.70:Jatropha curcas|FAR1|FAR1 family protein	XP_021727617.1:protein PINLIKES 1like [Chenopodium quinoa]	-	A0A0K9QZF3|A0A0K9QZF3_SPIOL:Uncharacterized protein;OS=Spinacia oleracea;|GO:0016021,|GO:0055085,

geneid	trend	GO	Pathway	TF	NR	NT	Uniprot
DN112911_c1_g1_i1	UP	[GO:0016021:integral component of membrane][GO:0009523:photosystem II][GO:0030145:manganese ion binding][GO:0015979:photosynthesis]	-	-	XP_010695963.1:PREDICTED: photosystem II core complex proteins psbY, chloroplastic [Beta vulgaris subsp. vulgaris] >KMS97236.1 hypothetical protein BVRB_7g176840 [Beta vulgaris subsp. vulgaris]	-	A0A0J8B8A9|A0A0J8B8A9_BETVU:Uncharacterized protein;OS=Beta vulgaris subsp. vulgaris.;|GO:0016021,GO:0009523,|GO:0030145,|GO:0015979,
DN128674_c0_g1_i2	UP	[GO:0005737:cytoplasm][GO:0005634:nucleus][GO:0004871:obsolete signal transducer activity][GO:0009638:phototropism]	-	-	XP_010677799.1:PREDICTED: root phototropism protein 2 [Beta vulgaris subsp. vulgaris] >KMT11299.1 hypothetical protein BVRB_5g109820 [Beta vulgaris subsp. vulgaris]	-	A0A0J8CGR1|A0A0J8CGR1_BETVU: Uncharacterized protein;OS=Beta vulgaris subsp. vulgaris.;|GO:0005737,GO:0005634,|GO:0004871,|GO:0009638,

geneid	trend	GO	Pathway	TF	NR	NT	Uniprot
DN125473_c0_g1_i8	UP	[GO:0008270:zinc ion binding]	-	-	XP_010694486.1:PREDICTED: DNAdependent metalloprotease WSS1 [Beta vulgaris subsp. vulgaris] >XP_010694487.1 PREDICTED: DNAdependent metalloprotease WSS1 [Beta vulgaris subsp. vulgaris] >XP_019108113.1 PREDICTED: DNAdependent metalloprotease WSS1 [Beta vulgaris subsp. vulgaris] >KMS98332.1 hypothetical protein BVRB_4g093460 [Beta vulgaris subsp. vulgaris]	gi|521492891|gb|GAHZ01037995.1|:TSA: Salicornia europaea Unigene42861_SeCKS transcribed RNA sequence	A0A0J8BEE3|A0A0J8BEE3_BETVU:Uncharacterized protein;OS=Beta vulgaris subsp. vulgaris.;|GO:0008270,

geneid	trend	GO	Pathway	TF	NR	NT	Uniprot
DN120634_c0_g1_i4	UP	[GO:0050589:leucocyanidin oxygenase activity][GO:0046872:metal ion binding]	-	676709864:Sisymbrium irio|STAT|STAT family protein	GAV79710.1:2OGFeII_Oxy domaincontaining protein/DIOX_N domaincontaining protein [Cephalotus follicularis]	-	B9S9D4|B9S9D4_RICCO:Leucoanthocyanidin dioxygenase, putative;OS=Ricinus communis;|GO:0050589,GO:0046872,
DN125632_c0_g1_i2	UP	[GO:0016021:integral component of membrane][GO:0005524:ATP binding][GO:0016887:ATPase activity]	-	MDP0000265619:Malus domestica|Nin-like|Nin-like family protein	XP_021714209.1:ABC transporter G family member 11like isoform X1 [Chenopodium quinoa]	gi|359497154|ref|XM_003635390.1|:PREDICTED: Vitis vinifera ABC transporter G family member 11-like (LOC100852871), mRNA	A0A0K9R4W0|A0A0K9R4W0_SPIOL: Uncharacterized protein;OS=Spinacia oleracea;|GO:0016021,|GO:0005524,GO:0016887,
DN119056_c0_g3_i5	UP	[GO:0005524:ATP binding][GO:0004143:diacylglycerol kinase activity][GO:0007205:protein kinase C-activating G protein-coupled receptor signaling pathway]	[ko00561:Glycerolipid metabolism][ko00564:Glycerophospholipid metabolism][ko04070:Phosphatidylinositol signaling system][ko04072:Phospholipase D	-	XP_021738335.1:diacylglycerol kinase 5like isoform X1 [Chenopodium quinoa]	gi|521493555|gb|GAHZ01037365.1|:TSA: Salicornia europaea Unigene42129_SeCKS transcribed RNA sequence	A0A0K9QM10|A0A0K9QM10_SPIOL: Diacylglycerol kinase;OS=Spinacia oleracea;|GO:0005524,GO:0004143,|GO:0007205,

geneid	trend	GO	Pathway	TF	NR	NT	Uniprot
			signaling pathway][ko05231: Choline metabolism in cancer]				
DN128931_c5_g5_i1	UP	[GO:0016021:integral component of membrane][GO:0005524:ATP binding][GO:0004672:protein kinase activity]	-	DCAR_008117:Daucus carota|bHLH|bHLH family protein	XP_021756548.1:cysteinerich receptorlike protein kinase 27 isoform X1 [Chenopodium quinoa]	-	A0A067JAH9|A0A067JAH9_JATCU:Uncharacterized protein;OS=Jatropha curcas;|GO:0016021,|GO:0005524,GO:0004672,
DN127759_c2_g1_i6	UP	[GO:0004089:carbonate dehydratase activity][GO:0008270:zinc ion binding][GO:0006730:one-carbon metabolic process]	[ko00910:Nitrogen metabolism]	Sopen10g033340.1: Solanum pennellii|MYB|MYB family protein	XP_021774553.1:alpha carbonic anhydrase 7like [Chenopodium quinoa] >XP_021775735.1 alpha carbonic anhydrase 7like [Chenopodium	-	A0A0J8B744|A0A0J8B744_BETVU:Uncharacterized protein;OS=Beta vulgaris subsp. vulgaris.;|GO:0004089,GO:0008270,|GO:0006730,

geneid	trend	GO	Pathway	TF	NR	NT	Uniprot
					quinoa]		
DN120069_c0_g2_i1	UP	[GO:0016021:integral component of membrane][GO:0005524:ATP binding][GO:0004672:protein kinase activity]	-	mrna27137.1-v1.0-hybrid:Fragaria vesca|STAT|STAT family protein	XP_010695988.1:PREDICTED: leucinerich repeat receptorlike serine/threonine/tyrosineprotein kinase SOBIR1 [Beta vulgaris subsp. vulgaris] >KMS97260.1 hypothetical protein BVRB_7g177070 [Beta vulgaris subsp. vulgaris]	gi|224133743|ref|XM_002321614.1|:Populus trichocarpa predicted protein, mRNA	A0A0J8E2C4|A0A0J8E2C4_BETVU:Uncharacterized protein;OS=Beta vulgaris subsp. vulgaris.;|GO:0016021,|GO:0005524,GO:0004672,
DN124774_c1_g10_i4	UP	-	[ko04141:Protein processing in endoplasmic reticulum]	NNU_003323-RA:Nelumbo nucifera|HSF|HSF family protein	XP_021732397.1:17.4 kDa class I heat shock proteinlike [Chenopodium quinoa]	-	Unreviewed|E1AWP1_TOBAC:HSP18.3;OS=Nicotiana tabacum;

geneid	trend	GO	Pathway	TF	NR	NT	Uniprot
DN117055_c0_g3_i1	UP	[GO:0048529:magnesium-protoporphyrin IX monomethyl ester (oxidative) cyclase activity][GO:0046872:metal ion binding][GO:0015995:chlorophyll biosynthetic process][GO:0015979:photosynthesis]	[ko00860:Porphyrin and chlorophyll metabolism]	-	KNA05315.1:hypothetical protein SOVF_191490 [Spinacia oleracea]	gi|118488486|gb|EF148071.1|:Populus trichocarpa clone WS0127_F03 unknown mRNA	A0A0K9QDI4|A0A0K9QDI4_SPIOL:Uncharacterized protein;OS=Spinacia oleracea;|GO:0048529,GO:0046872,|GO:0015995,GO:0015979,
DN120171_c0_g4_i1	UP	[GO:0009507:chloroplast][GO:0004497:monooxygenase activity][GO:0016984:ribulose-bisphosphate carboxylase activity][GO:0009853:photorespiration][GO:0019253:reductive pentose-phosphate cycle]	[ko00630:Glyoxylate and dicarboxylate metabolism][ko00710:Carbon fixation in photosynthetic organisms]	mrna16933.1-v1.0-hybrid:Fragaria vesca|M-type_MADS|M-type_MADS family protein	P16032.2:RecName: Full=Ribulose bisphosphate carboxylase small chain 1, chloroplastic; Short=RuBisCO small subunit 1; Flags: Precursor >AAA03693.1 ribulose1,5bisphosphate carboxylase/oxygenase small subunit [Mesembryanthemum crystallinum] >prf||1802403A	gi|521548629|gb|GAHZ01000026.1|:TSA: Salicornia europaea Unigene26_SeCKS transcribed RNA sequence	P16032|RBS1_MESCR:Ribulose bisphosphate carboxylase small chain 1, chloroplastic;OS=Mesembryanthemum crystallinum;|GO:0009507,|GO:0004497,GO:0016984,|GO:0009853,GO:0019253,

geneid	trend	GO	Pathway	TF	NR	NT	Uniprot
					RuBisCO:SUBUNIT=small		
DN121559_c3_g5_i1	UP	[GO:0004866:endopeptidase inhibitor activity]	-	-	XP_021722899.1:miraculinlike [Chenopodium quinoa]	-	M5X024|M5X024_PRUPE:Uncharacterized protein;OS=Prunus persica;|GO:0004866,
DN124496_c2_g8_i8	UP	-	-	DCAR_025729:Daucus carota|B3|B3 family protein	XP_010692733.1:PREDICTED: protein PLANT CADMIUM RESISTANCE 2like [Beta vulgaris subsp. vulgaris]	gi|521525561|gb|GAHZ01010883.1|:TSA: Salicornia europaea Unigene11498_SeCKS transcribed RNA sequence	A0A0K9RJW7|A0A0K9RJW7_SPIOL: Uncharacterized protein;OS=Spinacia oleracea;

geneid	trend	GO	Pathway	TF	NR	NT	Uniprot
DN126262_c4_g2_i5	UP	[GO:0016567:protein ubiquitination]	-	LPERR12G15710.1:Leersia perrieri|MYB|MYB family protein	XP_021851544.1:root phototropism protein 3 [Spinacia oleracea] >KNA20255.1 hypothetical protein SOVF_054220 [Spinacia oleracea]	gi|225464090|ref|XM_002269344.1|:PREDICTED: Vitis vinifera root phototropism protein 3-like (LOC100264249), mRNA	A0A0K9RL77|A0A0K9RL77_SPIOL:Uncharacterized protein;OS=Spinacia oleracea;|GO:0016567,
DN119365_c2_g6_i12	UP	-	-	-	-	-	-

geneid	trend	GO	Pathway	TF	NR	NT	Uniprot
DN114033_c0_g1_i2	UP	-	-	-	XP_010685041.1:PREDICTED: uncharacterized protein LOC104899533 isoform X1 [Beta vulgaris subsp. vulgaris] >XP_010685042.1 PREDICTED: uncharacterized protein LOC104899533 isoform X1 [Beta vulgaris subsp. vulgaris] >KMT05651.1 hypothetical protein BVRB_7g167850 isoform B [Beta vulgaris subsp. vulgaris]	-	A0A0J8ERE7|A0A0J8ERE7_BETVU:Uncharacterized protein;OS=Beta vulgaris subsp. vulgaris.;

geneid	trend	GO	Pathway	TF	NR	NT	Uniprot
DN124860_c2_g2_i1	UP	-	-	LPERR12G15710.1:Leersia perrieri|MYB|MYB family protein	KNA22061.1:hypothetical protein SOVF_037530 [Spinacia oleracea]	-	A0A0K9RR95|A0A0K9RR95_SPIOL:Uncharacterized protein;OS=Spinacia oleracea;
DN120312_c2_g1_i2	UP	-	-	-	XP_021837801.1:uncharacterized protein LOC110777502 isoform X1 [Spinacia oleracea] >KNA08248.1 hypothetical protein SOVF_164280 isoform A [Spinacia oleracea]	-	A0A0K9QNH2|A0A0K9QNH2_SPIOL: Uncharacterized protein;OS=Spinacia oleracea;
DN117105_c1_g1_i2	UP	[GO:0006355:regulation of transcription, DNA-templated][GO:0006351:transcription, DNA-templated]	-	Bv6_128680_pjam.t1:Beta vulgaris|GRAS|GRAS family protein	XP_010679714.1:PREDICTED: scarecrowlike protein 4 [Beta vulgaris subsp. vulgaris] >KMT09386.1 hypothetical protein BVRB_6g128680 [Beta vulgaris subsp.	-	A0A0J8F1D2|A0A0J8F1D2_BETVU:Uncharacterized protein;OS=Beta vulgaris subsp. vulgaris.;|GO:0006355,GO:0006351,

geneid	trend	GO	Pathway	TF	NR	NT	Uniprot
					vulgaris]		
DN120748_c 1_g2_i1	UP	-	-	XP_009123613.1:Br assica rapa|G2-like|G2-like family protein	KMT13888.1:hypot hetical protein BVRB_4g077300 [Beta vulgaris subsp. vulgaris]	gi|521703587|gb|GA IA01016131.1|:TSA: Salicornia europaea Unigene16839_Se20 0S transcribed RNA sequence	A0A0J8CPC8|A0A0 J8CPC8_BETVU:U ncharacterized protein;OS=Beta vulgaris subsp. vulgaris.;
DN124695_c 1_g1_i4	UP	[GO:0000932:P-body][GO:0005634: nucleus][GO:0000166:nucleotide binding][GO:0003723:RNA binding][GO:0006397:mRNA processing][GO:0045595:regulation of cell differentiation][GO:0043484:regulati on of RNA splicing]	-	-	XP_021737734.1:po lypyrimidine tractbinding protein homolog 3like isoform X1 [Chenopodium quinoa]	-	A0A0J8D5W4|A0A 0J8D5W4_BETVU: Uncharacterized protein;OS=Beta vulgaris subsp. vulgaris.;|GO:00009 32,GO:0005634,|GO :0000166,GO:00037 23,|GO:0006397,GO :0045595,GO:00434 84,

geneid	trend	GO	Pathway	TF	NR	NT	Uniprot
DN120503_c2_g2_i9	UP	[GO:0005634:nucleus][GO:0003677:DNA binding][GO:0003700:DNA-binding transcription factor activity][GO:0007275:multicellular organism development][GO:0006351:transcription, DNA-templated]	-	Sp_192560_dzus.t1: Spinacia oleracea|AP2|AP2 family protein	XP_010672391.1:PREDICTED: AP2like ethyleneresponsive transcription factor At2g41710 [Beta vulgaris subsp. vulgaris] >XP_010672863.1 PREDICTED: AP2like ethyleneresponsive transcription factor At2g41710 [Beta vulgaris subsp. vulgaris]	gi|521526092|gb|GAHZ01010434.1|:TSA: Salicornia europaea Unigene11049_SeCKS transcribed RNA sequence	A0A0K9QD46|A0A0K9QD46_SPIOL:Uncharacterized protein;OS=Spinacia oleracea;|GO:0005634,|GO:0003677,GO:0003700,|GO:0007275,GO:0006351,
DN122358_c2_g1_i9	UP	[GO:0004512:inositol-3-phosphate synthase activity][GO:0006021:inositol biosynthetic process][GO:0008654:phospholipid biosynthetic process]	[ko00521:Streptomycin biosynthesis][ko00562:Inositol phosphate metabolism]	-	XP_021861771.1:inositol3phosphate synthase [Spinacia oleracea] >KNA08398.1 hypothetical protein SOVF_162980 [Spinacia oleracea]	gi|975887|gb|U32511.1|MCU32511:Mesembryanthemum crystallinum myo-inositol-1-phosphate synthase mRNA, complete cds	A0A0K9QNY1|A0A0K9QNY1_SPIOL: Uncharacterized protein;OS=Spinacia oleracea;|GO:0004512,|GO:0006021,GO:0008654,

geneid	trend	GO	Pathway	TF	NR	NT	Uniprot
DN122213_c0_g2_i1	UP	[GO:0030132:clathrin coat of coated pit][GO:0030130:clathrin coat of trans-Golgi network vesicle][GO:0005198:structural molecule activity][GO:0006886:intracellular protein transport][GO:0016192:vesicle-mediated transport]	[ko04142:Lysosome][ko04144:Endocytosis][ko04721:Synaptic vesicle cycle][ko04961:Endocrine and other factor-regulated calcium reabsorption][ko05016:Huntington's disease][ko05100:Bacterial invasion of epithelial cells]	Araip.0P3IP:Arachis ipaensis|M-type_MADS|M-type_MADS family protein	XP_021859505.1:clathrin heavy chain 1like [Spinacia oleracea] >KNA23309.1 hypothetical protein SOVF_026060 [Spinacia oleracea]	gi|359486393|ref|XM_002269869.2|:PREDICTED: Vitis vinifera clathrin heavy chain 1-like (LOC100256972), mRNA	A0A0K9RWL2|A0A0K9RWL2_SPIOL:Clathrin heavy chain;OS=Spinacia oleracea;|GO:0030132,GO:0030130,|GO:0005198,|GO:0006886,GO:0016192,
DN93395_c0_g1_i1	UP	-	-	-	KNA09101.1:hypothetical protein SOVF_156590 [Spinacia oleracea]	-	A0A0K9QRI7|A0A0K9QRI7_SPIOL:Uncharacterized protein;OS=Spinacia oleracea;
DN127985_c0_g4_i3	UP	[GO:0016021:integral component of membrane][GO:0015299:solute:proton antiporter activity][GO:0006813:potassium ion transport]	-	-	XP_021717525.1:K(+) efflux antiporter 2, chloroplasticlike [Chenopodium quinoa]	gi|521750544|gb|GAIA01000876.1|:TSA: Salicornia europaea Unigene892_Se200S transcribed RNA sequence	A0A0K9R694|A0A0K9R694_SPIOL:Uncharacterized protein;OS=Spinacia oleracea;|GO:0016021,|GO:0015299,|GO:0006813,

geneid	trend	GO	Pathway	TF	NR	NT	Uniprot
DN113723_c0_g1_i1	UP	[GO:0016021:integral component of membrane]	-	Pbr006809.1:Pyrus bretschneideri|M-type_MADS|M-type_MADS family protein	XP_021844163.1:uncharacterized protein PAM68like [Spinacia oleracea] >KNA17799.1 hypothetical protein SOVF_076950 [Spinacia oleracea]	gi|478163868|gb|GAHK01012735.1|:TSA: Jatropha curcas Unigene186_JC-CK_1A transcribed RNA sequence	A0A0K9RFP7|A0A0K9RFP7_SPIOL:Uncharacterized protein;OS=Spinacia oleracea;|GO:0016021,
DN124772_c1_g1_i1	UP	[GO:0005794:Golgi apparatus][GO:0009505:plant-type cell wall][GO:0009506:plasmodesma][GO:0004252:serine-type endopeptidase activity]	-	CCG021324.1:Populus euphratica|Trihelix|Trihelix family protein	XP_021858819.1:subtilisinlike protease SBT1.8 [Spinacia oleracea]	gi|502086016|ref|XM_004488025.1|:PREDICTED: Cicer arietinum subtilisin-like protease-like (LOC101515370), mRNA	A0A0J8B754|A0A0J8B754_BETVU:Uncharacterized protein;OS=Beta vulgaris subsp. vulgaris.;|GO:0005794,GO:0009505,GO:0009506,|GO:0004252,
DN125310_c4_g3_i1	UP	[GO:0016020:membrane][GO:0016798:hydrolase activity, acting on glycosyl bonds][GO:0005975:carbohydrate metabolic process]	[ko00531:Glycosaminoglycan degradation][ko05205:Proteoglycans in cancer]	-	XP_010665516.1:PREDICTED: heparanaselike protein 3 [Beta vulgaris subsp. vulgaris]	gi|359476170|ref|XM_002283218.2|:PREDICTED: Vitis vinifera heparanase-like protein 3 (LOC100264535), mRNA	A0A0J8CYZ9|A0A0J8CYZ9_BETVU:Uncharacterized protein;OS=Beta vulgaris subsp. vulgaris.;|GO:0016020,|GO:0016798,|GO:0005975,

geneid	trend	GO	Pathway	TF	NR	NT	Uniprot
DN127555_c3_g1_i6	UP	[GO:0005618:cell wall][GO:0005576:extracellular region][GO:0016020:membrane][GO:0009664:plant-type cell wall organization]	-	676732396:Sisymbrium irio|bHLH|bHLH family protein	XP_010690765.1:PREDICTED: expansinA4 [Beta vulgaris subsp. vulgaris] >KMT18338.1 hypothetical protein BVRB_2g024950 [Beta vulgaris subsp. vulgaris]	gi|356501064|ref|XM_003519301.1|:PREDICTED: Glycine max expansin-A4-like (LOC100803242), mRNA	A0A0J8D2C1|A0A0J8D2C1_BETVU:Uncharacterized protein;OS=Beta vulgaris subsp. vulgaris.;|GO:0005618,GO:0005576,GO:0016020,|GO:0009664,
DN117684_c1_g1_i1	UP	-	-	Bv_011610_tatm.t1: Beta vulgaris|BBR-BPC|BBR-BPC family protein	XP_021738421.1:protein BASIC PENTACYSTEINE 4like [Chenopodium quinoa]	gi|521693352|gb|GAIA01020905.1|:TSA: Salicornia europaea Unigene21902_Se200S transcribed RNA sequence	A0A0J8B270|A0A0J8B270_BETVU:Uncharacterized protein;OS=Beta vulgaris subsp. vulgaris.;
DN121300_c0_g1_i4	UP	[GO:0005829:cytosol][GO:0016021:integral component of membrane][GO:0009626:plant-type hypersensitive response]	-	-	XP_021774380.1:protein LAZ1like [Chenopodium quinoa]	-	A0A0J8BMI5|A0A0J8BMI5_BETVU:Uncharacterized protein;OS=Beta vulgaris subsp. vulgaris.;|GO:0005829,GO:0016021,|GO:0009626,

geneid	trend	GO	Pathway	TF	NR	NT	Uniprot
DN119204_c1_g7_i4	UP	[GO:0016791:phosphatase activity][GO:0005975:carbohydrate metabolic process]	[ko00710:Carbon fixation in photosynthetic organisms]	-	XP_021769134.1:sedoheptulose1,7bisphosphatase, chloroplastic [Chenopodium quinoa]	gi|2529375|gb|L76556.1|SPIS17B:Spinacia oleracea chloroplast sedoheptulose-1,7-bisphosphatase mRNA, complete cds	A0A0J8D260|A0A0J8D260_BETVU:Uncharacterized protein;OS=Beta vulgaris subsp. vulgaris.;|GO:0016791,|GO:0005975,
DN118931_c2_g1_i1	UP	-	-	-	-	-	-
DN113019_c1_g1_i4	UP	[GO:0030132:clathrin coat of coated pit][GO:0030130:clathrin coat of trans-Golgi network vesicle][GO:0005198:structural molecule activity][GO:0006886:intracellular protein transport][GO:0016192:vesicle-mediated transport]	[ko04142:Lysosome][ko04144:Endocytosis][ko04721:Synaptic vesicle cycle][ko04961:Endocrine and other factor-regulated calcium reabsorption][ko05016:Huntington's disease][ko05100:Bacterial invasion of epithelial cells]	GRMZM2G395549_P01:Zea mays|bHLH|bHLH family protein	XP_021859505.1:clathrin heavy chain 1like [Spinacia oleracea] >KNA23309.1 hypothetical protein SOVF_026060 [Spinacia oleracea]	gi|521458355|gb|GAHZ01062129.1|:TSA: Salicornia europaea Unigene74848_SeCKS transcribed RNA sequence	A0A0K9RWL2|A0A0K9RWL2_SPIOL:Clathrin heavy chain;OS=Spinacia oleracea;|GO:0030132,GO:0030130,|GO:0005198,|GO:0006886,GO:0016192,

geneid	trend	GO	Pathway	TF	NR	NT	Uniprot
DN127197_c1_g2_i12	UP	[GO:0005886:plasma membrane]	-	BGIOSGA035841-PA:Oryza sativa subsp. indica|MYB_related|MYB_related family protein	XP_010687037.1:PREDICTED: BTB/POZ domaincontaining protein At1g30440 [Beta vulgaris subsp. vulgaris] >KMT03943.1 hypothetical protein BVRB_8g187520 [Beta vulgaris subsp. vulgaris]	gi|478156161|gb|GAHK01020442.1|:TSA: Jatropha curcas Unigene7898_JC-CK_1A transcribed RNA sequence	A0A0J8BVH9|A0A0J8BVH9_BETVU: Uncharacterized protein;OS=Beta vulgaris subsp. vulgaris.;|GO:0005886,
DN118188_c1_g1_i4	UP	[GO:0046872:metal ion binding][GO:0030001:metal ion transport]	-	-	XP_021866915.1:heavy metalassociated isoprenylated plant protein 39like [Spinacia oleracea] >KNA20675.1 hypothetical protein SOVF_050230 [Spinacia oleracea]	-	A0A0K9RME2|A0A0K9RME2_SPIOL: Uncharacterized protein;OS=Spinacia oleracea;|GO:0046872,|GO:0030001,

geneid	trend	GO	Pathway	TF	NR	NT	Uniprot
DN118741_c1_g1_i1	UP	[GO:0003993:acid phosphatase activity][GO:0046872:metal ion binding]	-	Achn233011:Actinidia chinensis|SRS|SRS family protein	XP_008387992.1:PREDICTED: purple acid phosphatase 2like [Malus domestica]	gi|521553930|gb|GAIA01080347.1|:TSA: Salicornia europaea Unigene97505_Se200S transcribed RNA sequence	A5B1B3|A5B1B3_VITVI:Purple acid phosphatase;OS=Vitis vinifera;GN=VIT_18s0001g13340 {ECO:0000313|EMBL:CBI19779.3};|GO:0003993,GO:0046872,

geneid	trend	GO	Pathway	TF	NR	NT	Uniprot
DN121629_c0_g2_i13	UP	[GO:0005634:nucleus][GO:0000989:obsolete transcription factor activity, transcription factor binding][GO:0008270:zinc ion binding][GO:0009640:photomorphogenesis][GO:0006355:regulation of transcription, DNA-templated]	-	AHYPO_013514-RA:Amaranthus hypochondriacus|DBB|DBB family protein	XP_021626783.1:Bbox zinc finger protein 19like isoform X2 [Manihot esculenta] >XP_021626785.1 Bbox zinc finger protein 19like isoform X2 [Manihot esculenta] >XP_021626786.1 Bbox zinc finger protein 19like isoform X2 [Manihot esculenta] >XP_021626787.1 Bbox zinc finger protein 19like isoform X2 [Manihot esculenta] >OAY37193.1 hypothetical protein MANES_11G082100 [Manihot esculenta]	-	B9H0Y0|B9H0Y0_POPTR:Zinc finger family protein;OS=Populus trichocarpa;|GO:0005634,|GO:0000989,GO:0008270,|GO:0009640,GO:0006355,

geneid	trend	GO	Pathway	TF	NR	NT	Uniprot
					>OAY37195.1 hypothetical protein MANES_11G082100 [Manihot esculenta] >OAY37196.1 hypothetical protein MANES_11G082100 [Manihot esculenta]		

geneid	trend	GO	Pathway	TF	NR	NT	Uniprot
DN122123_c0_g2_i8	UP	[GO:0016021:integral component of membrane]	-	Medtr0036s0260.1: Medicago truncatula|MYB_related|MYB_related family protein	XP_010691542.1:PREDICTED: probable zinc metallopeptidase EGY3, chloroplastic isoform X1 [Beta vulgaris subsp. vulgaris] >KMT01013.1 hypothetical protein BVRB_9g222740 [Beta vulgaris subsp. vulgaris]	gi|521663387|gb|GAIA01035561.1|:TSA: Salicornia europaea Unigene39377_Se200S transcribed RNA sequence	A0A0J8BLU0|A0A0J8BLU0_BETVU:Uncharacterized protein;OS=Beta vulgaris subsp. vulgaris.;|GO:0016021,
DN120251_c0_g1_i2	UP	[GO:0005622:intracellular][GO:0008270:zinc ion binding]	-	Bv8_198850_yjyh.t1:Beta vulgaris|CO-like|CO-like family protein	XP_010688513.1:PREDICTED: zinc finger protein CONSTANSLIKE 5 [Beta vulgaris subsp. vulgaris] >KMT03349.1 hypothetical protein BVRB_8g198850 [Beta vulgaris subsp. vulgaris]	gi|359485004|ref|XM_002277917.2|:PREDICTED: Vitis vinifera zinc finger protein CONSTANS-LIKE 5-like (LOC100244502), mRNA	A0A0J8BTL1|A0A0J8BTL1_BETVU:Uncharacterized protein;OS=Beta vulgaris subsp. vulgaris.;|GO:0005622,|GO:0008270,

geneid	trend	GO	Pathway	TF	NR	NT	Uniprot
DN116895_c1_g2_i10	UP	-	-	GSBRNA2T00089266001:Brassica napus|MYB_related|MYB_related family protein	XP_010668016.1:PREDICTED: protein ELF4LIKE 4 [Beta vulgaris subsp. vulgaris] >XP_010668017.1 PREDICTED: protein ELF4LIKE 4 [Beta vulgaris subsp. vulgaris] >XP_010668018.1 PREDICTED: protein ELF4LIKE 4 [Beta vulgaris subsp. vulgaris] >XP_019103280.1 PREDICTED: protein ELF4LIKE 4 [Beta vulgaris subsp. vulgaris] >ACK56108.1 ELF4like protein [Beta vulgaris] >KMS94973.1 hypothetical protein	gi|521731944|gb|GAIA01010710.1|:TSA: Salicornia europaea Unigene11308_Se200S transcribed RNA sequence	C6ZKH2|C6ZKH2_BETVU:ELF4-like protein;OS=Beta vulgaris;

geneid	trend	GO	Pathway	TF	NR	NT	Uniprot
					BVRB_013600 [Beta vulgaris subsp. vulgaris]		

geneid	trend	GO	Pathway	TF	NR	NT	Uniprot
DN126404_c6_g1_i1	UP	[GO:0016021:integral component of membrane]	-	-	XP_010679114.1:PREDICTED: BI1like protein [Beta vulgaris subsp. vulgaris] >KMT10231.1 hypothetical protein BVRB_5g119910 [Beta vulgaris subsp. vulgaris]	gi|357111354|ref|XM_003557431.1|:PREDICTED: Brachypodium distachyon BI1-like protein-like (LOC100833424), mRNA	A0A0J8F3B7|A0A0J8F3B7_BETVU:Uncharacterized protein;OS=Beta vulgaris subsp. vulgaris.;|GO:0016021,
DN117506_c0_g1_i3	UP	-	-	-	XP_021757694.1:uncharacterized protein LOC110722714 [Chenopodium quinoa]	-	A0A0K9QWC6|A0A0K9QWC6_SPIOL:Uncharacterized protein;OS=Spinacia oleracea;
DN114148_c3_g1_i1	UP	-	-	AHYPO_012608-RA:Amaranthus hypochondriacus|TCP|TCP family protein	XP_021761649.1:transcription factor TCP14like [Chenopodium quinoa]	-	A0A0K9QR91|A0A0K9QR91_SPIOL:Uncharacterized protein;OS=Spinacia oleracea;

geneid	trend	GO	Pathway	TF	NR	NT	Uniprot
DN113842_c0_g1_i1	UP	[GO:0005576:extracellular region][GO:0030145:manganese ion binding][GO:0045735:nutrient reservoir activity]	-	678365117:Utricularia gibba|bZIP|bZIP family protein	XP_021858456.1:auxinbinding protein ABP19alike [Spinacia oleracea]	gi|521707224|gb|GAIA01014485.1|:TSA: Salicornia europaea Unigene15144_Se200S transcribed RNA sequence	A0A0J8DZ97|A0A0J8DZ97_BETVU:Uncharacterized protein;OS=Beta vulgaris subsp. vulgaris.;|GO:0005576,|GO:0030145,GO:0045735,
DN122193_c1_g1_i3	UP	[GO:0042742:defense response to bacterium][GO:0050832:defense response to fungus]	-	-	XP_021864961.1:pathogenesisrelated protein PR4like [Spinacia oleracea] >KNA21920.1 hypothetical protein SOVF_037190 [Spinacia oleracea]	gi|19965|emb|X58547.1|:N.tabacum PR-4B mRNA for pathogenesis-related protein 4B	A0A0K9RQV4|A0A0K9RQV4_SPIOL: Uncharacterized protein;OS=Spinacia oleracea;|GO:0042742,GO:0050832,
DN123721_c0_g1_i5	UP	[GO:0005789:endoplasmic reticulum membrane][GO:0016021:integral component of membrane][GO:0004579:dolichyl-diphosphooligosaccharide-protein glycotransferase activity][GO:0006486:protein glycosylation]	-	Sme2.5_01445.1_g00001.1:Solanum melongena|MYB|MYB family protein	XP_021771025.1:protein WVD2like 7 [Chenopodium quinoa]	-	A0A0K9QF52|A0A0K9QF52_SPIOL:Dolichyl-diphosphooligosaccharide--protein glycosyltransferase subunit 1;OS=Spinacia oleracea;|GO:0005789,GO:0016021,|GO

geneid	trend	GO	Pathway	TF	NR	NT	Uniprot
							:0004579,|GO:0006486,
DN112181_c0_g1_i3	UP	[GO:0005737:cytoplasm][GO:0005634:nucleus]	-	-	XP_021713938.1:protein BOLA2like [Chenopodium quinoa]	-	A0A0J8C6M5|A0A0J8C6M5_BETVU: Uncharacterized protein;OS=Beta vulgaris subsp. vulgaris.;|GO:0005737,GO:0005634,
DN122970_c0_g6_i5	UP	-	-	Zjn_sc00009.1.g01550.1.am.mk:Zoysia japonica|C2H2|C2H2 family protein	XP_021758255.1:methylesterase 17like [Chenopodium quinoa]	-	M5X1P8|M5X1P8_PRUPE:Uncharacterized protein;OS=Prunus persica;
DN115104_c0_g10_i1	UP	-	-	-	-	-	-

geneid	trend	GO	Pathway	TF	NR	NT	Uniprot
DN111960_c0_g1_i1	UP	[GO:0005623:cell][GO:0009055:electron transfer activity][GO:0015035:protein disulfide oxidoreductase activity][GO:0045454:cell redox homeostasis]	-	-	XP_021726538.1:glutaredoxinC9like [Chenopodium quinoa]	-	A0A0J8ENN1|A0A0J8ENN1_BETVU: Uncharacterized protein;OS=Beta vulgaris subsp. vulgaris.;|GO:0005623,|GO:0009055,GO:0015035,|GO:0045454,
DN128213_c1_g1_i10	UP	[GO:0009570:chloroplast stroma][GO:0009535:chloroplast thylakoid membrane][GO:0005524:ATP binding][GO:0017038:protein import][GO:0006605:protein targeting]	[ko02024:Quorum sensing][ko03060:Protein export][ko03070:Bacterial secretion system]	-	XP_010679492.1:PREDICTED: protein translocase subunit SecA, chloroplastic [Beta vulgaris subsp. vulgaris]	gi|939975|emb|Z49124.1|:S.oleracea chloroplast secA mRNA	Q36795|SECA_SPIOL:Protein translocase subunit SecA, chloroplastic;OS=Spinacia oleracea;|GO:0009570,GO:0009535,|GO:0005524,|GO:0017038,GO:0006605,
DN121914_c0_g1_i3	UP	[GO:0005886:plasma membrane]	-	-	XP_021746525.1:protein JINGUBANGlike [Chenopodium quinoa]	gi|521652245|gb|GAIA01040614.1|:TSA: Salicornia europaea Unigene45631_Se200S transcribed RNA sequence	A0A0J8C5X1|A0A0J8C5X1_BETVU:Uncharacterized protein;OS=Beta vulgaris subsp. vulgaris.;|GO:00058

geneid	trend	GO	Pathway	TF	NR	NT	Uniprot
							86,
DN125073_c4_g1_i2	UP	[GO:0016021:integral component of membrane]	-	MDP0000321461:Malus domestica|G2-like|G2-like family protein	XP_021770841.1:sugar transporter ERD6like 16 [Chenopodium quinoa]	-	A0A0J8B4N7|A0A0J8B4N7_BETVU:Uncharacterized protein;OS=Beta vulgaris subsp. vulgaris.;|GO:0016021,|GO:0022891,
DN128302_c8_g3_i4	UP	-	-	-	-	-	-
DN125274_c1_g8_i4	UP	[GO:0000287:magnesium ion binding][GO:0010333:terpene synthase activity][GO:0008152:metabolic process]	-	Cotton_A_17926_BGI-A2_v1.0:Gossypium arboreum|GRAS|GRAS family protein	XP_010694277.1:PREDICTED: probable sesquiterpene synthase [Beta vulgaris subsp. vulgaris] >KMS98542.1 hypothetical protein BVRB_4g092310 [Beta vulgaris subsp.	-	A0A0J8BEZ1|A0A0J8BEZ1_BETVU:Uncharacterized protein;OS=Beta vulgaris subsp. vulgaris.;|GO:0000287,GO:0010333,|GO:0008152,

geneid	trend	GO	Pathway	TF	NR	NT	Uniprot
					vulgaris]		
DN127603_c 1_g1_i2	UP	-	-	678320713:Utricularia gibba|M-type_MADS|M-type_MADS family protein	XP_010666903.1:PREDICTED: protein TSS [Beta vulgaris subsp. vulgaris] >KMT20309.1 hypothetical protein BVRB_1g003100 [Beta vulgaris subsp. vulgaris]	-	A0A0J8D7F3|A0A0J8D7F3_BETVU:Uncharacterized protein;OS=Beta vulgaris subsp. vulgaris.;
DN117800_c 3_g3_i2	UP	[GO:0004332:fructose-bisphosphate aldolase activity][GO:0006096:glycolytic process]	[ko00010:Glycolysis / Gluconeogenesis][ko00030:Pentose phosphate pathway][ko00051:Fructose and mannose metabolism][ko0068	FANhyb_rscf00000068.1.g00012.1:Fragaria x ananassa|FAR1|FAR1 family protein	AFI25162.1:fructose 1,6 bisphosphate aldolase class 1, partial [Moringa oleifera]	gi|521752072|gb|GAIA01000090.1|:TSA: Salicornia europaea Unigene93_Se200S transcribed RNA sequence	I1V1V2|I1V1V2_MOROL:Fructose-bisphosphate aldolase;OS=Moringa oleifera;|GO:0004332,|GO:0006096,

geneid	trend	GO	Pathway	TF	NR	NT	Uniprot
			0:Methane metabolism][ko00710:Carbon fixation in photosynthetic organisms]				
DN121695_c1_g1_i4	UP	[GO:0048573:photoperiodism, flowering]	-	Bv3_060840_gszt.t1:Beta vulgaris|bHLH|bHLH family protein	KMT15380.1:hypothetical protein BVRB_3g060840 [Beta vulgaris subsp. vulgaris]	-	A0A0J8CNP6|A0A0J8CNP6_BETVU:Uncharacterized protein;OS=Beta vulgaris subsp. vulgaris.;|GO:0048573,
DN127312_c4_g10_i1	UP	[GO:0005524:ATP binding]	-	-	AEI69347.1:chloroplast rubisco activase [Ophiopogon japonicus]	gi|21950711|gb|AY117142.1|:Chenopodium quinoa rubisco activase (rca) mRNA, complete cds	F8UAA7|F8UAA7_9ASPA:Chloroplast rubisco activase;OS=Ophiopogon japonicus.;|GO:0005524,

geneid	trend	GO	Pathway	TF	NR	NT	Uniprot
DN107141_c0_g12_i1	UP	[GO:0005737:cytoplasm][GO:0004364:glutathione transferase activity][GO:0006749:glutathione metabolic process][GO:0009407:toxin catabolic process]	[ko00480:Glutathione metabolism][ko00980:Metabolism of xenobiotics by cytochrome P450][ko00982:Drug metabolism - cytochrome P450][ko01524:Platinum drug resistance][ko05204:Chemical carcinogenesis][ko05418:Fluid shear stress and atherosclerosis]	-	XP_002280532.1:PREDICTED: probable glutathione Stransferase [Vitis vinifera]	-	F6GTS9|F6GTS9_VITVI:Putative uncharacterized protein;OS=Vitis vinifera;GN=VIT_17s0000g02950{ECO:0000313|EMBL:CCB43253.1};|GO:0005737,|GO:0004364,|GO:0006749,GO:0009407,
DN110363_c0_g1_i1	UP	-	-	-	-	-	-

geneid	trend	GO	Pathway	TF	NR	NT	Uniprot
DN121117_c0_g4_i1	UP	[GO:0005622:intracellular][GO:0008270:zinc ion binding]	-	Bv2_028660_twpr.t1:Beta vulgaris|CO-like|CO-like family protein	XP_010694604.1:PREDICTED: zinc finger protein CONSTANSLIKE 2 [Beta vulgaris subsp. vulgaris] >ACC95129.1 COL1 [Beta vulgaris subsp. vulgaris] >KMT18744.1 hypothetical protein BVRB_2g028660 [Beta vulgaris subsp. vulgaris]	-	B3TZZ7|B3TZZ7_BETVU:COL1;OS=Beta vulgaris subsp. vulgaris.;|GO:0005622,|GO:0008270,
DN127471_c0_g3_i1	UP	[GO:0006457:protein folding]	-	Achn036321:Actinidia chinensis|G2-like|G2-like family protein	XP_021645314.1:dnaJ homolog subfamily B member 13like [Hevea brasiliensis]	gi|502143991|ref|XM_004505483.1|:PREDICTED: Cicer arietinum dnaJ homolog subfamily B member 4-like (LOC101497975), mRNA	B9RVT4|B9RVT4_RICCO:Protein SIS1, putative;OS=Ricinus communis;|GO:0006457,

geneid	trend	GO	Pathway	TF	NR	NT	Uniprot
DN125274_c1_g8_i1	UP	[GO:0000287:magnesium ion binding][GO:0010333:terpene synthase activity][GO:0008152:metabolic process]	-	Cotton_A_17926_BGI-A2_v1.0:Gossypium arboreum|GRAS|GRAS family protein	XP_010694277.1:PREDICTED: probable sesquiterpene synthase [Beta vulgaris subsp. vulgaris] >KMS98542.1 hypothetical protein BVRB_4g092310 [Beta vulgaris subsp. vulgaris]	-	A0A0J8BEZ1|A0A0J8BEZ1_BETVU:Uncharacterized protein;OS=Beta vulgaris subsp. vulgaris.;|GO:0000287,GO:0010333,|GO:0008152,
DN117271_c1_g2_i1	UP	-	-	-	-	-	-
DN112556_c1_g1_i1	UP	[GO:0008124:4-alpha-hydroxytetrahydrobiopterin dehydratase activity][GO:0006729:tetrahydrobiopterin biosynthetic process]	-	-	XP_006437027.1:hypothetical protein CICLE_v10032722mg [Citrus clementina] >ESR50267.1 hypothetical protein CICLE_v10032722mg [Citrus clementina]	gi|521570517|gb|GAIA01073428.1|:TSA: Salicornia europaea Unigene89255_Se200S transcribed RNA sequence	A0A067ERK0|A0A067ERK0_CITSI:Uncharacterized protein;OS=Citrus sinensis;|GO:0008124,|GO:0006729,

geneid	trend	GO	Pathway	TF	NR	NT	Uniprot
DN127312_c4_g8_i1	UP	[GO:0005524:ATP binding]	-	-	OIW02691.1:hypothetical protein TanjilG_29467 [Lupinus angustifolius]	-	G7KGT1|G7KGT1_MEDTR:Ribulose bisphosphate carboxylase/oxygenase activase;OS=Medicago truncatula;GN=MTR_5g080450 {ECO:0000313|EMBL:AES99408.1};|GO:0005524,
DN126404_c6_g1_i12	UP	[GO:0016021:integral component of membrane]	-	-	XP_021843492.1:BI1like protein [Spinacia oleracea] >KNA05528.1 hypothetical protein SOVF_189500 [Spinacia oleracea]	gi|357111354|ref|XM_003557431.1|:PREDICTED: Brachypodium distachyon BI1-like protein-like (LOC100833424), mRNA	A0A0K9QFR5|A0A0K9QFR5_SPIOL:Uncharacterized protein;OS=Spinacia oleracea;|GO:0016021,

geneid	trend	GO	Pathway	TF	NR	NT	Uniprot
DN121946_c2_g1_i1	UP	[GO:0047443:4-hydroxy-4-methyl-2-oxoglutarate aldolase activity][GO:0046872:metal ion binding][GO:0008948:oxaloacetate decarboxylase activity][GO:0008428:ribonuclease inhibitor activity][GO:0051252:regulation of RNA metabolic process]	-	-	XP_010533431.1:PREDICTED: putative 4hydroxy4methyl2oxoglutarate aldolase 2 [Tarenaya hassleriana] >XP_010533433.1 PREDICTED: putative 4hydroxy4methyl2oxoglutarate aldolase 2 [Tarenaya hassleriana]	-	V7D0J0|V7D0J0_PHAVU:4-hydroxy-4-methyl-2-oxoglutarate aldolase;OS=Phaseolus vulgaris;|GO:0047443,GO:0046872,GO:0008948,GO:0008428,|GO:0051252,

geneid	trend	GO	Pathway	TF	NR	NT	Uniprot
DN116103_c3_g4_i2	UP	[GO:0008483:transaminase activity]	[ko00250:Alanine, aspartate and glutamate metabolism][ko00260:Glycine, serine and threonine metabolism][ko00630:Glyoxylate and dicarboxylate metabolism][ko00680:Methane metabolism][ko04146:Peroxisome]	-	XP_021593021.1:serineglyoxylate aminotransferaselike [Manihot esculenta] >XP_021593064.1 serineglyoxylate aminotransferaselike [Manihot esculenta] >OAY61337.1 hypothetical protein MANES_01G181700 [Manihot esculenta] >OAY61338.1 hypothetical protein MANES_01G181700 [Manihot esculenta]	gi|521651870|gb|GAIA01040863.1|:TSA: Salicornia europaea Unigene45909_Se200S transcribed RNA sequence	A0A0A6ZHA3|A0A0A6ZHA3_GOSHI: Serine--glyoxylate aminotransferase;OS=Gossypium hirsutum;|GO:0008483,
DN120899_c5_g9_i1	UP	[GO:0048046:apoplast]	-	-	KNA07534.1:hypothetical protein SOVF_170950 [Spinacia oleracea]	-	A0A0K9QJV7|A0A0K9QJV7_SPIOL:Dirigent protein;OS=Spinacia oleracea;|GO:0048046,

geneid	trend	GO	Pathway	TF	NR	NT	Uniprot
DN123288_c1_g2_i1	UP	-	-	-	XP_021769868.1:uncharacterized protein LOC110734122 [Chenopodium quinoa]	-	A0A0K9RNZ9|A0A0K9RNZ9_SPIOL: Uncharacterized protein;OS=Spinacia oleracea;
DN117404_c0_g1_i1	UP	[GO:0016747:transferase activity, transferring acyl groups other than amino-acyl groups]	[ko00940:Phenylpropanoid biosynthesis][ko00941:Flavonoid biosynthesis][ko00945:Stilbenoid, diarylheptanoid and gingerol biosynthesis]	Zpz_sc00135.1.g00200.1.am.mk:Zoysia pacifica|Nin-like|Nin-like family protein	XP_021772284.1:omegahydroxypalmitate Oferuloyl transferaselike [Chenopodium quinoa]	gi|225469070|ref|XM_002270293.1|:PREDICTED: Vitis vinifera omega-hydroxypalmitate O-feruloyl transferase-like (LOC100249378), mRNA	A0A0J8CRQ3|A0A0J8CRQ3_BETVU: Uncharacterized protein;OS=Beta vulgaris subsp. vulgaris.;|GO:0016747,
DN114136_c0_g1_i1	UP	[GO:0016021:integral component of membrane]	-	-	XP_021748490.1:NAD(P)Hquinone oxidoreductase subunit T, chloroplasticlike [Chenopodium quinoa]	-	A0A0J8D1T5|A0A0J8D1T5_BETVU:Uncharacterized protein;OS=Beta vulgaris subsp. vulgaris.;|GO:0016021,
DN125651_c7_g2_i1	UP	-	-	-	-	-	-

geneid	trend	GO	Pathway	TF	NR	NT	Uniprot
DN115464_c1_g2_i2	UP	[GO:0005524:ATP binding][GO:0006457:protein folding]	[ko03040:Spliceosome][ko04010:MAPK signaling pathway][ko04141:Protein processing in endoplasmic reticulum][ko04144:Endocytosis][ko04213:Longevity regulating pathway - multiple species][ko04612:Antigen processing and presentation][ko04915:Estrogen signaling pathway][ko05134:Legionellosis][ko05145:Toxoplasmosis][ko05162:Measles][ko05164:Influenza A][ko05169:Epstein-Barr virus infection]	kfl00190_0180:Klebsormidium flaccidum|bHLH|bHLH family protein	XP_021748808.1:stromal 70 kDa heat shockrelated protein, chloroplasticlike [Chenopodium quinoa]	gi|225460327|ref|XM_002282766.1|:PREDICTED: Vitis vinifera stromal 70 kDa heat shock-related protein, chloroplastic-like (LOC100248235), mRNA	A0A067DN94|A0A067DN94_CITSI:Uncharacterized protein;OS=Citrus sinensis;|GO:0005524,|GO:0006457,

geneid	trend	GO	Pathway	TF	NR	NT	Uniprot
DN126446_c0_g2_i4	UP	[GO:0005829:cytosol]	-	-	XP_010686761.1:PREDICTED: HSPinteracting protein [Beta vulgaris subsp. vulgaris] >KMT04074.1 hypothetical protein BVRB_8g185280 [Beta vulgaris subsp. vulgaris]	gi|521522835|gb|GAHZ01012836.1|:TSA: Salicornia europaea Unigene13475_SeCKS transcribed RNA sequence	A0A0J8BVR4|A0A0J8BVR4_BETVU: Uncharacterized protein;OS=Beta vulgaris subsp. vulgaris.;|GO:0005829,
DN126451_c1_g6_i3	UP	[GO:0009507:chloroplast][GO:0005524:ATP binding][GO:0004672:protein kinase activity][GO:0042548:regulation of photosynthesis, light reaction]	-	-	XP_021723885.1:serine/threonineprotein kinase STN7, chloroplasticlike [Chenopodium quinoa]	gi|521453095|gb|GAHZ01067389.1|:TSA: Salicornia europaea Unigene81418_SeCKS transcribed RNA sequence	A0A0J8E2Q9|A0A0J8E2Q9_BETVU:Uncharacterized protein;OS=Beta vulgaris subsp. vulgaris.;|GO:0009507,|GO:0005524,GO:0004672,|GO:0042548,
DN125614_c0_g1_i10	UP	[GO:0016021:integral component of membrane]	-	-	XP_021732440.1:probable zinc metalloprotease EGY2, chloroplastic isoform X1	gi|224098532|ref|XM_002311173.1|:Populus trichocarpa predicted protein, mRNA	A0A0K9RPS9|A0A0K9RPS9_SPIOL:Uncharacterized protein;OS=Spinacia oleracea;|GO:00160

geneid	trend	GO	Pathway	TF	NR	NT	Uniprot
					[Chenopodium quinoa]		21,
DN128908_c5_g1_i1	UP	[GO:0005634:nucleus][GO:0000977:RNA polymerase II regulatory region sequence-specific DNA binding][GO:0003700:DNA-binding transcription factor activity][GO:0000165:MAPK cascade][GO:0045944:positive regulation of transcription by RNA polymerase II][GO:0006351:transcription, DNA-templated]	-	Bv9_214370_scjj.t1:Beta vulgaris|MIKC_MADS|MIKC_MADS family protein	XP_010690400.1:PREDICTED: MADSbox protein AGL24 [Beta vulgaris subsp. vulgaris] >XP_010690401.1 PREDICTED: MADSbox protein AGL24 [Beta vulgaris subsp. vulgaris] >XP_010690402.1 PREDICTED: MADSbox protein AGL24 [Beta vulgaris subsp. vulgaris] >KMT01428.1 hypothetical protein	-	A0A0J8BN34|A0A0J8BN34_BETVU:Uncharacterized protein;OS=Beta vulgaris subsp. vulgaris.;|GO:0005634,|GO:0000977,GO:0003700,|GO:0000165,GO:0045944,GO:0006351,

geneid	trend	GO	Pathway	TF	NR	NT	Uniprot
					BVRB_9g214370 [Beta vulgaris subsp. vulgaris]		
DN125455_c3_g1_i4	UP	[GO:0016874:ligase activity]	[ko00280:Valine, leucine and isoleucine degradation]	-	XP_021717696.1:methylcrotonoylCoA carboxylase beta chain, mitochondriallike [Chenopodium quinoa]	gi|521549652|gb|GAIA01082640.1|:TSA: Salicornia europaea Unigene100825_Se200S transcribed RNA sequence	A0A0K9RKN1|A0A0K9RKN1_SPIOL: Uncharacterized protein;OS=Spinacia oleracea;|GO:0016874,

geneid	trend	GO	Pathway	TF	NR	NT	Uniprot
DN107577_c0_g2_i1	UP	[GO:0016021:integral component of membrane][GO:0009523:photosystem II][GO:0030145:manganese ion binding][GO:0015979:photosynthesis]	[ko00195:Photosynthesis]	-	XP_010682367.1:PREDICTED: photosystem II core complex proteins psbY, chloroplastic [Beta vulgaris subsp. vulgaris] >KMT07620.1 hypothetical protein BVRB_6g147470 [Beta vulgaris subsp. vulgaris]	gi|502140289|ref|XM_004504093.1|:PREDICTED: Cicer arietinum photosystem II core complex proteins psbY, chloroplastic-like (LOC101506330), mRNA	A0A0J8C1H5|A0A0J8C1H5_BETVU:Uncharacterized protein;OS=Beta vulgaris subsp. vulgaris.;|GO:0016021,GO:0009523,|GO:0030145,|GO:0015979,
DN125791_c1_g6_i1	UP	-	-	-	EMS46064.1:hypothetical protein TRIUR3_04043 [Triticum urartu]	-	A0A1D5T4G6|A0A1D5T4G6_WHEAT:Uncharacterized protein;OS=Triticum aestivum;

geneid	trend	GO	Pathway	TF	NR	NT	Uniprot
DN126109_c4_g1_i1	UP	[GO:0009941:chloroplast envelope][GO:0009570:chloroplast stroma][GO:0016787:hydrolase activity][GO:0008152:metabolic process]	-	-	XP_010695958.1:PREDICTED: haloacid dehalogenaselike hydrolase domaincontaining protein At3g48420 [Beta vulgaris subsp. vulgaris] >KMS97233.1 hypothetical protein BVRB_7g176810 [Beta vulgaris subsp. vulgaris]	gi|521695369|gb|GAIA01019894.1|:TSA: Salicornia europaea Unigene20869_Se200S transcribed RNA sequence	A0A0J8BB53|A0A0J8BB53_BETVU:Uncharacterized protein;OS=Beta vulgaris subsp. vulgaris.;|GO:0009941,GO:0009570,|GO:0016787,|GO:0008152,
DN123384_c0_g2_i19	UP	[GO:0016021:integral component of membrane][GO:0016760:cellulose synthase (UDP-forming) activity][GO:0071555:cell wall organization][GO:0030244:cellulose biosynthetic process]	-	Sme2.5_02298.1_g00001.1:Solanum melongena|ARF|ARF family protein	XP_021765961.1:cellulose synthaselike protein G2 [Chenopodium quinoa]	-	A0A0K9RFU8|A0A0K9RFU8_SPIOL:Uncharacterized protein;OS=Spinacia oleracea;|GO:0016021,|GO:0016760,|GO:0071555,GO:0030244,

geneid	trend	GO	Pathway	TF	NR	NT	Uniprot
DN103858_c0_g1_i1	UP	-	-	mrna01851.1-v1.0-hybrid:Fragaria vesca|NAC|NAC family protein	XP_008360721.1:PREDICTED: uncharacterized protein LOC103424417 [Malus domestica]	-	A0A0K9QXW6|A0A0K9QXW6_SPIOL:Uncharacterized protein;OS=Spinacia oleracea;
DN125274_c1_g8_i5	UP	[GO:0000287:magnesium ion binding][GO:0010333:terpene synthase activity][GO:0008152:metabolic process]	-	Cotton_A_17926_BGI-A2_v1.0:Gossypium arboreum|GRAS|GRAS family protein	XP_010694277.1:PREDICTED: probable sesquiterpene synthase [Beta vulgaris subsp. vulgaris] >KMS98542.1 hypothetical protein BVRB_4g092310 [Beta vulgaris subsp. vulgaris]	-	A0A0J8BEZ1|A0A0J8BEZ1_BETVU:Uncharacterized protein;OS=Beta vulgaris subsp. vulgaris.;|GO:0000287,GO:0010333,|GO:0008152,
DN116124_c2_g14_i1	UP	[GO:0005576:extracellular region]	[ko04016:MAPK signaling pathway - plant][ko04075:Plant hormone signal transduction][ko04626:Plant-pathogen interaction]	PSME_00037622-RA:Pseudotsuga menziesii|HD-ZIP|HD-ZIP family protein	XP_021776323.1:pathogenesisrelated protein PRB13like, partial [Chenopodium quinoa]	-	A0A0J8BJ14|A0A0J8BJ14_BETVU:Uncharacterized protein;OS=Beta vulgaris subsp. vulgaris.;|GO:0005576,

geneid	trend	GO	Pathway	TF	NR	NT	Uniprot
DN114285_c0_g1_i1	UP	-	-	GRMZM5G866699_P02:Zea mays|NF-YB|NF-YB family protein	XP_010671464.1:PREDICTED: zinc finger protein CONSTANSLIKE 7 isoform X1 [Beta vulgaris subsp. vulgaris] >KMT16032.1 hypothetical protein BVRB_3g052070 isoform A [Beta vulgaris subsp. vulgaris]	-	A0A0J8CR38|A0A0J8CR38_BETVU:Uncharacterized protein;OS=Beta vulgaris subsp. vulgaris.;
DN124309_c0_g1_i9	UP	-	-	-	KNA03916.1:hypothetical protein SOVF_204650 [Spinacia oleracea]	-	A0A0K9QBJ0|A0A0K9QBJ0_SPIOL:Uncharacterized protein;OS=Spinacia oleracea;

geneid	trend	GO	Pathway	TF	NR	NT	Uniprot
DN126430_c3_g2_i6	UP	-	[ko00710:Carbon fixation in photosynthetic organisms]	MDP0000275412:Malus domestica|Trihelix|Trihelix family protein	AOA52388.1:glyceraldehyde3phosphate dehydrogenase [Haloxylon ammodendron]	gi|2529369|gb|L76552.1|SPIGDND:Spinacia oleracea NADP-dependent glyceraldehydephosphate dehydrogenase subunit A mRNA, chloroplast gene encoding chloroplast protein, complete cds	A0A1B2LUN5|A0A1B2LUN5_9CARY:Glyceraldehyde-3-phosphate dehydrogenase;OS=Haloxylon ammodendron.;
DN116628_c0_g1_i3	UP	[GO:0009535:chloroplast thylakoid membrane][GO:0010598:NAD(P)H dehydrogenase complex (plastoquinone)]	-	-	XP_021733867.1:photosynthetic NDH subunit of subcomplex B 4, chloroplasticlike [Chenopodium quinoa]	gi|460219952|gb|GABL01044771.1|:TSA: Camelina sativa comp52189_c0_seq1 transcribed RNA sequence	A0A0J8CAI8|A0A0J8CAI8_BETVU:Uncharacterized protein;OS=Beta vulgaris subsp. vulgaris.;|GO:0009535,GO:0010598,
DN116772_c5_g7_i1	UP	-	-	-	-	-	-

geneid	trend	GO	Pathway	TF	NR	NT	Uniprot
DN127252_c4_g3_i1	UP	[GO:0004565:beta-galactosidase activity][GO:0005975:carbohydrate metabolic process]	-	-	AMN14862.1:putative betagalactosidase 3, partial [Nepenthes mirabilis]	gi|227053552|gb|FJ859031.1|:Carica papaya cultivar Eksotika beta-galactosidase pBG(a) mRNA, complete cds	A0A140GMM4|A0A140GMM4_NEPMI:Beta-galactosidase;OS=Nepenthes mirabilis;|GO:0004565,|GO:0005975,
DN117658_c4_g7_i3	UP	[GO:0009535:chloroplast thylakoid membrane][GO:0016021:integral component of membrane][GO:0010598:NAD(P)H dehydrogenase complex (plastoquinone)]	-	-	XP_010669327.1:PREDICTED: NAD(P)Hquinone oxidoreductase subunit U, chloroplastic [Beta vulgaris subsp. vulgaris] >KMT17865.1 hypothetical protein BVRB_2g033980 [Beta vulgaris subsp. vulgaris]	gi|255550148|ref|XM_002516079.1|:Ricinus communis heat shock protein binding protein, putative, mRNA	A0A0J8CVW6|A0A0J8CVW6_BETVU:Uncharacterized protein;OS=Beta vulgaris subsp. vulgaris.;|GO:0009535,GO:0016021,GO:0010598,
DN128396_c4_g13_i2	UP	-	-	-	ACL81159.1:putative auxin/aluminumresponsive protein [Mirabilis jalapa]	-	B8YIB1|B8YIB1_MIRJA:Putative auxin/aluminum-responsive protein;OS=Mirabili

geneid	trend	GO	Pathway	TF	NR	NT	Uniprot
							s jalapa;
DN126095_c3_g2_i1	UP	[GO:0005524:ATP binding][GO:0004672:protein kinase activity]	-	Pp3c12_5150V3.2.p:Physcomitrella patens|BBR-BPC|BBR-BPC family protein	XP_010672128.1:PREDICTED: serine/threonineprotein kinase BLUS1 [Beta vulgaris subsp. vulgaris] >KMT15689.1 hypothetical protein BVRB_3g056750 [Beta vulgaris subsp. vulgaris]	-	A0A0J8CUF9|A0A0J8CUF9_BETVU:Uncharacterized protein;OS=Beta vulgaris subsp. vulgaris.;|GO:0005524,GO:0004672,

geneid	trend	GO	Pathway	TF	NR	NT	Uniprot
DN127098_c2_g1_i1	UP	[GO:0009507:chloroplast][GO:0005524:ATP binding][GO:0016301:kinase activity][GO:0046872:metal ion binding][GO:0050242:pyruvate, phosphate dikinase activity][GO:0015979:photosynthesis][GO:0006090:pyruvate metabolic process]	[ko00620:Pyruvate metabolism][ko00710:Carbon fixation in photosynthetic organisms][ko00720:Carbon fixation pathways in prokaryotes]	-	Q42910.1:RecName: Full=Pyruvate, phosphate dikinase, chloroplastic; AltName: Full=Pyruvate, orthophosphate dikinase; Flags: Precursor >CAA57872.1 pyruvate,orthophosphate dikinase [Mesembryanthemum crystallinum]	gi|559470|emb|X78347.1|:M.crystallinum PPDK mRNA	Q42910|PPDK_MESCR:Pyruvate, phosphate dikinase, chloroplastic;OS=Mesembryanthemum crystallinum;|GO:0009507,|GO:0005524,GO:0016301,GO:0046872,GO:0050242,|GO:0015979,GO:0006090,
DN112726_c1_g7_i1	UP	-	-	-	-	-	-
DN117745_c0_g1_i1	UP	-	-	-	XP_021836130.1:uncharacterized protein LOC110775844 [Spinacia oleracea] >KNA23402.1 hypothetical protein SOVF_025130 [Spinacia oleracea]	gi|478149793|gb|GAHK01026810.1|:TSA: Jatropha curcas Unigene14273_JC-CK_1A transcribed RNA sequence	A0A0K9RX23|A0A0K9RX23_SPIOL:Uncharacterized protein;OS=Spinacia oleracea;

geneid	trend	GO	Pathway	TF	NR	NT	Uniprot
DN109074_c0_g1_i1	UP	-	-	-	EEF33835.1:conserved hypothetical protein [Ricinus communis]	-	B9SRA4|B9SRA4_RICCO:Putative uncharacterized protein;OS=Ricinus communis;
DN118225_c3_g1_i1	UP	[GO:0016758:transferase activity, transferring hexosyl groups][GO:0008152:metabolic process]	-	Tp57577_TGAC_v2_mRNA33215:Trifolium pratense|bHLH|bHLH family protein	XP_021764994.1:UDPglycosyltransferase 88B1like [Chenopodium quinoa]	-	A0A0J8FK21|A0A0J8FK21_BETVU:Glycosyltransferase;OS=Beta vulgaris subsp. vulgaris.;|GO:0016758,|GO:0008152,
DN124915_c0_g1_i6	UP	[GO:0009941:chloroplast envelope][GO:0009570:chloroplast stroma][GO:0005739:mitochondrion][GO:0005524:ATP binding][GO:0016485:protein processing][GO:0009408:response to heat]	[ko04213:Longevity regulating pathway - multiple species]	Achn116231:Actinidia chinensis|ARF|ARF family protein	XP_010665999.1:PREDICTED: chaperone protein ClpB4, mitochondrial [Beta vulgaris subsp. vulgaris] >KMT19033.1 hypothetical protein BVRB_2g031280 [Beta vulgaris subsp. vulgaris]	gi|470114072|ref|XM_004293194.1|:PREDICTED: Fragaria vesca subsp. vesca chaperone protein ClpB3, mitochondrial-like (LOC101293371), mRNA	A0A0J8D4G2|A0A0J8D4G2_BETVU:Uncharacterized protein;OS=Beta vulgaris subsp. vulgaris.;|GO:0009941,GO:0009570,GO:0005739,|GO:0005524,|GO:0016485,GO:0009408,

geneid	trend	GO	Pathway	TF	NR	NT	Uniprot
DN127985_c0_g7_i2	UP	[GO:0016021:integral component of membrane][GO:0015299:solute:proton antiporter activity][GO:0006813:potassium ion transport]	-	-	XP_010671923.1:PREDICTED: K(+) efflux antiporter 2, chloroplastic [Beta vulgaris subsp. vulgaris] >KMT20607.1 hypothetical protein BVRB_1g005870 [Beta vulgaris subsp. vulgaris]	gi|224067865|ref|XM_002302536.1|:Populus trichocarpa potassium efflux antiporter, mRNA	A0A0J8D465|A0A0J8D465_BETVU:Uncharacterized protein;OS=Beta vulgaris subsp. vulgaris.;|GO:0016021,|GO:0015299,|GO:0006813,
DN117139_c4_g1_i4	UP	[GO:0016021:integral component of membrane]	-	-	KNA16430.1:hypothetical protein SOVF_089020 [Spinacia oleracea]	gi|224138099|ref|XM_002326482.1|:Populus trichocarpa predicted protein, mRNA	A0A0K9RAB5|A0A0K9RAB5_SPIOL:Uncharacterized protein;OS=Spinacia oleracea;|GO:0016021,
DN129195_c56_g4_i5	UP	-	[ko00902:Monoterpenoid biosynthesis]	-	XP_021849477.1:(+)neomenthol dehydrogenaselike [Spinacia oleracea] >KNA15032.1 hypothetical protein SOVF_101870 [Spinacia oleracea]	gi|359489757|ref|XM_002271991.2|:PREDICTED: Vitis vinifera (+)-neomenthol dehydrogenase-like (LOC100255075), mRNA	A0A0K9R8D1|A0A0K9R8D1_SPIOL:Uncharacterized protein;OS=Spinacia oleracea;

geneid	trend	GO	Pathway	TF	NR	NT	Uniprot
DN127670_c12_g2_i6	UP	-	-	-	-	-	-
DN125442_c0_g2_i3	UP	[GO:0051513:regulation of monopolar cell growth]	-	-	XP_010679143.1:PREDICTED: protein LONGIFOLIA 1 isoform X2 [Beta vulgaris subsp. vulgaris] >KMT10257.1 hypothetical protein BVRB_5g120140 [Beta vulgaris subsp. vulgaris]	gi|521688991|gb|GAIA01023271.1|:TSA: Salicornia europaea Unigene24880_Se200S transcribed RNA sequence	A0A0J8C9J6|A0A0J8C9J6_BETVU:Uncharacterized protein;OS=Beta vulgaris subsp. vulgaris.;|GO:0051513,
DN115194_c0_g10_i2	UP	[GO:0009570:chloroplast stroma][GO:0005829:cytosol][GO:0046872:metal ion binding][GO:0016491:oxidoreductase activity][GO:0006950:response to stress][GO:0009228:thiamine biosynthetic process][GO:0052837:thiazole biosynthetic process]	[ko00730:Thiamine metabolism]	-	XP_021758810.1:thiamine thiazole synthase, chloroplasticlike [Chenopodium quinoa]	gi|502140799|ref|XM_004504285.1|:PREDICTED: Cicer arietinum thiamine thiazole synthase, chloroplastic-like (LOC101512227), mRNA	A0A0J8CT45|A0A0J8CT45_BETVU:Thiamine thiazole synthase, chloroplastic;OS=Beta vulgaris subsp. vulgaris.;|GO:0009570,GO:0005829,|GO:0046872,GO:0016491,|GO:0006950,GO:0009228,GO:0052837,

geneid	trend	GO	Pathway	TF	NR	NT	Uniprot
DN119111_c1_g2_i3	UP	-	-	-	XP_010680569.1:PREDICTED: PLAT domaincontaining protein 3 [Beta vulgaris subsp. vulgaris] >KMT08807.1 hypothetical protein BVRB_6g135310 [Beta vulgaris subsp. vulgaris]	-	A0A0J8C5H7|A0A0J8C5H7_BETVU:Uncharacterized protein;OS=Beta vulgaris subsp. vulgaris.;
DN112346_c0_g3_i2	UP	-	-	-	-	-	-
DN120171_c0_g14_i1	UP	[GO:0009507:chloroplast][GO:0004497:monooxygenase activity][GO:0016984:ribulose-bisphosphate carboxylase activity][GO:0009853:photorespiration][GO:0019253:reductive pentose-phosphate cycle]	[ko00630:Glyoxylate and dicarboxylate metabolism][ko00710:Carbon fixation in photosynthetic organisms]	mrna16933.1-v1.0-hybrid:Fragaria vesca|M-type_MADS|M-type_MADS family protein	Q04450.1:RecName: Full=Ribulose bisphosphate carboxylase small chain 2, chloroplastic; Short=RuBisCO small subunit 2; Flags: Precursor >AAA03694.1 ribulose1,5bisphosphate	gi|521548633|gb|GAHZ01000024.1|:TSA: Salicornia europaea Unigene24_SeCKS transcribed RNA sequence	Q04450|RBS2_MESCR:Ribulose bisphosphate carboxylase small chain 2, chloroplastic;OS=Mesembryanthemum crystallinum;|GO:0009507,|GO:0004497,GO:0016984,|GO:0009853,GO:0019253,

geneid	trend	GO	Pathway	TF	NR	NT	Uniprot
					carboxylase/oxygenase small subunit [Mesembryanthemum crystallinum]		
DN115641_c1_g3_i2	UP	[GO:0009522:photosystem I][GO:0015979:photosynthesis]	-	-	XP_010691548.1:PREDICTED: uncharacterized protein LOC104904849 [Beta vulgaris subsp. vulgaris] >KMT01019.1 hypothetical protein BVRB_9g222810 [Beta vulgaris subsp. vulgaris]	-	A0A0J8BLZ5|A0A0J8BLZ5_BETVU:Uncharacterized protein;OS=Beta vulgaris subsp. vulgaris.;|GO:0009522,|GO:0015979,

geneid	trend	GO	Pathway	TF	NR	NT	Uniprot
DN122358_c2_g1_i3	UP	[GO:0004512:inositol-3-phosphate synthase activity][GO:0006021:inositol biosynthetic process][GO:0008654:phospholipid biosynthetic process]	[ko00521:Streptomycin biosynthesis][ko00562:Inositol phosphate metabolism]	-	XP_021861771.1:inositol3phosphate synthase [Spinacia oleracea] >KNA08398.1 hypothetical protein SOVF_162980 [Spinacia oleracea]	gi|975887|gb|U32511.1|MCU32511:Mesembryanthemum crystallinum myo-inositol-1-phosphate synthase mRNA, complete cds	A0A0K9QNY1|A0A0K9QNY1_SPIOL: Uncharacterized protein;OS=Spinacia oleracea;|GO:0004512,|GO:0006021,GO:0008654,
DN127412_c3_g19_i1	UP	-	-	-	-	-	-
DN127098_c2_g1_i3	UP	[GO:0009507:chloroplast][GO:0005524:ATP binding][GO:0016301:kinase activity][GO:0046872:metal ion binding][GO:0050242:pyruvate, phosphate dikinase activity][GO:0015979:photosynthesis][GO:0006090:pyruvate metabolic process]	[ko00620:Pyruvate metabolism][ko00710:Carbon fixation in photosynthetic organisms][ko00720:Carbon fixation pathways in prokaryotes]	-	CAA55143.1:pyruvate,orthophosphate dikinase [Mesembryanthemum crystallinum]	gi|559470|emb|X78347.1|:M.crystallinum PPDK mRNA	Q42910|PPDK_MESCR:Pyruvate, phosphate dikinase, chloroplastic;OS=Mesembryanthemum crystallinum;|GO:0009507,|GO:0005524,GO:0016301,GO:0046872,GO:0050242,|GO:0015979,GO:0006090,

geneid	trend	GO	Pathway	TF	NR	NT	Uniprot
DN118917_c3_g1_i3	UP	[GO:0005634:nucleus][GO:0000977:RNA polymerase II regulatory region sequence-specific DNA binding][GO:0003700:DNA-binding transcription factor activity][GO:0000900:translation repressor activity, mRNA regulatory element binding][GO:0048438:floral whorl development][GO:0000165:MAPK cascade][GO:0009910:negative regulation of flower development][GO:0045892:negative regulation of transcription, DNA-templated][GO:0045944:positive regulation of transcription by RNA polymerase II][GO:0009266:response to temperature stimulus][GO:0006351:transcription, DNA-templated]	-	Bv6_133680_nuri.t1:Beta vulgaris|MIKC_MADS|MIKC_MADS family protein	XP_010680338.1:PREDICTED: MADSbox protein JOINTLESS [Beta vulgaris subsp. vulgaris] >KMT09268.1 hypothetical protein BVRB_6g133680 [Beta vulgaris subsp. vulgaris]	-	A0A0J8CB89|A0A0J8CB89_BETVU:Uncharacterized protein;OS=Beta vulgaris subsp. vulgaris.;|GO:0005634,|GO:0000977,GO:0003700,GO:0000900,|GO:0048438,GO:0000165,GO:0009910,GO:0045892,GO:0045944,GO:0009266,GO:0006351,

geneid	trend	GO	Pathway	TF	NR	NT	Uniprot
DN116604_c0_g1_i2	UP	-	-	Bv3_069990_sghj.t1:Beta vulgaris|CPP|CPP family protein	XP_010694035.1:PREDICTED: protein tesmin/TSO1like CXC 2 [Beta vulgaris subsp. vulgaris] >KMS98644.1 hypothetical protein BVRB_3g069990 [Beta vulgaris subsp. vulgaris]	gi|356538171|ref|XM_003537530.1|:PREDICTED: Glycine max uncharacterized protein LOC100815907 (LOC100815907), mRNA	A0A0J8BB92|A0A0J8BB92_BETVU:Uncharacterized protein;OS=Beta vulgaris subsp. vulgaris.;
DN128494_c1_g3_i2	UP	-	-	cra_locus_5362_iso_4:Catharanthus roseus|MYB_related|MYB_related family protein	XP_010666903.1:PREDICTED: protein TSS [Beta vulgaris subsp. vulgaris] >KMT20309.1 hypothetical protein BVRB_1g003100 [Beta vulgaris subsp. vulgaris]	gi|521502684|gb|GAHZ01028236.1|:TSA: Salicornia europaea Unigene31091_SeCKS transcribed RNA sequence	A0A0J8D7F3|A0A0J8D7F3_BETVU:Uncharacterized protein;OS=Beta vulgaris subsp. vulgaris.;

geneid	trend	GO	Pathway	TF	NR	NT	Uniprot
DN127680_c0_g2_i7	UP	[GO:0016021:integral component of membrane][GO:0005524:ATP binding][GO:0042626:ATPase activity, coupled to transmembrane movement of substances]	-	RrC2480_p5:Raphanus raphanistrum|HSF|HSF family protein	XP_021853038.1:putative ABC transporter C family member 15 [Spinacia oleracea]	gi|255588744|ref|XM_002534659.1|:Ricinus communis multidrug resistance-associated protein 2, 6 (mrp2, 6), abc-transoprter, putative, mRNA	A0A0J8BIE8|A0A0J8BIE8_BETVU:Uncharacterized protein;OS=Beta vulgaris subsp. vulgaris.;|GO:0016021,|GO:0005524,GO:0042626,
DN119512_c4_g1_i2	UP	[GO:0051287:NAD binding][GO:0050661:NADP binding][GO:0016620:oxidoreductase activity, acting on the aldehyde or oxo group of donors, NAD or NADP as acceptor][GO:0006006:glucose metabolic process]	[ko00710:Carbon fixation in photosynthetic organisms]	MDP0000275412:Malus domestica|Trihelix|Trihelix family protein	XP_012464265.1:PREDICTED: glyceraldehyde3phosphate dehydrogenase B, chloroplastic [Gossypium raimondii] >KJB14201.1 hypothetical protein B456_002G114000 [Gossypium raimondii]	gi|512395725|gb|GAJZ01005445.1|:TSA: Prunus avium Pa_14655 transcribed RNA sequence	A0A0D2QBD6|A0A0D2QBD6_GOSRA:Glyceraldehyde-3-phosphate dehydrogenase;OS=Gossypium raimondii;|GO:0051287,GO:0050661,GO:0016620,|GO:0006006,

geneid	trend	GO	Pathway	TF	NR	NT	Uniprot
DN119776_c1_g6_i1	UP	[GO:0005524:ATP binding][GO:0004143:diacylglycerol kinase activity][GO:0007205:protein kinase C-activating G protein-coupled receptor signaling pathway]	[ko00561:Glycerolipid metabolism][ko00564:Glycerophospholipid metabolism][ko04070:Phosphatidylinositol signaling system][ko04072:Phospholipase D signaling pathway][ko05231:Choline metabolism in cancer]	-	XP_010689306.1:PREDICTED: diacylglycerol kinase 5 isoform X1 [Beta vulgaris subsp. vulgaris]	gi|521657779|gb|GAIA01038427.1|:TSA: Salicornia europaea Unigene43017_Se200S transcribed RNA sequence	A0A0J8EGW6|A0A0J8EGW6_BETVU: Diacylglycerol kinase;OS=Beta vulgaris subsp. vulgaris.;|GO:0005524,GO:0004143,|GO:0007205,
DN123384_c0_g2_i3	UP	[GO:0016021:integral component of membrane][GO:0016760:cellulose synthase (UDP-forming) activity][GO:0071555:cell wall organization][GO:0030244:cellulose biosynthetic process]	-	-	KNA17849.1:hypothetical protein SOVF_076680 [Spinacia oleracea]	-	A0A0K9RFU8|A0A0K9RFU8_SPIOL:Uncharacterized protein;OS=Spinacia oleracea;|GO:0016021,|GO:0016760,|GO:0071555,GO:0030244,
DN112577_c0_g1_i2	UP	-	-	-	-	-	-

geneid	trend	GO	Pathway	TF	NR	NT	Uniprot
DN124994_c1_g1_i2	UP	[GO:0005737:cytoplasm][GO:0042132:fructose 1,6-bisphosphate 1-phosphatase activity][GO:0030388:fructose 1,6-bisphosphate metabolic process][GO:0006002:fructose 6-phosphate metabolic process][GO:0006001:fructose catabolic process][GO:0006000:fructose metabolic process][GO:0006094:gluconeogenesis][GO:0005986:sucrose biosynthetic process]	[ko00010:Glycolysis / Gluconeogenesis][ko00030:Pentose phosphate pathway][ko00051:Fructose and mannose metabolism][ko00680:Methane metabolism][ko00710:Carbon fixation in photosynthetic organisms][ko04152:AMPK signaling pathway][ko04910:Insulin signaling pathway]	-	XP_021750548.1:fructose1,6bisphosphatase, chloroplastic [Chenopodium quinoa]	gi|521705780|gb|GAIA01015018.1|:TSA: Salicornia europaea Unigene15690_Se200S transcribed RNA sequence	A9PGB1|A9PGB1_POPTR:Chain D family protein;OS=Populus trichocarpa;|GO:0005737,|GO:0042132,|GO:0030388,GO:0006002,GO:0006001,GO:0006000,GO:0006094,GO:0005986,

geneid	trend	GO	Pathway	TF	NR	NT	Uniprot
DN122547_c0_g2_i1	UP	[GO:0020037:heme binding][GO:0005506:iron ion binding][GO:0004497:monooxygenase activity][GO:0016705:oxidoreductase activity, acting on paired donors, with incorporation or reduction of molecular oxygen]	-	Sme2.5_00297.1_g00019.1:Solanum melongena|MYB|MYB family protein	XP_004298682.1:PREDICTED: geraniol 8hydroxylaselike [Fragaria vesca subsp. vesca]	-	M5WU51|M5WU51_PRUPE:Uncharacterized protein;OS=Prunus persica;|GO:0020037,GO:0005506,GO:0004497,GO:0016705,
DN124366_c2_g1_i1	UP	-	-	-	-	-	-
DN127522_c1_g1_i3	UP	[GO:0003677:DNA binding][GO:0008080:N-acetyltransferase activity][GO:0008270:zinc ion binding]	-	Achn321981:Actinidia chinensis|Dof|Dof family protein	XP_010679053.1:PREDICTED: uncharacterized protein LOC104894503 [Beta vulgaris subsp. vulgaris] >XP_010679054.1 PREDICTED: uncharacterized protein LOC104894503 [Beta vulgaris subsp.	gi|521543072|gb|GAHZ01001894.1|:TSA: Salicornia europaea Unigene1944_SeCKS transcribed RNA sequence	A0A0J8C8Z3|A0A0J8C8Z3_BETVU:Uncharacterized protein;OS=Beta vulgaris subsp. vulgaris.;|GO:0003677,GO:0008080,GO:0008270,

geneid	trend	GO	Pathway	TF	NR	NT	Uniprot
					vulgaris] >KMT10175.1 hypothetical protein BVRB_5g119360 [Beta vulgaris subsp. vulgaris]		
DN121674_c1_g1_i3	UP	-	-	BGIOSGA035841-PA:Oryza sativa subsp. indica|MYB_related|MYB_related family protein	XP_021714432.1:BTB/POZ domaincontaining protein At5g67385like isoform X2 [Chenopodium quinoa]	gi|312282698|dbj|AK353129.1|:Thellungiella halophila mRNA, complete cds, clone: RTFL01-14-B17	A0A0J8CC97|A0A0J8CC97_BETVU:Uncharacterized protein;OS=Beta vulgaris subsp. vulgaris.;

geneid	trend	GO	Pathway	TF	NR	NT	Uniprot
DN122037_c2_g1_i3	UP	[GO:0009507:chloroplast][GO:0016021:integral component of membrane][GO:0004659:prenyltransferase activity][GO:0009772:photosynthetic electron transport in photosystem II][GO:0042372:phylloquinone biosynthetic process][GO:0010236:plastoquinone biosynthetic process]	[ko00130:Ubiquinone and other terpenoid-quinone biosynthesis]	-	XP_010690535.1:PREDICTED: 2carboxy1,4naphthoquinone phytyltransferase, chloroplastic [Beta vulgaris subsp. vulgaris] >KMT01547.1 hypothetical protein BVRB_9g215480 [Beta vulgaris subsp. vulgaris]	gi|521736686|gb|GAIA01008565.1|:TSA: Salicornia europaea Unigene8907_Se200S transcribed RNA sequence	A0A0J8BKM6|A0A0J8BKM6_BETVU: Uncharacterized protein;OS=Beta vulgaris subsp. vulgaris.;|GO:0009507,GO:0016021,|GO:0004659,|GO:0009772,GO:0042372,GO:0010236,
DN116124_c2_g18_i1	UP	[GO:0005576:extracellular region]	[ko04016:MAPK signaling pathway - plant][ko04075:Plant hormone signal transduction][ko04626:Plant-pathogen interaction]	-	XP_021776323.1:pathogenesisrelated protein PRB13like, partial [Chenopodium quinoa]	-	A0A0K9QY16|A0A0K9QY16_SPIOL:Uncharacterized protein;OS=Spinacia oleracea;|GO:0005576,

geneid	trend	GO	Pathway	TF	NR	NT	Uniprot
DN119512_c4_g1_i5	UP	[GO:0048046:apoplast][GO:0009941:chloroplast envelope][GO:0009570:chloroplast stroma][GO:0009535:chloroplast thylakoid membrane][GO:0010319:stromule][GO:0051287:NAD binding][GO:0050661:NADP binding][GO:0016620:oxidoreductase activity, acting on the aldehyde or oxo group of donors, NAD or NADP as acceptor][GO:0006006:glucose metabolic process]	[ko00710:Carbon fixation in photosynthetic organisms]	Achn306271:Actinidia chinensis|GRAS|GRAS family protein	XP_010686856.1:PREDICTED: glyceraldehyde3phosphate dehydrogenase B, chloroplastic [Beta vulgaris subsp. vulgaris] >KMT19244.1 hypothetical protein BVRB_1g014260 [Beta vulgaris subsp. vulgaris]	gi|521490462|gb|GAHZ01040424.1|:TSA: Salicornia europaea Unigene45791_SeCKS transcribed RNA sequence	A0A0J8D4K0|A0A0J8D4K0_BETVU:Glyceraldehyde-3-phosphate dehydrogenase;OS=Beta vulgaris subsp. vulgaris.;|GO:0048046,GO:0009941,GO:0009570,GO:0009535,GO:0010319,|GO:0051287,GO:0050661,GO:0016620,|GO:0006006,
DN128213_c1_g1_i11	UP	[GO:0005622:intracellular][GO:0016020:membrane][GO:0005524:ATP binding][GO:0017038:protein import][GO:0006605:protein targeting]	[ko02024:Quorum sensing][ko03060:Protein export][ko03070:Bacterial secretion system]	-	XP_021739734.1:protein translocase subunit SecA, chloroplasticlike [Chenopodium quinoa]	gi|939975|emb|Z49124.1|:S.oleracea chloroplast secA mRNA	V4TC12|V4TC12_9ROSI:Protein translocase subunit SecA;OS=Citrus clementina.;|GO:0005622,GO:0016020,|GO:0005524,|GO:0017038,GO:0006605,

geneid	trend	GO	Pathway	TF	NR	NT	Uniprot
DN127312_c4_g17_i2	UP	[GO:0005524:ATP binding]	-	GSBRNA2T00086041001:Brassica napus|ARF|ARF family protein	ABF38996.1:ribulose1,5bisphosphate carboxylase/oxygenase activase, partial [Pachysandra terminalis]	gi|21950711|gb|AY117142.1|:Chenopodium quinoa rubisco activase (rca) mRNA, complete cds	Q19RP2|Q19RP2_9MAGN:Ribulose-1,5-bisphosphate carboxylase/oxygenase se activase;OS=Pachysandra terminalis.;|GO:0005524,
DN121266_c0_g1_i1	UP	[GO:0005524:ATP binding][GO:0003676:nucleic acid binding]	-	-	XP_021862683.1:DEADbox ATPdependent RNA helicase 47, mitochondrial [Spinacia oleracea] >KNA16759.1 hypothetical protein SOVF_086240 [Spinacia oleracea]	gi|478159529|gb|GAHK01017074.1|:TSA: Jatropha curcas Unigene4528_JC-CK_1A transcribed RNA sequence	A0A0K9RCP2|A0A0K9RCP2_SPIOL:Uncharacterized protein;OS=Spinacia oleracea;|GO:0005524,GO:0003676,

geneid	trend	GO	Pathway	TF	NR	NT	Uniprot
DN128497_c4_g1_i7	UP	-	-	-	XP_021855853.1:uncharacterized protein LOC110795173 isoform X3 [Spinacia oleracea] >XP_021840277.1 uncharacterized protein LOC110780166 isoform X3 [Spinacia oleracea]	-	A0A0J8FT43|A0A0J8FT43_BETVU:Uncharacterized protein;OS=Beta vulgaris subsp. vulgaris.;
DN111711_c1_g1_i5	UP	-	-	-	XP_021769373.1:gibberellinregulated protein 1like [Chenopodium quinoa]	gi|521738633|gb|GAIA01007484.1|:TSA: Salicornia europaea Unigene7799_Se200S transcribed RNA sequence	A0A0J8BFU2|A0A0J8BFU2_BETVU:Uncharacterized protein;OS=Beta vulgaris subsp. vulgaris.;

geneid	trend	GO	Pathway	TF	NR	NT	Uniprot
DN124246_c2_g2_i3	UP	[GO:0016021:integral component of membrane]	-	-	XP_010673164.1:PREDICTED: uncharacterized protein LOC104889602 [Beta vulgaris subsp. vulgaris] >KMT20736.1 hypothetical protein BVRB_1g007090 [Beta vulgaris subsp. vulgaris]	-	A0A0J8FYN0|A0A0J8FYN0_BETVU:Uncharacterized protein;OS=Beta vulgaris subsp. vulgaris.;|GO:0016021,
DN122236_c3_g6_i8	UP	[GO:0005737:cytoplasm][GO:0042132:fructose 1,6-bisphosphate 1-phosphatase activity][GO:0046872:metal ion binding][GO:0006001:fructose catabolic process]	[ko00010:Glycolysis / Gluconeogenesis][ko00030:Pentose phosphate pathway][ko00051:Fructose and mannose metabolism][ko00680:Methane metabolism][ko00710:Carbon fixation in photosynthetic organisms][ko04152	-	BAF06820.1:Os01g0866400 [Oryza sativa Japonica Group] >BAS75386.1 Os01g0866400 [Oryza sativa Japonica Group]	gi|11139717|gb|AF317553.1|AF317553: Beta vulgaris cytosolic fructose-1,6-bisphosphatase mRNA, complete cds	A2WXB2|F16P2_ORYSI:Fructose-1,6-bisphosphatase, cytosolic;OS=Oryza sativa subsp. indica;|GO:0005737,|GO:0042132,GO:0046872,|GO:0006001,

geneid	trend	GO	Pathway	TF	NR	NT	Uniprot
			:AMPK signaling pathway][ko04910:Insulin signaling pathway]				
DN115589_c0_g1_i5	UP	-	-	-	XP_021776145.1:photosynthetic NDH subunit of subcomplex B 1, chloroplasticlike [Chenopodium quinoa]	gi|521541094|gb|GAHZ01002728.1|:TSA: Salicornia europaea Unigene2836_SeCKS transcribed RNA sequence	A0A0K9S0R8|A0A0K9S0R8_SPIOL:Uncharacterized protein;OS=Spinacia oleracea;
DN117689_c1_g1_i5	UP	[GO:0016021:integral component of membrane][GO:0016787:hydrolase activity][GO:0006629:lipid metabolic process]	-	-	XP_021768782.1:uncharacterized protein LOC110733070 [Chenopodium quinoa]	gi|521735544|gb|GAIA01009137.1|:TSA: Salicornia europaea Unigene9496_Se200S transcribed RNA sequence	A0A0J8C8K8|A0A0J8C8K8_BETVU:Uncharacterized protein;OS=Beta vulgaris subsp. vulgaris.;|GO:0016021,|GO:0016787,|G

geneid	trend	GO	Pathway	TF	NR	NT	Uniprot
							O:0006629,
DN119204_c1_g7_i2	UP	[GO:0016791:phosphatase activity][GO:0005975:carbohydrate metabolic process]	[ko00710:Carbon fixation in photosynthetic organisms]	-	XP_021769134.1:sedoheptulose1,7bisphosphatase, chloroplastic [Chenopodium quinoa]	gi|2529375|gb|L76556.1|SPIS17B:Spinacia oleracea chloroplast sedoheptulose-1,7-bisphosphatase mRNA, complete cds	A0A0J8D260|A0A0J8D260_BETVU:Uncharacterized protein;OS=Beta vulgaris subsp. vulgaris.;|GO:0016791,|GO:0005975,

geneid	trend	GO	Pathway	TF	NR	NT	Uniprot
DN114772_c0_g6_i2	UP	[GO:0070694:deoxyribonucleoside 5'-monophosphate N-glycosidase activity][GO:0043733:DNA-3-methyl base glycosylase activity][GO:0070635:nicotinamide riboside hydrolase activity][GO:0070636:nicotinic acid riboside hydrolase activity][GO:0000701:purine-specific mismatch base pair DNA N-glycosylase activity][GO:0017065:single-strand selective uracil DNA N-glycosylase activity][GO:0009691:cytokinin biosynthetic process]	-	Vang05g09490.1:Vigna angularis|WRKY|WRKY family protein	BAK52670.1:cytokinin riboside 5'monophosphate phosphoribohydrolase [Petunia x hybrida]	gi|521750654|gb|GAIA01000821.1|:TSA: Salicornia europaea Unigene837_Se200S transcribed RNA sequence	F8WLR9|F8WLR9_PETHY:Cytokinin riboside 5'-monophosphate phosphoribohydrolase;OS=Petunia hybrida;|GO:0070694,GO:0043733,GO:0070635,GO:0070636,GO:0000701,GO:0017065,|GO:0009691,
DN121341_c0_g1_i2	UP	[GO:0016021:integral component of membrane]	-	-	XP_021841495.1:radial spoke head 10 homolog Blike [Spinacia oleracea] >KNA24647.1 hypothetical protein	gi|297823304|ref|XM_002879489.1|:Arabidopsis lyrata subsp. lyrata hypothetical protein, mRNA	A0A0K9S0L4|A0A0K9S0L4_SPIOL:Uncharacterized protein;OS=Spinacia oleracea;|GO:0016021,

geneid	trend	GO	Pathway	TF	NR	NT	Uniprot
					SOVF_013740 [Spinacia oleracea]		
DN114988_c0_g1_i2	UP	-	-	-	XP_010693516.1:PREDICTED: uncharacterized protein LOC104906459 [Beta vulgaris subsp. vulgaris]	-	A0A0J8BGF5|A0A0J8BGF5_BETVU:Uncharacterized protein;OS=Beta vulgaris subsp. vulgaris.;
DN115459_c0_g4_i2	UP	[GO:0009941:chloroplast envelope][GO:0009570:chloroplast stroma][GO:0009535:chloroplast thylakoid membrane][GO:0003676:nucleic acid binding][GO:0000166:nucleotide binding]	[ko03013:RNA transport][ko03015:mRNA surveillance pathway][ko03018:RNA degradation]	CCG030364.1:Populus euphratica|NF-YA|NF-YA family protein	XP_015936795.1:30S ribosomal protein 2, chloroplastic [Arachis duranensis]	gi|255544261|ref|XM_002513147.1|:Ricinus communis ribonucleoprotein, chloroplast, putative, mRNA	A0A0J8CM43|A0A0J8CM43_BETVU: Uncharacterized protein;OS=Beta vulgaris subsp. vulgaris.;|GO:0009941,GO:0009570,GO:0009535,|GO:0003676,GO:0000166,

geneid	trend	GO	Pathway	TF	NR	NT	Uniprot
DN112815_c0_g1_i2	UP	-	-	-	XP_010667986.1:PREDICTED: 23 kDa jasmonateinduced protein [Beta vulgaris subsp. vulgaris] >KMS94988.1 hypothetical protein BVRB_013450 [Beta vulgaris subsp. vulgaris]	-	A0A0D3HL17|A0A0D3HL17_9ORYZ: Uncharacterized protein;OS=Oryza barthii.;
DN122023_c0_g3_i2	UP	[GO:0009976:tocopherol cyclase activity]	[ko00130:Ubiquinone and other terpenoid-quinone biosynthesis]	-	XP_021739186.1:tocopherol cyclase, chloroplasticlike [Chenopodium quinoa]	gi|359476043|ref|XM_002281388.2|:PREDICTED: Vitis vinifera tocopherol cyclase, chloroplastic-like (LOC100268095), mRNA	A0A0K9QFP0|A0A0K9QFP0_SPIOL:Uncharacterized protein;OS=Spinacia oleracea;|GO:0009976,
DN126919_c1_g1_i9	UP	-	-	-	XP_007154507.1:hypothetical protein PHAVU_003G124400g [Phaseolus vulgaris] >ESW26501.1	-	V7CC75|V7CC75_PHAVU:Uncharacterized protein;OS=Phaseolus vulgaris;

geneid	trend	GO	Pathway	TF	NR	NT	Uniprot
					hypothetical protein PHAVU_003G124400g [Phaseolus vulgaris]		
DN124622_c5_g1_i3	UP	-	-	GSMUA_Achr10P17130_001:Musa acuminata|bHLH|bHLH family protein	XP_012481672.1:PREDICTED: chaperone protein dnaJ 20, chloroplasticlike [Gossypium raimondii] >XP_016722421.1 PREDICTED: chaperone protein dnaJ 20, chloroplasticlike [Gossypium hirsutum] >KJB27726.1 hypothetical protein B456_005G028300 [Gossypium raimondii]	-	A0A0D2RDH2|A0A0D2RDH2_GOSRA:Uncharacterized protein;OS=Gossypium raimondii;

geneid	trend	GO	Pathway	TF	NR	NT	Uniprot
DN119311_c0_g1_i1	UP	[GO:0016758:transferase activity, transferring hexosyl groups][GO:0008152:metabolic process]	-	Tp57577_TGAC_v2_mRNA33215:Trifolium pratense|bHLH|bHLH family protein	XP_010686252.1:PREDICTED: UDPglycosyltransferase 86A1 [Beta vulgaris subsp. vulgaris] >KMT04460.1 hypothetical protein BVRB_8g181310 isoform B [Beta vulgaris subsp. vulgaris]	-	A0A0J8BSF8|A0A0J8BSF8_BETVU:Glycosyltransferase;OS=Beta vulgaris subsp. vulgaris.;|GO:0016758,|GO:0008152,
DN121618_c0_g2_i4	UP	[GO:0009941:chloroplast envelope][GO:0016491:oxidoreductase activity]	-	-	XP_021751179.1:15cisphytoene desaturase, chloroplastic/chromoplasticlike [Chenopodium quinoa]	gi|261264968|gb|GU048885.1|:Capsicum annuum clone BAC CaCM414G15, complete sequence	A0A0J8EL50|A0A0J8EL50_BETVU:Uncharacterized protein;OS=Beta vulgaris subsp. vulgaris.;|GO:0009941,|GO:0016491,
DN122687_c1_g1_i2	UP	[GO:0016021:integral component of membrane]	-	GSMUA_Achr1P00070_001:Musa acuminata|ERF|ERF family protein	OMO76482.1:hypothetical protein CCACVL1_15632 [Corchorus capsularis]	-	A0A067G8E6|A0A067G8E6_CITSI:Uncharacterized protein;OS=Citrus sinensis;|GO:0016021,

geneid	trend	GO	Pathway	TF	NR	NT	Uniprot
DN128129_c3_g9_i4	UP	-	-	-	KJB51172.1:hypothetical protein B456_008G204900 [Gossypium raimondii]	-	A0A0D2PYJ9|A0A0D2PYJ9_GOSRA:Uncharacterized protein;OS=Gossypium raimondii;
DN116782_c0_g1_i1	UP	[GO:0004842:ubiquitin-protein transferase activity]	-	Do007746.1:Dichanthelium oligosanthes|bHLH|bHLH family protein	XP_021719549.1:U box domaincontaining protein 8like [Chenopodium quinoa]	gi|521578137|gb|GAIA01069944.1|:TSA: Salicornia europaea Unigene84214_Se200S transcribed RNA sequence	W9SVD0|W9SVD0_9ROSA:U-box domain-containing protein 8;OS=Morus notabilis.;|GO:0004842,
DN115809_c2_g2_i4	UP	[GO:0005524:ATP binding][GO:0016887:ATPase activity]	-	Prupe.3G046700.1.p:Prunus persica|GeBP|GeBP family protein	XP_021642043.1:ABC transporter E family member 2 [Hevea brasiliensis] >XP_021642044.1 ABC transporter E family member 2 [Hevea brasiliensis] >XP_021642045.1 ABC transporter E family member 2 [Hevea brasiliensis]	gi|449503573|ref|XM_004162022.1|:PREDICTED: Cucumis sativus ABC transporter E family member 2-like (LOC101230133), mRNA	A0A068UHY8|A0A068UHY8_COFCA: Uncharacterized protein;OS=Coffea canephora;|GO:0005524,GO:0016887,

geneid	trend	GO	Pathway	TF	NR	NT	Uniprot
DN121386_c0_g1_i3	UP	[GO:0005509:calcium ion binding]	-	mrna23394.1-v1.0-hybrid:Fragaria vesca|NAC|NAC family protein	XP_010685415.1:PREDICTED: uncharacterized calciumbinding protein At1g02270like [Beta vulgaris subsp. vulgaris]	-	A0A0J8BY76|A0A0J8BY76_BETVU:Uncharacterized protein;OS=Beta vulgaris subsp. vulgaris.;|GO:0005509,
DN114503_c0_g1_i2	UP	[GO:0008168:methyltransferase activity]	-	-	XP_006470200.1:PREDICTED: uncharacterized protein LOC102631319 [Citrus sinensis] >KDO48995.1 hypothetical protein CISIN_1g023470mg [Citrus sinensis]	gi|512397344|gb|GAJZ01003826.1|:TSA: Prunus avium Pa_08681 transcribed RNA sequence	A0A067E540|A0A067E540_CITSI:Uncharacterized protein;OS=Citrus sinensis;|GO:0008168,
DN116539_c2_g2_i4	UP	[GO:0003676:nucleic acid binding]	-	XP_008245529.1:Prunus mume|E2F/DP|E2F/DP family protein	XP_010694802.1:PREDICTED: uncharacterized protein LOC104907559 [Beta vulgaris subsp. vulgaris]	-	F4NCI7|F4NCI7_BETVU:Uncharacterized protein;OS=Beta vulgaris subsp. vulgaris.;|GO:0003676,

geneid	trend	GO	Pathway	TF	NR	NT	Uniprot
DN123214_c4_g2_i9	UP	[GO:0046872:metal ion binding][GO:0004722:protein serine/threonine phosphatase activity]	[ko04010:MAPK signaling pathway]	-	XP_021774965.1:protein phosphatase 2C 57like isoform X1 [Chenopodium quinoa]	gi|3643089|gb|AF075582.1|AF075582:Mesembryanthemum crystallinum clone Mpc8 protein phosphatase-2C (PP2C) mRNA, complete cds	A0A0J8CV95|A0A0J8CV95_BETVU:Uncharacterized protein;OS=Beta vulgaris subsp. vulgaris.;|GO:0046872,GO:0004722,
DN120715_c3_g1_i2	UP	[GO:0009535:chloroplast thylakoid membrane][GO:0016021:integral component of membrane][GO:0009522:photosystem I][GO:0009523:photosystem II][GO:0016168:chlorophyll binding][GO:0046872:metal ion binding][GO:0009765:photosynthesis, light harvesting][GO:0018298:protein-chromophore linkage]	[ko00196:Photosynthesis - antenna proteins]	Bradi3g42627.3.p:Brachypodium distachyon|ERF|ERF family protein	XP_010694428.1:PREDICTED: chlorophyll ab binding protein 13, chloroplastic [Beta vulgaris subsp. vulgaris] >KMS98357.1 hypothetical protein BVRB_4g093140 [Beta vulgaris subsp. vulgaris]	gi|521544948|gb|GAHZ01001137.1|:TSA: Salicornia europaea Unigene1162_SeCKS transcribed RNA sequence	A0A0J8BEG8|A0A0J8BEG8_BETVU:Chlorophyll a-b binding protein, chloroplastic;OS=Beta vulgaris subsp. vulgaris.;|GO:0009535,GO:0016021,GO:0009522,GO:0009523,|GO:0016168,GO:0046872,|GO:0009765,GO:0018298,

geneid	trend	GO	Pathway	TF	NR	NT	Uniprot
DN117602_c0_g1_i3	UP	[GO:0016021:integral component of membrane][GO:0005886:plasma membrane][GO:0022857:transmembrane transporter activity][GO:0006810:transport]	-	-	XP_002278619.2:PREDICTED: uncharacterized protein At4g15545 isoform X1 [Vitis vinifera] >CBI26144.3 unnamed protein product, partial [Vitis vinifera]	gi|357447558|ref|XM_003594007.1|:Medicago truncatula hypothetical protein (MTR_2g020820) mRNA, complete cds	D7T6M3|D7T6M3_VITVI:Putative uncharacterized protein;OS=Vitis vinifera;GN=VIT_05s0020g02240 {ECO:0000313|EMBL:CBI26144.3};|GO:0016021,GO:0005886,|GO:0022857,|GO:0006810,
DN127312_c4_g23_i1	UP	[GO:0005524:ATP binding]	-	GSBRNA2T00086041001:Brassica napus|ARF|ARF family protein	OIW02691.1:hypothetical protein TanjilG_29467 [Lupinus angustifolius]	gi|21950711|gb|AY117142.1|:Chenopodium quinoa rubisco activase (rca) mRNA, complete cds	V7BAA0|V7BAA0_PHAVU:Uncharacterized protein;OS=Phaseolus vulgaris;|GO:0005524,
DN127471_c0_g3_i6	UP	[GO:0006457:protein folding]	-	Achn036321:Actinidia chinensis|G2-like|G2-like family protein	XP_021645314.1:dnaJ homolog subfamily B member 13like [Hevea brasiliensis]	gi|521527302|gb|GAHZ01009736.1|:TSA: Salicornia europaea Unigene10274_SeCKS transcribed RNA sequence	A0A0J8BKJ1|A0A0J8BKJ1_BETVU:Uncharacterized protein;OS=Beta vulgaris subsp. vulgaris.;|GO:0006457,

geneid	trend	GO	Pathway	TF	NR	NT	Uniprot
DN115321_c0_g1_i2	UP	[GO:0016021:integral component of membrane]	-	-	XP_010695745.1:PREDICTED: uncharacterized protein LOC104908341 [Beta vulgaris subsp. vulgaris]	-	A0A0J8BBY1|A0A0J8BBY1_BETVU: Uncharacterized protein;OS=Beta vulgaris subsp. vulgaris.;|GO:0016021,
DN110363_c0_g1_i2	UP	-	-	-	-	-	-

geneid	trend	GO	Pathway	TF	NR	NT	Uniprot
DN123784_c4_g1_i4	UP	[GO:0004838:L-tyrosine:2-oxoglutarate aminotransferase activity][GO:0030170:pyridoxal phosphate binding][GO:0006520:cellular amino acid metabolic process][GO:0010189:vitamin E biosynthetic process]	[ko00130:Ubiquinone and other terpenoid-quinone biosynthesis][ko00270:Cysteine and methionine metabolism][ko00350:Tyrosine metabolism][ko00360:Phenylalanine metabolism][ko00400:Phenylalanine, tyrosine and tryptophan biosynthesis][ko00401:Novobiocin biosynthesis][ko00950:Isoquinoline alkaloid biosynthesis][ko00960:Tropane, piperidine and pyridine alkaloid biosynthesis]	-	XP_010675472.1:PREDICTED: probable aminotransferase TAT2 isoform X2 [Beta vulgaris subsp. vulgaris]	gi|27525395|emb|AJ458993.1|:Coleus blumei mRNA for tyrosine aminotransferase (tat gene)	A0A0J8CHC8|A0A0J8CHC8_BETVU: Uncharacterized protein;OS=Beta vulgaris subsp. vulgaris.;|GO:0004838,GO:0030170,|GO:0006520,GO:0010189,

geneid	trend	GO	Pathway	TF	NR	NT	Uniprot
DN107172_c0_g1_i1	UP	-	-	-	XP_021768755.1:calvin cycle protein CP122, chloroplasticlike [Chenopodium quinoa]	gi|1617212|emb|Z72487.1|:S.oleracea mRNA for CP12	A0A059AWT8|A0A059AWT8_EUCGR:Uncharacterized protein;OS=Eucalyptus grandis;
DN128396_c4_g13_i1	UP	-	-	Tp57577_TGAC_v2_mRNA39490:Trifolium pratense|NAC|NAC family protein	ACL81159.1:putative auxin/aluminumresponsive protein [Mirabilis jalapa]	gi|220702720|gb|FJ536225.1|:Mirabilis jalapa putative auxin/aluminum-responsive protein mRNA, complete cds	B8YIB1|B8YIB1_MIRJA:Putative auxin/aluminum-responsive protein;OS=Mirabilis jalapa;
DN121965_c1_g2_i3	UP	[GO:0005737:cytoplasm][GO:0005634:nucleus][GO:0010309:acireductone dioxygenase [iron(II)-requiring] activity][GO:0005506:iron ion binding][GO:0019509:L-methionine salvage from methylthioadenosine]	[ko00270:Cysteine and methionine metabolism]	LPERR03G04120.1:Leersia perrieri|HSF|HSF family protein	XP_021725170.1:1,2dihydroxy3keto5methylthiopentene dioxygenase 1like [Chenopodium quinoa]	-	A0A0H2UIA1|A0A0H2UIA1_SOLLC:1,2-dihydroxy-3-keto-5-methylthiopentene dioxygenase;OS=Solanum lycopersicum;|GO:0005737,GO:0005634,|GO:0010309,GO:0005506,|GO:0019509,

geneid	trend	GO	Pathway	TF	NR	NT	Uniprot
DN124320_c3_g1_i3	UP	-	-	-	XP_010688351.1:PREDICTED: uncharacterized protein LOC104902318 [Beta vulgaris subsp. vulgaris] >KMT03219.1 hypothetical protein BVRB_8g197670 [Beta vulgaris subsp. vulgaris]	gi|388495223|gb|BT135883.1|:Medicago truncatula clone JCVI-FLMt-1C22 unknown mRNA	A0A0J8BT98|A0A0J8BT98_BETVU:Uncharacterized protein;OS=Beta vulgaris subsp. vulgaris.;
DN129008_c1_g1_i10	UP	[GO:0004185:serine-type carboxypeptidase activity]	-	Sme2.5_00187.1_g00004.1:Solanum melongena|MYB_related|MYB_related family protein	XP_021751255.1:serine carboxypeptidaselike 1 isoform X1 [Chenopodium quinoa]	gi|225429052|ref|XM_002269403.1|:PREDICTED: Vitis vinifera serine carboxypeptidase-like 18-like (LOC100253211), mRNA	B9S819|B9S819_RICCO:Serine carboxypeptidase, putative;OS=Ricinus communis;|GO:0004185,

geneid	trend	GO	Pathway	TF	NR	NT	Uniprot
DN123296_c2_g1_i3	UP	[GO:0005634:nucleus][GO:0000977:RNA polymerase II regulatory region sequence-specific DNA binding][GO:0003700:DNA-binding transcription factor activity][GO:0000165:MAPK cascade][GO:0045944:positive regulation of transcription by RNA polymerase II][GO:0006351:transcription, DNA-templated]	-	Bv6_150530_jqqr.t3:Beta vulgaris|MIKC_MADS|MIKC_MADS family protein	ABC55432.1:FLClike 1 splice variant 4 [Beta vulgaris subsp. maritima]	-	A2CHK0|A2CHK0_BETVM:FLC-like 1 splice variant 4;OS=Beta vulgaris subsp. maritima;|GO:0005634,|GO:0000977,GO:0003700,|GO:0000165,GO:0045944,GO:0006351,
DN127098_c2_g1_i16	UP	[GO:0009507:chloroplast][GO:0005524:ATP binding][GO:0016301:kinase activity][GO:0046872:metal ion binding][GO:0050242:pyruvate, phosphate dikinase activity][GO:0015979:photosynthesis][GO:0006090:pyruvate metabolic process]	[ko00620:Pyruvate metabolism][ko00710:Carbon fixation in photosynthetic organisms][ko00720:Carbon fixation pathways in prokaryotes]	-	Q42910.1:RecName: Full=Pyruvate, phosphate dikinase, chloroplastic; AltName: Full=Pyruvate, orthophosphate dikinase; Flags: Precursor >CAA57872.1 pyruvate,orthophosp	gi|559470|emb|X78347.1|:M.crystallinum PPDK mRNA	Q42910|PPDK_MESCR:Pyruvate, phosphate dikinase, chloroplastic;OS=Mesembryanthemum crystallinum;|GO:0009507,|GO:0005524,GO:0016301,GO:0046872,GO:0050242,|GO:0015979,GO:0006090,

geneid	trend	GO	Pathway	TF	NR	NT	Uniprot
					hate dikinase [Mesembryanthemum crystallinum]		
DN121962_c0_g1_i3	UP	[GO:0016811:hydrolase activity, acting on carbon-nitrogen (but not peptide) bonds, in linear amides]	[ko00460:Cyanoamino acid metabolism][ko00630:Glyoxylate and dicarboxylate metabolism][ko00910:Nitrogen metabolism]	-	XP_021714439.1:uncharacterized protein LOC110682416 isoform X1 [Chenopodium quinoa]	gi|255568564|ref|XM_002525210.1|:Ricinus communis Acetamidase, putative, mRNA	A0A0K9QZ91|A0A0K9QZ91_SPIOL:Uncharacterized protein;OS=Spinacia oleracea;|GO:0016811,
DN120811_c1_g2_i4	UP	[GO:0016788:hydrolase activity, acting on ester bonds]	-	-	XP_010685239.1:PREDICTED: GDSL esterase/lipase 2like [Beta vulgaris subsp. vulgaris]	-	A0A0J8BXN2|A0A0J8BXN2_BETVU: Uncharacterized protein;OS=Beta vulgaris subsp. vulgaris.;|GO:00167

geneid	trend	GO	Pathway	TF	NR	NT	Uniprot
							88,
DN123234_c 2_g2_i1	UP	[GO:0016021:integral component of membrane]	-	-	XP_010675230.1:P REDICTED: ALAinteracting subunit 3 [Beta vulgaris subsp. vulgaris] >KMT13323.1 hypothetical protein BVRB_4g084660 [Beta vulgaris subsp. vulgaris]	gi|449445934|ref|X M_004140679.1|:PR EDICTED: Cucumis sativus ALA-interacting subunit 5-like (LOC101204455), mRNA	A0A0J8CMQ1|A0A 0J8CMQ1_BETVU: Uncharacterized protein;OS=Beta vulgaris subsp. vulgaris.;|GO:00160 21,
DN121899_c 6_g1_i1	UP	-	-	-	XP_021756053.1:un characterized protein LOC110721211 isoform X1 [Chenopodium quinoa] >XP_021756054.1 uncharacterized protein	-	A0A0K9QW77|A0A 0K9QW77_SPIOL: Uncharacterized protein;OS=Spinacia oleracea;

geneid	trend	GO	Pathway	TF	NR	NT	Uniprot
					LOC110721211 isoform X1 [Chenopodium quinoa]		
DN128513_c1_g3_i5	UP	[GO:0005886:plasma membrane]	-	-	XP_010671007.1:PREDICTED: uncharacterized protein LOC104887920 isoform X1 [Beta vulgaris subsp. vulgaris] >KMT16594.1 hypothetical protein BVRB_3g048820 isoform A [Beta vulgaris subsp. vulgaris]	gi|521443817|gb|GAHZ01076667.1|:TSA: Salicornia europaea Unigene93671_SeCKS transcribed RNA sequence	A0A0J8CWX7|A0A0J8CWX7_BETVU: Uncharacterized protein;OS=Beta vulgaris subsp. vulgaris.;|GO:0005886,

geneid	trend	GO	Pathway	TF	NR	NT	Uniprot
DN124103_c0_g2_i5	UP	[GO:0016021:integral component of membrane][GO:0005215:transporter activity]	-	-	XP_021866383.1:metal transporter Nramp6like isoform X2 [Spinacia oleracea] >KNA10318.1 hypothetical protein SOVF_145490 [Spinacia oleracea]	gi|82581290|dbj|AB242564.1|:Chengiopanax sciadophylloides CsNramp1 mRNA for NRAMP family metal transporter, complete cds	A0A0K9QUD1|A0A0K9QUD1_SPIOL: Uncharacterized protein;OS=Spinacia oleracea;|GO:0016021,|GO:0005215,
DN120958_c2_g3_i4	UP	-	-	Sp_170630_gkqf.t1: Spinacia oleracea|TCP|TCP family protein	XP_021756900.1:transcription factor TCP4like [Chenopodium quinoa]	gi|521739087|gb|GAIA01007256.1|:TSA: Salicornia europaea Unigene7569_Se200S transcribed RNA sequence	A0A0K9QJY5|A0A0K9QJY5_SPIOL:Uncharacterized protein;OS=Spinacia oleracea;
DN98620_c0_g3_i1	UP	-	[ko04141:Protein processing in endoplasmic reticulum]	WALNUT_00030998-RA:Juglans regia|NF-YB|NF-YB family protein	XP_021845659.1:17.8 kDa class I heat shock proteinlike [Spinacia oleracea] >KNA25886.1 hypothetical protein SOVF_002240 [Spinacia oleracea]	gi|521537153|gb|GAHZ01004980.1|:TSA: Salicornia europaea Unigene5269_SeCKS transcribed RNA sequence	A0A0K9S2A8|A0A0K9S2A8_SPIOL:Uncharacterized protein;OS=Spinacia oleracea;

geneid	trend	GO	Pathway	TF	NR	NT	Uniprot
DN117714_c2_g4_i1	UP	[GO:0005622:intracellular][GO:0008270:zinc ion binding]	-	Bv_010830_jrft.t1:Beta vulgaris|CO-like|CO-like family protein	XP_010667660.1:PREDICTED: zinc finger protein CONSTANSLIKE 16 [Beta vulgaris subsp. vulgaris] >KMS95225.1 hypothetical protein BVRB_010830 [Beta vulgaris subsp. vulgaris]	gi|460386479|ref|XM_004238878.1|:PREDICTED: Solanum lycopersicum zinc finger protein CONSTANS-LIKE 16-like (LOC101253781), mRNA	A0A0J8DWJ0|A0A0J8DWJ0_BETVU: Uncharacterized protein;OS=Beta vulgaris subsp. vulgaris.;|GO:0005622,|GO:0008270,
DN115659_c1_g2_i1	UP	-	-	-	KNA13221.1:hypothetical protein SOVF_118660 [Spinacia oleracea]	-	A0A0K9R124|A0A0K9R124_SPIOL:Uncharacterized protein;OS=Spinacia oleracea;
DN120025_c0_g5_i7	UP	-	-	-	-	-	-

geneid	trend	GO	Pathway	TF	NR	NT	Uniprot
DN113843_c0_g1_i1	UP	-	-	-	XP_010665890.1:PREDICTED: uncharacterized protein LOC104883135 [Beta vulgaris subsp. vulgaris] >KMS96479.1 hypothetical protein BVRB_9g224720 [Beta vulgaris subsp. vulgaris]	-	A0A0J8B5G6|A0A0J8B5G6_BETVU:Uncharacterized protein;OS=Beta vulgaris subsp. vulgaris.;
DN128396_c3_g1_i1	UP	-	-	-	-	-	-
DN117118_c5_g1_i4	UP	[GO:0016021:integral component of membrane]	-	-	XP_021864742.1:uncharacterized protein LOC110803531 isoform X2 [Spinacia oleracea] >KNA15458.1 hypothetical protein SOVF_098120 [Spinacia oleracea]	gi|521746926|gb|GAIA01002400.1|:TSA: Salicornia europaea Unigene2454_Se200S transcribed RNA sequence	A0A0K9R7E7|A0A0K9R7E7_SPIOL:Uncharacterized protein;OS=Spinacia oleracea;|GO:0016021,

geneid	trend	GO	Pathway	TF	NR	NT	Uniprot
DN124915_c0_g1_i3	UP	[GO:0009941:chloroplast envelope][GO:0009570:chloroplast stroma][GO:0005739:mitochondrion][GO:0005524:ATP binding][GO:0016485:protein processing][GO:0009408:response to heat]	[ko04213:Longevity regulating pathway - multiple species]	Achn116231:Actinidia chinensis|ARF|ARF family protein	XP_010665999.1:PREDICTED: chaperone protein ClpB4, mitochondrial [Beta vulgaris subsp. vulgaris] >KMT19033.1 hypothetical protein BVRB_2g031280 [Beta vulgaris subsp. vulgaris]	gi|356507344|ref|XM_003522380.1|:PREDICTED: Glycine max chaperone protein ClpB4, mitochondrial-like (LOC100782242), mRNA	A0A0J8D4G2|A0A0J8D4G2_BETVU:Uncharacterized protein;OS=Beta vulgaris subsp. vulgaris.;|GO:0009941,GO:0009570,GO:0005739,|GO:0005524,|GO:0016485,GO:0009408,
DN122236_c3_g6_i7	UP	[GO:0016021:integral component of membrane][GO:0042132:fructose 1,6-bisphosphate 1-phosphatase activity][GO:0006001:fructose catabolic process]	[ko00010:Glycolysis / Gluconeogenesis][ko00030:Pentose phosphate pathway][ko00051:Fructose and mannose metabolism][ko00680:Methane metabolism][ko00710:Carbon fixation in photosynthetic organisms][ko04152	-	XP_015886438.1:PREDICTED: fructose1,6bisphosphatase, cytosolic [Ziziphus jujuba]	gi|11139717|gb|AF317553.1|AF317553: Beta vulgaris cytosolic fructose-1,6-bisphosphatase mRNA, complete cds	A0A061FJ67|A0A061FJ67_THECC:Inositol monophosphatase family protein isoform 3;OS=Theobroma cacao;|GO:0016021,|GO:0042132,|GO:0006001,

geneid	trend	GO	Pathway	TF	NR	NT	Uniprot
			:AMPK signaling pathway][ko04910:Insulin signaling pathway]				
DN122801_c1_g1_i2	UP	[GO:0005634:nucleus][GO:0043565:sequence-specific DNA binding][GO:0003700:DNA-binding transcription factor activity]	-	HL.SW.v1.0.G043571.1:Humulus lupulus|HD-ZIP|HD-ZIP family protein	NP_001280933.1:homeoboxleucine zipper protein HAT22like [Malus domestica] >ADL36706.1 HD domain class transcription factor [Malus domestica]	gi|512397285|gb|GAJZ01003885.1|:TSA: Prunus avium Pa_08893 transcribed RNA sequence	D9ZJ03|D9ZJ03_MALDO:HD domain class transcription factor;OS=Malus domestica;|GO:0005634,|GO:0043565,GO:0003700,

geneid	trend	GO	Pathway	TF	NR	NT	Uniprot
DN116001_c0_g1_i3	UP	-	-	-	XP_010665791.1:PREDICTED: DAG protein, chloroplastic [Beta vulgaris subsp. vulgaris] >XP_010665792.1 PREDICTED: DAG protein, chloroplastic [Beta vulgaris subsp. vulgaris] >KMS96580.1 hypothetical protein BVRB_8g201860 [Beta vulgaris subsp. vulgaris]	gi|521737048|gb|GAIA01008382.1|:TSA: Salicornia europaea Unigene8715_Se200S transcribed RNA sequence	A0A0J8E0G2|A0A0J8E0G2_BETVU:Uncharacterized protein;OS=Beta vulgaris subsp. vulgaris.;
DN120025_c0_g1_i1	UP	-	-	-	-	-	-

geneid	trend	GO	Pathway	TF	NR	NT	Uniprot
DN125907_c2_g5_i4	UP	[GO:0016021:integral component of membrane][GO:0005886:plasma membrane][GO:0008515:sucrose transmembrane transporter activity]	-	mrna24941.1-v1.0-hybrid:Fragaria vesca|DBB|DBB family protein	XP_021775815.1:bidirectional sugar transporter SWEET12like [Chenopodium quinoa]	-	A0A0J8CBG8|A0A0J8CBG8_BETVU: Bidirectional sugar transporter SWEET;OS=Beta vulgaris subsp. vulgaris.;|GO:0016021,GO:0005886,|GO:0008515,
DN127312_c4_g16_i2	UP	[GO:0005524:ATP binding]	-	GSBRNA2T00086041001:Brassica napus|ARF|ARF family protein	XP_007141402.1:hypothetical protein PHAVU_008G192400g [Phaseolus vulgaris] >ESW13396.1 hypothetical protein PHAVU_008G192400g [Phaseolus vulgaris]	gi|170128|gb|J03610.1|SPIRBCO:Spinach rubisco activase mRNA, complete cds	V7BAA0|V7BAA0_PHAVU:Uncharacterized protein;OS=Phaseolus vulgaris;|GO:0005524,

geneid	trend	GO	Pathway	TF	NR	NT	Uniprot
DN119650_c5_g1_i2	UP	[GO:0016021:integral component of membrane][GO:0016747:transferase activity, transferring acyl groups other than amino-acyl groups][GO:0006633:fatty acid biosynthetic process]	[ko00062:Fatty acid elongation]	-	XP_021847630.1:3ketoacylCoA synthase 4like [Spinacia oleracea] >KNA21425.1 hypothetical protein SOVF_043400 [Spinacia oleracea]	gi|199580041|gb|AC189356.2|:Brassica rapa subsp. pekinensis clone KBrB043O20, complete sequence	A0A0K9RPG4|A0A0K9RPG4_SPIOL:3-ketoacyl-CoA synthase;OS=Spinacia oleracea;|GO:0016021,|GO:0016747,|GO:0006633,
DN114201_c1_g8_i7	UP	[GO:0016831:carboxy-lyase activity][GO:0030170:pyridoxal phosphate binding][GO:0006520:cellular amino acid metabolic process]	[ko00350:Tyrosine metabolism][ko00950:Isoquinoline alkaloid biosynthesis]	-	XP_021722165.1:tyrosine decarboxylase 1like [Chenopodium quinoa]	gi|521666069|gb|GAIA01034503.1|:TSA: Salicornia europaea Unigene38193_Se200S transcribed RNA sequence	A0A0K9RYU8|A0A0K9RYU8_SPIOL: Uncharacterized protein;OS=Spinacia oleracea;|GO:0016831,GO:0030170,|GO:0006520,
DN111474_c0_g1_i2	UP	[GO:0006633:fatty acid biosynthetic process]	-	XP_011401860.1:Auxenochlorella protothecoides|CPP|CPP family protein	XP_006438854.1:hypothetical protein CICLE_v10033025mg [Citrus clementina] >ESR52094.1 hypothetical protein CICLE_v10033025mg [Citrus clementina]	-	A0A067DF69|A0A067DF69_CITSI:Acyl carrier protein;OS=Citrus sinensis;|GO:0006633,

geneid	trend	GO	Pathway	TF	NR	NT	Uniprot
DN127670_c9_g2_i1	UP	-	-	-	-	-	-
DN112556_c1_g1_i2	UP	[GO:0008124:4-alpha-hydroxytetrahydrobiopterin dehydratase activity][GO:0006729:tetrahydrobiopterin biosynthetic process]	-	-	XP_012467113.1:PREDICTED: uncharacterized protein LOC105785549 [Gossypium raimondii] >KJB07919.1 hypothetical protein B456_001G052600 [Gossypium raimondii]	gi|521570517|gb|GAIA01073428.1|:TSA: Salicornia europaea Unigene89255_Se200S transcribed RNA sequence	A0A0D2PM31|A0A0D2PM31_GOSRA: Uncharacterized protein;OS=Gossypium raimondii;|GO:0008124,|GO:0006729,
DN121629_c0_g2_i6	UP	[GO:0005622:intracellular][GO:0008270:zinc ion binding]	-	AHYPO_013514-RA:Amaranthus hypochondriacus|DBB|DBB family protein	XP_021753490.1:Bbox zinc finger protein 19like isoform X2 [Chenopodium quinoa]	gi|521511361|gb|GAHZ01019559.1|:TSA: Salicornia europaea Unigene20526_SeCKS transcribed RNA sequence	A0A0J8BJT3|A0A0J8BJT3_BETVU:Uncharacterized protein;OS=Beta vulgaris subsp. vulgaris.;|GO:0005622,|GO:0008270,

geneid	trend	GO	Pathway	TF	NR	NT	Uniprot
DN122228_c 1_g2_i4	UP	[GO:0005525:GTP binding][GO:0003924:GTPase activity][GO:0003676:nucleic acid binding]	-	KZV46390.1:Dorco ceras hygrometricum|LBD |LBD family protein	XP_021740971.1:pu tative elongation factor TypAlike SVR3, chloroplastic [Chenopodium quinoa]	gi|162424767|gb|EU 289224.1|:Suaeda salsa tyrosine phosphorylated protein A (TypA) mRNA, complete cds	W9R045|W9R045_9 ROSA:GTP-binding protein TypA/BipA-like protein;OS=Morus notabilis.;|GO:00055 25,GO:0003924,GO: 0003676,
DN123480_c 2_g1_i1	UP	[GO:0005524:ATP binding]	[ko04530:Tight junction][ko04612:A ntigen processing and presentation]	FANhyb_rscf00000 233.1.g00002.1:Frag aria x ananassa|GATA|GA TA family protein	XP_021732896.1:he at shock 70 kDa protein 8like [Chenopodium quinoa]	gi|49034965|gb|AC1 45222.18|:Medicago truncatula clone mth2-29a15, complete sequence	A0A0J8B7G9|A0A0 J8B7G9_BETVU:U ncharacterized protein;OS=Beta vulgaris subsp. vulgaris.;|GO:00055 24,
DN111531_c 0_g1_i1	UP	[GO:0003755:peptidyl-prolyl cis-trans isomerase activity][GO:0006457:protein folding]	-	-	XP_010695608.1:P REDICTED: peptidylprolyl cistrans isomerase CYP262, chloroplastic [Beta vulgaris subsp. vulgaris] >KMS97537.1 hypothetical protein	-	A0A0J8BC72|A0A0 J8BC72_BETVU:U ncharacterized protein;OS=Beta vulgaris subsp. vulgaris.;|GO:00037 55,|GO:0006457,

geneid	trend	GO	Pathway	TF	NR	NT	Uniprot
					BVRB_5g125980 [Beta vulgaris subsp. vulgaris]		
DN120171_c0_g19_i1	UP	[GO:0009507:chloroplast][GO:0004497:monooxygenase activity][GO:0016984:ribulose-bisphosphate carboxylase activity][GO:0009853:photorespiration][GO:0019253:reductive pentose-phosphate cycle]	[ko00630:Glyoxylate and dicarboxylate metabolism][ko00710:Carbon fixation in photosynthetic organisms]	mrna16933.1-v1.0-hybrid:Fragaria vesca|M-type_MADS|M-type_MADS family protein	XP_021734008.1:ribulose bisphosphate carboxylase small chain 1, chloroplastic [Chenopodium quinoa]	gi|521739485|gb|GAIA01006960.1|:TSA: Salicornia europaea Unigene7271_Se200S transcribed RNA sequence	P16032|RBS1_MESCR:Ribulose bisphosphate carboxylase small chain 1, chloroplastic;OS=Mesembryanthemum crystallinum;|GO:0009507,|GO:0004497,GO:0016984,|GO:0009853,GO:0019253,
DN128618_c0_g1_i18	UP	-	-	-	-	-	-

geneid	trend	GO	Pathway	TF	NR	NT	Uniprot
DN117208_c0_g2_i2	UP	[GO:0048046:apoplast][GO:0009941:chloroplast envelope][GO:0016020:membrane][GO:0005777:peroxisome][GO:0009506:plasmodesma][GO:0010287:plastoglobule][GO:0005840:ribosome][GO:0010319:stromule][GO:0005773:vacuole][GO:0003824:catalytic activity][GO:0050662:coenzyme binding][GO:0010297:heteropolysaccharide binding][GO:0003723:RNA binding][GO:0009658:chloroplast organization][GO:0007623:circadian rhythm][GO:0005996:monosaccharide metabolic process][GO:0032544:plastid translation][GO:0045893:positive regulation of transcription, DNA-templated][GO:0045727:positive regulation of translation][GO:0006364:rRNA processing]	-	-	XP_010674555.1:PREDICTED: chloroplast stemloop binding protein of 41 kDa b, chloroplastic [Beta vulgaris subsp. vulgaris] >KMT14184.1 hypothetical protein BVRB_4g080010 [Beta vulgaris subsp. vulgaris]	gi|521650915|gb|GAIA01041588.1|:TSA: Salicornia europaea Unigene46723_Se200S transcribed RNA sequence	A0A0J8CQA7|A0A0J8CQA7_BETVU: Uncharacterized protein;OS=Beta vulgaris subsp. vulgaris.;|GO:0048046,GO:0009941,GO:0016020,GO:0005777,GO:0009506,GO:0010287,GO:0005840,GO:0010319,GO:0005773,|GO:0003824,GO:0050662,GO:0010297,GO:0003723,|GO:0009658,GO:0007623,GO:0005996,GO:0032544,GO:0045893,GO:0045727,GO:0006364,

geneid	trend	GO	Pathway	TF	NR	NT	Uniprot
DN120325_c1_g1_i1	UP	[GO:0046872:metal ion binding][GO:0016491:oxidoreductase activity]	-	Zpz_sc00083.1.g00290.1.am.mk:Zoysia pacifica|Dof|Dof family protein	KMT00547.1:hypothetical protein BVRB_9g218460 [Beta vulgaris subsp. vulgaris]	-	A0A0J8BHV9|A0A0J8BHV9_BETVU: Uncharacterized protein;OS=Beta vulgaris subsp. vulgaris.;|GO:0046872,GO:0016491,
DN123930_c4_g2_i6	UP	[GO:0008430:selenium binding]	-	-	KNA09141.1:hypothetical protein SOVF_156170 [Spinacia oleracea]	gi|408777394|gb|JX472277.1|:Punica granatum cultivar Hongyushizi selenium-binding protein (SBP) mRNA, complete cds	A0A0K9QRM2|A0A0K9QRM2_SPIOL:Uncharacterized protein;OS=Spinacia oleracea;|GO:0008430,
DN128916_c2_g9_i2	UP	-	-	-	XP_002524154.1:PREDICTED: uncharacterized protein LOC8274801 [Ricinus communis] >EEF38217.1 conserved hypothetical protein [Ricinus communis]	-	B9SDT5|B9SDT5_RICCO:Putative uncharacterized protein;OS=Ricinus communis;

geneid	trend	GO	Pathway	TF	NR	NT	Uniprot
DN127312_c4_g22_i1	UP	[GO:0005524:ATP binding]	-	-	OIW02691.1:hypothetical protein TanjilG_29467 [Lupinus angustifolius]	-	A0A0L9UWT4|A0A0L9UWT4_PHAAN:Uncharacterized protein;OS=Phaseolus angularis;|GO:0005524,
DN111350_c0_g2_i1	UP	-	-	-	XP_017605757.1:PREDICTED: uncharacterized protein LOC108452472 isoform X3 [Gossypium arboreum]	-	A0A067K1B1|A0A067K1B1_JATCU:Uncharacterized protein;OS=Jatropha curcas;
DN120615_c0_g1_i1	UP	-	-	-	XP_010677282.1:PREDICTED: pentatricopeptide repeatcontaining protein At5g46580, chloroplastic [Beta vulgaris subsp. vulgaris] >KMT11704.1 hypothetical protein	gi|460400716|ref|XM_004245831.1|:PREDICTED: Solanum lycopersicum pentatricopeptide repeat-containing protein At5g46580, chloroplastic-like (LOC101264145), mRNA	A0A0J8CHW8|A0A0J8CHW8_BETVU:Uncharacterized protein;OS=Beta vulgaris subsp. vulgaris.;

geneid	trend	GO	Pathway	TF	NR	NT	Uniprot
					BVRB_5g106410 [Beta vulgaris subsp. vulgaris]		
DN127312_c4_g7_i1	UP	-	-	-	-	-	-
DN121172_c4_g5_i3	UP	[GO:0016021:integral component of membrane][GO:0004252:serine-type endopeptidase activity]	-	-	XP_021847145.1:RHOMBOIDlike protein 9, chloroplastic [Spinacia oleracea]	gi|502180640|ref|XM_004516627.1|:PREDICTED: Cicer arietinum uncharacterized LOC101511229 (LOC101511229), mRNA	A0A0K9QMQ6|A0A0K9QMQ6_SPIOL:Rhomboid-like protein;OS=Spinacia oleracea;|GO:0016021,|GO:0004252,

geneid	trend	GO	Pathway	TF	NR	NT	Uniprot
DN125071_c0_g1_i5	UP	[GO:0008168:methyltransferase activity][GO:0006694:steroid biosynthetic process]	[ko00100:Steroid biosynthesis]	-	XP_021736207.1:cycloartenolC24methyltransferaselike [Chenopodium quinoa] >XP_021736215.1 cycloartenolC24methyltransferaselike [Chenopodium quinoa]	gi|359487306|ref|XM_003633516.1|:PREDICTED: Vitis vinifera cycloartenol-C-24-methyltransferase-like, transcript variant 2 (LOC100263590), mRNA	A0A0K9RAF8|A0A0K9RAF8_SPIOL: Methyltransferase;OS=Spinacia oleracea;|GO:0008168,|GO:0006694,
DN125857_c5_g4_i3	UP	-	-	-	-	-	-
DN127139_c0_g2_i1	UP	[GO:0016021:integral component of membrane][GO:0005524:ATP binding][GO:0042626:ATPase activity, coupled to transmembrane movement of substances]	[ko02010:ABC transporters][ko04976:Bile secretion][ko05206: MicroRNAs in cancer]	Zjn_sc00086.1.g02330.1.am.mk:Zoysia japonica|ERF|ERF family protein	XP_021755311.1:ABC transporter B family member 13like [Chenopodium quinoa]	gi|359492271|ref|XM_002279435.2|:PREDICTED: Vitis vinifera ABC transporter B family member 13-like (LOC100259227), mRNA	A5CAU4|A5CAU4_VITVI:Putative uncharacterized protein;OS=Vitis vinifera;|GO:0016021,|GO:0005524,GO:0042626,

geneid	trend	GO	Pathway	TF	NR	NT	Uniprot
DN115897_c0_g1_i1	UP	[GO:0009941:chloroplast envelope][GO:0009535:chloroplast thylakoid membrane][GO:0016021:integral component of membrane][GO:0009538:photosystem I reaction center][GO:0010287:plastoglobule][GO:0015979:photosynthesis]	[ko00195:Photosynthesis]	-	XP_010673930.1:PREDICTED: photosystem I reaction center subunit XI, chloroplastic [Beta vulgaris subsp. vulgaris] >KMT14756.1 hypothetical protein BVRB_4g075220 [Beta vulgaris subsp. vulgaris]	gi|298571793|gb|HM188598.1|:Silene dioica isolate SdE393-10-b PSAL protein gene, partial cds	A0A0J8FG06|A0A0J8FG06_BETVU:Uncharacterized protein;OS=Beta vulgaris subsp. vulgaris.;|GO:0009941,GO:0009535,GO:0016021,GO:0009538,GO:0010287,|GO:0015979,
DN126422_c3_g2_i5	UP	[GO:0016491:oxidoreductase activity]	[ko00960:Tropane, piperidine and pyridine alkaloid biosynthesis]	XP_010558920.1:Tarenaya hassleriana|C2H2|C2H2 family protein	XP_010685684.1:PREDICTED: tropinone reductase homolog At2g29290like isoform X1 [Beta vulgaris subsp. vulgaris]	gi|521480611|gb|GAHZ01050275.1|:TSA: Salicornia europaea Unigene59398_SeCKS transcribed RNA sequence	A0A0J8BYZ8|A0A0J8BYZ8_BETVU:Uncharacterized protein;OS=Beta vulgaris subsp. vulgaris.;|GO:0016491,

geneid	trend	GO	Pathway	TF	NR	NT	Uniprot
DN122135_c1_g1_i5	UP	[GO:0008270:zinc ion binding]	-	-	XP_010691604.1:PREDICTED: protein DA1related 2 isoform X1 [Beta vulgaris subsp. vulgaris] >KMT18412.1 hypothetical protein BVRB_2g025600 isoform A [Beta vulgaris subsp. vulgaris]	gi|225449359|ref|XM_002277586.1|:PREDICTED: Vitis vinifera zinc ion binding protein-like (LOC100256525), mRNA	A0A0J8D2I4|A0A0J8D2I4_BETVU:Uncharacterized protein;OS=Beta vulgaris subsp. vulgaris.;|GO:0008270,

geneid	trend	GO	Pathway	TF	NR	NT	Uniprot
DN119057_c0_g6_i21	UP	[GO:0016491:oxidoreductase activity][GO:0008270:zinc ion binding]	[ko00010:Glycolysis / Gluconeogenesis][ko00071:Fatty acid degradation][ko00350:Tyrosine metabolism][ko00625:Chloroalkane and chloroalkene degradation][ko00626:Naphthalene degradation][ko00680:Methane metabolism][ko00830:Retinol metabolism][ko00980:Metabolism of xenobiotics by cytochrome P450][ko00982:Drug metabolism - cytochrome P450][ko05204:Chemical carcinogenesis]	-	GAV83260.1:ADH_zinc_N domaincontaining protein/ADH_N domaincontaining protein [Cephalotus follicularis]	-	A0A151RDF6|A0A151RDF6_CAJCA: Alcohol dehydrogenase-like 2;OS=Cajanus cajan;|GO:0016491, GO:0008270,

geneid	trend	GO	Pathway	TF	NR	NT	Uniprot
DN125588_c4_g2_i5	UP	[GO:0016021:integral component of membrane]	[ko04130:SNARE interactions in vesicular transport]	-	XP_021849007.1:syntaxin71like [Spinacia oleracea]	gi|521561038|gb|GAIA01076367.1|:TSA: Salicornia europaea Unigene92457_Se200S transcribed RNA sequence	A0A0J8D7U9|A0A0J8D7U9_BETVU:Uncharacterized protein;OS=Beta vulgaris subsp. vulgaris.;|GO:0016021,
DN126349_c8_g1_i2	UP	[GO:0046872:metal ion binding]	-	SapurV1A.0420s0040.1.p:Salix purpurea|C2H2|C2H2 family protein	XP_021616326.1:uncharacterized protein LOC110617680 [Manihot esculenta] >OAY47905.1 hypothetical protein MANES_06G115000 [Manihot esculenta]	-	A0A067K421|A0A067K421_JATCU:Uncharacterized protein;OS=Jatropha curcas;|GO:0046872,
DN122612_c1_g1_i4	UP	[GO:0008234:cysteine-type peptidase activity]	[ko04142:Lysosome][ko04210:Apoptosis]	-	XP_015876013.1:PREDICTED: cysteine proteinase 15Alike [Ziziphus jujuba] >XP_015884930.1 PREDICTED: cysteine proteinase 15Alike [Ziziphus	-	T2DN49|T2DN49_PHAVU:Cysteine proteinase RD19a-like protein;OS=Phaseolus vulgaris;|GO:0008234,

geneid	trend	GO	Pathway	TF	NR	NT	Uniprot
					jujuba]		
DN120377_c1_g1_i1	UP	-	-	-	-	-	-
DN123959_c2_g1_i5	UP	[GO:0016021:integral component of membrane][GO:0005215:transporter activity]	-	mrna00620.1-v1.0-hybrid:Fragaria vesca|BBR-BPC|BBR-BPC family protein	XP_010667212.1:PREDICTED: protein NRT1/ PTR FAMILY 2.7like [Beta vulgaris subsp. vulgaris]	-	A0A0J8B351|A0A0J8B351_BETVU:Uncharacterized protein;OS=Beta vulgaris subsp. vulgaris.;|GO:0016021,|GO:0005215,
DN127139_c0_g2_i2	UP	[GO:0016021:integral component of membrane][GO:0005524:ATP binding][GO:0042626:ATPase activity, coupled to transmembrane movement of substances]	[ko02010:ABC transporters][ko04976:Bile secretion][ko05206:MicroRNAs in cancer]	Zjn_sc00086.1.g02330.1.am.mk:Zoysia japonica|ERF|ERF family protein	XP_021755948.1:ABC transporter B family member 13like [Chenopodium quinoa]	gi|359492271|ref|XM_002279435.2|:PREDICTED: Vitis vinifera ABC transporter B family member 13-like (LOC100259227), mRNA	A0A0J8E9K1|A0A0J8E9K1_BETVU:Uncharacterized protein;OS=Beta vulgaris subsp. vulgaris.;|GO:0016021,|GO:0005524,GO:0042626,

geneid	trend	GO	Pathway	TF	NR	NT	Uniprot
DN117139_c3_g2_i1	UP	[GO:0016021:integral component of membrane]	-	-	XP_021852645.1:uncharacterized protein LOC110792138 [Spinacia oleracea]	gi|521674768|gb|GAIA01030097.1|:TSA: Salicornia europaea Unigene33009_Se200S transcribed RNA sequence	A0A0K9RAB5|A0A0K9RAB5_SPIOL: Uncharacterized protein;OS=Spinacia oleracea;|GO:0016021,
DN119031_c8_g4_i2	UP	-	[ko00480:Glutathione metabolism][ko00980:Metabolism of xenobiotics by cytochrome P450][ko00982:Drug metabolism - cytochrome P450][ko01524:Platinum drug resistance][ko05204:Chemical carcinogenesis][ko05418:Fluid shear stress and atherosclerosis]	-	XP_021769783.1:probable glutathione Stransferase parC [Chenopodium quinoa]	-	M5X2D7|M5X2D7_PRUPE:Uncharacterized protein;OS=Prunus persica;

geneid	trend	GO	Pathway	TF	NR	NT	Uniprot
DN126039_c2_g5_i2	UP	[GO:0003676:nucleic acid binding]	-	XP_008245529.1:Prunus mume|E2F/DP|E2F/DP family protein	XP_010694802.1:PREDICTED: uncharacterized protein LOC104907559 [Beta vulgaris subsp. vulgaris]	-	F4NCI4|F4NCI4_BETVU:Uncharacterized protein;OS=Beta vulgaris subsp. vulgaris.;|GO:0003676,
DN110068_c1_g5_i1	UP	-	-	-	-	gi|384096338|gb|JQ347294.1|:Asparagus adscendens antiviral protein mRNA, partial cds	-
DN114166_c2_g8_i1	UP	[GO:0016021:integral component of membrane]	-	-	XP_010693808.1:PREDICTED: uncharacterized protein LOC104906713 isoform X1 [Beta vulgaris subsp. vulgaris] >KMS98760.1 hypothetical protein BVRB_3g068390 isoform A [Beta vulgaris subsp.	gi|521676338|gb|GAIA01029190.1|:TSA: Salicornia europaea Unigene32013_Se200S transcribed RNA sequence	A0A0J8E6D8|A0A0J8E6D8_BETVU:Uncharacterized protein;OS=Beta vulgaris subsp. vulgaris.;|GO:0016021,

geneid	trend	GO	Pathway	TF	NR	NT	Uniprot
					vulgaris]		
DN125313_c0_g5_i3	UP	[GO:0004802:transketolase activity][GO:0008152:metabolic process]	[ko00030:Pentose phosphate pathway][ko00710:Carbon fixation in photosynthetic organisms][ko01051:Biosynthesis of ansamycins]	-	XP_010671991.1:PREDICTED: transketolase, chloroplastic [Beta vulgaris subsp. vulgaris] >KMT15886.1 hypothetical protein BVRB_3g055740 [Beta vulgaris subsp. vulgaris]	gi|2529341|gb|L76554.1|SPITRAN:Spinacia oleracea transketolase mRNA, chloroplast gene encoding chloroplast protein, complete cds	A0A0J8FJ23|A0A0J8FJ23_BETVU:Uncharacterized protein;OS=Beta vulgaris subsp. vulgaris.;|GO:0004802,|GO:0008152,
DN127036_c2_g1_i1	UP	-	-	-	-	-	-

geneid	trend	GO	Pathway	TF	NR	NT	Uniprot
DN124934_c2_g1_i1	UP	[GO:0005509:calcium ion binding]	-	676720782:Sisymbrium irio|FAR1|FAR1 family protein	XP_010692271.1:PREDICTED: probable calciumbinding protein CML23 [Beta vulgaris subsp. vulgaris]	gi|521611814|gb|GAIA01059001.1|:TSA: Salicornia europaea Unigene70085_Se200S transcribed RNA sequence	A0A0J8EAP3|A0A0J8EAP3_BETVU:Uncharacterized protein;OS=Beta vulgaris subsp. vulgaris.;|GO:0005509,
DN109859_c0_g1_i1	UP	[GO:0050660:flavin adenine dinucleotide binding][GO:0016614:oxidoreductase activity, acting on CH-OH group of donors]	-	-	XP_011005332.1:PREDICTED: reticuline oxidaselike protein [Populus euphratica]	-	B9GK10|B9GK10_POPTR:Uncharacterized protein;OS=Populus trichocarpa;|GO:0050660,GO:0016614,
DN119410_c1_g3_i1	UP	[GO:0004252:serine-type endopeptidase activity]	-	Aco013131.1:Ananas comosus|DBB|DBB family protein	XP_010685295.1:PREDICTED: subtilisinlike protease SBT1.4 [Beta vulgaris subsp. vulgaris]	-	A0A0J8EP70|A0A0J8EP70_BETVU:Uncharacterized protein;OS=Beta vulgaris subsp. vulgaris.;|GO:0004252,
DN116934_c0_g1_i7	UP	-	-	-	XP_021659366.1:uncharacterized protein LOC110649198 [Hevea brasiliensis]	gi|242385106|emb|FP093158.1|:Phyllostachys edulis cDNA clone: bphyst007n24, full	A0A067K1B2|A0A067K1B2_JATCU:Uncharacterized protein;OS=Jatropha curcas;

geneid	trend	GO	Pathway	TF	NR	NT	Uniprot
						insert sequence	
DN120332_c 2_g2_i2	UP	-	-	-	-	-	-
DN127616_c 4_g1_i10	UP	-	[ko04137:Mitophag y - animal][ko04217:Ne croptosis][ko04668: TNF signaling pathway]	-	XP_019106672.1:P REDICTED: probable 2carboxyDarabinitol 1phosphatase [Beta vulgaris subsp. vulgaris] >KMT04244.1 hypothetical protein BVRB_8g183200 [Beta vulgaris subsp. vulgaris]	gi|521546130|gb|GA HZ01000580.1|:TSA : Salicornia europaea Unigene594_SeCKS transcribed RNA sequence	A0A0J8BW62|A0A 0J8BW62_BETVU: Uncharacterized protein;OS=Beta vulgaris subsp. vulgaris.;
DN128383_c 2_g2_i1	UP	[GO:0016021:integral component of membrane][GO:0005215:transporter activity]	-	-	XP_021743644.1:pu rine permease 3like [Chenopodium quinoa]	-	A0A0J8D5V9|A0A0 J8D5V9_BETVU:U ncharacterized protein;OS=Beta vulgaris subsp. vulgaris.;|GO:00160 21,|GO:0005215,

geneid	trend	GO	Pathway	TF	NR	NT	Uniprot
DN121979_c5_g5_i3	UP	[GO:0016787:hydrolase activity][GO:0006629:lipid metabolic process]	-	-	XP_021729014.1:phospholipase A1Igamma3, chloroplasticlike isoform X3 [Chenopodium quinoa]	-	A0A0K9RV54|A0A0K9RV54_SPIOL:Uncharacterized protein;OS=Spinacia oleracea;|GO:0016787,|GO:0006629,
DN128963_c1_g1_i5	UP	[GO:0016021:integral component of membrane][GO:0036459:thiol-dependent ubiquitinyl hydrolase activity][GO:0016579:protein deubiquitination][GO:0006511:ubiquitin-dependent protein catabolic process]	[ko04137:Mitophagy - animal]	-	XP_010680306.1:PREDICTED: ubiquitin carboxylterminal hydrolase 27 isoform X1 [Beta vulgaris subsp. vulgaris]	gi|521473725|gb|GAHZ01057160.1|:TSA: Salicornia europaea Unigene68284_SeCKS transcribed RNA sequence	A0A0J8CAK0|A0A0J8CAK0_BETVU: Uncharacterized protein;OS=Beta vulgaris subsp. vulgaris.;|GO:0016021,|GO:0036459,|GO:0016579,GO:0006511,
DN115459_c0_g4_i5	UP	[GO:0000166:nucleotide binding][GO:0003723:RNA binding]	[ko03013:RNA transport][ko03015: mRNA surveillance pathway][ko03018: RNA degradation]	CCG030364.1:Populus euphratica|NF-YA|NF-YA family protein	XP_021674266.1:30S ribosomal protein 2, chloroplastic [Hevea brasiliensis]	gi|7578880|gb|AF240462.1|:Spinacia oleracea plastid-specific ribosomal protein 2 precursor (Psrp-2) mRNA, complete cds; nuclear gene for chloroplast product	A0A140TAN0|A0A140TAN0_SOYBN: Uncharacterized protein;OS=Glycine max;|GO:0000166,GO:0003723,

geneid	trend	GO	Pathway	TF	NR	NT	Uniprot
DN128066_c1_g1_i18	UP	[GO:0070204:2-succinyl-5-enolpyruvyl-6-hydroxy-3-cyclohexene-1-carboxylic-acid synthase activity][GO:0070205:2-succinyl-6-hydroxy-2,4-cyclohexadiene-1-carboxylate synthase activity][GO:0030976:thiamine pyrophosphate binding][GO:0009063:cellular amino acid catabolic process][GO:0009234:menaquinone biosynthetic process][GO:0042550:photosystem I stabilization][GO:0042372:phylloquinone biosynthetic process]	[ko00130:Ubiquinone and other terpenoid-quinone biosynthesis]	-	XP_021852080.1:protein PHYLLO, chloroplastic isoform X1 [Spinacia oleracea]	gi|359473492|ref|XM_002268725.2|:PREDICTED: Vitis vinifera protein PHYLLO, chloroplastic-like (LOC100241089), mRNA	A0A0J8C8Z6|A0A0J8C8Z6_BETVU:Uncharacterized protein;OS=Beta vulgaris subsp. vulgaris.;|GO:0070204,GO:0070205,GO:0030976,|GO:0009063,GO:0009234,GO:0042550,GO:0042372,

geneid	trend	GO	Pathway	TF	NR	NT	Uniprot
DN125524_c0_g2_i3	UP	[GO:0005737:cytoplasm][GO:0007015:actin filament organization][GO:0071483:cellular response to blue light][GO:0009904:chloroplast accumulation movement][GO:0009903:chloroplast avoidance movement]	-	Lus10012302:Linum usitatissimum|HB-other|HB-other family protein	XP_010694520.1:PREDICTED: J domaincontaining protein required for chloroplast accumulation response 1 isoform X1 [Beta vulgaris subsp. vulgaris] >KMS98314.1 hypothetical protein BVRB_4g093810 isoform B [Beta vulgaris subsp. vulgaris]	-	A0A0J8BAD0|A0A0J8BAD0_BETVU: Uncharacterized protein;OS=Beta vulgaris subsp. vulgaris.;|GO:0005737,|GO:0007015,GO:0071483,GO:0009904,GO:0009903,
DN116124_c2_g30_i1	UP	[GO:0005576:extracellular region]	[ko04016:MAPK signaling pathway - plant][ko04075:Plant hormone signal transduction][ko04626:Plant-pathogen interaction]	PSME_00037622-RA:Pseudotsuga menziesii|HD-ZIP|HD-ZIP family protein	XP_021776323.1:pathogenesisrelated protein PRB13like, partial [Chenopodium quinoa]	-	U5HUJ0|U5HUJ0_9CARY:Pathogenesis-related protein 1a;OS=Salsola ferganica.;|GO:0005576,

geneid	trend	GO	Pathway	TF	NR	NT	Uniprot
DN121629_c0_g2_i9	UP	[GO:0005622:intracellular][GO:0008270:zinc ion binding]	-	Bv9_216430_rwmw.t1:Beta vulgaris|DBB|DBB family protein	XP_021753490.1:Bbox zinc finger protein 19like isoform X2 [Chenopodium quinoa]	-	A0A0J8BJT3|A0A0J8BJT3_BETVU:Uncharacterized protein;OS=Beta vulgaris subsp. vulgaris.;|GO:0005622,|GO:0008270,
DN120598_c0_g1_i1	UP	[GO:0005768:endosome][GO:0016021:integral component of membrane][GO:0005802:trans-Golgi network]	-	-	XP_010667197.1:PREDICTED: myblike protein X [Beta vulgaris subsp. vulgaris] >XP_010667198.1 PREDICTED: myblike protein X [Beta vulgaris subsp. vulgaris] >KMS95548.1 hypothetical protein BVRB_007320 [Beta vulgaris subsp. vulgaris]	-	A0A0J8B6L8|A0A0J8B6L8_BETVU:Uncharacterized protein;OS=Beta vulgaris subsp. vulgaris.;|GO:0005768,GO:0016021,GO:0005802,

geneid	trend	GO	Pathway	TF	NR	NT	Uniprot
DN115311_c2_g1_i1	UP	[GO:0009570:chloroplast stroma][GO:0009535:chloroplast thylakoid membrane]	-	-	XP_010683679.1:PREDICTED: uncharacterized protein LOC104898322 [Beta vulgaris subsp. vulgaris] >KMT06745.1 hypothetical protein BVRB_7g159060 [Beta vulgaris subsp. vulgaris]	-	A0A0J8BZ10|A0A0J8BZ10_BETVU:Uncharacterized protein;OS=Beta vulgaris subsp. vulgaris.;|GO:0009570,GO:0009535,

geneid	trend	GO	Pathway	TF	NR	NT	Uniprot
DN123468_c5_g1_i1	UP	[GO:0019898:extrinsic component of membrane][GO:0009654:photosystem II oxygen evolving complex][GO:0005509:calcium ion binding][GO:0015979:photosynthesis]	[ko00195:Photosynthesis]	Pp3c2_23590V3.6.p:Physcomitrella patens|WRKY|WRKY family protein	XP_017648883.1:PREDICTED: oxygenevolving enhancer protein 2, chloroplasticlike isoform X1 [Gossypium arboreum] >XP_017648884.1 PREDICTED: oxygenevolving enhancer protein 2, chloroplasticlike isoform X2 [Gossypium arboreum] >KHG20721.1 Oxygenevolving enhancer 23, chloroplastic [Gossypium arboreum]	gi|356567469|ref|XM_003551894.1|:PREDICTED: Glycine max oxygen-evolving enhancer protein 2, chloroplastic-like (LOC100781848), mRNA	A0A0B0PBN3|A0A0B0PBN3_GOSAR: Oxygen-evolving enhancer 2-3, chloroplastic;OS=Gossypium arboreum;|GO:0019898,GO:0009654,|GO:0005509,|GO:0015979,

geneid	trend	GO	Pathway	TF	NR	NT	Uniprot
DN124590_c4_g5_i1	UP	[GO:0046872:metal ion binding][GO:0000166:nucleotide binding][GO:0003723:RNA binding]	-	Cotton_A_01797_BGI-A2_v1.0:Gossypium arboreum|C3H|C3H family protein	XP_021772120.1:zinc finger CCCH domaincontaining protein 53like [Chenopodium quinoa]	gi|512394734|gb|GAJZ01006436.1|:TSA: Prunus avium Pa_21648 transcribed RNA sequence	A0A0J8B4Z4|A0A0J8B4Z4_BETVU:Uncharacterized protein;OS=Beta vulgaris subsp. vulgaris.;|GO:0046872,GO:0000166,GO:0003723,
DN122646_c6_g17_i2	UP	[GO:0016021:integral component of membrane]	-	-	XP_021856040.1:uncharacterized protein LOC110795341 [Spinacia oleracea] >KNA06511.1 hypothetical protein SOVF_180400 [Spinacia oleracea]	gi|521454770|gb|GAHZ01065714.1|:TSA: Salicornia europaea Unigene79312_SeCKS transcribed RNA sequence	A0A0K9QIU1|A0A0K9QIU1_SPIOL:Uncharacterized protein;OS=Spinacia oleracea;|GO:0016021,
DN124541_c1_g1_i9	UP	[GO:0004176:ATP-dependent peptidase activity]	-	-	XP_012839125.1:PREDICTED: LON peptidase Nterminal domain and RING finger protein 1like [Erythranthe guttata] >EYU36744.1 hypothetical protein MIMGU_mgv1a011	-	A0A022R9Q1|A0A022R9Q1_ERYGU:Uncharacterized protein;OS=Erythranthe guttata;|GO:0004176,

geneid	trend	GO	Pathway	TF	NR	NT	Uniprot
					356mg [Erythranthe guttata]		
DN116124_c2_g3_i1	UP	-	-	-	-	-	-
DN123214_c4_g2_i5	UP	[GO:0004722:protein serine/threonine phosphatase activity]	[ko04010:MAPK signaling pathway]	-	AAC36700.1:protein phosphatase2C [Mesembryanthemum crystallinum]	gi|3643089|gb|AF075582.1|AF075582:Mesembryanthemum crystallinum clone Mpc8 protein phosphatase-2C (PP2C) mRNA, complete cds	O82471|O82471_MESCR:Protein phosphatase-2C;OS=Mesembryanthemum crystallinum;|GO:0004722,

geneid	trend	GO	Pathway	TF	NR	NT	Uniprot
DN127419_c2_g1_i5	UP	-	-	-	XP_010680254.1:PREDICTED: uncharacterized protein LOC104895442 [Beta vulgaris subsp. vulgaris] >KMT09198.1 hypothetical protein BVRB_6g133000 [Beta vulgaris subsp. vulgaris]	-	A0A0J8CB07|A0A0J8CB07_BETVU:Uncharacterized protein;OS=Beta vulgaris subsp. vulgaris.;
DN127098_c2_g1_i13	UP	[GO:0009507:chloroplast][GO:0005524:ATP binding][GO:0016301:kinase activity][GO:0046872:metal ion binding][GO:0050242:pyruvate, phosphate dikinase activity][GO:0015979:photosynthesis][GO:0006090:pyruvate metabolic process]	[ko00620:Pyruvate metabolism][ko00710:Carbon fixation in photosynthetic organisms][ko00720:Carbon fixation pathways in prokaryotes]	-	Q42910.1:RecName: Full=Pyruvate, phosphate dikinase, chloroplastic; AltName: Full=Pyruvate, orthophosphate dikinase; Flags: Precursor >CAA57872.1 pyruvate,orthophosphate dikinase [Mesembryanthemu	gi|559470|emb|X78347.1|:M.crystallinum PPDK mRNA	Q42910|PPDK_MESCR:Pyruvate, phosphate dikinase, chloroplastic;OS=Mesembryanthemum crystallinum;|GO:0009507,|GO:0005524,GO:0016301,GO:0046872,GO:0050242,|GO:0015979,GO:0006090,

geneid	trend	GO	Pathway	TF	NR	NT	Uniprot
					m crystallinum]		
DN120171_c0_g5_i1	UP	[GO:0009507:chloroplast][GO:0004497:monooxygenase activity][GO:0016984:ribulose-bisphosphate carboxylase activity][GO:0009853:photorespiration][GO:0019253:reductive pentose-phosphate cycle]	[ko00630:Glyoxylate and dicarboxylate metabolism][ko00710:Carbon fixation in photosynthetic organisms]	mrna16933.1-v1.0-hybrid:Fragaria vesca|M-type_MADS|M-type_MADS family protein	XP_021769982.1:ribulose bisphosphate carboxylase small chain 3, chloroplasticlike [Chenopodium quinoa]	gi|521739471|gb|GAIA01006967.1|:TSA: Salicornia europaea Unigene7278_Se200S transcribed RNA sequence	P32764|RBS3_SOLTU:Ribulose bisphosphate carboxylase small chain 3, chloroplastic;OS=Solanum tuberosum;|GO:0009507,|GO:0004497,GO:0016984,|GO:0009853,GO:0019253,
DN126800_c4_g5_i2	UP	-	-	-	-	-	-

geneid	trend	GO	Pathway	TF	NR	NT	Uniprot
DN127270_c4_g5_i1	UP	-	[ko00901:Indole alkaloid biosynthesis]	Zpz_sc00695.1.g00020.1.am.mk:Zoysia pacifica|C2H2|C2H2 family protein	XP_021723084.1:salicylic acidbinding protein 2like [Chenopodium quinoa]	-	V4SEQ8|V4SEQ8_9ROSI:Uncharacterized protein;OS=Citrus clementina.;
DN121594_c1_g4_i1	UP	-	-	-	XP_010671826.1:PREDICTED: uncharacterized protein LOC104888516 [Beta vulgaris subsp. vulgaris] >XP_010671827.1 PREDICTED: uncharacterized protein LOC104888516 [Beta vulgaris subsp. vulgaris] >KMT16348.1 hypothetical protein BVRB_3g054950 [Beta vulgaris subsp. vulgaris]	gi|255587049|ref|XM_002534068.1|:Ricinus communis conserved hypothetical protein, mRNA	A0A0J8CWL6|A0A0J8CWL6_BETVU: Uncharacterized protein;OS=Beta vulgaris subsp. vulgaris.;

geneid	trend	GO	Pathway	TF	NR	NT	Uniprot
DN119000_c2_g2_i4	UP	[GO:0016810:hydrolase activity, acting on carbon-nitrogen (but not peptide) bonds][GO:0006807:nitrogen compound metabolic process]	[ko00460:Cyanoamino acid metabolism]	NNU_002551-RA:Nelumbo nucifera|TCP|TCP family protein	XP_021720535.1:bifunctional nitrilase/nitrile hydratase NIT4A [Chenopodium quinoa] >XP_021720536.1 bifunctional nitrilase/nitrile hydratase NIT4A [Chenopodium quinoa] >XP_021720537.1 bifunctional nitrilase/nitrile hydratase NIT4A [Chenopodium quinoa] >XP_021720538.1 bifunctional nitrilase/nitrile hydratase NIT4A [Chenopodium quinoa] >XP_021720539.1	gi|359479486|ref|XM_002272801.2|:PREDICTED: Vitis vinifera bifunctional nitrilase/nitrile hydratase NIT4B-like (LOC100250098), mRNA	W9RSK5|W9RSK5_9ROSA:Bifunctional nitrilase/nitrile hydratase NIT4A;OS=Morus notabilis.;|GO:0016810,|GO:0006807,

geneid	trend	GO	Pathway	TF	NR	NT	Uniprot
					bifunctional nitrilase/nitrile hydratase NIT4A [Chenopodium quinoa]		

geneid	trend	GO	Pathway	TF	NR	NT	Uniprot
DN111999_c0_g1_i2	UP	[GO:0003677:DNA binding][GO:0004518:nuclease activity]	-	-	XP_010682877.1:PREDICTED: uncharacterized protein LOC104897642 [Beta vulgaris subsp. vulgaris] >KMT07548.1 hypothetical protein BVRB_6g151590 [Beta vulgaris subsp. vulgaris]	gi|521707990|gb|GAIA01013972.1|:TSA: Salicornia europaea Unigene14619_Se200S transcribed RNA sequence	A0A0J8C694|A0A0J8C694_BETVU:Uncharacterized protein;OS=Beta vulgaris subsp. vulgaris.;|GO:0003677,GO:0004518,
DN120136_c0_g3_i1	UP	[GO:0009941:chloroplast envelope][GO:0009535:chloroplast thylakoid membrane][GO:0009508:plastid chromosome]	-	-	XP_021720797.1:protein plastid transcriptionally active 16, chloroplasticlike isoform X1 [Chenopodium quinoa]	-	A0A0J8BUE7|A0A0J8BUE7_BETVU:Uncharacterized protein;OS=Beta vulgaris subsp. vulgaris.;|GO:0009941,GO:0009535,GO:0009508,
DN118547_c0_g3_i1	UP	-	-	-	XP_021772095.1:photosystem II 5 kDa protein, chloroplasticlike [Chenopodium	-	A9PIN3|A9PIN3_9ROSI:Putative uncharacterized protein;OS=Populus trichocarpa x

geneid	trend	GO	Pathway	TF	NR	NT	Uniprot
					quinoa]		Populus deltoides.;

geneid	trend	GO	Pathway	TF	NR	NT	Uniprot
DN115031_c1_g3_i1	UP	[GO:0016787:hydrolase activity]	-	-	OVA00002.1:hypothetical protein BVC80_8595g9 [Macleaya cordata]	gi|340842589|gb|HQ664648.1|:Mirabilis jalapa rpl23 pseudogene and tRNA-Ile (trnI-CAU) gene, complete sequence; hypothetical chloroplast RF2 (ycf2) gene, complete cds; tRNA-Leu (trnL-CAA) gene, complete sequence; NADH-plastoquinone oxidoreductase subunit 2 (ndhB) and ribosomal protein S7 (rps7) genes, complete cds; ribosomal protein S12 (rps12) gene, exons 2, 3 and partial cds; and 16S ribosomal RNA	T2DPD7|T2DPD7_PHAVU:Cell wall-associated hydrolase;OS=Phaseolus vulgaris;|GO:0016787,

geneid	trend	GO	Pathway	TF	NR	NT	Uniprot
						(rrn16), tRNA-Ile (trnI-GAU), tRNA-Ala (trnA-UGC), 23S ribosomal RNA (rrn23), 4.5S ribosomal RNA (rrn4.5), 5S ribosomal RNA (rrn5), tRNA-Arg (trnR-ACG), and tRNA-Asn (trnN-GUU) genes, complete sequence; plastid	

geneid	trend	GO	Pathway	TF	NR	NT	Uniprot
DN122911_c1_g1_i1	UP	[GO:0005829:cytosol][GO:0005730:nucleolus][GO:0003676:nucleic acid binding][GO:0000166:nucleotide binding]	[ko03040:Spliceosome][ko04657:IL-17 signaling pathway][ko05168:Herpes simplex infection]	-	XP_021775044.1:serine/argininerich splicing factor SR34Alike [Chenopodium quinoa] >XP_021775045.1 serine/argininerich splicing factor SR34Alike [Chenopodium quinoa] >XP_021775046.1 serine/argininerich splicing factor SR34Alike [Chenopodium quinoa]	gi|521548507|gb|GAHZ01000087.1|:TSA: Salicornia europaea Unigene89_SeCKS transcribed RNA sequence	A0A0J8B8Y6|A0A0J8B8Y6_BETVU:Uncharacterized protein;OS=Beta vulgaris subsp. vulgaris.;|GO:0005829,GO:0005730,|GO:0003676,GO:0000166,

geneid	trend	GO	Pathway	TF	NR	NT	Uniprot
DN122157_c1_g1_i4	UP	[GO:0043531:ADP binding]	-	-	XP_010685163.1:PREDICTED: probable disease resistance protein At1g15890 [Beta vulgaris subsp. vulgaris] >XP_019106476.1 PREDICTED: probable disease resistance protein At1g15890 [Beta vulgaris subsp. vulgaris]	-	A0A0K9RII6|A0A0K9RII6_SPIOL:Uncharacterized protein;OS=Spinacia oleracea;|GO:0043531,
DN121808_c3_g4_i4	UP	[GO:0016021:integral component of membrane][GO:0008270:zinc ion binding]	-	-	XP_021749120.1:E3 ubiquitinprotein ligase At4g11680like [Chenopodium quinoa]	gi|521533788|gb|GAHZ01006406.1|:TSA: Salicornia europaea Unigene6711_SeCKS transcribed RNA sequence	A0A0J8CS68|A0A0J8CS68_BETVU:Uncharacterized protein;OS=Beta vulgaris subsp. vulgaris.;|GO:0016021,|GO:0008270,

geneid	trend	GO	Pathway	TF	NR	NT	Uniprot
DN125985_c1_g1_i12	UP	[GO:0016787:hydrolase activity]	-	329122:Arabidopsis lyrata|C3H|C3H family protein	XP_021771392.1:nudix hydrolase 10like [Chenopodium quinoa]	gi|478164044|gb|GAHK01012559.1|:TSA: Jatropha curcas Unigene10_JC-CK_1A transcribed RNA sequence	A0A0K9RWD3|A0A0K9RWD3_SPIOL:Uncharacterized protein;OS=Spinacia oleracea;|GO:0016787,
DN118442_c0_g1_i1	UP	-	-	-	-	-	-
DN120444_c2_g2_i5	UP	-	-	-	KNA20958.1:hypothetical protein SOVF_047610 isoform C [Spinacia oleracea]	gi|255569226|ref|XM_002525536.1|:Ricinus communis HSP90 co-chaperone, putative, mRNA	A0A0K9RNA5|A0A0K9RNA5_SPIOL: Uncharacterized protein;OS=Spinacia oleracea;
DN117000_c0_g1_i3	UP	[GO:0016788:hydrolase activity, acting on ester bonds]	-	-	XP_021720221.1:GDSL esterase/lipase At3g48460like [Chenopodium quinoa]	gi|326525628|dbj|AK357647.1|:Hordeum vulgare subsp. vulgare mRNA for predicted protein, complete cds, clone: NIASHv1058H16	A0A0J8B867|A0A0J8B867_BETVU:Uncharacterized protein;OS=Beta vulgaris subsp. vulgaris.;|GO:0016788,

geneid	trend	GO	Pathway	TF	NR	NT	Uniprot
DN122228_c1_g5_i2	UP	[GO:0009570:chloroplast stroma][GO:0005525:GTP binding][GO:0003924:GTPase activity][GO:0031425:chloroplast RNA processing][GO:0010468:regulation of gene expression][GO:0000302:response to reactive oxygen species]	-	-	XP_010667054.1:PREDICTED: uncharacterized protein LOC104884155 [Beta vulgaris subsp. vulgaris] >KMS95632.1 hypothetical protein BVRB_006430 [Beta vulgaris subsp. vulgaris]	-	A0A0J8B767|A0A0J8B767_BETVU:Uncharacterized protein;OS=Beta vulgaris subsp. vulgaris.;|GO:0009570,|GO:0005525,GO:0003924,|GO:0031425,GO:0010468,GO:0000302,
DN128241_c5_g1_i15	UP	[GO:0005524:ATP binding][GO:0004326:tetrahydrofolylpolyglutamate synthase activity]	[ko00790:Folate biosynthesis][ko01523:Antifolate resistance]	-	XP_021853507.1:folylpolyglutamate synthase isoform X1 [Spinacia oleracea] >KNA20868.1 hypothetical protein SOVF_048480 isoform A [Spinacia oleracea]	-	A0A0K9RN08|A0A0K9RN08_SPIOL:Uncharacterized protein;OS=Spinacia oleracea;|GO:0005524,GO:0004326,

geneid	trend	GO	Pathway	TF	NR	NT	Uniprot
DN120417_c1_g1_i1	UP	[GO:0009570:chloroplast stroma][GO:0009535:chloroplast thylakoid membrane][GO:0004324:ferredoxin-NADP+ reductase activity][GO:0015979:photosynthesis]	[ko00195:Photosynthesis]	GRMZM2G320305_P01:Zea mays|MYB_related|MYB_related family protein	P41343.1:RecName: Full=FerredoxinNADP reductase, chloroplastic; Short=FNR; Flags: Precursor >AAA33029.1 ferredoxinNADP+ reductase precursor [Mesembryanthemum crystallinum] >prf||1604475A ferredoxin NADP reductase	gi|167255|gb|M25528.1|CIPFNRA:Mesembryanthemum crystallinum ferredoxin-NADP+ reductase precursor, mRNA, complete cds	P41343|FENR_MESCR:Ferredoxin--NADP reductase, chloroplastic;OS=Mesembryanthemum crystallinum;|GO:0009570,GO:0009535,|GO:0004324,|GO:0015979,

geneid	trend	GO	Pathway	TF	NR	NT	Uniprot
DN126949_c2_g7_i8	UP	[GO:0005634:nucleus][GO:0000977:RNA polymerase II regulatory region sequence-specific DNA binding][GO:0003700:DNA-binding transcription factor activity][GO:0000165:MAPK cascade][GO:0045944:positive regulation of transcription by RNA polymerase II][GO:0006351:transcription, DNA-templated]	-	Bv9_221550_mryh.t1:Beta vulgaris|MIKC_MADS|MIKC_MADS family protein	XP_010691403.1:PREDICTED: MADSbox protein JOINTLESS isoform X2 [Beta vulgaris subsp. vulgaris] >KMT00882.1 hypothetical protein BVRB_9g221550 isoform A [Beta vulgaris subsp. vulgaris]	-	A0A0J8BIR5|A0A0J8BIR5_BETVU:Uncharacterized protein;OS=Beta vulgaris subsp. vulgaris.;|GO:0005634,|GO:0000977,GO:0003700,|GO:0000165,GO:0045944,GO:0006351,
DN125279_c3_g2_i6	UP	[GO:0005739:mitochondrion]	-	PSME_00044865-RA:Pseudotsuga menziesii|E2F/DP|E2F/DP family protein	XP_010674380.1:PREDICTED: uncharacterized protein LOC104890548 [Beta vulgaris subsp. vulgaris] >KMT14012.1 hypothetical protein BVRB_4g078410 [Beta vulgaris subsp.	gi|521562420|gb|GAIA01075827.1|:TSA: Salicornia europaea Unigene91866_Se200S transcribed RNA sequence	A0A0J8CKI4|A0A0J8CKI4_BETVU:Uncharacterized protein;OS=Beta vulgaris subsp. vulgaris.;|GO:0005739,

geneid	trend	GO	Pathway	TF	NR	NT	Uniprot
					vulgaris]		
DN128213_c1_g1_i5	UP	[GO:0009570:chloroplast stroma][GO:0009535:chloroplast thylakoid membrane][GO:0005524:ATP binding][GO:0017038:protein import][GO:0006605:protein targeting]	[ko02024:Quorum sensing][ko03060:Protein export][ko03070:Bacterial secretion system]	-	XP_010679492.1:PREDICTED: protein translocase subunit SecA, chloroplastic [Beta vulgaris subsp. vulgaris]	gi|939975|emb|Z49124.1|:S.oleracea chloroplast secA mRNA	Q36795|SECA_SPIOL:Protein translocase subunit SecA, chloroplastic;OS=Spinacia oleracea;|GO:0009570,GO:0009535,|GO:0005524,|GO:0017038,GO:0006605,
DN106119_c0_g1_i1	UP	[GO:0009507:chloroplast][GO:0008080:N-acetyltransferase activity]	-	-	XP_021755832.1:serotonin Nacetyltransferase 2, chloroplasticlike [Chenopodium quinoa]	-	A0A0J8EYP7|A0A0J8EYP7_BETVU:Uncharacterized protein;OS=Beta vulgaris subsp. vulgaris.;|GO:00095

geneid	trend	GO	Pathway	TF	NR	NT	Uniprot
							07,|GO:0008080,
DN115066_c0_g1_i1	UP	-	-	-	-	-	-
DN122216_c3_g2_i8	UP	[GO:0016021:integral component of membrane][GO:0005524:ATP binding][GO:0004672:protein kinase activity]	[ko04064:NF-kappa B signaling pathway][ko04620:Toll-like receptor signaling pathway][ko04624:Toll and Imd signaling pathway][ko04722:Neurotrophin signaling pathway][ko05133:Pertussis][ko05140:Leishmaniasis][ko05142:Chagas disease (American trypanosomiasis)][k	-	XP_010694005.1:PREDICTED: putative receptorlike protein kinase At5g39000 [Beta vulgaris subsp. vulgaris] >KMT18668.1 hypothetical protein BVRB_2g027970 [Beta vulgaris subsp. vulgaris]	-	A0A0J8D3A9|A0A0J8D3A9_BETVU:Uncharacterized protein;OS=Beta vulgaris subsp. vulgaris.;|GO:0016021,|GO:0005524,GO:0004672,

geneid	trend	GO	Pathway	TF	NR	NT	Uniprot
			o05145:Toxoplasmosis][ko05152:Tuberculosis][ko05162:Measles][ko05169:Epstein-Barr virus infection]				
DN119541_c0_g1_i9	UP	[GO:0009535:chloroplast thylakoid membrane][GO:0010598:NAD(P)H dehydrogenase complex (plastoquinone)][GO:0009767:photosynthetic electron transport chain]	-	-	XP_010676290.1:PREDICTED: NAD(P)Hquinone oxidoreductase subunit S, chloroplastic [Beta vulgaris subsp. vulgaris] >KMT11913.1 hypothetical protein BVRB_5g098780	gi|460202941|gb|GABL01058898.1|:TSA: Camelina sativa comp57508_c0_seq2 transcribed RNA sequence	A0A0J8CIK5|A0A0J8CIK5_BETVU:Uncharacterized protein;OS=Beta vulgaris subsp. vulgaris.;|GO:0009535,GO:0010598,|GO:0009767,

geneid	trend	GO	Pathway	TF	NR	NT	Uniprot
					[Beta vulgaris subsp. vulgaris]		

geneid	trend	GO	Pathway	TF	NR	NT	Uniprot
DN118471_c5_g14_i3	UP	-	-	Sme2.5_02290.1_g00006.1:Solanum melongena|GATA|GATA family protein	OMP13171.1:hypothetical protein COLO4_02154 [Corchorus olitorius]	gi|340842549|gb|HQ664640.1|:Bougainvillea glabra rpl23 pseudogene and tRNA-Ile (trnI-CAU) gene, complete sequence; hypothetical chloroplast RF2 (ycf2) gene, complete cds; tRNA-Leu (trnL-CAA) gene, complete sequence; NADH-plastoquinone oxidoreductase subunit 2 (ndhB) and ribosomal protein S7 (rps7) genes, complete cds; ribosomal protein S12 (rps12) gene, exons 2, 3 and partial cds; tRNA-Val (trnV-GAC) and 16S ribosomal RNA (rrn16) genes, complete sequence; tRNA-Ile (trnI-GAU) and	A0A078G2W2|A0A078G2W2_BRANA: BnaC09g16480D protein;OS=Brassica napus;

geneid	trend	GO	Pathway	TF	NR	NT	Uniprot
DN114745_c0_g1_i1	UP	[GO:0010588:cotyledon vascular tissue pattern formation][GO:0010305:leaf vascular tissue pattern formation][GO:0010087:phloem or xylem histogenesis][GO:0080022:primary root development]	-	LPERR12G15710.1:Leersia perrieri|MYB|MYB family protein	XP_010680550.1:PREDICTED: BTB/POZ domaincontaining protein DOT3 [Beta vulgaris subsp. vulgaris]	-	A0A0J8C5G2|A0A0J8C5G2_BETVU:Uncharacterized protein;OS=Beta vulgaris subsp. vulgaris.;|GO:0010588,GO:0010305,GO:0010087,GO:0080022,
DN115615_c0_g2_i8	UP	-	-	-	-	-	-
DN115472_c2_g5_i4	UP	[GO:0004252:serine-type endopeptidase activity]	-	-	XP_021837886.1:protease Dolike 1, chloroplastic [Spinacia oleracea]	gi|521569910|gb|GAIA01073816.1|:TSA: Salicornia europaea Unigene89678_Se200S transcribed RNA sequence	A0A0K9QGC7|A0A0K9QGC7_SPIOL: Uncharacterized protein;OS=Spinacia oleracea;|GO:0004252,
DN118035_c0_g1_i6	UP	[GO:0016788:hydrolase activity, acting on ester bonds]	-	-	XP_021765378.1:GDSL esterase/lipase APGlike [Chenopodium quinoa]	gi|502156133|ref|XM_004510268.1|:PREDICTED: Cicer arietinum GDSL esterase/lipase APG-like (LOC101492933),	A0A0K9R9Z1|A0A0K9R9Z1_SPIOL:Uncharacterized protein;OS=Spinacia oleracea;|GO:0016788,

geneid	trend	GO	Pathway	TF	NR	NT	Uniprot
						mRNA	
DN105641_c0_g3_i7	UP	[GO:0009507:chloroplast][GO:0004497:monooxygenase activity][GO:0016984:ribulose-bisphosphate carboxylase activity][GO:0009853:photorespiration][GO:0019253:reductive pentose-phosphate cycle]	[ko00630:Glyoxylate and dicarboxylate metabolism][ko00710:Carbon fixation in photosynthetic organisms]	-	AAA33037.1:ribulose 1,5bisphosphate carboxylase/oxygenase small subunit [Mesembryanthemum crystallinum]	gi|167267|gb|M31640.1|CIPRBC:Ice plant ribulose-1-5-bisphosphate carboxylase (rbc5) gene, complete cds	Q08183|RBS3_MESCR:Ribulose bisphosphate carboxylase small chain 3, chloroplastic;OS=Mesembryanthemum crystallinum;|GO:0009507,|GO:0004497,GO:0016984,|GO:0009853,GO:0019253,

geneid	trend	GO	Pathway	TF	NR	NT	Uniprot
DN114569_c0_g1_i2	UP	[GO:0003677:DNA binding][GO:0006355:regulation of transcription, DNA-templated]	-	Aqcoe3G342600.1.p:Aquilegia coerulea|Dof|Dof family protein	XP_021842986.1:cyclic dof factor 1like isoform X2 [Spinacia oleracea] >XP_021842987.1 cyclic dof factor 1like isoform X2 [Spinacia oleracea] >XP_021842988.1 cyclic dof factor 1like isoform X2 [Spinacia oleracea]	gi|199580343|gb|AC189646.2|:Brassica rapa subsp. pekinensis clone KBrS009G14, complete sequence	F6H5U5|F6H5U5_VITVI:Putative uncharacterized protein;OS=Vitis vinifera;GN=VIT_14s0108g00980 {ECO:0000313|EMBL:CCB47566.1};|GO:0003677,|GO:0006355,
DN117439_c2_g1_i6	UP	[GO:0009570:chloroplast stroma][GO:0009534:chloroplast thylakoid][GO:0019898:extrinsic component of membrane][GO:0009654:photosystem II oxygen evolving complex][GO:0031977:thylakoid lumen][GO:0005509:calcium ion binding][GO:0015979:photosynthesis]	-	-	XP_010673078.1:PREDICTED: psbP domaincontaining protein 6, chloroplastic [Beta vulgaris subsp. vulgaris] >KMT15232.1 hypothetical protein BVRB_3g063420 [Beta vulgaris subsp. vulgaris]	gi|521570069|gb|GAIA01073657.1|:TSA: Salicornia europaea Unigene89502_Se200S transcribed RNA sequence	A0A0J8CNY4|A0A0J8CNY4_BETVU: Uncharacterized protein;OS=Beta vulgaris subsp. vulgaris.;|GO:0009570,GO:0009534,GO:0019898,GO:0009654,GO:0031977,|GO:0005509,|GO:0015979,

geneid	trend	GO	Pathway	TF	NR	NT	Uniprot
DN120421_c2_g8_i2	UP	[GO:0003677:DNA binding]	-	Bv9_214180_gjwr.t1:Beta vulgaris|MYB|MYB family protein	XP_010690379.1:PREDICTED: mybrelated protein 308 [Beta vulgaris subsp. vulgaris] >KMT01409.1 hypothetical protein BVRB_9g214180 [Beta vulgaris subsp. vulgaris]	gi|521651854|gb|GAIA01040879.1|:TSA: Salicornia europaea Unigene45927_Se200S transcribed RNA sequence	A0A0J8BN74|A0A0J8BN74_BETVU:Uncharacterized protein;OS=Beta vulgaris subsp. vulgaris.;|GO:0003677,
DN123450_c1_g11_i5	UP	-	-	-	XP_021762771.1:FRIGIDAlike protein 3 [Chenopodium quinoa] >XP_021762772.1 FRIGIDAlike protein 3 [Chenopodium quinoa] >XP_021762773.1 FRIGIDAlike protein 3 [Chenopodium quinoa]	gi|521737237|gb|GAIA01008198.1|:TSA: Salicornia europaea Unigene8528_Se200S transcribed RNA sequence	A0A0J8F349|A0A0J8F349_BETVU:Uncharacterized protein;OS=Beta vulgaris subsp. vulgaris.;

geneid	trend	GO	Pathway	TF	NR	NT	Uniprot
DN117978_c0_g4_i2	UP	[GO:0000287:magnesium ion binding][GO:0010333:terpene synthase activity][GO:0008152:metabolic process]	[ko00909:Sesquiterpenoid and triterpenoid biosynthesis]	Cotton_A_17926_BGI-A2_v1.0:Gossypium arboreum|GRAS|GRAS family protein	AHF22834.1:drimenol synthase [Persicaria hydropiper]	-	W0FFD7|W0FFD7_PERHD:Drimenol synthase;OS=Persicaria hydropiper;|GO:0000287,GO:0010333,|GO:0008152,
DN123781_c1_g8_i9	UP	[GO:0016021:integral component of membrane][GO:0005249:voltage-gated potassium channel activity]	-	PH01003957G0030:Phyllostachys heterocycla|E2F/DP|E2F/DP family protein	XP_021837948.1:potassium channel KAT1like [Spinacia oleracea] >KNA08948.1 hypothetical protein SOVF_158100 [Spinacia oleracea]	gi|8896128|gb|AF267754.1|AF267754:Mesembryanthemum crystallinum putative potassium channel protein Kmt1p mRNA, partial cds	A0A0K9QQI5|A0A0K9QQI5_SPIOL:Uncharacterized protein;OS=Spinacia oleracea;|GO:0016021,|GO:0005249,
DN128296_c3_g9_i2	UP	[GO:0030598:rRNA N-glycosylase activity][GO:0006952:defense response][GO:0017148:negative regulation of translation]	-	-	ABI64066.1:ribosome inactivating protein [Bougainvillea spectabilis]	gi|114325705|gb|DQ989495.1|:Bougainvillea spectabilis ribosome inactivating protein (RIP) gene, complete cds	Q09EH2|Q09EH2_9CARY:rRNA N-glycosidase;OS=Bougainvillea spectabilis.;|GO:0030598,|GO:0006952,GO:0017148,

geneid	trend	GO	Pathway	TF	NR	NT	Uniprot
DN126362_c3_g1_i7	UP	[GO:0009535:chloroplast thylakoid membrane][GO:0016021:integral component of membrane][GO:0009522:photosystem I][GO:0009523:photosystem II][GO:0016168:chlorophyll binding][GO:0046872:metal ion binding][GO:0009765:photosynthesis, light harvesting][GO:0018298:protein-chromophore linkage]	[ko00196:Photosynthesis - antenna proteins]	NNU_000849-RA:Nelumbo nucifera|M-type_MADS|M-type_MADS family protein	XP_010678492.1:PREDICTED: chlorophyll ab binding protein 4, chloroplastic [Beta vulgaris subsp. vulgaris] >CAE30280.1 chlorophyll a /b binding protein [Beta vulgaris] >KMT10798.1 hypothetical protein BVRB_5g114540 [Beta vulgaris subsp. vulgaris]	gi|33504459|emb|AJ579711.2|:Beta vulgaris mRNA for chloroplast chlorophyll a/b binding protein (cab11 gene)	Q7XAC6|Q7XAC6_BETVU:Chlorophyll a-b binding protein, chloroplastic;OS=Beta vulgaris;|GO:0009535,GO:0016021,GO:0009522,GO:0009523,|GO:0016168,GO:0046872,|GO:0009765,GO:0018298,
DN118627_c1_g1_i2	UP	-	-	Tp57577_TGAC_v2_mRNA10528:Trifolium pratense|MYB_related|MYB_related family protein	XP_006829398.1:ultravioletB receptor UVR8 isoform X2 [Amborella trichopoda] >ERM96814.1 hypothetical protein AMTR_s00128p00027720 [Amborella	-	W1NPX3|W1NPX3_AMBTC:Uncharacterized protein;OS=Amborella trichopoda.;

geneid	trend	GO	Pathway	TF	NR	NT	Uniprot
					trichopoda]		
DN127603_c 1_g1_i4	UP	-	-	cra_locus_5362_iso _4:Catharanthus roseus|MYB_related|MYB_related family protein	XP_010666903.1:P REDICTED: protein TSS [Beta vulgaris subsp. vulgaris] >KMT20309.1 hypothetical protein BVRB_1g003100 [Beta vulgaris subsp. vulgaris]	gi|359489731|ref|X M_002278334.2|:PR EDICTED: Vitis vinifera protein KIAA0664 homolog (LOC100263581), mRNA	A0A0J8D7F3|A0A0 J8D7F3_BETVU:U ncharacterized protein;OS=Beta vulgaris subsp. vulgaris.;
DN123870_c 4_g3_i1	UP	-	-	-	XP_021853272.1:pe ntatricopeptide repeatcontaining protein At3g46610 [Spinacia oleracea] >KNA17904.1 hypothetical protein SOVF_075500 [Spinacia oleracea]	gi|521500477|gb|GA HZ01030443.1|:TSA : Salicornia europaea Unigene33534_SeC KS transcribed RNA sequence	A0A0K9RG01|A0A 0K9RG01_SPIOL:U ncharacterized protein;OS=Spinacia oleracea;

geneid	trend	GO	Pathway	TF	NR	NT	Uniprot
DN123468_c5_g6_i2	UP	[GO:0019898:extrinsic component of membrane][GO:0009654:photosystem II oxygen evolving complex][GO:0005509:calcium ion binding][GO:0015979:photosynthesis]	[ko00195:Photosynthesis]	Pp3c2_23590V3.6.p:Physcomitrella patens|WRKY|WRKY family protein	XP_010093189.1:Oxygenevolving enhancer protein 2 [Morus notabilis] >EXB53677.1 Oxygenevolving enhancer protein 2 [Morus notabilis]	-	W9RF48|W9RF48_9ROSA:Oxygen-evolving enhancer protein 2;OS=Morus notabilis.;|GO:0019898,GO:0009654,|GO:0005509,|GO:0015979,
DN126430_c3_g2_i5	UP	-	[ko00710:Carbon fixation in photosynthetic organisms]	MDP0000275412:Malus domestica|Trihelix|Trihelix family protein	AOA52388.1:glyceraldehyde3phosphate dehydrogenase [Haloxylon ammodendron]	gi|2529369|gb|L76552.1|SPIGDND:Spinacia oleracea NADP-dependent glyceraldehydephosphate dehydrogenase subunit A mRNA, chloroplast gene encoding chloroplast protein, complete cds	A0A1B2LUN5|A0A1B2LUN5_9CARY:Glyceraldehyde-3-phosphate dehydrogenase;OS=Haloxylon ammodendron.;

geneid	trend	GO	Pathway	TF	NR	NT	Uniprot
DN123384_c0_g2_i9	UP	[GO:0016021:integral component of membrane][GO:0016760:cellulose synthase (UDP-forming) activity][GO:0071555:cell wall organization][GO:0030244:cellulose biosynthetic process]	-	Sme2.5_02298.1_g00001.1:Solanum melongena|ARF|ARF family protein	XP_010675676.1:PREDICTED: cellulose synthaselike protein G2 [Beta vulgaris subsp. vulgaris] >KMT12691.1 hypothetical protein BVRB_4g087890 [Beta vulgaris subsp. vulgaris]	-	A0A0J8F9Z4|A0A0J8F9Z4_BETVU:Uncharacterized protein;OS=Beta vulgaris subsp. vulgaris.;|GO:0016021,|GO:0016760,|GO:0071555,GO:0030244,
DN112996_c0_g1_i2	UP	[GO:0019898:extrinsic component of membrane][GO:0009654:photosystem II oxygen evolving complex][GO:0005509:calcium ion binding][GO:0015979:photosynthesis]	-	-	XP_009771604.1:PREDICTED: thylakoid lumenal 19 kDa protein, chloroplastic [Nicotiana sylvestris] >XP_016499294.1 PREDICTED: thylakoid lumenal 19 kDa protein, chloroplasticlike [Nicotiana tabacum]	-	A0A067L4V9|A0A067L4V9_JATCU:Uncharacterized protein;OS=Jatropha curcas;|GO:0019898,GO:0009654,|GO:0005509,|GO:0015979,

geneid	trend	GO	Pathway	TF	NR	NT	Uniprot
DN123350_c5_g4_i4	UP	[GO:0009535:chloroplast thylakoid membrane][GO:0005507:copper ion binding][GO:0009055:electron transfer activity][GO:0055114:oxidation-reduction process]	[ko00195:Photosynthesis]	-	XP_021860123.1:plastocyanin, chloroplastic [Spinacia oleracea] >P00289.2 RecName: Full=Plastocyanin, chloroplastic; Flags: Precursor >CAA28398.1 unnamed protein product [Spinacia oleracea] >KNA09426.1 hypothetical protein SOVF_153680 [Spinacia oleracea]	gi|359491971|ref|XM_003634300.1|:PREDICTED: Vitis vinifera plastocyanin, chloroplastic-like, transcript variant 2 (LOC100248911), mRNA	P00289|PLAS_SPIOL:Plastocyanin, chloroplastic;OS=Spinacia oleracea;|GO:0009535,|GO:0005507,GO:0009055,|GO:0055114,
DN119582_c1_g1_i1	UP	[GO:0016021:integral component of membrane][GO:0005886:plasma membrane][GO:0008643:carbohydrate transport]	-	mrna24941.1-v1.0-hybrid:Fragaria vesca|DBB|DBB family protein	XP_021775814.1:bidirectional sugar transporter N3like [Chenopodium quinoa]	gi|521742430|gb|GAIA01004953.1|:TSA: Salicornia europaea Unigene5240_Se200S transcribed RNA sequence	A0A0K9RJN8|A0A0K9RJN8_SPIOL:Bidirectional sugar transporter SWEET;OS=Spinacia oleracea;|GO:0016021,GO:0005886,|GO

geneid	trend	GO	Pathway	TF	NR	NT	Uniprot
							:0008643,
DN116095_c0_g2_i7	UP	[GO:0009055:electron transfer activity][GO:0005506:iron ion binding]	-	-	XP_021732923.1:chaperone protein dnaJ C76, chloroplasticlike [Chenopodium quinoa]	gi|521697901|gb|GAIA01018500.1|:TSA: Salicornia europaea Unigene19455_Se200S transcribed RNA sequence	A0A0K9QJ64|A0A0K9QJ64_SPIOL:Uncharacterized protein;OS=Spinacia oleracea;|GO:0009055,GO:0005506,
DN120433_c0_g1_i1	UP	[GO:0004190:aspartic-type endopeptidase activity]	-	Peaxi162Scf00170g01227.1:Petunia axillaris|FAR1|FAR1 family protein	XP_010676965.1:PREDICTED: basic 7S globulin 2 [Beta vulgaris subsp. vulgaris] >KMT12047.1 hypothetical protein BVRB_5g100060 [Beta vulgaris subsp. vulgaris]	-	A0A0J8CEV2|A0A0J8CEV2_BETVU:Uncharacterized protein;OS=Beta vulgaris subsp. vulgaris.;|GO:0004190,

geneid	trend	GO	Pathway	TF	NR	NT	Uniprot
DN127271_c2_g1_i13	UP	[GO:0005524:ATP binding][GO:0003883:CTP synthase activity][GO:0044210:'de novo' CTP biosynthetic process][GO:0006541:glutamine metabolic process]	[ko00240:Pyrimidine metabolism]	PK16897.10:Cannabis sativa|YABBY|YABBY family protein	XP_021837614.1:CTP synthase isoform X1 [Spinacia oleracea]	gi|115489035|ref|NM_001073537.1|:Oryza sativa Japonica Group Os12g0556600 (Os12g0556600) mRNA, partial cds	A0A0J8C8A9|A0A0J8C8A9_BETVU:CTP synthase;OS=Beta vulgaris subsp. vulgaris.;|GO:0005524,GO:0003883,|GO:0044210,GO:0006541,
DN114033_c0_g1_i1	UP	-	-	-	XP_010685041.1:PREDICTED: uncharacterized protein LOC104899533 isoform X1 [Beta vulgaris subsp. vulgaris] >XP_010685042.1 PREDICTED: uncharacterized protein LOC104899533 isoform X1 [Beta vulgaris subsp. vulgaris]	-	A0A0J8ERE7|A0A0J8ERE7_BETVU:Uncharacterized protein;OS=Beta vulgaris subsp. vulgaris.;

geneid	trend	GO	Pathway	TF	NR	NT	Uniprot
					>KMT05651.1 hypothetical protein BVRB_7g167850 isoform B [Beta vulgaris subsp. vulgaris]		
DN90916_c0_g2_i1	UP	[GO:0009733:response to auxin]	[ko04075:Plant hormone signal transduction]	-	XP_014524491.1:PREDICTED: auxininduced protein 6Blike [Vigna radiata var. radiata]	-	I1JXW7|I1JXW7_SOYBN:Uncharacterized protein;OS=Glycine max;|GO:0009733,

geneid	trend	GO	Pathway	TF	NR	NT	Uniprot
DN126764_c0_g1_i3	UP	-	-	AHYPO_010751-RA:Amaranthus hypochondriacus|LBD|LBD family protein	XP_021714438.1:LOB domaincontaining protein 38like [Chenopodium quinoa]	gi|470103628|ref|XM_004288186.1|:PREDICTED: Fragaria vesca subsp. vesca LOB domain-containing protein 38-like (LOC101307625), mRNA	A0A0K9Q7W4|A0A0K9Q7W4_SPIOL: Uncharacterized protein;OS=Spinacia oleracea;
DN128790_c5_g7_i1	UP	-	-	-	XP_021748000.1:thiosulfate sulfurtransferase 16, chloroplasticlike [Chenopodium quinoa]	-	A0A0K9RI51|A0A0K9RI51_SPIOL:Uncharacterized protein;OS=Spinacia oleracea;
DN117055_c0_g3_i2	UP	[GO:0048529:magnesium-protoporphyrin IX monomethyl ester (oxidative) cyclase activity][GO:0046872:metal ion binding][GO:0015995:chlorophyll biosynthetic process][GO:0015979:photosynthesis]	[ko00860:Porphyrin and chlorophyll metabolism]	-	KNA05315.1:hypothetical protein SOVF_191490 [Spinacia oleracea]	gi|521491807|gb|GAHZ01039079.1|:TSA: Salicornia europaea Unigene44266_SeCKS transcribed RNA sequence	A0A0K9QDI4|A0A0K9QDI4_SPIOL:Uncharacterized protein;OS=Spinacia oleracea;|GO:0048529,GO:0046872,|GO:0015995,GO:0015979,

geneid	trend	GO	Pathway	TF	NR	NT	Uniprot
DN128543_c1_g10_i1	UP	[GO:0009535:chloroplast thylakoid membrane][GO:0016021:integral component of membrane][GO:0009522:photosystem I][GO:0009523:photosystem II][GO:0016168:chlorophyll binding][GO:0046872:metal ion binding][GO:0009765:photosynthesis, light harvesting][GO:0018298:protein-chromophore linkage]	[ko00196:Photosynthesis - antenna proteins]	-	P12332.2:RecName: Full=Chlorophyll ab binding protein, chloroplastic; AltName: Full=LHCII type I CAB; Short=LHCP; Flags: Precursor >AAB42157.1 chlorophyla/bbinding protein precursor, partial [Silene latifolia subsp. alba]	gi|413968457|gb|JX576210.1|:Solanum tuberosum clone St-TNT31 chlorophyll a/b-binding protein mRNA, complete cds	P12332|CB21_SILLB:Chlorophyll a-b binding protein, chloroplastic;OS=Silene latifolia subsp. alba;|GO:0009535,GO:0016021,GO:0009522,GO:0009523,|GO:0016168,GO:0046872,|GO:0009765,GO:0018298,
DN114795_c0_g2_i2	UP	-	-	-	KNA15854.1:hypothetical protein SOVF_094380 isoform C [Spinacia oleracea]	-	A0A0K9RA19|A0A0K9RA19_SPIOL:Uncharacterized protein;OS=Spinacia oleracea;

geneid	trend	GO	Pathway	TF	NR	NT	Uniprot
DN120444_c2_g2_i3	UP	-	-	-	XP_021839089.1:uncharacterized protein OsI_027940like isoform X1 [Spinacia oleracea] >KNA20956.1 hypothetical protein SOVF_047610 isoform A [Spinacia oleracea]	-	A0A0K9RN54|A0A0K9RN54_SPIOL:Uncharacterized protein;OS=Spinacia oleracea;
DN118288_c0_g5_i3	UP	[GO:0009976:tocopherol cyclase activity]	[ko00130:Ubiquinone and other terpenoid-quinone biosynthesis]	-	XP_021907820.1:tocopherol cyclase, chloroplastic, partial [Carica papaya]	gi|255550999|ref|XM_002516502.1|:Ricinus communis Tocopherol cyclase, chloroplast precursor, putative, mRNA	B7X941|B7X941_HEVBR:Tocopherol cyclase;OS=Hevea brasiliensis;|GO:0009976,
DN125733_c0_g3_i1	UP	-	-	OB04G29050.1:Oryza brachyantha|HSF|HSF family protein	XP_021765152.1:uncharacterized protein LOC110729684 [Chenopodium quinoa]	-	A0A0K9RWR7|A0A0K9RWR7_SPIOL:Uncharacterized protein;OS=Spinacia oleracea;

geneid	trend	GO	Pathway	TF	NR	NT	Uniprot
DN120854_c0_g1_i2	UP	-	-	DCAR_008117:Daucus carota|bHLH|bHLH family protein	XP_010690216.1:PREDICTED: cysteinerich repeat secretory protein 38like [Beta vulgaris subsp. vulgaris]	-	A0A0J8BIG7|A0A0J8BIG7_BETVU:Uncharacterized protein;OS=Beta vulgaris subsp. vulgaris.;
DN128493_c1_g1_i1	UP	[GO:0009535:chloroplast thylakoid membrane][GO:0016021:integral component of membrane][GO:0009522:photosystem I][GO:0009523:photosystem II][GO:0016168:chlorophyll binding][GO:0046872:metal ion binding][GO:0009765:photosynthesis, light harvesting][GO:0018298:protein-chromophore linkage]	[ko00196:Photosynthesis - antenna proteins]	Bradi3g42627.3.p:Brachypodium distachyon|ERF|ERF family protein	XP_011100602.1:chlorophyll ab binding protein 5, chloroplastic [Sesamum indicum]	gi|225311906|dbj|AK319645.1|:Solanum lycopersicum cDNA, clone: LEFL1092AC01, HTC in leaf	A0A0E0KG01|A0A0E0KG01_ORYPU: Chlorophyll a-b binding protein, chloroplastic;OS=Oryza punctata;|GO:0009535,GO:0016021,GO:0009522,GO:0009523,|GO:0016168,GO:0046872,|GO:0009765,GO:0018298,

geneid	trend	GO	Pathway	TF	NR	NT	Uniprot
DN120071_c 8_g10_i1	UP	[GO:0050660:flavin adenine dinucleotide binding][GO:0016614:oxidoreductase activity, acting on CH-OH group of donors]	-	Pbr042464.1:Pyrus bretschneideri|GRA S|GRAS family protein	XP_010051874.1:P REDICTED: flavindependent oxidoreductase FOX2 [Eucalyptus grandis] >KCW75710.1 hypothetical protein EUGRSUZ_D00088 [Eucalyptus grandis]	gi|511325869|gb|GA KE01007008.1|:TSA : Thlaspi arvense Contig7135 transcribed RNA sequence	A0A059CCN6|A0A 059CCN6_EUCGR: Uncharacterized protein;OS=Eucalyp tus grandis;|GO:005066 0,GO:0016614,
DN118160_c 5_g1_i1	UP	[GO:0009538:photosystem I reaction center][GO:0015979:photosynthesis]	[ko00195:Photosynt hesis]	-	XP_011024524.1:P REDICTED: photosystem I reaction center subunit IV A, chloroplasticlike [Populus euphratica]	gi|157678947|dbj|A B002089.1|:Citrus sinensis psaE1 mRNA for PS1 reaction center subunit III, partial cds, clone: NE510	K4CU43|K4CU43_S OLLC:Uncharacteri zed protein;OS=Solanu m lycopersicum;|GO:0 009538,|GO:001597 9,
DN124515_c 1_g2_i3	UP	[GO:0004729:oxygen-dependent protoporphyrinogen oxidase activity][GO:0006779:porphyrin-cont aining compound biosynthetic process]	[ko00860:Porphyrin and chlorophyll metabolism]	-	XP_002273793.1:P REDICTED: protoporphyrinogen oxidase 1, chloroplastic [Vitis vinifera] >CBI37050.3	gi|292747472|dbj|A K337289.1|:Lotus japonicus cDNA, clone: LjFL1-054-AD02, HTC	D7U2T4|D7U2T4_ VITVI:Putative uncharacterized protein;OS=Vitis vinifera;GN=VIT_0 7s0005g02540 {ECO:0000313|EM

geneid	trend	GO	Pathway	TF	NR	NT	Uniprot
					unnamed protein product, partial [Vitis vinifera]		BL:CBI37050.3};|GO:0004729,|GO:0006779,
DN122348_c3_g1_i1	UP	[GO:0005623:cell][GO:0009055:electron transfer activity][GO:0015035:protein disulfide oxidoreductase activity][GO:0045454:cell redox homeostasis]	-	-	XP_021726538.1:glutaredoxinC9like [Chenopodium quinoa]	-	A0A0J8ENN1|A0A0J8ENN1_BETVU: Uncharacterized protein;OS=Beta vulgaris subsp. vulgaris.;|GO:0005623,|GO:0009055,GO:0015035,|GO:0045454,
DN106676_c0_g3_i1	UP	[GO:0004568:chitinase activity][GO:0005975:carbohydrate metabolic process][GO:0006032:chitin catabolic process]	[ko00520:Amino sugar and nucleotide sugar metabolism]	-	KNA14195.1:hypothetical protein SOVF_109800 [Spinacia oleracea]	-	A0A0K9R3W0|A0A0K9R3W0_SPIOL: Uncharacterized protein;OS=Spinacia oleracea;|GO:0004568,|GO:0005975,GO:0006032,

geneid	trend	GO	Pathway	TF	NR	NT	Uniprot
DN116301_c0_g2_i19	UP	[GO:0005524:ATP binding]	-	-	AAZ41846.1:Rubisco activase 2 [Mesembryanthemum crystallinum]	gi|71834883|gb|DQ087187.1|:Mesembryanthemum crystallinum Rubisco activase 2 mRNA, complete cds	Q45YI8|Q45YI8_MESCR:Rubisco activase 2;OS=Mesembryanthemum crystallinum;|GO:0005524,
DN116862_c1_g1_i1	UP	[GO:0008233:peptidase activity]	[ko04142:Lysosome][ko04612:Antigen processing and presentation]	-	XP_021716669.1:vacuolarprocessing enzymelike [Chenopodium quinoa]	-	Q949L7|Q949L7_BETVU:Putative vacuolar processing enzyme;OS=Beta vulgaris;|GO:0008233,
DN126109_c4_g1_i2	UP	[GO:0009941:chloroplast envelope][GO:0009570:chloroplast stroma][GO:0016787:hydrolase activity][GO:0008152:metabolic process]	-	-	XP_010695958.1:PREDICTED: haloacid dehalogenaselike hydrolase domaincontaining protein At3g48420 [Beta vulgaris subsp. vulgaris] >KMS97233.1 hypothetical protein BVRB_7g176810	gi|521695369|gb|GAIA01019894.1|:TSA: Salicornia europaea Unigene20869_Se200S transcribed RNA sequence	A0A0J8BB53|A0A0J8BB53_BETVU:Uncharacterized protein;OS=Beta vulgaris subsp. vulgaris.;|GO:0009941,GO:0009570,|GO:0016787,|GO:0008152,

geneid	trend	GO	Pathway	TF	NR	NT	Uniprot
					[Beta vulgaris subsp. vulgaris]		
DN121667_c3_g5_i1	UP	[GO:0019898:extrinsic component of membrane][GO:0009654:photosystem II oxygen evolving complex][GO:0005509:calcium ion binding][GO:0015979:photosynthesis]	[ko00195:Photosynthesis]	-	XP_010088099.1:Oxygenevolving enhancer protein 32 [Morus notabilis] >EXB31418.1 Oxygenevolving enhancer protein 32 [Morus notabilis]	gi|160953578|emb|CU224433.1|:Populus EST from leave	W9QPP1|W9QPP1_9ROSA:Oxygen-evolving enhancer protein 3-2;OS=Morus notabilis.;|GO:0019898,GO:0009654,|GO:0005509,|GO:0015979,
DN123214_c4_g2_i3	UP	[GO:0004722:protein serine/threonine phosphatase activity]	[ko04010:MAPK signaling pathway]	-	AAC36700.1:protein phosphatase2C [Mesembryanthemum crystallinum]	gi|3643089|gb|AF075582.1|AF075582:Mesembryanthemum crystallinum clone Mpc8 protein phosphatase-2C	O82471|O82471_MESCR:Protein phosphatase-2C;OS=Mesembryanthemum crystallinum;|GO:00

geneid	trend	GO	Pathway	TF	NR	NT	Uniprot
						(PP2C) mRNA, complete cds	04722,

geneid	trend	GO	Pathway	TF	NR	NT	Uniprot
DN118175_c0_g1_i2	UP	-	-	-	XP_010676863.1:PREDICTED: BTB/POZ domaincontaining protein At3g05675 [Beta vulgaris subsp. vulgaris] >XP_010676865.1 PREDICTED: BTB/POZ domaincontaining protein At3g05675 [Beta vulgaris subsp. vulgaris] >XP_010676866.1 PREDICTED: BTB/POZ domaincontaining protein At3g05675 [Beta vulgaris subsp. vulgaris] >XP_010676867.1 PREDICTED: BTB/POZ domaincontaining	gi|460365624|ref|XM_004228653.1|:PREDICTED: Solanum lycopersicum BTB/POZ domain-containing protein At3g05675-like, transcript variant 2 (LOC101244517), mRNA	A0A0J8CJZ9|A0A0J8CJZ9_BETVU:Uncharacterized protein;OS=Beta vulgaris subsp. vulgaris.;

geneid	trend	GO	Pathway	TF	NR	NT	Uniprot
					protein At3g05675 [Beta vulgaris subsp. vulgaris] >KMT12434.1 hypothetical protein BVRB_5g103650 [Beta vulgaris subsp. vulgaris]		

geneid	trend	GO	Pathway	TF	NR	NT	Uniprot
DN113269_c0_g1_i2	UP	-	-	-	XP_021716236.1:uncharacterized protein LOC110684135 [Chenopodium quinoa]	-	A0A0J8BUT8|A0A0J8BUT8_BETVU:Uncharacterized protein;OS=Beta vulgaris subsp. vulgaris.;
DN123871_c2_g1_i1	UP	-	-	-	XP_010693686.1:PREDICTED: BAG family molecular chaperone regulator 7 [Beta vulgaris subsp. vulgaris] >KMS98865.1 hypothetical protein BVRB_3g067600 [Beta vulgaris subsp. vulgaris]	gi|225466096|ref|XM_002265938.1|:PREDICTED: Vitis vinifera uncharacterized LOC100246705 (LOC100246705), mRNA	A0A0J8E6N4|A0A0J8E6N4_BETVU:Uncharacterized protein;OS=Beta vulgaris subsp. vulgaris.;
DN120268_c0_g1_i6	UP	-	-	-	XP_011071384.1:inactive protein RESTRICTED TEV MOVEMENT 1like [Sesamum indicum]	-	V4N1N0|V4N1N0_EUTSA:Uncharacterized protein;OS=Eutrema salsugineum;

geneid	trend	GO	Pathway	TF	NR	NT	Uniprot
DN127161_c3_g1_i9	UP	-	-	Tp1g05870:Thellungiella parvula|MYB_related|MYB_related family protein	XP_021721284.1:protein CHUP1, chloroplasticlike [Chenopodium quinoa] >XP_021721285.1 protein CHUP1, chloroplasticlike [Chenopodium quinoa]	gi|359472708|ref|XM_002281118.2|:PREDICTED: Vitis vinifera protein CHUP1, chloroplastic-like (LOC100259307), mRNA	A0A0J8CDG4|A0A0J8CDG4_BETVU: Uncharacterized protein;OS=Beta vulgaris subsp. vulgaris.;
DN128244_c4_g1_i1	UP	[GO:0016021:integral component of membrane][GO:0008168:methyltransferase activity][GO:0006694:steroid biosynthetic process]	[ko00100:Steroid biosynthesis]	-	XP_008221201.1:PREDICTED: 24methylenesterol Cmethyltransferase 2 [Prunus mume]	gi|359807408|ref|NM_001254202.1|:Glycine max 24-methylenesterol C-methyltransferase 2-like (SMT2-1), mRNA >gi|242755432|gb|FJ483973.1| Glycine max sterol 24-C methyltransferase 2-1 (SMT2-1) mRNA, complete cds	M5XSG0|M5XSG0_PRUPE:Methyltransferase;OS=Prunus persica;|GO:0016021,|GO:0008168,|GO:0006694,

geneid	trend	GO	Pathway	TF	NR	NT	Uniprot
DN118310_c2_g1_i5	UP	[GO:0016021:integral component of membrane][GO:0005524:ATP binding][GO:0016887:ATPase activity]	-	-	XP_021858978.1:ABC transporter G family member 22like isoform X1 [Spinacia oleracea] >XP_021858979.1 ABC transporter G family member 22like isoform X1 [Spinacia oleracea]	-	A0A164WLW0|A0A164WLW0_DAUCA:Uncharacterized protein;OS=Daucus carota subsp. sativus.;|GO:0016021,|GO:0005524,GO:0016887,
DN115837_c1_g1_i3	UP	[GO:0009507:chloroplast][GO:0046906:tetrapyrrole binding][GO:0010019:chloroplast-nucleus signaling pathway]	-	-	XP_021721688.1:tetrapyrrolebinding protein, chloroplasticlike [Chenopodium quinoa]	-	A0A0J8CUT5|A0A0J8CUT5_BETVU:Uncharacterized protein;OS=Beta vulgaris subsp. vulgaris.;|GO:0009507,|GO:0046906,|GO:0010019,

geneid	trend	GO	Pathway	TF	NR	NT	Uniprot
DN125763_c1_g2_i5	UP	[GO:0005634:nucleus][GO:0003677:DNA binding][GO:0006355:regulation of transcription, DNA-templated][GO:0006351:transcription, DNA-templated]	-	Bv4_084740_hqpd.t1:Beta vulgaris|MYB_related|MYB_related family protein	XP_010675239.1:PREDICTED: protein REVEILLE 2 isoform X1 [Beta vulgaris subsp. vulgaris] >KMT13333.1 hypothetical protein BVRB_4g084740 [Beta vulgaris subsp. vulgaris]	gi|255571278|ref|XM_002526543.1|:Ricinus communis DNA binding protein, putative, mRNA	A0A0J8CMR1|A0A0J8CMR1_BETVU: Uncharacterized protein;OS=Beta vulgaris subsp. vulgaris.;|GO:0005634,|GO:0003677,|GO:0006355,GO:0006351,
DN117380_c2_g3_i3	UP	[GO:0016021:integral component of membrane][GO:0022857:transmembrane transporter activity]	-	NNU_016725-RA:Nelumbo nucifera|GRAS|GRAS family protein	XP_021742013.1:WAT1related protein At3g28050like [Chenopodium quinoa]	-	A0A0K9RYB6|A0A0K9RYB6_SPIOL: Uncharacterized protein;OS=Spinacia oleracea;|GO:0016021,|GO:0022857,

geneid	trend	GO	Pathway	TF	NR	NT	Uniprot
DN121965_c1_g2_i9	UP	[GO:0005737:cytoplasm][GO:0005634:nucleus][GO:0010309:acireductone dioxygenase [iron(II)-requiring] activity][GO:0005506:iron ion binding][GO:0019509:L-methionine salvage from methylthioadenosine]	[ko00270:Cysteine and methionine metabolism]	LPERR03G04120.1:Leersia perrieri|HSF|HSF family protein	XP_021725170.1:1,2dihydroxy3keto5methylthiopentene dioxygenase 1like [Chenopodium quinoa]	-	A0A0K9R9Z4|A0A0K9R9Z4_SPIOL:1,2-dihydroxy-3-keto-5-methylthiopentene dioxygenase;OS=Spinacia oleracea;|GO:0005737,GO:0005634,|GO:0010309,GO:0005506,|GO:0019509,
DN113913_c0_g1_i1	UP	-	-	-	-	-	-
DN128864_c3_g3_i4	UP	[GO:0030598:rRNA N-glycosylase activity][GO:0006952:defense response][GO:0017148:negative regulation of translation]	-	-	BAG71411.1:type1 ribosomeinactivating protein [Spinacia oleracea]	-	B5MET5|B5MET5_SPIOL:rRNA N-glycosidase;OS=Spinacia oleracea;|GO:0030598,|GO:0006952,GO:0017148,
DN124997_c3_g11_i7	UP	[GO:0016021:integral component of membrane][GO:0022857:transmembrane transporter activity]	-	NNU_016725-RA:Nelumbo nucifera|GRAS|GRAS family protein	XP_021842788.1:uncharacterized protein LOC110782850 [Spinacia oleracea]	-	A0A067JMT7|A0A067JMT7_JATCU:WAT1-related protein;OS=Jatropha curcas;|GO:0016021

geneid	trend	GO	Pathway	TF	NR	NT	Uniprot
							,|GO:0022857,
DN120136_c0_g3_i3	UP	-	-	-	XP_021720797.1:protein plastid transcriptionally active 16, chloroplasticlike isoform X1 [Chenopodium quinoa]	-	A0A0K9RKG9|A0A0K9RKG9_SPIOL: Uncharacterized protein;OS=Spinacia oleracea;
DN126366_c4_g1_i3	UP	-	-	Tp57577_TGAC_v2_mRNA31725:Trifolium pratense|WRKY|WRKY family protein	XP_010670718.1:PREDICTED: uncharacterized protein LOC104887707 [Beta vulgaris subsp. vulgaris] >KMT16853.1 hypothetical protein BVRB_2g043330 [Beta vulgaris subsp. vulgaris]	gi|478166033|gb|GAHK01010570.1|:TSA: Jatropha curcas CL4245.Contig1_JC-CK_1A transcribed RNA sequence	A0A0J8CY30|A0A0J8CY30_BETVU:Uncharacterized protein;OS=Beta vulgaris subsp. vulgaris.;

geneid	trend	GO	Pathway	TF	NR	NT	Uniprot
DN120646_c0_g2_i3	UP	[GO:0005783:endoplasmic reticulum][GO:0005509:calcium ion binding][GO:0006457:protein folding]	[ko04141:Protein processing in endoplasmic reticulum][ko04145:Phagosome][ko04612:Antigen processing and presentation][ko05142:Chagas disease (American trypanosomiasis)][ko05166:HTLV-I infection]	-	XP_021772305.1:calreticulin3like [Chenopodium quinoa]	gi|388500321|gb|BT138432.1|:Lotus japonicus clone JCVI-FLLj-22H4 unknown mRNA	A0A151TAW8|A0A151TAW8_CAJCA:Calreticulin-3;OS=Cajanus cajan;|GO:0005783,|GO:0005509,|GO:0006457,
DN118394_c0_g1_i2	UP	[GO:0016021:integral component of membrane]	-	-	KNA08933.1:hypothetical protein SOVF_158220 isoform A [Spinacia oleracea]	-	A0A0K9QQH4|A0A0K9QQH4_SPIOL:Uncharacterized protein;OS=Spinacia oleracea;|GO:0016021,
DN118987_c1_g3_i3	UP	[GO:0005634:nucleus][GO:0043565:sequence-specific DNA binding][GO:0006355:regulation of transcription, DNA-templated]	[ko04612:Antigen processing and presentation][ko05152:Tuberculosis][ko05166:HTLV-I infection]	KFK32404.1:Arabis alpina|NF-YB|NF-YB family protein	KFK32404.1:nuclear transcription factor y subunit b3 [Arabis alpina]	gi|388523220|gb|JQ918286.1|:Medicago truncatula nuclear transcription factor Y subunit B13 (NF-YB13) mRNA,	A0A087GRA2|A0A087GRA2_ARAAL:Nuclear transcription factor y subunit b-3;OS=Arabis alpina;|GO:0005634,

geneid	trend	GO	Pathway	TF	NR	NT	Uniprot
						complete cds	|GO:0043565,|GO:0006355,
DN115858_c4_g3_i7	UP	[GO:0016021:integral component of membrane][GO:0032440:2-alkenal reductase [NAD(P)] activity][GO:0005524:ATP binding][GO:0004674:protein serine/threonine kinase activity][GO:0004714:transmembrane receptor protein tyrosine kinase activity]	[ko04016:MAPK signaling pathway - plant][ko04075:Plant hormone signal transduction][ko04626:Plant-pathogen interaction]	KZV24601.1:Dorcoceras hygrometricum|Trihelix|Trihelix family protein	OWM63581.1:hypothetical protein CDL15_Pgr008124 [Punica granatum]	gi|359485980|ref|XM_002262662.2|:PREDICTED: Vitis vinifera BRASSINOSTEROID INSENSITIVE 1-associated receptor kinase 1-like (LOC100253855), mRNA	B9S2Z2|B9S2Z2_RICCO:BRASSINOSTEROID INSENSITIVE 1-associated receptor kinase 1, putative;OS=Ricinus communis;|GO:0016021,|GO:0032440,GO:0005524,GO:0004674,GO:0004714,
DN127982_c0_g7_i1	UP	-	-	Cotton_A_10855_BGI-A2_v1.0:Gossypium arboreum|bHLH|bHLH family protein	KNA12966.1:hypothetical protein SOVF_120870 [Spinacia oleracea]	gi|521709187|gb|GAIA01013410.1|:TSA: Salicornia europaea Unigene14054_Se200S transcribed RNA sequence	A0A0K9R0C2|A0A0K9R0C2_SPIOL:Uncharacterized protein;OS=Spinacia oleracea;

geneid	trend	GO	Pathway	TF	NR	NT	Uniprot
DN113423_c0_g1_i4	UP	[GO:0016021:integral component of membrane]	-	-	XP_010692659.1:PREDICTED: uncharacterized protein LOC104905741 [Beta vulgaris subsp. vulgaris] >KMT18512.1 hypothetical protein BVRB_2g026500 [Beta vulgaris subsp. vulgaris]	gi|521529819|gb|GAHZ01008632.1|:TSA: Salicornia europaea Unigene9006_SeCKS transcribed RNA sequence	A0A0J8CY47|A0A0J8CY47_BETVU:Uncharacterized protein;OS=Beta vulgaris subsp. vulgaris.;|GO:0016021,
DN109870_c1_g1_i4	UP	[GO:0016021:integral component of membrane][GO:0008270:zinc ion binding]	-	-	KNA04353.1:hypothetical protein SOVF_199770 [Spinacia oleracea]	-	A0A0K9QCD8|A0A0K9QCD8_SPIOL: Uncharacterized protein;OS=Spinacia oleracea;|GO:0016021,|GO:0008270,

geneid	trend	GO	Pathway	TF	NR	NT	Uniprot
DN124482_c3_g1_i5	UP	[GO:0016021:integral component of membrane][GO:0020037:heme binding][GO:0005506:iron ion binding][GO:0004497:monooxygenase activity][GO:0016705:oxidoreductase activity, acting on paired donors, with incorporation or reduction of molecular oxygen]	-	Csa09g009740.1:Camelina sativa|HSF|HSF family protein	XP_021863527.1:cytochrome P450 71A26like isoform X1 [Spinacia oleracea]	-	A5B9L0|A5B9L0_VITVI:Putative uncharacterized protein;OS=Vitis vinifera;|GO:0016021,|GO:0020037,GO:0005506,GO:0004497,GO:0016705,
DN121629_c0_g2_i7	UP	[GO:0005622:intracellular][GO:0008270:zinc ion binding]	-	Manes.11G082100.2.p:Manihot esculenta|DBB|DBB family protein	XP_021753490.1:Bbox zinc finger protein 19like isoform X2 [Chenopodium quinoa]	-	A0A0J8BJT3|A0A0J8BJT3_BETVU:Uncharacterized protein;OS=Beta vulgaris subsp. vulgaris.;|GO:0005622,|GO:0008270,

geneid	trend	GO	Pathway	TF	NR	NT	Uniprot
DN123450_c1_g6_i4	UP	-	-	-	XP_010679002.1:PREDICTED: FRIGIDAlike protein 3 [Beta vulgaris subsp. vulgaris] >XP_010679003.1 PREDICTED: FRIGIDAlike protein 3 [Beta vulgaris subsp. vulgaris] >KMT10136.1 hypothetical protein BVRB_5g118980 [Beta vulgaris subsp. vulgaris]	gi|388491599|gb|BT134071.1|:Lotus japonicus clone JCVI-FLLj-22F12 unknown mRNA	A0A0J8F349|A0A0J8F349_BETVU:Uncharacterized protein;OS=Beta vulgaris subsp. vulgaris.;
DN123905_c1_g5_i1	UP	-	-	-	-	-	-

geneid	trend	GO	Pathway	TF	NR	NT	Uniprot
DN127759_c 2_g1_i5	UP	[GO:0004089:carbonate dehydratase activity][GO:0008270:zinc ion binding][GO:0006730:one-carbon metabolic process]	[ko00910:Nitrogen metabolism]	Gh_D13G2412:Goss ypium hirsutum|GATA|GA TA family protein	XP_010696308.1:P REDICTED: alpha carbonic anhydrase 7like [Beta vulgaris subsp. vulgaris]	-	A0A0J8B744|A0A0 J8B744_BETVU:Un characterized protein;OS=Beta vulgaris subsp. vulgaris.;|GO:00040 89,GO:0008270,|GO :0006730,
DN112838_c 1_g1_i1	UP	[GO:0019898:extrinsic component of membrane][GO:0009654:photosyste m II oxygen evolving complex][GO:0005509:calcium ion binding][GO:0015979:photosynthesis]	[ko00195:Photosynt hesis]	Pp3c2_23590V3.6.p :Physcomitrella patens|WRKY|WRK Y family protein	XP_010680144.1:P REDICTED: oxygenevolving enhancer protein 2, chloroplastic [Beta vulgaris subsp. vulgaris] >KMT09104.1 hypothetical protein BVRB_6g132120 [Beta vulgaris subsp. vulgaris]	gi|21264|emb|X0551 1.1|:Spinach mRNA 23 kDa protein of the photosynthetic oxygen-evolving complex (OEC)	A0A0J8CA65|A0A0 J8CA65_BETVU:U ncharacterized protein;OS=Beta vulgaris subsp. vulgaris.;|GO:00198 98,GO:0009654,|GO :0005509,|GO:00159 79,

geneid	trend	GO	Pathway	TF	NR	NT	Uniprot
DN124060_c1_g11_i14	UP	-	-	-	XP_010692820.1:PREDICTED: uncharacterized protein LOC104905876 isoform X1 [Beta vulgaris subsp. vulgaris] >KMS99550.1 hypothetical protein BVRB_1g022780 isoform A [Beta vulgaris subsp. vulgaris]	gi|521483031|gb|GAHZ01047855.1|:TSA: Salicornia europaea Unigene56349_SeCKS transcribed RNA sequence	A0A0J8BF49|A0A0J8BF49_BETVU:Uncharacterized protein;OS=Beta vulgaris subsp. vulgaris.;
DN123506_c0_g1_i2	UP	-	-	-	XP_021728042.1:uncharacterized protein LOC110695129 [Chenopodium quinoa]	-	A0A0K9RR82|A0A0K9RR82_SPIOL:Uncharacterized protein;OS=Spinacia oleracea;
DN125570_c4_g1_i19	UP	[GO:0016301:kinase activity]	-	Achn360501:Actinidia chinensis|FAR1|FAR1 family protein	CDY18874.1:BnaC04g04760D [Brassica napus]	gi|460368178|ref|XM_004229898.1|:PREDICTED: Solanum lycopersicum uncharacterized LOC101266162	A0A078G066|A0A078G066_BRANA:BnaC04g04760D protein;OS=Brassica napus;|GO:0016301,

geneid	trend	GO	Pathway	TF	NR	NT	Uniprot
						(LOC101266162), mRNA	
DN123894_c0_g1_i5	UP	-	-	-	XP_021717427.1:uncharacterized protein LOC110685243 [Chenopodium quinoa] >XP_021717428.1 uncharacterized protein LOC110685243 [Chenopodium quinoa] >XP_021717429.1 uncharacterized protein LOC110685243 [Chenopodium quinoa]	-	A0A0J8B5U4|A0A0J8B5U4_BETVU:Uncharacterized protein;OS=Beta vulgaris subsp. vulgaris.;

geneid	trend	GO	Pathway	TF	NR	NT	Uniprot
DN123719_c1_g1_i1	UP	[GO:0009507:chloroplast][GO:0005525:GTP binding][GO:0003924:GTPase activity][GO:0003746:translation elongation factor activity]	-	MDP0000428265:Malus domestica|NAC|NAC family protein	XP_010676186.1:PREDICTED: elongation factor G2, chloroplastic [Beta vulgaris subsp. vulgaris] >KMT12558.1 hypothetical protein BVRB_5g098040 [Beta vulgaris subsp. vulgaris]	gi|359496424|ref|XM_002264185.2|:PREDICTED: Vitis vinifera elongation factor G, chloroplastic-like (LOC100257414), mRNA	A0A0J8CKJ2|A0A0J8CKJ2_BETVU:Elongation factor G, chloroplastic;OS=Beta vulgaris subsp. vulgaris.;|GO:0009507,|GO:0005525,GO:0003924,GO:0003746,
DN128107_c2_g1_i5	UP	-	-	-	XP_015876061.1:PREDICTED: uncharacterized protein LOC107412757 [Ziziphus jujuba]	gi|478161421|gb|GAHK01015182.1|:TSA: Jatropha curcas Unigene2636_JC-CK_1A transcribed RNA sequence	W9RB03|W9RB03_9ROSA:Uncharacterized protein;OS=Morus notabilis.;

geneid	trend	GO	Pathway	TF	NR	NT	Uniprot
DN112838_c1_g1_i4	UP	[GO:0019898:extrinsic component of membrane][GO:0009654:photosystem II oxygen evolving complex][GO:0005509:calcium ion binding][GO:0015979:photosynthesis]	[ko00195:Photosynthesis]	Pp3c2_23590V3.6.p:Physcomitrella patens|WRKY|WRKY family protein	XP_010680144.1:PREDICTED: oxygenevolving enhancer protein 2, chloroplastic [Beta vulgaris subsp. vulgaris] >KMT09104.1 hypothetical protein BVRB_6g132120 [Beta vulgaris subsp. vulgaris]	gi|21264|emb|X05511.1|:Spinach mRNA 23 kDa protein of the photosynthetic oxygen-evolving complex (OEC)	A0A0J8CA65|A0A0J8CA65_BETVU:Uncharacterized protein;OS=Beta vulgaris subsp. vulgaris.;|GO:0019898,GO:0009654,|GO:0005509,|GO:0015979,
DN110112_c0_g2_i1	UP	[GO:0005739:mitochondrion][GO:0005840:ribosome][GO:0003735:structural constituent of ribosome][GO:0006412:translation]	-	-	YP_009241985.1:ribosomal protein S3 (mitochondrion) [Oryza minuta] >AMA06704.1 ribosomal protein S3 (mitochondrion) [Oryza minuta]	gi|5737725|gb|AF080085.1|:Corylus colurna ribosomal protein small 3 (rps3) gene, partial cds; mitochondrial gene for mitochondrial product	A0A141NB76|A0A141NB76_ORYMI:Ribosomal protein S3;OS=Oryza minuta.;|GO:0005739,GO:0005840,|GO:0003735,|GO:0006412,
DN119275_c1_g1_i3	UP	-	-	-	-	-	-

geneid	trend	GO	Pathway	TF	NR	NT	Uniprot
DN127809_c0_g2_i1	UP	[GO:0005622:intracellular][GO:0003746:translation elongation factor activity]	-	-	XP_021741470.1:uncharacterized protein LOC110707738 isoform X2 [Chenopodium quinoa]	gi|521732424|gb|GAIA01010604.1|:TSA: Salicornia europaea Unigene11189_Se200S transcribed RNA sequence	A0A0J8BT23|A0A0J8BT23_BETVU:Elongation factor Ts;OS=Beta vulgaris subsp. vulgaris.;|GO:0005622,|GO:0003746,
DN126018_c2_g1_i4	UP	-	-	-	-	-	-
DN127046_c4_g1_i2	UP	[GO:0005737:cytoplasm][GO:0005524:ATP binding][GO:0042026:protein refolding]	[ko03018:RNA degradation][ko04212:Longevity regulating pathway - worm][ko04940:Type I diabetes mellitus][ko05134:Legionellosis][ko05152:Tuberculosis]	DCAR_026693:Daucus carota|bZIP|bZIP family protein	XP_021862888.1:ruBisCO large subunitbinding protein subunit beta, chloroplasticlike [Spinacia oleracea] >XP_021862890.1 ruBisCO large subunitbinding protein subunit beta, chloroplasticlike [Spinacia oleracea] >KNA12027.1 hypothetical protein SOVF_129600 [Spinacia oleracea]	gi|449493561|ref|XM_004159302.1|:PREDICTED: Cucumis sativus ruBisCO large subunit-binding protein subunit beta, chloroplastic-like (LOC101217516), mRNA	A0A0K9QXN7|A0A0K9QXN7_SPIOL: Uncharacterized protein;OS=Spinacia oleracea;|GO:0005737,|GO:0005524,|GO:0042026,

geneid	trend	GO	Pathway	TF	NR	NT	Uniprot
DN119020_c1_g2_i1	UP	[GO:0008270:zinc ion binding]	[ko04141:Protein processing in endoplasmic reticulum]	-	XP_010689296.1:PREDICTED: E3 ubiquitinprotein ligase RMA1H1 [Beta vulgaris subsp. vulgaris] >KMT02197.1 hypothetical protein BVRB_9g206580 [Beta vulgaris subsp. vulgaris]	gi|460412806|ref|XM_004251741.1|:PREDICTED: Solanum lycopersicum E3 ubiquitin-protein ligase RMA1H1-like (LOC101253976), mRNA	A0A0J8BLW5|A0A0J8BLW5_BETVU: Uncharacterized protein;OS=Beta vulgaris subsp. vulgaris.;|GO:0008270,
DN126207_c0_g1_i3	UP	[GO:0016758:transferase activity, transferring hexosyl groups][GO:0008152:metabolic process]	-	PH01002606G0040: Phyllostachys heterocycla|HSF|HSF family protein	XP_010681315.1:PREDICTED: scopoletin glucosyltransferase [Beta vulgaris subsp. vulgaris] >KMT08362.1 hypothetical protein BVRB_6g140440 [Beta vulgaris subsp. vulgaris]	gi|502104360|ref|XM_004492458.1|:PREDICTED: Cicer arietinum soyasapogenol B glucuronide galactosyltransferase-like (LOC101510223), mRNA	A0A0J8C475|A0A0J8C475_BETVU:Glycosyltransferase;OS=Beta vulgaris subsp. vulgaris.;|GO:0016758,|GO:0008152,

geneid	trend	GO	Pathway	TF	NR	NT	Uniprot
DN118851_c4_g2_i14	UP	[GO:0016021:integral component of membrane]	[ko04721:Synaptic vesicle cycle][ko04723:Retrograde endocannabinoid signaling][ko04727:GABAergic synapse][ko05032:Morphine addiction][ko05033:Nicotine addiction]	-	XP_021745828.1:amino acid transporter AVT1Alike [Chenopodium quinoa]	-	A0A0K9QXA1|A0A0K9QXA1_SPIOL:Uncharacterized protein;OS=Spinacia oleracea;|GO:0016021,
DN106926_c0_g1_i4	UP	[GO:0005634:nucleus][GO:0003677:DNA binding][GO:0009908:flower development][GO:0009835:fruit ripening][GO:0010150:leaf senescence][GO:0009825:multidimensional cell growth][GO:0006355:regulation of transcription, DNA-templated][GO:0006351:transcription, DNA-templated]	-	Bv6_153540_qoyx.t1:Beta vulgaris|NAC|NAC family protein	XP_010683140.1:PREDICTED: NAC transcription factor 29 [Beta vulgaris subsp. vulgaris] >KMT07023.1 hypothetical protein BVRB_6g153540 [Beta vulgaris subsp. vulgaris]	gi|449530425|ref|XM_004172148.1|:PREDICTED: Cucumis sativus NAC transcription factor 29-like (LOC101217253), mRNA	A0A0J8C4T4|A0A0J8C4T4_BETVU:Uncharacterized protein;OS=Beta vulgaris subsp. vulgaris.;|GO:0005634,|GO:0003677,|GO:0009908,GO:0009835,GO:0010150,GO:0009825,GO:0006355,GO:0006351,

geneid	trend	GO	Pathway	TF	NR	NT	Uniprot
DN112973_c0_g1_i5	UP	-	-	-	XP_010665890.1:PREDICTED: uncharacterized protein LOC104883135 [Beta vulgaris subsp. vulgaris] >KMS96479.1 hypothetical protein BVRB_9g224720 [Beta vulgaris subsp. vulgaris]	-	A0A0K9RQD7|A0A0K9RQD7_SPIOL: Uncharacterized protein;OS=Spinacia oleracea;
DN125018_c1_g2_i1	UP	[GO:0008233:peptidase activity]	[ko04142:Lysosome][ko04612:Antigen processing and presentation]	-	XP_021849605.1:vacuolarprocessing enzymelike [Spinacia oleracea] >KNA12291.1 hypothetical protein SOVF_127310 [Spinacia oleracea]	gi|210142873|dbj|AK285655.1|:Glycine max cDNA, clone: GMFL01-14-D16	A0A0K9R0H8|A0A0K9R0H8_SPIOL:Uncharacterized protein;OS=Spinacia oleracea;|GO:0008233,

geneid	trend	GO	Pathway	TF	NR	NT	Uniprot
DN113941_c0_g3_i1	UP	[GO:0009535:chloroplast thylakoid membrane][GO:0016021:integral component of membrane][GO:0009522:photosystem I][GO:0009523:photosystem II][GO:0016168:chlorophyll binding][GO:0046872:metal ion binding][GO:0009765:photosynthesis, light harvesting][GO:0018298:protein-chromophore linkage]	[ko00196:Photosynthesis - antenna proteins]	Bradi3g42627.3.p:Brachypodium distachyon|ERF|ERF family protein	XP_009397219.1:PREDICTED: chlorophyll ab binding protein 151, chloroplastic [Musa acuminata subsp. malaccensis] >ABF70157.1 chlorophyll AB binding protein (CAB), putative [Musa acuminata]	gi|9587202|gb|AF279248.1|AF279248:Vigna radiata LHCII type II chlorophyll a/b-binding protein (CipLhcb2) mRNA, complete cds; nuclear gene for chloroplast product	Unreviewed|Q1EP00_MUSAC:Chlorophyll a-b binding protein, chloroplastic;OS=Musa acuminata;|GO:0009535,GO:0016021,GO:0009522,GO:0009523,|GO:0016168,GO:0046872,|GO:0009765,GO:0018298,

geneid	trend	GO	Pathway	TF	NR	NT	Uniprot
DN111348_c0_g1_i3	UP	[GO:0016538:cyclin-dependent protein serine/threonine kinase regulator activity][GO:0016301:kinase activity][GO:0007049:cell cycle][GO:0051301:cell division]	[ko04111:Cell cycle - yeast][ko05200:Pathways in cancer][ko05222:Small cell lung cancer]	Cotton_A_38757_BGI-A2_v1.0:Gossypium arboreum|MYB_related|MYB_related family protein	XP_017603676.1:PREDICTED: cyclindependent kinases regulatory subunit 1 [Gossypium arboreum] >XP_017603677.1 PREDICTED: cyclindependent kinases regulatory subunit 1 [Gossypium arboreum] >XP_017603678.1 PREDICTED: cyclindependent kinases regulatory subunit 1 [Gossypium arboreum] >KHG01535.1 Cyclindependent kinases regulatory subunit 1	gi|478175961|gb|GAHK01000642.1|:TSA: Jatropha curcas CL200.Contig1_JC-CK_1A transcribed RNA sequence	A0A0B0ML77|A0A0B0ML77_GOSAR: Cyclin-dependent kinases regulatory subunit;OS=Gossypium arboreum;|GO:0016538,GO:0016301,|GO:0007049,GO:0051301,

geneid	trend	GO	Pathway	TF	NR	NT	Uniprot
					[Gossypium arboreum] >KHG29549.1 Cyclindependent kinases regulatory subunit 1 [Gossypium arboreum]		

geneid	trend	GO	Pathway	TF	NR	NT	Uniprot
DN113000_c1_g3_i2	UP	[GO:0003824:catalytic activity][GO:0050662:coenzyme binding]	-	MDP0000265073:Malus domestica|NAC|NAC family protein	KNA11911.1:hypothetical protein SOVF_130830 [Spinacia oleracea]	-	A0A0K9QZD0|A0A0K9QZD0_SPIOL: Uncharacterized protein;OS=Spinacia oleracea;|GO:0003824,GO:0050662,
DN126098_c1_g3_i4	UP	[GO:0016021:integral component of membrane][GO:0020037:heme binding][GO:0005506:iron ion binding][GO:0004497:monooxygenase activity][GO:0016705:oxidoreductase activity, acting on paired donors, with incorporation or reduction of molecular oxygen]	-	Bradi2g44117.7.p:Brachypodium distachyon|bHLH|bHLH family protein	KNA12752.1:hypothetical protein SOVF_123110 [Spinacia oleracea]	-	A0A0K9R1T0|A0A0K9R1T0_SPIOL:Uncharacterized protein;OS=Spinacia oleracea;|GO:0016021,|GO:0020037,GO:0005506,GO:0004497,GO:0016705,

geneid	trend	GO	Pathway	TF	NR	NT	Uniprot
DN118083_c2_g4_i2	UP	[GO:0016021:integral component of membrane][GO:0020037:heme binding][GO:0005506:iron ion binding][GO:0004497:monooxygenase activity][GO:0016705:oxidoreductase activity, acting on paired donors, with incorporation or reduction of molecular oxygen]	[ko00905:Brassinosteroid biosynthesis]	Gh_A02G0666:Gossypium hirsutum|G2-like|G2-like family protein	XP_021757486.1:cytochrome P450 90B1like [Chenopodium quinoa]	gi|359476573|ref|XM_002267151.2|:PREDICTED: Vitis vinifera cytochrome P450 90B1-like (LOC100241065), mRNA	A0A061DH82|A0A061DH82_THECC: Cytochrome P450 superfamily protein isoform 1;OS=Theobroma cacao;|GO:0016021,|GO:0020037,GO:0005506,GO:0004497,GO:0016705,

geneid	trend	GO	Pathway	TF	NR	NT	Uniprot
DN117096_c0_g1_i1	UP	[GO:0016791:phosphatase activity]	[ko00630:Glyoxylate and dicarboxylate metabolism]	PK12308.1:Cannabis sativa|MYB_related|MYB_related family protein	XP_010673877.1:PREDICTED: phosphoglycolate phosphatase 1B, chloroplastic [Beta vulgaris subsp. vulgaris] >XP_010673878.1 PREDICTED: phosphoglycolate phosphatase 1B, chloroplastic [Beta vulgaris subsp. vulgaris] >KMT14707.1 hypothetical protein BVRB_4g074740 [Beta vulgaris subsp. vulgaris]	gi|512394856|gb|GAJZ01006314.1|:TSA: Prunus avium Pa_20139 transcribed RNA sequence	A0A0J8CMF9|A0A0J8CMF9_BETVU: Uncharacterized protein;OS=Beta vulgaris subsp. vulgaris.;|GO:0016791,
DN113749_c0_g1_i3	UP	-	-	-	XP_021847390.1:uncharacterized protein LOC110787127 [Spinacia oleracea] >KNA22496.1 hypothetical protein	-	A0A0K9RSG9|A0A0K9RSG9_SPIOL:Uncharacterized protein;OS=Spinacia oleracea;

geneid	trend	GO	Pathway	TF	NR	NT	Uniprot
					SOVF_033700 [Spinacia oleracea]		
DN124290_c1_g6_i9	UP	-	-	-	XP_020705091.1:uncharacterized protein LOC110116006 [Dendrobium catenatum]	gi|478171453|gb|GAHK01005150.1|:TSA: Jatropha curcas CL1737.Contig3_JC-CK_1A transcribed RNA sequence	A0A068UIE1|A0A068UIE1_COFCA:Uncharacterized protein;OS=Coffea canephora;
DN115872_c3_g2_i5	UP	[GO:0010181:FMN binding][GO:0016491:oxidoreductase activity]	-	-	XP_010671596.1:PREDICTED: uncharacterized protein At3g49140 [Beta vulgaris subsp. vulgaris] >KMT16157.1 hypothetical protein BVRB_3g053180 [Beta vulgaris subsp. vulgaris]	gi|521560763|gb|GAIA01076629.1|:TSA: Salicornia europaea Unigene92738_Se200S transcribed RNA sequence	A0A0J8CRD7|A0A0J8CRD7_BETVU: Uncharacterized protein;OS=Beta vulgaris subsp. vulgaris.;|GO:0010181,GO:0016491,

geneid	trend	GO	Pathway	TF	NR	NT	Uniprot
DN123990_c5_g4_i4	UP	[GO:0030170:pyridoxal phosphate binding][GO:0008483:transaminase activity]	[ko00250:Alanine, aspartate and glutamate metabolism][ko00260:Glycine, serine and threonine metabolism][ko00270:Cysteine and methionine metabolism][ko00280:Valine, leucine and isoleucine degradation]	Gh_A09G0861:Gossypium hirsutum|ERF|ERF family protein	XP_010683560.1:PREDICTED: alanineglyoxylate aminotransferase 2 homolog 3, mitochondrial [Beta vulgaris subsp. vulgaris] >KMT06598.1 hypothetical protein BVRB_7g157660 [Beta vulgaris subsp. vulgaris]	gi|521751349|gb|GAIA01000473.1|:TSA: Salicornia europaea Unigene482_Se200S transcribed RNA sequence	A0A0J8C3E5|A0A0J8C3E5_BETVU:Uncharacterized protein;OS=Beta vulgaris subsp. vulgaris.;|GO:0030170,GO:0008483,
DN115268_c0_g1_i2	UP	-	-	-	KNA08979.1:hypothetical protein SOVF_157790 [Spinacia oleracea]	-	A0A0K9QNY4|A0A0K9QNY4_SPIOL: Uncharacterized protein;OS=Spinacia oleracea;
DN120560_c1_g2_i4	UP	[GO:0016021:integral component of membrane]	[ko00062:Fatty acid elongation][ko01040:Biosynthesis of unsaturated fatty acids]	-	XP_021728184.1:verylongchain (3R)3hydroxyacylCoA dehydratase PASTICCINO 2like [Chenopodium quinoa]	gi|521739247|gb|GAIA01007145.1|:TSA: Salicornia europaea Unigene7457_Se200S transcribed RNA sequence	G5DXR7|G5DXR7_SILLA:Very-long-chain (3R)-3-hydroxyacyl-CoA dehydratase;OS=Silene

geneid	trend	GO	Pathway	TF	NR	NT	Uniprot
							latifolia;|GO:0016021,
DN121898_c2_g1_i3	UP	[GO:0016021:integral component of membrane][GO:0005215:transporter activity]	-	mrna00620.1-v1.0-hybrid:Fragaria vesca|BBR-BPC|BBR-BPC family protein	XP_021747687.1:protein NRT1/ PTR FAMILY 2.12like [Chenopodium quinoa]	-	A0A0K9RRF5|A0A0K9RRF5_SPIOL:Uncharacterized protein;OS=Spinacia oleracea;|GO:0016021,|GO:0005215,
DN123468_c5_g4_i1	UP	[GO:0019898:extrinsic component of membrane][GO:0009654:photosystem II oxygen evolving complex][GO:0005509:calcium ion binding][GO:0015979:photosynthesis]	[ko00195:Photosynthesis]	Pp3c2_23590V3.6.p:Physcomitrella patens|WRKY|WRKY family protein	BAG70022.1:23 kDa precursor protein of the oxygenevolving complex [Salicornia europaea]	gi|356567469|ref|XM_003551894.1|:PREDICTED: Glycine max oxygen-evolving enhancer protein 2, chloroplastic-like (LOC100781848), mRNA	B5BT07|B5BT07_SALEU:23 kDa protein of the oxygen-evolving complex;OS=Salicornia europaea;|GO:0019898,GO:0009654,|GO:0005509,|GO:0015979,

geneid	trend	GO	Pathway	TF	NR	NT	Uniprot
DN126651_c3_g5_i2	UP	[GO:0009507:chloroplast][GO:0015035:protein disulfide oxidoreductase activity][GO:0045454:cell redox homeostasis][GO:0006662:glycerol ether metabolic process]	-	AA81G00050:Aethionema arabicum|HRT-like|HRT-like family protein	XP_010678488.1:PREDICTED: thioredoxinlike 12, chloroplastic [Beta vulgaris subsp. vulgaris] >KMT10788.1 hypothetical protein BVRB_5g114440 [Beta vulgaris subsp. vulgaris]	-	A0A0J8CFJ7|A0A0J8CFJ7_BETVU:Uncharacterized protein;OS=Beta vulgaris subsp. vulgaris.;|GO:0009507,|GO:0015035,|GO:0045454,GO:0006662,
DN112747_c0_g1_i1	UP	[GO:0009538:photosystem I reaction center][GO:0015979:photosynthesis]	[ko00195:Photosynthesis]	-	XP_010684821.1:PREDICTED: photosystem I reaction center subunit IV, chloroplastic [Beta vulgaris subsp. vulgaris] >KMT05810.1 hypothetical protein BVRB_7g166490 [Beta vulgaris subsp. vulgaris]	gi|21294|emb|X14018.1|:Spinach psaE mRNA for subunit III precursor of photosystem I reaction centre	A0A0J8BW49|A0A0J8BW49_BETVU: Uncharacterized protein;OS=Beta vulgaris subsp. vulgaris.;|GO:0009538,|GO:0015979,

geneid	trend	GO	Pathway	TF	NR	NT	Uniprot
DN126700_c3_g2_i3	UP	[GO:0005737:cytoplasm][GO:0005524:ATP binding][GO:0042026:protein refolding]	[ko03018:RNA degradation][ko04212:Longevity regulating pathway - worm][ko04940:Type I diabetes mellitus][ko05134:Legionellosis][ko05152:Tuberculosis]	DCAR_026693:Daucus carota|bZIP|bZIP family protein	XP_010671843.1:PREDICTED: ruBisCO large subunitbinding protein subunit beta, chloroplastic [Beta vulgaris subsp. vulgaris] >KMT16357.1 hypothetical protein BVRB_3g055040 [Beta vulgaris subsp. vulgaris]	gi|478163148|gb|GAHK01013455.1|:TSA: Jatropha curcas Unigene907_JC-CK_1A transcribed RNA sequence	A0A0J8CRX1|A0A0J8CRX1_BETVU: Uncharacterized protein;OS=Beta vulgaris subsp. vulgaris.;|GO:0005737,|GO:0005524,|GO:0042026,

geneid	trend	GO	Pathway	TF	NR	NT	Uniprot
DN118317_c0_g1_i4	UP	[GO:0005634:nucleus][GO:0003682:chromatin binding][GO:0003677:DNA binding][GO:0003713:transcription coactivator activity][GO:0003700:DNA-binding transcription factor activity][GO:0008270:zinc ion binding][GO:0006338:chromatin remodeling][GO:0016573:histone acetylation][GO:0035066:positive regulation of histone acetylation][GO:0031540:regulation of anthocyanin biosynthetic process][GO:0006357:regulation of transcription by RNA polymerase II][GO:0006355:regulation of transcription, DNA-templated][GO:0009737:response to abscisic acid][GO:0009733:response to auxin][GO:0046686:response to cadmium ion][GO:0009723:response to ethylene][GO:0009739:response to gibberellin][GO:0009753:response to jasmonic acid][GO:0080167:response to karrikin][GO:0009751:response to salicylic acid][GO:0009651:response to salt stress][GO:0006351:transcription, DNA-templated]	-	AHYPO_000402-RA:Amaranthus hypochondriacus|MYB_related|MYB_related family protein	XP_021747695.1:transcription factor KUA1like [Chenopodium quinoa]	gi|327412632|emb|FR828549.1|:Rosa rugosa mRNA for putative MYB transcription factor (myb16 gene)	O04544|O04544_ARATH:At1g70000; OS=Arabidopsis thaliana;|GO:0005634,|GO:0003682,GO:0003677,GO:0003713,GO:0003700,GO:0008270,|GO:0006338,GO:0016573,GO:0035066,GO:0031540,GO:0006357,GO:0006355,GO:0009737,GO:0009733,GO:0046686,GO:0009723,GO:0009739,GO:0009753,GO:0080167,GO:0009751,GO:0009651,GO:0006351,

geneid	trend	GO	Pathway	TF	NR	NT	Uniprot
DN124890_c1_g2_i2	UP	[GO:0004332:fructose-bisphosphate aldolase activity][GO:0006096:glycolytic process]	[ko00010:Glycolysis / Gluconeogenesis][ko000030:Pentose phosphate pathway][ko00051:Fructose and mannose metabolism][ko00680:Methane metabolism][ko00710:Carbon fixation in photosynthetic organisms]	FANhyb_rscf00000068.1.g00012.1:Fragaria x ananassa|FAR1|FAR1 family protein	XP_021738721.1:fructosebisphosphate aldolase 1, chloroplastic [Chenopodium quinoa]	gi|521752072|gb|GAIA01000090.1|:TSA: Salicornia europaea Unigene93_Se200S transcribed RNA sequence	K4B3P9|K4B3P9_SOLLC:Fructose-bisphosphate aldolase;OS=Solanum lycopersicum;|GO:0004332,|GO:0006096,
DN122212_c2_g1_i4	UP	-	-	-	-	-	-
DN127738_c0_g2_i3	UP	[GO:0050660:flavin adenine dinucleotide binding][GO:0016614:oxidoreductase activity, acting on CH-OH group of donors]	-	CCG025416.1:Populus euphratica|G2-like|G2-like family protein	XP_021863737.1:protein HOTHEADlike [Spinacia oleracea]	-	A0A0K9Q7A0|A0A0K9Q7A0_SPIOL:Uncharacterized protein;OS=Spinacia oleracea;|GO:0050660,GO:0016614,

geneid	trend	GO	Pathway	TF	NR	NT	Uniprot
DN119478_c1_g1_i5	UP	[GO:0016021:integral component of membrane][GO:0042651:thylakoid membrane][GO:0010190:cytochrome b6f complex assembly]	-	-	XP_010670384.1:PREDICTED: uncharacterized protein LOC104887451 [Beta vulgaris subsp. vulgaris] >KMT17192.1 hypothetical protein BVRB_2g041000 [Beta vulgaris subsp. vulgaris]	-	A0A0J8CU96|A0A0J8CU96_BETVU:Uncharacterized protein;OS=Beta vulgaris subsp. vulgaris.;|GO:0016021,GO:0042651,|GO:0010190,
DN127868_c1_g1_i5	UP	[GO:0016021:integral component of membrane][GO:0005524:ATP binding][GO:0042277:peptide binding][GO:0004674:protein serine/threonine kinase activity][GO:1902025:nitrate import][GO:0090548:response to nitrate starvation]	-	Sme2.5_01196.1_g00006.1:Solanum melongena|STAT|STAT family protein	XP_021760432.1:receptor proteintyrosine kinase CEPR2like [Chenopodium quinoa]	-	A0A0J8D723|A0A0J8D723_BETVU:Uncharacterized protein;OS=Beta vulgaris subsp. vulgaris.;|GO:0016021,|GO:0005524,GO:0042277,GO:0004674,|GO:1902025,GO:0090548,

geneid	trend	GO	Pathway	TF	NR	NT	Uniprot
DN118084_c3_g13_i2	UP	[GO:0016491:oxidoreductase activity]	-	Cc01_g08720:Coffea canephora|NAC|NAC family protein	XP_021753384.1:secoisolariciresinol dehydrogenaselike [Chenopodium quinoa]	gi|521452294|gb|GAHZ01068190.1|:TSA: Salicornia europaea Unigene82288_SeCKS transcribed RNA sequence	A0A0K9R9V4|A0A0K9R9V4_SPIOL:Uncharacterized protein;OS=Spinacia oleracea;|GO:0016491,
DN122193_c1_g1_i1	UP	[GO:0042742:defense response to bacterium][GO:0050832:defense response to fungus]	-	-	XP_021864961.1:pathogenesisrelated protein PR4like [Spinacia oleracea] >KNA21920.1 hypothetical protein SOVF_037190 [Spinacia oleracea]	-	A0A0K9RQV4|A0A0K9RQV4_SPIOL: Uncharacterized protein;OS=Spinacia oleracea;|GO:0042742,GO:0050832,
DN120312_c2_g1_i1	UP	[GO:0009507:chloroplast][GO:0005886:plasma membrane]	-	-	XP_010666609.1:PREDICTED: uncharacterized protein LOC104883752 isoform X1 [Beta vulgaris subsp. vulgaris] >KMS95957.1 hypothetical protein BVRB_003200	gi|356537576|ref|XM_003537255.1|:PREDICTED: Glycine max uncharacterized protein LOC100816437 (LOC100816437), mRNA	A0A0J8B837|A0A0J8B837_BETVU:Uncharacterized protein;OS=Beta vulgaris subsp. vulgaris.;|GO:0009507,GO:0005886,

geneid	trend	GO	Pathway	TF	NR	NT	Uniprot
					isoform A [Beta vulgaris subsp. vulgaris]		
DN128865_c7_g6_i2	UP	[GO:0016491:oxidoreductase activity]	[ko00902:Monoterpenoid biosynthesis]	Niben101Scf02581g01014.1:Nicotiana benthamiana|MYB_related|MYB_related family protein	XP_021760280.1:(+)neomenthol dehydrogenaselike [Chenopodium quinoa] >XP_021717522.1 (+)neomenthol dehydrogenaselike [Chenopodium quinoa]	gi|255541519|ref|XM_002511778.1|:Ricinus communis carbonyl reductase, putative, mRNA	A0A068US79|A0A068US79_COFCA:Uncharacterized protein;OS=Coffea canephora;|GO:0016491,

geneid	trend	GO	Pathway	TF	NR	NT	Uniprot
DN125124_c3_g1_i1	UP	[GO:0003676:nucleic acid binding][GO:0000166:nucleotide binding]	-	CCG030364.1:Populus euphratica|NF-YA|NF-YA family protein	XP_021770788.1:28 kDa ribonucleoprotein, chloroplasticlike [Chenopodium quinoa]	gi|359477775|ref|XM_002285433.2|:PREDICTED: Vitis vinifera uncharacterized LOC100261382 (LOC100261382), mRNA	A0A0J8B6R4|A0A0J8B6R4_BETVU:Uncharacterized protein;OS=Beta vulgaris subsp. vulgaris.;|GO:0003676,GO:0000166,
DN124032_c4_g5_i4	UP	[GO:0003824:catalytic activity][GO:0008152:metabolic process]	[ko00280:Valine, leucine and isoleucine degradation][ko00640:Propanoate metabolism]	-	OMO57826.1:hypothetical protein COLO4_35079 [Corchorus olitorius]	gi|521508439|gb|GAHZ01022481.1|:TSA: Salicornia europaea Unigene24172_SeCKS transcribed RNA sequence	G7JTF7|G7JTF7_MEDTR:Branched chain alpha-keto acid dehydrogenase E1 beta subunit;OS=Medicago truncatula;GN=MTR_4g021470 {ECO:0000313|EMBL:AES87123.1};|GO:0003824,|GO:0008152,
DN122050_c0_g1_i1	UP	-	-	-	-	-	-

geneid	trend	GO	Pathway	TF	NR	NT	Uniprot
DN127046_c4_g1_i3	UP	[GO:0005737:cytoplasm][GO:0005524:ATP binding][GO:0042026:protein refolding]	[ko03018:RNA degradation][ko04212:Longevity regulating pathway - worm][ko04940:Type I diabetes mellitus][ko05134:Legionellosis][ko05152:Tuberculosis]	DCAR_026693:Daucus carota|bZIP|bZIP family protein	XP_021862888.1:ruBisCO large subunitbinding protein subunit beta, chloroplasticlike [Spinacia oleracea] >XP_021862890.1 ruBisCO large subunitbinding protein subunit beta, chloroplasticlike [Spinacia oleracea] >KNA12027.1 hypothetical protein SOVF_129600 [Spinacia oleracea]	gi|449493561|ref|XM_004159302.1|:PREDICTED: Cucumis sativus ruBisCO large subunit-binding protein subunit beta, chloroplastic-like (LOC101217516), mRNA	A0A0K9QXN7|A0A0K9QXN7_SPIOL: Uncharacterized protein;OS=Spinacia oleracea;|GO:0005737,|GO:0005524,|GO:0042026,
DN124476_c0_g1_i4	UP	[GO:0003993:acid phosphatase activity][GO:0046872:metal ion binding]	-	PDK_30s873781g005:Phoenix dactylifera|C3H|C3H family protein	XP_010683763.1:PREDICTED: purple acid phosphatase 22 isoform X1 [Beta vulgaris subsp. vulgaris] >KMT06815.1 hypothetical protein BVRB_7g159710	gi|357601489|gb|JN835292.1|:Gossypium hirsutum PAP18 mRNA, complete cds	A0A0J8BZ70|A0A0J8BZ70_BETVU:Purple acid phosphatase;OS=Beta vulgaris subsp. vulgaris.;|GO:0003993,GO:0046872,

geneid	trend	GO	Pathway	TF	NR	NT	Uniprot
					[Beta vulgaris subsp. vulgaris]		
DN113514_c1_g1_i5	UP	[GO:0016832:aldehyde-lyase activity][GO:0051287:NAD binding][GO:0004616:phosphogluconate dehydrogenase (decarboxylating) activity][GO:0008270:zinc ion binding][GO:0005975:carbohydrate metabolic process]	-	440731:Selaginella moellendorffii|ERF|ERF family protein	XP_010693952.1:PREDICTED: uncharacterized protein LOC104906826 [Beta vulgaris subsp. vulgaris] >XP_010693953.1 PREDICTED: uncharacterized protein LOC104906826 [Beta vulgaris subsp. vulgaris] >KMS98693.1 hypothetical protein	gi|521686022|gb|GAIA01024479.1|:TSA: Salicornia europaea Unigene26195_Se200S transcribed RNA sequence	A0A0J8BF81|A0A0J8BF81_BETVU:Uncharacterized protein;OS=Beta vulgaris subsp. vulgaris.;|GO:0016832,GO:0051287,GO:0004616,GO:0008270,|GO:0005975,

geneid	trend	GO	Pathway	TF	NR	NT	Uniprot
					BVRB_3g069420 [Beta vulgaris subsp. vulgaris]		
DN118105_c1_g2_i8	UP	[GO:0016021:integral component of membrane]	-	-	XP_021734094.1:WAT1related protein At3g02690, chloroplasticlike [Chenopodium quinoa]	gi|521551022|gb|GAIA01081971.1|:TSA: Salicornia europaea Unigene100081_Se200S transcribed RNA sequence	A0A0K9RJ91|A0A0K9RJ91_SPIOL:Uncharacterized protein;OS=Spinacia oleracea;|GO:0016021,

geneid	trend	GO	Pathway	TF	NR	NT	Uniprot
DN125524_c0_g2_i1	UP	[GO:0005737:cytoplasm][GO:0007015:actin filament organization][GO:0071483:cellular response to blue light][GO:0009904:chloroplast accumulation movement][GO:0009903:chloroplast avoidance movement]	-	Lus10012302:Linum usitatissimum|HB-other|HB-other family protein	XP_010694520.1:PREDICTED: J domaincontaining protein required for chloroplast accumulation response 1 isoform X1 [Beta vulgaris subsp. vulgaris] >KMS98314.1 hypothetical protein BVRB_4g093810 isoform B [Beta vulgaris subsp. vulgaris]	-	A0A0J8BAD0|A0A0J8BAD0_BETVU: Uncharacterized protein;OS=Beta vulgaris subsp. vulgaris.;|GO:0005737,|GO:0007015,GO:0071483,GO:0009904,GO:0009903,
DN128947_c2_g1_i2	UP	[GO:0004540:ribonuclease activity][GO:0003723:RNA binding][GO:0006396:RNA processing]	-	-	XP_021751543.1:ribonuclease E/Glike protein, chloroplastic isoform X1 [Chenopodium quinoa]	gi|521520063|gb|GAHZ01014112.1|:TSA: Salicornia europaea Unigene14770_SeCKS transcribed RNA sequence	A0A0J8EUQ5|A0A0J8EUQ5_BETVU: Uncharacterized protein;OS=Beta vulgaris subsp. vulgaris.;|GO:0004540,GO:0003723,|GO:0006396,

geneid	trend	GO	Pathway	TF	NR	NT	Uniprot
DN123450_c 1_g11_i4	UP	-	-	-	XP_021733466.1:FRIGIDAlike protein 3 [Chenopodium quinoa] >XP_021733467.1 FRIGIDAlike protein 3 [Chenopodium quinoa] >XP_021733468.1 FRIGIDAlike protein 3 [Chenopodium quinoa]	gi|521737237|gb|GAIA01008198.1|:TSA: Salicornia europaea Unigene8528_Se200S transcribed RNA sequence	A0A0J8F349|A0A0J8F349_BETVU:Uncharacterized protein;OS=Beta vulgaris subsp. vulgaris.;

geneid	trend	GO	Pathway	TF	NR	NT	Uniprot
DN108987_c0_g2_i9	UP	[GO:0016597:amino acid binding][GO:0008152:metabolic process]	-	Zmw_sc00881.1.g00060.1:Zoysia matrella|NAC|NAC family protein	XP_010679322.1:PREDICTED: ACT domaincontaining protein ACR3 [Beta vulgaris subsp. vulgaris] >XP_010679331.1 PREDICTED: ACT domaincontaining protein ACR3 [Beta vulgaris subsp. vulgaris] >KMT19679.1 hypothetical protein BVRB_1g010650 [Beta vulgaris subsp. vulgaris]	-	A0A0J8D5Q5|A0A0J8D5Q5_BETVU:Uncharacterized protein;OS=Beta vulgaris subsp. vulgaris.;|GO:0016597,|GO:0008152,

geneid	trend	GO	Pathway	TF	NR	NT	Uniprot
DN119963_c6_g1_i2	UP	[GO:0009941:chloroplast envelope][GO:0009535:chloroplast thylakoid membrane][GO:0045261:proton-transporting ATP synthase complex, catalytic core F(1)][GO:0046933:proton-transporting ATP synthase activity, rotational mechanism][GO:0015986:ATP synthesis coupled proton transport][GO:0009772:photosynthetic electron transport in photosystem II]	[ko00190:Oxidative phosphorylation][ko00195:Photosynthesis]	-	XP_010686838.1:PREDICTED: ATP synthase gamma chain, chloroplastic [Beta vulgaris subsp. vulgaris] >KMT04174.1 hypothetical protein BVRB_8g186220 [Beta vulgaris subsp. vulgaris]	gi|449479456|ref|XM_004155556.1|:PREDICTED: Cucumis sativus ATP synthase gamma chain, chloroplastic-like (LOC101218747), mRNA	A0A0J8BVZ7|A0A0J8BVZ7_BETVU:Uncharacterized protein;OS=Beta vulgaris subsp. vulgaris.;|GO:0009941,GO:0009535,GO:0045261,|GO:0046933,|GO:0015986,GO:0009772,
DN119479_c0_g2_i1	UP	[GO:0016630:protochlorophyllide reductase activity]	-	TRIUR3_22095-P1: Triticum urartu|C2H2|C2H2 family protein	XP_021767760.1:protochlorophyllide reductaselike [Chenopodium quinoa]	gi|224126712|ref|XM_002319872.1|:Populus trichocarpa predicted protein, mRNA	G5DVX6|G5DVX6_SILLA:Protochlorophyllide reductase B;OS=Silene latifolia;|GO:0016630,
DN114093_c5_g7_i1	UP	-	-	-	XP_021764988.1:Fbox protein At5g52880like [Chenopodium	-	A0A0J8CVM5|A0A0J8CVM5_BETVU: Uncharacterized protein;OS=Beta

geneid	trend	GO	Pathway	TF	NR	NT	Uniprot
					quinoa]		vulgaris subsp. vulgaris.;
DN116125_c1_g1_i1	UP	[GO:0016705:oxidoreductase activity, acting on paired donors, with incorporation or reduction of molecular oxygen][GO:0016123:xanthophyll biosynthetic process]	[ko00906:Carotenoid biosynthesis]	-	XP_010696541.1:PREDICTED: lycopene epsilon cyclase, chloroplastic [Beta vulgaris subsp. vulgaris] >KMS96852.1 hypothetical protein BVRB_8g199280 [Beta vulgaris subsp. vulgaris]	gi|18419660|gb|AF463497.1|:Spinacia oleracea lycopene epsilon-cyclase (lec) mRNA, complete cds	A0A0J8BAA9|A0A0J8BAA9_BETVU: Uncharacterized protein;OS=Beta vulgaris subsp. vulgaris.;|GO:0016705,|GO:0016123,
DN125335_c2_g5_i1	UP	-	-	Bv5_125870_rycp.t1:Beta vulgaris|Nin-like|Nin-like family protein	XP_010695590.1:PREDICTED: protein NLP7 [Beta vulgaris subsp. vulgaris] >KMS97563.1 hypothetical protein BVRB_5g125870 [Beta vulgaris subsp.	-	A0A0J8BC07|A0A0J8BC07_BETVU:Uncharacterized protein;OS=Beta vulgaris subsp. vulgaris.;

geneid	trend	GO	Pathway	TF	NR	NT	Uniprot
					vulgaris]		
DN115432_c0_g1_i1	UP	[GO:0005634:nucleus]	-	CCG029944.1:Populus euphratica|NF-YC|NF-YC family protein	XP_010245157.1:PREDICTED: dr1associated corepressorlike [Nelumbo nucifera]	-	B9SB51|B9SB51_RICCO:Negative cofactor 2 transcriptional co-repressor, putative;OS=Ricinus communis;|GO:0005634,
DN127453_c2_g1_i4	UP	[GO:0016021:integral component of membrane][GO:0008289:lipid binding]	-	-	XP_021720270.1:uncharacterized protein LOC110687931 [Chenopodium quinoa]	gi|478160693|gb|GAHK01015910.1|:TSA: Jatropha curcas Unigene3364_JC-CK_1A transcribed RNA sequence	A0A0K9R4F9|A0A0K9R4F9_SPIOL:Uncharacterized protein;OS=Spinacia oleracea;|GO:0016021,|GO:0008289,

geneid	trend	GO	Pathway	TF	NR	NT	Uniprot
DN120527_c1_g1_i6	UP	-	-	-	XP_010667552.1:PREDICTED: uncharacterized protein LOC104884574 [Beta vulgaris subsp. vulgaris] >KMS95302.1 hypothetical protein BVRB_009570 isoform A [Beta vulgaris subsp. vulgaris]	-	A0A0J8B676|A0A0J8B676_BETVU:Uncharacterized protein;OS=Beta vulgaris subsp. vulgaris.;
DN123758_c1_g1_i4	UP	[GO:0016021:integral component of membrane]	-	Achn317751:Actinidia chinensis|bHLH|bHLH family protein	XP_021844493.1:PGR5like protein 1A, chloroplastic [Spinacia oleracea] >KNA15411.1 hypothetical protein SOVF_098540 [Spinacia oleracea]	gi|521502004|gb|GAHZ01028916.1|:TSA: Salicornia europaea Unigene31845_SeCKS transcribed RNA sequence	A0A0K9R7D6|A0A0K9R7D6_SPIOL:Uncharacterized protein;OS=Spinacia oleracea;|GO:0016021,

geneid	trend	GO	Pathway	TF	NR	NT	Uniprot
DN120279_c6_g11_i1	UP	-	-	-	AJK93577.1:RD22like dehydrationresponsive protein [Suaeda glauca]	-	A0A0C5B956|A0A0C5B956_9CARY:Suaeda glauca RD22-like dehydration-responsive protein mRNA;OS=Suaeda glauca.;
DN125837_c3_g1_i1	UP	[GO:0016021:integral component of membrane][GO:0005524:ATP binding][GO:0016301:kinase activity][GO:0016462:pyrophosphatase activity][GO:0009814:defense response, incompatible interaction]	-	-	GAV80215.1:PRK domaincontaining protein/CYTH domaincontaining protein [Cephalotus follicularis]	gi|225425040|ref|XM_002270584.1|:PREDICTED: Vitis vinifera uridine-cytidine kinase C-like (LOC100266718), mRNA	A0A061EAS2|A0A061EAS2_THECC:Phosphoribulokinase / Uridine kinase family isoform 1;OS=Theobroma cacao;|GO:0016021,|GO:0005524,GO:0016301,GO:0016462,|GO:0009814,
DN118288_c0_g5_i4	UP	[GO:0009976:tocopherol cyclase activity]	[ko00130:Ubiquinone and other terpenoid-quinone biosynthesis]	-	XP_021739186.1:tocopherol cyclase, chloroplasticlike [Chenopodium quinoa]	gi|219842173|dbj|AB376095.1|:Hevea brasiliensis tc mRNA for tocopherol cyclase, complete cds	A0A0K9QFP0|A0A0K9QFP0_SPIOL:Uncharacterized protein;OS=Spinacia oleracea;|GO:0009976,

geneid	trend	GO	Pathway	TF	NR	NT	Uniprot
DN121726_c1_g1_i1	UP	-	-	XP_016511122.1:Nicotiana tabacum|bHLH|bHLH family protein	KZV51009.1:Metallocarboxypeptidase inhibitor [Dorcoceras hygrometricum]	gi|340842549|gb|HQ664640.1|:Bougainvillea glabra rpl23 pseudogene and tRNA-Ile (trnI-CAU) gene, complete sequence; hypothetical chloroplast RF2 (ycf2) gene, complete cds; tRNA-Leu (trnL-CAA) gene, complete sequence; NADH-plastoquinone oxidoreductase subunit 2 (ndhB) and ribosomal protein S7 (rps7) genes, complete cds; ribosomal protein S12 (rps12) gene, exons 2, 3 and partial cds; tRNA-Val (trnV-GAC) and 16S ribosomal RNA (rrn16) genes, complete sequence; tRNA-Ile (trnI-GAU) and	M1DI68|M1DI68_SOLTU:Uncharacterized protein;OS=Solanum tuberosum;

geneid	trend	GO	Pathway	TF	NR	NT	Uniprot
DN127868_c1_g1_i1	UP	[GO:0016021:integral component of membrane][GO:0005524:ATP binding][GO:0042277:peptide binding][GO:0004674:protein serine/threonine kinase activity][GO:1902025:nitrate import][GO:0090548:response to nitrate starvation]	-	Sme2.5_01196.1_g00006.1:Solanum melongena|STAT|STAT family protein	XP_021760432.1:receptor proteintyrosine kinase CEPR2like [Chenopodium quinoa]	-	A0A0J8D723|A0A0J8D723_BETVU:Uncharacterized protein;OS=Beta vulgaris subsp. vulgaris.;|GO:0016021,|GO:0005524,GO:0042277,GO:0004674,|GO:1902025,GO:0090548,
DN109186_c0_g1_i1	UP	-	-	-	-	-	-
DN128790_c5_g19_i3	UP	-	-	-	-	-	-

geneid	trend	GO	Pathway	TF	NR	NT	Uniprot
DN128354_c1_g2_i16	UP	[GO:0003677:DNA binding][GO:0004518:nuclease activity]	-	-	XP_010680737.1:PREDICTED: uncharacterized protein LOC104895820 isoform X1 [Beta vulgaris subsp. vulgaris] >XP_010680738.1 PREDICTED: uncharacterized protein LOC104895820 isoform X1 [Beta vulgaris subsp. vulgaris] >XP_010680739.1 PREDICTED: uncharacterized protein LOC104895820 isoform X1 [Beta vulgaris subsp. vulgaris] >XP_010680741.1 PREDICTED: uncharacterized protein LOC104895820 isoform X2 [Beta vulgaris subsp.	gi|255566109|ref|XM_002523997.1|:Ricinus communis conserved hypothetical protein, mRNA	A0A0J8C9R2|A0A0J8C9R2_BETVU:Uncharacterized protein;OS=Beta vulgaris subsp. vulgaris.;|GO:0003677,GO:0004518,

geneid	trend	GO	Pathway	TF	NR	NT	Uniprot
DN123786_c 1_g2_i1	UP	-	-	MDP0000153000:Malus domestica|bHLH|bHLH family protein	XP_021850169.1:transcription factor bHLH144like [Spinacia oleracea] >KNA23000.1 hypothetical protein SOVF_027880 [Spinacia oleracea]	gi|359483842|ref|XM_002275357.2|:PREDICTED: Vitis vinifera transcription factor bHLH144-like, transcript variant 2 (LOC100264025), mRNA	A0A0K9RTY3|A0A0K9RTY3_SPIOL: Uncharacterized protein;OS=Spinacia oleracea;
DN126451_c 1_g6_i6	UP	[GO:0005524:ATP binding][GO:0004672:protein kinase activity]	-	-	XP_021754724.1:serine/threonineprotein kinase STN7, chloroplasticlike [Chenopodium quinoa]	-	A0A0K9QAH6|A0A0K9QAH6_SPIOL: Uncharacterized protein;OS=Spinacia oleracea;|GO:0005524,GO:0004672,

geneid	trend	GO	Pathway	TF	NR	NT	Uniprot
DN123350_c5_g4_i2	UP	[GO:0009535:chloroplast thylakoid membrane][GO:0005507:copper ion binding][GO:0009055:electron transfer activity][GO:0055114:oxidation-reduction process]	[ko00195:Photosynthesis]	-	XP_021860123.1:plastocyanin, chloroplastic [Spinacia oleracea] >P00289.2 RecName: Full=Plastocyanin, chloroplastic; Flags: Precursor >CAA28398.1 unnamed protein product [Spinacia oleracea] >KNA09426.1 hypothetical protein SOVF_153680 [Spinacia oleracea]	gi|359491971|ref|XM_003634300.1|:PREDICTED: Vitis vinifera plastocyanin, chloroplastic-like, transcript variant 2 (LOC100248911), mRNA	P00289|PLAS_SPIOL:Plastocyanin, chloroplastic;OS=Spinacia oleracea;|GO:0009535,|GO:0005507,GO:0009055,|GO:0055114,
DN125181_c1_g2_i6	UP	[GO:0005829:cytosol][GO:0030117:membrane coat][GO:0009506:plasmodesma][GO:0006886:intracellular protein transport][GO:0016192:vesicle-mediated transport]	[ko04142:Lysosome]	-	XP_010676834.1:PREDICTED: AP4 complex subunit epsilon [Beta vulgaris subsp. vulgaris] >KMT12414.1 hypothetical protein	gi|224129593|ref|XM_002328719.1|:Populus trichocarpa predicted protein, mRNA	A0A0J8CJY5|A0A0J8CJY5_BETVU:Uncharacterized protein;OS=Beta vulgaris subsp. vulgaris.;|GO:0005829,GO:0030117,GO:0009506,|GO:00068

geneid	trend	GO	Pathway	TF	NR	NT	Uniprot
					BVRB_5g103460 [Beta vulgaris subsp. vulgaris]		86,GO:0016192,
DN119133_c3_g6_i9	UP	[GO:0003824:catalytic activity][GO:0008152:metabolic process]	[ko00280:Valine, leucine and isoleucine degradation][ko00640:Propanoate metabolism]	PH01003957G0030: Phyllostachys heterocycla|E2F/DP|E2F/DP family protein	KNA11018.1:hypothetical protein SOVF_139050 [Spinacia oleracea]	gi|511324566|gb|GAKE01008311.1|:TSA: Thlaspi arvense Contig8449 transcribed RNA sequence	A0A0K9QWD2|A0A0K9QWD2_SPIOL:Uncharacterized protein;OS=Spinacia oleracea;|GO:0003824,|GO:0008152,
DN115443_c2_g1_i6	UP	[GO:0003824:catalytic activity]	-	-	XP_021850093.1:protein HEATSTRESSASSOCIATED 32 [Spinacia oleracea]	gi|212675231|gb|FJ411078.1|:Garcinia mangostana HSA32 mRNA, partial cds	A0A0J8B1H0|A0A0J8B1H0_BETVU:Uncharacterized protein;OS=Beta vulgaris subsp. vulgaris.;|GO:0003824,

geneid	trend	GO	Pathway	TF	NR	NT	Uniprot
DN129028_c2_g6_i5	UP	[GO:0010181:FMN binding][GO:0016491:oxidoreductase activity]	-	-	XP_021726749.1:uncharacterized protein At3g49140like [Chenopodium quinoa]	gi|521562811|gb|GAIA01075437.1|:TSA: Salicornia europaea Unigene91434_Se200S transcribed RNA sequence	A0A0J8CRD7|A0A0J8CRD7_BETVU: Uncharacterized protein;OS=Beta vulgaris subsp. vulgaris.;|GO:0010181,GO:0016491,
DN128254_c0_g1_i8	UP	[GO:0043531:ADP binding][GO:0006952:defense response]	-	Migut.K01050.1.p: Mimulus guttatus|bHLH|bHLH family protein	XP_010671190.1:PREDICTED: probable disease resistance protein RF45 isoform X1 [Beta vulgaris subsp. vulgaris] >XP_019103985.1 PREDICTED: probable disease resistance protein RF45 isoform X1 [Beta vulgaris subsp. vulgaris] >KMT16752.1 hypothetical protein BVRB_3g050340 isoform B [Beta	-	A0A0J8FLG1|A0A0J8FLG1_BETVU:Uncharacterized protein;OS=Beta vulgaris subsp. vulgaris.;|GO:0043531,|GO:0006952,

geneid	trend	GO	Pathway	TF	NR	NT	Uniprot
					vulgaris subsp. vulgaris]		
DN127680_c0_g2_i12	UP	[GO:0016021:integral component of membrane][GO:0005524:ATP binding][GO:0042626:ATPase activity, coupled to transmembrane movement of substances]	-	RrC2480_p5:Raphanus raphanistrum|HSF|HSF family protein	XP_021853038.1:putative ABC transporter C family member 15 [Spinacia oleracea]	gi|478175585|gb|GAHK01001018.1|:TSA: Jatropha curcas CL296.Contig1_JC-CK_1A transcribed RNA sequence	A0A0J8BIE8|A0A0J8BIE8_BETVU:Uncharacterized protein;OS=Beta vulgaris subsp. vulgaris.;|GO:0016021,|GO:0005524,GO:0042626,

geneid	trend	GO	Pathway	TF	NR	NT	Uniprot
DN123784_c4_g1_i5	UP	[GO:0004838:L-tyrosine:2-oxoglutarate aminotransferase activity][GO:0030170:pyridoxal phosphate binding][GO:0006520:cellular amino acid metabolic process][GO:0010189:vitamin E biosynthetic process]	[ko00130:Ubiquinone and other terpenoid-quinone biosynthesis][ko00270:Cysteine and methionine metabolism][ko00350:Tyrosine metabolism][ko00360:Phenylalanine metabolism][ko00400:Phenylalanine, tyrosine and tryptophan biosynthesis][ko00401:Novobiocin biosynthesis][ko00950:Isoquinoline alkaloid biosynthesis][ko00960:Tropane, piperidine and pyridine alkaloid biosynthesis]	-	XP_010675472.1:PREDICTED: probable aminotransferase TAT2 isoform X2 [Beta vulgaris subsp. vulgaris]	gi|359493822|ref|XM_002282628.2|:PREDICTED: Vitis vinifera probable aminotransferase TAT2-like, transcript variant 1 (LOC100241782), mRNA	A0A0J8CHC8|A0A0J8CHC8_BETVU: Uncharacterized protein;OS=Beta vulgaris subsp. vulgaris.;|GO:0004838,GO:0030170,|GO:0006520,GO:0010189,

geneid	trend	GO	Pathway	TF	NR	NT	Uniprot
DN113874_c0_g2_i4	UP	[GO:0016021:integral component of membrane]	-	-	XP_021765661.1:uncharacterized protein LOC110730191 [Chenopodium quinoa]	gi|521525139|gb|GAHZ01011304.1|:TSA: Salicornia europaea Unigene11921_SeCKS transcribed RNA sequence	A0A0J8F6U0|A0A0J8F6U0_BETVU:Uncharacterized protein;OS=Beta vulgaris subsp. vulgaris.;|GO:0016021,
DN122208_c1_g1_i1	UP	[GO:0009941:chloroplast envelope][GO:0009535:chloroplast thylakoid membrane][GO:0009538:photosystem I reaction center][GO:0010287:plastoglobule][GO:0015979:photosynthesis]	[ko00195:Photosynthesis]	-	XP_010690394.1:PREDICTED: photosystem I reaction center subunit III, chloroplastic [Beta vulgaris subsp. vulgaris] >KMT01421.1 hypothetical protein BVRB_9g214300 [Beta vulgaris subsp. vulgaris]	gi|224057967|ref|XM_002299378.1|:Populus trichocarpa hypothetical protein, mRNA	A0A0J8EEM4|A0A0J8EEM4_BETVU:Uncharacterized protein;OS=Beta vulgaris subsp. vulgaris.;|GO:0009941,GO:0009535,GO:0009538,GO:0010287,|GO:0015979,

geneid	trend	GO	Pathway	TF	NR	NT	Uniprot
DN124514_c 1_g7_i6	UP	[GO:0004252:serine-type endopeptidase activity]	-	Achn120161:Actinid ia chinensis|bZIP|bZIP family protein	XP_010675634.1:P REDICTED: subtilisinlike protease SBT5.4 isoform X1 [Beta vulgaris subsp. vulgaris] >KMT13056.1 hypothetical protein BVRB_4g087680 [Beta vulgaris subsp. vulgaris]	-	A0A0J8FB28|A0A0 J8FB28_BETVU:Un characterized protein;OS=Beta vulgaris subsp. vulgaris.;|GO:00042 52,
DN109387_c 1_g1_i1	UP	[GO:0016021:integral component of membrane]	-	-	XP_012472375.1:P REDICTED: uncharacterized protein LOC105789545 [Gossypium raimondii] >KJB06576.1 hypothetical protein B456_001G092300 [Gossypium raimondii] >KJB06577.1	gi|359485352|ref|X M_003633215.1|:PR EDICTED: Vitis vinifera uncharacterized LOC100243650, transcript variant 2 (LOC100243650), mRNA	A0A0D2PRL2|A0A 0D2PRL2_GOSRA: Uncharacterized protein;OS=Gossypi um raimondii;|GO:0016 021,

geneid	trend	GO	Pathway	TF	NR	NT	Uniprot
					hypothetical protein B456_001G092300 [Gossypium raimondii]		
DN116423_c2_g1_i5	UP	-	-	-	-	-	-

geneid	trend	GO	Pathway	TF	NR	NT	Uniprot
DN120025_c0_g5_i2	UP	-	-	Do001369.1:Dichanthelium oligosanthes|MYB|MYB family protein	XP_010682446.1:PREDICTED: uncharacterized protein LOC104897290 [Beta vulgaris subsp. vulgaris] >XP_010682449.1 PREDICTED: uncharacterized protein LOC104897290 [Beta vulgaris subsp. vulgaris] >XP_010682451.1 PREDICTED: uncharacterized protein LOC104897290 [Beta vulgaris subsp. vulgaris] >XP_019105947.1 PREDICTED: uncharacterized protein	-	A0A0J8C6P1|A0A0J8C6P1_BETVU:Uncharacterized protein;OS=Beta vulgaris subsp. vulgaris.;

geneid	trend	GO	Pathway	TF	NR	NT	Uniprot
					LOC104897290 [Beta vulgaris subsp. vulgaris] >KMT07713.1 hypothetical protein BVRB_6g148340 [Beta vulgaris subsp. vulgaris]		

geneid	trend	GO	Pathway	TF	NR	NT	Uniprot
DN124022_c0_g1_i1	UP	[GO:0016021:integral component of membrane][GO:0009579:thylakoid]	-	-	XP_004306610.1:PREDICTED: protein CURVATURE THYLAKOID 1B, chloroplastic [Fragaria vesca subsp. vesca]	-	W9S6N7|W9S6N7_9ROSA:Uncharacterized protein;OS=Morus notabilis.;|GO:0016021,GO:0009579,
DN119428_c2_g1_i5	UP	[GO:0016021:integral component of membrane][GO:0005524:ATP binding][GO:0046872:metal ion binding]	[ko00190:Oxidative phosphorylation]	Lus10040166:Linum usitatissimum|GRAS|GRAS family protein	XP_021758477.1:ATPase 4, plasma membranetype [Chenopodium quinoa]	gi|33943130|gb|AY347715.1|:Juglans regia plasma membrane H+-ATPase (AHA1) mRNA, complete cds	A0A103XY06|A0A103XY06_CYNCS: Cation-transporting P-type ATPase;OS=Cynara cardunculus var. scolymus;|GO:0016021,|GO:0005524,GO:0046872,
DN117918_c0_g3_i1	UP	[GO:0009507:chloroplast][GO:0080085:signal recognition particle, chloroplast targeting][GO:0045038:protein import into chloroplast thylakoid membrane][GO:0009416:response to light stimulus]	-	-	KNA15378.1:hypothetical protein SOVF_098790 [Spinacia oleracea]	gi|521599620|gb|GAIA01064655.1|:TSA: Salicornia europaea Unigene77651_Se200S transcribed RNA sequence	A0A0K9R768|A0A0K9R768_SPIOL:Uncharacterized protein;OS=Spinacia oleracea;|GO:0009507,GO:0080085,|GO:0045038,GO:0009416,

geneid	trend	GO	Pathway	TF	NR	NT	Uniprot
DN123400_c0_g2_i3	UP	[GO:0009507:chloroplast][GO:0051537:2 iron, 2 sulfur cluster binding][GO:0009055:electron transfer activity][GO:0046872:metal ion binding][GO:0022900:electron transport chain]	[ko00195:Photosynthesis]	-	O04683.1:RecName: Full=Ferredoxin1, chloroplastic; AltName: Full=Ferredoxin I; Flags: Precursor >AAB61593.1 ferredoxin I precursor [Mesembryanthemum crystallinum]	gi|388510581|gb|BT143563.1|:Medicago truncatula clone JCVI-FLMt-7F8 unknown mRNA	O04683|FER1_MESCR:Ferredoxin-1, chloroplastic;OS=Mesembryanthemum crystallinum;|GO:0009507,|GO:0051537,GO:0009055,GO:0046872,|GO:0022900,
DN127818_c1_g1_i8	UP	-	-	-	XP_021719755.1:plastidlipidassociated protein, chloroplasticlike [Chenopodium quinoa]	-	A0A0K9QBN7|A0A0K9QBN7_SPIOL: Uncharacterized protein;OS=Spinacia oleracea;
DN120954_c5_g1_i2	UP	[GO:0005634:nucleus][GO:0003677:DNA binding][GO:0003700:DNA-binding transcription factor activity][GO:0006351:transcription, DNA-templated]	-	Bv6_127190_stch.t1:Beta vulgaris|ERF|ERF family protein	XP_021752063.1:LOW QUALITY PROTEIN: ethyleneresponsive transcription factor ERF010like [Chenopodium quinoa]	gi|161138203|gb|EF051463.1|:Glycine soja isolate 4-18 dehydration responsive element-binding protein 1-like gene, complete sequence	A0A0K9QM16|A0A0K9QM16_SPIOL: Uncharacterized protein;OS=Spinacia oleracea;|GO:0005634,|GO:0003677,GO:0003700,|GO:0006351,

geneid	trend	GO	Pathway	TF	NR	NT	Uniprot
DN112726_c1_g7_i2	UP	-	-	-	-	-	-
DN120289_c4_g1_i1	UP	[GO:0005840:ribosome][GO:0003735:structural constituent of ribosome][GO:0006412:translation]	[ko03010:Ribosome]	-	XP_021748334.1:50S ribosomal protein L5, chloroplastic [Chenopodium quinoa]	gi|7582404|gb|AF250923.1|AF250923:Spinacia oleracea plastid ribosomal protein PRPL5 (PrpL5) mRNA, partial cds; nuclear gene for chloroplast product	A0A0V0HKS8|A0A0V0HKS8_SOLCH: Putative 50S ribosomal protein L5, chloroplastic-like;OS=Solanum chacoense;|GO:0005840,|GO:0003735,|GO:0006412,
DN117689_c1_g1_i4	UP	[GO:0016021:integral component of membrane][GO:0016787:hydrolase activity][GO:0006629:lipid metabolic process]	-	-	XP_021768782.1:uncharacterized protein LOC110733070 [Chenopodium quinoa]	gi|521735544|gb|GAIA01009137.1|:TSA: Salicornia europaea Unigene9496_Se200S transcribed RNA sequence	A0A0J8C8K8|A0A0J8C8K8_BETVU:Uncharacterized protein;OS=Beta vulgaris subsp. vulgaris.;|GO:0016021,|GO:0016787,|GO:0006629,

geneid	trend	GO	Pathway	TF	NR	NT	Uniprot
DN123547_c5_g8_i1	UP	-	-	-	XP_021847932.1:chaperone protein dnaJ 8, chloroplastic [Spinacia oleracea] >KNA26024.1 hypothetical protein SOVF_000120 [Spinacia oleracea]	-	A0A0K9S429|A0A0K9S429_SPIOL:Uncharacterized protein;OS=Spinacia oleracea;
DN119619_c0_g1_i3	UP	[GO:0005524:ATP binding][GO:0004674:protein serine/threonine kinase activity]	-	PDK_30s682911g002:Phoenix dactylifera|C2H2|C2H2 family protein	XP_021756547.1:Gtype lectin Sreceptorlike serine/threonineprotein kinase LECRK3 [Chenopodium quinoa]	-	A5BLK2|A5BLK2_VITVI:Putative uncharacterized protein;OS=Vitis vinifera;|GO:0005524,GO:0004674,
DN125732_c2_g2_i8	UP	[GO:0008270:zinc ion binding][GO:1902456:regulation of stomatal opening]	[ko04115:p53 signaling pathway][ko04120:Ubiquitin mediated proteolysis][ko05162:Measles]	MDP0000124187:Malus domestica|C2H2|C2H2 family protein	XP_010689159.1:PREDICTED: E3 ubiquitinprotein ligase MIEL1 [Beta vulgaris subsp. vulgaris]	-	A0A0J8EH89|A0A0J8EH89_BETVU:Uncharacterized protein;OS=Beta vulgaris subsp. vulgaris.;|GO:0008270,|GO:1902456,

geneid	trend	GO	Pathway	TF	NR	NT	Uniprot
DN125763_c1_g2_i1	UP	[GO:0005634:nucleus][GO:0003677:DNA binding][GO:0006355:regulation of transcription, DNA-templated][GO:0006351:transcription, DNA-templated]	-	Bv4_084740_hqpd.t1:Beta vulgaris|MYB_related|MYB_related family protein	XP_010675239.1:PREDICTED: protein REVEILLE 2 isoform X1 [Beta vulgaris subsp. vulgaris] >KMT13333.1 hypothetical protein BVRB_4g084740 [Beta vulgaris subsp. vulgaris]	gi|255571278|ref|XM_002526543.1|:Ricinus communis DNA binding protein, putative, mRNA	A0A0J8CMR1|A0A0J8CMR1_BETVU: Uncharacterized protein;OS=Beta vulgaris subsp. vulgaris.;|GO:0005634,|GO:0003677,|GO:0006355,GO:0006351,
DN123159_c1_g1_i5	UP	[GO:0009570:chloroplast stroma][GO:0005524:ATP binding][GO:0051536:iron-sulfur cluster binding][GO:0016226:iron-sulfur cluster assembly]	-	RrC216_p2:Raphanus raphanistrum|bHLH|bHLH family protein	XP_021764457.1:feS cluster assembly factor HCF101, chloroplasticlike [Chenopodium quinoa] >XP_021764458.1 feS cluster assembly factor HCF101, chloroplasticlike [Chenopodium quinoa]	gi|521578103|gb|GAIA01069978.1|:TSA: Salicornia europaea Unigene84255_Se200S transcribed RNA sequence	A0A0J8EJ83|A0A0J8EJ83_BETVU:Uncharacterized protein;OS=Beta vulgaris subsp. vulgaris.;|GO:0009570,|GO:0005524,GO:0051536,|GO:0016226,

geneid	trend	GO	Pathway	TF	NR	NT	Uniprot
DN123533_c0_g1_i2	UP	[GO:0030154:cell differentiation][GO:0048831:regulation of shoot system development][GO:0006355:regulation of transcription, DNA-templated][GO:0006351:transcription, DNA-templated]	-	Bv_011420_aoao.t1: Beta vulgaris|GRAS|GRAS family protein	XP_010667697.1:PREDICTED: scarecrowlike protein 15 [Beta vulgaris subsp. vulgaris] >KMS95202.1 hypothetical protein BVRB_011420 [Beta vulgaris subsp. vulgaris]	-	A0A0J8B5W8|A0A0J8B5W8_BETVU: Uncharacterized protein;OS=Beta vulgaris subsp. vulgaris.;|GO:0030154,GO:0048831,GO:0006355,GO:0006351,
DN123760_c5_g3_i17	UP	[GO:0005777:peroxisome][GO:0016787:hydrolase activity]	-	-	XP_010685890.1:PREDICTED: shewanellalike protein phosphatase 1 [Beta vulgaris subsp. vulgaris] >KMT05376.1 hypothetical protein BVRB_7g175080 [Beta vulgaris subsp. vulgaris]	gi|478172846|gb|GAHK01003757.1|:TSA: Jatropha curcas CL1224.Contig1_JC-CK_1A transcribed RNA sequence	A0A0J8EQS9|A0A0J8EQS9_BETVU:Uncharacterized protein;OS=Beta vulgaris subsp. vulgaris.;|GO:0005777,|GO:0016787,

geneid	trend	GO	Pathway	TF	NR	NT	Uniprot
DN125651_c8_g2_i1	UP	[GO:0004525:ribonuclease III activity][GO:0006396:RNA processing]	-	-	XP_010676009.1:PREDICTED: ribonuclease 3like protein 3 [Beta vulgaris subsp. vulgaris]	-	A0A0J8CL27|A0A0J8CL27_BETVU:Uncharacterized protein;OS=Beta vulgaris subsp. vulgaris.;|GO:0004525,|GO:0006396,
DN119087_c4_g2_i2	UP	[GO:0009507:chloroplast][GO:0015035:protein disulfide oxidoreductase activity][GO:0045454:cell redox homeostasis][GO:0006662:glycerol ether metabolic process]	[ko04621:NOD-like receptor signaling pathway][ko05418:Fluid shear stress and atherosclerosis]	-	P09856.2:RecName: Full=Thioredoxin Ftype, chloroplastic; Short=TrxF; Flags: Precursor >CAA33082.1 unnamed protein product [Spinacia oleracea]	-	P09856|TRXF_SPIOL:Thioredoxin F-type, chloroplastic;OS=Spinacia oleracea;|GO:0009507,|GO:0015035,|GO:0045454,GO:0006662,
DN122861_c2_g1_i4	UP	[GO:0046872:metal ion binding][GO:0004655:porphobilinogen synthase activity][GO:0006779:porphyrin-containing compound biosynthetic process]	[ko00860:Porphyrin and chlorophyll metabolism]	-	XP_021759725.1:deltaaminolevulinic acid dehydratase, chloroplastic [Chenopodium quinoa] >XP_021759726.1 deltaaminolevulinic acid dehydratase,	gi|805061|emb|X57842.1|:S.oleraceae ala1 mRNA for delta-aminolevulinic acid dehydratase	A0A0K9S004|A0A0K9S004_SPIOL:Delta-aminolevulinic acid dehydratase;OS=Spinacia oleracea;|GO:0046872,GO:0004655,|GO:0006779,

geneid	trend	GO	Pathway	TF	NR	NT	Uniprot
					chloroplastic [Chenopodium quinoa]		
DN117500_c0_g8_i5	UP	[GO:0004089:carbonate dehydratase activity][GO:0008270:zinc ion binding][GO:0015976:carbon utilization]	[ko00910:Nitrogen metabolism]	Jcr4S00223.20:Jatropha curcas|bHLH|bHLH family protein	AEV42277.1:beta carbonic anhydrase 2 [Mesembryanthemum nodiflorum]	gi|302768556|ref|XM_002967652.1|:Selaginella moellendorffii hypothetical protein, mRNA	G9FZ36|G9FZ36_MESNO:Carbonic anhydrase;OS=Mesembryanthemum nodiflorum;|GO:0004089,GO:0008270,|GO:0015976,
DN118282_c2_g8_i4	UP	[GO:0016021:integral component of membrane][GO:0042578:phosphoric ester hydrolase activity][GO:0048193:Golgi vesicle transport]	-	-	KVH91022.1:Synaptojanin, Nterminal, partial [Cynara cardunculus var. scolymus]	-	A0A103XHX7|A0A103XHX7_CYNCS:Synaptojanin, N-terminal;OS=Cynara cardunculus var. scolymus;|GO:0016021,|GO:0042578,|GO:0048193,

geneid	trend	GO	Pathway	TF	NR	NT	Uniprot
DN128864_c 3_g1_i2	UP	[GO:0030598:rRNA N-glycosylase activity][GO:0051607:defense response to virus][GO:0017148:negative regulation of translation]	-	-	Q40772.1:RecName: Full=Antiviral protein 2; AltName: Full=PAPII; AltName: Full=Ribosomeinactivating protein; AltName: Full=rRNA Nglycosidase; Flags: Precursor >CAA55342.1 pokeweed antiviral protein II [Phytolacca americana] >prf||2014245A antiviral protein II	-	Q40772|RIP2_PHYAM:Antiviral protein 2;OS=Phytolacca americana;|GO:0030598,|GO:0051607,GO:0017148,
DN119886_c 5_g1_i2	UP	[GO:0009507:chloroplast][GO:0016021:integral component of membrane][GO:0009522:photosystem I][GO:0009767:photosynthetic electron transport chain]	[ko00195:Photosynthesis]	-	XP_021715688.1:photosystem I subunit Olike [Chenopodium quinoa]	-	A0A0J8BAP5|A0A0J8BAP5_BETVU:Uncharacterized protein;OS=Beta vulgaris subsp. vulgaris.;|GO:0009507,GO:0016021,GO:

geneid	trend	GO	Pathway	TF	NR	NT	Uniprot
							0009522,|GO:0009767,
DN125974_c0_g1_i1	UP	[GO:0005524:ATP binding][GO:0004672:protein kinase activity]	-	-	XP_021764364.1:serine/threonineprotein kinase Nek2like [Chenopodium quinoa] >XP_021764365.1 serine/threonineprotein kinase Nek2like [Chenopodium quinoa]	gi|359479670|ref|XM_003632280.1|:PREDICTED: Vitis vinifera serine/threonine-protein kinase Nek2-like (LOC100852976), mRNA	A0A0J8C938|A0A0J8C938_BETVU:Uncharacterized protein;OS=Beta vulgaris subsp. vulgaris.;|GO:0005524,GO:0004672,
DN126700_c2_g1_i4	UP	[GO:0043565:sequence-specific DNA binding][GO:0003700:DNA-binding transcription factor activity]	-	Bv2_034240_wzwm.t2:Beta vulgaris|WRKY|WRKY family protein	KMT17893.1:hypothetical protein BVRB_2g034240 isoform A [Beta vulgaris subsp. vulgaris]	-	A0A0J8D148|A0A0J8D148_BETVU:Uncharacterized protein;OS=Beta vulgaris subsp. vulgaris.;|GO:0043565,GO:0003700,

geneid	trend	GO	Pathway	TF	NR	NT	Uniprot
DN128415_c0_g14_i9	UP	-	[ko03013:RNA transport]	-	XP_021742340.1:protein HASTY 1like isoform X1 [Chenopodium quinoa]	gi|189163240|dbj|AP010474.1|:Lotus japonicus genomic DNA, chromosome 1, clone: LjB12K22, BM1097, complete sequence	A0A0K9QPY8|A0A0K9QPY8_SPIOL: Uncharacterized protein;OS=Spinacia oleracea;
DN119920_c2_g5_i4	UP	-	[ko00902:Monoterpenoid biosynthesis]	Niben101Scf02581g01014.1:Nicotiana benthamiana|MYB_related|MYB_related family protein	XP_021760280.1:(+)neomenthol dehydrogenaselike [Chenopodium quinoa] >XP_021717522.1 (+)neomenthol dehydrogenaselike [Chenopodium quinoa]	gi|359489757|ref|XM_002271991.2|:PREDICTED: Vitis vinifera (+)-neomenthol dehydrogenase-like (LOC100255075), mRNA	A0A0K9R8D1|A0A0K9R8D1_SPIOL:Uncharacterized protein;OS=Spinacia oleracea;
DN122762_c0_g2_i1	UP	-	-	-	KNA11927.1:hypothetical protein SOVF_130660 [Spinacia oleracea]	-	A0A0K9QXD1|A0A0K9QXD1_SPIOL: Uncharacterized protein;OS=Spinacia oleracea;

geneid	trend	GO	Pathway	TF	NR	NT	Uniprot
DN128616_c10_g17_i8	UP	[GO:0015935:small ribosomal subunit][GO:0003735:structural constituent of ribosome][GO:0006412:translation]	-	FANhyb_rscf00000347.1.g00002.1:Fragaria x ananassa|FAR1|FAR1 family protein	KYP48943.1:hypothetical protein KK1_029344, partial [Cajanus cajan]	gi|372000942|gb|HQ621631.1|:Portulacaria afra voucher 995326/0 (ZSS) 16S ribosomal RNA (rrn16) gene, partial sequence; chloroplast	M1DBW2|M1DBW2_SOLTU:Uncharacterized protein;OS=Solanum tuberosum;|GO:0015935,|GO:0003735,|GO:0006412,
DN124687_c3_g7_i9	UP	[GO:0005886:plasma membrane][GO:0009506:plasmodesma][GO:0005524:ATP binding][GO:0004672:protein kinase activity]	-	mrna32207.1-v1.0-hybrid:Fragaria vesca|TALE|TALE family protein	XP_010678745.1:PREDICTED: serine/threonineprotein kinase HT1 [Beta vulgaris subsp. vulgaris] >KMT10562.1 hypothetical protein BVRB_5g116850 [Beta vulgaris subsp. vulgaris]	gi|356513266|ref|XM_003525287.1|:PREDICTED: Glycine max dual specificity protein kinase zakA-like (LOC100807921), mRNA	A0A0J8CAD9|A0A0J8CAD9_BETVU: Uncharacterized protein;OS=Beta vulgaris subsp. vulgaris.;|GO:0005886,GO:0009506,|GO:0005524,GO:0004672,

geneid	trend	GO	Pathway	TF	NR	NT	Uniprot
DN124165_c0_g1_i12	UP	[GO:0016021:integral component of membrane]	-	311286:Arabidopsis lyrata|MYB_related|MYB_related family protein	XP_010690888.1:PREDICTED: uncharacterized protein At1g01500 [Beta vulgaris subsp. vulgaris] >XP_019107551.1 PREDICTED: uncharacterized protein At1g01500 [Beta vulgaris subsp. vulgaris] >KMT00449.1 hypothetical protein BVRB_9g217500 [Beta vulgaris subsp. vulgaris]	gi|460414878|ref|XM_004252741.1|:PREDICTED: Solanum lycopersicum uncharacterized LOC101245741 (LOC101245741), mRNA	A0A0J8BK50|A0A0J8BK50_BETVU:Uncharacterized protein;OS=Beta vulgaris subsp. vulgaris.;|GO:0016021,
DN119582_c1_g5_i2	UP	[GO:0016021:integral component of membrane][GO:0005886:plasma membrane][GO:0008643:carbohydrate transport]	-	mrna24941.1-v1.0-hybrid:Fragaria vesca|DBB|DBB family protein	XP_021846236.1:bidirectional sugar transporter N3like [Spinacia oleracea] >KNA19087.1 hypothetical protein SOVF_064870 [Spinacia oleracea]	gi|356516514|ref|XM_003526891.1|:PREDICTED: Glycine max bidirectional sugar transporter SWEET12-like (LOC100810426), mRNA	A0A0K9RJN8|A0A0K9RJN8_SPIOL:Bidirectional sugar transporter SWEET;OS=Spinacia oleracea;|GO:0016021,GO:0005886,|GO

geneid	trend	GO	Pathway	TF	NR	NT	Uniprot
							:0008643,
DN126652_c1_g1_i1	UP	-	-	Bv3_060900_tcwq.t1:Beta vulgaris|bHLH|bHLH family protein	XP_010672733.1:PREDICTED: transcription factor bHLH48 [Beta vulgaris subsp. vulgaris] >KMT15386.1 hypothetical protein BVRB_3g060900 [Beta vulgaris subsp. vulgaris]	gi|521443494|gb|GAHZ01076990.1|:TSA: Salicornia europaea Unigene94021_SeCKS transcribed RNA sequence	A0A0J8FHK3|A0A0J8FHK3_BETVU:Uncharacterized protein;OS=Beta vulgaris subsp. vulgaris.;

geneid	trend	GO	Pathway	TF	NR	NT	Uniprot
DN125079_c2_g4_i6	UP	[GO:0005634:nucleus][GO:0004402:histone acetyltransferase activity][GO:0003712:transcription coregulator activity][GO:0008270:zinc ion binding][GO:0006355:regulation of transcription, DNA-templated]	-	PSME_00040545-RA:Pseudotsuga menziesii|G2-like|G2-like family protein	XP_021751426.1:BTB/POZ and TAZ domaincontaining protein 4like [Chenopodium quinoa] >XP_021751427.1 BTB/POZ and TAZ domaincontaining protein 4like [Chenopodium quinoa]	gi|359480558|ref|XM_003632444.1|:PREDICTED: Vitis vinifera BTB/POZ and TAZ domain-containing protein 4-like (LOC100855250), mRNA	A0A0J8CCZ8|A0A0J8CCZ8_BETVU:Uncharacterized protein;OS=Beta vulgaris subsp. vulgaris.;|GO:0005634,|GO:0004402,GO:0003712,GO:0008270,|GO:0006355,
DN121990_c1_g1_i4	UP	[GO:0009941:chloroplast envelope][GO:0016021:integral component of membrane][GO:0015095:magnesium ion transmembrane transporter activity]	-	-	XP_010689970.1:PREDICTED: magnesium transporter MRS211, chloroplastic [Beta vulgaris subsp. vulgaris] >KMT01794.1 hypothetical protein BVRB_9g210760 [Beta vulgaris subsp. vulgaris]	gi|521697822|gb|GAIA01018579.1|:TSA: Salicornia europaea Unigene19534_Se200S transcribed RNA sequence	A0A0J8BPC0|A0A0J8BPC0_BETVU:Uncharacterized protein;OS=Beta vulgaris subsp. vulgaris.;|GO:0009941,GO:0016021,|GO:0015095,

geneid	trend	GO	Pathway	TF	NR	NT	Uniprot
DN122990_c3_g2_i2	UP	[GO:0005524:ATP binding][GO:0016887:ATPase activity]	-	-	XP_010674157.1:PREDICTED: ABC transporter F family member 5 [Beta vulgaris subsp. vulgaris] >KMT13815.1 hypothetical protein BVRB_4g076610 [Beta vulgaris subsp. vulgaris]	gi|460383503|ref|XM_004237414.1|:PREDICTED: Solanum lycopersicum ABC transporter F family member 5-like (LOC101256375), mRNA	A0A0J8CJE7|A0A0J8CJE7_BETVU:Uncharacterized protein;OS=Beta vulgaris subsp. vulgaris.;|GO:0005524,GO:0016887,
DN127312_c4_g13_i2	UP	[GO:0005524:ATP binding]	-	-	OIW02691.1:hypothetical protein TanjilG_29467 [Lupinus angustifolius]	-	B9SDY7|B9SDY7_RICCO:Ribulose bisphosphate carboxylase/oxygenase activase 1, chloroplast, putative;OS=Ricinus communis;|GO:0005524,

geneid	trend	GO	Pathway	TF	NR	NT	Uniprot
DN118273_c0_g1_i2	UP	[GO:0006355:regulation of transcription, DNA-templated][GO:0006351:transcription, DNA-templated]	-	Sp_142310_fwnd.t1:Spinacia oleracea|GRAS|GRAS family protein	XP_021863662.1:scarecrowlike protein 30 [Spinacia oleracea] >KNA10642.1 hypothetical protein SOVF_142310 [Spinacia oleracea]	gi|521590623|gb|GAIA01067577.1|:TSA: Salicornia europaea Unigene81362_Se200S transcribed RNA sequence	A0A0K9QTP6|A0A0K9QTP6_SPIOL:Uncharacterized protein;OS=Spinacia oleracea;|GO:0006355,GO:0006351,
DN115340_c0_g1_i1	UP	-	-	-	KNA08805.1:hypothetical protein SOVF_159400, partial [Spinacia oleracea]	gi|502085698|ref|XM_004487922.1|:PREDICTED: Cicer arietinum oxysterol-binding protein-related protein 4B-like (LOC101506673), mRNA	A0A0K9QNI6|A0A0K9QNI6_SPIOL:Uncharacterized protein;OS=Spinacia oleracea;
DN128281_c3_g6_i2	UP	-	-	-	XP_021770274.1:uncharacterized protein LOC110734428 [Chenopodium quinoa]	gi|521490625|gb|GAHZ01040261.1|:TSA: Salicornia europaea Unigene45604_SeCKS transcribed RNA sequence	A0A0J8C9K2|A0A0J8C9K2_BETVU:Uncharacterized protein;OS=Beta vulgaris subsp. vulgaris.;

geneid	trend	GO	Pathway	TF	NR	NT	Uniprot
DN113964_c0_g2_i2	UP	[GO:0009507:chloroplast][GO:0005524:ATP binding][GO:0016851:magnesium chelatase activity][GO:0015995:chlorophyll biosynthetic process][GO:0015979:photosynthesis]	[ko00860:Porphyrin and chlorophyll metabolism]	DCAR_002090:Daucus carota|GRAS|GRAS family protein	XP_021766048.1:magnesiumchelatase subunit ChlI, chloroplasticlike [Chenopodium quinoa]	gi|359483587|ref|XM_002276226.2|:PREDICTED: Vitis vinifera magnesium-chelatase subunit chlI, chloroplastic-like (LOC100267459), mRNA	A0A0K9RL14|A0A0K9RL14_SPIOL:Mg-protoporphyrin IX chelatase;OS=Spinacia oleracea;|GO:0009507,|GO:0005524,GO:0016851,|GO:0015995,GO:0015979,
DN124025_c0_g1_i3	UP	-	-	-	-	-	-
DN118035_c0_g1_i2	UP	[GO:0048046:apoplast][GO:0016788:hydrolase activity, acting on ester bonds]	-	-	XP_010690130.1:PREDICTED: GDSL esterase/lipase APG [Beta vulgaris subsp. vulgaris] >KMT01744.1 hypothetical protein BVRB_9g211950 [Beta vulgaris subsp. vulgaris]	gi|388511470|gb|BT144003.1|:Medicago truncatula clone JCVI-FLMt-16P9 unknown mRNA	A0A0J8BP66|A0A0J8BP66_BETVU:Uncharacterized protein;OS=Beta vulgaris subsp. vulgaris.;|GO:0048046,|GO:0016788,

geneid	trend	GO	Pathway	TF	NR	NT	Uniprot
DN122228_c1_g2_i6	UP	[GO:0005525:GTP binding][GO:0003924:GTPase activity][GO:0003676:nucleic acid binding]	-	KZV46390.1:Dorcoceras hygrometricum|LBD|LBD family protein	XP_015898978.1:PREDICTED: GTPbinding protein TypA/BipA homolog [Ziziphus jujuba]	gi|162424767|gb|EU289224.1|:Suaeda salsa tyrosine phosphorylated protein A (TypA) mRNA, complete cds	W9R045|W9R045_9ROSA:GTP-binding protein TypA/BipA-like protein;OS=Morus notabilis.;|GO:0005525,GO:0003924,GO:0003676,
DN121858_c2_g2_i1	UP	[GO:0004392:heme oxygenase (decyclizing) activity][GO:0006788:heme oxidation]	[ko00860:Porphyrin and chlorophyll metabolism]	-	XP_021760807.1:heme oxygenase 1, chloroplasticlike [Chenopodium quinoa]	-	A0A0J8BS81|A0A0J8BS81_BETVU:Uncharacterized protein;OS=Beta vulgaris subsp. vulgaris.;|GO:0004392,|GO:0006788,

geneid	trend	GO	Pathway	TF	NR	NT	Uniprot
DN126617_c0_g2_i5	UP	[GO:0009535:chloroplast thylakoid membrane][GO:0016021:integral component of membrane][GO:0009522:photosystem I][GO:0009523:photosystem II][GO:0016168:chlorophyll binding][GO:0046872:metal ion binding][GO:0009765:photosynthesis, light harvesting][GO:0018298:protein-chromophore linkage]	[ko00196:Photosynthesis - antenna proteins]	Bradi3g42627.3.p:Brachypodium distachyon|ERF|ERF family protein	XP_010913989.1:PREDICTED: chlorophyll ab binding protein 8, chloroplastic [Elaeis guineensis]	gi|359489061|ref|XM_002273165.2|:PREDICTED: Vitis vinifera chlorophyll a/b binding protein (LHCA3), mRNA	A0A067L9S9|A0A067L9S9_JATCU:Chlorophyll a-b binding protein, chloroplastic;OS=Jatropha curcas;|GO:0009535,GO:0016021,GO:0009522,GO:0009523,|GO:0016168,GO:0046872,|GO:0009765,GO:0018298,
DN116367_c0_g3_i2	UP	-	-	-	XP_010675832.1:PREDICTED: mannose/glucosespecific lectinlike [Beta vulgaris subsp. vulgaris]	-	A0A0J8FAD3|A0A0J8FAD3_BETVU:Uncharacterized protein;OS=Beta vulgaris subsp. vulgaris.;

geneid	trend	GO	Pathway	TF	NR	NT	Uniprot
DN126841_c0_g2_i2	UP	[GO:0005737:cytoplasm][GO:0005524:ATP binding][GO:0042026:protein refolding]	[ko03018:RNA degradation][ko04212:Longevity regulating pathway - worm][ko04940:Type I diabetes mellitus][ko05134:Legionellosis][ko05152:Tuberculosis]	DCAR_026693:Daucus carota|bZIP|bZIP family protein	XP_021765315.1:ruBisCO large subunitbinding protein subunit beta, chloroplasticlike [Chenopodium quinoa]	gi|225442530|ref|XM_002284098.1|:PREDICTED: Vitis vinifera ruBisCO large subunit-binding protein subunit beta, chloroplastic-like (LOC100261815), mRNA	A0A0J8BIV0|A0A0J8BIV0_BETVU:Uncharacterized protein;OS=Beta vulgaris subsp. vulgaris.;|GO:0005737,|GO:0005524,|GO:0042026,
DN125791_c1_g9_i2	UP	-	-	-	-	-	-
DN118708_c2_g2_i2	UP	[GO:0046872:metal ion binding]	-	462924824:Eragrostis tef|GRAS|GRAS family protein	XP_009586618.1:PREDICTED: protein yippeelike At5g53940 [Nicotiana tomentosiformis] >XP_016509903.1 PREDICTED: protein yippeelike At5g53940 [Nicotiana tabacum] >XP_019256932.1 PREDICTED:	-	M1B8Q5|M1B8Q5_SOLTU:Protein yippee-like;OS=Solanum tuberosum;|GO:0046872,

geneid	trend	GO	Pathway	TF	NR	NT	Uniprot
					protein yippeelike At5g53940 [Nicotiana attenuata]		
DN120725_c1_g2_i1	UP	[GO:0000786:nucleosome][GO:0005634:nucleus][GO:0003677:DNA binding][GO:0006334:nucleosome assembly]	-	-	XP_021856526.1:uncharacterized protein LOC110795808 [Spinacia oleracea]	-	A0A0J8BGP0|A0A0J8BGP0_BETVU:Uncharacterized protein;OS=Beta vulgaris subsp. vulgaris.;|GO:0000786,GO:0005634,|GO:0003677,|GO:0006334,
DN117008_c0_g8_i1	UP	[GO:0048046:apoplast][GO:0009570:chloroplast stroma][GO:0009579:thylakoid][GO:0005507:copper ion binding]	-	-	XP_021739827.1:uncharacterized protein At5g02240like [Chenopodium quinoa]	-	A0A0J8CC05|A0A0J8CC05_BETVU:Uncharacterized protein;OS=Beta vulgaris subsp. vulgaris.;|GO:00480

<table>
<tr><th>geneid</th><th>trend</th><th>GO</th><th>Pathway</th><th>TF</th><th>NR</th><th>NT</th><th>Uniprot</th></tr>
<tr><td></td><td></td><td></td><td></td><td></td><td></td><td></td><td>46,GO:0009570,GO:0009579,|GO:0005507,</td></tr>
<tr><td>DN126828_c2_g1_i2</td><td>UP</td><td>[GO:0050660:flavin adenine dinucleotide binding][GO:0016614:oxidoreductase activity, acting on CH-OH group of donors]</td><td>-</td><td>CCG025416.1:Populus euphratica|G2-like|G2-like family protein</td><td>XP_010693456.1:PREDICTED: protein HOTHEAD [Beta vulgaris subsp. vulgaris] >KMS99012.1 hypothetical protein BVRB_3g066190 [Beta vulgaris subsp. vulgaris]</td><td>gi|460390239|ref|XM_004240698.1|:PREDICTED: Solanum lycopersicum protein HOTHEAD-like, transcript variant 2 (LOC101244002), mRNA</td><td>A0A0J8BG77|A0A0J8BG77_BETVU:Uncharacterized protein;OS=Beta vulgaris subsp. vulgaris.;|GO:0050660,GO:0016614,</td></tr>
<tr><td>DN115929_c1_g4_i11</td><td>UP</td><td>[GO:0004144:diacylglycerol O-acyltransferase activity][GO:0045017:glycerolipid biosynthetic process]</td><td>-</td><td>-</td><td>XP_010666700.1:PREDICTED: Oacyltransferase WSD1like isoform X1 [Beta vulgaris subsp. vulgaris]</td><td>-</td><td>A0A0J8D7E9|A0A0J8D7E9_BETVU:Uncharacterized protein;OS=Beta vulgaris subsp. vulgaris.;|GO:0004144,|GO:0045017,</td></tr>
</table>

geneid	trend	GO	Pathway	TF	NR	NT	Uniprot
DN119874_c4_g6_i2	UP	[GO:0009941:chloroplast envelope][GO:0009535:chloroplast thylakoid membrane][GO:0016021:integral component of membrane][GO:0009522:photosystem I][GO:0009523:photosystem II][GO:0010287:plastoglobule][GO:0016168:chlorophyll binding][GO:0009768:photosynthesis, light harvesting in photosystem I][GO:0018298:protein-chromophore linkage]	[ko00196:Photosynthesis - antenna proteins]	Bradi3g42627.3.p:Brachypodium distachyon|ERF|ERF family protein	XP_021771044.1:chlorophyll ab binding protein 6A, chloroplasticlike [Chenopodium quinoa]	gi|521750578|gb|GAIA01000859.1|:TSA: Salicornia europaea Unigene875_Se200S transcribed RNA sequence	A0A0J8BGF2|A0A0J8BGF2_BETVU:Chlorophyll a-b binding protein, chloroplastic;OS=Beta vulgaris subsp. vulgaris.;|GO:0009941,GO:0009535,GO:0016021,GO:0009522,GO:0009523,GO:0010287,|GO:0016168,|GO:0009768,GO:0018298,
DN127818_c1_g1_i4	UP	-	-	-	XP_009357505.1:PREDICTED: plastidlipidassociated protein, chloroplasticlike [Pyrus x bretschneideri]	-	A0A0K9QBN7|A0A0K9QBN7_SPIOL: Uncharacterized protein;OS=Spinacia oleracea;

geneid	trend	GO	Pathway	TF	NR	NT	Uniprot
DN124165_c0_g1_i8	UP	[GO:0016021:integral component of membrane]	-	311286:Arabidopsis lyrata|MYB_related|MYB_related family protein	XP_010690888.1:PREDICTED: uncharacterized protein At1g01500 [Beta vulgaris subsp. vulgaris] >XP_019107551.1 PREDICTED: uncharacterized protein At1g01500 [Beta vulgaris subsp. vulgaris] >KMT00449.1 hypothetical protein BVRB_9g217500 [Beta vulgaris subsp. vulgaris]	gi|460414878|ref|XM_004252741.1|:PREDICTED: Solanum lycopersicum uncharacterized LOC101245741 (LOC101245741), mRNA	A0A0J8BK50|A0A0J8BK50_BETVU:Uncharacterized protein;OS=Beta vulgaris subsp. vulgaris.;|GO:0016021,

geneid	trend	GO	Pathway	TF	NR	NT	Uniprot
DN126651_c3_g5_i6	UP	[GO:0009507:chloroplast][GO:0015035:protein disulfide oxidoreductase activity][GO:0045454:cell redox homeostasis][GO:0006662:glycerol ether metabolic process]	-	AA81G00050:Aethionema arabicum|HRT-like|HRT-like family protein	XP_010678488.1:PREDICTED: thioredoxinlike 12, chloroplastic [Beta vulgaris subsp. vulgaris] >KMT10788.1 hypothetical protein BVRB_5g114440 [Beta vulgaris subsp. vulgaris]	-	A0A0J8CFJ7|A0A0J8CFJ7_BETVU:Uncharacterized protein;OS=Beta vulgaris subsp. vulgaris.;|GO:0009507,|GO:0015035,|GO:0045454,GO:0006662,
DN120171_c0_g15_i1	UP	[GO:0009507:chloroplast][GO:0004497:monooxygenase activity][GO:0016984:ribulose-bisphosphate carboxylase activity][GO:0009853:photorespiration][GO:0019253:reductive pentose-phosphate cycle]	[ko00630:Glyoxylate and dicarboxylate metabolism][ko00710:Carbon fixation in photosynthetic organisms]	-	XP_021769982.1:ribulose bisphosphate carboxylase small chain 3, chloroplasticlike [Chenopodium quinoa]	gi|21570|emb|X69763.1|:S.tuberosum rbcS3 gene for ribulose-(1,5)-bisphosphate carboxylase/oxygenase small subunit	P04714|RBS1_PETHY:Ribulose bisphosphate carboxylase small chain SSU8, chloroplastic;OS=Petunia hybrida;|GO:0009507,|GO:0004497,GO:0016984,|GO:0009853,GO:0019253,

geneid	trend	GO	Pathway	TF	NR	NT	Uniprot
DN119176_c4_g3_i2	UP	[GO:0005634:nucleus][GO:0003677:DNA binding][GO:0003700:DNA-binding transcription factor activity][GO:0006351:transcription, DNA-templated]	-	Bv_006850_gcqz.t1: Beta vulgaris|ERF|ERF family protein	XP_010667125.1:PREDICTED: ethyleneresponsive transcription factor RAP23 [Beta vulgaris subsp. vulgaris] >KMS95596.1 hypothetical protein BVRB_006850 [Beta vulgaris subsp. vulgaris]	gi|449532514|ref|XM_004173178.1|:PREDICTED: Cucumis sativus ethylene-responsive transcription factor RAP2-3-like (LOC101225200), mRNA	A0A0J8B3N9|A0A0J8B3N9_BETVU:Uncharacterized protein;OS=Beta vulgaris subsp. vulgaris.;|GO:0005634,|GO:0003677,GO:0003700,|GO:0006351,

geneid	trend	GO	Pathway	TF	NR	NT	Uniprot
DN118868_c0_g6_i1	UP	[GO:0005524:ATP binding]	[ko03040:Spliceosome][ko04010:MAPK signaling pathway][ko04141:Protein processing in endoplasmic reticulum][ko04144:Endocytosis][ko04213:Longevity regulating pathway - multiple species][ko04612:Antigen processing and presentation][ko04915:Estrogen signaling pathway][ko05134:Legionellosis][ko05145:Toxoplasmosis][ko05162:Measles][ko05164:Influenza A][ko05169:Epstein-Barr virus infection]	FANhyb_rscf00000233.1.g00002.1:Fragaria x ananassa|GATA|GATA family protein	XP_020242501.1:heat shock cognate 70 kDa protein 2like [Asparagus officinalis]	gi|392465166|dbj|AB689673.1|:Nicotiana tabacum NtHsp70 mRNA for Heat shock protein 70, complete cds	A0A0A0KQW6|A0A0A0KQW6_CUCSA:Uncharacterized protein;OS=Cucumis sativus;|GO:0005524,

geneid	trend	GO	Pathway	TF	NR	NT	Uniprot
DN119234_c0_g1_i2	UP	-	-	-	XP_021843559.1:B2 protein [Spinacia oleracea] >KNA18355.1 hypothetical protein SOVF_071680 [Spinacia oleracea]	gi|521738675|gb|GAIA01007463.1|:TSA: Salicornia europaea Unigene7778_Se200S transcribed RNA sequence	A0A0K9RFU4|A0A0K9RFU4_SPIOL:Uncharacterized protein;OS=Spinacia oleracea;
DN115533_c1_g1_i1	UP	[GO:0003677:DNA binding]	-	AHYPO_015608-RA:Amaranthus hypochondriacus|ZF-HD|ZF-HD family protein	XP_011085755.1:zincfinger homeodomain protein 9 [Sesamum indicum]	gi|357501808|ref|XM_003621145.1|:Medicago truncatula ZF-HD homeobox protein (MTR_7g010300) mRNA, complete cds	A0A0J8BEA3|A0A0J8BEA3_BETVU:Uncharacterized protein;OS=Beta vulgaris subsp. vulgaris.;|GO:0003677,
DN122228_c1_g2_i2	UP	[GO:0005525:GTP binding][GO:0003924:GTPase activity][GO:0003676:nucleic acid binding]	-	KZV46390.1:Dorcoceras hygrometricum|LBD|LBD family protein	XP_021740971.1:putative elongation factor TypAlike SVR3, chloroplastic [Chenopodium quinoa]	gi|162424767|gb|EU289224.1|:Suaeda salsa tyrosine phosphorylated protein A (TypA) mRNA, complete cds	W9R045|W9R045_9ROSA:GTP-binding protein TypA/BipA-like protein;OS=Morus notabilis.;|GO:0005525,GO:0003924,GO:0003676,

geneid	trend	GO	Pathway	TF	NR	NT	Uniprot
DN126362_c3_g1_i5	UP	[GO:0009535:chloroplast thylakoid membrane][GO:0016021:integral component of membrane][GO:0009522:photosystem I][GO:0009523:photosystem II][GO:0016168:chlorophyll binding][GO:0046872:metal ion binding][GO:0009765:photosynthesis, light harvesting][GO:0018298:protein-chromophore linkage]	[ko00196:Photosynthesis - antenna proteins]	Bradi3g42627.3.p:Brachypodium distachyon|ERF|ERF family protein	XP_010678492.1:PREDICTED: chlorophyll ab binding protein 4, chloroplastic [Beta vulgaris subsp. vulgaris] >CAE30280.1 chlorophyll a /b binding protein [Beta vulgaris] >KMT10798.1 hypothetical protein BVRB_5g114540 [Beta vulgaris subsp. vulgaris]	gi|33504459|emb|AJ579711.2|:Beta vulgaris mRNA for chloroplast chlorophyll a/b binding protein (cab11 gene)	Q7XAC6|Q7XAC6_BETVU:Chlorophyll a-b binding protein, chloroplastic;OS=Beta vulgaris;|GO:0009535,GO:0016021,GO:0009522,GO:0009523,|GO:0016168,GO:0046872,|GO:0009765,GO:0018298,
DN124420_c0_g3_i1	UP	[GO:0102132:3-oxo-pimeloyl-[acp] methyl ester reductase activity][GO:0004316:3-oxoacyl-[acyl-carrier-protein] reductase (NADPH) activity]	[ko00906:Carotenoid biosynthesis]	Cc01_g08720:Coffea canephora|NAC|NAC family protein	XP_021675230.1:xanthoxin dehydrogenaselike [Hevea brasiliensis]	-	B9SMW7|B9SMW7_RICCO:Short chain alcohol dehydrogenase, putative;OS=Ricinus communis;|GO:0102132,GO:0004316,
DN122748_c2_g6_i1	UP	-	-	-	-	-	-

geneid	trend	GO	Pathway	TF	NR	NT	Uniprot
DN121436_c1_g1_i1	UP	-	-	-	XP_010670216.1:PREDICTED: jasmonateinduced protein homolog [Beta vulgaris subsp. vulgaris]	-	A0A0J8CU80|A0A0J8CU80_BETVU:Uncharacterized protein;OS=Beta vulgaris subsp. vulgaris.;
DN120762_c6_g3_i6	UP	-	-	-	-	-	-
DN122593_c1_g6_i3	UP	[GO:0016765:transferase activity, transferring alkyl or aryl (other than methyl) groups]	[ko00900:Terpenoid backbone biosynthesis]	-	XP_010685566.1:PREDICTED: dehydrodolichyl diphosphate synthase 2 [Beta vulgaris subsp. vulgaris] >KMT05070.1 hypothetical protein BVRB_7g172260 [Beta vulgaris subsp. vulgaris]	-	A0A0J8BU46|A0A0J8BU46_BETVU:Alkyl transferase;OS=Beta vulgaris subsp. vulgaris.;|GO:0016765,

geneid	trend	GO	Pathway	TF	NR	NT	Uniprot
DN126982_c2_g1_i2	UP	[GO:0003984:acetolactate synthase activity][GO:0050660:flavin adenine dinucleotide binding][GO:0000287:magnesium ion binding][GO:0030976:thiamine pyrophosphate binding][GO:0009097:isoleucine biosynthetic process][GO:0009099:valine biosynthetic process]	[ko00290:Valine, leucine and isoleucine biosynthesis][ko00650:Butanoate metabolism][ko00660:C5-Branched dibasic acid metabolism][ko00770:Pantothenate and CoA biosynthesis]	-	ABM53021.1:acetolactate synthase [Amaranthus tuberculatus]	gi|312233975|gb|GU271215.1|:Salsola tragus biotype Reford_11 acetolactate synthase (ALS) gene, partial cds	A1Z0Z0|A1Z0Z0_AMATU:Acetolactate synthase;OS=Amaranthus tuberculatus;|GO:0003984,GO:0050660,GO:0000287,GO:0030976,|GO:0009097,GO:0009099,
DN122734_c1_g2_i2	UP	[GO:0003824:catalytic activity]	-	Medtr7g113010.1:Medicago truncatula|FAR1|FAR1 family protein	XP_021770817.1:uncharacterized protein LOC110735006 [Chenopodium quinoa]	gi|32970581|dbj|AK060563.1|:Oryza sativa Japonica Group cDNA clone:001-022-G09, full insert sequence	A0A0J8E710|A0A0J8E710_BETVU:Uncharacterized protein;OS=Beta vulgaris subsp. vulgaris.;|GO:0003824,

geneid	trend	GO	Pathway	TF	NR	NT	Uniprot
DN126838_c0_g1_i2	UP	[GO:0015930:glutamate synthase activity][GO:0006537:glutamate biosynthetic process]	[ko00630:Glyoxylate and dicarboxylate metabolism][ko00910:Nitrogen metabolism]	448225:Selaginella moellendorffii|C3H|C3H family protein	XP_010673008.1:PREDICTED: ferredoxindependent glutamate synthase, chloroplastic [Beta vulgaris subsp. vulgaris] >KMT15173.1 hypothetical protein BVRB_3g062860 [Beta vulgaris subsp. vulgaris]	gi|397172238|gb|JN967620.1|:Beta vulgaris subsp. vulgaris glutamate synthase mRNA, complete cds	A0A0J8CT99|A0A0J8CT99_BETVU:Uncharacterized protein;OS=Beta vulgaris subsp. vulgaris.;|GO:0015930,|GO:0006537,
DN126125_c0_g1_i1	UP	[GO:0009941:chloroplast envelope][GO:0009570:chloroplast stroma]	-	-	XP_010677196.1:PREDICTED: DNAbinding protein SMUBP2 [Beta vulgaris subsp. vulgaris] >KMT19936.1 hypothetical protein BVRB_1g009830 [Beta vulgaris subsp. vulgaris]	gi|460379048|ref|XM_004235229.1|:PREDICTED: Solanum lycopersicum DNA-binding protein SMUBP-2-like (LOC101246606), mRNA	A0A0J8FW82|A0A0J8FW82_BETVU:Uncharacterized protein;OS=Beta vulgaris subsp. vulgaris.;|GO:0009941,GO:0009570,

geneid	trend	GO	Pathway	TF	NR	NT	Uniprot
DN122733_c3_g2_i14	UP	[GO:0048046:apoplast][GO:0009941:chloroplast envelope][GO:0009570:chloroplast stroma][GO:0010319:stromule][GO:0009579:thylakoid][GO:0016857:racemase and epimerase activity, acting on carbohydrates and derivatives][GO:0005975:carbohydrate metabolic process][GO:0009624:response to nematode]	[ko00030:Pentose phosphate pathway][ko00040:Pentose and glucuronate interconversions][ko00710:Carbon fixation in photosynthetic organisms]	-	XP_010678478.1:PREDICTED: ribulosephosphate 3epimerase, chloroplastic [Beta vulgaris subsp. vulgaris] >KMT10779.1 hypothetical protein BVRB_5g114350 [Beta vulgaris subsp. vulgaris]	gi|512399395|gb|GAJZ01001775.1|:TSA: Prunus avium Pa_03473 transcribed RNA sequence	A0A0J8CF54|A0A0J8CF54_BETVU:Uncharacterized protein;OS=Beta vulgaris subsp. vulgaris.;|GO:0048046,GO:0009941,GO:0009570,GO:0010319,GO:0009579,|GO:0016857,|GO:0005975,GO:0009624,
DN118374_c0_g1_i2	UP	-	-	-	XP_021723093.1:Fbox only protein 6like [Chenopodium quinoa]	gi|521637193|gb|GAIA01048064.1|:TSA: Salicornia europaea Unigene56298_Se200S transcribed RNA sequence	A0A0J8C1U9|A0A0J8C1U9_BETVU:Uncharacterized protein;OS=Beta vulgaris subsp. vulgaris.;
DN119349_c0_g1_i5	UP	[GO:0004722:protein serine/threonine phosphatase activity]	-	KZV22762.1:Dorcoceras hygrometricum|CAMTA|CAMTA family protein	AAC36699.1:protein phosphatase2C [Mesembryanthemum crystallinum]	gi|3643087|gb|AF075581.1|AF075581:Mesembryanthemum crystallinum clone Mpc6 protein phosphatase-2C	O82470|O82470_MESCR:Protein phosphatase-2C;OS=Mesembryanthemum crystallinum;|GO:00

geneid	trend	GO	Pathway	TF	NR	NT	Uniprot
						(PP2C) mRNA, complete cds	04722,
DN128034_c 1_g2_i1	UP	[GO:0009507:chloroplast][GO:0015035:protein disulfide oxidoreductase activity][GO:0045454:cell redox homeostasis][GO:0006662:glycerol ether metabolic process]	[ko04621:NOD-like receptor signaling pathway][ko05418:Fluid shear stress and atherosclerosis]	DCAR_019739:Daucus carota|C3H|C3H family protein	XP_021841800.1:thioredoxin Mtype, chloroplastic [Spinacia oleracea] >P07591.2 RecName: Full=Thioredoxin Mtype, chloroplastic; Short=TrxM; Contains: RecName: Full=Thioredoxin Mtype Mc; Contains: RecName: Full=Thioredoxin Mtype Md; Flags: Precursor >CAA35826.1 unnamed protein	-	P07591|TRXM_SPIOL:Thioredoxin M-type, chloroplastic;OS=Spinacia oleracea;|GO:0009507,|GO:0015035,|GO:0045454,GO:0006662,

geneid	trend	GO	Pathway	TF	NR	NT	Uniprot
					product [Spinacia oleracea]		
DN114644_c0_g1_i2	UP	[GO:0009570:chloroplast stroma][GO:0009535:chloroplast thylakoid membrane][GO:0010275:NAD(P)H dehydrogenase complex assembly]	-	-	XP_010687776.1:PREDICTED: protein CHLORORESPIRATORY REDUCTION 6, chloroplastic [Beta vulgaris subsp. vulgaris] >KMT03612.1 hypothetical protein	gi|521746267|gb|GAIA01002829.1|:TSA: Salicornia europaea Unigene2961_Se200S transcribed RNA sequence	A0A0J8BQZ8|A0A0J8BQZ8_BETVU:Uncharacterized protein;OS=Beta vulgaris subsp. vulgaris.;|GO:0009570,GO:0009535,|GO:0010275,

geneid	trend	GO	Pathway	TF	NR	NT	Uniprot
					BVRB_8g192850 [Beta vulgaris subsp. vulgaris]		
DN110339_c0_g1_i1	UP	[GO:0008270:zinc ion binding]	-	-	XP_021732914.1:putative RINGH2 finger protein ATL71 [Chenopodium quinoa]	-	A0A0J8BLH7|A0A0J8BLH7_BETVU:Uncharacterized protein;OS=Beta vulgaris subsp. vulgaris.;|GO:0008270,
DN118627_c1_g1_i13	UP	-	-	Tp57577_TGAC_v2_mRNA10528:Trifolium pratense|MYB_related|MYB_related family protein	XP_021763531.1:ultravioletB receptor UVR8like [Chenopodium quinoa]	gi|90657533|gb|DQ415920.1|:Cleome spinosa clone BAC Cs3, complete sequence	A0A0J8CXT4|A0A0J8CXT4_BETVU:Uncharacterized protein;OS=Beta vulgaris subsp. vulgaris.;

geneid	trend	GO	Pathway	TF	NR	NT	Uniprot
DN128196_c2_g2_i1	UP	[GO:0016829:lyase activity][GO:0006520:cellular amino acid metabolic process]	[ko00260:Glycine, serine and threonine metabolism]	Gh_A11G2240:Gossypium hirsutum|MYB|MYB family protein	XP_010694951.1:PREDICTED: probable lowspecificity Lthreonine aldolase 1 [Beta vulgaris subsp. vulgaris] >XP_010694952.1 PREDICTED: probable lowspecificity Lthreonine aldolase 1 [Beta vulgaris subsp. vulgaris] >XP_019108198.1 PREDICTED: probable lowspecificity Lthreonine aldolase 1 [Beta vulgaris subsp. vulgaris] >KMS98028.1 hypothetical protein BVRB_4g096540 [Beta vulgaris subsp.	gi|359488056|ref|XM_003633648.1|:PREDICTED: Vitis vinifera L-allo-threonine aldolase-like, transcript variant 3 (LOC100265578), mRNA	A0A0J8BDA5|A0A0J8BDA5_BETVU: Uncharacterized protein;OS=Beta vulgaris subsp. vulgaris.;|GO:0016829,|GO:0006520,

geneid	trend	GO	Pathway	TF	NR	NT	Uniprot
					vulgaris]		
DN127279_c1_g1_i1	UP	-	-	-	XP_021753251.1:uncharacterized protein LOC110718675 [Chenopodium quinoa]	gi|449531690|ref|XM_004172771.1|:PREDICTED: Cucumis sativus uncharacterized LOC101221421 (LOC101221421),	A0A0J8BJJ4|A0A0J8BJJ4_BETVU:Uncharacterized protein;OS=Beta vulgaris subsp. vulgaris.;

geneid	trend	GO	Pathway	TF	NR	NT	Uniprot
						partial mRNA	
DN127777_c2_g1_i8	UP	[GO:0016491:oxidoreductase activity]	-	-	KNA22191.1:hypothetical protein SOVF_036430 [Spinacia oleracea]	gi|356514367|ref|XM_003525830.1|:PREDICTED: Glycine max uncharacterized protein LOC100791851 (LOC100791851), mRNA	A0A0K9RRM1|A0A0K9RRM1_SPIOL:Uncharacterized protein;OS=Spinacia oleracea;|GO:0016491,
DN109715_c1_g1_i1	UP	[GO:0005840:ribosome][GO:0003735:structural constituent of ribosome][GO:0006412:translation]	[ko03010:Ribosome]	Araip.FQ8DT:Arachis ipaensis|SBP|SBP family protein	XP_010689247.1:PREDICTED: 50S ribosomal protein L9, chloroplastic [Beta vulgaris subsp. vulgaris] >KMT02272.1 hypothetical protein BVRB_9g206220 [Beta vulgaris subsp. vulgaris]	gi|521699774|gb|GAIA01017541.1|:TSA: Salicornia europaea Unigene18461_Se200S transcribed RNA sequence	A0A0J8BM33|A0A0J8BM33_BETVU: Uncharacterized protein;OS=Beta vulgaris subsp. vulgaris.;|GO:0005840,|GO:0003735,|GO:0006412,

geneid	trend	GO	Pathway	TF	NR	NT	Uniprot
DN113095_c0_g1_i1	UP	-	-	-	XP_020215778.1:hemebindinglike protein At3g10130, chloroplastic [Cajanus cajan]	gi|148537546|dbj|AK246312.1|:Solanum lycopersicum cDNA, clone: FC06CD10, HTC in fruit	G7JRA5|G7JRA5_MEDTR:SOUL heme-binding family protein;OS=Medicago truncatula;GN=MTR_4g019150 {ECO:0000313|EMBL:AES87018.1};
DN116952_c2_g1_i3	UP	[GO:0016021:integral component of membrane][GO:0020037:heme binding][GO:0005506:iron ion binding][GO:0004497:monooxygenase activity][GO:0016705:oxidoreductase activity, acting on paired donors, with incorporation or reduction of molecular oxygen]	-	Bradi2g44117.7.p:Brachypodium distachyon|bHLH|bHLH family protein	XP_021733659.1:cytochrome P450 CYP72A219like isoform X2 [Chenopodium quinoa]	-	A0A0K9R1T0|A0A0K9R1T0_SPIOL:Uncharacterized protein;OS=Spinacia oleracea;|GO:0016021,|GO:0020037,GO:0005506,GO:0004497,GO:0016705,

geneid	trend	GO	Pathway	TF	NR	NT	Uniprot
DN105718_c0_g1_i3	UP	[GO:0009522:photosystem I][GO:0016168:chlorophyll binding][GO:0015979:photosynthesis]	[ko00195:Photosynthesis]	-	XP_010686093.1:PREDICTED: photosystem I reaction center subunit psaK, chloroplastic [Beta vulgaris subsp. vulgaris] >KMT05554.1 hypothetical protein BVRB_7g176760 [Beta vulgaris subsp. vulgaris]	gi|521707235|gb|GAIA01014474.1|:TSA: Salicornia europaea Unigene15133_Se200S transcribed RNA sequence	A0A0J8BZU6|A0A0J8BZU6_BETVU:Uncharacterized protein;OS=Beta vulgaris subsp. vulgaris.;|GO:0009522,|GO:0016168,|GO:0015979,
DN122988_c1_g1_i13	UP	[GO:0005524:ATP binding][GO:0004672:protein kinase activity]	-	KN539740.1_FGP005:Oryza longistaminata|MYB_related|MYB_related family protein	XP_021751741.1:uncharacterized protein LOC110717376 isoform X1 [Chenopodium quinoa] >XP_021751743.1 uncharacterized protein LOC110717376 isoform X1 [Chenopodium	gi|521635870|gb|GAIA01048667.1|:TSA: Salicornia europaea Unigene56959_Se200S transcribed RNA sequence	A0A0K9RLV9|A0A0K9RLV9_SPIOL: Uncharacterized protein;OS=Spinacia oleracea;|GO:0005524,GO:0004672,

geneid	trend	GO	Pathway	TF	NR	NT	Uniprot
					quinoa] >XP_021751744.1 uncharacterized protein LOC110717376 isoform X1 [Chenopodium quinoa]		
DN124231_c0_g2_i2	UP	-	-	-	XP_021634516.1:EG45like domain containing protein [Manihot esculenta] >OAY31863.1 hypothetical protein MANES_14G147000 [Manihot esculenta]	-	A0A0K9RBG4|A0A0K9RBG4_SPIOL: Uncharacterized protein;OS=Spinacia oleracea;

geneid	trend	GO	Pathway	TF	NR	NT	Uniprot
DN127161_c3_g1_i7	UP	-	-	Tp1g05870:Thellungiella parvula|MYB_related|MYB_related family protein	XP_021721284.1:protein CHUP1, chloroplasticlike [Chenopodium quinoa] >XP_021721285.1 protein CHUP1, chloroplasticlike [Chenopodium quinoa]	gi|359472708|ref|XM_002281118.2|:PREDICTED: Vitis vinifera protein CHUP1, chloroplastic-like (LOC100259307), mRNA	A0A0J8CDG4|A0A0J8CDG4_BETVU: Uncharacterized protein;OS=Beta vulgaris subsp. vulgaris.;
DN126362_c3_g2_i1	UP	[GO:0009535:chloroplast thylakoid membrane][GO:0016021:integral component of membrane][GO:0009522:photosystem I][GO:0009523:photosystem II][GO:0016168:chlorophyll binding][GO:0046872:metal ion binding][GO:0009765:photosynthesis, light harvesting][GO:0018298:protein-chromophore linkage]	[ko00196:Photosynthesis - antenna proteins]	Bradi3g42627.3.p:Brachypodium distachyon|ERF|ERF family protein	XP_008453211.1:PREDICTED: chlorophyll ab binding protein P4, chloroplastic [Cucumis melo]	gi|169930143|gb|EU401724.1|:Capsicum annuum chloroplast chlorophyll a/b binding protein mRNA, complete cds; nuclear gene for chloroplast product	A0A0A0LSB4|A0A0A0LSB4_CUCSA: Chlorophyll a-b binding protein, chloroplastic;OS=Cucumis sativus;|GO:0009535,GO:0016021,GO:0009522,GO:0009523,|GO:0016168,GO:0046872,|GO:0009765,GO:0018298,

geneid	trend	GO	Pathway	TF	NR	NT	Uniprot
DN128351_c5_g5_i2	UP	[GO:0009535:chloroplast thylakoid membrane][GO:0016021:integral component of membrane][GO:0009522:photosystem I][GO:0009523:photosystem II][GO:0016168:chlorophyll binding][GO:0046872:metal ion binding][GO:0009765:photosynthesis, light harvesting][GO:0018298:protein-chromophore linkage]	[ko00196:Photosynthesis - antenna proteins]	Peaxi162Scf00920g00027.1:Petunia axillaris|M-type_MADS|M-type_MADS family protein	NP_001312267.1:chlorophyll ab binding protein CP26, chloroplastic [Nicotiana tabacum] >XP_009616313.1 PREDICTED: chlorophyll ab binding protein CP26, chloroplastic [Nicotiana tomentosiformis] >ABG73417.1 chloroplast pigmentbinding protein CP26 [Nicotiana tabacum]	gi|521742345|gb|GAIA01004996.1|:TSA: Salicornia europaea Unigene5283_Se200S transcribed RNA sequence	Unreviewed|Q0PWS5_TOBAC:Chlorophyll a-b binding protein, chloroplastic;OS=Nicotiana tabacum;|GO:0009535,GO:0016021,GO:0009522,GO:0009523,|GO:0016168,GO:0046872,|GO:0009765,GO:0018298,
DN124845_c2_g2_i9	UP	[GO:0016021:integral component of membrane][GO:0019706:protein-cysteine S-palmitoyltransferase activity][GO:0008270:zinc ion binding]	-	-	XP_021845170.1:probable protein Sacyltransferase 22 [Spinacia oleracea] >KNA19875.1 hypothetical protein SOVF_057410 [Spinacia oleracea]	gi|521441415|gb|GAHZ01079069.1|:TSA: Salicornia europaea Unigene96757_SeCKS transcribed RNA sequence	A0A0K9RK53|A0A0K9RK53_SPIOL:S-acyltransferase;OS=Spinacia oleracea;|GO:0016021,|GO:0019706,GO:0008270,

geneid	trend	GO	Pathway	TF	NR	NT	Uniprot
DN118160_c5_g1_i2	UP	[GO:0009538:photosystem I reaction center][GO:0015979:photosynthesis]	[ko00195:Photosynthesis]	-	XP_011024524.1:PREDICTED: photosystem I reaction center subunit IV A, chloroplasticlike [Populus euphratica]	gi|157678947|dbj|AB002089.1|:Citrus sinensis psaE1 mRNA for PS1 reaction center subunit III, partial cds, clone: NE510	K4CU43|K4CU43_SOLLC:Uncharacterized protein;OS=Solanum lycopersicum;|GO:0009538,|GO:0015979,
DN119222_c2_g1_i3	UP	-	-	-	GAU16460.1:hypothetical protein TSUD_166920 [Trifolium subterraneum]	-	A0A0J8BZ67|A0A0J8BZ67_BETVU:Uncharacterized protein;OS=Beta vulgaris subsp. vulgaris.;
DN123468_c5_g6_i1	UP	[GO:0019898:extrinsic component of membrane][GO:0009654:photosystem II oxygen evolving complex][GO:0005509:calcium ion binding][GO:0015979:photosynthesis]	[ko00195:Photosynthesis]	Pp3c2_23590V3.6.p:Physcomitrella patens|WRKY|WRKY family protein	XP_009407699.1:PREDICTED: oxygenevolving enhancer protein 2, chloroplasticlike [Musa acuminata subsp. malaccensis]	-	W9RF48|W9RF48_9ROSA:Oxygen-evolving enhancer protein 2;OS=Morus notabilis.;|GO:0019898,GO:0009654,|GO:0005509,|GO:0015979,

geneid	trend	GO	Pathway	TF	NR	NT	Uniprot
DN125857_c5_g11_i1	UP	-	-	-	KNA16295.1:hypothetical protein SOVF_090490 [Spinacia oleracea]	-	A0A0K9R9Y6|A0A0K9R9Y6_SPIOL:Uncharacterized protein;OS=Spinacia oleracea;
DN128790_c5_g7_i4	UP	-	-	-	XP_021748000.1:thiosulfate sulfurtransferase 16, chloroplasticlike [Chenopodium quinoa]	-	A0A0K9RI51|A0A0K9RI51_SPIOL:Uncharacterized protein;OS=Spinacia oleracea;
DN113123_c0_g1_i1	UP	[GO:0016021:integral component of membrane]	-	-	XP_021768746.1:uncharacterized protein LOC110733038 [Chenopodium quinoa]	gi|521525084|gb|GAHZ01011359.1|:TSA: Salicornia europaea Unigene11976_SeCKS transcribed RNA sequence	A0A0J8F2R5|A0A0J8F2R5_BETVU:Uncharacterized protein;OS=Beta vulgaris subsp. vulgaris.;|GO:0016021,

geneid	trend	GO	Pathway	TF	NR	NT	Uniprot
DN121349_c1_g5_i1	UP	[GO:0009941:chloroplast envelope][GO:0009570:chloroplast stroma][GO:0005739:mitochondrion][GO:0005634:nucleus]	-	PSME_00016918-RA:Pseudotsuga menziesii|NAC|NAC family protein	XP_010681289.1:PREDICTED: uncharacterized protein LOC104896271 [Beta vulgaris subsp. vulgaris] >KMT08470.1 hypothetical protein BVRB_6g140250 [Beta vulgaris subsp. vulgaris]	-	A0A0J8C3V6|A0A0J8C3V6_BETVU:Uncharacterized protein;OS=Beta vulgaris subsp. vulgaris.;|GO:0009941,GO:0009570,GO:0005739,GO:0005634,

geneid	trend	GO	Pathway	TF	NR	NT	Uniprot
DN123296_c2_g1_i11	UP	[GO:0005634:nucleus][GO:0000977:RNA polymerase II regulatory region sequence-specific DNA binding][GO:0003700:DNA-binding transcription factor activity][GO:0000165:MAPK cascade][GO:0045944:positive regulation of transcription by RNA polymerase II][GO:0006351:transcription, DNA-templated]	-	Bv6_150530_jqqr.t3:Beta vulgaris|MIKC_MADS|MIKC_MADS family protein	ABC55432.1:FLClike 1 splice variant 4 [Beta vulgaris subsp. maritima]	-	A2CHK0|A2CHK0_BETVM:FLC-like 1 splice variant 4;OS=Beta vulgaris subsp. maritima;|GO:0005634,|GO:0000977,GO:0003700,|GO:0000165,GO:0045944,GO:0006351,
DN122009_c1_g1_i9	UP	[GO:0016021:integral component of membrane][GO:0015299:solute:proton antiporter activity][GO:0006813:potassium ion transport]	-	-	XP_010275512.1:PREDICTED: K(+) efflux antiporter 3, chloroplastic [Nelumbo nucifera]	gi|224105838|ref|XM_002313914.1|:Populus trichocarpa potassium efflux antiporter, mRNA	A0A0K9QQA9|A0A0K9QQA9_SPIOL:Uncharacterized protein;OS=Spinacia oleracea;|GO:0016021,|GO:0015299,|GO:0006813,

geneid	trend	GO	Pathway	TF	NR	NT	Uniprot
DN126251_c2_g5_i10	UP	[GO:0009507:chloroplast][GO:0005739:mitochondrion][GO:0004813:alanine-tRNA ligase activity][GO:0005524:ATP binding][GO:0000049:tRNA binding][GO:0008270:zinc ion binding][GO:0006419:alanyl-tRNA aminoacylation]	[ko00970:Aminoacyl-tRNA biosynthesis]	-	XP_021848519.1:alaninetRNA ligase, chloroplastic/mitochondrial isoform X1 [Spinacia oleracea] >KNA14563.1 hypothetical protein SOVF_106470 [Spinacia oleracea]	gi|359474806|ref|XM_002278915.2|:PREDICTED: Vitis vinifera probable alanyl-tRNA synthetase, chloroplastic-like (LOC100255425), mRNA	A0A0K9R4X5|A0A0K9R4X5_SPIOL:Probable alanine--tRNA ligase, chloroplastic;OS=Spinacia oleracea;|GO:0009507,GO:0005739,|GO:0004813,GO:0005524,GO:0000049,GO:0008270,|GO:0006419,
DN112726_c1_g4_i1	UP	-	-	-	-	-	-
DN121935_c3_g2_i2	UP	-	-	-	XP_021741383.1:deltalatroinsectotoxinLt1alike [Chenopodium quinoa]	gi|521747487|gb|GAIA01002120.1|:TSA: Salicornia europaea Unigene2165_Se200S transcribed RNA sequence	A0A0J8BTI7|A0A0J8BTI7_BETVU:Uncharacterized protein;OS=Beta vulgaris subsp. vulgaris.;

geneid	trend	GO	Pathway	TF	NR	NT	Uniprot
DN123884_c0_g1_i3	UP	[GO:0016021:integral component of membrane][GO:0009523:photosystem II][GO:0015979:photosynthesis]	-	-	XP_021757835.1:uncharacterized protein LOC110722841 [Chenopodium quinoa]	-	A0A0K9RTU3|A0A0K9RTU3_SPIOL: Uncharacterized protein;OS=Spinacia oleracea;|GO:0016021,GO:0009523,|GO:0015979,
DN116141_c2_g4_i2	UP	[GO:0006952:defense response][GO:0009607:response to biotic stimulus]	-	-	XP_021732306.1:pathogenesisrelated protein STH21like [Chenopodium quinoa]	-	A0A0J8ES22|A0A0J8ES22_BETVU:Uncharacterized protein;OS=Beta vulgaris subsp. vulgaris.;|GO:0006952,GO:0009607,
DN119920_c2_g5_i3	UP	-	[ko00902:Monoterpenoid biosynthesis]	-	XP_021760280.1:(+)neomenthol dehydrogenaselike [Chenopodium quinoa] >XP_021717522.1 (+)neomenthol dehydrogenaselike [Chenopodium quinoa]	gi|255541519|ref|XM_002511778.1|:Ricinus communis carbonyl reductase, putative, mRNA	A0A0J8D374|A0A0J8D374_BETVU:Uncharacterized protein;OS=Beta vulgaris subsp. vulgaris.;

geneid	trend	GO	Pathway	TF	NR	NT	Uniprot
DN123448_c2_g2_i1	UP	[GO:0005694:chromosome][GO:0005524:ATP binding][GO:0003677:DNA binding][GO:0003918:DNA topoisomerase type II (ATP-hydrolyzing) activity][GO:0006265:DNA topological change]	-	Neem_11770_f_1:Azadirachta indica|bZIP|bZIP family protein	XP_021744510.1:DNA gyrase subunit B, chloroplastic/mitochondriallike [Chenopodium quinoa]	gi|359481924|ref|XM_002266632.2|:PREDICTED: Vitis vinifera DNA gyrase subunit B, chloroplastic/mitochondrial-like (LOC100241769), mRNA	A0A0K9R140|A0A0K9R140_SPIOL:DNA gyrase subunit B;OS=Spinacia oleracea;|GO:0005694,|GO:0005524,GO:0003677,GO:0003918,|GO:0006265,
DN117469_c1_g1_i1	UP	-	-	-	XP_020226457.1:uncharacterized protein LOC109808052 isoform X2 [Cajanus cajan]	-	A0A0B2QZ08|A0A0B2QZ08_GLYSO: Uncharacterized protein;OS=Glycine soja;
DN121096_c3_g4_i4	UP	[GO:0016021:integral component of membrane][GO:0005886:plasma membrane]	-	-	XP_010675729.1:PREDICTED: CASPlike protein 2D1 [Beta vulgaris subsp. vulgaris] >KMT12753.1 hypothetical protein BVRB_4g088470 [Beta vulgaris subsp. vulgaris]	-	A0A0J8CL23|A0A0J8CL23_BETVU:CASP-like protein;OS=Beta vulgaris subsp. vulgaris.;|GO:0016021,GO:0005886,

geneid	trend	GO	Pathway	TF	NR	NT	Uniprot
DN127574_c1_g5_i6	UP	[GO:0003824:catalytic activity][GO:0030170:pyridoxal phosphate binding][GO:0009058:biosynthetic process]	[ko00220:Arginine biosynthesis][ko00250:Alanine, aspartate and glutamate metabolism][ko00260:Glycine, serine and threonine metabolism][ko00630:Glyoxylate and dicarboxylate metabolism][ko00710:Carbon fixation in photosynthetic organisms]	-	XP_010682426.1:PREDICTED: glutamateglyoxylate aminotransferase 2 [Beta vulgaris subsp. vulgaris] >XP_010682427.1 PREDICTED: glutamateglyoxylate aminotransferase 2 [Beta vulgaris subsp. vulgaris] >KMT07688.1 hypothetical protein BVRB_6g148110 [Beta vulgaris subsp. vulgaris]	gi|521742504|gb|GAIA01004916.1|:TSA: Salicornia europaea Unigene5203_Se200S transcribed RNA sequence	A0A0J8C6L6|A0A0J8C6L6_BETVU:Uncharacterized protein;OS=Beta vulgaris subsp. vulgaris.;|GO:0003824,GO:0030170,|GO:0009058,

geneid	trend	GO	Pathway	TF	NR	NT	Uniprot
DN113332_c2_g2_i3	UP	[GO:0000287:magnesium ion binding][GO:0004749:ribose phosphate diphosphokinase activity][GO:0009116:nucleoside metabolic process][GO:0009165:nucleotide biosynthetic process][GO:0009156:ribonucleoside monophosphate biosynthetic process]	[ko00030:Pentose phosphate pathway][ko00230:Purine metabolism]	-	XP_010691964.1:PREDICTED: ribosephosphate pyrophosphokinase 1 [Beta vulgaris subsp. vulgaris] >KMS99854.1 hypothetical protein BVRB_1g017070 [Beta vulgaris subsp. vulgaris]	gi|4902848|emb|AJ006940.1|:Spinacia oleracea mRNA for phosphoribosyl pyrophosphate synthase, isozyme 1	A0A0J8BEQ9|A0A0J8BEQ9_BETVU:Uncharacterized protein;OS=Beta vulgaris subsp. vulgaris.;|GO:0000287,GO:0004749,|GO:0009116,GO:0009165,GO:0009156,
DN126617_c0_g2_i2	UP	[GO:0009535:chloroplast thylakoid membrane][GO:0016021:integral component of membrane][GO:0009522:photosystem I][GO:0009523:photosystem II][GO:0016168:chlorophyll binding][GO:0046872:metal ion binding][GO:0009765:photosynthesis, light harvesting][GO:0018298:protein-chromophore linkage]	[ko00196:Photosynthesis - antenna proteins]	Bradi3g42627.3.p:Brachypodium distachyon|ERF|ERF family protein	XP_012084050.1:chlorophyll ab binding protein 8, chloroplastic [Jatropha curcas] >KDP45167.1 hypothetical protein JCGZ_15032 [Jatropha curcas]	gi|359489061|ref|XM_002273165.2|:PREDICTED: Vitis vinifera chlorophyll a/b binding protein (LHCA3), mRNA	A0A067L9S9|A0A067L9S9_JATCU:Chlorophyll a-b binding protein, chloroplastic;OS=Jatropha curcas;|GO:0009535,GO:0016021,GO:0009522,GO:0009523,|GO:0016168,GO:0046872,|GO:0009765,GO:0018298,

geneid	trend	GO	Pathway	TF	NR	NT	Uniprot
DN115532_c1_g8_i3	UP	[GO:0046872:metal ion binding][GO:0016491:oxidoreductase activity]	-	676709864:Sisymbrium irio|STAT|STAT family protein	XP_010665637.1:PREDICTED: protein SRG1like [Beta vulgaris subsp. vulgaris]	-	A0A0J8B6P3|A0A0J8B6P3_BETVU:Uncharacterized protein;OS=Beta vulgaris subsp. vulgaris.;|GO:0046872,GO:0016491,
DN120474_c0_g1_i7	UP	[GO:0004553:hydrolase activity, hydrolyzing O-glycosyl compounds][GO:0005975:carbohydrate metabolic process]	-	-	XP_010671663.1:PREDICTED: betaDxylosidase 1 [Beta vulgaris subsp. vulgaris] >KMT16215.1 hypothetical protein BVRB_3g053740 [Beta vulgaris subsp. vulgaris]	gi|32481072|gb|AY320257.1|:Chenopodium rubrum auxin-induced beta-glucosidase mRNA, complete cds	A0A0J8CR10|A0A0J8CR10_BETVU:Uncharacterized protein;OS=Beta vulgaris subsp. vulgaris.;|GO:0004553,|GO:0005975,

geneid	trend	GO	Pathway	TF	NR	NT	Uniprot
DN109932_c2_g3_i2	UP	-	-	XP_016483028.1:Nicotiana tabacum|bZIP|bZIP family protein	XP_020999426.1:uncharacterized protein LOC110281477 [Arachis duranensis]	gi|340842549|gb|HQ664640.1|:Bougainvillea glabra rpl23 pseudogene and tRNA-Ile (trnI-CAU) gene, complete sequence; hypothetical chloroplast RF2 (ycf2) gene, complete cds; tRNA-Leu (trnL-CAA) gene, complete sequence; NADH-plastoquinone oxidoreductase subunit 2 (ndhB) and ribosomal protein S7 (rps7) genes, complete cds; ribosomal protein S12 (rps12) gene, exons 2, 3 and partial cds; tRNA-Val (trnV-GAC) and 16S ribosomal RNA (rrn16) genes, complete sequence; tRNA-Ile (trnI-GAU) and	A0A166GEI3|A0A166GEI3_DAUCA:Uncharacterized protein;OS=Daucus carota subsp. sativus.;

geneid	trend	GO	Pathway	TF	NR	NT	Uniprot
DN117369_c1_g1_i3	UP	[GO:0009522:photosystem I][GO:0015979:photosynthesis]	[ko00195:Photosynthesis]	MDP0000319772:Malus domestica|MYB_related|MYB_related family protein	XP_010680205.1:PREDICTED: photosystem I reaction center subunit N, chloroplastic [Beta vulgaris subsp. vulgaris] >KMT09154.1 hypothetical protein BVRB_6g132600 [Beta vulgaris subsp. vulgaris]	gi|521651683|gb|GAIA01041050.1|:TSA: Salicornia europaea Unigene46126_Se200S transcribed RNA sequence	A0A0J8CA99|A0A0J8CA99_BETVU:Uncharacterized protein;OS=Beta vulgaris subsp. vulgaris.;|GO:0009522,|GO:0015979,
DN126267_c0_g3_i4	UP	[GO:0016021:integral component of membrane][GO:0005524:ATP binding][GO:0016887:ATPase activity]	-	MDP0000265619:Malus domestica|Nin-like|Nin-like family protein	XP_021721312.1:ABC transporter G family member 22like [Chenopodium quinoa]	gi|521485177|gb|GAHZ01045709.1|:TSA: Salicornia europaea Unigene53952_SeCKS transcribed RNA sequence	A0A067KIL4|A0A067KIL4_JATCU:Uncharacterized protein;OS=Jatropha curcas;|GO:0016021,|GO:0005524,GO:0016887,

geneid	trend	GO	Pathway	TF	NR	NT	Uniprot
DN123318_c1_g5_i2	UP	[GO:0016021:integral component of membrane][GO:0015297:antiporter activity][GO:0006855:drug transmembrane transport]	-	-	XP_002280423.1:PREDICTED: protein DETOXIFICATION 16 isoform X1 [Vitis vinifera]	-	F6GTS8|F6GTS8_VITVI:Protein DETOXIFICATION ;OS=Vitis vinifera;GN=VIT_17s0000g02970 {ECO:0000313|EMBL:CCB43252.1};|GO:0016021,|GO:0015297,GO:0015238,|GO:0006855,
DN117718_c3_g14_i2	UP	[GO:0016021:integral component of membrane][GO:0022857:transmembrane transporter activity]	-	-	XP_012087281.1:organic cation/carnitine transporter 7 isoform X1 [Jatropha curcas] >KDP25026.1 hypothetical protein JCGZ_22561 [Jatropha curcas]	-	A0A067JM06|A0A067JM06_JATCU:Uncharacterized protein;OS=Jatropha curcas;|GO:0016021,|GO:0022857,

geneid	trend	GO	Pathway	TF	NR	NT	Uniprot
DN127574_c1_g5_i4	UP	[GO:0003824:catalytic activity][GO:0030170:pyridoxal phosphate binding][GO:0009058:biosynthetic process]	[ko00220:Arginine biosynthesis][ko00250:Alanine, aspartate and glutamate metabolism][ko00260:Glycine, serine and threonine metabolism][ko00630:Glyoxylate and dicarboxylate metabolism][ko00710:Carbon fixation in photosynthetic organisms]	-	XP_010682426.1:PREDICTED: glutamateglyoxylate aminotransferase 2 [Beta vulgaris subsp. vulgaris] >XP_010682427.1 PREDICTED: glutamateglyoxylate aminotransferase 2 [Beta vulgaris subsp. vulgaris] >KMT07688.1 hypothetical protein BVRB_6g148110 [Beta vulgaris subsp. vulgaris]	gi|521742504|gb|GAIA01004916.1|:TSA: Salicornia europaea Unigene5203_Se200S transcribed RNA sequence	A0A0J8C6L6|A0A0J8C6L6_BETVU:Uncharacterized protein;OS=Beta vulgaris subsp. vulgaris.;|GO:0003824,GO:0030170,|GO:0009058,
DN125260_c1_g2_i1	UP	[GO:0043565:sequence-specific DNA binding][GO:0003700:DNA-binding transcription factor activity]	-	Dca14210.1:Dianthus caryophyllus|WRKY|WRKY family protein	XP_021866331.1:probable WRKY transcription factor 40 [Spinacia oleracea]	gi|298108802|gb|GU299481.1|:Artemisia annua WRKY3 transcription factor mRNA, complete cds	Q76LA9|Q76LA9_SPIOL:WRKY transcription factor 1;OS=Spinacia oleracea;|GO:0043565,GO:0003700,

geneid	trend	GO	Pathway	TF	NR	NT	Uniprot
DN120279_c6_g14_i1	UP	-	-	-	XP_021716270.1:LOW QUALITY PROTEIN: BURP domain protein RD22like [Chenopodium quinoa]	-	A0A0J8FQT2|A0A0J8FQT2_BETVU:Uncharacterized protein;OS=Beta vulgaris subsp. vulgaris.;
DN116331_c0_g2_i1	UP	-	-	AHYPO_006057-RA:Amaranthus hypochondriacus|bHLH|bHLH family protein	XP_021737367.1:transcription factor ICE1like [Chenopodium quinoa]	gi|521543481|gb|GAHZ01001689.1|:TSA: Salicornia europaea Unigene1730_SeCKS transcribed RNA sequence	A0A0S2CW82|A0A0S2CW82_PRUMU:Inducer of CBF expression a;OS=Prunus mume;
DN127140_c1_g2_i11	UP	[GO:0005576:extracellular region][GO:0009405:pathogenesis]	-	-	XP_002264858.1:PREDICTED: uncharacterized protein LOC100241465 [Vitis vinifera]	-	F6HZZ6|F6HZZ6_VITVI:Putative uncharacterized protein;OS=Vitis vinifera;GN=VIT_07s0005g06090{ECO:0000313|EMBL:CCB60262.1};|GO:0005576,|GO:0009405,

geneid	trend	GO	Pathway	TF	NR	NT	Uniprot
DN128690_c2_g1_i3	UP	[GO:0048046:apoplast][GO:0009506:plasmodesma][GO:0005524:ATP binding][GO:0003677:DNA binding][GO:0003910:DNA ligase (ATP) activity][GO:0071897:DNA biosynthetic process][GO:0051103:DNA ligation involved in DNA repair][GO:0006310:DNA recombination][GO:0006260:DNA replication][GO:0006303:double-strand break repair via nonhomologous end joining][GO:0010165:response to X-ray]	[ko03450:Non-homologous end-joining]	-	XP_010673725.1:PREDICTED: DNA ligase 4 [Beta vulgaris subsp. vulgaris] >KMT14578.1 hypothetical protein BVRB_4g073480 [Beta vulgaris subsp. vulgaris]	gi|225445942|ref|XM_002263931.1|:PREDICTED: Vitis vinifera DNA ligase 4-like (LOC100258105), mRNA	A0A0J8CRH1|A0A0J8CRH1_BETVU: DNA ligase;OS=Beta vulgaris subsp. vulgaris.;|GO:0048046,GO:0009506,|GO:0005524,GO:0003677,GO:0003910,|GO:0071897,GO:0051103,GO:0006310,GO:0006260,GO:0006303,GO:0010165,

geneid	trend	GO	Pathway	TF	NR	NT	Uniprot
DN119512_c4_g1_i7	UP	[GO:0048046:apoplast][GO:0009941:chloroplast envelope][GO:0009570:chloroplast stroma][GO:0009535:chloroplast thylakoid membrane][GO:0010319:stromule][GO:0051287:NAD binding][GO:0050661:NADP binding][GO:0016620:oxidoreductase activity, acting on the aldehyde or oxo group of donors, NAD or NADP as acceptor][GO:0006006:glucose metabolic process]	[ko00710:Carbon fixation in photosynthetic organisms]	MDP0000275412:Malus domestica|Trihelix|Trihelix family protein	XP_010686856.1:PREDICTED: glyceraldehyde3phosphate dehydrogenase B, chloroplastic [Beta vulgaris subsp. vulgaris] >KMT19244.1 hypothetical protein BVRB_1g014260 [Beta vulgaris subsp. vulgaris]	gi|512395725|gb|GAJZ01005445.1|:TSA: Prunus avium Pa_14655 transcribed RNA sequence	A0A0J8D4K0|A0A0J8D4K0_BETVU:Glyceraldehyde-3-phosphate dehydrogenase;OS=Beta vulgaris subsp. vulgaris.;|GO:0048046,GO:0009941,GO:0009570,GO:0009535,GO:0010319,|GO:0051287,GO:0050661,GO:0016620,|GO:0006006,
DN123687_c1_g1_i1	UP	[GO:0005739:mitochondrion][GO:0005525:GTP binding][GO:0003924:GTPase activity][GO:0003746:translation elongation factor activity][GO:0070125:mitochondrial translational elongation]	-	-	XP_009628327.1:PREDICTED: elongation factor TuA, chloroplastic [Nicotiana tomentosiformis]	gi|521491078|gb|GAHZ01039808.1|:TSA: Salicornia europaea Unigene45100_SeCKS transcribed RNA sequence	K4BK69|K4BK69_SOLLC:Elongation factor Tu;OS=Solanum lycopersicum;|GO:0005739,|GO:0005525,GO:0003924,GO:0003746,|GO:0070125,

geneid	trend	GO	Pathway	TF	NR	NT	Uniprot
DN118524_c3_g11_i1	UP	[GO:0009535:chloroplast thylakoid membrane][GO:0031361:integral component of thylakoid membrane][GO:0009055:electron transfer activity][GO:0020037:heme binding][GO:0005506:iron ion binding][GO:0055114:oxidation-reduction process][GO:0015979:photosynthesis]	[ko00195:Photosynthesis]	-	AFB70591.1:cytochrome f, partial (chloroplast) [Anredera baselloides]	gi|148607972|gb|EF534108.1|:Beta vulgaris chloroplast sequence	A0A0G2R7W8|A0A0G2R7W8_MESCR:Cytochrome f;OS=Mesembryanthemum crystallinum;|GO:0009535,GO:0031361,|GO:0009055,GO:0020037,GO:0005506,|GO:0055114,GO:0015979,
DN114832_c0_g1_i3	UP	[GO:0009535:chloroplast thylakoid membrane][GO:0009735:response to cytokinin]	-	-	XP_010670549.1:PREDICTED: uncharacterized protein LOC104887586 [Beta vulgaris subsp. vulgaris] >KMT17002.1 hypothetical protein BVRB_2g042050 [Beta vulgaris subsp. vulgaris]	-	A0A0J8CTU2|A0A0J8CTU2_BETVU:Uncharacterized protein;OS=Beta vulgaris subsp. vulgaris.;|GO:0009535,|GO:0009735,
DN121848_c	UP	-	-	-	-	-	-

geneid	trend	GO	Pathway	TF	NR	NT	Uniprot
1_g1_i1							
DN123468_c 5_g2_i2	UP	[GO:0019898:extrinsic component of membrane][GO:0009654:photosystem II oxygen evolving complex][GO:0005509:calcium ion binding][GO:0015979:photosynthesis]	[ko00195:Photosynthesis]	Pp3c2_23590V3.6.p:Physcomitrella patens|WRKY|WRKY family protein	XP_017648883.1:PREDICTED: oxygenevolving enhancer protein 2, chloroplasticlike isoform X1 [Gossypium arboreum] >XP_017648884.1 PREDICTED: oxygenevolving enhancer protein 2, chloroplasticlike isoform X2 [Gossypium arboreum] >KHG20721.1 Oxygenevolving enhancer 23, chloroplastic [Gossypium arboreum]	gi|356526941|ref|XM_003532026.1|:PREDICTED: Glycine max oxygen-evolving enhancer protein 2, chloroplastic-like (LOC100802315), mRNA	A0A0B0PBN3|A0A0B0PBN3_GOSAR: Oxygen-evolving enhancer 2-3, chloroplastic;OS=Gossypium arboreum;|GO:0019898,GO:0009654,|GO:0005509,|GO:0015979,

geneid	trend	GO	Pathway	TF	NR	NT	Uniprot
DN120039_c1_g2_i4	UP	[GO:0005524:ATP binding][GO:0004674:protein serine/threonine kinase activity]	[ko04016:MAPK signaling pathway - plant][ko04075:Plant hormone signal transduction]	Lus10043264:Linum usitatissimum|YABBY|YABBY family protein	XP_010680446.1:PREDICTED: serine/threonineprotein kinase SRK2I [Beta vulgaris subsp. vulgaris] >KMT09368.1 hypothetical protein BVRB_6g134600 [Beta vulgaris subsp. vulgaris]	gi|357114387|ref|XM_003558934.1|:PREDICTED: Brachypodium distachyon serine/threonine-protein kinase SAPK8-like, transcript variant 2 (LOC100827011), mRNA	A0A0J8CBL5|A0A0J8CBL5_BETVU:Uncharacterized protein;OS=Beta vulgaris subsp. vulgaris.;|GO:0005524,GO:0004674,
DN120624_c0_g2_i4	UP	[GO:0016021:integral component of membrane]	-	-	XP_010645864.1:PREDICTED: LOW QUALITY PROTEIN: uncharacterized protein LOC100246522 [Vitis vinifera]	gi|255569089|ref|XM_002525468.1|:Ricinus communis conserved hypothetical protein, mRNA	F6HMY3|F6HMY3_VITVI:Putative uncharacterized protein;OS=Vitis vinifera;GN=VIT_18s0001g03830{ECO:0000313|EMBL:CCB56038.1};|GO:0016021,
DN126142_c1_g4_i8	UP	[GO:0016491:oxidoreductase activity]	[ko00860:Porphyrin and chlorophyll metabolism]	LPERR03G23220.1:Leersia perrieri|G2-like|G2-like family protein	XP_021759692.1:chlorophyll(ide) b reductase NOL, chloroplasticlike [Chenopodium	gi|521538497|gb|GAHZ01004037.1|:TSA: Salicornia europaea Unigene4326_SeCKS transcribed RNA	A0A0K9RF66|A0A0K9RF66_SPIOL:Uncharacterized protein;OS=Spinacia oleracea;|GO:00164

geneid	trend	GO	Pathway	TF	NR	NT	Uniprot
					quinoa]	sequence	91,
DN127312_c 4_g17_i1	UP	[GO:0005524:ATP binding]	-	GSBRNA2T000860 41001:Brassica napus|ARF|ARF family protein	XP_021894604.1:rib ulose bisphosphate carboxylase/oxygena se activase, chloroplastic [Carica papaya]	gi|23320704|gb|AY1 30300.1|:Medicago sativa rubisco activase (Rca) mRNA, partial cds	V9VIF8|V9VIF8_ZI ZJJ:Ribulose bisphosphate carboxylase/oxygena se activase 1;OS=Ziziphus jujuba;|GO:0005524,
DN127161_c 3_g1_i14	UP	-	-	-	XP_021721284.1:pr otein CHUP1, chloroplasticlike [Chenopodium quinoa] >XP_021721285.1 protein CHUP1, chloroplasticlike [Chenopodium quinoa]	gi|521579490|gb|GA IA01069528.1|:TSA: Salicornia europaea Unigene83773_Se20 0S transcribed RNA sequence	A0A0J8CDG4|A0A 0J8CDG4_BETVU: Uncharacterized protein;OS=Beta vulgaris subsp. vulgaris.;

geneid	trend	GO	Pathway	TF	NR	NT	Uniprot
DN122419_c0_g3_i3	UP	-	-	-	XP_021731948.1:Fbox/kelchrepeat protein At1g80440like [Chenopodium quinoa]	gi|521651247|gb|GAIA01041423.1|:TSA: Salicornia europaea Unigene46538_Se200S transcribed RNA sequence	A0A0J8BIF1|A0A0J8BIF1_BETVU:Uncharacterized protein;OS=Beta vulgaris subsp. vulgaris.;
DN123328_c4_g1_i7	UP	[GO:0016021:integral component of membrane][GO:0070469:respirasome][GO:0009916:alternative oxidase activity][GO:0046872:metal ion binding]	-	-	XP_010686755.1:PREDICTED: ubiquinol oxidase 4, chloroplastic/chromoplastic [Beta vulgaris subsp. vulgaris] >KMT04070.1 hypothetical protein BVRB_8g185240 [Beta vulgaris subsp. vulgaris]	gi|521563038|gb|GAIA01075211.1|:TSA: Salicornia europaea Unigene91190_Se200S transcribed RNA sequence	A0A0J8BRE8|A0A0J8BRE8_BETVU:Ubiquinol oxidase;OS=Beta vulgaris subsp. vulgaris.;|GO:0016021,GO:0070469,|GO:0009916,GO:0046872,
DN116856_c3_g4_i9	UP	-	-	-	XP_021719862.1:uncharacterized protein LOC110687511 [Chenopodium quinoa]	gi|478165657|gb|GAHK01010946.1|:TSA: Jatropha curcas CL4440.Contig1_JC-CK_1A transcribed RNA sequence	A0A0K9QXX5|A0A0K9QXX5_SPIOL: Uncharacterized protein;OS=Spinacia oleracea;

geneid	trend	GO	Pathway	TF	NR	NT	Uniprot
DN118892_c0_g1_i1	UP	[GO:0009522:photosystem I][GO:0016168:chlorophyll binding][GO:0015979:photosynthesis]	[ko00195:Photosynthesis]	-	XP_004149569.1:PREDICTED: photosystem I reaction center subunit V, chloroplastic [Cucumis sativus] >KGN56044.1 hypothetical protein Csa_3G060980 [Cucumis sativus]	gi|192335343|gb|EU828956.1|:Linum usitatissimum clone LU0008E07 mRNA sequence	A0A0A0L4W3|A0A0A0L4W3_CUCSA: Uncharacterized protein;OS=Cucumis sativus;|GO:0009522,|GO:0016168,|GO:0015979,
DN123400_c0_g2_i1	UP	[GO:0009507:chloroplast][GO:0051537:2 iron, 2 sulfur cluster binding][GO:0009055:electron transfer activity][GO:0046872:metal ion binding][GO:0022900:electron transport chain]	[ko00195:Photosynthesis]	-	O04683.1:RecName: Full=Ferredoxin1, chloroplastic; AltName: Full=Ferredoxin I; Flags: Precursor >AAB61593.1 ferredoxin I precursor [Mesembryanthemum crystallinum]	gi|388510581|gb|BT143563.1|:Medicago truncatula clone JCVI-FLMt-7F8 unknown mRNA	O04683|FER1_MESCR:Ferredoxin-1, chloroplastic;OS=Mesembryanthemum crystallinum;|GO:0009507,|GO:0051537,GO:0009055,GO:0046872,|GO:0022900,

geneid	trend	GO	Pathway	TF	NR	NT	Uniprot
DN124515_c1_g2_i13	UP	[GO:0004729:oxygen-dependent protoporphyrinogen oxidase activity][GO:0006779:porphyrin-containing compound biosynthetic process]	[ko00860:Porphyrin and chlorophyll metabolism]	-	XP_021715303.1:protoporphyrinogen oxidase 1, chloroplasticlike [Chenopodium quinoa] >XP_021715304.1 protoporphyrinogen oxidase 1, chloroplasticlike [Chenopodium quinoa] >XP_021715305.1 protoporphyrinogen oxidase 1, chloroplasticlike [Chenopodium quinoa]	gi|292747472|dbj|AK337289.1|:Lotus japonicus cDNA, clone: LjFL1-054-AD02, HTC	D7U2T4|D7U2T4_VITVI:Putative uncharacterized protein;OS=Vitis vinifera;GN=VIT_07s0005g02540 {ECO:0000313|EMBL:CBI37050.3};|GO:0004729,|GO:0006779,

geneid	trend	GO	Pathway	TF	NR	NT	Uniprot
DN126841_c0_g1_i3	UP	[GO:0005737:cytoplasm][GO:0005524:ATP binding][GO:0042026:protein refolding]	[ko03018:RNA degradation][ko04212:Longevity regulating pathway - worm][ko04940:Type I diabetes mellitus][ko05134:Legionellosis][ko05152:Tuberculosis]	DCAR_026693:Daucus carota|bZIP|bZIP family protein	XP_010671843.1:PREDICTED: ruBisCO large subunitbinding protein subunit beta, chloroplastic [Beta vulgaris subsp. vulgaris] >KMT16357.1 hypothetical protein BVRB_3g055040 [Beta vulgaris subsp. vulgaris]	gi|521575944|gb|GAIA01071135.1|:TSA: Salicornia europaea Unigene85503_Se200S transcribed RNA sequence	A0A0J8CRX1|A0A0J8CRX1_BETVU: Uncharacterized protein;OS=Beta vulgaris subsp. vulgaris.;|GO:0005737,|GO:0005524,|GO:0042026,
DN116493_c3_g1_i9	UP	[GO:0000786:nucleosome][GO:0005634:nucleus][GO:0003677:DNA binding][GO:0006334:nucleosome assembly]	-	-	XP_010694434.1:PREDICTED: HMGYrelated protein A isoform X1 [Beta vulgaris subsp. vulgaris] >KMS98361.1 hypothetical protein BVRB_4g093180 [Beta vulgaris subsp. vulgaris]	-	A0A0J8BBS2|A0A0J8BBS2_BETVU:Uncharacterized protein;OS=Beta vulgaris subsp. vulgaris.;|GO:0000786,GO:0005634,|GO:0003677,|GO:0006334,

geneid	trend	GO	Pathway	TF	NR	NT	Uniprot
DN128767_c8_g3_i2	UP	[GO:0009507:chloroplast][GO:0051539:4 iron, 4 sulfur cluster binding][GO:0009055:electron transfer activity][GO:0030385:ferredoxin:thioredoxin reductase activity][GO:0046872:metal ion binding][GO:0055114:oxidation-reduction process]	-	-	XP_021859351.1:ferredoxinthioredoxin reductase catalytic chain, chloroplastic [Spinacia oleracea] >P41349.1 RecName: Full=Ferredoxinthioredoxin reductase catalytic chain, chloroplastic; Short=FTRC; AltName: Full=B1; AltName: Full=Ferredoxinthioredoxin reductase subunit B; Short=FTRB; Flags: Precursor >CAA54409.1 ferredoxinthioredoxin reductase SU B [Spinacia oleracea] >KNA20443.1 hypothetical protein	gi|505188|emb|X77164.1|:S.oleracea L. ftr B mRNA	P41349|FTRC2_SPIOL:Ferredoxin-thioredoxin reductase catalytic chain, chloroplastic;OS=Spinacia oleracea;|GO:0009507,|GO:0051539,GO:0009055,GO:0030385,GO:0046872,|GO:0055114,

geneid	trend	GO	Pathway	TF	NR	NT	Uniprot
					SOVF_052380 [Spinacia oleracea]		

geneid	trend	GO	Pathway	TF	NR	NT	Uniprot
DN119928_c0_g4_i1	UP	[GO:0005623:cell][GO:0009055:electron transfer activity][GO:0015035:protein disulfide oxidoreductase activity][GO:0045454:cell redox homeostasis]	-	-	CBI37229.3:unnamed protein product, partial [Vitis vinifera]	gi|460228730|gb|GABL01038040.1|:TSA: Camelina sativa comp48942_c0_seq1 transcribed RNA sequence	F6HZT9|F6HZT9_VITVI:Putative uncharacterized protein;OS=Vitis vinifera;GN=VIT_07s0005g04860 {ECO:0000313|EMBL:CCB60205.1};|GO:0005623,|GO:0009055,GO:0015035,|GO:0045454,

geneid	trend	GO	Pathway	TF	NR	NT	Uniprot
DN116108_c0_g1_i3	UP	[GO:0070694:deoxyribonucleoside 5'-monophosphate N-glycosidase activity][GO:0043733:DNA-3-methyl base glycosylase activity][GO:0070635:nicotinamide riboside hydrolase activity][GO:0070636:nicotinic acid riboside hydrolase activity][GO:0000701:purine-specific mismatch base pair DNA N-glycosylase activity][GO:0017065:single-strand selective uracil DNA N-glycosylase activity][GO:0009691:cytokinin biosynthetic process]	-	Vang05g09490.1:Vigna angularis|WRKY|WRKY family protein	XP_021738971.1:cytokinin riboside 5'monophosphate phosphoribohydrolase LOG8like isoform X2 [Chenopodium quinoa]	gi|521736091|gb|GAIA01008864.1|:TSA: Salicornia europaea Unigene9214_Se200S transcribed RNA sequence	A0A0J8D178|A0A0J8D178_BETVU:Cytokinin riboside 5'-monophosphate phosphoribohydrolase;OS=Beta vulgaris subsp. vulgaris.;|GO:0070694,GO:0043733,GO:0070635,GO:0070636,GO:0000701,GO:0017065,|GO:0009691,
DN120856_c2_g1_i2	UP	[GO:0051537:2 iron, 2 sulfur cluster binding][GO:0046872:metal ion binding][GO:0016491:oxidoreductase activity]	-	-	XP_008451029.1:PREDICTED: protochlorophyllidedependent translocon component 52, chloroplasticlike	-	A0A0A0LZA2|A0A0A0LZA2_CUCSA: Uncharacterized protein;OS=Cucumis sativus;|GO:0051537

geneid	trend	GO	Pathway	TF	NR	NT	Uniprot
					[Cucumis melo]		,GO:0046872,GO:0016491,
DN115642_c3_g1_i3	UP	-	-	-	XP_010670201.1:PREDICTED: uncharacterized protein LOC104887287 [Beta vulgaris subsp. vulgaris] >KMT17291.1 hypothetical protein BVRB_2g039890 [Beta vulgaris subsp. vulgaris]	gi|224141044|ref|XM_002323849.1|:Populus trichocarpa predicted protein, mRNA	A0A0J8FN88|A0A0J8FN88_BETVU:Uncharacterized protein;OS=Beta vulgaris subsp. vulgaris.;
DN126764_c0_g1_i2	UP	-	-	AHYPO_010751-RA:Amaranthus hypochondriacus|LBD|LBD family protein	XP_021714438.1:LOB domaincontaining protein 38like [Chenopodium quinoa]	gi|470103628|ref|XM_004288186.1|:PREDICTED: Fragaria vesca subsp. vesca LOB domain-containing protein 38-like	A0A0K9Q7W4|A0A0K9Q7W4_SPIOL:Uncharacterized protein;OS=Spinacia oleracea;

geneid	trend	GO	Pathway	TF	NR	NT	Uniprot
						(LOC101307625), mRNA	
DN122733_c 3_g2_i12	UP	[GO:0048046:apoplast][GO:0009941 :chloroplast envelope][GO:0009570:chloroplast stroma][GO:0010319:stromule][GO:0 009579:thylakoid][GO:0016857:race mase and epimerase activity, acting on carbohydrates and derivatives][GO:0005975:carbohydra te metabolic process][GO:0009624:response to nematode]	[ko00030:Pentose phosphate pathway][ko00040:P entose and glucuronate interconversions][ko 00710:Carbon fixation in photosynthetic organisms]	-	XP_021761594.1:rib ulosephosphate 3epimerase, chloroplastic [Chenopodium quinoa]	gi|512399395|gb|GA JZ01001775.1|:TSA: Prunus avium Pa_03473 transcribed RNA sequence	A0A0J8CF54|A0A0 J8CF54_BETVU:Un characterized protein;OS=Beta vulgaris subsp. vulgaris.;|GO:00480 46,GO:0009941,GO: 0009570,GO:001031 9,GO:0009579,|GO: 0016857,|GO:00059 75,GO:0009624,

geneid	trend	GO	Pathway	TF	NR	NT	Uniprot
DN125381_c1_g1_i3	UP	[GO:0016787:hydrolase activity][GO:0008152:metabolic process]	[ko04075:Plant hormone signal transduction]	-	XP_010674979.1:PREDICTED: gibberellin receptor GID1C [Beta vulgaris subsp. vulgaris] >KMT13544.1 hypothetical protein BVRB_4g083210 [Beta vulgaris subsp. vulgaris]	gi|349724383|emb|FQ393333.1|:Vitis vinifera clone SS0AFA26YI16	A0A0J8CNF0|A0A0J8CNF0_BETVU:Uncharacterized protein;OS=Beta vulgaris subsp. vulgaris.;|GO:0016787,|GO:0008152,
DN128285_c3_g1_i3	UP	[GO:0004089:carbonate dehydratase activity][GO:0008270:zinc ion binding][GO:0015976:carbon utilization]	[ko00910:Nitrogen metabolism]	Jcr4S00223.20:Jatropha curcas|bHLH|bHLH family protein	AEV42277.1:beta carbonic anhydrase 2 [Mesembryanthemum nodiflorum]	gi|359372873|gb|JN228099.1|:Mesembryanthemum nodiflorum beta carbonic anhydrase 2 (CA2) mRNA, complete cds	G9FZ36|G9FZ36_MESNO:Carbonic anhydrase;OS=Mesembryanthemum nodiflorum;|GO:0004089,GO:0008270,|GO:0015976,
DN128314_c2_g9_i2	UP	[GO:0003723:RNA binding]	-	-	XP_021845019.1:uncharacterized protein LOC110784877 [Spinacia oleracea] >KNA11272.1 hypothetical protein SOVF_136730	gi|521650865|gb|GAIA01041630.1|:TSA: Salicornia europaea Unigene46772_Se200S transcribed RNA sequence	A0A0K9QVG7|A0A0K9QVG7_SPIOL:Uncharacterized protein;OS=Spinacia oleracea;|GO:0003723,

geneid	trend	GO	Pathway	TF	NR	NT	Uniprot
					[Spinacia oleracea]		
DN124155_c0_g1_i6	UP	[GO:0009507:chloroplast]	-	Vradi05g10800.1:Vigna radiata|bZIP|bZIP family protein	XP_021729452.1:chaperone protein dnaJ 11, chloroplasticlike [Chenopodium quinoa]	gi|388516748|gb|BT146642.1|:Lotus japonicus clone JCVI-FLLj-8M13 unknown mRNA	A0A0J8BIA1|A0A0J8BIA1_BETVU:Uncharacterized protein;OS=Beta vulgaris subsp. vulgaris.;|GO:0009507,

geneid	trend	GO	Pathway	TF	NR	NT	Uniprot
DN122488_c0_g1_i1	UP	[GO:0016021:integral component of membrane][GO:0005622:intracellular][GO:0005262:calcium channel activity][GO:0005222:intracellular cAMP-activated cation channel activity][GO:0005242:inward rectifier potassium channel activity][GO:0007263:nitric oxide mediated signal transduction][GO:0009626:plant-type hypersensitive response]	[ko04626:Plant-pathogen interaction]	AHYPO_019315-RA:Amaranthus hypochondriacus|bHLH|bHLH family protein	XP_010674211.1:PREDICTED: cyclic nucleotidegated ion channel 2 [Beta vulgaris subsp. vulgaris]	gi|357502126|ref|XM_003621304.1|:Medicago truncatula Cyclic nucleotide-gated ion channel (MTR_7g012260) mRNA, complete cds	A0A0J8FWL5|A0A0J8FWL5_BETVU: Uncharacterized protein;OS=Beta vulgaris subsp. vulgaris.;|GO:0016021,GO:0005622,|GO:0005262,GO:0005222,GO:0005242,|GO:0007263,GO:0009626,
DN119008_c1_g3_i5	UP	-	-	-	XP_010681812.1:PREDICTED: uncharacterized protein LOC104896703 isoform X1 [Beta vulgaris subsp. vulgaris]	-	A0A162B1L2|A0A162B1L2_DAUCA:Uncharacterized protein;OS=Daucus carota subsp. sativus.;

geneid	trend	GO	Pathway	TF	NR	NT	Uniprot
DN121764_c2_g1_i6	UP	[GO:0016787:hydrolase activity]	[ko04310:Wnt signaling pathway]	Ote100112080011:Ocimum tenuiflorum|bHLH|bHLH family protein	KNA18490.1:hypothetical protein SOVF_070060 [Spinacia oleracea]	gi|255548206|ref|XM_002515114.1|:Ricinus communis pectin acetylesterase, putative, mRNA	A0A0K9RG85|A0A0K9RG85_SPIOL:Uncharacterized protein;OS=Spinacia oleracea;|GO:0016787,
DN118286_c0_g1_i6	UP	[GO:0009570:chloroplast stroma][GO:0016149:translation release factor activity, codon specific][GO:0009657:plastid organization][GO:0006396:RNA processing]	-	-	XP_010673888.1:PREDICTED: peptide chain release factor PrfB1, chloroplastic [Beta vulgaris subsp. vulgaris]	gi|521530764|gb|GAHZ01008074.1|:TSA: Salicornia europaea Unigene8415_SeCKS transcribed RNA sequence	A0A0J8FFW4|A0A0J8FFW4_BETVU: Uncharacterized protein;OS=Beta vulgaris subsp. vulgaris.;|GO:0009570,|GO:0016149,|GO:0009657,GO:0006396,
DN115285_c0_g1_i1	UP	[GO:0004462:lactoylglutathione lyase activity][GO:0046872:metal ion binding]	[ko00620:Pyruvate metabolism]	-	XP_021741465.1:probable lactoylglutathione lyase, chloroplastic [Chenopodium quinoa]	gi|359484558|ref|XM_003633073.1|:PREDICTED: Vitis vinifera probable lactoylglutathione lyase, chloroplast-like, transcript variant 2 (LOC100252461), mRNA	A0A0K9RAJ1|A0A0K9RAJ1_SPIOL:Lactoylglutathione lyase;OS=Spinacia oleracea;|GO:00044 62,GO:0046872,

geneid	trend	GO	Pathway	TF	NR	NT	Uniprot
DN121315_c0_g2_i6	UP	[GO:0009570:chloroplast stroma][GO:0009534:chloroplast thylakoid][GO:0044238:primary metabolic process]	-	-	XP_021763081.1:ribosomebinding factor PSRP1, chloroplastic [Chenopodium quinoa]	gi|521742540|gb|GAIA01004898.1|:TSA: Salicornia europaea Unigene5184_Se200S transcribed RNA sequence	A0A0J8B8J2|A0A0J8B8J2_BETVU:Uncharacterized protein;OS=Beta vulgaris subsp. vulgaris.;|GO:0009570,GO:0009534,|GO:0044238,
DN118061_c1_g1_i2	UP	[GO:0005524:ATP binding][GO:0004674:protein serine/threonine kinase activity][GO:0007165:signal transduction]	-	Lus10043264:Linum usitatissimum|YABBY|YABBY family protein	AAD31900.1:putative serine/threonine protein kinase [Mesembryanthemum crystallinum]	gi|4884973|gb|AF145482.1|AF145482:Mesembryanthemum crystallinum putative serine/threonine protein kinase mRNA, complete cds	Q9XFJ3|Q9XFJ3_MESCR:Non-specific serine/threonine protein kinase;OS=Mesembryanthemum crystallinum;|GO:0005524,GO:0004674,|GO:0007165,
DN118656_c3_g3_i2	UP	[GO:0016021:integral component of membrane][GO:0008270:zinc ion binding]	-	-	XP_021294192.1:RINGH2 finger protein ATL3like [Herrania umbratica]	-	A0A0P0KGG8|A0A0P0KGG8_9ROSA: RING-H2 finger protein-like protein;OS=Pyrus x bretschneideri;|GO:0016021,|GO:0008270,

geneid	trend	GO	Pathway	TF	NR	NT	Uniprot
DN120881_c0_g2_i1	UP	-	-	AA53G01052:Aethionema arabicum|MYB|MYB family protein	XP_010670360.1:PREDICTED: uncharacterized protein LOC104887434 [Beta vulgaris subsp. vulgaris] >XP_019103542.1 PREDICTED: uncharacterized protein LOC104887434 [Beta vulgaris subsp. vulgaris] >XP_019103543.1 PREDICTED: uncharacterized protein LOC104887434 [Beta vulgaris subsp. vulgaris] >XP_019103544.1 PREDICTED: uncharacterized protein	gi|449519686|ref|XM_004166818.1|:PREDICTED: Cucumis sativus putative rRNA methyltransferase YlbH-like (LOC101231492), mRNA	A0A0J8CTU5|A0A0J8CTU5_BETVU:Uncharacterized protein;OS=Beta vulgaris subsp. vulgaris.;

geneid	trend	GO	Pathway	TF	NR	NT	Uniprot
					LOC104887434 [Beta vulgaris subsp. vulgaris] >KMT17170.1 hypothetical protein BVRB_2g040800 [Beta vulgaris subsp. vulgaris]		

geneid	trend	GO	Pathway	TF	NR	NT	Uniprot
DN121829_c1_g1_i3	UP	[GO:0016021:integral component of membrane][GO:0009536:plastid][GO:0005215:transporter activity]	-	KZV22500.1:Dorcoceras hygrometricum|HB-other|HB-other family protein	AAF86906.1:triose phosphate/phosphate translocator precursor (plastid) [Mesembryanthemum crystallinum]	gi|9295272|gb|AF223358.1|:Mesembryanthemum crystallinum triose phosphate/phosphate translocator precursor (TPT) mRNA, complete cds	Q9MSB6|Q9MSB6_MESCR:Triose phosphate/phosphate translocator;OS=Mesembryanthemum crystallinum;|GO:0016021,GO:0009536,|GO:0005215,
DN118031_c1_g1_i1	UP	[GO:0005524:ATP binding][GO:0016887:ATPase activity][GO:0006810:transport]	-	-	KNA20748.1:hypothetical protein SOVF_049590 [Spinacia oleracea]	gi|521509017|gb|GAHZ01021903.1|:TSA: Salicornia europaea Unigene23546_SeCKS transcribed RNA sequence	A0A0K9RMN5|A0A0K9RMN5_SPIOL:Uncharacterized protein;OS=Spinacia oleracea;|GO:0005524,GO:0016887,|GO:0006810,
DN115131_c0_g1_i8	UP	[GO:0009535:chloroplast thylakoid membrane][GO:0031977:thylakoid lumen][GO:0006979:response to oxidative stress]	-	-	XP_010674669.1:PREDICTED: chloroplastic lipocalin [Beta vulgaris subsp. vulgaris] >KMT13688.1 hypothetical protein BVRB_4g080700	gi|521517872|gb|GAHZ01015104.1|:TSA: Salicornia europaea Unigene15792_SeCKS transcribed RNA sequence	A0A0J8CNS8|A0A0J8CNS8_BETVU:Uncharacterized protein;OS=Beta vulgaris subsp. vulgaris.;|GO:0009535,GO:0031977,|GO:0006979,

geneid	trend	GO	Pathway	TF	NR	NT	Uniprot
					[Beta vulgaris subsp. vulgaris]		
DN114384_c1_g1_i3	UP	[GO:0005524:ATP binding][GO:0004356:glutamate-ammonia ligase activity][GO:0006542:glutamine biosynthetic process]	[ko00220:Arginine biosynthesis][ko00250:Alanine, aspartate and glutamate metabolism][ko00630:Glyoxylate and dicarboxylate metabolism][ko00910:Nitrogen metabolism][ko02020:Two-component system][ko04217:Necroptosis][ko04724:Glutamatergic synapse][ko04727:GABAergic synapse]	-	AAD31898.1:glutamine synthetase leaf isozyme precursor [Mesembryanthemum crystallinum]	gi|4884969|gb|AF145480.1|AF145480:Mesembryanthemum crystallinum glutamine synthetase leaf isozyme precursor, mRNA, complete cds	Q9XFJ1|Q9XFJ1_MESCR:Glutamine synthetase;OS=Mesembryanthemum crystallinum;|GO:0005524,GO:0004356,|GO:0006542,

geneid	trend	GO	Pathway	TF	NR	NT	Uniprot
DN118442_c0_g1_i4	UP	-	-	-	KMS97608.1:hypothetical protein BVRB_5g125770 [Beta vulgaris subsp. vulgaris]	-	A0A0J8BC35|A0A0J8BC35_BETVU:Uncharacterized protein;OS=Beta vulgaris subsp. vulgaris.;
DN124494_c1_g4_i1	UP	[GO:0006457:protein folding][GO:0015031:protein transport]	-	-	XP_010672304.1:PREDICTED: uncharacterized protein LOC104888893 [Beta vulgaris subsp. vulgaris]	-	A0A0J8FJ05|A0A0J8FJ05_BETVU:Uncharacterized protein;OS=Beta vulgaris subsp. vulgaris.;|GO:0006457,GO:0015031,
DN122876_c7_g1_i3	UP	[GO:0016021:integral component of membrane][GO:0008270:zinc ion binding]	-	GSMUA_Achr7P25320_001:Musa acuminata|TCP|TCP family protein	AHB08887.1:RING H2 finger protein ATL2J [Suaeda glauca]	-	V5Q0U5|V5Q0U5_9CARY:RING-H2 finger protein ATL2J;OS=Suaeda glauca.;|GO:0016021,|GO:0008270,

geneid	trend	GO	Pathway	TF	NR	NT	Uniprot
DN122448_c1_g1_i4	UP	[GO:0016765:transferase activity, transferring alkyl or aryl (other than methyl) groups][GO:0008299:isoprenoid biosynthetic process][GO:0015979:photosynthesis]	[ko00900:Terpenoid backbone biosynthesis]	889097:Arabidopsis lyrata|STAT|STAT family protein	XP_010684494.1:PREDICTED: probable solanesyldiphosphate synthase 3, chloroplastic [Beta vulgaris subsp. vulgaris] >KMT05960.1 hypothetical protein BVRB_7g164490 [Beta vulgaris subsp. vulgaris]	gi|224061362|ref|XM_002300406.1|:Populus trichocarpa predicted protein, mRNA	A0A0J8BWM4|A0A0J8BWM4_BETVU:Uncharacterized protein;OS=Beta vulgaris subsp. vulgaris.;|GO:0016765,|GO:0008299,GO:0015979,
DN116934_c0_g1_i1	UP	-	-	-	XP_021659366.1:uncharacterized protein LOC110649198 [Hevea brasiliensis]	gi|242385106|emb|FP093158.1|:Phyllostachys edulis cDNA clone: bphyst007n24, full insert sequence	A0A067K1B2|A0A067K1B2_JATCU:Uncharacterized protein;OS=Jatropha curcas;
DN125386_c2_g1_i13	UP	[GO:0008374:O-acyltransferase activity][GO:0006629:lipid metabolic process]	-	Gh_D08G2402:Gossypium hirsutum|AP2|AP2 family protein	XP_021843458.1:phospholipase A(1) LCAT3 [Spinacia oleracea] >KNA18716.1 hypothetical protein	-	A0A0K9RGT8|A0A0K9RGT8_SPIOL:Uncharacterized protein;OS=Spinacia oleracea;|GO:0008374,|GO:0006629,

geneid	trend	GO	Pathway	TF	NR	NT	Uniprot
					SOVF_068220 [Spinacia oleracea]		
DN129195_c60_g8_i1	UP	[GO:0016021:integral component of membrane][GO:0022857:transmembrane transporter activity]	-	-	XP_021592722.1:organic cation/carnitine transporter 7like [Manihot esculenta] >OAY30051.1 hypothetical protein MANES_14G000300 [Manihot esculenta]	gi|478159684|gb|GAHK01016919.1|:TSA: Jatropha curcas Unigene4373_JC-CK_1A transcribed RNA sequence	U5G5F2|U5G5F2_POPTR:Uncharacterized protein;OS=Populus trichocarpa;|GO:0016021,|GO:0022857,
DN122191_c7_g1_i2	UP	-	-	-	XP_010688351.1:PREDICTED: uncharacterized protein LOC104902318 [Beta vulgaris subsp. vulgaris] >KMT03219.1 hypothetical protein	-	A0A0J8BT98|A0A0J8BT98_BETVU:Uncharacterized protein;OS=Beta vulgaris subsp. vulgaris.;

geneid	trend	GO	Pathway	TF	NR	NT	Uniprot
					BVRB_8g197670 [Beta vulgaris subsp. vulgaris]		
DN126293_c0_g1_i6	UP	-	-	-	XP_021747262.1:uncharacterized protein LOC110713110 isoform X2 [Chenopodium quinoa]	gi|224057175|ref|XM_002299121.1|:Populus trichocarpa predicted protein, mRNA	A0A0J8ELF1|A0A0J8ELF1_BETVU:Uncharacterized protein;OS=Beta vulgaris subsp. vulgaris.;
DN123885_c4_g1_i2	UP	[GO:0003677:DNA binding]	-	Sp_127650_nrid.t1:Spinacia oleracea|MYB|MYB family protein	KNA12256.1:hypothetical protein SOVF_127650 [Spinacia oleracea]	gi|521519284|gb|GAHZ01014489.1|:TSA: Salicornia europaea Unigene15151_SeCKS transcribed RNA sequence	A0A0K9R0E4|A0A0K9R0E4_SPIOL:Uncharacterized protein;OS=Spinacia oleracea;|GO:0003677,
DN124143_c3_g1_i1	UP	-	-	676725992:Sisymbrium irio|ERF|ERF family protein	XP_021756704.1:uncharacterized protein LOC110721813 [Chenopodium	-	A0A0J8BPN4|A0A0J8BPN4_BETVU:Uncharacterized protein;OS=Beta

geneid	trend	GO	Pathway	TF	NR	NT	Uniprot
					quinoa]		vulgaris subsp. vulgaris.;
DN113189_c0_g1_i3	UP	[GO:0016021:integral component of membrane]	-	-	XP_021846191.1:uncharacterized protein LOC110785992 isoform X2 [Spinacia oleracea] >XP_021846192.1 uncharacterized protein LOC110785992 isoform X2 [Spinacia oleracea]	-	V4KUC8|V4KUC8_EUTSA:Uncharacterized protein;OS=Eutrema salsugineum;|GO:0016021,
DN114449_c0_g1_i1	UP	-	-	-	XP_021769810.1:rubisco accumulation factor 1.1, chloroplasticlike [Chenopodium quinoa]	-	A0A0K9R1E1|A0A0K9R1E1_SPIOL:Uncharacterized protein;OS=Spinacia oleracea;

geneid	trend	GO	Pathway	TF	NR	NT	Uniprot
DN118228_c1_g3_i6	UP	[GO:0005634:nucleus][GO:0043565:sequence-specific DNA binding][GO:0003700:DNA-binding transcription factor activity]	-	Sp_108110_cazn.t1:Spinacia oleracea|HD-ZIP|HD-ZIP family protein	XP_021850966.1:homeoboxleucine zipper protein HAT5like [Spinacia oleracea] >KNA14370.1 hypothetical protein SOVF_108110 [Spinacia oleracea]	gi|470126859|ref|XM_004299352.1|:PREDICTED: Fragaria vesca subsp. vesca homeobox-leucine zipper protein HAT5-like, transcript variant 2 (LOC101292119), mRNA	A0A0K9R4D1|A0A0K9R4D1_SPIOL:Uncharacterized protein;OS=Spinacia oleracea;|GO:0005634,|GO:0043565,GO:0003700,
DN117247_c0_g1_i2	UP	[GO:0004842:ubiquitin-protein transferase activity]	-	FANhyb_rscf00000055.1.g00026.1:Fragaria x ananassa|GeBP|GeBP family protein	XP_010686933.1:PREDICTED: Ubox domaincontaining protein 44 [Beta vulgaris subsp. vulgaris] >KMT19258.1 hypothetical protein BVRB_1g014400 [Beta vulgaris subsp. vulgaris]	gi|502131038|ref|XM_004500797.1|:PREDICTED: Cicer arietinum U-box domain-containing protein 44-like (LOC101507091), mRNA	A0A0J8D515|A0A0J8D515_BETVU:Uncharacterized protein;OS=Beta vulgaris subsp. vulgaris.;|GO:0004842,

geneid	trend	GO	Pathway	TF	NR	NT	Uniprot
DN112824_c0_g2_i1	UP	[GO:0009505:plant-type cell wall][GO:0009506:plasmodesma]	-	-	OMO98084.1:Phosphateinduced protein 1 [Corchorus olitorius]	gi|224136773|ref|XM_002322376.1|:Populus trichocarpa predicted protein, mRNA	A0A0J8CV13|A0A0J8CV13_BETVU:Uncharacterized protein;OS=Beta vulgaris subsp. vulgaris.;|GO:0009505,GO:0009506,
DN120474_c0_g1_i8	UP	[GO:0004553:hydrolase activity, hydrolyzing O-glycosyl compounds][GO:0005975:carbohydrate metabolic process]	-	-	XP_021730258.1:betaDxylosidase 1like [Chenopodium quinoa]	gi|32481072|gb|AY320257.1|:Chenopodium rubrum auxin-induced beta-glucosidase mRNA, complete cds	A0A0J8CR10|A0A0J8CR10_BETVU:Uncharacterized protein;OS=Beta vulgaris subsp. vulgaris.;|GO:0004553,|GO:0005975,
DN126841_c0_g2_i3	UP	[GO:0005737:cytoplasm][GO:0005524:ATP binding][GO:0042026:protein refolding]	[ko03018:RNA degradation][ko04212:Longevity regulating pathway - worm][ko04940:Type I diabetes mellitus][ko05134:Legionellosis][ko05152:Tuberculosis]	DCAR_026693:Daucus carota|bZIP|bZIP family protein	XP_021765315.1:ruBisCO large subunitbinding protein subunit beta, chloroplasticlike [Chenopodium quinoa]	gi|225442530|ref|XM_002284098.1|:PREDICTED: Vitis vinifera ruBisCO large subunit-binding protein subunit beta, chloroplastic-like (LOC100261815), mRNA	A0A0J8BIV0|A0A0J8BIV0_BETVU:Uncharacterized protein;OS=Beta vulgaris subsp. vulgaris.;|GO:0005737,|GO:0005524,|GO:0042026,

geneid	trend	GO	Pathway	TF	NR	NT	Uniprot
DN122006_c5_g2_i5	UP	[GO:0016021:integral component of membrane][GO:0051537:2 iron, 2 sulfur cluster binding][GO:0010277:chlorophyllide a oxygenase [overall] activity]	[ko00860:Porphyrin and chlorophyll metabolism]	-	XP_010689248.1:PREDICTED: pheophorbide a oxygenase, chloroplastic [Beta vulgaris subsp. vulgaris] >KMT02273.1 hypothetical protein BVRB_9g206230 [Beta vulgaris subsp. vulgaris]	gi|521703238|gb|GAIA01016305.1|:TSA: Salicornia europaea Unigene17023_Se200S transcribed RNA sequence	A0A0J8BQQ3|A0A0J8BQQ3_BETVU: Uncharacterized protein;OS=Beta vulgaris subsp. vulgaris.;|GO:0016021,|GO:0051537,GO:0010277,
DN124761_c1_g3_i1	UP	[GO:0009570:chloroplast stroma][GO:0009535:chloroplast thylakoid membrane][GO:0022626:cytosolic ribosome][GO:0005634:nucleus][GO:0008266:poly(U) RNA binding][GO:0003735:structural constituent of ribosome][GO:0006412:translation]	[ko03010:Ribosome]	-	XP_021720722.1:50S ribosomal protein L4, chloroplasticlike [Chenopodium quinoa]	gi|2792019|emb|X93160.1|:S.oleracea mRNA for ribosomal protein L4	A0A0J8EQ55|A0A0J8EQ55_BETVU:Uncharacterized protein;OS=Beta vulgaris subsp. vulgaris.;|GO:0009570,GO:0009535,GO:0022626,GO:0005634,|GO:0008266,GO:0003735,|GO:0006412,

geneid	trend	GO	Pathway	TF	NR	NT	Uniprot
DN127886_c0_g2_i5	UP	[GO:0005524:ATP binding][GO:0004674:protein serine/threonine kinase activity]	-	Sme2.5_00580.1_g00006.1:Solanum melongena|CO-like|CO-like family protein	CBI25866.3:unnamed protein product, partial [Vitis vinifera]	gi|512396643|gb|GAJZ01004527.1|:TSA: Prunus avium Pa_10683 transcribed RNA sequence	F6HD69|F6HD69_VITVI:Putative uncharacterized protein;OS=Vitis vinifera;|GO:0005524,GO:0004674,
DN118158_c2_g1_i2	UP	[GO:0005739:mitochondrion]	-	Tp57577_TGAC_v2_mRNA8815:Trifolium pratense|LBD|LBD family protein	AGC78945.1:hypothetical protein (mitochondrion) [Vicia faba]	gi|384939108|emb|FQ014226.1|:Beta vulgaris subsp. maritima genotype male-sterile E mitochondrion, partial genome	R4ITY7|R4ITY7_VICFA:Uncharacterized protein;OS=Vicia faba;|GO:0005739,
DN123120_c2_g1_i8	UP	[GO:0003824:catalytic activity][GO:0008152:metabolic process]	-	GSMUA_Achr10P22190_001:Musa acuminata|bHLH|bHLH family protein	XP_010691375.1:PREDICTED: probable inactive nicotinamidase At3g16190 [Beta vulgaris subsp. vulgaris] >KMT00856.1 hypothetical protein BVRB_9g221300 [Beta vulgaris subsp. vulgaris]	-	A0A0J8ECW9|A0A0J8ECW9_BETVU: Uncharacterized protein;OS=Beta vulgaris subsp. vulgaris.;|GO:0003824,|GO:0008152,

geneid	trend	GO	Pathway	TF	NR	NT	Uniprot
DN128570_c5_g1_i8	UP	[GO:0016021:integral component of membrane][GO:0008270:zinc ion binding]	-	XP_016459247.1:Nicotiana tabacum|C2H2|C2H2 family protein	XP_010672410.1:PREDICTED: uncharacterized protein LOC104888989 [Beta vulgaris subsp. vulgaris] >KMT15493.1 hypothetical protein BVRB_3g058470 [Beta vulgaris subsp. vulgaris]	gi|460398411|ref|XM_004244706.1|:PREDICTED: Solanum lycopersicum uncharacterized LOC101251013 (LOC101251013), mRNA	A0A0J8CU64|A0A0J8CU64_BETVU:Uncharacterized protein;OS=Beta vulgaris subsp. vulgaris.;|GO:0016021,|GO:0008270,
DN116493_c3_g1_i2	UP	[GO:0000786:nucleosome][GO:0005634:nucleus][GO:0003677:DNA binding][GO:0006334:nucleosome assembly]	-	-	XP_010694434.1:PREDICTED: HMGYrelated protein A isoform X1 [Beta vulgaris subsp. vulgaris] >KMS98361.1 hypothetical protein BVRB_4g093180 [Beta vulgaris subsp. vulgaris]	-	A0A0J8BBS2|A0A0J8BBS2_BETVU:Uncharacterized protein;OS=Beta vulgaris subsp. vulgaris.;|GO:0000786,GO:0005634,|GO:0003677,|GO:0006334,

geneid	trend	GO	Pathway	TF	NR	NT	Uniprot
DN116204_c0_g1_i7	UP	[GO:0005759:mitochondrial matrix][GO:0000774:adenyl-nucleotide exchange factor activity][GO:0006457:protein folding]	-	MDP0000256621:Malus domestica|ARF|ARF family protein	XP_021863827.1:uncharacterized protein LOC110802688 isoform X1 [Spinacia oleracea] >KNA20105.1 hypothetical protein SOVF_055070 isoform A [Spinacia oleracea]	-	A0A0K9RKU5|A0A0K9RKU5_SPIOL: GrpE protein homolog;OS=Spinacia oleracea;|GO:0005759,|GO:0000774,|GO:0006457,
DN126664_c0_g2_i3	UP	[GO:0016021:integral component of membrane]	-	Bv5_101120_supk.t1:Beta vulgaris|ERF|ERF family protein	AAO74897.1:putative Na+/myoinositol symporter [Mesembryanthemum crystallinum]	gi|29501738|gb|AY233386.1|:Mesembryanthemum crystallinum putative Na+/myo-inositol symporter (Itr3) mRNA, complete cds	Q84UY4|Q84UY4_MESCR:Putative Na+/myo-inositol symporter;OS=Mesembryanthemum crystallinum;|GO:0016021,|GO:0022891,

geneid	trend	GO	Pathway	TF	NR	NT	Uniprot
DN116696_c2_g2_i2	UP	[GO:0010181:FMN binding][GO:0052853:long-chain-(S)-2-hydroxy-long-chain-acid oxidase activity][GO:0052854:medium-chain-(S)-2-hydroxy-acid oxidase activity][GO:0052852:very-long-chain-(S)-2-hydroxy-acid oxidase activity]	[ko00630:Glyoxylate and dicarboxylate metabolism][ko04146:Peroxisome]	FANhyb_rscf00000398.1.g00005.1:Fragaria x ananassa|G2-like|G2-like family protein	AAB40396.1:glycolate oxidase [Mesembryanthemum crystallinum]	gi|1773329|gb|U80071.1|MCU80071:Mesembryanthemum crystallinum glycolate oxidase (GOX) mRNA, complete cds	P93260|P93260_MESCR:Glycolate oxidase;OS=Mesembryanthemum crystallinum;|GO:0010181,GO:0052853,GO:0052854,GO:0052852,
DN123534_c2_g1_i2	UP	[GO:0005737:cytoplasm][GO:0005634:nucleus][GO:0004871:obsolete signal transducer activity][GO:0009638:phototropism]	-	BGIOSGA035841-PA:Oryza sativa subsp. indica|MYB_related|MYB_related family protein	XP_021715890.1:root phototropism protein 2like [Chenopodium quinoa]	-	A0A0J8CGR1|A0A0J8CGR1_BETVU:Uncharacterized protein;OS=Beta vulgaris subsp. vulgaris.;|GO:0005737,GO:0005634,|GO:0004871,|GO:0009638,
DN109987_c0_g2_i4	UP	[GO:0016021:integral component of membrane][GO:0005524:ATP binding][GO:0004672:protein kinase activity]	-	-	XP_021753979.1:somatic embryogenesis receptor kinase 2like [Chenopodium quinoa]	-	A0A0K9R2B7|A0A0K9R2B7_SPIOL:Uncharacterized protein;OS=Spinacia oleracea;|GO:0016021,|GO:0005524,GO:0004672,

geneid	trend	GO	Pathway	TF	NR	NT	Uniprot
DN127536_c6_g2_i1	UP	-	-	MDP0000574942:Malus domestica|MYB|MYB family protein	XP_021740247.1:protein EXORDIUMlike [Chenopodium quinoa]	gi|349708479|emb|FQ385247.1|:Vitis vinifera clone SS0AEB32YN20	A0A0K9R9T9|A0A0K9R9T9_SPIOL:Uncharacterized protein;OS=Spinacia oleracea;
DN120936_c3_g3_i2	UP	-	-	Sp_029270_fytx.t1:Spinacia oleracea|NAC|NAC family protein	AOC59206.1:NAC transcription factor 20 [Haloxylon ammodendron]	gi|521665074|gb|GAIA01034853.1|:TSA: Salicornia europaea Unigene38590_Se200S transcribed RNA sequence	A0A1B2RWJ2|A0A1B2RWJ2_9CARY: NAC transcription factor 20;OS=Haloxylon ammodendron.;
DN123884_c0_g2_i1	UP	[GO:0016021:integral component of membrane][GO:0009523:photosystem II][GO:0015979:photosynthesis]	-	-	XP_021757835.1:uncharacterized protein LOC110722841 [Chenopodium quinoa]	-	A0A0K9RTU3|A0A0K9RTU3_SPIOL: Uncharacterized protein;OS=Spinacia oleracea;|GO:0016021,GO:0009523,|GO:0015979,
DN118547_c0_g3_i2	UP	-	-	-	XP_021772095.1:photosystem II 5 kDa protein, chloroplasticlike [Chenopodium quinoa]	-	A0A068V1C0|A0A068V1C0_COFCA:Uncharacterized protein;OS=Coffea canephora;

geneid	trend	GO	Pathway	TF	NR	NT	Uniprot
DN128267_c1_g2_i2	UP	[GO:0009507:chloroplast][GO:0016021:integral component of membrane][GO:0009522:photosystem I][GO:0015979:photosynthesis]	-	-	AEX65542.1:photosystem I assembly protein Ycf4, partial (chloroplast) [Portulaca oleracea]	gi|11177503|gb|AF284694.1|:Carpobrotus chilensis ribulose-1,5-bisphosphate carboxylase/oxygenase large subunit (rbcL) gene, partial cds; acetyl-CoA carboxylase subunit (accD), PSI reaction center subunit VIII (psaI), and ycf4 (ycf4) genes, complete cds; and cemA (cemA) gene, partial cds; chloroplast genes for chloroplast products	H2BCF4|H2BCF4_POROL:Photosystem I assembly protein Ycf4;OS=Portulaca oleracea;|GO:0009507,GO:0016021,GO:0009522,|GO:0015979,

geneid	trend	GO	Pathway	TF	NR	NT	Uniprot
DN122884_c1_g6_i1	UP	[GO:0009535:chloroplast thylakoid membrane][GO:0016021:integral component of membrane][GO:0009522:photosystem I][GO:0009523:photosystem II][GO:0016168:chlorophyll binding][GO:0046872:metal ion binding][GO:0009765:photosynthesis, light harvesting][GO:0018298:protein-chromophore linkage]	[ko00196:Photosynthesis - antenna proteins]	-	XP_006429357.1:hypothetical protein CICLE_v10012370mg [Citrus clementina] >XP_006481019.1 PREDICTED: chlorophyll ab binding protein CP29.1, chloroplastic [Citrus sinensis] >ESR42597.1 hypothetical protein CICLE_v10012370mg [Citrus clementina]	gi|226358410|gb|FJ797565.1|:Gossypium hirsutum chloroplast chlorophyll A-B binding protein (CBP3) mRNA, complete cds; nuclear gene for chloroplast product	V4SPF6|V4SPF6_9ROSI:Chlorophyll a-b binding protein, chloroplastic;OS=Citrus clementina.;|GO:0009535,GO:0016021,GO:0009522,GO:0009523,|GO:0016168,GO:0046872,|GO:0009765,GO:0018298,
DN123612_c2_g1_i5	UP	[GO:0006457:protein folding][GO:0015031:protein transport]	-	-	XP_021774866.1:uncharacterized protein LOC110738749 [Chenopodium quinoa]	-	M1AAI8|M1AAI8_SOLTU:Uncharacterized protein;OS=Solanum tuberosum;|GO:0006457,GO:0015031,

geneid	trend	GO	Pathway	TF	NR	NT	Uniprot
DN117281_c0_g1_i1	UP	[GO:0016021:integral component of membrane][GO:0055085:transmembrane transport]	-	-	XP_021743730.1:mechanosensitive ion channel protein 2, chloroplasticlike [Chenopodium quinoa] >XP_021743731.1 mechanosensitive ion channel protein 2, chloroplasticlike [Chenopodium quinoa]	gi|521742437|gb|GAIA01004949.1|:TSA: Salicornia europaea Unigene5236_Se200S transcribed RNA sequence	A0A0J8BFR1|A0A0J8BFR1_BETVU:Uncharacterized protein;OS=Beta vulgaris subsp. vulgaris.;|GO:0016021,|GO:0055085,
DN127561_c0_g8_i9	UP	[GO:0005829:cytosol][GO:0009506:plasmodesma][GO:0003939:L-iditol 2-dehydrogenase activity][GO:0008270:zinc ion binding]	[ko00040:Pentose and glucuronate interconversions][ko00051:Fructose and mannose metabolism]	-	XP_010693460.1:PREDICTED: sorbitol dehydrogenase [Beta vulgaris subsp. vulgaris] >KMS99016.1 hypothetical protein BVRB_3g066230 [Beta vulgaris subsp. vulgaris]	gi|521449751|gb|GAHZ01070733.1|:TSA: Salicornia europaea Unigene86303_SeCKS transcribed RNA sequence	A0A0J8BDQ4|A0A0J8BDQ4_BETVU: Uncharacterized protein;OS=Beta vulgaris subsp. vulgaris.;|GO:0005829,GO:0009506,|GO:0003939,GO:0008270,

geneid	trend	GO	Pathway	TF	NR	NT	Uniprot
DN119925_c4_g5_i10	UP	-	[ko04016:MAPK signaling pathway - plant][ko04075:Plant hormone signal transduction]	PSME_00044429-RA:Pseudotsuga menziesii|MYB_related|MYB_related family protein	XP_010668998.1:PREDICTED: abscisic acid receptor PYL9 [Beta vulgaris subsp. vulgaris] >KMT20333.1 hypothetical protein BVRB_1g003320 [Beta vulgaris subsp. vulgaris]	-	A0A0J8D7Y5|A0A0J8D7Y5_BETVU:Uncharacterized protein;OS=Beta vulgaris subsp. vulgaris.;
DN121172_c4_g5_i2	UP	[GO:0016021:integral component of membrane][GO:0004252:serine-type endopeptidase activity]	-	-	XP_021847145.1:RHOMBOIDlike protein 9, chloroplastic [Spinacia oleracea]	gi|502180640|ref|XM_004516627.1|:PREDICTED: Cicer arietinum uncharacterized LOC101511229 (LOC101511229), mRNA	A0A0J8CXT0|A0A0J8CXT0_BETVU:Rhomboid-like protein;OS=Beta vulgaris subsp. vulgaris.;|GO:0016021,|GO:0004252,

geneid	trend	GO	Pathway	TF	NR	NT	Uniprot
DN121312_c3_g1_i3	UP	[GO:0009535:chloroplast thylakoid membrane][GO:0016021:integral component of membrane][GO:0009522:photosystem I][GO:0009523:photosystem II][GO:0016168:chlorophyll binding][GO:0046872:metal ion binding][GO:0009765:photosynthesis, light harvesting][GO:0018298:protein-chromophore linkage]	[ko00196:Photosynthesis - antenna proteins]	Bradi3g42627.3.p:Brachypodium distachyon|ERF|ERF family protein	XP_021776401.1:chlorophyll ab binding protein, chloroplastic [Chenopodium quinoa]	gi|521548619|gb|GAHZ01000031.1|:TSA: Salicornia europaea Unigene31_SeCKS transcribed RNA sequence	A0A0J8CWG9|A0A0J8CWG9_BETVU: Chlorophyll a-b binding protein, chloroplastic;OS=Beta vulgaris subsp. vulgaris.;|GO:0009535,GO:0016021,GO:0009522,GO:0009523,|GO:0016168,GO:0046872,|GO:0009765,GO:0018298,
DN109259_c0_g1_i2	UP	-	[ko04110:Cell cycle][ko04111:Cell cycle - yeast][ko05166:HTLV-I infection]	-	XP_021724866.1:mitotic checkpoint protein BUB3.2 [Chenopodium quinoa]	gi|521442352|gb|GAHZ01078132.1|:TSA: Salicornia europaea Unigene95707_SeCKS transcribed RNA sequence	A0A0J8BRL5|A0A0J8BRL5_BETVU:Uncharacterized protein;OS=Beta vulgaris subsp. vulgaris.;
DN116613_c4_g2_i13	UP	[GO:0017053:transcriptional repressor complex][GO:0007049:cell cycle]	-	-	GAV78250.1:hypothetical protein CFOL_v3_21718 [Cephalotus follicularis]	-	A0A0J8BT56|A0A0J8BT56_BETVU:Uncharacterized protein;OS=Beta vulgaris subsp.

geneid	trend	GO	Pathway	TF	NR	NT	Uniprot
							vulgaris.;|GO:0017053,|GO:0007049,
DN116696_c2_g2_i7	UP	[GO:0010181:FMN binding][GO:0052853:long-chain-(S)-2-hydroxy-long-chain-acid oxidase activity][GO:0052854:medium-chain-(S)-2-hydroxy-acid oxidase activity][GO:0052852:very-long-chain-(S)-2-hydroxy-acid oxidase activity]	[ko00630:Glyoxylate and dicarboxylate metabolism][ko04146:Peroxisome]	FANhyb_rscf00000398.1.g00005.1:Fragaria x ananassa|G2-like|G2-like family protein	AAB40396.1:glycolate oxidase [Mesembryanthemum crystallinum]	gi|1773329|gb|U80071.1|MCU80071:Mesembryanthemum crystallinum glycolate oxidase (GOX) mRNA, complete cds	P93260|P93260_MESCR:Glycolate oxidase;OS=Mesembryanthemum crystallinum;|GO:0010181,GO:0052853,GO:0052854,GO:0052852,
DN122305_c3_g1_i2	UP	[GO:0005623:cell][GO:0015035:protein disulfide oxidoreductase activity][GO:0045454:cell redox homeostasis][GO:0006662:glycerol ether metabolic process]	-	-	XP_021737089.1:thioredoxinlike protein CDSP32, chloroplastic [Chenopodium quinoa]	-	A0A0K9QI10|A0A0K9QI10_SPIOL:Uncharacterized protein;OS=Spinacia oleracea;|GO:0005623,|GO:0015035,|GO:0045454,GO:0006662,

geneid	trend	GO	Pathway	TF	NR	NT	Uniprot
DN115893_c4_g1_i1	UP	[GO:0016021:integral component of membrane]	-	-	XP_010667529.1:PREDICTED: uncharacterized protein LOC104884553 [Beta vulgaris subsp. vulgaris] >KMS95320.1 hypothetical protein BVRB_009370 [Beta vulgaris subsp. vulgaris]	-	A0A0J8DWT3|A0A0J8DWT3_BETVU: Uncharacterized protein;OS=Beta vulgaris subsp. vulgaris.;|GO:0016021,
DN120690_c2_g1_i3	UP	-	-	-	XP_010673460.1:PREDICTED: uncharacterized protein LOC104889834 [Beta vulgaris subsp. vulgaris] >KMT14302.1 hypothetical protein BVRB_4g070870 [Beta vulgaris subsp. vulgaris]	-	A0A0J8CLD1|A0A0J8CLD1_BETVU:Uncharacterized protein;OS=Beta vulgaris subsp. vulgaris.;

geneid	trend	GO	Pathway	TF	NR	NT	Uniprot
DN121670_c3_g1_i4	UP	-	-	-	-	gi|4884973|gb|AF145482.1|AF145482:Mesembryanthemum crystallinum putative serine/threonine protein kinase mRNA, complete cds	-
DN126451_c1_g6_i1	UP	[GO:0009507:chloroplast][GO:0005524:ATP binding][GO:0004672:protein kinase activity][GO:0042548:regulation of photosynthesis, light reaction]	-	-	XP_021723885.1:serine/threonineprotein kinase STN7, chloroplasticlike [Chenopodium quinoa]	gi|521453095|gb|GAHZ01067389.1|:TSA: Salicornia europaea Unigene81418_SeCKS transcribed RNA sequence	A0A0J8E2Q9|A0A0J8E2Q9_BETVU:Uncharacterized protein;OS=Beta vulgaris subsp. vulgaris.;|GO:0009507,|GO:0005524,GO:0004672,|GO:0042548,
DN123758_c1_g1_i5	UP	[GO:0016021:integral component of membrane]	-	-	XP_021844493.1:PGR5like protein 1A, chloroplastic [Spinacia oleracea] >KNA15411.1 hypothetical protein SOVF_098540 [Spinacia oleracea]	gi|521502004|gb|GAHZ01028916.1|:TSA: Salicornia europaea Unigene31845_SeCKS transcribed RNA sequence	A0A0K9R7D6|A0A0K9R7D6_SPIOL:Uncharacterized protein;OS=Spinacia oleracea;|GO:0016021,

geneid	trend	GO	Pathway	TF	NR	NT	Uniprot
DN120283_c0_g3_i1	UP	[GO:0005737:cytoplasm][GO:0005524:ATP binding][GO:0046914:transition metal ion binding][GO:1901671:positive regulation of superoxide dismutase activity][GO:0006457:protein folding]	-	LPERR06G05720.2: Leersia perrieri|ARF|ARF family protein	XP_021756702.1:20 kDa chaperonin, chloroplasticlike [Chenopodium quinoa]	gi|255550362|ref|XM_002516186.1|:Ricinus communis groes chaperonin, putative, mRNA	A0A0K9RUA6|A0A0K9RUA6_SPIOL: Uncharacterized protein;OS=Spinacia oleracea;|GO:0005737,|GO:0005524,GO:0046914,|GO:1901671,GO:0006457,
DN127574_c1_g5_i5	UP	[GO:0003824:catalytic activity][GO:0030170:pyridoxal phosphate binding][GO:0009058:biosynthetic process]	[ko00220:Arginine biosynthesis][ko00250:Alanine, aspartate and glutamate metabolism][ko00260:Glycine, serine and threonine metabolism][ko00630:Glyoxylate and dicarboxylate metabolism][ko00710:Carbon fixation in photosynthetic organisms]	-	XP_010682426.1:PREDICTED: glutamateglyoxylate aminotransferase 2 [Beta vulgaris subsp. vulgaris] >XP_010682427.1 PREDICTED: glutamateglyoxylate aminotransferase 2 [Beta vulgaris subsp. vulgaris] >KMT07688.1 hypothetical protein BVRB_6g148110 [Beta vulgaris subsp. vulgaris]	gi|521742504|gb|GAIA01004916.1|:TSA: Salicornia europaea Unigene5203_Se200S transcribed RNA sequence	A0A0J8C6L6|A0A0J8C6L6_BETVU:Uncharacterized protein;OS=Beta vulgaris subsp. vulgaris.;|GO:0003824,GO:0030170,|GO:0009058,

geneid	trend	GO	Pathway	TF	NR	NT	Uniprot
DN125807_c2_g1_i1	UP	[GO:0005524:ATP binding]	[ko03040:Spliceosome][ko04010:MAPK signaling pathway][ko04141:Protein processing in endoplasmic reticulum][ko04144:Endocytosis][ko04213:Longevity regulating pathway - multiple species][ko04612:Antigen processing and presentation][ko04915:Estrogen signaling pathway][ko05134:Legionellosis][ko05145:Toxoplasmosis][ko05162:Measles][ko05164:Influenza A][ko05169:Epstein-Barr virus infection]	FANhyb_rscf00000233.1.g00002.1:Fragaria x ananassa|GATA|GATA family protein	XP_006424799.1:hypothetical protein CICLE_v10027981mg [Citrus clementina] >ESR38039.1 hypothetical protein CICLE_v10027981mg [Citrus clementina]	gi|306595234|gb|GQ851948.1|:Phytolacca acinosa heat shock cognate 70 kDa protein (HSP1) mRNA, partial cds	V4UFD6|V4UFD6_9ROSI:Uncharacterized protein;OS=Citrus clementina.;|GO:0005524,

geneid	trend	GO	Pathway	TF	NR	NT	Uniprot
DN113354_c1_g1_i3	UP	[GO:0016021:integral component of membrane]	-	Zmw_sc06582.1.g00020.1:Zoysia matrella|C2H2|C2H2 family protein	XP_010691331.1:PREDICTED: uncharacterized protein LOC104904681 [Beta vulgaris subsp. vulgaris] >KMT00815.1 hypothetical protein BVRB_9g220910 [Beta vulgaris subsp. vulgaris]	-	A0A0J8BHD3|A0A0J8BHD3_BETVU: Uncharacterized protein;OS=Beta vulgaris subsp. vulgaris.;|GO:0016021,
DN113744_c1_g1_i1	UP	-	-	-	XP_021749531.1:uncharacterized protein LOC110715276 [Chenopodium quinoa]	-	A0A0J8FRU0|A0A0J8FRU0_BETVU:Uncharacterized protein;OS=Beta vulgaris subsp. vulgaris.;
DN119414_c0_g2_i1	UP	[GO:0050660:flavin adenine dinucleotide binding][GO:0016614:oxidoreductase activity, acting on CH-OH group of donors]	-	FANhyb_rscf00000362.1.g00002.1:Fragaria x ananassa|CPP|CPP family protein	XP_021768329.1:berberine bridge enzymelike 15 [Chenopodium quinoa]	gi|224061592|ref|XM_002300521.1|:Populus trichocarpa predicted protein, mRNA	A0A0K9QR43|A0A0K9QR43_SPIOL:Uncharacterized protein;OS=Spinacia oleracea;|GO:0050660,GO:0016614,

geneid	trend	GO	Pathway	TF	NR	NT	Uniprot
DN115733_c1_g5_i8	UP	[GO:0016021:integral component of membrane][GO:0055114:oxidation-reduction process]	-	PDK_30s976561g001:Phoenix dactylifera|bZIP|bZIP family protein	XP_021722631.1:probable ascorbatespecific transmembrane electron transporter 1 [Chenopodium quinoa]	-	A0A059BF36|A0A059BF36_EUCGR:Uncharacterized protein;OS=Eucalyptus grandis;|GO:0016021,|GO:0055114,
DN113469_c0_g1_i2	UP	-	-	-	XP_010685873.1:PREDICTED: BTB/POZ domaincontaining protein At5g60050 [Beta vulgaris subsp. vulgaris] >KMT05357.1 hypothetical protein BVRB_7g174890 [Beta vulgaris subsp. vulgaris]	gi|521528890|gb|GAHZ01009020.1|:TSA: Salicornia europaea Unigene9404_SeCKS transcribed RNA sequence	A0A0J8BW47|A0A0J8BW47_BETVU: Uncharacterized protein;OS=Beta vulgaris subsp. vulgaris.;
DN124893_c0_g1_i1	UP	-	-	-	XP_021851171.1:mannose/glucosespecific lectinlike [Spinacia oleracea]	-	A0A0K9QBJ0|A0A0K9QBJ0_SPIOL:Uncharacterized protein;OS=Spinacia oleracea;

geneid	trend	GO	Pathway	TF	NR	NT	Uniprot
DN120283_c0_g3_i3	UP	[GO:0005737:cytoplasm][GO:0005524:ATP binding][GO:0046914:transition metal ion binding][GO:1901671:positive regulation of superoxide dismutase activity][GO:0006457:protein folding]	-	LPERR06G05720.2: Leersia perrieri|ARF|ARF family protein	XP_021756702.1:20 kDa chaperonin, chloroplasticlike [Chenopodium quinoa]	gi|242374571|emb|FP095731.1|:Phyllostachys edulis cDNA clone: bphyem202e03, full insert sequence	A0A0K9RUA6|A0A0K9RUA6_SPIOL: Uncharacterized protein;OS=Spinacia oleracea;|GO:0005737,|GO:0005524,GO:0046914,|GO:1901671,GO:0006457,
DN123784_c4_g1_i6	UP	[GO:0004838:L-tyrosine:2-oxoglutarate aminotransferase activity][GO:0030170:pyridoxal phosphate binding][GO:0006520:cellular amino acid metabolic process][GO:0010189:vitamin E biosynthetic process]	[ko00130:Ubiquinone and other terpenoid-quinone biosynthesis][ko00270:Cysteine and methionine metabolism][ko00350:Tyrosine metabolism][ko00360:Phenylalanine metabolism][ko00400:Phenylalanine, tyrosine and tryptophan biosynthesis][ko00401:Novobiocin biosynthesis][ko009	-	XP_010675472.1:PREDICTED: probable aminotransferase TAT2 isoform X2 [Beta vulgaris subsp. vulgaris]	gi|27525395|emb|AJ458993.1|:Coleus blumei mRNA for tyrosine aminotransferase (tat gene)	A0A0J8CHC8|A0A0J8CHC8_BETVU: Uncharacterized protein;OS=Beta vulgaris subsp. vulgaris.;|GO:0004838,GO:0030170,|GO:0006520,GO:0010189,

geneid	trend	GO	Pathway	TF	NR	NT	Uniprot
			50:Isoquinoline alkaloid biosynthesis][ko00960:Tropane, piperidine and pyridine alkaloid biosynthesis]				

geneid	trend	GO	Pathway	TF	NR	NT	Uniprot
DN109544_c0_g2_i1	UP	-	-	-	XP_010680131.1:PREDICTED: B2 protein [Beta vulgaris subsp. vulgaris] >XP_010680134.1 PREDICTED: B2 protein [Beta vulgaris subsp. vulgaris] >KMT09095.1 hypothetical protein BVRB_6g132030 [Beta vulgaris subsp. vulgaris]	gi|521738675|gb|GAIA01007463.1|:TSA: Salicornia europaea Unigene7778_Se200S transcribed RNA sequence	A0A161ZSX1|A0A161ZSX1_DAUCA:Uncharacterized protein;OS=Daucus carota subsp. sativus.;
DN114474_c1_g3_i2	UP	[GO:0016020:membrane][GO:0005524:ATP binding][GO:0046872:metal ion binding][GO:0006457:protein folding][GO:0009408:response to heat]	[ko04141:Protein processing in endoplasmic reticulum]	ONIVA04G19030.1: Oryza nivara|MYB|MYB family protein	P43644.1:RecName: Full=DnaJ protein homolog ANJ1; Flags: Precursor	gi|289212|gb|L09124.1|ATPANJ1X:Atriplex nummularia homologous sequence (ANJ1) mRNA	P43644|DNJH_ATRNU:DnaJ protein homolog ANJ1;OS=Atriplex nummularia;|GO:0016020,|GO:0005524,GO:0046872,|GO:0006457,GO:0009408,
DN128979_c4_g1_i1	UP	-	-	-	-	-	-

geneid	trend	GO	Pathway	TF	NR	NT	Uniprot
DN117258_c0_g1_i4	UP	[GO:0005840:ribosome][GO:0003735:structural constituent of ribosome][GO:0006412:translation]	[ko03010:Ribosome]	-	XP_010694274.1:PREDICTED: 50S ribosomal protein L24, chloroplastic [Beta vulgaris subsp. vulgaris]	-	A0A0K9QFU9|A0A0K9QFU9_SPIOL: Uncharacterized protein;OS=Spinacia oleracea;|GO:0005840,|GO:0003735,|GO:0006412,
DN112973_c0_g1_i3	UP	-	-	-	XP_010665890.1:PREDICTED: uncharacterized protein LOC104883135 [Beta vulgaris subsp. vulgaris] >KMS96479.1 hypothetical protein BVRB_9g224720 [Beta vulgaris subsp. vulgaris]	-	A0A0J8B5G6|A0A0J8B5G6_BETVU:Uncharacterized protein;OS=Beta vulgaris subsp. vulgaris.;

geneid	trend	GO	Pathway	TF	NR	NT	Uniprot
DN122733_c3_g2_i8	UP	[GO:0048046:apoplast][GO:0009941:chloroplast envelope][GO:0009570:chloroplast stroma][GO:0010319:stromule][GO:0009579:thylakoid][GO:0016857:racemase and epimerase activity, acting on carbohydrates and derivatives][GO:0005975:carbohydrate metabolic process][GO:0009624:response to nematode]	[ko00030:Pentose phosphate pathway][ko00040:Pentose and glucuronate interconversions][ko00710:Carbon fixation in photosynthetic organisms]	-	XP_021761594.1:ribulosephosphate 3epimerase, chloroplastic [Chenopodium quinoa]	gi|3264789|gb|AF070942.1|:Expression vector pFL505 for Spinacia oleracea chloroplast ribulose-phosphate 3-epimerase transit form	A0A0J8CF54|A0A0J8CF54_BETVU:Uncharacterized protein;OS=Beta vulgaris subsp. vulgaris.;|GO:0048046,GO:0009941,GO:0009570,GO:0010319,GO:0009579,|GO:0016857,|GO:0005975,GO:0009624,
DN125124_c3_g1_i3	UP	[GO:0003676:nucleic acid binding][GO:0000166:nucleotide binding]	-	CCG030364.1:Populus euphratica|NF-YA|NF-YA family protein	XP_021770788.1:28 kDa ribonucleoprotein, chloroplasticlike [Chenopodium quinoa]	gi|359477775|ref|XM_002285433.2|:PREDICTED: Vitis vinifera uncharacterized LOC100261382 (LOC100261382), mRNA	A0A0J8B6R4|A0A0J8B6R4_BETVU:Uncharacterized protein;OS=Beta vulgaris subsp. vulgaris.;|GO:0003676,GO:0000166,

geneid	trend	GO	Pathway	TF	NR	NT	Uniprot
DN117139_c3_g1_i1	UP	[GO:0016021:integral component of membrane]	-	-	XP_010677247.1:PREDICTED: uncharacterized protein LOC104892921 [Beta vulgaris subsp. vulgaris] >KMT11666.1 hypothetical protein BVRB_5g106050 isoform A [Beta vulgaris subsp. vulgaris]	gi|521674768|gb|GAIA01030097.1|:TSA: Salicornia europaea Unigene33009_Se200S transcribed RNA sequence	A0A0J8F6U0|A0A0J8F6U0_BETVU:Uncharacterized protein;OS=Beta vulgaris subsp. vulgaris.;|GO:0016021,
DN122754_c1_g1_i12	UP	[GO:0046872:metal ion binding][GO:0004722:protein serine/threonine phosphatase activity]	-	KZV22762.1:Dorcoceras hygrometricum|CAMTA|CAMTA family protein	KNA11038.1:hypothetical protein SOVF_138810 [Spinacia oleracea]	gi|512399048|gb|GAJZ01002122.1|:TSA: Prunus avium Pa_04233 transcribed RNA sequence	A0A0K9QWF1|A0A0K9QWF1_SPIOL:Uncharacterized protein;OS=Spinacia oleracea;|GO:0046872,GO:0004722,

geneid	trend	GO	Pathway	TF	NR	NT	Uniprot
DN126821_c3_g7_i1	UP	[GO:0005524:ATP binding]	[ko03040:Spliceosome][ko04010:MAPK signaling pathway][ko04141:Protein processing in endoplasmic reticulum][ko04144:Endocytosis][ko04213:Longevity regulating pathway - multiple species][ko04612:Antigen processing and presentation][ko04915:Estrogen signaling pathway][ko05134:Legionellosis][ko05145:Toxoplasmosis][ko05162:Measles][ko05164:Influenza A][ko05169:Epstein-Barr virus infection]	FANhyb_rscf00000233.1.g00002.1:Fragaria x ananassa|GATA|GATA family protein	XP_010558794.1:PREDICTED: probable mediator of RNA polymerase II transcription subunit 37c [Tarenaya hassleriana]	gi|521705294|gb|GAIA01015354.1|:TSA: Salicornia europaea Unigene16032_Se200S transcribed RNA sequence	K3XFF0|K3XFF0_SETIT:Uncharacterized protein;OS=Setaria italica;|GO:0005524,

geneid	trend	GO	Pathway	TF	NR	NT	Uniprot
DN118919_c0_g2_i7	UP	[GO:0016021:integral component of membrane]	-	-	XP_021736623.1:uncharacterized protein LOC110703185 [Chenopodium quinoa]	gi|478166929|gb|GAHK01009674.1|:TSA: Jatropha curcas CL3792.Contig1_JC-CK_1A transcribed RNA sequence	A0A0J8C4F5|A0A0J8C4F5_BETVU:Uncharacterized protein;OS=Beta vulgaris subsp. vulgaris.;|GO:0016021,
DN122748_c2_g4_i1	UP	-	-	-	-	-	-
DN122803_c0_g10_i3	UP	[GO:0030598:rRNA N-glycosylase activity][GO:0006952:defense response][GO:0017148:negative regulation of translation]	-	-	ABI64066.1:ribosome inactivating protein [Bougainvillea spectabilis]	gi|114325705|gb|DQ989495.1|:Bougainvillea spectabilis ribosome inactivating protein (RIP) gene, complete cds	Q09EH2|Q09EH2_9CARY:rRNA N-glycosidase;OS=Bougainvillea spectabilis.;|GO:0030598,|GO:0006952, GO:0017148,
DN123945_c0_g2_i1	UP	[GO:0003676:nucleic acid binding][GO:0000166:nucleotide binding]	[ko05130:Pathogenic Escherichia coli infection]	CCG030364.1:Populus euphratica|NF-YA|NF-YA family protein	XP_021760134.1:RNAbinding protein CP29B, chloroplasticlike [Chenopodium quinoa]	-	A0A0J8BYC6|A0A0J8BYC6_BETVU: Uncharacterized protein;OS=Beta vulgaris subsp. vulgaris.;|GO:0003676,GO:0000166,

geneid	trend	GO	Pathway	TF	NR	NT	Uniprot
DN127281_c1_g1_i6	UP	[GO:0005524:ATP binding][GO:0019538:protein metabolic process]	-	Achn116231:Actinidia chinensis|ARF|ARF family protein	XP_010678345.1:PREDICTED: ATPdependent Clp protease ATPbinding subunit ClpA homolog CD4B, chloroplastic [Beta vulgaris subsp. vulgaris] >KMT10922.1 hypothetical protein BVRB_5g113350 [Beta vulgaris subsp. vulgaris]	gi|4105130|gb|AF043539.1|AF043539:Spinacia oleracea ClpC protease (clpC) mRNA, chloroplast gene encoding chloroplast protein, complete cds	A0A0J8CBD9|A0A0J8CBD9_BETVU: Uncharacterized protein;OS=Beta vulgaris subsp. vulgaris.;|GO:0005524,|GO:0019538,
DN123687_c3_g2_i1	UP	[GO:0005622:intracellular][GO:0005525:GTP binding][GO:0003924:GTPase activity][GO:0003746:translation elongation factor activity]	-	-	XP_016187654.1:elongation factor Tu, chloroplastic [Arachis ipaensis]	gi|459238|dbj|D11469.1|TOBTUFA2:Nicotiana sylvestris tufA gene for chloroplast elongation factor TuA, complete cds	A0A067K4G2|A0A067K4G2_JATCU:Elongation factor Tu;OS=Jatropha curcas;|GO:0005622,|GO:0005525,GO:0003924,GO:0003746,

geneid	trend	GO	Pathway	TF	NR	NT	Uniprot
DN126700_c 2_g1_i3	UP	[GO:0043565:sequence-specific DNA binding][GO:0003700:DNA-binding transcription factor activity]	-	Bv2_034240_wzwm .t2:Beta vulgaris|WRKY|WR KY family protein	KMT17893.1:hypot hetical protein BVRB_2g034240 isoform A [Beta vulgaris subsp. vulgaris]	-	A0A0J8D148|A0A0 J8D148_BETVU:Un characterized protein;OS=Beta vulgaris subsp. vulgaris.;|GO:00435 65,GO:0003700,
DN126525_c 1_g3_i3	UP	[GO:0003951:NAD+ kinase activity][GO:0019674:NAD metabolic process][GO:0006741:NADP biosynthetic process]	[ko00760:Nicotinate and nicotinamide metabolism]	AA31G00726:Aethi onema arabicum|STAT|ST AT family protein	XP_021760887.1:N AD kinase 2, chloroplasticlike [Chenopodium quinoa]	gi|359492365|ref|X M_002284571.2|:PR EDICTED: Vitis vinifera NAD kinase 2, chloroplastic-like (LOC100266161), mRNA	A0A0J8D1E1|A0A0 J8D1E1_BETVU:U ncharacterized protein;OS=Beta vulgaris subsp. vulgaris.;|GO:00039 51,|GO:0019674,GO :0006741,

geneid	trend	GO	Pathway	TF	NR	NT	Uniprot
DN110976_c0_g1_i1	UP	[GO:0009535:chloroplast thylakoid membrane][GO:0016021:integral component of membrane][GO:0009522:photosystem I][GO:0009523:photosystem II][GO:0016168:chlorophyll binding][GO:0046872:metal ion binding][GO:0009765:photosynthesis, light harvesting][GO:0018298:protein-chromophore linkage]	[ko00196:Photosynthesis - antenna proteins]	Bradi3g42627.3.p:Brachypodium distachyon|ERF|ERF family protein	XP_021732293.1:chlorophyll ab binding protein 7, chloroplasticlike isoform X2 [Chenopodium quinoa]	-	A0A0J8D0R7|A0A0J8D0R7_BETVU:Chlorophyll a-b binding protein, chloroplastic;OS=Beta vulgaris subsp. vulgaris.;|GO:0009535,GO:0016021,GO:0009522,GO:0009523,|GO:0016168,GO:0046872,|GO:0009765,GO:0018298,
DN116175_c2_g3_i2	UP	[GO:0005622:intracellular][GO:0005524:ATP binding][GO:0004707:MAP kinase activity]	[ko04016:MAPK signaling pathway - plant]	OGLUM05G28390.1:Oryza glumaepatula|HB-other|HB-other family protein	XP_021757551.1:mitogenactivated protein kinase homolog MMK2like [Chenopodium quinoa]	gi|521519390|gb|GAHZ01014449.1|:TSA: Salicornia europaea Unigene15111_SeCKS transcribed RNA sequence	A0A0J8CEU8|A0A0J8CEU8_BETVU:Mitogen-activated protein kinase;OS=Beta vulgaris subsp. vulgaris.;|GO:0005622,|GO:0005524,GO:0004707,

geneid	trend	GO	Pathway	TF	NR	NT	Uniprot
DN121346_c7_g7_i1	UP	[GO:0016020:membrane][GO:0046933:proton-transporting ATP synthase activity, rotational mechanism][GO:0015986:ATP synthesis coupled proton transport]	[ko00190:Oxidative phosphorylation][ko00195:Photosynthesis]	-	AJK93579.1:chloroplast ATP synthase delta [Suaeda glauca]	gi|521751211|gb|GAIA01000542.1|:TSA: Salicornia europaea Unigene552_Se200S transcribed RNA sequence	A0A0C5AX12|A0A0C5AX12_9CARY: Suaeda glauca chloroplast ATP synthase delta mRNA;OS=Suaeda glauca.;|GO:0016020,|GO:0046933,|GO:0015986,
DN128864_c3_g1_i5	UP	[GO:0030598:rRNA N-glycosylase activity][GO:0051607:defense response to virus][GO:0017148:negative regulation of translation]	-	-	Q40772.1:RecName: Full=Antiviral protein 2; AltName: Full=PAPII; AltName: Full=Ribosomeinactivating protein; AltName: Full=rRNA Nglycosidase; Flags: Precursor >CAA55342.1 pokeweed antiviral protein II [Phytolacca americana]	-	Q40772|RIP2_PHYAM:Antiviral protein 2;OS=Phytolacca americana;|GO:0030598,|GO:0051607,GO:0017148,

geneid	trend	GO	Pathway	TF	NR	NT	Uniprot
					>prf||2014245A antiviral protein II		
DN122597_c0_g2_i3	UP	[GO:0004794:L-threonine ammonia-lyase activity][GO:0030170:pyridoxal phosphate binding][GO:0008652:cellular amino acid biosynthetic process][GO:0009097:isoleucine biosynthetic process]	[ko00260:Glycine, serine and threonine metabolism][ko00290:Valine, leucine and isoleucine biosynthesis]	676765190:Sisymbrium irio|Trihelix|Trihelix family protein	XP_021857942.1:threonine dehydratase biosynthetic, chloroplastic isoform X2 [Spinacia oleracea] >KNA23108.1 hypothetical protein SOVF_027270 [Spinacia oleracea]	gi|359482291|ref|XM_002264275.2|:PREDICTED: Vitis vinifera threonine dehydratase biosynthetic, chloroplastic-like (LOC100248644), mRNA	A0A0K9RUH9|A0A0K9RUH9_SPIOL: Threonine dehydratase;OS=Spinacia oleracea;|GO:0004794,GO:0030170,|GO:0008652,GO:0009097,

geneid	trend	GO	Pathway	TF	NR	NT	Uniprot
DN114156_c0_g1_i1	UP	[GO:0016021:integral component of membrane]	-	Sme2.5_02290.1_g00006.1:Solanum melongena|GATA|GATA family protein	XP_010677139.1:PREDICTED: lightharvesting complexlike protein 3 isotype 2, chloroplastic [Beta vulgaris subsp. vulgaris] >KMT11824.1 hypothetical protein BVRB_5g105240 [Beta vulgaris subsp. vulgaris]	-	A0A0J8CI94|A0A0J8CI94_BETVU:Uncharacterized protein;OS=Beta vulgaris subsp. vulgaris.;|GO:0016021,
DN126525_c1_g8_i1	UP	[GO:0003951:NAD+ kinase activity][GO:0019674:NAD metabolic process][GO:0006741:NADP biosynthetic process]	[ko00760:Nicotinate and nicotinamide metabolism]	-	XP_021718190.1:NAD kinase 2, chloroplasticlike isoform X1 [Chenopodium quinoa]	gi|521457615|gb|GAHZ01062869.1|:TSA: Salicornia europaea Unigene75666_SeCKS transcribed RNA sequence	A0A0J8D1E1|A0A0J8D1E1_BETVU:Uncharacterized protein;OS=Beta vulgaris subsp. vulgaris.;|GO:0003951,|GO:0019674,GO:0006741,

geneid	trend	GO	Pathway	TF	NR	NT	Uniprot
DN122614_c3_g3_i6	UP	[GO:0005524:ATP binding][GO:0004849:uridine kinase activity][GO:0044211:CTP salvage][GO:0044206:UMP salvage]	[ko00240:Pyrimidine metabolism][ko00983:Drug metabolism - other enzymes]	-	XP_010676109.1:PREDICTED: uridine kinaselike protein 3 isoform X1 [Beta vulgaris subsp. vulgaris] >XP_019104740.1 PREDICTED: uridine kinaselike protein 3 isoform X1 [Beta vulgaris subsp. vulgaris]	gi|521452271|gb|GAHZ01068213.1|:TSA: Salicornia europaea Unigene82312_SeCKS transcribed RNA sequence	A0A0K9QBE8|A0A0K9QBE8_SPIOL: Uridine kinase;OS=Spinacia oleracea;|GO:0005524,GO:0004849,|GO:0044211,GO:0044206,
DN112114_c0_g1_i3	UP	-	-	-	XP_021859491.1:nonspecific lipidtransfer proteinlike protein At2g13820 isoform X1 [Spinacia oleracea]	-	A0A0K9R608|A0A0K9R608_SPIOL:Uncharacterized protein;OS=Spinacia oleracea;
DN126218_c3_g4_i4	UP	[GO:0006457:protein folding]	[ko04915:Estrogen signaling pathway]	-	XP_004510236.1:PREDICTED: peptidylprolyl cistrans isomerase FKBP62like [Cicer arietinum]	gi|255589765|ref|XM_002535035.1|:Ricinus communis peptidylprolyl isomerase, putative, mRNA	A0A067DFE2|A0A067DFE2_CITSI:Uncharacterized protein;OS=Citrus sinensis;|GO:0006457,

geneid	trend	GO	Pathway	TF	NR	NT	Uniprot
DN123624_c0_g3_i9	UP	[GO:0005840:ribosome][GO:0043022:ribosome binding][GO:0070569:uridylyltransferase activity][GO:0006364:rRNA processing]	-	Zpz_sc01241.1.g00120.1.am.mk:Zoysia pacifica|WRKY|WRKY family protein	XP_021860998.1:uncharacterized protein LOC110800013 [Spinacia oleracea]	gi|521670854|gb|GAIA01032097.1|:TSA: Salicornia europaea Unigene35227_Se200S transcribed RNA sequence	A0A0K9S1R3|A0A0K9S1R3_SPIOL:Uncharacterized protein;OS=Spinacia oleracea;|GO:0005840,|GO:0043022,GO:0070569,|GO:0006364,
DN120279_c6_g3_i1	UP	-	-	-	XP_021716270.1:LOW QUALITY PROTEIN: BURP domain protein RD22like [Chenopodium quinoa]	-	A0A0K9R0Z0|A0A0K9R0Z0_SPIOL:Uncharacterized protein;OS=Spinacia oleracea;
DN126451_c1_g6_i4	UP	[GO:0005524:ATP binding][GO:0004672:protein kinase activity]	-	-	XP_021754724.1:serine/threonineprotein kinase STN7, chloroplasticlike [Chenopodium quinoa]	-	A0A0K9QAH6|A0A0K9QAH6_SPIOL: Uncharacterized protein;OS=Spinacia oleracea;|GO:0005524,GO:0004672,

geneid	trend	GO	Pathway	TF	NR	NT	Uniprot
DN125802_c0_g1_i6	UP	[GO:0046872:metal ion binding][GO:0030001:metal ion transport]	-	-	XP_021605141.1:heavy metalassociated isoprenylated plant protein 39like [Manihot esculenta] >OAY56450.1 hypothetical protein MANES_02G017600 [Manihot esculenta]	-	A0A072TW71|A0A072TW71_MEDTR:Heavy metal transport/detoxification superfamily protein;OS=Medicago truncatula;GN=MTR_8g098915 {ECO:0000313|EMBL:KEH21133.1};|GO:0046872,|GO:0030001,
DN120248_c4_g1_i1	UP	[GO:0016021:integral component of membrane][GO:0009523:photosystem II][GO:0030145:manganese ion binding][GO:0015979:photosynthesis]	[ko00195:Photosynthesis]	-	XP_010250914.1:PREDICTED: photosystem II core complex proteins psbY, chloroplasticlike [Nelumbo nucifera]	gi|3337434|gb|AF060198.1|AF060198:Spinacia oleracea PsbY precursor (psbY) mRNA, nuclear gene encoding chloroplast protein, complete cds	A9PIV1|A9PIV1_9ROSI:Putative uncharacterized protein;OS=Populus trichocarpa x Populus deltoides.;|GO:0016021,GO:0009523,|GO:0030145,|GO:0015979,

geneid	trend	GO	Pathway	TF	NR	NT	Uniprot
DN128018_c4_g5_i1	UP	-	[ko04011:MAPK signaling pathway - yeast][ko04110:Cell cycle][ko04114:Oocyte meiosis][ko04151:PI3K-Akt signaling pathway][ko04390:Hippo signaling pathway][ko04391:Hippo signaling pathway - fly][ko04722:Neurotrophin signaling pathway][ko05169:Epstein-Barr virus infection][ko05203:Viral carcinogenesis]	GSBRNA2T00057732001:Brassica napus|E2F/DP|E2F/DP family protein	XP_010675565.1:PREDICTED: 1433 protein 1 [Beta vulgaris subsp. vulgaris] >KMT19765.1 hypothetical protein BVRB_1g008220 [Beta vulgaris subsp. vulgaris]	gi|3777446|emb|Y11688.1|:Solanum tuberosum mRNA for 14-3-3 protein, isolate 35G	A0A0J8D0V6|A0A0J8D0V6_BETVU:Uncharacterized protein;OS=Beta vulgaris subsp. vulgaris.;
DN126218_c3_g4_i8	UP	[GO:0006457:protein folding]	[ko04915:Estrogen signaling pathway]	Pahal.F00649.1:Panicum hallii|MYB_related|MYB_related family protein	KNA19433.1:hypothetical protein SOVF_061710 [Spinacia oleracea]	gi|478167275|gb|GAHK01009328.1|:TSA: Jatropha curcas CL3627.Contig1_JC-CK_1A transcribed RNA sequence	A0A0K9RIW2|A0A0K9RIW2_SPIOL:Uncharacterized protein;OS=Spinacia oleracea;|GO:0006457,

geneid	trend	GO	Pathway	TF	NR	NT	Uniprot
DN124890_c 1_g2_i4	UP	[GO:0004332:fructose-bisphosphate aldolase activity][GO:0006096:glycolytic process]	[ko00010:Glycolysis / Gluconeogenesis][ko000030:Pentose phosphate pathway][ko00051:Fructose and mannose metabolism][ko00680:Methane metabolism][ko00710:Carbon fixation in photosynthetic organisms]	FANhyb_rscf00000068.1.g00012.1:Fragaria x ananassa|FAR1|FAR1 family protein	XP_017619777.1:PREDICTED: fructosebisphosphate aldolase 1, chloroplasticlike [Gossypium arboreum] >KHG29854.1 putative fructosebisphosphate aldolase 2, chloroplastic like protein [Gossypium arboreum]	gi|521752072|gb|GAIA01000090.1|:TSA: Salicornia europaea Unigene93_Se200S transcribed RNA sequence	A0A0B0PZC7|A0A0B0PZC7_GOSAR: Fructose-bisphosphate aldolase;OS=Gossypium arboreum;|GO:0004332,|GO:0006096,
DN127245_c 1_g2_i1	UP	[GO:0016758:transferase activity, transferring hexosyl groups][GO:0008152:metabolic process]	-	AA31G00555:Aethionema arabicum|CO-like|CO-like family protein	XP_010666870.1:PREDICTED: UDPglycosyltransferase 90A1 [Beta vulgaris subsp. vulgaris] >KMS95780.1 hypothetical protein BVRB_005070 [Beta vulgaris subsp. vulgaris]	-	A0A0J8DY30|A0A0J8DY30_BETVU:Glycosyltransferase;OS=Beta vulgaris subsp. vulgaris.;|GO:0016758,|GO:0008152,

geneid	trend	GO	Pathway	TF	NR	NT	Uniprot
DN128351_c5_g5_i1	UP	[GO:0016021:integral component of membrane][GO:0009522:photosystem I][GO:0009783:photosystem II antenna complex][GO:0010287:plastoglobule][GO:0009517:PSII associated light-harvesting complex II][GO:0016168:chlorophyll binding][GO:0046872:metal ion binding][GO:0010196:nonphotochemical quenching][GO:0009765:photosynthesis, light harvesting][GO:0018298:protein-chromophore linkage]	[ko00196:Photosynthesis - antenna proteins]	Bradi3g42627.3.p:Brachypodium distachyon|ERF|ERF family protein	XP_010669870.1:PREDICTED: chlorophyll ab binding protein CP26, chloroplastic [Beta vulgaris subsp. vulgaris] >KMT20410.1 hypothetical protein BVRB_1g004060 [Beta vulgaris subsp. vulgaris]	gi|521742345|gb|GAIA01004996.1|:TSA: Salicornia europaea Unigene5283_Se200S transcribed RNA sequence	A0A0J8D2L3|A0A0J8D2L3_BETVU:Chlorophyll a-b binding protein, chloroplastic;OS=Beta vulgaris subsp. vulgaris.;|GO:0016021,GO:0009522,GO:0009783,GO:0010287,GO:0009517,|GO:0016168,GO:0046872,|GO:0010196,GO:0009765,GO:0018298,
DN112880_c0_g1_i1	UP	-	-	-	KNA25274.1:hypothetical protein SOVF_007160 [Spinacia oleracea]	-	A0A0K9S257|A0A0K9S257_SPIOL:Uncharacterized protein;OS=Spinacia oleracea;

geneid	trend	GO	Pathway	TF	NR	NT	Uniprot
DN122072_c0_g1_i6	UP	[GO:0006457:protein folding]	-	Pahal.F00649.1:Panicum hallii|MYB_related|MYB_related family protein	XP_015886778.1:PREDICTED: 70 kDa peptidylprolyl isomeraselike [Ziziphus jujuba]	-	A0A067KJY6|A0A067KJY6_JATCU:Uncharacterized protein;OS=Jatropha curcas;|GO:0006457,
DN118093_c0_g1_i1	UP	-	-	MDP0000315764:Malus domestica|M-type_MADS|M-type_MADS family protein	KNA17316.1:hypothetical protein SOVF_081060 [Spinacia oleracea]	gi|470136991|ref|XM_004304216.1|:PREDICTED: Fragaria vesca subsp. vesca UPF0301 protein Tfu_2389-like (LOC101304985), mRNA	A0A0K9RCS9|A0A0K9RCS9_SPIOL:Uncharacterized protein;OS=Spinacia oleracea;

geneid	trend	GO	Pathway	TF	NR	NT	Uniprot
DN119229_c3_g3_i8	UP	[GO:0016021:integral component of membrane][GO:0005524:ATP binding][GO:0004222:metalloendopeptidase activity]	-	FANhyb_rscf00001142.1.g00004.1:Fragaria x ananassa|C2H2|C2H2 family protein	XP_021732916.1:ATPdependent zinc metalloprotease FTSH 2, chloroplastic [Chenopodium quinoa] >XP_021732918.1 ATPdependent zinc metalloprotease FTSH 2, chloroplastic [Chenopodium quinoa] >XP_021732919.1 ATPdependent zinc metalloprotease FTSH 2, chloroplastic [Chenopodium quinoa]	gi|521490307|gb|GAHZ01040579.1|:TSA: Salicornia europaea Unigene45958_SeCKS transcribed RNA sequence	A0A0J8BKM2|A0A0J8BKM2_BETVU: Uncharacterized protein;OS=Beta vulgaris subsp. vulgaris.;|GO:0016021,|GO:0005524,GO:0004222,

geneid	trend	GO	Pathway	TF	NR	NT	Uniprot
DN120228_c1_g5_i2	UP	[GO:0005524:ATP binding][GO:0008974:phosphoribulokinase activity][GO:0005975:carbohydrate metabolic process]	[ko00710:Carbon fixation in photosynthetic organisms]	Niben101Scf12623g00009.1:Nicotiana benthamiana|BBR-BPC|BBR-BPC family protein	XP_010677417.1:PREDICTED: phosphoribulokinase, chloroplastic [Beta vulgaris subsp. vulgaris] >KMT11409.1 hypothetical protein BVRB_5g107370 [Beta vulgaris subsp. vulgaris]	gi|167265|gb|M73707.1|CIPPRK:Mesembryanthemum crystallinum phosphoribulokinase mRNA, complete CDS	A0A0J8CH28|A0A0J8CH28_BETVU:Phosphoribulokinase;OS=Beta vulgaris subsp. vulgaris.;|GO:0005524,GO:0008974,|GO:0005975,
DN125241_c4_g3_i1	UP	[GO:0009535:chloroplast thylakoid membrane][GO:0016021:integral component of membrane][GO:0009522:photosystem I][GO:0009523:photosystem II][GO:0016168:chlorophyll binding][GO:0009765:photosynthesis, light harvesting][GO:0018298:protein-chromophore linkage]	[ko00196:Photosynthesis - antenna proteins]	-	XP_015943384.1:chlorophyll ab binding protein CP24 10A, chloroplastic [Arachis duranensis] >XP_016179733.1 chlorophyll ab binding protein CP24 10A, chloroplastic [Arachis ipaensis]	gi|521490831|gb|GAHZ01040055.1|:TSA: Salicornia europaea Unigene45382_SeCKS transcribed RNA sequence	A0A151T249|A0A151T249_CAJCA:Chlorophyll a-b binding protein, chloroplastic;OS=Cajanus cajan;|GO:0009535,GO:0016021,GO:0009522,GO:0009523,|GO:0016168,|GO:0009765,GO:0018298,

geneid	trend	GO	Pathway	TF	NR	NT	Uniprot
DN119133_c3_g6_i10	UP	[GO:0003824:catalytic activity][GO:0008152:metabolic process]	[ko00280:Valine, leucine and isoleucine degradation][ko00640:Propanoate metabolism]	PH01003957G0030: Phyllostachys heterocycla|E2F/DP|E2F/DP family protein	XP_021714927.1:2oxoisovalerate dehydrogenase subunit beta 1, mitochondriallike [Chenopodium quinoa]	gi|388511998|gb|BT144267.1|:Medicago truncatula clone JCVI-FLMt-16M19 unknown mRNA	A0A166GT20|A0A166GT20_DAUCA:Uncharacterized protein;OS=Daucus carota subsp. sativus.;|GO:0003824,|GO:0008152,
DN126300_c1_g3_i1	UP	[GO:0016021:integral component of membrane]	-	-	XP_021731983.1:sulfite exporter TauE/SafE family protein 3like [Chenopodium quinoa]	gi|478154700|gb|GAHK01021903.1|:TSA: Jatropha curcas Unigene9361_JC-CK_1A transcribed RNA sequence	A0A0K9QKR8|A0A0K9QKR8_SPIOL: Uncharacterized protein;OS=Spinacia oleracea;|GO:0016021,
DN125386_c2_g1_i11	UP	[GO:0008374:O-acyltransferase activity][GO:0006629:lipid metabolic process]	-	Gh_D08G2402:Gossypium hirsutum|AP2|AP2 family protein	XP_021843458.1:phospholipase A(1) LCAT3 [Spinacia oleracea] >KNA18716.1 hypothetical protein SOVF_068220 [Spinacia oleracea]	-	A0A0K9RGT8|A0A0K9RGT8_SPIOL: Uncharacterized protein;OS=Spinacia oleracea;|GO:0008374,|GO:0006629,

geneid	trend	GO	Pathway	TF	NR	NT	Uniprot
DN128314_c2_g15_i2	UP	[GO:0003723:RNA binding]	-	-	XP_010686234.1:PREDICTED: uncharacterized protein LOC104900510 [Beta vulgaris subsp. vulgaris] >KMT04441.1 hypothetical protein BVRB_8g181150 [Beta vulgaris subsp. vulgaris]	-	A0A0J8EN71|A0A0J8EN71_BETVU:Uncharacterized protein;OS=Beta vulgaris subsp. vulgaris.;|GO:0003723,
DN123159_c1_g1_i2	UP	[GO:0009570:chloroplast stroma][GO:0005524:ATP binding][GO:0051536:iron-sulfur cluster binding][GO:0016226:iron-sulfur cluster assembly]	-	RrC216_p2:Raphanus raphanistrum|bHLH|bHLH family protein	XP_021764457.1:feS cluster assembly factor HCF101, chloroplasticlike [Chenopodium quinoa] >XP_021764458.1 feS cluster assembly factor HCF101, chloroplasticlike [Chenopodium quinoa]	gi|521578103|gb|GAIA01069978.1|:TSA: Salicornia europaea Unigene84255_Se200S transcribed RNA sequence	A0A0J8EJ83|A0A0J8EJ83_BETVU:Uncharacterized protein;OS=Beta vulgaris subsp. vulgaris.;|GO:0009570,|GO:0005524,GO:0051536,|GO:0016226,

geneid	trend	GO	Pathway	TF	NR	NT	Uniprot
DN122536_c2_g1_i1	UP	-	-	-	-	-	-
DN124317_c1_g1_i1	UP	[GO:0009941:chloroplast envelope][GO:0009570:chloroplast stroma][GO:0005525:GTP binding][GO:0003924:GTPase activity][GO:0003743:translation initiation factor activity]	-	Gh_D02G0276:Gossypium hirsutum|GRAS|GRAS family protein	XP_021718820.1:translation initiation factor IF2, chloroplasticlike [Chenopodium quinoa]	gi|356556944|ref|XM_003546732.1|:PREDICTED: Glycine max translation initiation factor IF-2, chloroplastic-like (LOC100778924), mRNA	A0A0J8B9V5|A0A0J8B9V5_BETVU:Uncharacterized protein;OS=Beta vulgaris subsp. vulgaris.;|GO:0009941,GO:0009570,|GO:0005525,GO:0003924,GO:0003743,
DN123859_c0_g4_i17	UP	[GO:0003873:6-phosphofructo-2-kinase activity][GO:0005524:ATP binding][GO:2001070:starch binding][GO:0006003:fructose 2,6-bisphosphate metabolic process][GO:0006000:fructose metabolic process]	[ko00051:Fructose and mannose metabolism][ko04066:HIF-1 signaling pathway][ko04152:AMPK signaling pathway]	-	XP_002276394.1:PREDICTED: 6phosphofructo2kinase/fructose2,6bisphosphatase [Vitis vinifera] >CBI15923.3 unnamed protein product, partial [Vitis vinifera]	gi|3170229|gb|AF041848.1|:Spinacia oleracea fructose-6-phosphate 2-kinase /fructose-2,6-bisphosphatase mRNA, complete cds	D7SJU7|D7SJU7_VITVI:Putative uncharacterized protein;OS=Vitis vinifera;GN=VIT_06s0004g06920 {ECO:0000313|EMBL:CBI15923.3};|GO:0003873,GO:0005524,GO:2001070,|GO:0006003,GO:0006000,

geneid	trend	GO	Pathway	TF	NR	NT	Uniprot
DN123764_c2_g2_i1	UP	[GO:0016021:integral component of membrane][GO:0016844:strictosidine synthase activity][GO:0009058:biosynthetic process]	-	-	XP_010666852.1:PREDICTED: protein STRICTOSIDINE SYNTHASELIKE 4 [Beta vulgaris subsp. vulgaris] >KMS95797.1 hypothetical protein BVRB_004960 [Beta vulgaris subsp. vulgaris]	-	A0A0J8B7N3|A0A0J8B7N3_BETVU:Uncharacterized protein;OS=Beta vulgaris subsp. vulgaris.;|GO:0016021,|GO:0016844,|GO:0009058,
DN127561_c0_g3_i3	UP	[GO:0005829:cytosol][GO:0009506:plasmodesma][GO:0003939:L-iditol 2-dehydrogenase activity][GO:0008270:zinc ion binding]	[ko00040:Pentose and glucuronate interconversions][ko00051:Fructose and mannose metabolism]	-	XP_010693460.1:PREDICTED: sorbitol dehydrogenase [Beta vulgaris subsp. vulgaris] >KMS99016.1 hypothetical protein BVRB_3g066230 [Beta vulgaris subsp. vulgaris]	gi|14699999|gb|AY037946.1|:Prunus cerasus sorbitol dehydrogenase mRNA, complete cds	A0A0J8BDQ4|A0A0J8BDQ4_BETVU:Uncharacterized protein;OS=Beta vulgaris subsp. vulgaris.;|GO:0005829,GO:0009506,|GO:0003939,GO:0008270,

geneid	trend	GO	Pathway	TF	NR	NT	Uniprot
DN126029_c1_g2_i1	UP	-	-	Achn002631:Actinidia chinensis|C2H2|C2H2 family protein	XP_021748517.1:uncharacterized protein LOC110714323 isoform X1 [Chenopodium quinoa]	-	A0A0J8EAL8|A0A0J8EAL8_BETVU:Uncharacterized protein;OS=Beta vulgaris subsp. vulgaris.;
DN114711_c1_g2_i2	UP	[GO:0051537:2 iron, 2 sulfur cluster binding][GO:0010277:chlorophyllide a oxygenase [overall] activity][GO:0005506:iron ion binding][GO:0016705:oxidoreductase activity, acting on paired donors, with incorporation or reduction of molecular oxygen]	[ko00860:Porphyrin and chlorophyll metabolism]	ORUFI10G20190.1:Oryza rufipogon|STAT|STAT family protein	XP_021743712.1:chlorophyllide a oxygenase, chloroplasticlike [Chenopodium quinoa]	gi|359492577|ref|XM_002284463.2|:PREDICTED: Vitis vinifera chlorophyllide a oxygenase, chloroplastic-like (LOC100247245), mRNA	A0A0K9RYA3|A0A0K9RYA3_SPIOL:Uncharacterized protein;OS=Spinacia oleracea;|GO:0051537,GO:0010277,GO:0005506,GO:0016705,
DN122026_c4_g1_i6	UP	[GO:0005783:endoplasmic reticulum][GO:0016021:integral component of membrane][GO:0046923:ER retention sequence binding][GO:0006621:protein retention in ER lumen]	-	Peaxi162Scf01312g00064.1:Petunia axillaris|M-type_MADS|M-type_MADS family protein	XP_021736929.1:putative ER lumen proteinretaining receptor C28H8.4 isoform X1 [Chenopodium quinoa]	-	A0A0J8B7X2|A0A0J8B7X2_BETVU:Uncharacterized protein;OS=Beta vulgaris subsp. vulgaris.;|GO:0005783,GO:0016021,|GO:0046923,|GO:00066

geneid	trend	GO	Pathway	TF	NR	NT	Uniprot
							21,

geneid	trend	GO	Pathway	TF	NR	NT	Uniprot
DN125998_c0_g2_i2	UP	[GO:0005737:cytoplasm][GO:0005634:nucleus][GO:0005524:ATP binding][GO:0009882:blue light photoreceptor activity][GO:0004672:protein kinase activity][GO:0046283:anthocyanin-containing compound metabolic process][GO:0010617:circadian regulation of calcium ion oscillation][GO:0009583:detection of light stimulus][GO:0055114:oxidation-reduction process][GO:0009640:photomorphogenesis][GO:0046777:protein autophosphorylation][GO:0010075:regulation of meristem growth][GO:0051510:regulation of unidimensional cell growth][GO:0009414:response to water deprivation][GO:0010343:singlet oxygen-mediated programmed cell death][GO:0010118:stomatal movement]	[ko04712:Circadian rhythm - plant]	KZV28906.1:Dorcoceras hygrometricum|C2H2|C2H2 family protein	XP_021740218.1:cryptochrome1like isoform X2 [Chenopodium quinoa] >XP_021740220.1 cryptochrome1like isoform X3 [Chenopodium quinoa]	gi|521745052|gb|GAIA01003475.1|:TSA: Salicornia europaea Unigene3688_Se200S transcribed RNA sequence	A0A0J8D131|A0A0J8D131_BETVU:Uncharacterized protein;OS=Beta vulgaris subsp. vulgaris.;|GO:0005737,GO:0005634,|GO:0005524,GO:0009882,GO:0004672,|GO:0046283,GO:0010617,GO:0009583,GO:0055114,GO:0009640,GO:0046777,GO:0010075,GO:0051510,GO:0009414,GO:0010343,GO:0010118,

geneid	trend	GO	Pathway	TF	NR	NT	Uniprot
DN128939_c4_g2_i1	UP	[GO:0016788:hydrolase activity, acting on ester bonds]	-	-	XP_010685239.1:PREDICTED: GDSL esterase/lipase 2like [Beta vulgaris subsp. vulgaris]	-	A0A0J8BXN2|A0A0J8BXN2_BETVU: Uncharacterized protein;OS=Beta vulgaris subsp. vulgaris.;|GO:0016788,
DN125678_c1_g1_i1	UP	[GO:0016021:integral component of membrane][GO:0005215:transporter activity]	-	Gh_D11G2978:Gossypium hirsutum|STAT|STAT family protein	XP_021765723.1:probable purine permease 11 [Chenopodium quinoa]	gi|147854852|emb|AM444518.2|:Vitis vinifera contig VV78X022735.4, whole genome shotgun sequence	A0A0J8F705|A0A0J8F705_BETVU:Uncharacterized protein;OS=Beta vulgaris subsp. vulgaris.;|GO:0016021,|GO:0005215,
DN128943_c5_g1_i5	UP	[GO:0016021:integral component of membrane][GO:0005524:ATP binding][GO:0004674:protein serine/threonine kinase activity][GO:0019199:transmembrane receptor protein kinase activity]	[ko04016:MAPK signaling pathway - plant][ko04075:Plant hormone signal transduction][ko04626:Plant-pathogen interaction]	KZV24601.1:Dorcoceras hygrometricum|Trihelix|Trihelix family protein	AIT39752.1:brassinosteroid insensitive 1associated receptor kinase 1, partial [Chrysanthemum boreale]	gi|521664252|gb|GAIA01035129.1|:TSA: Salicornia europaea Unigene38891_Se200S transcribed RNA sequence	A0A0A0QTG7|A0A0A0QTG7_9ASTR: Brassinosteroid insensitive 1-associated receptor kinase 1;OS=Chrysanthemum boreale.;|GO:0016021,|GO:0005524,GO:0004674,GO:001919

geneid	trend	GO	Pathway	TF	NR	NT	Uniprot
							9,
DN125200_c 3_g1_i8	UP	[GO:0004722:protein serine/threonine phosphatase activity]	-	KZV22762.1:Dorco ceras hygrometricum|CA MTA|CAMTA family protein	XP_010669220.1:P REDICTED: probable protein phosphatase 2C 12 [Beta vulgaris subsp. vulgaris] >KMT17976.1 hypothetical protein BVRB_2g033220 [Beta vulgaris subsp. vulgaris]	gi|3643087|gb|AF07 5581.1|AF075581:M esembryanthemum crystallinum clone Mpc6 protein phosphatase-2C (PP2C) mRNA, complete cds	A0A0J8FQC8|A0A0 J8FQC8_BETVU:U ncharacterized protein;OS=Beta vulgaris subsp. vulgaris.;|GO:00047 22,
DN127822_c 4_g2_i4	UP	-	-	-	XP_021737918.1:un characterized aarF domaincontaining protein kinase At5g05200, chloroplasticlike	gi|359486321|ref|X M_002275429.2|:PR EDICTED: Vitis vinifera uncharacterized LOC100243380	A0A0K9RA91|A0A 0K9RA91_SPIOL:U ncharacterized protein;OS=Spinacia oleracea;

geneid	trend	GO	Pathway	TF	NR	NT	Uniprot
					[Chenopodium quinoa]	(LOC100243380), mRNA	
DN121330_c5_g6_i4	UP	-	[ko05020:Prion diseases]	Pavir.8NG308000.1.p:Panicum virgatum|C3H|C3H family protein	XP_010539811.1:PREDICTED: hsp70Hsp90 organizing protein 3 [Tarenaya hassleriana]	gi|521705420|gb|GAIA01015228.1|:TSA: Salicornia europaea Unigene15902_Se200S transcribed RNA sequence	A0A067JNR3|A0A067JNR3_JATCU:Uncharacterized protein;OS=Jatropha curcas;
DN116332_c1_g1_i3	UP	-	-	-	-	-	-
DN126983_c6_g12_i1	UP	[GO:0008171:O-methyltransferase activity]	[ko00940:Phenylpropanoid biosynthesis]	-	XP_018504010.1:PREDICTED: caffeic acid 3Omethyltransferase like [Pyrus x bretschneideri]	-	C0L8A9|C0L8A9_BETPN:Putative caffeic acid O-methyltransferase;OS=Betula pendula;|GO:0008171,

geneid	trend	GO	Pathway	TF	NR	NT	Uniprot
DN111425_c0_g1_i6	UP	-	-	-	BAE00072.1:PGR5 [Amaranthus hybridus]	gi|521652535|gb|GAIA01040471.1|:TSA: Salicornia europaea Unigene45470_Se200S transcribed RNA sequence	Q4R1B3|Q4R1B3_AMAHY:PGR5;OS=Amaranthus hybridus;
DN128504_c1_g4_i4	UP	[GO:0019898:extrinsic component of membrane][GO:0016021:integral component of membrane][GO:0009654:photosystem II oxygen evolving complex][GO:0005509:calcium ion binding][GO:0015979:photosynthesis][GO:0042549:photosystem II stabilization]	[ko00195:Photosynthesis]	Dca27722.1:Dianthus caryophyllus|Nin-like|Nin-like family protein	XP_010673597.1:PREDICTED: oxygenevolving enhancer protein 1, chloroplastic [Beta vulgaris subsp. vulgaris] >KMT14449.1 hypothetical protein BVRB_4g072250 [Beta vulgaris subsp. vulgaris]	-	A0A0J8CR03|A0A0J8CR03_BETVU:Uncharacterized protein;OS=Beta vulgaris subsp. vulgaris.;|GO:0019898,GO:0016021,GO:0009654,|GO:0005509,|GO:0015979,GO:0042549,
DN124060_c1_g2_i1	UP	-	-	-	KHN33810.1:hypothetical protein glysoja_013816 [Glycine soja]	gi|512399259|gb|GAJZ01001911.1|:TSA: Prunus avium Pa_03809 transcribed RNA sequence	A0A0B2RKU8|A0A0B2RKU8_GLYSO:Uncharacterized protein;OS=Glycine soja;

geneid	trend	GO	Pathway	TF	NR	NT	Uniprot
DN119479_c0_g2_i2	UP	[GO:0016630:protochlorophyllide reductase activity]	-	TRIUR3_22095-P1: Triticum urartu|C2H2|C2H2 family protein	XP_021717319.1:protochlorophyllide reductaselike [Chenopodium quinoa]	gi|512396271|gb|GAJZ01004899.1|:TSA: Prunus avium Pa_12084 transcribed RNA sequence	G5DVX6|G5DVX6_SILLA:Protochlorophyllide reductase B;OS=Silene latifolia;|GO:0016630,
DN127698_c3_g1_i2	UP	[GO:0005783:endoplasmic reticulum][GO:0016021:integral component of membrane][GO:0016747:transferase activity, transferring acyl groups other than amino-acyl groups][GO:0006633:fatty acid biosynthetic process]	[ko00062:Fatty acid elongation]	-	XP_021746428.1:3ketoacylCoA synthase 10like [Chenopodium quinoa]	gi|28864533|gb|AY206684.1|:Tropaeolum majus fiddlehead-like protein mRNA, complete cds	A0A0J8BH71|A0A0J8BH71_BETVU:3-ketoacyl-CoA synthase;OS=Beta vulgaris subsp. vulgaris.;|GO:0005783,GO:0016021,|GO:0016747,|GO:0006633,
DN123299_c1_g1_i3	UP	[GO:0008270:zinc ion binding]	-	-	XP_021836415.1:protein NCA1 [Spinacia oleracea]	gi|521535449|gb|GAHZ01005739.1|:TSA: Salicornia europaea Unigene6032_SeCKS transcribed RNA sequence	A0A0K9RUP9|A0A0K9RUP9_SPIOL:Uncharacterized protein;OS=Spinacia oleracea;|GO:00082 70,

geneid	trend	GO	Pathway	TF	NR	NT	Uniprot
DN127270_c2_g1_i1	UP	[GO:0005622:intracellular][GO:0008270:zinc ion binding]	-	CCG032314.1:Populus euphratica|DBB|DBB family protein	GAV74446.1:zfB_box domaincontaining protein [Cephalotus follicularis]	gi|312032523|gb|AC243090.1|:Gossypioides kirkii clone GKH030A02-jgz, complete sequence	A0A0J8BE95|A0A0J8BE95_BETVU:Uncharacterized protein;OS=Beta vulgaris subsp. vulgaris.;|GO:0005622,|GO:0008270,
DN127261_c2_g11_i2	UP	[GO:0016021:integral component of membrane]	-	-	XP_021772984.1:LOW QUALITY PROTEIN: UPF0603 protein At1g54780, chloroplasticlike [Chenopodium quinoa]	gi|521750899|gb|GAIA01000699.1|:TSA: Salicornia europaea Unigene712_Se200S transcribed RNA sequence	A0A0K9QB22|A0A0K9QB22_SPIOL:Uncharacterized protein;OS=Spinacia oleracea;|GO:0016021,
DN116652_c1_g1_i1	UP	[GO:0016021:integral component of membrane][GO:0008270:zinc ion binding]	-	Zmw_sc01355.1.g00120.1:Zoysia matrella|bHLH|bHLH family protein	XP_006429572.1:hypothetical protein CICLE_v10013579mg, partial [Citrus clementina] >ESR42812.1 hypothetical protein CICLE_v10013579mg, partial [Citrus clementina]	-	V4SQ47|V4SQ47_9ROSI:Uncharacterized protein;OS=Citrus clementina.;|GO:0016021,|GO:0008270,

geneid	trend	GO	Pathway	TF	NR	NT	Uniprot
DN121268_c3_g3_i5	UP	[GO:0046872:metal ion binding][GO:0030001:metal ion transport]	-	-	XP_012841611.1:PREDICTED: pollenspecific leucinerich repeat extensinlike protein 1 [Erythranthe guttata]	-	A0A0D2T0C7|A0A0D2T0C7_GOSRA: Uncharacterized protein;OS=Gossypium raimondii;|GO:0046872,|GO:0030001,
DN125455_c3_g2_i15	UP	[GO:0005739:mitochondrion][GO:0050897:cobalt ion binding][GO:0016874:ligase activity][GO:0008270:zinc ion binding][GO:0006552:leucine catabolic process]	[ko00280:Valine, leucine and isoleucine degradation]	CCG019154.1:Populus euphratica|bZIP|bZIP family protein	XP_021854838.1:methylcrotonoylCoA carboxylase beta chain, mitochondrial [Spinacia oleracea]	gi|359475366|ref|XM_002282231.2|:PREDICTED: Vitis vinifera methylcrotonoyl-CoA carboxylase beta chain, mitochondrial-like (LOC100253805), mRNA	A0A0J8BGX7|A0A0J8BGX7_BETVU: Uncharacterized protein;OS=Beta vulgaris subsp. vulgaris.;|GO:0005739,|GO:0050897,GO:0016874,GO:0008270,|GO:0006552,
DN119798_c0_g6_i1	UP	[GO:0003682:chromatin binding][GO:0008270:zinc ion binding]	-	mrna00537.1-v1.0-hybrid:Fragaria vesca|HB-other|HB-other family protein	XP_021766828.1:chromatin remodeling protein EBSlike [Chenopodium quinoa] >XP_021719749.1 chromatin remodeling protein	gi|478158215|gb|GAHK01018388.1|:TSA: Jatropha curcas Unigene5842_JC-CK_1A transcribed RNA sequence	A0A0K9Q9Q8|A0A0K9Q9Q8_SPIOL:Uncharacterized protein;OS=Spinacia oleracea;|GO:0003682,GO:0008270,

geneid	trend	GO	Pathway	TF	NR	NT	Uniprot
					EBSlike [Chenopodium quinoa]		
DN120417_c1_g1_i2	UP	[GO:0009570:chloroplast stroma][GO:0009535:chloroplast thylakoid membrane][GO:0004324:ferredoxin-NADP+ reductase activity][GO:0015979:photosynthesis]	[ko00195:Photosynthesis]	GRMZM2G320305_P01:Zea mays|MYB_related|MYB_related family protein	P41343.1:RecName: Full=FerredoxinNADP reductase, chloroplastic; Short=FNR; Flags: Precursor >AAA33029.1 ferredoxinNADP+ reductase precursor [Mesembryanthemum crystallinum] >prf||1604475A ferredoxin NADP reductase	gi|167255|gb|M25528.1|CIPFNRA:Mesembryanthemum crystallinum ferredoxin-NADP+ reductase precursor, mRNA, complete cds	P41343|FENR_MESCR:Ferredoxin--NADP reductase, chloroplastic;OS=Mesembryanthemum crystallinum;|GO:0009570,GO:0009535,|GO:0004324,|GO:0015979,
DN127248_c0_g1_i5	UP	[GO:0016021:integral component of membrane]	[ko00900:Terpenoid backbone biosynthesis]	Pahal.C00083.1:Panicum hallii|G2-like|G2-like family protein	XP_021746078.1:farnesol kinase, chloroplasticlike [Chenopodium quinoa]	gi|478161680|gb|GAHK01014923.1|:TSA: Jatropha curcas Unigene2377_JC-CK_1A transcribed	A0A0J8BQB9|A0A0J8BQB9_BETVU: Uncharacterized protein;OS=Beta vulgaris subsp.

geneid	trend	GO	Pathway	TF	NR	NT	Uniprot
						RNA sequence	vulgaris.;|GO:0016021,
DN122903_c2_g1_i2	UP	-	-	GSMUA_Achr10P17130_001:Musa acuminata|bHLH|bHLH family protein	XP_021839366.1:chaperone protein dnaJ 11, chloroplasticlike [Spinacia oleracea] >KNA20631.1 hypothetical protein SOVF_050590 [Spinacia oleracea]	-	A0A0K9RMA3|A0A0K9RMA3_SPIOL:Uncharacterized protein;OS=Spinacia oleracea;
DN126838_c0_g1_i1	UP	[GO:0015930:glutamate synthase activity][GO:0006537:glutamate biosynthetic process]	[ko00630:Glyoxylate and dicarboxylate metabolism][ko00910:Nitrogen metabolism]	421270:Selaginella moellendorffii|C3H|C3H family protein	AFH78197.1:glutamate synthase [Beta vulgaris subsp. vulgaris]	gi|414978|gb|U03006.1|SOGOGAT:Spinacia oleracea ferredoxin-dependent glutamate synthase mRNA, partial cds	J7I3K0|J7I3K0_BETVU:Glutamate synthase;OS=Beta vulgaris;|GO:0015930,|GO:0006537,
DN117000_c0_g1_i2	UP	[GO:0016788:hydrolase activity, acting on ester bonds]	-	-	XP_021720221.1:GDSL esterase/lipase At3g48460like [Chenopodium quinoa]	-	A0A0J8B867|A0A0J8B867_BETVU:Uncharacterized protein;OS=Beta vulgaris subsp. vulgaris.;|GO:00167

geneid	trend	GO	Pathway	TF	NR	NT	Uniprot
							88,

geneid	trend	GO	Pathway	TF	NR	NT	Uniprot
DN124625_c0_g2_i9	UP	[GO:0046872:metal ion binding][GO:0004721:phosphoprotein phosphatase activity]	[ko03015:mRNA surveillance pathway][ko04022:cGMP-PKG signaling pathway][ko04024:cAMP signaling pathway][ko04113:Meiosis - yeast][ko04114:Oocyte meiosis][ko04218:Cellular senescence][ko04261:Adrenergic signaling in cardiomyocytes][ko04270:Vascular smooth muscle contraction][ko04390:Hippo signaling pathway][ko04510:Focal adhesion][ko04611:Platelet activation][ko04720:Long-term potentiation][ko04728:Dopaminergic synapse][ko04750:Inflammatory mediator regulation of TRP	Jcr4S06752.20:Jatropha curcas|FAR1|FAR1 family protein	XP_010676401.1:PREDICTED: serine/threonineprotein phosphatase PP1 [Beta vulgaris subsp. vulgaris] >KMT12017.1 hypothetical protein BVRB_5g099770 [Beta vulgaris subsp. vulgaris]	gi|521657003|gb|GAIA01038927.1|:TSA: Salicornia europaea Unigene43565_Se200S transcribed RNA sequence	A0A0J8CET1|A0A0J8CET1_BETVU:Serine/threonine-protein phosphatase;OS=Beta vulgaris subsp. vulgaris.;|GO:0046872,GO:0004721,

geneid	trend	GO	Pathway	TF	NR	NT	Uniprot
DN128685_c1_g3_i1	UP	[GO:0016620:oxidoreductase activity, acting on the aldehyde or oxo group of donors, NAD or NADP as acceptor]	[ko00010:Glycolysis / Gluconeogenesis][ko000030:Pentose phosphate pathway]	MDP0000606021:Malus domestica|bHLH|bHLH family protein	XP_010666027.1:PREDICTED: NADPdependent glyceraldehyde3phosphate dehydrogenase [Beta vulgaris subsp. vulgaris] >KMT19037.1 hypothetical protein BVRB_2g031320 [Beta vulgaris subsp. vulgaris]	gi|359475839|ref|XM_002285250.2|:PREDICTED: Vitis vinifera NADP-dependent glyceraldehyde-3-phosphate dehydrogenase-like (LOC100249114), mRNA	A0A0J8CZP0|A0A0J8CZP0_BETVU:Uncharacterized protein;OS=Beta vulgaris subsp. vulgaris.;|GO:0016620,
DN128314_c2_g4_i1	UP	[GO:0003723:RNA binding]	-	kfl00101_0290:Klebsormidium flaccidum|C3H|C3H family protein	XP_021720568.1:YTH domaincontaining family protein 2like isoform X3 [Chenopodium quinoa]	gi|521650865|gb|GAIA01041630.1|:TSA: Salicornia europaea Unigene46772_Se200S transcribed RNA sequence	A0A0J8EN71|A0A0J8EN71_BETVU:Uncharacterized protein;OS=Beta vulgaris subsp. vulgaris.;|GO:0003723,
DN118197_c0_g1_i1	UP	[GO:0008171:O-methyltransferase activity]	-	orange1.1g044941m:Citrus sinensis|C3H|C3H family protein	XP_021715143.1:caffeic acid 3Omethyltransferase like [Chenopodium quinoa]	-	A0A0K9RJQ9|A0A0K9RJQ9_SPIOL:Uncharacterized protein;OS=Spinacia oleracea;|GO:00081

geneid	trend	GO	Pathway	TF	NR	NT	Uniprot
							71,
DN126055_c 2_g1_i2	UP	-	-	-	-	-	-
DN124060_c 1_g11_i11	UP	-	-	-	XP_010692820.1:PREDICTED: uncharacterized protein LOC104905876 isoform X1 [Beta vulgaris subsp. vulgaris] >KMS99550.1 hypothetical protein BVRB_1g022780 isoform A [Beta vulgaris subsp. vulgaris]	gi|521670140|gb|GAIA01032456.1|:TSA: Salicornia europaea Unigene35617_Se200S transcribed RNA sequence	A0A0J8BF49|A0A0J8BF49_BETVU:Uncharacterized protein;OS=Beta vulgaris subsp. vulgaris.;

geneid	trend	GO	Pathway	TF	NR	NT	Uniprot
DN123080_c2_g3_i2	UP	[GO:0016868:intramolecular transferase activity, phosphotransferases][GO:0000287:magnesium ion binding][GO:0005975:carbohydrate metabolic process]	[ko00010:Glycolysis / Gluconeogenesis][ko00030:Pentose phosphate pathway][ko00052:Galactose metabolism][ko00230:Purine metabolism][ko00500:Starch and sucrose metabolism][ko00520:Amino sugar and nucleotide sugar metabolism][ko00521:Streptomycin biosynthesis]	-	KNA24627.1:hypothetical protein SOVF_014010 [Spinacia oleracea]	gi|225455656|ref|XM_002263777.1|:PREDICTED: Vitis vinifera phosphoglucomutase, chloroplastic-like (LOC100247590), mRNA	A0A0K9S0J9|A0A0K9S0J9_SPIOL:Uncharacterized protein;OS=Spinacia oleracea;|GO:0016868,GO:0000287,|GO:0005975,

geneid	trend	GO	Pathway	TF	NR	NT	Uniprot
DN126292_c0_g1_i5	UP	[GO:0009506:plasmodesma][GO:0003682:chromatin binding]	-	-	XP_010681574.1:PREDICTED: uncharacterized protein LOC104896520 isoform X1 [Beta vulgaris subsp. vulgaris] >KMT08292.1 hypothetical protein BVRB_6g142160 isoform B [Beta vulgaris subsp. vulgaris]	gi|478158569|gb|GAHK01018034.1|:TSA: Jatropha curcas Unigene5488_JC-CK_1A transcribed RNA sequence	A0A0J8C407|A0A0J8C407_BETVU:Uncharacterized protein;OS=Beta vulgaris subsp. vulgaris.;|GO:0009506,|GO:0003682,

geneid	trend	GO	Pathway	TF	NR	NT	Uniprot
DN114384_c1_g1_i1	UP	[GO:0005524:ATP binding][GO:0004356:glutamate-ammonia ligase activity][GO:0006542:glutamine biosynthetic process]	[ko00220:Arginine biosynthesis][ko00250:Alanine, aspartate and glutamate metabolism][ko00630:Glyoxylate and dicarboxylate metabolism][ko00910:Nitrogen metabolism][ko02020:Two-component system][ko04217:Necroptosis][ko04724:Glutamatergic synapse][ko04727:GABAergic synapse]	-	AAD31898.1:glutamine synthetase leaf isozyme precursor [Mesembryanthemum crystallinum]	gi|4884969|gb|AF145480.1|AF145480:Mesembryanthemum crystallinum glutamine synthetase leaf isozyme precursor, mRNA, complete cds	Q9XFJ1|Q9XFJ1_MESCR:Glutamine synthetase;OS=Mesembryanthemum crystallinum;|GO:0005524,GO:0004356,|GO:0006542,
DN127161_c3_g1_i17	UP	-	-	Tp1g05870:Thellungiella parvula|MYB_related|MYB_related family protein	XP_021721284.1:protein CHUP1, chloroplasticlike [Chenopodium quinoa] >XP_021721285.1 protein CHUP1, chloroplasticlike	gi|255566820|ref|XM_002524348.1|:Ricinus communis conserved hypothetical protein, mRNA	A0A0J8CDG4|A0A0J8CDG4_BETVU: Uncharacterized protein;OS=Beta vulgaris subsp. vulgaris.;

geneid	trend	GO	Pathway	TF	NR	NT	Uniprot
					[Chenopodium quinoa]		
DN122471_c0_g1_i10	UP	[GO:0008661:1-deoxy-D-xylulose-5-phosphate synthase activity][GO:0016114:terpenoid biosynthetic process]	[ko00730:Thiamine metabolism][ko00900:Terpenoid backbone biosynthesis]	-	XP_010666332.1:PREDICTED: probable 1deoxyDxylulose5phosphate synthase, chloroplastic [Beta vulgaris subsp. vulgaris] >KMS96169.1 hypothetical protein BVRB_001500 [Beta vulgaris subsp. vulgaris]	gi|386081791|gb|JQ710678.1|:Withania somnifera chloroplast 1-deoxy-D-xylulose-5-phosphate synthase (DXS) mRNA, complete cds; nuclear gene for chloroplast product	A0A0J8B4P2|A0A0J8B4P2_BETVU:Uncharacterized protein;OS=Beta vulgaris subsp. vulgaris.;|GO:0008661,|GO:0016114,

geneid	trend	GO	Pathway	TF	NR	NT	Uniprot
DN120304_c 1_g1_i1	UP	-	[ko04068:FoxO signaling pathway][ko04110: Cell cycle][ko04120:Ubiquitin mediated proteolysis][ko04150:mTOR signaling pathway][ko05168: Herpes simplex infection][ko05169: Epstein-Barr virus infection][ko05200: Pathways in cancer][ko05203:Viral carcinogenesis][ko05222:Small cell lung cancer]	-	EOY16514.1:Fbox/ RNIlike superfamily protein isoform 1 [Theobroma cacao] >EOY16515.1 Fbox/RNIlike superfamily protein isoform 1 [Theobroma cacao]	-	A0A061FPQ7|A0A061FPQ7_THECC:F-box/RNI-like superfamily protein isoform 1;OS=Theobroma cacao;

geneid	trend	GO	Pathway	TF	NR	NT	Uniprot
DN125291_c4_g4_i1	UP	[GO:0005737:cytoplasm][GO:0004364:glutathione transferase activity][GO:0006749:glutathione metabolic process][GO:0009407:toxin catabolic process]	[ko00480:Glutathione metabolism][ko00980:Metabolism of xenobiotics by cytochrome P450][ko00982:Drug metabolism - cytochrome P450][ko01524:Platinum drug resistance][ko05204:Chemical carcinogenesis][ko05418:Fluid shear stress and atherosclerosis]	-	XP_002264437.1:PREDICTED: probable glutathione Stransferase [Vitis vinifera]	gi|461496407|ref|NM_001247228.2|:Solanum lycopersicum glutathione S-transferase-like protein (LOC543836), mRNA	F6HR78|F6HR78_VITVI:Putative uncharacterized protein;OS=Vitis vinifera;GN=VIT_19s0093g00310{ECO:0000313|EMBL:CCB57186.1};|GO:0005737,|GO:0004364,|GO:0006749,GO:0009407,
DN123263_c0_g3_i1	UP	-	-	-	XP_021761237.1:calcium sensing receptor, chloroplasticlike [Chenopodium quinoa]	-	A0A0K9R5V6|A0A0K9R5V6_SPIOL:Uncharacterized protein;OS=Spinacia oleracea;

geneid	trend	GO	Pathway	TF	NR	NT	Uniprot
DN126598_c3_g2_i2	UP	[GO:0016021:integral component of membrane][GO:0005886:plasma membrane][GO:0005524:ATP binding][GO:0005509:calcium ion binding][GO:0030247:polysaccharide binding][GO:0004674:protein serine/threonine kinase activity][GO:0007166:cell surface receptor signaling pathway]	[ko04064:NF-kappa B signaling pathway][ko04620:Toll-like receptor signaling pathway][ko04621:NOD-like receptor signaling pathway][ko04624:Toll and Imd signaling pathway][ko04722:Neurotrophin signaling pathway][ko05133:Pertussis][ko05140:Leishmaniasis][ko05142:Chagas disease (American trypanosomiasis)][ko05145:Toxoplasmosis][ko05152:Tuberculosis][ko05162:Measles][ko05164:Influenza A]	-	XP_021678238.1:wallassociated receptor kinase 2like [Hevea brasiliensis]	-	F6H0G0|F6H0G0_VITVI:Putative uncharacterized protein;OS=Vitis vinifera;GN=VIT_18s0001g01370 {ECO:0000313|EMBL:CCB45432.1};|GO:0016021,GO:0005886,|GO:0005524,GO:0005509,GO:0030247,GO:0004674,|GO:0007166,

geneid	trend	GO	Pathway	TF	NR	NT	Uniprot
DN126049_c3_g1_i1	UP	-	-	Niben101Scf00646g07008.1:Nicotiana benthamiana|Trihelix|Trihelix family protein	AOA52386.1:actin 7 [Haloxylon ammodendron]	gi|521650517|gb|GAIA01041968.1|:TSA: Salicornia europaea Unigene47152_Se200S transcribed RNA sequence	A0A1B2LUP0|A0A1B2LUP0_9CARY: Actin 7;OS=Haloxylon ammodendron.;
DN108183_c0_g1_i1	UP	[GO:0000786:nucleosome][GO:0005634:nucleus][GO:0003677:DNA binding]	[ko05034:Alcoholism][ko05203:Viral carcinogenesis][ko05322:Systemic lupus erythematosus]	-	XP_012090395.1:histone H2B [Jatropha curcas] >XP_012090439.1 histone H2B [Jatropha curcas] >KDP45066.1 hypothetical protein JCGZ_01566 [Jatropha curcas] >KDP45069.1 hypothetical protein JCGZ_01569 [Jatropha curcas]	gi|116734896|gb|AC184093.2|:Populus trichocarpa clone Pop1-65E3, complete sequence	A0A067L9C8|A0A067L9C8_JATCU:Histone H2B;OS=Jatropha curcas;|GO:0000786,GO:0005634,|GO:0003677,

geneid	trend	GO	Pathway	TF	NR	NT	Uniprot
DN117560_c0_g1_i2	UP	[GO:0009941:chloroplast envelope][GO:0009534:chloroplast thylakoid][GO:0009840:chloroplastic endopeptidase Clp complex][GO:0004252:serine-type endopeptidase activity]	[ko04112:Cell cycle - Caulobacter][ko04212:Longevity regulating pathway - worm]	PSME_00013978-RA:Pseudotsuga menziesii|SBP|SBP family protein	XP_010693678.1:PREDICTED: ATPdependent Clp protease proteolytic subunit 3, chloroplastic [Beta vulgaris subsp. vulgaris] >KMT18622.1 hypothetical protein BVRB_2g027520 [Beta vulgaris subsp. vulgaris]	gi|521675408|gb|GAIA01029506.1|:TSA: Salicornia europaea Unigene32352_Se200S transcribed RNA sequence	A0A0J8CYD4|A0A0J8CYD4_BETVU: ATP-dependent Clp protease proteolytic subunit;OS=Beta vulgaris subsp. vulgaris.;|GO:0009941,GO:0009534,GO:0009840,|GO:0004252,
DN126364_c2_g1_i4	UP	[GO:0003824:catalytic activity][GO:0008152:metabolic process]	[ko00280:Valine, leucine and isoleucine degradation]	-	XP_010691379.1:PREDICTED: malonateCoA ligase isoform X2 [Beta vulgaris subsp. vulgaris]	gi|359482914|ref|XM_002279103.2|:PREDICTED: Vitis vinifera acyl-CoA synthetase family member 3, mitochondrial-like (LOC100258310), mRNA	A0A0J8BIP6|A0A0J8BIP6_BETVU:Uncharacterized protein;OS=Beta vulgaris subsp. vulgaris.;|GO:0003824,|GO:0008152,

geneid	trend	GO	Pathway	TF	NR	NT	Uniprot
DN119729_c4_g1_i1	UP	[GO:0005622:intracellular][GO:0008270:zinc ion binding]	-	Sp_027210_mzdq.t1:Spinacia oleracea|CO-like|CO-like family protein	XP_021850426.1:zinc finger protein CONSTANSLIKE 4 [Spinacia oleracea] >KNA23165.1 hypothetical protein SOVF_027210 [Spinacia oleracea]	gi|186911829|gb|EU437783.1|:Beta vulgaris subsp. vulgaris COL2 (COL2) gene, partial cds	A0A0K9RUG6|A0A0K9RUG6_SPIOL: Uncharacterized protein;OS=Spinacia oleracea;|GO:0005622,|GO:0008270,
DN121586_c0_g1_i2	UP	[GO:0016021:integral component of membrane]	-	-	XP_010690329.1:PREDICTED: protein CHAPERONELIKE PROTEIN OF POR1, chloroplastic [Beta vulgaris subsp. vulgaris] >KMT01357.1 hypothetical protein BVRB_9g213710 [Beta vulgaris subsp. vulgaris]	gi|217071889|gb|BT051641.1|:Medicago truncatula clone MTYF5_F6_F7_F81G-F-19 unknown mRNA >gi|388522646|gb|BT149591.1| Medicago truncatula clone JCVI-FLMt-11P19 unknown mRNA	A0A0J8BK07|A0A0J8BK07_BETVU:Uncharacterized protein;OS=Beta vulgaris subsp. vulgaris.;|GO:0016021,

geneid	trend	GO	Pathway	TF	NR	NT	Uniprot
DN122320_c2_g2_i10	UP	[GO:0009941:chloroplast envelope][GO:0016021:integral component of membrane][GO:0051741:2-methyl-6-phytyl-1,4-benzoquinone methyltransferase activity][GO:0010236:plastoquinone biosynthetic process][GO:0010189:vitamin E biosynthetic process]	[ko00130:Ubiquinone and other terpenoid-quinone biosynthesis]	-	XP_010679141.1:PREDICTED: 2methyl6phytyl1,4hydroquinone methyltransferase, chloroplastic [Beta vulgaris subsp. vulgaris] >KMT10256.1 hypothetical protein BVRB_5g120130 [Beta vulgaris subsp. vulgaris]	gi|21227|emb|X56963.1|:Spinach mRNA for chloroplast 37kD inner envelope membrane protein	A0A0J8F3D9|A0A0J8F3D9_BETVU:Uncharacterized protein;OS=Beta vulgaris subsp. vulgaris.;|GO:0009941,GO:0016021,|GO:0051741,|GO:0010236,GO:0010189,
DN127345_c1_g1_i6	UP	-	-	-	XP_021772848.1:dormancyassociated protein homolog 3like isoform X3 [Chenopodium quinoa] >XP_021772853.1 dormancyassociated protein homolog 3like isoform X3 [Chenopodium quinoa]	-	A0A0J8CQF6|A0A0J8CQF6_BETVU:Uncharacterized protein;OS=Beta vulgaris subsp. vulgaris.;

geneid	trend	GO	Pathway	TF	NR	NT	Uniprot
DN127252_c4_g1_i9	UP	[GO:0004565:beta-galactosidase activity][GO:0005975:carbohydrate metabolic process]	-	Thecc1EG029045t1: Theobroma cacao|C2H2|C2H2 family protein	XP_021751137.1:putative betagalactosidase [Chenopodium quinoa]	gi|225320515|dbj|AK324604.1|:Solanum lycopersicum cDNA, clone: LEFL1080AA05, HTC in leaf	A0A0J8B708|A0A0J8B708_BETVU:Beta-galactosidase;OS=Beta vulgaris subsp. vulgaris.;|GO:0004565,|GO:0005975,
DN118671_c4_g1_i8	UP	[GO:0009941:chloroplast envelope][GO:0016020:membrane][GO:0005739:mitochondrion][GO:0005524:ATP binding][GO:0004222:metalloendopeptidase activity][GO:0009408:response to heat]	[ko04139:Mitophagy - yeast]	FANhyb_rscf00001142.1.g00004.1:Fragaria x ananassa|C2H2|C2H2 family protein	XP_010671509.1:PREDICTED: ATPdependent zinc metalloprotease FTSH 11, chloroplastic/mitochondrial [Beta vulgaris subsp. vulgaris] >KMT16073.1 hypothetical protein BVRB_3g052420 [Beta vulgaris subsp. vulgaris]	gi|521736644|gb|GAIA01008588.1|:TSA: Salicornia europaea Unigene8932_Se200S transcribed RNA sequence	A0A0J8CVU9|A0A0J8CVU9_BETVU: Uncharacterized protein;OS=Beta vulgaris subsp. vulgaris.;|GO:0009941,GO:0016020,GO:0005739,|GO:0005524,GO:0004222,|GO:0009408,

geneid	trend	GO	Pathway	TF	NR	NT	Uniprot
DN118340_c1_g1_i1	UP	-	-	-	XP_021761972.1:uncharacterized protein LOC110726774 [Chenopodium quinoa]	gi|359483228|ref|XM_002274145.2|:PREDICTED: Vitis vinifera uncharacterized LOC100256501, transcript variant 1 (LOC100256501), mRNA	A0A0K9QTV4|A0A0K9QTV4_SPIOL: Uncharacterized protein;OS=Spinacia oleracea;
DN119772_c2_g6_i1	UP	-	-	-	XP_010680131.1:PREDICTED: B2 protein [Beta vulgaris subsp. vulgaris] >XP_010680134.1 PREDICTED: B2 protein [Beta vulgaris subsp. vulgaris] >KMT09095.1 hypothetical protein BVRB_6g132030 [Beta vulgaris subsp. vulgaris]	gi|521738675|gb|GAIA01007463.1|:TSA: Salicornia europaea Unigene7778_Se200 S transcribed RNA sequence	A0A0K9RFU4|A0A0K9RFU4_SPIOL:Uncharacterized protein;OS=Spinacia oleracea;

geneid	trend	GO	Pathway	TF	NR	NT	Uniprot
DN120431_c4_g1_i3	UP	-	-	-	XP_021853193.1:uncharacterized protein LOC110792683 [Spinacia oleracea] >KNA02968.1 hypothetical protein SOVF_213580 [Spinacia oleracea]	-	A0A0K9Q8D8|A0A0K9Q8D8_SPIOL:Uncharacterized protein;OS=Spinacia oleracea;
DN122597_c0_g3_i1	UP	[GO:0004794:L-threonine ammonia-lyase activity][GO:0030170:pyridoxal phosphate binding][GO:0008652:cellular amino acid biosynthetic process][GO:0009097:isoleucine biosynthetic process]	[ko00260:Glycine, serine and threonine metabolism][ko00290:Valine, leucine and isoleucine biosynthesis]	676765190:Sisymbrium irio|Trihelix|Trihelix family protein	XP_010683961.1:PREDICTED: threonine dehydratase biosynthetic, chloroplastic [Beta vulgaris subsp. vulgaris] >KMT06426.1 hypothetical protein BVRB_7g161000 [Beta vulgaris subsp. vulgaris]	gi|224068914|ref|XM_002326194.1|:Populus trichocarpa predicted protein, mRNA	A0A0J8ETB3|A0A0J8ETB3_BETVU:Threonine dehydratase;OS=Beta vulgaris subsp. vulgaris.;|GO:0004794,GO:0030170,|GO:0008652,GO:0009097,

geneid	trend	GO	Pathway	TF	NR	NT	Uniprot
DN117718_c4_g1_i2	UP	[GO:0008234:cysteine-type peptidase activity]	-	FANhyb_rscf00000253.1.g00010.1:Fragaria x ananassa|BBR-BPC|BBR-BPC family protein	XP_010679531.1:PREDICTED: cysteine proteinase COT44 [Beta vulgaris subsp. vulgaris] >KMT09753.1 hypothetical protein BVRB_6g127340 [Beta vulgaris subsp. vulgaris]	gi|521673797|gb|GAIA01030493.1|:TSA: Salicornia europaea Unigene33439_Se200S transcribed RNA sequence	A0A0J8CCR5|A0A0J8CCR5_BETVU:Uncharacterized protein;OS=Beta vulgaris subsp. vulgaris.;|GO:0008234,
DN121196_c0_g2_i9	UP	[GO:0016021:integral component of membrane][GO:0055085:transmembrane transport]	-	-	XP_021757626.1:mitochondrial adenine nucleotide transporter ADNT1like [Chenopodium quinoa]	gi|521703323|gb|GAIA01016263.1|:TSA: Salicornia europaea Unigene16979_Se200S transcribed RNA sequence	A0A0J8CEL2|A0A0J8CEL2_BETVU:Uncharacterized protein;OS=Beta vulgaris subsp. vulgaris.;|GO:0016021,|GO:0055085,
DN117945_c2_g1_i24	UP	[GO:0005737:cytoplasm][GO:0016149:translation release factor activity, codon specific]	[ko03015:mRNA surveillance pathway]	MDP0000153244:Malus domestica|SBP|SBP family protein	XP_021866209.1:eukaryotic peptide chain release factor subunit 13 [Spinacia oleracea] >KNA25828.1 hypothetical protein	gi|521650290|gb|GAIA01042081.1|:TSA: Salicornia europaea Unigene47278_Se200S transcribed RNA sequence	A0A0K9S297|A0A0K9S297_SPIOL:Uncharacterized protein;OS=Spinacia oleracea;|GO:0005737,|GO:0016149,

geneid	trend	GO	Pathway	TF	NR	NT	Uniprot
					SOVF_002950 [Spinacia oleracea]		
DN120279_c6_g13_i1	UP	-	-	-	XP_021716270.1:LOW QUALITY PROTEIN: BURP domain protein RD22like [Chenopodium quinoa]	-	A0A0K9R0Z0|A0A0K9R0Z0_SPIOL:Uncharacterized protein;OS=Spinacia oleracea;

geneid	trend	GO	Pathway	TF	NR	NT	Uniprot
DN128955_c0_g2_i2	UP	[GO:0009535:chloroplast thylakoid membrane][GO:0016021:integral component of membrane][GO:0009522:photosystem I][GO:0009523:photosystem II][GO:0016168:chlorophyll binding][GO:0009765:photosynthesis, light harvesting][GO:0018298:protein-chromophore linkage]	[ko00196:Photosynthesis - antenna proteins]	-	XP_015943384.1:chlorophyll ab binding protein CP24 10A, chloroplastic [Arachis duranensis] >XP_016179733.1 chlorophyll ab binding protein CP24 10A, chloroplastic [Arachis ipaensis]	gi|110377771|gb|DQ676844.1|:Nicotiana tabacum chloroplast pigment-binding protein CP24 (Lhcb6) mRNA, complete cds; nuclear gene for chloroplast product	A0A059BAM1|A0A059BAM1_EUCGR: Chlorophyll a-b binding protein, chloroplastic;OS=Eucalyptus grandis;|GO:0009535,GO:0016021,GO:0009522,GO:0009523,|GO:0016168,|GO:0009765,GO:0018298,
DN120559_c0_g3_i3	UP	[GO:0009535:chloroplast thylakoid membrane][GO:0016021:integral component of membrane][GO:0045038:protein import into chloroplast thylakoid membrane][GO:0051205:protein insertion into membrane][GO:0090342:regulation of cell aging][GO:0010027:thylakoid membrane organization]	[ko02024:Quorum sensing][ko03060:Protein export][ko03070:Bacterial secretion system]	-	XP_021716176.1:inner membrane protein PPF1, chloroplasticlike [Chenopodium quinoa]	gi|255581094|ref|XM_002531316.1|:Ricinus communis Inner membrane protein PPF-1, chloroplast precursor, putative, mRNA	A0A0J8BZ42|A0A0J8BZ42_BETVU:Uncharacterized protein;OS=Beta vulgaris subsp. vulgaris.;|GO:0009535,GO:0016021,|GO:0045038,GO:0051205,GO:0090342,GO:0010027,

geneid	trend	GO	Pathway	TF	NR	NT	Uniprot
DN125104_c0_g2_i14	UP	[GO:0005524:ATP binding][GO:0004672:protein kinase activity]	[ko04016:MAPK signaling pathway - plant]	Pahal.H01961.1:Panicum hallii|WRKY|WRKY family protein	XP_021845108.1:mitogenactivated protein kinase kinase kinase YODAlike [Spinacia oleracea] >XP_021845109.1 mitogenactivated protein kinase kinase kinase YODAlike [Spinacia oleracea] >XP_021845110.1 mitogenactivated protein kinase kinase kinase YODAlike [Spinacia oleracea] >XP_021845111.1 mitogenactivated protein kinase kinase kinase YODAlike [Spinacia oleracea] >KNA25513.1 hypothetical protein SOVF_005980 [Spinacia oleracea]	-	A0A0K9S1C0|A0A0K9S1C0_SPIOL:Uncharacterized protein;OS=Spinacia oleracea;|GO:0005524,GO:0004672,

geneid	trend	GO	Pathway	TF	NR	NT	Uniprot
DN116676_c0_g1_i3	UP	[GO:0008234:cysteine-type peptidase activity]	-	FANhyb_rscf00000253.1.g00010.1:Fragaria x ananassa|BBR-BPC|BBR-BPC family protein	XP_021753353.1:lowtemperatureinduced cysteine proteinaselike [Chenopodium quinoa]	gi|222425025|dbj|AB377534.1|:Spinacia oleracea SoCP3-41 mRNA for cysteine protease, complete cds	A0A0J8BMG6|A0A0J8BMG6_BETVU:Uncharacterized protein;OS=Beta vulgaris subsp. vulgaris.;|GO:0008234,
DN121007_c1_g1_i3	UP	[GO:0016021:integral component of membrane]	-	-	XP_010549311.1:PREDICTED: uncharacterized protein LOC104820536 isoform X1 [Tarenaya hassleriana]	-	V4U479|V4U479_9ROSI:Uncharacterized protein;OS=Citrus clementina.;|GO:0016021,
DN122448_c1_g1_i9	UP	[GO:0016765:transferase activity, transferring alkyl or aryl (other than methyl) groups][GO:0008299:isoprenoid biosynthetic process][GO:0015979:photosynthesis]	[ko00900:Terpenoid backbone biosynthesis]	889097:Arabidopsis lyrata|STAT|STAT family protein	XP_021773934.1:probable solanesyldiphosphate synthase 3, chloroplastic [Chenopodium quinoa]	gi|521741293|gb|GAIA01005754.1|:TSA: Salicornia europaea Unigene6043_Se200S transcribed RNA sequence	A0A0J8BWM4|A0A0J8BWM4_BETVU:Uncharacterized protein;OS=Beta vulgaris subsp. vulgaris.;|GO:0016765,|GO:0008299,GO:0015979,

geneid	trend	GO	Pathway	TF	NR	NT	Uniprot
DN124591_c0_g2_i5	UP	[GO:0009535:chloroplast thylakoid membrane][GO:0016021:integral component of membrane]	-	CCG000223.2:Populus euphratica|bHLH|bHLH family protein	XP_021732496.1:uncharacterized protein LOC110699301 [Chenopodium quinoa]	-	A0A0J8D1Y3|A0A0J8D1Y3_BETVU:Uncharacterized protein;OS=Beta vulgaris subsp. vulgaris.;|GO:0009535,GO:0016021,
DN125988_c1_g4_i3	UP	[GO:0016021:integral component of membrane]	-	-	XP_021753030.1:protein CHAPERONELIKE PROTEIN OF POR1, chloroplasticlike [Chenopodium quinoa]	gi|359475007|ref|XM_002277990.2|:PREDICTED: Vitis vinifera uncharacterized LOC100265837 (LOC100265837), mRNA	A0A0J8BK07|A0A0J8BK07_BETVU:Uncharacterized protein;OS=Beta vulgaris subsp. vulgaris.;|GO:0016021,

geneid	trend	GO	Pathway	TF	NR	NT	Uniprot
DN125283_c0_g1_i10	UP	[GO:0009507:chloroplast][GO:0016020:membrane][GO:0052663:antheraxanthin epoxidase activity][GO:0071949:FAD binding][GO:0009540:zeaxanthin epoxidase [overall] activity][GO:0052662:zeaxanthin epoxidase activity][GO:0009688:abscisic acid biosynthetic process]	[ko00906:Carotenoid biosynthesis]	Medtr4g134870.1:Medicago truncatula|ERF|ERF family protein	XP_010666612.1:PREDICTED: zeaxanthin epoxidase, chloroplastic [Beta vulgaris subsp. vulgaris] >KMS95959.1 hypothetical protein BVRB_003210 [Beta vulgaris subsp. vulgaris]	gi|5360185|gb|AF159948.1|AF159948:Prunus armeniaca zeaxanthin epoxidase (ZEAEPOX2) mRNA, complete cds	A0A0J8B447|A0A0J8B447_BETVU:Zeaxanthin epoxidase, chloroplastic;OS=Beta vulgaris subsp. vulgaris.;|GO:0009507,GO:0016020,|GO:0052663,GO:0071949,GO:0009540,GO:0052662,|GO:0009688,
DN116014_c0_g6_i2	UP	[GO:0016020:membrane]	-	-	XP_011071605.1:hypersensitiveinduced response protein 1 [Sesamum indicum]	gi|359491739|ref|XM_003634268.1|:PREDICTED: Vitis vinifera hypersensitive-induced response protein 1-like (LOC100245034), mRNA	A0A0D2LVS1|A0A0D2LVS1_GOSRA: Uncharacterized protein;OS=Gossypium raimondii;|GO:0016020,

geneid	trend	GO	Pathway	TF	NR	NT	Uniprot
DN126018_c2_g2_i10	UP	[GO:0046556:alpha-L-arabinofuranosidase activity][GO:0046373:L-arabinose metabolic process]	[ko00520:Amino sugar and nucleotide sugar metabolism]	-	XP_021861801.1:alphaLarabinofuranosidase 1like [Spinacia oleracea] >XP_021861802.1 alphaLarabinofuranosidase 1like [Spinacia oleracea] >XP_021861803.1 alphaLarabinofuranosidase 1like [Spinacia oleracea] >XP_021861804.1 alphaLarabinofuranosidase 1like [Spinacia oleracea] >KNA14857.1 hypothetical protein SOVF_103590 [Spinacia oleracea]	gi|254553043|dbj|AB266534.1|:Dionaea muscipula ARF mRNA for alpha-L-arabinofuranosidase, complete cds	A0A0K9R7U9|A0A0K9R7U9_SPIOL:Uncharacterized protein;OS=Spinacia oleracea;|GO:0046556,|GO:0046373,

geneid	trend	GO	Pathway	TF	NR	NT	Uniprot
DN122030_c2_g2_i1	UP	[GO:0009570:chloroplast stroma][GO:0080085:signal recognition particle, chloroplast targeting][GO:0008312:7S RNA binding][GO:0005525:GTP binding][GO:0003924:GTPase activity][GO:0006614:SRP-dependent cotranslational protein targeting to membrane]	[ko02024:Quorum sensing][ko03060:Protein export][ko03070:Bacterial secretion system]	-	XP_021766542.1:signal recognition particle 54 kDa protein, chloroplasticlike [Chenopodium quinoa]	gi|521562529|gb|GAIA01075718.1|:TSA: Salicornia europaea Unigene91748_Se200S transcribed RNA sequence	A0A0J8EU96|A0A0J8EU96_BETVU:Uncharacterized protein;OS=Beta vulgaris subsp. vulgaris.;|GO:0009570,GO:0080085,|GO:0008312,GO:0005525,GO:0003924,|GO:0006614,
DN117724_c0_g1_i1	UP	[GO:0016740:transferase activity][GO:0008299:isoprenoid biosynthetic process]	[ko00900:Terpenoid backbone biosynthesis][ko05164:Influenza A][ko05166:HTLV-I infection]	-	XP_021841818.1:farnesyl pyrophosphate synthase 1like [Spinacia oleracea] >KNA12159.1 hypothetical protein SOVF_128270 [Spinacia oleracea]	gi|300431228|gb|HM219226.1|:Aralia elata farnesyl diphosphate synthase mRNA, complete cds	A0A0K9QY15|A0A0K9QY15_SPIOL:Uncharacterized protein;OS=Spinacia oleracea;|GO:0016740,|GO:0008299,

geneid	trend	GO	Pathway	TF	NR	NT	Uniprot
DN120928_c2_g2_i1	UP	[GO:0005737:cytoplasm][GO:0005634:nucleus][GO:0008565:obsolete protein transporter activity][GO:0006606:protein import into nucleus]	-	MDP0000309902:Malus domestica|GATA|GATA family protein	XP_010024115.1:PREDICTED: importin subunit alpha1a [Eucalyptus grandis] >KCW60545.1 hypothetical protein EUGRSUZ_H03281 [Eucalyptus grandis] >KCW60546.1 hypothetical protein EUGRSUZ_H03281 [Eucalyptus grandis]	gi|84453223|dbj|AB236757.1|:Trifolium pratense RNA for putative importin alpha, complete cds, clone: C1528	A0A059B3T6|A0A059B3T6_EUCGR:Importin subunit alpha;OS=Eucalyptus grandis;|GO:0005737,GO:0005634,|GO:0008565,|GO:0006606,
DN116902_c0_g1_i1	UP	[GO:0004514:nicotinate-nucleotide diphosphorylase (carboxylating) activity][GO:0009435:NAD biosynthetic process]	[ko00760:Nicotinate and nicotinamide metabolism]	-	XP_021735454.1:nicotinatenucleotide pyrophosphorylase [carboxylating], chloroplasticlike [Chenopodium quinoa] >XP_021735455.1 nicotinatenucleotide pyrophosphorylase [carboxylating], chloroplasticlike	gi|521707218|gb|GAIA01014491.1|:TSA: Salicornia europaea Unigene15150_Se200S transcribed RNA sequence	A0A0K9R296|A0A0K9R296_SPIOL:Nicotinate-nucleotide pyrophosphorylase [carboxylating];OS=Spinacia oleracea;|GO:0004514,|GO:0009435,

geneid	trend	GO	Pathway	TF	NR	NT	Uniprot
					[Chenopodium quinoa]		
DN119157_c4_g1_i1	UP	-	-	Tp57577_TGAC_v2_mRNA39490:Trifolium pratense|NAC|NAC family protein	ALU58321.1:stemspecific protein TSJT1 [Mesembryanthemum crystallinum]	gi|460237218|gb|GABL01030866.1|:TSA: Camelina sativa comp44865_c0_seq1 transcribed RNA sequence	A0A0U3HQC3|A0A0U3HQC3_MESCR:Stem-specific protein TSJT1;OS=Mesembryanthemum crystallinum;
DN121583_c0_g1_i5	UP	[GO:0004834:tryptophan synthase activity][GO:0008652:cellular amino acid biosynthetic process]	[ko00260:Glycine, serine and threonine metabolism][ko00400:Phenylalanine, tyrosine and tryptophan biosynthesis]	-	XP_010694202.1:PREDICTED: tryptophan synthase beta chain 1 [Beta vulgaris subsp. vulgaris] >KMS98467.1 hypothetical protein BVRB_4g091620	gi|521664968|gb|GAIA01034908.1|:TSA: Salicornia europaea Unigene38652_Se200S transcribed RNA sequence	A0A0J8BER1|A0A0J8BER1_BETVU:Tryptophan synthase;OS=Beta vulgaris subsp. vulgaris.;|GO:0004834,|GO:0008652,

geneid	trend	GO	Pathway	TF	NR	NT	Uniprot
					[Beta vulgaris subsp. vulgaris]		
DN118900_c1_g3_i1	UP	-	-	-	-	-	-
DN116103_c3_g4_i5	UP	[GO:0008483:transaminase activity]	[ko00250:Alanine, aspartate and glutamate metabolism][ko00260:Glycine, serine and threonine metabolism][ko00630:Glyoxylate and dicarboxylate metabolism][ko00680:Methane metabolism][ko04146:Peroxisome]	-	XP_021593021.1:serineglyoxylate aminotransferaselike [Manihot esculenta] >XP_021593064.1 serineglyoxylate aminotransferaselike [Manihot esculenta] >OAY61337.1 hypothetical protein MANES_01G181700 [Manihot esculenta] >OAY61338.1 hypothetical protein	gi|521651870|gb|GAIA01040863.1|:TSA: Salicornia europaea Unigene45909_Se200S transcribed RNA sequence	A0A0A6ZHA3|A0A0A6ZHA3_GOSHI: Serine--glyoxylate aminotransferase;OS=Gossypium hirsutum;|GO:0008483,

geneid	trend	GO	Pathway	TF	NR	NT	Uniprot
					MANES_01G181700 [Manihot esculenta]		
DN116693_c0_g1_i2	UP	-	-	-	XP_021741487.1:uncharacterized protein LOC110707752 [Chenopodium quinoa]	gi|521472686|gb|GAHZ01058199.1|:TSA: Salicornia europaea Unigene69471_SeCKS transcribed RNA sequence	A0A0K9QQ72|A0A0K9QQ72_SPIOL:Uncharacterized protein;OS=Spinacia oleracea;

geneid	trend	GO	Pathway	TF	NR	NT	Uniprot
DN124836_c1_g2_i1	UP	[GO:0004096:catalase activity][GO:0020037:heme binding][GO:0046872:metal ion binding][GO:0042744:hydrogen peroxide catabolic process][GO:0006979:response to oxidative stress]	[ko00380:Tryptophan metabolism][ko00630:Glyoxylate and dicarboxylate metabolism][ko04011:MAPK signaling pathway - yeast][ko04016:MAPK signaling pathway - plant][ko04068:FoxO signaling pathway][ko04146:Peroxisome][ko04211:Longevity regulating pathway][ko04212:Longevity regulating pathway - worm][ko04213:Longevity regulating pathway - multiple species][ko05014:Amyotrophic lateral	Achn343181:Actinidia chinensis|Nin-like|Nin-like family protein	AAC19397.1:leaf catalase [Mesembryanthemum crystallinum]	gi|3202031|gb|AF069319.1|AF069319:Mesembryanthemum crystallinum leaf catalase mRNA, complete cds	O81336|O81336_MESCR:Catalase;OS=Mesembryanthemum crystallinum;|GO:0004096,GO:0020037,GO:0046872,|GO:0042744,GO:0006979,

geneid	trend	GO	Pathway	TF	NR	NT	Uniprot
			sclerosis (ALS)]				
DN128672_c1_g6_i1	UP	[GO:0016491:oxidoreductase activity][GO:0008270:zinc ion binding]	-	-	XP_021849248.1:2alkenal reductase (NADP(+)dependent)like [Spinacia oleracea] >KNA15052.1 hypothetical protein	gi|521741690|gb|GAIA01005415.1|:TSA: Salicornia europaea Unigene5704_Se200S transcribed RNA sequence	A0A0K9R8F5|A0A0K9R8F5_SPIOL:Uncharacterized protein;OS=Spinacia oleracea;|GO:0016491,GO:0008270,

geneid	trend	GO	Pathway	TF	NR	NT	Uniprot
					SOVF_101690 [Spinacia oleracea]		
DN127612_c 3_g8_i6	UP	[GO:0005739:mitochondrion][GO:00 09055:electron transfer activity][GO:0000062:fatty-acyl-CoA binding][GO:0050660:flavin adenine dinucleotide binding][GO:0008470:isovaleryl-Co A dehydrogenase activity][GO:0052890:oxidoreductase activity, acting on the CH-CH group of donors, with a flavin as acceptor][GO:0033539:fatty acid beta-oxidation using acyl-CoA dehydrogenase][GO:0055088:lipid homeostasis]	[ko00280:Valine, leucine and isoleucine degradation]	-	XP_002285017.1:P REDICTED: isovalerylCoA dehydrogenase, mitochondrial [Vitis vinifera]	gi|112005098|gb|DQ 831862.1|:Beta vulgaris M14 isovaleryl-CoA dehydrogenase mRNA, complete cds	D7SKV8|D7SKV8_ VITVI:Putative uncharacterized protein;OS=Vitis vinifera;GN=VIT_0 6s0004g03140 {ECO:0000313|EM BL:CBI16286.3};|G O:0005739,|GO:000 9055,GO:0000062,G O:0050660,GO:0008 470,GO:0052890,|G O:0033539,GO:0055 088,

geneid	trend	GO	Pathway	TF	NR	NT	Uniprot
DN116095_c0_g2_i4	UP	[GO:0009055:electron transfer activity][GO:0005506:iron ion binding]	-	-	XP_021732923.1:chaperone protein dnaJ C76, chloroplasticlike [Chenopodium quinoa]	gi|521697901|gb|GAIA01018500.1|:TSA: Salicornia europaea Unigene19455_Se200S transcribed RNA sequence	A0A0K9QJ64|A0A0K9QJ64_SPIOL:Uncharacterized protein;OS=Spinacia oleracea;|GO:0009055,GO:0005506,
DN120477_c1_g6_i1	UP	[GO:0046872:metal ion binding][GO:0003723:RNA binding]	-	WALNUT_00025139-RA:Juglans regia|C3H|C3H family protein	XP_018805967.1:PREDICTED: zinc finger CCCH domaincontaining protein 14like [Juglans regia]	gi|292780124|dbj|AK337948.1|:Lotus japonicus cDNA, clone: LjFL2-004-BE02, HTC	A0A0J8CNI7|A0A0J8CNI7_BETVU:Uncharacterized protein;OS=Beta vulgaris subsp. vulgaris.;|GO:0046872,GO:0003723,
DN120252_c0_g1_i1	UP	-	-	-	XP_021835350.1:pentatricopeptide repeatcontaining protein At5g25630 [Spinacia oleracea] >KNA21754.1 hypothetical protein SOVF_040510 [Spinacia oleracea]	gi|521706148|gb|GAIA01014834.1|:TSA: Salicornia europaea Unigene15501_Se200S transcribed RNA sequence	A0A0K9RS72|A0A0K9RS72_SPIOL:Uncharacterized protein;OS=Spinacia oleracea;

geneid	trend	GO	Pathway	TF	NR	NT	Uniprot
DN127048_c3_g1_i5	UP	[GO:0016021:integral component of membrane][GO:0015095:magnesium ion transmembrane transporter activity]	-	Lus10022218:Linum usitatissimum|bHLH|bHLH family protein	XP_010680685.1:PREDICTED: magnesium transporter MRS25 [Beta vulgaris subsp. vulgaris] >KMT08929.1 hypothetical protein BVRB_6g136460 [Beta vulgaris subsp. vulgaris]	gi|521706002|gb|GAIA01014907.1|:TSA: Salicornia europaea Unigene15577_Se200S transcribed RNA sequence	A0A0J8C9J5|A0A0J8C9J5_BETVU:Uncharacterized protein;OS=Beta vulgaris subsp. vulgaris.;|GO:0016021,|GO:0015095,
DN123630_c1_g1_i9	UP	[GO:0009507:chloroplast][GO:0045300:acyl-[acyl-carrier-protein] desaturase activity][GO:0006633:fatty acid biosynthetic process]	[ko00061:Fatty acid biosynthesis][ko01040:Biosynthesis of unsaturated fatty acids]	XP_011395797.1:Auxenochlorella protothecoides|G2-like|G2-like family protein	XP_021734327.1:stearoyl[acylcarrierprotein] 9desaturase, chloroplasticlike [Chenopodium quinoa]	gi|16588702|gb|AF315600.1|:Bassia scoparia ACP-stearoyl desaturase mRNA, complete cds	A0A0J8CLR8|A0A0J8CLR8_BETVU:Acyl-[acyl-carrier-protein] desaturase;OS=Beta vulgaris subsp. vulgaris.;|GO:0009507,|GO:0045300,|GO:0006633,
DN127412_c3_g15_i1	UP	-	-	-	-	-	-

geneid	trend	GO	Pathway	TF	NR	NT	Uniprot
DN127698_c3_g1_i1	UP	[GO:0005783:endoplasmic reticulum][GO:0016021:integral component of membrane][GO:0016747:transferase activity, transferring acyl groups other than amino-acyl groups][GO:0006633:fatty acid biosynthetic process]	[ko00062:Fatty acid elongation]	-	XP_021746428.1:3ketoacylCoA synthase 10like [Chenopodium quinoa]	gi|28864533|gb|AY206684.1|:Tropaeolum majus fiddlehead-like protein mRNA, complete cds	A0A0J8BH71|A0A0J8BH71_BETVU:3-ketoacyl-CoA synthase;OS=Beta vulgaris subsp. vulgaris.;|GO:0005783,GO:0016021,|GO:0016747,|GO:0006633,
DN120811_c1_g2_i2	UP	[GO:0016788:hydrolase activity, acting on ester bonds]	-	-	XP_010685239.1:PREDICTED: GDSL esterase/lipase 2like [Beta vulgaris subsp. vulgaris]	gi|521747012|gb|GAIA01002357.1|:TSA: Salicornia europaea Unigene2410_Se200S transcribed RNA sequence	A0A0J8BXN2|A0A0J8BXN2_BETVU: Uncharacterized protein;OS=Beta vulgaris subsp. vulgaris.;|GO:0016788,
DN127602_c1_g1_i1	UP	[GO:0005524:ATP binding][GO:0016787:hydrolase activity]	[ko04141:Protein processing in endoplasmic reticulum][ko05134:Legionellosis]	EMT26392:Aegilops tauschii|FAR1|FAR1 family protein	XP_010687252.1:PREDICTED: cell division cycle protein 48 homolog [Beta vulgaris subsp. vulgaris] >KMT03730.1 hypothetical protein BVRB_8g188790	gi|381145558|gb|JQ678763.1|:Camellia sinensis cell division cycle protein 48 (CDC48) mRNA, complete cds	A0A0J8BQG7|A0A0J8BQG7_BETVU: Uncharacterized protein;OS=Beta vulgaris subsp. vulgaris.;|GO:0005524,GO:0016787,

geneid	trend	GO	Pathway	TF	NR	NT	Uniprot
					[Beta vulgaris subsp. vulgaris]		
DN113354_c1_g1_i9	UP	[GO:0016021:integral component of membrane]	-	Zmw_sc06582.1.g00020.1:Zoysia matrella|C2H2|C2H2 family protein	XP_021862902.1:sulfite exporter TauE/SafE family protein 3like [Spinacia oleracea] >KNA07860.1 hypothetical protein SOVF_168000 [Spinacia oleracea]	-	A0A0K9QKR8|A0A0K9QKR8_SPIOL: Uncharacterized protein;OS=Spinacia oleracea;|GO:0016021,
DN120827_c1_g3_i1	UP	[GO:0005737:cytoplasm][GO:0005874:microtubule][GO:0005525:GTP binding][GO:0003924:GTPase activity][GO:0005200:structural constituent of cytoskeleton][GO:0007017:microtubule-based process]	[ko04145:Phagosome][ko04540:Gap junction][ko05130:Pathogenic Escherichia coli infection]	KN539001.1_FGP003:Oryza longistaminata|NAC|NAC family protein	XP_011076163.1:tubulin beta1 chain [Sesamum indicum]	gi|470144898|ref|XM_004308036.1|:PREDICTED: Fragaria vesca subsp. vesca tubulin beta-1 chain-like, transcript variant 2 (LOC101292553),	B9R9A9|B9R9A9_RICCO:Tubulin beta chain;OS=Ricinus communis;|GO:0005737,GO:0005874,|GO:0005525,GO:0003924,GO:0005200,|GO:0007017,

geneid	trend	GO	Pathway	TF	NR	NT	Uniprot
						mRNA	
DN116262_c2_g1_i1	UP	-	-	-	BAM64831.1:hypothetical protein [Beta vulgaris]	-	K4PYU4|K4PYU4_BETVU:Uncharacterized protein;OS=Beta vulgaris;
DN128295_c0_g3_i7	UP	[GO:0003824:catalytic activity][GO:0030170:pyridoxal phosphate binding]	[ko00270:Cysteine and methionine metabolism][ko00450:Selenocompound metabolism]	-	AFT91997.1:cystathionine gammasynthase [Populus tomentosa] >AFT92009.1 cystathionine gammasynthase [Populus tomentosa]	-	K0E818|K0E818_POPTO:Cystathionine gamma-synthase;OS=Populus tomentosa;|GO:0003824,GO:0030170,

geneid	trend	GO	Pathway	TF	NR	NT	Uniprot
DN121005_c0_g2_i7	UP	-	-	-	XP_010682761.1:PREDICTED: uncharacterized protein LOC104897560 isoform X1 [Beta vulgaris subsp. vulgaris] >KMT07457.1 hypothetical protein BVRB_6g150760 isoform A [Beta vulgaris subsp. vulgaris]	gi|521537624|gb|GAHZ01004638.1|:TSA: Salicornia europaea Unigene4927_SeCKS transcribed RNA sequence	A0A0J8C1W0|A0A0J8C1W0_BETVU: Uncharacterized protein;OS=Beta vulgaris subsp. vulgaris.;

geneid	trend	GO	Pathway	TF	NR	NT	Uniprot
DN114444_c0_g1_i3	UP	[GO:0009941:chloroplast envelope][GO:0009570:chloroplast stroma][GO:0009535:chloroplast thylakoid membrane][GO:0015934:large ribosomal subunit][GO:0005634:nucleus][GO:0003723:RNA binding][GO:0003735:structural constituent of ribosome][GO:0006412:translation]	[ko03010:Ribosome]	-	XP_010694860.1:PREDICTED: 50S ribosomal protein L1, chloroplastic [Beta vulgaris subsp. vulgaris] >KMT18785.1 hypothetical protein BVRB_2g028990 [Beta vulgaris subsp. vulgaris]	gi|7414577|emb|X76932.1|:S.oleracea mRNA for ribosomal protein L1	A0A0J8CYE7|A0A0J8CYE7_BETVU:Ribosomal protein;OS=Beta vulgaris subsp. vulgaris.;|GO:0009941,GO:0009570,GO:0009535,GO:0015934,GO:0005634,|GO:0003723,GO:0003735,|GO:0006412,
DN126651_c3_g5_i4	UP	[GO:0009507:chloroplast][GO:0015035:protein disulfide oxidoreductase activity][GO:0045454:cell redox homeostasis][GO:0006662:glycerol ether metabolic process]	-	AA81G00050:Aethionema arabicum|HRT-like|HRT-like family protein	XP_010678488.1:PREDICTED: thioredoxinlike 12, chloroplastic [Beta vulgaris subsp. vulgaris] >KMT10788.1 hypothetical protein BVRB_5g114440 [Beta vulgaris subsp. vulgaris]	-	A0A0J8CFJ7|A0A0J8CFJ7_BETVU:Uncharacterized protein;OS=Beta vulgaris subsp. vulgaris.;|GO:0009507,|GO:0015035,|GO:0045454,GO:0006662,

geneid	trend	GO	Pathway	TF	NR	NT	Uniprot
DN118355_c2_g8_i3	UP	[GO:0009507:chloroplast][GO:0016021:integral component of membrane][GO:0009522:photosystem I][GO:0009767:photosynthetic electron transport chain]	[ko00195:Photosynthesis]	-	XP_021715688.1:photosystem I subunit Olike [Chenopodium quinoa]	-	A0A0J8BAP5|A0A0J8BAP5_BETVU:Uncharacterized protein;OS=Beta vulgaris subsp. vulgaris.;|GO:0009507,GO:0016021,GO:0009522,|GO:0009767,
DN117928_c1_g12_i2	UP	[GO:0009507:chloroplast][GO:0003723:RNA binding][GO:0006397:mRNA processing][GO:0008380:RNA splicing][GO:0008033:tRNA processing]	-	Gh_D03G1542:Gossypium hirsutum|NAC|NAC family protein	AGC70798.1:maturase K (chloroplast) [Bougainvillea glabra]	gi|442769447|gb|JQ844141.1|:Bougainvillea glabra trnK gene, intron; and maturase K (matK) gene, complete cds; chloroplast	L7VUS8|L7VUS8_9CARY:Maturase K;OS=Bougainvillea glabra.;|GO:0009507,|GO:0003723,|GO:0006397,GO:0008380,GO:0008033,
DN127809_c0_g2_i7	UP	[GO:0005622:intracellular][GO:0003746:translation elongation factor activity]	-	-	XP_021741470.1:uncharacterized protein LOC110707738 isoform X2 [Chenopodium quinoa]	gi|470101994|ref|XM_004287397.1|:PREDICTED: Fragaria vesca subsp. vesca uncharacterized LOC101312281 (LOC101312281), mRNA	A0A0J8BT23|A0A0J8BT23_BETVU:Elongation factor Ts;OS=Beta vulgaris subsp. vulgaris.;|GO:0005622,|GO:0003746,

geneid	trend	GO	Pathway	TF	NR	NT	Uniprot
DN127816_c0_g2_i20	UP	[GO:0051537:2 iron, 2 sulfur cluster binding][GO:0009055:electron transfer activity][GO:0050660:flavin adenine dinucleotide binding][GO:0005506:iron ion binding][GO:0016614:oxidoreductase activity, acting on CH-OH group of donors]	[ko00230:Purine metabolism][ko00232:Caffeine metabolism][ko00983:Drug metabolism - other enzymes][ko04146:Peroxisome]	676732640:Sisymbrium irio|bZIP|bZIP family protein	XP_021862669.1:xanthine dehydrogenase 1like isoform X1 [Spinacia oleracea] >KNA16760.1 hypothetical protein SOVF_086250 [Spinacia oleracea]	gi|374095610|gb|JN650203.1|:Camellia sinensis xanthine dehydrogenase mRNA, complete cds	A0A0K9RBA2|A0A0K9RBA2_SPIOL: Uncharacterized protein;OS=Spinacia oleracea;|GO:0051537,GO:0009055,GO:0050660,GO:0005506,GO:0016614,
DN125620_c2_g4_i9	UP	[GO:0016021:integral component of membrane][GO:0051537:2 iron, 2 sulfur cluster binding][GO:0010277:chlorophyllide a oxygenase [overall] activity]	-	-	XP_021758003.1:protein TIC 55, chloroplasticlike [Chenopodium quinoa]	gi|521449069|gb|GAHZ01071415.1|:TSA: Salicornia europaea Unigene87056_SeCKS transcribed RNA sequence	A0A0J8BH75|A0A0J8BH75_BETVU:Uncharacterized protein;OS=Beta vulgaris subsp. vulgaris.;|GO:0016021,|GO:0051537,GO:0010277,
DN126664_c0_g2_i6	UP	[GO:0016021:integral component of membrane]	-	Bv5_101120_supk.t1:Beta vulgaris|ERF|ERF family protein	AAO74897.1:putative Na+/myoinositol symporter [Mesembryanthemum crystallinum]	gi|29501738|gb|AY233386.1|:Mesembryanthemum crystallinum putative Na+/myo-inositol symporter (Itr3) mRNA, complete	Q84UY4|Q84UY4_MESCR:Putative Na+/myo-inositol symporter;OS=Mesembryanthemum crystallinum;|GO:0016021,|GO:0022891,

geneid	trend	GO	Pathway	TF	NR	NT	Uniprot
						cds	
DN126861_c0_g6_i5	UP	[GO:0005623:cell][GO:0015035:protein disulfide oxidoreductase activity][GO:0045454:cell redox homeostasis][GO:0006662:glycerol ether metabolic process]	[ko04621:NOD-like receptor signaling pathway][ko05418:Fluid shear stress and atherosclerosis]	-	KMS97967.1:hypothetical protein BVRB_4g096860 [Beta vulgaris subsp. vulgaris]	gi|224114951|ref|XM_002316865.1|:Populus trichocarpa thioredoxin m (PtrTrxm8), mRNA	A0A0J8BDD0|A0A0J8BDD0_BETVU: Uncharacterized protein;OS=Beta vulgaris subsp. vulgaris.;|GO:0005623,|GO:0015035,|GO:0045454,GO:0006662,
DN125455_c3_g2_i5	UP	[GO:0005739:mitochondrion][GO:0050897:cobalt ion binding][GO:0016874:ligase activity][GO:0008270:zinc ion binding][GO:0006552:leucine catabolic process]	[ko00280:Valine, leucine and isoleucine degradation]	CCG019154.1:Populus euphratica|bZIP|bZIP family protein	XP_021854838.1:methylcrotonoylCoA carboxylase beta chain, mitochondrial [Spinacia oleracea]	gi|460369858|ref|XM_004230726.1|:PREDICTED: Solanum lycopersicum methylcrotonoyl-CoA carboxylase beta chain, mitochondrial-like (LOC101262668), mRNA	A0A0J8BGX7|A0A0J8BGX7_BETVU: Uncharacterized protein;OS=Beta vulgaris subsp. vulgaris.;|GO:0005739,|GO:0050897,GO:0016874,GO:0008270,|GO:0006552,

geneid	trend	GO	Pathway	TF	NR	NT	Uniprot
DN118035_c0_g1_i1	UP	[GO:0048046:apoplast][GO:0016788:hydrolase activity, acting on ester bonds]	-	Niben101Scf08326g00014.1:Nicotiana benthamiana|C2H2|C2H2 family protein	XP_010690130.1:PREDICTED: GDSL esterase/lipase APG [Beta vulgaris subsp. vulgaris] >KMT01744.1 hypothetical protein BVRB_9g211950 [Beta vulgaris subsp. vulgaris]	gi|460215749|gb|GABL01048154.1|:TSA: Camelina sativa comp53613_c0_seq1 transcribed RNA sequence	A0A0J8BP66|A0A0J8BP66_BETVU:Uncharacterized protein;OS=Beta vulgaris subsp. vulgaris.;|GO:0048046,|GO:0016788,
DN120662_c1_g4_i1	UP	[GO:0005737:cytoplasm][GO:0004364:glutathione transferase activity][GO:0006749:glutathione metabolic process]	-	-	XP_021756539.1:glutathione Stransferaselike [Chenopodium quinoa]	-	A5AJY2|A5AJY2_VITVI:Putative uncharacterized protein;OS=Vitis vinifera;GN=VIT_18s0001g00690 {ECO:0000313|EMBL:CBI18904.3};|GO:0005737,|GO:0004364,|GO:0006749,

geneid	trend	GO	Pathway	TF	NR	NT	Uniprot
DN122901_c0_g9_i3	UP	[GO:0000786:nucleosome][GO:0005634:nucleus][GO:0003677:DNA binding][GO:0006334:nucleosome assembly]	-	KN538979.1_FGP004:Oryza longistaminata|BBR-BPC|BBR-BPC family protein	CAC43291.1:putative linker histone H1 variant protein [Beta vulgaris]	-	Q949M1|Q949M1_BETVU:Putative linker histone H1 variant protein;OS=Beta vulgaris;|GO:0000786,GO:0005634,|GO:0003677,|GO:0006334,
DN111248_c0_g2_i2	UP	[GO:0016021:integral component of membrane]	-	-	XP_012462442.1:PREDICTED: uncharacterized protein LOC105782316 [Gossypium raimondii] >XP_016706184.1 PREDICTED: uncharacterized protein LOC107920828 [Gossypium hirsutum] >KJB78600.1 hypothetical protein	-	A0A0D2W1K9|A0A0D2W1K9_GOSRA:Uncharacterized protein;OS=Gossypium raimondii;|GO:0016021,

geneid	trend	GO	Pathway	TF	NR	NT	Uniprot
					B456_013G009800 [Gossypium raimondii]		
DN122471_c0_g1_i2	UP	[GO:0008661:1-deoxy-D-xylulose-5-phosphate synthase activity][GO:0016114:terpenoid biosynthetic process]	[ko00730:Thiamine metabolism][ko00900:Terpenoid backbone biosynthesis]	-	AAQ84169.1:1deoxyDxylulose 5phosphate synthase [Pueraria montana var. lobata]	gi|386081791|gb|JQ710678.1|:Withania somnifera chloroplast 1-deoxy-D-xylulose-5-phosphate synthase (DXS) mRNA, complete cds; nuclear gene for chloroplast product	Q6EJC9|Q6EJC9_PUEML:1-deoxy-D-xylulose 5-phosphate synthase;OS=Pueraria montana var. lobata;|GO:0008661,|GO:0016114,

geneid	trend	GO	Pathway	TF	NR	NT	Uniprot
DN124975_c3_g3_i4	UP	[GO:0019898:extrinsic component of membrane][GO:0009654:photosystem II oxygen evolving complex][GO:0005509:calcium ion binding][GO:0015979:photosynthesis]	[ko00195:Photosynthesis]	-	XP_021719592.1:oxygenevolving enhancer protein 3, chloroplastic [Chenopodium quinoa]	-	A0A0J8ELT3|A0A0J8ELT3_BETVU:Uncharacterized protein;OS=Beta vulgaris subsp. vulgaris.;|GO:0019898,GO:0009654,|GO:0005509,|GO:0015979,
DN127653_c2_g2_i15	UP	[GO:0005524:ATP binding][GO:0004386:helicase activity]	[ko03040:Spliceosome]	Lus10020727:Linum usitatissimum|bHLH|bHLH family protein	XP_010679427.1:PREDICTED: probable premRNAsplicing factor ATPdependent RNA helicase DEAH2 isoform X1 [Beta vulgaris subsp. vulgaris] >KMT10005.1 hypothetical protein BVRB_5g122200 [Beta vulgaris subsp. vulgaris]	gi|359480186|ref|XM_002278211.2|:PREDICTED: Vitis vinifera probable pre-mRNA-splicing factor ATP-dependent RNA helicase-like (LOC100253689), mRNA	A0A0J8C8K0|A0A0J8C8K0_BETVU:Uncharacterized protein;OS=Beta vulgaris subsp. vulgaris.;|GO:0005524,GO:0004386,

geneid	trend	GO	Pathway	TF	NR	NT	Uniprot
DN125291_c5_g1_i2	UP	-	-	Rsa1.0_04342.1_g00002.1:Raphanus sativus|LBD|LBD family protein	XP_020252239.1:uncharacterized protein LOC109829580 [Asparagus officinalis]	-	F4NCG1|F4NCG1_BETVU:Uncharacterized protein;OS=Beta vulgaris subsp. vulgaris.;
DN123254_c7_g3_i6	UP	[GO:0004310:farnesyl-diphosphate farnesyltransferase activity][GO:0051996:squalene synthase activity][GO:0006696:ergosterol biosynthetic process]	[ko00906:Carotenoid biosynthesis]	-	XP_021742036.1:phytoene synthase 2, chloroplastic [Chenopodium quinoa]	gi|60418980|gb|AY789515.1|:Salicornia europaea phytoene synthase (PSY) mRNA, complete cds	A0A0K9QSZ1|A0A0K9QSZ1_SPIOL:Uncharacterized protein;OS=Spinacia oleracea;|GO:0004310,GO:0051996,|GO:0006696,
DN127853_c1_g4_i3	UP	[GO:0016021:integral component of membrane][GO:0051537:2 iron, 2 sulfur cluster binding][GO:0010277:chlorophyllide a oxygenase [overall] activity]	-	-	XP_021771925.1:protochlorophyllidedependent translocon component 52, chloroplasticlike isoform X1 [Chenopodium quinoa]	-	A0A0K9RT34|A0A0K9RT34_SPIOL:Uncharacterized protein;OS=Spinacia oleracea;|GO:0016021,|GO:0051537,GO:0010277,

geneid	trend	GO	Pathway	TF	NR	NT	Uniprot
DN127396_c1_g5_i3	UP	[GO:0005840:ribosome][GO:0003735:structural constituent of ribosome][GO:0006412:translation]	-	-	XP_021713758.1:30S ribosomal protein S21, chloroplasticlike [Chenopodium quinoa]	gi|521700291|gb|GAIA01017283.1|:TSA: Salicornia europaea Unigene18146_Se200S transcribed RNA sequence	I3RZW2|I3RZW2_LOTJA:Uncharacterized protein;OS=Lotus japonicus;|GO:0005840,|GO:0003735,|GO:0006412,
DN126154_c1_g2_i2	UP	-	[ko04016:MAPK signaling pathway - plant][ko04075:Plant hormone signal transduction]	-	XP_010696236.1:PREDICTED: EIN3binding Fbox protein 1 [Beta vulgaris subsp. vulgaris] >KMT18934.1 hypothetical protein BVRB_2g030340 [Beta vulgaris subsp. vulgaris]	-	A0A0J8D3Q1|A0A0J8D3Q1_BETVU:Uncharacterized protein;OS=Beta vulgaris subsp. vulgaris.;
DN116087_c6_g1_i8	UP	-	-	-	-	-	-
DN122435_c4_g2_i1	UP	[GO:0016021:integral component of membrane]	-	-	XP_008464165.1:PREDICTED: uncharacterized protein LOC103502113	-	A0A0A0KA68|A0A0A0KA68_CUCSA: Uncharacterized protein;OS=Cucumis

geneid	trend	GO	Pathway	TF	NR	NT	Uniprot
					[Cucumis melo]		sativus;|GO:0016021,
DN123880_c0_g1_i1	UP	[GO:0016021:integral component of membrane]	[ko03060:Protein export][ko03070:Bacterial secretion system]	-	XP_021749255.1:secindependent protein translocase protein TATC, chloroplasticlike [Chenopodium quinoa]	gi|15004993|dbj|AB054096.1|:Arabidopsis thaliana apg2 mRNA for cpTatC, complete cds	A0A0J8C331|A0A0J8C331_BETVU:Uncharacterized protein;OS=Beta vulgaris subsp. vulgaris.;|GO:0016021,
DN119752_c4_g1_i3	UP	[GO:0004067:asparaginase activity]	-	-	XP_002514609.1:PREDICTED: isoaspartyl peptidase/Lasparaginase 1 [Ricinus communis] >EEF47715.1 lasparaginase, putative [Ricinus communis]	-	B9RLJ0|B9RLJ0_RICCO:L-asparaginase, putative;OS=Ricinus communis;|GO:0004067,
DN126781_c1_g1_i1	UP	[GO:0008080:N-acetyltransferase activity]	-	-	XP_021745642.1:uncharacterized protein LOC110711537 [Chenopodium	-	A0A0K9S126|A0A0K9S126_SPIOL:Uncharacterized protein;OS=Spinacia

geneid	trend	GO	Pathway	TF	NR	NT	Uniprot
					quinoa]		oleracea;|GO:00080 80,
DN113830_c 0_g1_i2	UP	-	-	GRMZM2G010929 _P02:Zea mays|WOX|WOX family protein	XP_021757273.1:th ylakoid lumenal 15 kDa protein 1, chloroplasticlike [Chenopodium quinoa]	gi|521723543|gb|GA IA01011121.1|:TSA: Salicornia europaea Unigene11734_Se20 0S transcribed RNA sequence	A0A0K9QY31|A0A 0K9QY31_SPIOL:U ncharacterized protein;OS=Spinacia oleracea;
DN113354_c 1_g1_i4	UP	[GO:0016021:integral component of membrane]	-	Zmw_sc06582.1.g00 020.1:Zoysia matrella|C2H2|C2H2 family protein	XP_010691331.1:P REDICTED: uncharacterized protein LOC104904681 [Beta vulgaris subsp. vulgaris] >KMT00815.1 hypothetical protein BVRB_9g220910 [Beta vulgaris subsp. vulgaris]	-	A0A0J8BHD3|A0A 0J8BHD3_BETVU: Uncharacterized protein;OS=Beta vulgaris subsp. vulgaris.;|GO:00160 21,

geneid	trend	GO	Pathway	TF	NR	NT	Uniprot
DN125124_c2_g1_i2	UP	[GO:0005886:plasma membrane]	-	Neem_1766_f_3:Azadirachta indica|C2H2|C2H2 family protein	XP_021738974.1:uncharacterized protein LOC110705418 [Chenopodium quinoa]	-	A0A0J8CW22|A0A0J8CW22_BETVU:Uncharacterized protein;OS=Beta vulgaris subsp. vulgaris.;|GO:0005886,
DN117945_c2_g2_i1	UP	[GO:0005737:cytoplasm][GO:0016149:translation release factor activity, codon specific]	[ko03015:mRNA surveillance pathway]	MDP0000153244:Malus domestica|SBP|SBP family protein	XP_010678624.1:PREDICTED: eukaryotic peptide chain release factor subunit 13 [Beta vulgaris subsp. vulgaris]	gi|521650290|gb|GAIA01042081.1|:TSA: Salicornia europaea Unigene47278_Se200S transcribed RNA sequence	C5WNT1|C5WNT1_SORBI:Uncharacterized protein;OS=Sorghum bicolor;|GO:0005737,|GO:0016149,
DN116259_c0_g14_i2	UP	-	-	-	XP_021848115.1:Fbox/kelchrepeat protein At1g80440 [Spinacia oleracea] >KNA14194.1 hypothetical protein SOVF_109840 [Spinacia oleracea]	-	A0A0K9R5I7|A0A0K9R5I7_SPIOL:Uncharacterized protein;OS=Spinacia oleracea;

geneid	trend	GO	Pathway	TF	NR	NT	Uniprot
DN122063_c1_g1_i9	UP	[GO:0051539:4 iron, 4 sulfur cluster binding][GO:0010181:FMN binding][GO:0051287:NAD binding][GO:0008137:NADH dehydrogenase (ubiquinone) activity]	[ko00190:Oxidative phosphorylation][ko04723:Retrograde endocannabinoid signaling][ko04932:Non-alcoholic fatty liver disease (NAFLD)][ko05010:Alzheimer's disease][ko05012:Parkinson's disease][ko05016:Huntington's disease]	MDP0000537221:Malus domestica|FAR1|FAR1 family protein	XP_021744645.1:NADH dehydrogenase [ubiquinone] flavoprotein 1, mitochondrial [Chenopodium quinoa] >XP_021746741.1 NADH dehydrogenase [ubiquinone] flavoprotein 1, mitochondrial [Chenopodium quinoa]	gi|225462160|ref|XM_002266625.1|:PREDICTED: Vitis vinifera NADH dehydrogenase [ubiquinone] flavoprotein 1, mitochondrial-like (LOC100266461), mRNA	A0A0J8C660|A0A0J8C660_BETVU:Uncharacterized protein;OS=Beta vulgaris subsp. vulgaris.;|GO:0051539,GO:0010181,GO:0051287,GO:0008137,
DN113982_c0_g3_i1	UP	-	-	-	XP_010678948.1:PREDICTED: uncharacterized protein At5g48480 [Beta vulgaris subsp. vulgaris] >KMT10080.1 hypothetical protein BVRB_5g118430 [Beta vulgaris subsp.	gi|388501825|gb|BT139184.1|:Medicago truncatula clone JCVI-FLMt-10M7 unknown mRNA	A0A0J8C8R6|A0A0J8C8R6_BETVU:Uncharacterized protein;OS=Beta vulgaris subsp. vulgaris.;

geneid	trend	GO	Pathway	TF	NR	NT	Uniprot
					vulgaris]		
DN122042_c 3_g1_i1	UP	[GO:0009538:photosystem I reaction center][GO:0015979:photosynthesis]	[ko00195:Photosynt hesis]	-	XP_010681150.1:P REDICTED: photosystem I reaction center subunit II, chloroplastic [Beta vulgaris subsp. vulgaris] >KMT08725.1 hypothetical protein BVRB_6g139980 [Beta vulgaris subsp. vulgaris]	gi|512394857|gb|GA JZ01006313.1|:TSA: Prunus avium Pa_20088 transcribed RNA sequence	A0A0J8C4K2|A0A0 J8C4K2_BETVU:U ncharacterized protein;OS=Beta vulgaris subsp. vulgaris.;|GO:00095 38,|GO:0015979,

geneid	trend	GO	Pathway	TF	NR	NT	Uniprot
DN118894_c4_g4_i2	UP	[GO:0005623:cell][GO:0015035:protein disulfide oxidoreductase activity][GO:0045454:cell redox homeostasis][GO:0006662:glycerol ether metabolic process]	[ko04621:NOD-like receptor signaling pathway][ko05418:Fluid shear stress and atherosclerosis]	Neem_16119_f_2:Azadirachta indica|bHLH|bHLH family protein	XP_021596332.1:thioredoxinlike protein CXXS1 [Manihot esculenta] >OAY26044.1 hypothetical protein MANES_16G017200 [Manihot esculenta]	-	Q9XF70|Q9XF70_HEVBR:Thioredoxin h;OS=Hevea brasiliensis;|GO:0005623,|GO:0015035,|GO:0045454,GO:0006662,
DN102446_c0_g1_i5	UP	-	-	-	KNA12441.1:hypothetical protein SOVF_125700 [Spinacia oleracea]	-	A0A0K9R0V2|A0A0K9R0V2_SPIOL:Uncharacterized protein;OS=Spinacia oleracea;
DN121320_c3_g6_i2	UP	-	-	-	-	-	-
DN103215_c0_g1_i1	UP	[GO:0016020:membrane][GO:0006952:defense response][GO:0009607:response to biotic stimulus]	-	-	XP_021740721.1:MLPlike protein 423 [Chenopodium quinoa]	-	A0A0J8C0L5|A0A0J8C0L5_BETVU:Uncharacterized protein;OS=Beta vulgaris subsp. vulgaris.;|GO:0016020,|GO:0006952,GO:0009607,

geneid	trend	GO	Pathway	TF	NR	NT	Uniprot
DN116411_c2_g1_i1	UP	-	-	MDP0000215590:Malus domestica|WRKY|WRKY family protein	XP_021735260.1:uncharacterized protein ycf45like [Chenopodium quinoa]	gi|521447352|gb|GAHZ01073132.1|:TSA: Salicornia europaea Unigene88956_SeCKS transcribed RNA sequence	A0A0J8BMK8|A0A0J8BMK8_BETVU: Uncharacterized protein;OS=Beta vulgaris subsp. vulgaris.;
DN127467_c3_g1_i1	UP	[GO:0016021:integral component of membrane][GO:0016020:membrane][GO:0020037:heme binding][GO:0005506:iron ion binding][GO:0016709:oxidoreductase activity, acting on paired donors, with incorporation or reduction of molecular oxygen, NAD(P)H as one donor, and incorporation of one atom of oxygen][GO:0044550:secondary metabolite biosynthetic process]	-	Cc10_g03650:Coffea canephora|G2-like|G2-like family protein	XP_002282035.1:PREDICTED: cytochrome P450 CYP82D47 [Vitis vinifera]	-	F6H021|F6H021_VITVI:Putative uncharacterized protein;OS=Vitis vinifera;GN=VIT_18s0001g11560 {ECO:0000313|EMBL:CCB45898.1};|GO:0016021,GO:0016020,|GO:0020037,GO:0005506,GO:0016709,|GO:0044550,

geneid	trend	GO	Pathway	TF	NR	NT	Uniprot
DN123263_c2_g1_i8	UP	[GO:0016021:integral component of membrane]	-	Gh_D08G0309:Gossypium hirsutum|GRAS|GRAS family protein	XP_021717697.1:major facilitator superfamily domaincontaining protein 12like [Chenopodium quinoa] >XP_021717698.1 major facilitator superfamily domaincontaining protein 12like [Chenopodium quinoa]	gi|359487444|ref|XM_002270489.2|:PREDICTED: Vitis vinifera uncharacterized LOC100259681 (LOC100259681), mRNA	A0A0J8BJJ3|A0A0J8BJJ3_BETVU:Uncharacterized protein;OS=Beta vulgaris subsp. vulgaris.;|GO:0016021,
DN127822_c4_g2_i11	UP	[GO:0010287:plastoglobule]	-	-	XP_021737918.1:uncharacterized aarF domaincontaining protein kinase At5g05200, chloroplasticlike [Chenopodium quinoa]	gi|359486321|ref|XM_002275429.2|:PREDICTED: Vitis vinifera uncharacterized LOC100243380 (LOC100243380), mRNA	A0A0J8FNP0|A0A0J8FNP0_BETVU:Uncharacterized protein;OS=Beta vulgaris subsp. vulgaris.;|GO:0010287,

geneid	trend	GO	Pathway	TF	NR	NT	Uniprot
DN122851_c0_g1_i6	UP	[GO:0009941:chloroplast envelope][GO:0009535:chloroplast thylakoid membrane][GO:0009772:photosynthetic electron transport in photosystem II]	-	-	XP_021760335.1:rhodaneselike domaincontaining protein 4, chloroplastic [Chenopodium quinoa]	-	A0A0J8FXC4|A0A0J8FXC4_BETVU:Uncharacterized protein;OS=Beta vulgaris subsp. vulgaris.;|GO:0009941,GO:0009535,|GO:0009772,
DN121420_c1_g3_i1	UP	[GO:0005886:plasma membrane]	-	MDP0000291152:Malus domestica|HSF|HSF family protein	XP_021768269.1:protein ABHD17Clike [Chenopodium quinoa]	gi|502162356|ref|XM_004512420.1|:PREDICTED: Cicer arietinum abhydrolase domain-containing protein FAM108C1-like (LOC101511838), mRNA	A0A0J8CC16|A0A0J8CC16_BETVU:Uncharacterized protein;OS=Beta vulgaris subsp. vulgaris.;|GO:0005886,
DN127147_c2_g1_i7	UP	-	-	-	-	-	-

geneid	trend	GO	Pathway	TF	NR	NT	Uniprot
DN127281_c 1_g1_i3	UP	[GO:0005524:ATP binding][GO:0019538:protein metabolic process]	-	Achn116231:Actinidia chinensis|ARF|ARF family protein	XP_010678345.1:PREDICTED: ATPdependent Clp protease ATPbinding subunit ClpA homolog CD4B, chloroplastic [Beta vulgaris subsp. vulgaris] >KMT10922.1 hypothetical protein BVRB_5g113350 [Beta vulgaris subsp. vulgaris]	gi|4105130|gb|AF043539.1|AF043539:Spinacia oleracea ClpC protease (clpC) mRNA, chloroplast gene encoding chloroplast protein, complete cds	A0A0J8CBD9|A0A0J8CBD9_BETVU: Uncharacterized protein;OS=Beta vulgaris subsp. vulgaris.;|GO:0005524,|GO:0019538,
DN118894_c 4_g4_i6	UP	[GO:0005623:cell][GO:0015035:protein disulfide oxidoreductase activity][GO:0045454:cell redox homeostasis][GO:0006662:glycerol ether metabolic process]	[ko04621:NOD-like receptor signaling pathway][ko05418:Fluid shear stress and atherosclerosis]	Neem_16119_f_2:Azadirachta indica|bHLH|bHLH family protein	XP_021596332.1:thioredoxinlike protein CXXS1 [Manihot esculenta] >OAY26044.1 hypothetical protein MANES_16G017200 [Manihot esculenta]	-	Q9XF70|Q9XF70_HEVBR:Thioredoxin h;OS=Hevea brasiliensis;|GO:0005623,|GO:0015035,|GO:0045454,GO:0006662,

geneid	trend	GO	Pathway	TF	NR	NT	Uniprot
DN125807_c2_g10_i1	UP	-	[ko03040:Spliceosome][ko04010:MAPK signaling pathway][ko04141:Protein processing in endoplasmic reticulum][ko04144:Endocytosis][ko04213:Longevity regulating pathway - multiple species][ko04612:Antigen processing and presentation][ko04915:Estrogen signaling pathway][ko05134:Legionellosis][ko05145:Toxoplasmosis][ko05162:Measles][ko05164:Influenza A][ko05169:Epstein-Barr virus infection]	FANhyb_rscf00000233.1.g00002.1:Fragaria x ananassa|GATA|GATA family protein	XP_008784530.1:PREDICTED: heat shock cognate 70 kDa protein 2 [Phoenix dactylifera]	gi|306595234|gb|GQ851948.1|:Phytolacca acinosa heat shock cognate 70 kDa protein (HSP1) mRNA, partial cds	Q40323|Q40323_MEDSA:70 kD heatshockprotein;OS=Medicago sativa;

geneid	trend	GO	Pathway	TF	NR	NT	Uniprot
DN126322_c6_g1_i6	UP	[GO:0016301:kinase activity]	-	-	XP_010681152.1:PREDICTED: phosphatidylinositol 4kinase gamma 7 [Beta vulgaris subsp. vulgaris] >KMT08728.1 hypothetical protein BVRB_6g140010 [Beta vulgaris subsp. vulgaris]	gi|225424994|ref|XM_002266989.1|:PREDICTED: Vitis vinifera probable phosphatidylinositol 4-kinase type 2-beta At1g26270-like, transcript variant 1 (LOC100247983), mRNA	A0A0J8C9Q1|A0A0J8C9Q1_BETVU:Uncharacterized protein;OS=Beta vulgaris subsp. vulgaris.;|GO:0016301,
DN122154_c0_g1_i2	UP	[GO:0004806:triglyceride lipase activity][GO:0006629:lipid metabolic process]	[ko00100:Steroid biosynthesis][ko00561:Glycerolipid metabolism][ko00564:Glycerophospholipid metabolism][ko00565:Ether lipid metabolism][ko00590:Arachidonic acid metabolism][ko00591:Linoleic acid metabolism][ko00592:alpha-Linolenic	XP_011401233.1:Auxenochlorella protothecoides|CAMTA|CAMTA family protein	XP_021744689.1:triacylglycerol lipase SDP1like [Chenopodium quinoa]	gi|356571964|ref|XM_003554093.1|:PREDICTED: Glycine max triacylglycerol lipase SDP1-like (LOC100816093), mRNA	A0A0J8CN09|A0A0J8CN09_BETVU:Uncharacterized protein;OS=Beta vulgaris subsp. vulgaris.;|GO:0004806,|GO:0006629,

geneid	trend	GO	Pathway	TF	NR	NT	Uniprot
			acid metabolism]				
DN122990_c3_g2_i1	UP	[GO:0005524:ATP binding][GO:0016887:ATPase activity]	-	Prupe.3G046700.1.p:Prunus persica|GeBP|GeBP family protein	XP_020212393.1:ABC transporter F family member 5 [Cajanus cajan]	gi|224093877|ref|XM_002309995.1|:Populus trichocarpa ABC transporter family protein, mRNA	V7AVF8|V7AVF8_PHAVU:Uncharacterized protein;OS=Phaseolus vulgaris;|GO:0005524,GO:0016887,

geneid	trend	GO	Pathway	TF	NR	NT	Uniprot
DN127345_c1_g1_i5	UP	-	-	-	XP_021772846.1:dormancyassociated protein homolog 3like isoform X1 [Chenopodium quinoa] >XP_021772851.1 dormancyassociated protein homolog 3like isoform X1 [Chenopodium quinoa]	-	I1LB93|I1LB93_SOYBN:Uncharacterized protein;OS=Glycine max;

geneid	trend	GO	Pathway	TF	NR	NT	Uniprot
DN129073_c2_g1_i2	UP	[GO:0009535:chloroplast thylakoid membrane][GO:0016021:integral component of membrane][GO:0045263:proton-transporting ATP synthase complex, coupling factor F(o)][GO:0005524:ATP binding][GO:0015078:proton transmembrane transporter activity][GO:0015986:ATP synthesis coupled proton transport]	[ko00190:Oxidative phosphorylation][ko00195:Photosynthesis]	-	XP_021738786.1:ATP synthase subunit b', chloroplasticlike [Chenopodium quinoa]	gi|460392351|ref|XM_004241732.1|:PREDICTED: Solanum lycopersicum ATP synthase subunit b', chloroplastic-like (LOC101263124), mRNA	P31853|ATPX_SPIOL:ATP synthase subunit b', chloroplastic;OS=Spinacia oleracea;|GO:0009535,GO:0016021,GO:0045263,|GO:0005524,GO:0015078,|GO:0015986,
DN116988_c0_g2_i1	UP	[GO:0016021:integral component of membrane][GO:0008270:zinc ion binding][GO:0006355:regulation of transcription, DNA-templated]	-	Achn050401:Actinidia chinensis|NF-YA|NF-YA family protein	XP_006426611.1:hypothetical protein CICLE_v10026319mg [Citrus clementina] >ESR39851.1 hypothetical protein CICLE_v10026319mg [Citrus clementina]	gi|255550120|ref|XM_002516065.1|:Ricinus communis DNA binding protein, putative, mRNA	V4SRT9|V4SRT9_9ROSI:Uncharacterized protein;OS=Citrus clementina.;|GO:0016021,|GO:0008270,|GO:0006355,

geneid	trend	GO	Pathway	TF	NR	NT	Uniprot
DN126063_c2_g3_i5	UP	[GO:0003824:catalytic activity][GO:0046872:metal ion binding]	-	-	XP_021768824.1:stromal processing peptidase, chloroplasticlike [Chenopodium quinoa]	gi|359491891|ref|XM_002277508.2|:PREDICTED: Vitis vinifera uncharacterized LOC100266746 (LOC100266746), mRNA	A0A0K9QHM4|A0A0K9QHM4_SPIOL:Uncharacterized protein;OS=Spinacia oleracea;|GO:0003824,GO:0046872,
DN126055_c2_g1_i1	UP	-	-	-	KNA07651.1:hypothetical protein SOVF_169960 [Spinacia oleracea]	-	A0A0K9QM67|A0A0K9QM67_SPIOL:Uncharacterized protein;OS=Spinacia oleracea;
DN125873_c1_g3_i5	UP	[GO:0016021:integral component of membrane][GO:0055085:transmembrane transport]	-	-	XP_021743730.1:mechanosensitive ion channel protein 2, chloroplasticlike [Chenopodium quinoa] >XP_021743731.1 mechanosensitive ion channel protein 2, chloroplasticlike [Chenopodium quinoa]	gi|521485021|gb|GAHZ01045865.1|:TSA: Salicornia europaea Unigene54125_SeCKS transcribed RNA sequence	A0A0J8BFR1|A0A0J8BFR1_BETVU:Uncharacterized protein;OS=Beta vulgaris subsp. vulgaris.;|GO:0016021,|GO:0055085,

geneid	trend	GO	Pathway	TF	NR	NT	Uniprot
DN119823_c1_g1_i3	UP	[GO:0016597:amino acid binding][GO:0008152:metabolic process]	-	Rsa1.0_00123.1_g00043.1:Raphanus sativus|MYB_related|MYB_related family protein	XP_021747268.1:ACT domaincontaining protein ACR11like [Chenopodium quinoa]	gi|521449528|gb|GAHZ01070956.1|:TSA: Salicornia europaea Unigene86548_SeCKS transcribed RNA sequence	A0A0K9S1C1|A0A0K9S1C1_SPIOL:Uncharacterized protein;OS=Spinacia oleracea;|GO:0016597,|GO:0008152,
DN119630_c0_g3_i7	UP	[GO:0003824:catalytic activity][GO:0008152:metabolic process]	[ko00010:Glycolysis / Gluconeogenesis][ko00260:Glycine, serine and threonine metabolism][ko00680:Methane metabolism]	-	XP_010690037.1:PREDICTED: probable 2carboxyDarabinitol 1phosphatase [Beta vulgaris subsp. vulgaris] >KMT01663.1 hypothetical protein BVRB_9g211240 [Beta vulgaris subsp. vulgaris]	gi|521705083|gb|GAIA01015528.1|:TSA: Salicornia europaea Unigene16212_Se200S transcribed RNA sequence	A0A0J8BNS1|A0A0J8BNS1_BETVU:Uncharacterized protein;OS=Beta vulgaris subsp. vulgaris.;|GO:0003824,|GO:0008152,

geneid	trend	GO	Pathway	TF	NR	NT	Uniprot
DN119871_c0_g2_i3	UP	[GO:0051745:4-hydroxy-3-methylbut-2-en-1-yl diphosphate reductase activity][GO:0046872:metal ion binding][GO:0050992:dimethylallyl diphosphate biosynthetic process][GO:0019288:isopentenyl diphosphate biosynthetic process, methylerythritol 4-phosphate pathway]	[ko00900:Terpenoid backbone biosynthesis]	Achn232491:Actinidia chinensis|FAR1|FAR1 family protein	XP_021738562.1:4hydroxy3methylbut2enyl diphosphate reductase, chloroplasticlike [Chenopodium quinoa]	gi|359475262|ref|XM_002284623.2|:PREDICTED: Vitis vinifera 4-hydroxy-3-methylbut-2-enyl diphosphate reductase-like (LOC100267479), mRNA	A0A0K9QTN2|A0A0K9QTN2_SPIOL: Uncharacterized protein;OS=Spinacia oleracea;|GO:0051745,GO:0046872,|GO:0050992,GO:0019288,
DN129144_c6_g2_i1	UP	[GO:0016021:integral component of membrane][GO:0016765:transferase activity, transferring alkyl or aryl (other than methyl) groups]	[ko00130:Ubiquinone and other terpenoid-quinone biosynthesis]	-	XP_021845146.1:homogentisate phytyltransferase 1, chloroplastic [Spinacia oleracea] >XP_021845148.1 homogentisate phytyltransferase 1, chloroplastic [Spinacia oleracea] >KNA23057.1 hypothetical protein SOVF_027980 [Spinacia oleracea]	gi|356546074|ref|XM_003541409.1|:PREDICTED: Glycine max homogentisate phytyltransferase 1, chloroplastic-like (LOC100787771), mRNA	A0A0K9RW28|A0A0K9RW28_SPIOL: Uncharacterized protein;OS=Spinacia oleracea;|GO:0016021,|GO:0016765,

geneid	trend	GO	Pathway	TF	NR	NT	Uniprot
DN128881_c3_g1_i3	UP	[GO:0003676:nucleic acid binding][GO:0000166:nucleotide binding]	-	GSMUA_Achr2P20130_001:Musa acuminata|WRKY|WRKY family protein	XP_021722567.1:protein MEI2like 5 isoform X2 [Chenopodium quinoa]	gi|521717859|gb|GAIA01011866.1|:TSA: Salicornia europaea Unigene12484_Se200S transcribed RNA sequence	A0A0J8B3Y7|A0A0J8B3Y7_BETVU:Uncharacterized protein;OS=Beta vulgaris subsp. vulgaris.;|GO:0003676,GO:0000166,
DN126066_c2_g1_i10	UP	[GO:0004553:hydrolase activity, hydrolyzing O-glycosyl compounds][GO:0005975:carbohydrate metabolic process]	[ko00460:Cyanoamino acid metabolism][ko00500:Starch and sucrose metabolism][ko00940:Phenylpropanoid biosynthesis]	676737120:Sisymbrium irio|NF-YC|NF-YC family protein	KMT06914.1:hypothetical protein BVRB_6g152510 [Beta vulgaris subsp. vulgaris]	-	A0A0J8C3U9|A0A0J8C3U9_BETVU:Uncharacterized protein;OS=Beta vulgaris subsp. vulgaris.;|GO:0004553,|GO:0005975,
DN118978_c1_g1_i5	UP	[GO:0016624:oxidoreductase activity, acting on the aldehyde or oxo group of donors, disulfide as acceptor]	[ko00280:Valine, leucine and isoleucine degradation][ko00640:Propanoate metabolism]	-	XP_010679310.1:PREDICTED: 2oxoisovalerate dehydrogenase subunit alpha 1, mitochondrial [Beta vulgaris subsp. vulgaris] >KMT19677.1 hypothetical protein BVRB_1g010630	-	A0A0J8D1I8|A0A0J8D1I8_BETVU:Uncharacterized protein;OS=Beta vulgaris subsp. vulgaris.;|GO:0016624,

geneid	trend	GO	Pathway	TF	NR	NT	Uniprot
					[Beta vulgaris subsp. vulgaris]		
DN118519_c6_g1_i3	UP	[GO:0046658:anchored component of plasma membrane][GO:0051716:cellular response to stimulus]	-	-	XP_010684190.2:PREDICTED: LOW QUALITY PROTEIN: plasma membraneassociated cationbinding protein 1 [Beta vulgaris subsp. vulgaris]	-	A0A0J8C1K6|A0A0J8C1K6_BETVU:Uncharacterized protein;OS=Beta vulgaris subsp. vulgaris.;|GO:0046658,|GO:0051716,
DN121268_c3_g3_i1	UP	[GO:0046872:metal ion binding][GO:0030001:metal ion transport]	-	-	XP_021852054.1:basic prolinerich protein [Spinacia oleracea] >KNA24086.1 hypothetical protein SOVF_019100	-	A0A0K9RX54|A0A0K9RX54_SPIOL:Uncharacterized protein;OS=Spinacia oleracea;|GO:0046872,|GO:0030001,

geneid	trend	GO	Pathway	TF	NR	NT	Uniprot
					[Spinacia oleracea]		
DN124213_c0_g1_i4	UP	[GO:0003676:nucleic acid binding]	-	GSMUA_Achr6P04510_001:Musa acuminata|GATA|GATA family protein	XP_021769636.1:protein argonaute 1like [Chenopodium quinoa] >XP_021769644.1 protein argonaute 1like [Chenopodium quinoa]	gi|521737238|gb|GAIA01008197.1|:TSA: Salicornia europaea Unigene8527_Se200S transcribed RNA sequence	A0A0K9QWS6|A0A0K9QWS6_SPIOL:Uncharacterized protein;OS=Spinacia oleracea;|GO:0003676,
DN127674_c2_g2_i2	UP	[GO:0016020:membrane][GO:0005524:ATP binding][GO:0046872:metal ion binding][GO:0006457:protein folding][GO:0009408:response to heat]	[ko04141:Protein processing in endoplasmic reticulum]	GSVIVT01018226001:Vitis vinifera|ERF|ERF family protein	XP_021720580.1:dnaJ protein homolog ANJ1 [Chenopodium quinoa]	gi|521650521|gb|GAIA01041966.1|:TSA: Salicornia europaea Unigene47150_Se200S transcribed RNA sequence	P43644|DNJH_ATRNU:DnaJ protein homolog ANJ1;OS=Atriplex nummularia;|GO:0016020,|GO:0005524,GO:0046872,|GO:0006457,GO:0009408,

geneid	trend	GO	Pathway	TF	NR	NT	Uniprot
DN121257_c1_g1_i2	UP	[GO:0005524:ATP binding][GO:0006457:protein folding][GO:0009408:response to heat]	-	ONIVA04G19030.1: Oryza nivara|MYB|MYB family protein	XP_021756546.1:chaperone protein dnaJ A7A, chloroplasticlike [Chenopodium quinoa]	-	A0A0J8BXC4|A0A0J8BXC4_BETVU: Uncharacterized protein;OS=Beta vulgaris subsp. vulgaris.;|GO:0005524,|GO:0006457,GO:0009408,
DN119643_c2_g3_i1	UP	[GO:0009535:chloroplast thylakoid membrane][GO:0016021:integral component of membrane][GO:0009523:photosystem II][GO:0015979:photosynthesis][GO:0042549:photosystem II stabilization]	[ko00195:Photosynthesis]	-	XP_021770252.1:photosystem II reaction center W protein, chloroplasticlike [Chenopodium quinoa]	-	A0A0J8F3G1|A0A0J8F3G1_BETVU:Uncharacterized protein;OS=Beta vulgaris subsp. vulgaris.;|GO:0009535,GO:0016021,GO:0009523,|GO:0015979,GO:0042549,

geneid	trend	GO	Pathway	TF	NR	NT	Uniprot
DN125290_c9_g1_i5	UP	-	-	-	XP_010670132.1:PREDICTED: uncharacterized protein LOC104887237 [Beta vulgaris subsp. vulgaris] >KMT17233.1 hypothetical protein BVRB_2g039340 [Beta vulgaris subsp. vulgaris]	gi|255580718|ref|XM_002531135.1|:Ricinus communis conserved hypothetical protein, mRNA	A0A0J8CZ85|A0A0J8CZ85_BETVU:Uncharacterized protein;OS=Beta vulgaris subsp. vulgaris.;
DN118603_c2_g1_i1	UP	[GO:0005524:ATP binding][GO:0004674:protein serine/threonine kinase activity]	-	RrC264_p4:Raphanus raphanistrum|NF-YC|NF-YC family protein	KMT05025.1:hypothetical protein BVRB_7g171850 [Beta vulgaris subsp. vulgaris]	-	A0A0J8BU19|A0A0J8BU19_BETVU:Uncharacterized protein;OS=Beta vulgaris subsp. vulgaris.;|GO:0005524,GO:0004674,

geneid	trend	GO	Pathway	TF	NR	NT	Uniprot
DN115222_c1_g2_i2	UP	[GO:0009941:chloroplast envelope][GO:0009535:chloroplast thylakoid membrane][GO:0016021:integral component of membrane][GO:0005886:plasma membrane][GO:0051537:2 iron, 2 sulfur cluster binding][GO:0045158:electron transporter, transferring electrons within cytochrome b6/f complex of photosystem II activity][GO:0046872:metal ion binding][GO:0009496:plastoquinol--plastocyanin reductase activity][GO:0010196:nonphotochemical quenching][GO:0015979:photosynthesis]	[ko00195:Photosynthesis]	-	XP_010689313.1:PREDICTED: cytochrome b6f complex ironsulfur subunit, chloroplastic [Beta vulgaris subsp. vulgaris] >KMT02215.1 hypothetical protein BVRB_9g206750 [Beta vulgaris subsp. vulgaris]	gi|225447690|ref|XM_002276734.1|:PREDICTED: Vitis vinifera cytochrome b6-f complex iron-sulfur subunit, chloroplastic-like, transcript variant 1 (LOC100256300), mRNA	A0A0J8BLD8|A0A0J8BLD8_BETVU:Cytochrome b6-f complex iron-sulfur subunit;OS=Beta vulgaris subsp. vulgaris.;|GO:0009941,GO:0009535,GO:0016021,GO:0005886,|GO:0051537,GO:0045158,GO:0046872,GO:0009496,|GO:0010196,GO:0015979,

geneid	trend	GO	Pathway	TF	NR	NT	Uniprot
DN122006_c5_g3_i2	UP	[GO:0051537:2 iron, 2 sulfur cluster binding][GO:0010277:chlorophyllide a oxygenase [overall] activity]	[ko00860:Porphyrin and chlorophyll metabolism]	-	XP_021724418.1:pheophorbide a oxygenase, chloroplasticlike [Chenopodium quinoa]	gi|349707332|emb|FQ395388.1|:Vitis vinifera clone SS0AFA16YD03	A0A0K9QXU3|A0A0K9QXU3_SPIOL: Uncharacterized protein;OS=Spinacia oleracea;|GO:0051537,GO:0010277,
DN126429_c5_g2_i9	UP	[GO:0009501:amyloplast][GO:0009507:chloroplast][GO:0004373:glycogen (starch) synthase activity][GO:0019252:starch biosynthetic process]	[ko00500:Starch and sucrose metabolism][ko02026:Biofilm formation - Escherichia coli]	BGIOSGA032671-PA:Oryza sativa subsp. indica|FAR1|FAR1 family protein	XP_010693244.1:PREDICTED: granulebound starch synthase 2, chloroplastic/amyloplastic [Beta vulgaris subsp. vulgaris] >XP_010693245.1 PREDICTED: granulebound starch synthase 2, chloroplastic/amyloplastic [Beta vulgaris subsp. vulgaris] >KMS99236.1 hypothetical protein BVRB_2g046740 [Beta vulgaris subsp. vulgaris]	gi|77176913|gb|DQ178026.1|:Amaranthus cruentus starch synthase II (SsII) mRNA, complete cds	A0A0J8BE99|A0A0J8BE99_BETVU:Starch synthase, chloroplastic/amyloplastic;OS=Beta vulgaris subsp. vulgaris.;|GO:0009501,GO:0009507,|GO:0004373,|GO:0019252,

geneid	trend	GO	Pathway	TF	NR	NT	Uniprot
DN101630_c0_g1_i2	UP	[GO:0009941:chloroplast envelope][GO:0009570:chloroplast stroma][GO:0016020:membrane][GO:0005634:nucleus][GO:0005524:ATP binding][GO:0004386:helicase activity][GO:0003723:RNA binding][GO:0008270:zinc ion binding]	-	GSBRNA2T00069970001:Brassica napus|MYB_related|MYB_related family protein	XP_010680184.1:PREDICTED: DEADbox ATPdependent RNA helicase 3, chloroplastic [Beta vulgaris subsp. vulgaris] >KMT09135.1 hypothetical protein BVRB_6g132420 [Beta vulgaris subsp. vulgaris]	gi|512399441|gb|GAJZ01001729.1|:TSA: Prunus avium Pa_03369 transcribed RNA sequence	A0A0J8C5V5|A0A0J8C5V5_BETVU:Uncharacterized protein;OS=Beta vulgaris subsp. vulgaris.;|GO:0009941,GO:0009570,GO:0016020,GO:0005634,|GO:0005524,GO:0004386,GO:0003723,GO:0008270,
DN122709_c0_g1_i4	UP	[GO:0016021:integral component of membrane]	-	-	XP_018851206.1:PREDICTED: ribosome biogenesis protein NSA2 homolog [Juglans regia]	gi|478161515|gb|GAHK01015088.1|:TSA: Jatropha curcas Unigene2542_JC-CK_1A transcribed RNA sequence	W9SF31|W9SF31_9ROSA:Uncharacterized protein;OS=Morus notabilis.;|GO:0016021,

geneid	trend	GO	Pathway	TF	NR	NT	Uniprot
DN116773_c0_g1_i5	UP	[GO:0009570:chloroplast stroma][GO:0052656:L-isoleucine transaminase activity][GO:0052654:L-leucine transaminase activity][GO:0052655:L-valine transaminase activity][GO:0009082:branched-chain amino acid biosynthetic process][GO:0008652:cellular amino acid biosynthetic process]	[ko00270:Cysteine and methionine metabolism][ko00280:Valine, leucine and isoleucine degradation][ko00290:Valine, leucine and isoleucine biosynthesis][ko00770:Pantothenate and CoA biosynthesis]	Achn299211:Actinidia chinensis|MYB_related|MYB_related family protein	XP_021858624.1:branchedchain amino acid aminotransferase 2, chloroplasticlike [Spinacia oleracea]	gi|521563591|gb|GAIA01074856.1|:TSA: Salicornia europaea Unigene90803_Se200S transcribed RNA sequence	A0A0J8CAL5|A0A0J8CAL5_BETVU:Branched-chain-amino-acid aminotransferase;OS=Beta vulgaris subsp. vulgaris.;|GO:0009570,|GO:0052656,GO:0052654,GO:0052655,|GO:0009082,GO:0008652,
DN111716_c0_g1_i1	UP	[GO:0005840:ribosome][GO:0003735:structural constituent of ribosome][GO:0006412:translation]	[ko03010:Ribosome]	-	XP_010677124.1:PREDICTED: 50S ribosomal protein L19, chloroplastic [Beta vulgaris subsp. vulgaris] >KMT11812.1 hypothetical protein BVRB_5g105130 [Beta vulgaris subsp. vulgaris]	-	A0A0J8CE33|A0A0J8CE33_BETVU:Uncharacterized protein;OS=Beta vulgaris subsp. vulgaris.;|GO:0005840,|GO:0003735,|GO:0006412,

geneid	trend	GO	Pathway	TF	NR	NT	Uniprot
DN123400_c0_g2_i4	UP	[GO:0009507:chloroplast][GO:0051537:2 iron, 2 sulfur cluster binding][GO:0009055:electron transfer activity][GO:0046872:metal ion binding][GO:0022900:electron transport chain]	[ko00195:Photosynthesis]	-	O04683.1:RecName: Full=Ferredoxin1, chloroplastic; AltName: Full=Ferredoxin I; Flags: Precursor >AAB61593.1 ferredoxin I precursor [Mesembryanthemum crystallinum]	gi|388510581|gb|BT143563.1|:Medicago truncatula clone JCVI-FLMt-7F8 unknown mRNA	O04683|FER1_MESCR:Ferredoxin-1, chloroplastic;OS=Mesembryanthemum crystallinum;|GO:0009507,|GO:0051537,GO:0009055,GO:0046872,|GO:0022900,
DN114912_c1_g2_i3	UP	[GO:0005623:cell][GO:0004601:peroxidase activity][GO:0045454:cell redox homeostasis]	-	-	NP_001289773.1:peroxiredoxin2B [Nelumbo nucifera] >ABN46981.1 thioredoxindependent peroxidase [Nelumbo nucifera]	gi|512400048|gb|GAJZ01001122.1|:TSA: Prunus avium Pa_02167 transcribed RNA sequence	A3FPF4|A3FPF4_NELNU:Thioredoxin-dependent peroxidase;OS=Nelumbo nucifera;|GO:0005623,|GO:0004601,|GO:0045454,

geneid	trend	GO	Pathway	TF	NR	NT	Uniprot
DN120421_c2_g7_i3	UP	-	-	-	XP_010680131.1:PREDICTED: B2 protein [Beta vulgaris subsp. vulgaris] >XP_010680134.1 PREDICTED: B2 protein [Beta vulgaris subsp. vulgaris] >KMT09095.1 hypothetical protein BVRB_6g132030 [Beta vulgaris subsp. vulgaris]	gi|225459928|ref|XM_002265007.1|:PREDICTED: Vitis vinifera B2 protein-like (LOC100256906), mRNA	A0A0J8C5R3|A0A0J8C5R3_BETVU:Uncharacterized protein;OS=Beta vulgaris subsp. vulgaris.;

geneid	trend	GO	Pathway	TF	NR	NT	Uniprot
DN128341_c3_g5_i2	UP	[GO:0009941:chloroplast envelope][GO:0009570:chloroplast stroma][GO:0005739:mitochondrion][GO:0005774:vacuolar membrane][GO:0005524:ATP binding][GO:0009704:de-etiolation][GO:0006457:protein folding][GO:0045037:protein import into chloroplast stroma][GO:0010157:response to chlorate][GO:0009651:response to salt stress][GO:0009414:response to water deprivation]	[ko04141:Protein processing in endoplasmic reticulum][ko04151:PI3K-Akt signaling pathway][ko04626:Plant-pathogen interaction][ko04657:IL-17 signaling pathway][ko04915:Estrogen signaling pathway][ko04918:Thyroid hormone synthesis][ko05200:Pathways in cancer][ko05215:Prostate cancer][ko05418:Fluid shear stress and atherosclerosis]	OMERI06G27590.3:Oryza meridionalis|bZIP|bZIP family protein	XP_021718317.1:heat shock protein 905, chloroplasticlike [Chenopodium quinoa]	gi|512399553|gb|GAJZ01001617.1|:TSA: Prunus avium Pa_03123 transcribed RNA sequence	A0A0J8C9R5|A0A0J8C9R5_BETVU:Uncharacterized protein;OS=Beta vulgaris subsp. vulgaris.;|GO:0009941,GO:0009570,GO:0005739,GO:0005774,|GO:0005524,|GO:0009704,GO:0006457,GO:0045037,GO:0010157,GO:0009651,GO:0009414,

geneid	trend	GO	Pathway	TF	NR	NT	Uniprot
DN120396_c4_g2_i2	UP	[GO:0043565:sequence-specific DNA binding][GO:0003700:DNA-binding transcription factor activity][GO:0045893:positive regulation of transcription, DNA-templated]	-	Bv4_089910_wkkq.t1:Beta vulgaris|WRKY|WRKY family protein	XP_010675876.1:PREDICTED: probable WRKY transcription factor 20 [Beta vulgaris subsp. vulgaris] >KMT12903.1 hypothetical protein BVRB_4g089910 [Beta vulgaris subsp. vulgaris]	gi|478159668|gb|GAHK01016935.1|:TSA: Jatropha curcas Unigene4389_JC-CK_1A transcribed RNA sequence	A0A0J8CLH0|A0A0J8CLH0_BETVU:Uncharacterized protein;OS=Beta vulgaris subsp. vulgaris.;|GO:0043565,GO:0003700,|GO:0045893,
DN125554_c4_g3_i4	UP	-	-	-	XP_021857713.1:FRIGIDAlike protein 4a [Spinacia oleracea] >KNA24200.1 hypothetical protein SOVF_016610 [Spinacia oleracea]	gi|521675712|gb|GAIA01029354.1|:TSA: Salicornia europaea Unigene32190_Se200S transcribed RNA sequence	A0A0K9RXD8|A0A0K9RXD8_SPIOL: Uncharacterized protein;OS=Spinacia oleracea;
DN123040_c3_g2_i2	UP	[GO:0046872:metal ion binding][GO:0003676:nucleic acid binding]	-	Sp_031720_pwzj.t1: Spinacia oleracea|C2H2|C2H2 family protein	XP_021759526.1:zinc finger protein ZAT10like [Chenopodium quinoa]	gi|521532849|gb|GAHZ01006876.1|:TSA: Salicornia europaea Unigene7188_SeCKS transcribed RNA sequence	A0A0K9RUY3|A0A0K9RUY3_SPIOL: Uncharacterized protein;OS=Spinacia oleracea;|GO:0046872,GO:0003676,

geneid	trend	GO	Pathway	TF	NR	NT	Uniprot
DN117703_c1_g2_i3	UP	-	-	-	XP_010676768.1:PREDICTED: uncharacterized protein LOC104892509 [Beta vulgaris subsp. vulgaris] >KMT12354.1 hypothetical protein BVRB_5g102890 [Beta vulgaris subsp. vulgaris]	-	A0A0J8CJS7|A0A0J8CJS7_BETVU:Uncharacterized protein;OS=Beta vulgaris subsp. vulgaris.;
DN116450_c2_g1_i3	DOWN	[GO:0016021:integral component of membrane]	[ko04142:Lysosome]	-	XP_021863047.1:cystinosin homolog [Spinacia oleracea] >KNA20439.1 hypothetical protein SOVF_052430 [Spinacia oleracea]	gi|224141270|ref|XM_002323962.1|:Populus trichocarpa predicted protein, mRNA	A0A0K9RNF2|A0A0K9RNF2_SPIOL:Uncharacterized protein;OS=Spinacia oleracea;|GO:0016021,

geneid	trend	GO	Pathway	TF	NR	NT	Uniprot
DN118669_c1_g3_i1	DOWN	[GO:0033180:proton-transporting V-type ATPase, V1 domain][GO:0005524:ATP binding][GO:0046961:proton-transporting ATPase activity, rotational mechanism][GO:0015991:ATP hydrolysis coupled proton transport][GO:0046034:ATP metabolic process]	[ko00190:Oxidative phosphorylation][ko04145:Phagosome][ko04150:mTOR signaling pathway][ko04721:Synaptic vesicle cycle][ko04966:Collecting duct acid secretion][ko05110:Vibrio cholerae infection][ko05120:Epithelial cell signaling in Helicobacter pylori infection][ko05323:Rheumatoid arthritis]	-	CAC33578.1:putative vacuolar ATP Synthase subunit A [Mesembryanthemum crystallinum]	gi|13235339|emb|AJ276326.1|:Mesembryanthemum crystallinum mRNA for putative vacuolar ATP synthase subunit A (vatA gene)	Q9AVU8|Q9AVU8_MESCR:Putative vacuolar ATP Synthase subunit A;OS=Mesembryanthemum crystallinum;|GO:0033180,|GO:0005524,GO:0046961,|GO:0015991,GO:0046034,
DN127317_c1_g4_i1	DOWN	[GO:0016021:integral component of membrane][GO:0020037:heme binding][GO:0046872:metal ion binding]	-	-	OWM82889.1:hypothetical protein CDL15_Pgr005289 [Punica granatum]	gi|521698730|gb|GAIA01018069.1|:TSA: Salicornia europaea Unigene19023_Se200S transcribed RNA sequence	A0A0J8BVI8|A0A0J8BVI8_BETVU:Uncharacterized protein;OS=Beta vulgaris subsp. vulgaris.;|GO:0016021,|GO:0020037,GO

geneid	trend	GO	Pathway	TF	NR	NT	Uniprot
							:0046872,
DN128845_c1_g1_i2	DOWN	[GO:0004553:hydrolase activity, hydrolyzing O-glycosyl compounds][GO:0005975:carbohydrate metabolic process]	[ko00460:Cyanoamino acid metabolism][ko00500:Starch and sucrose metabolism][ko00940:Phenylpropanoid biosynthesis]	Lus10009218:Linum usitatissimum|C2H2|C2H2 family protein	XP_010675614.1:PREDICTED: betaglucosidase 18like [Beta vulgaris subsp. vulgaris]	-	A0A0J8CLZ5|A0A0J8CLZ5_BETVU:Uncharacterized protein;OS=Beta vulgaris subsp. vulgaris.;|GO:0004553,|GO:0005975,
DN122356_c0_g1_i12	DOWN	[GO:0005634:nucleus][GO:0004842:ubiquitin-protein transferase activity][GO:0008270:zinc ion binding][GO:0031347:regulation of defense response]	-	Zosma25g00280.1:Zostera marina|B3|B3 family protein	KNA13187.1:hypothetical protein SOVF_119030, partial [Spinacia oleracea]	-	A0A0K9R319|A0A0K9R319_SPIOL:Uncharacterized protein;OS=Spinacia oleracea;|GO:0005634,|GO:0004842,GO:0008270,|GO:0031347,
DN126591_c0_g1_i4	DOWN	[GO:0008171:O-methyltransferase activity]	[ko00940:Phenylpropanoid biosynthesis]	-	XP_021730855.1:caffeic acid 3Omethyltransferase like [Chenopodium	-	A0A0K9R160|A0A0K9R160_SPIOL:Uncharacterized protein;OS=Spinacia

geneid	trend	GO	Pathway	TF	NR	NT	Uniprot
					quinoa]		oleracea;|GO:0008171,
DN128429_c0_g3_i3	DOWN	-	-	Brast01G380000.1.p:Brachypodium stacei|NAC|NAC family protein	XP_010687943.1:PREDICTED: serine/threonineprotein phosphatase 1 regulatory subunit 10 [Beta vulgaris subsp. vulgaris]	-	A0A0K9RMJ3|A0A0K9RMJ3_SPIOL:Uncharacterized protein;OS=Spinacia oleracea;
DN126591_c0_g1_i6	DOWN	[GO:0008171:O-methyltransferase activity]	[ko00940:Phenylpropanoid biosynthesis]	-	XP_021730855.1:caffeic acid 3Omethyltransferase like [Chenopodium quinoa]	-	A0A0K9R160|A0A0K9R160_SPIOL:Uncharacterized protein;OS=Spinacia oleracea;|GO:0008171,
DN121368_c0_g2_i1	DOWN	-	-	-	-	gi|347597319|gb|HQ538716.1|:Phytolacca acinosa metallothionein mRNA, complete cds	-

geneid	trend	GO	Pathway	TF	NR	NT	Uniprot
DN118591_c0_g1_i2	DOWN	-	-	-	ABC73397.1:thaumatinlike protein [Nepenthes gracilis] >ACU31844.1 thaumatinlike protein [Nepenthes gracilis]	gi|470137922|ref|XM_004304661.1|:PREDICTED: Fragaria vesca subsp. vesca thaumatin-like protein-like (LOC101297567), mRNA	Q0QJL7|Q0QJL7_NEPGR:Thaumatin-like protein;OS=Nepenthes gracilis;
DN123754_c4_g1_i6	DOWN	[GO:0004781:sulfate adenylyltransferase (ATP) activity][GO:0000103:sulfate assimilation]	[ko00230:Purine metabolism][ko00261:Monobactam biosynthesis][ko00450:Selenocompound metabolism][ko00920:Sulfur metabolism]	-	XP_021764054.1:ATP sulfurylase 1, chloroplasticlike [Chenopodium quinoa]	gi|255552026|ref|XM_002517012.1|:Ricinus communis sulfate adenylyltransferase, putative, mRNA	A0A0K9QLM5|A0A0K9QLM5_SPIOL:Uncharacterized protein;OS=Spinacia oleracea;|GO:0004781,|GO:0000103,
DN127160_c0_g5_i8	DOWN	-	-	mrna28785.1-v1.0-hybrid:Fragaria vesca|bHLH|bHLH family protein	XP_021838746.1:probable aldoketo reductase 2 isoform X1 [Spinacia oleracea]	gi|225433669|ref|XM_002265891.1|:PREDICTED: Vitis vinifera auxin-induced protein PCNT115-like, transcript variant 1 (LOC100246287),	A0A0J8CN69|A0A0J8CN69_BETVU:Uncharacterized protein;OS=Beta vulgaris subsp. vulgaris.;

geneid	trend	GO	Pathway	TF	NR	NT	Uniprot
						mRNA	
DN126749_c 1_g1_i2	DOWN	[GO:0003871:5-methyltetrahydropter oyltriglutamate-homocysteine S-methyltransferase activity][GO:0008270:zinc ion binding][GO:0009086:methionine biosynthetic process]	[ko00270:Cysteine and methionine metabolism][ko0045 0:Selenocompound metabolism]	-	XP_010677810.2:P REDICTED: 5methyltetrahydropt eroyltriglutamateho mocysteine methyltransferase [Beta vulgaris subsp. vulgaris] >KMT11311.1 hypothetical protein BVRB_5g109920 [Beta vulgaris subsp. vulgaris]	gi|1814402|gb|U848 89.1|MCU84889:Me sembryanthemum crystallinum methionine synthase (MetE) mRNA, complete cds	A0A0J8F5S9|A0A0J 8F5S9_BETVU:Unc haracterized protein;OS=Beta vulgaris subsp. vulgaris.;|GO:00038 71,GO:0008270,|GO :0009086,

geneid	trend	GO	Pathway	TF	NR	NT	Uniprot
DN124288_c0_g4_i11	DOWN	[GO:0005634:nucleus][GO:0003677:DNA binding][GO:0003700:DNA-binding transcription factor activity][GO:0006351:transcription, DNA-templated]	-	Sp_031310_cdtc.t1: Spinacia oleracea|ERF|ERF family protein	XP_021716591.1:ethyleneresponsive transcription factor RAP212like [Chenopodium quinoa]	gi|502156607|ref|XM_004510502.1|:PREDICTED: Cicer arietinum ethylene-responsive transcription factor RAP2-12-like (LOC101512899), transcript variant X2, mRNA	G9JXL3|G9JXL3_ATRCA:Dehydration-responsive element binding protein;OS=Atriplex canescens;|GO:0005634,|GO:0003677,GO:0003700,|GO:0006351,
DN127052_c4_g2_i6	DOWN	[GO:0001735:prenylcysteine oxidase activity][GO:0030328:prenylcysteine catabolic process]	[ko00900:Terpenoid backbone biosynthesis]	-	XP_021859846.1:farnesylcysteine lyase [Spinacia oleracea] >KNA17032.1 hypothetical protein SOVF_083490 [Spinacia oleracea]	gi|521515269|gb|GAHZ01016294.1|:TSA: Salicornia europaea Unigene17144_SeCKS transcribed RNA sequence	A0A0K9RDV8|A0A0K9RDV8_SPIOL: Uncharacterized protein;OS=Spinacia oleracea;|GO:0001735,|GO:0030328,
DN118447_c3_g6_i6	DOWN	[GO:0016021:integral component of membrane]	-	-	XP_021734187.1:tetraspanin3like [Chenopodium quinoa]	gi|460226435|gb|GABL01039922.1|:TSA: Camelina sativa comp49900_c0_seq1 transcribed RNA sequence	A0A0K9QCE4|A0A0K9QCE4_SPIOL: Uncharacterized protein;OS=Spinacia oleracea;|GO:0016021,

geneid	trend	GO	Pathway	TF	NR	NT	Uniprot
DN124496_c2_g1_i7	DOWN	[GO:0016758:transferase activity, transferring hexosyl groups][GO:0008152:metabolic process]	-	AA31G00555:Aethionema arabicum|CO-like|CO-like family protein	XP_021752804.1:UDPglycosyltransferase 74E2like [Chenopodium quinoa]	gi|242199343|gb|GQ221688.1|:Citrus sinensis UDP-glucosyltransferase family 1 protein (UGT3) gene, complete cds	A0A0J8CU34|A0A0J8CU34_BETVU:Glycosyltransferase;OS=Beta vulgaris subsp. vulgaris.;|GO:0016758,|GO:0008152,
DN118479_c0_g1_i10	DOWN	[GO:0016021:integral component of membrane][GO:0015297:antiporter activity]	-	TRIUR3_11943-P1:Triticum urartu|ERF|ERF family protein	XP_021722645.1:protein DETOXIFICATION 33like [Chenopodium quinoa]	gi|512397657|gb|GAJZ01003513.1|:TSA: Prunus avium Pa_07596 transcribed RNA sequence	A0A0K9R9I6|A0A0K9R9I6_SPIOL:Protein DETOXIFICATION;OS=Spinacia oleracea;|GO:0016021,|GO:0015297,GO:0015238,
DN124086_c0_g1_i2	DOWN	-	-	-	XP_010679967.1:PREDICTED: type2 histone deacetylase 1 [Beta vulgaris subsp. vulgaris] >KMT09638.1 hypothetical protein BVRB_6g130900 [Beta vulgaris subsp. vulgaris]	-	A0A0J8CCE1|A0A0J8CCE1_BETVU:Uncharacterized protein;OS=Beta vulgaris subsp. vulgaris.;

geneid	trend	GO	Pathway	TF	NR	NT	Uniprot
DN128482_c1_g3_i18	DOWN	[GO:0005524:ATP binding][GO:0004672:protein kinase activity]	-	Itr_sc000748.1_g00024.1:Ipomoea trifida|MYB_related|MYB_related family protein	XP_021773872.1:probable LRR receptorlike serine/threonineprotein kinase At1g53430 [Chenopodium quinoa]	gi|225461769|ref|XM_002283485.1|:PREDICTED: Vitis vinifera probable LRR receptor-like serine/threonine-protein kinase At1g53430-like (LOC100243601), mRNA	M5WZZ0|M5WZZ0_PRUPE:Uncharacterized protein;OS=Prunus persica;|GO:0005524,GO:0004672,
DN116948_c5_g2_i2	DOWN	[GO:0005509:calcium ion binding]	[ko04626:Plant-pathogen interaction]	-	XP_021833315.1:probable calciumbinding protein CML49 [Prunus avium]	gi|521711801|gb|GAIA01012194.1|:TSA: Salicornia europaea Unigene12814_Se200S transcribed RNA sequence	M5WGJ3|M5WGJ3_PRUPE:Uncharacterized protein;OS=Prunus persica;|GO:0005509,

geneid	trend	GO	Pathway	TF	NR	NT	Uniprot
DN120275_c0_g8_i3	DOWN	[GO:0005634:nucleus][GO:0005525:GTP binding][GO:0003924:GTPase activity][GO:0006886:intracellular protein transport][GO:0006913:nucleocytoplasmic transport][GO:0007264:small GTPase mediated signal transduction]	[ko03008:Ribosome biogenesis in eukaryotes][ko03013:RNA transport][ko05166:HTLV-I infection][ko05169:Epstein-Barr virus infection]	-	AEM97804.1:Ran3A1 [Dimocarpus longan] >AEM97805.1 Ran3A2 [Dimocarpus longan] >AEM97806.1 Ran3A3 [Dimocarpus longan] >AEM97807.1 Ran3A4 [Dimocarpus longan] >AEM97808.1 Ran3A5 [Dimocarpus longan] >AEM97809.1 Ran3A6 [Dimocarpus longan] >AEM97810.1 Ran3A7 [Dimocarpus longan] >AEM97811.1 Ran3A8 [Dimocarpus longan]	gi|77745508|gb|DQ222522.1|:Solanum tuberosum clone 129C03 Ran/TC4-like protein mRNA, complete cds	G3F7S3|G3F7S3_9ROSI:GTP-binding nuclear protein;OS=Dimocarpus longan.;|GO:0005634,|GO:0005525,GO:0003924,|GO:0006886,GO:0006913,GO:0007264,

geneid	trend	GO	Pathway	TF	NR	NT	Uniprot
DN124509_c1_g1_i1	DOWN	[GO:0004489:methylenetetrahydrofolate reductase (NAD(P)H) activity][GO:0006555:methionine metabolic process][GO:0035999:tetrahydrofolate interconversion]	[ko00670:One carbon pool by folate][ko00720:Carbon fixation pathways in prokaryotes][ko01523:Antifolate resistance]	MDP0000229611:Malus domestica|HD-ZIP|HD-ZIP family protein	XP_021752218.1:methylenetetrahydrofolate reductase 2like [Chenopodium quinoa]	gi|521502980|gb|GAHZ01027940.1|:TSA: Salicornia europaea Unigene30767_SeCKS transcribed RNA sequence	A0A0J8CU87|A0A0J8CU87_BETVU:Methylenetetrahydrofolate reductase;OS=Beta vulgaris subsp. vulgaris.;|GO:0004489,|GO:0006555,GO:0035999,
DN122548_c5_g1_i1	DOWN	[GO:0051537:2 iron, 2 sulfur cluster binding][GO:0009055:electron transfer activity][GO:0022900:electron transport chain]	[ko00195:Photosynthesis]	-	XP_021716351.1:ferredoxin, root RB2like [Chenopodium quinoa]	-	A0A0J8BZV9|A0A0J8BZV9_BETVU:Uncharacterized protein;OS=Beta vulgaris subsp. vulgaris.;|GO:0051537,GO:0009055,|GO:0022900,

geneid	trend	GO	Pathway	TF	NR	NT	Uniprot
DN128634_c3_g5_i3	DOWN	[GO:0016021:integral component of membrane][GO:0005524:ATP binding][GO:0016887:ATPase activity]	-	Rsa1.0_03996.1_g00001.1:Raphanus sativus|MYB_related|MYB_related family protein	XP_021627833.1:ABC transporter G family member 29like [Manihot esculenta] >XP_021627834.1 ABC transporter G family member 29like [Manihot esculenta] >XP_021627835.1 ABC transporter G family member 29like [Manihot esculenta] >OAY36609.1 hypothetical protein MANES_11G034200 [Manihot esculenta] >OAY36610.1 hypothetical protein MANES_11G034200 [Manihot esculenta]	gi|356526082|ref|XM_003531601.1|:PREDICTED: Glycine max ABC transporter G family member 36-like (LOC100798588), mRNA	A0A061GIL4|A0A061GIL4_THECC:ABC-2 and Plant PDR ABC-type transporter family protein isoform 4;OS=Theobroma cacao;|GO:0016021,|GO:0005524,GO:0016887,

geneid	trend	GO	Pathway	TF	NR	NT	Uniprot
DN116519_c1_g1_i1	DOWN	[GO:0003824:catalytic activity][GO:0008152:metabolic process]	-	Zmw_sc02382.1.g00020.1:Zoysia matrella|bHLH|bHLH family protein	XP_009596461.1:PREDICTED: oxalateCoA ligaselike [Nicotiana tomentosiformis] >XP_009596462.1 PREDICTED: oxalateCoA ligaselike [Nicotiana tomentosiformis] >XP_018625107.1 PREDICTED: oxalateCoA ligaselike [Nicotiana tomentosiformis]	gi|356574490|ref|XM_003555332.1|:PREDICTED: Glycine max putative peroxisomal-coenzyme A synthetase-like (LOC100783685), mRNA	A0A0J8CWC4|A0A0J8CWC4_BETVU: Uncharacterized protein;OS=Beta vulgaris subsp. vulgaris.;|GO:0003824,|GO:0008152,
DN118452_c3_g1_i1	DOWN	[GO:0008236:serine-type peptidase activity]	-	-	XP_021718124.1:probable serine protease EDA2 [Chenopodium quinoa]	gi|521447136|gb|GAHZ01073348.1|:TSA: Salicornia europaea Unigene89197_SeCKS transcribed RNA sequence	A0A0J8C740|A0A0J8C740_BETVU:Uncharacterized protein;OS=Beta vulgaris subsp. vulgaris.;|GO:0008236,

geneid	trend	GO	Pathway	TF	NR	NT	Uniprot
DN121217_c1_g2_i5	DOWN	[GO:0008270:zinc ion binding]	-	-	KNA18067.1:hypothetical protein SOVF_074350 isoform B [Spinacia oleracea]	-	A0A0K9RGU1|A0A0K9RGU1_SPIOL: Uncharacterized protein;OS=Spinacia oleracea;|GO:0008270,
DN127885_c5_g1_i5	DOWN	[GO:0005886:plasma membrane]	-	Sme2.5_07423.1_g00002.1:Solanum melongena|TCP|TCP family protein	XP_010669670.1:PREDICTED: protein IQDOMAIN 1 [Beta vulgaris subsp. vulgaris] >KMT17702.1 hypothetical protein BVRB_2g036470 [Beta vulgaris subsp. vulgaris]	gi|359486692|ref|XM_002277246.2|:PREDICTED: Vitis vinifera protein IQ-DOMAIN 1-like (LOC100256490), mRNA	A0A0J8CVT6|A0A0J8CVT6_BETVU:Uncharacterized protein;OS=Beta vulgaris subsp. vulgaris.;|GO:0005886,
DN126305_c0_g4_i1	DOWN	-	[ko05162:Measles]	Cc04_g06620:Coffea canephora|MYB|MYB family protein	XP_010678120.1:PREDICTED: guanine nucleotidebinding protein subunit betalike protein [Beta vulgaris subsp. vulgaris] >KMT11115.1	gi|359491000|ref|XM_003634149.1|:PREDICTED: Vitis vinifera guanine nucleotide-binding protein subunit beta-like protein, transcript variant 2 (LOC100249027),	A0A0J8CBC4|A0A0J8CBC4_BETVU:Uncharacterized protein;OS=Beta vulgaris subsp. vulgaris.;

geneid	trend	GO	Pathway	TF	NR	NT	Uniprot
					hypothetical protein BVRB_5g111970 [Beta vulgaris subsp. vulgaris]	mRNA	
DN120737_c0_g1_i6	DOWN	[GO:0016866:intramolecular transferase activity][GO:0030244:cellulose biosynthetic process]	[ko00520:Amino sugar and nucleotide sugar metabolism]	-	OMO79897.1:hypothetical protein COLO4_24299 [Corchorus olitorius]	gi|502094461|ref|XM_004490166.1|:PREDICTED: Cicer arietinum alpha-1,4-glucan-protein synthase [UDP-forming]-like (LOC101496708), mRNA	A0A0D2RIS0|A0A0D2RIS0_GOSRA:Uncharacterized protein;OS=Gossypium raimondii;|GO:0016866,|GO:0030244,
DN123998_c4_g1_i7	DOWN	[GO:0046872:metal ion binding][GO:0016491:oxidoreductase activity]	[ko00270:Cysteine and methionine metabolism]	676709864:Sisymbrium irio|STAT|STAT family protein	XP_021766818.1:1aminocyclopropane1carboxylate oxidaselike [Chenopodium quinoa]	gi|520801|gb|L35152.1|DINACCA:Dianthus caryophyllus amino-cyclopropane carboxylic acid oxidase mRNA, complete cds	A0A0J8EM69|A0A0J8EM69_BETVU:Uncharacterized protein;OS=Beta vulgaris subsp. vulgaris.;|GO:0046872,GO:0016491,

geneid	trend	GO	Pathway	TF	NR	NT	Uniprot
DN127228_c3_g1_i2	DOWN	[GO:0046872:metal ion binding][GO:0016491:oxidoreductase activity]	-	676709864:Sisymbrium irio|STAT|STAT family protein	KMT05032.1:hypothetical protein BVRB_7g171920 [Beta vulgaris subsp. vulgaris]	-	A0A0J8BVB0|A0A0J8BVB0_BETVU: Uncharacterized protein;OS=Beta vulgaris subsp. vulgaris.;|GO:0046872,GO:0016491,
DN122984_c4_g1_i3	DOWN	[GO:0005634:nucleus][GO:0043565: sequence-specific DNA binding][GO:0003700:DNA-binding transcription factor activity]	-	Sp_154640_utwj.t1: Spinacia oleracea|HD-ZIP|HD-ZIP family protein	ACL81158.1:homeobox leucine zipper protein [Mirabilis jalapa]	gi|220702718|gb|FJ536224.1|:Mirabilis jalapa homeobox leucine zipper protein mRNA, complete cds	B8YIB0|B8YIB0_MIRJA:Homeobox leucine zipper protein;OS=Mirabilis jalapa;|GO:0005634,|GO:0043565,GO:0003700,
DN98030_c0_g1_i1	DOWN	-	-	-	-	-	-
DN127964_c0_g1_i9	DOWN	-	-	-	XP_021849429.1:uncharacterized protein LOC110789089 [Spinacia oleracea] >KNA05349.1 hypothetical protein SOVF_191210	gi|521743582|gb|GAIA01004218.1|:TSA: Salicornia europaea Unigene4502_Se200S transcribed RNA sequence	A0A0K9QDJ8|A0A0K9QDJ8_SPIOL:Uncharacterized protein;OS=Spinacia oleracea;

geneid	trend	GO	Pathway	TF	NR	NT	Uniprot
					[Spinacia oleracea]		
DN125355_c6_g2_i1	DOWN	[GO:0005737:cytoplasm][GO:0003999:adenine phosphoribosyltransferase activity][GO:0006168:adenine salvage][GO:0009116:nucleoside metabolic process]	[ko00230:Purine metabolism]	Oropetium_20150105_18128A:Oropetium thomaeum|bHLH|bHLH family protein	XP_010688209.1:PREDICTED: adenine phosphoribosyltransferase 1like [Beta vulgaris subsp. vulgaris]	gi|521734961|gb|GAIA01009428.1|:TSA: Salicornia europaea Unigene9794_Se200S transcribed RNA sequence	A0A0J8D490|A0A0J8D490_BETVU:Uncharacterized protein;OS=Beta vulgaris subsp. vulgaris.;|GO:0005737,|GO:0003999,|GO:0006168,GO:0009116,
DN124467_c1_g1_i4	DOWN	[GO:0008270:zinc ion binding]	-	Bostr.7128s0454.1.p:Boechera stricta|bHLH|bHLH family protein	XP_021743658.1:pentatricopeptide repeatcontaining protein At4g15720like [Chenopodium quinoa]	gi|225432321|ref|XM_002276194.1|:PREDICTED: Vitis vinifera pentatricopeptide repeat-containing protein At4g15720-like (LOC100249494), mRNA	A0A0J8D5U8|A0A0J8D5U8_BETVU:Uncharacterized protein;OS=Beta vulgaris subsp. vulgaris.;|GO:0008270,

geneid	trend	GO	Pathway	TF	NR	NT	Uniprot
DN117349_c0_g7_i3	DOWN	[GO:0005524:ATP binding][GO:0004356:glutamate-ammonia ligase activity][GO:0006542:glutamine biosynthetic process]	[ko00220:Arginine biosynthesis][ko00250:Alanine, aspartate and glutamate metabolism][ko00630:Glyoxylate and dicarboxylate metabolism][ko00910:Nitrogen metabolism][ko02020:Two-component system][ko04217:Necroptosis][ko04724:Glutamatergic synapse][ko04727:GABAergic synapse]	-	XP_021757390.1:glutamine synthetase cytosolic isozyme [Chenopodium quinoa]	gi|4884971|gb|AF145481.1|AF145481:Mesembryanthemum crystallinum cytosolic glutamine synthetase mRNA, partial cds	A0A0S3RZR8|A0A0S3RZR8_PHAAN: Glutamine synthetase;OS=Vigna angularis var. angularis.;|GO:0005524,GO:0004356,|GO:0006542,
DN119725_c1_g1_i6	DOWN	[GO:0016788:hydrolase activity, acting on ester bonds]	-	-	XP_021841488.1:poly(U)specific endoribonucleaseBlike [Spinacia oleracea] >KNA05270.1 hypothetical protein SOVF_191960	gi|502152664|ref|XM_004508976.1|:PREDICTED: Cicer arietinum poly(U)-specific endoribonuclease-B-like (LOC101494319),	A0A0K9QDD8|A0A0K9QDD8_SPIOL: Uncharacterized protein;OS=Spinacia oleracea;|GO:0016788,

geneid	trend	GO	Pathway	TF	NR	NT	Uniprot
					[Spinacia oleracea]	mRNA	
DN115017_c0_g1_i2	DOWN	-	-	Vradi03g04520.1:Vigna radiata|bZIP|bZIP family protein	XP_021764779.1:uncharacterized protein LOC110729352 isoform X1 [Chenopodium quinoa]	gi|521538857|gb|GAHZ01003857.1|:TSA: Salicornia europaea Unigene4146_SeCKS transcribed RNA sequence	A0A0K9Q946|A0A0K9Q946_SPIOL:Uncharacterized protein;OS=Spinacia oleracea;
DN125219_c1_g1_i3	DOWN	[GO:0016021:integral component of membrane][GO:0005524:ATP binding][GO:0008553:proton-exporting ATPase activity, phosphorylative mechanism][GO:0046872:metal ion binding][GO:0006754:ATP biosynthetic process]	[ko00190:Oxidative phosphorylation]	Lus10040166:Linum usitatissimum|GRAS|GRAS family protein	XP_010688454.1:PREDICTED: plasma membrane ATPase 4 [Beta vulgaris subsp. vulgaris] >KMT03296.1 hypothetical protein BVRB_8g198370 [Beta vulgaris subsp. vulgaris]	gi|411107727|gb|JX628604.1|:Sesuvium portulacastrum plasma membrane H+-ATPase mRNA, complete cds	A0A0J8EK02|A0A0J8EK02_BETVU:Plasma membrane ATPase;OS=Beta vulgaris subsp. vulgaris.;|GO:0016021,|GO:0005524,GO:0008553,GO:0046872,|GO:0006754,

geneid	trend	GO	Pathway	TF	NR	NT	Uniprot
DN128926_c4_g9_i4	DOWN	-	-	-	KMS64936.1:hypothetical protein BVRB_041110, partial [Beta vulgaris subsp. vulgaris]	gi|193084603|gb|EU795174.1|:Uncultured bacterium HF0010_04H24 genomic sequence	A0A0J7YMY6|A0A0J7YMY6_BETVU: Uncharacterized protein;OS=Beta vulgaris subsp. vulgaris.;
DN125074_c5_g2_i3	DOWN	[GO:0042409:caffeoyl-CoA O-methyltransferase activity][GO:0046872:metal ion binding][GO:0009809:lignin biosynthetic process]	[ko00360:Phenylalanine metabolism][ko00940:Phenylpropanoid biosynthesis][ko00941:Flavonoid biosynthesis][ko00945:Stilbenoid, diarylheptanoid and gingerol biosynthesis]	ONIVA06G05580.1:Oryza nivara|ERF|ERF family protein	XP_021753326.1:caffeoylCoA Omethyltransferase 5like [Chenopodium quinoa]	gi|2995933|gb|AF053553.1|AF053553:Mesembryanthemum crystallinum caffeoyl-CoA O-methyltransferase mRNA, complete cds	O65162|CAMT_MESCR:Caffeoyl-CoA O-methyltransferase;OS=Mesembryanthemum crystallinum;|GO:0042409,GO:0046872,|GO:0009809,
DN109845_c2_g1_i1	DOWN	-	-	-	XP_010680753.1:PREDICTED: nodulinrelated protein 1 [Beta vulgaris subsp. vulgaris] >KMT08996.1 hypothetical protein	-	A0A0J8F0D5|A0A0J8F0D5_BETVU:Uncharacterized protein;OS=Beta vulgaris subsp. vulgaris.;

geneid	trend	GO	Pathway	TF	NR	NT	Uniprot
					BVRB_6g137060 [Beta vulgaris subsp. vulgaris]		
DN127292_c2_g1_i10	DOWN	[GO:0016021:integral component of membrane][GO:0005524:ATP binding][GO:0008553:proton-exporting ATPase activity, phosphorylative mechanism][GO:0046872:metal ion binding][GO:0006754:ATP biosynthetic process]	[ko00190:Oxidative phosphorylation]	Lus10040166:Linum usitatissimum|GRAS|GRAS family protein	XP_021978447.1:plasma membrane ATPase 1like isoform X1 [Helianthus annuus] >OTG19584.1 putative Ptype ATPase [Helianthus annuus]	gi|33943130|gb|AY347715.1|:Juglans regia plasma membrane H+-ATPase (AHA1) mRNA, complete cds	H9CTG5|H9CTG5_9MYRT:Plasma membrane ATPase;OS=Melastoma malabathricum.;|GO:0016021,|GO:0005524,GO:0008553,GO:0046872,|GO:0006754,

geneid	trend	GO	Pathway	TF	NR	NT	Uniprot
DN122780_c0_g5_i4	DOWN	[GO:0016021:integral component of membrane][GO:0005215:transporter activity][GO:0006857:oligopeptide transport]	-	mrna00620.1-v1.0-hybrid:Fragaria vesca|BBR-BPC|BBR-BPC family protein	XP_010691376.1:PREDICTED: protein NRT1/ PTR FAMILY 1.2 [Beta vulgaris subsp. vulgaris] >KMT00859.1 hypothetical protein BVRB_9g221330 [Beta vulgaris subsp. vulgaris]	-	A0A0J8BLG2|A0A0J8BLG2_BETVU:Uncharacterized protein;OS=Beta vulgaris subsp. vulgaris.;|GO:0016021,|GO:0005215,|GO:0006857,
DN107066_c0_g2_i2	DOWN	[GO:0005622:intracellular][GO:0006886:intracellular protein transport]	[ko04138:Autophagy - yeast][ko04721:Synaptic vesicle cycle]	-	XP_018461232.1:PREDICTED: alphasoluble NSF attachment protein 2like [Raphanus sativus]	-	A0A078BTW2|A0A078BTW2_BRANA:BnaA07g16770D protein;OS=Brassica napus;|GO:0005622,|GO:0006886,

geneid	trend	GO	Pathway	TF	NR	NT	Uniprot
DN123240_c1_g1_i4	DOWN	[GO:0022625:cytosolic large ribosomal subunit][GO:0030687:preribosome, large subunit precursor][GO:0070180:large ribosomal subunit rRNA binding][GO:0030295:protein kinase activator activity][GO:0003735:structural constituent of ribosome][GO:0002181:cytoplasmic translation][GO:0045860:positive regulation of protein kinase activity][GO:0006414:translational elongation]	[ko03010:Ribosome]	-	XP_012839856.1:PREDICTED: 60S acidic ribosomal protein P1like [Erythranthe guttata] >XP_012839867.1 PREDICTED: 60S acidic ribosomal protein P1like [Erythranthe guttata]	gi|512394336|gb|GAJZ01006834.1|:TSA: Prunus avium Pa_23419 transcribed RNA sequence	D7TVT2|D7TVT2_VITVI:Putative uncharacterized protein;OS=Vitis vinifera;GN=VIT_02s0025g02780 {ECO:0000313|EMBL:CBI34607.3};|GO:0022625,GO:0030687,|GO:0070180,GO:0030295,GO:0003735,|GO:0002181,GO:0045860,GO:0006414,
DN127807_c0_g4_i11	DOWN	[GO:0003824:catalytic activity]	-	-	XP_021726427.1:CBS domaincontaining protein CBSX5like [Chenopodium quinoa]	-	A0A0K9RBC0|A0A0K9RBC0_SPIOL: Uncharacterized protein;OS=Spinacia oleracea;|GO:0003824,

geneid	trend	GO	Pathway	TF	NR	NT	Uniprot
DN121919_c7_g1_i3	DOWN	[GO:0016021:integral component of membrane][GO:0005524:ATP binding][GO:0016887:ATPase activity][GO:0006810:transport]	-	Rsa1.0_03996.1_g00001.1:Raphanus sativus|MYB_related|MYB_related family protein	ASM90224.1:ABC2 type transporter [Sesuvium portulacastrum]	gi|449483990|ref|XM_004156704.1|:PREDICTED: Cucumis sativus pleiotropic drug resistance protein 1-like (LOC101231370), mRNA	A0A0J8B5I1|A0A0J8B5I1_BETVU:Uncharacterized protein;OS=Beta vulgaris subsp. vulgaris.;|GO:0016021,|GO:0005524,GO:0016887,|GO:0006810,
DN118165_c1_g1_i3	DOWN	[GO:0005783:endoplasmic reticulum][GO:0005509:calcium ion binding][GO:0006457:protein folding]	[ko04141:Protein processing in endoplasmic reticulum][ko04145:Phagosome][ko04612:Antigen processing and presentation][ko05142:Chagas disease (American trypanosomiasis)][ko05166:HTLV-I infection]	-	KVH99491.1:Calreticulin, partial [Cynara cardunculus var. scolymus]	gi|3288108|emb|AJ002057.1|:Beta vulgaris mRNA for calreticulin	A0A118JZ42|A0A118JZ42_CYNCS:Calreticulin;OS=Cynara cardunculus var. scolymus;|GO:0005783,|GO:0005509,|GO:0006457,

geneid	trend	GO	Pathway	TF	NR	NT	Uniprot
DN111654_c0_g1_i1	DOWN	-	-	-	XP_007223660.1:heterogeneous nuclear ribonucleoprotein A2 homolog 1 [Prunus persica] >ONI28070.1 hypothetical protein PRUPE_1G121000 [Prunus persica]	-	M5XFY3|M5XFY3_PRUPE:Uncharacterized protein;OS=Prunus persica;
DN114808_c1_g1_i2	DOWN	[GO:0016021:integral component of membrane]	-	-	XP_010686515.1:PREDICTED: tetraspanin19like [Beta vulgaris subsp. vulgaris]	-	A0A0J8BSW9|A0A0J8BSW9_BETVU: Uncharacterized protein;OS=Beta vulgaris subsp. vulgaris.;|GO:0016021,
DN120707_c2_g5_i1	DOWN	-	-	-	-	-	-
DN127776_c4_g4_i2	DOWN	[GO:0008792:arginine decarboxylase activity][GO:0006527:arginine catabolic process][GO:0008295:spermidine biosynthetic process]	[ko00330:Arginine and proline metabolism]	-	XP_021753478.1:arginine decarboxylaselike [Chenopodium quinoa]	gi|502149390|ref|XM_004507452.1|:PREDICTED: Cicer arietinum arginine decarboxylase-like (LOC101497888),	A0A0K9RMY8|A0A0K9RMY8_SPIOL:Arginine decarboxylase;OS=Spinacia oleracea;|GO:00087

geneid	trend	GO	Pathway	TF	NR	NT	Uniprot
						mRNA	92,|GO:0006527,GO:0008295,
DN126736_c0_g7_i1	DOWN	[GO:0003983:UTP:glucose-1-phosphate uridylyltransferase activity][GO:0006011:UDP-glucose metabolic process]	[ko00040:Pentose and glucuronate interconversions][ko00052:Galactose metabolism][ko00500:Starch and sucrose metabolism][ko00520:Amino sugar and nucleotide sugar metabolism]	-	XP_021713619.1:UTPglucose1phosphate uridylyltransferase [Chenopodium quinoa]	gi|224135236|ref|XM_002327563.1|:Populus trichocarpa predicted protein, mRNA	A0A0J8BDJ4|A0A0J8BDJ4_BETVU:Uncharacterized protein;OS=Beta vulgaris subsp. vulgaris.;|GO:0003983,|GO:0006011,
DN119629_c1_g4_i1	DOWN	[GO:0016021:integral component of membrane][GO:0015171:amino acid transmembrane transporter activity]	-	-	XP_010278618.1:PREDICTED: aminoacid permease BAT1 homolog isoform X2 [Nelumbo nucifera]	gi|356574237|ref|XM_003555209.1|:PREDICTED: Glycine max uncharacterized protein LOC100794968 (LOC100794968), mRNA	A0A0K9RVR6|A0A0K9RVR6_SPIOL: Uncharacterized protein;OS=Spinacia oleracea;|GO:0016021,|GO:0015171,

geneid	trend	GO	Pathway	TF	NR	NT	Uniprot
DN125302_c5_g3_i1	DOWN	-	-	-	KMT06327.1:hypothetical protein BVRB_7g160090 [Beta vulgaris subsp. vulgaris]	-	A0A0J8BYH6|A0A0J8BYH6_BETVU: Uncharacterized protein;OS=Beta vulgaris subsp. vulgaris.;
DN114265_c0_g1_i1	DOWN	-	-	-	XP_010682519.1:PREDICTED: Fbox/kelchrepeat protein At1g23390 [Beta vulgaris subsp. vulgaris] >KMT07219.1 hypothetical protein BVRB_6g148540 [Beta vulgaris subsp. vulgaris]	-	A0A0J8C4R2|A0A0J8C4R2_BETVU:Uncharacterized protein;OS=Beta vulgaris subsp. vulgaris.;
DN126350_c2_g4_i1	DOWN	-	-	-	XP_021855325.1:protein RALFlike 33 [Spinacia oleracea] >KNA06236.1 hypothetical protein SOVF_182740 [Spinacia oleracea]	-	A0A0K9QI34|A0A0K9QI34_SPIOL:Uncharacterized protein;OS=Spinacia oleracea;

geneid	trend	GO	Pathway	TF	NR	NT	Uniprot
DN125267_c0_g6_i2	DOWN	[GO:0016491:oxidoreductase activity][GO:0008270:zinc ion binding]	[ko00940:Phenylpropanoid biosynthesis]	-	XP_021852629.1:probable cinnamyl alcohol dehydrogenase 1 [Spinacia oleracea] >KNA07730.1 hypothetical protein SOVF_168400 [Spinacia oleracea]	gi|332384180|gb|HQ880207.1|:Camellia sinensis cinnamyl alcohol dehydrogenase 1 mRNA, complete cds	A0A0K9QKE9|A0A0K9QKE9_SPIOL: Uncharacterized protein;OS=Spinacia oleracea;|GO:0016491,GO:0008270,
DN108284_c0_g1_i1	DOWN	[GO:0005618:cell wall][GO:0005773:vacuole][GO:0032366:intracellular sterol transport]	-	-	XP_010685463.1:PREDICTED: putative phosphatidylglycerol/phosphatidylinositol transfer protein DDB_G0282179 [Beta vulgaris subsp. vulgaris] >KMT04966.1 hypothetical protein BVRB_7g171300 [Beta vulgaris subsp. vulgaris]	-	A0A0J8EPQ2|A0A0J8EPQ2_BETVU:Uncharacterized protein;OS=Beta vulgaris subsp. vulgaris.;|GO:0005618,GO:0005773,|GO:0032366,

geneid	trend	GO	Pathway	TF	NR	NT	Uniprot
DN116018_c1_g2_i1	DOWN	[GO:0016021:integral component of membrane][GO:0005886:plasma membrane][GO:0016760:cellulose synthase (UDP-forming) activity][GO:0008270:zinc ion binding][GO:0071555:cell wall organization][GO:0030244:cellulose biosynthetic process]	-	CCG027008.3:Populus euphratica|G2-like|G2-like family protein	XP_021771565.1:cellulose synthase A catalytic subunit 6 [UDPforming]like [Chenopodium quinoa]	gi|470108389|ref|XM_004290455.1|:PREDICTED: Fragaria vesca subsp. vesca cellulose synthase A catalytic subunit 6 [UDP-forming]-like (LOC101315010), mRNA	A0A0J8D5V8|A0A0J8D5V8_BETVU:Cellulose synthase;OS=Beta vulgaris subsp. vulgaris.;|GO:0016021,GO:0005886,|GO:0016760,GO:0008270,|GO:0071555,GO:0030244,
DN120393_c4_g4_i4	DOWN	[GO:0016021:integral component of membrane][GO:0003993:acid phosphatase activity][GO:0046872:metal ion binding]	-	DCAR_003235:Daucus carota|GRAS|GRAS family protein	XP_021847487.1:probable inactive purple acid phosphatase 2 [Spinacia oleracea]	gi|521540299|gb|GAHZ01003126.1|:TSA: Salicornia europaea Unigene3347_SeCKS transcribed RNA sequence	A0A0K9RL69|A0A0K9RL69_SPIOL:Purple acid phosphatase;OS=Spinacia oleracea;|GO:0016021,|GO:0003993,GO:0046872,
DN115330_c1_g1_i12	DOWN	[GO:0004001:adenosine kinase activity][GO:0006166:purine ribonucleoside salvage]	[ko00230:Purine metabolism]	-	ABK93986.1:unknown [Populus trichocarpa]	-	A9PC83|A9PC83_POPTR:Putative uncharacterized protein;OS=Populus trichocarpa;|GO:0004001,|GO:0006166,

geneid	trend	GO	Pathway	TF	NR	NT	Uniprot
DN108391_c0_g2_i2	DOWN	[GO:0003677:DNA binding]	-	AHYPO_005621-RA:Amaranthus hypochondriacus|MYB|MYB family protein	XP_021749839.1:transcription factor MYB62 [Chenopodium quinoa]	gi|460387496|ref|XM_004239365.1|:PREDICTED: Solanum lycopersicum transcription factor MYB28-like (LOC101244584), partial mRNA	A0A0K9QG06|A0A0K9QG06_SPIOL:Uncharacterized protein;OS=Spinacia oleracea;|GO:0003677,
DN126097_c4_g2_i4	DOWN	[GO:0005622:intracellular][GO:0004435:phosphatidylinositol phospholipase C activity][GO:0004871:obsolete signal transducer activity][GO:0035556:intracellular signal transduction][GO:0016042:lipid catabolic process]	[ko00562:Inositol phosphate metabolism][ko04020:Calcium signaling pathway][ko04070:Phosphatidylinositol signaling system][ko04919:Thyroid hormone signaling pathway][ko04933: AGE-RAGE signaling pathway in diabetic complications]	RrC2946_p1:Raphanus raphanistrum|C3H|C3H family protein	XP_010689655.1:PREDICTED: phosphoinositide phospholipase C 2 [Beta vulgaris subsp. vulgaris] >KMT18203.1 hypothetical protein BVRB_2g023740 [Beta vulgaris subsp. vulgaris]	gi|194719374|gb|EU870371.1|:Spinacia oleracea phospholipase C (plc) mRNA, complete cds	A0A0J8D202|A0A0J8D202_BETVU:Phosphoinositide phospholipase C;OS=Beta vulgaris subsp. vulgaris.;|GO:0005622,|GO:0004435,GO:0004871,|GO:0035556,GO:0016042,

geneid	trend	GO	Pathway	TF	NR	NT	Uniprot
DN123963_c0_g4_i4	DOWN	[GO:0016021:integral component of membrane]	-	-	XP_010247407.1:PREDICTED: probable inositol transporter 2 [Nelumbo nucifera]	-	A0A067GJ06|A0A067GJ06_CITSI:Uncharacterized protein;OS=Citrus sinensis;|GO:0016021,|GO:0022891,

geneid	trend	GO	Pathway	TF	NR	NT	Uniprot
DN121980_c1_g1_i6	DOWN	[GO:0016021:integral component of membrane][GO:0004030:aldehyde dehydrogenase [NAD(P)+] activity][GO:0006081:cellular aldehyde metabolic process]	[ko00010:Glycolysis / Gluconeogenesis][ko000053:Ascorbate and aldarate metabolism][ko00071:Fatty acid degradation][ko00280:Valine, leucine and isoleucine degradation][ko00310:Lysine degradation][ko00330:Arginine and proline metabolism][ko00340:Histidine metabolism][ko00380:Tryptophan metabolism][ko00410:beta-Alanine metabolism][ko00561:Glycerolipid metabolism][ko00620:Pyruvate	-	XP_010673629.1:PREDICTED: aldehyde dehydrogenase family 3 member F1 [Beta vulgaris subsp. vulgaris] >KMT14482.1 hypothetical protein BVRB_4g072550 [Beta vulgaris subsp. vulgaris]	-	A0A0J8CLU1|A0A0J8CLU1_BETVU:Aldehyde dehydrogenase;OS=Beta vulgaris subsp. vulgaris.;|GO:0016021,|GO:0004030,|GO:0006081,

geneid	trend	GO	Pathway	TF	NR	NT	Uniprot
			metabolism][ko00625:Chloroalkane and chloroalkene degradation][ko00903:Limonene and pinene degradation][ko00981:Insect hormone biosynthesis]				

geneid	trend	GO	Pathway	TF	NR	NT	Uniprot
DN118354_c1_g1_i1	DOWN	[GO:0016021:integral component of membrane][GO:0033179:proton-transporting V-type ATPase, V0 domain][GO:0005774:vacuolar membrane][GO:0015078:proton transmembrane transporter activity][GO:0015991:ATP hydrolysis coupled proton transport]	[ko00190:Oxidative phosphorylation][ko04142:Lysosome][ko04145:Phagosome][ko04721:Synaptic vesicle cycle][ko04966:Collecting duct acid secretion][ko05110:Vibrio cholerae infection][ko05120:Epithelial cell signaling in Helicobacter pylori infection][ko05152:Tuberculosis][ko05323:Rheumatoid arthritis]	TRIUR3_01525-P1:Triticum urartu|C2H2|C2H2 family protein	AAP15165.1:vacuolar H(+)ATPase subunit c [Suaeda salsa] >AEP40376.1 vacuolar proton pump ATPase subunit C [Suaeda corniculata]	gi|1495682|emb|X94999.1|:M.crystallinum mRNA for V-type ATPase c subunit	P68162|VATL_BETVU:V-type proton ATPase 16 kDa proteolipid subunit;OS=Beta vulgaris;|GO:0016021,GO:0033179,GO:0005774,|GO:0015078,|GO:0015991,

geneid	trend	GO	Pathway	TF	NR	NT	Uniprot
DN128767_c7_g2_i1	DOWN	[GO:0005509:calcium ion binding]	[ko04014:Ras signaling pathway][ko04015:Rap1 signaling pathway][ko04016:MAPK signaling pathway - plant][ko04020:Calcium signaling pathway][ko04022:cGMP-PKG signaling pathway][ko04024:cAMP signaling pathway][ko04070:Phosphatidylinositol signaling system][ko04114:Oocyte meiosis][ko04218:Cellular senescence][ko04261:Adrenergic signaling in cardiomyocytes][ko04270:Vascular smooth muscle contraction][ko04371:Apelin signaling pathway][ko04626:Plant-pathogen interaction][ko04713	Zjn_sc00028.1.g03130.1.am.mk:Zoysia japonica|NAC|NAC family protein	XP_016577941.1:PREDICTED: calmodulin7like [Capsicum annuum]	gi|8131968|gb|AF150059.1|AF150059:Brassica napus calmodulin (CaM1) mRNA, complete cds	A0A0V0H2X4|A0A0V0H2X4_SOLCH: Putative ovule protein;OS=Solanum chacoense;|GO:0005509,

geneid	trend	GO	Pathway	TF	NR	NT	Uniprot
DN128171_c3_g2_i2	DOWN	-	-	Itr_sc000071.1_g00023.1:Ipomoea trifida|ERF|ERF family protein	XP_021860533.1:protein CELLULOSE SYNTHASE INTERACTIVE 3 [Spinacia oleracea] >XP_021860534.1 protein CELLULOSE SYNTHASE INTERACTIVE 3 [Spinacia oleracea] >XP_021860535.1 protein CELLULOSE SYNTHASE INTERACTIVE 3 [Spinacia oleracea] >XP_021860536.1 protein CELLULOSE SYNTHASE INTERACTIVE 3 [Spinacia oleracea]	gi|512398806|gb|GAJZ01002364.1|:TSA: Prunus avium Pa_04699 transcribed RNA sequence	A0A0K9R2G9|A0A0K9R2G9_SPIOL:Uncharacterized protein;OS=Spinacia oleracea;

geneid	trend	GO	Pathway	TF	NR	NT	Uniprot
DN120572_c0_g1_i1	DOWN	[GO:0009507:chloroplast][GO:0016021:integral component of membrane]	-	-	XP_021750651.1:chaperone protein dnaJ C76, chloroplasticlike [Chenopodium quinoa]	-	A0A0J8BRI7|A0A0J8BRI7_BETVU:Uncharacterized protein;OS=Beta vulgaris subsp. vulgaris.;|GO:0009507,GO:0016021,
DN126149_c3_g1_i4	DOWN	[GO:0005829:cytosol][GO:0016831:carboxy-lyase activity][GO:0000287:magnesium ion binding][GO:0030976:thiamine pyrophosphate binding]	[ko00010:Glycolysis / Gluconeogenesis]	439190:Selaginella moellendorffii|bHLH|bHLH family protein	AAP96920.1:pyruvate decarboxylase [Dianthus caryophyllus]	gi|33149680|gb|AY263388.1|:Dianthus caryophyllus pyruvate decarboxylase mRNA, complete cds	A0A0J8CFP7|A0A0J8CFP7_BETVU:Uncharacterized protein;OS=Beta vulgaris subsp. vulgaris.;|GO:0005829,|GO:0016831,GO:0000287,GO:0030976,
DN125381_c0_g1_i3	DOWN	[GO:0016021:integral component of membrane][GO:0005215:transporter activity]	-	mrna00620.1-v1.0-hybrid:Fragaria vesca|BBR-BPC|BBR-BPC family protein	XP_010678590.1:PREDICTED: protein NRT1/ PTR FAMILY 2.9 [Beta vulgaris subsp. vulgaris] >KMT10400.1 hypothetical protein BVRB_5g115280	gi|449532492|ref|XM_004173167.1|:PREDICTED: Cucumis sativus probable peptide/nitrate transporter At5g62680-like (LOC101217494), mRNA	A0A0J8C9J3|A0A0J8C9J3_BETVU:Uncharacterized protein;OS=Beta vulgaris subsp. vulgaris.;|GO:0016021,|GO:0005215,

geneid	trend	GO	Pathway	TF	NR	NT	Uniprot
					[Beta vulgaris subsp. vulgaris]		
DN118105_c1_g3_i3	DOWN	-	[ko00561:Glycerolipid metabolism][ko04723:Retrograde endocannabinoid signaling][ko04923:Regulation of lipolysis in adipocytes]	Lus10033562:Linum usitatissimum|bHLH|bHLH family protein	XP_021732710.1:caffeoylshikimate esteraselike [Chenopodium quinoa]	-	A0A0J8E853|A0A0J8E853_BETVU:Uncharacterized protein;OS=Beta vulgaris subsp. vulgaris.;
DN123963_c0_g4_i2	DOWN	[GO:0016021:integral component of membrane]	-	-	XP_010247407.1:PREDICTED: probable inositol transporter 2 [Nelumbo nucifera]	-	A0A067G715|A0A067G715_CITSI:Uncharacterized protein;OS=Citrus sinensis;|GO:0016021,|GO:0022891,

geneid	trend	GO	Pathway	TF	NR	NT	Uniprot
DN116871_c0_g2_i1	DOWN	[GO:0008171:O-methyltransferase activity]	[ko00940:Phenylpropanoid biosynthesis]	-	XP_021725690.1:caffeic acid 3Omethyltransferase like [Chenopodium quinoa]	gi|3176966|gb|AF067968.1|AF067968:Mesembryanthemum crystallinum caffeic acid 3-O-methyltransferase mRNA, partial cds	A0A0K9RAX5|A0A0K9RAX5_SPIOL: Uncharacterized protein;OS=Spinacia oleracea;|GO:0008171,
DN119284_c0_g1_i1	DOWN	[GO:0005737:cytoplasm][GO:0004801:sedoheptulose-7-phosphate:D-glyceraldehyde-3-phosphate glyceronetransferase activity][GO:0005975:carbohydrate metabolic process][GO:0006098:pentose-phosphate shunt]	[ko00030:Pentose phosphate pathway]	-	XP_021845985.1:uncharacterized protein LOC110785777 [Spinacia oleracea] >KNA23727.1 hypothetical protein SOVF_021530 [Spinacia oleracea]	gi|225425279|ref|XM_002271478.1|:PREDICTED: Vitis vinifera transaldolase-like, transcript variant 1 (LOC100247241), mRNA	A0A0K9RY10|A0A0K9RY10_SPIOL:Uncharacterized protein;OS=Spinacia oleracea;|GO:0005737,|GO:0004801,|GO:0005975,GO:0006098,

geneid	trend	GO	Pathway	TF	NR	NT	Uniprot
DN126805_c1_g4_i2	DOWN	[GO:0005777:peroxisome][GO:0050660:flavin adenine dinucleotide binding][GO:0004362:glutathione-disulfide reductase activity][GO:0050661:NADP binding][GO:0045454:cell redox homeostasis][GO:0006749:glutathione metabolic process]	[ko00480:Glutathione metabolism][ko04918:Thyroid hormone synthesis]	-	XP_007034406.2:PREDICTED: glutathione reductase, chloroplastic [Theobroma cacao] >XP_007034407.2 PREDICTED: glutathione reductase, chloroplastic [Theobroma cacao]	gi|10862870|emb|AJ400816.1|:Mesembryanthemum crystallinum mRNA for cytosolic glutathione reductase (gr1 gene)	A0A061ELT7|A0A061ELT7_THECC:Glutathione-disulfide reductase isoform 1;OS=Theobroma cacao;|GO:0005777,|GO:0050660,GO:0004362,GO:0050661,|GO:0045454,GO:0006749,
DN123331_c0_g2_i3	DOWN	[GO:0016021:integral component of membrane]	-	-	XP_010680717.1:PREDICTED: uncharacterized protein LOC104895802 [Beta vulgaris subsp. vulgaris] >KMT19538.1 hypothetical protein BVRB_1g011590 [Beta vulgaris subsp. vulgaris]	gi|521703058|gb|GAIA01016396.1|:TSA: Salicornia europaea Unigene17115_Se200S transcribed RNA sequence	A0A0J8D5T5|A0A0J8D5T5_BETVU:Uncharacterized protein;OS=Beta vulgaris subsp. vulgaris.;|GO:0016021,

geneid	trend	GO	Pathway	TF	NR	NT	Uniprot
DN124879_c2_g5_i3	DOWN	[GO:0005829:cytosol][GO:0005634:nucleus][GO:0046872:metal ion binding][GO:0004722:protein serine/threonine phosphatase activity][GO:0009738:abscisic acid-activated signaling pathway][GO:0009788:negative regulation of abscisic acid-activated signaling pathway][GO:0010360:negative regulation of anion channel activity][GO:0010119:regulation of stomatal movement]	[ko04016:MAPK signaling pathway - plant][ko04075:Plant hormone signal transduction][ko04931:Insulin resistance]	XP_010536267.1:Tarenaya hassleriana|NAC|NAC family protein	XP_010691227.1:PREDICTED: protein phosphatase 2C 37 [Beta vulgaris subsp. vulgaris] >KMT18389.1 hypothetical protein BVRB_2g025410 [Beta vulgaris subsp. vulgaris]	-	A0A0J8D286|A0A0J8D286_BETVU:Uncharacterized protein;OS=Beta vulgaris subsp. vulgaris.;|GO:0005829,GO:0005634,|GO:0046872,GO:0004722,|GO:0009738,GO:0009788,GO:0010360,GO:0010119,

geneid	trend	GO	Pathway	TF	NR	NT	Uniprot
DN119623_c0_g1_i2	DOWN	-	-	-	XP_010690132.1:PREDICTED: protein CHUP1, chloroplastic [Beta vulgaris subsp. vulgaris] >XP_019107453.1 PREDICTED: protein CHUP1, chloroplastic [Beta vulgaris subsp. vulgaris] >KMT01747.1 hypothetical protein BVRB_9g211970 [Beta vulgaris subsp. vulgaris]	-	A0A0J8BL16|A0A0J8BL16_BETVU:Uncharacterized protein;OS=Beta vulgaris subsp. vulgaris.;
DN125332_c1_g2_i2	DOWN	[GO:0016788:hydrolase activity, acting on ester bonds]	-	-	XP_021716583.1:LOW QUALITY PROTEIN: GDSL esterase/lipase 2like [Chenopodium quinoa]	-	Q7Y055|Q7Y055_OXYRB:GDSL-lipase;OS=Oxybasis rubra;|GO:0016788,

geneid	trend	GO	Pathway	TF	NR	NT	Uniprot
DN127819_c3_g2_i8	DOWN	[GO:0003978:UDP-glucose 4-epimerase activity][GO:0006012:galactose metabolic process]	[ko00052:Galactose metabolism][ko00520:Amino sugar and nucleotide sugar metabolism]	-	XP_021754129.1:bifunctional UDPglucose 4epimerase and UDPxylose 4epimerase 1like [Chenopodium quinoa]	-	O65780|GALE1_CYATE:UDP-glucose 4-epimerase GEPI42;OS=Cyamopsis tetragonoloba;|GO:0003978,|GO:0006012,
DN115114_c1_g2_i2	DOWN	-	-	-	XP_021772877.1:stressrelated proteinlike isoform X1 [Chenopodium quinoa]	-	A0A0J8BME0|A0A0J8BME0_BETVU: Uncharacterized protein;OS=Beta vulgaris subsp. vulgaris.;
DN113751_c0_g1_i2	DOWN	-	-	-	XP_021726695.1:protein SRC2like [Chenopodium quinoa]	gi|292728400|dbj|AK336610.1|:Lotus japonicus cDNA, clone: LjFL1-014-AB12, HTC	A0A0K9RXU7|A0A0K9RXU7_SPIOL: Uncharacterized protein;OS=Spinacia oleracea;

geneid	trend	GO	Pathway	TF	NR	NT	Uniprot
DN124086_c0_g1_i6	DOWN	-	-	-	XP_010679967.1:PREDICTED: type2 histone deacetylase 1 [Beta vulgaris subsp. vulgaris] >KMT09638.1 hypothetical protein BVRB_6g130900 [Beta vulgaris subsp. vulgaris]	-	A0A0J8CCE1|A0A0J8CCE1_BETVU:Uncharacterized protein;OS=Beta vulgaris subsp. vulgaris.;
DN125731_c4_g10_i1	DOWN	-	-	-	XP_010670216.1:PREDICTED: jasmonateinduced protein homolog [Beta vulgaris subsp. vulgaris]	-	A0A0J8CU80|A0A0J8CU80_BETVU:Uncharacterized protein;OS=Beta vulgaris subsp. vulgaris.;
DN116718_c1_g7_i1	DOWN	-	-	-	BAJ11784.1:dehydration responsive protein, partial [Corchorus olitorius]	gi|193084603|gb|EU795174.1|:Uncultured bacterium HF0010_04H24 genomic sequence	E1CJ65|E1CJ65_9ROSI:Dehydration responsive protein;OS=Corchorus olitorius.;

geneid	trend	GO	Pathway	TF	NR	NT	Uniprot
DN126553_c2_g1_i9	DOWN	-	-	-	XP_010684822.1:PREDICTED: uncharacterized protein LOC104899346 isoform X1 [Beta vulgaris subsp. vulgaris]	gi|521540013|gb|GAHZ01003269.1|:TSA: Salicornia europaea Unigene3501_SeCKS transcribed RNA sequence	A0A0J8BX88|A0A0J8BX88_BETVU:Uncharacterized protein;OS=Beta vulgaris subsp. vulgaris.;
DN124464_c2_g2_i4	DOWN	[GO:0005737:cytoplasm][GO:0005524:ATP binding][GO:0046872:metal ion binding][GO:0004478:methionine adenosyltransferase activity][GO:0006730:one-carbon metabolic process][GO:0006556:S-adenosylmethionine biosynthetic process]	[ko00270:Cysteine and methionine metabolism]	-	XP_021734528.1:Sadenosylmethionine synthase 4 [Chenopodium quinoa]	gi|71000460|dbj|AB221009.1|:Beta vulgaris BvSAMS1 mRNA for S-adenosyl-L-methionine synthetase, complete cds	Q6F3F1|METK4_ATRNU:S-adenosylmethionine synthase 4;OS=Atriplex nummularia;|GO:0005737,|GO:0005524,GO:0046872,GO:0004478,|GO:0006730,GO:0006556,
DN123815_c0_g1_i3	DOWN	[GO:0016021:integral component of membrane][GO:0009055:electron transfer activity]	-	-	XP_021772217.1:blue copper proteinlike [Chenopodium quinoa]	gi|521697344|gb|GAIA01018910.1|:TSA: Salicornia europaea Unigene19869_Se200S transcribed RNA sequence	A0A0J8FFN4|A0A0J8FFN4_BETVU:Uncharacterized protein;OS=Beta vulgaris subsp. vulgaris.;|GO:0016021,|GO:0009055,

geneid	trend	GO	Pathway	TF	NR	NT	Uniprot
DN126107_c1_g4_i3	DOWN	[GO:0016021:integral component of membrane][GO:0005524:ATP binding][GO:0042626:ATPase activity, coupled to transmembrane movement of substances]	[ko01523:Antifolate resistance][ko01524:Platinum drug resistance][ko02010:ABC transporters][ko04976:Bile secretion]	RrC2480_p5:Raphanus raphanistrum|HSF|HSF family protein	XP_021848908.1:ABC transporter C family member 14like [Spinacia oleracea] >XP_021848909.1 ABC transporter C family member 14like [Spinacia oleracea] >XP_021848910.1 ABC transporter C family member 14like [Spinacia oleracea] >KNA20533.1 hypothetical protein SOVF_051460 [Spinacia oleracea]	gi|255564131|ref|XM_002523017.1|:Ricinus communis multidrug resistance-associated protein 2, 6 (mrp2, 6), abc-transoprter, putative, mRNA	A0A0K9RM16|A0A0K9RM16_SPIOL:Uncharacterized protein;OS=Spinacia oleracea;|GO:0016021,|GO:0005524,GO:0042626,

geneid	trend	GO	Pathway	TF	NR	NT	Uniprot
DN120331_c0_g1_i1	DOWN	[GO:0016021:integral component of membrane][GO:0016747:transferase activity, transferring acyl groups other than amino-acyl groups][GO:0006633:fatty acid biosynthetic process]	[ko00062:Fatty acid elongation]	-	XP_021767205.1:3ketoacylCoA synthase 1like [Chenopodium quinoa]	gi|470101839|ref|XM_004287328.1|:PREDICTED: Fragaria vesca subsp. vesca 3-ketoacyl-CoA synthase 11-like (LOC101312957), mRNA	A0A0K9QLI5|A0A0K9QLI5_SPIOL:3-ketoacyl-CoA synthase;OS=Spinacia oleracea;|GO:0016021,|GO:0016747,|GO:0006633,

geneid	trend	GO	Pathway	TF	NR	NT	Uniprot
DN123388_c2_g4_i10	DOWN	[GO:0016620:oxidoreductase activity, acting on the aldehyde or oxo group of donors, NAD or NADP as acceptor]	[ko00010:Glycolysis / Gluconeogenesis][ko000053:Ascorbate and aldarate metabolism][ko00071:Fatty acid degradation][ko00280:Valine, leucine and isoleucine degradation][ko00310:Lysine degradation][ko00330:Arginine and proline metabolism][ko00340:Histidine metabolism][ko00380:Tryptophan metabolism][ko00410:beta-Alanine metabolism][ko00561:Glycerolipid metabolism][ko00620:Pyruvate	Lus10030143:Linum usitatissimum|NAC|NAC family protein	XP_010682575.1:PREDICTED: aldehyde dehydrogenase family 2 member B7, mitochondrial isoform X1 [Beta vulgaris subsp. vulgaris] >KMT07274.1 hypothetical protein BVRB_6g149040 [Beta vulgaris subsp. vulgaris]	gi|521748252|gb|GAIA01001736.1|:TSA: Salicornia europaea Unigene1772_Se200S transcribed RNA sequence	A0A0J8C4W5|A0A0J8C4W5_BETVU: Uncharacterized protein;OS=Beta vulgaris subsp. vulgaris.;|GO:0016620,

geneid	trend	GO	Pathway	TF	NR	NT	Uniprot
			metabolism][ko00625:Chloroalkane and chloroalkene degradation][ko00903:Limonene and pinene degradation][ko00981:Insect hormone biosynthesis]				

geneid	trend	GO	Pathway	TF	NR	NT	Uniprot
DN122337_c3_g3_i3	DOWN	[GO:0016021:integral component of membrane][GO:0005506:iron ion binding][GO:0016491:oxidoreductase activity][GO:0006633:fatty acid biosynthetic process]	[ko00600:Sphingolipid metabolism]	-	XP_021867593.1:sphinganine C4monooxygenase 1like [Spinacia oleracea] >KNA14406.1 hypothetical protein SOVF_107720 [Spinacia oleracea]	gi|210141990|dbj|AK245909.1|:Glycine max cDNA, clone: GMFL01-45-O13	A0A0K9R4H8|A0A0K9R4H8_SPIOL:Uncharacterized protein;OS=Spinacia oleracea;|GO:0016021,|GO:0005506,GO:0016491,|GO:0006633,
DN126556_c0_g1_i8	DOWN	[GO:0016021:integral component of membrane][GO:0005524:ATP binding][GO:0000287:magnesium ion binding][GO:0004012:phospholipid-translocating ATPase activity]	-	-	XP_021842734.1:probable phospholipidtransporting ATPase 8 [Spinacia oleracea]	gi|225452443|ref|XM_002277653.1|:PREDICTED: Vitis vinifera putative phospholipid-transporting ATPase 8-like, transcript variant 1 (LOC100259356), mRNA	A0A0J8B3X2|A0A0J8B3X2_BETVU:Phospholipid-transporting ATPase;OS=Beta vulgaris subsp. vulgaris.;|GO:0016021,|GO:0005524,GO:0000287,GO:0004012,
DN128371_c3_g1_i2	DOWN	-	-	-	KMT19001.1:hypothetical protein BVRB_2g030970 [Beta vulgaris subsp. vulgaris]	-	A0A0J8FTI9|A0A0J8FTI9_BETVU:Uncharacterized protein;OS=Beta vulgaris subsp. vulgaris.;

geneid	trend	GO	Pathway	TF	NR	NT	Uniprot
DN125867_c2_g5_i7	DOWN	-	-	-	XP_021769068.1:uncharacterized protein LOC110733343 [Chenopodium quinoa]	-	A0A0J8EB67|A0A0J8EB67_BETVU:Uncharacterized protein;OS=Beta vulgaris subsp. vulgaris.;
DN112858_c0_g1_i1	DOWN	[GO:0046872:metal ion binding][GO:0030001:metal ion transport]	-	-	XP_021729477.1:heavy metalassociated isoprenylated plant protein 24like [Chenopodium quinoa]	-	A0A0J8BEB9|A0A0J8BEB9_BETVU:Uncharacterized protein;OS=Beta vulgaris subsp. vulgaris.;|GO:0046872,|GO:0030001,
DN126103_c2_g3_i1	DOWN	[GO:0016021:integral component of membrane][GO:0015297:antiporter activity]	-	-	XP_021728984.1:protein DETOXIFICATION 42like [Chenopodium quinoa]	gi|521444343|gb|GAHZ01076141.1|:TSA: Salicornia europaea Unigene93078_SeCKS transcribed RNA sequence	A0A0J8CLU8|A0A0J8CLU8_BETVU:Protein DETOXIFICATION;OS=Beta vulgaris subsp. vulgaris.;|GO:0016021,|GO:0015297,GO:0015238,

geneid	trend	GO	Pathway	TF	NR	NT	Uniprot
DN121215_c1_g1_i2	DOWN	[GO:0016021:integral component of membrane][GO:0055085:transmembrane transport]	-	-	XP_010666769.1:PREDICTED: peroxisomal adenine nucleotide carrier 1 [Beta vulgaris subsp. vulgaris] >KMS95855.1 hypothetical protein BVRB_004340 [Beta vulgaris subsp. vulgaris]	gi|521697863|gb|GAIA01018538.1|:TSA: Salicornia europaea Unigene19493_Se200S transcribed RNA sequence	A0A0J8DYA3|A0A0J8DYA3_BETVU: Uncharacterized protein;OS=Beta vulgaris subsp. vulgaris.;|GO:0016021,|GO:0055085,
DN122917_c0_g2_i3	DOWN	[GO:0016021:integral component of membrane][GO:0005215:transporter activity]	-	mrna31023.1-v1.0-hybrid:Fragaria vesca|C3H|C3H family protein	XP_021754114.1:protein NRT1/ PTR FAMILY 3.1like [Chenopodium quinoa]	gi|502168943|ref|XM_004514443.1|:PREDICTED: Cicer arietinum probable nitrite transporter At1g68570-like (LOC101498251), mRNA	A0A0K9QAH3|A0A0K9QAH3_SPIOL: Uncharacterized protein;OS=Spinacia oleracea;|GO:0016021,|GO:0005215,
DN127654_c3_g4_i6	DOWN	[GO:0016021:integral component of membrane]	-	mrna12442.1-v1.0-hybrid:Fragaria vesca|NAC|NAC family protein	XP_019103735.1:PREDICTED: uncharacterized protein LOC104887465 [Beta vulgaris subsp.	gi|359482029|ref|XM_002275830.2|:PREDICTED: Vitis vinifera IAA-amino acid hydrolase ILR1-like	A0A0J8CYE6|A0A0J8CYE6_BETVU:Uncharacterized protein;OS=Beta vulgaris subsp. vulgaris.;|GO:00160

geneid	trend	GO	Pathway	TF	NR	NT	Uniprot
					vulgaris]	(LOC100266557), mRNA	21,
DN125413_c5_g2_i7	DOWN	[GO:0016021:integral component of membrane][GO:0005622:intracellular][GO:0005215:transporter activity]	-	mrna15187.1-v1.0-hybrid:Fragaria vesca|bHLH|bHLH family protein	XP_021738651.1:phosphatidylinositol/phosphatidylcholine transfer protein SFH6like [Chenopodium quinoa]	gi|521703591|gb|GAIA01016129.1|:TSA: Salicornia europaea Unigene16837_Se200S transcribed RNA sequence	A0A0K9QSX2|A0A0K9QSX2_SPIOL: Uncharacterized protein;OS=Spinacia oleracea;|GO:0016021,GO:0005622,|GO:0005215,
DN123757_c1_g3_i18	DOWN	[GO:0004385:guanylate kinase activity]	[ko00230:Purine metabolism]	-	XP_010672782.1:PREDICTED: guanylate kinase 1 [Beta vulgaris subsp. vulgaris] >KMT15433.1 hypothetical protein BVRB_3g061370 [Beta vulgaris subsp. vulgaris]	gi|359481712|ref|XM_003632614.1|:PREDICTED: Vitis vinifera discs large homolog 1-like protein-like, transcript variant 2 (LOC100241746), mRNA	A0A0J8CTY5|A0A0J8CTY5_BETVU:Uncharacterized protein;OS=Beta vulgaris subsp. vulgaris.;|GO:0004385,

geneid	trend	GO	Pathway	TF	NR	NT	Uniprot
DN117171_c 0_g1_i1	DOWN	-	-	-	XP_021757699.1:B2 proteinlike [Chenopodium quinoa]	-	A0A0D2QX10|A0A 0D2QX10_GOSRA: Uncharacterized protein;OS=Gossypi um raimondii;
DN121001_c 0_g2_i3	DOWN	[GO:0005509:calcium ion binding]	-	-	XP_021765586.1:T LD domaincontaining protein 1like [Chenopodium quinoa]	gi|334187143|ref|N M_119568.3|:Arabid opsis thaliana Calcium-binding EF-hand family protein mRNA, complete cds	A0A0K9RLE9|A0A 0K9RLE9_SPIOL:U ncharacterized protein;OS=Spinacia oleracea;|GO:00055 09,
DN120092_c 1_g1_i12	DOWN	[GO:0016787:hydrolase activity][GO:0008152:metabolic process]	-	-	XP_021856941.1:un characterized protein LOC110796216 [Spinacia oleracea] >KNA20418.1 hypothetical protein SOVF_052570 [Spinacia oleracea]	gi|388496765|gb|BT 136654.1|:Medicago truncatula clone JCVI-FLMt-17L5 unknown mRNA	A0A0K9RLP1|A0A 0K9RLP1_SPIOL:U ncharacterized protein;OS=Spinacia oleracea;|GO:00167 87,|GO:0008152,
DN123963_c 0_g4_i3	DOWN	[GO:0016021:integral component of membrane]	-	-	CAN62202.1:hypoth etical protein VITISV_002203 [Vitis vinifera]	-	A5C973|A5C973_V ITVI:Putative uncharacterized protein;OS=Vitis vinifera;|GO:001602

geneid	trend	GO	Pathway	TF	NR	NT	Uniprot
							1,|GO:0022891,
DN114565_c0_g4_i2	DOWN	[GO:0005524:ATP binding]	[ko03040:Spliceosome][ko04010:MAPK signaling pathway][ko04141:Protein processing in endoplasmic reticulum][ko04144:Endocytosis][ko04213:Longevity regulating pathway - multiple species][ko04612:Antigen processing and presentation][ko04915:Estrogen signaling pathway][ko05134:Legionellosis][ko05145:Toxoplasmosis][k	FANhyb_rscf00000233.1.g00002.1:Fragaria x ananassa|GATA|GATA family protein	XP_021295264.1:heat shock cognate 70 kDa protein 2 isoform X1 [Herrania umbratica] >XP_021295265.1 heat shock cognate 70 kDa protein 2 isoform X2 [Herrania umbratica]	gi|2660771|gb|AF034618.1|AF034618:Spinacia oleracea cytosolic heat shock 70 protein (HSC70-1) gene, complete cds	A0A059A986|A0A059A986_EUCGR:Uncharacterized protein;OS=Eucalyptus grandis;|GO:0005524,

geneid	trend	GO	Pathway	TF	NR	NT	Uniprot
			o05162:Measles][ko05164:Influenza A][ko05169:Epstein-Barr virus infection]				

geneid	trend	GO	Pathway	TF	NR	NT	Uniprot
DN129178_c11_g1_i1	DOWN	[GO:0046872:metal ion binding][GO:0030001:metal ion transport]	-	-	XP_021722721.1:heavy metalassociated isoprenylated plant protein 6like isoform X1 [Chenopodium quinoa] >XP_021747648.1 heavy metalassociated isoprenylated plant protein 6like isoform X1 [Chenopodium quinoa]	-	A0A0K9QJY0|A0A0K9QJY0_SPIOL:Uncharacterized protein;OS=Spinacia oleracea;|GO:0046872,|GO:0030001,
DN119993_c0_g3_i1	DOWN	[GO:0005739:mitochondrion]	-	-	AGC78945.1:hypothetical protein (mitochondrion) [Vicia faba]	gi|411019496|gb|JQ372257.2|:Uncultured bacterium clone WIR21Fasta1_8536 16S ribosomal RNA gene, partial sequence	R4ITY7|R4ITY7_VICFA:Uncharacterized protein;OS=Vicia faba;|GO:0005739,

geneid	trend	GO	Pathway	TF	NR	NT	Uniprot
DN128648_c0_g4_i1	DOWN	[GO:0005737:cytoplasm][GO:0005634:nucleus][GO:0016442:RISC complex][GO:0004518:nuclease activity][GO:0003712:transcription coregulator activity][GO:0035194:posttranscriptional gene silencing by RNA][GO:0006401:RNA catabolic process]	[ko05169:Epstein-Barr virus infection][ko05203:Viral carcinogenesis]	-	XP_010250439.1:PREDICTED: staphylococcal nuclease domaincontaining protein 1 [Nelumbo nucifera]	gi|225451676|ref|XM_002278181.1|:PREDICTED: Vitis vinifera staphylococcal nuclease domain-containing protein 1-like (LOC100256648), mRNA	D7SVE1|D7SVE1_VITVI:Putative uncharacterized protein;OS=Vitis vinifera;GN=VIT_14s0068g00720 {ECO:0000313|EMBL:CBI21240.3};|GO:0005737,GO:0005634,GO:0016442,|GO:0004518,GO:0003712,|GO:0035194,GO:0006401,
DN116519_c1_g1_i3	DOWN	[GO:0008756:o-succinylbenzoate-CoA ligase activity][GO:0008152:metabolic process]	-	Zmw_sc02382.1.g00020.1:Zoysia matrella|bHLH|bHLH family protein	XP_002510783.1:PREDICTED: oxalateCoA ligase [Ricinus communis] >EEF51385.1 AMP dependent CoA ligase, putative [Ricinus communis]	gi|224922994|gb|AC235230.1|:Glycine max strain Williams 82 clone GM_WBb0026I18, complete sequence	B9R9A8|B9R9A8_RICCO:AMP dependent CoA ligase, putative;OS=Ricinus communis;|GO:0008756,|GO:0008152,

geneid	trend	GO	Pathway	TF	NR	NT	Uniprot
DN124971_c3_g4_i23	DOWN	[GO:0046872:metal ion binding][GO:0016491:oxidoreductase activity]	-	676709864:Sisymbrium irio|STAT|STAT family protein	XP_010665637.1:PREDICTED: protein SRG1like [Beta vulgaris subsp. vulgaris]	gi|255575140|ref|XM_002528429.1|:Ricinus communis Leucoanthocyanidin dioxygenase, putative, mRNA	A0A0J8B6P3|A0A0J8B6P3_BETVU:Uncharacterized protein;OS=Beta vulgaris subsp. vulgaris.;|GO:0046872,GO:0016491,
DN119379_c1_g4_i1	DOWN	-	-	Csa09g092790.1:Camelina sativa|BES1|BES1 family protein	XP_010686317.1:PREDICTED: protein trichome birefringencelike 37 [Beta vulgaris subsp. vulgaris]	gi|512395617|gb|GAJZ01005553.1|:TSA: Prunus avium Pa_14890 transcribed RNA sequence	A0A0K9RYP0|A0A0K9RYP0_SPIOL:Uncharacterized protein;OS=Spinacia oleracea;
DN53124_c0_g3_i1	DOWN	-	-	-	XP_021735769.1:thaumatinlike protein 1 [Chenopodium quinoa]	-	A0A199VDG3|A0A199VDG3_ANACO:Protein P21;OS=Ananas comosus;
DN119922_c1_g6_i2	DOWN	[GO:0005737:cytoplasm][GO:0005525:GTP binding][GO:0003924:GTPase activity][GO:0003746:translation elongation factor activity]	-	-	XP_020239395.1:elongation factor 1alpha [Cajanus cajan] >KYP42506.1 Elongation factor 1alpha [Cajanus	gi|306416020|gb|GQ848072.1|:Oryza sativa Japonica Group clone KCG182G02 elongation factor-1 alpha mRNA,	A0A151RIW9|A0A151RIW9_CAJCA: Elongation factor 1-alpha;OS=Cajanus cajan;|GO:0005737,|GO:0005525,GO:0003924,GO:0003746,

geneid	trend	GO	Pathway	TF	NR	NT	Uniprot
					cajan]	complete cds	
DN127292_c 2_g1_i9	DOWN	[GO:0016021:integral component of membrane][GO:0005524:ATP binding][GO:0008553:proton-exporting ATPase activity, phosphorylative mechanism][GO:0046872:metal ion binding][GO:0006754:ATP biosynthetic process]	[ko00190:Oxidative phosphorylation]	Lus10040166:Linum usitatissimum|GRAS |GRAS family protein	XP_021761683.1:ATPase 11, plasma membranetypelike [Chenopodium quinoa]	gi|521637751|gb|GAIA01047797.1|:TSA: Salicornia europaea Unigene56013_Se200S transcribed RNA sequence	A0A0K9RUS9|A0A0K9RUS9_SPIOL:Plasma membrane ATPase;OS=Spinacia oleracea;|GO:0016021,|GO:0005524,GO:0008553,GO:0046872,|GO:0006754,

geneid	trend	GO	Pathway	TF	NR	NT	Uniprot
DN125997_c3_g1_i4	DOWN	-	-	-	XP_021843979.1:uncharacterized protein LOC110783904 [Spinacia oleracea] >XP_021843980.1 uncharacterized protein LOC110783904 [Spinacia oleracea] >XP_021843981.1 uncharacterized protein LOC110783904 [Spinacia oleracea] >KNA22996.1 hypothetical protein SOVF_028860 [Spinacia oleracea]	gi|512395740|gb|GAJZ01005430.1|:TSA: Prunus avium Pa_14624 transcribed RNA sequence	A0A0K9RTX4|A0A0K9RTX4_SPIOL: Uncharacterized protein;OS=Spinacia oleracea;

geneid	trend	GO	Pathway	TF	NR	NT	Uniprot
DN111147_c1_g1_i1	DOWN	[GO:0004081:bis(5'-nucleosyl)-tetraphosphatase (asymmetrical) activity][GO:0008893:guanosine-3',5'-bis(diphosphate) 3'-diphosphatase activity][GO:0015967:diadenosine tetraphosphate catabolic process]	-	-	XP_021746945.1:nudix hydrolase 25like [Chenopodium quinoa]	gi|521529861|gb|GAHZ01008590.1|:TSA: Salicornia europaea Unigene8959_SeCKS transcribed RNA sequence	A0A061E9C3|A0A061E9C3_THECC:Nudix hydrolase;OS=Theobroma cacao;|GO:0004081,GO:0008893,|GO:0015967,
DN127925_c2_g1_i9	DOWN	[GO:0016021:integral component of membrane][GO:0005524:ATP binding][GO:0005388:calcium-transporting ATPase activity][GO:0046872:metal ion binding]	-	Lus10040166:Linum usitatissimum|GRAS|GRAS family protein	XP_010687432.1:PREDICTED: calciumtransporting ATPase 10, plasma membranetype isoform X1 [Beta vulgaris subsp. vulgaris]	gi|224097461|ref|XM_002310908.1|:Populus trichocarpa autoinhibited calcium ATPase, mRNA	A0A0J8EL09|A0A0J8EL09_BETVU:Calcium-transporting ATPase;OS=Beta vulgaris subsp. vulgaris.;|GO:0016021,|GO:0005524,GO:0005388,GO:0046872,

geneid	trend	GO	Pathway	TF	NR	NT	Uniprot
DN127571_c3_g2_i5	DOWN	[GO:0016301:kinase activity][GO:0000287:magnesium ion binding][GO:0030955:potassium ion binding][GO:0004743:pyruvate kinase activity]	[ko00010:Glycolysis / Gluconeogenesis][ko00230:Purine metabolism][ko00620:Pyruvate metabolism][ko04922:Glucagon signaling pathway][ko04930:Type II diabetes mellitus][ko05203:Viral carcinogenesis][ko05230:Central carbon metabolism in cancer]	-	XP_021839938.1:pyruvate kinase isozyme G, chloroplastic [Spinacia oleracea] >KNA14594.1 hypothetical protein SOVF_105980 [Spinacia oleracea]	gi|224118131|ref|XM_002317703.1|:Populus trichocarpa predicted protein, mRNA	A0A0K9R6L1|A0A0K9R6L1_SPIOL:Pyruvate kinase;OS=Spinacia oleracea;|GO:0016301,GO:0000287,GO:0030955,GO:0004743,
DN118572_c4_g2_i2	DOWN	[GO:0016491:oxidoreductase activity]	-	EcC042108.10:Eucalyptus camaldulensis|C2H2|C2H2 family protein	XP_021842806.1:nonfunctional NADPHdependent codeinone reductase 2like [Spinacia oleracea]	gi|224087093|ref|XM_002308027.1|:Populus trichocarpa predicted protein, mRNA	A0A0K9RZ86|A0A0K9RZ86_SPIOL:Uncharacterized protein;OS=Spinacia oleracea;|GO:0016491,

geneid	trend	GO	Pathway	TF	NR	NT	Uniprot
DN124018_c1_g3_i11	DOWN	-	-	Achn202071:Actinidia chinensis|MYB_related|MYB_related family protein	XP_021733635.1:protein KINESIN LIGHT CHAINRELATED 3like [Chenopodium quinoa]	gi|521570717|gb|GAIA01073328.1|:TSA: Salicornia europaea Unigene89146_Se200S transcribed RNA sequence	A0A0J8CEL7|A0A0J8CEL7_BETVU:Uncharacterized protein;OS=Beta vulgaris subsp. vulgaris.;
DN124386_c0_g1_i2	DOWN	[GO:0003676:nucleic acid binding][GO:0000166:nucleotide binding]	[ko03040:Spliceosome]	Achn013161:Actinidia chinensis|bHLH|bHLH family protein	XP_021848278.1:heterogeneous nuclear ribonucleoprotein 1like [Spinacia oleracea] >KNA25903.1 hypothetical protein SOVF_002430 [Spinacia oleracea]	gi|521484148|gb|GAHZ01046738.1|:TSA: Salicornia europaea Unigene55089_SeCKS transcribed RNA sequence	A0A0K9S2H0|A0A0K9S2H0_SPIOL:Uncharacterized protein;OS=Spinacia oleracea;|GO:0003676,GO:0000166,
DN119699_c3_g3_i2	DOWN	-	[ko04144:Endocytosis]	-	XP_021854045.1:clathrin interactor EPSIN 2 [Spinacia oleracea] >XP_021854046.1 clathrin interactor EPSIN 2 [Spinacia oleracea] >XP_021854047.1 clathrin interactor	gi|521567584|gb|GAIA01074557.1|:TSA: Salicornia europaea Unigene90482_Se200S transcribed RNA sequence	A0A0K9QVT9|A0A0K9QVT9_SPIOL: Uncharacterized protein;OS=Spinacia oleracea;

geneid	trend	GO	Pathway	TF	NR	NT	Uniprot
					EPSIN 2 [Spinacia oleracea]		
DN121643_c3_g3_i10	DOWN	[GO:0005524:ATP binding][GO:0005509:calcium ion binding][GO:0004672:protein kinase activity]	-	Zjn_sc00028.1.g03130.1.am.mk:Zoysia japonica|NAC|NAC family protein	XP_010673049.1:PREDICTED: CDPKrelated kinase 7 [Beta vulgaris subsp. vulgaris] >KMT15203.1 hypothetical protein BVRB_3g063140 [Beta vulgaris subsp. vulgaris]	gi|521598083|gb|GAIA01065162.1|:TSA: Salicornia europaea Unigene78208_Se200S transcribed RNA sequence	A0A0J8CTC0|A0A0J8CTC0_BETVU:Uncharacterized protein;OS=Beta vulgaris subsp. vulgaris.;|GO:0005524,GO:0005509,GO:0004672,

geneid	trend	GO	Pathway	TF	NR	NT	Uniprot
DN120357_c0_g2_i2	DOWN	[GO:0005524:ATP binding][GO:0004674:protein serine/threonine kinase activity]	-	mrna27940.1-v1.0-hybrid:Fragaria vesca|NAC|NAC family protein	XP_010683520.1:PREDICTED: serine/threonineprotein kinase HT1 [Beta vulgaris subsp. vulgaris] >KMT06558.1 hypothetical protein BVRB_7g157300 [Beta vulgaris subsp. vulgaris]	gi|224139345|ref|XM_002323031.1|:Populus trichocarpa predicted protein, mRNA	A0A0J8C3A9|A0A0J8C3A9_BETVU:Uncharacterized protein;OS=Beta vulgaris subsp. vulgaris.;|GO:0005524,GO:0004674,
DN119786_c0_g13_i2	DOWN	-	-	-	-	-	-
DN119670_c0_g1_i3	DOWN	[GO:0006950:response to stress]	-	-	XP_021848443.1:calmodulinbinding protein 60 A [Spinacia oleracea] >KNA08468.1 hypothetical protein SOVF_162320 [Spinacia oleracea]	gi|224133439|ref|XM_002321532.1|:Populus trichocarpa predicted protein, mRNA	A0A0K9QP63|A0A0K9QP63_SPIOL:Uncharacterized protein;OS=Spinacia oleracea;|GO:0006950,

geneid	trend	GO	Pathway	TF	NR	NT	Uniprot
DN126583_c2_g3_i10	DOWN	-	-	-	XP_021864624.1:B2 proteinlike [Spinacia oleracea] >KNA24030.1 hypothetical protein SOVF_019830 [Spinacia oleracea]	gi|224053762|ref|XM_002297931.1|:Populus trichocarpa predicted protein, mRNA	A0A0K9RWX0|A0A0K9RWX0_SPIOL:Uncharacterized protein;OS=Spinacia oleracea;
DN124467_c1_g1_i5	DOWN	[GO:0008270:zinc ion binding]	-	Bostr.7128s0454.1.p:Boechera stricta|bHLH|bHLH family protein	XP_021743658.1:pentatricopeptide repeatcontaining protein At4g15720like [Chenopodium quinoa]	gi|225432321|ref|XM_002276194.1|:PREDICTED: Vitis vinifera pentatricopeptide repeat-containing protein At4g15720-like (LOC100249494), mRNA	A0A0J8D5U8|A0A0J8D5U8_BETVU:Uncharacterized protein;OS=Beta vulgaris subsp. vulgaris.;|GO:0008270,
DN125811_c7_g13_i2	DOWN	[GO:0008168:methyltransferase activity][GO:0006480:N-terminal protein amino acid methylation]	-	-	OAY63434.1:Alpha Nterminal protein methyltransferase 1 [Ananas comosus]	-	A0A199UFB4|A0A199UFB4_ANACO:Alpha N-terminal protein methyltransferase 1;OS=Ananas comosus;|GO:0008168,|GO:0006480,

geneid	trend	GO	Pathway	TF	NR	NT	Uniprot
DN126350_c2_g5_i2	DOWN	-	-	-	-	-	-
DN124464_c2_g2_i8	DOWN	[GO:0005737:cytoplasm][GO:0005524:ATP binding][GO:0046872:metal ion binding][GO:0004478:methionine adenosyltransferase activity][GO:0006730:one-carbon metabolic process][GO:0006556:S-adenosylmethionine biosynthetic process]	[ko00270:Cysteine and methionine metabolism]	-	Q7XZR1.2:RecName: Full=Sadenosylmethionine synthase 1; Short=AdoMet synthase 1; AltName: Full=Methionine adenosyltransferase 1; Short=MAT 1 >BAC77697.2 SadenosylLmethionine synthase [Atriplex nummularia] >BAD29707.1 SadenosylLmethionine synthase 1 [Atriplex nummularia]	gi|1724103|gb|U79767.1|MCU79767:Mesembryanthemum crystallinum methionine adenosyltransferase mRNA, complete cds	Q7XZR1|METK1_ATRNU:S-adenosylmethionine synthase 1;OS=Atriplex nummularia;|GO:0005737,|GO:0005524,GO:0046872,GO:0004478,|GO:0006730,GO:0006556,

geneid	trend	GO	Pathway	TF	NR	NT	Uniprot
DN123380_c6_g1_i1	DOWN	-	-	-	XP_010679062.1:PREDICTED: late embryogenesis abundant protein 31 [Beta vulgaris subsp. vulgaris] >KMT10181.1 hypothetical protein BVRB_5g119420 [Beta vulgaris subsp. vulgaris]	-	A0A0J8F381|A0A0J8F381_BETVU:Uncharacterized protein;OS=Beta vulgaris subsp. vulgaris.;
DN125450_c2_g3_i3	DOWN	[GO:0000785:chromatin][GO:0005829:cytosol][GO:0005634:nucleus][GO:0003682:chromatin binding][GO:0009881:photoreceptor activity][GO:0009649:entrainment of circadian clock][GO:0010224:response to UV-B]	-	XP_010106869.1:Morus notabilis|HB-other|HB-other family protein	XP_010695065.1:PREDICTED: ultravioletB receptor UVR8 [Beta vulgaris subsp. vulgaris] >KMS97937.1 hypothetical protein BVRB_4g097290 [Beta vulgaris subsp. vulgaris]	gi|521540127|gb|GAHZ01003212.1|:TSA: Salicornia europaea Unigene3439_SeCKS transcribed RNA sequence	A0A0J8BD98|A0A0J8BD98_BETVU:Uncharacterized protein;OS=Beta vulgaris subsp. vulgaris.;|GO:0000785,GO:0005829,GO:0005634,|GO:0003682,GO:0009881,|GO:0009649,GO:0010224,

geneid	trend	GO	Pathway	TF	NR	NT	Uniprot
DN120910_c3_g1_i1	DOWN	[GO:0005829:cytosol][GO:0004332:fructose-bisphosphate aldolase activity][GO:0043621:protein self-association][GO:0030388:fructose 1,6-bisphosphate metabolic process][GO:0006096:glycolytic process]	[ko00010:Glycolysis / Gluconeogenesis][ko00030:Pentose phosphate pathway][ko00051:Fructose and mannose metabolism][ko00680:Methane metabolism][ko00710:Carbon fixation in photosynthetic organisms]	Csa06g039080.1:Camelina sativa|ERF|ERF family protein	P29356.1:RecName: Full=Fructosebisphosphate aldolase, cytoplasmic isozyme >CAA46649.1 fructosebisphosphate aldolase [Spinacia oleracea]	gi|514785953|ref|XM_004970909.1|:PREDICTED: Setaria italica fructose-bisphosphate aldolase cytoplasmic isozyme-like (LOC101756517), mRNA	P29356|ALF_SPIOL:Fructose-bisphosphate aldolase, cytoplasmic isozyme;OS=Spinacia oleracea;|GO:0005829,GO:0043234,|GO:0004332,GO:0043621,|GO:0030388,GO:0006096,
DN123530_c4_g3_i5	DOWN	[GO:0005634:nucleus][GO:0043565:sequence-specific DNA binding][GO:0003700:DNA-binding transcription factor activity]	-	SapurV1A.0003s0740.1.p:Salix purpurea|HD-ZIP|HD-ZIP family protein	ACZ05048.1:putative homeobox protein 12 [Phytolacca acinosa]	-	D5FV17|D5FV17_PHYAN:Putative homeobox protein 12;OS=Phytolacca acinosa;|GO:0005634,|GO:0043565,GO:0003700,
DN127105_c8_g1_i1	DOWN	[GO:0004185:serine-type carboxypeptidase activity]	-	KZV23174.1:Dorcoceras hygrometricum|C3H|C3H family protein	XP_018805989.1:PREDICTED: serine carboxypeptidaselike 45 [Juglans regia]	gi|521686287|gb|GAIA01024347.1|:TSA: Salicornia europaea Unigene26046_Se200S transcribed RNA	A0A0J8CT19|A0A0J8CT19_BETVU:Carboxypeptidase;OS=Beta vulgaris subsp. vulgaris.;|GO:00041

geneid	trend	GO	Pathway	TF	NR	NT	Uniprot
						sequence	85,
DN123245_c2_g1_i1	DOWN	[GO:0020037:heme binding][GO:0030151:molybdenum ion binding][GO:0043546:molybdopterin cofactor binding][GO:0050464:nitrate reductase (NADPH) activity][GO:0042128:nitrate assimilation][GO:0006809:nitric oxide biosynthetic process]	[ko00910:Nitrogen metabolism]	mrna31001.1-v1.0-hybrid:Fragaria vesca|C3H|C3H family protein	ABW05098.1:nitrate reductase [Beta vulgaris] >ACB73000.1 nitrate reductase [Beta vulgaris]	gi|177540044|gb|EU571714.1|:Beta vulgaris nitrate reductase (NR) gene, complete cds	A8IJ84|A8IJ84_BETVU:Nitrate reductase;OS=Beta vulgaris;|GO:0020037,GO:0030151,GO:0043546,GO:0050464,|GO:0042128,GO:0006809,

geneid	trend	GO	Pathway	TF	NR	NT	Uniprot
DN125703_c1_g1_i1	DOWN	[GO:0005737:cytoplasm][GO:0005634:nucleus][GO:0010427:abscisic acid binding][GO:0004864:protein phosphatase inhibitor activity][GO:0009738:abscisic acid-activated signaling pathway][GO:0080163:regulation of protein serine/threonine phosphatase activity]	-	-	XP_021847720.1:lachrymatoryfactor synthase [Spinacia oleracea]	-	I1MA03|I1MA03_SOYBN:Uncharacterized protein;OS=Glycine max;|GO:0005737,GO:0005634,|GO:0010427,GO:0004864,GO:0004872,|GO:0009738,GO:0080163,
DN124534_c1_g2_i1	DOWN	[GO:0005524:ATP binding][GO:0004672:protein kinase activity]	-	ONIVA10G07200.3:Oryza nivara|CAMTA|CAMTA family protein	XP_019105321.1:PREDICTED: serine/threonineprotein kinase HT1like isoform X1 [Beta vulgaris subsp. vulgaris] >XP_019105322.1 PREDICTED: serine/threonineprotein kinase HT1like isoform X1 [Beta vulgaris subsp. vulgaris]	gi|521706407|gb|GAIA01014705.1|:TSA: Salicornia europaea Unigene15367_Se200S transcribed RNA sequence	A0A0J8C926|A0A0J8C926_BETVU:Uncharacterized protein;OS=Beta vulgaris subsp. vulgaris.;|GO:0005524,GO:0004672,

geneid	trend	GO	Pathway	TF	NR	NT	Uniprot
					>KMT10210.1 hypothetical protein BVRB_5g119710 [Beta vulgaris subsp. vulgaris]		
DN126583_c2_g3_i5	DOWN	-	-	-	XP_010667908.1:PREDICTED: B2 protein [Beta vulgaris subsp. vulgaris] >KMS95040.1 hypothetical protein BVRB_013050 [Beta vulgaris subsp. vulgaris]	gi|388510813|gb|BT143679.1|:Medicago truncatula clone JCVI-FLMt-13P4 unknown mRNA	A0A0J8DW35|A0A0J8DW35_BETVU: Uncharacterized protein;OS=Beta vulgaris subsp. vulgaris.;

geneid	trend	GO	Pathway	TF	NR	NT	Uniprot
DN123471_c2_g3_i13	DOWN	[GO:0005777:peroxisome][GO:0046592:polyamine oxidase activity][GO:0006598:polyamine catabolic process]	[ko00330:Arginine and proline metabolism][ko00410:beta-Alanine metabolism]	676755044:Sisymbrium irio|bHLH|bHLH family protein	XP_010675226.1:PREDICTED: probable polyamine oxidase 4 [Beta vulgaris subsp. vulgaris] >KMT13320.1 hypothetical protein BVRB_4g084630 [Beta vulgaris subsp. vulgaris]	gi|521749399|gb|GAIA01001450.1|:TSA: Salicornia europaea Unigene1478_Se200S transcribed RNA sequence	A0A0J8CHY0|A0A0J8CHY0_BETVU: Uncharacterized protein;OS=Beta vulgaris subsp. vulgaris.;|GO:0005777,|GO:0046592,|GO:0006598,
DN127885_c5_g1_i1	DOWN	[GO:0005886:plasma membrane]	-	Sme2.5_07423.1_g00002.1:Solanum melongena|TCP|TCP family protein	XP_010669670.1:PREDICTED: protein IQDOMAIN 1 [Beta vulgaris subsp. vulgaris] >KMT17702.1 hypothetical protein BVRB_2g036470 [Beta vulgaris subsp. vulgaris]	gi|449486537|ref|XM_004157278.1|:PREDICTED: Cucumis sativus protein IQ-DOMAIN 1-like (LOC101209731), mRNA	A0A0J8CVT6|A0A0J8CVT6_BETVU:Uncharacterized protein;OS=Beta vulgaris subsp. vulgaris.;|GO:0005886,

geneid	trend	GO	Pathway	TF	NR	NT	Uniprot
DN125900_c0_g3_i1	DOWN	[GO:0016021:integral component of membrane][GO:0005524:ATP binding][GO:0004672:protein kinase activity]	[ko04075:Plant hormone signal transduction]	-	XP_021841039.1:systemin receptor SR160 [Spinacia oleracea]	gi|521508936|gb|GAHZ01021984.1|:TSA: Salicornia europaea Unigene23635_SeCKS transcribed RNA sequence	A0A0K9RSY6|A0A0K9RSY6_SPIOL:Uncharacterized protein;OS=Spinacia oleracea;|GO:0016021,|GO:0005524,GO:0004672,
DN125885_c1_g2_i3	DOWN	[GO:0016021:integral component of membrane]	-	-	XP_010675196.1:PREDICTED: uncharacterized protein LOC104891234 [Beta vulgaris subsp. vulgaris] >KMT13352.1 hypothetical protein BVRB_4g084430 [Beta vulgaris subsp. vulgaris]	-	A0A0J8CIG1|A0A0J8CIG1_BETVU:Uncharacterized protein;OS=Beta vulgaris subsp. vulgaris.;|GO:0016021,
DN121931_c2_g1_i1	DOWN	[GO:0016021:integral component of membrane][GO:0005524:ATP binding][GO:0005471:ATP:ADP antiporter activity]	-	-	BAD91180.1:putative plastidic adenylate transporter [Mesembryanthemum crystallinum]	gi|61651613|dbj|AB190777.1|:Mesembryanthemum crystallinum ANT1 mRNA for putative plastidic adenylate	Q59IV2|Q59IV2_MESCR:Putative plastidic adenylate transporter;OS=Mesembryanthemum crystallinum;|GO:00

geneid	trend	GO	Pathway	TF	NR	NT	Uniprot
						transporter, complete cds	16021,|GO:0005524, GO:0005471,
DN119636_c1_g1_i1	DOWN	[GO:0005887:integral component of plasma membrane][GO:0008515:sucrose transmembrane transporter activity][GO:0015293:symporter activity][GO:0005985:sucrose metabolic process]	-	Achn143971:Actinidia chinensis|HD-ZIP|HD-ZIP family protein	XP_021760460.1:sucrose transport proteinlike isoform X1 [Chenopodium quinoa]	gi|21318|emb|X67125.1|:S.oleraceae mRNA for sucrose permease	Q03411|SUT_SPIOL:Sucrose transport protein;OS=Spinacia oleracea;|GO:0005887,|GO:0008515,GO:0015293,|GO:0005985,
DN122756_c4_g4_i1	DOWN	[GO:0004190:aspartic-type endopeptidase activity]	-	Pbr030871.1:Pyrus bretschneideri|C2H2|C2H2 family protein	XP_021734363.1:aspartic proteinaselike protein 2 [Chenopodium quinoa]	gi|521483641|gb|GAHZ01047245.1|:TSA: Salicornia europaea Unigene55664_SeCKS transcribed RNA sequence	A0A0K9QSD2|A0A0K9QSD2_SPIOL: Uncharacterized protein;OS=Spinacia oleracea;|GO:0004190,

geneid	trend	GO	Pathway	TF	NR	NT	Uniprot
DN122083_c 4_g2_i3	DOWN	[GO:0046872:metal ion binding][GO:0016491:oxidoreductase activity]	-	676709864:Sisymbrium irio|STAT|STAT family protein	XP_019179826.1:PREDICTED: protein DOWNY MILDEW RESISTANCE 6like [Ipomoea nil]	gi|350540005|ref|NM_001246911.1|:Solanum lycopersicum flavonoid biosynthesis oxidoreductase protein (LOC100736526), mRNA >gi|307159103|gb|GQ925908.1| Solanum lycopersicum flavonoid biosynthesis oxidoreductase protein mRNA, complete cds	A0A0K9QR10|A0A0K9QR10_SPIOL:Uncharacterized protein;OS=Spinacia oleracea;|GO:0046872,GO:0016491,
DN122163_c 2_g1_i4	DOWN	-	-	-	XP_021849429.1:uncharacterized protein LOC110789089 [Spinacia oleracea] >KNA05349.1 hypothetical protein SOVF_191210 [Spinacia oleracea]	-	A0A0K9QDJ8|A0A0K9QDJ8_SPIOL:Uncharacterized protein;OS=Spinacia oleracea;

geneid	trend	GO	Pathway	TF	NR	NT	Uniprot
DN123921_c2_g2_i1	DOWN	[GO:0020037:heme binding][GO:0005506:iron ion binding][GO:0004497:monooxygenase activity][GO:0016705:oxidoreductase activity, acting on paired donors, with incorporation or reduction of molecular oxygen]	[ko00130:Ubiquinone and other terpenoid-quinone biosynthesis][ko00360:Phenylalanine metabolism][ko00940:Phenylpropanoid biosynthesis][ko00941:Flavonoid biosynthesis][ko00945:Stilbenoid, diarylheptanoid and gingerol biosynthesis]	Cc10_g03650:Coffea canephora|G2-like|G2-like family protein	XP_010685980.1:PREDICTED: transcinnamate 4monooxygenase [Beta vulgaris subsp. vulgaris] >KMT05454.1 hypothetical protein BVRB_7g175810 [Beta vulgaris subsp. vulgaris]	gi|225434328|ref|XM_002266202.1|:PREDICTED: Vitis vinifera trans-cinnamate 4-monooxygenase-like (LOC100253493), mRNA	A0A0J8BZK4|A0A0J8BZK4_BETVU:Uncharacterized protein;OS=Beta vulgaris subsp. vulgaris.;|GO:0020037,GO:0005506,GO:0004497,GO:0016705,
DN125177_c1_g4_i1	DOWN	[GO:0005634:nucleus][GO:0008289:lipid binding][GO:0043565:sequence-specific DNA binding][GO:0006355:regulation of transcription, DNA-templated]	-	Bv9_221020_azmu.t1:Beta vulgaris|HD-ZIP|HD-ZIP family protein	XP_010691342.1:PREDICTED: homeoboxleucine zipper protein GLABRA 2 [Beta vulgaris subsp. vulgaris] >KMT00826.1 hypothetical protein BVRB_9g221020 [Beta vulgaris subsp.	gi|470134099|ref|XM_004302846.1|:PREDICTED: Fragaria vesca subsp. vesca homeobox-leucine zipper protein GLABRA 2-like (LOC101315163), mRNA	A0A0J8ECT3|A0A0J8ECT3_BETVU:Uncharacterized protein;OS=Beta vulgaris subsp. vulgaris.;|GO:0005634,|GO:0008289,GO:0043565,|GO:0006355,

geneid	trend	GO	Pathway	TF	NR	NT	Uniprot
					vulgaris]		
DN116840_c6_g1_i2	DOWN	[GO:0004866:endopeptidase inhibitor activity]	-	-	XP_021722896.1:kunitz trypsin inhibitor 2like [Chenopodium quinoa]	-	A0A0K9RR37|A0A0K9RR37_SPIOL:Uncharacterized protein;OS=Spinacia oleracea;|GO:0004866,

geneid	trend	GO	Pathway	TF	NR	NT	Uniprot
DN120258_c1_g4_i7	DOWN	-	[ko00520:Amino sugar and nucleotide sugar metabolism]	MDP0000247737:Malus domestica|FAR1|FAR1 family protein	XP_021716189.1:UDPglucuronic acid decarboxylase 5 [Chenopodium quinoa] >XP_021716190.1 UDPglucuronic acid decarboxylase 5 [Chenopodium quinoa] >XP_021716191.1 UDPglucuronic acid decarboxylase 5 [Chenopodium quinoa] >XP_021720901.1 UDPglucuronic acid decarboxylase 5 [Chenopodium quinoa] >XP_021720902.1 UDPglucuronic acid decarboxylase 5 [Chenopodium quinoa]	gi|359486816|ref|XM_002283835.2|:PREDICTED: Vitis vinifera UDP-glucuronic acid decarboxylase 1-like (LOC100249678), mRNA	A0A0J8BZ49|A0A0J8BZ49_BETVU:Uncharacterized protein;OS=Beta vulgaris subsp. vulgaris.;

geneid	trend	GO	Pathway	TF	NR	NT	Uniprot
					>XP_021720903.1 UDPglucuronic acid decarboxylase 5 [Chenopodium quinoa]		

geneid	trend	GO	Pathway	TF	NR	NT	Uniprot
DN109510_c0_g1_i1	DOWN	[GO:0005634:nucleus][GO:0009734:auxin-activated signaling pathway][GO:0006355:regulation of transcription, DNA-templated][GO:0006351:transcription, DNA-templated]	[ko04075:Plant hormone signal transduction]	-	AAM96891.1:auxinresponsive protein IAA1, partial [Mirabilis jalapa]	gi|521705144|gb|GAIA01015497.1|:TSA: Salicornia europaea Unigene16181_Se200S transcribed RNA sequence	Q8L5G7|Q8L5G7_MIRJA:Auxin-responsive protein;OS=Mirabilis jalapa;|GO:0005634,|GO:0009734,GO:0006355,GO:0006351,
DN119000_c3_g15_i1	DOWN	-	-	-	XP_021838968.1:strigolactone esterase D14 [Spinacia oleracea] >KNA16712.1 hypothetical protein SOVF_086480 [Spinacia oleracea]	-	A0A0K9RD14|A0A0K9RD14_SPIOL:Uncharacterized protein;OS=Spinacia oleracea;

geneid	trend	GO	Pathway	TF	NR	NT	Uniprot
DN121164_c0_g1_i5	DOWN	-	-	Tp57577_TGAC_v2_mRNA41450:Trifolium pratense|C3H|C3H family protein	XP_010693550.1:PREDICTED: fimbrin5 [Beta vulgaris subsp. vulgaris] >XP_010693551.1 PREDICTED: fimbrin5 [Beta vulgaris subsp. vulgaris] >XP_010693552.1 PREDICTED: fimbrin5 [Beta vulgaris subsp. vulgaris] >KMS99097.1 hypothetical protein BVRB_3g066950 [Beta vulgaris subsp. vulgaris]	gi|512398418|gb|GAJZ01002752.1|:TSA: Prunus avium Pa_05719 transcribed RNA sequence	A0A0J8BGD5|A0A0J8BGD5_BETVU: Uncharacterized protein;OS=Beta vulgaris subsp. vulgaris.;

geneid	trend	GO	Pathway	TF	NR	NT	Uniprot
DN125212_c1_g1_i8	DOWN	[GO:0016021:integral component of membrane][GO:0005215:transporter activity]	-	mrna31023.1-v1.0-hybrid:Fragaria vesca|C3H|C3H family protein	XP_021744348.1:protein NRT1/ PTR FAMILY 6.4like [Chenopodium quinoa]	gi|521738019|gb|GAIA01007792.1|:TSA: Salicornia europaea Unigene8112_Se200S transcribed RNA sequence	A0A0J8CW21|A0A0J8CW21_BETVU: Uncharacterized protein;OS=Beta vulgaris subsp. vulgaris.;|GO:0016021,|GO:0005215,
DN118948_c1_g4_i1	DOWN	[GO:0005622:intracellular][GO:0005525:GTP binding][GO:0007264:small GTPase mediated signal transduction]	[ko04144:Endocytosis][ko04961:Endocrine and other factor-regulated calcium reabsorption][ko04962:Vasopressin-regulated water reabsorption][ko04972:Pancreatic secretion]	mrna29786.1-v1.0-hybrid:Fragaria vesca|WOX|WOX family protein	XP_010686242.1:PREDICTED: rasrelated protein RIC2 [Beta vulgaris subsp. vulgaris] >KMT04449.1 hypothetical protein BVRB_8g181210 [Beta vulgaris subsp. vulgaris]	gi|512393586|gb|GAJZ01007584.1|:TSA: Prunus avium Pa_45206 transcribed RNA sequence	A0A0J8BWR4|A0A0J8BWR4_BETVU: Uncharacterized protein;OS=Beta vulgaris subsp. vulgaris.;|GO:0005622,|GO:0005525,|GO:0007264,

geneid	trend	GO	Pathway	TF	NR	NT	Uniprot
DN119966_c0_g1_i2	DOWN	[GO:0005739:mitochondrion][GO:0004129:cytochrome-c oxidase activity]	[ko00190:Oxidative phosphorylation][ko04260:Cardiac muscle contraction][ko04932:Non-alcoholic fatty liver disease (NAFLD)][ko05010:Alzheimer's disease][ko05012:Parkinson's disease][ko05016:Huntington's disease]	OMERI03G21280.1:Oryza meridionalis|FAR1|FAR1 family protein	XP_010545443.1:PREDICTED: cytochrome c oxidase subunit 6b2 [Tarenaya hassleriana]	-	A0A087GBS1|A0A087GBS1_ARAAL: Uncharacterized protein;OS=Arabis alpina;|GO:0005739,|GO:0004129,
DN111555_c0_g1_i1	DOWN	[GO:0006952:defense response][GO:0009607:response to biotic stimulus]	-	-	XP_021732306.1:pathogenesisrelated protein STH21like [Chenopodium quinoa]	-	A0A171NYA8|A0A171NYA8_9CARY: PR10;OS=Halostachys caspica.;|GO:0006952,GO:0009607,

geneid	trend	GO	Pathway	TF	NR	NT	Uniprot
DN122573_c4_g5_i1	DOWN	[GO:0005634:nucleus][GO:0003677:DNA binding]	-	Bv3_060480_yxxg.t1:Beta vulgaris|SBP|SBP family protein	XP_010672689.1:PREDICTED: squamosa promoterbindinglike protein 9 [Beta vulgaris subsp. vulgaris] >KMT15343.1 hypothetical protein BVRB_3g060480 [Beta vulgaris subsp. vulgaris]	gi|521697830|gb|GAIA01018571.1|:TSA: Salicornia europaea Unigene19526_Se200S transcribed RNA sequence	A0A0J8CTR2|A0A0J8CTR2_BETVU:Uncharacterized protein;OS=Beta vulgaris subsp. vulgaris.;|GO:0005634,|GO:0003677,
DN118993_c3_g2_i4	DOWN	-	-	-	XP_010684822.1:PREDICTED: uncharacterized protein LOC104899346 isoform X1 [Beta vulgaris subsp. vulgaris]	-	A0A0J8BX88|A0A0J8BX88_BETVU:Uncharacterized protein;OS=Beta vulgaris subsp. vulgaris.;
DN124144_c0_g2_i1	DOWN	-	-	-	XP_021753418.1:uncharacterized protein LOC110718815 [Chenopodium quinoa]	-	A0A068TMT0|A0A068TMT0_COFCA: Uncharacterized protein;OS=Coffea canephora;

geneid	trend	GO	Pathway	TF	NR	NT	Uniprot
DN112135_c1_g1_i2	DOWN	[GO:0005622:intracellular][GO:0008270:zinc ion binding]	-	Aco008479.1:Ananas comosus|DBB|DBB family protein	EOY28479.1:Bbox type zinc finger family protein, putative isoform 1 [Theobroma cacao]	-	A0A061GMY3|A0A061GMY3_THECC: B-box type zinc finger family protein, putative isoform 1;OS=Theobroma cacao;|GO:0005622,|GO:0008270,
DN123003_c1_g2_i2	DOWN	[GO:0016021:integral component of membrane][GO:0008168:methyltransferase activity]	-	MDP0000277077:Malus domestica|NF-YB|NF-YB family protein	XP_010679252.1:PREDICTED: probable methyltransferase PMT26 [Beta vulgaris subsp. vulgaris] >KMT19667.1 hypothetical protein BVRB_1g010530 [Beta vulgaris subsp. vulgaris]	gi|521524730|gb|GAHZ01011569.1|:TSA: Salicornia europaea Unigene12187_SeCKS transcribed RNA sequence	A0A0J8D1I2|A0A0J8D1I2_BETVU:Uncharacterized protein;OS=Beta vulgaris subsp. vulgaris.;|GO:0016021,|GO:0008168,
DN118447_c3_g6_i5	DOWN	[GO:0016021:integral component of membrane]	-	-	XP_021734187.1:tetraspanin3like [Chenopodium quinoa]	-	A0A0K9QCE4|A0A0K9QCE4_SPIOL: Uncharacterized protein;OS=Spinacia

geneid	trend	GO	Pathway	TF	NR	NT	Uniprot
							oleracea;|GO:0016021,
DN119104_c2_g4_i7	DOWN	-	-	Csa09g092790.1:Camelina sativa|BES1|BES1 family protein	XP_021842613.1:protein trichome birefringencelike 41 [Spinacia oleracea]	gi|225316973|dbj|AK322727.1|:Solanum lycopersicum cDNA, clone: LEFL1042CA07, HTC in leaf	A0A0J8CMI4|A0A0J8CMI4_BETVU:Uncharacterized protein;OS=Beta vulgaris subsp. vulgaris.;
DN122976_c0_g2_i5	DOWN	[GO:0019005:SCF ubiquitin ligase complex]	-	mrna21668.1-v1.0-hybrid:Fragaria vesca|FAR1|FAR1 family protein	XP_010672252.1:PREDICTED: Fbox protein SKIP16 [Beta vulgaris subsp. vulgaris] >KMT15797.1 hypothetical protein BVRB_3g057720 [Beta vulgaris subsp. vulgaris]	-	A0A0J8CQE5|A0A0J8CQE5_BETVU:Uncharacterized protein;OS=Beta vulgaris subsp. vulgaris.;|GO:0019005,

geneid	trend	GO	Pathway	TF	NR	NT	Uniprot
DN123444_c0_g4_i1	DOWN	[GO:0005737:cytoplasm][GO:0004046:aminoacylase activity][GO:0046872:metal ion binding][GO:0008237:metallopeptidase activity][GO:0006520:cellular amino acid metabolic process]	[ko00220:Arginine biosynthesis]	-	XP_021846887.1:aminoacylase1 isoform X2 [Spinacia oleracea]	gi|224102764|ref|XM_002312757.1|:Populus trichocarpa predicted protein, mRNA	A0A0J8BNK5|A0A0J8BNK5_BETVU: Aminoacylase-1;OS=Beta vulgaris subsp. vulgaris.;|GO:0005737,|GO:0004046,GO:0046872,GO:0008237,|GO:0006520,
DN124684_c4_g3_i7	DOWN	-	-	-	XP_021745088.1:phosphatidylinositol/phosphatidylcholine transfer protein SFH2like [Chenopodium quinoa]	gi|521513546|gb|GAHZ01017374.1|:TSA: Salicornia europaea Unigene18313_SeCKS transcribed RNA sequence	A0A0J8CKM3|A0A0J8CKM3_BETVU: Uncharacterized protein;OS=Beta vulgaris subsp. vulgaris.;
DN125366_c4_g2_i1	DOWN	-	-	-	XP_021910017.1:uncharacterized protein At4g22758 [Carica papaya] >APQ44398.1 senescenceassociated protein [Carica papaya]	gi|521562606|gb|GAIA01075642.1|:TSA: Salicornia europaea Unigene91660_Se200S transcribed RNA sequence	M5XHL6|M5XHL6_PRUPE:Uncharacterized protein;OS=Prunus persica;

geneid	trend	GO	Pathway	TF	NR	NT	Uniprot
DN114714_c0_g2_i2	DOWN	-	-	-	XP_010675712.1:PREDICTED: polygalacturonase inhibitor 1like [Beta vulgaris subsp. vulgaris]	-	A0A0J8CL12|A0A0J8CL12_BETVU:Uncharacterized protein;OS=Beta vulgaris subsp. vulgaris.;
DN123940_c2_g2_i5	DOWN	[GO:0016021:integral component of membrane]	-	-	XP_021725515.1:lipid transferlike protein VAS [Chenopodium quinoa]	-	A0A0K9R849|A0A0K9R849_SPIOL:Uncharacterized protein;OS=Spinacia oleracea;|GO:0016021,
DN115701_c0_g1_i1	DOWN	-	-	-	XP_010676512.1:PREDICTED: protein P21like [Beta vulgaris subsp. vulgaris] >XP_010676517.1 PREDICTED: protein P21like [Beta vulgaris subsp. vulgaris] >KMT12125.1 hypothetical protein BVRB_5g100760	-	A0A0J8CEB7|A0A0J8CEB7_BETVU:Uncharacterized protein;OS=Beta vulgaris subsp. vulgaris.;

geneid	trend	GO	Pathway	TF	NR	NT	Uniprot
					[Beta vulgaris subsp. vulgaris]		
DN113088_c2_g1_i2	DOWN	[GO:0009570:chloroplast stroma][GO:0016597:amino acid binding][GO:0047769:arogenate dehydratase activity][GO:0004664:prephenate dehydratase activity][GO:0009094:L-phenylalanine biosynthetic process]	[ko00400:Phenylalanine, tyrosine and tryptophan biosynthesis]	-	XP_010690029.1:PREDICTED: arogenate dehydratase/prephenate dehydratase 6, chloroplastic [Beta vulgaris subsp. vulgaris] >KMT01656.1 hypothetical protein BVRB_9g211170 [Beta vulgaris subsp. vulgaris]	gi|356565692|ref|XM_003551024.1|:PREDICTED: Glycine max arogenate dehydratase/prephenate dehydratase 6, chloroplastic-like (LOC100781285), mRNA	A0A0J8EF91|A0A0J8EF91_BETVU:Arogenate dehydratase;OS=Beta vulgaris subsp. vulgaris.;|GO:0009570,|GO:0016597,GO:0047769,GO:0004664,|GO:0009094,

geneid	trend	GO	Pathway	TF	NR	NT	Uniprot
DN123568_c0_g1_i1	DOWN	[GO:0009507:chloroplast][GO:0003849:3-deoxy-7-phosphoheptulonate synthase activity][GO:0009073:aromatic amino acid family biosynthetic process][GO:0008652:cellular amino acid biosynthetic process]	[ko00400:Phenylalanine, tyrosine and tryptophan biosynthesis][ko02024:Quorum sensing]	-	XP_021863614.1:phospho2dehydro3deoxyheptonate aldolase 2, chloroplasticlike [Spinacia oleracea] >KNA25449.1 hypothetical protein SOVF_006640 [Spinacia oleracea]	gi|521733761|gb|GAIA01010031.1|:TSA: Salicornia europaea Unigene10493_Se200S transcribed RNA sequence	A0A0K9S2N1|A0A0K9S2N1_SPIOL:Phospho-2-dehydro-3-deoxyheptonate aldolase;OS=Spinacia oleracea;|GO:0009507,|GO:0003849,|GO:0009073,GO:0008652,
DN109856_c0_g1_i2	DOWN	[GO:0004190:aspartic-type endopeptidase activity]	-	Peaxi162Scf00170g01227.1:Petunia axillaris|FAR1|FAR1 family protein	XP_010667343.1:PREDICTED: basic 7S globulin [Beta vulgaris subsp. vulgaris] >KMS95450.1 hypothetical protein BVRB_008150 [Beta vulgaris subsp. vulgaris]	-	A0A0J8DX57|A0A0J8DX57_BETVU:Uncharacterized protein;OS=Beta vulgaris subsp. vulgaris.;|GO:0004190,

geneid	trend	GO	Pathway	TF	NR	NT	Uniprot
DN126926_c1_g5_i5	DOWN	[GO:0046872:metal ion binding][GO:0004722:protein serine/threonine phosphatase activity]	[ko04016:MAPK signaling pathway - plant][ko04075:Plant hormone signal transduction][ko04931:Insulin resistance]	KZV22762.1:Dorcoceras hygrometricum|CAMTA|CAMTA family protein	AAC36697.1:protein phosphatase2C [Mesembryanthemum crystallinum]	gi|3643081|gb|AF075579.1|AF075579:Mesembryanthemum crystallinum clone Mpc2 protein phosphatase-2C (PP2C) mRNA, complete cds	O82468|O82468_MESCR:Protein phosphatase-2C;OS=Mesembryanthemum crystallinum;|GO:0046872,GO:0004722,
DN114180_c0_g1_i5	DOWN	[GO:0003676:nucleic acid binding]	-	-	XP_021756397.1:uncharacterized protein LOC110721528 [Chenopodium quinoa] >XP_021756427.1 uncharacterized protein LOC110721557 [Chenopodium quinoa]	-	A0A0K9QEY4|A0A0K9QEY4_SPIOL:Uncharacterized protein;OS=Spinacia oleracea;|GO:0003676,
DN125450_c1_g1_i6	DOWN	-	-	-	-	-	-

geneid	trend	GO	Pathway	TF	NR	NT	Uniprot
DN127501_c0_g1_i3	DOWN	[GO:0016021:integral component of membrane]	[ko04152:AMPK signaling pathway][ko04211:Longevity regulating pathway][ko04920:Adipocytokine signaling pathway][ko04932:Non-alcoholic fatty liver disease (NAFLD)]	MDP0000270344:Malus domestica|BES1|BES1 family protein	XP_010687835.1:PREDICTED: heptahelical transmembrane protein 4 isoform X1 [Beta vulgaris subsp. vulgaris] >KMT19091.1 hypothetical protein BVRB_1g014890 [Beta vulgaris subsp. vulgaris]	gi|255582304|ref|XM_002531897.1|:Ricinus communis conserved hypothetical protein, mRNA	A0A0J8FTS7|A0A0J8FTS7_BETVU:Uncharacterized protein;OS=Beta vulgaris subsp. vulgaris.;|GO:0016021,
DN115114_c1_g2_i3	DOWN	-	-	-	XP_021772877.1:stressrelated proteinlike isoform X1 [Chenopodium quinoa]	-	A0A0K9QJW4|A0A0K9QJW4_SPIOL: Uncharacterized protein;OS=Spinacia oleracea;
DN121571_c4_g1_i4	DOWN	-	-	Achn056131:Actinidia chinensis|NF-YC|NF-YC family protein	XP_021754109.1:remorinlike [Chenopodium quinoa]	gi|238909001|gb|BT043148.2|:Zea mays full-length cDNA clone ZM_BFc0109K17 mRNA, complete cds	A0A0J8CEA6|A0A0J8CEA6_BETVU:Uncharacterized protein;OS=Beta vulgaris subsp. vulgaris.;

geneid	trend	GO	Pathway	TF	NR	NT	Uniprot
DN120496_c0_g2_i1	DOWN	[GO:0016021:integral component of membrane][GO:0005215:transporter activity]	-	Prupe.3G009400.1.p:Prunus persica|ERF|ERF family protein	XP_017229927.1:PREDICTED: probable aquaporin PIP28 [Daucus carota subsp. sativus]	gi|1657949|gb|U73467.1|MCU73467:Mesembryanthemum crystallinum water channel protein MipE mRNA, complete cds	A0A059APF1|A0A059APF1_EUCGR:Uncharacterized protein;OS=Eucalyptus grandis;|GO:0016021,|GO:0005215,
DN126170_c1_g2_i1	DOWN	-	-	-	XP_010679046.1:PREDICTED: myosinbinding protein 2 [Beta vulgaris subsp. vulgaris]	-	A0A0J8F370|A0A0J8F370_BETVU:Uncharacterized protein;OS=Beta vulgaris subsp. vulgaris.;
DN120612_c6_g16_i1	DOWN	-	-	-	-	-	-
DN121585_c2_g1_i9	DOWN	-	-	-	-	-	-
DN126583_c2_g3_i4	DOWN	-	-	-	XP_010667908.1:PREDICTED: B2 protein [Beta vulgaris subsp. vulgaris] >KMS95040.1 hypothetical protein	gi|388510813|gb|BT143679.1|:Medicago truncatula clone JCVI-FLMt-13P4 unknown mRNA	A0A0J8DW35|A0A0J8DW35_BETVU:Uncharacterized protein;OS=Beta vulgaris subsp. vulgaris.;

geneid	trend	GO	Pathway	TF	NR	NT	Uniprot
					BVRB_013050 [Beta vulgaris subsp. vulgaris]		
DN121931_c 2_g1_i4	DOWN	[GO:0016021:integral component of membrane][GO:0005524:ATP binding][GO:0005471:ATP:ADP antiporter activity]	-	-	BAD91180.1:putativ e plastidic adenylate transporter [Mesembryanthemu m crystallinum]	gi|61651613|dbj|AB 190777.1|:Mesembr yanthemum crystallinum ANT1 mRNA for putative plastidic adenylate transporter, complete cds	Q59IV2|Q59IV2_M ESCR:Putative plastidic adenylate transporter;OS=Mes embryanthemum crystallinum;|GO:00 16021,|GO:0005524, GO:0005471,
DN127259_c 2_g1_i4	DOWN	-	-	-	XP_021865553.1:gl ycinerich domaincontaining protein 1like isoform X3 [Spinacia oleracea] >KNA12464.1 hypothetical protein SOVF_125400 [Spinacia oleracea]	gi|521743119|gb|GA IA01004608.1|:TSA: Salicornia europaea Unigene4892_Se200 S transcribed RNA sequence	A0A0K9QYW3|A0 A0K9QYW3_SPIO L:Uncharacterized protein;OS=Spinacia oleracea;

geneid	trend	GO	Pathway	TF	NR	NT	Uniprot
DN117979_c3_g2_i3	DOWN	-	-	-	XP_021744977.1:uncharacterized protein At1g66480like [Chenopodium quinoa]	-	A0A0J8C7N7|A0A0J8C7N7_BETVU:Uncharacterized protein;OS=Beta vulgaris subsp. vulgaris.;
DN118876_c1_g1_i1	DOWN	[GO:0016021:integral component of membrane]	-	Kaladp0839s0004.1.p:Kalanchoe laxiflora|ERF|ERF family protein	XP_021719626.1:cysteinerich receptorlike protein kinase 10 isoform X1 [Chenopodium quinoa]	-	A0A0K9QH32|A0A0K9QH32_SPIOL:Uncharacterized protein;OS=Spinacia oleracea;|GO:0016021,
DN119284_c0_g1_i3	DOWN	[GO:0005737:cytoplasm][GO:0004801:sedoheptulose-7-phosphate:D-glyceraldehyde-3-phosphate glyceronetransferase activity][GO:0005975:carbohydrate metabolic process][GO:0006098:pentose-phosphate shunt]	[ko00030:Pentose phosphate pathway]	-	XP_021845985.1:uncharacterized protein LOC110785777 [Spinacia oleracea] >KNA23727.1 hypothetical protein SOVF_021530 [Spinacia oleracea]	gi|502090151|ref|XM_004489080.1|:PREDICTED: Cicer arietinum transaldolase-like (LOC101507457), mRNA	A0A0K9RY10|A0A0K9RY10_SPIOL:Uncharacterized protein;OS=Spinacia oleracea;|GO:0005737,|GO:0004801,|GO:0005975,GO:0006098,

geneid	trend	GO	Pathway	TF	NR	NT	Uniprot
DN124729_c4_g1_i1	DOWN	[GO:0016021:integral component of membrane]	-	-	XP_010691066.1:PREDICTED: uncharacterized protein At4g15970 isoform X2 [Beta vulgaris subsp. vulgaris]	gi|521513387|gb|GAHZ01017533.1|:TSA: Salicornia europaea Unigene18473_SeCKS transcribed RNA sequence	A0A0J8BKK9|A0A0J8BKK9_BETVU: Uncharacterized protein;OS=Beta vulgaris subsp. vulgaris.;|GO:0016021,
DN127480_c2_g2_i1	DOWN	-	-	-	XP_021843462.1:uncharacterized protein LOC110783431 [Spinacia oleracea] >KNA24409.1 hypothetical protein SOVF_015980 [Spinacia oleracea]	-	A0A0K9RZQ2|A0A0K9RZQ2_SPIOL: Uncharacterized protein;OS=Spinacia oleracea;
DN120189_c2_g2_i1	DOWN	-	-	-	XP_010674262.1:PREDICTED: uncharacterized protein LOC104890460 [Beta vulgaris subsp. vulgaris] >KMT13905.1 hypothetical protein BVRB_4g077460	gi|387965701|gb|JN606071.1|:Beta vulgaris subsp. vulgaris cultivar US H20 genomic sequence	A0A0J8CJP2|A0A0J8CJP2_BETVU:Uncharacterized protein;OS=Beta vulgaris subsp. vulgaris.;

geneid	trend	GO	Pathway	TF	NR	NT	Uniprot
					[Beta vulgaris subsp. vulgaris]		
DN127528_c3_g1_i1	DOWN	[GO:0010158:abaxial cell fate specification][GO:0007275:multicellular organism development]	-	Bv7_171330_nfki.t1:Beta vulgaris|YABBY|YABBY family protein	XP_010685466.1:PREDICTED: putative axial regulator YABBY 2 isoform X1 [Beta vulgaris subsp. vulgaris] >KMT04969.1 hypothetical protein BVRB_7g171330 isoform A [Beta vulgaris subsp. vulgaris]	gi|521751381|gb|GAIA01000457.1|:TSA: Salicornia europaea Unigene466_Se200S transcribed RNA sequence	A0A0J8BY87|A0A0J8BY87_BETVU:Uncharacterized protein;OS=Beta vulgaris subsp. vulgaris.;|GO:0010158,GO:0007275,
DN122950_c7_g8_i1	DOWN	-	-	-	-	-	-

geneid	trend	GO	Pathway	TF	NR	NT	Uniprot
DN113507_c1_g1_i3	DOWN	[GO:0046872:metal ion binding][GO:0004722:protein serine/threonine phosphatase activity]	-	KZV22762.1:Dorcoceras hygrometricum|CAMTA|CAMTA family protein	XP_019104666.1:PREDICTED: probable protein phosphatase 2C 39 isoform X3 [Beta vulgaris subsp. vulgaris] >KMT13144.1 hypothetical protein BVRB_4g086840 isoform C [Beta vulgaris subsp. vulgaris]	gi|521534795|gb|GAHZ01005914.1|:TSA: Salicornia europaea Unigene6219_SeCKS transcribed RNA sequence	A0A0J8CMA0|A0A0J8CMA0_BETVU: Uncharacterized protein;OS=Beta vulgaris subsp. vulgaris.;|GO:0046872,GO:0004722,
DN118616_c4_g4_i2	DOWN	-	-	-	EPS74505.1:hypothetical protein M569_00222, partial [Genlisea aurea]	gi|333499835|gb|JF766557.1|:Uncultured bacterium clone Y4-15 16S ribosomal RNA gene, partial sequence	A0A0V0H8Z9|A0A0V0H8Z9_SOLCH: Putative ovule protein;OS=Solanum chacoense;

geneid	trend	GO	Pathway	TF	NR	NT	Uniprot
DN116735_c0_g2_i9	DOWN	-	-	SMil_00007993-RA_Salv:Salvia miltiorrhiza|ERF|ERF family protein	XP_021855423.1:uncharacterized protein LOC110794773 [Spinacia oleracea] >KNA05846.1 hypothetical protein SOVF_186520 [Spinacia oleracea]	gi|521749993|gb|GAIA01001152.1|:TSA: Salicornia europaea Unigene1177_Se200S transcribed RNA sequence	A0A0K9QH01|A0A0K9QH01_SPIOL:Uncharacterized protein;OS=Spinacia oleracea;
DN123767_c3_g1_i3	DOWN	[GO:0008270:zinc ion binding]	-	-	XP_010685779.1:PREDICTED: putative E3 ubiquitinprotein ligase XBAT31 [Beta vulgaris subsp. vulgaris] >KMT05262.1 hypothetical protein BVRB_7g174010 [Beta vulgaris subsp. vulgaris]	gi|144962157|gb|EF470290.1|:Mirabilis jalapa ubiquitin ligase (XB3) mRNA, complete cds	A0A0J8BVV2|A0A0J8BVV2_BETVU: Uncharacterized protein;OS=Beta vulgaris subsp. vulgaris.;|GO:0008270,

geneid	trend	GO	Pathway	TF	NR	NT	Uniprot
DN112998_c0_g1_i6	DOWN	[GO:0016021:integral component of membrane][GO:0008324:cation transmembrane transporter activity]	-	maker-scaffold18721-augustus-gene-0.3-mRNA-1:Castanea mollissima|Dof|Dof family protein	XP_010673527.1:PREDICTED: metal tolerance protein 4 [Beta vulgaris subsp. vulgaris] >KMT14373.1 hypothetical protein BVRB_4g071530 [Beta vulgaris subsp. vulgaris]	gi|521554071|gb|GAIA01080206.1|:TSA: Salicornia europaea Unigene97353_Se200S transcribed RNA sequence	A0A0J8CQT4|A0A0J8CQT4_BETVU:Uncharacterized protein;OS=Beta vulgaris subsp. vulgaris.;|GO:0016021,|GO:0008324,
DN122473_c0_g2_i3	DOWN	[GO:0016021:integral component of membrane]	-	-	XP_019106567.1:PREDICTED: uncharacterized protein LOC104900147 [Beta vulgaris subsp. vulgaris] >KMT05286.1 hypothetical protein BVRB_7g174240 [Beta vulgaris subsp. vulgaris]	gi|359478977|ref|XM_002281167.2|:PREDICTED: Vitis vinifera uncharacterized LOC100248378 (LOC100248378), mRNA	A0A0J8EQJ3|A0A0J8EQJ3_BETVU:Uncharacterized protein;OS=Beta vulgaris subsp. vulgaris.;|GO:0016021,

geneid	trend	GO	Pathway	TF	NR	NT	Uniprot
DN125250_c0_g11_i2	DOWN	[GO:0016157:sucrose synthase activity][GO:0005985:sucrose metabolic process]	[ko00500:Starch and sucrose metabolism]	mrna11429.1-v1.0-hybrid:Fragaria vesca|HB-other|HB-other family protein	AFS17277.1:sucrose synthase 1 [Amaranthus cruentus/Amaranthus hypocondriacus mixed library]	gi|38425094|gb|AY457173.1|:Beta vulgaris sucrose synthase (SBSS2) mRNA, complete cds	J9WP95|J9WP95_9CARY:Sucrose synthase;OS=Amaranthus cruentus/Amaranthus hypocondriacus mixed library.;|GO:0016157,|GO:0005985,
DN120026_c3_g1_i3	DOWN	[GO:0008234:cysteine-type peptidase activity]	[ko04142:Lysosome][ko04210:Apoptosis]	FANhyb_rscf00000253.1.g00010.1:Fragaria x ananassa|BBR-BPC|BBR-BPC family protein	XP_021750293.1:thiol protease aleurainlike [Chenopodium quinoa]	gi|521650765|gb|GAIA01041730.1|:TSA: Salicornia europaea Unigene46882_Se200S transcribed RNA sequence	A0A0J8B8R9|A0A0J8B8R9_BETVU:Uncharacterized protein;OS=Beta vulgaris subsp. vulgaris.;|GO:0008234,
DN123791_c5_g1_i5	DOWN	[GO:0016021:integral component of membrane][GO:0055085:transmembrane transport]	-	DCAR_009102:Daucus carota|LBD|LBD family protein	XP_021837631.1:protein ZINC INDUCED FACILITATORLIKE 1like isoform X2 [Spinacia oleracea]	-	A0A0K9RQK3|A0A0K9RQK3_SPIOL: Uncharacterized protein;OS=Spinacia oleracea;|GO:0016021,|GO:0055085,

geneid	trend	GO	Pathway	TF	NR	NT	Uniprot
DN124690_c1_g3_i2	DOWN	[GO:0004108:citrate (Si)-synthase activity][GO:0006101:citrate metabolic process][GO:0006099:tricarboxylic acid cycle]	[ko00020:Citrate cycle (TCA cycle)][ko00630:Glyoxylate and dicarboxylate metabolism]	RrC6748_p2:Raphanus raphanistrum|MYB_related|MYB_related family protein	XP_010691175.1:PREDICTED: citrate synthase, mitochondrial isoform X1 [Beta vulgaris subsp. vulgaris]	gi|1556379|emb|X84228.1|:B.vulgaris mRNA for citrate synthase	A0A0J8BI86|A0A0J8BI86_BETVU:Citrate synthase;OS=Beta vulgaris subsp. vulgaris.;|GO:0004108,|GO:0006101,GO:0006099,
DN126557_c3_g7_i6	DOWN	[GO:0016020:membrane][GO:0020037:heme binding][GO:0005506:iron ion binding][GO:0016709:oxidoreductase activity, acting on paired donors, with incorporation or reduction of molecular oxygen, NAD(P)H as one donor, and incorporation of one atom of oxygen][GO:0044550:secondary metabolite biosynthetic process]	-	Csa09g009740.1:Camelina sativa|HSF|HSF family protein	XP_010674264.1:PREDICTED: cytochrome P450 71A1like [Beta vulgaris subsp. vulgaris]	-	F6GZR8|F6GZR8_VITVI:Putative uncharacterized protein;OS=Vitis vinifera;GN=VIT_18s0001g13770 {ECO:0000313|EMBL:CCB46010.1};|GO:0016020,|GO:0020037,GO:0005506,GO:0016709,|GO:0044550,

geneid	trend	GO	Pathway	TF	NR	NT	Uniprot
DN122109_c1_g1_i2	DOWN	-	-	-	XP_021751281.1:uncharacterized protein LOC110716938 [Chenopodium quinoa] >XP_021751282.1 uncharacterized protein LOC110716938 [Chenopodium quinoa]	gi|356524865|ref|XM_003531001.1|:PREDICTED: Glycine max uncharacterized protein LOC100787824 (LOC100787824), mRNA	A0A0J8BR72|A0A0J8BR72_BETVU:Uncharacterized protein;OS=Beta vulgaris subsp. vulgaris.;
DN120033_c1_g1_i6	DOWN	[GO:0016021:integral component of membrane]	[ko04020:Calcium signaling pathway][ko04218:Cellular senescence][ko04621:NOD-like receptor signaling pathway]	Zmw_sc01544.1.g00110.1:Zoysia matrella|GRAS|GRAS family protein	XP_010680913.1:PREDICTED: calcium uniporter protein 2, mitochondrial isoform X2 [Beta vulgaris subsp. vulgaris] >KMT08501.1 hypothetical protein BVRB_6g137880 isoform B [Beta vulgaris subsp. vulgaris]	gi|356562328|ref|XM_003549376.1|:PREDICTED: Glycine max uncharacterized protein LOC100808882 (LOC100808882), mRNA	A0A0J8EZ60|A0A0J8EZ60_BETVU:Uncharacterized protein;OS=Beta vulgaris subsp. vulgaris.;|GO:0016021,

geneid	trend	GO	Pathway	TF	NR	NT	Uniprot
DN127939_c1_g1_i1	DOWN	[GO:0005622:intracellular][GO:0004435:phosphatidylinositol phospholipase C activity][GO:0004871:obsolete signal transducer activity][GO:0035556:intracellular signal transduction][GO:0016042:lipid catabolic process]	[ko00562:Inositol phosphate metabolism][ko04020:Calcium signaling pathway][ko04070:Phosphatidylinositol signaling system][ko04919:Thyroid hormone signaling pathway][ko04933:AGE-RAGE signaling pathway in diabetic complications]	RrC2946_p1:Raphanus raphanistrum|C3H|C3H family protein	XP_010689668.1:PREDICTED: phosphoinositide phospholipase C 6 [Beta vulgaris subsp. vulgaris]	gi|460378981|ref|XM_004235196.1|:PREDICTED: Solanum lycopersicum phosphoinositide phospholipase C 6-like (LOC101261499), mRNA	A0A0J8CWT6|A0A0J8CWT6_BETVU: Phosphoinositide phospholipase C;OS=Beta vulgaris subsp. vulgaris.;|GO:0005622,|GO:0004435,GO:0004871,|GO:0035556,GO:0016042,
DN120033_c1_g1_i5	DOWN	[GO:0016021:integral component of membrane]	[ko04020:Calcium signaling pathway][ko04218:Cellular senescence][ko04621:NOD-like receptor signaling pathway]	Zmw_sc01544.1.g00110.1:Zoysia matrella|GRAS|GRAS family protein	XP_010680913.1:PREDICTED: calcium uniporter protein 2, mitochondrial isoform X2 [Beta vulgaris subsp. vulgaris] >KMT08501.1 hypothetical protein	gi|356562328|ref|XM_003549376.1|:PREDICTED: Glycine max uncharacterized protein LOC100808882 (LOC100808882), mRNA	A0A0J8EZ60|A0A0J8EZ60_BETVU:Uncharacterized protein;OS=Beta vulgaris subsp. vulgaris.;|GO:0016021,

geneid	trend	GO	Pathway	TF	NR	NT	Uniprot
					BVRB_6g137880 isoform B [Beta vulgaris subsp. vulgaris]		
DN128446_c3_g1_i4	DOWN	[GO:0003824:catalytic activity][GO:0008152:metabolic process]	-	-	XP_021844302.1:oxalateCoA ligase [Spinacia oleracea] >KNA11856.1 hypothetical protein SOVF_131270 [Spinacia oleracea]	gi|224137015|ref|XM_002322437.1|:Populus trichocarpa acyl:coa ligase (ACLL2), mRNA	A0A0K9QZ94|A0A0K9QZ94_SPIOL:Uncharacterized protein;OS=Spinacia oleracea;|GO:0003824,|GO:0008152,
DN108778_c1_g1_i1	DOWN	-	-	-	-	-	-
DN114225_c1_g1_i1	DOWN	[GO:0005509:calcium ion binding]	[ko04626:Plant-pathogen interaction]	-	XP_021847833.1:probable calciumbinding protein CML41 [Spinacia oleracea]	-	A0A0K9QFI1|A0A0K9QFI1_SPIOL:Uncharacterized protein;OS=Spinacia oleracea;|GO:0005509,

geneid	trend	GO	Pathway	TF	NR	NT	Uniprot
DN121454_c7_g1_i1	DOWN	-	-	-	-	-	-
DN125129_c1_g1_i2	DOWN	[GO:0008270:zinc ion binding]	-	678455432:Utricularia gibba|G2-like|G2-like family protein	XP_019107309.1:PREDICTED: uncharacterized protein LOC104902866 [Beta vulgaris subsp. vulgaris] >KMT02301.1 hypothetical protein BVRB_9g205260 [Beta vulgaris subsp. vulgaris]	gi|521718451|gb|GAIA01011570.1|:TSA: Salicornia europaea Unigene12185_Se200S transcribed RNA sequence	A0A0J8EH17|A0A0J8EH17_BETVU:Uncharacterized protein;OS=Beta vulgaris subsp. vulgaris.;|GO:0008270,
DN123380_c6_g1_i5	DOWN	-	-	-	XP_010679062.1:PREDICTED: late embryogenesis abundant protein 31 [Beta vulgaris subsp. vulgaris] >KMT10181.1 hypothetical protein BVRB_5g119420 [Beta vulgaris subsp. vulgaris]	-	A0A0J8F381|A0A0J8F381_BETVU:Uncharacterized protein;OS=Beta vulgaris subsp. vulgaris.;

geneid	trend	GO	Pathway	TF	NR	NT	Uniprot
DN123327_c2_g2_i1	DOWN	[GO:0046872:metal ion binding][GO:0030001:metal ion transport]	-	-	XP_010674786.1:PREDICTED: heavy metalassociated isoprenylated plant protein 32like [Beta vulgaris subsp. vulgaris]	-	A0A0J8CJS6|A0A0J8CJS6_BETVU:Uncharacterized protein;OS=Beta vulgaris subsp. vulgaris.;|GO:0046872,|GO:0030001,
DN125706_c3_g2_i4	DOWN	[GO:0004722:protein serine/threonine phosphatase activity]	-	KZV22762.1:Dorcoceras hygrometricum|CAMTA|CAMTA family protein	XP_009348094.1:PREDICTED: probable protein phosphatase 2C 10 [Pyrus x bretschneideri] >XP_009348095.1 PREDICTED: probable protein phosphatase 2C 10 [Pyrus x bretschneideri]	-	A0A061FGF6|A0A061FGF6_THECC:Phosphatase 2C family protein isoform 5;OS=Theobroma cacao;|GO:0004722,

geneid	trend	GO	Pathway	TF	NR	NT	Uniprot
DN126749_c1_g1_i3	DOWN	[GO:0005737:cytoplasm][GO:0003871:5-methyltetrahydropteroyltriglutamate-homocysteine S-methyltransferase activity][GO:0008270:zinc ion binding][GO:0009086:methionine biosynthetic process]	[ko00270:Cysteine and methionine metabolism][ko00450:Selenocompound metabolism]	-	P93263.1:RecName: Full=5methyltetrahydropteroyltriglutamatehomocysteine methyltransferase; AltName: Full=Cobalaminindependent methionine synthase isozyme; AltName: Full=VitaminB12independent methionine synthase isozyme >AAB41896.1 methionine synthase [Mesembryanthemum crystallinum]	gi|1814402|gb|U84889.1|MCU84889:Mesembryanthemum crystallinum methionine synthase (MetE) mRNA, complete cds	P93263|METE_MESCR:5-methyltetrahydropteroyltriglutamate--homocysteine methyltransferase;OS=Mesembryanthemum crystallinum;|GO:0005737,|GO:0003871,GO:0008270,|GO:0009086,
DN119786_c0_g13_i3	DOWN	-	-	-	-	-	-

geneid	trend	GO	Pathway	TF	NR	NT	Uniprot
DN115893_c4_g1_i12	DOWN	[GO:0016021:integral component of membrane]	-	-	XP_010667529.1:PREDICTED: uncharacterized protein LOC104884553 [Beta vulgaris subsp. vulgaris] >KMS95320.1 hypothetical protein BVRB_009370 [Beta vulgaris subsp. vulgaris]	-	A0A0J8DWT3|A0A0J8DWT3_BETVU: Uncharacterized protein;OS=Beta vulgaris subsp. vulgaris.;|GO:0016021,
DN124464_c2_g2_i10	DOWN	[GO:0005737:cytoplasm][GO:0005524:ATP binding][GO:0046872:metal ion binding][GO:0004478:methionine adenosyltransferase activity][GO:0006730:one-carbon metabolic process][GO:0006556:S-adenosylmethionine biosynthetic process]	[ko00270:Cysteine and methionine metabolism]	-	XP_021734528.1:Sadenosylmethionine synthase 4 [Chenopodium quinoa]	gi|1724103|gb|U79767.1|MCU79767:Mesembryanthemum crystallinum methionine adenosyltransferase mRNA, complete cds	Q6F3F1|METK4_ATRNU:S-adenosylmethionine synthase 4;OS=Atriplex nummularia;|GO:0005737,|GO:0005524,GO:0046872,GO:0004478,|GO:0006730,GO:0006556,

geneid	trend	GO	Pathway	TF	NR	NT	Uniprot
DN121547_c1_g1_i4	DOWN	-	-	-	XP_010688094.1:PREDICTED: EG45like domain containing protein isoform X2 [Beta vulgaris subsp. vulgaris] >KMT02958.1 hypothetical protein BVRB_8g195280 [Beta vulgaris subsp. vulgaris]	-	A0A0J8BST7|A0A0J8BST7_BETVU:Uncharacterized protein;OS=Beta vulgaris subsp. vulgaris.;
DN128867_c2_g1_i2	DOWN	-	-	-	-	-	-
DN128748_c1_g1_i1	DOWN	[GO:0004190:aspartic-type endopeptidase activity]	-	Peaxi162Scf00170g01227.1:Petunia axillaris|FAR1|FAR1 family protein	XP_021750345.1:basic 7S globulinlike [Chenopodium quinoa]	-	Q3KU27|Q3KU27_NICLS:Nectarin IV;OS=Nicotiana langsdorffii x Nicotiana sanderae;|GO:0004190,

geneid	trend	GO	Pathway	TF	NR	NT	Uniprot
DN123021_c1_g1_i1	DOWN	[GO:0004553:hydrolase activity, hydrolyzing O-glycosyl compounds][GO:0005975:carbohydrate metabolic process]	[ko00460:Cyanoamino acid metabolism][ko00500:Starch and sucrose metabolism][ko00940:Phenylpropanoid biosynthesis]	mrna03614.1-v1.0-hybrid:Fragaria vesca|C2H2|C2H2 family protein	XP_010677827.1:PREDICTED: uncharacterized protein LOC104893418 [Beta vulgaris subsp. vulgaris] >XP_010677828.1 PREDICTED: uncharacterized protein LOC104893418 [Beta vulgaris subsp. vulgaris] >KMT11326.1 hypothetical protein BVRB_5g110070 [Beta vulgaris subsp. vulgaris]	gi|70927644|gb|DQ103699.1|:Gossypium hirsutum endo-alpha-1,4-glucanase mRNA, complete cds	A0A0J8F5T9|A0A0J8F5T9_BETVU:Uncharacterized protein;OS=Beta vulgaris subsp. vulgaris.;|GO:0004553,|GO:0005975,
DN121068_c2_g3_i9	DOWN	[GO:0016021:integral component of membrane][GO:0015369:calcium:proton antiporter activity]	-	DCAR_013815:Daucus carota|ERF|ERF family protein	BAU21067.1:Ca2+/H+ exchanger [Salicornia europaea]	gi|356551862|ref|XM_003544244.1|:PREDICTED: Glycine max vacuolar cation/proton exchanger 1-like	A0A0U4VVR1|A0A0U4VVR1_SALEU:Ca2+/H+ exchanger;OS=Salicornia europaea;|GO:00160

geneid	trend	GO	Pathway	TF	NR	NT	Uniprot
						(LOC100816844), mRNA	21,|GO:0015369,
DN126155_c2_g6_i1	DOWN	[GO:0003824:catalytic activity]	[ko00564:Glycerophospholipid metabolism][ko00565:Ether lipid metabolism][ko04014:Ras signaling pathway][ko04024:cAMP signaling pathway][ko04071:Sphingolipid signaling pathway][ko04072:Phospholipase D signaling pathway][ko04144:Endocytosis][ko04666:Fc gamma R-mediated phagocytosis][ko047	-	XP_021856475.1:phospholipase D gamma 1like [Spinacia oleracea] >KNA03891.1 hypothetical protein SOVF_204840 [Spinacia oleracea]	gi|478159485|gb|GAHK01017118.1|:TSA: Jatropha curcas Unigene4572_JC-CK_1A transcribed RNA sequence	A0A0K9QBG9|A0A0K9QBG9_SPIOL: Uncharacterized protein;OS=Spinacia oleracea;|GO:0003824,

geneid	trend	GO	Pathway	TF	NR	NT	Uniprot
			24:Glutamatergic synapse][ko04912:GnRH signaling pathway][ko05231:Choline metabolism in cancer]				
DN117966_c0_g3_i1	DOWN	[GO:0004185:serine-type carboxypeptidase activity]	-	-	XP_021729852.1:serine carboxypeptidaselike 51 [Chenopodium quinoa]	gi|521633343|gb|GAIA01049761.1|:TSA: Salicornia europaea Unigene58197_Se200S transcribed RNA sequence	A0A061GZU6|A0A061GZU6_THECC: Carboxypeptidase;OS=Theobroma cacao;|GO:0004185,

geneid	trend	GO	Pathway	TF	NR	NT	Uniprot
DN118157_c4_g11_i7	DOWN	[GO:0003824:catalytic activity][GO:0030170:pyridoxal phosphate binding][GO:0009058:biosynthetic process]	[ko00220:Arginine biosynthesis][ko00250:Alanine, aspartate and glutamate metabolism][ko00710:Carbon fixation in photosynthetic organisms]	-	XP_021847467.1:alanine aminotransferase 2 [Spinacia oleracea] >KNA25948.1 hypothetical protein SOVF_001640 [Spinacia oleracea]	gi|521511812|gb|GAHZ01019108.1|:TSA: Salicornia europaea Unigene20057_SeCKS transcribed RNA sequence	A0A0K9S2L4|A0A0K9S2L4_SPIOL:Uncharacterized protein;OS=Spinacia oleracea;|GO:0003824,GO:0030170,|GO:0009058,
DN122740_c1_g1_i1	DOWN	-	-	-	-	-	-
DN125838_c1_g3_i2	DOWN	[GO:0048046:apoplast][GO:0005618:cell wall][GO:0016021:integral component of membrane][GO:0004553:hydrolase activity, hydrolyzing O-glycosyl compounds][GO:0016762:xyloglucan:xyloglucosyl transferase activity][GO:0042546:cell wall biogenesis][GO:0071555:cell wall organization][GO:0010411:xyloglucan metabolic process]	-	Sme2.5_00096.1_g00018.1:Solanum melongena|NAC|NAC family protein	XP_021851909.1:probable xyloglucan endotransglucosylase/hydrolase protein 30 [Spinacia oleracea] >KNA23029.1 hypothetical protein SOVF_028460 [Spinacia oleracea]	-	A0A0K9RVS5|A0A0K9RVS5_SPIOL:Xyloglucan endotransglucosylase/hydrolase;OS=Spinacia oleracea;|GO:0048046,GO:0005618,GO:0016021,|GO:0004553,GO:0016762,|GO:0042546,GO:0071555,GO:0010411,

geneid	trend	GO	Pathway	TF	NR	NT	Uniprot
DN128553_c4_g1_i1	DOWN	[GO:0016491:oxidoreductase activity][GO:0008270:zinc ion binding]	[ko00940:Phenylpropanoid biosynthesis]	-	ADU64756.1:cinnamyl alcohol dehydrogenase [Hevea brasiliensis] >ADU64757.1 cinnamyl alcohol dehydrogenase [Hevea brasiliensis]	-	E9M774|E9M774_HEVBR:Cinnamyl alcohol dehydrogenase;OS=Hevea brasiliensis;|GO:0016491,GO:0008270,

geneid	trend	GO	Pathway	TF	NR	NT	Uniprot
DN117874_c0_g1_i8	DOWN	[GO:0009507:chloroplast][GO:0000036:acyl carrier activity][GO:0016297:acyl-[acyl-carrier-protein] hydrolase activity]	[ko00061:Fatty acid biosynthesis]	-	XP_010688133.1:PREDICTED: palmitoylacyl carrier protein thioesterase, chloroplastic [Beta vulgaris subsp. vulgaris] >XP_010688134.1 PREDICTED: palmitoylacyl carrier protein thioesterase, chloroplastic [Beta vulgaris subsp. vulgaris] >XP_019106990.1 PREDICTED: palmitoylacyl carrier protein thioesterase, chloroplastic [Beta vulgaris subsp. vulgaris] >KMT02998.1 hypothetical protein BVRB_8g195640 [Beta vulgaris subsp.	gi|521519478|gb|GAHZ01014405.1|:TSA: Salicornia europaea Unigene15067_SeCKS transcribed RNA sequence	A0A0J8BSY2|A0A0J8BSY2_BETVU:Acyl-[acyl-carrier-protein] hydrolase;OS=Beta vulgaris subsp. vulgaris.;|GO:0009507,|GO:0000036,GO:0016297,

geneid	trend	GO	Pathway	TF	NR	NT	Uniprot
					vulgaris]		

geneid	trend	GO	Pathway	TF	NR	NT	Uniprot
DN127925_c2_g1_i3	DOWN	[GO:0016021:integral component of membrane][GO:0005524:ATP binding][GO:0005388:calcium-transporting ATPase activity][GO:0046872:metal ion binding]	-	Lus10040166:Linum usitatissimum|GRAS|GRAS family protein	XP_010687432.1:PREDICTED: calciumtransporting ATPase 10, plasma membranetype isoform X1 [Beta vulgaris subsp. vulgaris]	gi|478157351|gb|GAHK01019252.1|:TSA: Jatropha curcas Unigene6706_JC-CK_1A transcribed RNA sequence	A0A0J8EL09|A0A0J8EL09_BETVU:Calcium-transporting ATPase;OS=Beta vulgaris subsp. vulgaris.;|GO:0016021,|GO:0005524,GO:0005388,GO:0046872,
DN114298_c2_g1_i8	DOWN	[GO:0005840:ribosome][GO:0003735:structural constituent of ribosome][GO:0006412:translation]	[ko03010:Ribosome]	-	XP_010671181.1:PREDICTED: 60S ribosomal protein L131 [Beta vulgaris subsp. vulgaris] >KMT16742.1 hypothetical protein BVRB_3g050250 [Beta vulgaris subsp. vulgaris]	gi|521718379|gb|GAIA01011606.1|:TSA: Salicornia europaea Unigene12221_Se200S transcribed RNA sequence	A0A0J8CT12|A0A0J8CT12_BETVU:60S ribosomal protein L13;OS=Beta vulgaris subsp. vulgaris.;|GO:0005840,|GO:0003735,|GO:0006412,
DN125731_c4_g3_i1	DOWN	-	-	-	-	-	-
DN128616_c10_g13_i2	DOWN	-	-	-	KMS91954.1:hypothetical protein BVRB_033440,	gi|513798735|gb|KF150395.1|:Janthinobacterium lividum	A0A061S8J3|A0A061S8J3_9CHLO:Uncharacterized

geneid	trend	GO	Pathway	TF	NR	NT	Uniprot
					partial [Beta vulgaris subsp. vulgaris]	strain JN111 16S ribosomal RNA gene, partial sequence	protein;OS=Tetraselmis sp. GSL018.;
DN116255_c4_g9_i7	DOWN	[GO:0005524:ATP binding][GO:0004672:protein kinase activity]	-	OGLUM05G28390.1:Oryza glumaepatula|HB-other|HB-other family protein	XP_010694749.1:PREDICTED: mitogenactivated protein kinase 19 [Beta vulgaris subsp. vulgaris] >KMS98147.1 hypothetical protein BVRB_4g095340 isoform A [Beta vulgaris subsp. vulgaris]	gi|478164232|gb|GAHK01012371.1|:TSA: Jatropha curcas CL5229.Contig2_JC-CK_1A transcribed RNA sequence	A0A0J8BDV5|A0A0J8BDV5_BETVU: Uncharacterized protein;OS=Beta vulgaris subsp. vulgaris.;|GO:0005524,GO:0004672,
DN173112_c0_g1_i1	DOWN	[GO:0016021:integral component of membrane]	-	-	EYU28320.1:hypothetical protein MIMGU_mgv1a004005mg [Erythranthe guttata]	gi|255550408|ref|XM_002516209.1|:Ricinus communis conserved hypothetical protein, mRNA	A0A022QK39|A0A022QK39_ERYGU: Uncharacterized protein;OS=Erythranthe guttata;|GO:0016021,

geneid	trend	GO	Pathway	TF	NR	NT	Uniprot
DN126934_c0_g1_i1	DOWN	[GO:0043565:sequence-specific DNA binding][GO:0003700:DNA-binding transcription factor activity]	-	AHYPO_017174-RA:Amaranthus hypochondriacus|WRKY|WRKY family protein	XP_021839232.1:probable WRKY transcription factor 15 [Spinacia oleracea]	gi|511316844|gb|GAKE01016033.1|:TSA: Thlaspi arvense Contig16281 transcribed RNA sequence	A0A0K9QLS3|A0A0K9QLS3_SPIOL:Uncharacterized protein;OS=Spinacia oleracea;|GO:0043565,GO:0003700,
DN124690_c1_g3_i6	DOWN	[GO:0004108:citrate (Si)-synthase activity][GO:0006101:citrate metabolic process][GO:0006099:tricarboxylic acid cycle]	[ko00020:Citrate cycle (TCA cycle)][ko00630:Glyoxylate and dicarboxylate metabolism]	RrC6748_p2:Raphanus raphanistrum|MYB_related|MYB_related family protein	XP_010691175.1:PREDICTED: citrate synthase, mitochondrial isoform X1 [Beta vulgaris subsp. vulgaris]	gi|1556379|emb|X84228.1|:B.vulgaris mRNA for citrate synthase	A0A0J8BI86|A0A0J8BI86_BETVU:Citrate synthase;OS=Beta vulgaris subsp. vulgaris.;|GO:0004108,|GO:0006101,GO:0006099,
DN117220_c1_g10_i2	DOWN	-	-	-	XP_021640823.1:uncharacterized protein LOC110635701 isoform X2 [Hevea brasiliensis]	-	B9SDT5|B9SDT5_RICCO:Putative uncharacterized protein;OS=Ricinus communis;
DN128542_c0_g1_i6	DOWN	-	-	-	XP_010682959.1:PREDICTED: uncharacterized protein LOC104897720 isoform X1 [Beta	-	A0A0J8BZA1|A0A0J8BZA1_BETVU:Uncharacterized protein;OS=Beta vulgaris subsp. vulgaris.;

geneid	trend	GO	Pathway	TF	NR	NT	Uniprot
					vulgaris subsp. vulgaris]		
DN119061_c2_g1_i4	DOWN	[GO:0046872:metal ion binding][GO:0004722:protein serine/threonine phosphatase activity]	[ko04016:MAPK signaling pathway - plant][ko04075:Plant hormone signal transduction][ko04931:Insulin resistance]	KZV22762.1:Dorcoceras hygrometricum|CAMTA|CAMTA family protein	XP_021729058.1:probable protein phosphatase 2C 8 [Chenopodium quinoa]	-	A0A0K9QQG8|A0A0K9QQG8_SPIOL: Uncharacterized protein;OS=Spinacia oleracea;|GO:0046872,GO:0004722,

geneid	trend	GO	Pathway	TF	NR	NT	Uniprot
DN115243_c1_g1_i4	DOWN	[GO:0046872:metal ion binding][GO:0004721:phosphoprotein phosphatase activity]	[ko03015:mRNA surveillance pathway][ko04022:cGMP-PKG signaling pathway][ko04024:cAMP signaling pathway][ko04113:Meiosis - yeast][ko04114:Oocyte meiosis][ko04218:Cellular senescence][ko04261:Adrenergic signaling in cardiomyocytes][ko04270:Vascular smooth muscle contraction][ko04390:Hippo signaling pathway][ko04510:Focal adhesion][ko04611:Platelet activation][ko04720:Long-term potentiation][ko04728:Dopaminergic synapse][ko04750:Inflammatory mediator regulation of TRP	Jcr4S06752.20:Jatropha curcas|FAR1|FAR1 family protein	XP_021742589.1:serine/threonineprotein phosphatase PP1 isozyme 9like [Chenopodium quinoa]	gi|209879997|ref|XM_002141403.1|:Cryptosporidium muris RN66 serine/threonine protein phosphatase, putative, mRNA	A0A0K9RCG4|A0A0K9RCG4_SPIOL:Serine/threonine-protein phosphatase;OS=Spinacia oleracea;|GO:0046872,GO:0004721,

geneid	trend	GO	Pathway	TF	NR	NT	Uniprot
DN126775_c2_g1_i1	DOWN	[GO:0005737:cytoplasm][GO:0045548:phenylalanine ammonia-lyase activity][GO:0009800:cinnamic acid biosynthetic process][GO:0006559:L-phenylalanine catabolic process]	[ko00360:Phenylalanine metabolism][ko00940:Phenylpropanoid biosynthesis]	-	XP_010684079.1:PREDICTED: phenylalanine ammonialyase [Beta vulgaris subsp. vulgaris] >CAH17686.1 phenylalanine ammonia lyase [Beta vulgaris] >KMT06263.1 hypothetical protein BVRB_7g161880 [Beta vulgaris subsp. vulgaris]	gi|255556975|ref|XM_002519475.1|:Ricinus communis Phenylalanine ammonia-lyase, putative, mRNA	Q5QRU8|Q5QRU8_BETVU:Phenylalanine ammonia-lyase;OS=Beta vulgaris;|GO:0005737,|GO:0045548,|GO:0009800,GO:0006559,
DN128813_c3_g5_i2	DOWN	[GO:0046872:metal ion binding][GO:0016491:oxidoreductase activity]	-	Zpz_sc00083.1.g00290.1.am.mk:Zoysia pacifica|Dof|Dof family protein	XP_021863126.1:1aminocyclopropane1carboxylate oxidase homolog 1like [Spinacia oleracea]	-	A0A0K9QYK7|A0A0K9QYK7_SPIOL: Uncharacterized protein;OS=Spinacia oleracea;|GO:0046872,GO:0016491,

geneid	trend	GO	Pathway	TF	NR	NT	Uniprot
DN112369_c0_g2_i1	DOWN	[GO:0016021:integral component of membrane]	-	-	XP_021851037.1:transmembrane protein 136like [Spinacia oleracea]	-	A0A0J8FYL0|A0A0J8FYL0_BETVU:Uncharacterized protein;OS=Beta vulgaris subsp. vulgaris.;|GO:0016021,
DN115944_c0_g2_i1	DOWN	-	-	-	XP_021624061.1:polygalacturonase inhibitorlike [Manihot esculenta] >OAY40556.1 hypothetical protein MANES_09G031500 [Manihot esculenta]	-	A0A0K9RAH3|A0A0K9RAH3_SPIOL: Uncharacterized protein;OS=Spinacia oleracea;
DN128109_c1_g5_i2	DOWN	[GO:0016021:integral component of membrane][GO:0005524:ATP binding][GO:0016887:ATPase activity][GO:0006810:transport]	-	Rsa1.0_03996.1_g00001.1:Raphanus sativus|MYB_related|MYB_related family protein	XP_021746286.1:pleiotropic drug resistance protein 1like [Chenopodium quinoa]	gi|375273924|gb|JQ292813.1|:Petunia x hybrida ABCG/PDR subfamily ABC protein (PDR1) mRNA, complete cds	A0A0K9QNU0|A0A0K9QNU0_SPIOL: Uncharacterized protein;OS=Spinacia oleracea;|GO:0016021,|GO:0005524,GO:0016887,|GO:0006810,

geneid	trend	GO	Pathway	TF	NR	NT	Uniprot
DN122931_c1_g1_i3	DOWN	[GO:0005634:nucleus][GO:0003677:DNA binding][GO:0009734:auxin-activated signaling pathway][GO:0006355:regulation of transcription, DNA-templated][GO:0006351:transcription, DNA-templated]	[ko04075:Plant hormone signal transduction]	Bv1_002880_qzmp.t1:Beta vulgaris|ARF|ARF family protein	XP_021748876.1:auxin response factor 18like [Chenopodium quinoa] >XP_021748877.1 auxin response factor 18like [Chenopodium quinoa]	gi|521718421|gb|GAIA01011585.1|:TSA: Salicornia europaea Unigene12200_Se200S transcribed RNA sequence	A0A0J8D2B4|A0A0J8D2B4_BETVU:Auxin response factor;OS=Beta vulgaris subsp. vulgaris.;|GO:0005634,|GO:0003677,|GO:0009734,GO:0006355,GO:0006351,
DN119880_c0_g6_i1	DOWN	[GO:0003993:acid phosphatase activity]	[ko00740:Riboflavin metabolism][ko04142:Lysosome][ko04380:Osteoclast differentiation][ko05323:Rheumatoid arthritis]	GRMZM2G351232_P01:Zea mays|E2F/DP|E2F/DP family protein	XP_010674927.1:PREDICTED: purple acid phosphatase 3 [Beta vulgaris subsp. vulgaris] >KMT13491.1 hypothetical protein BVRB_4g082700 [Beta vulgaris subsp. vulgaris]	gi|449504799|ref|XM_004162250.1|:PREDICTED: Cucumis sativus purple acid phosphatase 17-like (LOC101228129), mRNA	A0A0J8FCC7|A0A0J8FCC7_BETVU:Uncharacterized protein;OS=Beta vulgaris subsp. vulgaris.;|GO:0003993,

geneid	trend	GO	Pathway	TF	NR	NT	Uniprot
DN126236_c2_g2_i14	DOWN	-	[ko04150:mTOR signaling pathway][ko04152: AMPK signaling pathway]	-	XP_010675860.1:PREDICTED: putative MO25like protein At5g47540 [Beta vulgaris subsp. vulgaris] >KMT12889.1 hypothetical protein BVRB_4g089790 isoform A [Beta vulgaris subsp. vulgaris]	gi|359483455|ref|XM_002267625.2|:PREDICTED: Vitis vinifera putative MO25-like protein At5g47540 (LOC100252109), mRNA	A0A0J8CLG7|A0A0J8CLG7_BETVU:Uncharacterized protein;OS=Beta vulgaris subsp. vulgaris.;
DN108208_c0_g1_i2	DOWN	[GO:0016788:hydrolase activity, acting on ester bonds]	-	KN538722.1_FGP037:Oryza longistaminata|HB-PHD|HB-PHD family protein	XP_021720400.1:GDSL esterase/lipase 2like [Chenopodium quinoa]	-	A0A0J8BXN2|A0A0J8BXN2_BETVU: Uncharacterized protein;OS=Beta vulgaris subsp. vulgaris.;|GO:0016788,

geneid	trend	GO	Pathway	TF	NR	NT	Uniprot
DN123590_c1_g2_i10	DOWN	[GO:0016021:integral component of membrane][GO:0005524:ATP binding][GO:0004672:protein kinase activity]	-	Rsa1.0_03766.1_g00001.1:Raphanus sativus|G2-like|G2-like family protein	XP_010689691.1:PREDICTED: protein STRUBBELIGRECEPTOR FAMILY 3 isoform X1 [Beta vulgaris subsp. vulgaris] >XP_010689692.1 PREDICTED: protein STRUBBELIGRECEPTOR FAMILY 3 isoform X1 [Beta vulgaris subsp. vulgaris] >XP_010689693.1 PREDICTED: protein STRUBBELIGRECEPTOR FAMILY 3 isoform X1 [Beta vulgaris subsp. vulgaris] >XP_019107272.1 PREDICTED: protein STRUBBELIGRECEPTOR FAMILY 3 isoform X1 [Beta vulgaris subsp. vulgaris]	gi|521530802|gb|GAHZ01008055.1|:TSA: Salicornia europaea Unigene8393_SeCKS transcribed RNA sequence	A0A0J8BLK9|A0A0J8BLK9_BETVU:Uncharacterized protein;OS=Beta vulgaris subsp. vulgaris.;|GO:0016021,|GO:0005524,GO:0004672,

geneid	trend	GO	Pathway	TF	NR	NT	Uniprot
DN117879_c0_g2_i7	DOWN	-	-	-	XP_010690907.1:PREDICTED: BURP domain protein USPL1like [Beta vulgaris subsp. vulgaris]	-	A0A0J8BGC5|A0A0J8BGC5_BETVU: Uncharacterized protein;OS=Beta vulgaris subsp. vulgaris.;
DN118150_c2_g7_i2	DOWN	[GO:0005737:cytoplasm][GO:0003991:acetylglutamate kinase activity][GO:0006526:arginine biosynthetic process]	[ko00220:Arginine biosynthesis]	-	XP_021717493.1:uncharacterized protein LOC110685313 [Chenopodium quinoa] >XP_021717494.1 uncharacterized protein LOC110685313 [Chenopodium quinoa] >XP_021717541.1 uncharacterized protein LOC110685341 [Chenopodium quinoa] >XP_021717542.1 uncharacterized	-	A0A0K9QMN0|A0A0K9QMN0_SPIOL:Uncharacterized protein;OS=Spinacia oleracea;|GO:0005737,|GO:0003991,|GO:0006526,

geneid	trend	GO	Pathway	TF	NR	NT	Uniprot
					protein LOC110685341 [Chenopodium quinoa]		
DN122536_c2_g1_i2	DOWN	-	-	-	-	-	-
DN127808_c2_g1_i1	DOWN	[GO:0016021:integral component of membrane]	-	-	XP_010693833.1:PREDICTED: uncharacterized protein LOC104906731 [Beta vulgaris subsp. vulgaris]	-	A0A0K9S2Z0|A0A0K9S2Z0_SPIOL:Uncharacterized protein;OS=Spinacia oleracea;|GO:0016021,
DN121539_c1_g1_i5	DOWN	-	-	-	-	-	-

geneid	trend	GO	Pathway	TF	NR	NT	Uniprot
DN120254_c0_g2_i1	DOWN	[GO:0016021:integral component of membrane][GO:0005524:ATP binding][GO:0030247:polysaccharide binding][GO:0004672:protein kinase activity]	[ko04064:NF-kappa B signaling pathway][ko04620:Toll-like receptor signaling pathway][ko04621:NOD-like receptor signaling pathway][ko04624:Toll and Imd signaling pathway][ko04722:Neurotrophin signaling pathway][ko05133:Pertussis][ko05140:Leishmaniasis][ko05142:Chagas disease (American trypanosomiasis)][ko05145:Toxoplasmosis][ko05152:Tuberculosis][ko05162:Measles][ko05164:Influenza A]	AA10G00404:Aethionema arabicum|WRKY|WRKY family protein	XP_021734429.1:wallassociated receptor kinaselike 1 [Chenopodium quinoa]	gi|147780652|emb|AM428423.2|:Vitis vinifera contig VV78X026305.4, whole genome shotgun sequence	A0A0K9S415|A0A0K9S415_SPIOL:Uncharacterized protein;OS=Spinacia oleracea;|GO:0016021,|GO:0005524,GO:0030247,GO:0004672,

geneid	trend	GO	Pathway	TF	NR	NT	Uniprot
DN119885_c1_g4_i2	DOWN	[GO:0016021:integral component of membrane][GO:0005215:transporter activity]	-	MDP0000288192:Malus domestica|HB-other|HB-other family protein	XP_021755823.1:glucose6phosphate/phosphate translocator 2, chloroplasticlike [Chenopodium quinoa]	gi|9295276|gb|AF223360.1|:Mesembryanthemum crystallinum glucose-6P/phosphate translocator precursor (GPT) mRNA, complete cds	A0A0K9QG79|A0A0K9QG79_SPIOL:Uncharacterized protein;OS=Spinacia oleracea;|GO:0016021,|GO:0005215,
DN128648_c0_g3_i1	DOWN	[GO:0016442:RISC complex][GO:0003712:transcription coregulator activity][GO:0035194:posttranscriptional gene silencing by RNA]	[ko05169:Epstein-Barr virus infection][ko05203:Viral carcinogenesis]	-	XP_021852255.1:ribonuclease TUDOR 2like [Spinacia oleracea] >KNA18582.1 hypothetical protein SOVF_069380 [Spinacia oleracea]	-	A0A0K9RI83|A0A0K9RI83_SPIOL:Uncharacterized protein;OS=Spinacia oleracea;|GO:0016442,|GO:0003712,|GO:0035194,

geneid	trend	GO	Pathway	TF	NR	NT	Uniprot
DN119939_c0_g1_i1	DOWN	[GO:0005618:cell wall][GO:0005576:extracellular region][GO:0016020:membrane][GO:0004252:serine-type endopeptidase activity]	-	Ote100132910021:Ocimum tenuiflorum|LSD|LSD family protein	XP_021852541.1:subtilisinlike protease SBT2.5 [Spinacia oleracea] >XP_021852542.1 subtilisinlike protease SBT2.5 [Spinacia oleracea] >XP_021852543.1 subtilisinlike protease SBT2.5 [Spinacia oleracea] >XP_021852544.1 subtilisinlike protease SBT2.5 [Spinacia oleracea]	gi|225470677|ref|XM_002269750.1|:PREDICTED: Vitis vinifera subtilisin-like protease (LOC100245233), mRNA	A0A0K9QLX5|A0A0K9QLX5_SPIOL: Uncharacterized protein;OS=Spinacia oleracea;|GO:0005618,GO:0005576,GO:0016020,|GO:0004252,
DN127770_c2_g2_i4	DOWN	-	-	Zjn_sc00002.1.g13080.1.am.mkhc:Zoysia japonica|bHLH|bHLH family protein	XP_010683226.1:PREDICTED: Fbox/kelchrepeat protein SKIP11 [Beta vulgaris subsp. vulgaris] >KMT07101.1 hypothetical protein BVRB_6g154280	-	A0A0J8EVA7|A0A0J8EVA7_BETVU: Uncharacterized protein;OS=Beta vulgaris subsp. vulgaris.;

geneid	trend	GO	Pathway	TF	NR	NT	Uniprot
					[Beta vulgaris subsp. vulgaris]		
DN125339_c2_g4_i3	DOWN	[GO:0009507:chloroplast][GO:0005739:mitochondrion][GO:0005524:ATP binding][GO:0050897:cobalt ion binding][GO:0004471:malate dehydrogenase (decarboxylating) (NAD+) activity][GO:0051287:NAD binding][GO:0008270:zinc ion binding][GO:0006108:malate metabolic process]	[ko00620:Pyruvate metabolism][ko00710:Carbon fixation in photosynthetic organisms]	DCAR_007584:Daucus carota|MYB_related|MYB_related family protein	XP_010671885.1:PREDICTED: NADdependent malic enzyme 59 kDa isoform, mitochondrial [Beta vulgaris subsp. vulgaris] >KMT20599.1 hypothetical protein BVRB_1g005810 [Beta vulgaris subsp. vulgaris]	gi|478169587|gb|GAHK01007016.1|:TSA: Jatropha curcas CL2551.Contig1_JC-CK_1A transcribed RNA sequence	A0A0J8D8C5|A0A0J8D8C5_BETVU:Malic enzyme;OS=Beta vulgaris subsp. vulgaris.;|GO:0009507,GO:0005739,|GO:0005524,GO:0050897,GO:0004471,GO:0051287,GO:0008270,|GO:0006108,

geneid	trend	GO	Pathway	TF	NR	NT	Uniprot
DN123689_c2_g2_i6	DOWN	[GO:0016491:oxidoreductase activity]	-	678321642:Utricularia gibba|bHLH|bHLH family protein	XP_010677556.1:PREDICTED: aldoketo reductase family 4 member C9 [Beta vulgaris subsp. vulgaris] >KMT11551.1 hypothetical protein BVRB_5g108680 [Beta vulgaris subsp. vulgaris]	gi|502146829|ref|XM_004506570.1|:PREDICTED: Cicer arietinum aldo-keto reductase family 4 member C9-like (LOC101510508), transcript variant X2, mRNA	A0A0J8F6H4|A0A0J8F6H4_BETVU:Uncharacterized protein;OS=Beta vulgaris subsp. vulgaris.;|GO:0016491,
DN118477_c0_g3_i3	DOWN	[GO:0046872:metal ion binding][GO:0016702:oxidoreductase activity, acting on single donors with incorporation of molecular oxygen, incorporation of two atoms of oxygen][GO:0006633:fatty acid biosynthetic process][GO:0031408:oxylipin biosynthetic process]	[ko00591:Linoleic acid metabolism][ko00592:alpha-Linolenic acid metabolism]	-	XP_021772004.1:linoleate 13Slipoxygenase 21, chloroplasticlike [Chenopodium quinoa]	gi|225450226|ref|XM_002263818.1|:PREDICTED: Vitis vinifera linoleate 13S-lipoxygenase 2-1, chloroplastic-like (LOC100257867), mRNA	E3WHD5|E3WHD5_9ROSI:Lipoxygenase;OS=Vitis hybrid cultivar.;|GO:0046872,GO:0016702,|GO:0006633,GO:0031408,

geneid	trend	GO	Pathway	TF	NR	NT	Uniprot
DN118440_c1_g1_i1	DOWN	[GO:0016702:oxidoreductase activity, acting on single donors with incorporation of molecular oxygen, incorporation of two atoms of oxygen]	[ko00906:Carotenoid biosynthesis]	-	XP_021867001.1:9cisepoxycarotenoid dioxygenase NCED1, chloroplastic [Spinacia oleracea]	gi|256561142|gb|GQ395772.1|:Caragana korshinskii 9-cis-epoxycarotenoid dioxygenase (NCED1) mRNA, complete cds	A0A0K9QZ39|A0A0K9QZ39_SPIOL:Uncharacterized protein;OS=Spinacia oleracea;|GO:0016702,
DN106568_c0_g1_i2	DOWN	-	-	-	-	-	-
DN114186_c0_g1_i1	DOWN	-	-	-	XP_021748895.1:elicitorresponsive protein 3like isoform X2 [Chenopodium quinoa]	-	A0A0J8D8C0|A0A0J8D8C0_BETVU:Uncharacterized protein;OS=Beta vulgaris subsp. vulgaris.;
DN119940_c0_g1_i3	DOWN	[GO:0003676:nucleic acid binding][GO:0000166:nucleotide binding]	-	-	XP_021766895.1:polyadenylatebinding protein RBP45like [Chenopodium quinoa]	gi|478163876|gb|GAHK01012727.1|:TSA: Jatropha curcas Unigene178_JC-CK_1A transcribed RNA sequence	Q84LL7|Q84LL7_BETVU:Salt tolerance protein 6;OS=Beta vulgaris;|GO:0003676,GO:0000166,

geneid	trend	GO	Pathway	TF	NR	NT	Uniprot
DN122958_c0_g1_i1	DOWN	[GO:0016021:integral component of membrane][GO:0005524:ATP binding][GO:0004672:protein kinase activity]	[ko04064:NF-kappa B signaling pathway][ko04620:Toll-like receptor signaling pathway][ko04621:NOD-like receptor signaling pathway][ko04624:Toll and Imd signaling pathway][ko04722:Neurotrophin signaling pathway][ko05133:Pertussis][ko05140:Leishmaniasis][ko05142:Chagas disease (American trypanosomiasis)][ko05145:Toxoplasmosis][ko05152:Tuberculosis][ko05162:Measles][ko05164:Influenza A]	312791:Arabidopsis lyrata|bHLH|bHLH family protein	KNA11482.1:hypothetical protein SOVF_134790 [Spinacia oleracea]	gi|521513182|gb|GAHZ01017738.1|:TSA: Salicornia europaea Unigene18679_SeCKS transcribed RNA sequence	A0A0K9QW24|A0A0K9QW24_SPIOL: Uncharacterized protein;OS=Spinacia oleracea;|GO:0016021,|GO:0005524,GO:0004672,

geneid	trend	GO	Pathway	TF	NR	NT	Uniprot
DN122888_c0_g1_i1	DOWN	[GO:0016021:integral component of membrane][GO:0005886:plasma membrane][GO:0015591:D-ribose transmembrane transporter activity][GO:0015148:D-xylose transmembrane transporter activity][GO:0005354:galactose transmembrane transporter activity][GO:0005355:glucose transmembrane transporter activity][GO:0015168:glycerol transmembrane transporter activity][GO:0015575:mannitol transmembrane transporter activity][GO:0005365:myo-inositol transmembrane transporter activity][GO:0015576:sorbitol transmembrane transporter activity][GO:0005351:carbohydrate:proton symporter activity][GO:0010311:lateral root formation]	-	-	XP_021731626.1:polyol transporter 5like [Chenopodium quinoa]	gi|1778092|gb|U64902.1|BVU64902:Beta vulgaris putative sugar transporter (BvcDNA-205) mRNA, complete cds	P93075|P93075_BETVU:BvcDNA-205 protein;OS=Beta vulgaris subsp. vulgaris.;|GO:0016021,GO:0005886,|GO:0015591,GO:0015148,GO:0005354,GO:0005355,GO:0015168,GO:0015575,GO:0005365,GO:0015576,GO:0005351,|GO:0010311,

geneid	trend	GO	Pathway	TF	NR	NT	Uniprot
DN126236_c2_g2_i8	DOWN	-	[ko04150:mTOR signaling pathway][ko04152:AMPK signaling pathway]	-	XP_010675860.1:PREDICTED: putative MO25like protein At5g47540 [Beta vulgaris subsp. vulgaris] >KMT12889.1 hypothetical protein BVRB_4g089790 isoform A [Beta vulgaris subsp. vulgaris]	gi|359483455|ref|XM_002267625.2|:PREDICTED: Vitis vinifera putative MO25-like protein At5g47540 (LOC100252109), mRNA	A0A0J8CLG7|A0A0J8CLG7_BETVU:Uncharacterized protein;OS=Beta vulgaris subsp. vulgaris.;
DN124018_c1_g3_i2	DOWN	-	-	Achn202071:Actinidia chinensis|MYB_related|MYB_related family protein	XP_021733635.1:protein KINESIN LIGHT CHAINRELATED 3like [Chenopodium quinoa]	gi|521570717|gb|GAIA01073328.1|:TSA: Salicornia europaea Unigene89146_Se200S transcribed RNA sequence	A0A0J8CEL7|A0A0J8CEL7_BETVU:Uncharacterized protein;OS=Beta vulgaris subsp. vulgaris.;

geneid	trend	GO	Pathway	TF	NR	NT	Uniprot
DN122899_c1_g3_i2	DOWN	[GO:0005783:endoplasmic reticulum][GO:0016020:membrane][GO:0016831:carboxy-lyase activity][GO:0030170:pyridoxal phosphate binding][GO:0019752:carboxylic acid metabolic process][GO:0030149:sphingolipid catabolic process]	[ko00600:Sphingolipid metabolism][ko04071:Sphingolipid signaling pathway]	-	XP_010682718.1:PREDICTED: sphingosine1phosphate lyase [Beta vulgaris subsp. vulgaris] >KMT07419.1 hypothetical protein BVRB_6g150440 [Beta vulgaris subsp. vulgaris]	gi|224075540|ref|XM_002304638.1|:Populus trichocarpa predicted protein, mRNA	A0A0J8C5A3|A0A0J8C5A3_BETVU:Uncharacterized protein;OS=Beta vulgaris subsp. vulgaris.;|GO:0005783,GO:0016020,|GO:0016831,GO:0030170,|GO:0019752,GO:0030149,
DN120632_c0_g1_i2	DOWN	[GO:0030060:L-malate dehydrogenase activity][GO:0005975:carbohydrate metabolic process][GO:0006108:malate metabolic process]	[ko00020:Citrate cycle (TCA cycle)][ko00270:Cysteine and methionine metabolism][ko00620:Pyruvate metabolism][ko00630:Glyoxylate and dicarboxylate metabolism][ko00710:Carbon fixation in photosynthetic organisms]	-	XP_021838684.1:malate dehydrogenase, chloroplasticlike [Spinacia oleracea] >KNA10786.1 hypothetical protein SOVF_140870 [Spinacia oleracea]	gi|521746976|gb|GAIA01002375.1|:TSA: Salicornia europaea Unigene2428_Se200S transcribed RNA sequence	A0A0K9QWA0|A0A0K9QWA0_SPIOL:Uncharacterized protein;OS=Spinacia oleracea;|GO:0030060,|GO:0005975,GO:0006108,

geneid	trend	GO	Pathway	TF	NR	NT	Uniprot
DN110571_c0_g3_i2	DOWN	[GO:0008270:zinc ion binding]	-	-	XP_010682265.1:PREDICTED: probable E3 ubiquitinprotein ligase XERICO [Beta vulgaris subsp. vulgaris] >KMT07753.1 hypothetical protein BVRB_6g146750 [Beta vulgaris subsp. vulgaris]	-	A0A0J8C6U0|A0A0J8C6U0_BETVU:Uncharacterized protein;OS=Beta vulgaris subsp. vulgaris.;|GO:0008270,

geneid	trend	GO	Pathway	TF	NR	NT	Uniprot
DN112623_c0_g1_i2	DOWN	-	[ko00270:Cysteine and methionine metabolism]	-	AOR52481.1:Sadenosylmethionine synthase, partial [Cycas debaoensis] >AOR52482.1 Sadenosylmethionine synthase, partial [Cycas debaoensis] >AOR52483.1 Sadenosylmethionine synthase, partial [Cycas debaoensis] >AOR52484.1 Sadenosylmethionine synthase, partial [Cycas debaoensis] >AOR52486.1 Sadenosylmethionine synthase, partial [Cycas debaoensis] >AOR52487.1 Sadenosylmethionine synthase, partial [Cycas debaoensis] >AOR52488.1 Sadenosylmethionine synthase, partial [Cycas debaoensis] >AOR52489.1 Sadenosylmethionine synthase, partial	gi|349730994|emb|FQ389202.1|:Vitis vinifera clone SS0AEB20YI17	A0A1C9U7T7|A0A1C9U7T7_9SPER:S-adenosylmethionine synthase;OS=Cycas debaoensis.;

geneid	trend	GO	Pathway	TF	NR	NT	Uniprot
DN122677_c6_g1_i8	DOWN	[GO:0016021:integral component of membrane][GO:0055085:transmembrane transport]	-	-	XP_010680909.1:PREDICTED: uncharacterized protein LOC104895957 isoform X2 [Beta vulgaris subsp. vulgaris]	-	A0A0J8C4M4|A0A0J8C4M4_BETVU: Uncharacterized protein;OS=Beta vulgaris subsp. vulgaris.;|GO:0016021,|GO:0055085,
DN124335_c3_g2_i10	DOWN	-	-	-	-	-	-
DN116735_c0_g2_i6	DOWN	-	-	SMil_00007993-RA_Salv:Salvia miltiorrhiza|ERF|ERF family protein	XP_021855423.1:uncharacterized protein LOC110794773 [Spinacia oleracea] >KNA05846.1 hypothetical protein SOVF_186520 [Spinacia oleracea]	gi|403043685|ref|NM_001254417.2|:Glycine max uncharacterized LOC100791242 (LOC100791242), mRNA	A0A0K9QH01|A0A0K9QH01_SPIOL:Uncharacterized protein;OS=Spinacia oleracea;

geneid	trend	GO	Pathway	TF	NR	NT	Uniprot
DN124522_c0_g8_i5	DOWN	[GO:0005737:cytoplasm][GO:0005634:nucleus][GO:0005524:ATP binding][GO:0004127:cytidylate kinase activity][GO:0009041:uridylate kinase activity][GO:0006207:'de novo' pyrimidine nucleobase biosynthetic process][GO:0006221:pyrimidine nucleotide biosynthetic process]	[ko00240:Pyrimidine metabolism]	-	XP_021835169.1:UMPCMP kinase 3 [Spinacia oleracea] >KNA12696.1 hypothetical protein SOVF_123580 [Spinacia oleracea]	gi|502155650|ref|XM_004510087.1|:PREDICTED: Cicer arietinum UMP/CMP kinase-like (LOC101513002), transcript variant X2, mRNA	A0A0K9QZL1|A0A0K9QZL1_SPIOL:UMP-CMP kinase;OS=Spinacia oleracea;|GO:0005737,GO:0005634,|GO:0005524,GO:0004127,GO:0009041,|GO:0006207,GO:0006221,
DN123380_c6_g1_i7	DOWN	-	-	-	XP_010679062.1:PREDICTED: late embryogenesis abundant protein 31 [Beta vulgaris subsp. vulgaris] >KMT10181.1 hypothetical protein BVRB_5g119420 [Beta vulgaris subsp. vulgaris]	-	A0A0J8F381|A0A0J8F381_BETVU:Uncharacterized protein;OS=Beta vulgaris subsp. vulgaris.;

geneid	trend	GO	Pathway	TF	NR	NT	Uniprot
DN127128_c5_g4_i1	DOWN	[GO:0016021:integral component of membrane][GO:0034256:chlorophyll(ide) b reductase activity][GO:0015996:chlorophyll catabolic process][GO:0010304:PSII associated light-harvesting complex II catabolic process]	[ko00860:Porphyrin and chlorophyll metabolism]	OPUNC03G25490.1:Oryza punctata|G2-like|G2-like family protein	XP_021723654.1:probable chlorophyll(ide) b reductase NYC1, chloroplastic [Chenopodium quinoa]	gi|356520528|ref|XM_003528866.1|:PREDICTED: Glycine max probable chlorophyll(ide) b reductase NYC1, chloroplastic-like (LOC100784978), mRNA	A0A0J8EJD4|A0A0J8EJD4_BETVU:Uncharacterized protein;OS=Beta vulgaris subsp. vulgaris.;|GO:0016021,|GO:0034256,|GO:0015996,GO:0010304,
DN127501_c0_g1_i2	DOWN	[GO:0016021:integral component of membrane]	[ko04152:AMPK signaling pathway][ko04211:Longevity regulating pathway][ko04920:Adipocytokine signaling pathway][ko04932:Non-alcoholic fatty liver disease (NAFLD)]	MDP0000270344:Malus domestica|BES1|BES1 family protein	XP_010687835.1:PREDICTED: heptahelical transmembrane protein 4 isoform X1 [Beta vulgaris subsp. vulgaris] >KMT19091.1 hypothetical protein BVRB_1g014890 [Beta vulgaris subsp. vulgaris]	-	A0A0J8FTS7|A0A0J8FTS7_BETVU:Uncharacterized protein;OS=Beta vulgaris subsp. vulgaris.;|GO:0016021,
DN126159_c0_g1_i7	DOWN	[GO:0004555:alpha,alpha-trehalase activity][GO:0005991:trehalose metabolic process]	[ko00500:Starch and sucrose metabolism]	-	XP_021767878.1:probable trehalase [Chenopodium quinoa]	-	A0A0K9RUX3|A0A0K9RUX3_SPIOL:Trehalase;OS=Spinacia

geneid	trend	GO	Pathway	TF	NR	NT	Uniprot
							oleracea;|GO:0004555,|GO:0005991,
DN117979_c3_g2_i1	DOWN	-	-	-	XP_010681969.1:PREDICTED: uncharacterized protein At1g66480 [Beta vulgaris subsp. vulgaris]	-	A0A0J8C7N7|A0A0J8C7N7_BETVU:Uncharacterized protein;OS=Beta vulgaris subsp. vulgaris.;
DN128322_c3_g2_i5	DOWN	-	-	-	XP_010685291.1:PREDICTED: uncharacterized protein LOC104899734 [Beta vulgaris subsp. vulgaris] >KMT04807.1 hypothetical protein BVRB_7g169780 [Beta vulgaris subsp. vulgaris]	gi|356519837|ref|XM_003528528.1|:PREDICTED: Glycine max uncharacterized protein LOC100807956 (LOC100807956), mRNA	A0A0J8BUJ4|A0A0J8BUJ4_BETVU:Uncharacterized protein;OS=Beta vulgaris subsp. vulgaris.;

geneid	trend	GO	Pathway	TF	NR	NT	Uniprot
DN119629_c0_g1_i3	DOWN	-	[ko00520:Amino sugar and nucleotide sugar metabolism]	MDP0000247737:Malus domestica|FAR1|FAR1 family protein	XP_021716189.1:UDPglucuronic acid decarboxylase 5 [Chenopodium quinoa] >XP_021716190.1 UDPglucuronic acid decarboxylase 5 [Chenopodium quinoa] >XP_021716191.1 UDPglucuronic acid decarboxylase 5 [Chenopodium quinoa] >XP_021720901.1 UDPglucuronic acid decarboxylase 5 [Chenopodium quinoa] >XP_021720902.1 UDPglucuronic acid decarboxylase 5 [Chenopodium quinoa]	gi|521650419|gb|GAIA01042017.1|:TSA: Salicornia europaea Unigene47208_Se200S transcribed RNA sequence	A0A0K9S2V0|A0A0K9S2V0_SPIOL:Uncharacterized protein;OS=Spinacia oleracea;

geneid	trend	GO	Pathway	TF	NR	NT	Uniprot
					>XP_021720903.1 UDPglucuronic acid decarboxylase 5 [Chenopodium quinoa]		
DN120624_c0_g3_i9	DOWN	[GO:0016021:integral component of membrane][GO:0004190:aspartic-type endopeptidase activity]	-	mrna24179.1-v1.0-hybrid:Fragaria vesca|NAC|NAC family protein	KNA08065.1:hypothetical protein SOVF_165960 isoform B [Spinacia oleracea]	-	A0A0K9QLC9|A0A0K9QLC9_SPIOL: Uncharacterized protein;OS=Spinacia oleracea;|GO:0016021,|GO:0004190,

geneid	trend	GO	Pathway	TF	NR	NT	Uniprot
DN109818_c0_g1_i2	DOWN	-	-	-	-	-	-
DN117852_c1_g3_i2	DOWN	-	-	Zmw_sc00451.1.g00140.1:Zoysia matrella|M-type_MADS|M-type_MADS family protein	XP_021731141.1:uncharacterized protein LOC110698043 [Chenopodium quinoa]	-	A0A0J8C545|A0A0J8C545_BETVU:Uncharacterized protein;OS=Beta vulgaris subsp. vulgaris.;
DN124528_c1_g3_i4	DOWN	[GO:0005622:intracellular][GO:0004871:obsolete signal transducer activity][GO:0000160:phosphorelay signal transduction system]	[ko04075:Plant hormone signal transduction]	-	XP_010674982.2:PREDICTED: histidinecontaining phosphotransfer protein 1 isoform X1 [Beta vulgaris subsp. vulgaris] >XP_019104588.1 PREDICTED: histidinecontaining phosphotransfer protein 1 isoform X1 [Beta vulgaris subsp. vulgaris] >KMT13547.1 hypothetical protein BVRB_4g083230	gi|359477451|ref|XM_002278375.2|:PREDICTED: Vitis vinifera histidine-containing phosphotransfer protein 1-like, transcript variant 1 (LOC100251055), mRNA	A0A0J8CJ27|A0A0J8CJ27_BETVU:Uncharacterized protein;OS=Beta vulgaris subsp. vulgaris.;|GO:0005622,|GO:0004871,|GO:0000160,

geneid	trend	GO	Pathway	TF	NR	NT	Uniprot
					isoform B [Beta vulgaris subsp. vulgaris]		
DN120657_c3_g1_i1	DOWN	-	-	-	-	-	-

geneid	trend	GO	Pathway	TF	NR	NT	Uniprot
DN116186_c2_g3_i5	DOWN	[GO:0005783:endoplasmic reticulum][GO:0016021:integral component of membrane][GO:0046923:ER retention sequence binding][GO:0006621:protein retention in ER lumen]	-	Peaxi162Scf01312g00064.1:Petunia axillaris|M-type_MADS|M-type_MADS family protein	OWM80917.1:hypothetical protein CDL15_Pgr006948 [Punica granatum]	gi|460370173|ref|XM_004230881.1|:PREDICTED: Solanum lycopersicum putative ER lumen protein retaining receptor C28H8.4-like (LOC101258414), mRNA	A0A0J8BJK7|A0A0J8BJK7_BETVU:Uncharacterized protein;OS=Beta vulgaris subsp. vulgaris.;|GO:0005783,GO:0016021,|GO:0046923,|GO:0006621,
DN125890_c1_g2_i3	DOWN	[GO:0016021:integral component of membrane][GO:0005506:iron ion binding][GO:0016491:oxidoreductase activity][GO:0006633:fatty acid biosynthetic process]	-	PSME_00012282-RA:Pseudotsuga menziesii|NAC|NAC family protein	XP_021731385.1:protein ECERIFERUM 3like [Chenopodium quinoa]	gi|521578491|gb|GAIA01069713.1|:TSA: Salicornia europaea Unigene83974_Se200S transcribed RNA sequence	A0A0J8BQ25|A0A0J8BQ25_BETVU:Uncharacterized protein;OS=Beta vulgaris subsp. vulgaris.;|GO:0016021,|GO:0005506,GO:0016491,|GO:0006633,

geneid	trend	GO	Pathway	TF	NR	NT	Uniprot
DN119092_c4_g1_i1	DOWN	[GO:0016758:transferase activity, transferring hexosyl groups][GO:0008152:metabolic process]	[ko00908:Zeatin biosynthesis]	Tp57577_TGAC_v2_mRNA33215:Trifolium pratense|bHLH|bHLH family protein	XP_010672886.1:PREDICTED: zeatin Oxylosyltransferase [Beta vulgaris subsp. vulgaris] >KMT15060.1 hypothetical protein BVRB_3g061820 [Beta vulgaris subsp. vulgaris]	gi|356537474|ref|XM_003537204.1|:PREDICTED: Glycine max zeatin O-glucosyltransferase-like (LOC100791957), mRNA	A0A0J8CMW1|A0A0J8CMW1_BETVU:Glycosyltransferase;OS=Beta vulgaris subsp. vulgaris.;|GO:0016758,|GO:0008152,
DN126592_c0_g1_i2	DOWN	[GO:0008171:O-methyltransferase activity]	[ko00940:Phenylpropanoid biosynthesis]	-	XP_021767231.1:caffeic acid 3Omethyltransferase like [Chenopodium quinoa]	gi|521498373|gb|GAHZ01032547.1|:TSA: Salicornia europaea Unigene35891_SeCKS transcribed RNA sequence	A0A0K9RKZ4|A0A0K9RKZ4_SPIOL:Uncharacterized protein;OS=Spinacia oleracea;|GO:0008171,
DN108548_c0_g1_i3	DOWN	[GO:0005737:cytoplasm][GO:0004349:glutamate 5-kinase activity][GO:0004350:glutamate-5-semialdehyde dehydrogenase activity][GO:0006561:proline biosynthetic process]	[ko00330:Arginine and proline metabolism]	mrna20854.1-v1.0-hybrid:Fragaria vesca|B3|B3 family protein	XP_010691515.1:PREDICTED: delta1pyrroline5carboxylate synthase isoform X1 [Beta vulgaris subsp. vulgaris] >KMT18404.1 hypothetical protein	gi|521665802|gb|GAIA01034637.1|:TSA: Salicornia europaea Unigene38340_Se200S transcribed RNA sequence	A0A0J8CXE2|A0A0J8CXE2_BETVU:Uncharacterized protein;OS=Beta vulgaris subsp. vulgaris.;|GO:0005737,|GO:0004349,GO:0004350,|GO:0006561,

geneid	trend	GO	Pathway	TF	NR	NT	Uniprot
					BVRB_2g025540 isoform A [Beta vulgaris subsp. vulgaris]		
DN122516_c1_g10_i1	DOWN	[GO:0043565:sequence-specific DNA binding][GO:0003700:DNA-binding transcription factor activity]	-	Bv3_050990_kqjn.t1:Beta vulgaris|bZIP|bZIP family protein	XP_010671337.1:PREDICTED: bZIP transcription factor 11 [Beta vulgaris subsp. vulgaris] >KMT16481.1 hypothetical protein BVRB_3g050990 [Beta vulgaris subsp. vulgaris]	gi|470106573|ref|XM_004289593.1|:PREDICTED: Fragaria vesca subsp. vesca uncharacterized LOC101313460 (LOC101313460), mRNA	A0A0J8FKP2|A0A0J8FKP2_BETVU:Uncharacterized protein;OS=Beta vulgaris subsp. vulgaris.;|GO:0043565,GO:0003700,
DN121601_c0_g1_i1	DOWN	[GO:0016021:integral component of membrane]	-	311286:Arabidopsis lyrata|MYB_related|MYB_related family protein	KMT20411.1:hypothetical protein BVRB_1g004070 [Beta vulgaris subsp. vulgaris]	-	A0A0J8FXM3|A0A0J8FXM3_BETVU:Uncharacterized protein;OS=Beta vulgaris subsp. vulgaris.;|GO:00160

geneid	trend	GO	Pathway	TF	NR	NT	Uniprot
							21,
DN127959_c2_g1_i2	DOWN	-	-	-	KMT15128.1:hypothetical protein BVRB_3g062460 [Beta vulgaris subsp. vulgaris]	gi|242388702|emb|FP100132.1|:Phyllostachys edulis cDNA clone: bphyem123g05, full insert sequence	A0A0J8CT63|A0A0J8CT63_BETVU:Uncharacterized protein;OS=Beta vulgaris subsp. vulgaris.;
DN128630_c8_g6_i1	DOWN	-	-	-	-	-	-
DN121470_c2_g1_i4	DOWN	[GO:0016021:integral component of membrane]	-	KHN40337.1:Glycine soja|HRT-like|HRT-like family protein	XP_021853522.1:NDR1/HIN1like protein 1 [Spinacia oleracea] >KNA06098.1 hypothetical protein SOVF_184230 [Spinacia oleracea]	-	A0A0K9QHF4|A0A0K9QHF4_SPIOL:Uncharacterized protein;OS=Spinacia oleracea;|GO:0016021,

geneid	trend	GO	Pathway	TF	NR	NT	Uniprot
DN122479_c1_g2_i3	DOWN	[GO:0008080:N-acetyltransferase activity]	-	-	XP_010688999.1:PREDICTED: Nacetyltransferase 9like protein [Beta vulgaris subsp. vulgaris] >KMT02416.1 hypothetical protein BVRB_9g204770 [Beta vulgaris subsp. vulgaris]	-	A0A0J8EHD4|A0A0J8EHD4_BETVU: Uncharacterized protein;OS=Beta vulgaris subsp. vulgaris.;|GO:0008080,
DN121585_c2_g1_i3	DOWN	-	-	-	-	-	-
DN109777_c0_g1_i1	DOWN	-	-	LOC_Os10g34884.1:Oryza sativa subsp. japonica|FAR1|FAR1 family protein	XP_010670661.1:PREDICTED: putative ripeningrelated protein 1 [Beta vulgaris subsp. vulgaris] >KMT20501.1 hypothetical protein BVRB_1g004870 [Beta vulgaris subsp. vulgaris]	-	A0A0J8FXY7|A0A0J8FXY7_BETVU:Uncharacterized protein;OS=Beta vulgaris subsp. vulgaris.;

geneid	trend	GO	Pathway	TF	NR	NT	Uniprot
DN128187_c6_g1_i9	DOWN	[GO:0016787:hydrolase activity]	-	Glyma.12G184400.1.p:Glycine max|bZIP|bZIP family protein	XP_008224614.1:PREDICTED: uncharacterized protein LOC103324341 [Prunus mume]	gi|224138621|ref|XM_002322824.1|:Populus trichocarpa predicted protein, mRNA	F6GTY2|F6GTY2_VITVI:Putative uncharacterized protein;OS=Vitis vinifera;GN=VIT_06s0004g03310 {ECO:0000313|EMBL:CCB43646.1};|GO:0016787,
DN127259_c2_g1_i5	DOWN	-	-	-	XP_021865553.1:glycinerich domaincontaining protein 1like isoform X3 [Spinacia oleracea] >KNA12464.1 hypothetical protein SOVF_125400 [Spinacia oleracea]	gi|521743119|gb|GAIA01004608.1|:TSA: Salicornia europaea Unigene4892_Se200S transcribed RNA sequence	A0A0K9QYW3|A0A0K9QYW3_SPIOL:Uncharacterized protein;OS=Spinacia oleracea;

geneid	trend	GO	Pathway	TF	NR	NT	Uniprot
DN123591_c2_g3_i3	DOWN	[GO:0006950:response to stress]	-	-	XP_010671709.1:PREDICTED: universal stress protein PHOS32 [Beta vulgaris subsp. vulgaris] >KMT16252.1 hypothetical protein BVRB_3g054060 [Beta vulgaris subsp. vulgaris]	-	A0A0K9QXP6|A0A0K9QXP6_SPIOL: Uncharacterized protein;OS=Spinacia oleracea;|GO:0006950,
DN121706_c3_g2_i2	DOWN	[GO:0005634:nucleus][GO:0003677: DNA binding][GO:0006355:regulation of transcription, DNA-templated]	-	Manes.02G018700.1.p:Manihot esculenta|TALE|TALE family protein	XP_021604201.1:homeobox protein BEL1 homolog [Manihot esculenta] >XP_021604202.1 homeobox protein BEL1 homolog [Manihot esculenta] >OAY56463.1 hypothetical protein MANES_02G018700 [Manihot esculenta]	gi|478171459|gb|GAHK01005144.1|:TSA: Jatropha curcas CL1736.Contig1_JC-CK_1A transcribed RNA sequence	V4VJ91|V4VJ91_9ROSI:Uncharacterized protein;OS=Citrus clementina.;|GO:0005634,|GO:0003677,|GO:0006355,

geneid	trend	GO	Pathway	TF	NR	NT	Uniprot
DN122309_c4_g2_i3	DOWN	[GO:0008168:methyltransferase activity]	-	-	KNA20158.1:hypothetical protein SOVF_054770 [Spinacia oleracea]	-	A0A0K9RKZ1|A0A0K9RKZ1_SPIOL: Uncharacterized protein;OS=Spinacia oleracea;|GO:0008168,
DN121001_c0_g2_i7	DOWN	[GO:0005509:calcium ion binding]	-	Itr_sc000795.1_g00005.1:Ipomoea trifida|ERF|ERF family protein	XP_010683627.1:PREDICTED: TLD domaincontaining protein 1 [Beta vulgaris subsp. vulgaris] >KMT06682.1 hypothetical protein BVRB_7g158460 [Beta vulgaris subsp. vulgaris]	-	A0A0J8BZM0|A0A0J8BZM0_BETVU: Uncharacterized protein;OS=Beta vulgaris subsp. vulgaris.;|GO:0005509,

geneid	trend	GO	Pathway	TF	NR	NT	Uniprot
DN129138_c7_g7_i1	DOWN	[GO:0005622:intracellular][GO:0008061:chitin binding][GO:0004568:chitinase activity][GO:0006040:amino sugar metabolic process][GO:0005975:carbohydrate metabolic process][GO:0016998:cell wall macromolecule catabolic process][GO:0006032:chitin catabolic process]	[ko00520:Amino sugar and nucleotide sugar metabolism]	-	XP_021657786.1:endochitinase PR4like [Hevea brasiliensis]	-	B9IQS9|B9IQS9_POPTR:Uncharacterized protein;OS=Populus trichocarpa;|GO:0005622,|GO:0008061,GO:0004568,|GO:0006040,GO:0005975,GO:0016998,GO:0006032,

geneid	trend	GO	Pathway	TF	NR	NT	Uniprot
DN122841_c4_g1_i6	DOWN	[GO:0016021:integral component of membrane][GO:0055085:transmembrane transport]	-	Jcr4S00206.70:Jatropha curcas|FAR1|FAR1 family protein	XP_010680628.1:PREDICTED: protein PINLIKES 7 isoform X1 [Beta vulgaris subsp. vulgaris] >XP_010680629.1 PREDICTED: protein PINLIKES 7 isoform X1 [Beta vulgaris subsp. vulgaris] >XP_019105728.1 PREDICTED: protein PINLIKES 7 isoform X1 [Beta vulgaris subsp. vulgaris] >KMT08875.1 hypothetical protein BVRB_6g135980 [Beta vulgaris subsp. vulgaris]	gi|297797790|ref|XM_002866734.1|:Arabidopsis lyrata subsp. lyrata predicted protein, mRNA	A0A0J8C515|A0A0J8C515_BETVU:Uncharacterized protein;OS=Beta vulgaris subsp. vulgaris.;|GO:0016021,|GO:0055085,
DN103832_c0_g3_i1	DOWN	-	-	-	-	-	-

geneid	trend	GO	Pathway	TF	NR	NT	Uniprot
DN126302_c0_g1_i1	DOWN	[GO:0005634:nucleus][GO:0009734:auxin-activated signaling pathway][GO:0006355:regulation of transcription, DNA-templated][GO:0006351:transcription, DNA-templated]	[ko04075:Plant hormone signal transduction]	-	XP_021747561.1:auxinresponsive protein IAA9like [Chenopodium quinoa] >XP_021747567.1 auxinresponsive protein IAA9like [Chenopodium quinoa]	gi|20257218|gb|AY090553.1|:Zinnia elegans auxin-regulated protein (IAA8) mRNA, complete cds	A0A0J8C4J8|A0A0J8C4J8_BETVU:Auxin-responsive protein;OS=Beta vulgaris subsp. vulgaris.;|GO:0005634,|GO:0009734,GO:0006355,GO:0006351,
DN115107_c2_g1_i2	DOWN	[GO:0005618:cell wall][GO:0005576:extracellular region][GO:0016020:membrane][GO:0009664:plant-type cell wall organization]	-	676732396:Sisymbrium irio|bHLH|bHLH family protein	AFZ78601.1:expansin protein [Populus tomentosa]	gi|478162897|gb|GAHK01013706.1|:TSA: Jatropha curcas Unigene1159_JC-CK_1A transcribed RNA sequence	L0ASW3|L0ASW3_POPTO:Expansin protein;OS=Populus tomentosa;|GO:0005618,GO:0005576,GO:0016020,|GO:0009664,
DN123793_c1_g2_i4	DOWN	[GO:0005634:nucleus][GO:0003677:DNA binding][GO:0006355:regulation of transcription, DNA-templated][GO:0006351:transcription, DNA-templated]	-	GSVIVT01014403001:Vitis vinifera|NAC|NAC family protein	XP_021840466.1:NAC domaincontaining protein 72like [Spinacia oleracea]	-	A5C6R7|A5C6R7_VITVI:Putative uncharacterized protein;OS=Vitis vinifera;|GO:0005634,|GO:0003677,|GO:0006355,GO:0006351,

geneid	trend	GO	Pathway	TF	NR	NT	Uniprot
DN114132_c1_g6_i3	DOWN	-	-	MDP0000240167:Malus domestica|CPP|CPP family protein	XP_021739436.1:uncharacterized protein LOC110705809 [Chenopodium quinoa]	-	A0A0K9RJL5|A0A0K9RJL5_SPIOL:Uncharacterized protein;OS=Spinacia oleracea;
DN123348_c0_g2_i12	DOWN	[GO:0016757:transferase activity, transferring glycosyl groups]	[ko00052:Galactose metabolism]	-	XP_021734495.1:galactinol synthase 2like [Chenopodium quinoa]	gi|521443710|gb|GAHZ01076774.1|:TSA: Salicornia europaea Unigene93790_SeCKS transcribed RNA sequence	A0A0J8CQ88|A0A0J8CQ88_BETVU:Hexosyltransferase;OS=Beta vulgaris subsp. vulgaris.;|GO:0016757,

geneid	trend	GO	Pathway	TF	NR	NT	Uniprot
DN125900_c0_g5_i1	DOWN	[GO:0005768:endosome][GO:0016021:integral component of membrane][GO:0005886:plasma membrane][GO:0005524:ATP binding][GO:0004674:protein serine/threonine kinase activity][GO:0005496:steroid binding][GO:0048657:anther wall tapetum cell differentiation][GO:0009742:brassinosteroid mediated signaling pathway][GO:0009729:detection of brassinosteroid stimulus][GO:0048366:leaf development][GO:0060548:negative regulation of cell death][GO:0010584:pollen exine formation][GO:0009911:positive regulation of flower development][GO:1900140:regulation of seedling development][GO:0010224:response to UV-B]	[ko04075:Plant hormone signal transduction]	Sme2.5_01196.1_g00006.1:Solanum melongena|STAT|STAT family protein	XP_010680326.1:PREDICTED: systemin receptor SR160 [Beta vulgaris subsp. vulgaris]	-	F6H4C0|F6H4C0_VITVI:Putative uncharacterized protein;OS=Vitis vinifera;GN=VIT_07s0031g01850 {ECO:0000313|EMBL:CCB47131.1};|GO:0005768,GO:0016021,GO:0005886,|GO:0005524,GO:0004674,GO:0005496,|GO:0048657,GO:0009742,GO:0009729,GO:0048366,GO:0060548,GO:0010584,GO:0009911,GO:1900140,GO:0010224,

geneid	trend	GO	Pathway	TF	NR	NT	Uniprot
DN125573_c1_g1_i3	DOWN	[GO:0004575:sucrose alpha-glucosidase activity][GO:0005975:carbohydrate metabolic process]	[ko00052:Galactose metabolism][ko00500:Starch and sucrose metabolism]	-	pir||S49256:betafructofuranosidase (EC 3.2.1.26) red goosefoot (fragment)	gi|521452619|gb|GAHZ01067865.1|:TSA: Salicornia europaea Unigene81936_SeCKS transcribed RNA sequence	Q8VXS6|Q8VXS6_BETVU:Beta-fructofuranosidase;OS=Beta vulgaris;|GO:0004575,|GO:0005975,
DN119009_c0_g1_i2	DOWN	[GO:0005507:copper ion binding][GO:0008131:primary amine oxidase activity][GO:0048038:quinone binding][GO:0009308:amine metabolic process]	[ko00260:Glycine, serine and threonine metabolism][ko00350:Tyrosine metabolism][ko00360:Phenylalanine metabolism][ko00410:beta-Alanine metabolism][ko00950:Isoquinoline alkaloid biosynthesis][ko00960:Tropane, piperidine and pyridine alkaloid biosynthesis]	-	XP_010682433.1:PREDICTED: uncharacterized protein LOC104897280 [Beta vulgaris subsp. vulgaris]	gi|521631770|gb|GAIA01050534.1|:TSA: Salicornia europaea Unigene59065_Se200S transcribed RNA sequence	A0A0K9QZ07|A0A0K9QZ07_SPIOL:Amine oxidase;OS=Spinacia oleracea;|GO:0005507,GO:0008131,GO:0048038,|GO:0009308,
DN118089_c4_g2_i6	DOWN	-	-	-	-	-	-

geneid	trend	GO	Pathway	TF	NR	NT	Uniprot
DN124214_c2_g3_i3	DOWN	[GO:0005829:cytosol][GO:0009505:plant-type cell wall][GO:0005773:vacuole][GO:0003993:acid phosphatase activity][GO:0046872:metal ion binding][GO:0055062:phosphate ion homeostasis]	-	Achn233011:Actinidia chinensis|SRS|SRS family protein	XP_021741804.1:bifunctional purple acid phosphatase 26like [Chenopodium quinoa] >XP_021741805.1 bifunctional purple acid phosphatase 26like [Chenopodium quinoa]	gi|521502737|gb|GAHZ01028183.1|:TSA: Salicornia europaea Unigene31035_SeCKS transcribed RNA sequence	A0A0J8BS79|A0A0J8BS79_BETVU:Purple acid phosphatase;OS=Beta vulgaris subsp. vulgaris.;|GO:0005829,GO:0009505,GO:0005773,|GO:0003993,GO:0046872,|GO:0055062,
DN118254_c1_g1_i1	DOWN	-	-	-	XP_010693674.1:PREDICTED: uncharacterized protein LOC104906597 [Beta vulgaris subsp. vulgaris] >KMS98958.1 hypothetical protein BVRB_3g067580 [Beta vulgaris subsp. vulgaris]	gi|357504112|ref|XM_003622297.1|:Medicago truncatula hypothetical protein (MTR_7g034800) mRNA, complete cds	A0A0J8BG38|A0A0J8BG38_BETVU:Uncharacterized protein;OS=Beta vulgaris subsp. vulgaris.;
DN126513_c0_g1_i3	DOWN	-	-	-	-	-	-

geneid	trend	GO	Pathway	TF	NR	NT	Uniprot
DN113131_c0_g1_i1	DOWN	[GO:0048046:apoplast]	-	Peaxi162Scf00045g01644.1:Petunia axillaris|B3|B3 family protein	XP_021858132.1:dirigent protein 22like [Spinacia oleracea] >KNA14909.1 hypothetical protein SOVF_103120 [Spinacia oleracea]	-	A0A0K9R7F5|A0A0K9R7F5_SPIOL:Dirigent protein;OS=Spinacia oleracea;|GO:0048046,
DN115495_c1_g1_i1	DOWN	-	-	-	XP_021776245.1:gammaglutamyl peptidase 5like [Chenopodium quinoa]	gi|521693646|gb|GAIA01020756.1|:TSA: Salicornia europaea Unigene21737_Se200S transcribed RNA sequence	A0A0K9RKM0|A0A0K9RKM0_SPIOL:Uncharacterized protein;OS=Spinacia oleracea;
DN110960_c0_g2_i7	DOWN	-	-	-	KNA24654.1:hypothetical protein SOVF_013430 [Spinacia oleracea]	gi|255547715|ref|XM_002514869.1|:Ricinus communis conserved hypothetical protein, mRNA	A0A0K9S0F6|A0A0K9S0F6_SPIOL:Uncharacterized protein;OS=Spinacia oleracea;

geneid	trend	GO	Pathway	TF	NR	NT	Uniprot
DN115783_c1_g3_i1	DOWN	[GO:0020037:heme binding][GO:0005506:iron ion binding][GO:0004497:monooxygenase activity][GO:0016705:oxidoreductase activity, acting on paired donors, with incorporation or reduction of molecular oxygen]	-	Thecc1EG000515t1: Theobroma cacao|B3|B3 family protein	XP_021846538.1:isoflavone 2'hydroxylaselike [Spinacia oleracea]	gi|521598257|gb|GAIA01065102.1|:TSA: Salicornia europaea Unigene78141_Se200S transcribed RNA sequence	A0A140JTI1|A0A140JTI1_9CARY:Cytochrome P450 81AG1;OS=Fallopia sachalinensis.;|GO:0020037,GO:0005506,GO:0004497,GO:0016705,
DN127801_c3_g1_i1	DOWN	-	-	-	XP_010694437.1:PREDICTED: uncharacterized protein LOC104907239 [Beta vulgaris subsp. vulgaris] >KMS98365.1 hypothetical protein BVRB_4g093210 [Beta vulgaris subsp. vulgaris]	gi|521717897|gb|GAIA01011847.1|:TSA: Salicornia europaea Unigene12464_Se200S transcribed RNA sequence	A0A0J8E5A9|A0A0J8E5A9_BETVU:Uncharacterized protein;OS=Beta vulgaris subsp. vulgaris.;

geneid	trend	GO	Pathway	TF	NR	NT	Uniprot
DN126583_c2_g3_i2	DOWN	-	-	-	XP_021864624.1:B2 proteinlike [Spinacia oleracea] >KNA24030.1 hypothetical protein SOVF_019830 [Spinacia oleracea]	gi|225426281|ref|XM_002266352.1|:PREDICTED: Vitis vinifera uncharacterized LOC100252953 (LOC100252953), mRNA	A0A0K9RWX0|A0A0K9RWX0_SPIOL:Uncharacterized protein;OS=Spinacia oleracea;
DN114160_c0_g1_i1	DOWN	[GO:0016021:integral component of membrane][GO:0005524:ATP binding][GO:0016787:hydrolase activity][GO:0046872:metal ion binding]	-	-	OWM69740.1:hypothetical protein CDL15_Pgr025589 [Punica granatum]	gi|326509058|dbj|AK355704.1|:Hordeum vulgare subsp. vulgare mRNA for predicted protein, partial cds, clone: NIASHv1024I02	A0A0K9QUA6|A0A0K9QUA6_SPIOL: Uncharacterized protein;OS=Spinacia oleracea;|GO:0016021,|GO:0005524,GO:0016787,GO:0046872,

geneid	trend	GO	Pathway	TF	NR	NT	Uniprot
DN128227_c2_g3_i5	DOWN	[GO:0051537:2 iron, 2 sulfur cluster binding][GO:0009055:electron transfer activity][GO:0050660:flavin adenine dinucleotide binding][GO:0005506:iron ion binding][GO:0016614:oxidoreductase activity, acting on CH-OH group of donors]	[ko00906:Carotenoid biosynthesis]	Zjn_sc00014.1.g06910.1.sm.mk:Zoysia japonica|LBD|LBD family protein	XP_010685490.1:PREDICTED: abscisicaldehyde oxidaselike isoform X1 [Beta vulgaris subsp. vulgaris]	gi|460198044|gb|GABL01063005.1|:TSA: Camelina sativa comp58726_c2_seq1 transcribed RNA sequence	A0A0J8BYB1|A0A0J8BYB1_BETVU: Uncharacterized protein;OS=Beta vulgaris subsp. vulgaris.;|GO:0051537,GO:0009055,GO:0050660,GO:0005506,GO:0016614,
DN112479_c1_g1_i1	DOWN	-	-	-	XP_021732810.1:uncharacterized protein At4g22758like [Chenopodium quinoa]	-	A0A0K9RX34|A0A0K9RX34_SPIOL:Uncharacterized protein;OS=Spinacia oleracea;
DN119997_c2_g2_i1	DOWN	[GO:0016021:integral component of membrane][GO:0016760:cellulose synthase (UDP-forming) activity][GO:0071555:cell wall organization][GO:0030244:cellulose biosynthetic process]	-	-	XP_010675676.1:PREDICTED: cellulose synthaselike protein G2 [Beta vulgaris subsp. vulgaris] >KMT12691.1 hypothetical protein BVRB_4g087890 [Beta vulgaris subsp.	-	A0A0J8F9Z4|A0A0J8F9Z4_BETVU:Uncharacterized protein;OS=Beta vulgaris subsp. vulgaris.;|GO:0016021,|GO:0016760,|GO:0071555,GO:0030244,

geneid	trend	GO	Pathway	TF	NR	NT	Uniprot
					vulgaris]		
DN125549_c2_g1_i5	DOWN	-	-	-	XP_010683216.1:PREDICTED: uncharacterized protein LOC104897939 isoform X2 [Beta vulgaris subsp. vulgaris] >KMT07092.1 hypothetical protein BVRB_6g154190 [Beta vulgaris subsp. vulgaris]	gi|521750532|gb|GAIA01000882.1|:TSA: Salicornia europaea Unigene899_Se200S transcribed RNA sequence	A0A0J8C0V9|A0A0J8C0V9_BETVU:Uncharacterized protein;OS=Beta vulgaris subsp. vulgaris.;
DN120821_c2_g1_i6	DOWN	-	-	-	-	-	-

geneid	trend	GO	Pathway	TF	NR	NT	Uniprot
DN125279_c2_g3_i3	DOWN	[GO:0016021:integral component of membrane]	-	-	KNA19093.1:hypothetical protein SOVF_064810 [Spinacia oleracea]	-	A0A0K9RHX2|A0A0K9RHX2_SPIOL: Uncharacterized protein;OS=Spinacia oleracea;|GO:0016021,
DN128505_c2_g3_i6	DOWN	-	-	-	XP_021763407.1:uncharacterized protein LOC110728087 [Chenopodium quinoa]	-	A0A0J8ERJ3|A0A0J8ERJ3_BETVU:Uncharacterized protein;OS=Beta vulgaris subsp. vulgaris.;
DN115144_c0_g1_i4	DOWN	[GO:0016491:oxidoreductase activity]	-	-	XP_010695883.1:PREDICTED: nonfunctional NADPHdependent codeinone reductase 2 [Beta vulgaris subsp. vulgaris] >KMS97356.1 hypothetical protein BVRB_6g155970 [Beta vulgaris subsp. vulgaris]	gi|470118051|ref|XM_004295109.1|:PREDICTED: Fragaria vesca subsp. vesca non-functional NADPH-dependent codeinone reductase 2-like, transcript variant 1 (LOC101307287), mRNA	A0A0J8B8Q7|A0A0J8B8Q7_BETVU:Uncharacterized protein;OS=Beta vulgaris subsp. vulgaris.;|GO:0016491,

geneid	trend	GO	Pathway	TF	NR	NT	Uniprot
DN118984_c 1_g3_i2	DOWN	-	-	-	XP_021751536.1:Fb ox protein At1g30200like [Chenopodium quinoa]	gi|521599668|gb|GA IA01064631.1|:TSA: Salicornia europaea Unigene77624_Se20 0S transcribed RNA sequence	A0A0J8CRE2|A0A0 J8CRE2_BETVU:U ncharacterized protein;OS=Beta vulgaris subsp. vulgaris.;
DN113281_c 3_g1_i2	DOWN	-	-	-	-	-	-
DN124100_c 6_g22_i1	DOWN	-	-	-	-	-	-
DN118864_c 1_g7_i1	DOWN	[GO:0016021:integral component of membrane][GO:0005215:transporter activity]	-	-	BAV00119.1:plasma membrane intrinsic protein [Dianthus caryophyllus]	gi|1592831|gb|L360 95.1|CIPMIPA:Mese mbryanthemum crystallinum mipA mRNA, complete cds	V5RFY5|V5RFY5_ CUCSA:Plasma intrinsic protein 1-2;OS=Cucumis sativus;|GO:0016021 ,|GO:0005215,
DN119027_c 0_g1_i6	DOWN	[GO:0016021:integral component of membrane]	[ko04075:Plant hormone signal transduction]	-	XP_017617777.1:P REDICTED: auxin transporterlike protein 2 [Gossypium arboreum] >KHG12121.1 Auxin	gi|224434585|dbj|A B488680.1|:Pisum sativum PsAUX1 mRNA for putative auxin transport facilitator protein, complete cds	A0A0B0NLZ6|A0A0 0B0NLZ6_GOSAR: Auxin transporter-like protein 2;OS=Gossypium arboreum;|GO:0016 021,

geneid	trend	GO	Pathway	TF	NR	NT	Uniprot
					transporterlike protein 2 [Gossypium arboreum]		
DN128383_c2_g2_i6	DOWN	-	-	-	XP_010677354.1:PREDICTED: uncharacterized protein LOC104893015 [Beta vulgaris subsp. vulgaris] >XP_010677355.1 PREDICTED: uncharacterized protein LOC104893015 [Beta vulgaris subsp. vulgaris] >KMT11354.1 hypothetical protein BVRB_5g106870 [Beta vulgaris subsp.	-	A0A0J8CGX8|A0A0J8CGX8_BETVU: Uncharacterized protein;OS=Beta vulgaris subsp. vulgaris.;

geneid	trend	GO	Pathway	TF	NR	NT	Uniprot
					vulgaris]		
DN117437_c0_g1_i2	DOWN	[GO:0003950:NAD+ ADP-ribosyltransferase activity]	-	-	XP_008239766.1:PREDICTED: probable inactive poly [ADPribose] polymerase SRO5 [Prunus mume]	-	D7SHS2|D7SHS2_VITVI:Putative uncharacterized protein;OS=Vitis vinifera;GN=VIT_17s0000g08370 {ECO:0000313|EMBL:CBI15032.3};|GO:0003950,

geneid	trend	GO	Pathway	TF	NR	NT	Uniprot
DN114826_c1_g1_i1	DOWN	-	-	-	XP_021732083.1:uncharacterized protein LOC110698881 [Chenopodium quinoa]	-	A0A0J8EDX3|A0A0J8EDX3_BETVU: Uncharacterized protein;OS=Beta vulgaris subsp. vulgaris.;
DN111494_c0_g1_i1	DOWN	[GO:0005576:extracellular region][GO:0020037:heme binding][GO:0046872:metal ion binding][GO:0004601:peroxidase activity][GO:0042744:hydrogen peroxide catabolic process][GO:0006979:response to oxidative stress]	[ko00940:Phenylpropanoid biosynthesis]	EPS67213.1:Genlisea aurea|NAC|NAC family protein	XP_021740467.1:peroxidase 3like [Chenopodium quinoa]	gi|460395777|ref|XM_004243410.1|:PREDICTED: Solanum lycopersicum peroxidase 3-like (LOC101249936), mRNA	A0A0J8B6A2|A0A0J8B6A2_BETVU:Peroxidase;OS=Beta vulgaris subsp. vulgaris.;|GO:0005576,|GO:0020037,GO:0046872,GO:0004601,|GO:0042744,GO:0006979,
DN125067_c0_g1_i7	DOWN	-	-	Vang0599s00010.1: Vigna angularis|NAC|NAC family protein	XP_021866657.1:uncharacterized protein LOC110805376 [Spinacia oleracea] >KNA15128.1 hypothetical protein SOVF_101080 [Spinacia oleracea]	gi|521637233|gb|GAIA01048044.1|:TSA: Salicornia europaea Unigene56278_Se200S transcribed RNA sequence	A0A0K9R6K6|A0A0K9R6K6_SPIOL:Uncharacterized protein;OS=Spinacia oleracea;

geneid	trend	GO	Pathway	TF	NR	NT	Uniprot
DN120551_c0_g2_i5	DOWN	[GO:0009793:embryo development ending in seed dormancy][GO:0098755:maintenance of seed dormancy by absisic acid][GO:0010029:regulation of seed germination][GO:0009845:seed germination]	-	-	XP_021745794.1:GEMlike protein 5 [Chenopodium quinoa]	-	A0A0J8C550|A0A0J8C550_BETVU:Uncharacterized protein;OS=Beta vulgaris subsp. vulgaris.;|GO:0009793,GO:0098755,GO:0010029,GO:0009845,
DN122008_c8_g2_i1	DOWN	-	-	-	-	-	-
DN123845_c2_g1_i1	DOWN	-	-	-	XP_021764252.1:uncharacterized protein LOC110728891 [Chenopodium quinoa]	-	A0A0K9RRE1|A0A0K9RRE1_SPIOL:Uncharacterized protein;OS=Spinacia oleracea;
DN125516_c0_g2_i1	DOWN	[GO:0005524:ATP binding][GO:0006457:protein folding][GO:0009408:response to heat]	[ko04141:Protein processing in endoplasmic reticulum]	ONIVA04G19030.1:Oryza nivara|MYB|MYB family protein	XP_010685460.1:PREDICTED: dnaJ protein homolog ANJ1 [Beta vulgaris subsp. vulgaris] >KMT04963.1 hypothetical protein BVRB_7g171270	-	A0A0J8BYC5|A0A0J8BYC5_BETVU:Uncharacterized protein;OS=Beta vulgaris subsp. vulgaris.;|GO:0005524,|GO:0006457,GO:0009408,

geneid	trend	GO	Pathway	TF	NR	NT	Uniprot
					[Beta vulgaris subsp. vulgaris]		
DN116186_c2_g3_i4	DOWN	[GO:0005783:endoplasmic reticulum][GO:0016021:integral component of membrane][GO:0046923:ER retention sequence binding][GO:0006621:protein retention in ER lumen]	-	Peaxi162Scf01312g00064.1:Petunia axillaris|M-type_MADS|M-type_MADS family protein	XP_010691857.1:PREDICTED: putative ER lumen proteinretaining receptor C28H8.4 [Beta vulgaris subsp. vulgaris] >KMT00258.1 hypothetical protein BVRB_1g016150 [Beta vulgaris subsp. vulgaris]	gi|509215242|gb|GAJL01259337.1|:TSA: Triticum aestivum TA_RNA_016025 transcribed RNA sequence	A0A0J8BJK7|A0A0J8BJK7_BETVU:Uncharacterized protein;OS=Beta vulgaris subsp. vulgaris.;|GO:0005783,GO:0016021,|GO:0046923,|GO:0006621,
DN123530_c4_g3_i2	DOWN	-	-	-	-	-	-

geneid	trend	GO	Pathway	TF	NR	NT	Uniprot
DN127462_c1_g4_i2	DOWN	[GO:0016020:membrane][GO:0005524:ATP binding][GO:0016301:kinase activity]	-	-	XP_010678633.1:PREDICTED: uncharacterized protein LOC104894151 [Beta vulgaris subsp. vulgaris] >KMT10444.1 hypothetical protein BVRB_5g115700 [Beta vulgaris subsp. vulgaris]	gi|521734654|gb|GAIA01009583.1|:TSA: Salicornia europaea Unigene9962_Se200S transcribed RNA sequence	A0A0J8CE34|A0A0J8CE34_BETVU:Uncharacterized protein;OS=Beta vulgaris subsp. vulgaris.;|GO:0016020,|GO:0005524,GO:0016301,
DN128212_c2_g1_i9	DOWN	[GO:0016021:integral component of membrane][GO:0009678:hydrogen-translocating pyrophosphatase activity][GO:0004427:inorganic diphosphatase activity]	[ko00190:Oxidative phosphorylation]	-	PAN23131.1:hypothetical protein PAHAL_D00745 [Panicum hallii]	gi|22532390|gb|AF533336.1|:Chenopodium rubrum vacuolar proton-pumping PPase (CVP1) mRNA, complete cds	C5Z6P9|C5Z6P9_SORBI:Uncharacterized protein;OS=Sorghum bicolor;|GO:0016021,|GO:0009678,GO:0004427,|GO:0015992,

geneid	trend	GO	Pathway	TF	NR	NT	Uniprot
DN115144_c0_g1_i3	DOWN	[GO:0016491:oxidoreductase activity]	-	-	XP_010695883.1:PREDICTED: nonfunctional NADPHdependent codeinone reductase 2 [Beta vulgaris subsp. vulgaris] >KMS97356.1 hypothetical protein BVRB_6g155970 [Beta vulgaris subsp. vulgaris]	-	A0A0J8B8Q7|A0A0J8B8Q7_BETVU:Uncharacterized protein;OS=Beta vulgaris subsp. vulgaris.;|GO:0016491,
DN122116_c3_g6_i1	DOWN	[GO:0016442:RISC complex][GO:0003712:transcription coregulator activity][GO:0035194:posttranscriptional gene silencing by RNA]	[ko05169:Epstein-Barr virus infection][ko05203:Viral carcinogenesis]	-	XP_021852255.1:ribonuclease TUDOR 2like [Spinacia oleracea] >KNA18582.1 hypothetical protein SOVF_069380 [Spinacia oleracea]	-	A0A0K9RI83|A0A0K9RI83_SPIOL:Uncharacterized protein;OS=Spinacia oleracea;|GO:0016442,|GO:0003712,|GO:0035194,
DN124346_c8_g27_i1	DOWN	-	-	-	AGU69247.1:salinityinduced protein [Salicornia brachiata]	-	T1YW73|T1YW73_9CARY:Salinity-induced protein;OS=Salicornia brachiata.;

geneid	trend	GO	Pathway	TF	NR	NT	Uniprot
DN121294_c0_g1_i4	DOWN	[GO:0033926:glycopeptide alpha-N-acetylgalactosaminidase activity]	-	-	XP_010691858.1:PREDICTED: probable alkaline/neutral invertase B [Beta vulgaris subsp. vulgaris] >XP_010691859.1 PREDICTED: probable alkaline/neutral invertase B [Beta vulgaris subsp. vulgaris] >KMT00261.1 hypothetical protein BVRB_1g016180 [Beta vulgaris subsp. vulgaris]	gi|521556227|gb|GAIA01079371.1|:TSA: Salicornia europaea Unigene96435_Se200S transcribed RNA sequence	A0A0J8EB43|A0A0J8EB43_BETVU:Uncharacterized protein;OS=Beta vulgaris subsp. vulgaris.;|GO:0033926,
DN124775_c2_g3_i2	DOWN	[GO:0005886:plasma membrane]	-	-	XP_021849965.1:uncharacterized protein LOC110789306 [Spinacia oleracea]	-	A0A0J8FY32|A0A0J8FY32_BETVU:Uncharacterized protein;OS=Beta vulgaris subsp. vulgaris.;|GO:00058

geneid	trend	GO	Pathway	TF	NR	NT	Uniprot
							86,
DN114669_c5_g4_i3	DOWN	[GO:0004185:serine-type carboxypeptidase activity]	-	KZV23174.1:Dorcoceras hygrometricum|C3H|C3H family protein	XP_021744587.1:serine carboxypeptidaselike 45 [Chenopodium quinoa]	-	A0A0K9RMX3|A0A0K9RMX3_SPIOL:Carboxypeptidase;OS=Spinacia oleracea;|GO:0004185,
DN122522_c0_g5_i2	DOWN	-	-	-	XP_021850991.1:protein IQDOMAIN 31 isoform X2 [Spinacia oleracea]	-	A0A0K9QFU5|A0A0K9QFU5_SPIOL:Uncharacterized protein;OS=Spinacia oleracea;
DN127617_c1_g5_i7	DOWN	[GO:0016021:integral component of membrane][GO:0005886:plasma membrane][GO:0008308:voltage-gated anion channel activity][GO:0006873:cellular ion homeostasis]	-	KN542606.1_FGP001:Oryza longistaminata|GRF|GRF family protein	XP_021726768.1:Stype anion channel SLAH2like [Chenopodium quinoa] >XP_021726769.1 Stype anion channel SLAH2like [Chenopodium	gi|356561467|ref|XM_003548955.1|:PREDICTED: Glycine max S-type anion channel SLAH3-like (LOC100788909), mRNA	A0A0J8CRB3|A0A0J8CRB3_BETVU:Uncharacterized protein;OS=Beta vulgaris subsp. vulgaris.;|GO:0016021,GO:0005886,|GO:0008308,|GO:0006873,

geneid	trend	GO	Pathway	TF	NR	NT	Uniprot
					quinoa]		
DN126337_c1_g1_i6	DOWN	-	-	-	XP_021759929.1:lectinlike isoform X1 [Chenopodium quinoa] >XP_021759930.1 lectinlike isoform X1 [Chenopodium quinoa] >XP_021759931.1 lectinlike isoform X1 [Chenopodium quinoa]	-	A0A0J8D4G9|A0A0J8D4G9_BETVU:Uncharacterized protein;OS=Beta vulgaris subsp. vulgaris.;
DN127228_c5_g17_i2	DOWN	[GO:0051213:dioxygenase activity][GO:0046872:metal ion binding]	-	-	NP_001242731.1:uncharacterized protein LOC100794065 [Glycine max] >ACU20277.1 unknown [Glycine	-	K7MQ88|K7MQ88_SOYBN:Uncharacterized protein;OS=Glycine max;|GO:0051213,GO:0046872,

geneid	trend	GO	Pathway	TF	NR	NT	Uniprot
					max]		
DN122740_c 1_g2_i1	DOWN	[GO:0004190:aspartic-type endopeptidase activity]	-	Peaxi162Scf00170g 01227.1:Petunia axillaris|FAR1|FAR 1 family protein	XP_021861429.1:ba sic 7S globulinlike [Spinacia oleracea] >KNA22753.1 hypothetical protein SOVF_031480 [Spinacia oleracea]	-	A0A0K9RTH0|A0A 0K9RTH0_SPIOL: Uncharacterized protein;OS=Spinacia oleracea;|GO:00041 90,

geneid	trend	GO	Pathway	TF	NR	NT	Uniprot
DN128870_c4_g2_i3	DOWN	[GO:0005524:ATP binding][GO:0016307:phosphatidylinositol phosphate kinase activity]	[ko00562:Inositol phosphate metabolism][ko04070:Phosphatidylinositol signaling system][ko04145:Phagosome][ko04810:Regulation of actin cytoskeleton]	Pbr011696.1:Pyrus bretschneideri|bHLH|bHLH family protein	XP_010673144.1:PREDICTED: putative 1phosphatidylinositol3phosphate 5kinase FAB1D [Beta vulgaris subsp. vulgaris] >XP_010673188.1 PREDICTED: putative 1phosphatidylinositol3phosphate 5kinase FAB1D [Beta vulgaris subsp. vulgaris] >KMT20037.1 hypothetical protein BVRB_1g000560 [Beta vulgaris subsp. vulgaris]	-	A0A0J8D2Q6|A0A0J8D2Q6_BETVU:Uncharacterized protein;OS=Beta vulgaris subsp. vulgaris.;|GO:0005524,GO:0016307,

geneid	trend	GO	Pathway	TF	NR	NT	Uniprot
DN121887_c2_g3_i3	DOWN	[GO:0016021:integral component of membrane]	-	-	XP_010686515.1:PREDICTED: tetraspanin19like [Beta vulgaris subsp. vulgaris]	-	A0A0J8BSW9|A0A0J8BSW9_BETVU: Uncharacterized protein;OS=Beta vulgaris subsp. vulgaris.;|GO:0016021,
DN115744_c0_g1_i1	DOWN	-	-	-	XP_021724288.1:uncharacterized protein LOC110691643 [Chenopodium quinoa]	-	A0A061DVP3|A0A061DVP3_THECC: VQ motif-containing protein, putative;OS=Theobroma cacao;
DN120745_c0_g1_i1	DOWN	-	-	Sp_172350_xysh.t1: Spinacia oleracea|NAC|NAC family protein	AOC59215.1:NAC transcription factor 42 [Haloxylon ammodendron]	-	A0A1B2RWF3|A0A1B2RWF3_9CARY: NAC transcription factor 42;OS=Haloxylon ammodendron.;

geneid	trend	GO	Pathway	TF	NR	NT	Uniprot
DN126749_c1_g1_i6	DOWN	[GO:0005737:cytoplasm][GO:0003871:5-methyltetrahydropteroyltriglutamate-homocysteine S-methyltransferase activity][GO:0008270:zinc ion binding][GO:0009086:methionine biosynthetic process]	[ko00270:Cysteine and methionine metabolism][ko00450:Selenocompound metabolism]	-	P93263.1:RecName: Full=5methyltetrahydropteroyltriglutamatehomocysteine methyltransferase; AltName: Full=Cobalaminindependent methionine synthase isozyme; AltName: Full=VitaminB12independent methionine synthase isozyme >AAB41896.1 methionine synthase [Mesembryanthemum crystallinum]	gi|1814402|gb|U84889.1|MCU84889:Mesembryanthemum crystallinum methionine synthase (MetE) mRNA, complete cds	P93263|METE_MESCR:5-methyltetrahydropteroyltriglutamate--homocysteine methyltransferase;OS=Mesembryanthemum crystallinum;|GO:0005737,|GO:0003871,GO:0008270,|GO:0009086,

geneid	trend	GO	Pathway	TF	NR	NT	Uniprot
DN116878_c0_g1_i1	DOWN	[GO:0005737:cytoplasm][GO:0005634:nucleus][GO:0043565:sequence-specific DNA binding][GO:0003700:DNA-binding transcription factor activity][GO:0006355:regulation of transcription, DNA-templated][GO:0006351:transcription, DNA-templated]	-	Achn188081:Actinidia chinensis|GRAS|GRAS family protein	XP_002272471.1:PREDICTED: scarecrowlike protein 8 [Vitis vinifera]	-	F6H6D7|F6H6D7_VITVI:Putative uncharacterized protein;OS=Vitis vinifera;GN=VIT_16s0050g00950 {ECO:0000313|EMBL:CCB47848.1};|GO:0005737,GO:0005634,|GO:0043565,GO:0003700,|GO:0006355,GO:0006351,
DN127353_c3_g2_i12	DOWN	[GO:0005737:cytoplasm][GO:0004615:phosphomannomutase activity][GO:0009298:GDP-mannose biosynthetic process]	[ko00051:Fructose and mannose metabolism][ko00520:Amino sugar and nucleotide sugar metabolism]	-	XP_021756552.1:phosphomannomutaselike [Chenopodium quinoa]	-	A0A0K9RL25|A0A0K9RL25_SPIOL:Phosphomannomutase;OS=Spinacia oleracea;|GO:0005737,|GO:0004615,|GO:0009298,
DN124424_c3_g2_i5	DOWN	[GO:0016021:integral component of membrane]	-	-	XP_021715826.1:uncharacterized protein LOC110683739 [Chenopodium quinoa]	gi|255587522|ref|XM_002534255.1|:Ricinus communis conserved hypothetical protein, mRNA	A0A0K9QWT1|A0A0K9QWT1_SPIOL:Uncharacterized protein;OS=Spinacia oleracea;|GO:0016021,

geneid	trend	GO	Pathway	TF	NR	NT	Uniprot
DN113690_c0_g1_i1	DOWN	-	[ko02010:ABC transporters][ko04976:Bile secretion][ko05206:MicroRNAs in cancer]	Tp57577_TGAC_v2_mRNA22346:Trifolium pratense|M-type_MADS|M-type_MADS family protein	KZN11631.1:hypothetical protein DCAR_004287 [Daucus carota subsp. sativus]	-	A0A1D1YCF7|A0A1D1YCF7_9ARAE:ABC transporter B family member 2;OS=Anthurium amnicola.;
DN118157_c4_g12_i2	DOWN	[GO:0003824:catalytic activity][GO:0030170:pyridoxal phosphate binding][GO:0009058:biosynthetic process]	[ko00220:Arginine biosynthesis][ko00250:Alanine, aspartate and glutamate metabolism][ko00710:Carbon fixation in photosynthetic organisms]	-	XP_021847467.1:alanine aminotransferase 2 [Spinacia oleracea] >KNA25948.1 hypothetical protein SOVF_001640 [Spinacia oleracea]	gi|521511812|gb|GAHZ01019108.1|:TSA: Salicornia europaea Unigene20057_SeCKS transcribed RNA sequence	A0A0K9S2L4|A0A0K9S2L4_SPIOL:Uncharacterized protein;OS=Spinacia oleracea;|GO:0003824,GO:0030170,|GO:0009058,
DN120840_c3_g1_i5	DOWN	[GO:0005768:endosome][GO:0016021:integral component of membrane][GO:0005802:trans-Golgi network]	-	676766192:Sisymbrium irio|BES1|BES1 family protein	XP_021835248.1:protein trichome birefringencelike 14 [Spinacia oleracea]	-	A0A0J8B7M9|A0A0J8B7M9_BETVU:Uncharacterized protein;OS=Beta vulgaris subsp. vulgaris.;|GO:0005768,GO:0016021,GO:0005802,

geneid	trend	GO	Pathway	TF	NR	NT	Uniprot
DN119042_c2_g1_i6	DOWN	-	-	Gh_D02G0149:Gossypium hirsutum|NAC|NAC family protein	XP_010669986.1:PREDICTED: coiledcoil domaincontaining protein 94 homolog isoform X1 [Beta vulgaris subsp. vulgaris] >KMT17357.1 hypothetical protein BVRB_2g038150 isoform A [Beta vulgaris subsp. vulgaris]	gi|521687833|gb|GAIA01023702.1|:TSA: Salicornia europaea Unigene25342_Se200S transcribed RNA sequence	A0A0J8CUS7|A0A0J8CUS7_BETVU:Uncharacterized protein;OS=Beta vulgaris subsp. vulgaris.;
DN124134_c3_g5_i5	DOWN	[GO:0016491:oxidoreductase activity][GO:0008270:zinc ion binding]	[ko00010:Glycolysis / Gluconeogenesis][ko00071:Fatty acid degradation][ko00350:Tyrosine metabolism][ko00592:alpha-Linolenic acid metabolism]	-	XP_021600872.1:alcohol dehydrogenaselike [Manihot esculenta] >OAY23545.1 hypothetical protein MANES_18G087000 [Manihot esculenta]	-	A0A0K9QY28|A0A0K9QY28_SPIOL:Uncharacterized protein;OS=Spinacia oleracea;|GO:0016491,GO:0008270,

geneid	trend	GO	Pathway	TF	NR	NT	Uniprot
DN109692_c1_g1_i2	DOWN	[GO:0009269:response to desiccation]	-	Lus10001876:Linum usitatissimum|NAC|NAC family protein	XP_010669772.1:PREDICTED: desiccation protectant protein Lea14 homolog [Beta vulgaris subsp. vulgaris] >KMT20399.1 hypothetical protein BVRB_1g003950 [Beta vulgaris subsp. vulgaris]	-	A0A0J8D7Q4|A0A0J8D7Q4_BETVU:Uncharacterized protein;OS=Beta vulgaris subsp. vulgaris.;|GO:0009269,
DN122309_c4_g2_i2	DOWN	[GO:0008168:methyltransferase activity]	-	-	KNA20158.1:hypothetical protein SOVF_054770 [Spinacia oleracea]	-	A0A0K9RKZ1|A0A0K9RKZ1_SPIOL:Uncharacterized protein;OS=Spinacia oleracea;|GO:0008168,

geneid	trend	GO	Pathway	TF	NR	NT	Uniprot
DN114217_c5_g3_i1	DOWN	[GO:0048046:apoplast][GO:0005618:cell wall][GO:0004553:hydrolase activity, hydrolyzing O-glycosyl compounds][GO:0016762:xyloglucan:xyloglucosyl transferase activity][GO:0042546:cell wall biogenesis][GO:0071555:cell wall organization][GO:0010411:xyloglucan metabolic process]	-	Sme2.5_00096.1_g00018.1:Solanum melongena|NAC|NAC family protein	XP_021718449.1:probable xyloglucan endotransglucosylase/hydrolase protein 6 [Chenopodium quinoa]	gi|356541542|ref|XM_003539186.1|:PREDICTED: Glycine max probable xyloglucan endotransglucosylase/hydrolase protein 6-like (LOC100791974), mRNA	G5DAC8|G5DAC8_DIOKA:Xyloglucan endotransglucosylase/hydrolase;OS=Diospyros kaki;|GO:0048046,GO:0005618,|GO:0004553,GO:0016762,|GO:0042546,GO:0071555,GO:0010411,
DN124464_c2_g3_i1	DOWN	[GO:0005524:ATP binding][GO:0046872:metal ion binding][GO:0004478:methionine adenosyltransferase activity][GO:0006730:one-carbon metabolic process][GO:0006556:S-adenosylmethionine biosynthetic process]	[ko00270:Cysteine and methionine metabolism]	-	XP_010276540.1:PREDICTED: Sadenosylmethionine synthase 2 [Nelumbo nucifera]	gi|11992266|gb|AF321001.1|:Suaeda salsa S-adenosylmethionine synthetase 2 mRNA, complete cds	A0A0D2P7N5|A0A0D2P7N5_GOSRA: S-adenosylmethionine synthase;OS=Gossypium raimondii;|GO:0005524,GO:0046872,GO:0004478,|GO:0006730,GO:0006556,

geneid	trend	GO	Pathway	TF	NR	NT	Uniprot
DN127643_c2_g5_i10	DOWN	[GO:0004565:beta-galactosidase activity][GO:0030246:carbohydrate binding][GO:0005975:carbohydrate metabolic process]	-	-	XP_021728326.1:betagalactosidase 8like [Chenopodium quinoa]	gi|521689353|gb|GAIA01022973.1|:TSA: Salicornia europaea Unigene24549_Se200S transcribed RNA sequence	A0A0J8BQ68|A0A0J8BQ68_BETVU:Beta-galactosidase;OS=Beta vulgaris subsp. vulgaris.;|GO:0004565,GO:0030246,|GO:0005975,
DN114704_c0_g1_i3	DOWN	[GO:0016021:integral component of membrane][GO:0005524:ATP binding][GO:0008553:proton-exporting ATPase activity, phosphorylative mechanism][GO:0046872:metal ion binding][GO:0006754:ATP biosynthetic process]	[ko00190:Oxidative phosphorylation]	Lus10040166:Linum usitatissimum|GRAS|GRAS family protein	XP_021761683.1:ATPase 11, plasma membranetypelike [Chenopodium quinoa]	gi|356550951|ref|XM_003543797.1|:PREDICTED: Glycine max plasma membrane ATPase 1-like (LOC100818268), mRNA	A0A0K9RUS9|A0A0K9RUS9_SPIOL:Plasma membrane ATPase;OS=Spinacia oleracea;|GO:0016021,|GO:0005524,GO:0008553,GO:0046872,|GO:0006754,

geneid	trend	GO	Pathway	TF	NR	NT	Uniprot
DN128165_c4_g4_i7	DOWN	[GO:0016021:integral component of membrane][GO:0005524:ATP binding][GO:0004674:protein serine/threonine kinase activity]	-	CCG018340.1:Populus euphratica|B3|B3 family protein	XP_021767444.1:probable LRR receptorlike serine/threonineprotein kinase At1g06840 isoform X3 [Chenopodium quinoa] >XP_021767452.1 probable LRR receptorlike serine/threonineprotein kinase At1g06840 isoform X3 [Chenopodium quinoa]	gi|255582017|ref|XM_002531760.1|:Ricinus communis ATP binding protein, putative, mRNA	A0A0K9R8K6|A0A0K9R8K6_SPIOL:Uncharacterized protein;OS=Spinacia oleracea;|GO:0016021,|GO:0005524,GO:0004674,
DN112388_c5_g6_i2	DOWN	-	-	-	-	-	-

geneid	trend	GO	Pathway	TF	NR	NT	Uniprot
DN118830_c0_g1_i3	DOWN	[GO:0005634:nucleus][GO:0004842:ubiquitin-protein transferase activity][GO:0008270:zinc ion binding][GO:0031347:regulation of defense response]	-	-	XP_010677203.1:PREDICTED: BOIrelated E3 ubiquitinprotein ligase 1 [Beta vulgaris subsp. vulgaris] >KMT11727.1 hypothetical protein BVRB_5g105630 [Beta vulgaris subsp. vulgaris]	-	A0A0J8CDT9|A0A0J8CDT9_BETVU:Uncharacterized protein;OS=Beta vulgaris subsp. vulgaris.;|GO:0005634,|GO:0004842,GO:0008270,|GO:0031347,
DN114298_c2_g1_i13	DOWN	[GO:0005840:ribosome][GO:0003735:structural constituent of ribosome][GO:0006412:translation]	[ko03010:Ribosome]	-	BAM64815.1:RPL13 [Beta vulgaris]	gi|521491904|gb|GAHZ01038982.1|:TSA: Salicornia europaea Unigene44159_SeCKS transcribed RNA sequence	K4Q1V6|K4Q1V6_BETVU:60S ribosomal protein L13;OS=Beta vulgaris;|GO:0005840,|GO:0003735,|GO:0006412,
DN126991_c0_g1_i3	DOWN	[GO:0016021:integral component of membrane][GO:0030246:carbohydrate binding]	-	-	GAV56809.1:Aa_trans domaincontaining protein, partial [Cephalotus follicularis]	gi|255707079|dbj|AB477096.1|:Beta vulgaris Bet/ProT mRNA for betaine/proline transporter,	W9R1P4|W9R1P4_9ROSA:Lysine histidine transporter 1;OS=Morus notabilis.;|GO:0016021,|GO:0030246,

geneid	trend	GO	Pathway	TF	NR	NT	Uniprot
						complete cds	
DN118157_c4_g11_i6	DOWN	[GO:0003824:catalytic activity][GO:0030170:pyridoxal phosphate binding][GO:0009058:biosynthetic process]	[ko00220:Arginine biosynthesis][ko00250:Alanine, aspartate and glutamate metabolism][ko00710:Carbon fixation in photosynthetic organisms]	-	XP_010685211.1:PREDICTED: alanine aminotransferase 2 [Beta vulgaris subsp. vulgaris] >KMT04732.1 hypothetical protein BVRB_7g169130 [Beta vulgaris subsp. vulgaris]	gi|521511812|gb|GAHZ01019108.1|:TSA: Salicornia europaea Unigene20057_SeCKS transcribed RNA sequence	A0A0J8BUA6|A0A0J8BUA6_BETVU: Uncharacterized protein;OS=Beta vulgaris subsp. vulgaris.;|GO:0003824,GO:0030170,|GO:0009058,
DN126635_c3_g1_i13	DOWN	[GO:0003993:acid phosphatase activity][GO:0046872:metal ion binding]	-	Achn233011:Actinidia chinensis|SRS|SRS family protein	XP_010672424.1:PREDICTED: purple acid phosphatase [Beta vulgaris subsp. vulgaris] >KMT20665.1 hypothetical protein BVRB_1g006410 [Beta vulgaris subsp. vulgaris]	gi|521738168|gb|GAIA01007715.1|:TSA: Salicornia europaea Unigene8034_Se200S transcribed RNA sequence	A0A0J8D3C8|A0A0J8D3C8_BETVU:Purple acid phosphatase;OS=Beta vulgaris subsp. vulgaris.;|GO:0003993,GO:0046872,

geneid	trend	GO	Pathway	TF	NR	NT	Uniprot
DN127991_c1_g8_i2	DOWN	[GO:0030598:rRNA N-glycosylase activity][GO:0006952:defense response][GO:0017148:negative regulation of translation]	-	-	AAL35962.1:bouganin [Bougainvillea spectabilis]	gi|17149253|gb|AF445416.1|AF445416: Bougainvillea spectabilis bouganin mRNA, complete cds	Q8W4U4|Q8W4U4_9CARY:rRNA N-glycosidase;OS= Bougainvillea spectabilis.;|GO:0030598,|GO:0006952, GO:0017148,
DN126337_c1_g1_i8	DOWN	-	-	-	XP_021759929.1:lectinlike isoform X1 [Chenopodium quinoa] >XP_021759930.1 lectinlike isoform X1 [Chenopodium quinoa] >XP_021759931.1 lectinlike isoform X1 [Chenopodium quinoa]	-	A0A0J8D4G9|A0A0J8D4G9_BETVU:Uncharacterized protein;OS=Beta vulgaris subsp. vulgaris.;
DN122415_c1_g1_i1	DOWN	-	[ko04111:Cell cycle - yeast][ko04390:Hippo signaling pathway][ko04391: Hippo signaling	-	XP_006381631.1:hypothetical protein POPTR_0006s14500g [Populus trichocarpa] >ERP59428.1	gi|356530424|ref|XM_003533734.1|:PREDICTED: Glycine max mps one binder kinase activator-like 1-like	U5G7B2|U5G7B2_POPTR:Uncharacterized protein;OS=Populus trichocarpa;

geneid	trend	GO	Pathway	TF	NR	NT	Uniprot
			pathway - fly][ko04392:Hippo signaling pathway -multiple species]		hypothetical protein POPTR_0006s14500g [Populus trichocarpa]	(LOC100794894), mRNA	
DN128475_c2_g1_i4	DOWN	-	-	-	-	-	-

geneid	trend	GO	Pathway	TF	NR	NT	Uniprot
DN112748_c0_g1_i1	DOWN	[GO:0016021:integral component of membrane][GO:0005622:intracellular][GO:0015450:P-P-bond-hydrolysis-driven protein transmembrane transporter activity][GO:0006605:protein targeting]	[ko03060:Protein export][ko04141:Protein processing in endoplasmic reticulum][ko04145:Phagosome][ko05110:Vibrio cholerae infection]	mrna06313.1-v1.0-hybrid:Fragaria vesca|NF-YC|NF-YC family protein	XP_006848251.1:protein transport protein Sec61 subunit gamma1 [Amborella trichopoda] >XP_011624359.1 protein transport protein Sec61 subunit gamma1 [Amborella trichopoda] >XP_020524588.1 protein transport protein Sec61 subunit gamma1 [Amborella trichopoda] >XP_020524589.1 protein transport protein Sec61 subunit gamma1 [Amborella trichopoda] >ERN09832.1	-	M0VP35|M0VP35_HORVD:Uncharacterized protein;OS=Hordeum vulgare var. distichum;|GO:0016021,GO:0005622,|GO:0015450,|GO:0006605,

geneid	trend	GO	Pathway	TF	NR	NT	Uniprot
					hypothetical protein AMTR_s00013p00046010 [Amborella trichopoda]		
DN127142_c4_g18_i1	DOWN	[GO:0004553:hydrolase activity, hydrolyzing O-glycosyl compounds][GO:0005975:carbohydrate metabolic process]	[ko00520:Amino sugar and nucleotide sugar metabolism]	-	CDP17849.1:unnamed protein product [Coffea canephora]	gi|357512472|ref|XM_003626477.1|:Medicago truncatula Acidic endochitinase (MTR_7g116850) mRNA, complete	A0A068VBB2|A0A068VBB2_COFCA: Uncharacterized protein;OS=Coffea canephora;|GO:0004553,|GO:0005975,

geneid	trend	GO	Pathway	TF	NR	NT	Uniprot
						cds	
DN118932_c0_g2_i2	DOWN	[GO:0005524:ATP binding][GO:0008641:ubiquitin-like modifier activating enzyme activity][GO:0006464:cellular protein modification process]	[ko04120:Ubiquitin mediated proteolysis][ko05012:Parkinson's disease]	-	XP_021769666.1:ubiquitinactivating enzyme E1 2like [Chenopodium quinoa]	gi|242036036|ref|XM_002465368.1|:Sorghum bicolor hypothetical protein, mRNA	A0A0K9QK53|A0A0K9QK53_SPIOL:Uncharacterized protein;OS=Spinacia oleracea;|GO:0005524,GO:0008641,|GO:0006464,
DN123026_c1_g4_i1	DOWN	-	-	-	XP_021843001.1:jasmonateinduced protein homolog [Spinacia oleracea] >KNA21727.1 hypothetical protein SOVF_040720 [Spinacia oleracea]	-	A0A0K9RSF1|A0A0K9RSF1_SPIOL:Uncharacterized protein;OS=Spinacia oleracea;
DN118958_c2_g1_i4	DOWN	[GO:0005887:integral component of plasma membrane][GO:0008515:sucrose transmembrane transporter activity]	-	Achn143971:Actinidia chinensis|HD-ZIP|HD-ZIP family protein	KMT20052.1:hypothetical protein BVRB_1g000710 [Beta vulgaris subsp. vulgaris]	gi|5822999|gb|U64967.1|BVU64967:Beta vulgaris sucrose-proton symporter (Bv8-6)	A0A0J8D2R8|A0A0J8D2R8_BETVU:Uncharacterized protein;OS=Beta vulgaris subsp.

geneid	trend	GO	Pathway	TF	NR	NT	Uniprot
						mRNA, complete cds	vulgaris.;|GO:0005887,|GO:0008515,
DN116303_c4_g1_i2	DOWN	-	-	Achn132541:Actinidia chinensis|ERF|ERF family protein	XP_010693501.1:PREDICTED: BAG family molecular chaperone regulator 3 [Beta vulgaris subsp. vulgaris] >KMS99049.1 hypothetical protein BVRB_3g066510 [Beta vulgaris subsp. vulgaris]	gi|356559872|ref|XR_137352.1|:PREDICTED: Glycine max uncharacterized LOC100818492 (LOC100818492), miscRNA	A0A0J8BCI5|A0A0J8BCI5_BETVU:Uncharacterized protein;OS=Beta vulgaris subsp. vulgaris.;
DN119383_c1_g6_i2	DOWN	[GO:0005524:ATP binding][GO:0004674:protein serine/threonine kinase activity]	-	Peinf101Scf01099g01019.1:Petunia inflata|HSF|HSF family protein	XP_021851492.1:SRSF protein kinase 1like [Spinacia oleracea] >KNA20111.1 hypothetical protein SOVF_055320 [Spinacia oleracea]	gi|521637487|gb|GAIA01047930.1|:TSA: Salicornia europaea Unigene56157_Se200S transcribed RNA sequence	A0A0K9RKT6|A0A0K9RKT6_SPIOL: Uncharacterized protein;OS=Spinacia oleracea;|GO:0005524,GO:0004674,

geneid	trend	GO	Pathway	TF	NR	NT	Uniprot
DN126708_c2_g1_i2	DOWN	[GO:0003824:catalytic activity][GO:0005992:trehalose biosynthetic process]	[ko00500:Starch and sucrose metabolism]	-	XP_021718967.1:probable alpha,alphatrehalose phosphate synthase [UDPforming] 9 isoform X1 [Chenopodium quinoa]	gi|123709445|emb|AM475307.1|:Vitis vinifera, whole genome shotgun sequence, contig VV78X222065.21, clone ENTAV 115	A0A0K9QJI2|A0A0K9QJI2_SPIOL:Uncharacterized protein;OS=Spinacia oleracea;|GO:0003824,|GO:0005992,
DN113817_c1_g1_i2	DOWN	[GO:0005576:extracellular region]	-	Sp_159490_wski.t1:Spinacia oleracea|bHLH|bHLH family protein	AAN86683.1:beta expansin EXPB2.1 [Mirabilis jalapa]	gi|30038050|gb|AY147412.1|:Mirabilis jalapa beta expansin EXPB2.1 (ExpB2.1) mRNA, complete cds	Q84L48|Q84L48_MIRJA:Beta expansin EXPB2.1;OS=Mirabilis jalapa;|GO:0005576,
DN80815_c0_g3_i1	DOWN	-	-	-	-	-	-
DN126324_c2_g1_i2	DOWN	-	-	-	KZV51009.1:Metallocarboxypeptidase inhibitor [Dorcoceras hygrometricum]	gi|520746981|gb|GALE01012307.1|:TSA: Spraguea lophii Contig_38297_c1_seq7 transcribed RNA sequence	A0A0J8BHP9|A0A0J8BHP9_BETVU:Uncharacterized protein;OS=Beta vulgaris subsp. vulgaris.;

geneid	trend	GO	Pathway	TF	NR	NT	Uniprot
DN125731_c4_g6_i1	DOWN	-	-	-	XP_010670303.1:PREDICTED: jasmonateinduced protein homolog [Beta vulgaris subsp. vulgaris] >KMT17313.1 hypothetical protein BVRB_2g040070 [Beta vulgaris subsp. vulgaris]	-	A0A0J8CZH0|A0A0J8CZH0_BETVU:Uncharacterized protein;OS=Beta vulgaris subsp. vulgaris.;

geneid	trend	GO	Pathway	TF	NR	NT	Uniprot
DN113227_c0_g1_i4	DOWN	[GO:0016307:phosphatidylinositol phosphate kinase activity]	[ko00562:Inositol phosphate metabolism][ko04011:MAPK signaling pathway - yeast][ko04070:Phosphatidylinositol signaling system][ko04072:Phospholipase D signaling pathway][ko04139:Mitophagy - yeast][ko04144:Endocytosis][ko04666:Fc gamma R-mediated phagocytosis][ko04810:Regulation of actin cytoskeleton][ko05231:Choline metabolism in cancer]	-	XP_021760505.1:phosphatidylinositol 4phosphate 5kinase 1like [Chenopodium quinoa]	-	A0A0K9R0F6|A0A0K9R0F6_SPIOL:Uncharacterized protein;OS=Spinacia oleracea;|GO:0016307,

geneid	trend	GO	Pathway	TF	NR	NT	Uniprot
DN116186_c2_g3_i2	DOWN	[GO:0005783:endoplasmic reticulum][GO:0016021:integral component of membrane][GO:0046923:ER retention sequence binding][GO:0006621:protein retention in ER lumen]	-	Peaxi162Scf01312g00064.1:Petunia axillaris|M-type_MADS|M-type_MADS family protein	XP_010691857.1:PREDICTED: putative ER lumen proteinretaining receptor C28H8.4 [Beta vulgaris subsp. vulgaris] >KMT00258.1 hypothetical protein BVRB_1g016150 [Beta vulgaris subsp. vulgaris]	gi|509215242|gb|GAJL01259337.1|:TSA: Triticum aestivum TA_RNA_016025 transcribed RNA sequence	A0A0J8BJK7|A0A0J8BJK7_BETVU:Uncharacterized protein;OS=Beta vulgaris subsp. vulgaris.;|GO:0005783,GO:0016021,|GO:0046923,|GO:0006621,
DN124100_c6_g1_i1	DOWN	-	-	-	-	-	-
DN120657_c3_g1_i2	DOWN	-	-	-	-	-	-
DN127959_c3_g1_i5	DOWN	[GO:0042409:caffeoyl-CoA O-methyltransferase activity][GO:0046872:metal ion binding][GO:0009809:lignin biosynthetic process]	[ko00360:Phenylalanine metabolism][ko00940:Phenylpropanoid biosynthesis][ko00941:Flavonoid biosynthesis][ko00945:Stilbenoid,	ONIVA06G05580.1: Oryza nivara|ERF|ERF family protein	OVA15646.1:Omethyltransferase [Macleaya cordata]	gi|381218035|gb|JQ684014.1|:Paeonia lactiflora cultivar Hongyanzhenghui caffeoyl-CoA O-methyltransferase mRNA, complete cds	Q8H9B6|CAMT_SOLTU:Caffeoyl-CoA O-methyltransferase; OS=Solanum tuberosum;|GO:0042409,GO:0046872,|GO:0009809,

geneid	trend	GO	Pathway	TF	NR	NT	Uniprot
			diarylheptanoid and gingerol biosynthesis]				
DN125573_c1_g1_i2	DOWN	[GO:0016021:integral component of membrane][GO:0004575:sucrose alpha-glucosidase activity][GO:0005975:carbohydrate metabolic process]	[ko00052:Galactose metabolism][ko00500:Starch and sucrose metabolism]	-	XP_021751853.1:acid betafructofuranosidaselike [Chenopodium quinoa]	gi|18072856|emb|AJ277456.1|:Beta vulgaris partial vi2 gene for beta-fructofuranosidase, exon 3	A0A0J8CVC3|A0A0J8CVC3_BETVU: Uncharacterized protein;OS=Beta vulgaris subsp. vulgaris.;|GO:0016021,|GO:0004575,|GO:0005975,
DN120140_c0_g1_i4	DOWN	[GO:0016758:transferase activity, transferring hexosyl groups][GO:0008152:metabolic process]	-	PH01002606G0040: Phyllostachys heterocycla|HSF|HSF family protein	XP_021865044.1:soyasapogenol B glucuronide galactosyltransferase like [Spinacia oleracea] >KNA07640.1 hypothetical protein SOVF_169980	-	A0A0K9QK68|A0A0K9QK68_SPIOL:Glycosyltransferase;OS=Spinacia oleracea;|GO:0016758,|GO:0008152,

geneid	trend	GO	Pathway	TF	NR	NT	Uniprot
					[Spinacia oleracea]		
DN125124_c4_g1_i1	DOWN	-	-	-	OIT08493.1:hypothetical protein A4A49_13909 [Nicotiana attenuata]	-	A0A061GP27|A0A061GP27_THECC:Serine-rich protein-related;OS=Theobroma cacao;
DN116131_c1_g1_i1	DOWN	[GO:0004857:enzyme inhibitor activity]	-	-	XP_010677216.1:PREDICTED: putative invertase inhibitor [Beta vulgaris subsp. vulgaris] >KMT11739.1 hypothetical protein BVRB_5g105730 [Beta vulgaris subsp. vulgaris]	-	A0A0J8CI04|A0A0J8CI04_BETVU:Uncharacterized protein;OS=Beta vulgaris subsp. vulgaris.;|GO:0004857,

geneid	trend	GO	Pathway	TF	NR	NT	Uniprot
DN125127_c0_g2_i5	DOWN	[GO:0016021:integral component of membrane][GO:0005622:intracellular][GO:0005524:ATP binding][GO:0004143:diacylglycerol kinase activity][GO:0035556:intracellular signal transduction][GO:0007205:protein kinase C-activating G protein-coupled receptor signaling pathway]	[ko00561:Glycerolipid metabolism][ko00564:Glycerophospholipid metabolism][ko04070:Phosphatidylinositol signaling system][ko04072:Phospholipase D signaling pathway][ko05231:Choline metabolism in cancer]	-	XP_010678411.1:PREDICTED: diacylglycerol kinase 1 [Beta vulgaris subsp. vulgaris] >XP_010678412.1 PREDICTED: diacylglycerol kinase 1 [Beta vulgaris subsp. vulgaris] >KMT10723.1 hypothetical protein BVRB_5g113810 [Beta vulgaris subsp. vulgaris]	gi|225457278|ref|XM_002281311.1|:PREDICTED: Vitis vinifera diacylglycerol kinase 1-like (LOC100255335), mRNA	A0A0J8CFD9|A0A0J8CFD9_BETVU:Diacylglycerol kinase;OS=Beta vulgaris subsp. vulgaris.;|GO:0016021,GO:0005622,|GO:0005524,GO:0004143,|GO:0035556,GO:0007205,
DN125680_c0_g1_i1	DOWN	[GO:0043531:ADP binding][GO:0006952:defense response]	[ko04626:Plant-pathogen interaction]	ONIVA06G28780.1:Oryza nivara|GATA|GATA family protein	XP_021732164.1:disease resistance protein RPM1like [Chenopodium quinoa]	-	A0A0K9QJQ4|A0A0K9QJQ4_SPIOL:Uncharacterized protein;OS=Spinacia oleracea;|GO:0043531,|GO:0006952,

geneid	trend	GO	Pathway	TF	NR	NT	Uniprot
DN126302_c0_g2_i3	DOWN	[GO:0005634:nucleus][GO:0009734:auxin-activated signaling pathway][GO:0006355:regulation of transcription, DNA-templated][GO:0006351:transcription, DNA-templated]	[ko04075:Plant hormone signal transduction]	Medtr3g064050.1:Medicago truncatula|ARF|ARF family protein	XP_021747561.1:auxinresponsive protein IAA9like [Chenopodium quinoa] >XP_021747567.1 auxinresponsive protein IAA9like [Chenopodium quinoa]	gi|521736340|gb|GAIA01008740.1|:TSA: Salicornia europaea Unigene9090_Se200S transcribed RNA sequence	A0A0J8C4J8|A0A0J8C4J8_BETVU:Auxin-responsive protein;OS=Beta vulgaris subsp. vulgaris.;|GO:0005634,|GO:0009734,GO:0006355,GO:0006351,
DN120491_c2_g1_i3	DOWN	[GO:0005634:nucleus][GO:0003677:DNA binding][GO:0008270:zinc ion binding][GO:0045893:positive regulation of transcription, DNA-templated]	-	-	XP_010695936.1:PREDICTED: uncharacterized protein LOC104908518 [Beta vulgaris subsp. vulgaris] >AFM94014.1 hypothetical protein [Beta vulgaris] >KMT18907.1 hypothetical protein BVRB_2g030080 [Beta vulgaris subsp. vulgaris]	gi|460390509|ref|XM_004240826.1|:PREDICTED: Solanum lycopersicum uncharacterized LOC101258159 (LOC101258159), mRNA	I6TMC3|I6TMC3_BETVU:Uncharacterized protein;OS=Beta vulgaris;|GO:0005634,|GO:0003677,GO:0008270,|GO:0045893,

geneid	trend	GO	Pathway	TF	NR	NT	Uniprot
DN127497_c4_g1_i10	DOWN	[GO:0003824:catalytic activity][GO:0008152:metabolic process]	[ko00061:Fatty acid biosynthesis][ko00071:Fatty acid degradation][ko02024:Quorum sensing][ko03320:PPAR signaling pathway][ko04146:Peroxisome][ko04216:Ferroptosis][ko04920:Adipocytokine signaling pathway]	TRIUR3_12546-P1:Triticum urartu|WRKY|WRKY family protein	XP_021770012.1:long chain acylCoA synthetase 1like isoform X1 [Chenopodium quinoa]	gi|521696965|gb|GAIA01019096.1|:TSA: Salicornia europaea Unigene20056_Se200S transcribed RNA sequence	A0A0J8C8V6|A0A0J8C8V6_BETVU:Uncharacterized protein;OS=Beta vulgaris subsp. vulgaris.;|GO:0003824,|GO:0008152,
DN125902_c0_g3_i4	DOWN	[GO:0003676:nucleic acid binding][GO:0015074:DNA integration]	-	Dca52167.1:Dianthus caryophyllus|M-type_MADS|M-type_MADS family protein	OMP02866.1:Reverse transcriptase, RNAdependent DNA polymerase [Corchorus capsularis]	-	M1ANG9|M1ANG9_SOLTU:Uncharacterized protein;OS=Solanum tuberosum;|GO:0003676,|GO:0015074,
DN99472_c0_g1_i1	DOWN	-	-	-	-	-	-
DN125958_c1_g1_i9	DOWN	-	-	OB01G21870.1:Oryza brachyantha|WRKY|	XP_021721786.1:protein ALP1like [Chenopodium	-	A2ZTW0|A2ZTW0_ORYSJ:Uncharacterized

geneid	trend	GO	Pathway	TF	NR	NT	Uniprot
				WRKY family protein	quinoa]		protein;OS=Oryza sativa subsp. japonica;
DN119223_c1_g3_i5	DOWN	-	-	-	XP_021759811.1:thaumatinlike protein 1b [Chenopodium quinoa]	gi|521742280|gb|GAIA01005030.1|:TSA: Salicornia europaea Unigene5317_Se200S transcribed RNA sequence	A0A0J8CCB6|A0A0J8CCB6_BETVU:Uncharacterized protein;OS=Beta vulgaris subsp. vulgaris.;
DN125413_c5_g2_i6	DOWN	[GO:0016021:integral component of membrane][GO:0005622:intracellular][GO:0005215:transporter activity]	-	mrna15187.1-v1.0-hybrid:Fragaria vesca|bHLH|bHLH family protein	XP_021738651.1:phosphatidylinositol/phosphatidylcholine transfer protein SFH6like [Chenopodium quinoa]	gi|521703591|gb|GAIA01016129.1|:TSA: Salicornia europaea Unigene16837_Se200S transcribed RNA sequence	A0A0K9QSX2|A0A0K9QSX2_SPIOL: Uncharacterized protein;OS=Spinacia oleracea;|GO:0016021,GO:0005622,|GO:0005215,
DN119908_c2_g2_i1	DOWN	-	-	-	-	-	-
DN118605_c1_g2_i1	DOWN	-	-	-	-	-	-

geneid	trend	GO	Pathway	TF	NR	NT	Uniprot
DN120299_c1_g3_i4	DOWN	[GO:0005829:cytosol][GO:0005634:nucleus][GO:0005886:plasma membrane][GO:0009536:plastid][GO:0005524:ATP binding][GO:0004674:protein serine/threonine kinase activity][GO:0007584:response to nutrient][GO:0009414:response to water deprivation][GO:0007165:signal transduction][GO:0010118:stomatal movement]	-	Lus10043264:Linum usitatissimum|YABBY|YABBY family protein	XP_010687109.1:PREDICTED: CBLinteracting serine/threonineprotein kinase 23 [Beta vulgaris subsp. vulgaris] >KMT03898.1 hypothetical protein BVRB_8g187980 [Beta vulgaris subsp. vulgaris]	gi|521527312|gb|GAHZ01009731.1|:TSA: Salicornia europaea Unigene10268_SeCKS transcribed RNA sequence	A0A0J8BVC7|A0A0J8BVC7_BETVU: Non-specific serine/threonine protein kinase;OS=Beta vulgaris subsp. vulgaris.;|GO:0005829,GO:0005634,GO:0005886,GO:0009536,|GO:0005524,GO:0004674,|GO:0010107,GO:0007584,GO:0009414,GO:0007165,GO:0010118,

geneid	trend	GO	Pathway	TF	NR	NT	Uniprot
DN125245_c0_g2_i2	DOWN	[GO:0005737:cytoplasm][GO:0005634:nucleus][GO:0019773:proteasome core complex, alpha-subunit complex][GO:0004298:threonine-type endopeptidase activity][GO:0006511:ubiquitin-dependent protein catabolic process]	[ko03050:Proteasome]	-	XP_021835536.1:proteasome subunit alpha type3 [Spinacia oleracea] >O24362.1 RecName: Full=Proteasome subunit alpha type3; AltName: Full=20S proteasome alpha subunit G; AltName: Full=20S proteasome subunit alpha7; AltName: Full=Proteasome component C8 >BAA21651.1 26S proteasome alpha subunit [Spinacia oleracea] >KNA09060.1 hypothetical protein SOVF_157090 [Spinacia oleracea]	gi|2285801|dbj|D78173.1|:Spinacia oleracea mRNA for 26S proteasome alpha subunit, complete cds	O24362|PSA3_SPIOL:Proteasome subunit alpha type-3;OS=Spinacia oleracea;|GO:0005737,GO:0005634,GO:0019773,|GO:0004298,|GO:0006511,

geneid	trend	GO	Pathway	TF	NR	NT	Uniprot
DN124522_c0_g8_i1	DOWN	[GO:0005737:cytoplasm][GO:0005634:nucleus][GO:0005524:ATP binding][GO:0004127:cytidylate kinase activity][GO:0009041:uridylate kinase activity][GO:0006207:'de novo' pyrimidine nucleobase biosynthetic process][GO:0006221:pyrimidine nucleotide biosynthetic process]	[ko00240:Pyrimidine metabolism]	-	XP_021835169.1:UMPCMP kinase 3 [Spinacia oleracea] >KNA12696.1 hypothetical protein SOVF_123580 [Spinacia oleracea]	gi|502155650|ref|XM_004510087.1|:PREDICTED: Cicer arietinum UMP/CMP kinase-like (LOC101513002), transcript variant X2, mRNA	A0A0K9QZL1|A0A0K9QZL1_SPIOL:UMP-CMP kinase;OS=Spinacia oleracea;|GO:0005737,GO:0005634,|GO:0005524,GO:0004127,GO:0009041,|GO:0006207,GO:0006221,

geneid	trend	GO	Pathway	TF	NR	NT	Uniprot
DN123372_c4_g3_i1	DOWN	[GO:0016021:integral component of membrane]	[ko04064:NF-kappa B signaling pathway][ko04620:Toll-like receptor signaling pathway][ko04621:NOD-like receptor signaling pathway][ko04624:Toll and Imd signaling pathway][ko04722:Neurotrophin signaling pathway][ko05133:Pertussis][ko05140:Leishmaniasis][ko05142:Chagas disease (American trypanosomiasis)][ko05145:Toxoplasmosis][ko05152:Tuberculosis][ko05162:Measles][ko05164:Influenza A]	Kaladp0839s0004.1.p:Kalanchoe laxiflora|ERF|ERF family protein	XP_010686941.1:PREDICTED: putative receptorlike protein kinase At4g00960 [Beta vulgaris subsp. vulgaris]	-	A0A0J8BS32|A0A0J8BS32_BETVU:Uncharacterized protein;OS=Beta vulgaris subsp. vulgaris.;|GO:0016021,

geneid	trend	GO	Pathway	TF	NR	NT	Uniprot
DN117982_c5_g3_i5	DOWN	-	-	-	KMT15127.1:hypothetical protein BVRB_3g062450 [Beta vulgaris subsp. vulgaris]	-	A0A0J8CNM8|A0A0J8CNM8_BETVU: Uncharacterized protein;OS=Beta vulgaris subsp. vulgaris.;
DN127259_c2_g1_i1	DOWN	-	-	-	XP_021865553.1:glycinerich domaincontaining protein 1like isoform X3 [Spinacia oleracea] >KNA12464.1 hypothetical protein SOVF_125400 [Spinacia oleracea]	gi|521743119|gb|GAIA01004608.1|:TSA: Salicornia europaea Unigene4892_Se200S transcribed RNA sequence	A0A0K9QYW3|A0A0K9QYW3_SPIOL:Uncharacterized protein;OS=Spinacia oleracea;
DN81897_c0_g1_i1	DOWN	[GO:0005737:cytoplasm][GO:0004349:glutamate 5-kinase activity][GO:0004350:glutamate-5-semialdehyde dehydrogenase activity][GO:0006561:proline biosynthetic process]	[ko00330:Arginine and proline metabolism]	mrna20854.1-v1.0-hybrid:Fragaria vesca|B3|B3 family protein	AEV46825.1:delta1 pyrroline5carboxylate synthetase [Boehmeria nivea]	gi|510787172|gb|KC602002.1|:Salicornia bigelovii pyrroline-5-carboxylate synthetase (p5cs) mRNA, partial cds	G9JKM5|G9JKM5_BOENI:Delta-1-pyrroline-5-carboxylate synthetase;OS=Boehmeria nivea;|GO:0005737,|GO:0004349,GO:0004350,|GO:0006561,

geneid	trend	GO	Pathway	TF	NR	NT	Uniprot
DN117220_c1_g3_i1	DOWN	-	-	-	XP_021640823.1:uncharacterized protein LOC110635701 isoform X2 [Hevea brasiliensis]	-	B9SDU1|B9SDU1_RICCO:Putative uncharacterized protein;OS=Ricinus communis;
DN114577_c0_g1_i1	DOWN	[GO:0003824:catalytic activity]	[ko00052:Galactose metabolism]	-	XP_021770039.1:stachyose synthaselike [Chenopodium quinoa]	gi|460367029|ref|XM_004229330.1|:PREDICTED: Solanum lycopersicum stachyose synthase-like (LOC101263843), mRNA	M1AI33|M1AI33_SOLTU:Uncharacterized protein;OS=Solanum tuberosum;|GO:0003824,
DN117683_c0_g1_i1	DOWN	-	-	Bv1_018470_kcda.t1:Beta vulgaris|bHLH|bHLH family protein	KMT00016.1:hypothetical protein BVRB_1g018470 isoform A [Beta vulgaris subsp. vulgaris]	-	A0A0J8EAG6|A0A0J8EAG6_BETVU:Uncharacterized protein;OS=Beta vulgaris subsp. vulgaris.;

geneid	trend	GO	Pathway	TF	NR	NT	Uniprot
DN124568_c0_g4_i4	DOWN	[GO:0004568:chitinase activity][GO:0005975:carbohydrate metabolic process][GO:0016998:cell wall macromolecule catabolic process][GO:0006032:chitin catabolic process]	-	-	XP_021733257.1:chitinaselike protein 1 [Chenopodium quinoa]	gi|255549219|ref|XM_002515618.1|:Ricinus communis chitinase, putative, mRNA	A0A0J8CG05|A0A0J8CG05_BETVU:Uncharacterized protein;OS=Beta vulgaris subsp. vulgaris.;|GO:0004568,|GO:0005975,GO:0016998,GO:0006032,
DN128294_c5_g3_i1	DOWN	[GO:0007165:signal transduction]	-	-	XP_010696260.1:PREDICTED: ninjafamily protein 3 [Beta vulgaris subsp. vulgaris] >KMS97101.1 hypothetical protein BVRB_7g178830 [Beta vulgaris subsp. vulgaris]	-	A0A0J8B7X7|A0A0J8B7X7_BETVU:Uncharacterized protein;OS=Beta vulgaris subsp. vulgaris.;|GO:0007165,

geneid	trend	GO	Pathway	TF	NR	NT	Uniprot
DN116157_c1_g1_i1	DOWN	[GO:0005634:nucleus][GO:0009734:auxin-activated signaling pathway][GO:0006355:regulation of transcription, DNA-templated][GO:0006351:transcription, DNA-templated]	[ko04075:Plant hormone signal transduction]	Araha.3284s0025.1.p:Arabidopsis halleri|ARF|ARF family protein	XP_021742258.1:auxininduced protein 22Dlike [Chenopodium quinoa]	gi|521567967|gb|GAIA01074366.1|:TSA: Salicornia europaea Unigene90270_Se200S transcribed RNA sequence	A0A0J8FCS6|A0A0J8FCS6_BETVU:Auxin-responsive protein;OS=Beta vulgaris subsp. vulgaris.;|GO:0005634,|GO:0009734,GO:0006355,GO:0006351,
DN120033_c1_g1_i3	DOWN	[GO:0016021:integral component of membrane]	[ko04020:Calcium signaling pathway][ko04218: Cellular senescence][ko04621:NOD-like receptor signaling pathway]	Zmw_sc01544.1.g00110.1:Zoysia matrella|GRAS|GRAS family protein	XP_010680913.1:PREDICTED: calcium uniporter protein 2, mitochondrial isoform X2 [Beta vulgaris subsp. vulgaris] >KMT08501.1 hypothetical protein BVRB_6g137880 isoform B [Beta vulgaris subsp. vulgaris]	gi|356562328|ref|XM_003549376.1|:PREDICTED: Glycine max uncharacterized protein LOC100808882 (LOC100808882), mRNA	A0A0J8EZ60|A0A0J8EZ60_BETVU:Uncharacterized protein;OS=Beta vulgaris subsp. vulgaris.;|GO:0016021,

geneid	trend	GO	Pathway	TF	NR	NT	Uniprot
DN118578_c0_g1_i1	DOWN	[GO:0004568:chitinase activity][GO:0005975:carbohydrate metabolic process][GO:0016998:cell wall macromolecule catabolic process][GO:0006032:chitin catabolic process]	-	-	XP_021838352.1:chitinaselike protein 1 [Spinacia oleracea] >KNA13195.1 hypothetical protein SOVF_118900 [Spinacia oleracea]	gi|255549219|ref|XM_002515618.1|:Ricinus communis chitinase, putative, mRNA	A0A0K9R103|A0A0K9R103_SPIOL:Uncharacterized protein;OS=Spinacia oleracea;|GO:0004568,|GO:0005975,GO:0016998,GO:0006032,
DN115532_c0_g1_i2	DOWN	[GO:0005509:calcium ion binding]	[ko04626:Plant-pathogen interaction]	676720782:Sisymbrium irio|FAR1|FAR1 family protein	XP_021767597.1:probable calciumbinding protein CML43 [Chenopodium quinoa]	-	A0A075C736|A0A075C736_9CARY:Calcium binding protein;OS=Reaumuria trigyna.;|GO:0005509,
DN124568_c0_g5_i1	DOWN	[GO:0004568:chitinase activity][GO:0005975:carbohydrate metabolic process][GO:0016998:cell wall macromolecule catabolic process][GO:0006032:chitin catabolic process]	-	-	XP_009617372.1:PREDICTED: chitinaselike protein 2 [Nicotiana tomentosiformis]	-	A0A0D5ZAW8|A0A0D5ZAW8_9ROSA:Chitinase;OS=Pyrus ussuriensis.;|GO:0004568,|GO:0005975,GO:0016998,GO:0006032,

geneid	trend	GO	Pathway	TF	NR	NT	Uniprot
DN113944_c0_g1_i2	DOWN	[GO:0005524:ATP binding][GO:0004674:protein serine/threonine kinase activity]	[ko04064:NF-kappa B signaling pathway][ko04620:Toll-like receptor signaling pathway][ko04621:NOD-like receptor signaling pathway][ko04624:Toll and Imd signaling pathway][ko04722:Neurotrophin signaling pathway][ko05133:Pertussis][ko05140:Leishmaniasis][ko05142:Chagas disease (American trypanosomiasis)][ko05145:Toxoplasmosis][ko05152:Tuberculosis][ko05162:Measles][ko05164:Influenza A]	mrna28322.1-v1.0-hybrid:Fragaria vesca|HD-ZIP|HD-ZIP family protein	XP_010683239.1:PREDICTED: protein kinase 2B, chloroplastic [Beta vulgaris subsp. vulgaris] >KMT07111.1 hypothetical protein BVRB_6g154390 [Beta vulgaris subsp. vulgaris]	gi|224111269|ref|XM_002315763.1|:Populus trichocarpa predicted protein, mRNA	A0A0J8EVB4|A0A0J8EVB4_BETVU:Uncharacterized protein;OS=Beta vulgaris subsp. vulgaris.;|GO:0005524,GO:0004674,

geneid	trend	GO	Pathway	TF	NR	NT	Uniprot
DN121733_c2_g3_i5	DOWN	[GO:0005524:ATP binding][GO:0004672:protein kinase activity]	-	FANhyb_rscf00000217.1.g00006.1:Fragaria x ananassa|WRKY|WRKY family protein	XP_021716532.1:probable serine/threonineprotein kinase PBL12 [Chenopodium quinoa]	-	A0A0K9S4F5|A0A0K9S4F5_SPIOL:Uncharacterized protein;OS=Spinacia oleracea;|GO:0005524,GO:0004672,
DN122730_c0_g2_i4	DOWN	[GO:0003824:catalytic activity][GO:0005992:trehalose biosynthetic process]	[ko00500:Starch and sucrose metabolism]	-	XP_021718969.1:probable alpha,alphatrehalosephosphate synthase [UDPforming] 9 isoform X2 [Chenopodium quinoa]	gi|224121669|ref|XM_002318607.1|:Populus trichocarpa predicted protein, mRNA	A0A0J8CEY8|A0A0J8CEY8_BETVU:Uncharacterized protein;OS=Beta vulgaris subsp. vulgaris.;|GO:0003824,|GO:0005992,
DN116547_c0_g1_i1	DOWN	[GO:0004190:aspartic-type endopeptidase activity]	-	-	XP_010692959.1:PREDICTED: aspartyl protease family protein At5g10770 [Beta vulgaris subsp. vulgaris] >KMS99364.1 hypothetical protein BVRB_2g044890 [Beta vulgaris subsp.	gi|521737162|gb|GAIA01008273.1|:TSA: Salicornia europaea Unigene8604_Se200S transcribed RNA sequence	A0A0J8BDE2|A0A0J8BDE2_BETVU:Uncharacterized protein;OS=Beta vulgaris subsp. vulgaris.;|GO:0004190,

geneid	trend	GO	Pathway	TF	NR	NT	Uniprot
					vulgaris]		
DN120776_c1_g5_i1	DOWN	-	-	-	XP_021759521.1:protein SNOWY COTYLEDON 3like [Chenopodium quinoa]	-	A0A0K9QMI7|A0A0K9QMI7_SPIOL:Uncharacterized protein;OS=Spinacia oleracea;
DN119465_c0_g2_i1	DOWN	-	-	-	XP_010675837.1:PREDICTED: uncharacterized protein LOC104891780 [Beta vulgaris subsp. vulgaris] >KMT12860.1 hypothetical protein BVRB_4g089520 [Beta vulgaris subsp.	-	A0A0J8CGG8|A0A0J8CGG8_BETVU: Uncharacterized protein;OS=Beta vulgaris subsp. vulgaris.;

geneid	trend	GO	Pathway	TF	NR	NT	Uniprot
					vulgaris]		
DN126441_c0_g5_i7	DOWN	[GO:0005524:ATP binding][GO:0003883:CTP synthase activity][GO:0044210:'de novo' CTP biosynthetic process][GO:0006541:glutamine metabolic process]	[ko00240:Pyrimidine metabolism]	GSBRNA2T00038789001:Brassica napus|NAC|NAC family protein	XP_021764419.1:CTP synthaselike [Chenopodium quinoa]	gi|470115200|ref|XM_004293743.1|:PREDICTED: Fragaria vesca subsp. vesca CTP synthase 1-like (LOC101298776), mRNA	A0A0J8F364|A0A0J8F364_BETVU:CTP synthase;OS=Beta vulgaris subsp. vulgaris.;|GO:0005524,GO:0003883,|GO:0044210,GO:0006541,
DN122008_c9_g13_i1	DOWN	-	-	-	-	-	-
DN118864_c1_g4_i1	DOWN	[GO:0016021:integral component of membrane][GO:0005215:transporter activity]	-	Prupe.3G009400.1.p:Prunus persica|ERF|ERF family protein	XP_015931337.1:probable aquaporin PIPtype 7a [Arachis duranensis] >XP_016166069.1 probable aquaporin	gi|470143928|ref|XM_004307571.1|:PREDICTED: Fragaria vesca subsp. vesca probable aquaporin PIP-type 7a-like	O65356|O65356_SAMSA:Aquaporin 1;OS=Samanea saman;|GO:0016021,|GO:0005215,

geneid	trend	GO	Pathway	TF	NR	NT	Uniprot
					PIPtype 7a [Arachis ipaensis]	(LOC101295462), mRNA	
DN128831_c1_g3_i2	DOWN	[GO:0009507:chloroplast]	-	-	XP_010667558.1:PREDICTED: MARbinding filamentlike protein 1 isoform X1 [Beta vulgaris subsp. vulgaris] >KMS95283.1 hypothetical protein BVRB_009610 [Beta vulgaris subsp. vulgaris]	gi|521503191|gb|GAHZ01027729.1|:TSA: Salicornia europaea Unigene30530_SeCKS transcribed RNA sequence	A0A0J8B5W6|A0A0J8B5W6_BETVU: Uncharacterized protein;OS=Beta vulgaris subsp. vulgaris.;|GO:0009507,

geneid	trend	GO	Pathway	TF	NR	NT	Uniprot
DN117396_c6_g1_i2	DOWN	[GO:0048037:cofactor binding][GO:0004776:succinate-CoA ligase (GDP-forming) activity][GO:0008152:metabolic process]	[ko00020:Citrate cycle (TCA cycle)][ko00640:Propanoate metabolism]	-	XP_002514510.1:PREDICTED: succinylCoA ligase [ADPforming] subunit alpha2, mitochondrial [Ricinus communis] >EEF47910.1 SuccinylCoA ligase [GDPforming] subunit alpha2, mitochondrial precursor, putative [Ricinus communis]	gi|521739971|gb|GAIA01006633.1|:TSA: Salicornia europaea Unigene6944_Se200S transcribed RNA sequence	B9RL91|B9RL91_RICCO:Succinyl-CoA ligase [GDP-forming] subunit alpha-2, mitochondrial, putative;OS=Ricinus communis;|GO:0048037,GO:0004776,|GO:0008152,
DN125731_c4_g11_i1	DOWN	-	-	-	XP_021843002.1:uncharacterized protein LOC110783009 [Spinacia oleracea] >XP_021843003.1 uncharacterized protein LOC110783009 [Spinacia oleracea] >KNA21726.1 hypothetical protein	-	A0A0K9RQC0|A0A0K9RQC0_SPIOL: Uncharacterized protein;OS=Spinacia oleracea;

geneid	trend	GO	Pathway	TF	NR	NT	Uniprot
					SOVF_040710 [Spinacia oleracea]		
DN123060_c0_g5_i7	DOWN	[GO:0016021:integral component of membrane][GO:0004222:metalloendopeptidase activity]	[ko04141:Protein processing in endoplasmic reticulum]	Zmw_sc02218.1.g00160.1:Zoysia matrella|bHLH|bHLH family protein	XP_021842909.1:membranebound transcription factor site2 protease homolog isoform X1 [Spinacia oleracea] >KNA22565.1 hypothetical protein SOVF_032400 [Spinacia oleracea]	-	A0A0K9RSQ3|A0A0K9RSQ3_SPIOL:Uncharacterized protein;OS=Spinacia oleracea;|GO:0016021,|GO:0004222,

geneid	trend	GO	Pathway	TF	NR	NT	Uniprot
DN114359_c1_g1_i4	DOWN	[GO:0005737:cytoplasm][GO:0004427:inorganic diphosphatase activity][GO:0000287:magnesium ion binding][GO:0006796:phosphate-containing compound metabolic process]	[ko00190:Oxidative phosphorylation]	GSBRNA2T00073198001:Brassica napus|MYB_related|MYB_related family protein	XP_011019913.1:PREDICTED: soluble inorganic pyrophosphatase [Populus euphratica]	gi|388522380|gb|BT149458.1|:Lotus japonicus clone JCVI-FLLj-14J2 unknown mRNA	A0A0K9QUH3|A0A0K9QUH3_SPIOL: Uncharacterized protein;OS=Spinacia oleracea;|GO:0005737,|GO:0004427,GO:0000287,|GO:0006796,
DN118387_c0_g2_i2	DOWN	[GO:0016021:integral component of membrane]	-	-	XP_021852013.1:probable glycosyltransferase At5g03795 [Spinacia oleracea] >KNA07083.1 hypothetical protein SOVF_175180 [Spinacia oleracea]	-	A0A0K9QK36|A0A0K9QK36_SPIOL:Uncharacterized protein;OS=Spinacia oleracea;|GO:0016021,
DN120085_c3_g1_i3	DOWN	[GO:0005524:ATP binding][GO:0004672:protein kinase activity]	-	FANhyb_rscf00000217.1.g00006.1:Fragaria x ananassa|WRKY|WRKY family protein	XP_021866482.1:serine/threonineprotein kinase RIPKlike [Spinacia oleracea]	gi|53857146|gb|AY220481.2|:Nicotiana tabacum Avr9/Cf-9 induced kinase 1 (ACIK1) mRNA, complete cds	A0A0J8B8C8|A0A0J8B8C8_BETVU:Uncharacterized protein;OS=Beta vulgaris subsp. vulgaris.;|GO:0005524,GO:0004672,

geneid	trend	GO	Pathway	TF	NR	NT	Uniprot
DN115520_c0_g1_i5	DOWN	[GO:0008270:zinc ion binding]	-	-	XP_021736134.1:uncharacterized protein LOC110702685 [Chenopodium quinoa] >XP_021736135.1 uncharacterized protein LOC110702685 [Chenopodium quinoa]	-	A0A0K9QEG0|A0A0K9QEG0_SPIOL: Uncharacterized protein;OS=Spinacia oleracea;|GO:0008270,
DN129138_c7_g25_i1	DOWN	[GO:0005622:intracellular][GO:0008061:chitin binding][GO:0004568:chitinase activity][GO:0006040:amino sugar metabolic process][GO:0005975:carbohydrate metabolic process][GO:0016998:cell wall macromolecule catabolic process][GO:0006032:chitin catabolic process]	[ko00520:Amino sugar and nucleotide sugar metabolism]	-	XP_021675880.1:endochitinase PR4like [Hevea brasiliensis]	-	B9IQT0|B9IQT0_POPTR:Uncharacterized protein;OS=Populus trichocarpa;|GO:0005622,|GO:0008061,GO:0004568,|GO:0006040,GO:0005975,GO:0016998,GO:0006032,
DN112388_c5_g16_i1	DOWN	-	-	-	-	-	-

geneid	trend	GO	Pathway	TF	NR	NT	Uniprot
DN128952_c2_g1_i2	DOWN	[GO:0005507:copper ion binding][GO:0008131:primary amine oxidase activity][GO:0048038:quinone binding][GO:0009308:amine metabolic process]	[ko00260:Glycine, serine and threonine metabolism][ko00350:Tyrosine metabolism][ko00360:Phenylalanine metabolism][ko00410:beta-Alanine metabolism][ko00950:Isoquinoline alkaloid biosynthesis][ko00960:Tropane, piperidine and pyridine alkaloid biosynthesis]	-	XP_021853658.1:uncharacterized protein LOC110793123 [Spinacia oleracea] >KNA12504.1 hypothetical protein SOVF_125350 [Spinacia oleracea]	gi|521482802|gb|GAHZ01048084.1|:TSA: Salicornia europaea Unigene56608_SeCKS transcribed RNA sequence	A0A0K9QZ07|A0A0K9QZ07_SPIOL:Amine oxidase;OS=Spinacia oleracea;|GO:0005507,GO:0008131,GO:0048038,|GO:0009308,
DN106538_c0_g1_i1	DOWN	[GO:0016021:integral component of membrane][GO:0005215:transporter activity]	-	Prupe.3G009400.1.p:Prunus persica|ERF|ERF family protein	XP_021858736.1:aquaporin PIP21like isoform X1 [Spinacia oleracea] >KNA10168.1 hypothetical protein SOVF_146900 [Spinacia oleracea]	gi|1657947|gb|U73466.1|MCU73466:Mesembryanthemum crystallinum water channel protein MipC mRNA, complete cds	A0A0K9QTZ8|A0A0K9QTZ8_SPIOL:Uncharacterized protein;OS=Spinacia oleracea;|GO:0016021,|GO:0005215,

geneid	trend	GO	Pathway	TF	NR	NT	Uniprot
DN124270_c4_g5_i4	DOWN	[GO:0016491:oxidoreductase activity][GO:0008270:zinc ion binding]	[ko00940:Phenylpropanoid biosynthesis]	-	XP_021714044.1:probable mannitol dehydrogenase [Chenopodium quinoa]	gi|1724109|gb|U79770.1|MCU79770:Mesembryanthemum crystallinum cinnamyl-alcohol dehydrogenase Eli3 mRNA, complete cds	A0A0J8CAR2|A0A0J8CAR2_BETVU: Uncharacterized protein;OS=Beta vulgaris subsp. vulgaris.;|GO:0016491,GO:0008270,
DN124690_c1_g3_i4	DOWN	[GO:0004108:citrate (Si)-synthase activity][GO:0006101:citrate metabolic process][GO:0006099:tricarboxylic acid cycle]	[ko00020:Citrate cycle (TCA cycle)][ko00630:Glyoxylate and dicarboxylate metabolism]	RrC6748_p2:Raphanus raphanistrum|MYB_related|MYB_related family protein	XP_010691175.1:PREDICTED: citrate synthase, mitochondrial isoform X1 [Beta vulgaris subsp. vulgaris]	gi|1556379|emb|X84228.1|:B.vulgaris mRNA for citrate synthase	A0A0J8BI86|A0A0J8BI86_BETVU:Citrate synthase;OS=Beta vulgaris subsp. vulgaris.;|GO:0004108,|GO:0006101,GO:0006099,
DN120468_c4_g7_i3	DOWN	[GO:0003824:catalytic activity][GO:0050662:coenzyme binding]	-	-	XP_021772066.1:3oxoDelta(4,5)steroid 5betareductaselike [Chenopodium quinoa]	gi|156187110|gb|EF579963.1|:Arabidopsis thaliana progesterone 5-beta-reductase mRNA, complete cds	B9R730|B9R730_RICCO:Putative uncharacterized protein;OS=Ricinus communis;|GO:0003824,GO:0050662,

geneid	trend	GO	Pathway	TF	NR	NT	Uniprot
DN126434_c 1_g2_i4	DOWN	[GO:0016021:integral component of membrane]	-	Cotton_A_07948_BGI-A2_v1.0:Gossypium arboreum|BBR-BPC|BBR-BPC family protein	XP_010686787.1:PREDICTED: calcium permeable stressgated cation channel 1 [Beta vulgaris subsp. vulgaris] >XP_010686788.1 PREDICTED: calcium permeable stressgated cation channel 1 [Beta vulgaris subsp. vulgaris] >KMT04111.1 hypothetical protein BVRB_8g185610 [Beta vulgaris subsp. vulgaris]	gi|521451727|gb|GAHZ01068757.1|:TSA: Salicornia europaea Unigene82902_SeCKS transcribed RNA sequence	A0A0J8EMA5|A0A0J8EMA5_BETVU: Uncharacterized protein;OS=Beta vulgaris subsp. vulgaris.;|GO:0016021,

geneid	trend	GO	Pathway	TF	NR	NT	Uniprot
DN129138_c7_g24_i1	DOWN	[GO:0004568:chitinase activity][GO:0005975:carbohydrate metabolic process][GO:0016998:cell wall macromolecule catabolic process][GO:0006032:chitin catabolic process]	[ko00520:Amino sugar and nucleotide sugar metabolism]	-	AEM97876.1:class IV chitinase [Corylus heterophylla]	-	Q7M1Q9|Q7M1Q9_PHYAM:Chitinase A;OS=Phytolacca americana;|GO:0004568,|GO:0005975,GO:0016998,GO:0006032,
DN122740_c1_g7_i2	DOWN	[GO:0004190:aspartic-type endopeptidase activity][GO:0030163:protein catabolic process][GO:0006508:proteolysis]	-	-	XP_021757105.1:basic 7S globulinlike [Chenopodium quinoa]	-	B9HAK0|B9HAK0_POPTR:Uncharacterized protein;OS=Populus trichocarpa;|GO:0004190,|GO:0030163,GO:0006508,
DN126550_c2_g1_i4	DOWN	[GO:0016021:integral component of membrane][GO:0055085:transmembrane transport]	-	MDP0000295305:Malus domestica|NF-YC|NF-YC family protein	XP_010666854.1:PREDICTED: cation/calcium exchanger 4like [Beta vulgaris subsp. vulgaris]	-	A0A0J8B3R3|A0A0J8B3R3_BETVU:Uncharacterized protein;OS=Beta vulgaris subsp. vulgaris.;|GO:0016021,|GO:0055085,

geneid	trend	GO	Pathway	TF	NR	NT	Uniprot
DN122291_c0_g1_i1	DOWN	[GO:0016021:integral component of membrane]	-	-	XP_010689745.1:PREDICTED: uncharacterized protein LOC104903416 [Beta vulgaris subsp. vulgaris]	-	F4NCG5|F4NCG5_BETVU:Uncharacterized protein;OS=Beta vulgaris subsp. vulgaris.;|GO:0016021,
DN122100_c0_g2_i2	DOWN	[GO:0048046:apoplast][GO:0005618:cell wall][GO:0016021:integral component of membrane][GO:0004553:hydrolase activity, hydrolyzing O-glycosyl compounds][GO:0016762:xyloglucan:xyloglucosyl transferase activity][GO:0042546:cell wall biogenesis][GO:0071555:cell wall organization][GO:0010411:xyloglucan metabolic process]	-	Sme2.5_00096.1_g00018.1:Solanum melongena|NAC|NAC family protein	XP_010687463.1:PREDICTED: probable xyloglucan endotransglucosylase/hydrolase protein 23 [Beta vulgaris subsp. vulgaris] >KMT03680.1 hypothetical protein BVRB_8g190170 [Beta vulgaris subsp. vulgaris]	gi|403044152|ref|NM_001255019.2|:Glycine max probable xyloglucan endotransglucosylase/hydrolase protein 23-like (LOC100800017), mRNA	A0A0J8BQC9|A0A0J8BQC9_BETVU:Xyloglucan endotransglucosylase/hydrolase;OS=Beta vulgaris subsp. vulgaris.;|GO:0048046,GO:0005618,GO:0016021,|GO:0004553,GO:0016762,|GO:0042546,GO:0071555,GO:0010411,

geneid	trend	GO	Pathway	TF	NR	NT	Uniprot
DN125610_c6_g1_i3	DOWN	[GO:0005576:extracellular region][GO:0020037:heme binding][GO:0046872:metal ion binding][GO:0004601:peroxidase activity][GO:0042744:hydrogen peroxide catabolic process][GO:0006979:response to oxidative stress]	[ko00940:Phenylpropanoid biosynthesis]	EPS67213.1:Genlisea aurea|NAC|NAC family protein	XP_010695722.1:PREDICTED: peroxidase P7 [Beta vulgaris subsp. vulgaris] >KMT18887.1 hypothetical protein BVRB_2g029880 [Beta vulgaris subsp. vulgaris]	gi|5381252|dbj|AB027752.1|:Nicotiana tabacum mRNA for peroxidase, complete cds, clone:tpoxC1	A0A0J8CZ48|A0A0J8CZ48_BETVU:Peroxidase;OS=Beta vulgaris subsp. vulgaris.;|GO:0005576,|GO:0020037,GO:0046872,GO:0004601,|GO:0042744,GO:0006979,
DN112244_c0_g2_i1	DOWN	[GO:0016021:integral component of membrane][GO:0008270:zinc ion binding]	-	676788138:Sisymbrium irio|C3H|C3H family protein	XP_021757310.1:RING H2 finger protein ATL3like [Chenopodium quinoa]	-	A0A059ARJ2|A0A059ARJ2_EUCGR:Uncharacterized protein;OS=Eucalyptus grandis;|GO:0016021,|GO:0008270,
DN124684_c4_g3_i4	DOWN	-	-	-	XP_021740767.1:phosphatidylinositol/phosphatidylcholine transfer protein SFH2like [Chenopodium quinoa]	gi|521513546|gb|GAHZ01017374.1|:TSA: Salicornia europaea Unigene18313_SeCKS transcribed RNA sequence	A0A0J8CKM3|A0A0J8CKM3_BETVU:Uncharacterized protein;OS=Beta vulgaris subsp. vulgaris.;

geneid	trend	GO	Pathway	TF	NR	NT	Uniprot
DN115175_c0_g3_i1	DOWN	[GO:0004106:chorismate mutase activity][GO:0009073:aromatic amino acid family biosynthetic process][GO:0046417:chorismate metabolic process]	[ko00400:Phenylalanine, tyrosine and tryptophan biosynthesis]	-	XP_021739246.1:chorismate mutase 2like [Chenopodium quinoa]	-	A0A0K9QDU8|A0A0K9QDU8_SPIOL: Uncharacterized protein;OS=Spinacia oleracea;|GO:0004106,|GO:0009073,GO:0046417,
DN125332_c1_g2_i1	DOWN	[GO:0016788:hydrolase activity, acting on ester bonds]	-	-	XP_021716583.1:LOW QUALITY PROTEIN: GDSL esterase/lipase 2like [Chenopodium quinoa]	-	Q7Y055|Q7Y055_OXYRB:GDSL-lipase ;OS=Oxybasis rubra;|GO:0016788,
DN127142_c4_g3_i1	DOWN	[GO:0004553:hydrolase activity, hydrolyzing O-glycosyl compounds][GO:0005975:carbohydrate metabolic process]	[ko00520:Amino sugar and nucleotide sugar metabolism]	-	CDP17849.1:unnamed protein product [Coffea canephora]	-	A0A068VBB2|A0A068VBB2_COFCA: Uncharacterized protein;OS=Coffea canephora;|GO:0004553,|GO:0005975,
DN123525_c2_g9_i1	DOWN	[GO:0046872:metal ion binding]	-	Sp_115610_qwhk.t1:Spinacia oleracea|C2H2|C2H2 family protein	XP_021853286.1:zinc finger protein ZAT10like [Spinacia oleracea] >KNA13553.1 hypothetical protein	gi|199580248|gb|AC189555.2|:Brassica rapa subsp. pekinensis clone KBrH006E24, complete sequence	A0A0K9R216|A0A0K9R216_SPIOL:Uncharacterized protein;OS=Spinacia oleracea;|GO:0046872,

geneid	trend	GO	Pathway	TF	NR	NT	Uniprot
					SOVF_115610 [Spinacia oleracea]		
DN126520_c2_g6_i2	DOWN	-	-	-	-	-	-
DN117299_c2_g2_i18	DOWN	[GO:0016021:integral component of membrane][GO:0015171:amino acid transmembrane transporter activity]	[ko05206:MicroRNAs in cancer]	OPUNC03G25490.2:Oryza punctata|G2-like|G2-like family protein	XP_021851267.1:cationic amino acid transporter 4, vacuolarlike isoform X1 [Spinacia oleracea] >KNA10213.1 hypothetical protein SOVF_146560 [Spinacia oleracea]	gi|223943028|gb|BT060901.1|:Zea mays full-length cDNA clone ZM_BFb0068I04 mRNA, complete cds	A0A0K9QU37|A0A0K9QU37_SPIOL:Uncharacterized protein;OS=Spinacia oleracea;|GO:0016021,|GO:0015171,

geneid	trend	GO	Pathway	TF	NR	NT	Uniprot
DN118649_c4_g7_i1	DOWN	[GO:0016021:integral component of membrane]	-	-	XP_010680806.1:PREDICTED: uncharacterized protein LOC104895866 [Beta vulgaris subsp. vulgaris] >KMT09045.1 hypothetical protein BVRB_6g137490 [Beta vulgaris subsp. vulgaris]	gi|519688851|gb|JX582746.1|:Gossypium hirsutum clone NBRI_GE15641 microsatellite sequence	A0A0J8C5L3|A0A0J8C5L3_BETVU:Uncharacterized protein;OS=Beta vulgaris subsp. vulgaris.;|GO:0016021,
DN118441_c4_g4_i4	DOWN	[GO:0046872:metal ion binding][GO:0004722:protein serine/threonine phosphatase activity]	[ko04016:MAPK signaling pathway - plant][ko04075:Plant hormone signal transduction][ko04931:Insulin resistance]	KZV22762.1:Dorcoceras hygrometricum|CAMTA|CAMTA family protein	XP_010685683.1:PREDICTED: probable protein phosphatase 2C 24 [Beta vulgaris subsp. vulgaris] >KMT05175.1 hypothetical protein BVRB_7g173230 [Beta vulgaris subsp. vulgaris]	-	A0A0J8BUE2|A0A0J8BUE2_BETVU:Uncharacterized protein;OS=Beta vulgaris subsp. vulgaris.;|GO:0046872,GO:0004722,

geneid	trend	GO	Pathway	TF	NR	NT	Uniprot
DN126097_c4_g1_i6	DOWN	[GO:0005622:intracellular][GO:0004435:phosphatidylinositol phospholipase C activity][GO:0004871:obsolete signal transducer activity][GO:0035556:intracellular signal transduction][GO:0016042:lipid catabolic process]	[ko00562:Inositol phosphate metabolism][ko04020:Calcium signaling pathway][ko04070:Phosphatidylinositol signaling system][ko04919:Thyroid hormone signaling pathway][ko04933:AGE-RAGE signaling pathway in diabetic complications]	RrC2946_p1:Raphanus raphanistrum|C3H|C3H family protein	XP_010689668.1:PREDICTED: phosphoinositide phospholipase C 6 [Beta vulgaris subsp. vulgaris]	gi|351726115|ref|NM_001248395.1|:Glycine max phosphoinositide-specific phospholipase C P25 (LOC547865), mRNA >gi|1399306|gb|U41475.1|GMU41475 Glycine max phosphoinositide-specific phospholipase C P25 mRNA, complete cds	A0A0J8CWT6|A0A0J8CWT6_BETVU: Phosphoinositide phospholipase C;OS=Beta vulgaris subsp. vulgaris.;|GO:0005622,|GO:0004435,GO:0004871,|GO:0035556,GO:0016042,
DN127254_c1_g1_i3	DOWN	[GO:0005739:mitochondrion][GO:0005524:ATP binding][GO:0003777:microtubule motor activity][GO:0009060:aerobic respiration][GO:0007018:microtubule-based movement][GO:0009845:seed germination]	-	Araip.YX9Q9:Arachis ipaensis|MYB_related|MYB_related family protein	XP_010676106.1:PREDICTED: kinesinlike protein KIN14F [Beta vulgaris subsp. vulgaris] >KMT12643.1 hypothetical protein BVRB_4g091020 [Beta vulgaris subsp.	gi|511326059|gb|GAKE01006818.1|:TSA: Thlaspi arvense Contig6939 transcribed RNA sequence	A0A0J8CKS8|A0A0J8CKS8_BETVU:Uncharacterized protein;OS=Beta vulgaris subsp. vulgaris.;|GO:0005739,|GO:0005524,GO:0003777,|GO:0009060,GO:0007018,GO:0009845,

geneid	trend	GO	Pathway	TF	NR	NT	Uniprot
					vulgaris]		
DN123195_c1_g1_i4	DOWN	[GO:0016021:integral component of membrane][GO:0009506:plasmodesma]	-	-	XP_010687826.1:PREDICTED: uncharacterized protein LOC104901901 [Beta vulgaris subsp. vulgaris] >KMT02704.1 hypothetical protein BVRB_8g192950 [Beta vulgaris subsp. vulgaris]	gi|521448773|gb|GAHZ01071711.1|:TSA: Salicornia europaea Unigene87385_SeCKS transcribed RNA sequence	A0A0J8BRT1|A0A0J8BRT1_BETVU:Uncharacterized protein;OS=Beta vulgaris subsp. vulgaris.;|GO:0016021,GO:0009506,

geneid	trend	GO	Pathway	TF	NR	NT	Uniprot
DN115517_c0_g1_i2	DOWN	[GO:0005634:nucleus][GO:0003677:DNA binding][GO:0003700:DNA-binding transcription factor activity][GO:0006351:transcription, DNA-templated]	-	Sp_011340_ydgz.t1:Spinacia oleracea|ERF|ERF family protein	XP_021856079.1:ethyleneresponsive transcription factor 2like [Spinacia oleracea] >KNA24911.1 hypothetical protein SOVF_011340 [Spinacia oleracea]	gi|270308999|dbj|AB490000.1|:Citrullus lanatus subsp. vulgaris CitERF1a mRNA for ethylene responsive transcription factor 1a, complete cds	A0A0K9RZH6|A0A0K9RZH6_SPIOL:Uncharacterized protein;OS=Spinacia oleracea;|GO:0005634,|GO:0003677,GO:0003700,|GO:0006351,
DN85608_c0_g2_i1	DOWN	-	-	-	-	gi|521736612|gb|GAIA01008604.1|:TSA: Salicornia europaea Unigene8948_Se200S transcribed RNA sequence	-
DN128476_c3_g2_i2	DOWN	[GO:0016021:integral component of membrane][GO:0005524:ATP binding][GO:0004674:protein serine/threonine kinase activity]	-	MDP0000261185:Malus domestica|FAR1|FAR1 family protein	XP_021860781.1:probable LRR receptorlike serine/threonineprotein kinase At1g74360 [Spinacia oleracea] >KNA19118.1 hypothetical protein SOVF_064300	gi|502151982|ref|XM_004508650.1|:PREDICTED: Cicer arietinum probable LRR receptor-like serine/threonine-protein kinase At1g74360-like (LOC101496260), mRNA	A0A0K9RI05|A0A0K9RI05_SPIOL:Uncharacterized protein;OS=Spinacia oleracea;|GO:0016021,|GO:0005524,GO:0004674,

geneid	trend	GO	Pathway	TF	NR	NT	Uniprot
					[Spinacia oleracea]		
DN119373_c0_g3_i1	DOWN	[GO:0016021:integral component of membrane][GO:0006979:response to oxidative stress]	-	-	XP_021278994.1:uncharacterized protein LOC110412707 [Herrania umbratica]	gi|521485434|gb|GAHZ01045452.1|:TSA: Salicornia europaea Unigene53671_SeCKS transcribed RNA sequence	A0A061GR99|A0A061GR99_THECC:Uncharacterized protein;OS=Theobroma cacao;|GO:0016021,|GO:0006979,

geneid	trend	GO	Pathway	TF	NR	NT	Uniprot
DN127818_c2_g2_i4	DOWN	[GO:0033180:proton-transporting V-type ATPase, V1 domain][GO:0005524:ATP binding][GO:0015991:ATP hydrolysis coupled proton transport][GO:0046034:ATP metabolic process]	[ko00190:Oxidative phosphorylation][ko04145:Phagosome][ko04150:mTOR signaling pathway][ko04721:Synaptic vesicle cycle][ko04966:Collecting duct acid secretion][ko05110:Vibrio cholerae infection][ko05120:Epithelial cell signaling in Helicobacter pylori infection][ko05323:Rheumatoid arthritis]	-	CAD27443.1:vacuolar ATPase subunit B [Mesembryanthemum crystallinum]	gi|26986105|emb|AJ438590.1|:Mesembryanthemum crystallinum mRNA for vacuolar ATPase subunit B (vha-B gene)	A0A0K9RZD4|A0A0K9RZD4_SPIOL: Uncharacterized protein;OS=Spinacia oleracea;|GO:0033180,|GO:0005524,GO:0016820,|GO:0015991,GO:0046034,

geneid	trend	GO	Pathway	TF	NR	NT	Uniprot
DN125413_c3_g1_i2	DOWN	[GO:0016021:integral component of membrane][GO:0005622:intracellular][GO:0005215:transporter activity]	-	mrna15187.1-v1.0-hybrid:Fragaria vesca|bHLH|bHLH family protein	XP_010679780.1:PREDICTED: phosphatidylinositol/phosphatidylcholine transfer protein SFH12 [Beta vulgaris subsp. vulgaris] >XP_010679781.1 PREDICTED: phosphatidylinositol/phosphatidylcholine transfer protein SFH12 [Beta vulgaris subsp. vulgaris] >KMT09460.1 hypothetical protein BVRB_6g129320 [Beta vulgaris subsp. vulgaris]	gi|521510669|gb|GAHZ01020251.1|:TSA: Salicornia europaea Unigene21305_SeCKS transcribed RNA sequence	A0A0J8C6V3|A0A0J8C6V3_BETVU:Uncharacterized protein;OS=Beta vulgaris subsp. vulgaris.;|GO:0016021,GO:0005622,|GO:0005215,

geneid	trend	GO	Pathway	TF	NR	NT	Uniprot
DN116864_c0_g1_i8	DOWN	-	-	-	XP_010670735.1:PREDICTED: uncharacterized protein LOC104887719 [Beta vulgaris subsp. vulgaris] >KMT16865.1 hypothetical protein BVRB_2g043450 isoform A [Beta vulgaris subsp. vulgaris]	-	A0A0J8CSW6|A0A0J8CSW6_BETVU: Uncharacterized protein;OS=Beta vulgaris subsp. vulgaris.;
DN108910_c0_g1_i1	DOWN	-	-	-	XP_021718047.1:uncharacterized protein LOC110685827 [Chenopodium quinoa]	-	A0A0J8CBU6|A0A0J8CBU6_BETVU: Uncharacterized protein;OS=Beta vulgaris subsp. vulgaris.;

geneid	trend	GO	Pathway	TF	NR	NT	Uniprot
DN127654_c3_g4_i5	DOWN	[GO:0005783:endoplasmic reticulum][GO:0010211:IAA-Leu conjugate hydrolase activity][GO:0010210:IAA-Phe conjugate hydrolase activity][GO:0009850:auxin metabolic process]	-	XP_011071286.1:Sesamum indicum|C2H2|C2H2 family protein	XP_010683632.1:PREDICTED: IAAamino acid hydrolase ILR1 [Beta vulgaris subsp. vulgaris]	gi|359482029|ref|XM_002275830.2|:PREDICTED: Vitis vinifera IAA-amino acid hydrolase ILR1-like (LOC100266557), mRNA	A0A0J8BZN7|A0A0J8BZN7_BETVU:Uncharacterized protein;OS=Beta vulgaris subsp. vulgaris.;|GO:0005783,|GO:0010211,GO:0010210,|GO:0009850,
DN127099_c6_g6_i2	DOWN	[GO:0005634:nucleus][GO:0004402:histone acetyltransferase activity][GO:0003712:transcription coregulator activity][GO:0008270:zinc ion binding][GO:0006355:regulation of transcription, DNA-templated]	-	GSMUA_Achr10P03980_001:Musa acuminata|C2H2|C2H2 family protein	XP_010695978.1:PREDICTED: BTB/POZ and TAZ domaincontaining protein 2 [Beta vulgaris subsp. vulgaris]	gi|521738988|gb|GAIA01007306.1|:TSA: Salicornia europaea Unigene7620_Se200S transcribed RNA sequence	A0A0K9QM81|A0A0K9QM81_SPIOL: Uncharacterized protein;OS=Spinacia oleracea;|GO:0005634,|GO:0004402,GO:0003712,GO:0008270,|GO:0006355,
DN122113_c2_g11_i1	DOWN	-	-	-	-	-	-

geneid	trend	GO	Pathway	TF	NR	NT	Uniprot
DN126103_c2_g3_i5	DOWN	[GO:0016021:integral component of membrane][GO:0015297:antiporter activity]	-	-	XP_021728984.1:protein DETOXIFICATION 42like [Chenopodium quinoa]	gi|521444343|gb|GAHZ01076141.1|:TSA: Salicornia europaea Unigene93078_SeCKS transcribed RNA sequence	A0A0J8CLU8|A0A0J8CLU8_BETVU:Protein DETOXIFICATION;OS=Beta vulgaris subsp. vulgaris.;|GO:0016021,|GO:0015297,GO:0015238,
DN119551_c1_g1_i2	DOWN	[GO:0005524:ATP binding]	-	Zmw_sc02343.1.g00060.1:Zoysia matrella|NAC|NAC family protein	XP_021714566.1:AAAATPase At3g50940like [Chenopodium quinoa]	gi|255547775|ref|XM_002514899.1|:Ricinus communis ATP binding protein, putative, mRNA	A0A0J8CBA2|A0A0J8CBA2_BETVU: Uncharacterized protein;OS=Beta vulgaris subsp. vulgaris.;|GO:0005524,
DN126994_c2_g15_i1	DOWN	-	-	-	-	-	-
DN121064_c2_g1_i2	DOWN	[GO:0006355:regulation of transcription, DNA-templated][GO:0006351:transcription, DNA-templated]	-	Sp_036000_ggiy.t1: Spinacia oleracea|GRAS|GRAS family protein	XP_021726697.1:scarecrowlike protein 8 [Chenopodium quinoa]	-	A0A0K9RTG0|A0A0K9RTG0_SPIOL: Uncharacterized protein;OS=Spinacia oleracea;|GO:0006355,GO:0006351,

geneid	trend	GO	Pathway	TF	NR	NT	Uniprot
DN124100_c6_g9_i1	DOWN	-	-	-	-	-	-
DN125339_c2_g1_i1	DOWN	[GO:0009507:chloroplast][GO:0005739:mitochondrion][GO:0005524:ATP binding][GO:0050897:cobalt ion binding][GO:0004471:malate dehydrogenase (decarboxylating) (NAD+) activity][GO:0051287:NAD binding][GO:0008270:zinc ion binding][GO:0006108:malate metabolic process]	[ko00620:Pyruvate metabolism][ko00710:Carbon fixation in photosynthetic organisms]	-	XP_010671885.1:PREDICTED: NADdependent malic enzyme 59 kDa isoform, mitochondrial [Beta vulgaris subsp. vulgaris] >KMT20599.1 hypothetical protein BVRB_1g005810 [Beta vulgaris subsp. vulgaris]	-	A0A0J8D8C5|A0A0J8D8C5_BETVU:Malic enzyme;OS=Beta vulgaris subsp. vulgaris.;|GO:0009507,GO:0005739,|GO:0005524,GO:0050897,GO:0004471,GO:0051287,GO:0008270,|GO:0006108,
DN119061_c2_g1_i5	DOWN	[GO:0046872:metal ion binding][GO:0004722:protein serine/threonine phosphatase activity]	[ko04016:MAPK signaling pathway - plant][ko04075:Plant hormone signal transduction][ko04931:Insulin resistance]	XP_010536267.1:Tarenaya hassleriana|NAC|NAC family protein	XP_021729058.1:probable protein phosphatase 2C 8 [Chenopodium quinoa]	-	A0A0K9QQG8|A0A0K9QQG8_SPIOL:Uncharacterized protein;OS=Spinacia oleracea;|GO:0046872,GO:0004722,

geneid	trend	GO	Pathway	TF	NR	NT	Uniprot
DN123815_c0_g1_i4	DOWN	[GO:0016021:integral component of membrane][GO:0009055:electron transfer activity]	-	-	XP_021734267.1:blue copper proteinlike [Chenopodium quinoa]	-	A0A0J8FFN4|A0A0J8FFN4_BETVU:Uncharacterized protein;OS=Beta vulgaris subsp. vulgaris.;|GO:0016021,|GO:0009055,
DN116885_c0_g2_i1	DOWN	[GO:0008270:zinc ion binding]	-	Pp3c15_4950V3.1.p:Physcomitrella patens|C2H2|C2H2 family protein	XP_021741260.1:zinc finger AN1 domaincontaining stressassociated protein 12like [Chenopodium quinoa]	-	A0A067GA40|A0A067GA40_CITSI:Uncharacterized protein;OS=Citrus sinensis;|GO:0008270,
DN124251_c0_g11_i1	DOWN	[GO:0004869:cysteine-type endopeptidase inhibitor activity]	[ko04970:Salivary secretion]	-	XP_010686713.1:PREDICTED: cysteine proteinase inhibitor 1 [Beta vulgaris subsp. vulgaris] >KMT04191.1 hypothetical protein BVRB_8g184860 [Beta vulgaris subsp. vulgaris]	-	A0A0J8EMH9|A0A0J8EMH9_BETVU: Cysteine proteinase inhibitor;OS=Beta vulgaris subsp. vulgaris.;|GO:0004869,

geneid	trend	GO	Pathway	TF	NR	NT	Uniprot
DN123327_c2_g2_i5	DOWN	[GO:0046872:metal ion binding][GO:0030001:metal ion transport]	-	-	XP_010674786.1:PREDICTED: heavy metalassociated isoprenylated plant protein 32like [Beta vulgaris subsp. vulgaris]	-	A0A0J8CJS6|A0A0J8CJS6_BETVU:Uncharacterized protein;OS=Beta vulgaris subsp. vulgaris.;|GO:0046872,|GO:0030001,
DN120639_c0_g2_i6	DOWN	[GO:0016021:integral component of membrane]	-	mrna32536.1-v1.0-hybrid:Fragaria vesca|bHLH|bHLH family protein	XP_021760455.1:embryogenesisassociated protein EMB8like [Chenopodium quinoa]	gi|521448119|gb|GAHZ01072365.1|:TSA: Salicornia europaea Unigene88104_SeCKS transcribed RNA sequence	A0A0J8FWM4|A0A0J8FWM4_BETVU: Uncharacterized protein;OS=Beta vulgaris subsp. vulgaris.;|GO:0016021,
DN114918_c0_g1_i1	DOWN	[GO:0005737:cytoplasm][GO:0004791:thioredoxin-disulfide reductase activity][GO:0045454:cell redox homeostasis]	-	-	OMO75478.1:Thioredoxinlike protein [Corchorus olitorius]	gi|502131229|ref|XM_004500860.1|:PREDICTED: Cicer arietinum probable nucleoredoxin 2-like (LOC101498505), transcript variant X2, mRNA	F6H0E3|F6H0E3_VITVI:Putative uncharacterized protein;OS=Vitis vinifera;GN=VIT_18s0001g01030 {ECO:0000313|EMBL:CCB45415.1};|GO:0005737,|GO:0004791,|GO:0045454,

geneid	trend	GO	Pathway	TF	NR	NT	Uniprot
DN108307_c0_g1_i2	DOWN	[GO:0016021:integral component of membrane][GO:0016491:oxidoreductase activity]	[ko00520:Amino sugar and nucleotide sugar metabolism]	mrna31001.1-v1.0-hybrid:Fragaria vesca|C3H|C3H family protein	XP_008231098.1:PREDICTED: NADHcytochrome b5 reductase 1like [Prunus mume]	-	M5W5N6|M5W5N6_PRUPE:Uncharacterized protein;OS=Prunus persica;|GO:0016021,|GO:0016491,
DN115781_c0_g3_i1	DOWN	[GO:0005576:extracellular region][GO:0020037:heme binding][GO:0046872:metal ion binding][GO:0004601:peroxidase activity][GO:0042744:hydrogen peroxide catabolic process][GO:0006979:response to oxidative stress]	[ko00940:Phenylpropanoid biosynthesis]	EPS67213.1:Genlisea aurea|NAC|NAC family protein	XP_021737720.1:peroxidase 12like [Chenopodium quinoa]	gi|1781325|emb|Y10464.1|:S.oleracea mRNA for peroxidase, clone PC42	A0A0K9QU56|A0A0K9QU56_SPIOL:Peroxidase;OS=Spinacia oleracea;|GO:0005576,|GO:0020037,GO:0046872,GO:0004601,|GO:0042744,GO:0006979,
DN124018_c1_g3_i13	DOWN	-	-	Achn202071:Actinidia chinensis|MYB_related|MYB_related family protein	XP_021718943.1:protein KINESIN LIGHT CHAINRELATED 3like [Chenopodium quinoa]	gi|359491583|ref|XM_002279627.2|:PREDICTED: Vitis vinifera uncharacterized LOC100255253 (LOC100255253), mRNA	A0A0J8CEL7|A0A0J8CEL7_BETVU:Uncharacterized protein;OS=Beta vulgaris subsp. vulgaris.;

geneid	trend	GO	Pathway	TF	NR	NT	Uniprot
DN128965_c3_g1_i1	DOWN	-	-	-	-	-	-
DN114571_c2_g3_i3	DOWN	-	-	-	-	-	-
DN123582_c2_g3_i4	DOWN	-	-	-	-	-	-

geneid	trend	GO	Pathway	TF	NR	NT	Uniprot
DN127998_c1_g3_i2	DOWN	[GO:0005783:endoplasmic reticulum][GO:0018472:1-hydroxy-2-naphthaldehyde dehydrogenase activity][GO:0034525:1-naphthaldehyde dehydrogenase activity][GO:0044104:2,5-dioxovalerate dehydrogenase (NAD+) activity][GO:0018474:2-carboxybenzaldehyde dehydrogenase activity][GO:0034520:2-naphthaldehyde dehydrogenase activity][GO:0004028:3-chloroallyl aldehyde dehydrogenase activity][GO:0034538:3-methylsalicylaldehyde dehydrogenase activity][GO:0034530:4-hydroxymethylsalicyaldehyde dehydrogenase activity][GO:0019115:benzaldehyde dehydrogenase activity][GO:0018473:cis-2-methyl-5-isopropylhexa-2,5-dienal dehydrogenase activity][GO:0034822:citronellal dehydrogenase activity][GO:0080019:fatty-acyl-CoA reductase (alcohol-forming) activity][GO:0034832:geranial dehydrogenase activity][GO:0043796:glyceraldehyde dehydrogenase (NADP) activity][GO:0043878:glyceraldehyde	[ko00073:Cutin, suberine and wax biosynthesis][ko04146:Peroxisome][ko04212:Longevity regulating pathway - worm]	-	XP_021716399.1:alcoholforming fatty acylCoA reductaselike [Chenopodium quinoa]	gi|297798545|ref|XM_002867111.1|:Arabidopsis lyrata subsp. lyrata predicted protein, mRNA	A0A0J8C022|A0A0J8C022_BETVU:Fatty acyl-CoA reductase;OS=Beta vulgaris subsp. vulgaris.;|GO:0005783,|GO:0018472,GO:0034525,GO:0044104,GO:0018474,GO:0034520,GO:0004028,GO:0034538,GO:0034530,GO:0019115,GO:0018473,GO:0034822,GO:0080019,GO:0034832,GO:0043796,GO:0043878,GO:0052814,GO:0043870,GO:0043745,GO:0034602,GO:0034601,GO:0034604,GO:0018475,|GO:0006629,GO:0010025,

geneid	trend	GO	Pathway	TF	NR	NT	Uniprot
DN118246_c0_g2_i1	DOWN	[GO:0005777:peroxisome][GO:0005507:copper ion binding][GO:0008131:primary amine oxidase activity][GO:0048038:quinone binding][GO:0009308:amine metabolic process]	[ko00260:Glycine, serine and threonine metabolism][ko00350:Tyrosine metabolism][ko00360:Phenylalanine metabolism][ko00410:beta-Alanine metabolism][ko00950:Isoquinoline alkaloid biosynthesis][ko00960:Tropane, piperidine and pyridine alkaloid biosynthesis]	-	XP_010682433.1:PREDICTED: uncharacterized protein LOC104897280 [Beta vulgaris subsp. vulgaris]	gi|460398431|ref|XM_004244715.1|:PREDICTED: Solanum lycopersicum copper methylamine oxidase-like (LOC101254319), mRNA	A0A0J8C1P7|A0A0J8C1P7_BETVU:Amine oxidase;OS=Beta vulgaris subsp. vulgaris.;|GO:0005777,|GO:0005507,GO:0008131,GO:0048038,|GO:0009308,
DN119025_c0_g2_i5	DOWN	[GO:0008270:zinc ion binding]	-	-	XP_021763155.1:probable E3 ubiquitinprotein ligase BAH1like 1 [Chenopodium quinoa]	gi|224068329|ref|XM_002302672.1|:Populus trichocarpa predicted protein, mRNA	A0A0J8ES68|A0A0J8ES68_BETVU:Uncharacterized protein;OS=Beta vulgaris subsp. vulgaris.;|GO:0008270,

geneid	trend	GO	Pathway	TF	NR	NT	Uniprot
DN122915_c2_g2_i5	DOWN	[GO:0046872:metal ion binding][GO:0016491:oxidoreductase activity]	[ko00941:Flavonoid biosynthesis]	676709864:Sisymbrium irio|STAT|STAT family protein	XP_010679937.1:PREDICTED: naringenin,2oxoglutarate 3dioxygenase [Beta vulgaris subsp. vulgaris] >KMT09612.1 hypothetical protein BVRB_6g130650 [Beta vulgaris subsp. vulgaris]	gi|134039063|gb|EF468104.1|:Dimocarpus longan flavanone-3-hydroxylase (f3h) mRNA, complete cds	A0A0J8C7R1|A0A0J8C7R1_BETVU:Uncharacterized protein;OS=Beta vulgaris subsp. vulgaris.;|GO:0046872,GO:0016491,
DN120597_c2_g1_i3	DOWN	[GO:0020037:heme binding][GO:0005506:iron ion binding][GO:0004497:monooxygenase activity][GO:0016705:oxidoreductase activity, acting on paired donors, with incorporation or reduction of molecular oxygen]	[ko00592:alpha-Linolenic acid metabolism]	-	XP_021738527.1:allene oxide synthase 2like isoform X2 [Chenopodium quinoa]	gi|123649877|emb|AM448726.1|:Vitis vinifera contig VV78X182466.9, whole genome shotgun sequence	A0A0K9QAG2|A0A0K9QAG2_SPIOL: Uncharacterized protein;OS=Spinacia oleracea;|GO:0020037,GO:0005506,GO:0004497,GO:0016705,

geneid	trend	GO	Pathway	TF	NR	NT	Uniprot
DN128916_c1_g2_i1	DOWN	-	-	-	XP_010102639.1:hypothetical protein L484_010931 [Morus notabilis] >EXB93789.1 hypothetical protein L484_010931 [Morus notabilis]	-	W9RRW0|W9RRW0_9ROSA:Uncharacterized protein;OS=Morus notabilis.;
DN128121_c3_g1_i2	DOWN	[GO:0016021:integral component of membrane][GO:0005215:transporter activity][GO:0006857:oligopeptide transport]	-	Sopen09g001330.1:Solanum pennellii|G2-like|G2-like family protein	KNA06466.1:hypothetical protein SOVF_180790 [Spinacia oleracea]	-	A0A0K9QIP4|A0A0K9QIP4_SPIOL:Uncharacterized protein;OS=Spinacia oleracea;|GO:0016021,|GO:0005215,|GO:0006857,
DN117616_c0_g1_i2	DOWN	[GO:0009507:chloroplast][GO:0016020:membrane][GO:0003849:3-deoxy-7-phosphoheptulonate synthase activity][GO:0009073:aromatic amino acid family biosynthetic process][GO:0008652:cellular amino acid biosynthetic process]	[ko00400:Phenylalanine, tyrosine and tryptophan biosynthesis][ko02024:Quorum sensing]	-	XP_010676056.1:PREDICTED: phospho2dehydro3deoxyheptonate aldolase 1, chloroplastic [Beta vulgaris subsp. vulgaris] >KMT19815.1 hypothetical protein	gi|521703112|gb|GAIA01016369.1|:TSA: Salicornia europaea Unigene17088_Se200S transcribed RNA sequence	A0A0J8D0Z8|A0A0J8D0Z8_BETVU:Phospho-2-dehydro-3-deoxyheptonate aldolase;OS=Beta vulgaris subsp. vulgaris.;|GO:0009507,GO:0016020,|GO:0003849,|GO:0009073,GO:0008652,

geneid	trend	GO	Pathway	TF	NR	NT	Uniprot
					BVRB_1g008670 [Beta vulgaris subsp. vulgaris]		
DN119721_c0_g2_i2	DOWN	[GO:0003824:catalytic activity][GO:0008152:metabolic process]	[ko00010:Glycolysis / Gluconeogenesis][ko00260:Glycine, serine and threonine metabolism][ko00680:Methane metabolism]	-	XP_021860792.1:phosphoglycerate mutaselike protein 4 [Spinacia oleracea] >KNA24192.1 hypothetical protein SOVF_017860 [Spinacia oleracea]	-	A0A0K9RZB2|A0A0K9RZB2_SPIOL:Uncharacterized protein;OS=Spinacia oleracea;|GO:0003824,|GO:0008152,

geneid	trend	GO	Pathway	TF	NR	NT	Uniprot
DN82717_c0_g1_i1	DOWN	[GO:0020037:heme binding][GO:0005506:iron ion binding][GO:0004497:monooxygenase activity][GO:0016705:oxidoreductase activity, acting on paired donors, with incorporation or reduction of molecular oxygen]	-	-	XP_021850062.1:alkane hydroxylase MAH1like [Spinacia oleracea]	-	A0A0J8C9H2|A0A0J8C9H2_BETVU:Uncharacterized protein;OS=Beta vulgaris subsp. vulgaris.;|GO:0020037,GO:0005506,GO:0004497,GO:0016705,

geneid	trend	GO	Pathway	TF	NR	NT	Uniprot
DN115243_c1_g1_i5	DOWN	[GO:0046872:metal ion binding][GO:0004721:phosphoprotein phosphatase activity]	[ko03015:mRNA surveillance pathway][ko04022:cGMP-PKG signaling pathway][ko04024:cAMP signaling pathway][ko04113:Meiosis - yeast][ko04114:Oocyte meiosis][ko04218:Cellular senescence][ko04261:Adrenergic signaling in cardiomyocytes][ko04270:Vascular smooth muscle contraction][ko04390:Hippo signaling pathway][ko04510:Focal adhesion][ko04611:Platelet activation][ko04720:Long-term potentiation][ko04728:Dopaminergic synapse][ko04750:Inflammatory mediator regulation of TRP	Jcr4S06752.20:Jatropha curcas|FAR1|FAR1 family protein	XP_021742589.1:serine/threonineprotein phosphatase PP1 isozyme 9like [Chenopodium quinoa]	gi|209879997|ref|XM_002141403.1|:Cryptosporidium muris RN66 serine/threonine protein phosphatase, putative, mRNA	A0A0K9RCG4|A0A0K9RCG4_SPIOL:Serine/threonine-protein phosphatase;OS=Spinacia oleracea;|GO:0046872,GO:0004721,

geneid	trend	GO	Pathway	TF	NR	NT	Uniprot
DN107431_c0_g1_i1	DOWN	-	-	-	XP_010684800.1:PREDICTED: uncharacterized protein LOC104899325 [Beta vulgaris subsp. vulgaris] >KMT05787.1 hypothetical protein BVRB_7g166260 [Beta vulgaris subsp. vulgaris]	-	A0A0J8BX59|A0A0J8BX59_BETVU:Uncharacterized protein;OS=Beta vulgaris subsp. vulgaris.;
DN119897_c6_g1_i4	DOWN	-	-	-	XP_021857297.1:BRO1 domaincontaining protein BROXlike [Spinacia oleracea] >XP_021857298.1 BRO1 domaincontaining protein BROXlike [Spinacia oleracea] >KNA16292.1 hypothetical protein SOVF_090290	-	A0A0K9RBX6|A0A0K9RBX6_SPIOL:Uncharacterized protein;OS=Spinacia oleracea;

geneid	trend	GO	Pathway	TF	NR	NT	Uniprot
					[Spinacia oleracea]		
DN125731_c4_g7_i1	DOWN	-	-	-	-	-	-
DN87124_c0_g1_i1	DOWN	[GO:0030598:rRNA N-glycosylase activity][GO:0006952:defense response][GO:0017148:negative regulation of translation]	-	-	AAL35962.1:bouganin [Bougainvillea spectabilis]	gi|17149253|gb|AF445416.1|AF445416: Bougainvillea spectabilis bouganin mRNA, complete cds	Q8W4U4|Q8W4U4_9CARY:rRNA N-glycosidase;OS=Bougainvillea spectabilis.;|GO:0030598,|GO:0006952, GO:0017148,

geneid	trend	GO	Pathway	TF	NR	NT	Uniprot
DN126891_c2_g2_i6	DOWN	[GO:0009507:chloroplast]	-	mrna13277.1-v1.0-hybrid:Fragaria vesca|MIKC_MADS|MIKC_MADS family protein	XP_010667558.1:PREDICTED: MARbinding filamentlike protein 1 isoform X1 [Beta vulgaris subsp. vulgaris] >KMS95283.1 hypothetical protein BVRB_009610 [Beta vulgaris subsp. vulgaris]	-	A0A0J8B5W6|A0A0J8B5W6_BETVU: Uncharacterized protein;OS=Beta vulgaris subsp. vulgaris.;|GO:0009507,
DN127595_c2_g1_i1	DOWN	[GO:0003989:acetyl-CoA carboxylase activity][GO:0005524:ATP binding][GO:0004075:biotin carboxylase activity][GO:0046872:metal ion binding][GO:0006633:fatty acid biosynthetic process]	[ko00061:Fatty acid biosynthesis][ko00254:Aflatoxin biosynthesis][ko00620:Pyruvate metabolism][ko00640:Propanoate metabolism][ko04152:AMPK signaling pathway][ko04910:Insulin signaling pathway][ko04922:Glucagon signaling	-	XP_010683396.1:PREDICTED: acetylCoA carboxylase 1 isoform X1 [Beta vulgaris subsp. vulgaris]	gi|225459363|ref|XM_002285772.1|:PREDICTED: Vitis vinifera acetyl-CoA carboxylase 1-like (LOC100247288), mRNA	A0A0J8CZX5|A0A0J8CZX5_BETVU:Uncharacterized protein;OS=Beta vulgaris subsp. vulgaris.;|GO:0003989,GO:0005524,GO:0004075,GO:0046872,|GO:0006633,

geneid	trend	GO	Pathway	TF	NR	NT	Uniprot
			pathway]				
DN122523_c0_g4_i1	DOWN	-	-	-	-	-	-
DN116018_c1_g3_i1	DOWN	[GO:0016021:integral component of membrane][GO:0005886:plasma membrane][GO:0016760:cellulose synthase (UDP-forming) activity][GO:0008270:zinc ion binding][GO:0071555:cell wall organization][GO:0030244:cellulose biosynthetic process]	-	CCG027008.3:Populus euphratica|G2-like|G2-like family protein	XP_021771565.1:cellulose synthase A catalytic subunit 6 [UDPforming]like [Chenopodium quinoa]	gi|356559630|ref|XM_003548054.1|:PREDICTED: Glycine max cellulose synthase A catalytic subunit 6 [UDP-forming]-like (LOC100798759), mRNA	A0A0J8F408|A0A0J8F408_BETVU:Cellulose synthase;OS=Beta vulgaris subsp. vulgaris.;|GO:0016021,GO:0005886,|GO:0016760,GO:0008270,|GO:0071555,GO:0030244,

geneid	trend	GO	Pathway	TF	NR	NT	Uniprot
DN126731_c2_g1_i1	DOWN	[GO:0016021:integral component of membrane][GO:0005524:ATP binding][GO:0004672:protein kinase activity]	-	Sme2.5_00580.1_g00006.1:Solanum melongena|CO-like|CO-like family protein	XP_021738612.1:LOW QUALITY PROTEIN: probable LRR receptorlike serine/threonineprotein kinase At2g16250 [Chenopodium quinoa]	gi|255536844|ref|XM_002509443.1|:Ricinus communis lrr receptor protein kinase, putative, mRNA	A0A0K9R4Q5|A0A0K9R4Q5_SPIOL:Uncharacterized protein;OS=Spinacia oleracea;|GO:0016021,|GO:0005524,GO:0004672,
DN119798_c0_g7_i6	DOWN	-	-	-	-	-	-
DN121524_c0_g1_i1	DOWN	[GO:0043565:sequence-specific DNA binding][GO:0003700:DNA-binding transcription factor activity]	-	Bv3_053880_mpkc.t1:Beta vulgaris|bZIP|bZIP family protein	XP_010671685.1:PREDICTED: bZIP transcription factor 53 [Beta vulgaris subsp. vulgaris] >KMT16231.1 hypothetical protein BVRB_3g053880 [Beta vulgaris subsp. vulgaris]	-	A0A0J8FJZ7|A0A0J8FJZ7_BETVU:Uncharacterized protein;OS=Beta vulgaris subsp. vulgaris.;|GO:0043565,GO:0003700,

geneid	trend	GO	Pathway	TF	NR	NT	Uniprot
DN119465_c0_g1_i7	DOWN	-	-	-	XP_002524160.1:PREDICTED: uncharacterized protein LOC8274807 [Ricinus communis] >EEF38223.1 conserved hypothetical protein [Ricinus communis]	-	B9SDU1|B9SDU1_RICCO:Putative uncharacterized protein;OS=Ricinus communis;
DN128542_c0_g1_i10	DOWN	-	-	-	XP_010682959.1:PREDICTED: uncharacterized protein LOC104897720 isoform X1 [Beta vulgaris subsp. vulgaris]	-	A0A0J8BZA1|A0A0J8BZA1_BETVU:Uncharacterized protein;OS=Beta vulgaris subsp. vulgaris.;
DN121806_c0_g7_i2	DOWN	[GO:0008234:cysteine-type peptidase activity]	-	FANhyb_rscf00000253.1.g00010.1:Fragaria x ananassa|BBR-BPC|BBR-BPC family protein	AHA62778.1:RCR3like cysteine protease [Mirabilis jalapa]	-	V5M3B7|V5M3B7_MIRJA:RCR3-like cysteine protease;OS=Mirabilis jalapa;|GO:0008234,

geneid	trend	GO	Pathway	TF	NR	NT	Uniprot
DN117251_c1_g2_i1	DOWN	[GO:0016620:oxidoreductase activity, acting on the aldehyde or oxo group of donors, NAD or NADP as acceptor]	[ko00940:Phenylpropanoid biosynthesis]	Lus10030143:Linum usitatissimum|NAC|NAC family protein	XP_010688154.1:PREDICTED: aldehyde dehydrogenase family 2 member C4 [Beta vulgaris subsp. vulgaris] >KMT03022.1 hypothetical protein BVRB_8g195850 [Beta vulgaris subsp. vulgaris]	gi|521734154|gb|GAIA01009835.1|:TSA: Salicornia europaea Unigene10290_Se200S transcribed RNA sequence	A0A0J8BP52|A0A0J8BP52_BETVU:Uncharacterized protein;OS=Beta vulgaris subsp. vulgaris.;|GO:0016620,
DN122008_c9_g7_i1	DOWN	-	-	-	-	-	-
DN126084_c1_g1_i6	DOWN	[GO:0005524:ATP binding][GO:0004672:protein kinase activity]	-	-	XP_010672750.1:PREDICTED: serine/threonineprotein kinase Nek6 [Beta vulgaris subsp. vulgaris] >KMT15398.1 hypothetical protein BVRB_3g061020 [Beta vulgaris subsp. vulgaris]	gi|521600368|gb|GAIA01064458.1|:TSA: Salicornia europaea Unigene77446_Se200S transcribed RNA sequence	A0A0J8CTV4|A0A0J8CTV4_BETVU:Uncharacterized protein;OS=Beta vulgaris subsp. vulgaris.;|GO:0005524,GO:0004672,

geneid	trend	GO	Pathway	TF	NR	NT	Uniprot
DN104858_c0_g1_i1	DOWN	-	-	-	XP_021732076.1:uncharacterized protein LOC110698867 [Chenopodium quinoa]	-	A0A0K9S1P3|A0A0K9S1P3_SPIOL:Uncharacterized protein;OS=Spinacia oleracea;
DN116217_c4_g1_i2	DOWN	[GO:0016021:integral component of membrane]	-	-	XP_010691065.1:PREDICTED: uncharacterized protein At4g15970 isoform X1 [Beta vulgaris subsp. vulgaris] >KMT00604.1 hypothetical protein BVRB_9g218990 [Beta vulgaris subsp. vulgaris]	gi|521440786|gb|GAHZ01079698.1|:TSA: Salicornia europaea Unigene97627_SeCKS transcribed RNA sequence	A0A0J8BKK9|A0A0J8BKK9_BETVU: Uncharacterized protein;OS=Beta vulgaris subsp. vulgaris.;|GO:0016021,
DN126393_c2_g1_i1	DOWN	[GO:0016021:integral component of membrane]	-	-	XP_010260816.1:PREDICTED: galactan beta1,4galactosyltransferase GALS3 [Nelumbo nucifera]	gi|511316813|gb|GAKE01016064.1|:TSA: Thlaspi arvense Contig16313 transcribed RNA sequence	W9QWR2|W9QWR2_9ROSA:Uncharacterized protein;OS=Morus notabilis.;|GO:0016021,

geneid	trend	GO	Pathway	TF	NR	NT	Uniprot
DN125838_c1_g3_i9	DOWN	[GO:0048046:apoplast][GO:0005618:cell wall][GO:0016021:integral component of membrane][GO:0004553:hydrolase activity, hydrolyzing O-glycosyl compounds][GO:0016762:xyloglucan:xyloglucosyl transferase activity][GO:0042546:cell wall biogenesis][GO:0071555:cell wall organization][GO:0010411:xyloglucan metabolic process]	-	-	XP_021851909.1:probable xyloglucan endotransglucosylase/hydrolase protein 30 [Spinacia oleracea] >KNA23029.1 hypothetical protein SOVF_028460 [Spinacia oleracea]	-	A0A0K9RVS5|A0A0K9RVS5_SPIOL:Xyloglucan endotransglucosylase/hydrolase;OS=Spinacia oleracea;|GO:0048046,GO:0005618,GO:0016021,|GO:0004553,GO:0016762,|GO:0042546,GO:0071555,GO:0010411,
DN125413_c5_g2_i1	DOWN	[GO:0016021:integral component of membrane][GO:0005622:intracellular][GO:0005215:transporter activity]	-	mrna15187.1-v1.0-hybrid:Fragaria vesca|bHLH|bHLH family protein	XP_021731549.1:phosphatidylinositol/phosphatidylcholine transfer protein SFH6like [Chenopodium quinoa]	gi|521703591|gb|GAIA01016129.1|:TSA: Salicornia europaea Unigene16837_Se200S transcribed RNA sequence	A0A0K9QSX2|A0A0K9QSX2_SPIOL: Uncharacterized protein;OS=Spinacia oleracea;|GO:0016021,GO:0005622,|GO:0005215,
DN116435_c0_g2_i2	DOWN	-	-	676766192:Sisymbrium irio|BES1|BES1 family protein	XP_021842470.1:protein trichome birefringencelike 41 [Spinacia oleracea]	gi|521666877|gb|GAIA01034095.1|:TSA: Salicornia europaea Unigene37626_Se200S transcribed RNA	A0A0J8CMH9|A0A0J8CMH9_BETVU: Uncharacterized protein;OS=Beta vulgaris subsp.

geneid	trend	GO	Pathway	TF	NR	NT	Uniprot
						sequence	vulgaris.;
DN110328_c0_g1_i2	DOWN	[GO:0016021:integral component of membrane]	-	-	XP_017406701.1:PREDICTED: low temperatureinduced protein lt101.2 [Vigna angularis] >KOM26588.1 hypothetical protein LR48_Vigan303s001000 [Vigna angularis]	gi|184160092|gb|EU382073.1|:Arabidopsis halleri subsp. halleri clone BAC_7C17, genomic sequence	A0A0L9T7P0|A0A0L9T7P0_PHAAN:Uncharacterized protein;OS=Phaseolus angularis;|GO:0016021,
DN114831_c0_g1_i2	DOWN	[GO:0003824:catalytic activity][GO:0050662:coenzyme binding]	-	MDP0000265073:Malus domestica|NAC|NAC family protein	XP_021862302.1:cinnamoylCoA reductase 1like isoform X1 [Spinacia oleracea] >KNA23415.1 hypothetical protein SOVF_024990 [Spinacia oleracea]	-	A0A0K9RV58|A0A0K9RV58_SPIOL:Uncharacterized protein;OS=Spinacia oleracea;|GO:0003824,GO:0050662,

geneid	trend	GO	Pathway	TF	NR	NT	Uniprot
DN123042_c0_g1_i4	DOWN	-	-	-	XP_021861079.1:uncharacterized protein LOC110800100 [Spinacia oleracea] >XP_021861080.1 uncharacterized protein LOC110800100 [Spinacia oleracea] >XP_021861081.1 uncharacterized protein LOC110800100 [Spinacia oleracea] >XP_021861082.1 uncharacterized protein LOC110800100 [Spinacia oleracea]	gi|521747383|gb|GAIA01002171.1|:TSA: Salicornia europaea Unigene2218_Se200S transcribed RNA sequence	F6GT09|F6GT09_VITVI:Putative uncharacterized protein;OS=Vitis vinifera;GN=VIT_17s0000g00570{ECO:0000313|EMBL:CCB43354.1};

geneid	trend	GO	Pathway	TF	NR	NT	Uniprot
DN125731_c4_g16_i1	DOWN	-	-	-	XP_010670303.1:PREDICTED: jasmonateinduced protein homolog [Beta vulgaris subsp. vulgaris] >KMT17313.1 hypothetical protein BVRB_2g040070 [Beta vulgaris subsp. vulgaris]	-	A0A0J8CZH0|A0A0J8CZH0_BETVU:Uncharacterized protein;OS=Beta vulgaris subsp. vulgaris.;
DN125856_c3_g1_i3	DOWN	-	-	-	XP_010675324.1:PREDICTED: IRKinteracting protein [Beta vulgaris subsp. vulgaris] >KMT13291.1 hypothetical protein BVRB_4g085500 [Beta vulgaris subsp. vulgaris]	-	A0A0J8FBS0|A0A0J8FBS0_BETVU:Uncharacterized protein;OS=Beta vulgaris subsp. vulgaris.;

geneid	trend	GO	Pathway	TF	NR	NT	Uniprot
DN116231_c0_g1_i7	DOWN	[GO:0009881:photoreceptor activity][GO:0010224:response to UV-B]	-	-	KHG12284.1:Herc4 [Gossypium arboreum]	gi|521597449|gb|GAIA01065302.1|:TSA: Salicornia europaea Unigene78360_Se200S transcribed RNA sequence	A0A0B0NKJ2|A0A0B0NKJ2_GOSAR: Herc4;OS=Gossypium arboreum;|GO:0009881,|GO:0010224,
DN129138_c7_g26_i1	DOWN	[GO:0008061:chitin binding][GO:0004568:chitinase activity][GO:0005975:carbohydrate metabolic process][GO:0016998:cell wall macromolecule catabolic process][GO:0006032:chitin catabolic process]	[ko00520:Amino sugar and nucleotide sugar metabolism]	-	XP_021657786.1:endochitinase PR4like [Hevea brasiliensis]	-	A0A059AUC3|A0A059AUC3_EUCGR: Uncharacterized protein;OS=Eucalyptus grandis;|GO:0008061,GO:0004568,|GO:0005975,GO:0016998,GO:0006032,
DN127763_c0_g1_i1	DOWN	[GO:0016021:integral component of membrane][GO:0005524:ATP binding][GO:0004674:protein serine/threonine kinase activity][GO:0048544:recognition of pollen]	-	Bv5_098930_sojm.t1:Beta vulgaris|ERF|ERF family protein	XP_021764969.1:Gtype lectin Sreceptorlike serine/threonineprotein kinase At5g24080 [Chenopodium quinoa]	gi|224125797|ref|XM_002329684.1|:Populus trichocarpa predicted protein, mRNA	A0A0J8CQU8|A0A0J8CQU8_BETVU: Serine/threonine-protein kinase;OS=Beta vulgaris subsp. vulgaris.;|GO:0016021,|GO:0005524,GO:0004674,|GO:0048544,

geneid	trend	GO	Pathway	TF	NR	NT	Uniprot
DN128475_c2_g1_i3	DOWN	-	-	-	-	-	-
DN128542_c0_g1_i14	DOWN	-	-	-	XP_010682959.1:PREDICTED: uncharacterized protein LOC104897720 isoform X1 [Beta vulgaris subsp. vulgaris]	-	A0A0J8BZA1|A0A0J8BZA1_BETVU:Uncharacterized protein;OS=Beta vulgaris subsp. vulgaris.;
DN116561_c5_g1_i1	DOWN	[GO:0016021:integral component of membrane][GO:0004575:sucrose alpha-glucosidase activity][GO:0005975:carbohydrate metabolic process]	[ko00052:Galactose metabolism][ko00500:Starch and sucrose metabolism]	-	XP_010672026.1:PREDICTED: betafructofuranosidase, soluble isoenzyme I [Beta vulgaris subsp. vulgaris] >KMT15923.1 hypothetical protein BVRB_3g056080 [Beta vulgaris subsp. vulgaris]	-	A0A0J8CVC3|A0A0J8CVC3_BETVU:Uncharacterized protein;OS=Beta vulgaris subsp. vulgaris.;|GO:0016021,|GO:0004575,|GO:0005975,

geneid	trend	GO	Pathway	TF	NR	NT	Uniprot
DN125194_c1_g1_i8	DOWN	[GO:0005634:nucleus][GO:0009734:auxin-activated signaling pathway][GO:0006355:regulation of transcription, DNA-templated][GO:0006351:transcription, DNA-templated]	[ko04075:Plant hormone signal transduction]	-	XP_010691245.1:PREDICTED: auxinresponsive protein IAA1 [Beta vulgaris subsp. vulgaris]	-	A0A0J8ECK9|A0A0J8ECK9_BETVU:Auxin-responsive protein;OS=Beta vulgaris subsp. vulgaris.;|GO:0005634,|GO:0009734,GO:0006355,GO:0006351,
DN121706_c3_g2_i3	DOWN	[GO:0005634:nucleus][GO:0003677:DNA binding][GO:0006355:regulation of transcription, DNA-templated]	-	Bv5_100130_otho.t1:Beta vulgaris|TALE|TALE family protein	XP_018829277.1:PREDICTED: homeobox protein BEL1 homolog isoform X1 [Juglans regia] >XP_018829278.1 PREDICTED: homeobox protein BEL1 homolog isoform X1 [Juglans regia] >XP_018829279.1 PREDICTED: homeobox protein BEL1 homolog	gi|478171089|gb|GAHK01005514.1|:TSA: Jatropha curcas CL1895.Contig1_JC-CK_1A transcribed RNA sequence	A0A0J8CIZ1|A0A0J8CIZ1_BETVU:Uncharacterized protein;OS=Beta vulgaris subsp. vulgaris.;|GO:0005634,|GO:0003677,|GO:0006355,

geneid	trend	GO	Pathway	TF	NR	NT	Uniprot
					isoform X1 [Juglans regia]		
DN118697_c5_g1_i1	DOWN	[GO:0016021:integral component of membrane][GO:0033843:xyloglucan 6-xylosyltransferase activity]	-	-	GAU11210.1:hypothetical protein TSUD_342000 [Trifolium subterraneum]	gi|356529598|ref|XM_003533329.1|:PREDICTED: Glycine max putative glycosyltransferase 2-like, transcript variant 3 (LOC100813899), mRNA	A0A0B2Q3P3|A0A0B2Q3P3_GLYSO: Putative glycosyltransferase 2;OS=Glycine soja;|GO:0016021,|GO:0033843,
DN129168_c4_g8_i4	DOWN	[GO:0016491:oxidoreductase activity]	-	-	XP_018812413.1:PREDICTED: uncharacterized protein	-	A0A0J8CNU9|A0A0J8CNU9_BETVU: Uncharacterized protein;OS=Beta

geneid	trend	GO	Pathway	TF	NR	NT	Uniprot
					LOC108984806 [Juglans regia]		vulgaris subsp. vulgaris.;|GO:0016491,
DN118142_c1_g1_i3	DOWN	[GO:0016021:integral component of membrane][GO:0015031:protein transport]	-	-	XP_021740484.1:secretory carrierassociated membrane protein 1like isoform X1 [Chenopodium quinoa]	gi|521651697|gb|GAIA01041036.1|:TSA: Salicornia europaea Unigene46109_Se200S transcribed RNA sequence	A0A0J8E0Z0|A0A0J8E0Z0_BETVU:Uncharacterized protein;OS=Beta vulgaris subsp. vulgaris.;|GO:0016021,|GO:0015031,
DN124391_c2_g10_i1	DOWN	[GO:0016758:transferase activity, transferring hexosyl groups][GO:0008152:metabolic process]	[ko00908:Zeatin biosynthesis]	Tp57577_TGAC_v2_mRNA33215:Trifolium pratense|bHLH|bHLH family protein	XP_021724162.1:zeatin Oglucosyltransferaselike [Chenopodium quinoa]	gi|356537474|ref|XM_003537204.1|:PREDICTED: Glycine max zeatin O-glucosyltransferase-like (LOC100791957), mRNA	A0A0J8CSP3|A0A0J8CSP3_BETVU:Glycosyltransferase;OS=Beta vulgaris subsp. vulgaris.;|GO:0016758,|GO:0008152,
DN127007_c3_g1_i1	DOWN	-	-	-	-	-	-
DN101356_c0_g1_i1	DOWN	-	-	-	-	-	-

geneid	trend	GO	Pathway	TF	NR	NT	Uniprot
DN127142_c4_g26_i1	DOWN	-	-	-	-	-	-
DN126441_c0_g3_i3	DOWN	[GO:0005524:ATP binding][GO:0003883:CTP synthase activity][GO:0044210:'de novo' CTP biosynthetic process][GO:0006541:glutamine metabolic process]	[ko00240:Pyrimidine metabolism]	PK16897.10:Cannabis sativa|YABBY|YABBY family protein	XP_010679044.1:PREDICTED: CTP synthase isoform X1 [Beta vulgaris subsp. vulgaris] >KMT10161.1 hypothetical protein BVRB_5g119220 [Beta vulgaris subsp. vulgaris]	gi|357517334|ref|XM_003628908.1|:Medicago truncatula CTP synthase like protein (MTR_8g070540) mRNA, complete cds	A0A0J8F364|A0A0J8F364_BETVU:CTP synthase;OS=Beta vulgaris subsp. vulgaris.;|GO:0005524,GO:0003883,|GO:0044210,GO:0006541,
DN117986_c1_g2_i2	DOWN	[GO:0005524:ATP binding][GO:0005509:calcium ion binding][GO:0004672:protein kinase activity]	[ko04626:Plant-pathogen interaction][ko05145:Toxoplasmosis]	Zjn_sc00028.1.g03130.1.am.mk:Zoysia japonica|NAC|NAC family protein	XP_010678598.1:PREDICTED: calciumdependent protein kinase 10 [Beta vulgaris subsp. vulgaris] >KMT10408.1 hypothetical protein BVRB_5g115380 [Beta vulgaris subsp. vulgaris]	gi|521546535|gb|GAHZ01000377.1|:TSA: Salicornia europaea Unigene386_SeCKS transcribed RNA sequence	A0A0J8CEH5|A0A0J8CEH5_BETVU:Uncharacterized protein;OS=Beta vulgaris subsp. vulgaris.;|GO:0005524,GO:0005509,GO:0004672,

geneid	trend	GO	Pathway	TF	NR	NT	Uniprot
DN124496_c2_g1_i3	DOWN	[GO:0016758:transferase activity, transferring hexosyl groups][GO:0008152:metabolic process]	-	Tp57577_TGAC_v2_mRNA33215:Trifolium pratense|bHLH|bHLH family protein	XP_021752804.1:UDPglycosyltransferase 74E2like [Chenopodium quinoa]	gi|242199343|gb|GQ221688.1|:Citrus sinensis UDP-glucosyltransferase family 1 protein (UGT3) gene, complete cds	A0A0J8CU34|A0A0J8CU34_BETVU:Glycosyltransferase;OS=Beta vulgaris subsp. vulgaris.;|GO:0016758,|GO:0008152,
DN126635_c3_g1_i6	DOWN	[GO:0003993:acid phosphatase activity][GO:0046872:metal ion binding]	-	Achn233011:Actinidia chinensis|SRS|SRS family protein	XP_010672424.1:PREDICTED: purple acid phosphatase [Beta vulgaris subsp. vulgaris] >KMT20665.1 hypothetical protein BVRB_1g006410 [Beta vulgaris subsp. vulgaris]	gi|224085757|ref|XM_002307654.1|:Populus trichocarpa predicted protein, mRNA	A0A0J8D3C8|A0A0J8D3C8_BETVU:Purple acid phosphatase;OS=Beta vulgaris subsp. vulgaris.;|GO:0003993,GO:0046872,
DN124729_c4_g1_i5	DOWN	[GO:0016021:integral component of membrane]	-	-	XP_021862970.1:uncharacterized protein At4g15970like [Spinacia oleracea]	-	A0A0J8BKK9|A0A0J8BKK9_BETVU:Uncharacterized protein;OS=Beta vulgaris subsp. vulgaris.;|GO:0016021,

geneid	trend	GO	Pathway	TF	NR	NT	Uniprot
DN112282_c0_g1_i1	DOWN	-	-	Csa09g092790.1:Camelina sativa|BES1|BES1 family protein	XP_021842604.1:protein trichome birefringencelike 42 isoform X1 [Spinacia oleracea] >KNA16000.1 hypothetical protein SOVF_093180 isoform A [Spinacia oleracea]	gi|521480175|gb|GAHZ01050711.1|:TSA: Salicornia europaea Unigene59879_SeCKS transcribed RNA sequence	A0A0K9R933|A0A0K9R933_SPIOL:Uncharacterized protein;OS=Spinacia oleracea;
DN125125_c1_g9_i1	DOWN	-	-	-	-	-	-
DN126441_c0_g3_i7	DOWN	[GO:0005524:ATP binding][GO:0003883:CTP synthase activity][GO:0044210:'de novo' CTP biosynthetic process][GO:0006541:glutamine metabolic process]	[ko00240:Pyrimidine metabolism]	PK16897.10:Cannabis sativa|YABBY|YABBY family protein	XP_010679044.1:PREDICTED: CTP synthase isoform X1 [Beta vulgaris subsp. vulgaris] >KMT10161.1 hypothetical protein BVRB_5g119220 [Beta vulgaris subsp. vulgaris]	gi|357517334|ref|XM_003628908.1|:Medicago truncatula CTP synthase like protein (MTR_8g070540) mRNA, complete cds	A0A0J8F364|A0A0J8F364_BETVU:CTP synthase;OS=Beta vulgaris subsp. vulgaris.;|GO:0005524,GO:0003883,|GO:0044210,GO:0006541,

geneid	trend	GO	Pathway	TF	NR	NT	Uniprot
DN125546_c3_g2_i12	DOWN	-	-	GSMUA_Achr4P28960_001:Musa acuminata|SBP|SBP family protein	XP_010680424.1:PREDICTED: uncharacterized protein LOC104895573 isoform X1 [Beta vulgaris subsp. vulgaris] >XP_010680425.1 PREDICTED: uncharacterized protein LOC104895573 isoform X1 [Beta vulgaris subsp. vulgaris] >KMT09346.1 hypothetical protein BVRB_6g134400 [Beta vulgaris subsp. vulgaris]	gi|349717811|emb|FQ381014.1|:Vitis vinifera clone SS0ACG1YK18	A0A0J8F1A1|A0A0J8F1A1_BETVU:Uncharacterized protein;OS=Beta vulgaris subsp. vulgaris.;

geneid	trend	GO	Pathway	TF	NR	NT	Uniprot
DN111165_c0_g1_i1	DOWN	[GO:0043565:sequence-specific DNA binding][GO:0003700:DNA-binding transcription factor activity]	-	Ciclev10027584m:Citrus clementina|bZIP|bZIP family protein	XP_006426133.1:hypothetical protein CICLE_v10027584mg [Citrus clementina] >ESR39373.1 hypothetical protein CICLE_v10027584mg [Citrus clementina]	-	V4RWX2|V4RWX2_9ROSI:Uncharacterized protein;OS=Citrus clementina.;|GO:0043565,GO:0003700,
DN128756_c3_g1_i2	DOWN	-	-	mrna13277.1-v1.0-hybrid:Fragaria vesca|MIKC_MADS|MIKC_MADS family protein	XP_021754111.1:paramyosinlike isoform X2 [Chenopodium quinoa]	-	A0A0K9RM17|A0A0K9RM17_SPIOL:Uncharacterized protein;OS=Spinacia oleracea;
DN120524_c0_g8_i2	DOWN	[GO:0009269:response to desiccation]	-	-	ACJ76842.1:salt tolerance protein I [Sesuvium portulacastrum]	gi|478162615|gb|GAHK01013988.1|:TSA: Jatropha curcas Unigene1441_JC-CK_1A transcribed RNA sequence	B8XXQ9|B8XXQ9_SESPO:Salt tolerance protein I;OS=Sesuvium portulacastrum;|GO:0009269,

geneid	trend	GO	Pathway	TF	NR	NT	Uniprot
DN127445_c4_g2_i4	DOWN	[GO:0005634:nucleus][GO:0003677:DNA binding][GO:0003700:DNA-binding transcription factor activity][GO:0006351:transcription, DNA-templated]	-	Bv6_133340_zkhw.t1:Beta vulgaris|ERF|ERF family protein	XP_021713958.1:ethyleneresponsive transcription factor ERF113like [Chenopodium quinoa]	gi|332002898|gb|CP002688.1|:Arabidopsis thaliana chromosome 5, complete sequence	A0A0J8CB32|A0A0J8CB32_BETVU:Uncharacterized protein;OS=Beta vulgaris subsp. vulgaris.;|GO:0005634,|GO:0003677,GO:0003700,|GO:0006351,
DN127467_c3_g2_i2	DOWN	[GO:0005789:endoplasmic reticulum membrane]	-	-	XP_010686270.1:PREDICTED: vesicleassociated protein 42 [Beta vulgaris subsp. vulgaris] >KMT04478.1 hypothetical protein BVRB_8g181470 [Beta vulgaris subsp. vulgaris]	-	A0A0J8BWY1|A0A0J8BWY1_BETVU:Uncharacterized protein;OS=Beta vulgaris subsp. vulgaris.;|GO:0005789,

geneid	trend	GO	Pathway	TF	NR	NT	Uniprot
DN114244_c0_g1_i2	DOWN	[GO:0016021:integral component of membrane][GO:0005338:nucleotide-sugar transmembrane transporter activity][GO:0015780:nucleotide-sugar transmembrane transport]	-	Do008886.1:Dichanthelium oligosanthes|NAC|NAC family protein	XP_021728151.1:UDPrhamnose/UDPgalactose transporter 4like [Chenopodium quinoa] >XP_021728152.1 UDPrhamnose/UDP galactose transporter 4like [Chenopodium quinoa] >XP_021728153.1 UDPrhamnose/UDP galactose transporter 4like [Chenopodium quinoa]	-	A0A0K9RVC5|A0A0K9RVC5_SPIOL: Uncharacterized protein;OS=Spinacia oleracea;|GO:0016021,|GO:0005338,|GO:0015780,
DN121694_c2_g2_i3	DOWN	-	-	-	XP_018860004.1:PREDICTED: Ubox domaincontaining protein 4like [Juglans regia]	gi|356569538|ref|XM_003552909.1|:PREDICTED: Glycine max U-box domain-containing protein 4-like (LOC100780954), mRNA	A0A161ZW28|A0A161ZW28_DAUCA: Uncharacterized protein;OS=Daucus carota subsp. sativus.;

geneid	trend	GO	Pathway	TF	NR	NT	Uniprot
DN125874_c 8_g1_i1	DOWN	[GO:0016021:integral component of membrane][GO:0055114:oxidation-re duction process]	-	-	XP_021735209.1:cy tochrome b561 and DOMON domaincontaining protein At5g35735like [Chenopodium quinoa]	gi|116012816|dbj|A K243451.1|:Oryza sativa Japonica Group cDNA, clone: J100070C01, full insert sequence	A0A0K9RL58|A0A 0K9RL58_SPIOL:U ncharacterized protein;OS=Spinacia oleracea;|GO:00160 21,|GO:0055114,
DN128780_c 2_g8_i2	DOWN	[GO:0004568:chitinase activity][GO:0005975:carbohydrate metabolic process][GO:0016998:cell wall macromolecule catabolic process][GO:0006032:chitin catabolic process]	[ko00520:Amino sugar and nucleotide sugar metabolism]	-	XP_021989730.1:en dochitinase EP3like [Helianthus annuus] >OTG12466.1 putative glycoside hydrolase, family 19, Lysozymelike domain protein [Helianthus annuus]	-	Q7M1Q9|Q7M1Q9_ PHYAM:Chitinase A;OS=Phytolacca americana;|GO:0004 568,|GO:0005975,G O:0016998,GO:0006 032,
DN117064_c 3_g1_i3	DOWN	[GO:0016021:integral component of membrane][GO:0005215:transporter activity][GO:0006857:oligopeptide transport]	-	Oropetium_2015010 5_01570A:Oropetiu m thomaeum|WRKY| WRKY family protein	XP_021685972.1:pr otein NRT1/ PTR FAMILY 8.3like isoform X1 [Hevea brasiliensis]	gi|521452864|gb|GA HZ01067620.1|:TSA : Salicornia europaea Unigene81668_SeC KS transcribed RNA sequence	F6HEU1|F6HEU1_ VITVI:Putative uncharacterized protein;OS=Vitis vinifera;GN=VIT_0 1s0011g06620 {ECO:0000313|EM BL:CCB50737.1};|G

geneid	trend	GO	Pathway	TF	NR	NT	Uniprot
							O:0016021,|GO:0005215,|GO:0006857,
DN119487_c1_g2_i8	DOWN	[GO:0016491:oxidoreductase activity]	[ko00061:Fatty acid biosynthesis][ko00780:Biotin metabolism][ko01040:Biosynthesis of unsaturated fatty acids]	-	XP_021741048.1:NADPHdependent aldehyde reductaselike protein, chloroplastic, partial [Chenopodium quinoa]	-	A0A0J8CUB0|A0A0J8CUB0_BETVU: Uncharacterized protein;OS=Beta vulgaris subsp. vulgaris.;|GO:0016491,
DN120445_c4_g1_i2	DOWN	-	-	-	-	-	-
DN113624_c0_g1_i2	DOWN	[GO:0016021:integral component of membrane]	[ko00520:Amino sugar and nucleotide sugar metabolism]	MDP0000247737:Malus domestica|FAR1|FAR1 family protein	XP_021856720.1:UDPglucuronic acid decarboxylase 2 [Spinacia oleracea] >KNA16834.1 hypothetical protein SOVF_084960	gi|521501538|gb|GAHZ01029382.1|:TSA: Salicornia europaea Unigene32343_SeCKS transcribed RNA sequence	A0A0K9RCV8|A0A0K9RCV8_SPIOL: Uncharacterized protein;OS=Spinacia oleracea;|GO:0016021,

geneid	trend	GO	Pathway	TF	NR	NT	Uniprot
					[Spinacia oleracea]		
DN122816_c0_g1_i3	DOWN	-	-	-	-	-	-
DN126395_c1_g1_i3	DOWN	[GO:0004252:serine-type endopeptidase activity]	-	Aco013131.1:Ananas comosus|DBB|DBB family protein	XP_010692331.1:PREDICTED: subtilisinlike protease SBT4.14 [Beta vulgaris subsp. vulgaris] >KMT00167.1 hypothetical protein BVRB_1g019850 [Beta vulgaris subsp. vulgaris]	-	A0A0J8BGQ9|A0A0J8BGQ9_BETVU: Uncharacterized protein;OS=Beta vulgaris subsp. vulgaris.;|GO:0004252,
DN124100_c6_g12_i1	DOWN	-	-	-	-	gi|160957909|emb|CU231924.1|:Populus EST from mild drought-stressed leaves	-

geneid	trend	GO	Pathway	TF	NR	NT	Uniprot
DN128914_c1_g1_i10	DOWN	[GO:0005509:calcium ion binding]	-	-	XP_016733381.1:PREDICTED: LRR receptorlike serine/threonineprotein kinase FLS2 [Gossypium hirsutum]	-	F6H3W3|F6H3W3_VITVI:Putative uncharacterized protein;OS=Vitis vinifera;GN=VIT_04s0008g06290 {ECO:0000313|EMBL:CCB46852.1};|GO:0005509,
DN125900_c0_g4_i1	DOWN	[GO:0016021:integral component of membrane][GO:0005524:ATP binding][GO:0004672:protein kinase activity]	[ko04075:Plant hormone signal transduction]	-	XP_010680326.1:PREDICTED: systemin receptor SR160 [Beta vulgaris subsp. vulgaris]	-	A0A0K9RSY6|A0A0K9RSY6_SPIOL:Uncharacterized protein;OS=Spinacia oleracea;|GO:0016021,|GO:0005524,GO:0004672,
DN110485_c0_g1_i2	DOWN	-	-	Rsa1.0_00048.1_g00027.1:Raphanus sativus|bHLH|bHLH family protein	KNA22290.1:hypothetical protein SOVF_035310 isoform A [Spinacia oleracea]	gi|521745514|gb|GAIA01003243.1|:TSA: Salicornia europaea Unigene3413_Se200S transcribed RNA sequence	A0A0K9RRW7|A0A0K9RRW7_SPIOL:Uncharacterized protein;OS=Spinacia oleracea;
DN119868_c2_g1_i2	DOWN	-	-	-	-	-	-

geneid	trend	GO	Pathway	TF	NR	NT	Uniprot
DN124479_c1_g2_i2	DOWN	[GO:0016021:integral component of membrane][GO:0005739:mitochondrion][GO:0005524:ATP binding][GO:0004674:protein serine/threonine kinase activity][GO:0042742:defense response to bacterium][GO:0050832:defense response to fungus][GO:0048281:inflorescence morphogenesis][GO:0009965:leaf morphogenesis][GO:0009664:plant-type cell wall organization][GO:0009944:polarity specification of adaxial/abaxial axis][GO:0030155:regulation of cell adhesion][GO:0051302:regulation of cell division][GO:0001558:regulation of cell growth][GO:2000027:regulation of animal organ morphogenesis][GO:0010103:stomatal complex morphogenesis][GO:0010148:transpiration][GO:0001944:vasculature development]	[ko04016:MAPK signaling pathway - plant]	XP_015897327.1:Ziziphus jujuba|Trihelix|Trihelix family protein	XP_021738909.1:LRR receptorlike serine/threonineprotein kinase ERECTA [Chenopodium quinoa]	gi|460399019|ref|XM_004244995.1|:PREDICTED: Solanum lycopersicum LRR receptor-like serine/threonine-protein kinase ERECTA-like (LOC101266437), mRNA	A0A0J8D110|A0A0J8D110_BETVU:Uncharacterized protein;OS=Beta vulgaris subsp. vulgaris.;|GO:0016021,GO:0005739,|GO:0005524,GO:0004674,|GO:0042742,GO:0050832,GO:0048281,GO:0009965,GO:0009664,GO:0009944,GO:0030155,GO:0051302,GO:0001558,GO:2000027,GO:0010103,GO:0010148,GO:0001944,

geneid	trend	GO	Pathway	TF	NR	NT	Uniprot
DN125706_c3_g2_i3	DOWN	[GO:0004722:protein serine/threonine phosphatase activity]	-	KZV22762.1:Dorcoceras hygrometricum|CAMTA|CAMTA family protein	XP_009348094.1:PREDICTED: probable protein phosphatase 2C 10 [Pyrus x bretschneideri] >XP_009348095.1 PREDICTED: probable protein phosphatase 2C 10 [Pyrus x bretschneideri]	gi|224084661|ref|XM_002307342.1|:Populus trichocarpa predicted protein, mRNA	B9RJK5|B9RJK5_RICCO:Protein phosphatase 2c, putative;OS=Ricinus communis;|GO:0004722,
DN123180_c4_g1_i2	DOWN	[GO:0005618:cell wall][GO:0005576:extracellular region][GO:0016020:membrane][GO:0009664:plant-type cell wall organization]	-	676732396:Sisymbrium irio|bHLH|bHLH family protein	XP_021761608.1:expansinA10like [Chenopodium quinoa]	gi|521739695|gb|GAIA01006855.1|:TSA: Salicornia europaea Unigene7166_Se200S transcribed RNA sequence	A0A0J8CB05|A0A0J8CB05_BETVU:Uncharacterized protein;OS=Beta vulgaris subsp. vulgaris.;|GO:0005618,GO:0005576,GO:0016020,|GO:0009664,

geneid	trend	GO	Pathway	TF	NR	NT	Uniprot
DN118063_c3_g5_i1	DOWN	[GO:0030598:rRNA N-glycosylase activity][GO:0006952:defense response][GO:0017148:negative regulation of translation]	-	-	AAS77872.1:antiviral protein [Phytolacca americana]	-	Q6PWU4|Q6PWU4_PHYAM:rRNA N-glycosidase;OS=Phytolacca americana;|GO:0030598,|GO:0006952,GO:0017148,

geneid	trend	GO	Pathway	TF	NR	NT	Uniprot
DN124052_c1_g4_i6	DOWN	[GO:0009941:chloroplast envelope][GO:0005789:endoplasmic reticulum membrane][GO:0016021:integral component of membrane][GO:0005811:lipid droplet][GO:0004144:diacylglycerol O-acyltransferase activity][GO:0005975:carbohydrate metabolic process][GO:0010030:positive regulation of seed germination][GO:0045995:regulation of embryonic development][GO:0009737:response to abscisic acid][GO:0009409:response to cold][GO:0009749:response to glucose][GO:0009651:response to salt stress][GO:0019432:triglyceride biosynthetic process]	[ko00561:Glycerolipid metabolism][ko00830:Retinol metabolism][ko04975:Fat digestion and absorption]	-	XP_010696537.1:PREDICTED: diacylglycerol Oacyltransferase 1 [Beta vulgaris subsp. vulgaris] >KMS96848.1 hypothetical protein BVRB_8g199240 [Beta vulgaris subsp. vulgaris]	gi|521499110|gb|GAHZ01031810.1|:TSA: Salicornia europaea Unigene35062_SeCKS transcribed RNA sequence	A0A0J8BA41|A0A0J8BA41_BETVU:O-acyltransferase;OS=Beta vulgaris subsp. vulgaris.;|GO:0009941,GO:0005789,GO:0016021,GO:0005811,|GO:0004144,|GO:0005975,GO:0010030,GO:0045995,GO:0009737,GO:0009409,GO:0009749,GO:0009651,GO:0019432,

geneid	trend	GO	Pathway	TF	NR	NT	Uniprot
DN126550_c2_g1_i1	DOWN	[GO:0016021:integral component of membrane][GO:0055085:transmembrane transport]	-	MDP0000295305:Malus domestica|NF-YC|NF-YC family protein	XP_010666854.1:PREDICTED: cation/calcium exchanger 4like [Beta vulgaris subsp. vulgaris]	-	A0A0J8B3R3|A0A0J8B3R3_BETVU:Uncharacterized protein;OS=Beta vulgaris subsp. vulgaris.;|GO:0016021,|GO:0055085,
DN125521_c1_g4_i3	DOWN	-	-	-	KNA21659.1:hypothetical protein SOVF_041230 [Spinacia oleracea]	-	A0A0K9RRY8|A0A0K9RRY8_SPIOL: Uncharacterized protein;OS=Spinacia oleracea;
DN116303_c4_g1_i3	DOWN	-	-	Achn132541:Actinidia chinensis|ERF|ERF family protein	XP_021765176.1:BAG family molecular chaperone regulator 3like [Chenopodium quinoa]	gi|356559872|ref|XR_137352.1|:PREDICTED: Glycine max uncharacterized LOC100818492 (LOC100818492), miscRNA	A0A0K9QQB7|A0A0K9QQB7_SPIOL: Uncharacterized protein;OS=Spinacia oleracea;
DN114858_c0_g3_i2	DOWN	-	-	-	XP_021741378.1:uncharacterized protein LOC110707665 [Chenopodium quinoa]	-	A0A0J8EKW4|A0A0J8EKW4_BETVU: Uncharacterized protein;OS=Beta vulgaris subsp. vulgaris.;

geneid	trend	GO	Pathway	TF	NR	NT	Uniprot
DN120567_c0_g1_i4	DOWN	[GO:0005829:cytosol][GO:0009409:response to cold][GO:0006970:response to osmotic stress][GO:0009414:response to water deprivation][GO:0048316:seed development]	-	-	XP_011078826.1:late embryogenesis abundant protein 46like [Sesamum indicum]	-	K4BGN5|K4BGN5_SOLLC:Uncharacterized protein;OS=Solanum lycopersicum;|GO:0005829,|GO:0009409,GO:0006970,GO:0009414,GO:0048316,
DN110571_c0_g3_i1	DOWN	[GO:0008270:zinc ion binding]	-	-	XP_010682265.1:PREDICTED: probable E3 ubiquitinprotein ligase XERICO [Beta vulgaris subsp. vulgaris] >KMT07753.1 hypothetical protein BVRB_6g146750 [Beta vulgaris subsp. vulgaris]	-	A0A0J8C6U0|A0A0J8C6U0_BETVU:Uncharacterized protein;OS=Beta vulgaris subsp. vulgaris.;|GO:0008270,

geneid	trend	GO	Pathway	TF	NR	NT	Uniprot
DN126653_c0_g1_i4	DOWN	[GO:0016021:integral component of membrane]	-	Bv5_101120_supk.t1:Beta vulgaris|ERF|ERF family protein	AAF91431.1:putative Na+/myoinositol symporter [Mesembryanthemum crystallinum]	gi|521510402|gb|GAHZ01020518.1|:TSA: Salicornia europaea Unigene21618_SeCKS transcribed RNA sequence	Q9LKH2|Q9LKH2_MESCR:Putative Na+/myo-inositol symporter;OS=Mesembryanthemum crystallinum;|GO:0016021,|GO:0022891,
DN114629_c0_g1_i1	DOWN	[GO:0016021:integral component of membrane]	-	-	XP_010691334.1:PREDICTED: uncharacterized protein LOC104904684 [Beta vulgaris subsp. vulgaris] >KMT00818.1 hypothetical protein BVRB_9g220940 [Beta vulgaris subsp. vulgaris]	gi|403043539|ref|NM_001254412.2|:Glycine max uncharacterized LOC100818121 (LOC100818121), mRNA	A0A0J8BL17|A0A0J8BL17_BETVU:Uncharacterized protein;OS=Beta vulgaris subsp. vulgaris.;|GO:0016021,
DN127528_c4_g7_i1	DOWN	-	-	-	-	-	-

geneid	trend	GO	Pathway	TF	NR	NT	Uniprot
DN128780_c2_g5_i1	DOWN	[GO:0004568:chitinase activity][GO:0005975:carbohydrate metabolic process][GO:0016998:cell wall macromolecule catabolic process][GO:0006032:chitin catabolic process]	[ko00520:Amino sugar and nucleotide sugar metabolism]	-	XP_015868633.1:PREDICTED: endochitinase EP3like [Ziziphus jujuba]	-	Q7M1Q9|Q7M1Q9_PHYAM:Chitinase A;OS=Phytolacca americana;|GO:0004568,|GO:0005975,GO:0016998,GO:0006032,
DN119137_c0_g1_i2	DOWN	-	-	-	XP_021738441.1:putative protein TPRXL [Chenopodium quinoa]	-	A0A0K9RB35|A0A0K9RB35_SPIOL:Uncharacterized protein;OS=Spinacia oleracea;
DN122116_c4_g1_i5	DOWN	[GO:0004190:aspartic-type endopeptidase activity]	-	-	XP_010680395.1:PREDICTED: aspartyl protease family protein 1 [Beta vulgaris subsp. vulgaris] >KMT09315.1 hypothetical protein BVRB_6g134130 [Beta vulgaris subsp. vulgaris]	gi|261264968|gb|GU048885.1|:Capsicum annuum clone BAC CaCM414G15, complete sequence	A0A0J8C6D8|A0A0J8C6D8_BETVU:Uncharacterized protein;OS=Beta vulgaris subsp. vulgaris.;|GO:0004190,
DN119725_c	DOWN	-	-	-	-	-	-

geneid	trend	GO	Pathway	TF	NR	NT	Uniprot
0_g3_i1							
DN121068_c2_g3_i2	DOWN	[GO:0016021:integral component of membrane][GO:0015369:calcium:proton antiporter activity]	-	DCAR_013815:Daucus carota|ERF|ERF family protein	XP_021753905.1:vacuolar cation/proton exchanger 3like [Chenopodium quinoa]	gi|356551862|ref|XM_003544244.1|:PREDICTED: Glycine max vacuolar cation/proton exchanger 1-like (LOC100816844), mRNA	Q6R2R4|Q6R2R4_SUASA:CAX;OS=Suaeda salsa;|GO:0016021,|GO:0015369,
DN117241_c1_g1_i1	DOWN	-	-	-	-	-	-
DN117235_c2_g1_i2	DOWN	-	-	-	XP_021740487.1:putative glycinerich cell wall structural protein 1 [Chenopodium quinoa]	-	M0STZ5|M0STZ5_MUSAM:Uncharacterized protein;OS=Musa acuminata subsp. malaccensis;
DN121560_c3_g3_i6	DOWN	[GO:0005634:nucleus][GO:0009734:auxin-activated signaling pathway][GO:0006355:regulation of transcription, DNA-templated][GO:0006351:transcription, DNA-templated]	[ko04075:Plant hormone signal transduction]	-	XP_021770258.1:auxininduced protein 22Dlike [Chenopodium quinoa]	gi|521656915|gb|GAIA01039007.1|:TSA: Salicornia europaea Unigene43651_Se200S transcribed RNA sequence	A0A0K9QV49|A0A0K9QV49_SPIOL:Auxin-responsive protein;OS=Spinacia oleracea;|GO:0005634,|GO:0009734,GO:0006355,GO:00063

geneid	trend	GO	Pathway	TF	NR	NT	Uniprot
							51,
DN117349_c0_g7_i8	DOWN	[GO:0005524:ATP binding][GO:0004356:glutamate-ammonia ligase activity][GO:0006542:glutamine biosynthetic process]	[ko00220:Arginine biosynthesis][ko00250:Alanine, aspartate and glutamate metabolism][ko00630:Glyoxylate and dicarboxylate metabolism][ko00910:Nitrogen metabolism][ko02020:Two-component system][ko04217:Necroptosis][ko04724:Glutamatergic synapse][ko04727:GABAergic synapse]	-	AAD31899.1:cytosolic glutamine synthetase, partial [Mesembryanthemum crystallinum]	gi|4884971|gb|AF145481.1|AF145481:Mesembryanthemum crystallinum cytosolic glutamine synthetase mRNA, partial cds	Q9XFJ2|Q9XFJ2_MESCR:Glutamine synthetase;OS=Mesembryanthemum crystallinum;|GO:0005524,GO:0004356,|GO:0006542,

geneid	trend	GO	Pathway	TF	NR	NT	Uniprot
DN128780_c2_g9_i1	DOWN	[GO:0004568:chitinase activity][GO:0005975:carbohydrate metabolic process][GO:0016998:cell wall macromolecule catabolic process][GO:0006032:chitin catabolic process]	[ko00520:Amino sugar and nucleotide sugar metabolism]	-	XP_020692273.1:chitinase 4like [Dendrobium catenatum]	-	Q7M1Q9|Q7M1Q9_PHYAM:Chitinase A;OS=Phytolacca americana;|GO:0004568,|GO:0005975,GO:0016998,GO:0006032,
DN127793_c1_g4_i2	DOWN	[GO:0003824:catalytic activity]	[ko00052:Galactose metabolism]	-	XP_021768710.1:stachyose synthaselike [Chenopodium quinoa]	gi|521558221|gb|GAIA01078344.1|:TSA: Salicornia europaea Unigene95273_Se200S transcribed RNA sequence	A0A0J8C8R8|A0A0J8C8R8_BETVU:Uncharacterized protein;OS=Beta vulgaris subsp. vulgaris.;|GO:0003824,
DN118441_c4_g4_i1	DOWN	[GO:0046872:metal ion binding][GO:0004722:protein serine/threonine phosphatase activity]	[ko04016:MAPK signaling pathway - plant][ko04075:Plant hormone signal transduction][ko04931:Insulin resistance]	XP_010536267.1:Tarenaya hassleriana|NAC|NAC family protein	XP_010685683.1:PREDICTED: probable protein phosphatase 2C 24 [Beta vulgaris subsp. vulgaris] >KMT05175.1 hypothetical protein BVRB_7g173230 [Beta vulgaris subsp.	-	A0A0J8BUE2|A0A0J8BUE2_BETVU:Uncharacterized protein;OS=Beta vulgaris subsp. vulgaris.;|GO:0046872,GO:0004722,

geneid	trend	GO	Pathway	TF	NR	NT	Uniprot
					vulgaris]		
DN123155_c2_g2_i11	DOWN	[GO:0046872:metal ion binding][GO:0004722:protein serine/threonine phosphatase activity]	-	KZV22762.1:Dorcoceras hygrometricum|CAMTA|CAMTA family protein	XP_010685523.1:PREDICTED: probable protein phosphatase 2C 25 [Beta vulgaris subsp. vulgaris] >KMT05026.1 hypothetical protein BVRB_7g171860 [Beta vulgaris subsp. vulgaris]	gi|521738200|gb|GAIA01007700.1|:TSA: Salicornia europaea Unigene8019_Se200S transcribed RNA sequence	A0A0J8EPX9|A0A0J8EPX9_BETVU:Uncharacterized protein;OS=Beta vulgaris subsp. vulgaris.;|GO:0046872,GO:0004722,
DN124473_c1_g2_i1	DOWN	[GO:0008171:O-methyltransferase activity]	-	-	XP_021770486.1:(R,S)reticuline 7Omethyltransferase like [Chenopodium quinoa]	-	A0A0J8BLP8|A0A0J8BLP8_BETVU:Uncharacterized protein;OS=Beta vulgaris subsp. vulgaris.;|GO:00081

geneid	trend	GO	Pathway	TF	NR	NT	Uniprot
							71,
DN110786_c0_g1_i1	DOWN	[GO:0033897:ribonuclease T2 activity][GO:0003723:RNA binding]	-	-	XP_010683182.1:PREDICTED: ribonuclease 1 [Beta vulgaris subsp. vulgaris] >KMT07058.1 hypothetical protein BVRB_6g153870 [Beta vulgaris subsp. vulgaris]	gi|521480454|gb|GAHZ01050432.1|:TSA: Salicornia europaea Unigene59568_SeCKS transcribed RNA sequence	A0A0J8C4W3|A0A0J8C4W3_BETVU: Uncharacterized protein;OS=Beta vulgaris subsp. vulgaris.;|GO:0033897,GO:0003723,

geneid	trend	GO	Pathway	TF	NR	NT	Uniprot
DN127604_c2_g3_i2	DOWN	[GO:0005886:plasma membrane]	-	-	XP_010681137.1:PREDICTED: uncharacterized protein LOC104896134 [Beta vulgaris subsp. vulgaris] >XP_010681138.1 PREDICTED: uncharacterized protein LOC104896134 [Beta vulgaris subsp. vulgaris] >XP_010681140.1 PREDICTED: uncharacterized protein LOC104896134 [Beta vulgaris subsp. vulgaris] >KMT08720.1 hypothetical protein BVRB_6g139930 [Beta vulgaris subsp.	gi|521669176|gb|GAIA01033042.1|:TSA: Salicornia europaea Unigene36266_Se200S transcribed RNA sequence	A0A0J8C4J7|A0A0J8C4J7_BETVU:Uncharacterized protein;OS=Beta vulgaris subsp. vulgaris.;|GO:0005886,

geneid	trend	GO	Pathway	TF	NR	NT	Uniprot
					vulgaris]		
DN126406_c 4_g3_i8	DOWN	[GO:0016758:transferase activity, transferring hexosyl groups][GO:0008152:metabolic process]	-	TRIUR3_17264-P1: Triticum urartu|GRAS|GRAS family protein	XP_010680524.1:P REDICTED: 7deoxyloganetin glucosyltransferaseli ke [Beta vulgaris subsp. vulgaris]	gi|225465723|ref|X M_002263241.1|:PR EDICTED: Vitis vinifera UDP-glycosyltransfe rase 85A2-like, transcript variant 2	A0A0J8EZU7|A0A0 J8EZU7_BETVU:Gl ycosyltransferase;O S=Beta vulgaris subsp. vulgaris.;|GO:00167 58,|GO:0008152,

geneid	trend	GO	Pathway	TF	NR	NT	Uniprot
						(LOC100243488), mRNA	
DN120133_c0_g2_i3	DOWN	[GO:0016020:membrane][GO:0016798:hydrolase activity, acting on glycosyl bonds][GO:0005975:carbohydrate metabolic process]	[ko00531:Glycosaminoglycan degradation][ko05205:Proteoglycans in cancer]	-	KNA04466.1:hypothetical protein SOVF_199420, partial [Spinacia oleracea]	gi|255586002|ref|XM_002533625.1|:Ricinus communis heparanase, putative, mRNA	A0A0K9QD39|A0A0K9QD39_SPIOL:Uncharacterized protein;OS=Spinacia oleracea;|GO:0016020,|GO:0016798,|GO:0005975,
DN111048_c0_g1_i4	DOWN	-	-	-	-	-	-
DN107007_c0_g1_i2	DOWN	[GO:0016021:integral component of membrane][GO:0005524:ATP binding][GO:0016887:ATPase activity][GO:0006810:transport]	-	Rsa1.0_03996.1_g00001.1:Raphanus sativus|MYB_related|MYB_related family protein	XP_010665917.1:PREDICTED: ABC transporter G family member 36 [Beta vulgaris subsp. vulgaris] >KMS96494.1 hypothetical protein BVRB_9g224870	gi|521637749|gb|GAIA01047798.1|:TSA: Salicornia europaea Unigene56014_Se200S transcribed RNA sequence	A0A0J8B5I1|A0A0J8B5I1_BETVU:Uncharacterized protein;OS=Beta vulgaris subsp. vulgaris.;|GO:0016021,|GO:0005524,GO:0016887,|GO:0006810,

geneid	trend	GO	Pathway	TF	NR	NT	Uniprot
					[Beta vulgaris subsp. vulgaris]		
DN122740_c0_g1_i6	DOWN	-	-	-	XP_021741568.1:probable strigolactone esterase DAD2 [Chenopodium quinoa]	-	A0A0K9RD14|A0A0K9RD14_SPIOL:Uncharacterized protein;OS=Spinacia oleracea;
DN115632_c0_g1_i2	DOWN	[GO:0016021:integral component of membrane][GO:0055114:oxidation-reduction process]	-	-	XP_021749592.1:cytochrome b561 and DOMON domaincontaining protein At4g12980like [Chenopodium quinoa]	-	A0A0K9RB36|A0A0K9RB36_SPIOL:Uncharacterized protein;OS=Spinacia oleracea;|GO:0016021,|GO:0055114,
DN128951_c12_g9_i1	DOWN	-	-	-	-	-	-

geneid	trend	GO	Pathway	TF	NR	NT	Uniprot
DN126882_c0_g2_i17	DOWN	[GO:0016021:integral component of membrane][GO:0005524:ATP binding][GO:0004672:protein kinase activity]	[ko04064:NF-kappa B signaling pathway][ko04620:Toll-like receptor signaling pathway][ko04621:NOD-like receptor signaling pathway][ko04624:Toll and Imd signaling pathway][ko04722:Neurotrophin signaling pathway][ko05133:Pertussis][ko05140:Leishmaniasis][ko05142:Chagas disease (American trypanosomiasis)][ko05145:Toxoplasmosis][ko05152:Tuberculosis][ko05162:Measles][ko05164:Influenza A]	312791:Arabidopsis lyrata|bHLH|bHLH family protein	XP_010681636.1:PREDICTED: LEAF RUST 10 DISEASERESISTANCE LOCUS RECEPTORLIKE PROTEIN KINASElike 1.1 [Beta vulgaris subsp. vulgaris] >KMT08229.1 hypothetical protein BVRB_6g142460 [Beta vulgaris subsp. vulgaris]	-	A0A0J8C7J6|A0A0J8C7J6_BETVU:Uncharacterized protein;OS=Beta vulgaris subsp. vulgaris.;|GO:0016021,|GO:0005524,GO:0004672,

geneid	trend	GO	Pathway	TF	NR	NT	Uniprot
DN118303_c4_g1_i4	DOWN	[GO:0016021:integral component of membrane]	-	-	XP_010695641.1:PREDICTED: uncharacterized protein LOC104908245 [Beta vulgaris subsp. vulgaris] >KMS97529.1 hypothetical protein BVRB_5g126170 [Beta vulgaris subsp. vulgaris]	-	A0A0J8B8F1|A0A0J8B8F1_BETVU:Uncharacterized protein;OS=Beta vulgaris subsp. vulgaris.;|GO:0016021,

geneid	trend	GO	Pathway	TF	NR	NT	Uniprot
DN124061_c 1_g2_i5	DOWN	[GO:0005634:nucleus][GO:0003677: DNA binding]	-	Bv6_150690_wmrr.t 1:Beta vulgaris|SBP|SBP family protein	XP_010682751.1:P REDICTED: squamosa promoterbindinglike protein 2 [Beta vulgaris subsp. vulgaris] >XP_010682752.1 PREDICTED: squamosa promoterbindinglike protein 2 [Beta vulgaris subsp. vulgaris] >XP_010682753.1 PREDICTED: squamosa promoterbindinglike protein 2 [Beta vulgaris subsp. vulgaris] >XP_010682754.1 PREDICTED: squamosa promoterbindinglike protein 2 [Beta vulgaris subsp. vulgaris] >XP_010682755.1 PREDICTED: squamosa	gi|521684943|gb|GA IA01025054.1|:TSA: Salicornia europaea Unigene26886_Se20 0S transcribed RNA sequence	A0A0J8C109|A0A0 J8C109_BETVU:Un characterized protein;OS=Beta vulgaris subsp. vulgaris.;|GO:00056 34,|GO:0003677,

geneid	trend	GO	Pathway	TF	NR	NT	Uniprot
DN123181_c1_g1_i3	DOWN	[GO:0050832:defense response to fungus][GO:0031640:killing of cells of other organism]	-	-	XP_021857496.1:defensinlike protein [Spinacia oleracea] >KNA06414.1 hypothetical protein SOVF_181290 [Spinacia oleracea]	-	A0A0K9QGJ2|A0A0K9QGJ2_SPIOL:Uncharacterized protein;OS=Spinacia oleracea;|GO:0050832,GO:0031640,
DN119632_c0_g1_i1	DOWN	[GO:0005524:ATP binding][GO:0004356:glutamate-ammonia ligase activity][GO:0006542:glutamine biosynthetic process]	[ko00220:Arginine biosynthesis][ko00250:Alanine, aspartate and glutamate metabolism][ko00630:Glyoxylate and dicarboxylate metabolism][ko00910:Nitrogen metabolism][ko02020:Two-component system][ko04217:Necroptosis][ko04724:Glutamatergic synapse][ko04727:GABAergic synapse]	-	NP_001313793.1:glutamine synthetase nodule isozymelike [Gossypium hirsutum] >XP_012459580.1 PREDICTED: glutamine synthetase nodule isozymelike [Gossypium raimondii] >ACJ11724.1 glutamine synthase [Gossypium hirsutum] >KJB77349.1 hypothetical protein B456_012G133400	gi|13173418|gb|AF343667.1|AF343667: Beta vulgaris cytosolic glutamine synthetase (gln1) mRNA, complete cds	A0A0D2VZ01|A0A0D2VZ01_GOSRA: Glutamine synthetase;OS=Gossypium raimondii;|GO:0005524,GO:0004356,|GO:0006542,

geneid	trend	GO	Pathway	TF	NR	NT	Uniprot
					[Gossypium raimondii] >KJB77350.1 hypothetical protein B456_012G133400 [Gossypium raimondii]		
DN126708_c0_g1_i2	DOWN	[GO:0016021:integral component of membrane][GO:0008270:zinc ion binding]	-	DCAR_024960:Daucus carota|C3H|C3H family protein	XP_021758691.1:E3 ubiquitinprotein ligase ATL6like [Chenopodium quinoa]	-	A0A0K9RQC3|A0A0K9RQC3_SPIOL: Uncharacterized protein;OS=Spinacia oleracea;|GO:0016021,|GO:0008270,
DN127500_c0_g1_i3	DOWN	[GO:0003676:nucleic acid binding][GO:0015074:DNA integration]	-	676783392:Sisymbrium irio|B3|B3 family protein	XP_021715949.1:uncharacterized protein LOC110683848	-	M1ANG9|M1ANG9_SOLTU:Uncharacterized

geneid	trend	GO	Pathway	TF	NR	NT	Uniprot
					[Chenopodium quinoa]		protein;OS=Solanum tuberosum;|GO:0003676,|GO:0015074,
DN119897_c6_g1_i8	DOWN	-	-	-	XP_021857297.1:BRO1 domaincontaining protein BROXlike [Spinacia oleracea] >XP_021857298.1 BRO1 domaincontaining protein BROXlike [Spinacia oleracea] >KNA16292.1 hypothetical protein SOVF_090290 [Spinacia oleracea]	-	A0A0K9RBX6|A0A0K9RBX6_SPIOL: Uncharacterized protein;OS=Spinacia oleracea;

geneid	trend	GO	Pathway	TF	NR	NT	Uniprot
DN125703_c2_g1_i1	DOWN	[GO:0043565:sequence-specific DNA binding][GO:0003700:DNA-binding transcription factor activity]	-	Bv8_185810_kdqu.t1:Beta vulgaris|WRKY|WRKY family protein	XP_010686806.1:PREDICTED: probable WRKY transcription factor 31 isoform X1 [Beta vulgaris subsp. vulgaris] >KMT04131.1 hypothetical protein BVRB_8g185810 [Beta vulgaris subsp. vulgaris]	gi|406856225|gb|JX416201.1|:Tamarix hispida WRKY transcription factor 12 (WRKY12) gene, complete cds	A0A0J8EMC3|A0A0J8EMC3_BETVU: Uncharacterized protein;OS=Beta vulgaris subsp. vulgaris.;|GO:0043565,GO:0003700,
DN116116_c2_g2_i3	DOWN	[GO:0016021:integral component of membrane]	[ko04075:Plant hormone signal transduction]	-	XP_010689616.1:PREDICTED: auxin transporterlike protein 3 [Beta vulgaris subsp. vulgaris] >KMT19998.1 hypothetical protein BVRB_1g000210 [Beta vulgaris subsp. vulgaris]	gi|388523056|gb|BT149796.1|:Medicago truncatula clone JCVI-FLMt-19K8 unknown mRNA	A0A0J8D713|A0A0J8D713_BETVU:Uncharacterized protein;OS=Beta vulgaris subsp. vulgaris.;|GO:0016021,

geneid	trend	GO	Pathway	TF	NR	NT	Uniprot
DN121348_c2_g1_i2	DOWN	[GO:0046872:metal ion binding][GO:0004722:protein serine/threonine phosphatase activity]	[ko04016:MAPK signaling pathway - plant][ko04075:Plant hormone signal transduction][ko04931:Insulin resistance]	-	AAC36697.1:protein phosphatase2C [Mesembryanthemum crystallinum]	gi|521503757|gb|GAHZ01027163.1|:TSA: Salicornia europaea Unigene29887_SeCKS transcribed RNA sequence	O82468|O82468_MESCR:Protein phosphatase-2C;OS=Mesembryanthemum m crystallinum;|GO:0046872,GO:0004722,
DN125731_c4_g12_i1	DOWN	-	-	-	-	-	-
DN123940_c2_g2_i3	DOWN	-	-	-	-	-	-
DN127959_c2_g1_i4	DOWN	-	-	-	KMT15128.1:hypothetical protein BVRB_3g062460 [Beta vulgaris subsp. vulgaris]	-	A0A0J8CT63|A0A0J8CT63_BETVU:Uncharacterized protein;OS=Beta vulgaris subsp. vulgaris.;

geneid	trend	GO	Pathway	TF	NR	NT	Uniprot
DN114985_c4_g2_i2	DOWN	[GO:0016787:hydrolase activity][GO:0008152:metabolic process]	-	OMERI12G01050.1:Oryza meridionalis|NAC|NAC family protein	XP_010671193.1:PREDICTED: probable carboxylesterase 12 [Beta vulgaris subsp. vulgaris] >XP_019103957.1 PREDICTED: probable carboxylesterase 12 [Beta vulgaris subsp. vulgaris] >XP_019103958.1 PREDICTED: probable carboxylesterase 12 [Beta vulgaris subsp. vulgaris] >XP_019103959.1 PREDICTED: probable carboxylesterase 12 [Beta vulgaris subsp. vulgaris]	-	A0A0J8CSI6|A0A0J8CSI6_BETVU:Uncharacterized protein;OS=Beta vulgaris subsp. vulgaris.;|GO:0016787,|GO:0008152,

geneid	trend	GO	Pathway	TF	NR	NT	Uniprot
DN120551_c0_g2_i3	DOWN	[GO:0009793:embryo development ending in seed dormancy][GO:0098755:maintenance of seed dormancy by absisic acid][GO:0010029:regulation of seed germination][GO:0009845:seed germination]	-	-	XP_021745794.1:GEMlike protein 5 [Chenopodium quinoa]	-	A0A0J8C550|A0A0J8C550_BETVU:Uncharacterized protein;OS=Beta vulgaris subsp. vulgaris.;|GO:0009793,GO:0098755,GO:0010029,GO:0009845,
DN116435_c0_g2_i1	DOWN	-	-	Csa09g092790.1:Camelina sativa|BES1|BES1 family protein	XP_021842470.1:protein trichome birefringencelike 41 [Spinacia oleracea]	gi|521666877|gb|GAIA01034095.1|:TSA: Salicornia europaea Unigene37626_Se200S transcribed RNA sequence	A0A0J8CMH9|A0A0J8CMH9_BETVU: Uncharacterized protein;OS=Beta vulgaris subsp. vulgaris.;
DN127293_c1_g8_i2	DOWN	-	[ko04075:Plant hormone signal transduction]	-	XP_010685279.1:PREDICTED: protein TIFY 10Alike [Beta vulgaris subsp. vulgaris]	-	A0A0B4RUR8|A0A0B4RUR8_HEVBR: JAZ2;OS=Hevea brasiliensis;
DN125544_c3_g1_i1	DOWN	-	-	OPUNC04G28460.1:Oryza punctata|NAC|NAC family protein	XP_006487559.1:PREDICTED: probable beta1,4xylosyltransferase IRX10 [Citrus	gi|224075446|ref|XM_002304601.1|:Populus trichocarpa predicted protein, mRNA	A0A067EW49|A0A067EW49_CITSI:Uncharacterized protein;OS=Citrus sinensis;

geneid	trend	GO	Pathway	TF	NR	NT	Uniprot
					sinensis]		
DN118949_c4_g1_i12	DOWN	[GO:0016747:transferase activity, transferring acyl groups other than amino-acyl groups]	-	MDP0000294614:Malus domestica|NAC|NAC family protein	XP_021765621.1:uncharacterized acetyltransferase At3g50280like [Chenopodium quinoa]	-	A0A0K9RMS2|A0A0K9RMS2_SPIOL:Uncharacterized protein;OS=Spinacia oleracea;|GO:0016747,
DN125386_c0_g1_i1	DOWN	[GO:0005509:calcium ion binding]	-	GSVIVT01013228001:Vitis vinifera|MYB_related|MYB_related family protein	XP_010694463.1:PREDICTED: calciumbinding protein PBP1 [Beta vulgaris subsp. vulgaris] >KMS98389.1 hypothetical protein BVRB_4g093410 [Beta vulgaris subsp. vulgaris]	gi|359474486|ref|XM_002279071.2|:PREDICTED: Vitis vinifera calcium-binding protein PBP1-like (LOC100260800), mRNA	A0A0J8BAJ0|A0A0J8BAJ0_BETVU:Uncharacterized protein;OS=Beta vulgaris subsp. vulgaris.;|GO:0005509,

geneid	trend	GO	Pathway	TF	NR	NT	Uniprot
DN123919_c2_g1_i1	DOWN	[GO:0005886:plasma membrane][GO:0008081:phosphoric diester hydrolase activity][GO:0006629:lipid metabolic process]	-	Kaladp0020s0147.1.p:Kalanchoe laxiflora|bZIP|bZIP family protein	XP_021714320.1:PI PLC X domaincontaining protein At5g67130like [Chenopodium quinoa]	-	A0A0J8B2Q3|A0A0J8B2Q3_BETVU:Uncharacterized protein;OS=Beta vulgaris subsp. vulgaris.;|GO:0005886,|GO:0008081,|GO:0006629,
DN123348_c0_g4_i1	DOWN	-	-	-	-	-	-
DN116640_c2_g7_i1	DOWN	[GO:0016021:integral component of membrane]	-	Bv5_101120_supk.t1:Beta vulgaris|ERF|ERF family protein	XP_010667424.1:PREDICTED: sugar transport protein 13 [Beta vulgaris subsp. vulgaris] >KMS95389.1 hypothetical protein BVRB_008680 [Beta vulgaris subsp. vulgaris]	gi|449433328|ref|XM_004134402.1|:PREDICTED: Cucumis sativus sugar transport protein 13-like (LOC101222532), mRNA	A0A0J8B2P4|A0A0J8B2P4_BETVU:Uncharacterized protein;OS=Beta vulgaris subsp. vulgaris.;|GO:0016021,|GO:0022891,
DN125598_c0_g1_i2	DOWN	[GO:0008146:sulfotransferase activity]	-	676777512:Sisymbrium irio|ERF|ERF family protein	XP_010672565.1:PREDICTED: cytosolic sulfotransferase	-	A0A0J8BW20|A0A0J8BW20_BETVU:Sulfotransferase;OS=Beta vulgaris

geneid	trend	GO	Pathway	TF	NR	NT	Uniprot
					5like [Beta vulgaris subsp. vulgaris]		subsp. vulgaris.;|GO:0008146,
DN125606_c3_g2_i1	DOWN	-	-	-	XP_021730198.1:xyloglucan galactosyltransferase XLT2like [Chenopodium quinoa]	gi|359490235|ref|XM_002270273.2|:PREDICTED: Vitis vinifera xyloglucan galactosyltransferase KATAMARI1 homolog (LOC100263561), mRNA	A0A0K9RE55|A0A0K9RE55_SPIOL:Uncharacterized protein;OS=Spinacia oleracea;
DN117048_c0_g3_i2	DOWN	[GO:0005524:ATP binding][GO:0019538:protein metabolic process][GO:1902347:response to strigolactone]	-	-	XP_021732477.1:protein SMAX1LIKE 8like [Chenopodium quinoa]	-	A0A0J8FQZ6|A0A0J8FQZ6_BETVU:Uncharacterized protein;OS=Beta vulgaris subsp. vulgaris.;|GO:0005524,|GO:0019538,GO:1902347,
DN128703_c2_g5_i1	DOWN	[GO:0003676:nucleic acid binding]	-	Itr_sc000795.1_g00005.1:Ipomoea trifida|ERF|ERF family protein	CCA66050.1:hypothetical protein [Beta vulgaris subsp. vulgaris]	-	F4NCJ4|F4NCJ4_BETVU:Uncharacterized protein;OS=Beta vulgaris subsp.

geneid	trend	GO	Pathway	TF	NR	NT	Uniprot
							vulgaris.;|GO:0003676,
DN111897_c0_g1_i3	DOWN	[GO:0016021:integral component of membrane][GO:0009055:electron transfer activity]	-	-	XP_010665738.1:PREDICTED: mavicyanin [Beta vulgaris subsp. vulgaris] >KMS96601.1 hypothetical protein BVRB_8g201310 [Beta vulgaris subsp. vulgaris]	-	A0A0J8B6I5|A0A0J8B6I5_BETVU:Uncharacterized protein;OS=Beta vulgaris subsp. vulgaris.;|GO:0016021,|GO:0009055,

geneid	trend	GO	Pathway	TF	NR	NT	Uniprot
DN120299_c1_g3_i1	DOWN	[GO:0005829:cytosol][GO:0005634:nucleus][GO:0005886:plasma membrane][GO:0009536:plastid][GO:0005524:ATP binding][GO:0004674:protein serine/threonine kinase activity][GO:0007584:response to nutrient][GO:0009414:response to water deprivation][GO:0007165:signal transduction][GO:0010118:stomatal movement]	-	Lus10043264:Linum usitatissimum|YABBY|YABBY family protein	XP_010687109.1:PREDICTED: CBLinteracting serine/threonineprotein kinase 23 [Beta vulgaris subsp. vulgaris] >KMT03898.1 hypothetical protein BVRB_8g187980 [Beta vulgaris subsp. vulgaris]	gi|521527312|gb|GAHZ01009731.1|:TSA: Salicornia europaea Unigene10268_SeCKS transcribed RNA sequence	A0A0J8BVC7|A0A0J8BVC7_BETVU: Non-specific serine/threonine protein kinase;OS=Beta vulgaris subsp. vulgaris.;|GO:0005829,GO:0005634,GO:0005886,GO:0009536,|GO:0005524,GO:0004674,|GO:0010107,GO:0007584,GO:0009414,GO:0007165,GO:0010118,
DN127581_c1_g7_i12	DOWN	[GO:0016021:integral component of membrane][GO:0005524:ATP binding][GO:0016887:ATPase activity]	-	Vradi02g06060.1:Vigna radiata|TCP|TCP family protein	KNA06455.1:hypothetical protein SOVF_180860, partial [Spinacia oleracea]	gi|255580206|ref|XM_002530888.1|:Ricinus communis Protein white, putative, mRNA	A0A0K9QGV7|A0A0K9QGV7_SPIOL: Uncharacterized protein;OS=Spinacia oleracea;|GO:0016021,|GO:0005524,GO:0016887,

geneid	trend	GO	Pathway	TF	NR	NT	Uniprot
DN110825_c0_g1_i1	DOWN	-	-	-	XP_021772444.1:uncharacterized protein LOC110736524 [Chenopodium quinoa]	-	A0A0J8CQ97|A0A0J8CQ97_BETVU:Uncharacterized protein;OS=Beta vulgaris subsp. vulgaris.;
DN123169_c4_g1_i12	DOWN	[GO:0016021:integral component of membrane]	-	Zmw_sc06582.1.g00020.1:Zoysia matrella|C2H2|C2H2 family protein	XP_010249715.1:PREDICTED: uncharacterized protein LOC104592187 [Nelumbo nucifera]	gi|478161786|gb|GAHK01014817.1|:TSA: Jatropha curcas Unigene2271_JC-CK_1A transcribed RNA sequence	D7SUE2|D7SUE2_VITVI:Putative uncharacterized protein;OS=Vitis vinifera;GN=VIT_04s0008g03820 {ECO:0000313|EMBL:CBI20891.3};|GO:0016021,
DN122812_c3_g2_i2	DOWN	-	-	-	-	-	-
DN128158_c1_g1_i3	DOWN	[GO:0016021:integral component of membrane]	-	-	XP_019105768.1:PREDICTED: myblike protein X [Beta vulgaris subsp. vulgaris] >KMT09020.1 hypothetical protein	-	A0A0J8C5J6|A0A0J8C5J6_BETVU:Uncharacterized protein;OS=Beta vulgaris subsp. vulgaris.;|GO:0016021,

geneid	trend	GO	Pathway	TF	NR	NT	Uniprot
					BVRB_6g137260 [Beta vulgaris subsp. vulgaris]		
DN125260_c1_g1_i4	DOWN	[GO:0043565:sequence-specific DNA binding][GO:0003700:DNA-binding transcription factor activity]	-	Dca50783.1:Dianthus caryophyllus|WRKY|WRKY family protein	KJB63354.1:hypothetical protein B456_010G118300 [Gossypium raimondii]	gi|386686574|gb|JQ820201.1|:Camellia sinensis WRKY transcription factor 40 mRNA, complete cds	A0A0D2U306|A0A0D2U306_GOSRA: Uncharacterized protein;OS=Gossypium raimondii;|GO:0043565,GO:0003700,
DN124640_c0_g1_i2	DOWN	[GO:0016747:transferase activity, transferring acyl groups other than amino-acyl groups]	-	MDP0000294614:Malus domestica|NAC|NAC family protein	XP_021765621.1:uncharacterized acetyltransferase At3g50280like [Chenopodium quinoa]	-	A0A0K9RMS2|A0A0K9RMS2_SPIOL: Uncharacterized protein;OS=Spinacia oleracea;|GO:0016747,

geneid	trend	GO	Pathway	TF	NR	NT	Uniprot
DN118725_c0_g1_i2	DOWN	-	-	-	XP_010681076.1:PREDICTED: uncharacterized protein LOC104896080 [Beta vulgaris subsp. vulgaris] >KMT08649.1 hypothetical protein BVRB_6g139260 [Beta vulgaris subsp. vulgaris]	gi|521521236|gb|GAHZ01013338.1|:TSA: Salicornia europaea Unigene13979_SeCKS transcribed RNA sequence	A0A0J8C8S9|A0A0J8C8S9_BETVU:Uncharacterized protein;OS=Beta vulgaris subsp. vulgaris.;
DN129077_c2_g1_i2	DOWN	-	-	-	-	-	-
DN125140_c0_g1_i1	DOWN	[GO:0016021:integral component of membrane]	-	-	XP_010677360.1:PREDICTED: uncharacterized protein LOC104893019 [Beta vulgaris subsp. vulgaris] >KMT11358.1 hypothetical protein BVRB_5g106910 [Beta vulgaris subsp.	-	A0A0J8CH26|A0A0J8CH26_BETVU:Uncharacterized protein;OS=Beta vulgaris subsp. vulgaris.;|GO:0016021,

geneid	trend	GO	Pathway	TF	NR	NT	Uniprot
					vulgaris]		
DN123199_c1_g2_i4	DOWN	[GO:0016021:integral component of membrane]	-	-	XP_021856392.1:probable glycosyltransferase At5g03795 [Spinacia oleracea]	-	A0A0J8B786|A0A0J8B786_BETVU:Uncharacterized protein;OS=Beta vulgaris subsp. vulgaris.;|GO:0016021,
DN111579_c0_g1_i1	DOWN	[GO:0004553:hydrolase activity, hydrolyzing O-glycosyl compounds][GO:0005975:carbohydrate metabolic process]	[ko00520:Amino sugar and nucleotide sugar metabolism]	-	XP_018836110.1:PREDICTED: acidic endochitinaselike [Juglans regia]	gi|502094103|ref|XM_004490083.1|:PREDICTED: Cicer arietinum acidic endochitinase-like (LOC101495610), mRNA	M5XGX5|M5XGX5_PRUPE:Uncharacterized protein;OS=Prunus persica;|GO:0004553,|GO:0005975,

geneid	trend	GO	Pathway	TF	NR	NT	Uniprot
DN126513_c1_g4_i1	DOWN	-	-	Lus10003548:Linum usitatissimum|NAC|NAC family protein	KNA21232.1:hypothetical protein SOVF_045080 [Spinacia oleracea]	-	A0A0K9RQX5|A0A0K9RQX5_SPIOL: Uncharacterized protein;OS=Spinacia oleracea;

geneid	trend	GO	Pathway	TF	NR	NT	Uniprot
DN127489_c0_g2_i3	DOWN	[GO:0005739:mitochondrion][GO:0070469:respirasome][GO:0009055:electron transfer activity][GO:0020037:heme binding][GO:0046872:metal ion binding][GO:0055114:oxidation-reduction process]	[ko00920:Sulfur metabolism][ko01524:Platinum drug resistance][ko02020:Two-component system][ko04115:p53 signaling pathway][ko04210:Apoptosis][ko04214:Apoptosis - fly][ko04215:Apoptosis - multiple species][ko04932:Non-alcoholic fatty liver disease (NAFLD)][ko05010:Alzheimer's disease][ko05012:Parkinson's disease][ko05014:Amyotrophic lateral sclerosis (ALS)][ko05016:Huntington's disease][ko05134:Legionellosis][ko05145:Toxoplasmosis][ko05152:Tuberculosis][ko05161:Hepatitis B][ko05164:Influenza a	-	XP_008795309.1:PREDICTED: cytochrome c [Phoenix dactylifera]	gi|521675355|gb|GAIA01029553.1|:TSA: Salicornia europaea Unigene32406_Se200S transcribed RNA sequence	A0A0D6R8Z7|A0A0D6R8Z7_ARACU: Uncharacterized protein;OS=Araucaria cunninghamii;|GO:0005739,GO:0070469,|GO:0009055,GO:0020037,GO:0046872,|GO:0055114,

geneid	trend	GO	Pathway	TF	NR	NT	Uniprot
DN121211_c 1_g9_i1	DOWN	[GO:0005634:nucleus][GO:0043565: sequence-specific DNA binding][GO:0003700:DNA-binding transcription factor activity]	-	Bv_003250_arsd.t1: Beta vulgaris|HSF|HSF family protein	XP_010666616.1:P REDICTED: heat shock factor protein HSF24 [Beta vulgaris subsp. vulgaris] >KMS95964.1 hypothetical protein BVRB_003250 [Beta vulgaris subsp. vulgaris]	gi|5821135|dbj|AB0 14483.1|:Nicotiana tabacum NtHSF1 mRNA for heat shock factor, complete cds	A0A0J8B452|A0A0 J8B452_BETVU:Un characterized protein;OS=Beta vulgaris subsp. vulgaris.;|GO:00056 34,|GO:0043565,GO :0003700,

geneid	trend	GO	Pathway	TF	NR	NT	Uniprot
DN125900_c0_g2_i1	DOWN	[GO:0005768:endosome][GO:0016021:integral component of membrane][GO:0005886:plasma membrane][GO:0005524:ATP binding][GO:0004674:protein serine/threonine kinase activity][GO:0005496:steroid binding][GO:0048657:anther wall tapetum cell differentiation][GO:0009742:brassinosteroid mediated signaling pathway][GO:0009729:detection of brassinosteroid stimulus][GO:0048366:leaf development][GO:0060548:negative regulation of cell death][GO:0010584:pollen exine formation][GO:0009911:positive regulation of flower development][GO:1900140:regulation of seedling development][GO:0010224:response to UV-B]	[ko04075:Plant hormone signal transduction]	mrna27137.1-v1.0-hybrid:Fragaria vesca|STAT|STAT family protein	XP_010680326.1:PREDICTED: systemin receptor SR160 [Beta vulgaris subsp. vulgaris]	gi|512400340|gb|GAJZ01000830.1|:TSA: Prunus avium Pa_01629 transcribed RNA sequence	F6H4C0|F6H4C0_VITVI:Putative uncharacterized protein;OS=Vitis vinifera;GN=VIT_07s0031g01850 {ECO:0000313|EMBL:CCB47131.1};|GO:0005768,GO:0016021,GO:0005886,|GO:0005524,GO:0004674,GO:0005496,|GO:0048657,GO:0009742,GO:0009729,GO:0048366,GO:0060548,GO:0010584,GO:0009911,GO:1900140,GO:0010224,

geneid	trend	GO	Pathway	TF	NR	NT	Uniprot
DN117527_c0_g4_i11	DOWN	[GO:0016021:integral component of membrane][GO:0008270:zinc ion binding]	-	-	XP_010689560.1:PREDICTED: uncharacterized protein LOC104903248 [Beta vulgaris subsp. vulgaris] >KMT02075.1 hypothetical protein BVRB_9g208040 [Beta vulgaris subsp. vulgaris]	-	A0A0J8BKZ3|A0A0J8BKZ3_BETVU:Uncharacterized protein;OS=Beta vulgaris subsp. vulgaris.;|GO:0016021,|GO:0008270,
DN121547_c1_g1_i2	DOWN	-	-	-	XP_010688094.1:PREDICTED: EG45like domain containing protein isoform X2 [Beta vulgaris subsp. vulgaris] >KMT02958.1 hypothetical protein BVRB_8g195280 [Beta vulgaris subsp. vulgaris]	-	A0A0J8BST7|A0A0J8BST7_BETVU:Uncharacterized protein;OS=Beta vulgaris subsp. vulgaris.;

geneid	trend	GO	Pathway	TF	NR	NT	Uniprot
DN124100_c6_g11_i1	DOWN	-	-	-	-	-	-
DN128780_c2_g14_i1	DOWN	-	-	-	-	-	-
DN128908_c4_g1_i1	DOWN	[GO:0016491:oxidoreductase activity]	-	-	OWM70964.1:hypothetical protein CDL15_Pgr013145 [Punica granatum]	gi|363806929|ref|NM_001255633.1|:Glycine max auxin-induced protein PCNT115-like (LOC100779689), mRNA >gi|255639462|gb|BT095792.1| Soybean clone JCVI-FLGm-19L4 unknown mRNA	A9PGV9|A9PGV9_POPTR:Putative uncharacterized protein;OS=Populus trichocarpa;|GO:0016491,
DN116295_c0_g2_i1	DOWN	-	-	-	-	-	-
DN123455_c1_g1_i1	DOWN	-	-	-	XP_021764790.1:myblike protein X [Chenopodium quinoa]	-	A0A0K9Q9D4|A0A0K9Q9D4_SPIOL:Uncharacterized protein;OS=Spinacia oleracea;

geneid	trend	GO	Pathway	TF	NR	NT	Uniprot
DN125521_c1_g4_i5	DOWN	-	-	-	XP_010672716.1:PREDICTED: polygalacturonase inhibitor 1 [Beta vulgaris subsp. vulgaris] >KMT15372.1 hypothetical protein BVRB_3g060770 [Beta vulgaris subsp. vulgaris]	-	A0A0J8CPC2|A0A0J8CPC2_BETVU:Uncharacterized protein;OS=Beta vulgaris subsp. vulgaris.;
DN127884_c2_g2_i8	DOWN	-	-	-	-	-	-
DN109640_c0_g1_i3	DOWN	-	-	-	XP_010690436.1:PREDICTED: prolinerich protein 4 [Beta vulgaris subsp. vulgaris] >KMT01451.1 hypothetical protein BVRB_9g214580 [Beta vulgaris subsp. vulgaris]	gi|521538282|gb|GAHZ01004231.1|:TSA: Salicornia europaea Unigene4520_SeCKS transcribed RNA sequence	A0A0J8EEP5|A0A0J8EEP5_BETVU:Uncharacterized protein;OS=Beta vulgaris subsp. vulgaris.;

geneid	trend	GO	Pathway	TF	NR	NT	Uniprot
DN125046_c0_g1_i2	DOWN	[GO:0016021:integral component of membrane][GO:0005509:calcium ion binding]	-	OB11G10730.1:Oryza brachyantha|WRKY|WRKY family protein	XP_021723277.1:vacuolarsorting receptor 1like [Chenopodium quinoa]	gi|512395009|gb|GAJZ01006161.1|:TSA: Prunus avium Pa_17800 transcribed RNA sequence	A0A0K9QR89|A0A0K9QR89_SPIOL:Uncharacterized protein;OS=Spinacia oleracea;|GO:0016021,|GO:0005509,
DN118603_c2_g2_i1	DOWN	[GO:0005524:ATP binding][GO:0004674:protein serine/threonine kinase activity]	-	RrC264_p4:Raphanus raphanistrum|NF-YC|NF-YC family protein	KMT05025.1:hypothetical protein BVRB_7g171850 [Beta vulgaris subsp. vulgaris]	-	A0A0J8BU19|A0A0J8BU19_BETVU:Uncharacterized protein;OS=Beta vulgaris subsp. vulgaris.;|GO:0005524,GO:0004674,
DN126994_c2_g29_i1	DOWN	-	-	-	-	-	-

geneid	trend	GO	Pathway	TF	NR	NT	Uniprot
DN114918_c0_g1_i2	DOWN	[GO:0005622:intracellular][GO:0045454:cell redox homeostasis][GO:0035556:intracellular signal transduction]	-	-	XP_006435249.1:hypothetical protein CICLE_v10001187mg [Citrus clementina] >ESR48489.1 hypothetical protein CICLE_v10001187mg [Citrus clementina] >KDO84956.1 hypothetical protein CISIN_1g013684mg [Citrus sinensis] >KDO84957.1 hypothetical protein CISIN_1g013684mg [Citrus sinensis]	gi|502131229|ref|XM_004500860.1|:PREDICTED: Cicer arietinum probable nucleoredoxin 2-like (LOC101498505), transcript variant X2, mRNA	A0A067HB84|A0A067HB84_CITSI:Uncharacterized protein;OS=Citrus sinensis;|GO:0005622,|GO:0045454,GO:0035556,
DN116167_c5_g11_i7	DOWN	[GO:0016021:integral component of membrane]	-	-	XP_019106730.1:PREDICTED: uncharacterized protein LOC104901121 isoform X2 [Beta vulgaris subsp.	-	A0A0J8BVU5|A0A0J8BVU5_BETVU:Uncharacterized protein;OS=Beta vulgaris subsp. vulgaris.;|GO:0016021,

geneid	trend	GO	Pathway	TF	NR	NT	Uniprot
					vulgaris]		
DN128568_c1_g7_i1	DOWN	[GO:0016021:integral component of membrane]	-	MDP0000131822:Malus domestica|MYB|MYB family protein	XP_010692891.1:PREDICTED: uncharacterized protein LOC104905931 [Beta vulgaris subsp. vulgaris] >KMS99496.1 hypothetical protein BVRB_1g023360 [Beta vulgaris subsp. vulgaris]	gi|502118148|ref|XM_004496067.1|:PREDICTED: Cicer arietinum uncharacterized LOC101496417 (LOC101496417), mRNA	A0A0J8BEX8|A0A0J8BEX8_BETVU:Uncharacterized protein;OS=Beta vulgaris subsp. vulgaris.;|GO:0016021,
DN129195_c59_g1_i2	DOWN	[GO:0016491:oxidoreductase activity]	[ko00902:Monoterpenoid biosynthesis]	-	XP_002267348.2:PREDICTED: (+)neomenthol dehydrogenase isoform X1 [Vitis vinifera] >CBI40297.3	-	D7UC32|D7UC32_VITVI:Putative uncharacterized protein;OS=Vitis vinifera;GN=VIT_15s0046g03590 {ECO:0000313|EM

geneid	trend	GO	Pathway	TF	NR	NT	Uniprot
					unnamed protein product, partial [Vitis vinifera]		BL:CBI40297.3};|GO:0016491,
DN112980_c0_g1_i1	DOWN	[GO:0016021:integral component of membrane][GO:0005524:ATP binding][GO:0004674:protein serine/threonine kinase activity]	-	mrna27137.1-v1.0-hybrid:Fragaria vesca|STAT|STAT family protein	XP_021761567.1:probable LRR receptorlike serine/threonineprotein kinase At1g74360 [Chenopodium quinoa]	-	A0A0K9RI05|A0A0K9RI05_SPIOL:Uncharacterized protein;OS=Spinacia oleracea;|GO:0016021,|GO:0005524,GO:0004674,
DN128682_c2_g5_i14	DOWN	[GO:0046872:metal ion binding][GO:0016702:oxidoreductase activity, acting on single donors with incorporation of molecular oxygen, incorporation of two atoms of oxygen][GO:0006633:fatty acid biosynthetic process][GO:0031408:oxylipin biosynthetic process]	[ko00591:Linoleic acid metabolism][ko00592:alpha-Linolenic acid metabolism]	Lus10031505:Linum usitatissimum|NF-YA|NF-YA family protein	XP_021772004.1:linoleate 13Slipoxygenase 21, chloroplasticlike [Chenopodium quinoa]	-	Q8W418|Q8W418_CITJA:Lipoxygenase;OS=Citrus jambhiri;|GO:0046872,GO:0016702,|GO:0006633,GO:0031408,

geneid	trend	GO	Pathway	TF	NR	NT	Uniprot
DN120465_c1_g1_i1	DOWN	-	-	-	KMT08309.1:hypothetical protein BVRB_6g141760 [Beta vulgaris subsp. vulgaris]	-	A0A0J8C7S2|A0A0J8C7S2_BETVU:Uncharacterized protein;OS=Beta vulgaris subsp. vulgaris.;
DN126316_c3_g1_i4	DOWN	[GO:0016021:integral component of membrane][GO:0008168:methyltransferase activity]	-	CCG010610.1:Populus euphratica|NAC|NAC family protein	XP_021851572.1:probable methyltransferase PMT21 [Spinacia oleracea] >XP_021851580.1 probable methyltransferase PMT21 [Spinacia oleracea] >KNA24870.1 hypothetical protein SOVF_011830 [Spinacia oleracea]	gi|460400803|ref|XM_004245874.1|:PREDICTED: Solanum lycopersicum probable methyltransferase PMT21-like, transcript variant 2 (LOC101251208), mRNA	A0A0K9RZD8|A0A0K9RZD8_SPIOL: Uncharacterized protein;OS=Spinacia oleracea;|GO:0016021,|GO:0008168,

geneid	trend	GO	Pathway	TF	NR	NT	Uniprot
DN117107_c0_g2_i3	DOWN	[GO:0016021:integral component of membrane][GO:0005215:transporter activity]	-	Prupe.3G009400.1.p:Prunus persica|ERF|ERF family protein	XP_006436425.1:hypothetical protein CICLE_v10032302mg [Citrus clementina] >XP_006485687.1 PREDICTED: probable aquaporin PIP25 [Citrus sinensis] >ESR49665.1 hypothetical protein CICLE_v10032302mg [Citrus clementina]	gi|224113030|ref|XM_002316331.1|:Populus trichocarpa aquaporin, MIP family, PIP subfamily, mRNA	A0A067E1I1|A0A067E1I1_CITSI:Uncharacterized protein;OS=Citrus sinensis;|GO:0016021,|GO:0005215,
DN123438_c0_g2_i8	DOWN	[GO:0016758:transferase activity, transferring hexosyl groups][GO:0008152:metabolic process]	-	TRIUR3_17264-P1:Triticum urartu|GRAS|GRAS family protein	XP_010676160.1:PREDICTED: 7deoxyloganetin glucosyltransferase [Beta vulgaris subsp. vulgaris] >KMT19826.1 hypothetical protein BVRB_1g008780 [Beta vulgaris subsp.	gi|521542941|gb|GAHZ01001959.1|:TSA: Salicornia europaea Unigene2010_SeCKS transcribed RNA sequence	A0A0J8FW01|A0A0J8FW01_BETVU:Glycosyltransferase;OS=Beta vulgaris subsp. vulgaris.;|GO:0016758,|GO:0008152,

geneid	trend	GO	Pathway	TF	NR	NT	Uniprot
					vulgaris]		
DN120579_c1_g2_i1	DOWN	-	-	-	-	-	-
DN127007_c3_g2_i1	DOWN	-	-	-	-	-	-
DN129195_c59_g4_i2	DOWN	[GO:0016491:oxidoreductase activity]	[ko00902:Monoterpenoid biosynthesis]	-	XP_016193789.1:(+)neomenthol dehydrogenaselike [Arachis ipaensis]	-	A0A151TBI6|A0A151TBI6_CAJCA:(+)-neomenthol dehydrogenase;OS=Cajanus cajan;|GO:0016491,

geneid	trend	GO	Pathway	TF	NR	NT	Uniprot
DN119000_c3_g2_i1	DOWN	-	-	-	XP_010688158.1:PREDICTED: strigolactone esterase RMS3 [Beta vulgaris subsp. vulgaris] >KMT03028.1 hypothetical protein BVRB_8g195900 [Beta vulgaris subsp. vulgaris]	-	A0A0J8BT29|A0A0J8BT29_BETVU:Uncharacterized protein;OS=Beta vulgaris subsp. vulgaris.;
DN117161_c8_g5_i1	DOWN	[GO:0005524:ATP binding][GO:0004674:protein serine/threonine kinase activity][GO:0007165:signal transduction]	-	Lus10043264:Linum usitatissimum|YABBY|YABBY family protein	XP_010691588.1:PREDICTED: CBLinteracting serine/threonineprotein kinase 5 [Beta vulgaris subsp. vulgaris]	gi|255572732|ref|XM_002527253.1|:Ricinus communis CBL-interacting serine/threonine-protein kinase, putative, mRNA	A0A0J8BM29|A0A0J8BM29_BETVU: Non-specific serine/threonine protein kinase;OS=Beta vulgaris subsp. vulgaris.;|GO:0005524,GO:0004674,|GO:0007165,

geneid	trend	GO	Pathway	TF	NR	NT	Uniprot
DN119637_c1_g2_i1	DOWN	[GO:0016021:integral component of membrane][GO:0005622:intracellular][GO:0005215:transporter activity]	-	mrna15187.1-v1.0-hybrid:Fragaria vesca|bHLH|bHLH family protein	XP_021731549.1:phosphatidylinositol/phosphatidylcholine transfer protein SFH6like [Chenopodium quinoa]	gi|470106569|ref|XM_004289591.1|:PREDICTED: Fragaria vesca subsp. vesca uncharacterized LOC101312875 (LOC101312875), mRNA	A0A0J8BHE1|A0A0J8BHE1_BETVU:Uncharacterized protein;OS=Beta vulgaris subsp. vulgaris.;|GO:0016021,GO:0005622,|GO:0005215,
DN114394_c0_g1_i1	DOWN	[GO:0016021:integral component of membrane][GO:0005524:ATP binding][GO:0016887:ATPase activity][GO:0006810:transport]	-	Rsa1.0_03996.1_g00001.1:Raphanus sativus|MYB_related|MYB_related family protein	XP_010665917.1:PREDICTED: ABC transporter G family member 36 [Beta vulgaris subsp. vulgaris] >KMS96494.1 hypothetical protein BVRB_9g224870 [Beta vulgaris subsp. vulgaris]	gi|521457518|gb|GAHZ01062966.1|:TSA: Salicornia europaea Unigene75775_SeCKS transcribed RNA sequence	A0A0J8B5I1|A0A0J8B5I1_BETVU:Uncharacterized protein;OS=Beta vulgaris subsp. vulgaris.;|GO:0016021,|GO:0005524,GO:0016887,|GO:0006810,
DN126994_c2_g4_i1	DOWN	-	-	-	-	-	-

geneid	trend	GO	Pathway	TF	NR	NT	Uniprot
DN122730_c0_g2_i6	DOWN	[GO:0003824:catalytic activity][GO:0005992:trehalose biosynthetic process]	[ko00500:Starch and sucrose metabolism]	-	XP_021718969.1:probable alpha,alphatrehalose phosphate synthase [UDPforming] 9 isoform X2 [Chenopodium quinoa]	gi|224121669|ref|XM_002318607.1|:Populus trichocarpa predicted protein, mRNA	A0A0J8CEY8|A0A0J8CEY8_BETVU:Uncharacterized protein;OS=Beta vulgaris subsp. vulgaris.;|GO:0003824,|GO:0005992,
DN116663_c2_g4_i1	DOWN	[GO:0004519:endonuclease activity][GO:0003676:nucleic acid binding][GO:0006308:DNA catabolic process]	-	TRIUR3_03984-P1: Triticum urartu|TCP|TCP family protein	XP_010696599.1:PREDICTED: endonuclease 4 [Beta vulgaris subsp. vulgaris] >XP_010696600.1 PREDICTED: endonuclease 4 [Beta vulgaris subsp. vulgaris] >KMS96783.1 hypothetical protein BVRB_8g199660 [Beta vulgaris subsp. vulgaris]	-	A0A0J8B9W4|A0A0J8B9W4_BETVU: Uncharacterized protein;OS=Beta vulgaris subsp. vulgaris.;|GO:0004519,GO:0003676,|GO:0006308,
DN127884_c2_g2_i7	DOWN	-	-	-	-	-	-

geneid	trend	GO	Pathway	TF	NR	NT	Uniprot
DN126720_c2_g1_i1	DOWN	[GO:0008883:glutamyl-tRNA reductase activity][GO:0050661:NADP binding][GO:0006782:protoporphyrinogen IX biosynthetic process]	[ko00860:Porphyrin and chlorophyll metabolism]	-	XP_019107661.1:PREDICTED: glutamyltRNA reductase 1, chloroplastic isoform X1 [Beta vulgaris subsp. vulgaris]	gi|512400296|gb|GAJZ01000874.1|:TSA: Prunus avium Pa_01701 transcribed RNA sequence	A0A0J8BGX3|A0A0J8BGX3_BETVU: Glutamyl-tRNA reductase;OS=Beta vulgaris subsp. vulgaris.;|GO:0008883,GO:0050661,|GO:0006782,
DN119872_c2_g5_i1	DOWN	[GO:0005737:cytoplasm][GO:0016020:membrane][GO:0047372:acylglycerol lipase activity][GO:0004620:phospholipase activity][GO:0016042:lipid catabolic process]	-	-	XP_021729610.1:patatinlike protein 2 isoform X2 [Chenopodium quinoa]	-	U5FP13|U5FP13_POPTR:Patatin;OS=Populus trichocarpa;|GO:0005737,GO:0016020,|GO:0047372,GO:0004620,|GO:0016042,
DN119061_c2_g1_i2	DOWN	[GO:0046872:metal ion binding][GO:0004722:protein serine/threonine phosphatase activity]	[ko04016:MAPK signaling pathway - plant][ko04075:Plant hormone signal transduction][ko04931:Insulin resistance]	-	XP_021840400.1:probable protein phosphatase 2C 51 isoform X1 [Spinacia oleracea] >KNA09519.1 hypothetical protein SOVF_152860 isoform B [Spinacia oleracea]	-	A0A0K9QQG8|A0A0K9QQG8_SPIOL: Uncharacterized protein;OS=Spinacia oleracea;|GO:0046872,GO:0004722,

geneid	trend	GO	Pathway	TF	NR	NT	Uniprot
DN117491_c1_g1_i6	DOWN	-	[ko00480:Glutathione metabolism][ko00980:Metabolism of xenobiotics by cytochrome P450][ko00982:Drug metabolism - cytochrome P450][ko01524:Platinum drug resistance][ko05204:Chemical carcinogenesis][ko05418:Fluid shear stress and atherosclerosis]	-	XP_021729368.1:probable glutathione Stransferase parC [Chenopodium quinoa]	-	A0A0K9QL02|A0A0K9QL02_SPIOL:Uncharacterized protein;OS=Spinacia oleracea;
DN126104_c0_g1_i1	DOWN	-	-	-	XP_010691776.1:PREDICTED: uncharacterized protein At1g15400 [Beta vulgaris subsp. vulgaris]	-	-
DN122751_c1_g11_i8	DOWN	-	-	-	-	-	-

geneid	trend	GO	Pathway	TF	NR	NT	Uniprot
DN125188_c0_g3_i1	DOWN	[GO:0046872:metal ion binding][GO:0004722:protein serine/threonine phosphatase activity]	[ko04016:MAPK signaling pathway - plant][ko04075:Plant hormone signal transduction][ko04931:Insulin resistance]	-	XP_010685683.1:PREDICTED: probable protein phosphatase 2C 24 [Beta vulgaris subsp. vulgaris] >KMT05175.1 hypothetical protein BVRB_7g173230 [Beta vulgaris subsp. vulgaris]	-	A0A0J8BUE2|A0A0J8BUE2_BETVU:Uncharacterized protein;OS=Beta vulgaris subsp. vulgaris.;|GO:0046872,GO:0004722,
DN126766_c1_g1_i16	DOWN	-	-	-	KNA07601.1:hypothetical protein SOVF_170270 [Spinacia oleracea]	-	A0A0K9QM20|A0A0K9QM20_SPIOL:Uncharacterized protein;OS=Spinacia oleracea;
DN108522_c0_g1_i2	DOWN	[GO:0004869:cysteine-type endopeptidase inhibitor activity]	-	-	ABG89856.1:cystatin [Amaranthus hypochondriacus]	-	Q0GPA4|Q0GPA4_AMAHP:Cysteine proteinase inhibitor;OS=Amaranthus hypochondriacus;|GO:0004869,

geneid	trend	GO	Pathway	TF	NR	NT	Uniprot
DN125571_c3_g9_i2	DOWN	[GO:0016021:integral component of membrane][GO:0009506:plasmodesma][GO:0005249:voltage-gated potassium channel activity][GO:0042391:regulation of membrane potential]	-	-	XP_021727318.1:potassium channel AKT2/3like [Chenopodium quinoa]	-	A0A0J8C2X6|A0A0J8C2X6_BETVU:Uncharacterized protein;OS=Beta vulgaris subsp. vulgaris.;|GO:0016021,GO:0009506,|GO:0005249,|GO:0042391,
DN125250_c0_g11_i7	DOWN	[GO:0016157:sucrose synthase activity][GO:0005985:sucrose metabolic process]	[ko00500:Starch and sucrose metabolism]	mrna11429.1-v1.0-hybrid:Fragaria vesca|HB-other|HB-other family protein	AFS17277.1:sucrose synthase 1 [Amaranthus cruentus/Amaranthus hypocondriacus mixed library]	gi|405132079|gb|JQ012918.1|:Amaranthus cruentus/Amaranthus hypocondriacus mixed library sucrose synthase 1 mRNA, complete cds	J9WP95|J9WP95_9CARY:Sucrose synthase;OS=Amaranthus cruentus/Amaranthus hypocondriacus mixed library.;|GO:0016157,|GO:0005985,
DN124273_c1_g5_i1	DOWN	-	-	-	-	-	-

geneid	trend	GO	Pathway	TF	NR	NT	Uniprot
DN103966_c0_g1_i2	DOWN	[GO:0004747:ribokinase activity][GO:0006014:D-ribose metabolic process]	[ko00051:Fructose and mannose metabolism][ko00500:Starch and sucrose metabolism][ko00520:Amino sugar and nucleotide sugar metabolism]	Pbr018801.2:Pyrus bretschneideri|MIKC_MADS|MIKC_MADS family protein	XP_021751776.1:probable fructokinase4 [Chenopodium quinoa]	-	A0A151SUH9|A0A151SUH9_CAJCA: Fructokinase-2;OS= Cajanus cajan;|GO:0004747,|GO:0006014,
DN125326_c1_g4_i1	DOWN	-	-	DCAR_004769:Daucus carota|bHLH|bHLH family protein	XP_010678954.1:PREDICTED: transcription factor IBH1 [Beta vulgaris subsp. vulgaris] >KMT10085.1 hypothetical protein BVRB_5g118480 [Beta vulgaris subsp. vulgaris]	-	A0A0J8C8S1|A0A0J8C8S1_BETVU:Uncharacterized protein;OS=Beta vulgaris subsp. vulgaris.;
DN128916_c1_g2_i2	DOWN	-	-	-	XP_021620201.1:uncharacterized protein LOC110620688 [Manihot esculenta] >OAY43500.1 hypothetical protein MANES_08G07490	-	B9S1D8|B9S1D8_RICCO:Putative uncharacterized protein;OS=Ricinus communis;

geneid	trend	GO	Pathway	TF	NR	NT	Uniprot
					0 [Manihot esculenta]		
DN128881_c3_g1_i9	DOWN	[GO:0016021:integral component of membrane][GO:0008270:zinc ion binding]	-	Oropetium_20150105_23793A:Oropetium thomaeum|C3H|C3H family protein	XP_021745986.1:uncharacterized protein LOC110711854 [Chenopodium quinoa]	gi|470117775|ref|XM_004294977.1|:PREDICTED: Fragaria vesca subsp. vesca uncharacterized LOC101315129 (LOC101315129), mRNA	A0A0J8BKZ3|A0A0J8BKZ3_BETVU:Uncharacterized protein;OS=Beta vulgaris subsp. vulgaris.;|GO:0016021,|GO:0008270,
DN115629_c3_g2_i2	DOWN	[GO:0006457:protein folding]	-	Achn036321:Actinidia chinensis|G2-like|G2-like family protein	XP_021844662.1:dnaJ homolog subfamily B member 1like [Spinacia oleracea] >KNA09714.1 hypothetical protein SOVF_151130 [Spinacia oleracea]	gi|255558263|ref|XM_002520113.1|:Ricinus communis Curved DNA-binding protein, putative, mRNA	A0A0K9QQY5|A0A0K9QQY5_SPIOL: Uncharacterized protein;OS=Spinacia oleracea;|GO:0006457,

geneid	trend	GO	Pathway	TF	NR	NT	Uniprot
DN118441_c4_g4_i3	DOWN	[GO:0046872:metal ion binding][GO:0004722:protein serine/threonine phosphatase activity]	[ko04016:MAPK signaling pathway - plant][ko04075:Plant hormone signal transduction][ko04931:Insulin resistance]	XP_010536267.1:Tarenaya hassleriana|NAC|NAC family protein	XP_010685683.1:PREDICTED: probable protein phosphatase 2C 24 [Beta vulgaris subsp. vulgaris] >KMT05175.1 hypothetical protein BVRB_7g173230 [Beta vulgaris subsp. vulgaris]	-	A0A0J8BUE2|A0A0J8BUE2_BETVU:Uncharacterized protein;OS=Beta vulgaris subsp. vulgaris.;|GO:0046872,GO:0004722,
DN86043_c0_g1_i3	DOWN	-	-	-	-	-	-
DN114289_c1_g3_i1	DOWN	[GO:0005509:calcium ion binding]	-	GSVIVT01013228001:Vitis vinifera|MYB_related|MYB_related family protein	XP_008233101.1:PREDICTED: calciumbinding protein KIC [Prunus mume]	-	I3T2B5|I3T2B5_LOTJA:Uncharacterized protein;OS=Lotus japonicus;|GO:0005509,
DN125188_c0_g3_i2	DOWN	-	-	-	-	-	-
DN124100_c6_g14_i1	DOWN	-	-	-	-	-	-

geneid	trend	GO	Pathway	TF	NR	NT	Uniprot
DN120937_c0_g1_i3	DOWN	[GO:0005737:cytoplasm][GO:0005524:ATP binding][GO:0004335:galactokinase activity][GO:0006012:galactose metabolic process]	[ko00520:Amino sugar and nucleotide sugar metabolism]	-	XP_021850313.1:galacturonokinase [Spinacia oleracea] >KNA18884.1 hypothetical protein SOVF_064080 [Spinacia oleracea]	gi|242077113|ref|XM_002448448.1|:Sorghum bicolor hypothetical protein, mRNA	A0A0K9RIX3|A0A0K9RIX3_SPIOL:Uncharacterized protein;OS=Spinacia oleracea;|GO:0005737,|GO:0005524,GO:0004335,|GO:0006012,
DN118084_c3_g13_i3	DOWN	[GO:0016491:oxidoreductase activity]	-	Cc01_g08720:Coffea canephora|NAC|NAC family protein	XP_021860399.1:secoisolariciresinol dehydrogenaselike [Spinacia oleracea] >KNA15779.1 hypothetical protein SOVF_095070 [Spinacia oleracea]	-	A0A0K9R9V4|A0A0K9R9V4_SPIOL:Uncharacterized protein;OS=Spinacia oleracea;|GO:0016491,
DN124346_c8_g11_i1	DOWN	-	-	-	-	-	-
DN123326_c4_g1_i6	DOWN	-	-	-	OMO50323.1:hypothetical protein CCACVL1_30507 [Corchorus capsularis]	-	A0A151SFD2|A0A151SFD2_CAJCA:Retrovirus-related Pol polyprotein from transposon TNT 1-94;OS=Cajanus

geneid	trend	GO	Pathway	TF	NR	NT	Uniprot
							cajan;
DN121806_c0_g5_i2	DOWN	[GO:0008234:cysteine-type peptidase activity]	-	FANhyb_rscf00000253.1.g00010.1:Fragaria x ananassa|BBR-BPC|BBR-BPC family protein	AHA62778.1:RCR3like cysteine protease [Mirabilis jalapa]	-	V5M3B7|V5M3B7_MIRJA:RCR3-like cysteine protease;OS=Mirabilis jalapa;|GO:0008234,
DN121767_c1_g5_i1	DOWN	-	-	-	XP_010669252.1:PREDICTED: pollenspecific protein SF21 isoform X1 [Beta vulgaris subsp. vulgaris]	-	A0A0J8D1H1|A0A0J8D1H1_BETVU:Uncharacterized protein;OS=Beta vulgaris subsp. vulgaris.;

geneid	trend	GO	Pathway	TF	NR	NT	Uniprot
DN117874_c0_g1_i6	DOWN	[GO:0009507:chloroplast][GO:0000036:acyl carrier activity][GO:0016297:acyl-[acyl-carrier-protein] hydrolase activity]	[ko00061:Fatty acid biosynthesis]	-	XP_010688133.1:PREDICTED: palmitoylacyl carrier protein thioesterase, chloroplastic [Beta vulgaris subsp. vulgaris] >XP_010688134.1 PREDICTED: palmitoylacyl carrier protein thioesterase, chloroplastic [Beta vulgaris subsp. vulgaris] >XP_019106990.1 PREDICTED: palmitoylacyl carrier protein thioesterase, chloroplastic [Beta vulgaris subsp. vulgaris] >KMT02998.1 hypothetical protein BVRB_8g195640 [Beta vulgaris subsp.	gi|282160398|gb|FJ936170.1|:Macadamia tetraphylla chloroplast acyl-ACP thioesterase (FATB) mRNA, complete cds; nuclear gene for chloroplast product	A0A0J8BSY2|A0A0J8BSY2_BETVU:Acyl-[acyl-carrier-protein] hydrolase;OS=Beta vulgaris subsp. vulgaris.;|GO:0009507,|GO:0000036,GO:0016297,

geneid	trend	GO	Pathway	TF	NR	NT	Uniprot
					vulgaris]		
DN113791_c1_g2_i2	DOWN	[GO:0003824:catalytic activity]	-	-	XP_021767900.1:uncharacterized protein LOC110732290 [Chenopodium quinoa]	-	A0A0J8CK63|A0A0J8CK63_BETVU:Uncharacterized protein;OS=Beta vulgaris subsp.

geneid	trend	GO	Pathway	TF	NR	NT	Uniprot
							vulgaris.;|GO:0003824,
DN112388_c5_g6_i1	DOWN	-	-	-	-	-	-
DN117335_c0_g1_i1	DOWN	[GO:0016021:integral component of membrane][GO:0005524:ATP binding][GO:0030247:polysaccharide binding][GO:0004672:protein kinase activity]	[ko04064:NF-kappa B signaling pathway][ko04620:Toll-like receptor signaling pathway][ko04621:NOD-like receptor signaling pathway][ko04624:Toll and Imd signaling pathway][ko04722:Neurotrophin signaling pathway][ko05133:Pertussis][ko05140:Leishmaniasis][ko05142:Chagas disease	-	KMT13953.1:hypothetical protein BVRB_4g077880 isoform C [Beta vulgaris subsp. vulgaris]	-	A0A0J8CPJ2|A0A0J8CPJ2_BETVU:Uncharacterized protein;OS=Beta vulgaris subsp. vulgaris.;|GO:0016021,|GO:0005524,GO:0030247,GO:0004672,

geneid	trend	GO	Pathway	TF	NR	NT	Uniprot
			(American trypanosomiasis)][ko05145:Toxoplasmosis][ko05152:Tuberculosis][ko05162:Measles][ko05164:Influenza A]				
DN112599_c0_g1_i2	DOWN	-	-	-	-	-	-

geneid	trend	GO	Pathway	TF	NR	NT	Uniprot
DN117616_c0_g1_i3	DOWN	[GO:0009507:chloroplast][GO:0016020:membrane][GO:0003849:3-deoxy-7-phosphoheptulonate synthase activity][GO:0009073:aromatic amino acid family biosynthetic process][GO:0008652:cellular amino acid biosynthetic process]	[ko00400:Phenylalanine, tyrosine and tryptophan biosynthesis][ko02024:Quorum sensing]	-	XP_010676056.1:PREDICTED: phospho2dehydro3deoxyheptonate aldolase 1, chloroplastic [Beta vulgaris subsp. vulgaris] >KMT19815.1 hypothetical protein BVRB_1g008670 [Beta vulgaris subsp. vulgaris]	gi|521491658|gb|GAHZ01039228.1|:TSA: Salicornia europaea Unigene44447_SeCKS transcribed RNA sequence	A0A0J8D0Z8|A0A0J8D0Z8_BETVU:Phospho-2-dehydro-3-deoxyheptonate aldolase;OS=Beta vulgaris subsp. vulgaris.;|GO:0009507,GO:0016020,|GO:0003849,|GO:0009073,GO:0008652,
DN118842_c0_g1_i11	DOWN	[GO:0052692:raffinose alpha-galactosidase activity][GO:0005975:carbohydrate metabolic process]	[ko00052:Galactose metabolism][ko00561:Glycerolipid metabolism][ko00600:Sphingolipid metabolism][ko00603:Glycosphingolipid biosynthesis - globo and isoglobo series]	Peaxi162Scf01390g00031.1:Petunia axillaris|MYB_related|MYB_related family protein	XP_021750574.1:LOW QUALITY PROTEIN: alphagalactosidase 1like [Chenopodium quinoa]	gi|512394250|gb|GAJZ01006920.1|:TSA: Prunus avium Pa_25899 transcribed RNA sequence	A0A0K9RFS4|A0A0K9RFS4_SPIOL:Alpha-galactosidase;OS=Spinacia oleracea;|GO:0052692,|GO:0005975,

geneid	trend	GO	Pathway	TF	NR	NT	Uniprot
DN125969_c2_g1_i1	DOWN	[GO:0000079:regulation of cyclin-dependent protein serine/threonine kinase activity]	-	Gh_A08G1256:Gossypium hirsutum|WRKY|WRKY family protein	XP_010684268.1:PREDICTED: cyclinP31 [Beta vulgaris subsp. vulgaris] >KMT06145.1 hypothetical protein BVRB_7g163150 [Beta vulgaris subsp. vulgaris]	gi|224383137|gb|FJ038351.1|:Neea floribunda voucher NL110119 RNA polymerase beta subunit (rpoB) gene, partial cds; chloroplast	A0A0J8BX77|A0A0J8BX77_BETVU:Uncharacterized protein;OS=Beta vulgaris subsp. vulgaris.;|GO:0000079,
DN116435_c0_g10_i1	DOWN	-	-	-	XP_010675285.1:PREDICTED: protein trichome birefringencelike 41 [Beta vulgaris subsp. vulgaris]	-	A0A0J8CMH9|A0A0J8CMH9_BETVU: Uncharacterized protein;OS=Beta vulgaris subsp. vulgaris.;
DN113836_c0_g1_i4	DOWN	[GO:0046872:metal ion binding][GO:0030001:metal ion transport]	-	-	XP_010674786.1:PREDICTED: heavy metalassociated isoprenylated plant protein 32like [Beta vulgaris subsp. vulgaris]	gi|521746726|gb|GAIA01002496.1|:TSA: Salicornia europaea Unigene2553_Se200S transcribed RNA sequence	A0A0J8CJS6|A0A0J8CJS6_BETVU:Uncharacterized protein;OS=Beta vulgaris subsp. vulgaris.;|GO:0046872,|GO:0030001,

geneid	trend	GO	Pathway	TF	NR	NT	Uniprot
DN128244_c6_g1_i3	DOWN	[GO:0016021:integral component of membrane][GO:0005622:intracellular][GO:0005524:ATP binding][GO:0004143:diacylglycerol kinase activity][GO:0035556:intracellular signal transduction][GO:0007205:protein kinase C-activating G protein-coupled receptor signaling pathway]	[ko00561:Glycerolipid metabolism][ko00564:Glycerophospholipid metabolism][ko04070:Phosphatidylinositol signaling system][ko04072:Phospholipase D signaling pathway][ko05231:Choline metabolism in cancer]	-	XP_021853092.1:diacylglycerol kinase 2 [Spinacia oleracea] >XP_021853093.1 diacylglycerol kinase 2 [Spinacia oleracea] >XP_021853094.1 diacylglycerol kinase 2 [Spinacia oleracea] >KNA05158.1 hypothetical protein SOVF_193030 [Spinacia oleracea]	gi|224093683|ref|XM_002309918.1|:Populus trichocarpa predicted protein, mRNA	A0A0K9QES0|A0A0K9QES0_SPIOL:Diacylglycerol kinase;OS=Spinacia oleracea;|GO:0016021,GO:0005622,|GO:0005524,GO:0004143,|GO:0035556,GO:0007205,
DN128845_c1_g1_i5	DOWN	[GO:0004553:hydrolase activity, hydrolyzing O-glycosyl compounds][GO:0005975:carbohydrate metabolic process]	[ko00460:Cyanoamino acid metabolism][ko00500:Starch and sucrose metabolism][ko00940:Phenylpropanoid biosynthesis]	Jcr4S00282.90:Jatropha curcas|NAC|NAC family protein	XP_010675614.1:PREDICTED: betaglucosidase 18like [Beta vulgaris subsp. vulgaris]	-	A0A0J8CLZ5|A0A0J8CLZ5_BETVU:Uncharacterized protein;OS=Beta vulgaris subsp. vulgaris.;|GO:0004553,|GO:0005975,

geneid	trend	GO	Pathway	TF	NR	NT	Uniprot
DN117075_c0_g1_i5	DOWN	[GO:0004725:protein tyrosine phosphatase activity]	-	EMT21109:Aegilops tauschii|GeBP|GeBP family protein	XP_015959034.1:probable tyrosineprotein phosphatase At1g05000 [Arachis duranensis]	gi|521546124|gb|GAHZ01000583.1|:TSA: Salicornia europaea Unigene597_SeCKS transcribed RNA sequence	A0A067EKA5|A0A067EKA5_CITSI:Uncharacterized protein;OS=Citrus sinensis;|GO:0004725,
DN117189_c1_g1_i4	DOWN	-	-	-	-	-	-
DN114614_c0_g5_i1	DOWN	[GO:0005886:plasma membrane]	-	-	XP_010684438.1:PREDICTED: uncharacterized protein LOC104898995 [Beta vulgaris subsp. vulgaris] >KMT19337.1 hypothetical protein BVRB_1g013400 [Beta vulgaris subsp. vulgaris]	-	A0A0J8D0M4|A0A0J8D0M4_BETVU:Uncharacterized protein;OS=Beta vulgaris subsp. vulgaris.;|GO:0005886,
DN114924_c2_g1_i2	DOWN	-	-	XP_015865732.1:Ziziphus jujuba|FAR1|FAR1 family protein	XP_021864234.1:pimeloyl[acylcarrier protein] methyl ester esteraselike isoform	-	M5VI00|M5VI00_PRUPE:Uncharacterized protein;OS=Prunus

geneid	trend	GO	Pathway	TF	NR	NT	Uniprot
					X1 [Spinacia oleracea]		persica;
DN122740_c0_g1_i2	DOWN	-	-	-	XP_021741568.1:probable strigolactone esterase DAD2 [Chenopodium quinoa]	-	A0A0K9RD14|A0A0K9RD14_SPIOL:Uncharacterized protein;OS=Spinacia oleracea;
DN109981_c0_g1_i1	DOWN	-	-	-	XP_021851302.1:uncharacterized protein LOC110790847 [Spinacia oleracea] >KNA13412.1 hypothetical protein SOVF_117320 [Spinacia oleracea]	gi|332189094|gb|CP002684.1|:Arabidopsis thaliana chromosome 1, complete sequence	A0A0K9R3M0|A0A0K9R3M0_SPIOL: Uncharacterized protein;OS=Spinacia oleracea;

geneid	trend	GO	Pathway	TF	NR	NT	Uniprot
DN126097_c4_g1_i17	DOWN	[GO:0005622:intracellular][GO:0004435:phosphatidylinositol phospholipase C activity][GO:0004871:obsolete signal transducer activity][GO:0035556:intracellular signal transduction][GO:0016042:lipid catabolic process]	[ko00562:Inositol phosphate metabolism][ko04020:Calcium signaling pathway][ko04070:Phosphatidylinositol signaling system][ko04919:Thyroid hormone signaling pathway][ko04933: AGE-RAGE signaling pathway in diabetic complications]	RrC2946_p1:Raphanus raphanistrum|C3H|C3H family protein	XP_010689668.1:PREDICTED: phosphoinositide phospholipase C 6 [Beta vulgaris subsp. vulgaris]	gi|225321262|dbj|AK325473.1|:Solanum lycopersicum cDNA, clone: LEFL1097AC01, HTC in leaf	A0A0K9QJD6|A0A0K9QJD6_SPIOL:Phosphoinositide phospholipase C;OS=Spinacia oleracea;|GO:0005622,|GO:0004435,GO:0004871,|GO:0035556,GO:0016042,
DN125080_c3_g7_i1	DOWN	-	-	-	-	-	-
DN118488_c0_g3_i5	DOWN	[GO:0016021:integral component of membrane][GO:0009055:electron transfer activity]	-	-	XP_021727313.1:uclacyanin2like [Chenopodium quinoa]	-	A0A151RB51|A0A151RB51_CAJCA:Uclacyanin-2;OS=Cajanus cajan;|GO:0016021,|GO:0009055,

geneid	trend	GO	Pathway	TF	NR	NT	Uniprot
DN119267_c2_g1_i1	DOWN	[GO:0016788:hydrolase activity, acting on ester bonds]	-	KN538722.1_FGP037:Oryza longistaminata|HB-PHD|HB-PHD family protein	XP_010673692.1:PREDICTED: GDSL esterase/lipase At5g08460 [Beta vulgaris subsp. vulgaris] >KMT20041.1 hypothetical protein BVRB_1g000600 [Beta vulgaris subsp. vulgaris]	-	A0A0J8FWL2|A0A0J8FWL2_BETVU: Uncharacterized protein;OS=Beta vulgaris subsp. vulgaris.;|GO:0016788,
DN122866_c0_g2_i4	DOWN	[GO:0009507:chloroplast][GO:0005886:plasma membrane][GO:0005774:vacuolar membrane]	-	MDP0000730019:Malus domestica|SBP|SBP family protein	XP_010674627.1:PREDICTED: uncharacterized protein LOC104890745 [Beta vulgaris subsp. vulgaris] >XP_019104530.1 PREDICTED: uncharacterized protein LOC104890745 [Beta vulgaris subsp. vulgaris]	gi|502125214|ref|XM_004498789.1|:PREDICTED: Cicer arietinum uncharacterized LOC101491352 (LOC101491352), mRNA	A0A0J8CJB8|A0A0J8CJB8_BETVU:Uncharacterized protein;OS=Beta vulgaris subsp. vulgaris.;|GO:0009507,GO:0005886,GO:0005774,

geneid	trend	GO	Pathway	TF	NR	NT	Uniprot
					>KMT13642.1 hypothetical protein BVRB_4g080280 [Beta vulgaris subsp. vulgaris]		
DN121547_c1_g1_i3	DOWN	-	-	-	XP_010688094.1:PREDICTED: EG45like domain containing protein isoform X2 [Beta vulgaris subsp. vulgaris] >KMT02958.1 hypothetical protein BVRB_8g195280 [Beta vulgaris subsp. vulgaris]	-	A0A0J8BST7|A0A0J8BST7_BETVU:Uncharacterized protein;OS=Beta vulgaris subsp. vulgaris.;

geneid	trend	GO	Pathway	TF	NR	NT	Uniprot
DN127133_c0_g1_i7	DOWN	[GO:0016021:integral component of membrane][GO:0005524:ATP binding][GO:0000287:magnesium ion binding][GO:0004012:phospholipid-translocating ATPase activity]	-	MDP0000754989:Malus domestica|WRKY|WRKY family protein	XP_021773395.1:phospholipidtransporting ATPase 1like [Chenopodium quinoa]	gi|470141426|ref|XM_004306386.1|:PREDICTED: Fragaria vesca subsp. vesca phospholipid-transporting ATPase 1-like (LOC101314786), mRNA	A0A0K9QML1|A0A0K9QML1_SPIOL:Phospholipid-transporting ATPase;OS=Spinacia oleracea;|GO:0016021,|GO:0005524,GO:0000287,GO:0004012,

geneid	trend	GO	Pathway	TF	NR	NT	Uniprot
DN119247_c0_g1_i5	DOWN	[GO:0005524:ATP binding][GO:0004672:protein kinase activity]	-	mrna09125.1-v1.0-hybrid:Fragaria vesca|bHLH|bHLH family protein	XP_010689101.1:PREDICTED: lysM domain receptorlike kinase 3 isoform X2 [Beta vulgaris subsp. vulgaris] >XP_010689102.1 PREDICTED: lysM domain receptorlike kinase 3 isoform X2 [Beta vulgaris subsp. vulgaris] >XP_010689103.1 PREDICTED: lysM domain receptorlike kinase 3 isoform X2 [Beta vulgaris subsp. vulgaris] >XP_019107332.1 PREDICTED: lysM domain receptorlike kinase 3 isoform X2 [Beta vulgaris subsp. vulgaris] >KMT02310.1	gi|521507903|gb|GAHZ01023017.1|:TSA: Salicornia europaea Unigene24748_SeCKS transcribed RNA sequence	A0A0J8BLN0|A0A0J8BLN0_BETVU:Uncharacterized protein;OS=Beta vulgaris subsp. vulgaris.;|GO:0005524,GO:0004672,

geneid	trend	GO	Pathway	TF	NR	NT	Uniprot
					hypothetical protein BVRB_9g205340 [Beta vulgaris subsp. vulgaris]		

geneid	trend	GO	Pathway	TF	NR	NT	Uniprot
DN111770_c0_g1_i1	DOWN	[GO:0005634:nucleus][GO:0003677:DNA binding][GO:0003700:DNA-binding transcription factor activity][GO:0006351:transcription, DNA-templated]	-	Bv8_182220_noqg.t1:Beta vulgaris|ERF|ERF family protein	XP_021726573.1:pathogenesisrelated genes transcriptional activator PTI5like [Chenopodium quinoa]	gi|521743205|gb|GAIA01004531.1|:TSA: Salicornia europaea Unigene4815_Se200S transcribed RNA sequence	A0A0J8BX42|A0A0J8BX42_BETVU:Uncharacterized protein;OS=Beta vulgaris subsp. vulgaris.;|GO:0005634,|GO:0003677,GO:0003700,|GO:0006351,
DN123525_c1_g2_i2	DOWN	[GO:0009269:response to desiccation]	-	-	XP_014505352.1:PREDICTED: uncharacterized protein LOC106765293 [Vigna radiata var. radiata]	gi|521742172|gb|GAIA01005084.1|:TSA: Salicornia europaea Unigene5371_Se200S transcribed RNA sequence	B8XXQ9|B8XXQ9_SESPO:Salt tolerance protein I;OS=Sesuvium portulacastrum;|GO:0009269,
DN128634_c3_g4_i1	DOWN	[GO:0016021:integral component of membrane][GO:0005524:ATP binding][GO:0016887:ATPase activity][GO:0006810:transport]	-	Rsa1.0_03996.1_g00001.1:Raphanus sativus|MYB_related|MYB_related family protein	XP_021749875.1:ABC transporter G family member 35like [Chenopodium quinoa]	gi|478165088|gb|GAHK01011515.1|:TSA: Jatropha curcas CL4734.Contig1_JC-CK_1A transcribed RNA sequence	A0A0J8B5I1|A0A0J8B5I1_BETVU:Uncharacterized protein;OS=Beta vulgaris subsp. vulgaris.;|GO:0016021,|GO:0005524,GO:0016887,|GO:0006810,

geneid	trend	GO	Pathway	TF	NR	NT	Uniprot
DN123940_c2_g2_i4	DOWN	[GO:0016021:integral component of membrane]	-	-	XP_021725515.1:lipid transferlike protein VAS [Chenopodium quinoa]	-	A0A0K9R849|A0A0K9R849_SPIOL:Uncharacterized protein;OS=Spinacia oleracea;|GO:0016021,
DN119556_c0_g1_i3	DOWN	[GO:0005634:nucleus][GO:0003677:DNA binding][GO:0003700:DNA-binding transcription factor activity][GO:0006351:transcription, DNA-templated]	-	Niben101Scf06378g04004.1:Nicotiana benthamiana|ERF|ERF family protein	XP_021732554.1:dehydrationresponsive elementbinding protein 2Clike isoform X1 [Chenopodium quinoa]	gi|255559548|ref|XM_002520748.1|:Ricinus communis Dehydration-responsive element-binding protein 2C, putative, mRNA	A0A0K1LXK2|A0A0K1LXK2_9CARY:Dehydration responsive element binding transcription factor 2A;OS=Haloxylon ammodendron.;|GO:0005634,|GO:0003677,GO:0003700,|GO:0006351,
DN108568_c0_g1_i1	DOWN	[GO:0005737:cytoplasm][GO:0045548:phenylalanine ammonia-lyase activity][GO:0009800:cinnamic acid biosynthetic process][GO:0006559:L-phenylalanine catabolic process]	[ko00360:Phenylalanine metabolism][ko00940:Phenylpropanoid biosynthesis]	-	XP_021730766.1:phenylalanine ammonialyaselike [Chenopodium quinoa]	gi|18072846|emb|AJ276132.1|:Beta vulgaris partial mRNA for phenylalanine ammonia-lyase (pal1 gene)	A0A0K9R3X8|A0A0K9R3X8_SPIOL:Phenylalanine ammonia-lyase;OS=Spinacia oleracea;|GO:0005737,|GO:0045548,|GO:0009800,GO:0006

geneid	trend	GO	Pathway	TF	NR	NT	Uniprot
							559,
DN120170_c2_g1_i4	DOWN	[GO:0046872:metal ion binding][GO:0016702:oxidoreductase activity, acting on single donors with incorporation of molecular oxygen, incorporation of two atoms of oxygen][GO:0006633:fatty acid biosynthetic process][GO:0031408:oxylipin biosynthetic process]	[ko00591:Linoleic acid metabolism][ko00592:alpha-Linolenic acid metabolism]	Aan016747:Artemisia annua|ERF|ERF family protein	XP_010673986.1:PREDICTED: linoleate 13Slipoxygenase 21, chloroplasticlike, partial [Beta vulgaris subsp. vulgaris]	gi|388522776|gb|BT149656.1|:Lotus japonicus clone JCVI-FLLj-24E12 unknown mRNA	A0A0J8FF20|A0A0J8FF20_BETVU:Lipoxygenase;OS=Beta vulgaris subsp. vulgaris.;|GO:0046872,GO:0016702,|GO:0006633,GO:0031408,
DN126193_c1_g1_i2	DOWN	[GO:0016021:integral component of membrane]	-	-	XP_010685345.1:PREDICTED: forminlike protein 20 [Beta vulgaris subsp. vulgaris] >KMT04859.1 hypothetical protein BVRB_7g170270	-	A0A0J8BXY2|A0A0J8BXY2_BETVU: Uncharacterized protein;OS=Beta vulgaris subsp. vulgaris.;|GO:0016021,

geneid	trend	GO	Pathway	TF	NR	NT	Uniprot
					[Beta vulgaris subsp. vulgaris]		
DN120085_c3_g1_i4	DOWN	[GO:0005524:ATP binding][GO:0004672:protein kinase activity]	-	FANhyb_rscf00000217.1.g00006.1:Fragaria x ananassa|WRKY|WRKY family protein	XP_021866482.1:serine/threonineprotein kinase RIPKlike [Spinacia oleracea]	-	A0A0J8B8C8|A0A0J8B8C8_BETVU:Uncharacterized protein;OS=Beta vulgaris subsp. vulgaris.;|GO:0005524,GO:0004672,

geneid	trend	GO	Pathway	TF	NR	NT	Uniprot
DN127489_c0_g2_i7	DOWN	[GO:0005739:mitochondrion][GO:0070469:respirasome][GO:0009055:electron transfer activity][GO:0020037:heme binding][GO:0046872:metal ion binding][GO:0055114:oxidation-reduction process]	[ko00920:Sulfur metabolism][ko01524:Platinum drug resistance][ko02020:Two-component system][ko04115:p53 signaling pathway][ko04210:Apoptosis][ko04214:Apoptosis - fly][ko04215:Apoptosis - multiple species][ko04932:Non-alcoholic fatty liver disease (NAFLD)][ko05010:Alzheimer's disease][ko05012:Parkinson's disease][ko05014:Amyotrophic lateral sclerosis (ALS)][ko05016:Huntington's disease][ko05134:Legionellosis][ko05145:Toxoplasmosis][ko05152:Tuberculosis][ko05161:Hepatitis B][ko05164:Influenza	-	XP_008795309.1:PREDICTED: cytochrome c [Phoenix dactylifera]	gi|521502666|gb|GAHZ01028254.1|:TSA: Salicornia europaea Unigene31110_SeCKS transcribed RNA sequence	A0A0D6R8Z7|A0A0D6R8Z7_ARACU: Uncharacterized protein;OS=Araucaria cunninghamii;|GO:0005739,GO:0070469,|GO:0009055,GO:0020037,GO:0046872,|GO:0055114,

geneid	trend	GO	Pathway	TF	NR	NT	Uniprot
DN119164_c0_g1_i1	DOWN	[GO:0015031:protein transport][GO:0006979:response to oxidative stress]	[ko04144:Endocytosis]	676753378:Sisymbrium irio|B3|B3 family protein	XP_009770596.1:PREDICTED: uncharacterized protein LOC104221270 isoform X1 [Nicotiana sylvestris] >XP_016476226.1 PREDICTED: uncharacterized protein LOC107797817 [Nicotiana tabacum]	-	K4BXC9|K4BXC9_SOLLC:Uncharacterized protein;OS=Solanum lycopersicum;|GO:0015031,GO:0006979,
DN125074_c5_g2_i10	DOWN	[GO:0042409:caffeoyl-CoA O-methyltransferase activity][GO:0046872:metal ion binding][GO:0009809:lignin biosynthetic process]	[ko00360:Phenylalanine metabolism][ko00940:Phenylpropanoid biosynthesis][ko00941:Flavonoid biosynthesis][ko00945:Stilbenoid, diarylheptanoid and gingerol biosynthesis]	ONIVA06G05580.1:Oryza nivara|ERF|ERF family protein	O65162.1:RecName: Full=CaffeoylCoA Omethyltransferase; AltName: Full=TranscaffeoylCoA 3Omethyltransferase; Short=CCoAMT; Short=CCoAOMT >AAC08395.1 caffeoylCoA	gi|2995933|gb|AF053553.1|AF053553:Mesembryanthemum crystallinum caffeoyl-CoA O-methyltransferase mRNA, complete cds	O65162|CAMT_MESCR:Caffeoyl-CoA O-methyltransferase;OS=Mesembryanthemum crystallinum;|GO:0042409,GO:0046872,|GO:0009809,

geneid	trend	GO	Pathway	TF	NR	NT	Uniprot
					Omethyltransferase [Mesembryanthemum crystallinum]		
DN121507_c0_g2_i6	DOWN	-	-	-	KNA04441.1:hypothetical protein SOVF_199830 [Spinacia oleracea]	-	A0A0K9QD15|A0A0K9QD15_SPIOL:Uncharacterized protein;OS=Spinacia oleracea;
DN126106_c3_g5_i2	DOWN	[GO:0016021:integral component of membrane]	-	CCG025544.1:Populus euphratica|MYB_related|MYB_related family protein	XP_010685175.1:PREDICTED: probable xyloglucan glycosyltransferase 5 [Beta vulgaris subsp. vulgaris] >KMT04694.1 hypothetical protein BVRB_7g168770	gi|225443287|ref|XM_002274363.1|:PREDICTED: Vitis vinifera probable xyloglucan glycosyltransferase 5-like (LOC100265643), mRNA	A0A0J8BXG3|A0A0J8BXG3_BETVU: Uncharacterized protein;OS=Beta vulgaris subsp. vulgaris.;|GO:0016021,

geneid	trend	GO	Pathway	TF	NR	NT	Uniprot
					[Beta vulgaris subsp. vulgaris]		
DN122931_c1_g1_i1	DOWN	[GO:0005634:nucleus][GO:0003677:DNA binding][GO:0009734:auxin-activated signaling pathway][GO:0006355:regulation of transcription, DNA-templated][GO:0006351:transcription, DNA-templated]	[ko04075:Plant hormone signal transduction]	Bv1_002880_qzmp.t1:Beta vulgaris|ARF|ARF family protein	XP_021748876.1:auxin response factor 18like [Chenopodium quinoa] >XP_021748877.1 auxin response factor 18like [Chenopodium quinoa]	gi|521718421|gb|GAIA01011585.1|:TSA: Salicornia europaea Unigene12200_Se200S transcribed RNA sequence	A0A0J8D2B4|A0A0J8D2B4_BETVU:Auxin response factor;OS=Beta vulgaris subsp. vulgaris.;|GO:0005634,|GO:0003677,|GO:0009734,GO:0006355,GO:0006351,
DN125521_c1_g4_i4	DOWN	-	-	-	KNA21659.1:hypothetical protein SOVF_041230 [Spinacia oleracea]	-	A0A0K9RRY8|A0A0K9RRY8_SPIOL: Uncharacterized protein;OS=Spinacia oleracea;
DN127721_c	DOWN	-	-	-	-	-	-

geneid	trend	GO	Pathway	TF	NR	NT	Uniprot
1_g3_i2							
DN112960_c0_g1_i1	DOWN	[GO:0033897:ribonuclease T2 activity][GO:0003723:RNA binding]	-	-	XP_021842969.1:ribonuclease 1like [Spinacia oleracea] >KNA22868.1 hypothetical protein SOVF_029770 [Spinacia oleracea]	gi|521696281|gb|GAIA01019439.1|:TSA: Salicornia europaea Unigene20399_Se200S transcribed RNA sequence	A0A0K9RTT6|A0A0K9RTT6_SPIOL:Uncharacterized protein;OS=Spinacia oleracea;|GO:0033897,GO:0003723,
DN124018_c1_g3_i15	DOWN	-	-	Achn202071:Actinidia chinensis|MYB_related|MYB_related family protein	XP_021733635.1:protein KINESIN LIGHT CHAINRELATED 3like [Chenopodium quinoa]	gi|359491583|ref|XM_002279627.2|:PREDICTED: Vitis vinifera uncharacterized LOC100255253 (LOC100255253), mRNA	A0A0J8CEL7|A0A0J8CEL7_BETVU:Uncharacterized protein;OS=Beta vulgaris subsp. vulgaris.;
DN84241_c0_g1_i1	DOWN	-	-	-	XP_021758666.1:glycinerich cell wall structural proteinlike [Chenopodium quinoa]	-	-

geneid	trend	GO	Pathway	TF	NR	NT	Uniprot
DN126316_c3_g1_i5	DOWN	[GO:0016021:integral component of membrane][GO:0008168:methyltransferase activity]	-	CCG010610.1:Populus euphratica|NAC|NAC family protein	XP_021851572.1:probable methyltransferase PMT21 [Spinacia oleracea] >XP_021851580.1 probable methyltransferase PMT21 [Spinacia oleracea] >KNA24870.1 hypothetical protein SOVF_011830 [Spinacia oleracea]	gi|460400803|ref|XM_004245874.1|:PREDICTED: Solanum lycopersicum probable methyltransferase PMT21-like, transcript variant 2 (LOC101251208), mRNA	A0A0K9RZD8|A0A0K9RZD8_SPIOL: Uncharacterized protein;OS=Spinacia oleracea;|GO:0016021,|GO:0008168,
DN112897_c0_g2_i1	DOWN	[GO:0005576:extracellular region][GO:0030145:manganese ion binding][GO:0045735:nutrient reservoir activity]	-	678365117:Utricularia gibba|bZIP|bZIP family protein	AFK48662.1:unknown [Lotus japonicus]	-	I3T820|I3T820_LOTJA:Uncharacterized protein;OS=Lotus japonicus;|GO:0005576,|GO:0030145,GO:0045735,

geneid	trend	GO	Pathway	TF	NR	NT	Uniprot
DN113223_c0_g1_i1	DOWN	[GO:0016021:integral component of membrane][GO:0005524:ATP binding][GO:0004674:protein serine/threonine kinase activity][GO:0019199:transmembrane receptor protein kinase activity]	[ko04016:MAPK signaling pathway - plant][ko04075:Plant hormone signal transduction][ko04626:Plant-pathogen interaction]	KZV24601.1:Dorcoceras hygrometricum|Trihelix|Trihelix family protein	OMO68155.1:hypothetical protein CCACVL1_20059 [Corchorus capsularis]	-	W9RV20|W9RV20_9ROSA:BRASSINOSTEROID INSENSITIVE 1-associated receptor kinase 1;OS=Morus notabilis.;|GO:0016021,|GO:0005524,GO:0004674,GO:0019199,
DN118712_c0_g2_i1	DOWN	[GO:0047776:citramalate lyase activity][GO:0046872:metal ion binding][GO:0030603:oxaloacetase activity][GO:0016740:transferase activity][GO:0008152:metabolic process]	-	-	XP_021760942.1:petal death proteinlike [Chenopodium quinoa]	-	Q05957|PDP_DIACA:Petal death protein;OS=Dianthus caryophyllus;|GO:0047776,GO:0046872,GO:0030603,GO:0016740,|GO:0008152,
DN124795_c1_g1_i2	DOWN	[GO:0005789:endoplasmic reticulum membrane][GO:0016021:integral component of membrane]	[ko05010:Alzheimer's disease]	-	XP_010687720.1:PREDICTED: reticulonlike protein B21 isoform X1 [Beta vulgaris subsp. vulgaris]	gi|521483495|gb|GAHZ01047391.1|:TSA: Salicornia europaea Unigene55831_SeCKS transcribed RNA sequence	A0A0J8BQV2|A0A0J8BQV2_BETVU: Reticulon-like protein;OS=Beta vulgaris subsp. vulgaris.;|GO:0005789,GO:0016021,

geneid	trend	GO	Pathway	TF	NR	NT	Uniprot
DN121542_c2_g1_i4	DOWN	[GO:0004842:ubiquitin-protein transferase activity]	-	Do007746.1:Dichanthelium oligosanthes|bHLH|bHLH family protein	XP_021733644.1:U box domaincontaining protein 19like [Chenopodium quinoa]	-	A0A0J8CEZ7|A0A0J8CEZ7_BETVU:Uncharacterized protein;OS=Beta vulgaris subsp. vulgaris.;|GO:0004842,
DN121733_c2_g1_i5	DOWN	[GO:0005524:ATP binding][GO:0004672:protein kinase activity]	-	FANhyb_rscf00000217.1.g00006.1:Fragaria x ananassa|WRKY|WRKY family protein	XP_021864011.1:probable serine/threonineprotein kinase PBL12 [Spinacia oleracea] >KNA26125.1 hypothetical protein SOVF_000420 [Spinacia oleracea]	gi|356526887|ref|XM_003532000.1|:PREDICTED: Glycine max probable receptor-like protein kinase At5g15080-like (LOC100787127), mRNA	A0A0K9S302|A0A0K9S302_SPIOL:Uncharacterized protein;OS=Spinacia oleracea;|GO:0005524,GO:0004672,
DN126983_c6_g13_i2	DOWN	[GO:0008171:O-methyltransferase activity]	[ko00940:Phenylpropanoid biosynthesis]	-	XP_010671332.1:PREDICTED: caffeic acid 3Omethyltransferase [Beta vulgaris subsp. vulgaris] >KMT16477.1 hypothetical protein BVRB_3g050950	-	A0A0J8CS99|A0A0J8CS99_BETVU:Uncharacterized protein;OS=Beta vulgaris subsp. vulgaris.;|GO:0008171,

geneid	trend	GO	Pathway	TF	NR	NT	Uniprot
					[Beta vulgaris subsp. vulgaris]		
DN112388_c5_g7_i1	DOWN	-	-	-	-	-	-
DN126994_c2_g33_i1	DOWN	-	-	-	-	-	-
DN127772_c2_g6_i9	DOWN	[GO:0016021:integral component of membrane][GO:0005886:plasma membrane][GO:0016760:cellulose synthase (UDP-forming) activity][GO:0046872:metal ion binding][GO:0071555:cell wall organization][GO:0030244:cellulose biosynthetic process]	-	FANhyb_rscf00001203.1.g00002.1:Fragaria x ananassa|LBD|LBD family protein	XP_021911761.1:LOW QUALITY PROTEIN: cellulose synthase A catalytic subunit 3 [UDPforming]like [Carica papaya]	gi|225450118|ref|XM_002278961.1|:PREDICTED: Vitis vinifera cellulose synthase A catalytic subunit 3 [UDP-forming]-like (LOC100256811), mRNA	A0A061DWE0|A0A061DWE0_THECC: Cellulose synthase;OS=Theobroma cacao;|GO:0016021,GO:0005886,|GO:0016760,GO:0046872,|GO:0071555,GO:0030244,

geneid	trend	GO	Pathway	TF	NR	NT	Uniprot
DN115987_c0_g1_i2	DOWN	-	-	-	XP_021766118.1:uncharacterized protein LOC110730603 [Chenopodium quinoa] >XP_021766119.1 uncharacterized protein LOC110730603 [Chenopodium quinoa]	-	A0A0K9RLT2|A0A0K9RLT2_SPIOL:Uncharacterized protein;OS=Spinacia oleracea;
DN124762_c10_g1_i2	DOWN	-	-	-	BAT78682.1:hypothetical protein VIGAN_02139400 [Vigna angularis var. angularis]	-	A0A0S3RDS0|A0A0S3RDS0_PHAAN:Uncharacterized protein;OS=Vigna angularis var. angularis.;
DN123110_c0_g2_i2	DOWN	[GO:0004866:endopeptidase inhibitor activity]	-	-	XP_010683193.1:PREDICTED: kunitz trypsin inhibitor 2 [Beta vulgaris subsp. vulgaris] >KMT07072.1 hypothetical protein BVRB_6g153990	-	A0A0J8C0T9|A0A0J8C0T9_BETVU:Uncharacterized protein;OS=Beta vulgaris subsp. vulgaris.;|GO:0004866,

geneid	trend	GO	Pathway	TF	NR	NT	Uniprot
					[Beta vulgaris subsp. vulgaris]		
DN108568_c0_g2_i1	DOWN	[GO:0005737:cytoplasm][GO:0045548:phenylalanine ammonia-lyase activity][GO:0009800:cinnamic acid biosynthetic process][GO:0006559:L-phenylalanine catabolic process]	[ko00360:Phenylalanine metabolism][ko00940:Phenylpropanoid biosynthesis]	-	XP_010684079.1:PREDICTED: phenylalanine ammonialyase [Beta vulgaris subsp. vulgaris] >CAH17686.1 phenylalanine ammonia lyase [Beta vulgaris] >KMT06263.1 hypothetical protein BVRB_7g161880 [Beta vulgaris subsp. vulgaris]	gi|460416375|ref|XM_004253470.1|:PREDICTED: Solanum lycopersicum phenylalanine ammonia-lyase-like (LOC101253989), partial mRNA	Q5QRU8|Q5QRU8_BETVU:Phenylalanine ammonia-lyase;OS=Beta vulgaris;|GO:0005737,|GO:0045548,|GO:0009800,GO:0006559,

geneid	trend	GO	Pathway	TF	NR	NT	Uniprot
DN115174_c5_g1_i2	DOWN	[GO:0016021:integral component of membrane][GO:0016757:transferase activity, transferring glycosyl groups]	-	-	XP_010666578.1:PREDICTED: xyloglucan 6xylosyltransferase 2 [Beta vulgaris subsp. vulgaris] >KMS96012.1 hypothetical protein BVRB_003040 [Beta vulgaris subsp. vulgaris]	gi|292770812|dbj|AK337587.1|:Lotus japonicus cDNA, clone: LjFL1-069-CC03, HTC	A0A0J8B8A6|A0A0J8B8A6_BETVU:Uncharacterized protein;OS=Beta vulgaris subsp. vulgaris.;|GO:0016021,|GO:0016757,
DN121142_c2_g2_i10	DOWN	[GO:0016021:integral component of membrane][GO:0006810:transport]	[ko04146:Peroxisome]	-	XP_021837843.1:peroxisomal nicotinamide adenine dinucleotide carrier [Spinacia oleracea] >KNA13923.1 hypothetical protein SOVF_112280 [Spinacia oleracea]	gi|359486714|ref|XM_002279452.2|:PREDICTED: Vitis vinifera mitochondrial substrate carrier family protein Q-like (LOC100261501), mRNA	A0A0K9R332|A0A0K9R332_SPIOL:Uncharacterized protein;OS=Spinacia oleracea;|GO:0016021,|GO:0006810,

geneid	trend	GO	Pathway	TF	NR	NT	Uniprot
DN124134_c3_g8_i1	DOWN	[GO:0016491:oxidoreductase activity][GO:0008270:zinc ion binding]	[ko00010:Glycolysis / Gluconeogenesis][ko000071:Fatty acid degradation][ko00350:Tyrosine metabolism][ko00592:alpha-Linolenic acid metabolism]	-	AAP96921.1:alcohol dehydrogenase [Dianthus caryophyllus]	gi|33149682|gb|AY263389.1|:Dianthus caryophyllus alcohol dehydrogenase mRNA, complete cds	Q5QJY8|Q5QJY8_DIACA:Alcohol dehydrogenase;OS=Dianthus caryophyllus;|GO:0016491,GO:0008270,
DN129001_c3_g5_i1	DOWN	[GO:0005634:nucleus][GO:0009734:auxin-activated signaling pathway][GO:0006355:regulation of transcription, DNA-templated][GO:0006351:transcription, DNA-templated]	[ko04075:Plant hormone signal transduction]	FANhyb_rscf00000379.1.g00008.1:Fragaria x ananassa|bZIP|bZIP family protein	ACL81167.1:auxinresponsive protein IAA1, partial [Mirabilis jalapa]	gi|220702736|gb|FJ536233.1|:Mirabilis jalapa clone R493 auxin-responsive protein IAA1 mRNA, partial cds	B8YIB9|B8YIB9_MIRJA:Auxin-responsive protein;OS=Mirabilis jalapa;|GO:0005634,|GO:0009734,GO:0006355,GO:0006351,
DN117802_c5_g3_i3	DOWN	[GO:0016021:integral component of membrane][GO:0005739:mitochondrion]	-	-	KUM48329.1:hypothetical protein ABT39_MTgene5329 (mitochondrion) [Picea glauca]	-	A0A101LZN5|A0A101LZN5_PICGL:Uncharacterized protein;OS=Picea glauca;|GO:0016021,GO:0005739,

geneid	trend	GO	Pathway	TF	NR	NT	Uniprot
DN121142_c2_g4_i1	DOWN	[GO:0016021:integral component of membrane][GO:0006810:transport]	[ko04146:Peroxisome]	MDP0000322264:Malus domestica|M-type_MADS|M-type_MADS family protein	XP_021732416.1:peroxisomal nicotinamide adenine dinucleotide carrierlike [Chenopodium quinoa]	gi|521493393|gb|GAHZ01037527.1|:TSA: Salicornia europaea Unigene42307_SeCKS transcribed RNA sequence	B9SLI5|B9SLI5_RICCO:Peroxisomal membrane protein pmp34, putative;OS=Ricinus communis;|GO:0016021,|GO:0006810,
DN124298_c1_g2_i3	DOWN	[GO:0016021:integral component of membrane][GO:0005524:ATP binding][GO:0004672:protein kinase activity]	-	KZV29721.1:Dorcoceras hygrometricum|STAT|STAT family protein	XP_021739110.1:receptorlike protein kinase HSL1 [Chenopodium quinoa]	-	A0A0J8D1R2|A0A0J8D1R2_BETVU:Uncharacterized protein;OS=Beta vulgaris subsp. vulgaris.;|GO:0016021,|GO:0005524,GO:0004672,
DN116680_c0_g3_i7	DOWN	[GO:0020037:heme binding][GO:0005506:iron ion binding][GO:0004497:monooxygenase activity][GO:0016705:oxidoreductase activity, acting on paired donors, with incorporation or reduction of molecular oxygen]	-	676766070:Sisymbrium irio|bHLH|bHLH family protein	ALT04747.1:hypothetical protein [Beta vulgaris]	-	A0A0U3BG65|A0A0U3BG65_BETVU:Uncharacterized protein;OS=Beta vulgaris;|GO:0020037,GO:0005506,GO:0004497,GO:0016705,

geneid	trend	GO	Pathway	TF	NR	NT	Uniprot
DN124267_c0_g1_i1	DOWN	-	-	-	-	-	-
DN125890_c1_g1_i1	DOWN	[GO:0016021:integral component of membrane][GO:0005506:iron ion binding][GO:0016491:oxidoreductase activity][GO:0006633:fatty acid biosynthetic process]	-	-	XP_021756859.1:protein ECERIFERUM 3like [Chenopodium quinoa]	gi|521452999|gb|GAHZ01067485.1|:TSA: Salicornia europaea Unigene81517_SeCKS transcribed RNA sequence	A0A0J8BQ25|A0A0J8BQ25_BETVU:Uncharacterized protein;OS=Beta vulgaris subsp. vulgaris.;|GO:0016021,|GO:0005506,GO:0016491,|GO:0006633,
DN116679_c2_g2_i5	DOWN	[GO:0004805:trehalose-phosphatase activity][GO:0005992:trehalose biosynthetic process]	[ko00500:Starch and sucrose metabolism]	-	KNA17803.1:hypothetical protein SOVF_076230 [Spinacia oleracea]	gi|521482710|gb|GAHZ01048176.1|:TSA: Salicornia europaea Unigene56706_SeCKS transcribed RNA sequence	A0A0K9REB3|A0A0K9REB3_SPIOL:Trehalose 6-phosphate phosphatase;OS=Spinacia oleracea;|GO:0004805,|GO:0005992,

geneid	trend	GO	Pathway	TF	NR	NT	Uniprot
DN122849_c2_g3_i10	DOWN	[GO:0005829:cytosol][GO:0016020:membrane][GO:0009505:plant-type cell wall][GO:0016157:sucrose synthase activity][GO:0010431:seed maturation][GO:0005982:starch metabolic process][GO:0005985:sucrose metabolic process]	[ko00500:Starch and sucrose metabolism]	mrna11429.1-v1.0-hybrid:Fragaria vesca|HB-other|HB-other family protein	XP_021763213.1:sucrose synthase [Chenopodium quinoa]	gi|478158948|gb|GAHK01017655.1|:TSA: Jatropha curcas Unigene5109_JC-CK_1A transcribed RNA sequence	A0A0J8ESB8|A0A0J8ESB8_BETVU:Sucrose synthase;OS=Beta vulgaris subsp. vulgaris.;|GO:0005829,GO:0016020,GO:0009505,|GO:0016157,|GO:0010431,GO:0005982,GO:0005985,
DN112472_c2_g1_i2	DOWN	[GO:0003824:catalytic activity][GO:0050662:coenzyme binding]	[ko00940:Phenylpropanoid biosynthesis]	TRIUR3_04273-P1: Triticum urartu|ERF|ERF family protein	XP_010691595.1:PREDICTED: cinnamoylCoA reductase 1 [Beta vulgaris subsp. vulgaris] >KMT01051.1 hypothetical protein BVRB_9g223090 [Beta vulgaris subsp. vulgaris]	gi|316939059|gb|HQ229954.1|:Hevea brasiliensis cinnamoyl-CoA reductase (CCR) mRNA, complete cds	A0A0J8EDK7|A0A0J8EDK7_BETVU: Uncharacterized protein;OS=Beta vulgaris subsp. vulgaris.;|GO:0003824,GO:0050662,

geneid	trend	GO	Pathway	TF	NR	NT	Uniprot
DN124740_c2_g1_i4	DOWN	-	-	-	KNA17513.1:hypothetical protein SOVF_079260 isoform A [Spinacia oleracea]	gi|521544140|gb|GAHZ01001359.1|:TSA: Salicornia europaea Unigene1385_SeCKS transcribed RNA sequence	A0A0K9RDJ8|A0A0K9RDJ8_SPIOL:Uncharacterized protein;OS=Spinacia oleracea;
DN79060_c0_g1_i1	DOWN	-	-	-	-	-	-
DN111512_c0_g1_i3	DOWN	-	-	-	XP_010692836.1:PREDICTED: uncharacterized protein LOC104905882 [Beta vulgaris subsp. vulgaris] >KMS99561.1 hypothetical protein BVRB_1g022880 [Beta vulgaris subsp. vulgaris]	-	A0A0J8BF54|A0A0J8BF54_BETVU:Uncharacterized protein;OS=Beta vulgaris subsp. vulgaris.;

geneid	trend	GO	Pathway	TF	NR	NT	Uniprot
DN117349_c0_g4_i1	DOWN	[GO:0005524:ATP binding][GO:0004356:glutamate-ammonia ligase activity][GO:0006542:glutamine biosynthetic process]	[ko00220:Arginine biosynthesis][ko00250:Alanine, aspartate and glutamate metabolism][ko00630:Glyoxylate and dicarboxylate metabolism][ko00910:Nitrogen metabolism][ko02020:Two-component system][ko04217:Necroptosis][ko04724:Glutamatergic synapse][ko04727:GABAergic synapse]	-	XP_008461372.1:PREDICTED: glutamine synthetase nodule isozymelike [Cucumis melo]	gi|521569822|gb|GAIA01073903.1|:TSA: Salicornia europaea Unigene89771_Se200S transcribed RNA sequence	A0A0B0PTQ0|A0A0B0PTQ0_GOSAR: Glutamine synthetase;OS=Gossypium arboreum;|GO:0005524,GO:0004356,|GO:0006542,

geneid	trend	GO	Pathway	TF	NR	NT	Uniprot
DN118153_c0_g2_i1	DOWN	[GO:0016021:integral component of membrane][GO:0005886:plasma membrane][GO:0016760:cellulose synthase (UDP-forming) activity][GO:0046872:metal ion binding][GO:0071555:cell wall organization][GO:0030244:cellulose biosynthetic process]	-	FANhyb_rscf00001203.1.g00002.1:Fragaria x ananassa|LBD|LBD family protein	XP_021758182.1:cellulose synthase A catalytic subunit 1 [UDPforming]like [Chenopodium quinoa]	gi|242205329|gb|GQ200734.1|:Gossypium hirsutum cultivar Xu-142 cellulose synthase catalytic subunit (cesA7) mRNA, partial cds	A0A0K9QW16|A0A0K9QW16_SPIOL: Cellulose synthase;OS=Spinacia oleracea;|GO:0016021,GO:0005886,|GO:0016760,GO:0046872,|GO:0071555,GO:0030244,
DN122803_c0_g3_i1	DOWN	[GO:0030598:rRNA N-glycosylase activity][GO:0006952:defense response][GO:0017148:negative regulation of translation]	-	-	AAL35962.1:bouganin [Bougainvillea spectabilis]	-	Q8W4U4|Q8W4U4_9CARY:rRNA N-glycosidase;OS= Bougainvillea spectabilis.;|GO:0030598,|GO:0006952, GO:0017148,
DN119164_c0_g1_i3	DOWN	[GO:0015031:protein transport][GO:0006979:response to oxidative stress]	[ko04144:Endocytosis]	676753378:Sisymbrium irio|B3|B3 family protein	XP_021723839.1:uncharacterized protein LOC110691235 isoform X1 [Chenopodium quinoa]	-	A0A0J8C8F8|A0A0J8C8F8_BETVU:Uncharacterized protein;OS=Beta vulgaris subsp. vulgaris.;|GO:0015031,GO:0006979,

geneid	trend	GO	Pathway	TF	NR	NT	Uniprot
DN117800_c4_g4_i8	DOWN	[GO:0016021:integral component of membrane]	[ko00520:Amino sugar and nucleotide sugar metabolism]	MDP0000247737:Malus domestica|FAR1|FAR1 family protein	XP_021856720.1:UDPglucuronic acid decarboxylase 2 [Spinacia oleracea] >KNA16834.1 hypothetical protein SOVF_084960 [Spinacia oleracea]	gi|356503899|ref|XM_003520690.1|:PREDICTED: Glycine max UDP-glucuronic acid decarboxylase 1-like (LOC100782935), mRNA	A0A0K9RCV8|A0A0K9RCV8_SPIOL: Uncharacterized protein;OS=Spinacia oleracea;|GO:0016021,
DN112378_c0_g1_i1	DOWN	[GO:0005524:ATP binding][GO:0004672:protein kinase activity]	-	GSVIVT01019630001:Vitis vinifera|MIKC_MADS|MIKC_MADS family protein	XP_010675874.2:PREDICTED: probable serine/threonineprotein kinase WNK11 [Beta vulgaris subsp. vulgaris]	gi|449530884|ref|XM_004172374.1|:PREDICTED: Cucumis sativus probable serine/threonine-protein kinase WNK11-like (LOC101221172), mRNA	A0A0J8FAK5|A0A0J8FAK5_BETVU:Uncharacterized protein;OS=Beta vulgaris subsp. vulgaris.;|GO:0005524,GO:0004672,
DN123981_c0_g3_i6	DOWN	[GO:0016758:transferase activity, transferring hexosyl groups][GO:0008152:metabolic process]	-	-	XP_021735839.1:anthocyanidin 3Oglucoside 2"Oglucosyltransferaselike [Chenopodium quinoa]	-	A0A0K9QDA4|A0A0K9QDA4_SPIOL: Glycosyltransferase; OS=Spinacia oleracea;|GO:0016758,|GO:0008152,

geneid	trend	GO	Pathway	TF	NR	NT	Uniprot
DN127662_c3_g3_i2	DOWN	[GO:0043565:sequence-specific DNA binding][GO:0003700:DNA-binding transcription factor activity]	-	Bv_002510_sgmx.t1:Beta vulgaris|WRKY|WRKY family protein	XP_010666507.1:PREDICTED: probable WRKY transcription factor 71 [Beta vulgaris subsp. vulgaris] >KMS96051.1 hypothetical protein BVRB_002510 [Beta vulgaris subsp. vulgaris]	gi|460374432|ref|XM_004232967.1|:PREDICTED: Solanum lycopersicum probable WRKY transcription factor 28-like (LOC101264783), mRNA	A0A0J8B518|A0A0J8B518_BETVU:Uncharacterized protein;OS=Beta vulgaris subsp. vulgaris.;|GO:0043565,GO:0003700,
DN119556_c0_g1_i2	DOWN	[GO:0005634:nucleus][GO:0003677:DNA binding][GO:0003700:DNA-binding transcription factor activity][GO:0006351:transcription, DNA-templated]	-	XP_016461559.1:Nicotiana tabacum|ERF|ERF family protein	XP_019245341.1:PREDICTED: dehydrationresponsive elementbinding protein 2Alike [Nicotiana attenuata] >OIT03028.1 dehydrationresponsive elementbinding protein 2a [Nicotiana attenuata]	gi|255559548|ref|XM_002520748.1|:Ricinus communis Dehydration-responsive element-binding protein 2C, putative, mRNA	I3NN76|I3NN76_SOLLC:DREB2;OS=Solanum lycopersicum;|GO:0005634,|GO:0003677,GO:0003700,|GO:0006351,
DN127959_c2_g1_i1	DOWN	-	-	-	-	-	-

geneid	trend	GO	Pathway	TF	NR	NT	Uniprot
DN119461_c1_g1_i1	DOWN	[GO:0004553:hydrolase activity, hydrolyzing O-glycosyl compounds][GO:0005975:carbohydrate metabolic process]	[ko00460:Cyanoamino acid metabolism][ko00500:Starch and sucrose metabolism][ko00940:Phenylpropanoid biosynthesis]	676737120:Sisymbrium irio|NF-YC|NF-YC family protein	XP_021751155.1:betaglucosidase 12like [Chenopodium quinoa]	gi|359478416|ref|XM_003632070.1|:PREDICTED: Vitis vinifera beta-glucosidase 12-like (LOC100250160), mRNA	A0A0K9S1T7|A0A0K9S1T7_SPIOL:Uncharacterized protein;OS=Spinacia oleracea;|GO:0004553,|GO:0005975,
DN112658_c0_g1_i3	DOWN	[GO:0005576:extracellular region]	[ko04016:MAPK signaling pathway - plant][ko04075:Plant hormone signal transduction][ko04626:Plant-pathogen interaction]	-	XP_021903735.1:pathogenesisrelated protein 1like [Carica papaya] >AMP83210.1 pathogenesisrelated protein 1 [Carica papaya]	-	A0A142CDV7|A0A142CDV7_CARPA: Pathogenesis-related protein 1;OS=Carica papaya;|GO:0005576,
DN125434_c3_g5_i5	DOWN	[GO:0016021:integral component of membrane][GO:0005509:calcium ion binding][GO:0005216:ion channel activity]	-	-	XP_021850725.1:two pore calcium channel protein 1A [Spinacia oleracea] >KNA19477.1 hypothetical protein SOVF_061250 [Spinacia oleracea]	gi|521562954|gb|GAIA01075295.1|:TSA: Salicornia europaea Unigene91279_Se200S transcribed RNA sequence	A0A0K9RKT4|A0A0K9RKT4_SPIOL: Uncharacterized protein;OS=Spinacia oleracea;|GO:0016021,|GO:0005509,GO:0005216,

geneid	trend	GO	Pathway	TF	NR	NT	Uniprot
DN123417_c1_g3_i3	DOWN	[GO:0016758:transferase activity, transferring hexosyl groups][GO:0008152:metabolic process]	-	Tp57577_TGAC_v2_mRNA33215:Trifolium pratense|bHLH|bHLH family protein	XP_019106621.1:PREDICTED: UDPglycosyltransferase 86A2like [Beta vulgaris subsp. vulgaris] >KMT04457.1 hypothetical protein BVRB_8g181290 [Beta vulgaris subsp. vulgaris]	gi|511329053|gb|GAKE01003824.1|:TSA: Thlaspi arvense Contig3897 transcribed RNA sequence	A0A0J8BTK7|A0A0J8BTK7_BETVU:Glycosyltransferase;OS=Beta vulgaris subsp. vulgaris.;|GO:0016758,|GO:0008152,
DN123195_c1_g1_i6	DOWN	[GO:0016021:integral component of membrane][GO:0009506:plasmodesma]	-	-	XP_010687826.1:PREDICTED: uncharacterized protein LOC104901901 [Beta vulgaris subsp. vulgaris] >KMT02704.1 hypothetical protein BVRB_8g192950 [Beta vulgaris subsp. vulgaris]	gi|521448773|gb|GAHZ01071711.1|:TSA: Salicornia europaea Unigene87385_SeCKS transcribed RNA sequence	A0A0J8BRT1|A0A0J8BRT1_BETVU:Uncharacterized protein;OS=Beta vulgaris subsp. vulgaris.;|GO:0016021,GO:0009506,

geneid	trend	GO	Pathway	TF	NR	NT	Uniprot
DN121765_c2_g1_i1	DOWN	[GO:0045735:nutrient reservoir activity]	-	-	XP_010685841.1:PREDICTED: glutelin typeB 5 [Beta vulgaris subsp. vulgaris] >KMT05326.1 hypothetical protein BVRB_7g174610 [Beta vulgaris subsp. vulgaris]	gi|225435089|ref|XM_002284423.1|:PREDICTED: Vitis vinifera glutelin type-A 1-like (LOC100256985), mRNA	A0A0J8EQN3|A0A0J8EQN3_BETVU: Uncharacterized protein;OS=Beta vulgaris subsp. vulgaris.;|GO:0045735,
DN120263_c0_g1_i5	DOWN	[GO:0005774:vacuolar membrane][GO:0004650:polygalacturonase activity][GO:0005975:carbohydrate metabolic process]	-	Itr_sc000031.1_g00012.1:Ipomoea trifida|bHLH|bHLH family protein	XP_010672734.1:PREDICTED: polygalacturonase QRT2 [Beta vulgaris subsp. vulgaris] >KMT15387.1 hypothetical protein BVRB_3g060910 [Beta vulgaris subsp. vulgaris]	gi|521736344|gb|GAIA01008738.1|:TSA: Salicornia europaea Unigene9088_Se200S transcribed RNA sequence	A0A0J8CPD5|A0A0J8CPD5_BETVU:Uncharacterized protein;OS=Beta vulgaris subsp. vulgaris.;|GO:0005774,|GO:0004650,|GO:0005975,
DN111708_c1_g1_i2	DOWN	-	-	-	-	-	-

geneid	trend	GO	Pathway	TF	NR	NT	Uniprot
DN121816_c1_g1_i3	DOWN	[GO:0016021:integral component of membrane][GO:0008270:zinc ion binding]	-	Oropetium_20150105_23793A:Oropetium thomaeum|C3H|C3H family protein	XP_021745986.1:uncharacterized protein LOC110711854 [Chenopodium quinoa]	-	V4RT85|V4RT85_9ROSI:Uncharacterized protein;OS=Citrus clementina.;|GO:0016021,|GO:0008270,
DN118844_c2_g1_i1	DOWN	[GO:0016021:integral component of membrane][GO:0005249:voltage-gated potassium channel activity]	-	-	KNA13563.1:hypothetical protein SOVF_115010 [Spinacia oleracea]	-	A0A0K9R225|A0A0K9R225_SPIOL:Uncharacterized protein;OS=Spinacia oleracea;|GO:0016021,|GO:0005249,
DN124522_c0_g14_i1	DOWN	-	-	-	-	-	-
DN117251_c1_g2_i5	DOWN	[GO:0016620:oxidoreductase activity, acting on the aldehyde or oxo group of donors, NAD or NADP as acceptor]	[ko00940:Phenylpropanoid biosynthesis]	Lus10030143:Linum usitatissimum|NAC|NAC family protein	XP_010688154.1:PREDICTED: aldehyde dehydrogenase family 2 member C4 [Beta vulgaris subsp. vulgaris] >KMT03022.1 hypothetical protein BVRB_8g195850	gi|521734154|gb|GAIA01009835.1|:TSA: Salicornia europaea Unigene10290_Se200S transcribed RNA sequence	A0A0J8BP52|A0A0J8BP52_BETVU:Uncharacterized protein;OS=Beta vulgaris subsp. vulgaris.;|GO:0016620,

geneid	trend	GO	Pathway	TF	NR	NT	Uniprot
					[Beta vulgaris subsp. vulgaris]		
DN117915_c3_g2_i1	DOWN	[GO:0003914:DNA (6-4) photolyase activity][GO:0009411:response to UV]	[ko04710:Circadian rhythm]	KZV28906.1:Dorcoceras hygrometricum|C2H2|C2H2 family protein	XP_010691197.1:PREDICTED: (64)DNA photolyase isoform X1 [Beta vulgaris subsp. vulgaris] >KMT00718.1 hypothetical protein BVRB_9g220030 isoform B [Beta vulgaris subsp. vulgaris]	gi|521454350|gb|GAHZ01066134.1|:TSA: Salicornia europaea Unigene79827_SeCKS transcribed RNA sequence	A0A0J8BKS5|A0A0J8BKS5_BETVU:Uncharacterized protein;OS=Beta vulgaris subsp. vulgaris.;|GO:0003914,|GO:0009411,

geneid	trend	GO	Pathway	TF	NR	NT	Uniprot
DN127142_c3_g1_i1	DOWN	[GO:0004553:hydrolase activity, hydrolyzing O-glycosyl compounds][GO:0005975:carbohydrate metabolic process]	[ko00520:Amino sugar and nucleotide sugar metabolism]	-	XP_021726781.1:acidic endochitinaselike [Chenopodium quinoa]	gi|521541767|gb|GAHZ01002390.1|:TSA: Salicornia europaea Unigene2454_SeCKS transcribed RNA sequence	A0A0J8CR98|A0A0J8CR98_BETVU:Uncharacterized protein;OS=Beta vulgaris subsp. vulgaris.;|GO:0004553,|GO:0005975,
DN127671_c4_g2_i7	DOWN	[GO:0016021:integral component of membrane][GO:0043231:intracellular membrane-bounded organelle][GO:0005524:ATP binding][GO:0042626:ATPase activity, coupled to transmembrane movement of substances][GO:0006869:lipid transport]	-	-	XP_003527064.1:PREDICTED: ABC transporter A family member 2like [Glycine max] >KRH54517.1 hypothetical protein GLYMA_06G191300 [Glycine max]	-	I1KCQ5|I1KCQ5_SOYBN:Uncharacterized protein;OS=Glycine max;|GO:0016021,GO:0043231,|GO:0005524,GO:0042626,|GO:0006869,

geneid	trend	GO	Pathway	TF	NR	NT	Uniprot
DN127998_c1_g1_i1	DOWN	[GO:0005783:endoplasmic reticulum][GO:0018472:1-hydroxy-2-naphthaldehyde dehydrogenase activity][GO:0034525:1-naphthaldehyde dehydrogenase activity][GO:0044104:2,5-dioxovalerate dehydrogenase (NAD+) activity][GO:0018474:2-carboxybenzaldehyde dehydrogenase activity][GO:0034520:2-naphthaldehyde dehydrogenase activity][GO:0004028:3-chloroallyl aldehyde dehydrogenase activity][GO:0034538:3-methylsalicylaldehyde dehydrogenase activity][GO:0034530:4-hydroxymethylsalicyaldehyde dehydrogenase activity][GO:0019115:benzaldehyde dehydrogenase activity][GO:0018473:cis-2-methyl-5-isopropylhexa-2,5-dienal dehydrogenase activity][GO:0034822:citronellal dehydrogenase activity][GO:0080019:fatty-acyl-CoA reductase (alcohol-forming) activity][GO:0034832:geranial dehydrogenase activity][GO:0043796:glyceraldehyde dehydrogenase (NADP) activity][GO:0043878:glyceraldehyde	[ko00073:Cutin, suberine and wax biosynthesis][ko04146:Peroxisome][ko04212:Longevity regulating pathway - worm]	-	XP_021722084.1:alcoholforming fatty acylCoA reductaselike [Chenopodium quinoa]	gi|478157506|gb|GAHK01019097.1|:TSA: Jatropha curcas Unigene6551_JC-CK_1A transcribed RNA sequence	A0A0J8C022|A0A0J8C022_BETVU:Fatty acyl-CoA reductase;OS=Beta vulgaris subsp. vulgaris.;|GO:0005783,|GO:0018472,GO:0034525,GO:0044104,GO:0018474,GO:0034520,GO:0004028,GO:0034538,GO:0034530,GO:0019115,GO:0018473,GO:0034822,GO:0080019,GO:0034832,GO:0043796,GO:0043878,GO:0052814,GO:0043870,GO:0043745,GO:0034602,GO:0034601,GO:0034604,GO:0018475,|GO:0006629,GO:0010025,

geneid	trend	GO	Pathway	TF	NR	NT	Uniprot
DN119092_c5_g1_i1	DOWN	[GO:0016787:hydrolase activity][GO:0008152:metabolic process]	-	ORUFI12G02480.1: Oryza rufipogon|NAC|NAC family protein	XP_021754367.1:probable carboxylesterase 6 [Chenopodium quinoa]	-	A0A0D2U5F2|A0A0D2U5F2_GOSRA: Uncharacterized protein;OS=Gossypium raimondii;|GO:0016787,|GO:0008152,
DN126294_c1_g2_i11	DOWN	[GO:0020037:heme binding][GO:0005506:iron ion binding][GO:0004497:monooxygenase activity][GO:0016705:oxidoreductase activity, acting on paired donors, with incorporation or reduction of molecular oxygen]	[ko00906:Carotenoid biosynthesis]	Gh_A02G0666:Gossypium hirsutum|G2-like|G2-like family protein	XP_010676702.1:PREDICTED: abscisic acid 8'hydroxylase 1like [Beta vulgaris subsp. vulgaris]	gi|367465453|gb|JF316738.1|:Citrus sinensis ABA 8'-hydroxylase (CYP707A1) mRNA, complete cds	A0A0J8CEQ4|A0A0J8CEQ4_BETVU:Uncharacterized protein;OS=Beta vulgaris subsp. vulgaris.;|GO:0020037,GO:0005506,GO:0004497,GO:0016705,
DN119797_c3_g1_i1	DOWN	[GO:0016758:transferase activity, transferring hexosyl groups][GO:0008152:metabolic process]	-	Tp57577_TGAC_v2_mRNA33215:Trifolium pratense|bHLH|bHLH family protein	XP_010679006.1:PREDICTED: UDPglycosyltransferase 74G1 [Beta vulgaris subsp. vulgaris] >KMT10139.1 hypothetical protein BVRB_5g119010	-	A0A0J8CD45|A0A0J8CD45_BETVU:Glycosyltransferase;OS=Beta vulgaris subsp. vulgaris.;|GO:0016758,|GO:0008152,

geneid	trend	GO	Pathway	TF	NR	NT	Uniprot
					[Beta vulgaris subsp. vulgaris]		
DN122627_c0_g1_i1	DOWN	[GO:0016021:integral component of membrane][GO:0008146:sulfotransferase activity]	-	KN538864.1_FGP012:Oryza longistaminata|bZIP|bZIP family protein	XP_021859945.1:uncharacterized protein LOC110799053 [Spinacia oleracea]	gi|521658499|gb|GAIA01038067.1|:TSA: Salicornia europaea Unigene42602_Se200S transcribed RNA sequence	A0A0K9QZL8|A0A0K9QZL8_SPIOL:Sulfotransferase;OS=Spinacia oleracea;|GO:0016021,|GO:0008146,
DN109250_c0_g2_i1	DOWN	-	-	-	-	-	-
DN122116_c4_g1_i13	DOWN	[GO:0004190:aspartic-type endopeptidase activity]	-	mrna24179.1-v1.0-hybrid:Fragaria vesca|NAC|NAC family protein	XP_010680395.1:PREDICTED: aspartyl protease family protein 1 [Beta vulgaris subsp. vulgaris] >KMT09315.1 hypothetical protein BVRB_6g134130	gi|261264968|gb|GU048885.1|:Capsicum annuum clone BAC CaCM414G15, complete sequence	A0A0J8C6D8|A0A0J8C6D8_BETVU:Uncharacterized protein;OS=Beta vulgaris subsp. vulgaris.;|GO:0004190,

geneid	trend	GO	Pathway	TF	NR	NT	Uniprot
					[Beta vulgaris subsp. vulgaris]		
DN112423_c1_g1_i2	DOWN	[GO:0006950:response to stress]	-	-	XP_010678366.1:PREDICTED: uncharacterized protein LOC104893927 isoform X2 [Beta vulgaris subsp. vulgaris] >KMT10846.1 hypothetical protein BVRB_5g113500 isoform A [Beta vulgaris subsp. vulgaris]	-	A0A0J8F4T2|A0A0J8F4T2_BETVU:Uncharacterized protein;OS=Beta vulgaris subsp. vulgaris.;|GO:0006950,

geneid	trend	GO	Pathway	TF	NR	NT	Uniprot
DN120263_c0_g1_i3	DOWN	[GO:0005774:vacuolar membrane][GO:0004650:polygalacturonase activity][GO:0005975:carbohydrate metabolic process]	-	Itr_sc000031.1_g00012.1:Ipomoea trifida|bHLH|bHLH family protein	XP_010672734.1:PREDICTED: polygalacturonase QRT2 [Beta vulgaris subsp. vulgaris] >KMT15387.1 hypothetical protein BVRB_3g060910 [Beta vulgaris subsp. vulgaris]	gi|521736344|gb|GAIA01008738.1|:TSA: Salicornia europaea Unigene9088_Se200S transcribed RNA sequence	A0A0J8CPD5|A0A0J8CPD5_BETVU:Uncharacterized protein;OS=Beta vulgaris subsp. vulgaris.;|GO:0005774,|GO:0004650,|GO:0005975,
DN123195_c1_g1_i5	DOWN	[GO:0016021:integral component of membrane][GO:0009506:plasmodesma]	-	-	XP_010687826.1:PREDICTED: uncharacterized protein LOC104901901 [Beta vulgaris subsp. vulgaris] >KMT02704.1 hypothetical protein BVRB_8g192950 [Beta vulgaris subsp. vulgaris]	-	A0A0J8BRT1|A0A0J8BRT1_BETVU:Uncharacterized protein;OS=Beta vulgaris subsp. vulgaris.;|GO:0016021,GO:0009506,

geneid	trend	GO	Pathway	TF	NR	NT	Uniprot
DN121929_c1_g3_i1	DOWN	[GO:0030410:nicotianamine synthase activity][GO:0030418:nicotianamine biosynthetic process]	-	-	XP_010674658.1:PREDICTED: nicotianamine synthase [Beta vulgaris subsp. vulgaris] >KMT13674.1 hypothetical protein BVRB_4g080570 [Beta vulgaris subsp. vulgaris]	gi|255542893|ref|XM_002512464.1|:Ricinus communis Nicotianamine synthase, putative, mRNA	A0A0J8CNS6|A0A0J8CNS6_BETVU:Uncharacterized protein;OS=Beta vulgaris subsp. vulgaris.;|GO:0030410,|GO:0030418,
DN120468_c4_g7_i1	DOWN	[GO:0003824:catalytic activity][GO:0050662:coenzyme binding]	-	-	XP_021772066.1:3oxoDelta(4,5)steroid 5betareductaselike [Chenopodium quinoa]	-	A0A078H9D1|A0A078H9D1_BRANA:BnaA01g13700D protein;OS=Brassica napus;|GO:0003824,GO:0050662,
DN125656_c1_g1_i4	DOWN	[GO:0016021:integral component of membrane][GO:0005524:ATP binding][GO:0042626:ATPase activity, coupled to transmembrane movement of substances]	[ko02010:ABC transporters][ko04976:Bile secretion][ko05206:MicroRNAs in cancer]	Zjn_sc00086.1.g02330.1.am.mk:Zoysia japonica|ERF|ERF family protein	XP_021721381.1:ABC transporter B family member 19 [Chenopodium quinoa]	gi|356550794|ref|XM_003543721.1|:PREDICTED: Glycine max ABC transporter B family member 19-like (LOC100797124), mRNA	A0A0K9RV17|A0A0K9RV17_SPIOL:Uncharacterized protein;OS=Spinacia oleracea;|GO:0016021,|GO:0005524,GO:0042626,

geneid	trend	GO	Pathway	TF	NR	NT	Uniprot
DN124225_c1_g6_i2	DOWN	-	-	-	-	-	-
DN126783_c1_g1_i7	DOWN	[GO:0008289:lipid binding]	-	GSBRNA2T00031982001:Brassica napus|DBB|DBB family protein	GAV66415.1:PH domaincontaining protein/START domaincontaining protein/DUF1336 domaincontaining protein [Cephalotus follicularis]	gi|359486882|ref|XM_002274017.2|:PREDICTED: Vitis vinifera uncharacterized LOC100259813 (LOC100259813), mRNA	A0A0J8BKW3|A0A0J8BKW3_BETVU: Uncharacterized protein;OS=Beta vulgaris subsp. vulgaris.;|GO:0008289,
DN120776_c1_g6_i1	DOWN	[GO:0016021:integral component of membrane][GO:0008168:methyltransferase activity]	-	-	XP_021864743.1:uncharacterized protein LOC110803532 [Spinacia oleracea] >KNA18011.1 hypothetical protein SOVF_074180 [Spinacia oleracea]	gi|521512908|gb|GAHZ01018012.1|:TSA: Salicornia europaea Unigene18953_SeCKS transcribed RNA sequence	A0A0K9REU0|A0A0K9REU0_SPIOL: Uncharacterized protein;OS=Spinacia oleracea;|GO:0016021,|GO:0008168,

geneid	trend	GO	Pathway	TF	NR	NT	Uniprot
DN121733_c2_g1_i1	DOWN	[GO:0005524:ATP binding][GO:0004672:protein kinase activity]	-	FANhyb_rscf00000217.1.g00006.1:Fragaria x ananassa|WRKY|WRKY family protein	XP_010685306.1:PREDICTED: putative receptorlike protein kinase At1g72540 [Beta vulgaris subsp. vulgaris] >KMT04824.1 hypothetical protein BVRB_7g169950 [Beta vulgaris subsp. vulgaris]	gi|224922999|gb|AC235235.1|:Glycine max strain Williams 82 clone GM_WBb0028H01, complete sequence	A0A0J8BXU4|A0A0J8BXU4_BETVU: Uncharacterized protein;OS=Beta vulgaris subsp. vulgaris.;|GO:0005524,GO:0004672,
DN116996_c1_g6_i1	DOWN	-	-	-	-	-	-
DN113261_c0_g2_i2	DOWN	-	-	-	-	-	-
DN112266_c0_g2_i1	DOWN	[GO:0006952:defense response][GO:0009607:response to biotic stimulus]	-	-	XP_010684600.1:PREDICTED: major allergen Pru ar 1 [Beta vulgaris subsp. vulgaris] >KMT05929.1 hypothetical protein BVRB_7g165090	-	A0A0J8BRC2|A0A0J8BRC2_BETVU:Uncharacterized protein;OS=Beta vulgaris subsp. vulgaris.;|GO:0006952,GO:0009607,

geneid	trend	GO	Pathway	TF	NR	NT	Uniprot
					[Beta vulgaris subsp. vulgaris]		
DN122084_c0_g4_i4	DOWN	[GO:0003987:acetate-CoA ligase activity][GO:0016208:AMP binding][GO:0019427:acetyl-CoA biosynthetic process from acetate]	[ko00010:Glycolysis / Gluconeogenesis][ko00620:Pyruvate metabolism][ko00640:Propanoate metabolism][ko00680:Methane metabolism][ko00720:Carbon fixation pathways in prokaryotes]	-	XP_021646625.1:acetylcoenzyme A synthetase, chloroplastic/glyoxysomallike isoform X1 [Hevea brasiliensis]	gi|255543444|ref|XM_002512739.1|:Ricinus communis acetyl-CoA synthetase, putative, mRNA	A0A067L0C0|A0A067L0C0_JATCU:Uncharacterized protein;OS=Jatropha curcas;|GO:0003987,GO:0016208,|GO:0019427,
DN121243_c0_g1_i2	DOWN	-	-	-	XP_021753987.1:IRKinteracting proteinlike [Chenopodium quinoa]	gi|521697608|gb|GAIA01018777.1|:TSA: Salicornia europaea Unigene19735_Se200S transcribed RNA	A0A0K9R5D9|A0A0K9R5D9_SPIOL:Uncharacterized protein;OS=Spinacia oleracea;

geneid	trend	GO	Pathway	TF	NR	NT	Uniprot
						sequence	
DN127839_c4_g3_i2	DOWN	[GO:0016021:integral component of membrane]	-	Achn250761:Actinidia chinensis|TCP|TCP family protein	XP_021842098.1:uncharacterized protein LOC110782288 [Spinacia oleracea] >KNA11615.1 hypothetical protein SOVF_133500 [Spinacia oleracea]	gi|502119429|ref|XM_004496560.1|:PREDICTED: Cicer arietinum uncharacterized LOC101512823 (LOC101512823), mRNA	A0A0K9QWJ8|A0A0K9QWJ8_SPIOL: Uncharacterized protein;OS=Spinacia oleracea;|GO:0016021,
DN115532_c0_g1_i3	DOWN	[GO:0005509:calcium ion binding]	[ko04626:Plant-pathogen interaction]	676720782:Sisymbrium irio|FAR1|FAR1 family protein	XP_021767597.1:probable calciumbinding protein CML43 [Chenopodium quinoa]	-	A0A075C736|A0A075C736_9CARY:Calcium binding protein;OS=Reaumuria trigyna.;|GO:0005509,

geneid	trend	GO	Pathway	TF	NR	NT	Uniprot
DN111704_c0_g1_i3	DOWN	[GO:0005576:extracellular region][GO:0030145:manganese ion binding][GO:0045735:nutrient reservoir activity]	-	678365117:Utricularia gibba|bZIP|bZIP family protein	XP_021744584.1:germinlike protein [Chenopodium quinoa]	-	A0A0J8CUD5|A0A0J8CUD5_BETVU: Uncharacterized protein;OS=Beta vulgaris subsp. vulgaris.;|GO:0005576,|GO:0030145,GO:0045735,
DN117527_c0_g4_i14	DOWN	[GO:0016021:integral component of membrane][GO:0008270:zinc ion binding]	-	-	XP_010689560.1:PREDICTED: uncharacterized protein LOC104903248 [Beta vulgaris subsp. vulgaris] >KMT02075.1 hypothetical protein BVRB_9g208040 [Beta vulgaris subsp. vulgaris]	-	A0A0J8BKZ3|A0A0J8BKZ3_BETVU:Uncharacterized protein;OS=Beta vulgaris subsp. vulgaris.;|GO:0016021,|GO:0008270,

geneid	trend	GO	Pathway	TF	NR	NT	Uniprot
DN116315_c2_g1_i2	DOWN	[GO:0005874:microtubule][GO:0005525:GTP binding][GO:0003924:GTPase activity][GO:0005200:structural constituent of cytoskeleton][GO:0007017:microtubule-based process]	[ko04145:Phagosome][ko04540:Gap junction][ko05130:Pathogenic Escherichia coli infection]	KN539001.1_FGP003:Oryza longistaminata|NAC|NAC family protein	XP_010673063.1:PREDICTED: tubulin beta6 chain isoform X1 [Beta vulgaris subsp. vulgaris]	gi|225440130|ref|XM_002283020.1|:PREDICTED: Vitis vinifera tubulin beta-1 chain-like (LOC100244729), mRNA	K4D2Y1|K4D2Y1_SOLLC:Tubulin beta chain;OS=Solanum lycopersicum;|GO:0005874,|GO:0005525,GO:0003924,GO:0005200,|GO:0007017,
DN118869_c2_g3_i1	DOWN	[GO:0016021:integral component of membrane]	-	-	XP_010686967.1:PREDICTED: uncharacterized protein LOC104901121 isoform X1 [Beta vulgaris subsp. vulgaris]	-	A0A0J8BVU5|A0A0J8BVU5_BETVU:Uncharacterized protein;OS=Beta vulgaris subsp. vulgaris.;|GO:0016021,
DN121660_c4_g7_i2	DOWN	-	-	-	-	-	-
DN128713_c5_g6_i1	DOWN	-	-	-	ACN31360.1:unknown [Zea mays]	-	C0PB15|C0PB15_MAIZE:Uncharacterized protein;OS=Zea mays;

geneid	trend	GO	Pathway	TF	NR	NT	Uniprot
DN124262_c0_g1_i6	DOWN	-	-	Achn250761:Actinidia chinensis|TCP|TCP family protein	XP_012441714.1:PREDICTED: uncharacterized protein LOC105766694 [Gossypium raimondii] >KJB62177.1 hypothetical protein B456_009G405000 [Gossypium raimondii]	-	A0A0D2QW30|A0A0D2QW30_GOSRA:Uncharacterized protein;OS=Gossypium raimondii;
DN125608_c0_g1_i7	DOWN	[GO:0016021:integral component of membrane][GO:0005524:ATP binding][GO:0004672:protein kinase activity]	-	Itr_sc000748.1_g00024.1:Ipomoea trifida|MYB_related|MYB_related family protein	XP_021772420.1:probable LRR receptorlike serine/threonineprotein kinase RFK1 isoform X1 [Chenopodium quinoa]	gi|460376127|ref|XM_004233804.1|:PREDICTED: Solanum lycopersicum uncharacterized LOC101253557 (LOC101253557), mRNA	A0A166CU56|A0A166CU56_DAUCA:Uncharacterized protein;OS=Daucus carota subsp. sativus.;|GO:0016021,|GO:0005524,GO:0004672,

geneid	trend	GO	Pathway	TF	NR	NT	Uniprot
DN117599_c0_g1_i2	DOWN	[GO:0016831:carboxy-lyase activity][GO:0030170:pyridoxal phosphate binding][GO:0006520:cellular amino acid metabolic process]	[ko00350:Tyrosine metabolism][ko00950:Isoquinoline alkaloid biosynthesis]	-	XP_010666091.1:PREDICTED: tyrosine/DOPA decarboxylase 1 [Beta vulgaris subsp. vulgaris] >KMS96359.1 hypothetical protein BVRB_9g225880 [Beta vulgaris subsp. vulgaris]	-	A0A0J8B553|A0A0J8B553_BETVU:Uncharacterized protein;OS=Beta vulgaris subsp. vulgaris.;|GO:0016831,GO:0030170,|GO:0006520,
DN127849_c1_g4_i4	DOWN	[GO:0016021:integral component of membrane][GO:0005524:ATP binding][GO:0042626:ATPase activity, coupled to transmembrane movement of substances]	[ko02010:ABC transporters][ko04976:Bile secretion][ko05206:MicroRNAs in cancer]	Zjn_sc00086.1.g02330.1.am.mk:Zoysia japonica|ERF|ERF family protein	ASM90198.1:multidrug resistance 16 [Sesuvium portulacastrum]	gi|470103821|ref|XM_004288278.1|:PREDICTED: Fragaria vesca subsp. vesca ABC transporter B family member 4-like (LOC101311221), mRNA	A0A0J8BPC6|A0A0J8BPC6_BETVU:Uncharacterized protein;OS=Beta vulgaris subsp. vulgaris.;|GO:0016021,|GO:0005524,GO:0042626,

geneid	trend	GO	Pathway	TF	NR	NT	Uniprot
DN119162_c3_g1_i2	DOWN	[GO:0046872:metal ion binding][GO:0045486:naringenin 3-dioxygenase activity][GO:0009813:flavonoid biosynthetic process][GO:0009416:response to light stimulus]	[ko00941:Flavonoid biosynthesis]	676709864:Sisymbrium irio|STAT|STAT family protein	XP_002275563.1:PREDICTED: flavanone 3dioxygenase [Vitis vinifera] >CAN80262.1 hypothetical protein VITISV_027403 [Vitis vinifera]	gi|339715869|gb|HM543570.1|:Prunus persica flavanone 3-hydroxylase (F3H) mRNA, complete cds	A5ANR7|A5ANR7_VITVI:Putative uncharacterized protein;OS=Vitis vinifera;GN=VIT_18s0001g14310 {ECO:0000313|EMBL:CCB46039.1};|GO:0046872,GO:0045486,|GO:0009813,GO:0009416,
DN111502_c0_g1_i1	DOWN	-	-	-	XP_002307232.2:hypothetical protein POPTR_0005s13840g [Populus trichocarpa] >EEE94228.2 hypothetical protein POPTR_0005s13840g [Populus trichocarpa]	-	B9H6A5|B9H6A5_POPTR:Uncharacterized protein;OS=Populus trichocarpa;

geneid	trend	GO	Pathway	TF	NR	NT	Uniprot
DN117083_c0_g2_i2	DOWN	[GO:0046872:metal ion binding][GO:0016491:oxidoreductase activity]	-	Zpz_sc00083.1.g00290.1.am.mk:Zoysia pacifica|Dof|Dof family protein	XP_021759327.1:2oxoglutaratedependent dioxygenase DAOlike [Chenopodium quinoa]	-	A0A0J8C4G8|A0A0J8C4G8_BETVU:Uncharacterized protein;OS=Beta vulgaris subsp. vulgaris.;|GO:0046872,GO:0016491,
DN127528_c4_g2_i4	DOWN	-	-	-	OIW06231.1:hypothetical protein TanjilG_03856 [Lupinus angustifolius]	-	-
DN127085_c1_g4_i2	DOWN	[GO:0016021:integral component of membrane][GO:0004190:aspartic-type endopeptidase activity]	-	-	XP_021838961.1:aspartic proteinase PCS1 [Spinacia oleracea] >KNA19908.1 hypothetical protein SOVF_057310 [Spinacia oleracea]	gi|521442117|gb|GAHZ01078367.1|:TSA: Salicornia europaea Unigene95970_SeCKS transcribed RNA sequence	A0A0K9RK83|A0A0K9RK83_SPIOL:Uncharacterized protein;OS=Spinacia oleracea;|GO:0016021,|GO:0004190,

geneid	trend	GO	Pathway	TF	NR	NT	Uniprot
DN123838_c3_g8_i7	DOWN	[GO:0016021:integral component of membrane]	-	GSMUA_Achr1P00070_001:Musa acuminata|ERF|ERF family protein	XP_010675618.1:PREDICTED: uncharacterized protein LOC104891608 [Beta vulgaris subsp. vulgaris] >KMT13037.1 hypothetical protein BVRB_4g087510 [Beta vulgaris subsp. vulgaris]	gi|38016520|gb|AY429438.1|:Gossypium barbadense fiber protein Fb34 mRNA, complete cds	A0A0J8CHL7|A0A0J8CHL7_BETVU:Uncharacterized protein;OS=Beta vulgaris subsp. vulgaris.;|GO:0016021,
DN126986_c3_g1_i4	DOWN	[GO:0016021:integral component of membrane]	-	-	XP_010672073.1:PREDICTED: protein Oglucosyltransferase 1 isoform X1 [Beta vulgaris subsp. vulgaris] >XP_019103926.1 PREDICTED: protein Oglucosyltransferase 1 isoform X1 [Beta vulgaris subsp. vulgaris]	gi|449518612|ref|XM_004166283.1|:PREDICTED: Cucumis sativus protein O-glucosyltransferase 1-like (LOC101203870), mRNA	A0A0J8FYA5|A0A0J8FYA5_BETVU:Uncharacterized protein;OS=Beta vulgaris subsp. vulgaris.;|GO:0016021,

geneid	trend	GO	Pathway	TF	NR	NT	Uniprot
					>KMT20626.1 hypothetical protein BVRB_1g006040 [Beta vulgaris subsp. vulgaris]		
DN121806_c0_g5_i1	DOWN	[GO:0008234:cysteine-type peptidase activity]	-	FANhyb_rscf00000253.1.g00010.1:Fragaria x ananassa|BBR-BPC|BBR-BPC family protein	AHA62778.1:RCR3like cysteine protease [Mirabilis jalapa]	-	V5M3B7|V5M3B7_MIRJA:RCR3-like cysteine protease;OS=Mirabilis jalapa;|GO:0008234,

geneid	trend	GO	Pathway	TF	NR	NT	Uniprot
DN125546_c3_g2_i10	DOWN	-	-	-	XP_010680424.1:PREDICTED: uncharacterized protein LOC104895573 isoform X1 [Beta vulgaris subsp. vulgaris] >XP_010680425.1 PREDICTED: uncharacterized protein LOC104895573 isoform X1 [Beta vulgaris subsp. vulgaris] >KMT09346.1 hypothetical protein BVRB_6g134400 [Beta vulgaris subsp. vulgaris]	gi|521482573|gb|GAHZ01048313.1|:TSA: Salicornia europaea Unigene56863_SeCKS transcribed RNA sequence	A0A0J8F1A1|A0A0J8F1A1_BETVU:Uncharacterized protein;OS=Beta vulgaris subsp. vulgaris.;

geneid	trend	GO	Pathway	TF	NR	NT	Uniprot
DN116561_c5_g1_i4	DOWN	[GO:0016021:integral component of membrane][GO:0004575:sucrose alpha-glucosidase activity][GO:0005975:carbohydrate metabolic process]	-	OB01G21870.1:Oryza brachyantha|WRKY|WRKY family protein	XP_021737768.1:uncharacterized protein LOC110704285 [Chenopodium quinoa]	-	A0A0J8CVC3|A0A0J8CVC3_BETVU: Uncharacterized protein;OS=Beta vulgaris subsp. vulgaris.;|GO:0016021,|GO:0004575,|GO:0005975,
DN122084_c0_g4_i3	DOWN	[GO:0003987:acetate-CoA ligase activity][GO:0016208:AMP binding][GO:0019427:acetyl-CoA biosynthetic process from acetate]	[ko00010:Glycolysis / Gluconeogenesis][ko00620:Pyruvate metabolism][ko00640:Propanoate metabolism][ko00680:Methane metabolism][ko00720:Carbon fixation pathways in prokaryotes]	-	KDP41901.1:hypothetical protein JCGZ_26919 [Jatropha curcas]	gi|449484576|ref|XM_004156872.1|:PREDICTED: Cucumis sativus acetate--CoA ligase ACS, chloroplastic/glyoxysomal-like (LOC101203579), mRNA	A0A067L0C0|A0A067L0C0_JATCU:Uncharacterized protein;OS=Jatropha curcas;|GO:0003987,GO:0016208,|GO:0019427,

geneid	trend	GO	Pathway	TF	NR	NT	Uniprot
DN118019_c0_g1_i2	DOWN	[GO:0016021:integral component of membrane][GO:0055085:transmembrane transport]	-	-	XP_010683591.1:PREDICTED: mitochondrial thiamine pyrophosphate carrierlike isoform X1 [Beta vulgaris subsp. vulgaris] >XP_010683592.1 PREDICTED: mitochondrial thiamine pyrophosphate carrierlike isoform X1 [Beta vulgaris subsp. vulgaris] >XP_019106267.1 PREDICTED: mitochondrial thiamine pyrophosphate carrierlike isoform X1 [Beta vulgaris subsp. vulgaris]	gi|521717832|gb|GAIA01011880.1|:TSA: Salicornia europaea Unigene12498_Se200S transcribed RNA sequence	A0A0K9S0N1|A0A0K9S0N1_SPIOL:Uncharacterized protein;OS=Spinacia oleracea;|GO:0016021,|GO:0055085,

geneid	trend	GO	Pathway	TF	NR	NT	Uniprot
DN118111_c2_g1_i2	DOWN	[GO:0048046:apoplast][GO:0005618:cell wall][GO:0004553:hydrolase activity, hydrolyzing O-glycosyl compounds][GO:0016762:xyloglucan:xyloglucosyl transferase activity][GO:0042546:cell wall biogenesis][GO:0071555:cell wall organization][GO:0010411:xyloglucan metabolic process]	-	Sme2.5_00096.1_g00018.1:Solanum melongena|NAC|NAC family protein	XP_010677382.1:PREDICTED: probable xyloglucan endotransglucosylase/hydrolase protein 30 [Beta vulgaris subsp. vulgaris] >KMT11384.1 hypothetical protein BVRB_5g107150 [Beta vulgaris subsp. vulgaris]	gi|147787675|emb|AM443312.2|:Vitis vinifera contig VV78X035602.8, whole genome shotgun sequence	A0A0J8CH06|A0A0J8CH06_BETVU:Xyloglucan endotransglucosylase/hydrolase;OS=Beta vulgaris subsp. vulgaris.;|GO:0048046,GO:0005618,|GO:0004553,GO:0016762,|GO:0042546,GO:0071555,GO:0010411,
DN124134_c3_g5_i6	DOWN	[GO:0016491:oxidoreductase activity][GO:0008270:zinc ion binding]	[ko00010:Glycolysis / Gluconeogenesis][ko00071:Fatty acid degradation][ko00350:Tyrosine metabolism][ko00592:alpha-Linolenic acid metabolism]	-	XP_021600872.1:alcohol dehydrogenaselike [Manihot esculenta] >OAY23545.1 hypothetical protein MANES_18G087000 [Manihot esculenta]	-	A0A0K9QY28|A0A0K9QY28_SPIOL:Uncharacterized protein;OS=Spinacia oleracea;|GO:0016491,GO:0008270,

geneid	trend	GO	Pathway	TF	NR	NT	Uniprot
DN110485_c0_g1_i3	DOWN	-	-	Csa10g007600.1:Camelina sativa|bHLH|bHLH family protein	KNA22291.1:hypothetical protein SOVF_035310 isoform B [Spinacia oleracea]	gi|521745514|gb|GAIA01003243.1|:TSA: Salicornia europaea Unigene3413_Se200S transcribed RNA sequence	A0A0K9RRW1|A0A0K9RRW1_SPIOL:Uncharacterized protein;OS=Spinacia oleracea;
DN122131_c1_g3_i1	DOWN	[GO:0016491:oxidoreductase activity]	[ko00330:Arginine and proline metabolism][ko00410:beta-Alanine metabolism]	676755044:Sisymbrium irio|bHLH|bHLH family protein	XP_010688837.1:PREDICTED: probable polyamine oxidase 2 isoform X2 [Beta vulgaris subsp. vulgaris] >KMT02426.1 hypothetical protein BVRB_9g203800 [Beta vulgaris subsp. vulgaris]	gi|460373722|ref|XM_004232616.1|:PREDICTED: Solanum lycopersicum probable polyamine oxidase 4-like (LOC101264170), mRNA	A0A0J8EHE3|A0A0J8EHE3_BETVU:Uncharacterized protein;OS=Beta vulgaris subsp. vulgaris.;|GO:0016491,
DN122441_c1_g2_i2	DOWN	[GO:0005634:nucleus][GO:0043565:sequence-specific DNA binding][GO:0003700:DNA-binding transcription factor activity][GO:0006351:transcription, DNA-templated]	-	Bv5_102770_jswm.t1:Beta vulgaris|Trihelix|Trihelix family protein	XP_021769680.1:trihelix transcription factor GT3blike [Chenopodium quinoa]	gi|478153830|gb|GAHK01022773.1|:TSA: Jatropha curcas Unigene10231_JC-CK_1A transcribed RNA sequence	A0A0J8CFL2|A0A0J8CFL2_BETVU:Uncharacterized protein;OS=Beta vulgaris subsp. vulgaris.;|GO:0005634,|GO:0043565,GO:0003700,|GO:00063

geneid	trend	GO	Pathway	TF	NR	NT	Uniprot
							51,
DN128015_c1_g1_i6	DOWN	[GO:0048046:apoplast][GO:0005507:copper ion binding][GO:0052716:hydroquinone:oxygen oxidoreductase activity][GO:0046274:lignin catabolic process]	-	Rsa1.0_03034.1_g00001.1:Raphanus sativus|ERF|ERF family protein	XP_010685615.1:PREDICTED: laccase4 [Beta vulgaris subsp. vulgaris] >KMT05118.1 hypothetical protein BVRB_7g172690 [Beta vulgaris subsp. vulgaris]	gi|1685086|gb|U43542.1|NTU43542:Nicotiana tabacum diphenol oxidase mRNA, complete cds	A0A0J8BYU1|A0A0J8BYU1_BETVU: Laccase;OS=Beta vulgaris subsp. vulgaris.;|GO:0048046,|GO:0005507,GO:0052716,|GO:0046274,
DN127476_c0_g1_i2	DOWN	-	-	-	-	-	-
DN113753_c2_g1_i5	DOWN	[GO:0009055:electron transfer activity]	-	-	XP_021760777.1:umecyaninlike [Chenopodium quinoa]	-	A0A166B7R2|A0A166B7R2_DAUCA:Uncharacterized protein;OS=Daucus carota subsp. sativus.;|GO:000905

geneid	trend	GO	Pathway	TF	NR	NT	Uniprot
							5,
DN114864_c2_g1_i2	DOWN	-	-	Bv7_168260_jzca.t1:Beta vulgaris|bHLH|bHLH family protein	XP_010685111.1:PREDICTED: transcription factor bHLH78 [Beta vulgaris subsp. vulgaris] >KMT05583.1 hypothetical protein BVRB_7g168260 [Beta vulgaris subsp. vulgaris]	gi|521633890|gb|GAIA01049504.1|:TSA: Salicornia europaea Unigene57912_Se200S transcribed RNA sequence	A0A0J8C085|A0A0J8C085_BETVU:Uncharacterized protein;OS=Beta vulgaris subsp. vulgaris.;
DN120085_c3_g1_i2	DOWN	[GO:0005524:ATP binding][GO:0004672:protein kinase activity]	-	FANhyb_rscf00000217.1.g00006.1:Fragaria x ananassa|WRKY|WRKY family protein	XP_021866482.1:serine/threonineprotein kinase RIPKlike [Spinacia oleracea]	gi|53857146|gb|AY220481.2|:Nicotiana tabacum Avr9/Cf-9 induced kinase 1 (ACIK1) mRNA, complete cds	A0A0J8B8C8|A0A0J8B8C8_BETVU:Uncharacterized protein;OS=Beta vulgaris subsp. vulgaris.;|GO:0005524,GO:0004672,

geneid	trend	GO	Pathway	TF	NR	NT	Uniprot
DN122576_c1_g3_i3	DOWN	[GO:0050660:flavin adenine dinucleotide binding][GO:0004499:N,N-dimethylaniline monooxygenase activity][GO:0050661:NADP binding]	[ko00380:Tryptophan metabolism]	-	XP_021735654.1:indole3pyruvate monooxygenase YUCCA6like [Chenopodium quinoa] >XP_021719351.1 indole3pyruvate monooxygenase YUCCA6like [Chenopodium quinoa]	-	A0A0K9RS63|A0A0K9RS63_SPIOL:Flavin-containing monooxygenase;OS=Spinacia oleracea;|GO:0050660,GO:0004499,GO:0050661,
DN125856_c3_g1_i8	DOWN	-	-	-	XP_010675324.1:PREDICTED: IRKinteracting protein [Beta vulgaris subsp. vulgaris] >KMT13291.1 hypothetical protein BVRB_4g085500 [Beta vulgaris subsp. vulgaris]	-	A0A0J8FBS0|A0A0J8FBS0_BETVU:Uncharacterized protein;OS=Beta vulgaris subsp. vulgaris.;

geneid	trend	GO	Pathway	TF	NR	NT	Uniprot
DN124216_c1_g1_i1	DOWN	[GO:0004097:catechol oxidase activity][GO:0046872:metal ion binding][GO:0046148:pigment biosynthetic process]	[ko00350:Tyrosine metabolism][ko00950:Isoquinoline alkaloid biosynthesis]	-	XP_021865904.1:polyphenol oxidase, chloroplasticlike [Spinacia oleracea]	gi|115392290|gb|DQ851217.1|:Prunus mexicana putative polyphenol oxidase (ppo) gene, partial cds	I4DD58|I4DD58_POROL:Uncharacterized protein;OS=Portulaca oleracea;|GO:0004097,GO:0046872,|GO:0046148,

geneid	trend	GO	Pathway	TF	NR	NT	Uniprot
DN123921_c3_g2_i1	DOWN	[GO:0005623:cell][GO:0005524:ATP binding][GO:0005536:glucose binding][GO:0004396:hexokinase activity][GO:0001678:cellular glucose homeostasis][GO:0006096:glycolytic process]	[ko00010:Glycolysis / Gluconeogenesis][ko00051:Fructose and mannose metabolism][ko00052:Galactose metabolism][ko00500:Starch and sucrose metabolism][ko00520:Amino sugar and nucleotide sugar metabolism][ko00521:Streptomycin biosynthesis][ko00524:Neomycin, kanamycin and gentamicin biosynthesis][ko04066:HIF-1 signaling pathway][ko04910:Insulin signaling pathway][ko04930:Type II diabetes mellitus][ko04973:C	KHN04400.1:Glycine soja|G2-like|G2-like family protein	XP_021751499.1:hexokinase3like [Chenopodium quinoa]	gi|521505392|gb|GAHZ01025528.1|:TSA: Salicornia europaea Unigene27688_SeCKS transcribed RNA sequence	A0A0J8CM25|A0A0J8CM25_BETVU: Phosphotransferase; OS=Beta vulgaris subsp. vulgaris.;|GO:0005623,|GO:0005524,GO:0005536,GO:0004396,|GO:0001678,GO:0006096,

geneid	trend	GO	Pathway	TF	NR	NT	Uniprot
			arbohydrate digestion and absorption][ko05230:Central carbon metabolism in cancer]				

geneid	trend	GO	Pathway	TF	NR	NT	Uniprot
DN114629_c0_g1_i3	DOWN	[GO:0016021:integral component of membrane]	-	-	XP_010691334.1:PREDICTED: uncharacterized protein LOC104904684 [Beta vulgaris subsp. vulgaris] >KMT00818.1 hypothetical protein BVRB_9g220940 [Beta vulgaris subsp. vulgaris]	gi|403043539|ref|NM_001254412.2|:Glycine max uncharacterized LOC100818121 (LOC100818121), mRNA	A0A0J8BL17|A0A0J8BL17_BETVU:Uncharacterized protein;OS=Beta vulgaris subsp. vulgaris.;|GO:0016021,
DN127772_c2_g8_i6	DOWN	[GO:0032040:small-subunit processome]	-	Achn356291:Actinidia chinensis|MIKC_MADS|MIKC_MADS family protein	XP_010681128.1:PREDICTED: nucleolar protein 14 [Beta vulgaris subsp. vulgaris] >KMT08702.1 hypothetical protein BVRB_6g139750 [Beta vulgaris subsp. vulgaris]	gi|521670917|gb|GAIA01032066.1|:TSA: Salicornia europaea Unigene35193_Se200S transcribed RNA sequence	A0A0J8C595|A0A0J8C595_BETVU:Uncharacterized protein;OS=Beta vulgaris subsp. vulgaris.;|GO:0032040,

geneid	trend	GO	Pathway	TF	NR	NT	Uniprot
DN117978_c0_g4_i4	DOWN	[GO:0000287:magnesium ion binding][GO:0010333:terpene synthase activity][GO:0008152:metabolic process]	[ko00909:Sesquiterpenoid and triterpenoid biosynthesis]	Cotton_A_17926_BGI-A2_v1.0:Gossypium arboreum|GRAS|GRAS family protein	AHF22834.1:drimenol synthase [Persicaria hydropiper]	-	W0FFD7|W0FFD7_PERHD:Drimenol synthase;OS=Persicaria hydropiper;|GO:0000287,GO:0010333,|GO:0008152,
DN125958_c1_g1_i10	DOWN	-	-	XP_009771234.1:Nicotiana sylvestris|ERF|ERF family protein	XP_019102784.1:PREDICTED: uncharacterized protein LOC109133657 [Beta vulgaris subsp. vulgaris]	-	A0A0K9RY82|A0A0K9RY82_SPIOL:Uncharacterized protein;OS=Spinacia oleracea;
DN121165_c2_g3_i9	DOWN	[GO:0004722:protein serine/threonine phosphatase activity]	-	KZV22762.1:Dorcoceras hygrometricum|CAMTA|CAMTA family protein	XP_010674092.1:PREDICTED: probable protein phosphatase 2C 34 [Beta vulgaris subsp. vulgaris] >KMT14266.1 hypothetical protein BVRB_4g076220 [Beta vulgaris subsp. vulgaris]	gi|225321289|dbj|AK325500.1|:Solanum lycopersicum cDNA, clone: LEFL1097BB08, HTC in leaf	A0A0J8FEL1|A0A0J8FEL1_BETVU:Uncharacterized protein;OS=Beta vulgaris subsp. vulgaris.;|GO:0004722,

geneid	trend	GO	Pathway	TF	NR	NT	Uniprot
DN125877_c1_g1_i2	DOWN	[GO:0044212:transcription regulatory region DNA binding]	-	Bv4_079240_zfig.t1:Beta vulgaris|MYB|MYB family protein	XP_010674459.1:PREDICTED: transcription factor MYB86 [Beta vulgaris subsp. vulgaris] >KMT14099.1 hypothetical protein BVRB_4g079240 [Beta vulgaris subsp. vulgaris]	gi|521546277|gb|GAHZ01000506.1|:TSA: Salicornia europaea Unigene519_SeCKS transcribed RNA sequence	A0A0J8CQ17|A0A0J8CQ17_BETVU:Uncharacterized protein;OS=Beta vulgaris subsp. vulgaris.;|GO:0044212,
DN128780_c2_g3_i1	DOWN	-	-	-	-	-	-
DN102181_c0_g1_i1	DOWN	[GO:0004568:chitinase activity][GO:0005975:carbohydrate metabolic process][GO:0016998:cell wall macromolecule catabolic process][GO:0006032:chitin catabolic process]	[ko00520:Amino sugar and nucleotide sugar metabolism]	-	XP_004953034.1:PREDICTED: chitinase 6like [Setaria italica] >KQL30395.1 hypothetical protein SETIT_018061mg [Setaria italica]	-	Q7M1Q9|Q7M1Q9_PHYAM:Chitinase A;OS=Phytolacca americana;|GO:0004568,|GO:0005975,GO:0016998,GO:0006032,

geneid	trend	GO	Pathway	TF	NR	NT	Uniprot
DN124100_c6_g16_i1	DOWN	-	-	-	-	gi|418731483|gb|JX839758.1|:Solanum tuberosum clone StDT188 abscisic stress ripening protein 4 mRNA, partial cds	-
DN119752_c4_g1_i8	DOWN	[GO:0004067:asparaginase activity]	-	-	XP_002514609.1:PREDICTED: isoaspartyl peptidase/Lasparaginase 1 [Ricinus communis] >EEF47715.1 lasparaginase, putative [Ricinus communis]	gi|224115911|ref|XM_002332007.1|:Populus trichocarpa predicted protein, mRNA	B9RLJ0|B9RLJ0_RICCO:L-asparaginase, putative;OS=Ricinus communis;|GO:0004067,
DN121719_c0_g1_i1	DOWN	[GO:0003824:catalytic activity][GO:0050662:coenzyme binding]	-	-	XP_021772066.1:3oxoDelta(4,5)steroid 5betareductaselike [Chenopodium quinoa]	gi|478161917|gb|GAHK01014686.1|:TSA: Jatropha curcas Unigene2140_JC-CK_1A transcribed RNA sequence	A0A067LC28|A0A067LC28_JATCU:Uncharacterized protein;OS=Jatropha curcas;|GO:0003824,GO:0050662,

geneid	trend	GO	Pathway	TF	NR	NT	Uniprot
DN121733_c2_g3_i9	DOWN	[GO:0005524:ATP binding][GO:0004672:protein kinase activity]	-	PSME_00040474-RA:Pseudotsuga menziesii|M-type_MADS|M-type_MADS family protein	XP_021716532.1:probable serine/threonineprotein kinase PBL12 [Chenopodium quinoa]	-	A0A0J8EPB5|A0A0J8EPB5_BETVU:Uncharacterized protein;OS=Beta vulgaris subsp. vulgaris.;|GO:0005524,GO:0004672,
DN122280_c1_g3_i3	DOWN	-	-	-	-	-	-
DN119554_c0_g4_i1	DOWN	-	-	-	-	-	-
DN124244_c4_g1_i2	DOWN	[GO:0005634:nucleus][GO:0003677:DNA binding][GO:0003700:DNA-binding transcription factor activity][GO:0006351:transcription, DNA-templated]	-	DCAR_028674:Daucus carota|ERF|ERF family protein	XP_017221967.1:PREDICTED: dehydrationresponsive elementbinding protein 3like [Daucus carota subsp. sativus] >KZM83904.1 hypothetical protein DCAR_028674 [Daucus carota subsp. sativus]	-	A0A175YJK8|A0A175YJK8_DAUCA:Uncharacterized protein;OS=Daucus carota subsp. sativus.;|GO:0005634,|GO:0003677,GO:0003700,|GO:0006351,

geneid	trend	GO	Pathway	TF	NR	NT	Uniprot
DN118191_c0_g1_i5	DOWN	[GO:0005794:Golgi apparatus][GO:0016021:integral component of membrane][GO:0015020:glucuronosyltransferase activity][GO:0009834:plant-type secondary cell wall biogenesis][GO:0045492:xylan biosynthetic process]	-	-	XP_021728089.1:UDPglucuronate:xylan alphaglucuronosyltransferase 1like [Chenopodium quinoa]	gi|521655962|gb|GAIA01039188.1|:TSA: Salicornia europaea Unigene43854_Se200S transcribed RNA sequence	A0A0J8F0X4|A0A0J8F0X4_BETVU:Hexosyltransferase;OS=Beta vulgaris subsp. vulgaris.;|GO:0005794,GO:0016021,|GO:0015020,|GO:0009834,GO:0045492,
DN123919_c2_g1_i5	DOWN	[GO:0005886:plasma membrane][GO:0008081:phosphoric diester hydrolase activity][GO:0006629:lipid metabolic process]	-	Kaladp0020s0147.1.p:Kalanchoe laxiflora|bZIP|bZIP family protein	XP_010667411.1:PREDICTED: PIPLCX domaincontaining protein At5g67130 [Beta vulgaris subsp. vulgaris] >KMS95399.1 hypothetical protein BVRB_008570 [Beta vulgaris subsp. vulgaris]	-	A0A0J8B2Q3|A0A0J8B2Q3_BETVU:Uncharacterized protein;OS=Beta vulgaris subsp. vulgaris.;|GO:0005886,|GO:0008081,|GO:0006629,

geneid	trend	GO	Pathway	TF	NR	NT	Uniprot
DN118758_c0_g1_i3	DOWN	[GO:0004747:ribokinase activity][GO:0006014:D-ribose metabolic process]	[ko00051:Fructose and mannose metabolism][ko00500:Starch and sucrose metabolism][ko00520:Amino sugar and nucleotide sugar metabolism]	mrna13356.1-v1.0-hybrid:Fragaria vesca|MIKC_MADS|MIKC_MADS family protein	XP_010672224.1:PREDICTED: probable fructokinase4 [Beta vulgaris subsp. vulgaris] >KMT15770.1 hypothetical protein BVRB_3g057460 isoform A [Beta vulgaris subsp. vulgaris]	gi|1052972|gb|U37838.1|BVU37838:Beta vulgaris fructokinase mRNA, complete cds	A0A0J8CPR6|A0A0J8CPR6_BETVU:Uncharacterized protein;OS=Beta vulgaris subsp. vulgaris.;|GO:0004747,|GO:0006014,
DN116990_c0_g2_i1	DOWN	[GO:0005634:nucleus][GO:0003677:DNA binding][GO:0006355:regulation of transcription, DNA-templated][GO:0006351:transcription, DNA-templated]	-	AHYPO_014626-RA:Amaranthus hypochondriacus|G2-like|G2-like family protein	XP_021713727.1:mybrelated protein 1like isoform X2 [Chenopodium quinoa]	gi|302398966|gb|HM122639.1|:Malus x domestica MYBR domain class transcription factor (MYBR14) mRNA, complete cds	B9S7B5|B9S7B5_RICCO:Putative uncharacterized protein;OS=Ricinus communis;|GO:0005634,|GO:0003677,|GO:0006355,GO:0006351,

geneid	trend	GO	Pathway	TF	NR	NT	Uniprot
DN127735_c0_g2_i7	DOWN	[GO:0008270:zinc ion binding]	-	MDP0000266172:Malus domestica|Nin-like|Nin-like family protein	XP_021714578.1:pentatricopeptide repeatcontaining protein At5g44230like [Chenopodium quinoa] >XP_021714579.1 pentatricopeptide repeatcontaining protein At5g44230like [Chenopodium quinoa] >XP_021714580.1 pentatricopeptide repeatcontaining protein At5g44230like [Chenopodium quinoa] >XP_021714581.1 pentatricopeptide repeatcontaining protein	gi|224133789|ref|XM_002327645.1|:Populus trichocarpa predicted protein, mRNA	F6GWS8|F6GWS8_VITVI:Putative uncharacterized protein;OS=Vitis vinifera;GN=VIT_04s0023g02490 {ECO:0000313|EMBL:CCB44413.1};|GO:0008270,

geneid	trend	GO	Pathway	TF	NR	NT	Uniprot
					At5g44230like [Chenopodium quinoa]		
DN123478_c1_g1_i8	DOWN	[GO:0050660:flavin adenine dinucleotide binding][GO:0004499:N,N-dimethylaniline monooxygenase activity][GO:0050661:NADP binding]	[ko00982:Drug metabolism - cytochrome P450]	-	KNA14954.1:hypothetical protein SOVF_102690 [Spinacia oleracea]	-	A0A0K9R7J8|A0A0K9R7J8_SPIOL:Uncharacterized protein;OS=Spinacia oleracea;|GO:0050660,GO:0004499,GO:0050661,

geneid	trend	GO	Pathway	TF	NR	NT	Uniprot
DN125382_c0_g3_i2	DOWN	[GO:0016021:integral component of membrane][GO:0005215:transporter activity][GO:0006857:oligopeptide transport]	-	Sopen09g001330.1: Solanum pennellii|G2-like|G2-like family protein	XP_010680056.1:PREDICTED: protein NRT1/ PTR FAMILY 8.3 [Beta vulgaris subsp. vulgaris] >KMT09724.1 hypothetical protein BVRB_6g131730 [Beta vulgaris subsp. vulgaris]	-	A0A0J8CBX0|A0A0J8CBX0_BETVU: Uncharacterized protein;OS=Beta vulgaris subsp. vulgaris.;|GO:0016021,|GO:0005215,|GO:0006857,
DN116564_c4_g4_i7	DOWN	-	-	-	XP_021751950.1:uncharacterized protein LOC110717532 isoform X1 [Chenopodium quinoa]	gi|521505652|gb|GAHZ01025268.1|:TSA: Salicornia europaea Unigene27387_SeCKS transcribed RNA sequence	A0A0J8CBJ4|A0A0J8CBJ4_BETVU:Uncharacterized protein;OS=Beta vulgaris subsp. vulgaris.;
DN113210_c0_g1_i3	DOWN	[GO:0043565:sequence-specific DNA binding][GO:0003700:DNA-binding transcription factor activity]	[ko04016:MAPK signaling pathway - plant][ko04626:Plant-pathogen interaction]	Sp_060460_fefj.t1:Spinacia oleracea|WRKY|WRKY family protein	XP_021767146.1:WRKY transcription factor 22like [Chenopodium quinoa]	gi|460368149|ref|XM_004229884.1|:PREDICTED: Solanum lycopersicum WRKY transcription factor 22-like (LOC101261749), mRNA	A0A0K9RJ46|A0A0K9RJ46_SPIOL:Uncharacterized protein;OS=Spinacia oleracea;|GO:0043565,GO:0003700,

geneid	trend	GO	Pathway	TF	NR	NT	Uniprot
DN128780_c2_g8_i1	DOWN	[GO:0004568:chitinase activity][GO:0005975:carbohydrate metabolic process][GO:0016998:cell wall macromolecule catabolic process][GO:0006032:chitin catabolic process]	[ko00520:Amino sugar and nucleotide sugar metabolism]	-	OIV98483.1:hypothetical protein TanjilG_18767 [Lupinus angustifolius]	-	Q7M1Q9|Q7M1Q9_PHYAM:Chitinase A;OS=Phytolacca americana;|GO:0004568,|GO:0005975,GO:0016998,GO:0006032,
DN128238_c1_g5_i4	DOWN	-	-	-	-	-	-
DN116210_c4_g1_i1	DOWN	-	-	-	-	-	-
DN128833_c0_g1_i2	DOWN	-	-	-	-	-	-

geneid	trend	GO	Pathway	TF	NR	NT	Uniprot
DN121980_c1_g1_i1	DOWN	[GO:0016021:integral component of membrane][GO:0004030:aldehyde dehydrogenase [NAD(P)+] activity][GO:0006081:cellular aldehyde metabolic process]	[ko00010:Glycolysis / Gluconeogenesis][ko000053:Ascorbate and aldarate metabolism][ko00071:Fatty acid degradation][ko00280:Valine, leucine and isoleucine degradation][ko00310:Lysine degradation][ko00330:Arginine and proline metabolism][ko00340:Histidine metabolism][ko00380:Tryptophan metabolism][ko00410:beta-Alanine metabolism][ko00561:Glycerolipid metabolism][ko00620:Pyruvate	-	XP_010673629.1:PREDICTED: aldehyde dehydrogenase family 3 member F1 [Beta vulgaris subsp. vulgaris] >KMT14482.1 hypothetical protein BVRB_4g072550 [Beta vulgaris subsp. vulgaris]	-	A0A0J8CLU1|A0A0J8CLU1_BETVU:Aldehyde dehydrogenase;OS=Beta vulgaris subsp. vulgaris.;|GO:0016021,|GO:0004030,|GO:0006081,

geneid	trend	GO	Pathway	TF	NR	NT	Uniprot
			metabolism][ko00625:Chloroalkane and chloroalkene degradation][ko00903:Limonene and pinene degradation][ko00981:Insect hormone biosynthesis]				

geneid	trend	GO	Pathway	TF	NR	NT	Uniprot
DN113217_c1_g7_i1	DOWN	[GO:0003964:RNA-directed DNA polymerase activity]	-	Rsa1.0_04342.1_g00002.1:Raphanus sativus|LBD|LBD family protein	XP_010684619.1:PREDICTED: uncharacterized protein LOC104899180 [Beta vulgaris subsp. vulgaris]	-	O82216|O82216_ARATH:Putative non-LTR retroelement reverse transcriptase;OS=Arabidopsis thaliana;GN=At2g23880 {ECO:0000313|EMBL:AAC63678.1};|GO:0003964,
DN127346_c2_g7_i3	DOWN	-	-	-	XP_021755345.1:EG45like domain containing protein [Chenopodium quinoa]	-	A0A0J8BQ32|A0A0J8BQ32_BETVU:Uncharacterized protein;OS=Beta vulgaris subsp. vulgaris.;
DN111677_c0_g1_i2	DOWN	[GO:0008270:zinc ion binding]	-	-	KNA05680.1:hypothetical protein SOVF_188000 [Spinacia oleracea]	-	A0A0K9QEJ2|A0A0K9QEJ2_SPIOL:Uncharacterized protein;OS=Spinacia oleracea;|GO:0008270,
DN109860_c	DOWN	-	-	-	-	-	-

geneid	trend	GO	Pathway	TF	NR	NT	Uniprot
1_g1_i1							
DN112388_c5_g19_i1	DOWN	-	-	-	-	-	-
DN128300_c4_g1_i1	DOWN	[GO:0016758:transferase activity, transferring hexosyl groups][GO:0008152:metabolic process]	-	-	XP_021766006.1:anthocyanidin 3Oglucoside 2"Oglucosyltransferaselike [Chenopodium quinoa]	-	A0A0K9QF01|A0A0K9QF01_SPIOL:Uncharacterized protein;OS=Spinacia oleracea;|GO:0016758,|GO:0008152,
DN122780_c0_g7_i4	DOWN	[GO:0016021:integral component of membrane][GO:0005215:transporter activity][GO:0006857:oligopeptide transport]	-	mrna00620.1-v1.0-hybrid:Fragaria vesca|BBR-BPC|BBR-BPC family protein	XP_010691376.1:PREDICTED: protein NRT1/ PTR FAMILY 1.2 [Beta vulgaris subsp. vulgaris] >KMT00859.1 hypothetical protein BVRB_9g221330 [Beta vulgaris subsp. vulgaris]	-	A0A0J8BLG2|A0A0J8BLG2_BETVU:Uncharacterized protein;OS=Beta vulgaris subsp. vulgaris.;|GO:0016021,|GO:0005215,|GO:0006857,

geneid	trend	GO	Pathway	TF	NR	NT	Uniprot
DN108568_c0_g2_i3	DOWN	[GO:0005737:cytoplasm][GO:0045548:phenylalanine ammonia-lyase activity][GO:0009800:cinnamic acid biosynthetic process][GO:0006559:L-phenylalanine catabolic process]	[ko00360:Phenylalanine metabolism][ko00940:Phenylpropanoid biosynthesis]	-	XP_010684079.1:PREDICTED: phenylalanine ammonialyase [Beta vulgaris subsp. vulgaris] >CAH17686.1 phenylalanine ammonia lyase [Beta vulgaris] >KMT06263.1 hypothetical protein BVRB_7g161880 [Beta vulgaris subsp. vulgaris]	gi|460416375|ref|XM_004253470.1|:PREDICTED: Solanum lycopersicum phenylalanine ammonia-lyase-like (LOC101253989), partial mRNA	Q5QRU8|Q5QRU8_BETVU:Phenylalanine ammonia-lyase;OS=Beta vulgaris;|GO:0005737,|GO:0045548,|GO:0009800,GO:0006559,
DN105983_c1_g1_i1	DOWN	-	-	-	-	-	-
DN118656_c3_g1_i1	DOWN	[GO:0016021:integral component of membrane][GO:0004190:aspartic-type endopeptidase activity]	-	-	XP_010666402.1:PREDICTED: LOW QUALITY PROTEIN: aspartic proteinase PCS1 [Beta vulgaris subsp. vulgaris]	-	A0A0K9RK83|A0A0K9RK83_SPIOL:Uncharacterized protein;OS=Spinacia oleracea;|GO:0016021,|GO:0004190,

geneid	trend	GO	Pathway	TF	NR	NT	Uniprot
DN128006_c11_g16_i1	DOWN	-	-	-	-	-	-
DN118725_c0_g1_i4	DOWN	-	-	-	XP_010681076.1:PREDICTED: uncharacterized protein LOC104896080 [Beta vulgaris subsp. vulgaris] >KMT08649.1 hypothetical protein BVRB_6g139260 [Beta vulgaris subsp. vulgaris]	gi|521521236|gb|GAHZ01013338.1|:TSA: Salicornia europaea Unigene13979_SeCKS transcribed RNA sequence	A0A0J8C8S9|A0A0J8C8S9_BETVU:Uncharacterized protein;OS=Beta vulgaris subsp. vulgaris.;
DN123606_c1_g2_i3	DOWN	[GO:0016021:integral component of membrane][GO:0005215:transporter activity]	-	GSMUA_Achr10P22520_001:Musa acuminata|TALE|TALE family protein	XP_010691634.1:PREDICTED: protein NRT1/ PTR FAMILY 5.6 [Beta vulgaris subsp. vulgaris] >KMT18416.1 hypothetical protein BVRB_2g025620 [Beta vulgaris subsp. vulgaris]	-	A0A0J8FRN7|A0A0J8FRN7_BETVU:Uncharacterized protein;OS=Beta vulgaris subsp. vulgaris.;|GO:0016021,|GO:0005215,

geneid	trend	GO	Pathway	TF	NR	NT	Uniprot
DN121816_c1_g1_i4	DOWN	[GO:0016021:integral component of membrane][GO:0008270:zinc ion binding]	-	Oropetium_20150105_23793A:Oropetium thomaeum|C3H|C3H family protein	XP_021745986.1:uncharacterized protein LOC110711854 [Chenopodium quinoa]	-	A0A0J8BKZ3|A0A0J8BKZ3_BETVU:Uncharacterized protein;OS=Beta vulgaris subsp. vulgaris.;|GO:0016021,|GO:0008270,
DN119101_c2_g1_i2	DOWN	-	-	-	-	-	-
DN127825_c4_g2_i4	DOWN	[GO:0016021:integral component of membrane][GO:0005524:ATP binding][GO:0016887:ATPase activity][GO:0005215:transporter activity]	-	MDP0000265619:Malus domestica|Nin-like|Nin-like family protein	XP_021858728.1:ABC transporter A family member 7like [Spinacia oleracea]	gi|356519042|ref|XR_136634.1|:PREDICTED: Glycine max ABC transporter A family member 7-like (LOC100805474), miscRNA	A0A0K9QZZ8|A0A0K9QZZ8_SPIOL:Uncharacterized protein;OS=Spinacia oleracea;|GO:0016021,|GO:0005524,GO:0016887,GO:0005215,
DN100873_c0_g3_i1	DOWN	[GO:0004013:adenosylhomocysteinase activity][GO:0051287:NAD binding][GO:0006730:one-carbon metabolic process][GO:0019510:S-adenosylhomocysteine catabolic process]	[ko00270:Cysteine and methionine metabolism]	-	XP_010551666.1:PREDICTED: adenosylhomocysteinase 1like [Tarenaya hassleriana]	gi|1724101|gb|U79766.1|MCU79766:Mesembryanthemum crystallinum S-adenosyl-L-homocystein hydrolase mRNA, complete	A0A059CBS9|A0A059CBS9_EUCGR:Adenosylhomocysteinase;OS=Eucalyptus grandis;|GO:0004013,GO:0051287,|GO:0006730,GO:001951

geneid	trend	GO	Pathway	TF	NR	NT	Uniprot
						cds	0,
DN124100_c6_g21_i1	DOWN	-	-	-	-	gi|292494383|dbj|AB434496.1|:Prunus mume ASR1 mRNA for abscisic acid stress ripening protein homolog, complete cds	-
DN119852_c0_g2_i14	DOWN	[GO:0008270:zinc ion binding]	-	KN540739.1_FGP003:Oryza longistaminata|M-type_MADS|M-type_MADS family protein	XP_021734584.1:E3 ubiquitinprotein ligase RGLG3like [Chenopodium quinoa]	gi|359477064|ref|XM_002266798.2|:PREDICTED: Vitis vinifera E3 ubiquitin-protein ligase RGLG1-like, transcript variant 1 (LOC100249896), mRNA	A0A0K9Q7A7|A0A0K9Q7A7_SPIOL:Uncharacterized protein;OS=Spinacia oleracea;|GO:0008270,

geneid	trend	GO	Pathway	TF	NR	NT	Uniprot
DN119833_c0_g1_i1	DOWN	[GO:0003676:nucleic acid binding]	-	PH01001260G0280: Phyllostachys heterocycla|bHLH|bHLH family protein	XP_020963321.1:uncharacterized protein LOC110264969 [Arachis ipaensis]	-	F4NCK1|F4NCK1_BETVU:Uncharacterized protein;OS=Beta vulgaris subsp. vulgaris.;|GO:0003676,
DN104928_c0_g1_i2	DOWN	[GO:0016021:integral component of membrane][GO:0005886:plasma membrane][GO:0005524:ATP binding][GO:0042626:ATPase activity, coupled to transmembrane movement of substances]	-	Rsa1.0_03996.1_g00001.1:Raphanus sativus|MYB_related|MYB_related family protein	XP_003546230.1:PREDICTED: pleiotropic drug resistance protein 1like [Glycine max] >KRH09797.1 hypothetical protein GLYMA_15G012000 [Glycine max]	gi|255543330|ref|XM_002512682.1|:Ricinus communis ATP-binding cassette transporter, putative, mRNA	I1MCJ9|I1MCJ9_SOYBN:Uncharacterized protein;OS=Glycine max;|GO:0016021,GO:0005886,|GO:0005524,GO:0042626,
DN116931_c3_g7_i1	DOWN	-	-	-	-	-	-

geneid	trend	GO	Pathway	TF	NR	NT	Uniprot
DN120597_c2_g1_i2	DOWN	[GO:0020037:heme binding][GO:0005506:iron ion binding][GO:0004497:monooxygenase activity][GO:0016705:oxidoreductase activity, acting on paired donors, with incorporation or reduction of molecular oxygen]	[ko00592:alpha-Linolenic acid metabolism]	-	XP_021738527.1:allene oxide synthase 2like isoform X2 [Chenopodium quinoa]	gi|225428597|ref|XM_002281123.1|:PREDICTED: Vitis vinifera allene oxide synthase, chloroplastic-like (LOC100252012), mRNA	A0A0K9QAG2|A0A0K9QAG2_SPIOL: Uncharacterized protein;OS=Spinacia oleracea;|GO:0020037,GO:0005506,GO:0004497,GO:0016705,
DN115040_c3_g4_i1	DOWN	[GO:0005524:ATP binding]	[ko00020:Citrate cycle (TCA cycle)][ko00720:Carbon fixation pathways in prokaryotes]	-	XP_021727061.1:ATPcitrate synthase alpha chain protein 1 [Chenopodium quinoa]	gi|521526383|gb|GAHZ01010262.1|:TSA: Salicornia europaea Unigene10876_SeCKS transcribed RNA sequence	A0A0K9QLK4|A0A0K9QLK4_SPIOL: Uncharacterized protein;OS=Spinacia oleracea;|GO:0005524,
DN121822_c1_g2_i2	DOWN	-	-	-	-	gi|478163487|gb|GAHK01013116.1|:TSA: Jatropha curcas Unigene568_JC-CK_1A transcribed RNA sequence	-
DN115428_c1_g1_i2	DOWN	[GO:0046872:metal ion binding][GO:0030001:metal ion transport]	-	-	XP_021727300.1:heavy metalassociated isoprenylated plant protein 20like	-	V4TM22|V4TM22_9ROSI:Uncharacterized protein;OS=Citrus

geneid	trend	GO	Pathway	TF	NR	NT	Uniprot
					[Chenopodium quinoa]		clementina.;|GO:0046872,|GO:0030001,
DN120684_c4_g1_i4	DOWN	-	-	-	XP_021734253.1:Fbox protein At2g32560like isoform X2 [Chenopodium quinoa]	gi|356554182|ref|XM_003545380.1|:PREDICTED: Glycine max F-box protein At2g32560-like (LOC100790643), mRNA	V5Q1I6|V5Q1I6_9CARY:F-box family protein;OS=Suaeda glauca.;
DN119557_c0_g9_i1	DOWN	-	-	Sp_048420_mmdm.t1:Spinacia oleracea|TCP|TCP family protein	XP_021763056.1:transcription factor CYCLOIDEAlike [Chenopodium quinoa]	-	A0A0K9RPV7|A0A0K9RPV7_SPIOL:Uncharacterized protein;OS=Spinacia oleracea;
DN127916_c0_g2_i1	DOWN	[GO:0009733:response to auxin]	[ko04075:Plant hormone signal transduction]	-	KNA12702.1:hypothetical protein SOVF_123490 [Spinacia oleracea]	-	A0A0K9R1N8|A0A0K9R1N8_SPIOL:Uncharacterized protein;OS=Spinacia oleracea;|GO:0009733,

geneid	trend	GO	Pathway	TF	NR	NT	Uniprot
DN117741_c12_g1_i1	DOWN	-	-	-	XP_021755345.1:EG45like domain containing protein [Chenopodium quinoa]	-	A0A0J8BQ32|A0A0J8BQ32_BETVU:Uncharacterized protein;OS=Beta vulgaris subsp. vulgaris.;
DN118688_c0_g1_i1	DOWN	-	-	-	-	-	-
DN116334_c0_g1_i4	DOWN	[GO:0043565:sequence-specific DNA binding][GO:0003700:DNA-binding transcription factor activity]	-	Bv2_031460_zqqw.t1:Beta vulgaris|WRKY|WRKY family protein	XP_010667511.1:PREDICTED: probable WRKY transcription factor 40 [Beta vulgaris subsp. vulgaris] >KMT18115.1 hypothetical protein BVRB_2g031460 [Beta vulgaris subsp. vulgaris]	gi|521696852|gb|GAIA01019153.1|:TSA: Salicornia europaea Unigene20113_Se200S transcribed RNA sequence	A0A0J8CWM5|A0A0J8CWM5_BETVU:Uncharacterized protein;OS=Beta vulgaris subsp. vulgaris.;|GO:0043565,GO:0003700,
DN120430_c0_g1_i2	DOWN	-	-	-	-	-	-

geneid	trend	GO	Pathway	TF	NR	NT	Uniprot
DN123949_c0_g4_i1	DOWN	[GO:0005874:microtubule]	-	KN539732.1_FGP002:Oryza longistaminata|E2F/DP|E2F/DP family protein	XP_010678655.1:PREDICTED: microtubuleassociated protein TORTIFOLIA1 [Beta vulgaris subsp. vulgaris] >KMT10475.1 hypothetical protein BVRB_5g115990 [Beta vulgaris subsp. vulgaris]	gi|359491626|ref|XM_002280564.2|:PREDICTED: Vitis vinifera microtubule-associated protein TORTIFOLIA1-like (LOC100263859), mRNA	A0A0J8C9R3|A0A0J8C9R3_BETVU:Uncharacterized protein;OS=Beta vulgaris subsp. vulgaris.;|GO:0005874,
DN121559_c3_g1_i3	DOWN	[GO:0004866:endopeptidase inhibitor activity]	-	-	XP_010683193.1:PREDICTED: kunitz trypsin inhibitor 2 [Beta vulgaris subsp. vulgaris] >KMT07072.1 hypothetical protein BVRB_6g153990 [Beta vulgaris subsp. vulgaris]	-	A0A0J8C0T9|A0A0J8C0T9_BETVU:Uncharacterized protein;OS=Beta vulgaris subsp. vulgaris.;|GO:0004866,

geneid	trend	GO	Pathway	TF	NR	NT	Uniprot
DN124641_c3_g1_i3	DOWN	[GO:0016747:transferase activity, transferring acyl groups other than amino-acyl groups]	-	Zpz_sc00135.1.g00200.1.am.mk:Zoysia pacifica|Nin-like|Nin-like family protein	XP_021766404.1:benzyl alcohol Obenzoyltransferase like [Chenopodium quinoa]	-	A0A0J8BMA6|A0A0J8BMA6_BETVU: Uncharacterized protein;OS=Beta vulgaris subsp. vulgaris.;|GO:0016747,
DN89172_c1_g1_i1	DOWN	[GO:0046872:metal ion binding][GO:0016491:oxidoreductase activity]	-	676709864:Sisymbrium irio|STAT|STAT family protein	XP_010679241.1:PREDICTED: 2'deoxymugineicacid 2'dioxygenase [Beta vulgaris subsp. vulgaris] >KMT10354.1 hypothetical protein BVRB_5g121030 isoform B [Beta vulgaris subsp. vulgaris]	-	A0A0J8CDT2|A0A0J8CDT2_BETVU:Uncharacterized protein;OS=Beta vulgaris subsp. vulgaris.;|GO:0046872,GO:0016491,

geneid	trend	GO	Pathway	TF	NR	NT	Uniprot
DN127849_c1_g4_i3	DOWN	[GO:0016021:integral component of membrane][GO:0005524:ATP binding][GO:0042626:ATPase activity, coupled to transmembrane movement of substances]	[ko02010:ABC transporters][ko04976:Bile secretion][ko05206:MicroRNAs in cancer]	Zjn_sc00086.1.g02330.1.am.mk:Zoysia japonica|ERF|ERF family protein	ASM90198.1:multidrug resistance 16 [Sesuvium portulacastrum]	gi|470103821|ref|XM_004288278.1|:PREDICTED: Fragaria vesca subsp. vesca ABC transporter B family member 4-like (LOC101311221), mRNA	A0A0J8BPC6|A0A0J8BPC6_BETVU:Uncharacterized protein;OS=Beta vulgaris subsp. vulgaris.;|GO:0016021,|GO:0005524,GO:0042626,
DN121389_c0_g1_i1	DOWN	-	-	-	-	-	-
DN114322_c0_g1_i4	DOWN	-	-	-	XP_021775279.1:uncharacterized protein LOC110739131 isoform X1 [Chenopodium quinoa]	-	A0A0K9R3E0|A0A0K9R3E0_SPIOL:Uncharacterized protein;OS=Spinacia oleracea;

geneid	trend	GO	Pathway	TF	NR	NT	Uniprot
DN122915_c2_g2_i1	DOWN	[GO:0046872:metal ion binding][GO:0016491:oxidoreductase activity]	[ko00941:Flavonoid biosynthesis]	Zpz_sc00083.1.g00290.1.am.mk:Zoysia pacifica|Dof|Dof family protein	XP_010679937.1:PREDICTED: naringenin,2oxoglutarate 3dioxygenase [Beta vulgaris subsp. vulgaris] >KMT09612.1 hypothetical protein BVRB_6g130650 [Beta vulgaris subsp. vulgaris]	gi|134039063|gb|EF468104.1|:Dimocarpus longan flavanone-3-hydroxylase (f3h) mRNA, complete cds	A0A0J8C7R1|A0A0J8C7R1_BETVU:Uncharacterized protein;OS=Beta vulgaris subsp. vulgaris.;|GO:0046872,GO:0016491,
DN126900_c1_g7_i2	DOWN	[GO:0016021:integral component of membrane]	-	-	XP_021843724.1:glucomannan 4betamannosyltransferase 2like [Spinacia oleracea] >KNA10420.1 hypothetical protein SOVF_144400 [Spinacia oleracea]	gi|512400436|gb|GAJZ01000734.1|:TSA: Prunus avium Pa_01420 transcribed RNA sequence	A0A0K9QT44|A0A0K9QT44_SPIOL:Uncharacterized protein;OS=Spinacia oleracea;|GO:0016021,
DN127558_c1_g2_i1	DOWN	-	-	-	-	-	-

geneid	trend	GO	Pathway	TF	NR	NT	Uniprot
DN77198_c0_g1_i2	DOWN	[GO:0005783:endoplasmic reticulum][GO:0018472:1-hydroxy-2-naphthaldehyde dehydrogenase activity][GO:0034525:1-naphthaldehyde dehydrogenase activity][GO:0044104:2,5-dioxovalerate dehydrogenase (NAD+) activity][GO:0018474:2-carboxybenzaldehyde dehydrogenase activity][GO:0034520:2-naphthaldehyde dehydrogenase activity][GO:0004028:3-chloroallyl aldehyde dehydrogenase activity][GO:0034538:3-methylsalicylaldehyde dehydrogenase activity][GO:0034530:4-hydroxymethylsalicyaldehyde dehydrogenase activity][GO:0019115:benzaldehyde dehydrogenase activity][GO:0018473:cis-2-methyl-5-isopropylhexa-2,5-dienal dehydrogenase activity][GO:0034822:citronellal dehydrogenase activity][GO:0080019:fatty-acyl-CoA reductase (alcohol-forming) activity][GO:0034832:geranial dehydrogenase activity][GO:0043796:glyceraldehyde dehydrogenase (NADP) activity][GO:0043878:glyceraldehyde	[ko00073:Cutin, suberine and wax biosynthesis][ko04146:Peroxisome][ko04212:Longevity regulating pathway - worm]	-	XP_021716399.1:alcoholforming fatty acylCoA reductaselike [Chenopodium quinoa]	-	A0A0J8C022|A0A0J8C022_BETVU:Fatty acyl-CoA reductase;OS=Beta vulgaris subsp. vulgaris.;|GO:0005783,|GO:0018472,GO:0034525,GO:0044104,GO:0018474,GO:0034520,GO:0004028,GO:0034538,GO:0034530,GO:0019115,GO:0018473,GO:0034822,GO:0080019,GO:0034832,GO:0043796,GO:0043878,GO:0052814,GO:0043870,GO:0043745,GO:0034602,GO:0034601,GO:0034604,GO:0018475,|GO:0006629,GO:0010025,

geneid	trend	GO	Pathway	TF	NR	NT	Uniprot
DN124168_c2_g1_i2	DOWN	[GO:0030246:carbohydrate binding][GO:0004553:hydrolase activity, hydrolyzing O-glycosyl compounds][GO:0005975:carbohydrate metabolic process]	-	mrna31843.1-v1.0-hybrid:Fragaria vesca|WRKY|WRKY family protein	XP_021601989.1:endoglucanase 6like [Manihot esculenta] >OAY22429.1 hypothetical protein MANES_S001800 [Manihot esculenta]	gi|254031736|gb|GQ261871.1|:Dimocarpus longan endo-1,4-beta-glucanase (EG1) mRNA, complete cds	A0A199UCP3|A0A199UCP3_MANES: Endoglucanase;OS=Manihot esculenta;|GO:0030246,GO:0004553,|GO:0005975,
DN109841_c0_g1_i2	DOWN	[GO:0003824:catalytic activity][GO:0008152:metabolic process]	-	-	XP_021760635.1:petal death proteinlike [Chenopodium quinoa]	-	A0A0K9QKY2|A0A0K9QKY2_SPIOL: Uncharacterized protein;OS=Spinacia oleracea;|GO:0003824,|GO:0008152,
DN123873_c0_g2_i4	DOWN	[GO:0005623:cell][GO:0016671:oxidoreductase activity, acting on a sulfur group of donors, disulfide as acceptor][GO:0045454:cell redox homeostasis][GO:0019419:sulfate reduction]	[ko00920:Sulfur metabolism]	-	XP_021766865.1:5'adenylylsulfate reductase 1, chloroplasticlike [Chenopodium quinoa] >XP_021736490.1 5'adenylylsulfate reductase 1, chloroplasticlike [Chenopodium quinoa]	gi|521563730|gb|GAIA01074717.1|:TSA: Salicornia europaea Unigene90653_Se200S transcribed RNA sequence	A0A0K9QUF8|A0A0K9QUF8_SPIOL: Uncharacterized protein;OS=Spinacia oleracea;|GO:0005623,|GO:0016671,|GO:0045454,GO:0019419,

geneid	trend	GO	Pathway	TF	NR	NT	Uniprot
DN121526_c0_g2_i21	DOWN	-	[ko04144:Endocytosis]	OPUNC06G25210.3:Oryza punctata|bZIP|bZIP family protein	XP_021775512.1:protein EARLYRESPONSIVE TO DEHYDRATION 7, chloroplasticlike [Chenopodium quinoa]	-	A0A0K9RDR8|A0A0K9RDR8_SPIOL: Uncharacterized protein;OS=Spinacia oleracea;
DN122216_c2_g1_i4	DOWN	-	-	-	-	-	-
DN124813_c6_g4_i2	DOWN	[GO:0016021:integral component of membrane][GO:0005215:transporter activity]	-	ONIVA01G08700.1:Oryza nivara|BES1|BES1 family protein	XP_010680950.1:PREDICTED: probable aquaporin NIP51 [Beta vulgaris subsp. vulgaris] >KMT08535.1 hypothetical protein BVRB_6g138160 [Beta vulgaris subsp. vulgaris]	-	A0A0J8C433|A0A0J8C433_BETVU:Uncharacterized protein;OS=Beta vulgaris subsp. vulgaris.;|GO:0016021,|GO:0005215,
DN127192_c0_g1_i1	DOWN	-	-	-	-	-	-

geneid	trend	GO	Pathway	TF	NR	NT	Uniprot
DN120736_c1_g1_i4	DOWN	-	-	Bv9_222310_qpjr.t1:Beta vulgaris|bHLH|bHLH family protein	XP_010691492.1:PREDICTED: transcription factor bHLH25 [Beta vulgaris subsp. vulgaris] >KMT00966.1 hypothetical protein BVRB_9g222310 [Beta vulgaris subsp. vulgaris]	-	A0A0J8ED99|A0A0J8ED99_BETVU:Uncharacterized protein;OS=Beta vulgaris subsp. vulgaris.;
DN117292_c3_g2_i2	DOWN	-	-	-	-	-	-
DN121619_c1_g4_i2	DOWN	[GO:0048046:apoplast][GO:0005507:copper ion binding][GO:0052716:hydroquinone:oxygen oxidoreductase activity][GO:0046274:lignin catabolic process]	-	Rsa1.0_03034.1_g00001.1:Raphanus sativus|ERF|ERF family protein	XP_010685719.1:PREDICTED: laccase17 [Beta vulgaris subsp. vulgaris] >KMT05208.1 hypothetical protein BVRB_7g173530 [Beta vulgaris subsp. vulgaris]	-	A0A0J8BZ29|A0A0J8BZ29_BETVU:Laccase;OS=Beta vulgaris subsp. vulgaris.;|GO:0048046,|GO:0005507,GO:0052716,|GO:0046274,

geneid	trend	GO	Pathway	TF	NR	NT	Uniprot
DN119346_c0_g1_i3	DOWN	[GO:0004126:cytidine deaminase activity][GO:0008270:zinc ion binding]	[ko00240:Pyrimidine metabolism][ko00983:Drug metabolism - other enzymes]	-	XP_010667333.1:PREDICTED: cytidine deaminase 1 [Beta vulgaris subsp. vulgaris] >KMS95458.1 hypothetical protein BVRB_008100 [Beta vulgaris subsp. vulgaris]	-	A0A0J8B6D3|A0A0J8B6D3_BETVU:Cytidine deaminase;OS=Beta vulgaris subsp. vulgaris.;|GO:0004126,GO:0008270,
DN124100_c6_g25_i1	DOWN	-	-	-	-	gi|418731483|gb|JX839758.1|:Solanum tuberosum clone StDT188 abscisic stress ripening protein 4 mRNA, partial cds	-
DN105826_c0_g1_i1	DOWN	[GO:0008168:methyltransferase activity]	-	EMT20798:Aegilops tauschii|MYB_related|MYB_related family protein	KNA17621.1:hypothetical protein SOVF_078430 [Spinacia oleracea]	-	A0A0K9RDN0|A0A0K9RDN0_SPIOL:Uncharacterized protein;OS=Spinacia oleracea;|GO:0008168,

geneid	trend	GO	Pathway	TF	NR	NT	Uniprot
DN128383_c2_g2_i11	DOWN	-	-	-	XP_010677354.1:PREDICTED: uncharacterized protein LOC104893015 [Beta vulgaris subsp. vulgaris] >XP_010677355.1 PREDICTED: uncharacterized protein LOC104893015 [Beta vulgaris subsp. vulgaris] >KMT11354.1 hypothetical protein BVRB_5g106870 [Beta vulgaris subsp. vulgaris]	-	A0A0J8CGX8|A0A0J8CGX8_BETVU: Uncharacterized protein;OS=Beta vulgaris subsp. vulgaris.;

geneid	trend	GO	Pathway	TF	NR	NT	Uniprot
DN123180_c3_g2_i2	DOWN	[GO:0005634:nucleus][GO:2000022:regulation of jasmonic acid mediated signaling pathway][GO:0009753:response to jasmonic acid][GO:0009611:response to wounding]	[ko04075:Plant hormone signal transduction]	-	XP_010682667.1:PREDICTED: protein TIFY 9 [Beta vulgaris subsp. vulgaris] >KMT07363.1 hypothetical protein BVRB_6g149890 [Beta vulgaris subsp. vulgaris]	gi|521470503|gb|GAHZ01060382.1|:TSA: Salicornia europaea Unigene72573_SeCKS transcribed RNA sequence	A0A0J8C5M1|A0A0J8C5M1_BETVU: Uncharacterized protein;OS=Beta vulgaris subsp. vulgaris.;|GO:0005634,|GO:2000022,GO:0009753,GO:0009611,
DN121806_c0_g2_i1	DOWN	[GO:0008234:cysteine-type peptidase activity]	-	FANhyb_rscf00000253.1.g00010.1:Fragaria x ananassa|BBR-BPC|BBR-BPC family protein	AHA62778.1:RCR3like cysteine protease [Mirabilis jalapa]	-	V5M3B7|V5M3B7_MIRJA:RCR3-like cysteine protease;OS=Mirabilis jalapa;|GO:0008234,

geneid	trend	GO	Pathway	TF	NR	NT	Uniprot
DN128914_c1_g1_i16	DOWN	[GO:0016021:integral component of membrane]	-	-	XP_021852833.1:probable leucinerich repeat receptorlike protein kinase At2g33170 [Spinacia oleracea] >XP_021840185.1 probable leucinerich repeat receptorlike protein kinase At2g33170 [Spinacia oleracea] >KNA24771.1 hypothetical protein SOVF_012540 [Spinacia oleracea]	-	A0A0K9RZ29|A0A0K9RZ29_SPIOL:Uncharacterized protein;OS=Spinacia oleracea;|GO:0016021,
DN111573_c0_g1_i2	DOWN	[GO:0004108:citrate (Si)-synthase activity][GO:0006101:citrate metabolic process][GO:0006099:tricarboxylic acid cycle]	-	-	XP_021859149.1:uncharacterized protein At1g15400like [Spinacia oleracea]	-	A0A0J8BI86|A0A0J8BI86_BETVU:Citrate synthase;OS=Beta vulgaris subsp. vulgaris.;|GO:0004108,|GO:0006101,GO:0006099,

geneid	trend	GO	Pathway	TF	NR	NT	Uniprot
DN119029_c3_g12_i1	DOWN	-	-	-	-	-	-
DN105724_c0_g1_i2	DOWN	-	-	-	-	-	-
DN121234_c2_g1_i1	DOWN	[GO:0045430:chalcone isomerase activity][GO:0009813:flavonoid biosynthetic process]	[ko00941:Flavonoid biosynthesis]	Csa07g061600.1:Camelina sativa|NAC|NAC family protein	XP_021755848.1:chalconeflavonone isomeraselike isoform X1 [Chenopodium quinoa]	-	A0A0K9QY08|A0A0K9QY08_SPIOL:Chalcone-flavonone isomerase family protein;OS=Spinacia oleracea;|GO:0045430,|GO:0009813,
DN105333_c0_g1_i1	DOWN	[GO:0048046:apoplast][GO:0005622:intracellular][GO:0016491:oxidoreductase activity][GO:0005093:Rab GDP-dissociation inhibitor activity][GO:0015031:protein transport][GO:0007264:small GTPase mediated signal transduction]	-	Sme2.5_02464.1_g00004.1:Solanum melongena|MIKC_MADS|MIKC_MADS family protein	XP_010691404.1:PREDICTED: guanosine nucleotide diphosphate dissociation inhibitor At5g09550 [Beta vulgaris subsp. vulgaris] >KMT00881.1 hypothetical protein BVRB_9g221540 [Beta vulgaris subsp. vulgaris]	-	A0A0J8ECZ7|A0A0J8ECZ7_BETVU:Uncharacterized protein;OS=Beta vulgaris subsp. vulgaris.;|GO:0048046,GO:0005622,|GO:0016491,GO:0005093,|GO:0015031,GO:0007264,

geneid	trend	GO	Pathway	TF	NR	NT	Uniprot
DN114581_c1_g1_i1	DOWN	[GO:0003677:DNA binding]	-	Lsa007623:Lactuca sativa|MYB|MYB family protein	XP_021768829.1:transcription factor MYB14like [Chenopodium quinoa]	gi|145360506|ref|NM_128674.3|:Arabidopsis thaliana myb domain protein 14 mRNA, complete cds	A0A078JZ82|A0A078JZ82_BRANA:BnaAnng35730D protein;OS=Brassica napus;|GO:0003677,
DN120965_c3_g6_i8	DOWN	[GO:0016021:integral component of membrane]	-	Csa09g092790.1:Camelina sativa|BES1|BES1 family protein	XP_021855818.1:protein trichome birefringencelike 33 [Spinacia oleracea] >KNA05048.1 hypothetical protein SOVF_193950 [Spinacia oleracea]	gi|359486372|ref|XM_002277348.2|:PREDICTED: Vitis vinifera uncharacterized LOC100262072 (LOC100262072), mRNA	A0A0K9QEC4|A0A0K9QEC4_SPIOL: Uncharacterized protein;OS=Spinacia oleracea;|GO:0016021,
DN128780_c2_g6_i2	DOWN	[GO:0004568:chitinase activity][GO:0005975:carbohydrate metabolic process][GO:0016998:cell wall macromolecule catabolic process][GO:0006032:chitin catabolic process]	[ko00520:Amino sugar and nucleotide sugar metabolism]	-	OIV98483.1:hypothetical protein TanjilG_18767 [Lupinus angustifolius]	-	Q7M1Q9|Q7M1Q9_PHYAM:Chitinase A;OS=Phytolacca americana;|GO:0004568,|GO:0005975,GO:0016998,GO:0006032,

geneid	trend	GO	Pathway	TF	NR	NT	Uniprot
DN125117_c2_g2_i9	DOWN	[GO:0016021:integral component of membrane][GO:0015098:molybdate ion transmembrane transporter activity]	-	-	XP_021854545.1:molybdate transporter 1 [Spinacia oleracea] >KNA05899.1 hypothetical protein SOVF_186100 [Spinacia oleracea]	-	A0A0K9QF43|A0A0K9QF43_SPIOL:Uncharacterized protein;OS=Spinacia oleracea;|GO:0016021,|GO:0015098,
DN117527_c0_g4_i3	DOWN	[GO:0016021:integral component of membrane][GO:0008270:zinc ion binding]	-	-	XP_021745986.1:uncharacterized protein LOC110711854 [Chenopodium quinoa]	-	A0A0J8BKZ3|A0A0J8BKZ3_BETVU:Uncharacterized protein;OS=Beta vulgaris subsp. vulgaris.;|GO:0016021,|GO:0008270,
DN126737_c3_g4_i1	DOWN	-	-	Zmw_sc01994.1.g00160.1:Zoysia matrella|ZF-HD|ZF-HD family protein	XP_012090061.1:protein GAST1 [Jatropha curcas] >KDP22135.1 hypothetical protein JCGZ_25966 [Jatropha curcas]	gi|478162735|gb|GAHK01013868.1|:TSA: Jatropha curcas Unigene1321_JC-CK_1A transcribed RNA sequence	M5WL91|M5WL91_PRUPE:Uncharacterized protein;OS=Prunus persica;

geneid	trend	GO	Pathway	TF	NR	NT	Uniprot
DN115455_c0_g1_i2	DOWN	[GO:0016747:transferase activity, transferring acyl groups other than amino-acyl groups]	-	-	XP_021759935.1:fatty alcohol:caffeoylCoA acyltransferaselike [Chenopodium quinoa]	-	A0A0J8D8N6|A0A0J8D8N6_BETVU:Uncharacterized protein;OS=Beta vulgaris subsp. vulgaris.;|GO:0016747,
DN119223_c1_g3_i3	DOWN	-	-	-	-	-	-
DN126900_c1_g7_i1	DOWN	[GO:0016021:integral component of membrane]	-	-	XP_021843724.1:glucomannan 4betamannosyltransferase 2like [Spinacia oleracea] >KNA10420.1 hypothetical protein SOVF_144400 [Spinacia oleracea]	gi|512400436|gb|GAJZ01000734.1|:TSA: Prunus avium Pa_01420 transcribed RNA sequence	A0A0K9QT44|A0A0K9QT44_SPIOL:Uncharacterized protein;OS=Spinacia oleracea;|GO:0016021,

geneid	trend	GO	Pathway	TF	NR	NT	Uniprot
DN105541_c0_g1_i1	DOWN	[GO:0005634:nucleus][GO:0043565:sequence-specific DNA binding][GO:0006355:regulation of transcription, DNA-templated]	-	Bv8_182430_wwwi.t1:Beta vulgaris|HD-ZIP|HD-ZIP family protein	XP_010686481.1:PREDICTED: homeoboxleucine zipper protein ATHB52 [Beta vulgaris subsp. vulgaris] >KMT04587.1 hypothetical protein BVRB_8g182430 [Beta vulgaris subsp. vulgaris]	-	A0A0J8BTX8|A0A0J8BTX8_BETVU:Uncharacterized protein;OS=Beta vulgaris subsp. vulgaris.;|GO:0005634,|GO:0043565,|GO:0006355,
DN121526_c0_g2_i9	DOWN	-	[ko04144:Endocytosis]	OPUNC06G25210.3:Oryza punctata|bZIP|bZIP family protein	XP_021775512.1:protein EARLYRESPONSIVE TO DEHYDRATION 7, chloroplasticlike [Chenopodium quinoa]	-	A0A0K9RDR8|A0A0K9RDR8_SPIOL:Uncharacterized protein;OS=Spinacia oleracea;

geneid	trend	GO	Pathway	TF	NR	NT	Uniprot
DN125668_c0_g2_i10	DOWN	[GO:0016021:integral component of membrane][GO:0004310:farnesyl-diphosphate farnesyltransferase activity][GO:0051996:squalene synthase activity][GO:0006696:ergosterol biosynthetic process]	[ko00100:Steroid biosynthesis][ko00909:Sesquiterpenoid and triterpenoid biosynthesis]	-	ABQ96265.1:squalene synthase [Psammosilene tunicoides]	gi|478161531|gb|GAHK01015072.1|:TSA: Jatropha curcas Unigene2526_JC-CK_1A transcribed RNA sequence	A5YT92|A5YT92_9CARY:Squalene synthase;OS=Psammosilene tunicoides.;|GO:0016021,|GO:0004310,GO:0051996,|GO:0006696,
DN125811_c7_g13_i1	DOWN	-	-	-	-	-	-

geneid	trend	GO	Pathway	TF	NR	NT	Uniprot
DN118019_c0_g1_i7	DOWN	[GO:0016021:integral component of membrane][GO:0055085:transmembrane transport]	-	-	XP_010683591.1:PREDICTED: mitochondrial thiamine pyrophosphate carrierlike isoform X1 [Beta vulgaris subsp. vulgaris] >XP_010683592.1 PREDICTED: mitochondrial thiamine pyrophosphate carrierlike isoform X1 [Beta vulgaris subsp. vulgaris] >XP_019106267.1 PREDICTED: mitochondrial thiamine pyrophosphate carrierlike isoform X1 [Beta vulgaris subsp. vulgaris]	gi|521717832|gb|GAIA01011880.1|:TSA: Salicornia europaea Unigene12498_Se200S transcribed RNA sequence	A0A0K9S0N1|A0A0K9S0N1_SPIOL:Uncharacterized protein;OS=Spinacia oleracea;|GO:0016021,|GO:0055085,

geneid	trend	GO	Pathway	TF	NR	NT	Uniprot
DN125219_c1_g1_i5	DOWN	-	[ko00190:Oxidative phosphorylation]	Lus10040166:Linum usitatissimum|GRAS|GRAS family protein	ONM12759.1:H(+) ATPase 5 [Zea mays]	gi|411107727|gb|JX628604.1|:Sesuvium portulacastrum plasma membrane H+-ATPase mRNA, complete cds	A0A1D6RJI1|A0A1D6RJI1_WHEAT:Uncharacterized protein;OS=Triticum aestivum;
DN128881_c3_g1_i11	DOWN	[GO:0016021:integral component of membrane][GO:0008270:zinc ion binding]	-	Oropetium_20150105_23793A:Oropetium thomaeum|C3H|C3H family protein	XP_021745986.1:uncharacterized protein LOC110711854 [Chenopodium quinoa]	-	A0A0J8BKZ3|A0A0J8BKZ3_BETVU:Uncharacterized protein;OS=Beta vulgaris subsp. vulgaris.;|GO:0016021,|GO:0008270,
DN121436_c3_g13_i3	DOWN	[GO:0003676:nucleic acid binding][GO:0008270:zinc ion binding][GO:0015074:DNA integration]	-	676783392:Sisymbrium irio|B3|B3 family protein	XP_017415202.1:PREDICTED: retrovirusrelated Pol polyprotein from transposon TNT 194 isoform X1 [Vigna angularis]	-	A5BCC7|A5BCC7_VITVI:Putative uncharacterized protein;OS=Vitis vinifera;|GO:0003676,GO:0008270,|GO:0015074,

geneid	trend	GO	Pathway	TF	NR	NT	Uniprot
DN116020_c4_g2_i1	DOWN	[GO:0005856:cytoskeleton]	[ko04013:MAPK signaling pathway - fly][ko04015:Rap1 signaling pathway][ko04810: Regulation of actin cytoskeleton][ko05131:Shigellosis][ko05132:Salmonella infection]	-	XP_019422928.1:PREDICTED: profilin4 [Lupinus angustifolius] >OIV93398.1 hypothetical protein TanjilG_02935 [Lupinus angustifolius]	-	A0A068U433|A0A068U433_COFCA:Profilin;OS=Coffea canephora;|GO:0005856,
DN116583_c1_g3_i1	DOWN	[GO:0019139:cytokinin dehydrogenase activity][GO:0050660:flavin adenine dinucleotide binding][GO:0016614:oxidoreductase activity, acting on CH-OH group of donors][GO:0009690:cytokinin metabolic process]	[ko00908:Zeatin biosynthesis]	PGSC0003DMP400000652:Solanum tuberosum|bHLH|bHLH family protein	XP_021759904.1:cytokinin dehydrogenase 5like [Chenopodium quinoa]	gi|521735182|gb|GAIA01009318.1|:TSA: Salicornia europaea Unigene9683_Se200S transcribed RNA sequence	A0A0J8D8K7|A0A0J8D8K7_BETVU:Uncharacterized protein;OS=Beta vulgaris subsp. vulgaris.;|GO:0019139,GO:0050660,GO:0016614,|GO:0009690,

geneid	trend	GO	Pathway	TF	NR	NT	Uniprot
DN110960_c0_g2_i3	DOWN	-	-	-	KNA24654.1:hypothetical protein SOVF_013430 [Spinacia oleracea]	gi|449450583|ref|XM_004142994.1|:PREDICTED: Cucumis sativus F-box protein At1g67340-like (LOC101205201), mRNA >gi|449494546|ref|XM_004159529.1| PREDICTED: Cucumis sativus F-box protein At1g67340-like (LOC101229302), mRNA	A0A0K9S0F6|A0A0K9S0F6_SPIOL:Uncharacterized protein;OS=Spinacia oleracea;
DN122392_c2_g2_i1	DOWN	[GO:0016787:hydrolase activity]	[ko04310:Wnt signaling pathway]	Ote100112080011:Ocimum tenuiflorum|bHLH|bHLH family protein	XP_010677538.1:PREDICTED: pectin acetylesterase 8 isoform X1 [Beta vulgaris subsp. vulgaris] >KMT11532.1 hypothetical protein BVRB_5g108510	-	A0A0J8CD75|A0A0J8CD75_BETVU:Uncharacterized protein;OS=Beta vulgaris subsp. vulgaris.;|GO:0016787,

geneid	trend	GO	Pathway	TF	NR	NT	Uniprot
					[Beta vulgaris subsp. vulgaris]		
DN122899_c1_g3_i5	DOWN	[GO:0005783:endoplasmic reticulum][GO:0016020:membrane][GO:0016831:carboxy-lyase activity][GO:0030170:pyridoxal phosphate binding][GO:0019752:carboxylic acid metabolic process][GO:0030149:sphingolipid catabolic process]	[ko00600:Sphingolipid metabolism][ko04071:Sphingolipid signaling pathway]	-	XP_010682718.1:PREDICTED: sphingosine1phosphate lyase [Beta vulgaris subsp. vulgaris] >KMT07419.1 hypothetical protein BVRB_6g150440 [Beta vulgaris subsp. vulgaris]	gi|224075540|ref|XM_002304638.1|:Populus trichocarpa predicted protein, mRNA	A0A0J8C5A3|A0A0J8C5A3_BETVU:Uncharacterized protein;OS=Beta vulgaris subsp. vulgaris.;|GO:0005783,GO:0016020,|GO:0016831,GO:0030170,|GO:0019752,GO:0030149,
DN121733_c2_g3_i8	DOWN	[GO:0005524:ATP binding][GO:0004672:protein kinase activity]	-	FANhyb_rscf00000217.1.g00006.1:Fragaria x ananassa|WRKY|WRKY family protein	XP_021716532.1:probable serine/threonineprotein kinase PBL12 [Chenopodium	-	A0A0K9S4F5|A0A0K9S4F5_SPIOL:Uncharacterized protein;OS=Spinacia oleracea;|GO:00055

geneid	trend	GO	Pathway	TF	NR	NT	Uniprot
					quinoa]		24,GO:0004672,
DN119965_c 2_g2_i1	DOWN	[GO:0009507:chloroplast][GO:0005524:ATP binding][GO:0008878:glucose-1-phosphate adenylyltransferase activity][GO:0005978:glycogen biosynthetic process][GO:0019252:starch biosynthetic process]	[ko00500:Starch and sucrose metabolism][ko00520:Amino sugar and nucleotide sugar metabolism][ko02026:Biofilm formation - Escherichia coli]	DCAR_013815:Daucus carota|ERF|ERF family protein	XP_010689353.1:PREDICTED: glucose1phosphate adenylyltransferase small subunit, chloroplastic/amyloplastic [Beta vulgaris subsp. vulgaris] >KMT02178.1 hypothetical protein BVRB_9g206950 [Beta vulgaris subsp. vulgaris]	gi|89089751|gb|DQ399915.1|:Nicotiana tabacum ADP-glucose pyrophosphorylase small subunit (AGP) mRNA, complete cds	A0A0J8BQG2|A0A0J8BQG2_BETVU: Glucose-1-phosphate adenylyltransferase; OS=Beta vulgaris subsp. vulgaris.;|GO:0009507,|GO:0005524,GO:0008878,|GO:0005978,GO:0019252,
DN115389_c 0_g1_i1	DOWN	-	-	-	XP_010690293.1:PREDICTED: Fbox protein SKIP23 [Beta vulgaris subsp. vulgaris] >KMT01313.1 hypothetical protein	-	A0A0J8BMR1|A0A0J8BMR1_BETVU: Uncharacterized protein;OS=Beta vulgaris subsp. vulgaris.;

geneid	trend	GO	Pathway	TF	NR	NT	Uniprot
					BVRB_9g213290 [Beta vulgaris subsp. vulgaris]		
DN101592_c0_g2_i2	DOWN	[GO:0003987:acetate-CoA ligase activity][GO:0016208:AMP binding][GO:0019427:acetyl-CoA biosynthetic process from acetate]	[ko00010:Glycolysis / Gluconeogenesis][ko00620:Pyruvate metabolism][ko00640:Propanoate metabolism][ko00680:Methane metabolism][ko00720:Carbon fixation pathways in prokaryotes]	-	XP_009403818.1:PREDICTED: acetylcoenzyme A synthetase, chloroplastic/glyoxysomallike [Musa acuminata subsp. malaccensis]	gi|356560403|ref|XM_003548434.1|:PREDICTED: Glycine max acetyl-coenzyme A synthetase-like (LOC100789257), mRNA	M0T581|M0T581_MUSAM:Uncharacterized protein;OS=Musa acuminata subsp. malaccensis;|GO:0003987,GO:0016208,|GO:0019427,
DN123943_c1_g2_i3	DOWN	-	-	-	-	-	-
DN120084_c3_g3_i5	DOWN	-	-	-	-	-	-

geneid	trend	GO	Pathway	TF	NR	NT	Uniprot
DN122974_c7_g13_i1	DOWN	[GO:0045735:nutrient reservoir activity]	-	-	XP_010678486.1:PREDICTED: uncharacterized protein LOC104894026 [Beta vulgaris subsp. vulgaris] >KMT10786.1 hypothetical protein BVRB_5g114420 [Beta vulgaris subsp. vulgaris]	-	A0A0J8F4N5|A0A0J8F4N5_BETVU:Uncharacterized protein;OS=Beta vulgaris subsp. vulgaris.;|GO:0045735,
DN122081_c1_g1_i3	DOWN	-	-	KFK43895.1:Arabis alpina|STAT|STAT family protein	XP_010679487.1:PREDICTED: uncharacterized protein LOC104894841 [Beta vulgaris subsp. vulgaris] >KMT10059.1 hypothetical protein BVRB_5g122690 [Beta vulgaris subsp. vulgaris]	gi|521526499|gb|GAHZ01010146.1|:TSA: Salicornia europaea Unigene10745_SeCKS transcribed RNA sequence	A0A0K9QZW9|A0A0K9QZW9_SPIOL:Uncharacterized protein;OS=Spinacia oleracea;

geneid	trend	GO	Pathway	TF	NR	NT	Uniprot
DN112063_c0_g1_i1	DOWN	-	-	-	XP_021719503.1:uncharacterized protein LOC110687199 [Chenopodium quinoa]	-	A0A0J8B6Y4|A0A0J8B6Y4_BETVU:Uncharacterized protein;OS=Beta vulgaris subsp. vulgaris.;
DN125481_c0_g2_i2	DOWN	[GO:0016021:integral component of membrane][GO:0005524:ATP binding][GO:0004672:protein kinase activity]	[ko04064:NF-kappa B signaling pathway][ko04620:Toll-like receptor signaling pathway][ko04621:NOD-like receptor signaling pathway][ko04624:Toll and Imd signaling pathway][ko04722:Neurotrophin signaling pathway][ko05133:Pertussis][ko05140:Leishmaniasis][ko05142:Chagas disease (American	Kaladp0839s0004.1.p:Kalanchoe laxiflora|ERF|ERF family protein	XP_010686904.1:PREDICTED: putative receptorlike protein kinase At4g00960 [Beta vulgaris subsp. vulgaris]	gi|521518144|gb|GAHZ01014966.1|:TSA: Salicornia europaea Unigene15646_SeCKS transcribed RNA sequence	G7IE17|G7IE17_MEDTR:Cysteine-rich receptor-kinase-like protein;OS=Medicago truncatula;GN=MTR_1g104890 {ECO:0000313|EMBL:AES62686.1};|GO:0016021,|GO:0005524,GO:0004672,

geneid	trend	GO	Pathway	TF	NR	NT	Uniprot
			trypanosomiasis)][ko05145:Toxoplasmosis][ko05152:Tuberculosis][ko05162:Measles][ko05164:Influenza A]				
DN128721_c1_g2_i6	DOWN	[GO:0031011:Ino80 complex][GO:0006281:DNA repair][GO:0006355:regulation of transcription, DNA-templated]	-	-	XP_010322076.1:PREDICTED: actinrelated protein 5 isoform X2 [Solanum lycopersicum]	-	K4C570|K4C570_SOLLC:Uncharacterized protein;OS=Solanum lycopersicum;|GO:0031011,|GO:0006281,GO:0006355,

geneid	trend	GO	Pathway	TF	NR	NT	Uniprot
DN125458_c6_g2_i1	DOWN	[GO:0016021:integral component of membrane]	-	676746044:Sisymbrium irio|MIKC_MADS|MIKC_MADS family protein	OVA16898.1:GDPfucose protein Ofucosyltransferase [Macleaya cordata]	gi|224120707|ref|XM_002330896.1|:Populus trichocarpa predicted protein, mRNA	M0SDI3|M0SDI3_MUSAM:Uncharacterized protein;OS=Musa acuminata subsp. malaccensis;|GO:0016021,
DN117474_c0_g3_i17	DOWN	[GO:0016021:integral component of membrane][GO:0005524:ATP binding][GO:0004672:protein kinase activity]	-	SMil_00011498-RA_Salv:Salvia miltiorrhiza|bHLH|bHLH family protein	XP_021864481.1:probable LRR receptorlike serine/threonineprotein kinase At1g56140 isoform X1 [Spinacia oleracea]	gi|521493522|gb|GAHZ01037398.1|:TSA: Salicornia europaea Unigene42165_SeCKS transcribed RNA sequence	A0A0J8C0I1|A0A0J8C0I1_BETVU:Uncharacterized protein;OS=Beta vulgaris subsp. vulgaris.;|GO:0016021,|GO:0005524,GO:0004672,
DN120639_c0_g2_i14	DOWN	[GO:0016021:integral component of membrane]	-	mrna32536.1-v1.0-hybrid:Fragaria vesca|bHLH|bHLH family protein	XP_021760455.1:embryogenesisassociated protein EMB8like [Chenopodium quinoa]	gi|521448119|gb|GAHZ01072365.1|:TSA: Salicornia europaea Unigene88104_SeCKS transcribed RNA sequence	A0A0J8FWM4|A0A0J8FWM4_BETVU:Uncharacterized protein;OS=Beta vulgaris subsp. vulgaris.;|GO:0016021,
DN112388_c5_g25_i1	DOWN	-	-	-	-	-	-

geneid	trend	GO	Pathway	TF	NR	NT	Uniprot
DN122915_c2_g2_i2	DOWN	[GO:0046872:metal ion binding][GO:0016491:oxidoreductase activity]	[ko00941:Flavonoid biosynthesis]	676709864:Sisymbrium irio|STAT|STAT family protein	XP_010679937.1:PREDICTED: naringenin,2oxoglutarate 3dioxygenase [Beta vulgaris subsp. vulgaris] >KMT09612.1 hypothetical protein BVRB_6g130650 [Beta vulgaris subsp. vulgaris]	gi|134039063|gb|EF468104.1|:Dimocarpus longan flavanone-3-hydroxylase (f3h) mRNA, complete cds	A0A0J8C7R1|A0A0J8C7R1_BETVU:Uncharacterized protein;OS=Beta vulgaris subsp. vulgaris.;|GO:0046872,GO:0016491,
DN119193_c4_g1_i3	DOWN	[GO:0016021:integral component of membrane]	-	-	XP_021774155.1:GABA transporter 1like [Chenopodium quinoa] >XP_021774157.1 GABA transporter 1like [Chenopodium quinoa]	gi|470115733|ref|XM_004293999.1|:PREDICTED: Fragaria vesca subsp. vesca GABA transporter 1-like (LOC101303198), mRNA	A0A0J8BL69|A0A0J8BL69_BETVU:Uncharacterized protein;OS=Beta vulgaris subsp. vulgaris.;|GO:0016021,
DN125731_c4_g1_i1	DOWN	-	-	-	-	-	-
DN122280_c1_g3_i5	DOWN	-	-	-	-	-	-

geneid	trend	GO	Pathway	TF	NR	NT	Uniprot
DN98842_c0_g2_i1	DOWN	-	-	Zmw_sc01994.1.g00160.1:Zoysia matrella|ZF-HD|ZF-HD family protein	XP_021744520.1:protein GAST1like [Chenopodium quinoa]	gi|357518798|ref|XM_003629640.1|:Medicago truncatula Gibberellin induced protein (MTR_8g085470) mRNA, complete cds	A0A0J8CN04|A0A0J8CN04_BETVU:Uncharacterized protein;OS=Beta vulgaris subsp. vulgaris.;
DN118954_c0_g4_i4	DOWN	[GO:0009507:chloroplast][GO:0004851:uroporphyrin-III C-methyltransferase activity][GO:0055114:oxidation-reduction process][GO:0019354:siroheme biosynthetic process]	-	-	XP_010667452.1:PREDICTED: uncharacterized protein LOC104884494 [Beta vulgaris subsp. vulgaris] >KMS95363.1 hypothetical protein BVRB_008870 [Beta vulgaris subsp. vulgaris]	gi|521742619|gb|GAIA01004858.1|:TSA: Salicornia europaea Unigene5144_Se200S transcribed RNA sequence	A0A0J8B653|A0A0J8B653_BETVU:Uncharacterized protein;OS=Beta vulgaris subsp. vulgaris.;|GO:0009507,|GO:0004851,|GO:0055114,GO:0019354,
DN123152_c0_g1_i3	DOWN	[GO:0008234:cysteine-type peptidase activity]	-	FANhyb_rscf00000253.1.g00010.1:Fragaria x ananassa|BBR-BPC|BBR-BPC family	AHA62778.1:RCR3like cysteine protease [Mirabilis jalapa]	-	V5M3B7|V5M3B7_MIRJA:RCR3-like cysteine protease;OS=Mirabilis

geneid	trend	GO	Pathway	TF	NR	NT	Uniprot
				protein			jalapa;|GO:0008234,
DN125052_c0_g1_i1	DOWN	-	-	Cotton_A_16694_BGI-A2_v1.0:Gossypium arboreum|bHLH|bHLH family protein	XP_010680350.1:PREDICTED: protein NETWORKED 2D [Beta vulgaris subsp. vulgaris] >KMT09275.1 hypothetical protein BVRB_6g133750 [Beta vulgaris subsp. vulgaris]	gi|359496915|ref|XM_003635322.1|:PREDICTED: Vitis vinifera uncharacterized LOC100853146 (LOC100853146), mRNA	A0A0J8C698|A0A0J8C698_BETVU:Uncharacterized protein;OS=Beta vulgaris subsp. vulgaris.;
DN119809_c0_g3_i3	DOWN	[GO:0003676:nucleic acid binding][GO:0008270:zinc ion binding][GO:0015074:DNA integration]	-	Sopen05g016700.1: Solanum pennellii|ZF-HD|ZF-HD family protein	XP_020272202.1:LOW QUALITY PROTEIN: uncharacterized protein LOC109847382 [Asparagus officinalis]	-	E2DN01|E2DN01_BETVU:Putative uncharacterized protein;OS=Beta vulgaris;|GO:0003676,GO:0008270,|GO:0015074,

geneid	trend	GO	Pathway	TF	NR	NT	Uniprot
DN116315_c1_g1_i1	DOWN	[GO:0005737:cytoplasm][GO:0005874:microtubule][GO:0005525:GTP binding][GO:0003924:GTPase activity][GO:0005200:structural constituent of cytoskeleton][GO:0007017:microtubule-based process]	-	-	XP_010271905.1:PREDICTED: tubulin beta1 chain [Nelumbo nucifera] >XP_010271906.1 PREDICTED: tubulin beta1 chain [Nelumbo nucifera]	gi|460240246|gb|KC243684.1|:Salix arbutifolia beta-tubulin 17 mRNA, complete cds	A5ATG8|A5ATG8_VITVI:Tubulin beta chain;OS=Vitis vinifera;GN=VIT_08s0040g00980 {ECO:0000313|EMBL:CCB57123.1};|GO:0005737,GO:0005874,|GO:0005525,GO:0003924,GO:0005200,|GO:0007017,
DN118291_c0_g1_i1	DOWN	[GO:0016021:integral component of membrane][GO:0016757:transferase activity, transferring glycosyl groups]	[ko00515:Mannose type O-glycan biosynthesis]	Zmw_sc00532.1.g00310.1:Zoysia matrella|C2H2|C2H2 family protein	XP_021851441.1:uncharacterized protein LOC110790991 [Spinacia oleracea] >KNA12727.1 hypothetical protein SOVF_123330 [Spinacia oleracea]	-	A0A0K9R1R0|A0A0K9R1R0_SPIOL:Uncharacterized protein;OS=Spinacia oleracea;|GO:0016021,|GO:0016757,

geneid	trend	GO	Pathway	TF	NR	NT	Uniprot
DN125757_c0_g2_i1	DOWN	-	-	-	XP_010678868.1:PREDICTED: C2 and GRAM domaincontaining protein At5g50170 [Beta vulgaris subsp. vulgaris] >KMT10701.1 hypothetical protein BVRB_5g118100 [Beta vulgaris subsp. vulgaris]	-	A0A0J8F4I3|A0A0J8F4I3_BETVU:Uncharacterized protein;OS=Beta vulgaris subsp. vulgaris.;
DN116024_c1_g2_i2	DOWN	[GO:0005634:nucleus][GO:0003677:DNA binding][GO:0006355:regulation of transcription, DNA-templated][GO:0006351:transcription, DNA-templated]	-	AHYPO_009703-RA:Amaranthus hypochondriacus|B3|B3 family protein	XP_021728607.1:B3 domaincontaining protein At2g36080like isoform X2 [Chenopodium quinoa]	gi|253560641|gb|GQ227992.1|:Camellia sinensis RAV transcription factor mRNA, complete cds	F6HKN6|F6HKN6_VITVI:Putative uncharacterized protein;OS=Vitis vinifera;GN=VIT_08s0007g02810 {ECO:0000313|EMBL:CCB55458.1};|GO:0005634,|GO:0003677,|GO:0006355, GO:0006351,
DN123155_c1_g1_i1	DOWN	-	-	-	-	-	-

geneid	trend	GO	Pathway	TF	NR	NT	Uniprot
DN121857_c1_g1_i3	DOWN	[GO:0005789:endoplasmic reticulum membrane][GO:0016021:integral component of membrane][GO:0004420:hydroxymethylglutaryl-CoA reductase (NADPH) activity][GO:0050661:NADP binding][GO:0015936:coenzyme A metabolic process][GO:0008299:isoprenoid biosynthetic process]	[ko00900:Terpenoid backbone biosynthesis][ko04152:AMPK signaling pathway][ko04976:Bile secretion]	RrC14701_p1:Raphanus raphanistrum|HSF|HSF family protein	GAV85418.1:HMGCoA_red domaincontaining protein [Cephalotus follicularis]	gi|356577270|ref|XM_003556703.1|:PREDICTED: Glycine max 3-hydroxy-3-methylglutaryl-coenzyme A reductase 1-like (LOC100779932), mRNA	A0A067JY31|A0A067JY31_JATCU:3-hydroxy-3-methylglutaryl coenzyme A reductase;OS=Jatropha curcas;|GO:0005789,GO:0016021,|GO:0004420,GO:0050661,|GO:0015936,GO:0008299,
DN118691_c2_g1_i3	DOWN	-	-	-	-	-	-
DN115629_c3_g2_i3	DOWN	[GO:0006457:protein folding]	-	Achn036321:Actinidia chinensis|G2-like|G2-like family protein	XP_021844662.1:dnaJ homolog subfamily B member 1like [Spinacia oleracea] >KNA09714.1 hypothetical protein SOVF_151130 [Spinacia oleracea]	gi|255558263|ref|XM_002520113.1|:Ricinus communis Curved DNA-binding protein, putative, mRNA	A0A0K9QQY5|A0A0K9QQY5_SPIOL: Uncharacterized protein;OS=Spinacia oleracea;|GO:0006457,

geneid	trend	GO	Pathway	TF	NR	NT	Uniprot
DN126871_c1_g3_i12	DOWN	[GO:0016021:integral component of membrane][GO:0005524:ATP binding][GO:0004672:protein kinase activity]	-	mrna09125.1-v1.0-hybrid:Fragaria vesca|bHLH|bHLH family protein	XP_010694279.1:PREDICTED: calcium/calmodulinregulated receptorlike kinase 1 [Beta vulgaris subsp. vulgaris] >XP_010694280.1 PREDICTED: calcium/calmodulinregulated receptorlike kinase 1 [Beta vulgaris subsp. vulgaris] >XP_010694281.1 PREDICTED: calcium/calmodulinregulated receptorlike kinase 1 [Beta vulgaris subsp. vulgaris] >XP_010694282.1 PREDICTED: calcium/calmodulinregulated receptorlike	gi|470116310|ref|XM_004294278.1|:PREDICTED: Fragaria vesca subsp. vesca probable leucine-rich repeat receptor-like serine/threonine-protein kinase At5g15730-like (LOC101291802), mRNA	A0A0J8E5S9|A0A0J8E5S9_BETVU:Uncharacterized protein;OS=Beta vulgaris subsp. vulgaris.;|GO:0016021,|GO:0005524,GO:0004672,

geneid	trend	GO	Pathway	TF	NR	NT	Uniprot
					kinase 1 [Beta vulgaris subsp. vulgaris] >KMS98545.1 hypothetical protein BVRB_4g092340 [Beta vulgaris subsp. vulgaris]		

geneid	trend	GO	Pathway	TF	NR	NT	Uniprot
DN128061_c0_g1_i3	DOWN	[GO:0016021:integral component of membrane][GO:0005524:ATP binding][GO:0042626:ATPase activity, coupled to transmembrane movement of substances]	[ko01523:Antifolate resistance][ko01524:Platinum drug resistance][ko02010:ABC transporters][ko04976:Bile secretion]	RrC2480_p5:Raphanus raphanistrum|HSF|HSF family protein	XP_002276212.2:PREDICTED: ABC transporter C family member 8 isoform X1 [Vitis vinifera]	gi|359482525|ref|XM_002276176.2|:PREDICTED: Vitis vinifera ABC transporter C family member 8-like (LOC100246410), mRNA	F6HY32|F6HY32_VITVI:Putative uncharacterized protein;OS=Vitis vinifera;GN=VIT_09s0002g02430 {ECO:0000313|EMBL:CCB59355.1};|GO:0016021,|GO:0005524,GO:0042626,
DN126602_c3_g2_i1	DOWN	[GO:0003824:catalytic activity]	[ko00052:Galactose metabolism]	-	XP_021771609.1:galactinolsucrose galactosyltransferase like [Chenopodium quinoa]	gi|224131913|ref|XM_002328103.1|:Populus trichocarpa predicted protein, mRNA	A0A0J8FV17|A0A0J8FV17_BETVU:Uncharacterized protein;OS=Beta vulgaris subsp. vulgaris.;|GO:0003824,

geneid	trend	GO	Pathway	TF	NR	NT	Uniprot
DN122030_c1_g2_i3	DOWN	[GO:0046872:metal ion binding][GO:0030001:metal ion transport]	-	Zjn_sc00020.1.g02760.1.am.mk:Zoysia japonica|MYB_related|MYB_related family protein	XP_010671305.1:PREDICTED: heavy metalassociated isoprenylated plant protein 9 isoform X1 [Beta vulgaris subsp. vulgaris] >KMT16459.1 hypothetical protein BVRB_3g050800 isoform B [Beta vulgaris subsp. vulgaris]	gi|502095841|ref|XM_004490494.1|:PREDICTED: Cicer arietinum myb-like protein X-like (LOC101513750), mRNA	A0A0K9R2B9|A0A0K9R2B9_SPIOL:Uncharacterized protein;OS=Spinacia oleracea;|GO:0046872,|GO:0030001,
DN116806_c0_g1_i1	DOWN	-	-	-	-	-	-
DN128786_c1_g1_i11	DOWN	-	-	-	XP_021759112.1:uncharacterized protein LOC110724013 [Chenopodium quinoa]	-	D7THD8|D7THD8_VITVI:Putative uncharacterized protein;OS=Vitis vinifera;GN=VIT_08s0007g01560 {ECO:0000313|EMBL:CBI30454.3};

geneid	trend	GO	Pathway	TF	NR	NT	Uniprot
DN119997_c4_g1_i4	DOWN	[GO:0008061:chitin binding][GO:0004568:chitinase activity][GO:0005975:carbohydrate metabolic process][GO:0016998:cell wall macromolecule catabolic process][GO:0006032:chitin catabolic process]	[ko00520:Amino sugar and nucleotide sugar metabolism]	-	ACD69683.1:chitinase, partial [Mangifera indica]	-	D3TIC1|D3TIC1_MANIN:Chitinase;OS=Mangifera indica;|GO:0008061,GO:0004568,|GO:0005975,GO:0016998,GO:0006032,
DN119283_c1_g1_i1	DOWN	[GO:0016021:integral component of membrane]	-	-	XP_021850766.1:uncharacterized protein LOC110790291 [Spinacia oleracea]	-	A0A0J8CAA1|A0A0J8CAA1_BETVU: Uncharacterized protein;OS=Beta vulgaris subsp. vulgaris.;|GO:0016021,
DN121439_c0_g1_i6	DOWN	[GO:0016021:integral component of membrane][GO:0006952:defense response][GO:0009607:response to biotic stimulus]	-	29656.m000483:Ricinus communis|NF-YC|NF-YC family protein	XP_021864783.1:MLOlike protein 4 isoform X1 [Spinacia oleracea] >KNA21881.1 hypothetical protein SOVF_039050 [Spinacia oleracea]	gi|521655972|gb|GAIA01039183.1|:TSA: Salicornia europaea Unigene43848_Se200S transcribed RNA sequence	A0A0K9RQR9|A0A0K9RQR9_SPIOL: MLO-like protein;OS=Spinacia oleracea;|GO:0016021,|GO:0006952,GO:0009607,

geneid	trend	GO	Pathway	TF	NR	NT	Uniprot
DN128299_c5_g9_i2	DOWN	[GO:0020037:heme binding][GO:0005506:iron ion binding][GO:0004497:monooxygenase activity][GO:0016705:oxidoreductase activity, acting on paired donors, with incorporation or reduction of molecular oxygen]	-	Cc10_g03650:Coffea canephora|G2-like|G2-like family protein	XP_010687564.1:PREDICTED: cytochrome P450 76C4like [Beta vulgaris subsp. vulgaris]	-	A0A0U3BG65|A0A0U3BG65_BETVU: Uncharacterized protein;OS=Beta vulgaris;|GO:0020037,GO:0005506,GO:0004497,GO:0016705,
DN122293_c2_g3_i10	DOWN	-	-	Rsa1.0_00048.1_g00027.1:Raphanus sativus|bHLH|bHLH family protein	XP_021725594.1:isoflavone reductase homolog [Chenopodium quinoa]	-	A0A0K9RRW7|A0A0K9RRW7_SPIOL:Uncharacterized protein;OS=Spinacia oleracea;
DN127671_c4_g2_i1	DOWN	[GO:0016021:integral component of membrane][GO:0005524:ATP binding][GO:0016887:ATPase activity][GO:0005215:transporter activity]	-	RrC2480_p5:Raphanus raphanistrum|HSF|HSF family protein	XP_010689556.1:PREDICTED: ABC transporter A family member 2 [Beta vulgaris subsp. vulgaris] >KMT02071.1 hypothetical protein BVRB_9g208000 [Beta vulgaris subsp. vulgaris]	gi|521494056|gb|GAHZ01036864.1|:TSA: Salicornia europaea Unigene41568_SeCKS transcribed RNA sequence	A0A0J8EGH0|A0A0J8EGH0_BETVU: Uncharacterized protein;OS=Beta vulgaris subsp. vulgaris.;|GO:0016021,|GO:0005524,GO:0016887,GO:0005215,

geneid	trend	GO	Pathway	TF	NR	NT	Uniprot
DN119853_c0_g3_i1	DOWN	[GO:0016021:integral component of membrane][GO:0008270:zinc ion binding]	[ko04141:Protein processing in endoplasmic reticulum]	-	XP_021752846.1:E3 ubiquitin protein ligase RIN2like [Chenopodium quinoa]	gi|521530832|gb|GAHZ01008040.1|:TSA: Salicornia europaea Unigene8378_SeCKS transcribed RNA sequence	A0A0J8BBX2|A0A0J8BBX2_BETVU: Uncharacterized protein;OS=Beta vulgaris subsp. vulgaris.;|GO:0016021,|GO:0008270,
DN118493_c0_g2_i1	DOWN	[GO:0051287:NAD binding][GO:0003979:UDP-glucose 6-dehydrogenase activity]	[ko00040:Pentose and glucuronate interconversions][ko00053:Ascorbate and aldarate metabolism][ko00520:Amino sugar and nucleotide sugar metabolism]	OGLUM03G34520.2:Oryza glumaepatula|WRKY|WRKY family protein	XP_010678413.1:PREDICTED: UDPglucose 6dehydrogenase 1 [Beta vulgaris subsp. vulgaris] >XP_019105198.1 PREDICTED: UDPglucose 6dehydrogenase 1 [Beta vulgaris subsp. vulgaris] >KMT10725.1 hypothetical protein BVRB_5g113830 [Beta vulgaris subsp. vulgaris]	gi|292684202|dbj|AK339764.1|:Lotus japonicus cDNA, clone: LjFL3-080-AB04, HTC	A0A0J8CAE4|A0A0J8CAE4_BETVU:UDP-glucose 6-dehydrogenase;OS=Beta vulgaris subsp. vulgaris.;|GO:0051287,GO:0003979,

geneid	trend	GO	Pathway	TF	NR	NT	Uniprot
DN124950_c2_g1_i1	DOWN	[GO:0030410:nicotianamine synthase activity][GO:0030418:nicotianamine biosynthetic process]	-	-	XP_021734458.1:nicotianamine synthaselike [Chenopodium quinoa]	-	A0A0K9QJP5|A0A0K9QJP5_SPIOL:Uncharacterized protein;OS=Spinacia oleracea;|GO:0030410,|GO:0030418,
DN116226_c0_g1_i1	DOWN	[GO:0005524:ATP binding][GO:0004672:protein kinase activity]	-	GSVIVT01019630001:Vitis vinifera|MIKC_MADS|MIKC_MADS family protein	XP_010675874.2:PREDICTED: probable serine/threonineprotein kinase WNK11 [Beta vulgaris subsp. vulgaris]	gi|255576305|ref|XM_002529000.1|:Ricinus communis ATP binding protein, putative, mRNA	A0A0J8FAK5|A0A0J8FAK5_BETVU:Uncharacterized protein;OS=Beta vulgaris subsp. vulgaris.;|GO:0005524,GO:0004672,
DN107296_c0_g2_i2	DOWN	[GO:0008168:methyltransferase activity]	-	EMT20798:Aegilops tauschii|MYB_related|MYB_related family protein	XP_010689978.1:PREDICTED: caffeine synthase 1 [Beta vulgaris subsp. vulgaris] >KMT18246.1 hypothetical protein BVRB_2g024130 [Beta vulgaris subsp. vulgaris]	-	A0A0J8FR51|A0A0J8FR51_BETVU:Uncharacterized protein;OS=Beta vulgaris subsp. vulgaris.;|GO:0008168,

geneid	trend	GO	Pathway	TF	NR	NT	Uniprot
DN121526_c0_g2_i13	DOWN	-	[ko04144:Endocytosis]	PH01000276G1150: Phyllostachys heterocycla|bZIP|bZIP family protein	XP_022146239.1:protein EARLYRESPONSIVE TO DEHYDRATION 7, chloroplasticlike [Momordica charantia]	-	A0A103XSF4|A0A103XSF4_CYNCS:Senescence/spartin-associated;OS=Cynara cardunculus var. scolymus;
DN104526_c1_g1_i1	DOWN	[GO:0005524:ATP binding][GO:0004674:protein serine/threonine kinase activity]	-	XP_016443488.1:Nicotiana tabacum|bHLH|bHLH family protein	KMT15300.1:hypothetical protein BVRB_3g060090 [Beta vulgaris subsp. vulgaris]	gi|144925108|gb|AC165135.29|:Medicago truncatula clone mth2-20f2, complete sequence	A0A0J8CNF9|A0A0J8CNF9_BETVU:Uncharacterized protein;OS=Beta vulgaris subsp. vulgaris.;|GO:0005524,GO:0004674,
DN123054_c2_g3_i4	DOWN	[GO:0008171:O-methyltransferase activity]	-	-	KNA03838.1:hypothetical protein SOVF_205310 [Spinacia oleracea]	-	A0A0K9QAW2|A0A0K9QAW2_SPIOL:Uncharacterized protein;OS=Spinacia oleracea;|GO:0008171,
DN117189_c1_g1_i1	DOWN	-	-	-	-	-	-

geneid	trend	GO	Pathway	TF	NR	NT	Uniprot
DN126366_c2_g1_i3	DOWN	-	-	GSVIVT01033372001:Vitis vinifera|NAC|NAC family protein	XP_021741758.1:ricin Blike lectin R40G3 [Chenopodium quinoa]	-	A0A0K9RMJ3|A0A0K9RMJ3_SPIOL:Uncharacterized protein;OS=Spinacia oleracea;
DN116540_c0_g2_i1	DOWN	-	-	-	-	-	-
DN128238_c1_g3_i3	DOWN	-	-	-	-	-	-
DN124251_c0_g14_i1	DOWN	-	-	-	-	-	-
DN115984_c0_g1_i6	DOWN	[GO:0020037:heme binding][GO:0005506:iron ion binding][GO:0016710:trans-cinnamate 4-monooxygenase activity]	[ko00130:Ubiquinone and other terpenoid-quinone biosynthesis][ko00360:Phenylalanine metabolism][ko00940:Phenylpropanoid biosynthesis][ko00941:Flavonoid biosynthesis][ko00945:Stilbenoid, diarylheptanoid and gingerol	Csa09g009740.1:Camelina sativa|HSF|HSF family protein	AAD11427.1:cinnamate 4hydroxylase [Mesembryanthemum crystallinum]	gi|4206115|gb|AF097664.1|AF097664:Mesembryanthemum crystallinum cinnamate 4-hydroxylase mRNA, complete cds	Q9ZSR0|Q9ZSR0_MESCR:Cinnamate 4-hydroxylase;OS=Mesembryanthemum crystallinum;|GO:0020037,GO:0005506,GO:0016710,

geneid	trend	GO	Pathway	TF	NR	NT	Uniprot
			biosynthesis]				
DN126660_c0_g1_i1	DOWN	[GO:0016021:integral component of membrane][GO:0005524:ATP binding][GO:0004672:protein kinase activity]	-	EMT05641:Aegilops tauschii|FAR1|FAR1 family protein	XP_021866985.1:probable leucinerich repeat receptorlike protein kinase At5g49770 isoform X1 [Spinacia oleracea] >KNA06111.1 hypothetical protein SOVF_184090 [Spinacia oleracea]	-	A0A0K9QHR6|A0A0K9QHR6_SPIOL: Uncharacterized protein;OS=Spinacia oleracea;|GO:0016021,|GO:0005524,GO:0004672,

geneid	trend	GO	Pathway	TF	NR	NT	Uniprot
DN121353_c8_g1_i2	DOWN	[GO:0016788:hydrolase activity, acting on ester bonds]	-	-	XP_021720400.1:GDSL esterase/lipase 2like [Chenopodium quinoa]	-	A0A0J8BXN2|A0A0J8BXN2_BETVU: Uncharacterized protein;OS=Beta vulgaris subsp. vulgaris.;|GO:0016788,
DN126748_c3_g3_i4	DOWN	[GO:0005618:cell wall][GO:0005576:extracellular region][GO:0016020:membrane][GO:0009664:plant-type cell wall organization]	-	676732396:Sisymbrium irio|bHLH|bHLH family protein	ADN03964.1:EXP1 [Phytolacca acinosa]	gi|28624705|gb|AY079206.1|:Mirabilis jalapa cell wall protein Exp4 precursor, mRNA, complete cds	E2D554|E2D554_PHYAN:EXP1;OS=Phytolacca acinosa;|GO:0005618,GO:0005576,GO:0016020,|GO:0009664,

geneid	trend	GO	Pathway	TF	NR	NT	Uniprot
DN124472_c0_g2_i4	DOWN	-	[ko00902:Monoterpenoid biosynthesis]	-	XP_010053674.1:PREDICTED: (+)neomenthol dehydrogenase [Eucalyptus grandis] >XP_018727894.1 PREDICTED: (+)neomenthol dehydrogenase [Eucalyptus grandis] >KCW78033.1 hypothetical protein EUGRSUZ_D02258 [Eucalyptus grandis] >KCW78034.1 hypothetical protein EUGRSUZ_D02258 [Eucalyptus grandis]	gi|502157294|ref|XM_004510767.1|:PREDICTED: Cicer arietinum (+)-neomenthol dehydrogenase-like (LOC101492139), mRNA	A0A059CHV7|A0A059CHV7_EUCGR: Uncharacterized protein;OS=Eucalyptus grandis;
DN121127_c0_g2_i3	DOWN	[GO:0008171:O-methyltransferase activity]	-	-	XP_021717347.1:(R,S)reticuline 7Omethyltransferase like [Chenopodium quinoa]	-	A0A0J8BLP8|A0A0J8BLP8_BETVU:Uncharacterized protein;OS=Beta vulgaris subsp. vulgaris.;|GO:0008171,

geneid	trend	GO	Pathway	TF	NR	NT	Uniprot
DN117146_c5_g1_i3	DOWN	-	-	Sp_080370_rfri.t1:Spinacia oleracea|LBD|LBD family protein	XP_021755125.1:LOB domaincontaining protein 40like [Chenopodium quinoa]	-	A0A0K9REJ1|A0A0K9REJ1_SPIOL:Uncharacterized protein;OS=Spinacia oleracea;
DN123359_c1_g2_i6	DOWN	[GO:0016787:hydrolase activity]	[ko04310:Wnt signaling pathway]	Ote100112080011:Ocimum tenuiflorum|bHLH|bHLH family protein	XP_010676220.1:PREDICTED: pectin acetylesterase 8 [Beta vulgaris subsp. vulgaris] >KMT12596.1 hypothetical protein BVRB_5g098360 isoform A [Beta vulgaris subsp. vulgaris]	gi|521734818|gb|GAIA01009500.1|:TSA: Salicornia europaea Unigene9870_Se200S transcribed RNA sequence	A0A0J8F9Q0|A0A0J8F9Q0_BETVU:Uncharacterized protein;OS=Beta vulgaris subsp. vulgaris.;|GO:0016787,

geneid	trend	GO	Pathway	TF	NR	NT	Uniprot
DN95355_c0_g1_i1	DOWN	[GO:0016021:integral component of membrane]	-	-	XP_010687986.1:PREDICTED: uncharacterized protein LOC104902029 [Beta vulgaris subsp. vulgaris] >KMT02860.1 hypothetical protein BVRB_8g194380 [Beta vulgaris subsp. vulgaris]	-	A0A0J8BNB8|A0A0J8BNB8_BETVU: Uncharacterized protein;OS=Beta vulgaris subsp. vulgaris.;|GO:0016021,
DN120667_c1_g6_i1	DOWN	[GO:0005634:nucleus][GO:2000032:regulation of secondary shoot formation][GO:0010223:secondary shoot formation]	-	Bv5_126100_ryfn.t1:Beta vulgaris|TCP|TCP family protein	XP_010695622.1:PREDICTED: transcription factor TCP18 [Beta vulgaris subsp. vulgaris] >KMS97548.1 hypothetical protein BVRB_5g126100 [Beta vulgaris subsp. vulgaris]	-	A0A0J8BBZ7|A0A0J8BBZ7_BETVU:Uncharacterized protein;OS=Beta vulgaris subsp. vulgaris.;|GO:0005634,|GO:2000032,GO:0010223,

geneid	trend	GO	Pathway	TF	NR	NT	Uniprot
DN116470_c0_g1_i1	DOWN	[GO:0005576:extracellular region][GO:0020037:heme binding][GO:0046872:metal ion binding][GO:0004601:peroxidase activity][GO:0042744:hydrogen peroxide catabolic process][GO:0006979:response to oxidative stress]	[ko00940:Phenylpropanoid biosynthesis]	EPS67213.1:Genlisea aurea|NAC|NAC family protein	ACN42168.1:peroxidase 1 [Sesuvium portulacastrum]	gi|224382176|gb|FJ644943.1|:Sesuvium portulacastrum peroxidase 1 (POD1) mRNA, complete cds	C0KXH4|C0KXH4_SESPO:Peroxidase; OS=Sesuvium portulacastrum;|GO:0005576,|GO:0020037,GO:0046872,GO:0004601,|GO:0042744,GO:0006979,
DN121872_c4_g1_i1	DOWN	[GO:0016021:integral component of membrane][GO:0003676:nucleic acid binding][GO:0004523:RNA-DNA hybrid ribonuclease activity][GO:0015074:DNA integration]	-	LOC_Os04g22990.1:Oryza sativa subsp. japonica|FAR1|FAR1 family protein	XP_003632035.1:PREDICTED: uncharacterized protein LOC100854167 [Vitis vinifera]	-	A5C6U7|A5C6U7_VITVI:Putative uncharacterized protein;OS=Vitis vinifera;|GO:0016021,|GO:0003676,GO:0004523,|GO:0015074,
DN120938_c1_g5_i1	DOWN	-	-	-	-	-	-

geneid	trend	GO	Pathway	TF	NR	NT	Uniprot
DN127849_c1_g5_i2	DOWN	[GO:0016021:integral component of membrane][GO:0005524:ATP binding][GO:0042626:ATPase activity, coupled to transmembrane movement of substances]	[ko02010:ABC transporters][ko04976:Bile secretion][ko05206:MicroRNAs in cancer]	Tp57577_TGAC_v2_mRNA22346:Trifolium pratense|M-type_MADS|M-type_MADS family protein	XP_010688391.1:PREDICTED: ABC transporter B family member 11 isoform X1 [Beta vulgaris subsp. vulgaris] >XP_010688392.1 PREDICTED: ABC transporter B family member 11 isoform X1 [Beta vulgaris subsp. vulgaris] >KMT03250.1 hypothetical protein BVRB_8g197930 [Beta vulgaris subsp. vulgaris]	gi|224064761|ref|XM_002301514.1|:Populus trichocarpa multidrug/pheromone exporter, MDR family, ABC transporter family, mRNA	A0A0J8BPC6|A0A0J8BPC6_BETVU:Uncharacterized protein;OS=Beta vulgaris subsp. vulgaris.;|GO:0016021,|GO:0005524,GO:0042626,
DN111836_c1_g1_i2	DOWN	-	-	-	-	-	-
DN117086_c3_g3_i1	DOWN	-	-	-	-	-	-

geneid	trend	GO	Pathway	TF	NR	NT	Uniprot
DN127167_c2_g1_i14	DOWN	[GO:0016021:integral component of membrane][GO:0005886:plasma membrane][GO:0005524:ATP binding][GO:0042626:ATPase activity, coupled to transmembrane movement of substances]	-	-	XP_010690527.2:PREDICTED: ABC transporter G family member 11like [Beta vulgaris subsp. vulgaris]	gi|502181024|ref|XM_004516685.1|:PREDICTED: Cicer arietinum ABC transporter G family member 11-like (LOC101508979), mRNA	F6HTK2|F6HTK2_VITVI:Putative uncharacterized protein;OS=Vitis vinifera;GN=VIT_03s0017g01300 {ECO:0000313|EMBL:CCB58012.1};|GO:0016021,GO:0005886,|GO:0005524,GO:0042626,
DN118611_c1_g2_i1	DOWN	-	-	-	XP_010682870.1:PREDICTED: protein phosphatase 1 regulatory subunit 12A [Beta vulgaris subsp. vulgaris] >KMT07542.1 hypothetical protein BVRB_6g151530 [Beta vulgaris subsp. vulgaris]	-	A0A0J8C256|A0A0J8C256_BETVU:Uncharacterized protein;OS=Beta vulgaris subsp. vulgaris.;
DN113351_c5_g1_i1	DOWN	-	-	-	-	-	-

geneid	trend	GO	Pathway	TF	NR	NT	Uniprot
DN118660_c1_g8_i4	DOWN	[GO:0004834:tryptophan synthase activity][GO:0008652:cellular amino acid biosynthetic process]	[ko00260:Glycine, serine and threonine metabolism][ko00400:Phenylalanine, tyrosine and tryptophan biosynthesis]	-	KNA11302.1:hypothetical protein SOVF_136280 isoform A [Spinacia oleracea]	-	A0A0K9QVL8|A0A0K9QVL8_SPIOL:Tryptophan synthase;OS=Spinacia oleracea;|GO:0004834,|GO:0008652,
DN124529_c2_g6_i7	DOWN	[GO:0016021:integral component of membrane][GO:0015297:antiporter activity]	-	TRIUR3_11943-P1: Triticum urartu|ERF|ERF family protein	XP_021836465.1:protein DETOXIFICATION 40like [Spinacia oleracea] >KNA22506.1 hypothetical protein SOVF_033530 [Spinacia oleracea]	gi|224115729|ref|XM_002317072.1|:Populus trichocarpa predicted protein, mRNA	A0A0K9RSI0|A0A0K9RSI0_SPIOL:Protein DETOXIFICATION;OS=Spinacia oleracea;|GO:0016021,|GO:0015297,GO:0015238,
DN121816_c1_g1_i5	DOWN	[GO:0016021:integral component of membrane][GO:0008270:zinc ion binding]	-	-	XP_021745986.1:uncharacterized protein LOC110711854 [Chenopodium quinoa]	-	A0A0J8BKZ3|A0A0J8BKZ3_BETVU:Uncharacterized protein;OS=Beta vulgaris subsp. vulgaris.;|GO:0016021,|GO:0008270,

geneid	trend	GO	Pathway	TF	NR	NT	Uniprot
DN124251_c0_g4_i1	DOWN	[GO:0004869:cysteine-type endopeptidase inhibitor activity]	-	-	XP_010057838.1:PREDICTED: cysteine proteinase inhibitor 1 [Eucalyptus grandis] >KCW75168.1 hypothetical protein EUGRSUZ_E03919 [Eucalyptus grandis]	-	A0A059C9M1|A0A059C9M1_EUCGR: Cysteine proteinase inhibitor;OS=Eucalyptus grandis;|GO:0004869,
DN110371_c0_g1_i1	DOWN	-	-	-	XP_021765115.1:uncharacterized protein LOC110729652 [Chenopodium quinoa]	-	A0A0J8CWB9|A0A0J8CWB9_BETVU: Uncharacterized protein;OS=Beta vulgaris subsp. vulgaris.;
DN85313_c0_g1_i1	DOWN	-	-	-	-	-	-
DN125211_c1_g4_i12	DOWN	[GO:0005773:vacuole]	-	-	XP_021747950.1:uncharacterized protein LOC110713815 [Chenopodium quinoa]	-	A0A0J8EW61|A0A0J8EW61_BETVU: Uncharacterized protein;OS=Beta vulgaris subsp. vulgaris.;|GO:0005773,

geneid	trend	GO	Pathway	TF	NR	NT	Uniprot
DN122401_c7_g1_i6	DOWN	-	-	-	XP_021767973.1:VAN3binding proteinlike [Chenopodium quinoa]	-	A0A0J8CEC2|A0A0J8CEC2_BETVU:Uncharacterized protein;OS=Beta vulgaris subsp. vulgaris.;
DN117110_c0_g5_i1	DOWN	-	-	-	XP_002524160.1:PREDICTED: uncharacterized protein LOC8274807 [Ricinus communis] >EEF38223.1 conserved hypothetical protein [Ricinus communis]	-	B9SDU1|B9SDU1_RICCO:Putative uncharacterized protein;OS=Ricinus communis;
DN125366_c5_g5_i1	DOWN	-	-	-	-	-	-

geneid	trend	GO	Pathway	TF	NR	NT	Uniprot
DN126602_c2_g2_i1	DOWN	[GO:0019139:cytokinin dehydrogenase activity][GO:0050660:flavin adenine dinucleotide binding][GO:0016614:oxidoreductase activity, acting on CH-OH group of donors][GO:0009690:cytokinin metabolic process]	[ko00908:Zeatin biosynthesis]	PGSC0003DMP400000652:Solanum tuberosum|bHLH|bHLH family protein	XP_021744102.1:cytokinin dehydrogenase 7like [Chenopodium quinoa]	gi|511319945|gb|GAKE01012932.1|:TSA : Thlaspi arvense Contig13130 transcribed RNA sequence	A0A0J8CW83|A0A0J8CW83_BETVU: Uncharacterized protein;OS=Beta vulgaris subsp. vulgaris.;|GO:0019139,GO:0050660,GO:0016614,|GO:0009690,
DN122081_c1_g1_i2	DOWN	-	-	KFK43895.1:Arabis alpina|STAT|STAT family protein	KNA12238.1:hypothetical protein SOVF_127760 [Spinacia oleracea]	gi|521526499|gb|GAHZ01010146.1|:TSA : Salicornia europaea Unigene10745_SeCKS transcribed RNA sequence	A0A0K9QZW9|A0A0K9QZW9_SPIOL:Uncharacterized protein;OS=Spinacia oleracea;
DN127223_c0_g1_i1	DOWN	[GO:0000287:magnesium ion binding][GO:0010333:terpene synthase activity][GO:0008152:metabolic process]	[ko00902:Monoterpenoid biosynthesis]	MDP0000245233:Malus domestica|bHLH|bHLH family protein	XP_021850089.1:(3S,6E)nerolidol synthase 1like isoform X2 [Spinacia oleracea]	-	A0A0K9RG31|A0A0K9RG31_SPIOL:Uncharacterized protein;OS=Spinacia oleracea;|GO:0000287,GO:0010333,|GO:0008152,
DN102684_c0_g1_i1	DOWN	-	-	-	-	-	-

geneid	trend	GO	Pathway	TF	NR	NT	Uniprot
DN128828_c1_g1_i3	DOWN	[GO:0030131:clathrin adaptor complex][GO:0008565:obsolete protein transporter activity][GO:0006886:intracellular protein transport][GO:0016192:vesicle-mediated transport]	[ko04144:Endocytosis][ko04721:Synaptic vesicle cycle][ko04961:Endocrine and other factor-regulated calcium reabsorption][ko05016:Huntington's disease]	FANhyb_rscf00000217.1.g00006.1:Fragaria x ananassa|WRKY|WRKY family protein	XP_010689981.1:PREDICTED: AP2 complex subunit alpha1like [Beta vulgaris subsp. vulgaris]	gi|255573013|ref|XM_002527391.1|:Ricinus communis AP-2 complex subunit alpha, putative, mRNA	A0A0J8BPD0|A0A0J8BPD0_BETVU:Uncharacterized protein;OS=Beta vulgaris subsp. vulgaris.;|GO:0030131,|GO:0008565,|GO:0006886,GO:0016192,
DN116568_c0_g1_i1	DOWN	-	-	-	XP_010672718.1:PREDICTED: polygalacturonase inhibitor 1 [Beta vulgaris subsp. vulgaris] >KMT15373.1 hypothetical protein BVRB_3g060780 [Beta vulgaris subsp. vulgaris]	-	A0A0J8CTT7|A0A0J8CTT7_BETVU:Uncharacterized protein;OS=Beta vulgaris subsp. vulgaris.;

geneid	trend	GO	Pathway	TF	NR	NT	Uniprot
DN127140_c1_g2_i3	DOWN	[GO:0005576:extracellular region][GO:0009405:pathogenesis]	-	-	XP_002264858.1:PREDICTED: uncharacterized protein LOC100241465 [Vitis vinifera]	-	F6HZZ6|F6HZZ6_VITVI:Putative uncharacterized protein;OS=Vitis vinifera;GN=VIT_07s0005g06090 {ECO:0000313|EMBL:CCB60262.1};|GO:0005576,|GO:0009405,
DN101825_c0_g1_i2	DOWN	[GO:0016021:integral component of membrane]	-	-	XP_021735331.1:uncharacterized protein LOC110702001 [Chenopodium quinoa]	-	A0A061ENX2|A0A061ENX2_THECC: Uncharacterized protein;OS=Theobroma cacao;|GO:0016021,
DN126196_c1_g2_i6	DOWN	[GO:0016021:integral component of membrane][GO:0005524:ATP binding][GO:0004674:protein serine/threonine kinase activity]	-	Sme2.5_01196.1_g00006.1:Solanum melongena|STAT|STAT family protein	XP_021740781.1:LRR receptorlike serine/threonineprotein kinase [Chenopodium quinoa]	gi|521737905|gb|GAIA01007848.1|:TSA: Salicornia europaea Unigene8170_Se200S transcribed RNA sequence	A0A0J8CKS4|A0A0J8CKS4_BETVU:Uncharacterized protein;OS=Beta vulgaris subsp. vulgaris.;|GO:0016021,|GO:0005524,GO:0004674,

geneid	trend	GO	Pathway	TF	NR	NT	Uniprot
DN129138_c7_g4_i1	DOWN	[GO:0004568:chitinase activity][GO:0005975:carbohydrate metabolic process][GO:0016998:cell wall macromolecule catabolic process][GO:0006032:chitin catabolic process]	[ko00520:Amino sugar and nucleotide sugar metabolism]	-	XP_010094102.1:Endochitinase [Morus notabilis] >EXB55192.1 Endochitinase [Morus notabilis]	-	Q7M1Q9|Q7M1Q9_PHYAM:Chitinase A;OS=Phytolacca americana;|GO:0004568,|GO:0005975,GO:0016998,GO:0006032,
DN123155_c1_g1_i5	DOWN	[GO:0006950:response to stress]	-	-	XP_010680967.1:PREDICTED: abscisic stressripening protein 2 [Beta vulgaris subsp. vulgaris] >KMT19566.1 hypothetical protein BVRB_1g011850 [Beta vulgaris subsp. vulgaris]	gi|521651526|gb|GAIA01041192.1|:TSA: Salicornia europaea Unigene46283_Se200S transcribed RNA sequence	A0A0J8FV71|A0A0J8FV71_BETVU:Uncharacterized protein;OS=Beta vulgaris subsp. vulgaris.;|GO:0006950,
DN128505_c2_g3_i3	DOWN	[GO:0005576:extracellular region][GO:0030145:manganese ion binding][GO:0045735:nutrient reservoir activity]	-	678365117:Utricularia gibba|bZIP|bZIP family protein	XP_021714510.1:germinlike protein subfamily 2 member 4 [Chenopodium quinoa]	-	A0A0K9R898|A0A0K9R898_SPIOL:Uncharacterized protein;OS=Spinacia oleracea;|GO:00055

geneid	trend	GO	Pathway	TF	NR	NT	Uniprot
							76,|GO:0030145,GO:0045735,
DN119812_c4_g1_i3	DOWN	[GO:0046872:metal ion binding][GO:0004722:protein serine/threonine phosphatase activity]	[ko04016:MAPK signaling pathway - plant][ko04075:Plant hormone signal transduction][ko04931:Insulin resistance]	KZV22762.1:Dorcoceras hygrometricum|CAMTA|CAMTA family protein	XP_015873860.1:PREDICTED: probable protein phosphatase 2C 24 [Ziziphus jujuba]	-	A0A067LDT9|A0A067LDT9_JATCU: Uncharacterized protein;OS=Jatropha curcas;|GO:0046872,GO:0004722,
DN117050_c0_g1_i3	DOWN	[GO:0016021:integral component of membrane][GO:0005215:transporter activity]	-	GSMUA_Achr10P22520_001:Musa acuminata|TALE|TALE family protein	XP_010691634.1:PREDICTED: protein NRT1/ PTR FAMILY 5.6 [Beta vulgaris subsp. vulgaris] >KMT18416.1 hypothetical protein BVRB_2g025620 [Beta vulgaris subsp. vulgaris]	gi|356537957|ref|XM_003537425.1|:PREDICTED: Glycine max probable peptide/nitrate transporter At3g53960-like (LOC100794418), mRNA	A0A0J8FRN7|A0A0J8FRN7_BETVU:Uncharacterized protein;OS=Beta vulgaris subsp. vulgaris.;|GO:0016021,|GO:0005215,

geneid	trend	GO	Pathway	TF	NR	NT	Uniprot
DN121983_c8_g6_i1	DOWN	-	-	-	KMT15827.1:hypothetical protein BVRB_3g057960 [Beta vulgaris subsp. vulgaris]	-	A0A0J8CQH3|A0A0J8CQH3_BETVU: Uncharacterized protein;OS=Beta vulgaris subsp. vulgaris.;
DN118738_c1_g2_i3	DOWN	[GO:0003824:catalytic activity][GO:0050662:coenzyme binding]	-	MDP0000265073:Malus domestica|NAC|NAC family protein	XP_021862341.1:cinnamoylCoA reductase 1like [Spinacia oleracea]	gi|199580018|gb|AC189336.2|:Brassica rapa subsp. pekinensis clone KBrB039G17, complete sequence	A0A0J8CLC1|A0A0J8CLC1_BETVU:Uncharacterized protein;OS=Beta vulgaris subsp. vulgaris.;|GO:0003824,GO:0050662,
DN118691_c1_g4_i1	DOWN	-	-	-	XP_021731374.1:EG45like domain containing protein [Chenopodium quinoa]	-	A0A0J8BQ32|A0A0J8BQ32_BETVU:Uncharacterized protein;OS=Beta vulgaris subsp. vulgaris.;
DN124168_c2_g1_i5	DOWN	[GO:0030246:carbohydrate binding][GO:0008810:cellulase activity][GO:0030245:cellulose catabolic process]	-	EMT11687:Aegilops tauschii|ERF|ERF family protein	XP_011101037.1:endoglucanase 6 [Sesamum indicum]	gi|148763626|gb|EF608067.1|:Mangifera indica endo-beta-1,4-glucanase mRNA, complete cds	A0A0R6MNA7|A0A0R6MNA7_HEVBR:Endoglucanase;OS=Hevea brasiliensis;|GO:0030246,GO:0008810,|

geneid	trend	GO	Pathway	TF	NR	NT	Uniprot
							GO:0030245,
DN113610_c0_g1_i2	DOWN	[GO:0003676:nucleic acid binding][GO:0008270:zinc ion binding]	-	-	XP_021726128.1:uncharacterized protein LOC110693273 [Chenopodium quinoa]	-	F4NCI9|F4NCI9_BETVU:Uncharacterized protein;OS=Beta vulgaris subsp. vulgaris.;|GO:0003676,GO:0008270,
DN118492_c0_g1_i1	DOWN	[GO:0005622:intracellular][GO:0008270:zinc ion binding][GO:0035556:intracellular signal transduction]	-	-	XP_004142834.1:PREDICTED: uncharacterized protein LOC101213112 [Cucumis sativus] >KGN51301.1 hypothetical protein Csa_5G515310 [Cucumis sativus]	-	A0A0A0KU87|A0A0A0KU87_CUCSA: Uncharacterized protein;OS=Cucumis sativus;|GO:0005622,|GO:0008270,|GO:0035556,
DN113760_c0_g1_i1	DOWN	[GO:0008146:sulfotransferase activity]	-	676777512:Sisymbrium irio|ERF|ERF family protein	XP_021855367.1:cytosolic sulfotransferase	-	A0A0J8BEN1|A0A0J8BEN1_BETVU:Sulfotransferase;OS=

geneid	trend	GO	Pathway	TF	NR	NT	Uniprot
					5like [Spinacia oleracea]		Beta vulgaris subsp. vulgaris.;|GO:0008146,
DN111841_c0_g4_i2	DOWN	[GO:0005737:cytoplasm][GO:0003999:adenine phosphoribosyltransferase activity][GO:0006168:adenine salvage][GO:0009116:nucleoside metabolic process]	[ko00230:Purine metabolism]	Oropetium_20150105_18128A:Oropetium thomaeum|bHLH|bHLH family protein	XP_021769363.1:adenine phosphoribosyltransferase 5like [Chenopodium quinoa]	gi|255553230|ref|XM_002517612.1|:Ricinus communis Adenine phosphoribosyltransferase, putative, mRNA	A0A0J8CYY0|A0A0J8CYY0_BETVU:Uncharacterized protein;OS=Beta vulgaris subsp. vulgaris.;|GO:0005737,|GO:0003999,|GO:0006168,GO:0009116,
DN119737_c0_g1_i4	DOWN	-	[ko04075:Plant hormone signal transduction]	RrC1700_p1:Raphanus raphanistrum|ERF|ERF family protein	XP_021714393.1:probable indole3acetic acidamido synthetase GH3.1 isoform X1 [Chenopodium quinoa]	gi|521532536|gb|GAHZ01007143.1|:TSA: Salicornia europaea Unigene7459_SeCKS transcribed RNA sequence	A0A0J8CC67|A0A0J8CC67_BETVU:Uncharacterized protein;OS=Beta vulgaris subsp. vulgaris.;
DN112897_c0_g2_i2	DOWN	[GO:0005576:extracellular region][GO:0030145:manganese ion binding][GO:0045735:nutrient reservoir activity]	-	678365117:Utricularia gibba|bZIP|bZIP family protein	AFK48662.1:unknown [Lotus japonicus]	-	I3T820|I3T820_LOTJA:Uncharacterized protein;OS=Lotus japonicus;|GO:0005576,|GO:0030145,G

geneid	trend	GO	Pathway	TF	NR	NT	Uniprot
							O:0045735,
DN124746_c 1_g3_i3	DOWN	[GO:0016702:oxidoreductase activity, acting on single donors with incorporation of molecular oxygen, incorporation of two atoms of oxygen]	[ko00906:Carotenoi d biosynthesis]	-	XP_021851772.1:pr obable carotenoid cleavage dioxygenase 4, chloroplastic [Spinacia oleracea] >KNA23039.1 hypothetical protein SOVF_028540 [Spinacia oleracea]	-	A0A0K9RVT5|A0A 0K9RVT5_SPIOL: Uncharacterized protein;OS=Spinacia oleracea;|GO:00167 02,
DN123993_c 0_g2_i8	DOWN	[GO:0016021:integral component of membrane]	-	Gh_D13G2412:Goss ypium hirsutum|GATA|GA TA family protein	XP_010682053.1:P REDICTED: uncharacterized protein LOC104896958 isoform X1 [Beta vulgaris subsp. vulgaris]	gi|521538674|gb|GA HZ01003949.1|:TSA : Salicornia europaea Unigene4238_SeCK S transcribed RNA sequence	A0A0J8EXH9|A0A 0J8EXH9_BETVU: Uncharacterized protein;OS=Beta vulgaris subsp. vulgaris.;|GO:00160 21,
DN117969_c	DOWN	-	-	-	-	-	-

geneid	trend	GO	Pathway	TF	NR	NT	Uniprot
5_g2_i1							
DN127426_c 2_g1_i1	DOWN	[GO:0003987:acetate-CoA ligase activity][GO:0016208:AMP binding][GO:0005524:ATP binding][GO:0019427:acetyl-CoA biosynthetic process from acetate]	[ko00010:Glycolysis / Gluconeogenesis][ko00620:Pyruvate metabolism][ko00640:Propanoate metabolism][ko00680:Methane metabolism][ko00720:Carbon fixation pathways in prokaryotes]	-	XP_010682211.1:PREDICTED: acetylcoenzyme A synthetase, chloroplastic/glyoxysomal [Beta vulgaris subsp. vulgaris]	gi|521637211|gb|GAIA01048055.1|:TSA: Salicornia europaea Unigene56289_Se200S transcribed RNA sequence	A0A0K9R5X9|A0A0K9R5X9_SPIOL:Acetyl-coenzyme A synthetase;OS=Spinacia oleracea;|GO:0003987,GO:0016208,GO:0005524,|GO:0019427,
DN109382_c 0_g1_i1	DOWN	[GO:0016021:integral component of membrane]	-	676746044:Sisymbrium irio|MIKC_MADS|MIKC_MADS family protein	XP_010261204.1:PREDICTED: uncharacterized protein At1g04910 isoform X1 [Nelumbo nucifera]	gi|521630323|gb|GAIA01051073.1|:TSA: Salicornia europaea Unigene59665_Se200S transcribed RNA sequence	B9SIX5|B9SIX5_RICCO:Putative uncharacterized protein;OS=Ricinus communis;|GO:0016021,
DN127706_c 1_g2_i2	DOWN	[GO:0008270:zinc ion binding]	-	-	XP_021908061.1:uncharacterized protein LOC110822292 [Carica papaya]	gi|521526872|gb|GAHZ01009951.1|:TSA: Salicornia europaea Unigene10508_SeCKS transcribed RNA	V4UHE2|V4UHE2_9ROSI:Uncharacterized protein;OS=Citrus clementina.;|GO:000

geneid	trend	GO	Pathway	TF	NR	NT	Uniprot
						sequence	8270,
DN124227_c0_g1_i1	DOWN	-	-	-	XP_021764393.1:uncharacterized protein LOC110729004 [Chenopodium quinoa]	-	A0A0K9RW49|A0A0K9RW49_SPIOL: Uncharacterized protein;OS=Spinacia oleracea;
DN119699_c3_g4_i1	DOWN	-	-	-	XP_010056457.1:PREDICTED: protein GAST1 [Eucalyptus grandis] >KCW90091.1 hypothetical protein EUGRSUZ_A02289 [Eucalyptus grandis]	-	A0A059DI63|A0A059DI63_EUCGR:Uncharacterized protein;OS=Eucalyptus grandis;
DN100423_c0_g2_i1	DOWN	-	-	-	-	-	-
DN119596_c2_g1_i5	DOWN	[GO:0004185:serine-type carboxypeptidase activity]	-	Sme2.5_00187.1_g00004.1:Solanum melongena|MYB_related|MYB_related family protein	KMT02607.1:hypothetical protein BVRB_9g202920 [Beta vulgaris subsp. vulgaris]	gi|255558660|ref|XM_002520309.1|:Ricinus communis serine carboxypeptidase,	A0A0J8BMX7|A0A0J8BMX7_BETVU: Carboxypeptidase;OS=Beta vulgaris subsp.

geneid	trend	GO	Pathway	TF	NR	NT	Uniprot
						putative, mRNA	vulgaris.;|GO:0004185,
DN120390_c1_g1_i1	DOWN	[GO:0016021:integral component of membrane][GO:0020037:heme binding][GO:0005506:iron ion binding][GO:0004497:monooxygenase activity][GO:0016705:oxidoreductase activity, acting on paired donors, with incorporation or reduction of molecular oxygen]	-	CCG000365.1:Populus euphratica|NAC|NAC family protein	XP_021850062.1:alkane hydroxylase MAH1like [Spinacia oleracea]	gi|521707701|gb|GAIA01014118.1|:TSA: Salicornia europaea Unigene14772_Se200S transcribed RNA sequence	A0A0K9QR84|A0A0K9QR84_SPIOL:Uncharacterized protein;OS=Spinacia oleracea;|GO:0016021,|GO:0020037,GO:0005506,GO:0004497,GO:0016705,
DN127054_c4_g1_i1	DOWN	[GO:0016021:integral component of membrane][GO:0005509:calcium ion binding][GO:0016174:NAD(P)H oxidase activity][GO:0004601:peroxidase activity]	[ko04016:MAPK signaling pathway - plant][ko04626:Plant-pathogen interaction]	-	KNA08419.1:hypothetical protein SOVF_162770 [Spinacia oleracea]	gi|371767602|gb|JN808448.1|:Phaseolus vulgaris NADPH oxidase (RbohB) mRNA, partial cds	A0A0K9QME8|A0A0K9QME8_SPIOL:Uncharacterized protein;OS=Spinacia oleracea;|GO:0016021,|GO:0005509,GO:0016174,GO:0004601,

geneid	trend	GO	Pathway	TF	NR	NT	Uniprot
DN119628_c1_g2_i1	DOWN	-	-	-	XP_010679295.1:PREDICTED: uncharacterized protein LOC104894701 [Beta vulgaris subsp. vulgaris] >KMT19674.1 hypothetical protein BVRB_1g010600 [Beta vulgaris subsp. vulgaris]	-	A0A0J8D5Q1|A0A0J8D5Q1_BETVU:Uncharacterized protein;OS=Beta vulgaris subsp. vulgaris.;
DN128951_c12_g4_i2	DOWN	-	-	-	-	-	-
DN120213_c0_g1_i1	DOWN	[GO:0016207:4-coumarate-CoA ligase activity][GO:0009699:phenylpropanoid biosynthetic process]	[ko00130:Ubiquinone and other terpenoid-quinone biosynthesis][ko00360:Phenylalanine metabolism][ko00940:Phenylpropanoid biosynthesis]	Spipo12G0053800:Spirodela polyrhiza|C2H2|C2H2 family protein	XP_021755918.1:4coumarateCoA ligase 1like [Chenopodium quinoa]	gi|502149478|ref|XM_004507487.1|:PREDICTED: Cicer arietinum 4-coumarate--CoA ligase-like (LOC101509548), mRNA	A0A0J8BPZ1|A0A0J8BPZ1_BETVU:Uncharacterized protein;OS=Beta vulgaris subsp. vulgaris.;|GO:0016207,|GO:0009699,

geneid	trend	GO	Pathway	TF	NR	NT	Uniprot
DN118020_c3_g2_i4	DOWN	[GO:0005524:ATP binding][GO:0004674:protein serine/threonine kinase activity]	[ko04016:MAPK signaling pathway - plant]	Gh_D11G0701:Gossypium hirsutum|NAC|NAC family protein	XP_010673003.1:PREDICTED: serine/threonineprotein kinase OXI1 [Beta vulgaris subsp. vulgaris] >KMT15160.1 hypothetical protein BVRB_3g062740 [Beta vulgaris subsp. vulgaris]	gi|521540920|gb|GAHZ01002816.1|:TSA: Salicornia europaea Unigene2945_SeCKS transcribed RNA sequence	A0A0J8CN39|A0A0J8CN39_BETVU:Uncharacterized protein;OS=Beta vulgaris subsp. vulgaris.;|GO:0005524,GO:0004674,
DN113229_c0_g1_i1	DOWN	[GO:0016021:integral component of membrane][GO:0005524:ATP binding][GO:0016887:ATPase activity][GO:0006810:transport]	-	Rsa1.0_03996.1_g00001.1:Raphanus sativus|MYB_related|MYB_related family protein	XP_021731921.1:pleiotropic drug resistance protein 1like [Chenopodium quinoa]	gi|521527947|gb|GAHZ01009415.1|:TSA: Salicornia europaea Unigene9864_SeCKS transcribed RNA sequence	A0A0K9QP53|A0A0K9QP53_SPIOL:Uncharacterized protein;OS=Spinacia oleracea;|GO:0016021,|GO:0005524,GO:0016887,|GO:0006810,

<table>
<tr><th>geneid</th><th>trend</th><th>GO</th><th>Pathway</th><th>TF</th><th>NR</th><th>NT</th><th>Uniprot</th></tr>
<tr><td>DN120847_c0_g1_i1</td><td>DOWN</td><td>[GO:0016021:integral component of membrane]</td><td>-</td><td>Vradi03g04520.1:Vigna radiata|bZIP|bZIP family protein</td><td>XP_010683745.1:PREDICTED: uncharacterized protein LOC104898376 [Beta vulgaris subsp. vulgaris] >KMT06804.1 hypothetical protein BVRB_7g159620 [Beta vulgaris subsp. vulgaris]</td><td>-</td><td>A0A0J8C3H0|A0A0J8C3H0_BETVU:Uncharacterized protein;OS=Beta vulgaris subsp. vulgaris.;|GO:0016021,</td></tr>
<tr><td>DN114322_c0_g1_i5</td><td>DOWN</td><td>-</td><td>-</td><td>-</td><td>XP_021775279.1:uncharacterized protein LOC110739131 isoform X1 [Chenopodium quinoa]</td><td>-</td><td>A0A0K9R3E0|A0A0K9R3E0_SPIOL:Uncharacterized protein;OS=Spinacia oleracea;</td></tr>
<tr><td>DN128542_c0_g1_i4</td><td>DOWN</td><td>-</td><td>-</td><td>-</td><td>XP_010682959.1:PREDICTED: uncharacterized protein LOC104897720 isoform X1 [Beta vulgaris subsp.</td><td>-</td><td>A0A0J8BZA1|A0A0J8BZA1_BETVU:Uncharacterized protein;OS=Beta vulgaris subsp. vulgaris.;</td></tr>
</table>

geneid	trend	GO	Pathway	TF	NR	NT	Uniprot
					vulgaris]		
DN125212_c1_g1_i7	DOWN	[GO:0016021:integral component of membrane][GO:0005215:transporter activity]	-	mrna31023.1-v1.0-hybrid:Fragaria vesca|C3H|C3H family protein	XP_010669517.1:PREDICTED: protein NRT1/ PTR FAMILY 6.4 [Beta vulgaris subsp. vulgaris] >KMT17797.1 hypothetical protein BVRB_2g035420 [Beta vulgaris subsp. vulgaris]	gi|521738019|gb|GAIA01007792.1|:TSA: Salicornia europaea Unigene8112_Se200S transcribed RNA sequence	A0A0J8CW21|A0A0J8CW21_BETVU: Uncharacterized protein;OS=Beta vulgaris subsp. vulgaris.;|GO:0016021,|GO:0005215,

geneid	trend	GO	Pathway	TF	NR	NT	Uniprot
DN119997_c4_g1_i6	DOWN	[GO:0008061:chitin binding][GO:0004568:chitinase activity][GO:0005975:carbohydrate metabolic process][GO:0016998:cell wall macromolecule catabolic process][GO:0006032:chitin catabolic process]	[ko00520:Amino sugar and nucleotide sugar metabolism]	-	XP_010069260.1:PREDICTED: endochitinase EP3 [Eucalyptus grandis] >KCW57557.1 hypothetical protein EUGRSUZ_H00326 [Eucalyptus grandis]	-	A0A059AUC3|A0A059AUC3_EUCGR: Uncharacterized protein;OS=Eucalyptus grandis;|GO:0008061,GO:0004568,|GO:0005975,GO:0016998,GO:0006032,
DN129138_c7_g13_i1	DOWN	[GO:0004568:chitinase activity][GO:0005975:carbohydrate metabolic process][GO:0016998:cell wall macromolecule catabolic process][GO:0006032:chitin catabolic process]	[ko00520:Amino sugar and nucleotide sugar metabolism]	-	XP_015868633.1:PREDICTED: endochitinase EP3like [Ziziphus jujuba]	-	Q7M1Q9|Q7M1Q9_PHYAM:Chitinase A;OS=Phytolacca americana;|GO:0004568,|GO:0005975,GO:0016998,GO:0006032,
DN122030_c1_g2_i1	DOWN	[GO:0046872:metal ion binding][GO:0030001:metal ion transport]	-	Zjn_sc00020.1.g02760.1.am.mk:Zoysia japonica|MYB_related|MYB_related family protein	XP_021858541.1:heavy metalassociated isoprenylated plant protein 9 [Spinacia oleracea] >KNA13094.1 hypothetical protein SOVF_119880	gi|502095841|ref|XM_004490494.1|:PREDICTED: Cicer arietinum myb-like protein X-like (LOC101513750), mRNA	A0A0K9R2B9|A0A0K9R2B9_SPIOL:Uncharacterized protein;OS=Spinacia oleracea;|GO:0046872,|GO:0030001,

geneid	trend	GO	Pathway	TF	NR	NT	Uniprot
					[Spinacia oleracea]		
DN121010_c0_g1_i1	DOWN	[GO:0016747:transferase activity, transferring acyl groups other than amino-acyl groups]	-	-	XP_021852926.1:benzyl alcohol Obenzoyltransferase like [Spinacia oleracea] >KNA08666.1 hypothetical protein SOVF_160600 [Spinacia oleracea]	-	A0A0K9QQ73|A0A0K9QQ73_SPIOL:Uncharacterized protein;OS=Spinacia oleracea;|GO:0016747,
DN119706_c4_g3_i1	DOWN	-	-	-	-	-	-

geneid	trend	GO	Pathway	TF	NR	NT	Uniprot
DN126986_c3_g1_i2	DOWN	[GO:0016021:integral component of membrane]	-	-	XP_008393993.1:PREDICTED: Oglucosyltransferase rumi homolog [Malus domestica] >XP_008353980.1 PREDICTED: Oglucosyltransferase rumi homolog [Malus domestica]	gi|297824638|ref|XM_002880156.1|:Arabidopsis lyrata subsp. lyrata hypothetical protein, mRNA	M5X7Y2|M5X7Y2_PRUPE:Uncharacterized protein;OS=Prunus persica;|GO:0016021,
DN111343_c0_g2_i2	DOWN	[GO:0016021:integral component of membrane]	-	-	XP_021848902.1:translocator protein homolog [Spinacia oleracea] >KNA22091.1 hypothetical protein SOVF_037110 [Spinacia oleracea]	-	A0A0K9RRC3|A0A0K9RRC3_SPIOL: Uncharacterized protein;OS=Spinacia oleracea;|GO:0016021,
DN128881_c3_g1_i10	DOWN	[GO:0016021:integral component of membrane][GO:0008270:zinc ion binding]	-	Oropetium_20150105_23793A:Oropetium thomaeum|C3H|C3H family protein	XP_021745986.1:uncharacterized protein LOC110711854 [Chenopodium quinoa]	gi|470117775|ref|XM_004294977.1|:PREDICTED: Fragaria vesca subsp. vesca uncharacterized LOC101315129 (LOC101315129),	A0A0J8BKZ3|A0A0J8BKZ3_BETVU:Uncharacterized protein;OS=Beta vulgaris subsp. vulgaris.;|GO:0016021,|GO:0008270,

geneid	trend	GO	Pathway	TF	NR	NT	Uniprot
						mRNA	
DN125757_c0_g4_i3	DOWN	-	-	-	XP_021762843.1:C2 and GRAM domaincontaining protein At5g50170like [Chenopodium quinoa]	-	A0A0K9RHW2|A0A0K9RHW2_SPIOL:Uncharacterized protein;OS=Spinacia oleracea;
DN122008_c8_g2_i2	DOWN	-	-	-	-	-	-
DN128786_c1_g1_i3	DOWN	-	-	-	XP_021759112.1:uncharacterized protein LOC110724013 [Chenopodium quinoa]	-	D7THD8|D7THD8_VITVI:Putative uncharacterized protein;OS=Vitis vinifera;GN=VIT_08s0007g01560 {ECO:0000313|EMBL:CBI30454.3};

geneid	trend	GO	Pathway	TF	NR	NT	Uniprot
DN128058_c2_g5_i9	DOWN	[GO:0016747:transferase activity, transferring acyl groups other than amino-acyl groups]	-	Cagra.1914s0012.1.p:Capsella grandiflora|GeBP|GeBP family protein	XP_012569164.1:PREDICTED: acyltransferaselike protein At3g26840, chloroplastic [Cicer arietinum]	-	G7KW54|G7KW54_MEDTR:Esterase/lipase/thioesterase family protein;OS=Medicago truncatula;GN=MTR_7g083200 {ECO:0000313|EMBL:AES80654.1};|GO:0016747,
DN115761_c1_g2_i7	DOWN	[GO:0008270:zinc ion binding]	-	KN540739.1_FGP003:Oryza longistaminata|M-type_MADS|M-type_MADS family protein	XP_010682336.1:PREDICTED: E3 ubiquitinprotein ligase RGLG2 isoform X1 [Beta vulgaris subsp. vulgaris] >XP_010682338.1 PREDICTED: E3 ubiquitinprotein ligase RGLG2 isoform X1 [Beta vulgaris subsp. vulgaris]	gi|521539473|gb|GAHZ01003542.1|:TSA: Salicornia europaea Unigene3814_SeCKS transcribed RNA sequence	A0A0J8C5V9|A0A0J8C5V9_BETVU:Uncharacterized protein;OS=Beta vulgaris subsp. vulgaris.;|GO:0008270,

geneid	trend	GO	Pathway	TF	NR	NT	Uniprot
					>KMT07589.1 hypothetical protein BVRB_6g147210 [Beta vulgaris subsp. vulgaris]		
DN122008_c9_g6_i1	DOWN	-	-	-	-	-	-

geneid	trend	GO	Pathway	TF	NR	NT	Uniprot
DN114217_c5_g2_i1	DOWN	[GO:0048046:apoplast][GO:0005618:cell wall][GO:0004553:hydrolase activity, hydrolyzing O-glycosyl compounds][GO:0016762:xyloglucan:xyloglucosyl transferase activity][GO:0042546:cell wall biogenesis][GO:0071555:cell wall organization][GO:0010411:xyloglucan metabolic process]	-	Sme2.5_00096.1_g00018.1:Solanum melongena|NAC|NAC family protein	XP_009354593.1:PREDICTED: probable xyloglucan endotransglucosylase/hydrolase protein 6 [Pyrus x bretschneideri] >XP_009354596.1 PREDICTED: probable xyloglucan endotransglucosylase/hydrolase protein 6 [Pyrus x bretschneideri]	gi|308229783|gb|HM749061.1|:Gossypium hirsutum cultivar Coker 312 xyloglucan endotransglucosylase/hydrolase (XTH2) mRNA, complete cds	G5DAC8|G5DAC8_DIOKA:Xyloglucan endotransglucosylase/hydrolase;OS=Diospyros kaki;|GO:0048046,GO:0005618,|GO:0004553,GO:0016762,|GO:0042546,GO:0071555,GO:0010411,
DN122974_c7_g12_i1	DOWN	[GO:0045735:nutrient reservoir activity]	-	-	XP_021733866.1:11S globulin seed storage protein 2like [Chenopodium quinoa]	-	A0A0J8F4N5|A0A0J8F4N5_BETVU:Uncharacterized protein;OS=Beta vulgaris subsp. vulgaris.;|GO:0045735,

geneid	trend	GO	Pathway	TF	NR	NT	Uniprot
DN110475_c0_g1_i1	DOWN	-	-	-	XP_021846847.1:uncharacterized protein LOC110786604 [Spinacia oleracea]	-	A0A0J8EF31|A0A0J8EF31_BETVU:Uncharacterized protein;OS=Beta vulgaris subsp. vulgaris.;
DN121693_c0_g5_i2	DOWN	[GO:0016758:transferase activity, transferring hexosyl groups][GO:0008152:metabolic process]	[ko00908:Zeatin biosynthesis]	Tp57577_TGAC_v2_mRNA33215:Trifolium pratense|bHLH|bHLH family protein	XP_021741034.1:zeatin Oglucosyltransferase like [Chenopodium quinoa]	-	A0A0J8CMW1|A0A0J8CMW1_BETVU:Glycosyltransferase;OS=Beta vulgaris subsp. vulgaris.;|GO:0016758,|GO:0008152,
DN116837_c2_g1_i2	DOWN	[GO:0016021:integral component of membrane][GO:0005215:transporter activity]	-	-	AAD31847.1:water channel protein MipI [Mesembryanthemum crystallinum]	gi|4884865|gb|AF133531.1|AF133531:Mesembryanthemum crystallinum water channel protein MipI (MipI) mRNA, complete cds	Q9XF75|Q9XF75_MESCR:Water channel protein MipI;OS=Mesembryanthemum crystallinum;|GO:0016021,|GO:0005215,

geneid	trend	GO	Pathway	TF	NR	NT	Uniprot
DN123972_c0_g5_i5	DOWN	[GO:0005737:cytoplasm][GO:0016021:integral component of membrane]	-	Dca5233.1:Dianthus caryophyllus|MIKC_MADS|MIKC_MADS family protein	XP_003516326.1:PREDICTED: uncharacterized protein At1g04910like [Glycine max] >KRH75793.1 hypothetical protein GLYMA_01G109800 [Glycine max]	gi|502146860|ref|XM_004506583.1|:PREDICTED: Cicer arietinum uncharacterized LOC101488794 (LOC101488794), mRNA	K7K347|K7K347_SOYBN:Uncharacterized protein;OS=Glycine max;|GO:0005737,GO:0016021,
DN128453_c2_g2_i3	DOWN	[GO:0005524:ATP binding][GO:0004003:ATP-dependent DNA helicase activity][GO:0003677:DNA binding][GO:0006289:nucleotide-excision repair][GO:0006367:transcription initiation from RNA polymerase II promoter]	-	-	KNA08100.1:hypothetical protein SOVF_165390 [Spinacia oleracea]	-	A0A0K9QLI0|A0A0K9QLI0_SPIOL:Uncharacterized protein;OS=Spinacia oleracea;|GO:0005524,GO:0004003,GO:0003677,|GO:0006289,GO:0006367,

geneid	trend	GO	Pathway	TF	NR	NT	Uniprot
DN129195_c59_g1_i3	DOWN	-	[ko00902:Monoterpenoid biosynthesis]	Niben101Scf02581g01014.1:Nicotiana benthamiana|MYB_related|MYB_related family protein	XP_012843683.1:PREDICTED: (+)neomenthol dehydrogenaselike isoform X1 [Erythranthe guttata] >EYU32077.1 hypothetical protein MIMGU_mgv1a010747mg [Erythranthe guttata]	-	A0A022QTR3|A0A022QTR3_ERYGU: Uncharacterized protein;OS=Erythranthe guttata;
DN98441_c0_g1_i1	DOWN	[GO:0016021:integral component of membrane][GO:0005215:transporter activity]	-	-	XP_021662216.1:probable aquaporin PIP22 [Hevea brasiliensis]	gi|502118465|ref|XM_004496224.1|:PREDICTED: Cicer arietinum aquaporin PIP2-1-like (LOC101488859), mRNA	A0A067LAW1|A0A067LAW1_JATCU: Uncharacterized protein;OS=Jatropha curcas;|GO:0016021,|GO:0005215,
DN121966_c5_g1_i1	DOWN	-	-	-	-	-	-

geneid	trend	GO	Pathway	TF	NR	NT	Uniprot
DN127160_c0_g5_i9	DOWN	-	-	mrna28785.1-v1.0-hybrid:Fragaria vesca|bHLH|bHLH family protein	XP_021745924.1:probable aldoketo reductase 4 [Chenopodium quinoa]	gi|225433669|ref|XM_002265891.1|:PREDICTED: Vitis vinifera auxin-induced protein PCNT115-like, transcript variant 1 (LOC100246287), mRNA	A0A0J8CN69|A0A0J8CN69_BETVU:Uncharacterized protein;OS=Beta vulgaris subsp. vulgaris.;
DN123314_c4_g1_i2	DOWN	[GO:0003824:catalytic activity]	[ko00052:Galactose metabolism]	-	XP_021760751.1:probable galactinolsucrose galactosyltransferase 6 isoform X2 [Chenopodium quinoa]	gi|359484222|ref|XM_002285382.2|:PREDICTED: Vitis vinifera probable galactinol--sucrose galactosyltransferase 6-like (LOC100268134), mRNA	A0A0J8BN50|A0A0J8BN50_BETVU:Uncharacterized protein;OS=Beta vulgaris subsp. vulgaris.;|GO:0003824,
DN126341_c5_g8_i4	DOWN	[GO:0005576:extracellular region][GO:0030145:manganese ion binding][GO:0045735:nutrient reservoir activity]	-	-	XP_021858456.1:auxinbinding protein ABP19alike [Spinacia oleracea]	-	A0A0J8B4R9|A0A0J8B4R9_BETVU:Uncharacterized protein;OS=Beta vulgaris subsp. vulgaris.;|GO:00055

geneid	trend	GO	Pathway	TF	NR	NT	Uniprot
							76,|GO:0030145,GO:0045735,
DN120474_c0_g2_i1	DOWN	[GO:0016021:integral component of membrane][GO:0004553:hydrolase activity, hydrolyzing O-glycosyl compounds][GO:0005975:carbohydrate metabolic process]	[ko00520:Amino sugar and nucleotide sugar metabolism]	-	XP_010687471.1:PREDICTED: betaxylosidase/alpha Larabinofuranosidase 2 [Beta vulgaris subsp. vulgaris] >KMT03688.1 hypothetical protein BVRB_8g190250 [Beta vulgaris subsp. vulgaris]	gi|255545292|ref|XM_002513661.1|:Ricinus communis Beta-glucosidase, putative, mRNA	A0A0J8BUU1|A0A0J8BUU1_BETVU: Uncharacterized protein;OS=Beta vulgaris subsp. vulgaris.;|GO:0016021,|GO:0004553,|GO:0005975,

geneid	trend	GO	Pathway	TF	NR	NT	Uniprot
DN125485_c0_g3_i4	DOWN	[GO:0097008:(3E)-4,8-dimethyl-1,3,7-nonatriene synthase activity][GO:0097007:4,8,12-trimethyltrideca-1,3,7,11-tetraene synthase activity][GO:0020037:heme binding][GO:0005506:iron ion binding][GO:0046246:terpene biosynthetic process]	[ko00904:Diterpenoid biosynthesis]	Cc10_g03650:Coffea canephora|G2-like|G2-like family protein	XP_010696237.1:PREDICTED: cytochrome P450 82G1 [Beta vulgaris subsp. vulgaris] >KMS97083.1 hypothetical protein BVRB_7g178650 [Beta vulgaris subsp. vulgaris]	gi|521441879|gb|GAHZ01078605.1|:TSA: Salicornia europaea Unigene96237_SeCKS transcribed RNA sequence	A0A0J8BAT5|A0A0J8BAT5_BETVU:Uncharacterized protein;OS=Beta vulgaris subsp. vulgaris.;|GO:0097008,GO:0097007,GO:0020037,GO:0005506,|GO:0046246,
DN120100_c0_g7_i1	DOWN	-	-	-	XP_010685294.1:PREDICTED: uncharacterized protein LOC104899736 [Beta vulgaris subsp. vulgaris] >KMT04809.1 hypothetical protein BVRB_7g169800 [Beta vulgaris subsp. vulgaris]	gi|224090468|ref|XM_002308955.1|:Populus trichocarpa predicted protein, mRNA	A0A0J8BXT3|A0A0J8BXT3_BETVU:Uncharacterized protein;OS=Beta vulgaris subsp. vulgaris.;

geneid	trend	GO	Pathway	TF	NR	NT	Uniprot
DN128181_c1_g1_i1	DOWN	-	-	-	KDP20462.1:hypothetical protein JCGZ_06007 [Jatropha curcas]	gi|9844608|emb|AJ297573.1|:Phyllocoptes gracilis 18S rRNA gene (partial), 5.8S rRNA gene, 28S rRNA gene (partial), internal transcribed spacer 1 (ITS1) and internal transcribed spacer 2 (ITS2)	R7QUP6|R7QUP6_CHOCR:Uncharacterized protein;OS=Chondrus crispus;
DN114383_c0_g1_i1	DOWN	[GO:0004553:hydrolase activity, hydrolyzing O-glycosyl compounds][GO:0005975:carbohydrate metabolic process]	-	Pbr002083.1:Pyrus bretschneideri|ERF|ERF family protein	XP_010675772.1:PREDICTED: glucan endo1,3betaglucosidase 13 [Beta vulgaris subsp. vulgaris] >KMT12799.1 hypothetical protein BVRB_4g088930 [Beta vulgaris subsp. vulgaris]	gi|512393684|gb|GAJZ01007486.1|:TSA: Prunus avium Pa_44925 transcribed RNA sequence	A0A0J8CL71|A0A0J8CL71_BETVU:Uncharacterized protein;OS=Beta vulgaris subsp. vulgaris.;|GO:0004553,|GO:0005975,

geneid	trend	GO	Pathway	TF	NR	NT	Uniprot
DN115424_c0_g1_i2	DOWN	[GO:0051287:NAD binding][GO:0016616:oxidoreductase activity, acting on the CH-OH group of donors, NAD or NADP as acceptor]	[ko00260:Glycine, serine and threonine metabolism][ko00630:Glyoxylate and dicarboxylate metabolism]	GSBRNA2T00090396001:Brassica napus|bHLH|bHLH family protein	XP_019103210.1:PREDICTED: glyoxylate/hydroxypyruvate reductase HPR3 isoform X2 [Beta vulgaris subsp. vulgaris]	-	A0A0J8B2G0|A0A0J8B2G0_BETVU:Uncharacterized protein;OS=Beta vulgaris subsp. vulgaris.;|GO:0051287,GO:0016616,
DN122974_c7_g12_i2	DOWN	[GO:0045735:nutrient reservoir activity]	-	-	XP_021761604.1:glutelin typeA 3like [Chenopodium quinoa]	-	A0A0J8F4N5|A0A0J8F4N5_BETVU:Uncharacterized protein;OS=Beta vulgaris subsp. vulgaris.;|GO:0045735,
DN127296_c0_g1_i4	DOWN	[GO:0005096:GTPase activator activity][GO:0007165:signal transduction]	-	-	XP_010675320.1:PREDICTED: rho GTPaseactivating protein REN1 isoform X1 [Beta vulgaris subsp. vulgaris] >KMT19730.1 hypothetical protein BVRB_1g007850 [Beta vulgaris subsp.	-	A0A0J8D0S5|A0A0J8D0S5_BETVU:Uncharacterized protein;OS=Beta vulgaris subsp. vulgaris.;|GO:0005096,|GO:0007165,

geneid	trend	GO	Pathway	TF	NR	NT	Uniprot
					vulgaris]		
DN111224_c1_g1_i1	DOWN	[GO:0003978:UDP-glucose 4-epimerase activity][GO:0006012:galactose metabolic process]	[ko00052:Galactose metabolism][ko00520:Amino sugar and nucleotide sugar metabolism]	-	XP_021754129.1:bifunctional UDPglucose 4epimerase and UDPxylose 4epimerase 1like [Chenopodium quinoa]	gi|224074890|ref|XM_002304442.1|:Populus trichocarpa predicted protein, mRNA	A0A0J8CEC3|A0A0J8CEC3_BETVU:Uncharacterized protein;OS=Beta vulgaris subsp. vulgaris.;|GO:0003978,|GO:0006012,

geneid	trend	GO	Pathway	TF	NR	NT	Uniprot
DN119804_c0_g1_i1	DOWN	[GO:0016021:integral component of membrane][GO:0020037:heme binding][GO:0005506:iron ion binding][GO:0004497:monooxygenase activity][GO:0016705:oxidoreductase activity, acting on paired donors, with incorporation or reduction of molecular oxygen]	-	Cc10_g03650:Coffea canephora|G2-like|G2-like family protein	EOY14628.1:Cytochrome P450, family 82, subfamily C, polypeptide 4 [Theobroma cacao]	-	A0A061FCJ9|A0A061FCJ9_THECC:Cytochrome P450, family 82, subfamily C, polypeptide 4;OS=Theobroma cacao;|GO:0016021,|GO:0020037,GO:0005506,GO:0004497,GO:0016705,
DN127142_c4_g22_i1	DOWN	[GO:0004553:hydrolase activity, hydrolyzing O-glycosyl compounds][GO:0005975:carbohydrate metabolic process]	[ko00520:Amino sugar and nucleotide sugar metabolism]	-	KNA13287.1:hypothetical protein SOVF_118080 [Spinacia oleracea]	-	A0A0K9R3A9|A0A0K9R3A9_SPIOL:Uncharacterized protein;OS=Spinacia oleracea;|GO:0004553,|GO:0005975,

geneid	trend	GO	Pathway	TF	NR	NT	Uniprot
DN127657_c2_g1_i2	DOWN	[GO:0004013:adenosylhomocysteinase activity][GO:0051287:NAD binding][GO:0006730:one-carbon metabolic process][GO:0019510:S-adenosylhomocysteine catabolic process]	[ko00270:Cysteine and methionine metabolism]	Seita.6G063700.1.p: Setaria italica|bHLH|bHLH family protein	P93253.1:RecName: Full=Adenosylhomocysteinase; Short=AdoHcyase; AltName: Full=SadenosylLhomocysteine hydrolase >AAB38499.1 SadenosylLhomocystein hydrolase [Mesembryanthemum crystallinum]	gi|1724101|gb|U79766.1|MCU79766:Mesembryanthemum crystallinum S-adenosyl-L-homocystein hydrolase mRNA, complete cds	P93253|SAHH_MESCR:Adenosylhomocysteinase;OS=Mesembryanthemum crystallinum;|GO:0004013,GO:0051287,|GO:0006730,GO:0019510,
DN121183_c2_g5_i1	DOWN	-	-	-	-	-	-
DN128231_c3_g2_i2	DOWN	[GO:0003824:catalytic activity]	[ko00052:Galactose metabolism]	-	XP_021741776.1:probable galactinolsucrose galactosyltransferase 6 [Chenopodium quinoa]	gi|521452359|gb|GAHZ01068125.1|:TSA: Salicornia europaea Unigene82219_SeCKS transcribed RNA sequence	A0A0J8BN50|A0A0J8BN50_BETVU:Uncharacterized protein;OS=Beta vulgaris subsp. vulgaris.;|GO:0003824,

geneid	trend	GO	Pathway	TF	NR	NT	Uniprot
DN119021_c0_g1_i1	DOWN	[GO:0016021:integral component of membrane][GO:0009705:plant-type vacuole membrane]	-	Bv5_101120_supk.t1:Beta vulgaris|ERF|ERF family protein	XP_010686712.1:PREDICTED: monosaccharidesensing protein 2 [Beta vulgaris subsp. vulgaris] >KMT19227.1 hypothetical protein BVRB_1g014090 [Beta vulgaris subsp. vulgaris]	gi|449475696|ref|XM_004154478.1|:PREDICTED: Cucumis sativus monosaccharide-sensing protein 2-like (LOC101212786), mRNA	A0A0J8D069|A0A0J8D069_BETVU:Uncharacterized protein;OS=Beta vulgaris subsp. vulgaris.;|GO:0016021,GO:0009705,|GO:0022891,
DN116103_c2_g1_i5	DOWN	-	-	Zmw_sc01994.1.g00160.1:Zoysia matrella|ZF-HD|ZF-HD family protein	XP_010673717.1:PREDICTED: protein GAST1 isoform X1 [Beta vulgaris subsp. vulgaris] >KMT14572.1 hypothetical protein BVRB_4g073420 [Beta vulgaris subsp. vulgaris]	gi|359482200|ref|XM_003632678.1|:PREDICTED: Vitis vinifera protein GAST1-like (LOC100852723), mRNA	A0A0J8CM35|A0A0J8CM35_BETVU: Uncharacterized protein;OS=Beta vulgaris subsp. vulgaris.;
DN111836_c1_g1_i4	DOWN	-	-	-	-	-	-

geneid	trend	GO	Pathway	TF	NR	NT	Uniprot
DN118057_c1_g5_i1	DOWN	-	-	-	XP_010684938.1:PREDICTED: protein SULFUR DEFICIENCYINDUCED 1 [Beta vulgaris subsp. vulgaris]	gi|388491309|gb|BT133926.1|:Lotus japonicus clone JCVI-FLLj-2P12 unknown mRNA	A0A0J8C068|A0A0J8C068_BETVU:Uncharacterized protein;OS=Beta vulgaris subsp. vulgaris.;
DN121165_c2_g3_i8	DOWN	[GO:0004722:protein serine/threonine phosphatase activity]	-	-	XP_010674092.1:PREDICTED: probable protein phosphatase 2C 34 [Beta vulgaris subsp. vulgaris] >KMT14266.1 hypothetical protein BVRB_4g076220 [Beta vulgaris subsp. vulgaris]	gi|225321289|dbj|AK325500.1|:Solanum lycopersicum cDNA, clone: LEFL1097BB08, HTC in leaf	A0A0J8FEL1|A0A0J8FEL1_BETVU:Uncharacterized protein;OS=Beta vulgaris subsp. vulgaris.;|GO:0004722,
DN125757_c0_g1_i1	DOWN	-	-	-	XP_021762843.1:C2 and GRAM domaincontaining protein At5g50170like [Chenopodium quinoa]	-	A0A0K9RHW2|A0A0K9RHW2_SPIOL:Uncharacterized protein;OS=Spinacia oleracea;

geneid	trend	GO	Pathway	TF	NR	NT	Uniprot
DN127932_c1_g1_i1	DOWN	[GO:0016021:integral component of membrane][GO:0005215:transporter activity]	-	DCAR_011771:Daucus carota|MYB_related|MYB_related family protein	XP_021745833.1:protein NRT1/ PTR FAMILY 3.1like [Chenopodium quinoa]	-	A0A0J8CN99|A0A0J8CN99_BETVU:Uncharacterized protein;OS=Beta vulgaris subsp. vulgaris.;|GO:0016021,|GO:0005215,
DN119523_c0_g1_i1	DOWN	[GO:0048046:apoplast][GO:0005507:copper ion binding][GO:0052716:hydroquinone:oxygen oxidoreductase activity][GO:0046274:lignin catabolic process]	-	Rsa1.0_03034.1_g00001.1:Raphanus sativus|ERF|ERF family protein	XP_010690891.1:PREDICTED: laccase17 [Beta vulgaris subsp. vulgaris] >KMT18357.1 hypothetical protein BVRB_2g025130 [Beta vulgaris subsp. vulgaris]	gi|225449453|ref|XM_002278239.1|:PREDICTED: Vitis vinifera laccase-17-like (LOC100251378), mRNA	A0A0J8CXN4|A0A0J8CXN4_BETVU:Laccase;OS=Beta vulgaris subsp. vulgaris.;|GO:0048046,|GO:0005507,GO:0052716,|GO:0046274,

geneid	trend	GO	Pathway	TF	NR	NT	Uniprot
DN125678_c2_g2_i1	DOWN	[GO:0016021:integral component of membrane][GO:0008375:acetylglucosaminyltransferase activity]	-	Achn337101:Actinidia chinensis|Dof|Dof family protein	XP_010694976.1:PREDICTED: betaglucuronosyltransferase GlcAT14B [Beta vulgaris subsp. vulgaris] >KMS97985.1 hypothetical protein BVRB_4g096670 [Beta vulgaris subsp. vulgaris]	-	A0A0J8E4D2|A0A0J8E4D2_BETVU:Uncharacterized protein;OS=Beta vulgaris subsp. vulgaris.;|GO:0016021,|GO:0008375,
DN124859_c2_g3_i2	DOWN	-	-	-	-	-	-
DN118864_c1_g4_i2	DOWN	[GO:0016021:integral component of membrane][GO:0005215:transporter activity]	-	Prupe.3G009400.1.p:Prunus persica|ERF|ERF family protein	XP_015931337.1:probable aquaporin PIPtype 7a [Arachis duranensis] >XP_016166069.1 probable aquaporin PIPtype 7a [Arachis ipaensis]	gi|470143928|ref|XM_004307571.1|:PREDICTED: Fragaria vesca subsp. vesca probable aquaporin PIP-type 7a-like (LOC101295462), mRNA	O65356|O65356_SAMSA:Aquaporin 1;OS=Samanea saman;|GO:0016021,|GO:0005215,

geneid	trend	GO	Pathway	TF	NR	NT	Uniprot
DN119738_c1_g2_i5	DOWN	[GO:0016021:integral component of membrane][GO:0015297:antiporter activity]	-	TRIUR3_11943-P1: Triticum urartu|ERF|ERF family protein	XP_021852220.1:protein DETOXIFICATION 27like [Spinacia oleracea] >KNA21049.1 hypothetical protein SOVF_046790 [Spinacia oleracea]	gi|521543878|gb|GAHZ01001490.1|:TSA: Salicornia europaea Unigene1521_SeCKS transcribed RNA sequence	A0A0K9RQ63|A0A0K9RQ63_SPIOL:Protein DETOXIFICATION;OS=Spinacia oleracea;|GO:0016021,|GO:0015297,GO:0015238,
DN113273_c1_g1_i1	DOWN	-	-	-	-	-	-
DN128128_c3_g4_i1	DOWN	-	-	-	-	-	-
DN123703_c2_g4_i2	DOWN	[GO:0016021:integral component of membrane][GO:0005886:plasma membrane][GO:0015112:nitrate transmembrane transporter activity][GO:0006857:oligopeptide transport][GO:0010167:response to nitrate][GO:0009414:response to water deprivation]	-	mrna31023.1-v1.0-hybrid:Fragaria vesca|C3H|C3H family protein	XP_021768447.1:protein NRT1/ PTR FAMILY 6.3like [Chenopodium quinoa]	-	A0A0J8F9I8|A0A0J8F9I8_BETVU:Uncharacterized protein;OS=Beta vulgaris subsp. vulgaris.;|GO:0016021,GO:0005886,|GO:0015112,|GO:0006857,GO:0010167,GO:0009414,

geneid	trend	GO	Pathway	TF	NR	NT	Uniprot
DN116025_c0_g1_i3	DOWN	-	-	-	-	-	-
DN123419_c0_g1_i4	DOWN	[GO:0016021:integral component of membrane]	-	-	XP_002303016.1:hypothetical protein POPTR_0002s23870g [Populus trichocarpa] >EEE82289.1 hypothetical protein POPTR_0002s23870g [Populus trichocarpa]	gi|470137708|ref|XM_004304557.1|:PREDICTED: Fragaria vesca subsp. vesca uncharacterized LOC101315363 (LOC101315363), mRNA	B9GNF9|B9GNF9_POPTR:Uncharacterized protein;OS=Populus trichocarpa;|GO:0016021,

geneid	trend	GO	Pathway	TF	NR	NT	Uniprot
DN129123_c0_g7_i2	DOWN	[GO:0016021:integral component of membrane][GO:0000325:plant-type vacuole][GO:0009506:plasmodesma][GO:0005774:vacuolar membrane][GO:0005509:calcium ion binding][GO:0005432:calcium:sodium antiporter activity][GO:0055074:calcium ion homeostasis][GO:0071472:cellular response to salt stress]	-	Zmw_sc02027.1.g00100.1:Zoysia matrella|bZIP|bZIP family protein	XP_010675392.1:PREDICTED: uncharacterized protein LOC104891403 [Beta vulgaris subsp. vulgaris] >KMT13179.1 hypothetical protein BVRB_4g085850 [Beta vulgaris subsp. vulgaris]	gi|521508627|gb|GAHZ01022293.1|:TSA: Salicornia europaea Unigene23973_SeCKS transcribed RNA sequence	A0A0J8CMD2|A0A0J8CMD2_BETVU: Uncharacterized protein;OS=Beta vulgaris subsp. vulgaris.;|GO:0016021,GO:0000325,GO:0009506,GO:0005774,|GO:0005509,GO:0005432,|GO:0055074,GO:0071472,
DN119489_c4_g8_i3	DOWN	-	-	-	-	-	-
DN122397_c4_g3_i1	DOWN	-	-	-	-	-	-

geneid	trend	GO	Pathway	TF	NR	NT	Uniprot
DN129116_c5_g1_i1	DOWN	[GO:0050356:tropine dehydrogenase activity][GO:0050358:tropinone reductase activity]	[ko00960:Tropane, piperidine and pyridine alkaloid biosynthesis]	XP_010558920.1:Tarenaya hassleriana|C2H2|C2H2 family protein	XP_021755762.1:tropinone reductase homolog At5g06060like [Chenopodium quinoa] >XP_021755764.1 tropinone reductase homolog At5g06060like [Chenopodium quinoa] >XP_021755765.1 tropinone reductase homolog At5g06060like [Chenopodium quinoa]	-	A7DY56|TRN1_COCOF:Tropinone reductase;OS=Cochlearia officinalis;|GO:0050356,GO:0050358,

geneid	trend	GO	Pathway	TF	NR	NT	Uniprot
DN114841_c0_g1_i1	DOWN	[GO:0048046:apoplast][GO:0005618:cell wall][GO:0004553:hydrolase activity, hydrolyzing O-glycosyl compounds][GO:0016762:xyloglucan:xyloglucosyl transferase activity][GO:0042546:cell wall biogenesis][GO:0071555:cell wall organization][GO:0010411:xyloglucan metabolic process]	-	Sme2.5_00096.1_g00018.1:Solanum melongena|NAC|NAC family protein	XP_010673363.1:PREDICTED: xyloglucan endotransglucosylase/hydrolase 2 [Beta vulgaris subsp. vulgaris] >KMT14799.1 hypothetical protein BVRB_3g065250 [Beta vulgaris subsp. vulgaris]	gi|521665693|gb|GAIA01034692.1|:TSA: Salicornia europaea Unigene38400_Se200S transcribed RNA sequence	A0A0J8CRX9|A0A0J8CRX9_BETVU: Xyloglucan endotransglucosylase/hydrolase;OS=Beta vulgaris subsp. vulgaris.;|GO:0048046,GO:0005618,|GO:0004553,GO:0016762,|GO:0042546,GO:0071555,GO:0010411,
DN119738_c1_g2_i3	DOWN	[GO:0016021:integral component of membrane][GO:0015297:antiporter activity]	-	TRIUR3_11943-P1: Triticum urartu|ERF|ERF family protein	XP_010681038.1:PREDICTED: protein DETOXIFICATION 27 [Beta vulgaris subsp. vulgaris] >KMT08611.1 hypothetical protein BVRB_6g138880 [Beta vulgaris subsp. vulgaris]	gi|521543878|gb|GAHZ01001490.1|:TSA: Salicornia europaea Unigene1521_SeCKS transcribed RNA sequence	A0A0K9RQ63|A0A0K9RQ63_SPIOL:Protein DETOXIFICATION;OS=Spinacia oleracea;|GO:0016021,|GO:0015297,GO:0015238,

geneid	trend	GO	Pathway	TF	NR	NT	Uniprot
DN123455_c1_g1_i3	DOWN	-	-	MDP0000574105:Malus domestica|LBD|LBD family protein	XP_021764790.1:myblike protein X [Chenopodium quinoa]	-	A0A0K9Q9D4|A0A0K9Q9D4_SPIOL:Uncharacterized protein;OS=Spinacia oleracea;
DN118252_c0_g4_i2	DOWN	[GO:0016021:integral component of membrane][GO:0005509:calcium ion binding][GO:0016174:NAD(P)H oxidase activity][GO:0004601:peroxidase activity][GO:0009408:response to heat][GO:0009845:seed germination]	[ko04016:MAPK signaling pathway - plant][ko04626:Plant-pathogen interaction]	-	XP_021742555.1:respiratory burst oxidase homolog protein Blike [Chenopodium quinoa]	gi|521674929|gb|GAIA01029936.1|:TSA: Salicornia europaea Unigene32832_Se200S transcribed RNA sequence	A0A0J8FEF7|A0A0J8FEF7_BETVU:Uncharacterized protein;OS=Beta vulgaris subsp. vulgaris.;|GO:0016021,|GO:0005509,GO:0016174,GO:0004601,|GO:0009408,GO:0009845,
DN96253_c0_g1_i1	DOWN	-	-	-	XP_010679295.1:PREDICTED: uncharacterized protein LOC104894701 [Beta vulgaris subsp. vulgaris] >KMT19674.1 hypothetical protein BVRB_1g010600	-	A0A0J8D5Q1|A0A0J8D5Q1_BETVU:Uncharacterized protein;OS=Beta vulgaris subsp. vulgaris.;

geneid	trend	GO	Pathway	TF	NR	NT	Uniprot
					[Beta vulgaris subsp. vulgaris]		
DN106136_c0_g1_i1	DOWN	[GO:0004568:chitinase activity][GO:0005975:carbohydrate metabolic process][GO:0016998:cell wall macromolecule catabolic process][GO:0006032:chitin catabolic process]	[ko00520:Amino sugar and nucleotide sugar metabolism]	-	P42820.1:RecName: Full=Acidic endochitinase SP2; Flags: Precursor >AAA32916.1 chitinase [Beta vulgaris]	-	Q7M1Q9|Q7M1Q9_PHYAM:Chitinase A;OS=Phytolacca americana;|GO:0004568,|GO:0005975,GO:0016998,GO:0006032,
DN17029_c0_g1_i1	DOWN	-	-	-	-	-	-
DN122685_c2_g3_i3	DOWN	-	-	-	-	-	-

geneid	trend	GO	Pathway	TF	NR	NT	Uniprot
DN102099_c0_g1_i1	DOWN	-	[ko00902:Monoterpenoid biosynthesis]	-	XP_019055105.1:PREDICTED: LOW QUALITY PROTEIN: (+)neomenthol dehydrogenaselike [Nelumbo nucifera]	-	A0A0L9VR26|A0A0L9VR26_PHAAN: Uncharacterized protein;OS=Phaseolus angularis;
DN88671_c0_g2_i1	DOWN	-	-	-	XP_010691165.1:PREDICTED: uncharacterized protein LOC104904576 [Beta vulgaris subsp. vulgaris] >KMT00689.1 hypothetical protein BVRB_9g219770 [Beta vulgaris subsp. vulgaris]	-	A0A0J8BKW7|A0A0J8BKW7_BETVU: Uncharacterized protein;OS=Beta vulgaris subsp. vulgaris.;
DN106388_c0_g1_i1	DOWN	-	-	-	-	-	-

geneid	trend	GO	Pathway	TF	NR	NT	Uniprot
DN122974_c7_g12_i3	DOWN	[GO:0045735:nutrient reservoir activity]	-	-	XP_021733866.1:11S globulin seed storage protein 2like [Chenopodium quinoa]	-	A0A0J8F4N5|A0A0J8F4N5_BETVU:Uncharacterized protein;OS=Beta vulgaris subsp. vulgaris.;|GO:0045735,
DN108359_c0_g1_i1	DOWN	-	-	-	XP_015065205.1:PREDICTED: uncharacterized protein LOC107010435 [Solanum pennellii]	-	B9H2E9|B9H2E9_POPTR:Uncharacterized protein;OS=Populus trichocarpa;
DN121427_c0_g2_i10	DOWN	[GO:0016021:integral component of membrane][GO:0005778:peroxisomal membrane][GO:0016560:protein import into peroxisome matrix, docking]	[ko04146:Peroxisome]	MDP0000166392:Malus domestica|MYB_related|MYB_related family protein	XP_010678761.1:PREDICTED: peroxisomal membrane protein PEX14 isoform X2 [Beta vulgaris subsp. vulgaris]	-	A0A0J8CAF2|A0A0J8CAF2_BETVU:Uncharacterized protein;OS=Beta vulgaris subsp. vulgaris.;|GO:0016021,GO:0005778,|GO:0016560,

geneid	trend	GO	Pathway	TF	NR	NT	Uniprot
DN122398_c0_g1_i1	DOWN	[GO:0004553:hydrolase activity, hydrolyzing O-glycosyl compounds][GO:0005975:carbohydrate metabolic process]	[ko00460:Cyanoamino acid metabolism][ko00500:Starch and sucrose metabolism][ko00940:Phenylpropanoid biosynthesis]	676737120:Sisymbrium irio|NF-YC|NF-YC family protein	KMT06914.1:hypothetical protein BVRB_6g152510 [Beta vulgaris subsp. vulgaris]	-	A0A0J8C3U9|A0A0J8C3U9_BETVU:Uncharacterized protein;OS=Beta vulgaris subsp. vulgaris.;|GO:0004553,|GO:0005975,
DN127560_c3_g3_i1	DOWN	[GO:0016210:naringenin-chalcone synthase activity][GO:0009813:flavonoid biosynthetic process]	[ko00941:Flavonoid biosynthesis][ko04712:Circadian rhythm - plant]	-	Q9ZRS4.1:RecName: Full=Chalcone synthase; AltName: Full=Naringeninchalcone synthase >CAA10511.1 chalcone synthase [Catharanthus roseus]	gi|77994621|gb|DQ205352.1|:Rheum palmatum chalcone synthase 1 (CHS1) mRNA, complete cds	Q9ZRS4|CHSY_CATRO:Chalcone synthase;OS=Catharanthus roseus;|GO:0016210,|GO:0009813,
DN127168_c1_g6_i1	DOWN	[GO:0016021:integral component of membrane][GO:0005524:ATP binding][GO:0016887:ATPase activity]	-	MDP0000265619:Malus domestica|Nin-like|Nin-like family protein	XP_021716840.1:LOW QUALITY PROTEIN: ABC transporter G family member 6like [Chenopodium quinoa]	-	A0A0J8FP55|A0A0J8FP55_BETVU:Uncharacterized protein;OS=Beta vulgaris subsp. vulgaris.;|GO:0016021,|GO:0005524,GO:0016887,

geneid	trend	GO	Pathway	TF	NR	NT	Uniprot
DN124946_c0_g1_i2	DOWN	-	-	DCAR_006074:Daucus carota|MYB_related|MYB_related family protein	XP_010670661.1:PREDICTED: putative ripeningrelated protein 1 [Beta vulgaris subsp. vulgaris] >KMT20501.1 hypothetical protein BVRB_1g004870 [Beta vulgaris subsp. vulgaris]	-	A0A0J8FXY7|A0A0J8FXY7_BETVU:Uncharacterized protein;OS=Beta vulgaris subsp. vulgaris.;
DN116488_c4_g4_i1	DOWN	-	-	-	XP_021725000.1:uncharacterized protein LOC110692302 [Chenopodium quinoa] >XP_021730738.1 uncharacterized protein LOC110697658 [Chenopodium quinoa]	gi|28416780|gb|BT004675.1|:Arabidopsis thaliana At4g16146 gene, complete cds	A0A0D2TAM8|A0A0D2TAM8_GOSRA:Uncharacterized protein;OS=Gossypium raimondii;

geneid	trend	GO	Pathway	TF	NR	NT	Uniprot
DN115687_c1_g2_i1	DOWN	[GO:0000287:magnesium ion binding][GO:0010333:terpene synthase activity][GO:0008152:metabolic process]	[ko00904:Diterpenoid biosynthesis]	-	XP_019106684.1:PREDICTED: (E,E)geranyllinalool synthaselike [Beta vulgaris subsp. vulgaris] >KMT04332.1 hypothetical protein BVRB_8g184070 [Beta vulgaris subsp. vulgaris]	-	A0A0J8BT41|A0A0J8BT41_BETVU:Uncharacterized protein;OS=Beta vulgaris subsp. vulgaris.;|GO:0000287,GO:0010333,|GO:0008152,
DN127683_c1_g7_i2	DOWN	-	-	-	XP_021755289.1:thaumatinlike protein 1b [Chenopodium quinoa]	gi|356552061|ref|XM_003544342.1|:PREDICTED: Glycine max thaumatin-like protein 1-like (LOC100787631), mRNA	A0A0J8BJP1|A0A0J8BJP1_BETVU:Uncharacterized protein;OS=Beta vulgaris subsp. vulgaris.;
DN114658_c0_g1_i6	DOWN	[GO:0004805:trehalose-phosphatase activity][GO:0005992:trehalose biosynthetic process]	[ko00500:Starch and sucrose metabolism]	-	XP_021768421.1:probable trehalosephosphate phosphatase F [Chenopodium quinoa] >XP_021768422.1	gi|470125336|ref|XM_004298611.1|:PREDICTED: Fragaria vesca subsp. vesca probable trehalose-phosphate phosphatase F-like	A0A0J8CKH6|A0A0J8CKH6_BETVU: Trehalose 6-phosphate phosphatase;OS=Beta vulgaris subsp. vulgaris.;|GO:00048

geneid	trend	GO	Pathway	TF	NR	NT	Uniprot
					probable trehalosephosphate phosphatase F [Chenopodium quinoa]	(LOC101314657), mRNA	05,|GO:0005992,
DN129104_c1_g1_i1	DOWN	[GO:0008168:methyltransferase activity]	-	EMT20798:Aegilops tauschii|MYB_related|MYB_related family protein	XP_021848471.1:salicylate carboxymethyltransferaselike [Spinacia oleracea]	gi|58201441|gb|AY741495.1|:Streptosolen jamesonii SAMT (SAMT) mRNA, partial cds	A0A0J8CSG5|A0A0J8CSG5_BETVU:Uncharacterized protein;OS=Beta vulgaris subsp. vulgaris.;|GO:0008168,
DN111173_c0_g1_i1	DOWN	[GO:0016021:integral component of membrane][GO:0045492:xylan biosynthetic process]	-	Itr_sc000772.1_g00020.1:Ipomoea trifida|FAR1|FAR1 family protein	XP_021752329.1:protein IRX15LIKElike [Chenopodium quinoa]	-	A0A0J8CP22|A0A0J8CP22_BETVU:Uncharacterized protein;OS=Beta vulgaris subsp. vulgaris.;|GO:0016021,|GO:0045492,

geneid	trend	GO	Pathway	TF	NR	NT	Uniprot
DN116266_c0_g1_i2	DOWN	[GO:0005634:nucleus][GO:0043565:sequence-specific DNA binding][GO:0003700:DNA-binding transcription factor activity]	-	Bv_000370_cufa.t1:Beta vulgaris|HD-ZIP|HD-ZIP family protein	XP_021763384.1:homeoboxleucine zipper protein ATHB12like [Chenopodium quinoa]	gi|521570380|gb|GAIA01073498.1|:TSA: Salicornia europaea Unigene89334_Se200S transcribed RNA sequence	A0A0J8B8M1|A0A0J8B8M1_BETVU:Uncharacterized protein;OS=Beta vulgaris subsp. vulgaris.;|GO:0005634,|GO:0043565,GO:0003700,
DN121091_c0_g4_i2	DOWN	[GO:0016021:integral component of membrane]	-	-	XP_021836262.1:putative DNA helicase ino80 [Spinacia oleracea] >KNA24380.1 hypothetical protein SOVF_016140 [Spinacia oleracea]	-	A0A0K9RXV8|A0A0K9RXV8_SPIOL:Uncharacterized protein;OS=Spinacia oleracea;|GO:0016021,
DN115022_c1_g1_i2	DOWN	-	-	-	XP_002277345.1:PREDICTED: uncharacterized protein LOC100260838 [Vitis vinifera]	gi|502140636|ref|XM_004504227.1|:PREDICTED: Cicer arietinum uncharacterized LOC101494080 (LOC101494080), mRNA	F6H530|F6H530_VITVI:Putative uncharacterized protein;OS=Vitis vinifera;GN=VIT_12s0028g01720{ECO:0000313|EMBL:CCB47398.1};

geneid	trend	GO	Pathway	TF	NR	NT	Uniprot
DN122230_c4_g1_i2	DOWN	-	-	XP_008235702.1:Prunus mume|bHLH|bHLH family protein	XP_021742053.1:protein RICE SALT SENSITIVE 3like isoform X1 [Chenopodium quinoa]	gi|225898033|dbj|AB493511.1|:Arabidopsis thaliana At1g60060 mRNA for hypothetical protein, partial cds, clone: RAAt1g60060	A0A0K9REW3|A0A0K9REW3_SPIOL:Uncharacterized protein;OS=Spinacia oleracea;
DN122397_c5_g8_i2	DOWN	-	-	-	-	-	-
DN121091_c0_g4_i1	DOWN	[GO:0016021:integral component of membrane]	-	-	XP_021836262.1:putative DNA helicase ino80 [Spinacia oleracea] >KNA24380.1 hypothetical protein SOVF_016140 [Spinacia oleracea]	-	A0A0K9RXV8|A0A0K9RXV8_SPIOL:Uncharacterized protein;OS=Spinacia oleracea;|GO:0016021,
DN120030_c1_g1_i5	DOWN	[GO:0005524:ATP binding][GO:0004672:protein kinase activity]	-	FANhyb_rscf00000217.1.g00006.1:Fragaria x ananassa|WRKY|WRKY family protein	XP_021864011.1:probable serine/threonineprotein kinase PBL12 [Spinacia oleracea] >KNA26125.1	gi|53857146|gb|AY220481.2|:Nicotiana tabacum Avr9/Cf-9 induced kinase 1 (ACIK1) mRNA, complete cds	A0A0K9S302|A0A0K9S302_SPIOL:Uncharacterized protein;OS=Spinacia oleracea;|GO:0005524,GO:0004672,

geneid	trend	GO	Pathway	TF	NR	NT	Uniprot
					hypothetical protein SOVF_000420 [Spinacia oleracea]		
DN122026_c5_g24_i1	DOWN	-	-	-	XP_021755346.1:EG45like domain containing protein [Chenopodium quinoa]	-	A0A022RRW3|A0A022RRW3_ERYGU:Uncharacterized protein;OS=Erythranthe guttata;
DN111740_c0_g1_i1	DOWN	-	-	-	XP_010679579.1:PREDICTED: uncharacterized protein LOC104894915 [Beta vulgaris subsp. vulgaris] >KMT09793.1 hypothetical protein BVRB_6g127730 [Beta vulgaris subsp. vulgaris]	-	A0A0J8CCV2|A0A0J8CCV2_BETVU: Uncharacterized protein;OS=Beta vulgaris subsp. vulgaris.;

geneid	trend	GO	Pathway	TF	NR	NT	Uniprot
DN127099_c6_g6_i8	DOWN	[GO:0005634:nucleus][GO:0004402:histone acetyltransferase activity][GO:0003712:transcription coregulator activity][GO:0008270:zinc ion binding][GO:0006355:regulation of transcription, DNA-templated]	-	GSMUA_Achr10P03980_001:Musa acuminata|C2H2|C2H2 family protein	XP_021720204.1:BTB/POZ and TAZ domaincontaining protein 1like [Chenopodium quinoa]	gi|521738988|gb|GAIA01007306.1|:TSA: Salicornia europaea Unigene7620_Se200S transcribed RNA sequence	A0A0J8BB65|A0A0J8BB65_BETVU:Uncharacterized protein;OS=Beta vulgaris subsp. vulgaris.;|GO:0005634,|GO:0004402,GO:0003712,GO:0008270,|GO:0006355,
DN111663_c0_g1_i2	DOWN	-	-	-	-	-	-
DN110005_c1_g1_i2	DOWN	-	-	-	-	-	-
DN115960_c0_g1_i1	DOWN	[GO:0004553:hydrolase activity, hydrolyzing O-glycosyl compounds][GO:0005975:carbohydrate metabolic process]	[ko00460:Cyanoamino acid metabolism][ko00500:Starch and sucrose metabolism][ko00940:Phenylpropanoid biosynthesis]	Lus10009218:Linum usitatissimum|C2H2|C2H2 family protein	XP_021846064.1:betaglucosidase 44like [Spinacia oleracea] >KNA10671.1 hypothetical protein SOVF_142210 [Spinacia oleracea]	gi|512399917|gb|GAJZ01001253.1|:TSA: Prunus avium Pa_02496 transcribed RNA sequence	A0A0K9QW01|A0A0K9QW01_SPIOL: Uncharacterized protein;OS=Spinacia oleracea;|GO:0004553,|GO:0005975,

geneid	trend	GO	Pathway	TF	NR	NT	Uniprot
DN121733_c2_g1_i6	DOWN	[GO:0005524:ATP binding][GO:0004672:protein kinase activity]	-	FANhyb_rscf00000217.1.g00006.1:Fragaria x ananassa|WRKY|WRKY family protein	XP_021720455.1:serine/threonineprotein kinase RIPKlike [Chenopodium quinoa]	gi|449447360|ref|XM_004141389.1|:PREDICTED: Cucumis sativus putative receptor-like protein kinase At1g72540-like (LOC101218136), mRNA >gi|449486800|ref|XM_004157359.1| PREDICTED: Cucumis sativus putative receptor-like protein kinase At1g72540-like (LOC101218136), mRNA	A0A0K9S302|A0A0K9S302_SPIOL:Uncharacterized protein;OS=Spinacia oleracea;|GO:0005524,GO:0004672,

geneid	trend	GO	Pathway	TF	NR	NT	Uniprot
DN125951_c0_g4_i3	DOWN	[GO:0016758:transferase activity, transferring hexosyl groups][GO:0008152:metabolic process]	[ko00908:Zeatin biosynthesis]	Tp57577_TGAC_v2_mRNA33215:Trifolium pratense|bHLH|bHLH family protein	XP_010668171.1:PREDICTED: zeatin Oglucosyltransferase [Beta vulgaris subsp. vulgaris] >KMS94841.1 hypothetical protein BVRB_014800 [Beta vulgaris subsp. vulgaris]	-	A0A0J8B1G9|A0A0J8B1G9_BETVU:Glycosyltransferase;OS=Beta vulgaris subsp. vulgaris.;|GO:0016758,|GO:0008152,
DN115083_c0_g1_i2	DOWN	[GO:0016021:integral component of membrane][GO:0020037:heme binding][GO:0005506:iron ion binding][GO:0004497:monooxygenase activity][GO:0016705:oxidoreductase activity, acting on paired donors, with incorporation or reduction of molecular oxygen]	[ko00941:Flavonoid biosynthesis][ko00944:Flavone and flavonol biosynthesis]	RrC1570_p1:Raphanus raphanistrum|bHLH|bHLH family protein	AMQ23620.1:flavonoid 3'hydroxylase [Silene littorea]	gi|325551318|gb|HQ290518.1|:Camellia nitidissima flavonoid-3'-hydroxylase (F3'H) mRNA, complete cds	A0A142D8F1|A0A142D8F1_9CARY:Flavonoid 3'hydroxylase;OS=Silene littorea.;|GO:0016021,|GO:0020037,GO:0005506,GO:0004497,GO:0016705,
DN127289_c1_g3_i9	DOWN	-	-	-	-	-	-

geneid	trend	GO	Pathway	TF	NR	NT	Uniprot
DN106139_c0_g1_i1	DOWN	-	-	-	XP_010675751.1:PREDICTED: uncharacterized protein LOC104891715 [Beta vulgaris subsp. vulgaris] >KMT12787.1 hypothetical protein BVRB_4g088810 [Beta vulgaris subsp. vulgaris]	-	A0A0J8CGV3|A0A0J8CGV3_BETVU: Uncharacterized protein;OS=Beta vulgaris subsp. vulgaris.;
DN123703_c2_g4_i3	DOWN	[GO:0016021:integral component of membrane][GO:0005886:plasma membrane][GO:0015112:nitrate transmembrane transporter activity][GO:0006857:oligopeptide transport][GO:0010167:response to nitrate][GO:0009414:response to water deprivation]	-	mrna31023.1-v1.0-hybrid:Fragaria vesca|C3H|C3H family protein	XP_021768447.1:protein NRT1/ PTR FAMILY 6.3like [Chenopodium quinoa]	-	A0A0J8F9I8|A0A0J8F9I8_BETVU:Uncharacterized protein;OS=Beta vulgaris subsp. vulgaris.;|GO:0016021,GO:0005886,|GO:0015112,|GO:0006857,GO:0010167,GO:0009414,

geneid	trend	GO	Pathway	TF	NR	NT	Uniprot
DN127716_c2_g2_i4	DOWN	[GO:0008146:sulfotransferase activity]	-	676777512:Sisymbrium irio|ERF|ERF family protein	XP_021718354.1:cytosolic sulfotransferase 12like [Chenopodium quinoa]	-	A0A0J8C4J2|A0A0J8C4J2_BETVU:Sulfotransferase;OS=Beta vulgaris subsp. vulgaris.;|GO:0008146,
DN128996_c5_g7_i3	DOWN	[GO:0031225:anchored component of membrane][GO:0016021:integral component of membrane][GO:0016049:cell growth][GO:0010215:cellulose microfibril organization]	-	Sopen01g046240.1:Solanum pennellii|M-type_MADS|M-type_MADS family protein	XP_021839524.1:COBRAlike protein 4 [Spinacia oleracea] >KNA23357.1 hypothetical protein SOVF_025620 [Spinacia oleracea]	gi|521511127|gb|GAHZ01019793.1|:TSA: Salicornia europaea Unigene20760_SeCKS transcribed RNA sequence	A0A0K9RWY1|A0A0K9RWY1_SPIOL:Uncharacterized protein;OS=Spinacia oleracea;|GO:0031225,GO:0016021,|GO:0016049,GO:0010215,
DN111416_c0_g1_i1	DOWN	-	-	-	-	-	-
DN124434_c0_g2_i3	DOWN	[GO:0005576:extracellular region][GO:0004650:polygalacturonase activity][GO:0005975:carbohydrate metabolic process][GO:0071555:cell wall organization]	-	Pbr023394.1:Pyrus bretschneideri|C2H2|C2H2 family protein	XP_010691173.1:PREDICTED: probable polygalacturonase At1g80170 isoform X1 [Beta vulgaris subsp. vulgaris] >KMT00695.1	gi|521523545|gb|GAHZ01012441.1|:TSA: Salicornia europaea Unigene13066_SeCKS transcribed RNA sequence	A0A0J8BH27|A0A0J8BH27_BETVU:Uncharacterized protein;OS=Beta vulgaris subsp. vulgaris.;|GO:0005576,|GO:0004650,|GO:0005975,GO:0071

geneid	trend	GO	Pathway	TF	NR	NT	Uniprot
					hypothetical protein BVRB_9g219830 [Beta vulgaris subsp. vulgaris]		555,
DN121969_c2_g3_i5	DOWN	[GO:0046872:metal ion binding][GO:0016491:oxidoreductase activity]	-	676709864:Sisymbrium irio|STAT|STAT family protein	XP_021737922.1:protein SRG1like [Chenopodium quinoa]	-	A0A0K9R3K3|A0A0K9R3K3_SPIOL:Uncharacterized protein;OS=Spinacia oleracea;|GO:0046872,GO:0016491,
DN121767_c1_g1_i12	DOWN	-	-	-	XP_021749610.1:pollenspecific protein SF21like [Chenopodium quinoa]	gi|210142770|dbj|AK285552.1|:Glycine max cDNA, clone: GMFL01-12-E20	A0A0J8D1H1|A0A0J8D1H1_BETVU:Uncharacterized protein;OS=Beta vulgaris subsp. vulgaris.;
DN123742_c1_g5_i3	DOWN	[GO:0003676:nucleic acid binding]	-	XP_008245529.1:Prunus mume|E2F/DP|E2F/DP family protein	XP_010682933.1:PREDICTED: uncharacterized protein	-	F4NCI7|F4NCI7_BETVU:Uncharacterized protein;OS=Beta vulgaris subsp.

geneid	trend	GO	Pathway	TF	NR	NT	Uniprot
					LOC104897695 [Beta vulgaris subsp. vulgaris]		vulgaris.;|GO:0003676,
DN117153_c0_g1_i2	DOWN	[GO:0016021:integral component of membrane][GO:0016746:transferase activity, transferring acyl groups][GO:0008152:metabolic process]	[ko00561:Glycerolipid metabolism][ko00564:Glycerophospholipid metabolism]	-	XP_021772437.1:probable glycerol3phosphate acyltransferase 3 [Chenopodium quinoa]	gi|356553598|ref|XM_003545094.1|:PREDICTED: Glycine max glycerol-3-phosphate acyltransferase 1-like (LOC100803875), mRNA	A0A0J8CCU3|A0A0J8CCU3_BETVU: Uncharacterized protein;OS=Beta vulgaris subsp. vulgaris.;|GO:0016021,|GO:0016746,|GO:0008152,
DN115622_c1_g2_i9	DOWN	-	-	-	XP_010665522.1:PREDICTED: CRIB domaincontaining protein RIC10 isoform X2 [Beta vulgaris subsp. vulgaris] >KMS96712.1 hypothetical protein BVRB_8g200180 isoform A [Beta	gi|521500808|gb|GAHZ01030112.1|:TSA: Salicornia europaea Unigene33160_SeCKS transcribed RNA sequence	A0A0J8BA08|A0A0J8BA08_BETVU:Uncharacterized protein;OS=Beta vulgaris subsp. vulgaris.;

geneid	trend	GO	Pathway	TF	NR	NT	Uniprot
					vulgaris subsp. vulgaris]		
DN122397_c5_g2_i12	DOWN	-	-	-	-	-	-
DN120388_c2_g1_i5	DOWN	[GO:0004553:hydrolase activity, hydrolyzing O-glycosyl compounds][GO:0005975:carbohydrate metabolic process]	-	-	XP_010676175.1:PREDICTED: acid betafructofuranosidase isoform X2 [Beta vulgaris subsp. vulgaris] >KMT12545.1 hypothetical protein BVRB_5g097930 [Beta vulgaris subsp. vulgaris]	gi|21322511|emb|AJ422051.1|:Beta vulgaris mRNA for acid vacuolar invertase (vacinv gene)	A0A0J8CFL4|A0A0J8CFL4_BETVU:Uncharacterized protein;OS=Beta vulgaris subsp. vulgaris.;|GO:0004553,|GO:0005975,

geneid	trend	GO	Pathway	TF	NR	NT	Uniprot
DN103841_c0_g1_i3	DOWN	[GO:0000910:cytokinesis][GO:0000226:microtubule cytoskeleton organization]	-	KZV23864.1:Dorcoceras hygrometricum|LBD|LBD family protein	XP_010682781.1:PREDICTED: 65kDa microtubuleassociated protein 8 [Beta vulgaris subsp. vulgaris] >KMT07471.1 hypothetical protein BVRB_6g150890 [Beta vulgaris subsp. vulgaris]	gi|521697998|gb|GAIA01018431.1|:TSA: Salicornia europaea Unigene19386_Se200S transcribed RNA sequence	A0A0J8EW66|A0A0J8EW66_BETVU: Uncharacterized protein;OS=Beta vulgaris subsp. vulgaris.;|GO:0000910,GO:0000226,
DN113695_c0_g1_i3	DOWN	-	-	-	XP_021752134.1:uncharacterized protein LOC110717681 [Chenopodium quinoa]	-	A0A0K9R8W5|A0A0K9R8W5_SPIOL: Uncharacterized protein;OS=Spinacia oleracea;
DN112977_c0_g1_i2	DOWN	[GO:0005737:cytoplasm][GO:0005886:plasma membrane][GO:0010181:FMN binding][GO:0003955:NAD(P)H dehydrogenase (quinone) activity][GO:0055114:oxidation-reduction process]	[ko00130:Ubiquinone and other terpenoid-quinone biosynthesis]	-	XP_021608505.1:NAD(P)H dehydrogenase (quinone) FQR1 isoform X1 [Manihot esculenta] >OAY54996.1 hypothetical protein MANES_03G11960	gi|224124151|ref|XM_002319222.1|:Populus trichocarpa predicted protein, mRNA	U5GWE6|U5GWE6_POPTR:FLAVODOXIN-LIKE QUINONE REDUCTASE 1 family protein;OS=Populus trichocarpa;|GO:0005737,GO:0005886,|

geneid	trend	GO	Pathway	TF	NR	NT	Uniprot
					0 [Manihot esculenta]		GO:0010181,GO:0003955,|GO:0055114,
DN123314_c4_g1_i1	DOWN	[GO:0003824:catalytic activity]	[ko00052:Galactose metabolism]	-	XP_021760751.1:probable galactinolsucrose galactosyltransferase 6 isoform X2 [Chenopodium quinoa]	gi|521738202|gb|GAIA01007699.1|:TSA: Salicornia europaea Unigene8018_Se200S transcribed RNA sequence	A0A0J8BN50|A0A0J8BN50_BETVU:Uncharacterized protein;OS=Beta vulgaris subsp. vulgaris.;|GO:0003824,

geneid	trend	GO	Pathway	TF	NR	NT	Uniprot
DN119427_c0_g1_i2	DOWN	[GO:0016021:integral component of membrane][GO:0055085:transmembrane transport]	-	Jcr4S00206.70:Jatropha curcas|FAR1|FAR1 family protein	XP_010680628.1:PREDICTED: protein PINLIKES 7 isoform X1 [Beta vulgaris subsp. vulgaris] >XP_010680629.1 PREDICTED: protein PINLIKES 7 isoform X1 [Beta vulgaris subsp. vulgaris] >XP_019105728.1 PREDICTED: protein PINLIKES 7 isoform X1 [Beta vulgaris subsp. vulgaris] >KMT08875.1 hypothetical protein BVRB_6g135980 [Beta vulgaris subsp. vulgaris]	gi|521703437|gb|GAIA01016206.1|:TSA: Salicornia europaea Unigene16917_Se200S transcribed RNA sequence	A0A0J8C515|A0A0J8C515_BETVU:Uncharacterized protein;OS=Beta vulgaris subsp. vulgaris.;|GO:0016021,|GO:0055085,

geneid	trend	GO	Pathway	TF	NR	NT	Uniprot
DN119760_c5_g2_i1	DOWN	[GO:0016021:integral component of membrane]	-	-	KNA22374.1:hypothetical protein SOVF_034590 [Spinacia oleracea]	gi|521563765|gb|GAIA01074682.1|:TSA: Salicornia europaea Unigene90614_Se200S transcribed RNA sequence	A0A0K9RTX7|A0A0K9RTX7_SPIOL: Uncharacterized protein;OS=Spinacia oleracea;|GO:0016021,
DN122397_c5_g2_i2	DOWN	-	-	-	-	gi|521518426|gb|GAHZ01014842.1|:TSA: Salicornia europaea Unigene15516_SeCKS transcribed RNA sequence	-

geneid	trend	GO	Pathway	TF	NR	NT	Uniprot
DN122425_c0_g1_i1	DOWN	[GO:0016021:integral component of membrane][GO:0020037:heme binding][GO:0005506:iron ion binding][GO:0016709:oxidoreductase activity, acting on paired donors, with incorporation or reduction of molecular oxygen, NAD(P)H as one donor, and incorporation of one atom of oxygen][GO:0016132:brassinosteroid biosynthetic process][GO:0009965:leaf morphogenesis][GO:0042814:monopolar cell growth][GO:0048441:petal development][GO:0048443:stamen development]	[ko00905:Brassinosteroid biosynthesis]	Gh_A02G0666:Gossypium hirsutum|G2-like|G2-like family protein	XP_010679812.1:PREDICTED: 3epi6deoxocathasterone 23monooxygenase [Beta vulgaris subsp. vulgaris] >KMT09488.1 hypothetical protein BVRB_6g129580 [Beta vulgaris subsp. vulgaris]	gi|511318860|gb|GAKE01014017.1|:TSA: Thlaspi arvense Contig14235 transcribed RNA sequence	A0A0J8CBZ6|A0A0J8CBZ6_BETVU:Uncharacterized protein;OS=Beta vulgaris subsp. vulgaris.;|GO:0016021,|GO:0020037,GO:0005506,GO:0016709,|GO:0016132,GO:0009965,GO:0042814,GO:0048441,GO:0048443,

geneid	trend	GO	Pathway	TF	NR	NT	Uniprot
DN127647_c0_g1_i1	DOWN	[GO:0016021:integral component of membrane]	-	-	XP_021738731.1:uncharacterized protein LOC110705184 [Chenopodium quinoa]	-	A0A0K9RY57|A0A0K9RY57_SPIOL:Uncharacterized protein;OS=Spinacia oleracea;|GO:0016021,
DN116882_c2_g2_i3	DOWN	-	-	-	-	-	-
DN125811_c7_g10_i1	DOWN	-	-	-	AAT07462.1:thaumatinlike protein, partial [Mirabilis jalapa]	gi|46949211|gb|AY589711.1|:Mirabilis jalapa clone FS-82 thaumatin-like protein mRNA, partial cds	Q6PNZ5|Q6PNZ5_MIRJA:Thaumatin-like protein;OS=Mirabilis jalapa;
DN108492_c0_g1_i1	DOWN	[GO:0003824:catalytic activity]	[ko00052:Galactose metabolism]	-	XP_021741776.1:probable galactinolsucrose galactosyltransferase 6 [Chenopodium quinoa]	gi|357519204|ref|XM_003629843.1|:Medicago truncatula Stachyose synthase (MTR_8g088020) mRNA, complete cds	A0A0J8BN50|A0A0J8BN50_BETVU:Uncharacterized protein;OS=Beta vulgaris subsp. vulgaris.;|GO:0003824,
DN125811_c7_g5_i2	DOWN	-	-	-	-	-	-

geneid	trend	GO	Pathway	TF	NR	NT	Uniprot
DN124985_c1_g2_i2	DOWN	-	-	-	XP_010679575.1:PREDICTED: uncharacterized protein LOC104894909 [Beta vulgaris subsp. vulgaris]	-	A0A0J8CC76|A0A0J8CC76_BETVU:Uncharacterized protein;OS=Beta vulgaris subsp. vulgaris.;
DN126983_c6_g4_i2	DOWN	[GO:0008171:O-methyltransferase activity]	[ko00940:Phenylpropanoid biosynthesis]	-	KNA08046.1:hypothetical protein SOVF_166240 [Spinacia oleracea]	-	A0A0K9QN72|A0A0K9QN72_SPIOL:Uncharacterized protein;OS=Spinacia oleracea;|GO:0008171,
DN124293_c4_g1_i5	DOWN	[GO:0016021:integral component of membrane]	-	CCG025544.1:Populus euphratica|MYB_related|MYB_related family protein	XP_021774159.1:glucomannan 4betamannosyltransferase 2like [Chenopodium quinoa]	gi|292684181|dbj|AK339743.1|:Lotus japonicus cDNA, clone: LjFL3-075-AD05, HTC	A0A0K9QT44|A0A0K9QT44_SPIOL:Uncharacterized protein;OS=Spinacia oleracea;|GO:0016021,
DN112305_c0_g1_i2	DOWN	-	-	-	-	-	-
DN117620_c4_g6_i6	DOWN	-	-	-	XP_021862305.1:polygalacturonase QRT3like [Spinacia	-	A0A0K9RZE8|A0A0K9RZE8_SPIOL:Uncharacterized

geneid	trend	GO	Pathway	TF	NR	NT	Uniprot
					oleracea]		protein;OS=Spinacia oleracea;
DN116785_c0_g1_i1	DOWN	[GO:0016758:transferase activity, transferring hexosyl groups][GO:0008152:metabolic process]	[ko00908:Zeatin biosynthesis]	Tp57577_TGAC_v2_mRNA33215:Trifolium pratense|bHLH|bHLH family protein	XP_010672878.1:PREDICTED: zeatin Oglucosyltransferase [Beta vulgaris subsp. vulgaris] >KMT15051.1 hypothetical protein BVRB_3g061740 [Beta vulgaris subsp. vulgaris]	gi|224138073|ref|XM_002322687.1|:Populus trichocarpa predicted protein, mRNA	A0A0J8FGR9|A0A0J8FGR9_BETVU:Glycosyltransferase;OS=Beta vulgaris subsp. vulgaris.;|GO:0016758,|GO:0008152,
DN120376_c0_g9_i6	DOWN	[GO:0016021:integral component of membrane]	[ko04721:Synaptic vesicle cycle][ko04723:Retrograde endocannabinoid signaling][ko04727:GABAergic synapse][ko05032:Morphine addiction][ko05033:Nicotine addiction]	-	XP_010693246.1:PREDICTED: vacuolar amino acid transporter 1 [Beta vulgaris subsp. vulgaris] >KMS99237.1 hypothetical protein BVRB_2g046750 [Beta vulgaris subsp. vulgaris]	-	A0A0J8BGQ5|A0A0J8BGQ5_BETVU: Uncharacterized protein;OS=Beta vulgaris subsp. vulgaris.;|GO:0016021,

geneid	trend	GO	Pathway	TF	NR	NT	Uniprot
DN124134_c3_g8_i2	DOWN	[GO:0016491:oxidoreductase activity][GO:0008270:zinc ion binding]	[ko00010:Glycolysis / Gluconeogenesis][ko00071:Fatty acid degradation][ko00350:Tyrosine metabolism][ko00592:alpha-Linolenic acid metabolism]	-	AAP96921.1:alcohol dehydrogenase [Dianthus caryophyllus]	gi|512401092|gb|GAJZ01000078.1|:TSA: Prunus avium Pa_00186 transcribed RNA sequence	Q5QJY8|Q5QJY8_DIACA:Alcohol dehydrogenase;OS=Dianthus caryophyllus;|GO:0016491,GO:0008270,
DN122712_c0_g1_i2	DOWN	[GO:0016021:integral component of membrane][GO:0008519:ammonium transmembrane transporter activity]	-	Csa11g014890.1:Camelina sativa|GRAS|GRAS family protein	XP_010674250.1:PREDICTED: ammonium transporter 1 member 2like [Beta vulgaris subsp. vulgaris]	gi|359476542|ref|XM_002266540.2|:PREDICTED: Vitis vinifera ammonium transporter 1 member 2-like (LOC100256949), mRNA	A0A0J8CPE4|A0A0J8CPE4_BETVU:Ammonium transporter;OS=Beta vulgaris subsp. vulgaris.;|GO:0016021,|GO:0008519,
DN122397_c5_g2_i10	DOWN	-	-	-	-	-	-

geneid	trend	GO	Pathway	TF	NR	NT	Uniprot
DN126521_c0_g1_i1	DOWN	[GO:0016021:integral component of membrane]	-	-	XP_021842473.1:uncharacterized protein LOC110782616 [Spinacia oleracea] >KNA23833.1 hypothetical protein SOVF_020600 [Spinacia oleracea]	-	A0A0K9RWK0|A0A0K9RWK0_SPIOL:Uncharacterized protein;OS=Spinacia oleracea;|GO:0016021,
DN121552_c6_g13_i2	DOWN	-	-	-	-	-	-
DN170491_c0_g1_i1	DOWN	[GO:0003676:nucleic acid binding][GO:0008270:zinc ion binding][GO:0015074:DNA integration]	-	Rsa1.0_02878.1_g00005.1:Raphanus sativus|LBD|LBD family protein	KZV44616.1:hypothetical protein F511_33024 [Dorcoceras hygrometricum]	-	Q9C536|Q9C536_ARATH:Copia-type polyprotein, putative;OS=Arabidopsis thaliana;|GO:0003676,GO:0008270,|GO:0015074,

geneid	trend	GO	Pathway	TF	NR	NT	Uniprot
DN122545_c2_g2_i2	DOWN	[GO:0004185:serine-type carboxypeptidase activity]	-	KZV23174.1:Dorcoceras hygrometricum|C3H|C3H family protein	XP_010679983.1:PREDICTED: serine carboxypeptidaselike 42 [Beta vulgaris subsp. vulgaris] >KMT19451.1 hypothetical protein BVRB_1g010810 [Beta vulgaris subsp. vulgaris]	gi|225457766|ref|XM_002278157.1|:PREDICTED: Vitis vinifera serine carboxypeptidase-like 42 (LOC100262647), mRNA	A0A0J8FUT6|A0A0J8FUT6_BETVU:Carboxypeptidase;OS=Beta vulgaris subsp. vulgaris.;|GO:0004185,
DN123056_c1_g7_i3	DOWN	[GO:0016021:integral component of membrane][GO:0005215:transporter activity]	-	Sopen09g001330.1: Solanum pennellii|G2-like|G2-like family protein	XP_021837213.1:protein NRT1/ PTR FAMILY 3.1like [Spinacia oleracea] >KNA22385.1 hypothetical protein SOVF_034480 [Spinacia oleracea]	gi|478162000|gb|GAHK01014603.1|:TSA: Jatropha curcas Unigene2057_JC-CK_1A transcribed RNA sequence	A0A0K9RS59|A0A0K9RS59_SPIOL:Uncharacterized protein;OS=Spinacia oleracea;|GO:0016021,|GO:0005215,

geneid	trend	GO	Pathway	TF	NR	NT	Uniprot
DN127716_c2_g4_i1	DOWN	[GO:0008146:sulfotransferase activity]	-	29851.m002493:Ricinus communis|B3|B3 family protein	XP_010681134.1:PREDICTED: cytosolic sulfotransferase 12 [Beta vulgaris subsp. vulgaris] >KMT08715.1 hypothetical protein BVRB_6g139880 [Beta vulgaris subsp. vulgaris]	-	A0A0J8C4J2|A0A0J8C4J2_BETVU:Sulfotransferase;OS=Beta vulgaris subsp. vulgaris.;|GO:0008146,
DN128195_c1_g1_i3	DOWN	[GO:0052656:L-isoleucine transaminase activity][GO:0052654:L-leucine transaminase activity][GO:0052655:L-valine transaminase activity][GO:0009082:branched-chain amino acid biosynthetic process][GO:0008652:cellular amino acid biosynthetic process]	[ko00270:Cysteine and methionine metabolism][ko00280:Valine, leucine and isoleucine degradation][ko00290:Valine, leucine and isoleucine biosynthesis][ko00770:Pantothenate and CoA biosynthesis]	Achn299211:Actinidia chinensis|MYB_related|MYB_related family protein	XP_021754683.1:branchedchainaminoacid aminotransferase 2, chloroplasticlike [Chenopodium quinoa]	gi|356510641|ref|XM_003523998.1|:PREDICTED: Glycine max branched-chain-amino-acid aminotransferase 2, chloroplastic-like (LOC100818776), mRNA	A0A0K9QIB4|A0A0K9QIB4_SPIOL:Branched-chain-amino-acid aminotransferase;OS=Spinacia oleracea;|GO:0052656,GO:0052654,GO:0052655,|GO:0009082,GO:0008652,

geneid	trend	GO	Pathway	TF	NR	NT	Uniprot
DN124293_c4_g2_i2	DOWN	[GO:0016021:integral component of membrane]	-	CCG025544.1:Populus euphratica|MYB_related|MYB_related family protein	XP_021774159.1:glucomannan 4betamannosyltransferase 2like [Chenopodium quinoa]	gi|255580119|ref|XM_002530846.1|:Ricinus communis transferase, transferring glycosyl groups, putative, mRNA	A0A0K9QT44|A0A0K9QT44_SPIOL:Uncharacterized protein;OS=Spinacia oleracea;|GO:0016021,
DN112593_c0_g1_i3	DOWN	-	-	GSMUA_Achr10P17130_001:Musa acuminata|bHLH|bHLH family protein	KVI08265.1:DnaJ domaincontaining protein, partial [Cynara cardunculus var. scolymus]	-	A0A118K4Y3|A0A118K4Y3_CYNCS: DnaJ domain-containing protein;OS=Cynara cardunculus var. scolymus;
DN128380_c1_g7_i1	DOWN	-	-	-	XP_021755549.1:cytochrome P450 CYP736A12like [Chenopodium quinoa]	-	-

geneid	trend	GO	Pathway	TF	NR	NT	Uniprot
DN128061_c0_g1_i1	DOWN	[GO:0016021:integral component of membrane][GO:0032440:2-alkenal reductase [NAD(P)] activity][GO:0005524:ATP binding][GO:0042626:ATPase activity, coupled to transmembrane movement of substances]	-	RrC2480_p5:Raphanus raphanistrum|HSF|HSF family protein	XP_021610154.1:ABC transporter C family member 8 [Manihot esculenta] >OAY53133.1 hypothetical protein MANES_04G138200 [Manihot esculenta]	gi|359482525|ref|XM_002276176.2|:PREDICTED: Vitis vinifera ABC transporter C family member 8-like (LOC100246410), mRNA	B9SN54|B9SN54_RICCO:Multidrug resistance-associated protein 1, 3 (Mrp1, 3), abc-transoprter, putative;OS=Ricinus communis;|GO:0016021,|GO:0032440,GO:0005524,GO:0042626,
DN120234_c1_g1_i2	DOWN	[GO:0016021:integral component of membrane][GO:0020037:heme binding][GO:0005506:iron ion binding][GO:0004497:monooxygenase activity][GO:0016705:oxidoreductase activity, acting on paired donors, with incorporation or reduction of molecular oxygen]	-	OPUNC10G09700.4:Oryza punctata|E2F/DP|E2F/DP family protein	AKI33900.1:cytochrome P450 76AD1like protein, partial [Pisonia umbellifera]	-	A0A0G2URJ5|A0A0G2URJ5_9CARY:Cytochrome P450 76AD1-like protein;OS=Pisonia umbellifera.;|GO:0016021,|GO:0020037,GO:0005506,GO:0004497,GO:0016705,

geneid	trend	GO	Pathway	TF	NR	NT	Uniprot
DN127560_c3_g17_i1	DOWN	[GO:0016747:transferase activity, transferring acyl groups other than amino-acyl groups][GO:0009058:biosynthetic process]	[ko00941:Flavonoid biosynthesis][ko04712:Circadian rhythm - plant]	-	XP_021837196.1:chalcone synthaselike [Spinacia oleracea] >KNA19553.1 hypothetical protein SOVF_060670 [Spinacia oleracea]	gi|215513675|gb|FJ384161.1|:Cardamine maritima isolate M3 chalcone synthase gene, exon 2 and partial cds	A0A0K9RJ90|A0A0K9RJ90_SPIOL:Uncharacterized protein;OS=Spinacia oleracea;|GO:0016747,|GO:0009058,
DN122008_c9_g8_i1	DOWN	-	-	-	-	-	-
DN119937_c0_g3_i1	DOWN	-	-	Rsa1.0_00048.1_g00027.1:Raphanus sativus|bHLH|bHLH family protein	XP_010690854.1:PREDICTED: isoflavone reductaselike protein [Beta vulgaris subsp. vulgaris]	gi|10764490|gb|AF282850.1|AF282850: Betula pendula allergenic isoflavone reductase-like protein Bet v 6.0102 (BETV6.0102) mRNA, complete cds	A0A0J8BG85|A0A0J8BG85_BETVU:Uncharacterized protein;OS=Beta vulgaris subsp. vulgaris.;
DN120776_c1_g3_i1	DOWN	[GO:0016021:integral component of membrane][GO:0008168:methyltransferase activity]	-	-	XP_021758761.1:uncharacterized protein LOC110723714 [Chenopodium quinoa]	-	A0A0K9REU0|A0A0K9REU0_SPIOL: Uncharacterized protein;OS=Spinacia oleracea;|GO:0016021,|GO:0008168,

geneid	trend	GO	Pathway	TF	NR	NT	Uniprot
DN110478_c0_g2_i2	DOWN	[GO:0016021:integral component of membrane][GO:0005886:plasma membrane]	-	-	XP_010686498.1:PREDICTED: CASPlike protein 1C1 [Beta vulgaris subsp. vulgaris] >KMT04245.1 hypothetical protein BVRB_8g183210 [Beta vulgaris subsp. vulgaris]	-	A0A0J8BRW0|A0A0J8BRW0_BETVU: CASP-like protein;OS=Beta vulgaris subsp. vulgaris.;|GO:0016021,GO:0005886,
DN119806_c0_g2_i2	DOWN	-	-	-	XP_021854286.1:uncharacterized protein LOC110793691 [Spinacia oleracea] >KNA22057.1 hypothetical protein SOVF_037490 [Spinacia oleracea]	-	A0A0K9RTA3|A0A0K9RTA3_SPIOL: Uncharacterized protein;OS=Spinacia oleracea;
DN106011_c0_g2_i1	DOWN	-	-	-	XP_014519484.1:PREDICTED: decapping 5like protein [Vigna radiata var. radiata]	-	A0A0B0PB27|A0A0B0PB27_GOSAR:Lsm14b-b: Protein LSM14 B-B;OS=Gossypium arboreum;

geneid	trend	GO	Pathway	TF	NR	NT	Uniprot
DN127035_c4_g4_i5	DOWN	[GO:0016788:hydrolase activity, acting on ester bonds]	-	Potri.012G137500.1:Populus trichocarpa|FAR1|FAR1 family protein	XP_010666213.1:PREDICTED: GDSL esterase/lipase At5g55050 [Beta vulgaris subsp. vulgaris] >KMS96262.1 hypothetical protein BVRB_000690 [Beta vulgaris subsp. vulgaris]	gi|357476428|ref|XM_003608452.1|:Medicago truncatula Cytochrome B561-related protein (MTR_4g096780) mRNA, complete cds	A0A0J8B8U9|A0A0J8B8U9_BETVU:Uncharacterized protein;OS=Beta vulgaris subsp. vulgaris.;|GO:0016788,
DN125250_c0_g10_i2	DOWN	[GO:0016157:sucrose synthase activity][GO:0005985:sucrose metabolic process]	[ko00500:Starch and sucrose metabolism]	mrna11429.1-v1.0-hybrid:Fragaria vesca|HB-other|HB-other family protein	AFS17277.1:sucrose synthase 1 [Amaranthus cruentus/Amaranthus hypocondriacus mixed library]	gi|7007262|emb|X82504.1|:C.rubrum mRNA for sucrose synthase	J9WP95|J9WP95_9CARY:Sucrose synthase;OS=Amaranthus cruentus/Amaranthus hypocondriacus mixed library.;|GO:0016157,|GO:0005985,

geneid	trend	GO	Pathway	TF	NR	NT	Uniprot
DN127142_c4_g22_i2	DOWN	[GO:0004553:hydrolase activity, hydrolyzing O-glycosyl compounds][GO:0005975:carbohydrate metabolic process]	[ko00520:Amino sugar and nucleotide sugar metabolism]	-	XP_010671559.1:PREDICTED: hevamineA [Beta vulgaris subsp. vulgaris] >KMT16117.1 hypothetical protein BVRB_3g052790 [Beta vulgaris subsp. vulgaris]	-	A0A0J8CR98|A0A0J8CR98_BETVU:Uncharacterized protein;OS=Beta vulgaris subsp. vulgaris.;|GO:0004553,|GO:0005975,
DN121060_c0_g3_i1	DOWN	[GO:0016787:hydrolase activity][GO:0016042:lipid catabolic process]	-	KHN31892.1:Glycine soja|bHLH|bHLH family protein	XP_021852131.1:probable inactive patatinlike protein 9 [Spinacia oleracea]	-	A0A0K9REM9|A0A0K9REM9_SPIOL: Patatin;OS=Spinacia oleracea;|GO:0016787,|GO:0016042,
DN119252_c4_g2_i2	DOWN	-	-	-	-	-	-
DN117950_c0_g1_i3	DOWN	[GO:0003676:nucleic acid binding]	-	Dca50986.1:Dianthus caryophyllus|FAR1|FAR1 family protein	XP_022030758.1:uncharacterized protein LOC110931681 [Helianthus annuus]	-	A0A0V0IAV6|A0A0V0IAV6_SOLCH: Putative ovule protein;OS=Solanum chacoense;|GO:0003676,

geneid	trend	GO	Pathway	TF	NR	NT	Uniprot
DN120100_c0_g7_i4	DOWN	-	-	-	XP_010685294.1:PREDICTED: uncharacterized protein LOC104899736 [Beta vulgaris subsp. vulgaris] >KMT04809.1 hypothetical protein BVRB_7g169800 [Beta vulgaris subsp. vulgaris]	gi|224090468|ref|XM_002308955.1|:Populus trichocarpa predicted protein, mRNA	A0A0J8BXT3|A0A0J8BXT3_BETVU:Uncharacterized protein;OS=Beta vulgaris subsp. vulgaris.;
DN103604_c0_g1_i1	DOWN	-	-	-	XP_010673923.1:PREDICTED: uncharacterized protein LOC104890222 [Beta vulgaris subsp. vulgaris] >KMT14751.1 hypothetical protein BVRB_4g075170 [Beta vulgaris subsp. vulgaris]	gi|521706306|gb|GAIA01014755.1|:TSA: Salicornia europaea Unigene15418_Se200S transcribed RNA sequence	A0A0J8FG01|A0A0J8FG01_BETVU:Uncharacterized protein;OS=Beta vulgaris subsp. vulgaris.;

geneid	trend	GO	Pathway	TF	NR	NT	Uniprot
DN103814_c0_g1_i1	DOWN	-	-	-	XP_021861758.1:WEB family protein At1g75720 [Spinacia oleracea] >KNA18540.1 hypothetical protein SOVF_069800 [Spinacia oleracea]	-	A0A0K9RGD5|A0A0K9RGD5_SPIOL: Uncharacterized protein;OS=Spinacia oleracea;
DN124946_c0_g1_i1	DOWN	-	-	DCAR_006074:Daucus carota|MYB_related|MYB_related family protein	XP_010670661.1:PREDICTED: putative ripeningrelated protein 1 [Beta vulgaris subsp. vulgaris] >KMT20501.1 hypothetical protein BVRB_1g004870 [Beta vulgaris subsp. vulgaris]	-	A0A0J8FXY7|A0A0J8FXY7_BETVU:Uncharacterized protein;OS=Beta vulgaris subsp. vulgaris.;
DN121306_c4_g9_i1	DOWN	-	-	-	-	-	-

geneid	trend	GO	Pathway	TF	NR	NT	Uniprot
DN118633_c2_g2_i1	DOWN	[GO:0005634:nucleus][GO:0043565:sequence-specific DNA binding][GO:0006355:regulation of transcription, DNA-templated]	-	Bv6_139660_wjii.t1:Beta vulgaris|HD-ZIP|HD-ZIP family protein	XP_010681120.1:PREDICTED: homeoboxleucine zipper protein ATHB40 [Beta vulgaris subsp. vulgaris] >KMT08693.1 hypothetical protein BVRB_6g139660 [Beta vulgaris subsp. vulgaris]	gi|521711753|gb|GAIA01012218.1|:TSA: Salicornia europaea Unigene12838_Se200S transcribed RNA sequence	A0A0J8C9L6|A0A0J8C9L6_BETVU:Uncharacterized protein;OS=Beta vulgaris subsp. vulgaris.;|GO:0005634,|GO:0043565,|GO:0006355,
DN121715_c1_g1_i4	DOWN	[GO:0020037:heme binding][GO:0005506:iron ion binding][GO:0004497:monooxygenase activity][GO:0016705:oxidoreductase activity, acting on paired donors, with incorporation or reduction of molecular oxygen]	-	-	XP_021755505.1:alkane hydroxylase MAH1like [Chenopodium quinoa]	-	A0A067KGN9|A0A067KGN9_JATCU: Uncharacterized protein;OS=Jatropha curcas;|GO:0020037,GO:0005506,GO:0004497,GO:0016705,

geneid	trend	GO	Pathway	TF	NR	NT	Uniprot
DN116600_c0_g1_i1	DOWN	[GO:0016021:integral component of membrane][GO:0008270:zinc ion binding]	-	Sme2.5_00572.1_g00001.1:Solanum melongena|HD-ZIP|HD-ZIP family protein	XP_010676294.1:PREDICTED: RINGH2 finger protein ATL22 [Beta vulgaris subsp. vulgaris] >KMT11919.1 hypothetical protein BVRB_5g098830 [Beta vulgaris subsp. vulgaris]	-	A0A0J8CIJ9|A0A0J8CIJ9_BETVU:Uncharacterized protein;OS=Beta vulgaris subsp. vulgaris.;|GO:0016021,|GO:0008270,
DN127035_c4_g5_i1	DOWN	[GO:0016788:hydrolase activity, acting on ester bonds]	-	-	XP_010666213.1:PREDICTED: GDSL esterase/lipase At5g55050 [Beta vulgaris subsp. vulgaris] >KMS96262.1 hypothetical protein BVRB_000690 [Beta vulgaris subsp. vulgaris]	gi|521745190|gb|GAIA01003406.1|:TSA: Salicornia europaea Unigene3613_Se200S transcribed RNA sequence	A0A0J8B8U9|A0A0J8B8U9_BETVU:Uncharacterized protein;OS=Beta vulgaris subsp. vulgaris.;|GO:0016788,

geneid	trend	GO	Pathway	TF	NR	NT	Uniprot
DN121243_c0_g1_i4	DOWN	-	-	-	XP_021753987.1:IRKinteracting proteinlike [Chenopodium quinoa]	-	A0A0K9R5D9|A0A0K9R5D9_SPIOL:Uncharacterized protein;OS=Spinacia oleracea;
DN108404_c0_g3_i2	DOWN	[GO:0005524:ATP binding][GO:0003777:microtubule motor activity][GO:0007018:microtubule-based movement]	-	-	XP_021728220.1:kinesinlike protein KIN14R [Chenopodium quinoa]	-	A0A0K9R366|A0A0K9R366_SPIOL:Uncharacterized protein;OS=Spinacia oleracea;|GO:0005524,GO:0003777,|GO:0007018,
DN105221_c0_g1_i1	DOWN	[GO:0046658:anchored component of plasma membrane][GO:0009055:electron transfer activity]	-	-	XP_009612880.1:PREDICTED: blue copper proteinlike [Nicotiana tomentosiformis]	-	K4CBT1|K4CBT1_SOLLC:Uncharacterized protein;OS=Solanum lycopersicum;|GO:0046658,|GO:0009055,

geneid	trend	GO	Pathway	TF	NR	NT	Uniprot
DN118981_c0_g8_i1	DOWN	[GO:0004665:prephenate dehydrogenase (NADP+) activity][GO:0008977:prephenate dehydrogenase (NAD+) activity][GO:0006571:tyrosine biosynthetic process]	[ko00400:Phenylalanine, tyrosine and tryptophan biosynthesis]	-	AMR60658.1:arogenate dehydrogenase, partial [Mirabilis jalapa]	gi|521452250|gb|GAHZ01068234.1|:TSA: Salicornia europaea Unigene82334_SeCKS transcribed RNA sequence	A0A0K9QIW7|A0A0K9QIW7_SPIOL: Uncharacterized protein;OS=Spinacia oleracea;|GO:0004665,GO:0008977,|GO:0006571,
DN124971_c3_g2_i1	DOWN	[GO:0046872:metal ion binding][GO:0016491:oxidoreductase activity]	-	676709864:Sisymbrium irio|STAT|STAT family protein	XP_021764180.1:protein SRG1like [Chenopodium quinoa]	gi|224061380|ref|XM_002300415.1|:Populus trichocarpa predicted protein, mRNA	A0A0J8B6P3|A0A0J8B6P3_BETVU:Uncharacterized protein;OS=Beta vulgaris subsp. vulgaris.;|GO:0046872,GO:0016491,
DN127167_c2_g1_i12	DOWN	[GO:0016021:integral component of membrane][GO:0005524:ATP binding][GO:0016887:ATPase activity]	-	MDP0000265619:Malus domestica|Nin-like|Nin-like family protein	XP_010690527.2:PREDICTED: ABC transporter G family member 11like [Beta vulgaris subsp. vulgaris]	gi|502142152|ref|XM_004504760.1|:PREDICTED: Cicer arietinum ABC transporter G family member 11-like (LOC101503225), mRNA	A0A0K9RUY2|A0A0K9RUY2_SPIOL: Uncharacterized protein;OS=Spinacia oleracea;|GO:0016021,|GO:0005524,GO:0016887,

geneid	trend	GO	Pathway	TF	NR	NT	Uniprot
DN112621_c0_g1_i1	DOWN	[GO:0016021:integral component of membrane]	-	-	KMT12931.1:hypothetical protein BVRB_4g090150 [Beta vulgaris subsp. vulgaris]	gi|356552612|ref|XM_003544611.1|:PREDICTED: Glycine max uncharacterized protein LOC100801579 (LOC100801579), mRNA	A0A0J8FAP0|A0A0J8FAP0_BETVU:Uncharacterized protein;OS=Beta vulgaris subsp. vulgaris.;|GO:0016021,
DN107438_c0_g1_i1	DOWN	[GO:0031225:anchored component of membrane][GO:0016021:integral component of membrane][GO:0005886:plasma membrane][GO:0005774:vacuolar membrane][GO:0016049:cell growth][GO:0010215:cellulose microfibril organization][GO:0009834:plant-type secondary cell wall biogenesis]	-	Sopen01g046270.1:Solanum pennellii|M-type_MADS|M-type_MADS family protein	OVA00833.1:COBRA [Macleaya cordata]	gi|521656075|gb|GAIA01039135.1|:TSA: Salicornia europaea Unigene43792_Se200S transcribed RNA sequence	A0A0J8D2Y8|A0A0J8D2Y8_BETVU:Uncharacterized protein;OS=Beta vulgaris subsp. vulgaris.;|GO:0031225,GO:0016021,GO:0005886,GO:0005774,|GO:0016049,GO:0010215,GO:0009834,
DN121522_c1_g1_i6	DOWN	-	-	-	-	-	-

geneid	trend	GO	Pathway	TF	NR	NT	Uniprot
DN121542_c2_g1_i1	DOWN	[GO:0004842:ubiquitin-protein transferase activity]	-	Do007746.1:Dichanthelium oligosanthes|bHLH|bHLH family protein	XP_021733644.1:U box domaincontaining protein 19like [Chenopodium quinoa]	gi|521499962|gb|GAHZ01030958.1|:TSA: Salicornia europaea Unigene34115_SeCKS transcribed RNA sequence	A0A0J8CEZ7|A0A0J8CEZ7_BETVU:Uncharacterized protein;OS=Beta vulgaris subsp. vulgaris.;|GO:0004842,
DN123961_c0_g1_i1	DOWN	-	-	-	XP_010676582.1:PREDICTED: synaptonemal complex protein 1like isoform X2 [Beta vulgaris subsp. vulgaris]	gi|356552883|ref|XM_003544744.1|:PREDICTED: Glycine max synaptonemal complex protein 1-like (LOC100785508), mRNA	A0A0K9RRJ2|A0A0K9RRJ2_SPIOL:Uncharacterized protein;OS=Spinacia oleracea;
DN88736_c0_g1_i1	DOWN	-	-	-	-	-	-
DN107426_c0_g1_i1	DOWN	-	-	-	-	-	-

geneid	trend	GO	Pathway	TF	NR	NT	Uniprot
DN120974_c1_g3_i5	DOWN	[GO:0005634:nucleus][GO:0003677:DNA binding]	-	Sp_098310_sjnz.t1: Spinacia oleracea|SBP|SBP family protein	XP_021845911.1:squamosa promoterbindinglike protein 13A [Spinacia oleracea] >XP_021845912.1 squamosa promoterbindinglike protein 13A [Spinacia oleracea] >XP_021845913.1 squamosa promoterbindinglike protein 13A [Spinacia oleracea] >KNA15434.1 hypothetical protein SOVF_098310 [Spinacia oleracea]	-	A0A0K9R8X4|A0A0K9R8X4_SPIOL:Uncharacterized protein;OS=Spinacia oleracea;|GO:0005634,|GO:0003677,

geneid	trend	GO	Pathway	TF	NR	NT	Uniprot
DN119962_c0_g2_i1	DOWN	[GO:0016021:integral component of membrane][GO:0005886:plasma membrane][GO:0016760:cellulose synthase (UDP-forming) activity][GO:0046872:metal ion binding][GO:0071555:cell wall organization][GO:0030244:cellulose biosynthetic process][GO:0042742:defense response to bacterium][GO:0050832:defense response to fungus][GO:0009834:plant-type secondary cell wall biogenesis]	-	XP_010091789.1:Morus notabilis|G2-like|G2-like family protein	XP_009588096.1:PREDICTED: cellulose synthase A catalytic subunit 4 [UDPforming] isoform X1 [Nicotiana tomentosiformis]	gi|470107288|ref|XM_004289933.1|:PREDICTED: Fragaria vesca subsp. vesca cellulose synthase A catalytic subunit 4 [UDP-forming]-like (LOC101299045), mRNA	A0A0J8B7A9|A0A0J8B7A9_BETVU:Cellulose synthase;OS=Beta vulgaris subsp. vulgaris.;|GO:0016021,GO:0005886,|GO:0016760,GO:0046872,|GO:0071555,GO:0030244,GO:0042742,GO:0050832,GO:0009834,
DN123231_c0_g6_i8	DOWN	[GO:0005524:ATP binding][GO:0003777:microtubule motor activity][GO:0007018:microtubule-based movement]	-	Araip.YX9Q9:Arachis ipaensis|MYB_related|MYB_related family protein	XP_021772951.1:kinesinlike protein KIN14S [Chenopodium quinoa]	-	A0A0J8CJA0|A0A0J8CJA0_BETVU:Uncharacterized protein;OS=Beta vulgaris subsp. vulgaris.;|GO:0005524,GO:0003777,|GO:0007018,

geneid	trend	GO	Pathway	TF	NR	NT	Uniprot
DN121885_c1_g1_i4	DOWN	[GO:0016021:integral component of membrane][GO:0005886:plasma membrane][GO:0008643:carbohydrate transport]	-	mrna24941.1-v1.0-hybrid:Fragaria vesca|DBB|DBB family protein	XP_021771668.1:bidirectional sugar transporter SWEET1like [Chenopodium quinoa]	-	A0A0K9QJC5|A0A0K9QJC5_SPIOL:Bidirectional sugar transporter SWEET;OS=Spinacia oleracea;|GO:0016021,GO:0005886,|GO:0008643,
DN128863_c1_g1_i4	DOWN	[GO:0000287:magnesium ion binding][GO:0010333:terpene synthase activity][GO:0008152:metabolic process]	[ko00904:Diterpenoid biosynthesis]	MDP0000245233:Malus domestica|bHLH|bHLH family protein	XP_019106684.1:PREDICTED: (E,E)geranyllinalool synthaselike [Beta vulgaris subsp. vulgaris] >KMT04332.1 hypothetical protein BVRB_8g184070 [Beta vulgaris subsp. vulgaris]	-	A0A0J8BT41|A0A0J8BT41_BETVU:Uncharacterized protein;OS=Beta vulgaris subsp. vulgaris.;|GO:0000287,GO:0010333,|GO:0008152,
DN127293_c1_g1_i3	DOWN	-	-	-	-	-	-

geneid	trend	GO	Pathway	TF	NR	NT	Uniprot
DN121623_c0_g1_i9	DOWN	[GO:0016021:integral component of membrane]	-	-	XP_021614185.1:protein IQDOMAIN 1 [Manihot esculenta] >OAY48942.1 hypothetical protein MANES_05G017200 [Manihot esculenta]	gi|521502741|gb|GAHZ01028179.1|:TSA: Salicornia europaea Unigene31029_SeCKS transcribed RNA sequence	B9SVG1|B9SVG1_RICCO:Calmodulin binding protein, putative;OS=Ricinus communis;|GO:0016021,
DN120388_c2_g1_i4	DOWN	[GO:0004553:hydrolase activity, hydrolyzing O-glycosyl compounds][GO:0005975:carbohydrate metabolic process]	-	-	XP_010676175.1:PREDICTED: acid betafructofuranosidase isoform X2 [Beta vulgaris subsp. vulgaris] >KMT12545.1 hypothetical protein BVRB_5g097930 [Beta vulgaris subsp. vulgaris]	gi|21322511|emb|AJ422051.1|:Beta vulgaris mRNA for acid vacuolar invertase (vacinv gene)	A0A0J8CFL4|A0A0J8CFL4_BETVU:Uncharacterized protein;OS=Beta vulgaris subsp. vulgaris.;|GO:0004553,|GO:0005975,

geneid	trend	GO	Pathway	TF	NR	NT	Uniprot
DN124189_c0_g3_i1	DOWN	[GO:0005509:calcium ion binding][GO:0005544:calcium-dependent phospholipid binding]	-	-	XP_010680554.1:PREDICTED: annexin D2 [Beta vulgaris subsp. vulgaris] >KMT08792.1 hypothetical protein BVRB_6g135160 [Beta vulgaris subsp. vulgaris]	gi|521512199|gb|GAHZ01018721.1|:TSA: Salicornia europaea Unigene19664_SeCKS transcribed RNA sequence	A0A0J8C5G7|A0A0J8C5G7_BETVU:Annexin;OS=Beta vulgaris subsp. vulgaris.;|GO:0005509,GO:0005544,
DN119868_c2_g1_i6	DOWN	-	-	-	-	-	-

geneid	trend	GO	Pathway	TF	NR	NT	Uniprot
DN98220_c0_g1_i3	DOWN	-	[ko03010:Ribosome]	-	XP_011087800.1:60S ribosomal protein L3 [Sesamum indicum] >XP_011087801.1 60S ribosomal protein L3 [Sesamum indicum] >XP_011087802.1 60S ribosomal protein L3 [Sesamum indicum] >XP_020551938.1 60S ribosomal protein L3 [Sesamum indicum] >XP_020551939.1 60S ribosomal protein L3 [Sesamum indicum] >XP_020551940.1 60S ribosomal protein L3 [Sesamum indicum]	gi|1181130|emb|Z69336.1|:C.elegans mRNA for ribosomal protein L3	A0A1D1YTM4|A0A1D1YTM4_9ARAE:60S ribosomal protein L3;OS=Anthurium amnicola.;

geneid	trend	GO	Pathway	TF	NR	NT	Uniprot
DN127772_c2_g8_i2	DOWN	[GO:0032040:small-subunit processome]	-	Achn356291:Actinidia chinensis|MIKC_MADS|MIKC_MADS family protein	XP_010667324.1:PREDICTED: uncharacterized protein LOC104884377 [Beta vulgaris subsp. vulgaris]	gi|521670917|gb|GAIA01032066.1|:TSA: Salicornia europaea Unigene35193_Se200S transcribed RNA sequence	A0A0J8C595|A0A0J8C595_BETVU:Uncharacterized protein;OS=Beta vulgaris subsp. vulgaris.;|GO:0032040,
DN127142_c4_g4_i1	DOWN	[GO:0004553:hydrolase activity, hydrolyzing O-glycosyl compounds][GO:0005975:carbohydrate metabolic process]	[ko00520:Amino sugar and nucleotide sugar metabolism]	-	KNA13287.1:hypothetical protein SOVF_118080 [Spinacia oleracea]	gi|460368216|ref|XM_004229916.1|:PREDICTED: Solanum lycopersicum acidic endochitinase-like (LOC101246963), mRNA	A0A0K9R3A9|A0A0K9R3A9_SPIOL:Uncharacterized protein;OS=Spinacia oleracea;|GO:0004553,|GO:0005975,
DN118299_c1_g1_i2	DOWN	-	-	-	KNA08571.1:hypothetical protein SOVF_161530 [Spinacia oleracea]	-	A0A0K9QPU7|A0A0K9QPU7_SPIOL: Uncharacterized protein;OS=Spinacia oleracea;
DN111439_c0_g1_i6	DOWN	[GO:0020037:heme binding][GO:0046872:metal ion binding][GO:0019825:oxygen binding]	-	-	XP_021731656.1:nonsymbiotic hemoglobin 1like [Chenopodium quinoa]	gi|444792482|gb|KC142173.1|:Spinacia oleracea non-symbiotic hemoglobin mRNA, complete cds	L7ZC15|L7ZC15_SPIOL:Non-symbiotic hemoglobin;OS=Spinacia oleracea;|GO:00200

geneid	trend	GO	Pathway	TF	NR	NT	Uniprot
							37,GO:0046872,GO:0019825,
DN113730_c0_g1_i1	DOWN	-	-	MDP0000574942:Malus domestica|MYB|MYB family protein	KNA21831.1:hypothetical protein SOVF_039720 [Spinacia oleracea]	gi|521651255|gb|GAIA01041419.1|:TSA: Salicornia europaea Unigene46534_Se200S transcribed RNA sequence	A0A0K9RQL6|A0A0K9RQL6_SPIOL: Uncharacterized protein;OS=Spinacia oleracea;
DN128315_c7_g4_i11	DOWN	-	-	-	-	-	-
DN126341_c5_g8_i5	DOWN	[GO:0005576:extracellular region][GO:0030145:manganese ion binding][GO:0045735:nutrient reservoir activity]	-	-	XP_021750074.1:auxinbinding protein ABP19alike [Chenopodium quinoa]	-	A0A0K9RFJ5|A0A0K9RFJ5_SPIOL:Uncharacterized protein;OS=Spinacia oleracea;|GO:0005576,|GO:0030145,GO:0045735,

geneid	trend	GO	Pathway	TF	NR	NT	Uniprot
DN128786_c1_g1_i7	DOWN	-	-	-	XP_021750546.1:uncharacterized protein LOC110716241 [Chenopodium quinoa]	-	D7THD8|D7THD8_VITVI:Putative uncharacterized protein;OS=Vitis vinifera;GN=VIT_08s0007g01560 {ECO:0000313|EMBL:CBI30454.3};
DN119707_c1_g1_i6	DOWN	[GO:0016021:integral component of membrane]	-	-	XP_021845185.1:uncharacterized protein LOC110785047 [Spinacia oleracea] >KNA04822.1 hypothetical protein SOVF_196160 [Spinacia oleracea]	-	A0A0K9QC24|A0A0K9QC24_SPIOL:Uncharacterized protein;OS=Spinacia oleracea;|GO:0016021,
DN116172_c1_g1_i1	DOWN	-	-	-	-	-	-

geneid	trend	GO	Pathway	TF	NR	NT	Uniprot
DN121252_c1_g5_i2	DOWN	[GO:0016021:integral component of membrane][GO:0005215:transporter activity][GO:0006857:oligopeptide transport]	-	Sopen09g001330.1: Solanum pennellii|G2-like|G2-like family protein	XP_010680056.1:PREDICTED: protein NRT1/ PTR FAMILY 8.3 [Beta vulgaris subsp. vulgaris] >KMT09724.1 hypothetical protein BVRB_6g131730 [Beta vulgaris subsp. vulgaris]	-	A0A0J8CBX0|A0A0J8CBX0_BETVU: Uncharacterized protein;OS=Beta vulgaris subsp. vulgaris.;|GO:0016021,|GO:0005215,|GO:0006857,
DN120208_c1_g2_i1	DOWN	-	-	-	-	-	-
DN120465_c0_g2_i4	DOWN	-	-	-	KMT04735.1:hypothetical protein BVRB_7g169160 [Beta vulgaris subsp. vulgaris]	-	A0A0J8BT62|A0A0J8BT62_BETVU:Uncharacterized protein;OS=Beta vulgaris subsp. vulgaris.;
DN122397_c5_g2_i13	DOWN	-	-	-	-	-	-
DN85322_c0_g3_i1	DOWN	-	-	-	-	-	-

geneid	trend	GO	Pathway	TF	NR	NT	Uniprot
DN682_c0_g3_i1	DOWN	-	-	-	-	gi|134305329|gb|EF471314.1|:Eriocheir sinensis tropomyosin mRNA, complete cds	-
DN119104_c2_g3_i5	DOWN	-	-	Csa09g092790.1:Camelina sativa|BES1|BES1 family protein	XP_021736225.1:protein trichome birefringencelike 41 [Chenopodium quinoa]	-	A0A0J8CMI4|A0A0J8CMI4_BETVU:Uncharacterized protein;OS=Beta vulgaris subsp. vulgaris.;
DN118084_c3_g13_i4	DOWN	[GO:0016491:oxidoreductase activity]	-	Cc01_g08720:Coffea canephora|NAC|NAC family protein	XP_021753384.1:secoisolariciresinol dehydrogenaselike [Chenopodium quinoa]	gi|521452294|gb|GAHZ01068190.1|:TSA: Salicornia europaea Unigene82288_SeCKS transcribed RNA sequence	A0A0K9R9V4|A0A0K9R9V4_SPIOL:Uncharacterized protein;OS=Spinacia oleracea;|GO:0016491,
DN128938_c2_g6_i3	DOWN	-	-	-	XP_016465625.1:PREDICTED: uncharacterized protein LOC107788459 [Nicotiana tabacum]	-	Unreviewed|Q948Y4_TOBAC:Putative translocation transactivator/inclusion body protein;OS=Nicotiana tabacum;

geneid	trend	GO	Pathway	TF	NR	NT	Uniprot
DN123400_c0_g1_i3	DOWN	[GO:0005794:Golgi apparatus][GO:0016021:integral component of membrane][GO:0015020:glucuronosyltransferase activity][GO:0009834:plant-type secondary cell wall biogenesis][GO:0045492:xylan biosynthetic process]	-	-	XP_021742962.1:UDPglucuronate:xylan alphaglucuronosyltransferase 2like isoform X1 [Chenopodium quinoa]	-	A0A0J8BHS8|A0A0J8BHS8_BETVU:Hexosyltransferase;OS=Beta vulgaris subsp. vulgaris.;|GO:0005794,GO:0016021,|GO:0015020,|GO:0009834,GO:0045492,
DN127647_c0_g1_i5	DOWN	[GO:0016021:integral component of membrane]	-	-	XP_021866951.1:uncharacterized protein LOC110805639 [Spinacia oleracea] >KNA24470.1 hypothetical protein SOVF_015340 [Spinacia oleracea]	-	A0A0K9RY57|A0A0K9RY57_SPIOL:Uncharacterized protein;OS=Spinacia oleracea;|GO:0016021,
DN128786_c1_g1_i2	DOWN	-	-	-	XP_021759112.1:uncharacterized protein LOC110724013 [Chenopodium quinoa]	-	D7THD8|D7THD8_VITVI:Putative uncharacterized protein;OS=Vitis vinifera;GN=VIT_08s0007g01560 {ECO:0000313|EMBL:CBI30454.3};

geneid	trend	GO	Pathway	TF	NR	NT	Uniprot
DN108591_c0_g1_i1	DOWN	-	-	-	-	-	-
DN104612_c0_g2_i1	DOWN	[GO:0016021:integral component of membrane][GO:0005743:mitochondrial inner membrane][GO:0008137:NADH dehydrogenase (ubiquinone) activity]	[ko00190:Oxidative phosphorylation][ko04723:Retrograde endocannabinoid signaling][ko05012:Parkinson's disease]	-	NP_050063.1:NADH dehydrogenase subunit 1 [Pedinomonas minor] >AAD19672.1 NADH dehydrogenase subunit 1 (mitochondrion) [Pedinomonas minor]	-	Q9G8V3|Q9G8V3_RHDSA:NADH-ubiquinone oxidoreductase chain 1;OS=Rhodomonas salina;|GO:0016021,GO:0005743,|GO:0008137,
DN122252_c2_g1_i3	DOWN	[GO:0016747:transferase activity, transferring acyl groups other than amino-acyl groups][GO:0009058:biosynthetic process]	[ko00941:Flavonoid biosynthesis][ko04712:Circadian rhythm - plant]	-	XP_021747699.1:chalcone synthaselike [Chenopodium quinoa]	gi|478686564|gb|KC261503.1|:Tulipa fosteriana CHS1 mRNA, complete cds	A0A0K9RJ90|A0A0K9RJ90_SPIOL:Uncharacterized protein;OS=Spinacia oleracea;|GO:0016747,|GO:0009058,

geneid	trend	GO	Pathway	TF	NR	NT	Uniprot
DN113291_c0_g2_i1	DOWN	[GO:0016021:integral component of membrane][GO:0008289:lipid binding][GO:0006869:lipid transport]	-	-	XP_021764265.1:nonspecific lipidtransfer proteinlike protein At2g13820 [Chenopodium quinoa]	-	A0A067L6V1|A0A067L6V1_JATCU:Non-specific lipid transfer protein A;OS=Jatropha curcas;|GO:0016021,|GO:0008289,|GO:0006869,
DN128530_c19_g15_i2	DOWN	-	-	-	KRH17812.1:hypothetical protein GLYMA_13G0180002, partial [Glycine max]	gi|345287582|gb|HQ843443.1|:Bougainvillea glabra 26S ribosomal RNA gene, partial sequence	A0A0R0GUN2|A0A0R0GUN2_SOYBN:Uncharacterized protein;OS=Glycine max;
DN116736_c1_g4_i1	DOWN	-	-	-	-	-	-
DN119981_c5_g1_i1	DOWN	-	-	-	-	-	-

geneid	trend	GO	Pathway	TF	NR	NT	Uniprot
DN127196_c0_g1_i2	DOWN	-	-	-	XP_010683264.1:PREDICTED: uncharacterized protein LOC104897980 [Beta vulgaris subsp. vulgaris] >XP_010683265.1 PREDICTED: uncharacterized protein LOC104897980 [Beta vulgaris subsp. vulgaris] >KMT07138.1 hypothetical protein BVRB_6g154660 [Beta vulgaris subsp. vulgaris]	gi|470124904|ref|XM_004298401.1|:PREDICTED: Fragaria vesca subsp. vesca uncharacterized LOC101310896 (LOC101310896), mRNA	A0A0J8C524|A0A0J8C524_BETVU:Uncharacterized protein;OS=Beta vulgaris subsp. vulgaris.;

geneid	trend	GO	Pathway	TF	NR	NT	Uniprot
DN125485_c0_g1_i3	DOWN	[GO:0097008:(3E)-4,8-dimethyl-1,3,7-nonatriene synthase activity][GO:0097007:4,8,12-trimethyltrideca-1,3,7,11-tetraene synthase activity][GO:0020037:heme binding][GO:0005506:iron ion binding][GO:0046246:terpene biosynthetic process]	[ko00904:Diterpenoid biosynthesis]	Thecc1EG000515t1:Theobroma cacao|B3|B3 family protein	XP_021864443.1:cytochrome P450 82G1like [Spinacia oleracea]	-	A0A0J8BAT5|A0A0J8BAT5_BETVU:Uncharacterized protein;OS=Beta vulgaris subsp. vulgaris.;|GO:0097008,GO:0097007,GO:0020037,GO:0005506,|GO:0046246,
DN87496_c0_g2_i1	DOWN	-	-	-	-	-	-
DN88671_c0_g2_i2	DOWN	-	-	-	XP_010691165.1:PREDICTED: uncharacterized protein LOC104904576 [Beta vulgaris subsp. vulgaris] >KMT00689.1 hypothetical protein BVRB_9g219770 [Beta vulgaris subsp. vulgaris]	-	A0A0J8BKW7|A0A0J8BKW7_BETVU: Uncharacterized protein;OS=Beta vulgaris subsp. vulgaris.;

geneid	trend	GO	Pathway	TF	NR	NT	Uniprot
DN119524_c2_g1_i10	DOWN	[GO:0016021:integral component of membrane][GO:0006810:transport]	-	-	XP_021610714.1:mitochondrial phosphate carrier protein 3, mitochondriallike [Manihot esculenta] >OAY52544.1 hypothetical protein MANES_04G092000 [Manihot esculenta]	gi|460379114|ref|XM_004235262.1|:PREDICTED: Solanum lycopersicum phosphate carrier protein, mitochondrial-like (LOC101255971), mRNA	A0A061FZC3|A0A061FZC3_THECC:Phosphate transporter 3,1;OS=Theobroma cacao;|GO:0016021,|GO:0006810,
DN116103_c2_g1_i2	DOWN	-	-	Zmw_sc01994.1.g00160.1:Zoysia matrella|ZF-HD|ZF-HD family protein	XP_010673717.1:PREDICTED: protein GAST1 isoform X1 [Beta vulgaris subsp. vulgaris] >KMT14572.1 hypothetical protein BVRB_4g073420 [Beta vulgaris subsp. vulgaris]	gi|356571976|ref|XM_003554099.1|:PREDICTED: Glycine max protein GAST1-like, transcript variant 2 (LOC100818749), mRNA	A0A0J8CM35|A0A0J8CM35_BETVU: Uncharacterized protein;OS=Beta vulgaris subsp. vulgaris.;

geneid	trend	GO	Pathway	TF	NR	NT	Uniprot
DN126877_c6_g2_i5	DOWN	-	-	-	KMT15128.1:hypothetical protein BVRB_3g062460 [Beta vulgaris subsp. vulgaris]	-	A0A0J8CT63|A0A0J8CT63_BETVU:Uncharacterized protein;OS=Beta vulgaris subsp. vulgaris.;
DN106153_c0_g1_i1	DOWN	-	[ko05205:Proteoglycans in cancer][ko05206:MicroRNAs in cancer]	-	XP_008648420.1:uncharacterized protein LOC100501384 [Zea mays] >AQK78411.1 MA3 domaincontaining protein [Zea mays]	-	A0A1D6CQ58|A0A1D6CQ58_WHEAT:Uncharacterized protein;OS=Triticum aestivum;
DN124178_c5_g1_i2	DOWN	[GO:0003677:DNA binding]	-	Bv5_121120_roao.t1:Beta vulgaris|MYB|MYB family protein	XP_010679296.1:PREDICTED: mybrelated protein Myb4 [Beta vulgaris subsp. vulgaris] >KMT09893.1 hypothetical protein BVRB_5g121120 [Beta vulgaris subsp. vulgaris]	-	A0A0J8CD61|A0A0J8CD61_BETVU:Uncharacterized protein;OS=Beta vulgaris subsp. vulgaris.;|GO:0003677,

geneid	trend	GO	Pathway	TF	NR	NT	Uniprot
DN112627_c0_g1_i1	DOWN	-	-	-	XP_021732273.1:uncharacterized protein LOC110699086 [Chenopodium quinoa]	-	A0A0J8BF24|A0A0J8BF24_BETVU:Uncharacterized protein;OS=Beta vulgaris subsp. vulgaris.;
DN121171_c3_g1_i1	DOWN	-	-	FANhyb_rscf00002064.1.g00002.1:Fragaria x ananassa|B3|B3 family protein	XP_021738006.1:uncharacterized protein LOC110704518 [Chenopodium quinoa]	gi|502124744|ref|XM_004498597.1|:PREDICTED: Cicer arietinum uncharacterized LOC101508759 (LOC101508759), mRNA	A0A0J8FLQ1|A0A0J8FLQ1_BETVU:Uncharacterized protein;OS=Beta vulgaris subsp. vulgaris.;

geneid	trend	GO	Pathway	TF	NR	NT	Uniprot
DN124568_c0_g1_i2	DOWN	[GO:0005618:cell wall][GO:0005576:extracellular region][GO:0016021:integral component of membrane][GO:0045330:aspartyl esterase activity][GO:0004857:enzyme inhibitor activity][GO:0030599:pectinesterase activity][GO:0042545:cell wall modification][GO:0045490:pectin catabolic process]	[ko00040:Pentose and glucuronate interconversions]	MDP0000320566:Malus domestica|NAC|NAC family protein	XP_021758812.1:pectinesteraselike [Chenopodium quinoa]	-	Unreviewed|V9TLK1_TOBAC:Pectinesterase;OS=Nicotiana tabacum;|GO:0005618,GO:0005576,GO:0016021,|GO:0045330,GO:0004857,GO:0030599,|GO:0042545,GO:0045490,
DN117397_c1_g1_i5	DOWN	-	-	-	XP_021733266.1:pirinlike protein [Chenopodium quinoa]	gi|460405070|ref|XM_004247952.1|:PREDICTED: Solanum lycopersicum pirin-like protein-like (LOC101261553), mRNA	A0A0J8CG99|A0A0J8CG99_BETVU:Uncharacterized protein;OS=Beta vulgaris subsp. vulgaris.;
DN124847_c0_g1_i2	DOWN	[GO:0006952:defense response][GO:0009607:response to biotic stimulus]	-	-	KNA16670.1:hypothetical protein SOVF_087080 [Spinacia oleracea]	-	A0A0K9RB14|A0A0K9RB14_SPIOL:Uncharacterized protein;OS=Spinacia oleracea;|GO:00069

geneid	trend	GO	Pathway	TF	NR	NT	Uniprot
							52,GO:0009607,
DN120191_c0_g3_i2	DOWN	[GO:0016021:integral component of membrane][GO:0050662:coenzyme binding][GO:0016857:racemase and epimerase activity, acting on carbohydrates and derivatives][GO:0005975:carbohydrate metabolic process]	[ko00520:Amino sugar and nucleotide sugar metabolism]	MDP0000247737:Malus domestica|FAR1|FAR1 family protein	KNA25764.1:hypothetical protein SOVF_003910 [Spinacia oleracea]	gi|225433490|ref|XM_002265052.1|:PREDICTED: Vitis vinifera UDP-glucuronate 4-epimerase 6-like (LOC100241904), mRNA	A0A0K9S3G2|A0A0K9S3G2_SPIOL:Uncharacterized protein;OS=Spinacia oleracea;|GO:0016021,|GO:0050662,GO:0016857,|GO:0005975,
DN124746_c1_g4_i1	DOWN	[GO:0010287:plastoglobule][GO:0016702:oxidoreductase activity, acting on single donors with incorporation of molecular oxygen, incorporation of two atoms of oxygen]	[ko00906:Carotenoid biosynthesis]	-	XP_010677394.1:PREDICTED: probable carotenoid cleavage dioxygenase 4, chloroplastic [Beta vulgaris subsp. vulgaris] >KMT11394.1 hypothetical protein BVRB_5g107230	-	A0A0J8CH14|A0A0J8CH14_BETVU:Uncharacterized protein;OS=Beta vulgaris subsp. vulgaris.;|GO:0010287,|GO:0016702,

geneid	trend	GO	Pathway	TF	NR	NT	Uniprot
					[Beta vulgaris subsp. vulgaris]		
DN95333_c0_g1_i2	DOWN	[GO:0006952:defense response][GO:0009607:response to biotic stimulus]	-	-	XP_010687260.1:PREDICTED: major allergen Pru ar 1 [Beta vulgaris subsp. vulgaris] >KMT03737.1 hypothetical protein BVRB_8g188860 [Beta vulgaris subsp. vulgaris]	-	A0A0J8BRC2|A0A0J8BRC2_BETVU:Uncharacterized protein;OS=Beta vulgaris subsp. vulgaris.;|GO:0006952,GO:0009607,
DN119252_c2_g1_i1	DOWN	-	-	-	-	-	-
DN116172_c1_g1_i2	DOWN	-	-	-	-	-	-

geneid	trend	GO	Pathway	TF	NR	NT	Uniprot
DN122252_c2_g1_i6	DOWN	[GO:0016747:transferase activity, transferring acyl groups other than amino-acyl groups][GO:0009058:biosynthetic process]	[ko00941:Flavonoid biosynthesis][ko04712:Circadian rhythm - plant]	-	XP_021747699.1:chalcone synthaselike [Chenopodium quinoa]	gi|306415504|gb|HQ161731.1|:Lilium hybrid cultivar Siberia chalcone synthase mRNA, complete cds	A0A0K9RJ90|A0A0K9RJ90_SPIOL:Uncharacterized protein;OS=Spinacia oleracea;|GO:0016747,|GO:0009058,
DN122008_c9_g3_i1	DOWN	-	-	-	-	-	-
DN124234_c3_g1_i1	DOWN	[GO:0016021:integral component of membrane][GO:0020037:heme binding][GO:0005506:iron ion binding][GO:0004497:monooxygenase activity][GO:0016705:oxidoreductase activity, acting on paired donors, with incorporation or reduction of molecular oxygen]	[ko00940:Phenylpropanoid biosynthesis]	676766070:Sisymbrium irio|bHLH|bHLH family protein	XP_021718110.1:cytochrome P450 84A1like [Chenopodium quinoa]	gi|502161258|ref|XM_004512040.1|:PREDICTED: Cicer arietinum cytochrome P450 84A1-like (LOC101491312), mRNA	E0WCK5|E0WCK5_EUCGL:Ferulate 5-hydroxylase;OS=Eucalyptus globulus;|GO:0016021,|GO:0020037,GO:0005506,GO:0004497,GO:0016705,

geneid	trend	GO	Pathway	TF	NR	NT	Uniprot
DN121715_c1_g1_i2	DOWN	[GO:0020037:heme binding][GO:0005506:iron ion binding][GO:0004497:monooxygenase activity][GO:0016705:oxidoreductase activity, acting on paired donors, with incorporation or reduction of molecular oxygen]	-	CCG000365.1:Populus euphratica|NAC|NAC family protein	XP_021850062.1:alkane hydroxylase MAH1like [Spinacia oleracea]	-	A0A0J8C9H2|A0A0J8C9H2_BETVU:Uncharacterized protein;OS=Beta vulgaris subsp. vulgaris.;|GO:0020037,GO:0005506,GO:0004497,GO:0016705,
DN123742_c1_g4_i1	DOWN	[GO:0003676:nucleic acid binding]	-	-	XP_010694404.1:PREDICTED: uncharacterized protein LOC104907208 [Beta vulgaris subsp. vulgaris]	-	F4NCI7|F4NCI7_BETVU:Uncharacterized protein;OS=Beta vulgaris subsp. vulgaris.;|GO:0003676,
DN114941_c0_g1_i3	DOWN	-	-	-	-	-	-

geneid	trend	GO	Pathway	TF	NR	NT	Uniprot
DN113891_c0_g2_i1	DOWN	-	-	-	XP_021849583.1:remorinlike [Spinacia oleracea]	gi|350536268|ref|NM_001247309.1|:Solanum lycopersicum remorin 2 (rem-2), mRNA >gi|4883529|gb|AF123266.2|AF123266 Lycopersicon esculentum remorin 2 (rem-2) mRNA, complete cds	A0A0J8D392|A0A0J8D392_BETVU:Uncharacterized protein;OS=Beta vulgaris subsp. vulgaris.;
DN117888_c2_g1_i1	DOWN	-	-	-	-	-	-
DN124496_c2_g10_i1	DOWN	-	-	DCAR_025729:Daucus carota|B3|B3 family protein	XP_010692733.1:PREDICTED: protein PLANT CADMIUM RESISTANCE 2like [Beta vulgaris subsp. vulgaris]	-	A0A0K9RJW7|A0A0K9RJW7_SPIOL: Uncharacterized protein;OS=Spinacia oleracea;
DN85552_c0_g1_i1	DOWN	-	-	-	-	-	-

geneid	trend	GO	Pathway	TF	NR	NT	Uniprot
DN119146_c0_g3_i1	DOWN	[GO:0016021:integral component of membrane][GO:0020037:heme binding][GO:0005506:iron ion binding][GO:0004497:monooxygenase activity][GO:0016705:oxidoreductase activity, acting on paired donors, with incorporation or reduction of molecular oxygen]	-	Cc10_g03650:Coffea canephora|G2-like|G2-like family protein	XP_010682779.1:PREDICTED: cytochrome P450 78A5 [Beta vulgaris subsp. vulgaris] >KMT07469.1 hypothetical protein BVRB_6g150870 [Beta vulgaris subsp. vulgaris]	-	A0A0J8C5E8|A0A0J8C5E8_BETVU:Uncharacterized protein;OS=Beta vulgaris subsp. vulgaris.;|GO:0016021,|GO:0020037,GO:0005506,GO:0004497,GO:0016705,
DN126748_c3_g3_i1	DOWN	[GO:0005618:cell wall][GO:0005576:extracellular region][GO:0016020:membrane][GO:0009664:plant-type cell wall organization]	-	676732396:Sisymbrium irio|bHLH|bHLH family protein	ADN03964.1:EXP1 [Phytolacca acinosa]	gi|28624705|gb|AY079206.1|:Mirabilis jalapa cell wall protein Exp4 precursor, mRNA, complete cds	E2D554|E2D554_PHYAN:EXP1;OS=Phytolacca acinosa;|GO:0005618,GO:0005576,GO:0016020,|GO:0009664,
DN116771_c0_g2_i3	DOWN	[GO:0016021:integral component of membrane][GO:0008375:acetylglucosaminyltransferase activity]	-	Achn337101:Actinidia chinensis|Dof|Dof family protein	XP_021843674.1:betaglucuronosyltransferase GlcAT14B [Spinacia oleracea] >KNA19095.1 hypothetical protein	gi|255585737|ref|XM_002533504.1|:Ricinus communis acetylglucosaminyltransferase, putative, mRNA	A0A0K9RHY7|A0A0K9RHY7_SPIOL: Uncharacterized protein;OS=Spinacia oleracea;|GO:00160 21,|GO:0008375,

geneid	trend	GO	Pathway	TF	NR	NT	Uniprot
					SOVF_064790 [Spinacia oleracea]		
DN108401_c0_g1_i1	DOWN	[GO:0016442:RISC complex][GO:0003712:transcription coregulator activity][GO:0035194:posttranscriptional gene silencing by RNA]	[ko05169:Epstein-Barr virus infection][ko05203:Viral carcinogenesis]	-	XP_015955763.1:ribonuclease TUDOR 1 [Arachis duranensis] >XP_015955764.1 ribonuclease TUDOR 1 [Arachis duranensis]	-	A0A0S3QXZ8|A0A0S3QXZ8_PHAAN: Uncharacterized protein;OS=Vigna angularis var. angularis.;|GO:0016442,|GO:0003712,|GO:0035194,
DN126023_c1_g2_i6	DOWN	[GO:0010181:FMN binding][GO:0016491:oxidoreductase activity]	[ko00592:alpha-Linolenic acid metabolism]	Zmw_sc00338.1.g00010.1:Zoysia matrella|NAC|NAC family protein	XP_015889051.1:PREDICTED: 12oxophytodienoate reductase 3like [Ziziphus jujuba]	-	A0A0B0P2H6|A0A0B0P2H6_GOSAR: 12-oxophytodienoate reductase 3;OS=Gossypium arboreum;|GO:0010181,GO:0016491,

geneid	trend	GO	Pathway	TF	NR	NT	Uniprot
DN115157_c0_g1_i1	DOWN	-	-	Pbr002083.1:Pyrus bretschneideri|ERF|ERF family protein	XP_010674501.1:PREDICTED: PLASMODESMATA CALLOSEBINDING PROTEIN 4 [Beta vulgaris subsp. vulgaris]	-	A0A0J8CQ50|A0A0J8CQ50_BETVU:Uncharacterized protein;OS=Beta vulgaris subsp. vulgaris.;
DN123054_c2_g3_i7	DOWN	[GO:0008171:O-methyltransferase activity]	-	-	XP_010690296.1:PREDICTED: (RS)norcoclaurine 6Omethyltransferase [Beta vulgaris subsp. vulgaris] >KMT01317.1 hypothetical protein BVRB_9g213330 [Beta vulgaris subsp. vulgaris]	-	A0A0J8BJX2|A0A0J8BJX2_BETVU:Uncharacterized protein;OS=Beta vulgaris subsp. vulgaris.;|GO:0008171,
DN92943_c0_g1_i1	DOWN	-	-	-	-	-	-

geneid	trend	GO	Pathway	TF	NR	NT	Uniprot
DN123703_c2_g1_i8	DOWN	[GO:0016021:integral component of membrane][GO:0005886:plasma membrane][GO:0015112:nitrate transmembrane transporter activity][GO:0006857:oligopeptide transport][GO:0010167:response to nitrate][GO:0009414:response to water deprivation]	-	mrna31023.1-v1.0-hybrid:Fragaria vesca|C3H|C3H family protein	XP_021768447.1:protein NRT1/ PTR FAMILY 6.3like [Chenopodium quinoa]	gi|449459765|ref|XM_004147569.1|:PREDICTED: Cucumis sativus nitrate transporter 1.1-like (LOC101222104), mRNA >gi|449528354|ref|XM_004171122.1| PREDICTED: Cucumis sativus nitrate transporter 1.1-like (LOC101222104), mRNA	A0A0J8F9I8|A0A0J8F9I8_BETVU:Uncharacterized protein;OS=Beta vulgaris subsp. vulgaris.;|GO:0016021,GO:0005886,|GO:0015112,|GO:0006857,GO:0010167,GO:0009414,
DN124867_c3_g1_i11	DOWN	-	[ko00350:Tyrosine metabolism][ko00360:Phenylalanine metabolism]	-	XP_021744792.1:macrophage migration inhibitory factor homolog [Chenopodium quinoa]	-	A0A0K9RS80|A0A0K9RS80_SPIOL:Uncharacterized protein;OS=Spinacia oleracea;

geneid	trend	GO	Pathway	TF	NR	NT	Uniprot
DN121071_c0_g1_i1	DOWN	[GO:0031225:anchored component of membrane][GO:0016021:integral component of membrane][GO:0016049:cell growth][GO:0010215:cellulose microfibril organization]	-	Sopen01g046270.1:Solanum pennellii|M-type_MADS|M-type_MADS family protein	KDP24286.1:hypothetical protein JCGZ_25582 [Jatropha curcas]	-	A0A067JJZ4|A0A067JJZ4_JATCU:Uncharacterized protein;OS=Jatropha curcas;|GO:0031225,GO:0016021,|GO:0016049,GO:0010215,
DN127828_c1_g15_i2	DOWN	-	[ko00480:Glutathione metabolism][ko00980:Metabolism of xenobiotics by cytochrome P450][ko00982:Drug metabolism - cytochrome P450][ko01524:Platinum drug resistance][ko05204:Chemical carcinogenesis][ko05418:Fluid shear stress and atherosclerosis]	-	ADN03963.1:GST1 [Phytolacca acinosa]	-	E2D553|E2D553_PHYAN:GST1;OS=Phytolacca acinosa;

geneid	trend	GO	Pathway	TF	NR	NT	Uniprot
DN126417_c1_g1_i4	DOWN	[GO:0016021:integral component of membrane][GO:0015297:antiporter activity]	-	Zpz_sc01010.1.g00110.1.am.mk:Zoysia pacifica|MYB|MYB family protein	XP_010681038.1:PREDICTED: protein DETOXIFICATION 27 [Beta vulgaris subsp. vulgaris] >KMT08611.1 hypothetical protein BVRB_6g138880 [Beta vulgaris subsp. vulgaris]	-	A0A0J8EZG0|A0A0J8EZG0_BETVU:Protein DETOXIFICATION ;OS=Beta vulgaris subsp. vulgaris.;|GO:0016021,|GO:0015297,GO:0015238,
DN109527_c1_g5_i1	DOWN	-	-	-	-	-	-
DN105642_c0_g1_i1	DOWN	[GO:0016491:oxidoreductase activity]	[ko00040:Pentose and glucuronate interconversions][ko00051:Fructose and mannose metabolism][ko00052:Galactose metabolism][ko00561:Glycerolipid metabolism]	-	GAQ80469.1:alcohol dehydrogenase (NADP+) [Klebsormidium nitens]	-	F0Y0Z9|F0Y0Z9_AURAN:Putative uncharacterized protein;OS=Aureococcus anophagefferens;|GO:0016491,

geneid	trend	GO	Pathway	TF	NR	NT	Uniprot
DN120512_c1_g1_i3	DOWN	-	-	-	XP_010692836.1:PREDICTED: uncharacterized protein LOC104905882 [Beta vulgaris subsp. vulgaris] >KMS99561.1 hypothetical protein BVRB_1g022880 [Beta vulgaris subsp. vulgaris]	-	A0A0J8BF54|A0A0J8BF54_BETVU:Uncharacterized protein;OS=Beta vulgaris subsp. vulgaris.;
DN113891_c0_g1_i1	DOWN	-	-	Achn056131:Actinidia chinensis|NF-YC|NF-YC family protein	XP_021717540.1:remorinlike [Chenopodium quinoa]	gi|502157244|ref|XM_004510749.1|:PREDICTED: Cicer arietinum remorin-like (LOC101514413), mRNA	M5X106|M5X106_PRUPE:Uncharacterized protein;OS=Prunus persica;
DN116799_c0_g1_i6	DOWN	[GO:0016021:integral component of membrane]	-	Zmw_sc02218.1.g00160.1:Zoysia matrella|bHLH|bHLH family protein	XP_021845251.1:uncharacterized protein LOC110785115 [Spinacia oleracea] >KNA09651.1 hypothetical protein	-	A0A0K9QT15|A0A0K9QT15_SPIOL:Uncharacterized protein;OS=Spinacia oleracea;|GO:0016021,

geneid	trend	GO	Pathway	TF	NR	NT	Uniprot
					SOVF_151610 [Spinacia oleracea]		
DN124100_c6_g3_i2	DOWN	-	-	-	-	-	-
DN125118_c3_g5_i1	DOWN	[GO:0003676:nucleic acid binding][GO:0004523:RNA-DNA hybrid ribonuclease activity]	-	-	CCA66009.1:hypothetical protein [Beta vulgaris subsp. vulgaris]	-	F4NCH2|F4NCH2_BETVU:Uncharacterized protein;OS=Beta vulgaris subsp. vulgaris.;|GO:0003676,GO:0004523,

geneid	trend	GO	Pathway	TF	NR	NT	Uniprot
DN122425_c0_g1_i4	DOWN	[GO:0016021:integral component of membrane][GO:0020037:heme binding][GO:0005506:iron ion binding][GO:0016709:oxidoreductase activity, acting on paired donors, with incorporation or reduction of molecular oxygen, NAD(P)H as one donor, and incorporation of one atom of oxygen][GO:0016132:brassinosteroid biosynthetic process][GO:0009965:leaf morphogenesis][GO:0042814:monopolar cell growth][GO:0048441:petal development][GO:0048443:stamen development]	[ko00905:Brassinosteroid biosynthesis]	Gh_A02G0666:Gossypium hirsutum|G2-like|G2-like family protein	XP_010679812.1:PREDICTED: 3epi6deoxocathasterone 23monooxygenase [Beta vulgaris subsp. vulgaris] >KMT09488.1 hypothetical protein BVRB_6g129580 [Beta vulgaris subsp. vulgaris]	gi|511318860|gb|GAKE01014017.1|:TSA: Thlaspi arvense Contig14235 transcribed RNA sequence	A0A0J8CBZ6|A0A0J8CBZ6_BETVU:Uncharacterized protein;OS=Beta vulgaris subsp. vulgaris.;|GO:0016021,|GO:0020037,GO:0005506,GO:0016709,|GO:0016132,GO:0009965,GO:0042814,GO:0048441,GO:0048443,
DN91199_c0_g1_i1	DOWN	-	-	-	-	-	-

geneid	trend	GO	Pathway	TF	NR	NT	Uniprot
DN126658_c0_g3_i2	DOWN	[GO:0016788:hydrolase activity, acting on ester bonds]	-	OB04G28180.1:Oryza brachyantha|GRAS|GRAS family protein	XP_004492179.1:PREDICTED: uncharacterized protein LOC101497919 [Cicer arietinum]	-	Q7XDX0|Q7XDX0_ORYSJ:Transposon protein, putative, Pong sub-class;OS=Oryza sativa subsp. japonica;GN=LOC_Os10g31130 {ECO:0000313|EMBL:AAP54021.1};|GO:0016788,
DN120376_c0_g9_i4	DOWN	[GO:0016021:integral component of membrane]	[ko04721:Synaptic vesicle cycle][ko04723:Retrograde endocannabinoid signaling][ko04727:GABAergic synapse][ko05032:Morphine addiction][ko05033:Nicotine addiction]	-	XP_010691502.1:PREDICTED: amino acid transporter ANT1like [Beta vulgaris subsp. vulgaris] >KMT00974.1 hypothetical protein BVRB_9g222370 [Beta vulgaris subsp. vulgaris]	-	A0A0J8BLR7|A0A0J8BLR7_BETVU:Uncharacterized protein;OS=Beta vulgaris subsp. vulgaris.;|GO:0016021,

geneid	trend	GO	Pathway	TF	NR	NT	Uniprot
DN128542_c0_g1_i1	DOWN	-	-	-	XP_010682959.1:PREDICTED: uncharacterized protein LOC104897720 isoform X1 [Beta vulgaris subsp. vulgaris]	-	A0A0J8BZA1|A0A0J8BZA1_BETVU:Uncharacterized protein;OS=Beta vulgaris subsp. vulgaris.;
DN120421_c1_g1_i2	DOWN	[GO:0004190:aspartic-type endopeptidase activity]	-	Aco017242.1:Ananas comosus|Trihelix|Trihelix family protein	XP_021853223.1:aspartic proteinase nepenthesin1 [Spinacia oleracea]	gi|521534704|gb|GAHZ01006005.1|:TSA: Salicornia europaea Unigene6310_SeCKS transcribed RNA sequence	A0A0J8CKC3|A0A0J8CKC3_BETVU: Uncharacterized protein;OS=Beta vulgaris subsp. vulgaris.;|GO:0004190,
DN125251_c1_g3_i3	DOWN	-	-	-	-	-	-
DN129175_c15_g7_i3	DOWN	-	-	-	-	-	-

geneid	trend	GO	Pathway	TF	NR	NT	Uniprot
DN117880_c0_g2_i1	DOWN	[GO:0005576:extracellular region][GO:0020037:heme binding][GO:0046872:metal ion binding][GO:0004601:peroxidase activity][GO:0042744:hydrogen peroxide catabolic process][GO:0006979:response to oxidative stress]	[ko00940:Phenylpropanoid biosynthesis]	EPS67213.1:Genlisea aurea|NAC|NAC family protein	XP_021714590.1:peroxidase 72like [Chenopodium quinoa]	gi|7453850|gb|AF244922.1|AF244922:Spinacia oleracea peroxidase prx13 precursor, mRNA, complete cds	A0A0J8C6W9|A0A0J8C6W9_BETVU: Peroxidase;OS=Beta vulgaris subsp. vulgaris.;|GO:0005576,|GO:0020037,GO:0046872,GO:0004601,|GO:0042744,GO:0006979,
DN122230_c4_g1_i13	DOWN	-	-	-	XP_004135232.1:PREDICTED: uncharacterized protein LOC101212200 [Cucumis sativus] >KGN51824.1 hypothetical protein Csa_5G602230 [Cucumis sativus]	gi|225898033|dbj|AB493511.1|:Arabidopsis thaliana At1g60060 mRNA for hypothetical protein, partial cds, clone: RAAt1g60060	A0A0A0KSE0|A0A0A0KSE0_CUCSA: Uncharacterized protein;OS=Cucumis sativus;

geneid	trend	GO	Pathway	TF	NR	NT	Uniprot
DN124746_c1_g3_i2	DOWN	[GO:0016702:oxidoreductase activity, acting on single donors with incorporation of molecular oxygen, incorporation of two atoms of oxygen]	[ko00906:Carotenoid biosynthesis]	-	XP_021851772.1:probable carotenoid cleavage dioxygenase 4, chloroplastic [Spinacia oleracea] >KNA23039.1 hypothetical protein SOVF_028540 [Spinacia oleracea]	-	A0A0K9RVT5|A0A0K9RVT5_SPIOL: Uncharacterized protein;OS=Spinacia oleracea;|GO:0016702,
DN129077_c2_g1_i3	DOWN	-	-	-	-	-	-
DN118252_c0_g4_i5	DOWN	[GO:0016021:integral component of membrane][GO:0005509:calcium ion binding][GO:0016174:NAD(P)H oxidase activity][GO:0004601:peroxidase activity][GO:0009408:response to heat][GO:0009845:seed germination]	[ko04016:MAPK signaling pathway - plant][ko04626:Plant-pathogen interaction]	-	XP_010674039.1:PREDICTED: respiratory burst oxidase homolog protein B [Beta vulgaris subsp. vulgaris] >KMT14211.1 hypothetical protein BVRB_4g075730 [Beta vulgaris subsp. vulgaris]	gi|521674929|gb|GAIA01029936.1|:TSA: Salicornia europaea Unigene32832_Se200S transcribed RNA sequence	A0A0J8FEF7|A0A0J8FEF7_BETVU:Uncharacterized protein;OS=Beta vulgaris subsp. vulgaris.;|GO:0016021,|GO:0005509,GO:0016174,GO:0004601,|GO:0009408,GO:0009845,

geneid	trend	GO	Pathway	TF	NR	NT	Uniprot
DN126905_c2_g1_i4	DOWN	[GO:0016021:integral component of membrane]	-	Do008886.1:Dichanthelium oligosanthes|NAC|NAC family protein	XP_010689087.1:PREDICTED: uncharacterized membrane protein At1g06890 [Beta vulgaris subsp. vulgaris] >KMT02300.1 hypothetical protein BVRB_9g205250 [Beta vulgaris subsp. vulgaris]	gi|224131999|ref|XM_002328124.1|:Populus trichocarpa predicted protein, mRNA	A0A0J8BLL6|A0A0J8BLL6_BETVU:Uncharacterized protein;OS=Beta vulgaris subsp. vulgaris.;|GO:0016021,
DN119612_c4_g1_i1	DOWN	[GO:0005524:ATP binding][GO:0004672:protein kinase activity]	-	GSVIVT01019630001:Vitis vinifera|MIKC_MADS|MIKC_MADS family protein	XP_010689238.1:PREDICTED: probable serine/threonineprotein kinase WNK6 [Beta vulgaris subsp. vulgaris] >KMT02282.1 hypothetical protein BVRB_9g206000 [Beta vulgaris subsp. vulgaris]	-	A0A0J8BM43|A0A0J8BM43_BETVU: Uncharacterized protein;OS=Beta vulgaris subsp. vulgaris.;|GO:0005524,GO:0004672,

geneid	trend	GO	Pathway	TF	NR	NT	Uniprot
DN118165_c2_g2_i1	DOWN	-	-	-	XP_010691165.1:PREDICTED: uncharacterized protein LOC104904576 [Beta vulgaris subsp. vulgaris] >KMT00689.1 hypothetical protein BVRB_9g219770 [Beta vulgaris subsp. vulgaris]	-	A0A0J8BKW7|A0A0J8BKW7_BETVU: Uncharacterized protein;OS=Beta vulgaris subsp. vulgaris.;
DN125273_c0_g5_i1	DOWN	[GO:0016020:membrane][GO:0046872:metal ion binding][GO:0030570:pectate lyase activity][GO:0045490:pectin catabolic process]	[ko00040:Pentose and glucuronate interconversions][ko02024:Quorum sensing]	Pbr039133.1:Pyrus bretschneideri|AP2|AP2 family protein	XP_021720205.1:probable pectate lyase 18 [Chenopodium quinoa]	gi|521479186|gb|GAHZ01051700.1|:TSA: Salicornia europaea Unigene60976_SeCKS transcribed RNA sequence	A0A0J8B8B9|A0A0J8B8B9_BETVU:Pectate lyase;OS=Beta vulgaris subsp. vulgaris.;|GO:0016020,|GO:0046872,GO:0030570,|GO:0045490,
DN127790_c1_g8_i3	DOWN	-	-	-	-	-	-

geneid	trend	GO	Pathway	TF	NR	NT	Uniprot
DN117146_c6_g4_i3	DOWN	[GO:0005576:extracellular region][GO:0030145:manganese ion binding][GO:0045735:nutrient reservoir activity]	-	-	XP_021863187.1:putative germinlike protein 21 [Spinacia oleracea]	-	A0A0J8D8V3|A0A0J8D8V3_BETVU:Uncharacterized protein;OS=Beta vulgaris subsp. vulgaris.;|GO:0005576,|GO:0030145,GO:0045735,
DN119833_c0_g5_i1	DOWN	-	-	-	-	-	-
DN123788_c3_g1_i1	DOWN	-	-	-	XP_010052189.1:PREDICTED: uncharacterized protein LOC104440898 [Eucalyptus grandis] >KCW76108.1 hypothetical protein EUGRSUZ_D00486 [Eucalyptus grandis]	-	A0A059CCC6|A0A059CCC6_EUCGR:Uncharacterized protein;OS=Eucalyptus grandis;
DN111836_c1_g1_i3	DOWN	-	-	-	-	-	-

geneid	trend	GO	Pathway	TF	NR	NT	Uniprot
DN125031_c0_g2_i12	DOWN	[GO:0016874:ligase activity][GO:0008270:zinc ion binding]	-	KN540739.1_FGP003:Oryza longistaminata|M-type_MADS|M-type_MADS family protein	AFN89139.1:RINGtype ubiquitin ligase [Mesembryanthemum crystallinum]	gi|396924950|gb|JN558780.1|:Mesembryanthemum crystallinum RING-type ubiquitin ligase (CPN1) mRNA, complete cds	J7FH94|J7FH94_MESCR:RING-type ubiquitin ligase;OS=Mesembryanthemum crystallinum;|GO:0016874,GO:0008270,
DN126877_c6_g2_i2	DOWN	-	-	-	-	-	-
DN120753_c1_g1_i1	DOWN	-	-	-	-	-	-
DN123090_c1_g1_i10	DOWN	[GO:0005634:nucleus][GO:0003677:DNA binding][GO:0003700:DNA-binding transcription factor activity][GO:0007275:multicellular organism development][GO:0006351:transcription, DNA-templated]	-	XP_010109804.1:Morus notabilis|AP2|AP2 family protein	XP_021631445.1:floral homeotic protein APETALA 2 isoform X2 [Manihot esculenta]	gi|388329712|gb|JQ398741.1|:Camellia sinensis transcription factor APETALA2 mRNA, complete cds	W9S3F7|W9S3F7_9ROSA:Floral homeotic protein APETALA 2;OS=Morus notabilis.;|GO:0005634,|GO:0003677,GO:0003700,|GO:0007275,GO:0006351,

geneid	trend	GO	Pathway	TF	NR	NT	Uniprot
DN127142_c4_g1_i4	DOWN	[GO:0004553:hydrolase activity, hydrolyzing O-glycosyl compounds][GO:0005975:carbohydrate metabolic process]	[ko00520:Amino sugar and nucleotide sugar metabolism]	Zjn_sc00096.1.g00310.1.sm.mk:Zoysia japonica|C2H2|C2H2 family protein	XP_021764959.1:acidic endochitinaselike [Chenopodium quinoa]	gi|502094103|ref|XM_004490083.1|:PREDICTED: Cicer arietinum acidic endochitinase-like (LOC101495610), mRNA	A0A0J8CR98|A0A0J8CR98_BETVU:Uncharacterized protein;OS=Beta vulgaris subsp. vulgaris.;|GO:0004553,|GO:0005975,
DN126983_c6_g7_i2	DOWN	[GO:0008171:O-methyltransferase activity]	[ko00940:Phenylpropanoid biosynthesis]	-	XP_021844176.1:caffeic acid 3Omethyltransferase like [Spinacia oleracea]	-	A0A0K9QFL0|A0A0K9QFL0_SPIOL:Uncharacterized protein;OS=Spinacia oleracea;|GO:0008171,
DN110379_c0_g1_i1	DOWN	[GO:0016021:integral component of membrane][GO:0005524:ATP binding][GO:0016887:ATPase activity][GO:0006810:transport]	-	Rsa1.0_03996.1_g00001.1:Raphanus sativus|MYB_related|MYB_related family protein	XP_021736787.1:pleiotropic drug resistance protein 1like [Chenopodium quinoa]	gi|521718059|gb|GAIA01011766.1|:TSA: Salicornia europaea Unigene12383_Se200S transcribed RNA sequence	A0A0K9QP53|A0A0K9QP53_SPIOL:Uncharacterized protein;OS=Spinacia oleracea;|GO:0016021,|GO:0005524,GO:0016887,|GO:0006810,

geneid	trend	GO	Pathway	TF	NR	NT	Uniprot
DN104542_c0_g1_i1	DOWN	-	-	-	XP_021757549.1:protein SPIRAL1like 5 [Chenopodium quinoa]	-	A0A0J8CIY2|A0A0J8CIY2_BETVU:Uncharacterized protein;OS=Beta vulgaris subsp. vulgaris.;
DN104698_c0_g1_i1	DOWN	-	-	-	-	-	-
DN113127_c0_g3_i4	DOWN	-	-	-	-	-	-
DN108246_c0_g1_i1	DOWN	[GO:0016021:integral component of membrane][GO:0008270:zinc ion binding]	-	-	KMS98241.1:hypothetical protein BVRB_4g094420 [Beta vulgaris subsp. vulgaris]	-	A0A0J8BBH2|A0A0J8BBH2_BETVU: Uncharacterized protein;OS=Beta vulgaris subsp. vulgaris.;|GO:0016021,|GO:0008270,
DN122397_c5_g11_i1	DOWN	-	-	-	-	-	-
DN112354_c4_g7_i1	DOWN	-	-	-	-	-	-
DN106005_c0_g1_i1	DOWN	-	-	-	-	-	-

geneid	trend	GO	Pathway	TF	NR	NT	Uniprot
DN127451_c2_g2_i12	DOWN	[GO:0008270:zinc ion binding][GO:0006355:regulation of transcription, DNA-templated]	-	Dca51545.1:Dianthus caryophyllus|FAR1|FAR1 family protein	XP_021841647.1:protein FAR1RELATED SEQUENCE 5like [Spinacia oleracea]	-	A0A199V309|A0A199V309_ANACO:Protein FAR1-RELATED SEQUENCE 5;OS=Ananas comosus;|GO:0008270,|GO:0006355,
DN112454_c0_g1_i1	DOWN	-	-	-	XP_010675675.1:PREDICTED: fatty acid desaturase 4, chloroplastic [Beta vulgaris subsp. vulgaris] >KMT12690.1 hypothetical protein BVRB_4g087880 [Beta vulgaris subsp. vulgaris]	-	A0A0J8CG00|A0A0J8CG00_BETVU:Uncharacterized protein;OS=Beta vulgaris subsp. vulgaris.;
DN122338_c2_g1_i4	DOWN	-	-	-	XP_021769390.1:uncharacterized protein LOC110733628 [Chenopodium quinoa]	-	A0A068UHH9|A0A068UHH9_COFCA:Uncharacterized protein;OS=Coffea canephora;

geneid	trend	GO	Pathway	TF	NR	NT	Uniprot
DN111603_c0_g1_i1	DOWN	[GO:0008408:3'-5' exonuclease activity][GO:0003677:DNA binding][GO:0003887:DNA-directed DNA polymerase activity][GO:0001882:nucleoside binding][GO:0000166:nucleotide binding][GO:0006270:DNA replication initiation]	[ko00230:Purine metabolism][ko00240:Pyrimidine metabolism][ko03030:DNA replication]	-	XP_010667413.1:PREDICTED: DNA polymerase alpha catalytic subunit [Beta vulgaris subsp. vulgaris] >KMS95401.1 hypothetical protein BVRB_008590 [Beta vulgaris subsp. vulgaris]	-	A0A0J8B325|A0A0J8B325_BETVU:DNA polymerase;OS=Beta vulgaris subsp. vulgaris.;|GO:0008408,GO:0003677,GO:0003887,GO:0001882,GO:0000166,|GO:0006270,
DN127674_c1_g1_i1	DOWN	-	-	-	XP_021715637.1:fasciclinlike arabinogalactan protein 17 isoform X1 [Chenopodium quinoa]	gi|356572649|ref|XM_003554432.1|:PREDICTED: Glycine max fasciclin-like arabinogalactan protein 16-like (LOC100818575), mRNA	F6I3P9|F6I3P9_VITVI:Putative uncharacterized protein;OS=Vitis vinifera;GN=VIT_08s0032g00910 {ECO:0000313|EMBL:CCB61559.1};
DN123058_c0_g2_i1	DOWN	[GO:0005524:ATP binding][GO:0004672:protein kinase activity]	-	GSBRNA2T00046376001:Brassica napus|S1Fa-like|S1Fa-like family protein	XP_010691277.1:PREDICTED: receptor like protein kinase S.2 [Beta vulgaris subsp. vulgaris]	-	A0A0J8BH99|A0A0J8BH99_BETVU:Uncharacterized protein;OS=Beta vulgaris subsp. vulgaris.;|GO:00055

geneid	trend	GO	Pathway	TF	NR	NT	Uniprot
							24,GO:0004672,
DN111356_c0_g2_i1	DOWN	-	-	-	KMT14213.1:hypothetical protein BVRB_4g075750 [Beta vulgaris subsp. vulgaris]	-	A0A0J8CQB5|A0A0J8CQB5_BETVU: Uncharacterized protein;OS=Beta vulgaris subsp. vulgaris.;
DN104612_c0_g1_i1	DOWN	-	-	-	-	gi|169203889|gb|EU520641.1|:Phrynus sp. 1 SEM-2008 mitochondrion, complete genome	-

geneid	trend	GO	Pathway	TF	NR	NT	Uniprot
DN115634_c0_g2_i1	DOWN	[GO:0005743:mitochondrial inner membrane][GO:0045275:respiratory chain complex III][GO:0009055:electron transfer activity][GO:0046872:metal ion binding][GO:0008121:ubiquinol-cytochrome-c reductase activity][GO:0006122:mitochondrial electron transport, ubiquinol to cytochrome c]	[ko00190:Oxidative phosphorylation][ko02020:Two-component system][ko04260:Cardiac muscle contraction][ko04932:Non-alcoholic fatty liver disease (NAFLD)][ko05010:Alzheimer's disease][ko05012:Parkinson's disease][ko05016:Huntington's disease]	-	YP_008816157.1:apocytochrome b (mitochondrion) [Roya obtusa] >AGZ90367.1 apocytochrome b (mitochondrion) [Roya obtusa]	-	A0A097PBM5|A0A097PBM5_CYAPA:Cytochrome b;OS=Cyanophora paradoxa.;|GO:0005743,GO:0045275,|GO:0009055,GO:0046872,GO:0008121,|GO:0006122,
DN127142_c4_g1_i1	DOWN	[GO:0004553:hydrolase activity, hydrolyzing O-glycosyl compounds][GO:0005975:carbohydrate metabolic process]	[ko00520:Amino sugar and nucleotide sugar metabolism]	-	XP_021726781.1:acidic endochitinaselike [Chenopodium quinoa]	-	Q00MX4|Q00MX4_MALDO:Pathogenesis-related protein 8;OS=Malus domestica;|GO:0004553,|GO:0005975,
DN119252_c4_g2_i1	DOWN	-	-	-	-	-	-

geneid	trend	GO	Pathway	TF	NR	NT	Uniprot
DN125485_c0_g3_i2	DOWN	[GO:0097008:(3E)-4,8-dimethyl-1,3,7-nonatriene synthase activity][GO:0097007:4,8,12-trimethyltrideca-1,3,7,11-tetraene synthase activity][GO:0020037:heme binding][GO:0005506:iron ion binding][GO:0046246:terpene biosynthetic process]	[ko00904:Diterpenoid biosynthesis]	Cc10_g03650:Coffea canephora|G2-like|G2-like family protein	XP_010696237.1:PREDICTED: cytochrome P450 82G1 [Beta vulgaris subsp. vulgaris] >KMS97083.1 hypothetical protein BVRB_7g178650 [Beta vulgaris subsp. vulgaris]	gi|521747893|gb|GAIA01001914.1|:TSA: Salicornia europaea Unigene1952_Se200S transcribed RNA sequence	A0A0J8BAT5|A0A0J8BAT5_BETVU:Uncharacterized protein;OS=Beta vulgaris subsp. vulgaris.;|GO:0097008,GO:0097007,GO:0020037,GO:0005506,|GO:0046246,
DN128440_c3_g6_i1	DOWN	[GO:0016021:integral component of membrane][GO:0005886:plasma membrane][GO:0016760:cellulose synthase (UDP-forming) activity][GO:0008270:zinc ion binding][GO:0071555:cell wall organization][GO:0030244:cellulose biosynthetic process]	-	FANhyb_rscf00001203.1.g00002.1:Fragaria x ananassa|LBD|LBD family protein	XP_010687624.1:PREDICTED: cellulose synthase A catalytic subunit 7 [UDPforming] [Beta vulgaris subsp. vulgaris] >KMT03462.1 hypothetical protein BVRB_8g191460 [Beta vulgaris subsp. vulgaris]	gi|521686575|gb|GAIA01024202.1|:TSA: Salicornia europaea Unigene25888_Se200S transcribed RNA sequence	A0A0J8BQH0|A0A0J8BQH0_BETVU: Cellulose synthase;OS=Beta vulgaris subsp. vulgaris.;|GO:0016021,GO:0005886,|GO:0016760,GO:0008270,|GO:0071555,GO:0030244,

geneid	trend	GO	Pathway	TF	NR	NT	Uniprot
DN123155_c1_g1_i3	DOWN	-	-	-	-	gi|8272397|dbj|AB021792.1|:Pyrus pyrifolia PPFRU21 mRNA for abscisic stress ripening protein, partial cds	-
DN67151_c0_g2_i1	DOWN	-	-	-	-	-	-

geneid	trend	GO	Pathway	TF	NR	NT	Uniprot
DN118006_c1_g1_i6	DOWN	[GO:0016021:integral component of membrane][GO:0005338:nucleotide-sugar transmembrane transporter activity][GO:0015780:nucleotide-sugar transmembrane transport]	-	Do008886.1:Dichanthelium oligosanthes|NAC|NAC family protein	XP_021846508.1:UDPrhamnose/UDPgalactose transporter 4like [Spinacia oleracea] >XP_021846509.1 UDPrhamnose/UDPgalactose transporter 4like [Spinacia oleracea] >XP_021846510.1 UDPrhamnose/UDPgalactose transporter 4like [Spinacia oleracea] >XP_021846511.1 UDPrhamnose/UDPgalactose transporter 4like [Spinacia oleracea] >KNA23413.1 hypothetical protein SOVF_025050 [Spinacia oleracea]	gi|359480731|ref|XM_002278777.2|:PREDICTED: Vitis vinifera UDP-galactose transporter 2-like (LOC100257791), mRNA	A0A0K9RVC5|A0A0K9RVC5_SPIOL: Uncharacterized protein;OS=Spinacia oleracea;|GO:0016021,|GO:0005338,|GO:0015780,

geneid	trend	GO	Pathway	TF	NR	NT	Uniprot
DN110505_c0_g1_i1	DOWN	[GO:0003978:UDP-glucose 4-epimerase activity][GO:0006012:galactose metabolic process]	[ko00052:Galactose metabolism][ko00520:Amino sugar and nucleotide sugar metabolism]	-	XP_010273140.1:PREDICTED: bifunctional UDPglucose 4epimerase and UDPxylose 4epimerase 1like isoform X2 [Nelumbo nucifera]	gi|521687960|gb|GAIA01023638.1|:TSA: Salicornia europaea Unigene25275_Se200S transcribed RNA sequence	A0A067JZP0|A0A067JZP0_JATCU:Uncharacterized protein;OS=Jatropha curcas;|GO:0003978,|GO:0006012,
DN114717_c0_g2_i1	DOWN	[GO:0016021:integral component of membrane][GO:0020037:heme binding][GO:0005506:iron ion binding][GO:0016705:oxidoreductase activity, acting on paired donors, with incorporation or reduction of molecular oxygen]	-	CCG000365.1:Populus euphratica|NAC|NAC family protein	XP_021845446.1:cytochrome P450 94C1 [Spinacia oleracea]	-	A0A061GZR4|A0A061GZR4_THECC: Cytochrome P450 94A1;OS=Theobroma cacao;|GO:0016021,|GO:0020037,GO:0005506,GO:0016705,
DN125811_c7_g7_i1	DOWN	-	-	-	-	-	-

geneid	trend	GO	Pathway	TF	NR	NT	Uniprot
DN112897_c0_g3_i1	DOWN	[GO:0005576:extracellular region][GO:0030145:manganese ion binding][GO:0045735:nutrient reservoir activity]	-	678365117:Utricularia gibba|bZIP|bZIP family protein	XP_021911189.1:germinlike protein subfamily 2 member 4 [Carica papaya]	-	A0A067H744|A0A067H744_CITSI:Uncharacterized protein;OS=Citrus sinensis;|GO:0005576,|GO:0030145,GO:0045735,
DN104971_c1_g2_i1	DOWN	[GO:0009733:response to auxin]	-	Rsa1.0_03425.1_g00001.1:Raphanus sativus|ZF-HD|ZF-HD family protein	XP_021756613.1:auxinresponsive protein SAUR50like [Chenopodium quinoa]	-	A0A0J8EK99|A0A0J8EK99_BETVU:Uncharacterized protein;OS=Beta vulgaris subsp. vulgaris.;|GO:0009733,
DN125811_c7_g3_i1	DOWN	-	-	-	OWM72007.1:hypothetical protein CDL15_Pgr017890 [Punica granatum]	-	A0A059CH61|A0A059CH61_EUCGR:Uncharacterized protein;OS=Eucalyptus grandis;

geneid	trend	GO	Pathway	TF	NR	NT	Uniprot
DN123703_c2_g1_i3	DOWN	[GO:0016021:integral component of membrane][GO:0005886:plasma membrane][GO:0015112:nitrate transmembrane transporter activity][GO:0006857:oligopeptide transport][GO:0010167:response to nitrate][GO:0009414:response to water deprivation]	-	mrna31023.1-v1.0-hybrid:Fragaria vesca|C3H|C3H family protein	XP_021768447.1:protein NRT1/ PTR FAMILY 6.3like [Chenopodium quinoa]	gi|357482000|ref|XM_003611238.1|:Medicago truncatula Nitrate transporter (MTR_5g012290) mRNA, complete cds	A0A0J8F9I8|A0A0J8F9I8_BETVU:Uncharacterized protein;OS=Beta vulgaris subsp. vulgaris.;|GO:0016021,GO:0005886,|GO:0015112,|GO:0006857,GO:0010167,GO:0009414,
DN127289_c0_g1_i2	DOWN	-	-	-	-	-	-
DN120768_c0_g2_i10	DOWN	[GO:0016021:integral component of membrane][GO:0008271:secondary active sulfate transmembrane transporter activity]	-	GSMUA_Achr9P13010_001:Musa acuminata|MIKC_MADS|MIKC_MADS family protein	XP_021723678.1:probable sulfate transporter 3.5 [Chenopodium quinoa]	-	A0A0K9RRK3|A0A0K9RRK3_SPIOL:Uncharacterized protein;OS=Spinacia oleracea;|GO:0016021,|GO:0008271,
DN122079_c4_g1_i1	DOWN	-	-	-	KNA21712.1:hypothetical protein SOVF_038540 [Spinacia oleracea]	gi|157273661|gb|EF672640.1|:Gossypium hirsutum fasciclin-like arabinogalactan	A0A0K9RSD7|A0A0K9RSD7_SPIOL:Uncharacterized protein;OS=Spinacia oleracea;

geneid	trend	GO	Pathway	TF	NR	NT	Uniprot
						protein 14 (FLA14) mRNA, complete cds	
DN114502_c1_g1_i1	DOWN	[GO:0016021:integral component of membrane]	-	676763678:Sisymbrium irio|GRAS|GRAS family protein	XP_010683745.1:PREDICTED: uncharacterized protein LOC104898376 [Beta vulgaris subsp. vulgaris] >KMT06804.1 hypothetical protein BVRB_7g159620 [Beta vulgaris subsp. vulgaris]	-	A0A0J8C3H0|A0A0J8C3H0_BETVU:Uncharacterized protein;OS=Beta vulgaris subsp. vulgaris.;|GO:0016021,
DN128759_c1_g12_i4	DOWN	[GO:0003824:catalytic activity][GO:0030170:pyridoxal phosphate binding][GO:0009058:biosynthetic process]	[ko00270:Cysteine and methionine metabolism][ko04016:MAPK signaling pathway - plant]	-	XP_021767144.1:1aminocyclopropane1carboxylate synthaselike [Chenopodium quinoa]	gi|379317179|gb|GU182503.3|:Narcissus tazetta var. chinensis cultivar Zhangzhou 1-aminocyclopropane-1-carboxylic acid synthase (ACS) mRNA, complete	A0A0J8D842|A0A0J8D842_BETVU:Uncharacterized protein;OS=Beta vulgaris subsp. vulgaris.;|GO:0003824,GO:0030170,|GO:0009058,

geneid	trend	GO	Pathway	TF	NR	NT	Uniprot
						cds	
DN129195_c59_g4_i4	DOWN	[GO:0016491:oxidoreductase activity]	[ko00902:Monoterpenoid biosynthesis]	Niben101Scf02581g01014.1:Nicotiana benthamiana|MYB_related|MYB_related family protein	CBI40299.3:unnamed protein product, partial [Vitis vinifera]	-	F6I6H7|F6I6H7_VITVI:Putative uncharacterized protein;OS=Vitis vinifera;GN=VIT_15s0046g03570 {ECO:0000313|EMBL:CCB62370.1};|GO:0016491,
DN95662_c0_g1_i1	DOWN	-	-	-	-	-	-
DN121767_c1_g1_i14	DOWN	-	-	-	XP_021749610.1:pollenspecific protein SF21like [Chenopodium quinoa]	-	A0A0J8D1H1|A0A0J8D1H1_BETVU:Uncharacterized protein;OS=Beta vulgaris subsp. vulgaris.;

geneid	trend	GO	Pathway	TF	NR	NT	Uniprot
DN119962_c0_g1_i5	DOWN	[GO:0016021:integral component of membrane][GO:0005886:plasma membrane][GO:0016760:cellulose synthase (UDP-forming) activity][GO:0046872:metal ion binding][GO:0071555:cell wall organization][GO:0030244:cellulose biosynthetic process][GO:0042742:defense response to bacterium][GO:0050832:defense response to fungus][GO:0009834:plant-type secondary cell wall biogenesis]	-	CCG027008.3:Populus euphratica|G2-like|G2-like family protein	XP_021722190.1:cellulose synthase A catalytic subunit 4 [UDPforming]like [Chenopodium quinoa]	gi|356530214|ref|XM_003533631.1|:PREDICTED: Glycine max cellulose synthase A catalytic subunit 4 [UDP-forming]-like (LOC100781996), mRNA	A0A0J8B7A9|A0A0J8B7A9_BETVU:Cellulose synthase;OS=Beta vulgaris subsp. vulgaris.;|GO:0016021,GO:0005886,|GO:0016760,GO:0046872,|GO:0071555,GO:0030244,GO:0042742,GO:0050832,GO:0009834,
DN128505_c2_g3_i4	DOWN	[GO:0005576:extracellular region][GO:0030145:manganese ion binding][GO:0045735:nutrient reservoir activity]	-	678365117:Utricularia gibba|bZIP|bZIP family protein	XP_021714510.1:germinlike protein subfamily 2 member 4 [Chenopodium quinoa]	-	A0A0K9R898|A0A0K9R898_SPIOL:Uncharacterized protein;OS=Spinacia oleracea;|GO:0005576,|GO:0030145,GO:0045735,

geneid	trend	GO	Pathway	TF	NR	NT	Uniprot
DN119314_c1_g1_i2	DOWN	[GO:0016021:integral component of membrane][GO:0004553:hydrolase activity, hydrolyzing O-glycosyl compounds][GO:0005975:carbohydrate metabolic process]	-	-	XP_010685866.1:PREDICTED: chitinase 2 [Beta vulgaris subsp. vulgaris] >KMT05350.1 hypothetical protein BVRB_7g174820 [Beta vulgaris subsp. vulgaris]	gi|449532530|ref|XM_004173186.1|:PREDICTED: Cucumis sativus chitinase 2-like (LOC101227481), partial mRNA	A0A0J8BUV0|A0A0J8BUV0_BETVU: Uncharacterized protein;OS=Beta vulgaris subsp. vulgaris.;|GO:0016021,|GO:0004553,|GO:0005975,
DN120860_c6_g15_i1	DOWN	-	-	-	-	-	-

geneid	trend	GO	Pathway	TF	NR	NT	Uniprot
DN115324_c0_g1_i1	DOWN	[GO:0016021:integral component of membrane][GO:0005524:ATP binding][GO:0004672:protein kinase activity]	[ko04064:NF-kappa B signaling pathway][ko04620:Toll-like receptor signaling pathway][ko04621:NOD-like receptor signaling pathway][ko04624:Toll and Imd signaling pathway][ko04722:Neurotrophin signaling pathway][ko05133:Pertussis][ko05140:Leishmaniasis][ko05142:Chagas disease (American trypanosomiasis)][ko05145:Toxoplasmosis][ko05152:Tuberculosis][ko05162:Measles][ko05164:Influenza A]	mrna09125.1-v1.0-hybrid:Fragaria vesca|bHLH|bHLH family protein	XP_010676257.1:PREDICTED: probable receptorlike protein kinase At1g11050 [Beta vulgaris subsp. vulgaris] >KMT12591.1 hypothetical protein BVRB_5g098310 [Beta vulgaris subsp. vulgaris]	-	A0A0J8F9P6|A0A0J8F9P6_BETVU:Uncharacterized protein;OS=Beta vulgaris subsp. vulgaris.;|GO:0016021,|GO:0005524,GO:0004672,

geneid	trend	GO	Pathway	TF	NR	NT	Uniprot
DN127735_c0_g2_i5	DOWN	[GO:0008270:zinc ion binding]	-	MDP0000266172:Malus domestica|Nin-like|Nin-like family protein	XP_021714578.1:pentatricopeptide repeatcontaining protein At5g44230like [Chenopodium quinoa] >XP_021714579.1 pentatricopeptide repeatcontaining protein At5g44230like [Chenopodium quinoa] >XP_021714580.1 pentatricopeptide repeatcontaining protein At5g44230like [Chenopodium quinoa] >XP_021714581.1 pentatricopeptide repeatcontaining protein	gi|224133789|ref|XM_002327645.1|:Populus trichocarpa predicted protein, mRNA	F6GWS8|F6GWS8_VITVI:Putative uncharacterized protein;OS=Vitis vinifera;GN=VIT_04s0023g02490 {ECO:0000313|EMBL:CCB44413.1};|GO:0008270,

geneid	trend	GO	Pathway	TF	NR	NT	Uniprot
					At5g44230like [Chenopodium quinoa]		
DN122258_c3_g5_i3	DOWN	[GO:0008061:chitin binding][GO:0004568:chitinase activity][GO:0005975:carbohydrate metabolic process][GO:0016998:cell wall macromolecule catabolic process][GO:0006032:chitin catabolic process]	[ko00520:Amino sugar and nucleotide sugar metabolism]	mrna20854.1-v1.0-hybrid:Fragaria vesca|B3|B3 family protein	AHA62771.1:chitinase [Mirabilis jalapa]	gi|3176964|gb|AF067967.1|AF067967:Mesembryanthemum crystallinum pyrroline-5-carboxylate synthetase (P5CS) mRNA, complete cds	V5M258|V5M258_MIRJA:Chitinase;OS=Mirabilis jalapa;|GO:0008061,GO:0004568,|GO:0005975,GO:0016998,GO:0006032,

geneid	trend	GO	Pathway	TF	NR	NT	Uniprot
DN69980_c0_g1_i1	DOWN	-	[ko03010:Ribosome]	-	O65353.1:RecName: Full=60S ribosomal protein L5 >AAC17448.1 RPL5Arelated protein [Helianthus annuus]	gi|214012054|gb|FJ263908.1|:Artemia franciscana clone 19MB8.08 ribosomal protein L5 mRNA, partial cds	A0A1D1XTV4|A0A1D1XTV4_9ARAE: 60S ribosomal protein L5;OS=Anthurium amnicola.;
DN126301_c1_g1_i1	DOWN	[GO:0005794:Golgi apparatus][GO:0016021:integral component of membrane][GO:0016413:O-acetyltransferase activity][GO:0010411:xyloglucan metabolic process]	-	Csa09g092790.1:Camelina sativa|BES1|BES1 family protein	XP_010682616.1:PREDICTED: protein ALTERED XYLOGLUCAN 4 [Beta vulgaris subsp. vulgaris] >KMT07323.1 hypothetical protein BVRB_6g149510 [Beta vulgaris subsp. vulgaris]	gi|502093254|ref|XM_004489829.1|:PREDICTED: Cicer arietinum uncharacterized LOC101490987 (LOC101490987), mRNA	A0A0J8C5I7|A0A0J8C5I7_BETVU:Uncharacterized protein;OS=Beta vulgaris subsp. vulgaris.;|GO:0005794,GO:0016021,|GO:0016413,|GO:0010411,

geneid	trend	GO	Pathway	TF	NR	NT	Uniprot
DN120683_c2_g1_i1	DOWN	[GO:0016324:apical plasma membrane][GO:0010215:cellulose microfibril organization][GO:0051650:establishment of vesicle localization][GO:0009860:pollen tube growth][GO:0030833:regulation of actin filament polymerization][GO:0017157:regulation of exocytosis]	-	-	XP_010673928.1:PREDICTED: CRIB domaincontaining protein RIC4 isoform X2 [Beta vulgaris subsp. vulgaris]	gi|521737812|gb|GAIA01007895.1|:TSA: Salicornia europaea Unigene8219_Se200S transcribed RNA sequence	A0A0J8CLV8|A0A0J8CLV8_BETVU:Uncharacterized protein;OS=Beta vulgaris subsp. vulgaris.;|GO:0016324,|GO:0010215,GO:0051650,GO:0009860,GO:0030833,GO:0017157,
DN128041_c1_g1_i2	DOWN	-	-	-	-	-	-
DN101166_c0_g2_i1	DOWN	-	-	-	-	-	-
DN49759_c0_g1_i1	DOWN	-	[ko03010:Ribosome]	-	KJB47513.1:hypothetical protein B456_008G029900 [Gossypium raimondii]	-	A0A1D1XNL9|A0A1D1XNL9_9ARAE:40S ribosomal protein S25;OS=Anthurium amnicola.;
DN117458_c1_g3_i2	DOWN	-	-	-	-	-	-

geneid	trend	GO	Pathway	TF	NR	NT	Uniprot
DN109651_c0_g1_i1	DOWN	-	-	DCAR_008117:Daucus carota|bHLH|bHLH family protein	XP_010684085.1:PREDICTED: cysteinerich repeat secretory protein 38 [Beta vulgaris subsp. vulgaris] >KMT06271.1 hypothetical protein BVRB_7g161960 [Beta vulgaris subsp. vulgaris]	gi|312283492|dbj|AK353526.1|:Thellungiella halophila mRNA, complete cds, clone: RTFL01-49-C01	A0A0J8ESV7|A0A0J8ESV7_BETVU:Uncharacterized protein;OS=Beta vulgaris subsp. vulgaris.;
DN116401_c4_g14_i4	DOWN	-	-	-	XP_010666717.1:PREDICTED: protein TIFY 5A [Beta vulgaris subsp. vulgaris] >KMS95890.1 hypothetical protein BVRB_004000 [Beta vulgaris subsp. vulgaris]	-	A0A0J8DYD7|A0A0J8DYD7_BETVU:Uncharacterized protein;OS=Beta vulgaris subsp. vulgaris.;

geneid	trend	GO	Pathway	TF	NR	NT	Uniprot
DN116412_c0_g1_i3	DOWN	[GO:0005618:cell wall][GO:0016021:integral component of membrane][GO:0005886:plasma membrane][GO:0016760:cellulose synthase (UDP-forming) activity][GO:0046872:metal ion binding][GO:0071555:cell wall organization][GO:0030244:cellulose biosynthetic process][GO:0042742:defense response to bacterium][GO:0050832:defense response to fungus][GO:0009834:plant-type secondary cell wall biogenesis][GO:0006970:response to osmotic stress][GO:0009414:response to water deprivation]	-	FANhyb_rscf00001203.1.g00002.1:Fragaria x ananassa|LBD|LBD family protein	XP_021852858.1:cellulose synthase A catalytic subunit 8 [UDPforming] [Spinacia oleracea]	gi|502078354|ref|XM_004485861.1|:PREDICTED: Cicer arietinum cellulose synthase A catalytic subunit 8 [UDP-forming]-like (LOC101510081), transcript variant X2, mRNA	A0A0J8BWP9|A0A0J8BWP9_BETVU: Cellulose synthase;OS=Beta vulgaris subsp. vulgaris.;|GO:0005618,GO:0016021,GO:0005886,|GO:0016760,GO:0046872,|GO:0071555,GO:0030244,GO:0042742,GO:0050832,GO:0009834,GO:0006970,GO:0009414,
DN126513_c0_g1_i5	DOWN	-	-	-	-	-	-
DN127790_c	DOWN	-	-	-	-	-	-

geneid	trend	GO	Pathway	TF	NR	NT	Uniprot
1_g8_i1							
DN128356_c4_g6_i1	DOWN	-	-	-	-	-	-
DN112553_c0_g1_i1	DOWN	[GO:0005634:nucleus][GO:0003677:DNA binding][GO:0003700:DNA-binding transcription factor activity][GO:0006351:transcription, DNA-templated]	-	Bv5_121050_fnik.t1:Beta vulgaris|ERF|ERF family protein	XP_010679244.1:PREDICTED: ethyleneresponsive transcription factor ERF098 [Beta vulgaris subsp. vulgaris] >KMT10356.1 hypothetical protein BVRB_5g121050 [Beta vulgaris subsp. vulgaris]	gi|224085624|ref|XM_002307604.1|:Populus trichocarpa AP2/ERF domain-containing transcription factor (ERF18), mRNA	A0A0J8F3M4|A0A0J8F3M4_BETVU:Uncharacterized protein;OS=Beta vulgaris subsp. vulgaris.;|GO:0005634,|GO:0003677,GO:0003700,|GO:0006351,
DN121969_c2_g3_i9	DOWN	[GO:0046872:metal ion binding][GO:0016491:oxidoreductase activity]	-	-	XP_021737922.1:protein SRG1like [Chenopodium quinoa]	-	A0A0K9R3K3|A0A0K9R3K3_SPIOL:Uncharacterized protein;OS=Spinacia oleracea;|GO:0046872,GO:0016491,

geneid	trend	GO	Pathway	TF	NR	NT	Uniprot
DN115537_c2_g1_i1	DOWN	[GO:0050660:flavin adenine dinucleotide binding][GO:0016614:oxidoreductase activity, acting on CH-OH group of donors]	-	FANhyb_rscf00000362.1.g00002.1:Fragaria x ananassa|CPP|CPP family protein	XP_021725597.1:berberine bridge enzymelike 15 [Chenopodium quinoa]	gi|470115000|ref|XM_004293644.1|:PREDICTED: Fragaria vesca subsp. vesca cannabidiolic acid synthase-like 1-like (LOC101294538), mRNA	A0A0K9QR43|A0A0K9QR43_SPIOL:Uncharacterized protein;OS=Spinacia oleracea;|GO:0050660,GO:0016614,
DN112235_c1_g1_i8	DOWN	-	-	-	XP_021852628.1:uncharacterized protein LOC110792127 [Spinacia oleracea] >KNA07731.1 hypothetical protein SOVF_168410 [Spinacia oleracea]	-	A0A0K9QMD5|A0A0K9QMD5_SPIOL:Uncharacterized protein;OS=Spinacia oleracea;

geneid	trend	GO	Pathway	TF	NR	NT	Uniprot
DN125332_c2_g1_i6	DOWN	[GO:0005576:extracellular region][GO:0020037:heme binding][GO:0046872:metal ion binding][GO:0004601:peroxidase activity][GO:0042744:hydrogen peroxide catabolic process][GO:0006979:response to oxidative stress]	[ko00940:Phenylpropanoid biosynthesis]	Sp_046550_watp.t1: Spinacia oleracea|MYB|MYB family protein	XP_021838020.1:peroxidase 60like [Spinacia oleracea] >CAA71489.1 peroxidase [Spinacia oleracea]	-	A0A0K9RSI5|A0A0K9RSI5_SPIOL:Peroxidase;OS=Spinacia oleracea;|GO:0005576,|GO:0020037,GO:0046872,GO:0004601,|GO:0042744,GO:0006979,
DN127018_c1_g2_i2	DOWN	[GO:0004657:proline dehydrogenase activity][GO:0006562:proline catabolic process]	[ko00330:Arginine and proline metabolism]	-	XP_010691926.1:PREDICTED: proline dehydrogenase 2, mitochondriallike [Beta vulgaris subsp. vulgaris]	gi|521502071|gb|GAHZ01028849.1|:TSA: Salicornia europaea Unigene31774_SeCKS transcribed RNA sequence	A0A0K9QPJ1|A0A0K9QPJ1_SPIOL:Uncharacterized protein;OS=Spinacia oleracea;|GO:0004657,|GO:0006562,
DN115164_c0_g1_i1	DOWN	[GO:0048046:apoplast][GO:0005507:copper ion binding][GO:0052716:hydroquinone:oxygen oxidoreductase activity][GO:0046274:lignin catabolic process]	-	Rsa1.0_03034.1_g00001.1:Raphanus sativus|ERF|ERF family protein	XP_021732412.1:laccase4like [Chenopodium quinoa]	gi|521489127|gb|GAHZ01041759.1|:TSA: Salicornia europaea Unigene48054_SeCKS transcribed RNA sequence	A0A0J8CXD3|A0A0J8CXD3_BETVU: Laccase;OS=Beta vulgaris subsp. vulgaris.;|GO:0048046,|GO:0005507,GO:0052716,|GO:0046274,

geneid	trend	GO	Pathway	TF	NR	NT	Uniprot
DN127647_c0_g1_i6	DOWN	[GO:0016021:integral component of membrane]	-	-	XP_021866951.1:uncharacterized protein LOC110805639 [Spinacia oleracea] >KNA24470.1 hypothetical protein SOVF_015340 [Spinacia oleracea]	-	A0A0K9RY57|A0A0K9RY57_SPIOL:Uncharacterized protein;OS=Spinacia oleracea;|GO:0016021,
DN111153_c0_g2_i1	DOWN	-	-	Sp_127320_zaeh.t1:Spinacia oleracea|ZF-HD|ZF-HD family protein	XP_021859560.1:mini zinc finger protein 1like [Spinacia oleracea] >KNA12285.1 hypothetical protein SOVF_127320 [Spinacia oleracea]	-	A0A0K9QYF5|A0A0K9QYF5_SPIOL: Uncharacterized protein;OS=Spinacia oleracea;
DN114360_c0_g1_i2	DOWN	[GO:0003824:catalytic activity]	-	XP_015865732.1:Ziziphus jujuba|FAR1|FAR1 family protein	XP_021714525.1:epoxide hydrolase 4like [Chenopodium quinoa]	gi|484678603|gb|GAHV01000030.1|:TSA: Nelumbo nucifera Seq31 transcribed RNA sequence	A0A0J8C757|A0A0J8C757_BETVU:Uncharacterized protein;OS=Beta vulgaris subsp. vulgaris.;|GO:0003824,

geneid	trend	GO	Pathway	TF	NR	NT	Uniprot
DN128453_c2_g2_i5	DOWN	-	-	-	KNA13188.1:hypothetical protein SOVF_119040 isoform A [Spinacia oleracea]	-	A0A0K9R104|A0A0K9R104_SPIOL:Uncharacterized protein;OS=Spinacia oleracea;
DN127772_c2_g8_i3	DOWN	[GO:0032040:small-subunit processome]	-	Achn356291:Actinidia chinensis|MIKC_MADS|MIKC_MADS family protein	XP_010667324.1:PREDICTED: uncharacterized protein LOC104884377 [Beta vulgaris subsp. vulgaris]	gi|521670917|gb|GAIA01032066.1|:TSA: Salicornia europaea Unigene35193_Se200S transcribed RNA sequence	A0A0J8C595|A0A0J8C595_BETVU:Uncharacterized protein;OS=Beta vulgaris subsp. vulgaris.;|GO:0032040,
DN109527_c1_g3_i1	DOWN	-	-	-	-	-	-
DN123750_c0_g1_i1	DOWN	[GO:0005634:nucleus][GO:0043565:sequence-specific DNA binding][GO:0003700:DNA-binding transcription factor activity]	-	Sp_003720_make.t1:Spinacia oleracea|HSF|HSF family protein	XP_021738979.1:heat shock factor protein HSF30like isoform X1 [Chenopodium quinoa] >XP_021738980.1 heat shock factor protein HSF30like isoform X1	gi|470109539|ref|XM_004291004.1|:PREDICTED: Fragaria vesca subsp. vesca heat shock factor protein HSF30-like, transcript variant 2 (LOC101312795), mRNA	A0A0K9S258|A0A0K9S258_SPIOL:Uncharacterized protein;OS=Spinacia oleracea;|GO:0005634,|GO:0043565,GO:0003700,

geneid	trend	GO	Pathway	TF	NR	NT	Uniprot
					[Chenopodium quinoa]		

geneid	trend	GO	Pathway	TF	NR	NT	Uniprot
DN92669_c0_g1_i1	DOWN	-	[ko04141:Protein processing in endoplasmic reticulum][ko04151:PI3K-Akt signaling pathway][ko04217:Necroptosis][ko04612:Antigen processing and presentation][ko04621:NOD-like receptor signaling pathway][ko04626:Plant-pathogen interaction][ko04657:IL-17 signaling pathway][ko04659:Th17 cell differentiation][ko04914:Progesterone-mediated oocyte maturation][ko04915:Estrogen signaling pathway][ko05200:Pathways in	Do001279.1:Dichanthelium oligosanthes|bZIP|bZIP family protein	AGU99972.1:HSP90 [Populus tomentosa]	gi|354550151|gb|JN627862.1|:Stratiomys singularior heat shock protein 83S2 (hsp83S2) gene, complete cds	A0A1D1Y7I3|A0A1D1Y7I3_9ARAE:Heat shock protein 83;OS=Anthurium amnicola.;

geneid	trend	GO	Pathway	TF	NR	NT	Uniprot
			cancer][ko05215:Prostate cancer][ko05418:Fluid shear stress and atherosclerosis]				

geneid	trend	GO	Pathway	TF	NR	NT	Uniprot
DN118872_c1_g1_i1	DOWN	[GO:0009733:response to auxin]	[ko04075:Plant hormone signal transduction]	-	XP_021755011.1:auxinresponsive protein SAUR71like [Chenopodium quinoa]	-	A0A0J8BEY9|A0A0J8BEY9_BETVU:Uncharacterized protein;OS=Beta vulgaris subsp. vulgaris.;|GO:0009733,
DN125061_c1_g1_i8	DOWN	[GO:0008270:zinc ion binding]	-	-	XP_010684185.1:PREDICTED: uncharacterized protein LOC104898779 isoform X1 [Beta vulgaris subsp. vulgaris] >XP_019106354.1 PREDICTED: uncharacterized protein LOC104898779 isoform X1 [Beta vulgaris subsp. vulgaris] >KMT06192.1 hypothetical protein	-	A0A0J8BY84|A0A0J8BY84_BETVU:Uncharacterized protein;OS=Beta vulgaris subsp. vulgaris.;|GO:0008270,

geneid	trend	GO	Pathway	TF	NR	NT	Uniprot
					BVRB_7g162630 isoform B [Beta vulgaris subsp. vulgaris]		

geneid	trend	GO	Pathway	TF	NR	NT	Uniprot
DN109738_c0_g1_i1	DOWN	[GO:0016021:integral component of membrane][GO:0005743:mitochondrial inner membrane][GO:0005886:plasma membrane][GO:0045277:respiratory chain complex IV][GO:0005507:copper ion binding][GO:0004129:cytochrome-c oxidase activity][GO:0020037:heme binding][GO:0005506:iron ion binding][GO:0009060:aerobic respiration][GO:0022900:electron transport chain][GO:0006119:oxidative phosphorylation]	[ko00190:Oxidative phosphorylation][ko04260:Cardiac muscle contraction][ko04932:Non-alcoholic fatty liver disease (NAFLD)][ko05010:Alzheimer's disease][ko05012:Parkinson's disease][ko05016:Huntington's disease]	-	AKJ25554.1:cytochrome c oxidase subunit 1 (mitochondrion) [Geranium endressii] >AKJ25558.1 cytochrome c oxidase subunit 1 (mitochondrion) [Geranium nodosum] >AKJ25559.1 cytochrome c oxidase subunit 1 (mitochondrion) [Geranium platyanthum] >AKJ25560.1 cytochrome c oxidase subunit 1 (mitochondrion) [Geranium phaeum] >AKJ25561.1 cytochrome c oxidase subunit 1 (mitochondrion) [Geranium pratense] >AKJ25562.1 cytochrome c oxidase subunit 1 (mitochondrion)	gi|183013995|emb|AM404181.1|:Antedon mediterranea complete mitochondial genome	A0A0E3DAW8|A0A0E3DAW8_9FLOR:Cytochrome c oxidase subunit 1;OS=Asparagopsis taxiformis.;|GO:0016021,GO:0005743,GO:0005886,GO:0045277,|GO:0005507,GO:0004129,GO:0020037,GO:0005506,|GO:0009060,GO:0022900,GO:0006119,

geneid	trend	GO	Pathway	TF	NR	NT	Uniprot
DN122397_c5_g2_i6	DOWN	-	-	-	-	-	-
DN108906_c0_g1_i2	DOWN	-	-	-	-	-	-
DN117439_c2_g3_i1	DOWN	-	-	-	-	-	-
DN121192_c0_g3_i2	DOWN	-	-	Bv7_168260_jzca.t1:Beta vulgaris|bHLH|bHLH family protein	XP_021720145.1:transcription factor bHLH62like [Chenopodium quinoa]	gi|460403116|ref|XM_004246999.1|:PREDICTED: Solanum lycopersicum transcription factor bHLH62-like (LOC101265273), mRNA	A0A0J8C085|A0A0J8C085_BETVU:Uncharacterized protein;OS=Beta vulgaris subsp. vulgaris.;
DN121660_c4_g6_i1	DOWN	-	-	-	-	-	-
DN109276_c0_g1_i1	DOWN	-	-	-	-	gi|307141007|gb|GU932669.1|:Haemaphysalis longicornis paramyosin mRNA, partial cds	A0A1D1XRP2|A0A1D1XRP2_9ARAE: Paramyosin, long form;OS=Anthurium amnicola.;

geneid	trend	GO	Pathway	TF	NR	NT	Uniprot
DN127944_c10_g2_i2	DOWN	[GO:0046872:metal ion binding][GO:0016491:oxidoreductase activity]	-	676709864:Sisymbrium irio|STAT|STAT family protein	XP_010679238.1:PREDICTED: 2'deoxymugineicacid 2'dioxygenase [Beta vulgaris subsp. vulgaris] >XP_010679239.1 PREDICTED: 2'deoxymugineicacid 2'dioxygenase [Beta vulgaris subsp. vulgaris] >XP_010679240.1 PREDICTED: 2'deoxymugineicacid 2'dioxygenase [Beta vulgaris subsp. vulgaris] >KMT10352.1 hypothetical protein BVRB_5g121020 [Beta vulgaris subsp. vulgaris]	-	A0A0J8C9U3|A0A0J8C9U3_BETVU:Uncharacterized protein;OS=Beta vulgaris subsp. vulgaris.;|GO:0046872,GO:0016491,

geneid	trend	GO	Pathway	TF	NR	NT	Uniprot
DN110405_c0_g3_i2	DOWN	[GO:0016021:integral component of membrane][GO:0005886:plasma membrane][GO:0016760:cellulose synthase (UDP-forming) activity][GO:0046872:metal ion binding][GO:0071555:cell wall organization][GO:0030244:cellulose biosynthetic process][GO:0042742:defense response to bacterium][GO:0050832:defense response to fungus][GO:0009834:plant-type secondary cell wall biogenesis]	-	CCG027008.3:Populus euphratica|G2-like|G2-like family protein	XP_021722190.1:cellulose synthase A catalytic subunit 4 [UDPforming]like [Chenopodium quinoa]	gi|521747907|gb|GAIA01001907.1|:TSA: Salicornia europaea Unigene1945_Se200S transcribed RNA sequence	A0A0J8B7A9|A0A0J8B7A9_BETVU:Cellulose synthase;OS=Beta vulgaris subsp. vulgaris.;|GO:0016021,GO:0005886,|GO:0016760,GO:0046872,|GO:0071555,GO:0030244,GO:0042742,GO:0050832,GO:0009834,
DN128786_c1_g1_i9	DOWN	-	-	-	XP_021759112.1:uncharacterized protein LOC110724013 [Chenopodium quinoa]	-	A0A059D5P9|A0A059D5P9_EUCGR:Uncharacterized protein;OS=Eucalyptus grandis;

geneid	trend	GO	Pathway	TF	NR	NT	Uniprot
DN118191_c0_g1_i6	DOWN	[GO:0005794:Golgi apparatus][GO:0016021:integral component of membrane][GO:0015020:glucuronosyltransferase activity][GO:0009834:plant-type secondary cell wall biogenesis][GO:0045492:xylan biosynthetic process]	-	-	XP_021728089.1:UDPglucuronate:xylan alphaglucuronosyltransferase 1like [Chenopodium quinoa]	gi|521655962|gb|GAIA01039188.1|:TSA: Salicornia europaea Unigene43854_Se200S transcribed RNA sequence	A0A0J8F0X4|A0A0J8F0X4_BETVU:Hexosyltransferase;OS=Beta vulgaris subsp. vulgaris.;|GO:0005794,GO:0016021,|GO:0015020,|GO:0009834,GO:0045492,
DN100430_c0_g1_i1	DOWN	-	[ko00330:Arginine and proline metabolism]	-	ACN31538.1:unknown [Zea mays]	gi|317039993|gb|GU382867.1|:Hippa adactyla voucher NMNS 4368-027 arginine kinase (AK) gene, partial cds	A0A1D1XCV8|A0A1D1XCV8_9ARAE: Arginine kinase;OS=Anthurium amnicola.;

geneid	trend	GO	Pathway	TF	NR	NT	Uniprot
DN119427_c0_g1_i7	DOWN	[GO:0016021:integral component of membrane][GO:0055085:transmembrane transport]	-	Jcr4S00206.70:Jatropha curcas|FAR1|FAR1 family protein	XP_010680628.1:PREDICTED: protein PINLIKES 7 isoform X1 [Beta vulgaris subsp. vulgaris] >XP_010680629.1 PREDICTED: protein PINLIKES 7 isoform X1 [Beta vulgaris subsp. vulgaris] >XP_019105728.1 PREDICTED: protein PINLIKES 7 isoform X1 [Beta vulgaris subsp. vulgaris] >KMT08875.1 hypothetical protein BVRB_6g135980 [Beta vulgaris subsp. vulgaris]	gi|521703437|gb|GAIA01016206.1|:TSA: Salicornia europaea Unigene16917_Se200S transcribed RNA sequence	A0A0J8C515|A0A0J8C515_BETVU:Uncharacterized protein;OS=Beta vulgaris subsp. vulgaris.;|GO:0016021,|GO:0055085,

geneid	trend	GO	Pathway	TF	NR	NT	Uniprot
DN128539_c1_g1_i1	DOWN	[GO:0016491:oxidoreductase activity]	-	-	XP_021731293.1:aldose reductaselike [Chenopodium quinoa]	-	A0A199UW30|A0A199UW30_ANACO:Aldose reductase;OS=Ananas comosus;|GO:0016491,
DN122258_c3_g5_i2	DOWN	[GO:0008061:chitin binding][GO:0004568:chitinase activity][GO:0005975:carbohydrate metabolic process][GO:0016998:cell wall macromolecule catabolic process][GO:0006032:chitin catabolic process]	[ko00520:Amino sugar and nucleotide sugar metabolism]	mrna20854.1-v1.0-hybrid:Fragaria vesca|B3|B3 family protein	AHA62771.1:chitinase [Mirabilis jalapa]	gi|357454534|ref|XM_003597500.1|:Medicago truncatula Endochitinase PR4 (MTR_2g099470) mRNA, complete cds	V5M258|V5M258_MIRJA:Chitinase;OS=Mirabilis jalapa;|GO:0008061,GO:0004568,|GO:0005975,GO:0016998,GO:0006032,
DN112333_c0_g2_i1	DOWN	-	-	-	-	-	-

geneid	trend	GO	Pathway	TF	NR	NT	Uniprot
DN120736_c1_g1_i5	DOWN	-	-	GSVIVT01036959001:Vitis vinifera|bHLH|bHLH family protein	XP_002268443.3:PREDICTED: transcription factor bHLH18 [Vitis vinifera] >XP_010647980.1 PREDICTED: transcription factor bHLH18 [Vitis vinifera] >XP_019074348.1 PREDICTED: transcription factor bHLH18 [Vitis vinifera] >CBI25069.3 unnamed protein product, partial [Vitis vinifera]	-	A5BUH3|A5BUH3_VITVI:Putative uncharacterized protein;OS=Vitis vinifera;
DN117579_c3_g2_i1	DOWN	-	-	-	-	-	-

geneid	trend	GO	Pathway	TF	NR	NT	Uniprot
DN123125_c2_g1_i1	DOWN	[GO:0045492:xylan biosynthetic process]	-	Itr_sc000772.1_g00020.1:Ipomoea trifida|FAR1|FAR1 family protein	XP_010676715.1:PREDICTED: glucuronoxylan 4Omethyltransferase 1like [Beta vulgaris subsp. vulgaris]	gi|521563561|gb|GAIA01074886.1|:TSA: Salicornia europaea Unigene90838_Se200S transcribed RNA sequence	F6HK27|F6HK27_VITVI:Putative uncharacterized protein;OS=Vitis vinifera;GN=VIT_12s0035g01340 {ECO:0000313|EMBL:CCB55030.1};|GO:0045492,
DN107498_c0_g1_i2	DOWN	[GO:0003677:DNA binding]	-	Ciclev10009336m:Citrus clementina|MYB|MYB family protein	XP_021725068.1:transcription factor MYB36like [Chenopodium quinoa]	gi|224142835|ref|XM_002324711.1|:Populus trichocarpa predicted protein (MYB085), mRNA	A0A067ELE3|A0A067ELE3_CITSI:Uncharacterized protein;OS=Citrus sinensis;|GO:0003677,
DN121469_c3_g1_i1	DOWN	-	-	-	-	-	-
DN122713_c2_g1_i1	DOWN	-	-	-	-	-	-
DN114362_c0_g1_i3	DOWN	[GO:0006952:defense response][GO:0009607:response to biotic stimulus]	-	-	XP_021741515.1:MLPlike protein 43 [Chenopodium quinoa]	-	A0A0K9R979|A0A0K9R979_SPIOL:Uncharacterized protein;OS=Spinacia oleracea;|GO:00069

geneid	trend	GO	Pathway	TF	NR	NT	Uniprot
							52,GO:0009607,
DN79076_c0_g1_i1	DOWN	[GO:0005788:endoplasmic reticulum lumen][GO:0003756:protein disulfide isomerase activity][GO:0045454:cell redox homeostasis]	[ko04141:Protein processing in endoplasmic reticulum][ko04612:Antigen processing and presentation]	GRMZM2G008647_P02:Zea mays|MYB_related|MYB_related family protein	AAA32662.1:putative endomembrane protein precursor [Medicago sativa]	-	P29828|PDI_MEDSA:Protein disulfide-isomerase;OS=Medicago sativa;|GO:0005788,|GO:0003756,|GO:0045454,
DN128938_c2_g10_i1	DOWN	-	-	-	-	-	A0A0V0H8I6|A0A0V0H8I6_SOLCH:Putative ovule protein;OS=Solanum chacoense;
DN128786_c1_g1_i12	DOWN	-	-	-	XP_021750546.1:uncharacterized protein LOC110716241 [Chenopodium quinoa]	-	D7THD8|D7THD8_VITVI:Putative uncharacterized protein;OS=Vitis vinifera;GN=VIT_08s0007g01560{ECO:0000313|EMBL:CBI30454.3};

geneid	trend	GO	Pathway	TF	NR	NT	Uniprot
DN119739_c1_g6_i5	DOWN	[GO:0016021:integral component of membrane][GO:0070469:respirasome][GO:0009916:alternative oxidase activity][GO:0046872:metal ion binding]	-	-	XP_010677502.1:PREDICTED: ubiquinol oxidase 2, mitochondriallike [Beta vulgaris subsp. vulgaris]	gi|242384781|emb|FP093028.1|:Phyllostachys edulis cDNA clone: bphyem122a05, full insert sequence	A0A0J8CD04|A0A0J8CD04_BETVU:Ubiquinol oxidase;OS=Beta vulgaris subsp. vulgaris.;|GO:0016021,GO:0070469,|GO:0009916,GO:0046872,
DN116837_c2_g1_i1	DOWN	[GO:0016021:integral component of membrane][GO:0005215:transporter activity]	-	-	AAD31847.1:water channel protein MipI [Mesembryanthemum crystallinum]	gi|4884865|gb|AF133531.1|AF133531:Mesembryanthemum crystallinum water channel protein MipI (MipI) mRNA, complete cds	Q9XF75|Q9XF75_MESCR:Water channel protein MipI;OS=Mesembryanthemum crystallinum;|GO:0016021,|GO:0005215,
DN119876_c9_g16_i1	DOWN	-	-	-	-	-	-
DN121969_c2_g3_i2	DOWN	[GO:0046872:metal ion binding][GO:0016491:oxidoreductase activity]	-	-	XP_021737922.1:protein SRG1like [Chenopodium quinoa]	-	A0A0K9R3K3|A0A0K9R3K3_SPIOL:Uncharacterized protein;OS=Spinacia oleracea;|GO:0046872,GO:0016491,

geneid	trend	GO	Pathway	TF	NR	NT	Uniprot
DN126877_c6_g2_i6	DOWN	-	-	-	KMT15128.1:hypothetical protein BVRB_3g062460 [Beta vulgaris subsp. vulgaris]	-	A0A0J8CT63|A0A0J8CT63_BETVU:Uncharacterized protein;OS=Beta vulgaris subsp. vulgaris.;
DN110506_c0_g1_i2	DOWN	[GO:0003677:DNA binding]	-	AHYPO_011563-RA:Amaranthus hypochondriacus|MYB|MYB family protein	XP_021760419.1:transcription factor MYB63like [Chenopodium quinoa]	-	A0A0J8D727|A0A0J8D727_BETVU:Uncharacterized protein;OS=Beta vulgaris subsp. vulgaris.;|GO:0003677,
DN122974_c7_g14_i1	DOWN	[GO:0045735:nutrient reservoir activity]	-	-	XP_018835514.1:PREDICTED: glutelin typeA 3like [Juglans regia] >XP_018835515.1 PREDICTED: glutelin typeA 3like [Juglans regia] >XP_018835516.1 PREDICTED: glutelin typeA 3like [Juglans regia]	-	M5XQ02|M5XQ02_PRUPE:Uncharacterized protein;OS=Prunus persica;|GO:0045735,

geneid	trend	GO	Pathway	TF	NR	NT	Uniprot
DN125813_c5_g1_i3	DOWN	-	-	-	XP_015899916.1:PREDICTED: pirinlike protein [Ziziphus jujuba]	gi|460413074|ref|XM_004251873.1|:PREDICTED: Solanum lycopersicum pirin-like protein At1g50590-like (LOC101268741), mRNA	B9SSQ9|B9SSQ9_RICCO:Pirin, putative;OS=Ricinus communis;
DN125811_c7_g8_i1	DOWN	-	-	-	XP_008238906.1:PREDICTED: protein P21like [Prunus mume]	gi|46949211|gb|AY589711.1|:Mirabilis jalapa clone FS-82 thaumatin-like protein mRNA, partial cds	B9RC64|B9RC64_RICCO:Osmotin, putative;OS=Ricinus communis;
DN124033_c2_g1_i1	DOWN	[GO:0016758:transferase activity, transferring hexosyl groups][GO:0008152:metabolic process]	-	-	XP_021766006.1:anthocyanidin 3Oglucoside 2"Oglucosyltransferaselike [Chenopodium quinoa]	-	A0A0J8BSS4|A0A0J8BSS4_BETVU:Glycosyltransferase;OS=Beta vulgaris subsp. vulgaris.;|GO:0016758,|GO:0008152,
DN112580_c0_g2_i2	DOWN	-	-	-	XP_009762320.1:PREDICTED: dehydrationresponsive protein RD22	-	V7AX31|V7AX31_PHAVU:Uncharacterized protein;OS=Phaseol

geneid	trend	GO	Pathway	TF	NR	NT	Uniprot
					[Nicotiana sylvestris]		us vulgaris;
DN118957_c0_g1_i1	DOWN	[GO:0003824:catalytic activity][GO:0009116:nucleoside metabolic process]	-	-	XP_010668210.1:PREDICTED: bark storage protein B [Beta vulgaris subsp. vulgaris] >KMT18015.1 hypothetical protein BVRB_2g032090 [Beta vulgaris subsp. vulgaris]	-	A0A0J8CWE1|A0A0J8CWE1_BETVU: Uncharacterized protein;OS=Beta vulgaris subsp. vulgaris.;|GO:0003824,|GO:0009116,
DN116505_c0_g1_i2	DOWN	[GO:0050660:flavin adenine dinucleotide binding][GO:0016614:oxidoreductase activity, acting on CH-OH group of donors]	-	FANhyb_rscf00000362.1.g00002.1:Fragaria x ananassa|CPP|CPP family protein	KNA22661.1:hypothetical protein SOVF_031390 [Spinacia oleracea]	-	A0A0K9RT00|A0A0K9RT00_SPIOL:Uncharacterized protein;OS=Spinacia oleracea;|GO:0050660,GO:0016614,
DN91879_c0_g1_i1	DOWN	-	-	-	XP_021731624.1:organspecific protein P4like [Chenopodium quinoa]	-	A0A0J8BK42|A0A0J8BK42_BETVU:Uncharacterized protein;OS=Beta vulgaris subsp.

geneid	trend	GO	Pathway	TF	NR	NT	Uniprot
							vulgaris.;
DN127807_c0_g2_i3	DOWN	[GO:0008289:lipid binding][GO:0006869:lipid transport]	-	-	XP_010679071.1:PREDICTED: nonspecific lipid transfer protein GPIanchored 2 [Beta vulgaris subsp. vulgaris] >KMT10188.1 hypothetical protein BVRB_5g119490 [Beta vulgaris subsp. vulgaris]	-	A0A0J8CDW0|A0A0J8CDW0_BETVU: Uncharacterized protein;OS=Beta vulgaris subsp. vulgaris.;|GO:0008289,|GO:0006869,
DN128972_c0_g1_i2	DOWN	[GO:0016021:integral component of membrane][GO:0005524:ATP binding][GO:0016887:ATPase activity][GO:0006810:transport]	-	Rsa1.0_03996.1_g00001.1:Raphanus sativus|MYB_related|MYB_related family protein	KNA08458.1:hypothetical protein SOVF_162410 isoform A [Spinacia oleracea]	gi|224053413|ref|XM_002297771.1|:Populus trichocarpa predicted protein, mRNA	A0A0K9QP53|A0A0K9QP53_SPIOL:Uncharacterized protein;OS=Spinacia oleracea;|GO:0016021,|GO:0005524,GO:0016887,|GO:0006810,

geneid	trend	GO	Pathway	TF	NR	NT	Uniprot
DN123742_c1_g5_i1	DOWN	[GO:0003676:nucleic acid binding]	-	XP_008245529.1:Prunus mume|E2F/DP|E2F/DP family protein	XP_010682933.1:PREDICTED: uncharacterized protein LOC104897695 [Beta vulgaris subsp. vulgaris]	-	F4NCI7|F4NCI7_BETVU:Uncharacterized protein;OS=Beta vulgaris subsp. vulgaris.;|GO:0003676,
DN126929_c1_g1_i1	DOWN	[GO:0016702:oxidoreductase activity, acting on single donors with incorporation of molecular oxygen, incorporation of two atoms of oxygen]	[ko00906:Carotenoid biosynthesis]	-	XP_021851772.1:probable carotenoid cleavage dioxygenase 4, chloroplastic [Spinacia oleracea] >KNA23039.1 hypothetical protein SOVF_028540 [Spinacia oleracea]	-	A0A0K9RVT5|A0A0K9RVT5_SPIOL: Uncharacterized protein;OS=Spinacia oleracea;|GO:0016702,
DN120423_c1_g1_i2	DOWN	[GO:0005576:extracellular region][GO:0005507:copper ion binding][GO:0016491:oxidoreductase activity]	-	Rsa1.0_03034.1_g00001.1:Raphanus sativus|ERF|ERF family protein	XP_010680584.1:PREDICTED: Lascorbate oxidase [Beta vulgaris subsp. vulgaris]	-	A0A0J8C9Y3|A0A0J8C9Y3_BETVU:Uncharacterized protein;OS=Beta vulgaris subsp. vulgaris.;|GO:0005576,|GO:0005507,GO:0016491,

geneid	trend	GO	Pathway	TF	NR	NT	Uniprot
DN102678_c0_g1_i1	DOWN	-	-	-	XP_021758313.1:uncharacterized protein LOC110723278 [Chenopodium quinoa]	-	A0A0K9R2L9|A0A0K9R2L9_SPIOL:Uncharacterized protein;OS=Spinacia oleracea;
DN120027_c0_g2_i1	DOWN	[GO:0016021:integral component of membrane][GO:0015369:calcium:proton antiporter activity]	-	DCAR_013815:Daucus carota|ERF|ERF family protein	XP_010687286.1:PREDICTED: vacuolar cation/proton exchanger 2 [Beta vulgaris subsp. vulgaris] >KMT03761.1 hypothetical protein BVRB_8g189090 [Beta vulgaris subsp. vulgaris]	gi|521657407|gb|GAIA01038608.1|:TSA: Salicornia europaea Unigene43215_Se200S transcribed RNA sequence	A0A0J8ELB7|A0A0J8ELB7_BETVU:Uncharacterized protein;OS=Beta vulgaris subsp. vulgaris.;|GO:0016021,|GO:0015369,
DN118837_c5_g4_i1	DOWN	-	-	-	-	-	-

geneid	trend	GO	Pathway	TF	NR	NT	Uniprot
DN119709_c4_g4_i1	DOWN	-	[ko00480:Glutathione metabolism][ko00980:Metabolism of xenobiotics by cytochrome P450][ko00982:Drug metabolism - cytochrome P450][ko01524:Platinum drug resistance][ko05204:Chemical carcinogenesis][ko05418:Fluid shear stress and atherosclerosis]	-	GAX86357.1:hypothetical protein CEUSTIGMA_g13769.t1 [Chlamydomonas eustigma]	-	R1E1F2|R1E1F2_EMIHU:Uncharacterized protein;OS=Emiliania huxleyi;
DN116840_c6_g2_i3	DOWN	-	-	-	-	-	-

geneid	trend	GO	Pathway	TF	NR	NT	Uniprot
DN127128_c5_g4_i2	DOWN	[GO:0016021:integral component of membrane][GO:0034256:chlorophyll(ide) b reductase activity][GO:0015996:chlorophyll catabolic process][GO:0010304:PSII associated light-harvesting complex II catabolic process]	[ko00860:Porphyrin and chlorophyll metabolism]	OPUNC03G25490.1:Oryza punctata|G2-like|G2-like family protein	XP_021723654.1:probable chlorophyll(ide) b reductase NYC1, chloroplastic [Chenopodium quinoa]	gi|521734838|gb|GAIA01009490.1|:TSA: Salicornia europaea Unigene9860_Se200S transcribed RNA sequence	A0A0J8EJD4|A0A0J8EJD4_BETVU:Uncharacterized protein;OS=Beta vulgaris subsp. vulgaris.;|GO:0016021,|GO:0034256,|GO:0015996,GO:0010304,
DN123972_c0_g5_i1	DOWN	[GO:0005737:cytoplasm][GO:0016021:integral component of membrane]	-	Rsa1.0_04342.1_g00002.1:Raphanus sativus|LBD|LBD family protein	XP_021985807.1:uncharacterized protein LOC110882004 [Helianthus annuus]	-	F6HKX9|F6HKX9_VITVI:Putative uncharacterized protein;OS=Vitis vinifera;GN=VIT_08s0007g01650{ECO:0000313|EMBL:CCB55521.1};|GO:0005737,GO:0016021,
DN121314_c0_g2_i2	DOWN	-	-	MDP0000283975:Malus domestica|NAC|NAC family protein	KJB74126.1:hypothetical protein B456_011G274400 [Gossypium raimondii]	-	A0A0D2UYL8|A0A0D2UYL8_GOSRA:Uncharacterized protein;OS=Gossypium raimondii;

geneid	trend	GO	Pathway	TF	NR	NT	Uniprot
DN122349_c0_g1_i2	DOWN	[GO:0005634:nucleus][GO:0003677:DNA binding]	-	Sp_092060_ukro.t1:Spinacia oleracea|SBP|SBP family protein	XP_021756356.1:squamosa promoterbindinglike protein 8 [Chenopodium quinoa]	-	A0A0K9R9E3|A0A0K9R9E3_SPIOL:Uncharacterized protein;OS=Spinacia oleracea;|GO:0005634,|GO:0003677,
DN113464_c0_g1_i1	DOWN	-	-	DCAR_008117:Daucus carota|bHLH|bHLH family protein	XP_021863469.1:cysteinerich repeat secretory protein 55 [Spinacia oleracea] >KNA16947.1 hypothetical protein SOVF_084240 [Spinacia oleracea]	-	A0A0K9RDN1|A0A0K9RDN1_SPIOL:Uncharacterized protein;OS=Spinacia oleracea;
DN115945_c0_g2_i1	DOWN	-	[ko03013:RNA transport][ko05134:Legionellosis]	-	BAJ90196.1:predicted protein [Hordeum vulgare subsp. vulgare]	gi|94468779|gb|DQ440206.1|:Aedes aegypti clone AET-205 translation elongation factor EF-1 alpha/Tu mRNA, complete cds	A0A1D1YYY9|A0A1D1YYY9_9ARAE:Elongation factor 1-alpha 1;OS=Anthurium amnicola.;

geneid	trend	GO	Pathway	TF	NR	NT	Uniprot
DN109405_c0_g1_i1	DOWN	-	[ko01521:EGFR tyrosine kinase inhibitor resistance][ko03010:Ribosome][ko04066:HIF-1 signaling pathway][ko04150:mTOR signaling pathway][ko04151:PI3K-Akt signaling pathway][ko04371:Apelin signaling pathway][ko04910:Insulin signaling pathway][ko05205:Proteoglycans in cancer]	Lus10020794:Linum usitatissimum|NAC|NAC family protein	XP_008444852.1:PREDICTED: LOW QUALITY PROTEIN: 40S ribosomal protein S6like [Cucumis melo]	gi|460050142|gb|GAGD01008124.1|:TSA: Amblyomma americanum seq_A03_wt_5d_016.ab1 transcribed RNA sequence	A0A1D1YB48|A0A1D1YB48_9ARAE: 40S ribosomal protein S6;OS=Anthurium amnicola.;
DN119977_c0_g1_i1	DOWN	-	-	-	-	-	-
DN108906_c0_g2_i1	DOWN	-	-	-	-	-	-
DN128228_c0_g4_i1	DOWN	-	-	-	-	-	-

geneid	trend	GO	Pathway	TF	NR	NT	Uniprot
DN115597_c0_g1_i4	DOWN	-	-	-	XP_021726506.1:uncharacterized protein LOC110693664 [Chenopodium quinoa]	-	A0A0J8BX80|A0A0J8BX80_BETVU:Uncharacterized protein;OS=Beta vulgaris subsp. vulgaris.;
DN112333_c0_g1_i2	DOWN	-	-	-	-	-	-
DN120744_c0_g3_i1	DOWN	[GO:0005524:ATP binding][GO:0005509:calcium ion binding][GO:0004674:protein serine/threonine kinase activity]	[ko04626:Plant-pathogen interaction][ko05145:Toxoplasmosis]	MDP0000318339:Malus domestica|FAR1|FAR1 family protein	XP_010674273.1:PREDICTED: calciumdependent protein kinase SK5 [Beta vulgaris subsp. vulgaris]	gi|224145671|ref|XM_002325690.1|:Populus trichocarpa calcium dependent protein kinase 12 (CPK12), mRNA	J3SIC5|J3SIC5_BETVU:Calmcium/calmodulin-dependent protein kinase CDPK2;OS=Beta vulgaris subsp. vulgaris.;|GO:0005524,GO:0005509,GO:0004674,
DN128228_c0_g1_i1	DOWN	-	-	-	-	-	-
DN125813_c5_g1_i5	DOWN	-	-	-	XP_015899916.1:PREDICTED: pirinlike protein [Ziziphus jujuba]	gi|297242516|gb|HM068865.1|:Carica papaya pirin mRNA, complete cds	A0A0K9RPB0|A0A0K9RPB0_SPIOL:Uncharacterized protein;OS=Spinacia oleracea;

geneid	trend	GO	Pathway	TF	NR	NT	Uniprot
DN118949_c4_g1_i1	DOWN	[GO:0016747:transferase activity, transferring acyl groups other than amino-acyl groups]	-	MDP0000294614:Malus domestica|NAC|NAC family protein	XP_021765621.1:uncharacterized acetyltransferase At3g50280like [Chenopodium quinoa]	-	A0A0K9RLH5|A0A0K9RLH5_SPIOL: Uncharacterized protein;OS=Spinacia oleracea;|GO:0016747,
DN122315_c0_g2_i1	DOWN	[GO:0004553:hydrolase activity, hydrolyzing O-glycosyl compounds][GO:0005975:carbohydrate metabolic process]	-	Pbr002083.1:Pyrus bretschneideri|ERF|ERF family protein	XP_021716143.1:glucan endo1,3betaglucosidaselike [Chenopodium quinoa]	-	A0A061GX19|A0A061GX19_THECC: O-Glycosyl hydrolases family 17 protein isoform 2;OS=Theobroma cacao;|GO:0004553,|GO:0005975,
DN121882_c1_g1_i2	DOWN	[GO:0016021:integral component of membrane][GO:0005385:zinc ion transmembrane transporter activity]	-	Achn296391:Actinidia chinensis|NF-YC|NF-YC family protein	XP_021735924.1:zinc transporter 4, chloroplasticlike [Chenopodium quinoa]	-	A0A0K9QFX9|A0A0K9QFX9_SPIOL: Uncharacterized protein;OS=Spinacia oleracea;|GO:0016021,|GO:0005385,
DN124738_c11_g3_i4	DOWN	-	-	-	-	-	-

geneid	trend	GO	Pathway	TF	NR	NT	Uniprot
DN116266_c0_g1_i1	DOWN	[GO:0005634:nucleus][GO:0043565:sequence-specific DNA binding][GO:0003700:DNA-binding transcription factor activity]	-	Bv_000370_cufa.t1:Beta vulgaris|HD-ZIP|HD-ZIP family protein	XP_021775014.1:homeoboxleucine zipper protein ATHB12like [Chenopodium quinoa]	-	A0A0J8B8M1|A0A0J8B8M1_BETVU:Uncharacterized protein;OS=Beta vulgaris subsp. vulgaris.;|GO:0005634,|GO:0043565,GO:0003700,
DN44785_c0_g1_i1	DOWN	-	[ko03010:Ribosome]	Achn022801:Actinidia chinensis|C2H2|C2H2 family protein	XP_016569631.1:PREDICTED: 40S ribosomal protein S23like [Capsicum annuum]	gi|402691834|ref|NM_213279.2|:Danio rerio ribosomal protein S2 (rps2), mRNA	A0A1D1YB27|A0A1D1YB27_9ARAE:40S ribosomal protein S2;OS=Anthurium amnicola.;
DN99309_c0_g1_i1	DOWN	-	-	-	-	-	-
DN125458_c6_g2_i3	DOWN	[GO:0016021:integral component of membrane]	-	676746044:Sisymbrium irio|MIKC_MADS|MIKC_MADS family protein	OVA16898.1:GDPfucose protein Ofucosyltransferase [Macleaya cordata]	gi|224120707|ref|XM_002330896.1|:Populus trichocarpa predicted protein, mRNA	M0SDI3|M0SDI3_MUSAM:Uncharacterized protein;OS=Musa acuminata subsp. malaccensis;|GO:0016021,

geneid	trend	GO	Pathway	TF	NR	NT	Uniprot
DN79193_c0_g1_i1	DOWN	[GO:0000786:nucleosome][GO:0005634:nucleus][GO:0003677:DNA binding]	[ko04217:Necroptosis][ko05034:Alcoholism][ko05322:Systemic lupus erythematosus]	PSME_00049691-RA:Pseudotsuga menziesii|TCP|TCP family protein	XP_021987444.1:probable histone H2A variant 1 [Helianthus annuus] >OTG09949.1 putative histone H2A variant 1 [Helianthus annuus]	gi|302849395|ref|XM_002956182.1|:Volvox carteri f. nagariensis hypothetical protein, mRNA	A0A022S3N5|A0A022S3N5_ERYGU:Histone H2A;OS=Erythranthe guttata;|GO:0000786,GO:0005634,|GO:0003677,
DN81245_c0_g1_i1	DOWN	-	-	-	XP_016547266.1:PREDICTED: phytanoylCoA dioxygenase [Capsicum annuum]	-	A0A068TWW8|A0A068TWW8_COFCA:Uncharacterized protein;OS=Coffea canephora;
DN117455_c3_g2_i5	DOWN	-	-	-	-	-	-
DN115622_c1_g2_i6	DOWN	-	-	-	XP_021853800.1:CRIB domaincontaining protein RIC10like isoform X1 [Spinacia oleracea] >KNA08367.1 hypothetical protein SOVF_163160	gi|521500808|gb|GAHZ01030112.1|:TSA: Salicornia europaea Unigene33160_SeCKS transcribed RNA sequence	A0A0K9QMA3|A0A0K9QMA3_SPIOL:Uncharacterized protein;OS=Spinacia oleracea;

geneid	trend	GO	Pathway	TF	NR	NT	Uniprot
					isoform A [Spinacia oleracea]		
DN114284_c2_g1_i2	DOWN	[GO:0008171:O-methyltransferase activity]	-	-	XP_021717347.1:(R,S)reticuline 7Omethyltransferase like [Chenopodium quinoa]	-	A0A0J8BLP8|A0A0J8BLP8_BETVU:Uncharacterized protein;OS=Beta vulgaris subsp. vulgaris.;|GO:0008171,
DN126722_c1_g1_i2	DOWN	[GO:0016021:integral component of membrane]	-	-	XP_010678454.1:PREDICTED: uncharacterized protein LOC104894001 [Beta vulgaris subsp. vulgaris] >KMT10758.1 hypothetical protein BVRB_5g114160 [Beta vulgaris subsp.	-	A0A0J8CFG8|A0A0J8CFG8_BETVU:Uncharacterized protein;OS=Beta vulgaris subsp. vulgaris.;|GO:0016021,

geneid	trend	GO	Pathway	TF	NR	NT	Uniprot
					vulgaris]		
DN124705_c5_g4_i1	DOWN	-	-	-	-	-	-
DN114741_c0_g1_i1	DOWN	[GO:0008725:DNA-3-methyladenine glycosylase activity][GO:0006284:base-excision repair]	[ko03410:Base excision repair]	-	XP_010681154.1:PREDICTED: uncharacterized protein LOC104896146 [Beta vulgaris subsp. vulgaris] >KMT08733.1 hypothetical protein BVRB_6g140040 [Beta vulgaris subsp. vulgaris]	-	A0A0J8C9Q5|A0A0J8C9Q5_BETVU:Uncharacterized protein;OS=Beta vulgaris subsp. vulgaris.;|GO:0008725,|GO:0006284,

geneid	trend	GO	Pathway	TF	NR	NT	Uniprot
DN125813_c5_g1_i1	DOWN	-	-	-	XP_015899916.1:PREDICTED: pirinlike protein [Ziziphus jujuba]	gi|297242516|gb|HM068865.1|:Carica papaya pirin mRNA, complete cds	A0A067L424|A0A067L424_JATCU:Uncharacterized protein;OS=Jatropha curcas;
DN123981_c0_g1_i6	DOWN	[GO:0016758:transferase activity, transferring hexosyl groups][GO:0008152:metabolic process]	-	-	XP_021735839.1:anthocyanidin 3Oglucoside 2"Oglucosyltransferaselike [Chenopodium quinoa]	-	A0A0K9QDA4|A0A0K9QDA4_SPIOL: Glycosyltransferase; OS=Spinacia oleracea;|GO:0016758,|GO:0008152,
DN128972_c2_g2_i2	DOWN	[GO:0016021:integral component of membrane][GO:0005524:ATP binding][GO:0016887:ATPase activity][GO:0006810:transport]	-	Rsa1.0_03996.1_g00001.1:Raphanus sativus|MYB_related|MYB_related family protein	KNA08458.1:hypothetical protein SOVF_162410 isoform A [Spinacia oleracea]	gi|168044447|ref|XM_001774641.1|:Physcomitrella patens subsp. patens ATP-binding cassette transporter, subfamily G, member 21, group PDR protein PpABCG21 (ppabcg21) mRNA, complete cds	A0A0K9QP53|A0A0K9QP53_SPIOL:Uncharacterized protein;OS=Spinacia oleracea;|GO:0016021,|GO:0005524,GO:0016887,|GO:0006810,

geneid	trend	GO	Pathway	TF	NR	NT	Uniprot
DN112289_c0_g1_i3	DOWN	-	-	Rsa1.0_04342.1_g00002.1:Raphanus sativus|LBD|LBD family protein	XP_021850472.1:uncharacterized protein LOC110790035 [Spinacia oleracea]	-	A0A0V0IYZ4|A0A0V0IYZ4_SOLCH:Putative ovule protein;OS=Solanum chacoense;
DN128833_c0_g1_i4	DOWN	[GO:0006950:response to stress]	-	-	CBY16488.1:ASR5 protein, partial [Solanum chilense]	gi|521742359|gb|GAIA01004989.1|:TSA: Salicornia europaea Unigene5276_Se200S transcribed RNA sequence	E8ZFX5|E8ZFX5_SOLCI:ASR5 protein;OS=Solanum chilense;|GO:0006950,
DN116462_c0_g1_i1	DOWN	-	-	Sopen10g007160.1: Solanum pennellii|M-type_MADS|M-type_MADS family protein	XP_021769264.1:uncharacterized protein LOC110733512 [Chenopodium quinoa]	gi|255559564|ref|XM_002520756.1|:Ricinus communis conserved hypothetical protein, mRNA	A0A0J8BDM0|A0A0J8BDM0_BETVU: Uncharacterized protein;OS=Beta vulgaris subsp. vulgaris.;
DN123231_c0_g6_i3	DOWN	[GO:0005524:ATP binding][GO:0003777:microtubule motor activity][GO:0007018:microtubule-based movement]	-	-	XP_021772951.1:kinesinlike protein KIN14S [Chenopodium quinoa]	-	A0A0J8CJA0|A0A0J8CJA0_BETVU:Uncharacterized protein;OS=Beta vulgaris subsp. vulgaris.;|GO:0005524,GO:0003777,|GO:0007018,

geneid	trend	GO	Pathway	TF	NR	NT	Uniprot
DN111667_c2_g1_i2	DOWN	-	-	-	-	-	-
DN104121_c0_g2_i1	DOWN	-	[ko04152:AMPK signaling pathway][ko04921:Oxytocin signaling pathway]	MDP0000428265:Malus domestica|NAC|NAC family protein	BAK02186.1:predicted protein [Hordeum vulgare subsp. vulgare]	gi|510195120|gb|GAKD01006997.1|:TSA: Melita plumulosa mira_rep_c7217 transcribed RNA sequence	A0A1D1Z8P1|A0A1D1Z8P1_9ARAE:Elongation factor 2;OS=Anthurium amnicola.;

geneid	trend	GO	Pathway	TF	NR	NT	Uniprot
DN131056_c0_g1_i1	DOWN	[GO:0005524:ATP binding]	[ko04015:Rap1 signaling pathway][ko04145:Phagosome][ko04210:Apoptosis][ko04390:Hippo signaling pathway][ko04391:Hippo signaling pathway - fly][ko04510:Focal adhesion][ko04520:Adherens junction][ko04530:Tight junction][ko04611:Platelet activation][ko04670:Leukocyte transendothelial migration][ko04745:Phototransduction - fly][ko04810:Regulation of actin cytoskeleton][ko04919:Thyroid hormone signaling pathway][ko04921:Oxytocin signaling pathway][ko05100:Bacterial invasion of epithelial	Niben101Scf00646g07008.1:Nicotiana benthamiana|Trihelix|Trihelix family protein	BAJ90061.1:predicted protein [Hordeum vulgare subsp. vulgare]	gi|498972847|ref|XM_004527356.1|:PREDICTED: Ceratitis capitata actin, cytoplasmic A3a-like (LOC101458512), transcript variant X2, mRNA	F2D4P0|F2D4P0_HORVD:Predicted protein;OS=Hordeum vulgare var. distichum;|GO:0005524,

geneid	trend	GO	Pathway	TF	NR	NT	Uniprot
DN118652_c0_g1_i1	DOWN	[GO:0048046:apoplast][GO:0005507:copper ion binding][GO:0052716:hydroquinone:oxygen oxidoreductase activity][GO:0003676:nucleic acid binding][GO:0046274:lignin catabolic process]	-	Rsa1.0_03034.1_g00001.1:Raphanus sativus|ERF|ERF family protein	XP_021743328.1:laccase17like [Chenopodium quinoa]	gi|356527381|ref|XM_003532242.1|:PREDICTED: Glycine max laccase-17-like (LOC100783768), mRNA	A0A0J8BXR7|A0A0J8BXR7_BETVU: Laccase;OS=Beta vulgaris subsp. vulgaris.;|GO:0048046,|GO:0005507,GO:0052716,GO:0003676,|GO:0046274,

geneid	trend	GO	Pathway	TF	NR	NT	Uniprot
DN115830_c1_g1_i5	DOWN	[GO:0005634:nucleus][GO:0000977:RNA polymerase II regulatory region sequence-specific DNA binding][GO:0003700:DNA-binding transcription factor activity][GO:0048440:carpel development][GO:0010076:maintenance of floral meristem identity][GO:0000165:MAPK cascade][GO:0048441:petal development][GO:0045944:positive regulation of transcription by RNA polymerase II][GO:0048442:sepal development][GO:0048443:stamen development][GO:0006351:transcription, DNA-templated]	-	Bv6_139710_fhny.t1:Beta vulgaris|MIKC_MADS|MIKC_MADS family protein	XP_010681125.1:PREDICTED: MADSbox protein CMB1 [Beta vulgaris subsp. vulgaris] >KMT08698.1 hypothetical protein BVRB_6g139710 [Beta vulgaris subsp. vulgaris]	gi|22091480|emb|AJ271151.1|:Daucus carota subspecies sativus mRNA for putative MADS box transcription factor (mads5 gene)	A0A0J8C9M1|A0A0J8C9M1_BETVU: Uncharacterized protein;OS=Beta vulgaris subsp. vulgaris.;|GO:0005634,|GO:0000977,GO:0003700,|GO:0048440,GO:0010076,GO:0000165,GO:0048441,GO:0045944,GO:0048442,GO:0048443,GO:0006351,

geneid	trend	GO	Pathway	TF	NR	NT	Uniprot
DN86967_c0_g1_i1	DOWN	-	[ko04141:Protein processing in endoplasmic reticulum][ko04213: Longevity regulating pathway - multiple species]	-	-	-	-
DN121349_c1_g2_i1	DOWN	-	-	-	XP_021745877.1:CRIB domaincontaining protein RIC4like [Chenopodium quinoa]	-	A0A0J8CSK5|A0A0J8CSK5_BETVU:Uncharacterized protein;OS=Beta vulgaris subsp. vulgaris.;
DN123090_c1_g1_i6	DOWN	[GO:0005634:nucleus][GO:0003677: DNA binding][GO:0003700:DNA-binding transcription factor activity][GO:0007275:multicellular organism development][GO:0006351:transcription, DNA-templated]	-	XP_010109804.1:Morus notabilis|AP2|AP2 family protein	XP_010109804.1:Floral homeotic protein APETALA 2 [Morus notabilis] >EXC24730.1 Floral homeotic protein APETALA 2 [Morus notabilis]	gi|388329712|gb|JQ398741.1|:Camellia sinensis transcription factor APETALA2 mRNA, complete cds	W9S3F7|W9S3F7_9ROSA:Floral homeotic protein APETALA 2;OS=Morus notabilis.;|GO:0005634,|GO:0003677,GO:0003700,|GO:0007275,GO:0006351,

geneid	trend	GO	Pathway	TF	NR	NT	Uniprot
DN86587_c0_g5_i1	DOWN	-	-	Dca56676.1:Dianthus caryophyllus|B3|B3 family protein	XP_021729317.1:uncharacterized protein LOC110696333 [Chenopodium quinoa]	-	A0A0A9QML7|A0A0A9QML7_ARUDO:Uncharacterized protein;OS=Arundo donax;
DN116032_c1_g1_i2	DOWN	[GO:0031225:anchored component of membrane][GO:0016049:cell growth][GO:0010215:cellulose microfibril organization]	-	Sopen01g046240.1:Solanum pennellii|M-type_MADS|M-type_MADS family protein	XP_006374429.1:phytochelatin synthetaselike family protein [Populus trichocarpa] >ABK96050.1 unknown [Populus trichocarpa] >ERP52226.1 phytochelatin synthetaselike family protein [Populus trichocarpa]	gi|224124109|ref|XM_002330071.1|:Populus trichocarpa predicted protein, mRNA	A9PI47|A9PI47_POPTR:Phytochelatin synthetase-like family protein;OS=Populus trichocarpa;|GO:0031225,|GO:0016049,GO:0010215,

geneid	trend	GO	Pathway	TF	NR	NT	Uniprot
DN123978_c1_g4_i1	DOWN	[GO:0016021:integral component of membrane][GO:0005524:ATP binding][GO:0030247:polysaccharide binding][GO:0004672:protein kinase activity]	[ko04064:NF-kappa B signaling pathway][ko04620:Toll-like receptor signaling pathway][ko04621:NOD-like receptor signaling pathway][ko04624:Toll and Imd signaling pathway][ko04722:Neurotrophin signaling pathway][ko05133:Pertussis][ko05140:Leishmaniasis][ko05142:Chagas disease (American trypanosomiasis)][ko05145:Toxoplasmosis][ko05152:Tuberculosis][ko05162:Measles][ko05164:Influenza A]	AA10G00404:Aethionema arabicum|WRKY|WRKY family protein	XP_021734913.1:uncharacterized protein LOC110701590 [Chenopodium quinoa]	-	A0A0J8CLW9|A0A0J8CLW9_BETVU:Uncharacterized protein;OS=Beta vulgaris subsp. vulgaris.;|GO:0016021,|GO:0005524,GO:0030247,GO:0004672,

geneid	trend	GO	Pathway	TF	NR	NT	Uniprot
DN128286_c3_g22_i3	DOWN	-	-	-	XP_021853125.1:uncharacterized protein LOC110792607 [Spinacia oleracea]	-	A0A078DME7|A0A078DME7_BRANA:BnaA08g18620D protein;OS=Brassica napus;
DN129134_c10_g14_i4	DOWN	[GO:0016021:integral component of membrane][GO:0020037:heme binding][GO:0005506:iron ion binding][GO:0016705:oxidoreductase activity, acting on paired donors, with incorporation or reduction of molecular oxygen]	-	676766070:Sisymbrium irio|bHLH|bHLH family protein	XP_021863529.1:cytochrome P450 71A3like [Spinacia oleracea]	-	A0A0J8B8F4|A0A0J8B8F4_BETVU:Uncharacterized protein;OS=Beta vulgaris subsp. vulgaris.;|GO:0016021,|GO:0020037,GO:0005506,GO:0016705,

geneid	trend	GO	Pathway	TF	NR	NT	Uniprot
DN125332_c2_g1_i2	DOWN	[GO:0005576:extracellular region][GO:0020037:heme binding][GO:0046872:metal ion binding][GO:0004601:peroxidase activity][GO:0042744:hydrogen peroxide catabolic process][GO:0006979:response to oxidative stress]	[ko00940:Phenylpropanoid biosynthesis]	EPS67213.1:Genlisea aurea|NAC|NAC family protein	XP_021838020.1:peroxidase 60like [Spinacia oleracea] >CAA71489.1 peroxidase [Spinacia oleracea]	-	P93546|P93546_SPIOL:Peroxidase;OS=Spinacia oleracea;|GO:0005576,|GO:0020037,GO:0046872,GO:0004601,|GO:0042744,GO:0006979,
DN121736_c1_g10_i2	DOWN	-	-	-	-	-	-
DN168635_c0_g1_i1	DOWN	[GO:0008270:zinc ion binding]	-	-	AFK36347.1:unknown [Lotus japonicus]	gi|241998843|ref|XM_002434020.1|:Ixodes scapularis muscle LIM protein isoform A isoform, putative, mRNA	I3S7V5|I3S7V5_LOTJA:Uncharacterized protein;OS=Lotus japonicus;|GO:0008270,

geneid	trend	GO	Pathway	TF	NR	NT	Uniprot
DN116047_c1_g3_i1	DOWN	[GO:0016021:integral component of membrane]	-	-	XP_010693638.1:PREDICTED: uncharacterized protein LOC104906566 [Beta vulgaris subsp. vulgaris]	-	F6HFJ7|F6HFJ7_VITVI:Putative uncharacterized protein;OS=Vitis vinifera;GN=VIT_01s0011g04650 {ECO:0000313|EMBL:CCB50846.1};|GO:0016021,
DN125434_c3_g4_i1	DOWN	-	-	-	-	-	-
DN105877_c0_g1_i1	DOWN	[GO:0000166:nucleotide binding][GO:0003723:RNA binding]	-	-	KDP40836.1:hypothetical protein JCGZ_24835 [Jatropha curcas]	-	A0A067KXB0|A0A067KXB0_JATCU: Uncharacterized protein;OS=Jatropha curcas;|GO:0000166,GO:0003723,
DN120579_c0_g2_i2	DOWN	-	-	-	-	-	-

geneid	trend	GO	Pathway	TF	NR	NT	Uniprot
DN123978_c1_g4_i5	DOWN	[GO:0016021:integral component of membrane][GO:0005524:ATP binding][GO:0030247:polysaccharide binding][GO:0004672:protein kinase activity]	[ko04064:NF-kappa B signaling pathway][ko04620:Toll-like receptor signaling pathway][ko04621:NOD-like receptor signaling pathway][ko04624:Toll and Imd signaling pathway][ko04722:Neurotrophin signaling pathway][ko05133:Pertussis][ko05140:Leishmaniasis][ko05142:Chagas disease (American trypanosomiasis)][ko05145:Toxoplasmosis][ko05152:Tuberculosis][ko05162:Measles][ko05164:Influenza A]	AA10G00404:Aethionema arabicum|WRKY|WRKY family protein	XP_021734913.1:uncharacterized protein LOC110701590 [Chenopodium quinoa]	-	A0A0J8CLW9|A0A0J8CLW9_BETVU:Uncharacterized protein;OS=Beta vulgaris subsp. vulgaris.;|GO:0016021,|GO:0005524,GO:0030247,GO:0004672,

geneid	trend	GO	Pathway	TF	NR	NT	Uniprot
DN125226_c2_g1_i1	DOWN	[GO:0005576:extracellular region][GO:0005773:vacuole][GO:0004650:polygalacturonase activity][GO:0005975:carbohydrate metabolic process][GO:0071555:cell wall organization]	-	KZV57651.1:Dorcoceras hygrometricum|MYB_related|MYB_related family protein	XP_010679488.1:PREDICTED: probable polygalacturonase isoform X1 [Beta vulgaris subsp. vulgaris]	gi|224130825|ref|XM_002320898.1|:Populus trichocarpa predicted protein, mRNA	A0A0J8F303|A0A0J8F303_BETVU:Uncharacterized protein;OS=Beta vulgaris subsp. vulgaris.;|GO:0005576,GO:0005773,|GO:0004650,|GO:0005975,GO:0071555,
DN82526_c0_g3_i1	DOWN	[GO:0006952:defense response][GO:0009607:response to biotic stimulus]	-	-	AAC14179.1:major latex protein homolog [Mesembryanthemum crystallinum]	-	O65178|O65178_MESCR:Major latex protein homolog;OS=Mesembryanthemum crystallinum;|GO:0006952,GO:0009607,
DN105361_c0_g2_i1	DOWN	-	[ko03410:Base excision repair][ko04140:Autophagy - animal][ko04217:Necroptosis]	-	EOY15683.1:Leucine rich repeat protein kinase family protein, putative [Theobroma cacao]	-	A0A1D1Z9A5|A0A1D1Z9A5_9ARAE: High mobility group protein DSP1;OS=Anthurium amnicola.;

geneid	trend	GO	Pathway	TF	NR	NT	Uniprot
DN188400_c0_g1_i1	DOWN	[GO:0008234:cysteine-type peptidase activity]	-	-	OIW19233.1:hypothetical protein TanjilG_20358 [Lupinus angustifolius]	-	D8SAV1|D8SAV1_SELML:Putative uncharacterized protein;OS=Selaginella moellendorffii;|GO:0008234,
DN125918_c2_g1_i5	DOWN	-	-	-	XP_020273363.1:uncharacterized protein LOC109848329 [Asparagus officinalis]	-	A0A0J8CDD8|A0A0J8CDD8_BETVU:Uncharacterized protein;OS=Beta vulgaris subsp. vulgaris.;
DN127807_c0_g2_i6	DOWN	-	-	-	-	-	-
DN128972_c1_g2_i1	DOWN	[GO:0016021:integral component of membrane][GO:0005524:ATP binding][GO:0016887:ATPase activity][GO:0006810:transport]	-	Rsa1.0_03996.1_g00001.1:Raphanus sativus|MYB_related|MYB_related family protein	KNA08458.1:hypothetical protein SOVF_162410 isoform A [Spinacia oleracea]	gi|521718059|gb|GAIA01011766.1|:TSA: Salicornia europaea Unigene12383_Se200S transcribed RNA sequence	A0A0K9QP53|A0A0K9QP53_SPIOL:Uncharacterized protein;OS=Spinacia oleracea;|GO:0016021,|GO:0005524,GO:0016887,|GO:0006810,

geneid	trend	GO	Pathway	TF	NR	NT	Uniprot
DN110367_c0_g1_i1	DOWN	-	-	-	XP_021767051.1:putative ripeningrelated protein 1 [Chenopodium quinoa] >XP_021746932.1 putative ripeningrelated protein 1 [Chenopodium quinoa] >XP_021748915.1 putative ripeningrelated protein 1 [Chenopodium quinoa] >XP_021748916.1 putative ripeningrelated protein 1 [Chenopodium quinoa] >XP_021751453.1	-	A0A0K9Q9J2|A0A0K9Q9J2_SPIOL:Uncharacterized protein;OS=Spinacia oleracea;

geneid	trend	GO	Pathway	TF	NR	NT	Uniprot
					putative ripeningrelated protein 1 [Chenopodium quinoa]		
DN112834_c0_g2_i1	DOWN	[GO:0005524:ATP binding][GO:0004672:protein kinase activity]	-	Zmw_sc01274.1.g00150.1:Zoysia matrella|Trihelix|Trihelix family protein	XP_010677489.1:PREDICTED: probable receptorlike protein kinase At1g33260 [Beta vulgaris subsp. vulgaris] >KMT11473.1 hypothetical protein	-	A0A0J8CHD8|A0A0J8CHD8_BETVU: Uncharacterized protein;OS=Beta vulgaris subsp. vulgaris.;|GO:0005524,GO:0004672,

geneid	trend	GO	Pathway	TF	NR	NT	Uniprot
					BVRB_5g107980 [Beta vulgaris subsp. vulgaris]		
DN121127_c0_g2_i6	DOWN	[GO:0008171:O-methyltransferase activity]	-	-	XP_021717347.1:(R,S)reticuline 7Omethyltransferase like [Chenopodium quinoa]	-	A0A0J8BLP8|A0A0J8BLP8_BETVU:Uncharacterized protein;OS=Beta vulgaris subsp. vulgaris.;|GO:0008171,

geneid	trend	GO	Pathway	TF	NR	NT	Uniprot
DN117321_c1_g1_i1	DOWN	[GO:0005576:extracellular region][GO:0020037:heme binding][GO:0046872:metal ion binding][GO:0004601:peroxidase activity][GO:0042744:hydrogen peroxide catabolic process][GO:0006979:response to oxidative stress]	[ko00940:Phenylpropanoid biosynthesis]	MDP0000449389:Malus domestica|M-type_MADS|M-type_MADS family protein	XP_021842095.1:peroxidase 66like [Spinacia oleracea] >KNA11623.1 hypothetical protein SOVF_133580 [Spinacia oleracea]	gi|521628089|gb|GAIA01051989.1|:TSA: Salicornia europaea Unigene61009_Se200S transcribed RNA sequence	A0A0K9QY54|A0A0K9QY54_SPIOL:Peroxidase;OS=Spinacia oleracea;|GO:0005576,|GO:0020037,GO:0046872,GO:0004601,|GO:0042744,GO:0006979,
DN112697_c0_g4_i1	DOWN	-	-	CCG019201.1:Populus euphratica|HSF|HSF family protein	XP_021745419.1:fasciclinlike arabinogalactan protein 11 [Chenopodium quinoa]	-	A0A0K9QFL5|A0A0K9QFL5_SPIOL:Uncharacterized protein;OS=Spinacia oleracea;
DN127085_c1_g1_i4	DOWN	[GO:0016021:integral component of membrane][GO:0008271:secondary active sulfate transmembrane transporter activity]	-	-	XP_021723678.1:probable sulfate transporter 3.5 [Chenopodium quinoa]	gi|521689235|gb|GAIA01023091.1|:TSA: Salicornia europaea Unigene24682_Se200S transcribed RNA sequence	A0A0K9RRK3|A0A0K9RRK3_SPIOL: Uncharacterized protein;OS=Spinacia oleracea;|GO:0016021,|GO:0008271,
DN104283_c6_g4_i1	DOWN	-	-	-	-	-	-

geneid	trend	GO	Pathway	TF	NR	NT	Uniprot
DN111337_c0_g1_i3	DOWN	[GO:0020037:heme binding][GO:0005506:iron ion binding][GO:0004497:monooxygenase activity][GO:0016705:oxidoreductase activity, acting on paired donors, with incorporation or reduction of molecular oxygen]	-	Cc10_g03650:Coffea canephora|G2-like|G2-like family protein	XP_020419542.1:cytochrome P450 CYP736A12 [Prunus persica] >ONI09126.1 hypothetical protein PRUPE_5G219700 [Prunus persica]	-	M5WWE2|M5WWE2_PRUPE:Uncharacterized protein;OS=Prunus persica;|GO:0020037,GO:0005506,GO:0004497,GO:0016705,
DN107826_c1_g1_i6	DOWN	-	[ko04013:MAPK signaling pathway - fly][ko04110:Cell cycle][ko04114:Oocyte meiosis][ko04151:PI3K-Akt signaling pathway][ko04212:Longevity regulating pathway - worm][ko04390:Hippo signaling pathway][ko04391: Hippo signaling pathway - fly][ko05161:Hepati	GSBRNA2T00057732001:Brassica napus|E2F/DP|E2F/DP family protein	BAJ95666.1:predicted protein [Hordeum vulgare subsp. vulgare]	gi|116630880|emb|CT837434.1|:Oryza sativa (indica cultivar-group) cDNA clone:OSIGCRA106K10, full insert sequence	A0A1D1YYN0|A0A1D1YYN0_9ARAE:14-3-3 protein zeta;OS=Anthurium amnicola.;

geneid	trend	GO	Pathway	TF	NR	NT	Uniprot
			tis B][ko05169:Epstein-Barr virus infection][ko05203:Viral carcinogenesis]				
DN122272_c1_g1_i7	DOWN	-	-	-	XP_021865376.1:uncharacterized protein LOC110804116 [Spinacia oleracea] >KNA03271.1 hypothetical protein SOVF_210800 [Spinacia oleracea]	gi|521678164|gb|GAIA01028276.1|:TSA: Salicornia europaea Unigene30966_Se200S transcribed RNA sequence	A0A0K9Q9T1|A0A0K9Q9T1_SPIOL:Uncharacterized protein;OS=Spinacia oleracea;

geneid	trend	GO	Pathway	TF	NR	NT	Uniprot
DN119781_c0_g1_i2	DOWN	[GO:0016021:integral component of membrane][GO:0005886:plasma membrane][GO:0009506:plasmodesma][GO:0005524:ATP binding][GO:0009678:hydrogen-translocating pyrophosphatase activity][GO:0004427:inorganic diphosphatase activity][GO:0004674:protein serine/threonine kinase activity][GO:0006952:defense response][GO:0006468:protein phosphorylation]	[ko04064:NF-kappa B signaling pathway][ko04620:Toll-like receptor signaling pathway][ko04621:NOD-like receptor signaling pathway][ko04624:Toll and Imd signaling pathway][ko04722:Neurotrophin signaling pathway][ko05133:Pertussis][ko05140:Leishmaniasis][ko05142:Chagas disease (American trypanosomiasis)][ko05145:Toxoplasmosis][ko05152:Tuberculosis][ko05162:Measles][ko05164:Influenza A]	Kaladp0839s0004.1.p:Kalanchoe laxiflora|ERF|ERF family protein	XP_010267803.1:PREDICTED: putative receptorlike protein kinase At4g00960 isoform X2 [Nelumbo nucifera]	gi|521508180|gb|GAHZ01022740.1|:TSA: Salicornia europaea Unigene24455_SeCKS transcribed RNA sequence	F6HVX5|F6HVX5_VITVI:Putative uncharacterized protein;OS=Vitis vinifera;GN=VIT_10s0071g01200 {ECO:0000313|EMBL:CCB58838.1};|GO:0016021,GO:0005886,GO:0009506,|GO:0005524,GO:0009678,GO:0004427,GO:0004674,|GO:0006952,GO:0006468,GO:0015992,

geneid	trend	GO	Pathway	TF	NR	NT	Uniprot
DN108781_c0_g1_i3	DOWN	[GO:0016021:integral component of membrane][GO:0004970:ionotropic glutamate receptor activity]	-	XP_013628248.1:Brassica oleracea|B3|B3 family protein	XP_010682646.1:PREDICTED: glutamate receptor 2.6 [Beta vulgaris subsp. vulgaris] >KMT07344.1 hypothetical protein BVRB_6g149710 [Beta vulgaris subsp. vulgaris]	-	A0A0J8C530|A0A0J8C530_BETVU:Uncharacterized protein;OS=Beta vulgaris subsp. vulgaris.;|GO:0016021,|GO:0004970,
DN112929_c0_g1_i2	DOWN	[GO:0005634:nucleus][GO:0003677:DNA binding][GO:0003700:DNA-binding transcription factor activity][GO:0006351:transcription, DNA-templated]	-	Sp_169520_rsxc.t2:Spinacia oleracea|MIKC_MADS|MIKC_MADS family protein	XP_021737016.1:truncated transcription factor CAULIFLOWER Alike isoform X2 [Chenopodium quinoa]	gi|32478048|gb|AY306162.1|:Phytolacca americana FRUITFULL-like MADS-box (PaFL2) mRNA, partial cds	Q7XBL4|Q7XBL4_PHYAM:FRUITFULL-like MADS-box;OS=Phytolacca americana;|GO:0005634,|GO:0003677,GO:0003700,|GO:0006351,
DN127807_c0_g1_i1	DOWN	-	-	-	-	-	-

geneid	trend	GO	Pathway	TF	NR	NT	Uniprot
DN115174_c6_g4_i4	DOWN	[GO:0005634:nucleus][GO:0003677:DNA binding][GO:0006355:regulation of transcription, DNA-templated][GO:0006351:transcription, DNA-templated]	-	Dca51258.1:Dianthus caryophyllus|NAC|NAC family protein	XP_021758777.1:NAC transcription factor 56like [Chenopodium quinoa]	gi|350528640|gb|JN675710.1|:Prunus salicina NAC domain class transcription factor mRNA, complete cds	A0A0M4FP05|A0A0M4FP05_MANES:NAC transcription factors 21;OS=Manihot esculenta;|GO:0005634,|GO:0003677,|GO:0006355,GO:0006351,
DN119427_c0_g1_i9	DOWN	[GO:0016021:integral component of membrane][GO:0055085:transmembrane transport]	-	Jcr4S00206.70:Jatropha curcas|FAR1|FAR1 family protein	XP_018856104.1:PREDICTED: protein PINLIKES 7like [Juglans regia]	gi|521703437|gb|GAIA01016206.1|:TSA: Salicornia europaea Unigene16917_Se200S transcribed RNA sequence	A0A0J8C515|A0A0J8C515_BETVU:Uncharacterized protein;OS=Beta vulgaris subsp. vulgaris.;|GO:0016021,|GO:0055085,
DN119526_c2_g1_i1	DOWN	-	-	-	-	-	-
DN115628_c0_g6_i2	DOWN	-	-	-	-	-	-
DN126604_c2_g1_i1	DOWN	-	-	-	-	-	-

geneid	trend	GO	Pathway	TF	NR	NT	Uniprot
DN116555_c0_g11_i1	DOWN	-	-	-	EOY07334.1:PAR1 protein [Theobroma cacao]	-	A0A061EYI9|A0A061EYI9_THECC:PAR1 protein;OS=Theobroma cacao;
DN117882_c0_g1_i1	DOWN	[GO:0046872:metal ion binding]	-	Bv4_092260_ddzu.t1:Beta vulgaris|C2H2|C2H2 family protein	XP_010694273.1:PREDICTED: uncharacterized protein LOC104907097 [Beta vulgaris subsp. vulgaris] >KMS98537.1 hypothetical protein BVRB_4g092260 [Beta vulgaris subsp. vulgaris]	gi|170676245|gb|EU560900.1|:Capsicum annuum clone DEG190-B transcription factor mRNA, complete cds	A0A0J8BEY8|A0A0J8BEY8_BETVU:Uncharacterized protein;OS=Beta vulgaris subsp. vulgaris.;|GO:0046872,
DN113695_c0_g1_i1	DOWN	-	-	-	XP_021752134.1:uncharacterized protein LOC110717681 [Chenopodium quinoa]	-	A0A0K9R8W5|A0A0K9R8W5_SPIOL:Uncharacterized protein;OS=Spinacia oleracea;

geneid	trend	GO	Pathway	TF	NR	NT	Uniprot
DN120521_c1_g1_i2	DOWN	-	-	XP_013601330.1:Brassica oleracea|ERF|ERF family protein	XP_021738571.1:deSIlike protein At4g17486 [Chenopodium quinoa]	-	A0A0K9RPX6|A0A0K9RPX6_SPIOL:Uncharacterized protein;OS=Spinacia oleracea;
DN122713_c2_g3_i1	DOWN	-	-	-	-	-	-
DN123573_c0_g1_i1	DOWN	[GO:0003993:acid phosphatase activity][GO:0046872:metal ion binding]	-	PDK_30s873781g005:Phoenix dactylifera|C3H|C3H family protein	XP_021764754.1:purple acid phosphatase 22like [Chenopodium quinoa]	-	A0A0J8EUF0|A0A0J8EUF0_BETVU:Purple acid phosphatase;OS=Beta vulgaris subsp. vulgaris.;|GO:0003993,GO:0046872,
DN118058_c0_g3_i1	DOWN	-	[ko04260:Cardiac muscle contraction][ko04261:Adrenergic signaling in cardiomyocytes][ko05416:Viral myocarditis]	Mapoly0012s0190.1.p:Marchantia polymorpha|C3H|C3H family protein	XP_001755337.1:predicted protein [Physcomitrella patens] >EDQ79817.1 predicted protein [Physcomitrella patens]	gi|241997621|ref|XM_002433415.1|:Ixodes scapularis myosin heavy chain, skeletal muscle or cardiac muscle, putative, mRNA	A0A1D1XV65|A0A1D1XV65_9ARAE: Myosin heavy chain, muscle;OS=Anthurium amnicola.;

geneid	trend	GO	Pathway	TF	NR	NT	Uniprot
DN116525_c1_g1_i2	DOWN	[GO:0046872:metal ion binding][GO:0016491:oxidoreductase activity]	[ko00904:Diterpenoid biosynthesis]	Zpz_sc00083.1.g00290.1.am.mk:Zoysia pacifica|Dof|Dof family protein	XP_021854850.1:gibberellin 2betadioxygenase 8like isoform X1 [Spinacia oleracea] >XP_021854851.1 gibberellin 2betadioxygenase 8like isoform X1 [Spinacia oleracea] >XP_021854852.1 gibberellin 2betadioxygenase 8like isoform X1 [Spinacia oleracea]	gi|460405863|ref|XM_004248346.1|:PREDICTED: Solanum lycopersicum gibberellin 2-beta-dioxygenase 8-like (LOC101255340), mRNA	Q5D0G3|Q5D0G3_SPIOL:Gibberellin 2-oxidase 3;OS=Spinacia oleracea;|GO:0046872,GO:0016491,
DN122401_c7_g1_i8	DOWN	-	-	-	XP_021767973.1:VAN3binding proteinlike [Chenopodium quinoa]	-	A0A0J8CEC2|A0A0J8CEC2_BETVU:Uncharacterized protein;OS=Beta vulgaris subsp. vulgaris.;

geneid	trend	GO	Pathway	TF	NR	NT	Uniprot
DN120579_c0_g2_i1	DOWN	[GO:0016021:integral component of membrane]	-	-	ADV02768.1:putative low temperature and salt responsive protein isoform 1 [Ipomoea batatas] >ADV02769.1 putative low temperature and salt responsive protein isoform 2 [Ipomoea batatas]	gi|24745953|dbj|AB061265.1|:Solanum tuberosum StLTSR mRNA for low temperature and salt responsive protein, complete cds	V9HZQ9|V9HZQ9_IPOBA:Putative low temperature and salt responsive protein isoform 1;OS=Ipomoea batatas;|GO:0016021,
DN100305_c1_g2_i1	DOWN	-	[ko03010:Ribosome]	XP_010103855.1:Morus notabilis|bZIP|bZIP family protein	XP_002466868.1:60S ribosomal protein L10 [Sorghum bicolor] >EER93866.1 hypothetical protein SORBI_3001G177800 [Sorghum bicolor]	gi|182888863|gb|BC164313.1|:Danio rerio ribosomal protein L10, mRNA (cDNA clone MGC:191488 IMAGE:100059797), complete cds	A0A1D1ZHF4|A0A1D1ZHF4_9ARAE:60S ribosomal protein L10;OS=Anthurium amnicola.;
DN122081_c1_g1_i4	DOWN	-	-	KFK43895.1:Arabis alpina|STAT|STAT family protein	KNA12238.1:hypothetical protein SOVF_127760 [Spinacia oleracea]	gi|521526499|gb|GAHZ01010146.1|:TSA: Salicornia europaea Unigene10745_SeCKS transcribed RNA	A0A0K9QZW9|A0A0K9QZW9_SPIOL:Uncharacterized protein;OS=Spinacia oleracea;

geneid	trend	GO	Pathway	TF	NR	NT	Uniprot
						sequence	
DN110612_c0_g1_i1	DOWN	[GO:0005634:nucleus][GO:0003677: DNA binding][GO:0003700:DNA-binding transcription factor activity][GO:0006351:transcription, DNA-templated]	-	Bv1_002890_zepi.t1:Beta vulgaris|ERF|ERF family protein	XP_010696241.1:PREDICTED: ethyleneresponsive transcription factor ERF023 [Beta vulgaris subsp. vulgaris] >KMT20286.1 hypothetical protein BVRB_1g002890 [Beta vulgaris subsp. vulgaris]	-	A0A0J8FX79|A0A0J8FX79_BETVU:Uncharacterized protein;OS=Beta vulgaris subsp. vulgaris.;|GO:0005634,|GO:0003677,GO:0003700,|GO:0006351,
DN120889_c0_g2_i4	DOWN	-	-	-	-	-	-
DN120768_c0_g2_i2	DOWN	[GO:0016021:integral component of membrane][GO:0008271:secondary active sulfate transmembrane transporter activity]	-	GSMUA_Achr9P13010_001:Musa acuminata|MIKC_MADS|MIKC_MADS family protein	XP_021723678.1:probable sulfate transporter 3.5 [Chenopodium quinoa]	-	A0A0K9RRK3|A0A0K9RRK3_SPIOL:Uncharacterized protein;OS=Spinacia oleracea;|GO:0016021,|GO:0008271,

geneid	trend	GO	Pathway	TF	NR	NT	Uniprot
DN111653_c0_g2_i1	DOWN	-	[ko03010:Ribosome]	MDP0000279204:Malus domestica|MYB_related|MYB_related family protein	GAQ80594.1:40S ribosomal protein S3 family protein [Klebsormidium nitens]	-	A0A1D1XCH0|A0A1D1XCH0_9ARAE:40S ribosomal protein S3;OS=Anthurium amnicola.;
DN113273_c1_g2_i1	DOWN	-	-	-	-	-	-
DN120667_c1_g2_i1	DOWN	-	-	-	-	-	-
DN103642_c2_g4_i1	DOWN	-	-	-	-	-	-
DN112388_c5_g26_i1	DOWN	-	-	-	-	-	-
DN114022_c0_g1_i4	DOWN	[GO:0016788:hydrolase activity, acting on ester bonds]	-	-	XP_021720400.1:GDSL esterase/lipase 2like [Chenopodium quinoa]	-	A0A0K9RT83|A0A0K9RT83_SPIOL:Uncharacterized protein;OS=Spinacia oleracea;|GO:0016788,
DN105810_c3_g1_i1	DOWN	-	-	-	-	-	-

geneid	trend	GO	Pathway	TF	NR	NT	Uniprot
DN85036_c0_g2_i1	DOWN	[GO:0005840:ribosome][GO:0003735:structural constituent of ribosome][GO:0006412:translation]	[ko03010:Ribosome]	-	ACG44046.1:60S ribosomal protein L11 [Zea mays]	gi|114864834|gb|DQ910351.1|:Anopheles funestus clone AF-42 60S ribosomal protein L11 mRNA, complete cds	B6U3W3|B6U3W3_MAIZE:60S ribosomal protein L11;OS=Zea mays;|GO:0005840,|GO:0003735,|GO:0006412,
DN115174_c6_g4_i5	DOWN	[GO:0005634:nucleus][GO:0003677:DNA binding][GO:0006355:regulation of transcription, DNA-templated][GO:0006351:transcription, DNA-templated]	-	Dca51258.1:Dianthus caryophyllus|NAC|NAC family protein	XP_008386130.1:PREDICTED: NAC transcription factor 56like [Malus domestica]	gi|350528640|gb|JN675710.1|:Prunus salicina NAC domain class transcription factor mRNA, complete cds	A0A0M4FP05|A0A0M4FP05_MANES:NAC transcription factors 21;OS=Manihot esculenta;|GO:0005634,|GO:0003677,|GO:0006355,GO:0006351,
DN121617_c1_g2_i4	DOWN	[GO:0030246:carbohydrate binding][GO:0016853:isomerase activity][GO:0005975:carbohydrate metabolic process]	[ko00010:Glycolysis / Gluconeogenesis]	MDP0000239688:Malus domestica|bZIP|bZIP family protein	XP_021757607.1:putative glucose6phosphate 1epimerase [Chenopodium quinoa]	gi|521702913|gb|GAIA01016469.1|:TSA: Salicornia europaea Unigene17191_Se200S transcribed RNA sequence	A0A0K9R2E5|A0A0K9R2E5_SPIOL:Uncharacterized protein;OS=Spinacia oleracea;|GO:0030246,GO:0016853,|GO:0005975,
DN122397_c	DOWN	-	-	-	-	-	-

geneid	trend	GO	Pathway	TF	NR	NT	Uniprot
4_g3_i4							
DN108771_c0_g1_i1	DOWN	-	-	-	-	-	-
DN84165_c0_g2_i1	DOWN	[GO:0004450:isocitrate dehydrogenase (NADP+) activity][GO:0000287:magnesium ion binding][GO:0051287:NAD binding][GO:0006102:isocitrate metabolic process][GO:0006099:tricarboxylic acid cycle]	[ko00020:Citrate cycle (TCA cycle)][ko00480:Glutathione metabolism][ko00720:Carbon fixation pathways in prokaryotes][ko04146:Peroxisome]	PEQU_16234:Phalaenopsis equestris|G2-like|G2-like family protein	XP_009124158.1:PREDICTED: cytosolic isocitrate dehydrogenase [NADP]like [Brassica rapa]	gi|502122514|ref|XM_004497725.1|:PREDICTED: Cicer arietinum thioredoxin-like protein 4A-like (LOC101500748), mRNA	M4FAT6|M4FAT6_BRARP:Isocitrate dehydrogenase [NADP];OS=Brassica rapa subsp. pekinensis;|GO:0004450,GO:0000287,GO:0051287,|GO:0006102,GO:0006099,
DN118372_c2_g1_i1	DOWN	[GO:0016298:lipase activity][GO:0006629:lipid metabolic process]	-	-	XP_021847433.1:GDSL esterase/lipase 4like [Spinacia oleracea]	-	A0A0K9RV20|A0A0K9RV20_SPIOL:Uncharacterized protein;OS=Spinacia oleracea;|GO:0016298,|GO:0006629,

geneid	trend	GO	Pathway	TF	NR	NT	Uniprot
DN128832_c2_g7_i2	DOWN	[GO:0016021:integral component of membrane][GO:0016020:membrane][GO:0020037:heme binding][GO:0005506:iron ion binding][GO:0016709:oxidoreductase activity, acting on paired donors, with incorporation or reduction of molecular oxygen, NAD(P)H as one donor, and incorporation of one atom of oxygen][GO:0098542:defense response to other organism][GO:0042343:indole glucosinolate metabolic process][GO:0044550:secondary metabolite biosynthetic process]	-	OPUNC10G09700.3:Oryza punctata|E2F/DP|E2F/DP family protein	XP_021839387.1:isoflavone 3'hydroxylaselike [Spinacia oleracea]	-	F6GZP6|F6GZP6_VITVI:Putative uncharacterized protein;OS=Vitis vinifera;GN=VIT_18s0001g09510{ECO:0000313|EMBL:CCB45778.1};|GO:0016021,GO:0016020,|GO:0020037,GO:0005506,GO:0016709,|GO:0098542,GO:0042343,GO:0044550,

geneid	trend	GO	Pathway	TF	NR	NT	Uniprot
DN117320_c0_g1_i4	DOWN	[GO:0005576:extracellular region][GO:0020037:heme binding][GO:0046872:metal ion binding][GO:0004601:peroxidase activity][GO:0042744:hydrogen peroxide catabolic process][GO:0006979:response to oxidative stress]	[ko00940:Phenylpropanoid biosynthesis]	EPS67213.1:Genlisea aurea|NAC|NAC family protein	KNA06553.1:hypothetical protein SOVF_179990 [Spinacia oleracea]	gi|255560567|ref|XM_002521252.1|:Ricinus communis Peroxidase 3 precursor, putative, mRNA	A0A0K9QIM6|A0A0K9QIM6_SPIOL:Peroxidase;OS=Spinacia oleracea;|GO:0005576,|GO:0020037,GO:0046872,GO:0004601,|GO:0042744,GO:0006979,
DN115628_c0_g3_i1	DOWN	-	-	-	-	-	-
DN106000_c0_g1_i1	DOWN	-	-	-	-	-	-
DN124096_c1_g2_i5	DOWN	[GO:0005524:ATP binding][GO:0004672:protein kinase activity]	-	AA10G00404:Aethionema arabicum|WRKY|WRKY family protein	XP_021772357.1:wallassociated receptor kinaselike 1 [Chenopodium quinoa]	-	A0A0J8CMI5|A0A0J8CMI5_BETVU:Uncharacterized protein;OS=Beta vulgaris subsp. vulgaris.;|GO:0005524,GO:0004672,

geneid	trend	GO	Pathway	TF	NR	NT	Uniprot
DN2709_c0_g1_i1	DOWN	[GO:0005524:ATP binding][GO:0004386:helicase activity][GO:0003676:nucleic acid binding]	[ko03018:RNA degradation]	Manes.01G259600.1.p:Manihot esculenta|MYB|MYB family protein	BAJ93616.1:predicted protein [Hordeum vulgare subsp. vulgare]	-	F2DEU5|F2DEU5_HORVD:Predicted protein;OS=Hordeum vulgare var. distichum;|GO:0005524,GO:0004386,GO:0003676,
DN113697_c0_g1_i5	DOWN	[GO:0016757:transferase activity, transferring glycosyl groups]	[ko00052:Galactose metabolism]	-	XP_021734495.1:galactinol synthase 2like [Chenopodium quinoa]	gi|521742038|gb|GAIA01005209.1|:TSA: Salicornia europaea Unigene5498_Se200S transcribed RNA sequence	A0A0J8CQ88|A0A0J8CQ88_BETVU:Hexosyltransferase;OS=Beta vulgaris subsp. vulgaris.;|GO:0016757,
DN128011_c3_g4_i2	DOWN	[GO:0010181:FMN binding][GO:0003955:NAD(P)H dehydrogenase (quinone) activity]	[ko00130:Ubiquinone and other terpenoid-quinone biosynthesis]	-	XP_009132440.1:PREDICTED: NAD(P)H dehydrogenase (quinone) FQR1 [Brassica rapa]	-	M4EJQ9|M4EJQ9_BRARP:Uncharacterized protein;OS=Brassica rapa subsp. pekinensis;|GO:0010181,GO:0003955,

geneid	trend	GO	Pathway	TF	NR	NT	Uniprot
DN120933_c0_g2_i4	DOWN	[GO:0016021:integral component of membrane][GO:0005215:transporter activity][GO:0055085:transmembrane transport]	-	Vradi06g11150.1:Vigna radiata|WRKY|WRKY family protein	AAC19400.1:permease 1 [Mesembryanthemum crystallinum]	gi|3202037|gb|AF069322.1|AF069322:Mesembryanthemum crystallinum clone R30-9 permease 1 mRNA, complete cds	O81339|O81339_MESCR:Permease 1;OS=Mesembryanthemum crystallinum;|GO:0016021,|GO:0005215,|GO:0055085,
DN139584_c0_g1_i1	DOWN	-	-	-	-	-	-

geneid	trend	GO	Pathway	TF	NR	NT	Uniprot
DN128832_c2_g7_i6	DOWN	[GO:0016021:integral component of membrane][GO:0016020:membrane][GO:0020037:heme binding][GO:0005506:iron ion binding][GO:0016709:oxidoreductase activity, acting on paired donors, with incorporation or reduction of molecular oxygen, NAD(P)H as one donor, and incorporation of one atom of oxygen][GO:0098542:defense response to other organism][GO:0042343:indole glucosinolate metabolic process][GO:0044550:secondary metabolite biosynthetic process]	-	Thecc1EG000515t1:Theobroma cacao|B3|B3 family protein	XP_021839387.1:isoflavone 3'hydroxylaselike [Spinacia oleracea]	-	F6GZP6|F6GZP6_VITVI:Putative uncharacterized protein;OS=Vitis vinifera;GN=VIT_18s0001g09510 {ECO:0000313|EMBL:CCB45778.1};|GO:0016021,GO:0016020,|GO:0020037,GO:0005506,GO:0016709,|GO:0098542,GO:0042343,GO:0044550,

geneid	trend	GO	Pathway	TF	NR	NT	Uniprot
DN125096_c0_g4_i2	DOWN	[GO:0000910:cytokinesis][GO:0000226:microtubule cytoskeleton organization]	-	KZV23864.1:Dorcoceras hygrometricum|LBD|LBD family protein	XP_021746610.1:65 kDa microtubuleassociated protein 8like [Chenopodium quinoa]	gi|521563786|gb|GAIA01074661.1|:TSA: Salicornia europaea Unigene90592_Se200S transcribed RNA sequence	A0A0J8EW66|A0A0J8EW66_BETVU: Uncharacterized protein;OS=Beta vulgaris subsp. vulgaris.;|GO:0000910,GO:0000226,
DN121660_c4_g5_i1	DOWN	-	-	-	-	-	-
DN95396_c0_g1_i1	DOWN	[GO:0016020:membrane][GO:0020037:heme binding][GO:0005506:iron ion binding][GO:0016709:oxidoreductase activity, acting on paired donors, with incorporation or reduction of molecular oxygen, NAD(P)H as one donor, and incorporation of one atom of oxygen][GO:0044550:secondary metabolite biosynthetic process]	-	Sme2.5_00297.1_g00019.1:Solanum melongena|MYB|MYB family protein	XP_006358766.1:PREDICTED: cytochrome P450 83B1like [Solanum tuberosum]	-	K4CWT6|K4CWT6_SOLLC:Uncharacterized protein;OS=Solanum lycopersicum;|GO:0016020,|GO:0020037,GO:0005506,GO:0016709,|GO:0044550,
DN191544_c0_g1_i1	DOWN	-	-	-	-	-	-

geneid	trend	GO	Pathway	TF	NR	NT	Uniprot
DN112656_c0_g1_i1	DOWN	[GO:0005576:extracellular region][GO:0020037:heme binding][GO:0046872:metal ion binding][GO:0004601:peroxidase activity][GO:0042744:hydrogen peroxide catabolic process][GO:0006979:response to oxidative stress]	[ko00940:Phenylpropanoid biosynthesis]	EPS67213.1:Genlisea aurea|NAC|NAC family protein	XP_010681225.1:PREDICTED: peroxidase 12 [Beta vulgaris subsp. vulgaris] >KMT19595.1 hypothetical protein BVRB_1g012110 [Beta vulgaris subsp. vulgaris]	gi|1781321|emb|Y10462.1|:S.oleracea mRNA for peroxidase, clone PB11	A0A0J8D0F9|A0A0J8D0F9_BETVU:Peroxidase;OS=Beta vulgaris subsp. vulgaris.;|GO:0005576,|GO:0020037,GO:0046872,GO:0004601,|GO:0042744,GO:0006979,
DN150363_c0_g1_i1	DOWN	[GO:0000786:nucleosome][GO:0005634:nucleus][GO:0003677:DNA binding][GO:0006334:nucleosome assembly]	-	-	KFK23041.1:hypothetical protein AALP_AAs67754U000100, partial [Arabis alpina]	-	A0A087FZI9|A0A087FZI9_ARAAL:Uncharacterized protein;OS=Arabis alpina;|GO:0000786,GO:0005634,|GO:0003677,|GO:0006334,
DN128256_c0_g5_i7	DOWN	-	-	Rsa1.0_00424.1_g00007.1:Raphanus sativus|C2H2|C2H2 family protein	XP_021856570.1:uncharacterized protein LOC110795851 [Spinacia oleracea]	-	A0A0J8BVA4|A0A0J8BVA4_BETVU: Uncharacterized protein;OS=Beta vulgaris subsp. vulgaris.;

geneid	trend	GO	Pathway	TF	NR	NT	Uniprot
DN121617_c1_g2_i3	DOWN	[GO:0030246:carbohydrate binding][GO:0016853:isomerase activity][GO:0005975:carbohydrate metabolic process]	[ko00010:Glycolysis / Gluconeogenesis]	MDP0000239688:Malus domestica|bZIP|bZIP family protein	XP_021757607.1:putative glucose6phosphate 1epimerase [Chenopodium quinoa]	gi|521740951|gb|GAIA01006084.1|:TSA: Salicornia europaea Unigene6393_Se200S transcribed RNA sequence	A0A0K9R2E5|A0A0K9R2E5_SPIOL:Uncharacterized protein;OS=Spinacia oleracea;|GO:0030246,GO:0016853,|GO:0005975,
DN116303_c3_g4_i5	DOWN	-	-	-	-	-	-
DN115628_c0_g4_i1	DOWN	-	-	-	-	-	-
DN118177_c0_g1_i1	DOWN	[GO:0016021:integral component of membrane][GO:0055114:oxidation-reduction process]	-	-	XP_021735209.1:cytochrome b561 and DOMON domaincontaining protein At5g35735like [Chenopodium quinoa]	-	A0A0K9RL58|A0A0K9RL58_SPIOL:Uncharacterized protein;OS=Spinacia oleracea;|GO:0016021,|GO:0055114,

geneid	trend	GO	Pathway	TF	NR	NT	Uniprot
DN116764_c0_g4_i11	DOWN	[GO:0005576:extracellular region][GO:0020037:heme binding][GO:0046872:metal ion binding][GO:0004601:peroxidase activity][GO:0042744:hydrogen peroxide catabolic process][GO:0006979:response to oxidative stress]	[ko00940:Phenylpropanoid biosynthesis]	EPS67213.1:Genlisea aurea|NAC|NAC family protein	XP_021714056.1:peroxidase 64like [Chenopodium quinoa]	gi|521578430|gb|GAIA01069743.1|:TSA: Salicornia europaea Unigene84004_Se200S transcribed RNA sequence	A0A0K9QS66|A0A0K9QS66_SPIOL:Peroxidase;OS=Spinacia oleracea;|GO:0005576,|GO:0020037,GO:0046872,GO:0004601,|GO:0042744,GO:0006979,
DN120989_c1_g4_i1	DOWN	[GO:0004553:hydrolase activity, hydrolyzing O-glycosyl compounds][GO:0005975:carbohydrate metabolic process]	-	-	KNA18307.1:hypothetical protein SOVF_072030 [Spinacia oleracea]	gi|21322513|emb|AJ422052.1|:Beta vulgaris mRNA for exocellular acid invertase 1 (exinv1 gene)	A0A0K9RHI7|A0A0K9RHI7_SPIOL:Uncharacterized protein;OS=Spinacia oleracea;|GO:0004553,|GO:0005975,
DN121774_c3_g3_i3	DOWN	[GO:0010181:FMN binding][GO:0003955:NAD(P)H dehydrogenase (quinone) activity]	[ko00130:Ubiquinone and other terpenoid-quinone biosynthesis]	-	XP_010694306.1:PREDICTED: NAD(P)H dehydrogenase (quinone) FQR1 isoform X1 [Beta vulgaris subsp. vulgaris]	-	A0A0J8BEX6|A0A0J8BEX6_BETVU:Uncharacterized protein;OS=Beta vulgaris subsp. vulgaris.;|GO:0010181,GO:0003955,

geneid	trend	GO	Pathway	TF	NR	NT	Uniprot
DN127115_c2_g2_i1	DOWN	[GO:0005576:extracellular region][GO:0005509:calcium ion binding][GO:0004623:phospholipase A2 activity][GO:0050482:arachidonic acid secretion][GO:0016042:lipid catabolic process][GO:0006644:phospholipid metabolic process]	[ko00564:Glycerophospholipid metabolism][ko00565:Ether lipid metabolism][ko00590:Arachidonic acid metabolism][ko00591:Linoleic acid metabolism][ko00592:alpha-Linolenic acid metabolism][ko04014:Ras signaling pathway][ko04270: Vascular smooth muscle contraction][ko04972:Pancreatic secretion][ko04975: Fat digestion and absorption]	-	XP_011017752.1:PREDICTED: phospholipase A2alphalike [Populus euphratica]	gi|32454819|gb|AY319966.1|:Mirabilis jalapa phospholipase A2 mRNA, partial cds	A9PB93|A9PB93_POPTR:Uncharacterized protein;OS=Populus trichocarpa;|GO:0005576,|GO:0005509, GO:0004623,|GO:0050482,GO:0016042, GO:0006644,

geneid	trend	GO	Pathway	TF	NR	NT	Uniprot
DN125485_c0_g3_i3	DOWN	[GO:0097008:(3E)-4,8-dimethyl-1,3,7-nonatriene synthase activity][GO:0097007:4,8,12-trimethyltrideca-1,3,7,11-tetraene synthase activity][GO:0020037:heme binding][GO:0005506:iron ion binding][GO:0046246:terpene biosynthetic process]	[ko00904:Diterpenoid biosynthesis]	Cc10_g03650:Coffea canephora|G2-like|G2-like family protein	XP_010696237.1:PREDICTED: cytochrome P450 82G1 [Beta vulgaris subsp. vulgaris] >KMS97083.1 hypothetical protein BVRB_7g178650 [Beta vulgaris subsp. vulgaris]	gi|521441879|gb|GAHZ01078605.1|:TSA: Salicornia europaea Unigene96237_SeCKS transcribed RNA sequence	A0A0J8BAT5|A0A0J8BAT5_BETVU:Uncharacterized protein;OS=Beta vulgaris subsp. vulgaris.;|GO:0097008,GO:0097007,GO:0020037,GO:0005506,|GO:0046246,
DN124134_c3_g8_i6	DOWN	[GO:0016491:oxidoreductase activity][GO:0008270:zinc ion binding]	[ko00010:Glycolysis / Gluconeogenesis][ko00071:Fatty acid degradation][ko00350:Tyrosine metabolism][ko00592:alpha-Linolenic acid metabolism]	-	AAP96921.1:alcohol dehydrogenase [Dianthus caryophyllus]	gi|283826826|gb|GU233768.1|:Mangifera indica alcohol dehydrogenase 3 mRNA, complete cds	Q5QJY8|Q5QJY8_DIACA:Alcohol dehydrogenase;OS=Dianthus caryophyllus;|GO:0016491,GO:0008270,
DN121338_c7_g10_i1	DOWN	[GO:0008234:cysteine-type peptidase activity]	-	-	XP_021836618.1:uncharacterized protein LOC110776370 [Spinacia oleracea]	-	M5XWE4|M5XWE4_PRUPE:Uncharacterized protein;OS=Prunus persica;|GO:000823

geneid	trend	GO	Pathway	TF	NR	NT	Uniprot
							4,
DN128300_c4_g6_i1	DOWN	[GO:0016758:transferase activity, transferring hexosyl groups][GO:0008152:metabolic process]	-	-	XP_021735840.1:UDPglycosyltransferase 79B30like [Chenopodium quinoa]	-	A0A0K9RWT5|A0A0K9RWT5_SPIOL:Glycosyltransferase;OS=Spinacia oleracea;|GO:0016758,|GO:0008152,
DN116022_c2_g5_i2	DOWN	-	-	-	XP_010670795.1:PREDICTED: uncharacterized protein LOC104887762 isoform X2 [Beta vulgaris subsp. vulgaris] >KMT16921.1 hypothetical protein BVRB_2g043930 [Beta vulgaris subsp. vulgaris]	-	A0A0J8FM29|A0A0J8FM29_BETVU:Uncharacterized protein;OS=Beta vulgaris subsp. vulgaris.;
DN19398_c0_g1_i1	DOWN	-	-	-	-	-	-

geneid	trend	GO	Pathway	TF	NR	NT	Uniprot
DN90169_c0_g1_i1	DOWN	[GO:0000786:nucleosome][GO:0005634:nucleus][GO:0003677:DNA binding]	[ko05034:Alcoholism][ko05203:Viral carcinogenesis][ko05322:Systemic lupus erythematosus]	-	XP_006665103.1:PREDICTED: histone H2B [Oryza brachyantha] >XP_019435463.1 PREDICTED: histone H2B [Lupinus angustifolius] >KFK22720.1 hypothetical protein AALP_AAs74787U000100 [Arabis alpina]	gi|410927065|ref|XM_003976938.1|:PREDICTED: Takifugu rubripes histone H2B 1/2-like (LOC101068558), mRNA	A0A087FYL8|A0A087FYL8_ARAAL:Histone H2B;OS=Arabis alpina;|GO:0000786,GO:0005634,|GO:0003677,
DN115174_c6_g4_i3	DOWN	[GO:0005634:nucleus][GO:0003677:DNA binding][GO:0006355:regulation of transcription, DNA-templated][GO:0006351:transcription, DNA-templated]	-	Dca51258.1:Dianthus caryophyllus|NAC|NAC family protein	XP_008386130.1:PREDICTED: NAC transcription factor 56like [Malus domestica]	gi|350528640|gb|JN675710.1|:Prunus salicina NAC domain class transcription factor mRNA, complete cds	A0A0M4FP05|A0A0M4FP05_MANES:NAC transcription factors 21;OS=Manihot esculenta;|GO:0005634,|GO:0003677,|GO:0006355,GO:0006351,
DN105810_c3_g3_i1	DOWN	-	-	-	-	-	-

geneid	trend	GO	Pathway	TF	NR	NT	Uniprot
DN122397_c5_g2_i14	DOWN	-	-	-	-	-	-
DN115597_c0_g1_i5	DOWN	-	-	-	XP_021726506.1:uncharacterized protein LOC110693664 [Chenopodium quinoa]	-	A0A0J8BX80|A0A0J8BX80_BETVU:Uncharacterized protein;OS=Beta vulgaris subsp. vulgaris.;
DN82637_c0_g1_i1	DOWN	[GO:0000287:magnesium ion binding][GO:0010333:terpene synthase activity][GO:0008152:metabolic process]	[ko00902:Monoterpenoid biosynthesis]	MDP0000245233:Malus domestica|bHLH|bHLH family protein	ADR74212.1:(3S)linalool/(E)nerolidol synthase [Vitis vinifera]	-	F6I7G1|F6I7G1_VITVI:Putative uncharacterized protein;OS=Vitis vinifera;|GO:0000287,GO:0010333,|GO:0008152,
DN107831_c0_g3_i1	DOWN	-	[ko04624:Toll and Imd signaling pathway][ko05205:Proteoglycans in cancer]	-	-	gi|241264283|ref|XM_002405743.1|:Ixodes scapularis ankyrin 2,3/unc44, putative, mRNA	-
DN111221_c0_g1_i1	DOWN	-	-	-	-	-	-
DN122397_c5_g6_i1	DOWN	-	-	-	-	-	-

geneid	trend	GO	Pathway	TF	NR	NT	Uniprot
DN118915_c2_g2_i2	DOWN	[GO:0016021:integral component of membrane]	-	Sp_073850_ixmz.t1:Spinacia oleracea|MYB_related|MYB_related family protein	XP_021772597.1:amino acid permease 4like [Chenopodium quinoa]	gi|470109233|ref|XM_004290857.1|:PREDICTED: Fragaria vesca subsp. vesca amino acid permease 2-like (LOC101293061), mRNA	A0A0J8CPU4|A0A0J8CPU4_BETVU:Uncharacterized protein;OS=Beta vulgaris subsp. vulgaris.;|GO:0016021,
DN126220_c1_g8_i6	DOWN	-	-	-	-	-	-
DN102310_c0_g1_i1	DOWN	-	-	-	XP_010669448.1:PREDICTED: floweringpromoting factor 1like protein 2 [Beta vulgaris subsp. vulgaris] >KMT17728.1 hypothetical protein BVRB_2g034800 [Beta vulgaris subsp. vulgaris]	-	A0A0J8D0R9|A0A0J8D0R9_BETVU:Uncharacterized protein;OS=Beta vulgaris subsp. vulgaris.;
DN117062_c4_g3_i4	DOWN	-	-	-	-	-	-
DN177011_c	DOWN	-	-	-	-	-	-

geneid	trend	GO	Pathway	TF	NR	NT	Uniprot
0_g1_i1							
DN82724_c0_g1_i1	DOWN	-	-	-	-	-	-
DN92479_c0_g1_i1	DOWN	-	-	-	-	-	-
DN121332_c4_g3_i1	DOWN	-	-	-	-	-	-
DN119427_c0_g1_i8	DOWN	[GO:0016021:integral component of membrane][GO:0055085:transmembrane transport]	-	Jcr4S00206.70:Jatropha curcas|FAR1|FAR1 family protein	XP_010680628.1:PREDICTED: protein PINLIKES 7 isoform X1 [Beta vulgaris subsp. vulgaris] >XP_010680629.1 PREDICTED: protein PINLIKES 7 isoform X1 [Beta vulgaris subsp. vulgaris] >XP_019105728.1 PREDICTED: protein PINLIKES 7 isoform X1 [Beta	-	A0A0J8C515|A0A0J8C515_BETVU:Uncharacterized protein;OS=Beta vulgaris subsp. vulgaris.;|GO:0016021,|GO:0055085,

geneid	trend	GO	Pathway	TF	NR	NT	Uniprot
					vulgaris subsp. vulgaris] >KMT08875.1 hypothetical protein BVRB_6g135980 [Beta vulgaris subsp. vulgaris]		
DN125710_c2_g3_i6	DOWN	[GO:0009570:chloroplast stroma][GO:0016597:amino acid binding][GO:0047769:arogenate dehydratase activity][GO:0004664:prephenate dehydratase activity][GO:0009094:L-phenylalanine biosynthetic process]	[ko00400:Phenylalanine, tyrosine and tryptophan biosynthesis]	-	XP_010686824.1:PREDICTED: arogenate dehydratase/prephenate dehydratase 1, chloroplastic [Beta vulgaris subsp. vulgaris] >KMT04155.1	gi|521703531|gb|GAIA01016159.1|:TSA: Salicornia europaea Unigene16867_Se200S transcribed RNA sequence	A0A0J8BRM3|A0A0J8BRM3_BETVU: Arogenate dehydratase;OS=Beta vulgaris subsp. vulgaris.;|GO:0009570,|GO:0016597,GO:0047769,GO:0004664,|GO:0009094,

geneid	trend	GO	Pathway	TF	NR	NT	Uniprot
					hypothetical protein BVRB_8g186040 [Beta vulgaris subsp. vulgaris]		
DN109965_c0_g1_i1	DOWN	-	-	-	-	-	-
DN120768_c0_g2_i13	DOWN	[GO:0016021:integral component of membrane][GO:0008271:secondary active sulfate transmembrane transporter activity]	-	GSMUA_Achr9P13010_001:Musa acuminata|MIKC_MADS|MIKC_MADS family protein	XP_021723678.1:probable sulfate transporter 3.5 [Chenopodium quinoa]	-	A0A0J8EJ94|A0A0J8EJ94_BETVU:Uncharacterized protein;OS=Beta vulgaris subsp. vulgaris.;|GO:0016021,|GO:0008271,
DN72278_c0_g1_i1	DOWN	-	[ko03010:Ribosome]	-	XP_008775991.1:PREDICTED: 60S ribosomal protein L182 [Phoenix dactylifera]	-	A0A1D1ZF53|A0A1D1ZF53_9ARAE:60S ribosomal protein L18;OS=Anthurium amnicola.;

geneid	trend	GO	Pathway	TF	NR	NT	Uniprot
DN43497_c0_g1_i1	DOWN	[GO:0016491:oxidoreductase activity]	[ko00120:Primary bile acid biosynthesis][ko04146:Peroxisome]	-	BAJ94619.1:predicted protein [Hordeum vulgare subsp. vulgare]	-	F2DHP8|F2DHP8_HORVD:Predicted protein;OS=Hordeum vulgare var. distichum;|GO:0016491,
DN123972_c0_g1_i2	DOWN	[GO:0005634:nucleus][GO:0043565:sequence-specific DNA binding][GO:0003700:DNA-binding transcription factor activity]	-	Sp_179660_yhcr.t1:Spinacia oleracea|HD-ZIP|HD-ZIP family protein	XP_021835736.1:homeoboxleucine zipper protein HOX11like [Spinacia oleracea] >KNA06584.1 hypothetical protein SOVF_179660 [Spinacia oleracea]	gi|521742989|gb|GAIA01004673.1|:TSA: Salicornia europaea Unigene4957_Se200S transcribed RNA sequence	A0A0K9QH04|A0A0K9QH04_SPIOL:Uncharacterized protein;OS=Spinacia oleracea;|GO:0005634,|GO:0043565,GO:0003700,

geneid	trend	GO	Pathway	TF	NR	NT	Uniprot
DN125485_c0_g3_i1	DOWN	[GO:0097008:(3E)-4,8-dimethyl-1,3,7-nonatriene synthase activity][GO:0097007:4,8,12-trimethyltrideca-1,3,7,11-tetraene synthase activity][GO:0020037:heme binding][GO:0005506:iron ion binding][GO:0046246:terpene biosynthetic process]	[ko00904:Diterpenoid biosynthesis]	Cc10_g03650:Coffea canephora|G2-like|G2-like family protein	XP_010696237.1:PREDICTED: cytochrome P450 82G1 [Beta vulgaris subsp. vulgaris] >KMS97083.1 hypothetical protein BVRB_7g178650 [Beta vulgaris subsp. vulgaris]	gi|199580141|gb|AC189446.2|:Brassica rapa subsp. pekinensis clone KBrB070M16, complete sequence	A0A0J8BAT5|A0A0J8BAT5_BETVU:Uncharacterized protein;OS=Beta vulgaris subsp. vulgaris.;|GO:0097008,GO:0097007,GO:0020037,GO:0005506,|GO:0046246,
DN128676_c0_g10_i1	DOWN	-	-	-	-	-	-
DN117097_c0_g1_i3	DOWN	-	-	-	-	-	-
DN122681_c1_g1_i14	DOWN	[GO:0006511:ubiquitin-dependent protein catabolic process]	-	-	XP_021767088.1:SKP1like protein 21 isoform X1 [Chenopodium quinoa]	gi|521621825|gb|GAIA01054876.1|:TSA: Salicornia europaea Unigene64532_Se200S transcribed RNA sequence	A0A0K9RIZ6|A0A0K9RIZ6_SPIOL:Uncharacterized protein;OS=Spinacia oleracea;|GO:0006511,

geneid	trend	GO	Pathway	TF	NR	NT	Uniprot
DN60389_c0_g1_i1	DOWN	[GO:0004128:cytochrome-b5 reductase activity, acting on NAD(P)H]	[ko00520:Amino sugar and nucleotide sugar metabolism]	mrna31001.1-v1.0-hybrid:Fragaria vesca|C3H|C3H family protein	BAA13047.1:nitrate reductase [Spinacia oleracea]	-	H3G7G2|H3G7G2_PHYRM:NADH-cytochrome b5 reductase;OS=Phytophthora ramorum;|GO:0004128,
DN123446_c1_g1_i1	DOWN	[GO:0070531:BRCA1-A complex][GO:0070552:BRISC complex][GO:0004843:thiol-dependent ubiquitin-specific protease activity][GO:0006281:DNA repair][GO:0070536:protein K63-linked deubiquitination]	[ko03440:Homologous recombination][ko04621:NOD-like receptor signaling pathway]	-	XP_021731875.1:uncharacterized protein LOC110698682 [Chenopodium quinoa]	-	A0A0J8BKR4|A0A0J8BKR4_BETVU: Uncharacterized protein;OS=Beta vulgaris subsp. vulgaris.;|GO:0070531,GO:0070552,|GO:0004843,|GO:0006281,GO:0070536,
DN105055_c0_g1_i1	DOWN	-	-	-	-	-	-

geneid	trend	GO	Pathway	TF	NR	NT	Uniprot
DN56166_c0_g1_i1	DOWN	[GO:0005829:cytosol][GO:0015935:small ribosomal subunit][GO:0003723:RNA binding][GO:0003735:structural constituent of ribosome][GO:0042254:ribosome biogenesis][GO:0006412:translation]	[ko03010:Ribosome]	Achn386081:Actinidia chinensis|Dof|Dof family protein	XP_002306780.1:hypothetical protein POPTR_0005s23280g [Populus trichocarpa] >XP_011032690.1 PREDICTED: 40S ribosomal protein S18like [Populus euphratica] >ABK92692.1 unknown [Populus trichocarpa] >EEE93776.1 hypothetical protein POPTR_0005s23280g [Populus trichocarpa]	gi|159145773|gb|EU116929.1|:Barentsia elongata putative ribosomal protein S18 mRNA, complete cds	A9P8I9|A9P8I9_POPTR:Uncharacterized protein;OS=Populus trichocarpa;|GO:0005829,GO:0015935,|GO:0003723,GO:0003735,|GO:0042254,GO:0006412,

geneid	trend	GO	Pathway	TF	NR	NT	Uniprot
DN123094_c1_g1_i1	DOWN	[GO:0016021:integral component of membrane][GO:0055114:oxidation-reduction process]	-	678320713:Utricularia gibba|M-type_MADS|M-type_MADS family protein	XP_010679032.1:PREDICTED: cytochrome b561 and DOMON domaincontaining protein At3g07570 [Beta vulgaris subsp. vulgaris] >KMT10155.1 hypothetical protein BVRB_5g119160 [Beta vulgaris subsp. vulgaris]	-	A0A0J8C8X5|A0A0J8C8X5_BETVU:Uncharacterized protein;OS=Beta vulgaris subsp. vulgaris.;|GO:0016021,|GO:0055114,
DN128819_c2_g1_i2	DOWN	-	-	LOC_Os05g25600.1:Oryza sativa subsp. japonica|HB-other|HB-other family protein	XP_019103204.1:PREDICTED: serine/threonineprotein phosphatase 7 long form homolog [Beta vulgaris subsp. vulgaris]	-	V4RXX8|V4RXX8_9ROSI:Uncharacterized protein;OS=Citrus clementina.;
DN127723_c8_g11_i1	DOWN	-	-	-	-	-	-
DN114523_c1_g1_i8	DOWN	-	-	-	-	-	-

geneid	trend	GO	Pathway	TF	NR	NT	Uniprot
DN125020_c3_g1_i1	DOWN	-	-	-	-	-	-
DN124178_c5_g1_i1	DOWN	[GO:0003677:DNA binding]	-	Bv5_121120_roao.t1:Beta vulgaris|MYB|MYB family protein	XP_010679296.1:PREDICTED: mybrelated protein Myb4 [Beta vulgaris subsp. vulgaris] >KMT09893.1 hypothetical protein BVRB_5g121120 [Beta vulgaris subsp. vulgaris]	-	A0A0J8CD61|A0A0J8CD61_BETVU:Uncharacterized protein;OS=Beta vulgaris subsp. vulgaris.;|GO:0003677,
DN69058_c0_g1_i1	DOWN	-	[ko05202:Transcriptional misregulation in cancer]	-	BAK06609.1:predicted protein [Hordeum vulgare subsp. vulgare]	-	A0A1D1YVQ8|A0A1D1YVQ8_9ARAE:Mitochondrial-processing peptidase subunit beta;OS=Anthurium amnicola.;

geneid	trend	GO	Pathway	TF	NR	NT	Uniprot
DN71768_c0_g1_i1	DOWN	-	[ko05134:Legionellosis]	-	XP_001774148.1:predicted protein [Physcomitrella patens] >EDQ61062.1 predicted protein [Physcomitrella patens] >AFZ39149.1 EF1Bgamma class glutathione Stransferase [Physcomitrella patens]	-	A0A1D1XTP4|A0A1D1XTP4_9ARAE: Elongation factor 1-gamma;OS=Anthurium amnicola.;
DN116223_c0_g1_i2	DOWN	[GO:0016021:integral component of membrane][GO:0022857:transmembrane transporter activity]	-	Glyma.13G227100.1.p:Glycine max|ERF|ERF family protein	XP_010687695.1:PREDICTED: WAT1related protein At4g30420 [Beta vulgaris subsp. vulgaris] >KMT03534.1 hypothetical protein BVRB_8g192110 [Beta vulgaris subsp. vulgaris]	-	A0A0J8BU94|A0A0J8BU94_BETVU:WAT1-related protein;OS=Beta vulgaris subsp. vulgaris.;|GO:0016021,|GO:0022857,

geneid	trend	GO	Pathway	TF	NR	NT	Uniprot
DN121857_c1_g1_i5	DOWN	[GO:0005789:endoplasmic reticulum membrane][GO:0016021:integral component of membrane][GO:0005778:peroxisomal membrane][GO:0004420:hydroxymethylglutaryl-CoA reductase (NADPH) activity][GO:0050661:NADP binding][GO:0015936:coenzyme A metabolic process][GO:0008299:isoprenoid biosynthetic process][GO:0016126:sterol biosynthetic process]	[ko00900:Terpenoid backbone biosynthesis][ko04152:AMPK signaling pathway][ko04976: Bile secretion]	RrC14701_p1:Raphanus raphanistrum|HSF|HSF family protein	XP_010270571.1:PREDICTED: 3hydroxy3methylglutarylcoenzyme A reductase 1 [Nelumbo nucifera]	gi|356577270|ref|XM_003556703.1|:PREDICTED: Glycine max 3-hydroxy-3-methylglutaryl-coenzyme A reductase 1-like (LOC100779932), mRNA	I1NDS3|I1NDS3_SOYBN:3-hydroxy-3-methylglutaryl coenzyme A reductase;OS=Glycine max;|GO:0005789,GO:0016021,GO:0005778,|GO:0004420,GO:0050661,|GO:0015936,GO:0008299,GO:0016126,
DN120395_c0_g1_i4	DOWN	-	-	-	-	-	-

geneid	trend	GO	Pathway	TF	NR	NT	Uniprot
DN127565_c1_g1_i1	DOWN	[GO:0051213:dioxygenase activity][GO:0008198:ferrous iron binding][GO:0016701:oxidoreductase activity, acting on single donors with incorporation of molecular oxygen][GO:0008270:zinc ion binding][GO:0006725:cellular aromatic compound metabolic process]	[ko00965:Betalain biosynthesis]	Tp57577_TGAC_v2_mRNA17191:Trifolium pratense|ZF-HD|ZF-HD family protein	AKI33627.1:4,5dioxygenaselike protein, partial [Bougainvillea stipitata]	-	A0A0G2UQN4|A0A0G2UQN4_9CARY:4,5-dioxygenase-like protein;OS=Bougainvillea stipitata.;|GO:0051213,GO:0008198,GO:0016701,GO:0008270,|GO:0006725,
DN117530_c3_g7_i1	DOWN	[GO:0005634:nucleus][GO:0003677:DNA binding]	-	augustus_masked-scaffold03373-abinit-gene-0.2-mRNA-1:Castanea mollissima|WOX|WOX family protein	XP_009360081.1:PREDICTED: WUSCHELrelated homeobox 1like [Pyrus x bretschneideri]	gi|449461024|ref|XM_004148196.1|:PREDICTED: Cucumis sativus WUSCHEL-related homeobox 3-like (LOC101220383), mRNA >gi|449522912|ref|XM_004168422.1| PREDICTED: Cucumis sativus WUSCHEL-related	A0A0B2NSP2|A0A0B2NSP2_GLYSO:WUSCHEL-related homeobox 1;OS=Glycine soja;|GO:0005634,|GO:0003677,

geneid	trend	GO	Pathway	TF	NR	NT	Uniprot
						homeobox 3-like (LOC101231771), mRNA	

geneid	trend	GO	Pathway	TF	NR	NT	Uniprot
DN13234_c0_g1_i1	DOWN	[GO:0005524:ATP binding]	[ko04015:Rap1 signaling pathway][ko04145:Phagosome][ko04210:Apoptosis][ko04390:Hippo signaling pathway][ko04391:Hippo signaling pathway - fly][ko04510:Focal adhesion][ko04520:Adherens junction][ko04530:Tight junction][ko04611:Platelet activation][ko04670:Leukocyte transendothelial migration][ko04745:Phototransduction - fly][ko04810:Regulation of actin cytoskeleton][ko04919:Thyroid hormone signaling pathway][ko04921:Oxytocin signaling pathway][ko05100:Bacterial invasion of epithelial	Niben101Scf00646g07008.1:Nicotiana benthamiana|Trihelix|Trihelix family protein	BAJ90061.1:predicted protein [Hordeum vulgare subsp. vulgare]	gi|512423761|gb|GAJW01000209.1|:TSA: Aphis craccivora Aphis_trimmed_contig_212 transcribed RNA sequence	F2D4P0|F2D4P0_HORVD:Predicted protein;OS=Hordeum vulgare var. distichum;|GO:0005524,

geneid	trend	GO	Pathway	TF	NR	NT	Uniprot
DN118105_c1_g3_i4	DOWN	-	[ko00561:Glycerolipid metabolism][ko04723:Retrograde endocannabinoid signaling][ko04923: Regulation of lipolysis in adipocytes]	Lus10033562:Linum usitatissimum|bHLH|bHLH family protein	XP_010693140.1:PREDICTED: caffeoylshikimate esterase [Beta vulgaris subsp. vulgaris] >KMS99305.1 hypothetical protein BVRB_2g046090 [Beta vulgaris subsp. vulgaris]	-	A0A0J8E853|A0A0J8E853_BETVU:Uncharacterized protein;OS=Beta vulgaris subsp. vulgaris.;
DN128100_c5_g1_i1	DOWN	[GO:0050660:flavin adenine dinucleotide binding][GO:0016614:oxidoreductase activity, acting on CH-OH group of donors]	-	FANhyb_rscf00000362.1.g00002.1:Fragaria x ananassa|CPP|CPP family protein	XP_021758284.1:berberine bridge enzymelike 8 [Chenopodium quinoa]	-	A5AHZ3|A5AHZ3_VITVI:Putative uncharacterized protein;OS=Vitis vinifera;|GO:0050660,GO:0016614,

geneid	trend	GO	Pathway	TF	NR	NT	Uniprot
DN127565_c4_g5_i1	DOWN	[GO:0005737:cytoplasm][GO:0005874:microtubule][GO:0005815:microtubule organizing center][GO:0009506:plasmodesma][GO:0000922:spindle pole][GO:0043622:cortical microtubule organization][GO:0040007:growth][GO:0007020:microtubule nucleation][GO:0009624:response to nematode]	-	-	XP_010684379.1:PREDICTED: gammatubulin complex component 4 [Beta vulgaris subsp. vulgaris] >XP_010684381.1 PREDICTED: gammatubulin complex component 4 [Beta vulgaris subsp. vulgaris] >KMT06059.1 hypothetical protein BVRB_7g163840 [Beta vulgaris subsp. vulgaris]	gi|521529342|gb|GAHZ01008924.1|:TSA: Salicornia europaea Unigene9301_SeCKS transcribed RNA sequence	A0A0J8C176|A0A0J8C176_BETVU:Gamma-tubulin complex component;OS=Beta vulgaris subsp. vulgaris.;|GO:0005737,GO:0005874,GO:0005815,GO:0009506,GO:0000922,|GO:0043622,GO:0040007,GO:0007020,GO:0009624,

geneid	trend	GO	Pathway	TF	NR	NT	Uniprot
DN214306_c0_g1_i1	DOWN	-	-	Sme2.5_00010.1_g00004.1:Solanum melongena|HSF|HSF family protein	XP_002977832.1:hypothetical protein SELMODRAFT_107557 [Selaginella moellendorffii] >XP_002979493.1 hypothetical protein SELMODRAFT_233374 [Selaginella moellendorffii] >EFJ19382.1 hypothetical protein SELMODRAFT_233374 [Selaginella moellendorffii] >EFJ21170.1 hypothetical protein SELMODRAFT_107557 [Selaginella moellendorffii]	gi|219815475|gb|FJ534553.1|:Tyrophagus putrescentiae troponin C mRNA, complete cds	A0A1D1ZDP5|A0A1D1ZDP5_9ARAE: Troponin C, isoform 3;OS=Anthurium amnicola.;
DN106935_c0_g1_i2	DOWN	-	-	-	-	-	-
DN123200_c1_g3_i2	DOWN	-	-	-	-	-	-

geneid	trend	GO	Pathway	TF	NR	NT	Uniprot
DN88771_c0_g2_i1	DOWN	-	[ko03010:Ribosome]	MDP0000259898:Malus domestica|MYB|MYB family protein	KXZ43313.1:hypothetical protein GPECTOR_94g635 [Gonium pectorale]	-	A0A1D1XMF7|A0A1D1XMF7_9ARAE:40S ribosomal protein S7;OS=Anthurium amnicola.;
DN147108_c0_g1_i1	DOWN	-	-	-	-	-	-
DN126048_c2_g2_i1	DOWN	[GO:0005524:ATP binding][GO:0004672:protein kinase activity]	-	AA10G00404:Aethionema arabicum|WRKY|WRKY family protein	XP_021860590.1:wallassociated receptor kinaselike 2 [Spinacia oleracea] >KNA06178.1 hypothetical protein SOVF_183470 [Spinacia oleracea]	-	A0A0K9QHM3|A0A0K9QHM3_SPIOL:Uncharacterized protein;OS=Spinacia oleracea;|GO:0005524,GO:0004672,

geneid	trend	GO	Pathway	TF	NR	NT	Uniprot
DN122672_c0_g1_i2	DOWN	-	-	Aqcoe2G051500.1.p:Aquilegia coerulea|WRKY|WRKY family protein	XP_021853062.1:uncharacterized protein LOC110792551 [Spinacia oleracea] >XP_021853063.1 uncharacterized protein LOC110792551 [Spinacia oleracea] >XP_021853064.1 uncharacterized protein LOC110792551 [Spinacia oleracea] >XP_021853065.1 uncharacterized protein LOC110792551 [Spinacia oleracea] >XP_021853066.1 uncharacterized protein LOC110792551 [Spinacia oleracea] >XP_021853067.1 uncharacterized protein LOC110792551 [Spinacia oleracea] >XP_021853068.1 uncharacterized	-	A0A0K9RG10|A0A0K9RG10_SPIOL:Uncharacterized protein;OS=Spinacia oleracea;

geneid	trend	GO	Pathway	TF	NR	NT	Uniprot
DN191328_c0_g1_i1	DOWN	[GO:0005615:extracellular space][GO:0004867:serine-type endopeptidase inhibitor activity]	[ko05146:Amoebiasis]	-	XP_011021086.1:PREDICTED: serpinZXlike [Populus euphratica]	-	B9N6P6|B9N6P6_POPTR:Uncharacterized protein;OS=Populus trichocarpa;|GO:0005615,|GO:0004867,
DN123966_c0_g2_i1	DOWN	-	-	Sme2.5_03593.1_g00002.1:Solanum melongena|C2H2|C2H2 family protein	XP_009802111.1:PREDICTED: uncharacterized protein LOC104247724 [Nicotiana sylvestris]	-	Q6L4B3|Q6L4B3_SOLDE:Polyprotein, putative;OS=Solanum demissum;
DN115248_c0_g1_i2	DOWN	[GO:0005576:extracellular region][GO:0020037:heme binding][GO:0046872:metal ion binding][GO:0004601:peroxidase activity][GO:0042744:hydrogen peroxide catabolic process][GO:0006979:response to oxidative stress]	[ko00940:Phenylpropanoid biosynthesis]	EPS67213.1:Genlisea aurea|NAC|NAC family protein	XP_021714435.1:peroxidase 50like [Chenopodium quinoa]	gi|4138646|emb|AJ011939.1|:Trifolium repens mRNA for peroxidase	A0A0J8CCY2|A0A0J8CCY2_BETVU: Peroxidase;OS=Beta vulgaris subsp. vulgaris.;|GO:0005576,|GO:0020037,GO:0046872,GO:0004601,|GO:0042744,GO:0006979,

geneid	trend	GO	Pathway	TF	NR	NT	Uniprot
DN87585_c0_g1_i1	DOWN	-	-	-	-	-	-
DN119783_c3_g4_i5	DOWN	-	-	-	KNA19324.1:hypothetical protein SOVF_062580 isoform B [Spinacia oleracea]	-	A0A0K9RK78|A0A0K9RK78_SPIOL:Uncharacterized protein;OS=Spinacia oleracea;
DN115170_c0_g1_i1	DOWN	[GO:0004843:thiol-dependent ubiquitin-specific protease activity][GO:0016579:protein deubiquitination]	[ko04622:RIG-I-like receptor signaling pathway]	-	XP_018678099.1:PREDICTED: OTU domaincontaining protein 5 isoform X2 [Musa acuminata subsp. malaccensis] >XP_018678100.1 PREDICTED: OTU domaincontaining protein 5 isoform X2 [Musa acuminata subsp. malaccensis]	-	M0S9N0|M0S9N0_MUSAM:Uncharacterized protein;OS=Musa acuminata subsp. malaccensis;|GO:0004843,|GO:0016579,

geneid	trend	GO	Pathway	TF	NR	NT	Uniprot
DN120933_c0_g2_i1	DOWN	[GO:0016021:integral component of membrane][GO:0005215:transporter activity][GO:0055085:transmembrane transport]	-	Vradi06g11150.1:Vigna radiata|WRKY|WRKY family protein	XP_010691555.1:PREDICTED: nucleobaseascorbate transporter 4 [Beta vulgaris subsp. vulgaris] >KMT01024.1 hypothetical protein BVRB_9g222860 [Beta vulgaris subsp. vulgaris]	gi|3202037|gb|AF069322.1|AF069322:Mesembryanthemum crystallinum clone R30-9 permease 1 mRNA, complete cds	A0A0J8BM02|A0A0J8BM02_BETVU: Uncharacterized protein;OS=Beta vulgaris subsp. vulgaris.;|GO:0016021,|GO:0005215,|GO:0055085,
DN106303_c0_g1_i1	DOWN	[GO:0005524:ATP binding][GO:0009374:biotin binding][GO:0004075:biotin carboxylase activity][GO:0003677:DNA binding][GO:0016829:lyase activity][GO:0046872:metal ion binding][GO:0004736:pyruvate carboxylase activity][GO:0006094:gluconeogenesis][GO:0006090:pyruvate metabolic process]	[ko00020:Citrate cycle (TCA cycle)][ko00620:Pyruvate metabolism][ko00720:Carbon fixation pathways in prokaryotes]	-	XP_002503347.1:pyruvate carboyxlase [Micromonas commoda] >ACO64605.1 pyruvate carboyxlase [Micromonas commoda]	gi|195359263|ref|XM_002045296.1|:Drosophila sechellia GM24372 (Dsec\GM24372), mRNA	A0A0A0VAT8|A0A0A0VAT8_SACJA: Pyruvate carboxylase;OS=Saccharina japonica;|GO:0005524,GO:0009374,GO:0004075,GO:0003677,GO:0016829,GO:0046872,GO:0004736,|GO:0006094,GO:0006090,

geneid	trend	GO	Pathway	TF	NR	NT	Uniprot
DN111920_c0_g3_i5	DOWN	-	-	-	XP_009801039.1:PREDICTED: uncharacterized protein LOC104246841 [Nicotiana sylvestris] >XP_016448705.1 PREDICTED: uncharacterized protein LOC107773794 [Nicotiana tabacum]	-	M1BA40|M1BA40_SOLTU:Uncharacterized protein;OS=Solanum tuberosum;
DN118585_c0_g1_i8	DOWN	[GO:0008168:methyltransferase activity][GO:0003723:RNA binding]	-	-	XP_002527285.1:PREDICTED: hemolysin A [Ricinus communis] >EEF35129.1 Hemolysin A, putative [Ricinus communis]	gi|255572703|ref|XM_002527239.1|:Ricinus communis Hemolysin A, putative, mRNA	B9SMR6|B9SMR6_RICCO:Hemolysin A, putative;OS=Ricinus communis;|GO:0008168,GO:0003723,

geneid	trend	GO	Pathway	TF	NR	NT	Uniprot
DN110707_c0_g2_i3	DOWN	-	[ko04340:Hedgehog signaling pathway][ko04341: Hedgehog signaling pathway - fly]	-	XP_010695634.1:PREDICTED: BTB/POZ domaincontaining protein At3g56230 [Beta vulgaris subsp. vulgaris] >KMT18872.1 hypothetical protein BVRB_2g029750 [Beta vulgaris subsp. vulgaris]	-	A0A0J8CZ30|A0A0J8CZ30_BETVU:Uncharacterized protein;OS=Beta vulgaris subsp. vulgaris.;
DN127587_c1_g2_i13	DOWN	[GO:0008270:zinc ion binding]	-	Lus10030908:Linum usitatissimum|bHLH|bHLH family protein	XP_009378828.1:PREDICTED: putative pentatricopeptide repeatcontaining protein At5g13230, mitochondrial [Pyrus x bretschneideri]	-	M5Y125|M5Y125_PRUPE:Uncharacterized protein;OS=Prunus persica;|GO:0008270,
DN128959_c0_g1_i1	DOWN	-	-	-	-	-	-

geneid	trend	GO	Pathway	TF	NR	NT	Uniprot
DN129170_c7_g1_i4	DOWN	[GO:0005524:ATP binding][GO:0004672:protein kinase activity]	-	462864640:Eragrostis tef|MYB_related|MYB_related family protein	XP_021773177.1:serine/threonineprotein kinase Nek3like [Chenopodium quinoa]	gi|521679032|gb|GAIA01027785.1|:TSA: Salicornia europaea Unigene30413_Se200S transcribed RNA sequence	A0A0K9RYR2|A0A0K9RYR2_SPIOL: Uncharacterized protein;OS=Spinacia oleracea;|GO:0005524,GO:0004672,
DN109467_c0_g1_i1	DOWN	-	-	-	OMO97311.1:hypothetical protein COLO4_14695 [Corchorus olitorius]	-	B9SE35|B9SE35_RICCO:Putative uncharacterized protein;OS=Ricinus communis;

geneid	trend	GO	Pathway	TF	NR	NT	Uniprot
DN115428_c1_g2_i1	DOWN	[GO:0005737:cytoplasm][GO:0016020:membrane][GO:0046914:transition metal ion binding][GO:0055073:cadmium ion homeostasis][GO:0046916:cellular transition metal ion homeostasis][GO:0071585:detoxification of cadmium ion][GO:0030001:metal ion transport]	-	-	NP_197247.1:Heavy metal transport/detoxification superfamily protein [Arabidopsis thaliana] >XP_002871767.1 heavy metalassociated isoprenylated plant protein 21 [Arabidopsis lyrata subsp. lyrata] >XP_006286597.1 hypothetical protein CARUB_v10002198mg [Capsella rubella] >Q9LF57.1 RecName: Full=Heavy metalassociated isoprenylated plant protein 21; Short=AtHIP21; Short=AtHIPP21; Flags: Precursor >CAC01889.1 farnesylated protein ATFP6like protein [Arabidopsis thaliana]	-	Q9LF57|HIP21_ARATH:Heavy metal-associated isoprenylated plant protein 21;OS=Arabidopsis thaliana;GN=At5g17450 {ECO:0000312|TAIR:AT5G17450};|GO:0005737,GO:0016020,|GO:0046914,|GO:0055073,GO:0046916,GO:0071585,GO:0030001,

geneid	trend	GO	Pathway	TF	NR	NT	Uniprot
DN121660_c4_g6_i2	DOWN	-	-	-	-	-	-
DN121740_c3_g4_i4	DOWN	[GO:0016021:integral component of membrane]	-	-	XP_021843120.1:uncharacterized protein LOC110783124 isoform X2 [Spinacia oleracea]	gi|521628019|gb|GAIA01052024.1|:TSA: Salicornia europaea Unigene61052_Se200S transcribed RNA sequence	A0A0K9RS94|A0A0K9RS94_SPIOL:Uncharacterized protein;OS=Spinacia oleracea;|GO:0016021,
DN132218_c0_g1_i1	DOWN	[GO:0005524:ATP binding]	[ko04013:MAPK signaling pathway - fly][ko04120:Ubiquitin mediated proteolysis][ko04141:Protein processing in endoplasmic reticulum][ko04624:Toll and Imd signaling pathway]	EMT28005:Aegilops tauschii|MYB_related|MYB_related family protein	KVD98068.1:Ubiquitinconjugating enzyme, active sitecontaining protein [Cynara cardunculus var. scolymus]	gi|508936051|gb|GAJC01010213.1|:TSA: Leptopilina heterotoma comp309_c0_seq1 transcribed RNA sequence	A0A103D5A8|A0A103D5A8_CYNCS: Ubiquitin-conjugating enzyme, active site-containing protein;OS=Cynara cardunculus var. scolymus;|GO:0005524,
DN96828_c0_g1_i1	DOWN	[GO:0016021:integral component of membrane][GO:0005524:ATP binding][GO:0016887:ATPase activity][GO:0006810:transport]	-	Rsa1.0_03996.1_g00001.1:Raphanus sativus|MYB_related|MYB_related family protein	XP_009619822.1:PREDICTED: pleiotropic drug resistance protein 1like [Nicotiana tomentosiformis]	gi|41052473|dbj|AB109389.1|:Nicotiana tabacum NtPDR2 mRNA for PDR-type ABC transporter 2, partial	A0A059BSU9|A0A059BSU9_EUCGR: Uncharacterized protein;OS=Eucalyptus grandis;|GO:001602

geneid	trend	GO	Pathway	TF	NR	NT	Uniprot
						cds	1,|GO:0005524,GO:0016887,|GO:0006810,
DN188988_c0_g1_i1	DOWN	[GO:0005623:cell][GO:0015035:protein disulfide oxidoreductase activity][GO:0045454:cell redox homeostasis][GO:0006662:glycerol ether metabolic process]	[ko04621:NOD-like receptor signaling pathway][ko05418:Fluid shear stress and atherosclerosis]	-	XP_021747937.1:thioredoxin Htypelike [Chenopodium quinoa]	-	A0A0K9QT74|A0A0K9QT74_SPIOL:Uncharacterized protein;OS=Spinacia oleracea;|GO:0005623,|GO:0015035,|GO:0045454,GO:0006662,
DN126440_c1_g5_i1	DOWN	-	-	-	KMT12117.1:hypothetical protein BVRB_5g100690 [Beta vulgaris subsp. vulgaris]	-	A0A0J8CF09|A0A0J8CF09_BETVU:Uncharacterized protein;OS=Beta vulgaris subsp. vulgaris.;
DN122537_c7_g1_i1	DOWN	-	-	-	-	-	-

geneid	trend	GO	Pathway	TF	NR	NT	Uniprot
DN116334_c0_g1_i5	DOWN	[GO:0043565:sequence-specific DNA binding][GO:0003700:DNA-binding transcription factor activity]	-	Sp_162090_wpom.t1:Spinacia oleracea|WRKY|WRKY family protein	XP_021864705.1:probable WRKY transcription factor 40 [Spinacia oleracea] >XP_021840235.1 probable WRKY transcription factor 40 [Spinacia oleracea] >KNA08497.1 hypothetical protein SOVF_162090 [Spinacia oleracea]	gi|317174862|gb|HQ527081.1|:Gossypium herbaceum clone NBRI_B_3048 simple sequence repeat marker, mRNA sequence	A0A0K9QMM8|A0A0K9QMM8_SPIOL:Uncharacterized protein;OS=Spinacia oleracea;|GO:0043565,GO:0003700,
DN226450_c0_g1_i1	DOWN	-	[ko03010:Ribosome]	Gh_A13G1934:Gossypium hirsutum|bZIP|bZIP family protein	XP_006409766.1:hypothetical protein EUTSA_v10017225mg [Eutrema salsugineum] >ESQ51219.1 hypothetical protein EUTSA_v10017225mg [Eutrema salsugineum]	gi|510109365|gb|GAIW01010795.1|:TSA: Ganaspis sp. G1 comp88_c0_seq1 transcribed RNA sequence	A0A1D1Y8S7|A0A1D1Y8S7_9ARAE: 60S ribosomal protein L10a;OS=Anthurium amnicola.;

geneid	trend	GO	Pathway	TF	NR	NT	Uniprot
DN119427_c0_g1_i5	DOWN	[GO:0016021:integral component of membrane][GO:0055085:transmembrane transport]	-	Jcr4S00206.70:Jatropha curcas|FAR1|FAR1 family protein	XP_018856104.1:PREDICTED: protein PINLIKES 7like [Juglans regia]	-	A0A0J8C515|A0A0J8C515_BETVU:Uncharacterized protein;OS=Beta vulgaris subsp. vulgaris.;|GO:0016021,|GO:0055085,
DN111403_c0_g1_i1	DOWN	[GO:0005634:nucleus][GO:0000977:RNA polymerase II regulatory region sequence-specific DNA binding][GO:0003700:DNA-binding transcription factor activity][GO:0000165:MAPK cascade][GO:0045944:positive regulation of transcription by RNA polymerase II][GO:0006351:transcription, DNA-templated]	-	Bv1_019790_iszi.t1:Beta vulgaris|MIKC_MADS|MIKC_MADS family protein	XP_010692324.1:PREDICTED: truncated transcription factor CAULIFLOWER A isoform X1 [Beta vulgaris subsp. vulgaris] >KMT00161.1 hypothetical protein BVRB_1g019790 [Beta vulgaris subsp. vulgaris]	gi|521742336|gb|GAIA01005001.1|:TSA: Salicornia europaea Unigene5288_Se200S transcribed RNA sequence	A0A0J8EAU8|A0A0J8EAU8_BETVU: Uncharacterized protein;OS=Beta vulgaris subsp. vulgaris.;|GO:0005634,|GO:0000977,GO:0003700,|GO:0000165,GO:0045944,GO:0006351,
DN109249_c1_g7_i1	DOWN	-	-	-	-	-	-

geneid	trend	GO	Pathway	TF	NR	NT	Uniprot
DN116820_c0_g1_i6	DOWN	-	[ko04626:Plant-pathogen interaction]	-	XP_021288060.1:disease resistance protein RPM1like isoform X1 [Herrania umbratica]	-	-
DN128395_c1_g1_i1	DOWN	[GO:0016788:hydrolase activity, acting on ester bonds]	[ko00562:Inositol phosphate metabolism][ko00564:Glycerophospholipid metabolism][ko00565:Ether lipid metabolism][ko02024:Quorum sensing][ko04919:Thyroid hormone signaling pathway]	Csa09g050690.1:Camelina sativa|NF-YC|NF-YC family protein	XP_010691938.1:PREDICTED: nonspecific phospholipase C4 [Beta vulgaris subsp. vulgaris] >KMS99829.1 hypothetical protein BVRB_1g016810 [Beta vulgaris subsp. vulgaris]	-	A0A0J8BEP0|A0A0J8BEP0_BETVU:Uncharacterized protein;OS=Beta vulgaris subsp. vulgaris.;|GO:0016788,
DN122143_c1_g1_i3	DOWN	-	-	-	XP_010530699.1:PREDICTED: uncharacterized protein LOC104807210 [Tarenaya hassleriana]	-	A0A0K9RE53|A0A0K9RE53_SPIOL:Uncharacterized protein;OS=Spinacia oleracea;

geneid	trend	GO	Pathway	TF	NR	NT	Uniprot
DN125521_c1_g5_i1	DOWN	-	-	-	-	-	-
DN97025_c0_g1_i3	DOWN	[GO:0005887:integral component of plasma membrane][GO:0008515:sucrose transmembrane transporter activity]	-	Achn143971:Actinidia chinensis|HD-ZIP|HD-ZIP family protein	XP_010673573.1:PREDICTED: sucrose transport protein SUC2like [Beta vulgaris subsp. vulgaris]	gi|210141035|dbj|AK244954.1|:Glycine max cDNA, clone: GMFL01-17-K15	A0A0J8D1P2|A0A0J8D1P2_BETVU:Uncharacterized protein;OS=Beta vulgaris subsp. vulgaris.;|GO:0005887,|GO:0008515,

geneid	trend	GO	Pathway	TF	NR	NT	Uniprot
DN118735_c6_g3_i4	DOWN	[GO:0005576:extracellular region][GO:0050832:defense response to fungus][GO:0031640:killing of cells of other organism]	-	-	P81418.1:RecName: Full=Antimicrobial peptide 1; AltName: Full=AFPS1; AltName: Full=Antifungal protein 1; AltName: Full=PAFPS; AltName: Full=PaAMP1; Short=AMP1; Flags: Precursor >AAC05129.1 antifungal protein precursor [Phytolacca americana] >AAD17942.1 antifungal protein [Phytolacca americana] >BAA88893.1 antifungal protein precursor [Phytolacca	-	P81418|PAFP_PHYAM:Antimicrobial peptide 1;OS=Phytolacca americana;|GO:0005576,|GO:0050832,GO:0031640,

geneid	trend	GO	Pathway	TF	NR	NT	Uniprot
					americana]		

geneid	trend	GO	Pathway	TF	NR	NT	Uniprot
DN89578_c0_g2_i1	DOWN	-	[ko03010:Ribosome]	Achn302621:Actinidia chinensis|BES1|BES1 family protein	ABK21348.1:unknown [Picea sitchensis] >ABK21383.1 unknown [Picea sitchensis] >ABK21513.1 unknown [Picea sitchensis] >ABK21531.1 unknown [Picea sitchensis] >ABK21681.1 unknown [Picea sitchensis] >ABK22315.1 unknown [Picea sitchensis] >ABK22334.1 unknown [Picea sitchensis] >ABK24964.1 unknown [Picea sitchensis] >ABK26177.1 unknown [Picea sitchensis] >ABK26197.1 unknown [Picea sitchensis] >ABR16244.1 unknown [Picea	-	A0A1D1Z646|A0A1D1Z646_9ARAE:60S ribosomal protein L36;OS=Anthurium amnicola.;

geneid	trend	GO	Pathway	TF	NR	NT	Uniprot
DN126366_c2_g1_i1	DOWN	-	-	GSVIVT01033372001:Vitis vinifera|NAC|NAC family protein	XP_010687943.1:PREDICTED: serine/threonineprotein phosphatase 1 regulatory subunit 10 [Beta vulgaris subsp. vulgaris]	-	A0A0K9RMJ3|A0A0K9RMJ3_SPIOL:Uncharacterized protein;OS=Spinacia oleracea;

geneid	trend	GO	Pathway	TF	NR	NT	Uniprot
DN124998_c1_g4_i2	DOWN	-	-	-	-	gi|340842619|gb|HQ664654.1|:Phytolacca americana rpl23 pseudogene and tRNA-Ile (trnI-CAU) gene, complete sequence; hypothetical chloroplast RF2 (ycf2) gene, complete cds; tRNA-Leu (trnL-CAA) gene, complete sequence; NADH-plastoquinone oxidoreductase subunit 2 (ndhB) gene, complete cds; ribosomal protein S7 (rps7) gene, partial cds; ribosomal protein S12 (rps12) gene, exon 2 and partial cds; 16S ribosomal RNA (rrn16) gene, complete sequence; tRNA-Ile (trnI-GAU), tRNA-Ala (trnA-UGC), and	-

geneid	trend	GO	Pathway	TF	NR	NT	Uniprot
DN116525_c1_g1_i12	DOWN	[GO:0046872:metal ion binding][GO:0016491:oxidoreductase activity]	[ko00904:Diterpenoid biosynthesis]	AA31G00726:Aethionema arabicum|STAT|STAT family protein	XP_021854850.1:gibberellin 2betadioxygenase 8like isoform X1 [Spinacia oleracea] >XP_021854851.1 gibberellin 2betadioxygenase 8like isoform X1 [Spinacia oleracea] >XP_021854852.1 gibberellin 2betadioxygenase 8like isoform X1 [Spinacia oleracea]	-	Q5D0G3|Q5D0G3_SPIOL:Gibberellin 2-oxidase 3;OS=Spinacia oleracea;|GO:0046872,GO:0016491,

geneid	trend	GO	Pathway	TF	NR	NT	Uniprot
DN121802_c0_g3_i4	DOWN	[GO:0005524:ATP binding][GO:0004674:protein serine/threonine kinase activity]	[ko04068:FoxO signaling pathway][ko04310: Wnt signaling pathway][ko04340: Hedgehog signaling pathway][ko04341: Hedgehog signaling pathway - fly][ko04390:Hippo signaling pathway][ko04391: Hippo signaling pathway - fly][ko04392:Hippo signaling pathway -multiple species][ko04710:Circadian rhythm][ko04711:Circadian rhythm - fly]	-	XP_010688272.1:PREDICTED: casein kinase 1like protein 4 [Beta vulgaris subsp. vulgaris] >XP_010688273.1 PREDICTED: casein kinase 1like protein 4 [Beta vulgaris subsp. vulgaris] >KMT03143.1 hypothetical protein BVRB_8g196990 [Beta vulgaris subsp. vulgaris]	-	A0A0J8BTB6|A0A0J8BTB6_BETVU:Uncharacterized protein;OS=Beta vulgaris subsp. vulgaris.;|GO:0005524,GO:0004674,
DN119243_c3_g10_i2	DOWN	-	-	-	-	-	-

geneid	trend	GO	Pathway	TF	NR	NT	Uniprot
DN119004_c0_g7_i1	DOWN	-	[ko04140:Autophagy - animal][ko04142:Lysosome][ko04145:Phagosome][ko04210:Apoptosis][ko04612:Antigen processing and presentation][ko05205:Proteoglycans in cancer][ko05323:Rheumatoid arthritis][ko05418:Fluid shear stress and atherosclerosis]	FANhyb_rscf00000253.1.g00010.1:Fragaria x ananassa|BBR-BPC|BBR-BPC family protein	BAK02675.1:predicted protein [Hordeum vulgare subsp. vulgare]	-	A0A1D1ZBY8|A0A1D1ZBY8_9ARAE: Cathepsin L;OS=Anthurium amnicola.;
DN92387_c0_g1_i1	DOWN	-	[ko03010:Ribosome]	-	XP_010922435.1:PREDICTED: 60S ribosomal protein L35 [Elaeis guineensis]	-	A0A1D1YAC0|A0A1D1YAC0_9ARAE: 60S ribosomal protein L35;OS=Anthurium amnicola.;

geneid	trend	GO	Pathway	TF	NR	NT	Uniprot
DN125308_c1_g1_i8	DOWN	-	-	-	XP_021857734.1:uncharacterized protein LOC110796945 isoform X2 [Spinacia oleracea] >KNA13980.1 hypothetical protein SOVF_111630 [Spinacia oleracea]	-	A0A0K9R396|A0A0K9R396_SPIOL:Uncharacterized protein;OS=Spinacia oleracea;
DN103751_c0_g1_i1	DOWN	[GO:0005524:ATP binding]	[ko03060:Protein export][ko04141:Protein processing in endoplasmic reticulum][ko04918:Thyroid hormone synthesis][ko05020:Prion diseases]	FANhyb_rscf00000233.1.g00002.1:Fragaria x ananassa|GATA|GATA family protein	BAJ93696.1:predicted protein [Hordeum vulgare subsp. vulgare]	gi|501915807|gb|GAJB01005665.1|:TSA: Alexandrium tamarense Atam42274 transcribed RNA sequence	F2DF25|F2DF25_HORVD:Predicted protein;OS=Hordeum vulgare var. distichum;|GO:0005524,
DN104844_c0_g1_i1	DOWN	-	-	Sp_105690_ioxf.t1: Spinacia oleracea|bHLH|bHLH family protein	KNA14639.1:hypothetical protein SOVF_105690 [Spinacia oleracea]	-	A0A0K9R6S4|A0A0K9R6S4_SPIOL:Uncharacterized protein;OS=Spinacia oleracea;
DN108919_c1_g2_i1	DOWN	-	-	-	-	-	-

geneid	trend	GO	Pathway	TF	NR	NT	Uniprot
DN79702_c0_g1_i1	DOWN	-	-	-	-	-	-
DN125649_c4_g2_i7	DOWN	[GO:0016021:integral component of membrane][GO:0004190:aspartic-type endopeptidase activity]	-	-	XP_021714399.1:aspartic proteinase nepenthesin1like [Chenopodium quinoa]	gi|225438907|ref|XM_002279158.1|:PREDICTED: Vitis vinifera aspartic proteinase-like protein 2-like (LOC100262817), mRNA	A0A0K9RKC6|A0A0K9RKC6_SPIOL: Uncharacterized protein;OS=Spinacia oleracea;|GO:0016021,|GO:0004190,
DN67344_c0_g1_i1	DOWN	[GO:0005840:ribosome][GO:0003735:structural constituent of ribosome][GO:0006412:translation]	[ko03010:Ribosome]	Csa04g009800.1:Camelina sativa|M-type_MADS|M-type_MADS family protein	XP_001692985.1:ribosomal protein L8 [Chlamydomonas reinhardtii] >EDP03554.1 ribosomal protein L8 [Chlamydomonas reinhardtii]	gi|308486156|ref|XM_003105228.1|:Caenorhabditis remanei CRE-RPL-2 protein (Cre-rpl-2) mRNA, complete cds	A0A061SA28|A0A061SA28_9CHLO:Large subunit ribosomal protein L8e;OS=Tetraselmis sp. GSL018.;|GO:0005840,|GO:0003735,|GO:0006412,
DN191494_c0_g1_i1	DOWN	-	-	-	-	-	-

geneid	trend	GO	Pathway	TF	NR	NT	Uniprot
DN119126_c1_g2_i3	DOWN	[GO:0016021:integral component of membrane][GO:0005524:ATP binding][GO:0004672:protein kinase activity]	-	Achn335281:Actinidia chinensis|NAC|NAC family protein	XP_010669046.1:PREDICTED: receptorlike protein kinase HSL1 [Beta vulgaris subsp. vulgaris]	-	A0A0J8FQN3|A0A0J8FQN3_BETVU:Uncharacterized protein;OS=Beta vulgaris subsp. vulgaris.;|GO:0016021,|GO:0005524,GO:0004672,
DN114985_c4_g2_i1	DOWN	[GO:0016787:hydrolase activity][GO:0008152:metabolic process]	-	-	XP_015874288.1:PREDICTED: probable carboxylesterase 12 [Ziziphus jujuba]	-	A0A068V4E4|A0A068V4E4_COFCA:Uncharacterized protein;OS=Coffea canephora;|GO:0016787,|GO:0008152,
DN226796_c0_g1_i1	DOWN	-	-	-	-	-	-
DN116972_c0_g3_i3	DOWN	-	-	Rsa1.0_00424.1_g00007.1:Raphanus sativus|C2H2|C2H2 family protein	XP_020252239.1:uncharacterized protein LOC109829580 [Asparagus officinalis]	-	A0A0V0IYZ4|A0A0V0IYZ4_SOLCH:Putative ovule protein;OS=Solanum chacoense;

geneid	trend	GO	Pathway	TF	NR	NT	Uniprot
DN120933_c0_g2_i2	DOWN	[GO:0016021:integral component of membrane][GO:0005215:transporter activity][GO:0055085:transmembrane transport]	-	-	XP_010691555.1:PREDICTED: nucleobaseascorbate transporter 4 [Beta vulgaris subsp. vulgaris] >KMT01024.1 hypothetical protein BVRB_9g222860 [Beta vulgaris subsp. vulgaris]	gi|3202037|gb|AF069322.1|AF069322:Mesembryanthemum crystallinum clone R30-9 permease 1 mRNA, complete cds	A0A0J8BM02|A0A0J8BM02_BETVU: Uncharacterized protein;OS=Beta vulgaris subsp. vulgaris.;|GO:0016021,|GO:0005215,|GO:0055085,
DN113965_c0_g1_i2	DOWN	-	-	-	-	-	-
DN117979_c0_g1_i1	DOWN	-	-	-	-	-	-
DN114421_c4_g1_i1	DOWN	-	-	-	-	-	-

geneid	trend	GO	Pathway	TF	NR	NT	Uniprot
DN119549_c3_g1_i7	DOWN	[GO:0005524:ATP binding][GO:0016772:transferase activity, transferring phosphorus-containing groups]	-	-	OWM77678.1:hypothetical protein CDL15_Pgr017078 [Punica granatum]	gi|359483316|ref|XM_002268212.2|:PREDICTED: Vitis vinifera probable pyruvate, phosphate dikinase regulatory protein, chloroplastic-like, transcript variant 1 (LOC100267490), mRNA	A0A067G7F9|A0A067G7F9_CITSI:Uncharacterized protein;OS=Citrus sinensis;|GO:0005524,GO:0016772,
DN115368_c8_g4_i2	DOWN	-	-	-	-	-	-
DN59317_c0_g1_i1	DOWN	-	[ko03010:Ribosome]	-	KHN42697.1:Protein transport protein Sec16B [Glycine soja]	-	A0A1D1YUY4|A0A1D1YUY4_9ARAE:40S ribosomal protein S30;OS=Anthurium amnicola.;

geneid	trend	GO	Pathway	TF	NR	NT	Uniprot
DN101381_c1_g1_i1	DOWN	[GO:0008168:methyltransferase activity]	-	EMT20798:Aegilops tauschii|MYB_related|MYB_related family protein	XP_018820877.1:PREDICTED: salicylate carboxymethyltransferaselike [Juglans regia]	-	D7SP88|D7SP88_VITVI:Putative uncharacterized protein;OS=Vitis vinifera;GN=VIT_04s0023g02310 {ECO:0000313|EMBL:CBI17467.3};|GO:0008168,
DN125472_c1_g3_i3	DOWN	-	-	Tp57577_TGAC_v2_mRNA31725:Trifolium pratense|WRKY|WRKY family protein	XP_019102841.1:PREDICTED: uncharacterized protein LOC109133677 [Beta vulgaris subsp. vulgaris]	-	M1C4J6|M1C4J6_SOLTU:Uncharacterized protein;OS=Solanum tuberosum;
DN91198_c0_g1_i1	DOWN	[GO:0000786:nucleosome][GO:0005634:nucleus][GO:0003677:DNA binding][GO:0006334:nucleosome assembly]	[ko05034:Alcoholism][ko05203:Viral carcinogenesis][ko05322:Systemic lupus erythematosus]	Aan019826:Artemisia annua|CO-like|CO-like family protein	KJB21638.1:hypothetical protein B456_004G005600 [Gossypium raimondii]	gi|391344003|ref|XM_003746247.1|:PREDICTED: Metaseiulus occidentalis uncharacterized LOC100903522 (LOC100903522), mRNA	A0A0D2P4R2|A0A0D2P4R2_GOSRA: Histone H4;OS=Gossypium raimondii;|GO:0000786,GO:0005634,|GO:0003677,|GO:0006334,

geneid	trend	GO	Pathway	TF	NR	NT	Uniprot
DN109871_c0_g1_i1	DOWN	[GO:0016021:integral component of membrane]	-	Tp57577_TGAC_v2_mRNA3016:Trifolium pratense|Trihelix|Trihelix family protein	KJB36419.1:hypothetical protein B456_006G159900 [Gossypium raimondii]	gi|521448158|gb|GAHZ01072326.1|:TSA: Salicornia europaea Unigene88062_SeCKS transcribed RNA sequence	A0A0D2RX30|A0A0D2RX30_GOSRA: Uncharacterized protein;OS=Gossypium raimondii;|GO:0016021,
DN122338_c2_g1_i2	DOWN	-	-	-	XP_021769390.1:uncharacterized protein LOC110733628 [Chenopodium quinoa]	gi|224143206|ref|XM_002324845.1|:Populus trichocarpa predicted protein, mRNA	A0A068UHH9|A0A068UHH9_COFCA: Uncharacterized protein;OS=Coffea canephora;
DN106744_c0_g1_i2	DOWN	[GO:0003677:DNA binding][GO:0006355:regulation of transcription, DNA-templated][GO:0090378:seed trichome elongation]	-	Cotton_A_33049_BGI-A2_v1.0:Gossypium arboreum|MYB|MYB family protein	XP_012462415.1:PREDICTED: transcription repressor MYB6like [Gossypium raimondii] >KJB81321.1 hypothetical protein B456_013G139200 [Gossypium raimondii]	gi|512396378|gb|GAJZ01004792.1|:TSA: Prunus avium Pa_11466 transcribed RNA sequence	Q43436|Q43436_GOSHI:MYB1;OS=Gossypium hirsutum;|GO:0003677,|GO:0006355,GO:0090378,
DN85268_c0	DOWN	-	-	-	-	-	-

geneid	trend	GO	Pathway	TF	NR	NT	Uniprot
_g1_i2							
DN117460_c0_g1_i2	DOWN	[GO:0005829:cytosol][GO:0033926:glycopeptide alpha-N-acetylgalactosaminidase activity]	-	-	XP_010690448.1:PREDICTED: probable alkaline/neutral invertase B [Beta vulgaris subsp. vulgaris] >XP_010690449.1 PREDICTED: probable alkaline/neutral invertase B [Beta vulgaris subsp. vulgaris] >XP_010690450.1 PREDICTED: probable alkaline/neutral invertase B [Beta vulgaris subsp. vulgaris] >KMT01462.1 hypothetical protein	gi|470106988|ref|XM_004289786.1|:PREDICTED: Fragaria vesca subsp. vesca uncharacterized LOC101301732 (LOC101301732), mRNA	A0A0J8BKD1|A0A0J8BKD1_BETVU: Uncharacterized protein;OS=Beta vulgaris subsp. vulgaris.;|GO:0005829,|GO:0033926,

geneid	trend	GO	Pathway	TF	NR	NT	Uniprot
					BVRB_9g214690 [Beta vulgaris subsp. vulgaris]		

geneid	trend	GO	Pathway	TF	NR	NT	Uniprot
DN113883_c0_g1_i4	DOWN	[GO:0000166:nucleotide binding][GO:0017069:snRNA binding][GO:0000398:mRNA splicing, via spliceosome]	[ko03040:Spliceosome]	KZV25770.1:Dorcoceras hygrometricum|WRKY|WRKY family protein	XP_021861779.1:U1 small nuclear ribonucleoprotein A [Spinacia oleracea] >KNA23317.1 hypothetical protein SOVF_025990 [Spinacia oleracea]	gi|521737005|gb|GAIA01008404.1|:TSA: Salicornia europaea Unigene8738_Se200S transcribed RNA sequence	A0A0K9RWU6|A0A0K9RWU6_SPIOL:Uncharacterized protein;OS=Spinacia oleracea;|GO:0000166,GO:0017069,|GO:0000398,
DN128036_c10_g1_i10	DOWN	[GO:0030246:carbohydrate binding][GO:0003824:catalytic activity][GO:0005975:carbohydrate metabolic process]	-	-	XP_021747845.1:probable rhamnogalacturonate lyase B [Chenopodium quinoa]	-	A0A0J8C0D3|A0A0J8C0D3_BETVU:Uncharacterized protein;OS=Beta vulgaris subsp. vulgaris.;|GO:0030246,GO:0003824,|GO:0005975,
DN79277_c0_g3_i1	DOWN	[GO:0005840:ribosome][GO:0000166:nucleotide binding][GO:0003735:structural constituent of ribosome][GO:0006412:translation]	[ko03010:Ribosome]	mrna26696.1-v1.0-hybrid:Fragaria vesca|bHLH|bHLH family protein	XP_011400543.1:60S ribosomal protein L23A [Auxenochlorella protothecoides] >KFM27560.1 60S ribosomal protein L23A [Auxenochlorella	gi|15293914|gb|AF401578.1|:Ictalurus punctatus ribosomal protein L23a mRNA, partial cds	A0A087SPA6|A0A087SPA6_AUXPR:60S ribosomal protein L23A;OS=Auxenochlorella protothecoides;|GO:0005840,|GO:0000166,GO:0003735,|GO:0006412,

geneid	trend	GO	Pathway	TF	NR	NT	Uniprot
					protothecoides]		
DN128720_c0_g1_i9	DOWN	[GO:0003677:DNA binding]	-	Sp_000340_siic.t1:Spinacia oleracea|bHLH|bHLH family protein	KNA13392.1:hypothetical protein SOVF_117470 [Spinacia oleracea]	-	A0A0K9R3K9|A0A0K9R3K9_SPIOL:Uncharacterized protein;OS=Spinacia oleracea;|GO:0003677,
DN125443_c1_g5_i3	DOWN	-	-	-	-	-	-
DN98412_c0_g2_i1	DOWN	-	-	-	-	-	-

geneid	trend	GO	Pathway	TF	NR	NT	Uniprot
DN60022_c0_g1_i1	DOWN	[GO:0005623:cell][GO:0004601:peroxidase activity][GO:0051920:peroxiredoxin activity][GO:0045454:cell redox homeostasis]	-	-	XP_013904140.1:peroxiredoxin (alkyl hydroperoxidereductase subunit C) [Monoraphidium neglectum] >KIZ05121.1 peroxiredoxin (alkyl hydroperoxidereductase subunit C) [Monoraphidium neglectum]	-	A0A0D2NJX0|A0A0D2NJX0_9CHLO: Peroxiredoxin (Alkyl hydroperoxidereductase subunit C);OS=Monoraphidium neglectum.;|GO:0005623,|GO:0004601, GO:0051920,|GO:0045454,
DN118411_c3_g5_i2	DOWN	-	-	-	-	-	-
DN126198_c0_g1_i14	DOWN	[GO:0016787:hydrolase activity]	[ko05034:Alcoholism][ko05203:Viral carcinogenesis]	-	XP_010667134.1:PREDICTED: histone deacetylase 2 isoform X2 [Beta vulgaris subsp. vulgaris] >KMS95576.1 hypothetical protein BVRB_006940 [Beta vulgaris subsp. vulgaris]	gi|521735281|gb|GAIA01009268.1|:TSA: Salicornia europaea Unigene9631_Se200S transcribed RNA sequence	A0A0J8B3M0|A0A0J8B3M0_BETVU: Uncharacterized protein;OS=Beta vulgaris subsp. vulgaris.;|GO:0016787,

geneid	trend	GO	Pathway	TF	NR	NT	Uniprot
DN127212_c2_g4_i1	DOWN	-	-	-	-	-	-
DN117064_c3_g1_i8	DOWN	[GO:0016021:integral component of membrane][GO:0005215:transporter activity][GO:0006857:oligopeptide transport]	-	Oropetium_20150105_01570A:Oropetium thomaeum|WRKY|WRKY family protein	XP_021292805.1:protein NRT1/ PTR FAMILY 8.3like [Herrania umbratica]	gi|521452864|gb|GAHZ01067620.1|:TSA: Salicornia europaea Unigene81668_SeCKS transcribed RNA sequence	A0A061EA41|A0A061EA41_THECC:Peptide transporter 2 isoform 1;OS=Theobroma cacao;|GO:0016021,|GO:0005215,|GO:0006857,
DN114746_c0_g1_i1	DOWN	-	-	-	XP_010656991.1:PREDICTED: leucinerich repeat extensinlike protein 4 [Vitis vinifera] >CBI21875.3 unnamed protein product, partial [Vitis vinifera]	-	E0CTG0|E0CTG0_VITVI:Putative uncharacterized protein;OS=Vitis vinifera;GN=VIT_12s0028g03390 {ECO:0000313|EMBL:CBI21875.3};

geneid	trend	GO	Pathway	TF	NR	NT	Uniprot
DN122085_c0_g1_i4	DOWN	[GO:0042578:phosphoric ester hydrolase activity][GO:0036092:phosphatidylinositol-3-phosphate biosynthetic process][GO:0007033:vacuole organization]	-	-	XP_021847434.1:phosphoinositide phosphatase SAC2like [Spinacia oleracea]	-	A0A0J8BUE3|A0A0J8BUE3_BETVU:Uncharacterized protein;OS=Beta vulgaris subsp. vulgaris.;|GO:0042578,|GO:0036092,GO:0007033,
DN126457_c3_g1_i6	DOWN	-	-	FANhyb_rscf00000149.1.g00006.1:Fragaria x ananassa|GRAS|GRAS family protein	XP_010691192.1:PREDICTED: protein BCCIP homolog [Beta vulgaris subsp. vulgaris] >KMT00712.1 hypothetical protein BVRB_9g219980 [Beta vulgaris subsp. vulgaris]	gi|388520830|gb|BT148683.1|:Medicago truncatula clone JCVI-FLMt-18J24 unknown mRNA	A0A0J8BI99|A0A0J8BI99_BETVU:Protein BCCIP homolog;OS=Beta vulgaris subsp. vulgaris.;

geneid	trend	GO	Pathway	TF	NR	NT	Uniprot
DN128061_c0_g1_i2	DOWN	[GO:0016021:integral component of membrane][GO:0005524:ATP binding][GO:0042626:ATPase activity, coupled to transmembrane movement of substances]	-	-	XP_006466060.1:PREDICTED: ABC transporter C family member 8 isoform X1 [Citrus sinensis] >XP_015387761.1 PREDICTED: ABC transporter C family member 8 isoform X1 [Citrus sinensis]	-	A0A067FDA4|A0A067FDA4_CITSI:Uncharacterized protein;OS=Citrus sinensis;|GO:0016021,|GO:0005524,GO:0042626,
DN111089_c0_g2_i2	DOWN	-	-	-	-	-	-
DN123462_c0_g2_i3	DOWN	[GO:0009570:chloroplast stroma][GO:0005829:cytosol][GO:0009508:plastid chromosome][GO:0003676:nucleic acid binding]	-	-	XP_021759329.1:protein PLASTID TRANSCRIPTIONALLY ACTIVE 10like [Chenopodium quinoa]	gi|521704068|gb|GAIA01015890.1|:TSA: Salicornia europaea Unigene16586_Se200S transcribed RNA sequence	A0A0J8CGT2|A0A0J8CGT2_BETVU:Uncharacterized protein;OS=Beta vulgaris subsp. vulgaris.;|GO:0009570,GO:0005829,GO:0009508,|GO:0003676,

geneid	trend	GO	Pathway	TF	NR	NT	Uniprot
DN113008_c0_g4_i2	DOWN	[GO:0003824:catalytic activity][GO:0050662:coenzyme binding]	-	MDP0000265073:Malus domestica|NAC|NAC family protein	XP_010673664.1:PREDICTED: tetraketide alphapyrone reductase 1 isoform X1 [Beta vulgaris subsp. vulgaris] >KMT14527.1 hypothetical protein BVRB_4g072970 isoform B [Beta vulgaris subsp. vulgaris]	gi|357455444|ref|XM_003597955.1|:Medicago truncatula Dihydroflavonol-4-reductase (MTR_3g005170) mRNA, complete cds	A0A0J8CLZ4|A0A0J8CLZ4_BETVU:Uncharacterized protein;OS=Beta vulgaris subsp. vulgaris.;|GO:0003824,GO:0050662,
DN60812_c0_g1_i1	DOWN	[GO:0005840:ribosome][GO:0003735:structural constituent of ribosome][GO:0006414:translational elongation]	[ko03010:Ribosome]	-	GAX78858.1:hypothetical protein CEUSTIGMA_g6296.t1 [Chlamydomonas eustigma]	-	A0A024GBI1|A0A024GBI1_9STRA:Uncharacterized protein;OS=Albugo candida.;|GO:0005840,|GO:0003735,|GO:0006414,

geneid	trend	GO	Pathway	TF	NR	NT	Uniprot
DN127388_c0_g1_i5	DOWN	[GO:0046872:metal ion binding]	-	-	XP_021729504.1:phospholipase SGR2like isoform X2 [Chenopodium quinoa]	gi|449442115|ref|XM_004138780.1|:PREDICTED: Cucumis sativus SEC23-interacting protein-like (LOC101221207), mRNA	A0A0J8D5G2|A0A0J8D5G2_BETVU:Uncharacterized protein;OS=Beta vulgaris subsp. vulgaris.;|GO:0046872,
DN110753_c0_g1_i1	DOWN	-	-	-	-	-	-
DN125356_c4_g2_i3	DOWN	-	-	-	-	-	-
DN122974_c7_g12_i4	DOWN	[GO:0045735:nutrient reservoir activity]	-	-	XP_021761604.1:glutelin typeA 3like [Chenopodium quinoa]	-	A0A0J8F4N5|A0A0J8F4N5_BETVU:Uncharacterized protein;OS=Beta vulgaris subsp. vulgaris.;|GO:0045735,

geneid	trend	GO	Pathway	TF	NR	NT	Uniprot
DN119658_c0_g2_i4	DOWN	[GO:0016602:CCAAT-binding factor complex][GO:0003677:DNA binding][GO:0003700:DNA-binding transcription factor activity]	[ko04612:Antigen processing and presentation][ko05152:Tuberculosis]	Aradu.YE2F8:Arachis duranensis|NF-YA|NF-YA family protein	GAU17519.1:hypothetical protein TSUD_340630 [Trifolium subterraneum]	gi|502112190|ref|XM_004494202.1|:PREDICTED: Cicer arietinum nuclear transcription factor Y subunit A-10-like (LOC101506076), mRNA	F6I3K7|F6I3K7_VITVI:Putative uncharacterized protein;OS=Vitis vinifera;GN=VIT_08s0032g01190 {ECO:0000313|EMBL:CCB61571.1};|GO:0016602,|GO:0003677,GO:0003700,
DN122397_c5_g14_i1	DOWN	-	-	-	-	-	-
DN93177_c0_g1_i1	DOWN	[GO:0004185:serine-type carboxypeptidase activity]	-	Sme2.5_00187.1_g00004.1:Solanum melongena|MYB_related|MYB_related family protein	XP_021735430.1:serine carboxypeptidaselike 34 [Chenopodium quinoa] >XP_021735431.1 serine carboxypeptidaselike 34 [Chenopodium quinoa]	-	A0A0J8BMN8|A0A0J8BMN8_BETVU: Carboxypeptidase;OS=Beta vulgaris subsp. vulgaris.;|GO:0004185,
DN112881_c0_g2_i1	DOWN	-	-	-	-	-	-

geneid	trend	GO	Pathway	TF	NR	NT	Uniprot
DN123436_c3_g2_i1	DOWN	-	-	-	XP_021858760.1:uncharacterized protein LOC110797933 [Spinacia oleracea] >KNA15193.1 hypothetical protein SOVF_100560 [Spinacia oleracea]	gi|359477018|ref|XM_002274272.2|:PREDICTED: Vitis vinifera 5' exonuclease Apollo-like (LOC100254013), mRNA	A0A0K9R6Q8|A0A0K9R6Q8_SPIOL:Uncharacterized protein;OS=Spinacia oleracea;
DN118488_c0_g7_i1	DOWN	-	-	-	-	-	-
DN114856_c0_g2_i1	DOWN	[GO:0005737:cytoplasm][GO:0005874:microtubule][GO:0005525:GTP binding][GO:0003924:GTPase activity][GO:0005200:structural constituent of cytoskeleton][GO:0007017:microtubule-based process]	[ko04145:Phagosome][ko04210:Apoptosis][ko04530:Tight junction][ko04540:Gap junction][ko05130:Pathogenic Escherichia coli infection]	KN539001.1_FGP003:Oryza longistaminata|NAC|NAC family protein	BAK03519.1:predicted protein [Hordeum vulgare subsp. vulgare]	gi|194899301|ref|XM_001979163.1|:Drosophila erecta GG14119 (Dere\GG14119), mRNA	F2E847|F2E847_HORVD:Tubulin alpha chain;OS=Hordeum vulgare var. distichum;|GO:0005737,GO:0005874,|GO:0005525,GO:0003924,GO:0005200,|GO:0007017,
DN116806_c0_g1_i3	DOWN	-	-	-	-	-	-
DN126319_c1_g1_i2	DOWN	-	-	-	-	-	-

geneid	trend	GO	Pathway	TF	NR	NT	Uniprot
DN122617_c4_g1_i3	DOWN	-	-	-	-	-	-
DN114928_c0_g1_i5	DOWN	[GO:0005777:peroxisome][GO:0080023:3R-hydroxyacyl-CoA dehydratase activity][GO:0033542:fatty acid beta-oxidation, unsaturated, even number]	-	-	XP_021851085.1:enoylCoA hydratase 2, peroxisomal [Spinacia oleracea] >XP_021851086.1 enoylCoA hydratase 2, peroxisomal [Spinacia oleracea]	gi|521706627|gb|GAIA01014596.1|:TSA: Salicornia europaea Unigene15256_Se200S transcribed RNA sequence	A0A0J8B6M6|A0A0J8B6M6_BETVU: Uncharacterized protein;OS=Beta vulgaris subsp. vulgaris.;|GO:0005777,|GO:0080023,|GO:0033542,
DN118870_c6_g4_i3	DOWN	-	-	-	KYP76305.1:hypothetical protein KK1_020539 [Cajanus cajan]	-	A0A151UAF9|A0A151UAF9_CAJCA: Uncharacterized protein;OS=Cajanus cajan;
DN114523_c1_g1_i4	DOWN	[GO:0016021:integral component of membrane][GO:0005215:transporter activity][GO:0006857:oligopeptide transport]	-	-	XP_010105518.1:Peptide transporter [Morus notabilis] >EXC05013.1 Peptide transporter [Morus notabilis]	-	W9SG60|W9SG60_9ROSA:Peptide transporter;OS=Morus notabilis.;|GO:0016021,|GO:0005215,|GO:0006857,

geneid	trend	GO	Pathway	TF	NR	NT	Uniprot
DN123419_c0_g1_i7	DOWN	-	-	-	XP_010682899.1:PREDICTED: uncharacterized protein LOC104897659 [Beta vulgaris subsp. vulgaris]	gi|521503249|gb|GAHZ01027671.1|:TSA: Salicornia europaea Unigene30465_SeCKS transcribed RNA sequence	A0A0J8EWG9|A0A0J8EWG9_BETVU: Uncharacterized protein;OS=Beta vulgaris subsp. vulgaris.;
DN114955_c0_g1_i1	DOWN	[GO:0016788:hydrolase activity, acting on ester bonds]	-	Potri.012G137500.1: Populus trichocarpa|FAR1|FAR1 family protein	XP_010687164.1:PREDICTED: GDSL esterase/lipase At4g18970 [Beta vulgaris subsp. vulgaris] >KMT03853.1 hypothetical protein BVRB_8g188250 [Beta vulgaris subsp. vulgaris]	-	A0A0J8BV85|A0A0J8BV85_BETVU:Uncharacterized protein;OS=Beta vulgaris subsp. vulgaris.;|GO:0016788,

geneid	trend	GO	Pathway	TF	NR	NT	Uniprot
DN123384_c0_g3_i5	DOWN	[GO:0016021:integral component of membrane][GO:0016760:cellulose synthase (UDP-forming) activity][GO:0071555:cell wall organization][GO:0030244:cellulose biosynthetic process]	-	FANhyb_rscf00001203.1.g00002.1:Fragaria x ananassa|LBD|LBD family protein	XP_010675676.1:PREDICTED: cellulose synthaselike protein G2 [Beta vulgaris subsp. vulgaris]>KMT12691.1 hypothetical protein BVRB_4g087890 [Beta vulgaris subsp. vulgaris]	-	A0A0J8F9Z4|A0A0J8F9Z4_BETVU:Uncharacterized protein;OS=Beta vulgaris subsp. vulgaris.;|GO:0016021,|GO:0016760,|GO:0071555,GO:0030244,
DN125680_c0_g1_i4	DOWN	-	-	-	-	-	-
DN75710_c0_g1_i1	DOWN	-	-	-	-	-	-
DN112837_c0_g2_i1	DOWN	[GO:0005874:microtubule][GO:0005819:spindle][GO:0032147:activation of protein kinase activity][GO:0060236:regulation of mitotic spindle organization]	-	-	XP_020535769.1:protein TPX2 isoform X1 [Jatropha curcas]	-	A0A061EE26|A0A061EE26_THECC:TPX2 family protein;OS=Theobroma cacao;|GO:0005874,GO:0005819,|GO:0032147,GO:0060236,

geneid	trend	GO	Pathway	TF	NR	NT	Uniprot
DN126049_c0_g1_i1	DOWN	-	[ko04015:Rap1 signaling pathway][ko04145:Phagosome][ko04210:Apoptosis][ko04390:Hippo signaling pathway][ko04391:Hippo signaling pathway - fly][ko04510:Focal adhesion][ko04520:Adherens junction][ko04530:Tight junction][ko04611:Platelet activation][ko04670:Leukocyte transendothelial migration][ko04745:Phototransduction - fly][ko04810:Regulation of actin cytoskeleton][ko04919:Thyroid hormone signaling pathway][ko04921:Oxytocin signaling pathway][ko05100:Bacterial invasion of epithelial	Peinf101Scf00255g01007.1:Petunia inflata|Trihelix|Trihelix family protein	BAJ90061.1:predicted protein [Hordeum vulgare subsp. vulgare]	gi|109729853|gb|DQ657949.1|:Aedes albopictus actin mRNA, complete cds	A0A1D1YW40|A0A1D1YW40_9ARAE:Actin-2;OS=Anthurium amnicola.;

geneid	trend	GO	Pathway	TF	NR	NT	Uniprot
DN49078_c0_g1_i1	DOWN	[GO:0016021:integral component of membrane][GO:0033179:proton-transporting V-type ATPase, V0 domain][GO:0005774:vacuolar membrane][GO:0015078:proton transmembrane transporter activity][GO:0015991:ATP hydrolysis coupled proton transport]	[ko00190:Oxidative phosphorylation][ko04142:Lysosome][ko04145:Phagosome][ko04721:Synaptic vesicle cycle][ko04966:Collecting duct acid secretion][ko05110:Vibrio cholerae infection][ko05120:Epithelial cell signaling in Helicobacter pylori infection][ko05152:Tuberculosis][ko05323:Rheumatoid arthritis]	TRIUR3_01525-P1:Triticum urartu|C2H2|C2H2 family protein	AFK39667.1:unknown [Lotus japonicus]	-	I3SHC5|I3SHC5_LOTJA:V-type proton ATPase proteolipid subunit;OS=Lotus japonicus;|GO:0016021,GO:0033179,GO:0005774,|GO:0015078,|GO:0015991,
DN114789_c0_g1_i1	DOWN	[GO:0016021:integral component of membrane][GO:0005385:zinc ion transmembrane transporter activity]	-	Achn296391:Actinidia chinensis|NF-YC|NF-YC family protein	KNA22456.1:hypothetical protein SOVF_033360 [Spinacia oleracea]	-	A0A0K9RSC7|A0A0K9RSC7_SPIOL:Uncharacterized protein;OS=Spinacia oleracea;|GO:0016021,|GO:0005385,

geneid	trend	GO	Pathway	TF	NR	NT	Uniprot
DN125680_c0_g1_i15	DOWN	[GO:0043531:ADP binding][GO:0006952:defense response]	[ko04626:Plant-pathogen interaction]	-	XP_010092366.1:Disease resistance protein RPM1 [Morus notabilis] >EXB51042.1 Disease resistance protein RPM1 [Morus notabilis]	-	W9RCT0|W9RCT0_9ROSA:Disease resistance protein RPM1;OS=Morus notabilis.;|GO:0043531,|GO:0006952,
DN94640_c0_g1_i1	DOWN	-	-	Achn114091:Actinidia chinensis|Trihelix|Trihelix family protein	BAJ99614.1:predicted protein [Hordeum vulgare subsp. vulgare]	-	A0A1D1Z5S2|A0A1D1Z5S2_9ARAE:Elongation factor 1-beta;OS=Anthurium amnicola.;
DN127804_c3_g10_i2	DOWN	-	-	-	-	-	-
DN124918_c3_g3_i7	DOWN	[GO:0009966:regulation of signal transduction]	[ko04136:Autophagy - other][ko04138:Autophagy - yeast][ko04140:Autophagy - animal]	-	OMO93629.1:TAP42like protein [Corchorus olitorius]	gi|521457980|gb|GAHZ01062504.1|:TSA: Salicornia europaea Unigene75266_SeCKS transcribed RNA sequence	A0A061G149|A0A061G149_THECC:2A phosphatase associated protein of 46 kD;OS=Theobroma cacao;|GO:0009966,

geneid	trend	GO	Pathway	TF	NR	NT	Uniprot
DN113281_c2_g2_i1	DOWN	[GO:0005634:nucleus][GO:0003677:DNA binding][GO:0006355:regulation of transcription, DNA-templated][GO:0006351:transcription, DNA-templated]	-	C.cajan_02029:Cajanus cajan|NAC|NAC family protein	XP_021767210.1:NAC domaincontaining protein 43like [Chenopodium quinoa]	gi|521578388|gb|GAIA01069764.1|:TSA: Salicornia europaea Unigene84026_Se200S transcribed RNA sequence	F6I2X7|F6I2X7_VITVI:Putative uncharacterized protein;OS=Vitis vinifera;GN=VIT_15s0048g02660 {ECO:0000313|EMBL:CCB61294.1};|GO:0005634,|GO:0003677,|GO:0006355, GO:0006351,
DN114082_c0_g1_i1	DOWN	[GO:0016021:integral component of membrane][GO:0045492:xylan biosynthetic process]	-	Itr_sc000772.1_g00020.1:Ipomoea trifida|FAR1|FAR1 family protein	XP_021774845.1:protein IRX15LIKElike [Chenopodium quinoa]	-	A0A0J8CP22|A0A0J8CP22_BETVU:Uncharacterized protein;OS=Beta vulgaris subsp. vulgaris.;|GO:0016021,|GO:0045492,
DN121767_c1_g1_i2	DOWN	-	-	-	XP_021749610.1:pollenspecific protein SF21like [Chenopodium quinoa]	gi|356564068|ref|XM_003550231.1|:PREDICTED: Glycine max pollen-specific protein SF21-like, transcript variant 2 (LOC100781823),	A0A0J8D1H1|A0A0J8D1H1_BETVU:Uncharacterized protein;OS=Beta vulgaris subsp. vulgaris.;

geneid	trend	GO	Pathway	TF	NR	NT	Uniprot
						mRNA	
DN122170_c0_g1_i1	DOWN	-	-	-	XP_010696340.1:PREDICTED: uncharacterized protein LOC104908870 [Beta vulgaris subsp. vulgaris] >KMS97024.1 hypothetical protein BVRB_7g179400 [Beta vulgaris subsp. vulgaris]	gi|521536378|gb|GAHZ01005417.1|:TSA: Salicornia europaea Unigene5707_SeCKS transcribed RNA sequence	A0A0J8B715|A0A0J8B715_BETVU:Uncharacterized protein;OS=Beta vulgaris subsp. vulgaris.;
DN6055_c0_g1_i1	DOWN	-	-	-	-	-	-

geneid	trend	GO	Pathway	TF	NR	NT	Uniprot
DN127686_c0_g1_i22	DOWN	[GO:0035091:phosphatidylinositol binding]	-	-	XP_010696458.1:PREDICTED: uncharacterized protein LOC104908981 isoform X1 [Beta vulgaris subsp. vulgaris] >KMT18961.1 hypothetical protein BVRB_2g030590 [Beta vulgaris subsp. vulgaris]	gi|356507679|ref|XM_003522544.1|:PREDICTED: Glycine max uncharacterized protein LOC100792151 (LOC100792151), mRNA	A0A0J8FTC4|A0A0J8FTC4_BETVU:Uncharacterized protein;OS=Beta vulgaris subsp. vulgaris.;|GO:0035091,
DN128687_c2_g3_i7	DOWN	[GO:0003824:catalytic activity][GO:0008152:metabolic process]	[ko00130:Ubiquinone and other terpenoid-quinone biosynthesis][ko00360:Phenylalanine metabolism][ko00940:Phenylpropanoid biosynthesis]	Zmw_sc02382.1.g00020.1:Zoysia matrella|bHLH|bHLH family protein	XP_010689117.1:PREDICTED: 4coumarateCoA ligaselike 7 [Beta vulgaris subsp. vulgaris] >KMT02326.1 hypothetical protein BVRB_9g205510 [Beta vulgaris subsp. vulgaris]	gi|359479355|ref|XM_002276317.2|:PREDICTED: Vitis vinifera 4-coumarate--CoA ligase-like 7 (LOC100267198), mRNA	A0A0J8EH45|A0A0J8EH45_BETVU:Uncharacterized protein;OS=Beta vulgaris subsp. vulgaris.;|GO:0003824,|GO:0008152,

geneid	trend	GO	Pathway	TF	NR	NT	Uniprot
DN126783_c1_g1_i5	DOWN	[GO:0008289:lipid binding]	-	RrC8034_p2:Raphanus raphanistrum|DBB|DBB family protein	XP_021836862.1:protein ENHANCED DISEASE RESISTANCE 2like [Spinacia oleracea]	gi|225435337|ref|XR_078201.1|:PREDICTED: Vitis vinifera uncharacterized LOC100250069 (LOC100250069), miscRNA	A0A0K9R802|A0A0K9R802_SPIOL:Uncharacterized protein;OS=Spinacia oleracea;|GO:0008289,
DN116540_c1_g1_i1	DOWN	[GO:0003677:DNA binding]	-	Bv3_067360_mfpk.t1:Beta vulgaris|bHLH|bHLH family protein	XP_010693647.1:PREDICTED: transcription factor bHLH120 [Beta vulgaris subsp. vulgaris] >KMS98935.1 hypothetical protein BVRB_3g067360 [Beta vulgaris subsp. vulgaris]	-	A0A0J8E6W0|A0A0J8E6W0_BETVU: Uncharacterized protein;OS=Beta vulgaris subsp. vulgaris.;|GO:0003677,

geneid	trend	GO	Pathway	TF	NR	NT	Uniprot
DN121350_c3_g1_i6	DOWN	[GO:0048046:apoplast][GO:0005507:copper ion binding][GO:0052716:hydroquinone:oxygen oxidoreductase activity][GO:0016722:oxidoreductase activity, oxidizing metal ions][GO:0046274:lignin catabolic process]	-	Rsa1.0_03034.1_g00001.1:Raphanus sativus|ERF|ERF family protein	XP_015077948.1:PREDICTED: laccase5like [Solanum pennellii]	gi|186520893|ref|NM_120795.4|:Arabidopsis thaliana laccase 13 mRNA, complete cds	K4C5J7|K4C5J7_SOLLC:Laccase;OS=Solanum lycopersicum;|GO:0048046,|GO:0005507,GO:0052716,GO:0016722,|GO:0046274,
DN126419_c2_g6_i13	DOWN	-	-	-	-	-	-
DN110655_c0_g1_i3	DOWN	[GO:0016021:integral component of membrane][GO:0008168:methyltransferase activity][GO:0045492:xylan biosynthetic process]	-	Itr_sc000772.1_g00020.1:Ipomoea trifida|FAR1|FAR1 family protein	XP_010676715.1:PREDICTED: glucuronoxylan 4Omethyltransferase 1like [Beta vulgaris subsp. vulgaris]	gi|521563561|gb|GAIA01074886.1|:TSA: Salicornia europaea Unigene90838_Se200S transcribed RNA sequence	A0A061E3Z1|A0A061E3Z1_THECC:S-adenosyl-L-methionine-dependent methyltransferase mraW;OS=Theobroma cacao;|GO:0016021,|GO:0008168,|GO:0045492,

geneid	trend	GO	Pathway	TF	NR	NT	Uniprot
DN116702_c2_g1_i5	DOWN	-	[ko03015:mRNA surveillance pathway]	-	XP_021751284.1:mRNA cap guanineN7 methyltransferase 2like [Chenopodium quinoa]	-	A0A0J8DWL5|A0A0J8DWL5_BETVU: Uncharacterized protein;OS=Beta vulgaris subsp. vulgaris.;
DN113932_c0_g1_i2	DOWN	[GO:0003676:nucleic acid binding][GO:0008270:zinc ion binding][GO:0015074:DNA integration]	-	-	XP_021866109.1:uncharacterized protein LOC110804808 [Spinacia oleracea]	-	B8YLY6|B8YLY6_MEDTR:Gag-Pol polyprotein;OS=Medicago truncatula;|GO:0003676,GO:0008270,|GO:0015074,

geneid	trend	GO	Pathway	TF	NR	NT	Uniprot
DN123978_c1_g4_i2	DOWN	[GO:0016021:integral component of membrane][GO:0005524:ATP binding][GO:0030247:polysaccharide binding][GO:0004672:protein kinase activity]	[ko04064:NF-kappa B signaling pathway][ko04620:Toll-like receptor signaling pathway][ko04621:NOD-like receptor signaling pathway][ko04624:Toll and Imd signaling pathway][ko04722:Neurotrophin signaling pathway][ko05133:Pertussis][ko05140:Leishmaniasis][ko05142:Chagas disease (American trypanosomiasis)][ko05145:Toxoplasmosis][ko05152:Tuberculosis][ko05162:Measles][ko05164:Influenza A]	-	KMT14770.1:hypothetical protein BVRB_4g075360 [Beta vulgaris subsp. vulgaris]	-	A0A0J8CLW9|A0A0J8CLW9_BETVU: Uncharacterized protein;OS=Beta vulgaris subsp. vulgaris.;|GO:0016021,|GO:0005524,GO:0030247,GO:0004672,

geneid	trend	GO	Pathway	TF	NR	NT	Uniprot
DN112732_c0_g1_i4	DOWN	[GO:0016788:hydrolase activity, acting on ester bonds]	-	Niben101Scf08326g00014.1:Nicotiana benthamiana|C2H2|C2H2 family protein	XP_021720961.1:GDSL esterase/lipase At2g31550like [Chenopodium quinoa]	-	A0A0J8BZC1|A0A0J8BZC1_BETVU:Uncharacterized protein;OS=Beta vulgaris subsp. vulgaris.;|GO:0016788,
DN116075_c2_g7_i1	DOWN	-	-	-	-	-	-
DN126719_c1_g1_i1	DOWN	[GO:0005829:cytosol][GO:0003939:L-iditol 2-dehydrogenase activity][GO:0008270:zinc ion binding]	[ko00040:Pentose and glucuronate interconversions][ko00051:Fructose and mannose metabolism]	-	GAQ80199.1:Sorbitol dehydrogenase [Klebsormidium nitens]	-	M1VE10|M1VE10_CYAM1:NAD-dependent sorbitol dehydrogenase;OS=Cyanidioschyzon merolae;|GO:0005829,|GO:0003939,GO:0008270,
DN116045_c0_g1_i5	DOWN	[GO:0005634:nucleus][GO:0003677:DNA binding][GO:0006355:regulation of transcription, DNA-templated][GO:0006351:transcription, DNA-templated]	-	Bv9_220730_ohkq.t1:Beta vulgaris|B3|B3 family protein	XP_021846843.1:B3 domaincontaining protein Os01g0723500like [Spinacia oleracea]	-	A0A0J8BL74|A0A0J8BL74_BETVU:Uncharacterized protein;OS=Beta vulgaris subsp. vulgaris.;|GO:0005634,|GO:0003677,|G

geneid	trend	GO	Pathway	TF	NR	NT	Uniprot
							O:0006355,GO:0006351,
DN119852_c0_g2_i1	DOWN	[GO:0004842:ubiquitin-protein transferase activity][GO:0008270:zinc ion binding][GO:0042742:defense response to bacterium][GO:0009867:jasmonic acid mediated signaling pathway][GO:0009611:response to wounding]	-	KN540739.1_FGP003:Oryza longistaminata|M-type_MADS|M-type_MADS family protein	XP_010674439.1:PREDICTED: E3 ubiquitinprotein ligase RGLG1 [Beta vulgaris subsp. vulgaris] >XP_010674440.1 PREDICTED: E3 ubiquitinprotein ligase RGLG1 [Beta vulgaris subsp. vulgaris] >KMT14074.1 hypothetical protein BVRB_4g079000 [Beta vulgaris subsp. vulgaris]	-	A0A0J8CPZ4|A0A0J8CPZ4_BETVU:Uncharacterized protein;OS=Beta vulgaris subsp. vulgaris.;|GO:0004842,GO:0008270,|GO:0042742,GO:0009867,GO:0009611,

geneid	trend	GO	Pathway	TF	NR	NT	Uniprot
DN128280_c1_g2_i1	DOWN	[GO:0016021:integral component of membrane][GO:0005524:ATP binding][GO:0005509:calcium ion binding][GO:0030247:polysaccharide binding][GO:0004672:protein kinase activity]	[ko04064:NF-kappa B signaling pathway][ko04620:Toll-like receptor signaling pathway][ko04621:NOD-like receptor signaling pathway][ko04624:Toll and Imd signaling pathway][ko04722:Neurotrophin signaling pathway][ko05133:Pertussis][ko05140:Leishmaniasis][ko05142:Chagas disease (American trypanosomiasis)][ko05145:Toxoplasmosis][ko05152:Tuberculosis][ko05162:Measles][ko05164:Influenza A]	-	KMT14771.1:hypothetical protein BVRB_4g075370 [Beta vulgaris subsp. vulgaris]	-	A0A0J8FG21|A0A0J8FG21_BETVU:Uncharacterized protein;OS=Beta vulgaris subsp. vulgaris.;|GO:0016021,|GO:0005524,GO:0005509,GO:0030247,GO:0004672,

geneid	trend	GO	Pathway	TF	NR	NT	Uniprot
DN123110_c0_g5_i1	DOWN	-	-	-	-	-	-
DN123563_c0_g1_i6	DOWN	[GO:0016272:prefoldin complex][GO:0006457:protein folding]	-	-	XP_021605063.1:prefoldin subunit 1 [Manihot esculenta] >XP_021605064.1 prefoldin subunit 1 [Manihot esculenta] >OAY58676.1 hypothetical protein MANES_02G198400 [Manihot esculenta]	-	A0A0K9QLI8|A0A0K9QLI8_SPIOL:Uncharacterized protein;OS=Spinacia oleracea;|GO:0016272,|GO:0006457,
DN122105_c5_g1_i4	DOWN	[GO:0005634:nucleus][GO:0043565:sequence-specific DNA binding][GO:0003700:DNA-binding transcription factor activity]	-	Bv3_060000_xtmt.t1:Beta vulgaris|HD-ZIP|HD-ZIP family protein	XP_010672636.1:PREDICTED: homeoboxleucine zipper protein HOX11 [Beta vulgaris subsp. vulgaris]	gi|224138097|ref|XM_002322693.1|:Populus trichocarpa predicted protein, mRNA	A0A0J8FHB6|A0A0J8FHB6_BETVU:Uncharacterized protein;OS=Beta vulgaris subsp. vulgaris.;|GO:0005634,|GO:0043565,GO:0003700,

geneid	trend	GO	Pathway	TF	NR	NT	Uniprot
DN126428_c0_g3_i6	DOWN	[GO:0016592:mediator complex]	[ko04919:Thyroid hormone signaling pathway]	-	XP_010674857.1:PREDICTED: mediator of RNA polymerase II transcription subunit 14 [Beta vulgaris subsp. vulgaris] >KMT13627.1 hypothetical protein BVRB_4g082280 [Beta vulgaris subsp. vulgaris]	gi|123718705|emb|AM439399.1|:Vitis vinifera, whole genome shotgun sequence, contig VV78X154827.8, clone ENTAV 115	A0A0J8CJA3|A0A0J8CJA3_BETVU:Uncharacterized protein;OS=Beta vulgaris subsp. vulgaris.;|GO:0016592,|GO:0001104,
DN77094_c0_g1_i1	DOWN	-	-	-	-	-	-

geneid	trend	GO	Pathway	TF	NR	NT	Uniprot
DN127182_c3_g1_i4	DOWN	[GO:0016021:integral component of membrane][GO:0000220:vacuolar proton-transporting V-type ATPase, V0 domain][GO:0015078:proton transmembrane transporter activity][GO:0015991:ATP hydrolysis coupled proton transport]	[ko00190:Oxidative phosphorylation][ko04142:Lysosome][ko04145:Phagosome][ko04721:Synaptic vesicle cycle][ko04966:Collecting duct acid secretion][ko05110:Vibrio cholerae infection][ko05120:Epithelial cell signaling in Helicobacter pylori infection][ko05152:Tuberculosis][ko05323:Rheumatoid arthritis]	FANhyb_rscf00000814.1.g00005.1:Fragaria x ananassa|SRS|SRS family protein	XP_021862439.1:Vtype proton ATPase subunit a3like [Spinacia oleracea]	gi|225458114|ref|XM_002280751.1|:PREDICTED: Vitis vinifera vacuolar proton translocating ATPase 100 kDa subunit-like (LOC100255659), mRNA	A0A0K9QT51|A0A0K9QT51_SPIOL:V-type proton ATPase subunit a;OS=Spinacia oleracea;|GO:0016021,GO:0000220,|GO:0015078,|GO:0015991,
DN104237_c0_g1_i1	DOWN	[GO:0003993:acid phosphatase activity][GO:0046872:metal ion binding]	-	PDK_30s873781g005:Phoenix dactylifera|C3H|C3H family protein	XP_021764750.1:probable purple acid phosphatase 20 [Chenopodium quinoa]	-	A0A0J8C3I1|A0A0J8C3I1_BETVU:Purple acid phosphatase;OS=Beta vulgaris subsp. vulgaris.;|GO:0003993,GO:0046872,

geneid	trend	GO	Pathway	TF	NR	NT	Uniprot
DN112938_c0_g2_i1	DOWN	-	-	-	-	-	-
DN121396_c0_g1_i11	DOWN	[GO:0016021:integral component of membrane]	-	Sp_073850_ixmz.t1:Spinacia oleracea|MYB_related|MYB_related family protein	KHG29087.1:putative amino acid permease 7 [Gossypium arboreum]	gi|356566279|ref|XM_003551313.1|:PREDICTED: Glycine max probable amino acid permease 7-like (LOC100818535), mRNA	A0A0B0PW67|A0A0B0PW67_GOSAR:Putative amino acid permease 7;OS=Gossypium arboreum;|GO:0016021,
DN127349_c1_g6_i4	DOWN	-	-	kfl00029_0340:Klebsormidium flaccidum|C2H2|C2H2 family protein	XP_021866324.1:transcription factor GTE10like isoform X2 [Spinacia oleracea] >KNA24476.1 hypothetical protein SOVF_015290 [Spinacia oleracea]	gi|521480356|gb|GAHZ01050530.1|:TSA: Salicornia europaea Unigene59684_SeCKS transcribed RNA sequence	A0A0K9RY78|A0A0K9RY78_SPIOL:Uncharacterized protein;OS=Spinacia oleracea;
DN87691_c0_g1_i1	DOWN	-	-	-	-	-	-

geneid	trend	GO	Pathway	TF	NR	NT	Uniprot
DN123252_c1_g1_i4	DOWN	[GO:0005634:nucleus][GO:0000977:RNA polymerase II regulatory region sequence-specific DNA binding][GO:0003700:DNA-binding transcription factor activity][GO:0000165:MAPK cascade][GO:0045944:positive regulation of transcription by RNA polymerase II][GO:0006351:transcription, DNA-templated]	-	Bv6_139720_hfgn.t1:Beta vulgaris|MIKC_MADS|MIKC_MADS family protein	XP_010681126.1:PREDICTED: truncated transcription factor CAULIFLOWER A [Beta vulgaris subsp. vulgaris] >KMT08699.1 hypothetical protein BVRB_6g139720 [Beta vulgaris subsp. vulgaris]	gi|32478044|gb|AY306160.1|:Phytolacca americana euAP1 APETALA1-like MADS-box (PaAP1) mRNA, partial cds	A0A0J8C8W8|A0A0J8C8W8_BETVU: Uncharacterized protein;OS=Beta vulgaris subsp. vulgaris.;|GO:0005634,|GO:0000977,GO:0003700,|GO:0000165,GO:0045944,GO:0006351,

geneid	trend	GO	Pathway	TF	NR	NT	Uniprot
DN119781_c0_g1_i14	DOWN	[GO:0016021:integral component of membrane][GO:0005886:plasma membrane][GO:0009506:plasmodesma][GO:0005524:ATP binding][GO:0009678:hydrogen-translocating pyrophosphatase activity][GO:0004427:inorganic diphosphatase activity][GO:0004674:protein serine/threonine kinase activity][GO:0006952:defense response][GO:0006468:protein phosphorylation]	[ko04064:NF-kappa B signaling pathway][ko04620:Toll-like receptor signaling pathway][ko04621:NOD-like receptor signaling pathway][ko04624:Toll and Imd signaling pathway][ko04722:Neurotrophin signaling pathway][ko05133:Pertussis][ko05140:Leishmaniasis][ko05142:Chagas disease (American trypanosomiasis)][ko05145:Toxoplasmosis][ko05152:Tuberculosis][ko05162:Measles][ko05164:Influenza A]	Kaladp0839s0004.1.p:Kalanchoe laxiflora|ERF|ERF family protein	XP_010267803.1:PREDICTED: putative receptorlike protein kinase At4g00960 isoform X2 [Nelumbo nucifera]	gi|224113942|ref|XM_002316586.1|:Populus trichocarpa predicted protein, mRNA	F6HVX5|F6HVX5_VITVI:Putative uncharacterized protein;OS=Vitis vinifera;GN=VIT_10s0071g01200 {ECO:0000313|EMBL:CCB58838.1};|GO:0016021,GO:0005886,GO:0009506,|GO:0005524,GO:0009678,GO:0004427,GO:0004674,|GO:0006952,GO:0006468,GO:0015992,

geneid	trend	GO	Pathway	TF	NR	NT	Uniprot
DN60695_c0_g1_i1	DOWN	-	[ko03010:Ribosome]	-	XP_004296403.1:PREDICTED: 60S acidic ribosomal protein P0like [Fragaria vesca subsp. vesca]	gi|67083830|gb|DQ066213.1|:Ixodes scapularis isolate ISUFL22 60S acidic ribosomal protein P0 mRNA, complete cds	A0A1D1YJ31|A0A1D1YJ31_9ARAE:60S acidic ribosomal protein P0;OS=Anthurium amnicola.;
DN98881_c0_g2_i1	DOWN	-	[ko03010:Ribosome]	-	BAJ85408.1:predicted protein [Hordeum vulgare subsp. vulgare]	-	A0A1D1XIR1|A0A1D1XIR1_9ARAE:60S ribosomal protein L4;OS=Anthurium amnicola.;
DN125332_c2_g1_i1	DOWN	[GO:0005576:extracellular region][GO:0020037:heme binding][GO:0046872:metal ion binding][GO:0004601:peroxidase activity][GO:0042744:hydrogen peroxide catabolic process][GO:0006979:response to oxidative stress]	[ko00940:Phenylpropanoid biosynthesis]	-	XP_010685987.1:PREDICTED: peroxidase 57 [Beta vulgaris subsp. vulgaris] >CAK22416.1 cationic peroxidase [Beta vulgaris] >KMT05460.1 hypothetical protein BVRB_7g175860 [Beta vulgaris subsp. vulgaris]	-	Q1H8N1|Q1H8N1_BETVU:Peroxidase;OS=Beta vulgaris;|GO:0005576,|GO:0020037,GO:0046872,GO:0004601,|GO:0042744,GO:0006979,

geneid	trend	GO	Pathway	TF	NR	NT	Uniprot
DN125974_c0_g1_i8	DOWN	[GO:0005524:ATP binding][GO:0004672:protein kinase activity]	-	-	XP_021764364.1:serine/threonineprotein kinase Nek2like [Chenopodium quinoa] >XP_021764365.1 serine/threonineprotein kinase Nek2like [Chenopodium quinoa]	gi|359479670|ref|XM_003632280.1|:PREDICTED: Vitis vinifera serine/threonine-protein kinase Nek2-like (LOC100852976), mRNA	A0A0J8C938|A0A0J8C938_BETVU:Uncharacterized protein;OS=Beta vulgaris subsp. vulgaris.;|GO:0005524,GO:0004672,
DN121617_c1_g2_i7	DOWN	[GO:0030246:carbohydrate binding][GO:0016853:isomerase activity][GO:0005975:carbohydrate metabolic process]	[ko00010:Glycolysis / Gluconeogenesis]	MDP0000239688:Malus domestica|bZIP|bZIP family protein	XP_021757607.1:putative glucose6phosphate 1epimerase [Chenopodium quinoa]	gi|521740951|gb|GAIA01006084.1|:TSA: Salicornia europaea Unigene6393_Se200S transcribed RNA sequence	A0A0K9R2E5|A0A0K9R2E5_SPIOL:Uncharacterized protein;OS=Spinacia oleracea;|GO:0030246,GO:0016853,|GO:0005975,
DN127701_c2_g2_i1	DOWN	-	-	-	-	-	-

geneid	trend	GO	Pathway	TF	NR	NT	Uniprot
DN126441_c0_g3_i8	DOWN	[GO:0005524:ATP binding][GO:0003883:CTP synthase activity][GO:0044210:'de novo' CTP biosynthetic process][GO:0006541:glutamine metabolic process]	[ko00240:Pyrimidine metabolism]	PK16897.10:Cannabis sativa|YABBY|YABBY family protein	XP_010679044.1:PREDICTED: CTP synthase isoform X1 [Beta vulgaris subsp. vulgaris] >KMT10161.1 hypothetical protein BVRB_5g119220 [Beta vulgaris subsp. vulgaris]	gi|357517334|ref|XM_003628908.1|:Medicago truncatula CTP synthase like protein (MTR_8g070540) mRNA, complete cds	A0A0J8F364|A0A0J8F364_BETVU:CTP synthase;OS=Beta vulgaris subsp. vulgaris.;|GO:0005524,GO:0003883,|GO:0044210,GO:0006541,
DN126459_c2_g2_i1	DOWN	[GO:0016021:integral component of membrane][GO:0005524:ATP binding][GO:0042626:ATPase activity, coupled to transmembrane movement of substances]	-	-	KNA11606.1:hypothetical protein SOVF_133410 [Spinacia oleracea]	gi|521498839|gb|GAHZ01032081.1|:TSA: Salicornia europaea Unigene35368_SeCKS transcribed RNA sequence	A0A0K9QYM4|A0A0K9QYM4_SPIOL:Uncharacterized protein;OS=Spinacia oleracea;|GO:0016021,|GO:0005524,GO:0042626,
DN103259_c0_g2_i2	DOWN	-	-	augustus_masked-scaffold02666-abinit-gene-0.6-mRNA-1:Castanea mollissima|ZF-HD|ZF-HD family protein	OMO61911.1:hypothetical protein COLO4_33302 [Corchorus olitorius]	-	A0A067JT59|A0A067JT59_JATCU:Uncharacterized protein;OS=Jatropha curcas;

geneid	trend	GO	Pathway	TF	NR	NT	Uniprot
DN106285_c0_g1_i2	DOWN	-	-	-	KCW54793.1:hypothetical protein EUGRSUZ_I00734 [Eucalyptus grandis]	-	A0A059ALR5|A0A059ALR5_EUCGR: Uncharacterized protein;OS=Eucalyptus grandis;
DN126449_c0_g5_i12	DOWN	[GO:0005634:nucleus][GO:0004484:mRNA guanylyltransferase activity][GO:0004651:polynucleotide 5'-phosphatase activity][GO:0004725:protein tyrosine phosphatase activity][GO:0008138:protein tyrosine/serine/threonine phosphatase activity][GO:0006370:7-methylguanosine mRNA capping]	[ko03015:mRNA surveillance pathway]	mrna27834.1-v1.0-hybrid:Fragaria vesca|M-type_MADS|M-type_MADS family protein	XP_021774569.1:mRNAcapping enzymelike [Chenopodium quinoa]	gi|521663575|gb|GAIA01035468.1|:TSA: Salicornia europaea Unigene39274_Se200S transcribed RNA sequence	A0A0J8B756|A0A0J8B756_BETVU:Uncharacterized protein;OS=Beta vulgaris subsp. vulgaris.;|GO:0005634,|GO:0004484,GO:0004651,GO:0004725,GO:0008138,|GO:0006370,
DN126113_c0_g3_i1	DOWN	-	-	-	XP_010688460.1:PREDICTED: AP5 complex subunit mu isoform X1 [Beta vulgaris subsp. vulgaris]	gi|255551540|ref|XM_002516770.1|:Ricinus communis conserved hypothetical protein, mRNA	A0A0J8BPW3|A0A0J8BPW3_BETVU: Uncharacterized protein;OS=Beta vulgaris subsp. vulgaris.;

geneid	trend	GO	Pathway	TF	NR	NT	Uniprot
DN104513_c0_g1_i1	DOWN	-	-	-	XP_021862733.1:uncharacterized protein LOC110801657 [Spinacia oleracea]	-	A0A0J8FM09|A0A0J8FM09_BETVU:Uncharacterized protein;OS=Beta vulgaris subsp. vulgaris.;
DN124540_c0_g1_i4	DOWN	[GO:0006950:response to stress]	-	Bv3_057240_hnxc.t1:Beta vulgaris|HSF|HSF family protein	XP_010673093.1:PREDICTED: putative disease resistance RPP13like protein 1 [Beta vulgaris subsp. vulgaris] >KMT15250.1 hypothetical protein BVRB_3g063590 [Beta vulgaris subsp. vulgaris]	-	A0A0J8CNA5|A0A0J8CNA5_BETVU: Uncharacterized protein;OS=Beta vulgaris subsp. vulgaris.;|GO:0006950,

geneid	trend	GO	Pathway	TF	NR	NT	Uniprot
DN123231_c0_g4_i1	DOWN	[GO:0005524:ATP binding][GO:0003777:microtubule motor activity][GO:0007018:microtubule-based movement]	-	Araip.YX9Q9:Arachis ipaensis|MYB_related|MYB_related family protein	XP_010674755.1:PREDICTED: kinesinlike protein KIN14S isoform X1 [Beta vulgaris subsp. vulgaris] >KMT13776.1 hypothetical protein BVRB_4g081510 isoform B [Beta vulgaris subsp. vulgaris]	-	A0A0J8CJA0|A0A0J8CJA0_BETVU:Uncharacterized protein;OS=Beta vulgaris subsp. vulgaris.;|GO:0005524,GO:0003777,|GO:0007018,

geneid	trend	GO	Pathway	TF	NR	NT	Uniprot
DN125968_c2_g5_i14	DOWN	[GO:0000794:condensed nuclear chromosome][GO:0005737:cytoplasm][GO:0030870:Mre11 complex][GO:0000790:nuclear chromatin][GO:0000784:nuclear chromosome, telomeric region][GO:0035861:site of double-strand break][GO:0004017:adenylate kinase activity][GO:0016887:ATPase activity][GO:0003691:double-stranded telomeric DNA binding][GO:0051880:G-quadruplex DNA binding][GO:0043047:single-stranded telomeric DNA binding][GO:0070192:chromosome organization involved in meiotic cell cycle][GO:0032508:DNA duplex unwinding][GO:0000724:double-strand break repair via homologous recombination][GO:0090305:nucleic acid phosphodiester bond hydrolysis][GO:0007131:reciprocal meiotic recombination][GO:0016233:telomere capping][GO:0000722:telomere maintenance via recombination][GO:0007004:telomere maintenance via telomerase]	[ko03440:Homologous recombination][ko03450:Non-homologous end-joining][ko04218:Cellular senescence]	-	XP_018844064.1:PREDICTED: DNA repair protein RAD50 [Juglans regia]	gi|255563609|ref|XM_002522761.1|:Ricinus communis DNA repair protein RAD50, putative, mRNA	B9GFJ5|B9GFJ5_POPTR:DNA repair-recombination family protein;OS=Populus trichocarpa;|GO:0000794,GO:0005737,GO:0030870,GO:0000790,GO:0000784,GO:0035861,|GO:0004017,GO:0016887,GO:0003691,GO:0051880,GO:0043047,|GO:0070192,GO:0032508,GO:0000724,GO:0090305,GO:0007131,GO:0016233,GO:0000722,GO:0007004,

geneid	trend	GO	Pathway	TF	NR	NT	Uniprot
DN92872_c0_g1_i1	DOWN	-	-	-	-	-	-
DN118119_c7_g6_i3	DOWN	-	-	-	-	-	-
DN128315_c7_g4_i8	DOWN	-	-	-	-	-	-
DN76877_c0_g1_i1	DOWN	[GO:0016021:integral component of membrane]	-	GSMUA_Achr1P00070_001:Musa acuminata|ERF|ERF family protein	XP_007021340.2:PREDICTED: uncharacterized protein LOC18593872 [Theobroma cacao]	gi|38016520|gb|AY429438.1|:Gossypium barbadense fiber protein Fb34 mRNA, complete cds	A0A061F673|A0A061F673_THECC:Uncharacterized protein;OS=Theobroma cacao;|GO:0016021,
DN123531_c3_g3_i1	DOWN	[GO:0009507:chloroplast][GO:0005886:plasma membrane][GO:0051287:NAD binding][GO:0050136:NADH dehydrogenase (quinone) activity][GO:0048038:quinone binding][GO:0006810:transport]	[ko00190:Oxidative phosphorylation]	-	AFU94920.1:NdhH, partial (chloroplast) [Euphorbia maculata]	gi|290489827|gb|GQ998381.1|:Heuchera sanguinea voucher FLAS:M.J. Moore 329 NADH-plastoquinone oxidoreductase subunit 7 protein (ndhH) gene, complete cds; chloroplast	K7P8L9|K7P8L9_VACMA:NADH-quinone oxidoreductase subunit D;OS=Vaccinium macrocarpon;|GO:0009507,GO:0005886,|GO:0051287,GO:0050136,GO:0048038,|GO:0006810,

geneid	trend	GO	Pathway	TF	NR	NT	Uniprot
DN115836_c0_g1_i1	DOWN	[GO:0046658:anchored component of plasma membrane][GO:0005774:vacuolar membrane]	-	GSVIVT01030085001:Vitis vinifera|NF-YB|NF-YB family protein	XP_010689281.1:PREDICTED: fasciclinlike arabinogalactan protein 2 [Beta vulgaris subsp. vulgaris] >KMT02265.1 hypothetical protein BVRB_9g206520 [Beta vulgaris subsp. vulgaris]	-	A0A0J8BLI2|A0A0J8BLI2_BETVU:Uncharacterized protein;OS=Beta vulgaris subsp. vulgaris.;|GO:0046658,GO:0005774,
DN104427_c0_g1_i1	DOWN	-	-	-	XP_021727926.1:nucleosome assembly protein 1;3like [Chenopodium quinoa]	-	A0A1D1Y7D0|A0A1D1Y7D0_9ARAE: Nucleosome assembly protein 1-like 4;OS=Anthurium amnicola.;
DN85581_c0_g1_i1	DOWN	-	-	-	-	-	-

geneid	trend	GO	Pathway	TF	NR	NT	Uniprot
DN114093_c5_g1_i2	DOWN	[GO:0005737:cytoplasm][GO:0005524:ATP binding][GO:0004825:methionine-tRNA ligase activity][GO:0000049:tRNA binding][GO:0006431:methionyl-tRNA aminoacylation]	[ko00450:Selenocompound metabolism][ko00970:Aminoacyl-tRNA biosynthesis]	-	CDP11143.1:unnamed protein product [Coffea canephora]	-	A0A068UU54|A0A068UU54_COFCA: Uncharacterized protein;OS=Coffea canephora;|GO:0005737,|GO:0005524,GO:0004825,GO:0000049,|GO:0006431,
DN118199_c0_g4_i3	DOWN	[GO:0016021:integral component of membrane]	-	-	XP_021826195.1:uncharacterized protein LOC110767074 [Prunus avium] >XP_021826196.1 uncharacterized protein LOC110767074 [Prunus avium]	gi|521533120|gb|GAHZ01006740.1|:TSA : Salicornia europaea Unigene7047_SeCKS transcribed RNA sequence	M5XDJ5|M5XDJ5_PRUPE:Uncharacterized protein;OS=Prunus persica;|GO:0016021,
DN104600_c0_g1_i3	DOWN	[GO:0046872:metal ion binding][GO:0030001:metal ion transport]	-	-	XP_010671307.1:PREDICTED: heavy metalassociated isoprenylated plant protein 28 [Beta vulgaris subsp. vulgaris]	-	A0A151RS08|A0A151RS08_CAJCA:Uncharacterized protein;OS=Cajanus cajan;|GO:0046872,|GO:0030001,

geneid	trend	GO	Pathway	TF	NR	NT	Uniprot
DN123436_c3_g5_i4	DOWN	[GO:0016021:integral component of membrane][GO:0008324:cation transmembrane transporter activity]	-	-	XP_021650894.1:probable cation transporter HKT7 [Hevea brasiliensis]	gi|449466066|ref|XM_004150700.1|:PREDICTED: Cucumis sativus cation transporter HKT8-like (LOC101204165), partial mRNA	Q84UZ6|Q84UZ6_MESCR:High affinity potassium transporter 2;OS=Mesembryanthemum crystallinum;|GO:0016021,|GO:0008324,
DN122031_c1_g2_i2	DOWN	[GO:0016021:integral component of membrane][GO:0016491:oxidoreductase activity]	[ko00600:Sphingolipid metabolism]	-	XP_010668480.1:PREDICTED: 3dehydrosphinganine reductase TSC10Alike [Beta vulgaris subsp. vulgaris]	-	A0A0K9QGV9|A0A0K9QGV9_SPIOL: Uncharacterized protein;OS=Spinacia oleracea;|GO:0016021,|GO:0016491,
DN116708_c3_g3_i2	DOWN	[GO:0016021:integral component of membrane][GO:0005886:plasma membrane]	-	-	XP_010682994.1:PREDICTED: CASPlike protein 1F2 [Beta vulgaris subsp. vulgaris]	-	A0A061EE21|A0A061EE21_THECC:CASP-like protein;OS=Theobroma cacao;|GO:0016021, GO:0005886,

geneid	trend	GO	Pathway	TF	NR	NT	Uniprot
DN124061_c 1_g2_i1	DOWN	[GO:0005634:nucleus][GO:0003677: DNA binding]	-	Bv6_150690_wmrr.t 1:Beta vulgaris|SBP|SBP family protein	XP_010682751.1:P REDICTED: squamosa promoterbindinglike protein 2 [Beta vulgaris subsp. vulgaris] >XP_010682752.1 PREDICTED: squamosa promoterbindinglike protein 2 [Beta vulgaris subsp. vulgaris] >XP_010682753.1 PREDICTED: squamosa promoterbindinglike protein 2 [Beta vulgaris subsp. vulgaris] >XP_010682754.1 PREDICTED: squamosa promoterbindinglike protein 2 [Beta vulgaris subsp. vulgaris] >XP_010682755.1 PREDICTED: squamosa	gi|521684943|gb|GA IA01025054.1|:TSA: Salicornia europaea Unigene26886_Se20 0S transcribed RNA sequence	A0A0J8C109|A0A0 J8C109_BETVU:Un characterized protein;OS=Beta vulgaris subsp. vulgaris.;|GO:00056 34,|GO:0003677,

geneid	trend	GO	Pathway	TF	NR	NT	Uniprot
DN115465_c0_g3_i1	DOWN	[GO:0009317:acetyl-CoA carboxylase complex][GO:0003989:acetyl-CoA carboxylase activity][GO:0006633:fatty acid biosynthetic process]	[ko00061:Fatty acid biosynthesis][ko00620:Pyruvate metabolism][ko00640:Propanoate metabolism][ko00720:Carbon fixation pathways in prokaryotes]	-	XP_021740179.1:biotin carboxyl carrier protein of acetylCoA carboxylase 1, chloroplasticlike [Chenopodium quinoa]	gi|381342490|ref|NM_001248245.2|:Glycine max biotin carboxyl carrier protein subunit (ACCB-2), mRNA	A0A0K9QZ36|A0A0K9QZ36_SPIOL:Uncharacterized protein;OS=Spinacia oleracea;|GO:0009317,|GO:0003989,|GO:0006633,
DN124723_c0_g2_i7	DOWN	-	-	-	XP_021856855.1:DNA repair RAD52like protein 1, mitochondrial [Spinacia oleracea] >KNA05357.1 hypothetical protein SOVF_191090 [Spinacia oleracea]	gi|224127741|ref|XM_002329130.1|:Populus trichocarpa predicted protein, mRNA	A0A0K9QDK3|A0A0K9QDK3_SPIOL: Uncharacterized protein;OS=Spinacia oleracea;
DN117053_c0_g1_i4	DOWN	[GO:0004425:indole-3-glycerol-phosphate synthase activity][GO:0006568:tryptophan metabolic process]	[ko00400:Phenylalanine, tyrosine and tryptophan biosynthesis]	-	XP_021715193.1:indole3glycerol phosphate synthase, chloroplasticlike isoform X2 [Chenopodium quinoa]	gi|357162898|ref|XM_003579510.1|:PREDICTED: Brachypodium distachyon indole-3-glycerol phosphate synthase,	A0A0K9Q9F6|A0A0K9Q9F6_SPIOL:Uncharacterized protein;OS=Spinacia oleracea;|GO:0004425,|GO:0006568,

geneid	trend	GO	Pathway	TF	NR	NT	Uniprot
						chloroplastic-like, transcript variant 1 (LOC100842503), mRNA	
DN120030_c1_g1_i7	DOWN	[GO:0005524:ATP binding][GO:0004672:protein kinase activity]	-	FANhyb_rscf00000217.1.g00006.1:Fragaria x ananassa|WRKY|WRKY family protein	XP_021716533.1:serine/threonineprotein kinase RIPKlike [Chenopodium quinoa]	gi|53857146|gb|AY220481.2|:Nicotiana tabacum Avr9/Cf-9 induced kinase 1 (ACIK1) mRNA, complete cds	A0A0K9S302|A0A0K9S302_SPIOL:Uncharacterized protein;OS=Spinacia oleracea;|GO:0005524,GO:0004672,
DN117093_c4_g2_i1	DOWN	-	-	-	-	-	-
DN127573_c4_g9_i1	DOWN	[GO:0016021:integral component of membrane][GO:0005524:ATP binding][GO:0004672:protein kinase activity]	-	FANhyb_rscf00000217.1.g00006.1:Fragaria x ananassa|WRKY|WRKY family protein	XP_010685195.1:PREDICTED: protein STRUBBELIGRECEPTOR FAMILY 7 [Beta vulgaris subsp. vulgaris] >KMT04712.1 hypothetical protein BVRB_7g168940 [Beta vulgaris subsp.	-	A0A0J8BU90|A0A0J8BU90_BETVU:Uncharacterized protein;OS=Beta vulgaris subsp. vulgaris.;|GO:0016021,|GO:0005524,GO:0004672,

geneid	trend	GO	Pathway	TF	NR	NT	Uniprot
					vulgaris]		
DN115656_c1_g1_i1	DOWN	-	-	-	XP_021724299.1:uncharacterized protein LOC110691654 [Chenopodium quinoa]	-	A0A0J8B601|A0A0J8B601_BETVU:Uncharacterized protein;OS=Beta vulgaris subsp. vulgaris.;
DN127819_c3_g5_i2	DOWN	[GO:0003978:UDP-glucose 4-epimerase activity][GO:0006012:galactose metabolic process]	[ko00052:Galactose metabolism][ko00520:Amino sugar and nucleotide sugar metabolism]	-	XP_020697880.1:bifunctional UDPglucose 4epimerase and UDPxylose 4epimerase 1like [Dendrobium catenatum]	gi|139001603|dbj|AB272752.1|:Malus x domestica UGE mRNA for UDP-galactose, complete cds	A0A0K9Q8S6|A0A0K9Q8S6_SPIOL:Uncharacterized protein;OS=Spinacia oleracea;|GO:0003978,|GO:0006012,

geneid	trend	GO	Pathway	TF	NR	NT	Uniprot
DN127630_c1_g7_i7	DOWN	[GO:0005737:cytoplasm][GO:0008234:cysteine-type peptidase activity][GO:0006914:autophagy][GO:0015031:protein transport]	[ko04136:Autophagy - other][ko04138:Autophagy - yeast][ko04140:Autophagy - animal]	-	XP_021855598.1:cysteine protease ATG4like isoform X2 [Spinacia oleracea] >KNA05567.1 hypothetical protein SOVF_189160 isoform A [Spinacia oleracea]	-	A0A0K9QE75|A0A0K9QE75_SPIOL:Uncharacterized protein;OS=Spinacia oleracea;|GO:0005737,|GO:0008234,|GO:0006914,GO:0015031,
DN128192_c3_g4_i3	DOWN	[GO:0006355:regulation of transcription, DNA-templated]	-	Neem_21187_f_2:Azadirachta indica|FAR1|FAR1 family protein	XP_010677651.1:PREDICTED: protein FAR1RELATED SEQUENCE 5like [Beta vulgaris subsp. vulgaris]	-	M5X6N3|M5X6N3_PRUPE:Uncharacterized protein;OS=Prunus persica;|GO:0006355,

geneid	trend	GO	Pathway	TF	NR	NT	Uniprot
DN116154_c0_g1_i2	DOWN	[GO:0000139:Golgi membrane][GO:0016021:integral component of membrane][GO:0047262:polygalacturonate 4-alpha-galacturonosyltransferase activity][GO:0071555:cell wall organization][GO:0045489:pectin biosynthetic process]	[ko00520:Amino sugar and nucleotide sugar metabolism]	Itr_sc000159.1_g00028.1:Ipomoea trifida|MIKC_MADS|MIKC_MADS family protein	XP_021757199.1:probable galacturonosyltransferase 11 [Chenopodium quinoa]	gi|225456535|ref|XM_002262822.1|:PREDICTED: Vitis vinifera probable galacturonosyltransferase 11-like (LOC100262181), mRNA	A0A0J8F4T6|A0A0J8F4T6_BETVU:Hexosyltransferase;OS=Beta vulgaris subsp. vulgaris.;|GO:0000139,GO:0016021,|GO:0047262,|GO:0071555,GO:0045489,
DN123436_c3_g5_i1	DOWN	[GO:0016021:integral component of membrane][GO:0008324:cation transmembrane transporter activity]	-	-	ASM90218.1:sodium transporter HKT1 [Sesuvium portulacastrum]	gi|449466066|ref|XM_004150700.1|:PREDICTED: Cucumis sativus cation transporter HKT8-like (LOC101204165), partial mRNA	A0A0J8BPU8|A0A0J8BPU8_BETVU:Uncharacterized protein;OS=Beta vulgaris subsp. vulgaris.;|GO:0016021,|GO:0008324,
DN124693_c0_g3_i13	DOWN	[GO:0005634:nucleus][GO:0003677:DNA binding][GO:0006351:transcription, DNA-templated]	-	-	XP_021861170.1:probable mediator of RNA polymerase II transcription subunit 26b isoform X1 [Spinacia oleracea]	gi|478166522|gb|GAHK01010081.1|:TSA: Jatropha curcas CL3999.Contig1_JC-CK_1A transcribed RNA sequence	A0A0K9Q9Q3|A0A0K9Q9Q3_SPIOL:Uncharacterized protein;OS=Spinacia oleracea;|GO:0005634,|GO:0003677,|G

geneid	trend	GO	Pathway	TF	NR	NT	Uniprot
							O:0006351,
DN125764_c 1_g1_i7	DOWN	[GO:0016021:integral component of membrane][GO:0008289:lipid binding][GO:0006355:regulation of transcription, DNA-templated][GO:0006950:respon se to stress]	-	-	XP_010692291.1:P REDICTED: synaptotagmin5 [Beta vulgaris subsp. vulgaris] >XP_010692292.1 PREDICTED: synaptotagmin5 [Beta vulgaris subsp. vulgaris] >KMT00133.1 hypothetical protein BVRB_1g019550 [Beta vulgaris subsp. vulgaris]	gi|521514641|gb|GA HZ01016610.1|:TSA : Salicornia europaea Unigene17495_SeC KS transcribed RNA sequence	A0A0J8BJ99|A0A0J 8BJ99_BETVU:Unc haracterized protein;OS=Beta vulgaris subsp. vulgaris.;|GO:00160 21,|GO:0008289,|G O:0006355,GO:0006 950,

geneid	trend	GO	Pathway	TF	NR	NT	Uniprot
DN72576_c0_g1_i1	DOWN	-	[ko00190:Oxidative phosphorylation][ko05010:Alzheimer's disease][ko05012:Parkinson's disease][ko05016:Huntington's disease]	-	ASL24776.1:mitochondrial ATP synthase subunit 9 [Allium cepa]	gi|338224325|gb|HM217802.1|:Scylla paramamosain ATP synthase c-subunit-like mRNA, partial sequence	A0A1D1Y9C8|A0A1D1Y9C8_9ARAE: ATP synthase lipid-binding protein, mitochondrial;OS=Anthurium amnicola.;
DN123110_c0_g2_i4	DOWN	[GO:0004866:endopeptidase inhibitor activity]	-	-	XP_021722896.1:kunitz trypsin inhibitor 2like [Chenopodium quinoa]	-	A0A0J8C0T9|A0A0J8C0T9_BETVU:Uncharacterized protein;OS=Beta vulgaris subsp. vulgaris.;|GO:0004866,
DN126875_c1_g1_i2	DOWN	[GO:0005622:intracellular][GO:0005215:transporter activity]	-	Jcr4S00429.50:Jatropha curcas|Trihelix|Trihelix family protein	XP_010685011.1:PREDICTED: phosphatidylinositol/phosphatidylcholine transfer protein SFH9 isoform X1 [Beta vulgaris subsp. vulgaris]	gi|359479895|ref|XM_002271070.2|:PREDICTED: Vitis vinifera uncharacterized LOC100265430 (LOC100265430), mRNA	A0A0K9RAM9|A0A0K9RAM9_SPIOL:Uncharacterized protein;OS=Spinacia oleracea;|GO:0005622,|GO:0005215,

geneid	trend	GO	Pathway	TF	NR	NT	Uniprot
DN102195_c0_g2_i1	DOWN	[GO:0016491:oxidoreductase activity]	[ko00521:Streptomycin biosynthesis][ko00562:Inositol phosphate metabolism]	-	GAQ79372.1:hypothetical protein KFL_000290220 [Klebsormidium nitens]	-	W2LW60|W2LW60_PHYPR:Uncharacterized protein;OS=Phytophthora parasitica;|GO:0016491,
DN119427_c0_g1_i1	DOWN	[GO:0016021:integral component of membrane][GO:0055085:transmembrane transport]	-	Jcr4S00206.70:Jatropha curcas|FAR1|FAR1 family protein	XP_010680628.1:PREDICTED: protein PINLIKES 7 isoform X1 [Beta vulgaris subsp. vulgaris] >XP_010680629.1 PREDICTED: protein PINLIKES 7 isoform X1 [Beta vulgaris subsp. vulgaris] >XP_019105728.1 PREDICTED: protein PINLIKES 7 isoform X1 [Beta vulgaris subsp. vulgaris]	gi|521703437|gb|GAIA01016206.1|:TSA: Salicornia europaea Unigene16917_Se200S transcribed RNA sequence	A0A0J8C515|A0A0J8C515_BETVU:Uncharacterized protein;OS=Beta vulgaris subsp. vulgaris.;|GO:0016021,|GO:0055085,

geneid	trend	GO	Pathway	TF	NR	NT	Uniprot
					>KMT08875.1 hypothetical protein BVRB_6g135980 [Beta vulgaris subsp. vulgaris]		
DN106393_c1_g1_i3	DOWN	-	-	-	-	-	-

geneid	trend	GO	Pathway	TF	NR	NT	Uniprot
DN108398_c0_g1_i1	DOWN	[GO:0005618:cell wall][GO:0045330:aspartyl esterase activity][GO:0030599:pectinesterase activity][GO:0042545:cell wall modification][GO:0045490:pectin catabolic process]	-	MDP0000320566:Malus domestica|NAC|NAC family protein	XP_010695152.1:PREDICTED: probable pectinesterase 68 [Beta vulgaris subsp. vulgaris]	-	A0A0J8B983|A0A0J8B983_BETVU:Pectinesterase;OS=Beta vulgaris subsp. vulgaris.;|GO:0005618,|GO:0045330,GO:0030599,|GO:0042545,GO:0045490,
DN115244_c0_g2_i4	DOWN	-	-	-	-	-	-
DN119130_c1_g1_i1	DOWN	[GO:0003854:3-beta-hydroxy-delta5-steroid dehydrogenase activity][GO:0006694:steroid biosynthetic process]	-	Rsa1.0_02839.1_g00002.1:Raphanus sativus|LBD|LBD family protein	XP_021765635.1:cinnamoylCoA reductaselike SNL6 isoform X1 [Chenopodium quinoa]	gi|521750705|gb|GAIA01000796.1|:TSA: Salicornia europaea Unigene812_Se200S transcribed RNA sequence	A0A0J8B9H1|A0A0J8B9H1_BETVU:Uncharacterized protein;OS=Beta vulgaris subsp. vulgaris.;|GO:0003854,|GO:0006694,
DN126377_c1_g3_i3	DOWN	[GO:0016021:integral component of membrane]	-	-	XP_021851058.1:uncharacterized protein LOC110790582 [Spinacia oleracea] >KNA13173.1 hypothetical protein SOVF_118940	gi|225458170|ref|XM_002281048.1|:PREDICTED: Vitis vinifera uncharacterized LOC100257473 (LOC100257473),	A0A0K9R0Z2|A0A0K9R0Z2_SPIOL:Uncharacterized protein;OS=Spinacia oleracea;|GO:0016021,

geneid	trend	GO	Pathway	TF	NR	NT	Uniprot
					[Spinacia oleracea]	mRNA	
DN128491_c1_g1_i14	DOWN	[GO:0005524:ATP binding][GO:0003676:nucleic acid binding]	[ko03040:Spliceosome]	-	XP_010670756.1:PREDICTED: DExHbox ATPdependent RNA helicase DExH12like [Beta vulgaris subsp. vulgaris]	gi|478166734|gb|GAHK01009869.1|:TSA: Jatropha curcas CL3885.Contig1_JC-CK_1A transcribed RNA sequence	A0A0J8CXP5|A0A0J8CXP5_BETVU:Uncharacterized protein;OS=Beta vulgaris subsp. vulgaris.;|GO:0005524,GO:0003676,
DN106203_c0_g1_i1	DOWN	[GO:0005840:ribosome][GO:0042254:ribosome biogenesis]	[ko03010:Ribosome]	-	ACG24489.1:60S ribosomal protein L7a [Zea mays]	-	B6SI06|B6SI06_MAIZE:60S ribosomal protein L7a;OS=Zea mays;|GO:0005840,|GO:0042254,
DN125951_c0_g2_i6	DOWN	-	-	LOC_Os11g12360.1:Oryza sativa subsp. japonica|M-type_MADS|M-type_MADS family protein	XP_019163187.1:PREDICTED: uncharacterized protein LOC109159542 [Ipomoea nil]	-	A0A151S6U3|A0A151S6U3_CAJCA:Uncharacterized protein;OS=Cajanus cajan;

geneid	trend	GO	Pathway	TF	NR	NT	Uniprot
DN116027_c5_g3_i2	DOWN	-	-	-	-	-	-
DN109871_c0_g5_i1	DOWN	-	-	-	XP_010683989.1:PREDICTED: uncharacterized protein LOC104898591 [Beta vulgaris subsp. vulgaris] >KMT06460.1 hypothetical protein BVRB_7g161290 [Beta vulgaris subsp. vulgaris]	-	A0A0J8BY75|A0A0J8BY75_BETVU:Uncharacterized protein;OS=Beta vulgaris subsp. vulgaris.;
DN127655_c2_g2_i5	DOWN	[GO:0005886:plasma membrane][GO:0005524:ATP binding][GO:0004672:protein kinase activity][GO:0004842:ubiquitin-protein transferase activity]	-	Pbr031344.1:Pyrus bretschneideri|C2H2|C2H2 family protein	XP_021765089.1:U box domaincontaining protein 32like isoform X3 [Chenopodium quinoa]	-	A0A0J8CWA0|A0A0J8CWA0_BETVU:Uncharacterized protein;OS=Beta vulgaris subsp. vulgaris.;|GO:0005886,|GO:0005524,GO:0004672,GO:0004842,
DN110086_c	DOWN	-	-	-	-	-	-

geneid	trend	GO	Pathway	TF	NR	NT	Uniprot
0_g1_i2							
DN113281_c2_g2_i3	DOWN	[GO:0005634:nucleus][GO:0003677:DNA binding][GO:0006355:regulation of transcription, DNA-templated][GO:0006351:transcription, DNA-templated]	-	Thecc1EG005047t1:Theobroma cacao|NAC|NAC family protein	XP_021767210.1:NAC domaincontaining protein 43like [Chenopodium quinoa]	gi|521578388|gb|GAIA01069764.1|:TSA: Salicornia europaea Unigene84026_Se200S transcribed RNA sequence	F6I2X7|F6I2X7_VITVI:Putative uncharacterized protein;OS=Vitis vinifera;GN=VIT_15s0048g02660 {ECO:0000313|EMBL:CCB61294.1};|GO:0005634,|GO:0003677,|GO:0006355, GO:0006351,
DN117146_c6_g4_i2	DOWN	[GO:0005576:extracellular region][GO:0030145:manganese ion binding][GO:0045735:nutrient reservoir activity]	-	678365117:Utricularia gibba|bZIP|bZIP family protein	XP_010672578.1:PREDICTED: putative germinlike protein 21 [Beta vulgaris subsp. vulgaris] >KMT20683.1 hypothetical protein BVRB_1g006580 [Beta vulgaris subsp. vulgaris]	-	A0A0J8D8V3|A0A0J8D8V3_BETVU:Uncharacterized protein;OS=Beta vulgaris subsp. vulgaris.;|GO:0005576,|GO:0030145,GO:0045735,

geneid	trend	GO	Pathway	TF	NR	NT	Uniprot
DN128036_c10_g1_i13	DOWN	[GO:0030246:carbohydrate binding][GO:0016829:lyase activity][GO:0005975:carbohydrate metabolic process]	-	-	XP_021631259.1:rhamnogalacturonate lyase Blike [Manihot esculenta] >OAY35694.1 hypothetical protein MANES_12G122300 [Manihot esculenta]	-	B9SML0|B9SML0_RICCO:Lyase, putative;OS=Ricinus communis;|GO:0030246,GO:0016829,|GO:0005975,
DN119303_c7_g1_i1	DOWN	-	-	-	XP_010676059.1:PREDICTED: BURP domain protein USPL1like [Beta vulgaris subsp. vulgaris]	-	A0A0J8CLM4|A0A0J8CLM4_BETVU:Uncharacterized protein;OS=Beta vulgaris subsp. vulgaris.;
DN125284_c2_g1_i12	DOWN	-	-	-	XP_021749540.1:EVI5like protein [Chenopodium quinoa]	gi|521477194|gb|GAHZ01053691.1|:TSA: Salicornia europaea Unigene63510_SeCKS transcribed RNA sequence	A0A0J8D2E2|A0A0J8D2E2_BETVU:Uncharacterized protein;OS=Beta vulgaris subsp. vulgaris.;
DN111067_c0_g3_i1	DOWN	-	-	Sp_204350_kxxj.t1:Spinacia oleracea|bHLH|bHLH family protein	XP_021722565.1:transcription factor ALClike [Chenopodium	-	A0A0K9QBM4|A0A0K9QBM4_SPIOL:Uncharacterized protein;OS=Spinacia

geneid	trend	GO	Pathway	TF	NR	NT	Uniprot
					quinoa]		oleracea;
DN110344_c0_g1_i1	DOWN	[GO:0016788:hydrolase activity, acting on ester bonds][GO:0008152:metabolic process]	[ko00562:Inositol phosphate metabolism][ko00564:Glycerophospholipid metabolism][ko00565:Ether lipid metabolism][ko02024:Quorum sensing][ko04919:Thyroid hormone signaling pathway]	Csa09g050690.1:Camelina sativa|NF-YC|NF-YC family protein	XP_021735551.1:nonspecific phospholipase C3like [Chenopodium quinoa]	-	A0A067KX62|A0A067KX62_JATCU: Uncharacterized protein;OS=Jatropha curcas;|GO:0016788,|GO:0008152,
DN119596_c2_g1_i4	DOWN	[GO:0004185:serine-type carboxypeptidase activity]	-	Sme2.5_00187.1_g00004.1:Solanum melongena|MYB_related|MYB_related family protein	XP_010688684.1:PREDICTED: serine carboxypeptidaselike 34 [Beta vulgaris subsp. vulgaris]	gi|356538554|ref|XM_003537720.1|:PREDICTED: Glycine max serine carboxypeptidase-like 34-like (LOC100781503), mRNA	A0A0J8BMX7|A0A0J8BMX7_BETVU: Carboxypeptidase;OS=Beta vulgaris subsp. vulgaris.;|GO:0004185,

geneid	trend	GO	Pathway	TF	NR	NT	Uniprot
DN116024_c1_g2_i11	DOWN	[GO:0005634:nucleus][GO:0003677:DNA binding][GO:0006355:regulation of transcription, DNA-templated][GO:0006351:transcription, DNA-templated]	-	AHYPO_009703-RA:Amaranthus hypochondriacus|B3|B3 family protein	XP_021728607.1:B3 domaincontaining protein At2g36080like isoform X2 [Chenopodium quinoa]	-	F6HKN6|F6HKN6_VITVI:Putative uncharacterized protein;OS=Vitis vinifera;GN=VIT_08s0007g02810 {ECO:0000313|EMBL:CCB55458.1};|GO:0005634,|GO:0003677,|GO:0006355, GO:0006351,
DN120889_c0_g2_i5	DOWN	-	-	-	-	-	-
DN126983_c6_g7_i1	DOWN	[GO:0008171:O-methyltransferase activity]	[ko00940:Phenylpropanoid biosynthesis]	-	XP_021844176.1:caffeic acid 3Omethyltransferase like [Spinacia oleracea]	-	A0A0K9QFL0|A0A0K9QFL0_SPIOL:Uncharacterized protein;OS=Spinacia oleracea;|GO:0008171,

geneid	trend	GO	Pathway	TF	NR	NT	Uniprot
DN125322_c0_g1_i4	DOWN	[GO:0022627:cytosolic small ribosomal subunit][GO:0003735:structural constituent of ribosome][GO:0000028:ribosomal small subunit assembly][GO:0006412:translation]	[ko03010:Ribosome]	-	XP_010685248.1:PREDICTED: 40S ribosomal protein SA [Beta vulgaris subsp. vulgaris] >KMT04768.1 hypothetical protein BVRB_7g169470 [Beta vulgaris subsp. vulgaris]	gi|449516226|ref|XM_004165101.1|:PREDICTED: Cucumis sativus 40S ribosomal protein SA-like (LOC101212340), mRNA	A0A0J8BXT6|A0A0J8BXT6_BETVU:40S ribosomal protein SA;OS=Beta vulgaris subsp. vulgaris.;|GO:0022627,|GO:0003735,|GO:0000028,GO:0006412,
DN124854_c0_g1_i5	DOWN	[GO:0016021:integral component of membrane][GO:0015743:malate transport]	-	-	XP_010685434.1:PREDICTED: aluminumactivated malate transporter 2 [Beta vulgaris subsp. vulgaris] >KMT04941.1 hypothetical protein BVRB_7g171070 [Beta vulgaris subsp. vulgaris]	-	A0A0J8EPN0|A0A0J8EPN0_BETVU:Uncharacterized protein;OS=Beta vulgaris subsp. vulgaris.;|GO:0016021,|GO:0015743,

geneid	trend	GO	Pathway	TF	NR	NT	Uniprot
DN113558_c0_g1_i1	DOWN	-	-	-	XP_021720950.1:twocomponent response regulatorlike APRR5 [Chenopodium quinoa]	-	A0A0J8BZA6|A0A0J8BZA6_BETVU:Uncharacterized protein;OS=Beta vulgaris subsp. vulgaris.;
DN116255_c4_g9_i1	DOWN	[GO:0005524:ATP binding][GO:0004672:protein kinase activity]	-	OGLUM05G28390.1:Oryza glumaepatula|HB-other|HB-other family protein	XP_010694749.1:PREDICTED: mitogenactivated protein kinase 19 [Beta vulgaris subsp. vulgaris] >KMS98147.1 hypothetical protein BVRB_4g095340 isoform A [Beta vulgaris subsp. vulgaris]	gi|478164232|gb|GAHK01012371.1|:TSA: Jatropha curcas CL5229.Contig2_JC-CK_1A transcribed RNA sequence	A0A0J8BDV5|A0A0J8BDV5_BETVU: Uncharacterized protein;OS=Beta vulgaris subsp. vulgaris.;|GO:0005524,GO:0004672,

geneid	trend	GO	Pathway	TF	NR	NT	Uniprot
DN114666_c0_g1_i3	DOWN	[GO:0005783:endoplasmic reticulum][GO:0010211:IAA-Leu conjugate hydrolase activity][GO:0010210:IAA-Phe conjugate hydrolase activity][GO:0009850:auxin metabolic process]	-	XP_011071286.1:Sesamum indicum|C2H2|C2H2 family protein	XP_010683632.1:PREDICTED: IAAamino acid hydrolase ILR1 [Beta vulgaris subsp. vulgaris]	gi|512399173|gb|GAJZ01001997.1|:TSA: Prunus avium Pa_03996 transcribed RNA sequence	A0A0J8BZN7|A0A0J8BZN7_BETVU:Uncharacterized protein;OS=Beta vulgaris subsp. vulgaris.;|GO:0005783,|GO:0010211,GO:0010210,|GO:0009850,
DN126156_c0_g1_i5	DOWN	[GO:0016021:integral component of membrane][GO:0015079:potassium ion transmembrane transporter activity]	-	CCG011654.1:Populus euphratica|C3H|C3H family protein	ASM90205.1:K+ uptake permease 12 [Sesuvium portulacastrum]	gi|521669961|gb|GAIA01032547.1|:TSA: Salicornia europaea Unigene35714_Se200S transcribed RNA sequence	A0A0K9Q9U9|A0A0K9Q9U9_SPIOL:Potassium transporter;OS=Spinacia oleracea;|GO:0016021,|GO:0015079,
DN118949_c4_g1_i4	DOWN	[GO:0016747:transferase activity, transferring acyl groups other than amino-acyl groups]	-	MDP0000294614:Malus domestica|NAC|NAC family protein	XP_021765621.1:uncharacterized acetyltransferase At3g50280like [Chenopodium quinoa]	-	A0A0K9RMS2|A0A0K9RMS2_SPIOL: Uncharacterized protein;OS=Spinacia oleracea;|GO:0016747,

geneid	trend	GO	Pathway	TF	NR	NT	Uniprot
DN121446_c2_g2_i7	DOWN	[GO:0005524:ATP binding][GO:0004672:protein kinase activity]	-	-	XP_019105321.1:PREDICTED: serine/threonineprotein kinase HT1like isoform X1 [Beta vulgaris subsp. vulgaris] >XP_019105322.1 PREDICTED: serine/threonineprotein kinase HT1like isoform X1 [Beta vulgaris subsp. vulgaris] >KMT10210.1 hypothetical protein BVRB_5g119710 [Beta vulgaris subsp. vulgaris]	-	A0A0J8C926|A0A0J8C926_BETVU:Uncharacterized protein;OS=Beta vulgaris subsp. vulgaris.;|GO:0005524,GO:0004672,
DN125813_c5_g1_i4	DOWN	-	-	-	KNA20667.1:hypothetical protein SOVF_050300 [Spinacia oleracea]	gi|502099320|ref|XM_004491395.1|:PREDICTED: Cicer arietinum pirin-like protein-like (LOC101493702),	A0A0K9RPB0|A0A0K9RPB0_SPIOL:Uncharacterized protein;OS=Spinacia oleracea;

geneid	trend	GO	Pathway	TF	NR	NT	Uniprot
						mRNA	
DN124854_c0_g1_i8	DOWN	[GO:0016021:integral component of membrane][GO:0015743:malate transport]	-	-	KNA09491.1:hypothetical protein SOVF_153100 [Spinacia oleracea]	-	A0A0K9QSH2|A0A0K9QSH2_SPIOL: Uncharacterized protein;OS=Spinacia oleracea;|GO:0016021,|GO:0015743,
DN117527_c0_g4_i2	DOWN	[GO:0016021:integral component of membrane][GO:0008270:zinc ion binding]	-	-	XP_010689560.1:PREDICTED: uncharacterized protein LOC104903248 [Beta vulgaris subsp. vulgaris] >KMT02075.1 hypothetical protein BVRB_9g208040 [Beta vulgaris subsp. vulgaris]	-	A0A0J8BKZ3|A0A0J8BKZ3_BETVU:Uncharacterized protein;OS=Beta vulgaris subsp. vulgaris.;|GO:0016021,|GO:0008270,
DN118870_c6_g6_i2	DOWN	-	-	-	-	-	-

geneid	trend	GO	Pathway	TF	NR	NT	Uniprot
DN108404_c0_g1_i13	DOWN	-	-	-	-	-	-
DN117097_c0_g1_i1	DOWN	[GO:0005634:nucleus][GO:0003677:DNA binding][GO:0003700:DNA-binding transcription factor activity][GO:0006351:transcription, DNA-templated]	-	Bv1_006280_gdiw.t1:Beta vulgaris|MIKC_MADS|MIKC_MADS family protein	XP_021758358.1:floral homeotic protein DEFICIENSlike [Chenopodium quinoa]	-	A0A089VQF4|A0A089VQF4_9CARY:APETALA3;OS=Blitum nuttallianum.;|GO:0005634,|GO:0003677,GO:0003700,|GO:0006351,
DN118674_c1_g5_i2	DOWN	[GO:0005576:extracellular region][GO:0020037:heme binding][GO:0046872:metal ion binding][GO:0004601:peroxidase activity][GO:0042744:hydrogen peroxide catabolic process][GO:0006979:response to oxidative stress]	[ko00940:Phenylpropanoid biosynthesis]	EPS67213.1:Genlisea aurea|NAC|NAC family protein	OWM88823.1:hypothetical protein CDL15_Pgr020777 [Punica granatum]	gi|7453854|gb|AF244924.1|AF244924:Spinacia oleracea peroxidase prx15 precursor, mRNA, complete cds	A0A0J8F1K3|A0A0J8F1K3_BETVU:Peroxidase;OS=Beta vulgaris subsp. vulgaris.;|GO:0005576,|GO:0020037,GO:0046872,GO:0004601,|GO:0042744,GO:0006979,

geneid	trend	GO	Pathway	TF	NR	NT	Uniprot
DN119582_c1_g6_i1	DOWN	[GO:0016021:integral component of membrane][GO:0005886:plasma membrane][GO:0008643:carbohydrate transport]	-	-	XP_021775814.1:bidirectional sugar transporter N3like [Chenopodium quinoa]	gi|521742430|gb|GAIA01004953.1|:TSA: Salicornia europaea Unigene5240_Se200S transcribed RNA sequence	A0A0J8CG15|A0A0J8CG15_BETVU:Bidirectional sugar transporter SWEET;OS=Beta vulgaris subsp. vulgaris.;|GO:0016021,GO:0005886,|GO:0008643,
DN120768_c0_g2_i9	DOWN	[GO:0016021:integral component of membrane][GO:0008271:secondary active sulfate transmembrane transporter activity]	-	GSMUA_Achr9P13010_001:Musa acuminata|MIKC_MADS|MIKC_MADS family protein	XP_021723678.1:probable sulfate transporter 3.5 [Chenopodium quinoa]	-	A0A0J8EJ94|A0A0J8EJ94_BETVU:Uncharacterized protein;OS=Beta vulgaris subsp. vulgaris.;|GO:0016021,|GO:0008271,
DN119710_c0_g13_i1	DOWN	-	-	-	-	-	-
DN124905_c10_g2_i1	DOWN	-	-	-	-	-	-

geneid	trend	GO	Pathway	TF	NR	NT	Uniprot
DN126821_c2_g3_i1	DOWN	[GO:0005524:ATP binding]	[ko03040:Spliceosome][ko04010:MAPK signaling pathway][ko04141:Protein processing in endoplasmic reticulum][ko04144:Endocytosis][ko04213:Longevity regulating pathway - multiple species][ko04612:Antigen processing and presentation][ko04915:Estrogen signaling pathway][ko05134:Legionellosis][ko05145:Toxoplasmosis][ko05162:Measles][ko05164:Influenza A][ko05169:Epstein-Barr virus infection]	FANhyb_rscf00000233.1.g00002.1:Fragaria x ananassa|GATA|GATA family protein	BAJ98899.1:predicted protein [Hordeum vulgare subsp. vulgare]	gi|324499423|gb|HQ434763.2|:Ostrinia furnacalis heat shock 70 kDa cognate protein (HSC70) mRNA, complete cds	F2DUX8|F2DUX8_HORVD:Predicted protein;OS=Hordeum vulgare var. distichum;|GO:0005524,

geneid	trend	GO	Pathway	TF	NR	NT	Uniprot
DN124854_c0_g1_i7	DOWN	[GO:0016021:integral component of membrane][GO:0015743:malate transport]	-	-	XP_010685434.1:PREDICTED: aluminumactivated malate transporter 2 [Beta vulgaris subsp. vulgaris] >KMT04941.1 hypothetical protein BVRB_7g171070 [Beta vulgaris subsp. vulgaris]	-	A0A0J8EPN0|A0A0J8EPN0_BETVU:Uncharacterized protein;OS=Beta vulgaris subsp. vulgaris.;|GO:0016021,|GO:0015743,
DN90332_c0_g1_i1	DOWN	-	-	-	-	-	-
DN111947_c0_g1_i1	DOWN	[GO:0016021:integral component of membrane][GO:0005524:ATP binding][GO:0016887:ATPase activity][GO:0006810:transport]	-	Rsa1.0_03996.1_g00001.1:Raphanus sativus|MYB_related|MYB_related family protein	KNA08458.1:hypothetical protein SOVF_162410 isoform A [Spinacia oleracea]	-	A0A0K9QP53|A0A0K9QP53_SPIOL:Uncharacterized protein;OS=Spinacia oleracea;|GO:0016021,|GO:0005524,GO:0016887,|GO:0006810,

geneid	trend	GO	Pathway	TF	NR	NT	Uniprot
DN125382_c0_g1_i1	DOWN	[GO:0016021:integral component of membrane][GO:0005215:transporter activity][GO:0006857:oligopeptide transport]	-	-	KVH87396.1:Major facilitator superfamily domain, general substrate transporter [Cynara cardunculus var. scolymus]	-	A0A103XAZ6|A0A103XAZ6_CYNCS: Major facilitator superfamily domain, general substrate transporter;OS=Cynara cardunculus var. scolymus;|GO:0016021,|GO:0005215,|GO:0006857,
DN117077_c0_g1_i3	DOWN	[GO:0043565:sequence-specific DNA binding][GO:0003700:DNA-binding transcription factor activity][GO:0006351:transcription, DNA-templated]	[ko04075:Plant hormone signal transduction]	Sp_086720_jmtc.t1: Spinacia oleracea|bZIP|bZIP family protein	XP_021722056.1:transcription factor TGA9like isoform X3 [Chenopodium quinoa]	gi|460391422|ref|XM_004241272.1|:PREDICTED: Solanum lycopersicum TGACG-sequence-specific DNA-binding protein TGA-2.1-like (LOC101251993), mRNA	A0A0K9RCX9|A0A0K9RCX9_SPIOL: Uncharacterized protein;OS=Spinacia oleracea;|GO:0043565,GO:0003700,|GO:0006351,
DN125811_c7_g5_i1	DOWN	-	-	-	-	-	-

geneid	trend	GO	Pathway	TF	NR	NT	Uniprot
DN123981_c0_g1_i4	DOWN	[GO:0016758:transferase activity, transferring hexosyl groups][GO:0008152:metabolic process]	-	-	XP_021735839.1:anthocyanidin 3Oglucoside 2"Oglucosyltransferaselike [Chenopodium quinoa]	-	A0A0K9RWT5|A0A0K9RWT5_SPIOL:Glycosyltransferase;OS=Spinacia oleracea;|GO:0016758,|GO:0008152,
DN129148_c8_g11_i1	DOWN	-	-	-	-	-	-
DN111186_c1_g1_i1	DOWN	[GO:0016021:integral component of membrane][GO:0005886:plasma membrane][GO:0008643:carbohydrate transport]	-	mrna24941.1-v1.0-hybrid:Fragaria vesca|DBB|DBB family protein	XP_021846236.1:bidirectional sugar transporter N3like [Spinacia oleracea] >KNA19087.1 hypothetical protein SOVF_064870 [Spinacia oleracea]	-	A0A0K9RJN8|A0A0K9RJN8_SPIOL:Bidirectional sugar transporter SWEET;OS=Spinacia oleracea;|GO:0016021,GO:0005886,|GO:0008643,

geneid	trend	GO	Pathway	TF	NR	NT	Uniprot
DN124392_c3_g1_i5	DOWN	[GO:0016021:integral component of membrane][GO:0005524:ATP binding][GO:0042626:ATPase activity, coupled to transmembrane movement of substances]	[ko02010:ABC transporters]	Tp57577_TGAC_v2_mRNA22346:Trifolium pratense|M-type_MADS|M-type_MADS family protein	XP_017642452.1:PREDICTED: ABC transporter B family member 25, mitochondrial [Gossypium arboreum] >KHG26291.1 ABC transporter B family member 25, mitochondrial [Gossypium arboreum]	gi|521703387|gb|GAIA01016231.1|:TSA: Salicornia europaea Unigene16944_Se200S transcribed RNA sequence	A0A0B0PSE1|A0A0B0PSE1_GOSAR:ABC transporter B family member 25, mitochondrial;OS=Gossypium arboreum;|GO:0016021,|GO:0005524,GO:0042626,
DN110019_c0_g1_i2	DOWN	-	-	-	XP_010684671.1:PREDICTED: uncharacterized protein LOC104899217 [Beta vulgaris subsp. vulgaris] >KMT05898.1 hypothetical protein BVRB_7g165560 [Beta vulgaris subsp. vulgaris]	-	A0A0J8C133|A0A0J8C133_BETVU:Uncharacterized protein;OS=Beta vulgaris subsp. vulgaris.;

geneid	trend	GO	Pathway	TF	NR	NT	Uniprot
DN121774_c3_g3_i1	DOWN	[GO:0010181:FMN binding][GO:0003955:NAD(P)H dehydrogenase (quinone) activity]	[ko00130:Ubiquinone and other terpenoid-quinone biosynthesis]	-	XP_006284522.1:hypothetical protein CARUB_v10005724mg, partial [Capsella rubella] >EOA17420.1 hypothetical protein CARUB_v10005724mg, partial [Capsella rubella]	gi|255587933|ref|XM_002534399.1|:Ricinus communis Minor allergen Alt a, putative, mRNA	R0GYM8|R0GYM8_9BRAS:Uncharacterized protein;OS=Capsella rubella.;|GO:0010181,GO:0003955,
DN103642_c2_g6_i1	DOWN	-	-	-	-	-	-
DN106930_c0_g1_i2	DOWN	-	-	-	-	-	-
DN112349_c0_g1_i1	DOWN	[GO:0004857:enzyme inhibitor activity]	-	-	KMT03388.1:hypothetical protein BVRB_8g190770 [Beta vulgaris subsp. vulgaris]	-	A0A0J8BTX6|A0A0J8BTX6_BETVU:Uncharacterized protein;OS=Beta vulgaris subsp. vulgaris.;|GO:0004857,

geneid	trend	GO	Pathway	TF	NR	NT	Uniprot
DN113393_c0_g1_i5	DOWN	[GO:0003676:nucleic acid binding][GO:0015074:DNA integration]	-	Itr_sc000560.1_g00013.1:Ipomoea trifida|Nin-like|Nin-like family protein	XP_019231431.1:PREDICTED: uncharacterized protein LOC109212247 isoform X2 [Nicotiana attenuata]	-	A5C5R8|A5C5R8_VITVI:Putative uncharacterized protein;OS=Vitis vinifera;|GO:0003676,|GO:0015074,
DN117742_c2_g2_i2	DOWN	[GO:0009507:chloroplast][GO:0005739:mitochondrion][GO:0005525:GTP binding]	-	mrna03235.1-v1.0-hybrid:Fragaria vesca|ARR-B|ARR-B family protein	XP_021741015.1:short integuments 2, mitochondriallike [Chenopodium quinoa]	-	A0A0J8CSY7|A0A0J8CSY7_BETVU:Uncharacterized protein;OS=Beta vulgaris subsp. vulgaris.;|GO:0009507,GO:0005739,|GO:0005525,
DN124854_c0_g1_i6	DOWN	[GO:0016021:integral component of membrane][GO:0015743:malate transport]	-	-	XP_010685434.1:PREDICTED: aluminumactivated malate transporter 2 [Beta vulgaris subsp. vulgaris] >KMT04941.1 hypothetical protein BVRB_7g171070 [Beta vulgaris subsp.	gi|224054457|ref|XM_002298234.1|:Populus trichocarpa predicted protein, mRNA	A0A0J8EPN0|A0A0J8EPN0_BETVU:Uncharacterized protein;OS=Beta vulgaris subsp. vulgaris.;|GO:0016021,|GO:0015743,

geneid	trend	GO	Pathway	TF	NR	NT	Uniprot
					vulgaris]		
DN126501_c0_g1_i1	DOWN	-	-	-	XP_010682230.1:PREDICTED: uncharacterized protein LOC104897107 [Beta vulgaris subsp. vulgaris]	-	A0A0K9QVA1|A0A0K9QVA1_SPIOL: Uncharacterized protein;OS=Spinacia oleracea;

geneid	trend	GO	Pathway	TF	NR	NT	Uniprot
DN121603_c1_g3_i6	DOWN	[GO:0016021:integral component of membrane][GO:0005886:plasma membrane][GO:0016760:cellulose synthase (UDP-forming) activity][GO:0008270:zinc ion binding][GO:0071555:cell wall organization][GO:0030244:cellulose biosynthetic process]	-	FANhyb_rscf00001203.1.g00002.1:Fragaria x ananassa|LBD|LBD family protein	XP_021756780.1:cellulose synthase A catalytic subunit 7 [UDPforming]like [Chenopodium quinoa]	gi|359484895|ref|XM_002276854.2|:PREDICTED: Vitis vinifera cellulose synthase A catalytic subunit 7 [UDP-forming]-like, transcript variant 2 (LOC100248512), mRNA	A0A0K9QMU6|A0A0K9QMU6_SPIOL:Cellulose synthase;OS=Spinacia oleracea;|GO:0016021,GO:0005886,|GO:0016760,GO:0008270,|GO:0071555,GO:0030244,
DN127371_c2_g2_i3	DOWN	[GO:0016021:integral component of membrane][GO:0005524:ATP binding][GO:0004672:protein kinase activity]	-	GSMUA_Achr5P10740_001:Musa acuminata|LBD|LBD family protein	XP_021754117.1:probable LRR receptorlike serine/threonineprotein kinase At1g63430 isoform X2 [Chenopodium quinoa]	gi|521735030|gb|GAIA01009394.1|:TSA: Salicornia europaea Unigene9759_Se200S transcribed RNA sequence	A0A0J8CEA3|A0A0J8CEA3_BETVU:Uncharacterized protein;OS=Beta vulgaris subsp. vulgaris.;|GO:0016021,|GO:0005524,GO:0004672,

geneid	trend	GO	Pathway	TF	NR	NT	Uniprot
DN125877_c1_g5_i2	DOWN	[GO:0031083:BLOC-1 complex]	-	-	XP_021851364.1:biogenesis of lysosomerelated organelles complex 1 subunit 1 [Spinacia oleracea] >KNA18634.1 hypothetical protein SOVF_068660 [Spinacia oleracea]	-	A0A0K9RI86|A0A0K9RI86_SPIOL:Uncharacterized protein;OS=Spinacia oleracea;|GO:0031083,
DN124392_c3_g1_i9	DOWN	[GO:0016021:integral component of membrane][GO:0005524:ATP binding][GO:0042626:ATPase activity, coupled to transmembrane movement of substances]	[ko02010:ABC transporters]	Tp57577_TGAC_v2_mRNA22346:Trifolium pratense|M-type_MADS|M-type_MADS family protein	XP_021751097.1:ABC transporter B family member 25, mitochondriallike [Chenopodium quinoa]	gi|521703387|gb|GAIA01016231.1|:TSA: Salicornia europaea Unigene16944_Se200S transcribed RNA sequence	A0A0K9R945|A0A0K9R945_SPIOL:Uncharacterized protein;OS=Spinacia oleracea;|GO:0016021,|GO:0005524,GO:0042626,
DN127560_c3_g3_i2	DOWN	[GO:0016210:naringenin-chalcone synthase activity][GO:0009058:biosynthetic process]	[ko00941:Flavonoid biosynthesis][ko04712:Circadian rhythm - plant]	-	BAB40787.2:chalcone synthase [Lilium hybrid division I]	gi|507310951|gb|KC820130.1|:Apium graveolens chalcone synthase protein (CHS) mRNA, partial cds	Q9AVC0|Q9AVC0_9LILI:Chalcone synthase;OS=Lilium hybrid division I.;|GO:0016210,|GO:0009058,

geneid	trend	GO	Pathway	TF	NR	NT	Uniprot
DN128775_c1_g1_i4	DOWN	-	[ko00480:Glutathione metabolism][ko00980:Metabolism of xenobiotics by cytochrome P450][ko00982:Drug metabolism - cytochrome P450][ko01524:Platinum drug resistance][ko05204:Chemical carcinogenesis][ko05418:Fluid shear stress and atherosclerosis]	-	XP_021638097.1:glutathione Stransferase L3like isoform X2 [Hevea brasiliensis]	gi|509467285|gb|GAJL01014896.1|:TSA: Triticum aestivum TA_RNA_121579 transcribed RNA sequence	M5WH15|M5WH15_PRUPE:Uncharacterized protein;OS=Prunus persica;
DN86587_c0_g2_i1	DOWN	-	-	-	-	-	-

geneid	trend	GO	Pathway	TF	NR	NT	Uniprot
DN127531_c1_g2_i16	DOWN	-	-	-	XP_010683787.1:PREDICTED: BTB/POZ domaincontaining protein FBL11 isoform X3 [Beta vulgaris subsp. vulgaris] >KMT06841.1 hypothetical protein BVRB_7g159950 isoform B [Beta vulgaris subsp. vulgaris]	-	A0A0J8EUH4|A0A0J8EUH4_BETVU: Uncharacterized protein;OS=Beta vulgaris subsp. vulgaris.;
DN125095_c0_g1_i23	DOWN	[GO:0005524:ATP binding][GO:0003777:microtubule motor activity][GO:0007018:microtubule-based movement]	-	XP_009122937.1:Brassica rapa|MYB_related|MYB_related family protein	XP_021844160.1:kinesinlike protein KIN7N [Spinacia oleracea] >KNA24521.1 hypothetical protein SOVF_015230 [Spinacia oleracea]	gi|357153683|ref|XR_138399.1|:PREDICTED: Brachypodium distachyon uncharacterized LOC100828626 (LOC100828626), miscRNA	A0A0K9RYC7|A0A0K9RYC7_SPIOL: Uncharacterized protein;OS=Spinacia oleracea;|GO:0005524,GO:0003777,|GO:0007018,

geneid	trend	GO	Pathway	TF	NR	NT	Uniprot
DN117308_c5_g1_i2	DOWN	[GO:0016787:hydrolase activity][GO:0006629:lipid metabolic process]	-	Achn227371:Actinidia chinensis|bHLH|bHLH family protein	XP_010686369.1:PREDICTED: uncharacterized protein LOC104900601 [Beta vulgaris subsp. vulgaris]	-	A0A0J8BX94|A0A0J8BX94_BETVU:Uncharacterized protein;OS=Beta vulgaris subsp. vulgaris.;|GO:0016787,|GO:0006629,
DN112948_c0_g1_i2	DOWN	[GO:0016021:integral component of membrane][GO:0015171:amino acid transmembrane transporter activity]	-	-	XP_021734617.1:aminoacid permease BAT1 homolog [Chenopodium quinoa]	gi|502137114|ref|XM_004502917.1|:PREDICTED: Cicer arietinum amino-acid permease BAT1 homolog (LOC101497428), transcript variant X2, mRNA	A0A0J8CKL0|A0A0J8CKL0_BETVU:Uncharacterized protein;OS=Beta vulgaris subsp. vulgaris.;|GO:0016021,|GO:0015171,
DN105810_c3_g2_i1	DOWN	-	-	-	-	-	-
DN121617_c1_g2_i5	DOWN	[GO:0030246:carbohydrate binding][GO:0016853:isomerase activity][GO:0005975:carbohydrate metabolic process]	[ko00010:Glycolysis / Gluconeogenesis]	MDP0000239688:Malus domestica|bZIP|bZIP family protein	XP_021757607.1:putative glucose6phosphate 1epimerase [Chenopodium	gi|521740951|gb|GAIA01006084.1|:TSA: Salicornia europaea Unigene6393_Se200S transcribed RNA	A0A0K9R2E5|A0A0K9R2E5_SPIOL:Uncharacterized protein;OS=Spinacia oleracea;|GO:00302

geneid	trend	GO	Pathway	TF	NR	NT	Uniprot
					quinoa]	sequence	46,GO:0016853,|GO:0005975,
DN101824_c1_g1_i1	DOWN	[GO:0005634:nucleus][GO:0043565:sequence-specific DNA binding][GO:0003700:DNA-binding transcription factor activity]	-	maker-scaffold10979-augustus-gene-0.4-mRNA-1:Castanea mollissima|HSF|HSF family protein	XP_021717633.1:heat stress transcription factor A6blike [Chenopodium quinoa] >XP_021717635.1 heat stress transcription factor A6blike [Chenopodium quinoa] >XP_021717642.1 heat stress transcription factor A6blike [Chenopodium quinoa]	gi|521665782|gb|GAIA01034647.1|:TSA: Salicornia europaea Unigene38350_Se200S transcribed RNA sequence	A0A067GG69|A0A067GG69_CITSI:Uncharacterized protein;OS=Citrus sinensis;|GO:0005634,|GO:0043565,GO:0003700,

geneid	trend	GO	Pathway	TF	NR	NT	Uniprot
DN127121_c2_g2_i2	DOWN	[GO:0005789:endoplasmic reticulum membrane][GO:0016021:integral component of membrane][GO:0050660:flavin adenine dinucleotide binding][GO:0010181:FMN binding][GO:0050661:NADP binding][GO:0003958:NADPH-hemoprotein reductase activity]	-	GRMZM2G104268_P01:Zea mays|FAR1|FAR1 family protein	AJW67229.1:NADPHcytochrome P450 reductase [Camptotheca acuminata]	gi|521563577|gb|GAIA01074870.1|:TSA: Salicornia europaea Unigene90819_Se200S transcribed RNA sequence	Unreviewed|A0A0D5BVX3_CAMAC:NADPH--cytochrome P450 reductase;OS=Camptotheca acuminata;|GO:0005789,GO:0016021,|GO:0050660,GO:0010181,GO:0050661,GO:0003958,
DN117839_c2_g3_i3	DOWN	[GO:0008171:O-methyltransferase activity]	[ko00945:Stilbenoid, diarylheptanoid and gingerol biosynthesis]	-	XP_010694747.1:PREDICTED: transresveratrol diOmethyltransferaselike [Beta vulgaris subsp. vulgaris]	-	A0A0K9QLC2|A0A0K9QLC2_SPIOL: Uncharacterized protein;OS=Spinacia oleracea;|GO:0008171,
DN104543_c0_g1_i1	DOWN	-	-	-	-	-	-

geneid	trend	GO	Pathway	TF	NR	NT	Uniprot
DN120736_c1_g1_i2	DOWN	-	-	GSVIVT01036959001:Vitis vinifera|bHLH|bHLH family protein	XP_002268443.3:PREDICTED: transcription factor bHLH18 [Vitis vinifera] >XP_010647980.1 PREDICTED: transcription factor bHLH18 [Vitis vinifera] >XP_019074348.1 PREDICTED: transcription factor bHLH18 [Vitis vinifera] >CBI25069.3 unnamed protein product, partial [Vitis vinifera]	-	A5BUH3|A5BUH3_VITVI:Putative uncharacterized protein;OS=Vitis vinifera;

geneid	trend	GO	Pathway	TF	NR	NT	Uniprot
DN124622_c4_g1_i3	DOWN	[GO:0016021:integral component of membrane][GO:0022857:transmembrane transporter activity]	-	NNU_016725-RA:Nelumbo nucifera|GRAS|GRAS family protein	XP_010687695.1:PREDICTED: WAT1related protein At4g30420 [Beta vulgaris subsp. vulgaris] >KMT03534.1 hypothetical protein BVRB_8g192110 [Beta vulgaris subsp. vulgaris]	-	A0A0J8BU94|A0A0J8BU94_BETVU:WAT1-related protein;OS=Beta vulgaris subsp. vulgaris.;|GO:0016021,|GO:0022857,

geneid	trend	GO	Pathway	TF	NR	NT	Uniprot
DN104707_c0_g1_i1	DOWN	-	-	Cotton_A_16694_BGI-A2_v1.0:Gossypium arboreum|bHLH|bHLH family protein	XP_010696420.1:PREDICTED: protein NETWORKED 3A [Beta vulgaris subsp. vulgaris] >XP_010696421.1 PREDICTED: protein NETWORKED 3A [Beta vulgaris subsp. vulgaris] >XP_010696422.1 PREDICTED: protein NETWORKED 3A [Beta vulgaris subsp. vulgaris] >KMS96964.1 hypothetical protein BVRB_7g180070 [Beta vulgaris subsp. vulgaris]	gi|351723586|ref|NM_001250355.1|:Glycine max uncharacterized LOC100527524 (LOC100527524), mRNA >gi|255632539|gb|BT092371.1| Soybean clone JCVI-FLGm-10D14 unknown mRNA	A0A0J8B6S6|A0A0J8B6S6_BETVU:Uncharacterized protein;OS=Beta vulgaris subsp. vulgaris.;

geneid	trend	GO	Pathway	TF	NR	NT	Uniprot
DN123027_c1_g3_i4	DOWN	-	-	-	XP_009335445.1:PREDICTED: uncharacterized protein LOC103928158 [Pyrus x bretschneideri]	-	M5WPQ5|M5WPQ5_PRUPE:Uncharacterized protein;OS=Prunus persica;
DN103642_c2_g2_i1	DOWN	-	-	-	-	-	-
DN126715_c3_g2_i10	DOWN	-	[ko03040:Spliceosome]	-	XP_021850026.1:premRNAprocessing protein 40A isoform X1 [Spinacia oleracea] >XP_021850027.1 premRNAprocessing protein 40A isoform X1 [Spinacia oleracea] >KNA15150.1 hypothetical protein SOVF_100850 [Spinacia oleracea]	gi|224127227|ref|XM_002319983.1|:Populus trichocarpa predicted protein, mRNA	A0A0K9R6L6|A0A0K9R6L6_SPIOL:Uncharacterized protein;OS=Spinacia oleracea;
DN103187_c	DOWN	-	-	-	-	-	-

geneid	trend	GO	Pathway	TF	NR	NT	Uniprot
0_g3_i1							
DN119865_c 0_g2_i2	DOWN	[GO:0005672:transcription factor TFIIA complex][GO:0006355:regulation of transcription, DNA-templated][GO:0006367:transcription initiation from RNA polymerase II promoter]	[ko03022:Basal transcription factors][ko05203:Viral carcinogenesis]	-	XP_010277568.1:PREDICTED: transcription initiation factor IIA subunit 2 [Nelumbo nucifera] >XP_010277569.1 PREDICTED: transcription initiation factor IIA subunit 2 [Nelumbo nucifera] >XP_010277570.1 PREDICTED: transcription initiation factor IIA subunit 2 [Nelumbo nucifera]	-	R0GZG0|R0GZG0_9BRAS:Transcription initiation factor IIA subunit 2;OS=Capsella rubella.;|GO:0005672,|GO:0006355,GO:0006367,
DN127085_c 1_g1_i2	DOWN	[GO:0016021:integral component of membrane][GO:0008271:secondary active sulfate transmembrane transporter activity]	-	-	XP_021723678.1:probable sulfate transporter 3.5 [Chenopodium quinoa]	gi|521689235|gb|GAIA01023091.1|:TSA: Salicornia europaea Unigene24682_Se200S transcribed RNA	A0A0J8EJ94|A0A0J8EJ94_BETVU:Uncharacterized protein;OS=Beta vulgaris subsp.

geneid	trend	GO	Pathway	TF	NR	NT	Uniprot
						sequence	vulgaris.;|GO:0016021,|GO:0008271,
DN121789_c4_g1_i3	DOWN	-	-	-	-	-	-
DN127851_c2_g4_i6	DOWN	[GO:0016021:integral component of membrane][GO:0016760:cellulose synthase (UDP-forming) activity][GO:0071555:cell wall organization][GO:0030244:cellulose biosynthetic process]	-	Sme2.5_02298.1_g00001.1:Solanum melongena|ARF|ARF family protein	XP_010695585.1:PREDICTED: cellulose synthaselike protein G3 [Beta vulgaris subsp. vulgaris] >KMS97558.1 hypothetical protein BVRB_5g125820 [Beta vulgaris subsp. vulgaris]	gi|359474112|ref|XM_002265090.2|:PREDICTED: Vitis vinifera cellulose synthase-like protein G3 (LOC100253121), mRNA	A0A0J8BC03|A0A0J8BC03_BETVU:Uncharacterized protein;OS=Beta vulgaris subsp. vulgaris.;|GO:0016021,|GO:0016760,|GO:0071555,GO:0030244,

geneid	trend	GO	Pathway	TF	NR	NT	Uniprot
DN119288_c1_g3_i3	DOWN	[GO:0005524:ATP binding][GO:0004674:protein serine/threonine kinase activity]	[ko04064:NF-kappa B signaling pathway][ko04620:Toll-like receptor signaling pathway][ko04621:NOD-like receptor signaling pathway][ko04624:Toll and Imd signaling pathway][ko04722:Neurotrophin signaling pathway][ko05133:Pertussis][ko05140:Leishmaniasis][ko05142:Chagas disease (American trypanosomiasis)][ko05145:Toxoplasmosis][ko05152:Tuberculosis][ko05162:Measles][ko05164:Influenza A]	EMT28879:Aegilops tauschii|ERF|ERF family protein	XP_021726413.1:putative serine/threonineprotein kinase isoform X1 [Chenopodium quinoa]	gi|224125453|ref|XM_002329773.1|:Populus trichocarpa predicted protein, mRNA	A0A0K9RL94|A0A0K9RL94_SPIOL:Uncharacterized protein;OS=Spinacia oleracea;|GO:0005524,GO:0004674,

geneid	trend	GO	Pathway	TF	NR	NT	Uniprot
DN104283_c6_g3_i1	DOWN	-	-	-	-	-	-
DN122032_c1_g2_i3	DOWN	[GO:0016021:integral component of membrane][GO:0005886:plasma membrane]	-	-	KNA06998.1:hypothetical protein SOVF_175910 [Spinacia oleracea]	-	A0A0K9QJW8|A0A0K9QJW8_SPIOL: CASP-like protein;OS=Spinacia oleracea;|GO:0016021,GO:0005886,
DN150136_c0_g1_i1	DOWN	-	-	-	-	-	-
DN105361_c0_g2_i2	DOWN	-	[ko03410:Base excision repair][ko04140:Autophagy - animal][ko04217:Necroptosis]	-	-	-	A0A1D1Z9A5|A0A1D1Z9A5_9ARAE: High mobility group protein DSP1;OS=Anthurium amnicola.;
DN127982_c0_g7_i7	DOWN	-	-	Cotton_A_10855_BGI-A2_v1.0:Gossypium arboreum|bHLH|bHLH family protein	KNA12966.1:hypothetical protein SOVF_120870 [Spinacia oleracea]	gi|521709187|gb|GAIA01013410.1|:TSA: Salicornia europaea Unigene14054_Se200S transcribed RNA sequence	A0A0K9R0C2|A0A0K9R0C2_SPIOL:Uncharacterized protein;OS=Spinacia oleracea;

geneid	trend	GO	Pathway	TF	NR	NT	Uniprot
DN110194_c0_g1_i1	DOWN	[GO:0005524:ATP binding][GO:0004672:protein kinase activity]	-	mrna01596.1-v1.0-hybrid:Fragaria vesca|WRKY|WRKY family protein	XP_021766908.1:probable serine/threonineprotein kinase PBL23 [Chenopodium quinoa]	gi|478151674|gb|GAHK01024929.1|:TSA: Jatropha curcas Unigene12388_JC-CK_1A transcribed RNA sequence	A0A0J8E0V3|A0A0J8E0V3_BETVU:Uncharacterized protein;OS=Beta vulgaris subsp. vulgaris.;|GO:0005524,GO:0004672,
DN119797_c0_g3_i1	DOWN	-	-	-	-	-	-
DN121603_c1_g3_i7	DOWN	[GO:0016021:integral component of membrane][GO:0005886:plasma membrane][GO:0016760:cellulose synthase (UDP-forming) activity][GO:0008270:zinc ion binding][GO:0071555:cell wall organization][GO:0030244:cellulose biosynthetic process]	-	FANhyb_rscf00001203.1.g00002.1:Fragaria x ananassa|LBD|LBD family protein	XP_021756780.1:cellulose synthase A catalytic subunit 7 [UDPforming]like [Chenopodium quinoa]	gi|359484895|ref|XM_002276854.2|:PREDICTED: Vitis vinifera cellulose synthase A catalytic subunit 7 [UDP-forming]-like, transcript variant 2 (LOC100248512), mRNA	A0A0K9QMU6|A0A0K9QMU6_SPIOL:Cellulose synthase;OS=Spinacia oleracea;|GO:0016021,GO:0005886,|GO:0016760,GO:0008270,|GO:0071555,GO:0030244,

geneid	trend	GO	Pathway	TF	NR	NT	Uniprot
DN117321_c1_g1_i2	DOWN	[GO:0005576:extracellular region][GO:0020037:heme binding][GO:0046872:metal ion binding][GO:0004601:peroxidase activity][GO:0042744:hydrogen peroxide catabolic process][GO:0006979:response to oxidative stress]	[ko00940:Phenylpropanoid biosynthesis]	MDP0000449389:Malus domestica|M-type_MADS|M-type_MADS family protein	XP_010693446.1:PREDICTED: peroxidase 66like [Beta vulgaris subsp. vulgaris]	gi|521628089|gb|GAIA01051989.1|:TSA: Salicornia europaea Unigene61009_Se200S transcribed RNA sequence	A0A0K9QY54|A0A0K9QY54_SPIOL:Peroxidase;OS=Spinacia oleracea;|GO:0005576,|GO:0020037,GO:0046872,GO:0004601,|GO:0042744,GO:0006979,
DN115862_c1_g1_i1	DOWN	[GO:0016021:integral component of membrane][GO:0016844:strictosidine synthase activity][GO:0009058:biosynthetic process][GO:0010584:pollen exine formation]	-	KN538684.1_FGP011:Oryza longistaminata|MYB_related|MYB_related family protein	XP_010672238.1:PREDICTED: protein STRICTOSIDINE SYNTHASELIKE 13 [Beta vulgaris subsp. vulgaris] >KMT15781.1 hypothetical protein BVRB_3g057570 [Beta vulgaris subsp. vulgaris]	-	A0A0J8FIT0|A0A0J8FIT0_BETVU:Uncharacterized protein;OS=Beta vulgaris subsp. vulgaris.;|GO:0016021,|GO:0016844,|GO:0009058,GO:0010584,

geneid	trend	GO	Pathway	TF	NR	NT	Uniprot
DN121930_c0_g1_i4	DOWN	-	-	-	XP_021725685.1:uncharacterized protein LOC110692913 [Chenopodium quinoa]	-	A0A0J8CSH6|A0A0J8CSH6_BETVU:Uncharacterized protein;OS=Beta vulgaris subsp. vulgaris.;
DN120099_c1_g1_i4	DOWN	[GO:0016491:oxidoreductase activity]	[ko00330:Arginine and proline metabolism][ko00410:beta-Alanine metabolism]	676755044:Sisymbrium irio|bHLH|bHLH family protein	AAM43922.1:polyamine oxidase [Amaranthus hypochondriacus]	gi|521692116|gb|GAIA01021651.1|:TSA: Salicornia europaea Unigene22796_Se200S transcribed RNA sequence	Q8LL67|Q8LL67_AMAHP:Polyamine oxidase;OS=Amaranthus hypochondriacus;|GO:0016491,
DN103259_c0_g2_i1	DOWN	-	-	augustus_masked-scaffold02666-abinit-gene-0.6-mRNA-1:Castanea mollissima|ZF-HD|ZF-HD family protein	OMO61911.1:hypothetical protein COLO4_33302 [Corchorus olitorius]	-	A0A067JT59|A0A067JT59_JATCU:Uncharacterized protein;OS=Jatropha curcas;
DN121350_c3_g1_i5	DOWN	[GO:0048046:apoplast][GO:0005507:copper ion binding][GO:0052716:hydroquinone:oxygen oxidoreductase activity][GO:0046274:lignin catabolic process]	-	Rsa1.0_03034.1_g00001.1:Raphanus sativus|ERF|ERF family protein	XP_010692917.1:PREDICTED: laccase12 [Beta vulgaris subsp. vulgaris]	gi|460388611|ref|XM_004239913.1|:PREDICTED: Solanum lycopersicum laccase-5-like (LOC101263212), mRNA	A0A0J8BDM3|A0A0J8BDM3_BETVU:Laccase;OS=Beta vulgaris subsp. vulgaris.;|GO:0048046,|GO:0005507,GO:0052716,|GO:00462

geneid	trend	GO	Pathway	TF	NR	NT	Uniprot
							74,
DN121350_c3_g1_i1	DOWN	[GO:0048046:apoplast][GO:0005507:copper ion binding][GO:0052716:hydroquinone:oxygen oxidoreductase activity][GO:0046274:lignin catabolic process]	-	Rsa1.0_03034.1_g00001.1:Raphanus sativus|ERF|ERF family protein	XP_010692917.1:PREDICTED: laccase12 [Beta vulgaris subsp. vulgaris]	-	A0A0J8BDM3|A0A0J8BDM3_BETVU: Laccase;OS=Beta vulgaris subsp. vulgaris.;|GO:0048046,|GO:0005507,GO:0052716,|GO:0046274,
DN126423_c3_g1_i1	DOWN	[GO:0009507:chloroplast][GO:0031977:thylakoid lumen][GO:0003755:peptidyl-prolyl cis-trans isomerase activity][GO:0006457:protein folding]	-	-	XP_010687764.1:PREDICTED: peptidylprolyl cistrans isomerase CYP28, chloroplastic [Beta vulgaris subsp. vulgaris] >KMT03602.1 hypothetical protein BVRB_8g192750	-	A0A0J8BQY9|A0A0J8BQY9_BETVU: Uncharacterized protein;OS=Beta vulgaris subsp. vulgaris.;|GO:0009507,GO:0031977,|GO:0003755,|GO:0006457,

geneid	trend	GO	Pathway	TF	NR	NT	Uniprot
					[Beta vulgaris subsp. vulgaris]		
DN106927_c0_g1_i1	DOWN	[GO:0005777:peroxisome][GO:0016491:oxidoreductase activity][GO:0080024:indolebutyric acid metabolic process][GO:0080026:response to indolebutyric acid][GO:0048767:root hair elongation]	[ko00830:Retinol metabolism][ko04146:Peroxisome]	-	XP_010665748.1:PREDICTED: tropinone reductaselike 3 [Beta vulgaris subsp. vulgaris] >XP_019102797.1 PREDICTED: tropinone reductaselike 3 [Beta vulgaris subsp. vulgaris] >KMS96611.1 hypothetical protein BVRB_8g201410 [Beta vulgaris subsp.	-	A0A0J8B6J4|A0A0J8B6J4_BETVU:Uncharacterized protein;OS=Beta vulgaris subsp. vulgaris.;|GO:0005777,|GO:0016491,|GO:0080024,GO:0080026,GO:0048767,

geneid	trend	GO	Pathway	TF	NR	NT	Uniprot
					vulgaris]		
DN123221_c1_g2_i4	DOWN	[GO:0003677:DNA binding]	-	LOC_Os08g09810.1:Oryza sativa subsp. japonica|WRKY|WRKY family protein	KMS98900.1:hypothetical protein BVRB_3g067910 [Beta vulgaris subsp. vulgaris]	-	A0A0J8E6R9|A0A0J8E6R9_BETVU:Uncharacterized protein;OS=Beta vulgaris subsp. vulgaris.;|GO:0003677,
DN125490_c2_g3_i1	DOWN	[GO:0016021:integral component of membrane][GO:0022857:transmembrane transporter activity]	-	Glyma.13G227100.1.p:Glycine max|ERF|ERF family protein	EOY23738.1:Nodulin MtN21 /EamAlike transporter family protein, putative [Theobroma cacao]	-	A0A061G2X7|A0A061G2X7_THECC:WAT1-related protein;OS=Theobroma cacao;|GO:0016021,|

geneid	trend	GO	Pathway	TF	NR	NT	Uniprot
							GO:0022857,
DN127568_c2_g4_i1	DOWN	-	-	-	XP_021843114.1:protein IQDOMAIN 1 [Spinacia oleracea]	-	A0A0J8CQJ6|A0A0J8CQJ6_BETVU:Uncharacterized protein;OS=Beta vulgaris subsp. vulgaris.;
DN120066_c0_g7_i1	DOWN	-	-	-	-	-	-
DN117125_c0_g2_i6	DOWN	[GO:0003676:nucleic acid binding][GO:0008270:zinc ion binding]	-	-	KMT17803.1:hypothetical protein BVRB_2g035460 isoform B [Beta vulgaris subsp. vulgaris]	gi|521447289|gb|GAHZ01073195.1|:TSA: Salicornia europaea Unigene89025_SeCKS transcribed RNA sequence	A0A0J8D0Y2|A0A0J8D0Y2_BETVU:Uncharacterized protein;OS=Beta vulgaris subsp. vulgaris.;|GO:0003676,GO:0008270,

geneid	trend	GO	Pathway	TF	NR	NT	Uniprot
DN126821_c2_g3_i2	DOWN	[GO:0005524:ATP binding]	[ko03040:Spliceosome][ko04010:MAPK signaling pathway][ko04141:Protein processing in endoplasmic reticulum][ko04144:Endocytosis][ko04213:Longevity regulating pathway - multiple species][ko04612:Antigen processing and presentation][ko04915:Estrogen signaling pathway][ko05134:Legionellosis][ko05145:Toxoplasmosis][ko05162:Measles][ko05164:Influenza A][ko05169:Epstein-Barr virus infection]	FANhyb_rscf00000233.1.g00002.1:Fragaria x ananassa|GATA|GATA family protein	BAJ98899.1:predicted protein [Hordeum vulgare subsp. vulgare]	gi|324499423|gb|HQ434763.2|:Ostrinia furnacalis heat shock 70 kDa cognate protein (HSC70) mRNA, complete cds	F2DUX8|F2DUX8_HORVD:Predicted protein;OS=Hordeum vulgare var. distichum;|GO:0005524,

geneid	trend	GO	Pathway	TF	NR	NT	Uniprot
DN119391_c1_g2_i1	DOWN	[GO:0016021:integral component of membrane]	-	-	XP_018819741.1:PREDICTED: putative ALAinteracting subunit 2 [Juglans regia] >XP_018819742.1 PREDICTED: putative ALAinteracting subunit 2 [Juglans regia] >XP_018819743.1 PREDICTED: putative ALAinteracting subunit 2 [Juglans regia] >XP_018819744.1 PREDICTED: putative ALAinteracting subunit 2 [Juglans regia]	-	B9T807|B9T807_RICCO:Putative uncharacterized protein;OS=Ricinus communis;|GO:0016021,

geneid	trend	GO	Pathway	TF	NR	NT	Uniprot
DN118869_c2_g3_i4	DOWN	[GO:0016021:integral component of membrane]	-	-	XP_010686967.1:PREDICTED: uncharacterized protein LOC104901121 isoform X1 [Beta vulgaris subsp. vulgaris]	-	A0A0J8BVU5|A0A0J8BVU5_BETVU: Uncharacterized protein;OS=Beta vulgaris subsp. vulgaris.;|GO:0016021,
DN117582_c0_g1_i2	DOWN	[GO:0020037:heme binding][GO:0005506:iron ion binding][GO:0004497:monooxygenase activity][GO:0016705:oxidoreductase activity, acting on paired donors, with incorporation or reduction of molecular oxygen]	-	Sme2.5_00297.1_g00019.1:Solanum melongena|MYB|MYB family protein	AKI33941.1:cytochrome P450 76AD1like protein, partial [Bougainvillea spectabilis]	gi|356968421|gb|HQ656026.1|:Mirabilis jalapa CYP76AD3 mRNA, complete cds	A0A0G2UT73|A0A0G2UT73_9CARY: Cytochrome P450 76AD1-like protein;OS=Bougainvillea spectabilis.;|GO:0020037,GO:0005506,GO:0004497,GO:0016705,
DN118058_c0_g4_i1	DOWN	-	[ko04260:Cardiac muscle contraction][ko04261:Adrenergic signaling in cardiomyocytes][ko05416:Viral	-	-	gi|158299189|ref|XM_319309.4|:Anopheles gambiae str. PEST AGAP010147-PA (AgaP_AGAP010147) mRNA, complete	A0A1D1Z8S6|A0A1D1Z8S6_9ARAE:Myosin heavy chain, muscle;OS=Anthurium amnicola.;

geneid	trend	GO	Pathway	TF	NR	NT	Uniprot
			myocarditis]			cds	
DN125836_c1_g4_i7	DOWN	[GO:0046872:metal ion binding][GO:0008233:peptidase activity]	-	-	XP_021731733.1:presequence protease 1, chloroplastic/mitochondriallike [Chenopodium quinoa]	gi|449495594|ref|XM_004159841.1|:PREDICTED: Cucumis sativus presequence protease 1, chloroplastic/mitochondrial-like (LOC101228559), mRNA	A0A023EB19|A0A023EB19_SILLA:Presequence protease 1;OS=Silene latifolia;|GO:0046872,GO:0008233,
DN106730_c0_g4_i1	DOWN	-	-	-	-	-	-
DN117735_c4_g1_i1	DOWN	-	-	-	XP_021775039.1:uncharacterized protein LOC110738893 [Chenopodium quinoa]	-	A0A0J8B5L9|A0A0J8B5L9_BETVU:Uncharacterized protein;OS=Beta vulgaris subsp. vulgaris.;

geneid	trend	GO	Pathway	TF	NR	NT	Uniprot
DN115530_c0_g1_i2	DOWN	[GO:0016021:integral component of membrane][GO:0015018:galactosylgalactosylxylosylprotein 3-beta-glucuronosyltransferase activity]	-	-	XP_021751603.1:probable beta1,4xylosyltransferase IRX9 [Chenopodium quinoa]	gi|63087725|emb|AJ969050.1|:Populus balsamifera mRNA for glycosyltransferase (pglcat5 gene)	A0A0J8B5J9|A0A0J8B5J9_BETVU:Uncharacterized protein;OS=Beta vulgaris subsp. vulgaris.;|GO:0016021,|GO:0015018,
DN116413_c0_g1_i2	DOWN	[GO:0016021:integral component of membrane][GO:0020037:heme binding][GO:0005506:iron ion binding][GO:0004497:monooxygenase activity][GO:0016705:oxidoreductase activity, acting on paired donors, with incorporation or reduction of molecular oxygen]	[ko00905:Brassinosteroid biosynthesis]	Bradi2g44117.7.p:Brachypodium distachyon|bHLH|bHLH family protein	XP_010691194.1:PREDICTED: cytochrome P450 734A1 [Beta vulgaris subsp. vulgaris] >KMT00715.1 hypothetical protein BVRB_9g220010 [Beta vulgaris subsp. vulgaris]	gi|225442828|ref|XM_002281329.1|:PREDICTED: Vitis vinifera cytochrome P450 734A1-like (LOC100263507), mRNA	A0A0J8BH47|A0A0J8BH47_BETVU:Uncharacterized protein;OS=Beta vulgaris subsp. vulgaris.;|GO:0016021,|GO:0020037,GO:0005506,GO:0004497,GO:0016705,

geneid	trend	GO	Pathway	TF	NR	NT	Uniprot
DN114741_c0_g1_i6	DOWN	[GO:0008725:DNA-3-methyladenine glycosylase activity][GO:0006284:base-excision repair]	[ko03410:Base excision repair]	-	XP_010681154.1:PREDICTED: uncharacterized protein LOC104896146 [Beta vulgaris subsp. vulgaris] >KMT08733.1 hypothetical protein BVRB_6g140040 [Beta vulgaris subsp. vulgaris]	-	A0A0J8C9Q5|A0A0J8C9Q5_BETVU:Uncharacterized protein;OS=Beta vulgaris subsp. vulgaris.;|GO:0008725,|GO:0006284,
DN123291_c3_g4_i1	DOWN	-	-	-	-	-	A0A1D5T4G6|A0A1D5T4G6_WHEAT:Uncharacterized protein;OS=Triticum aestivum;
DN126635_c3_g1_i2	DOWN	[GO:0003993:acid phosphatase activity][GO:0046872:metal ion binding]	-	Achn233011:Actinidia chinensis|SRS|SRS family protein	XP_010672424.1:PREDICTED: purple acid phosphatase [Beta vulgaris subsp. vulgaris] >KMT20665.1 hypothetical protein BVRB_1g006410	gi|224085757|ref|XM_002307654.1|:Populus trichocarpa predicted protein, mRNA	A0A0J8D3C8|A0A0J8D3C8_BETVU:Purple acid phosphatase;OS=Beta vulgaris subsp. vulgaris.;|GO:0003993,GO:0046872,

geneid	trend	GO	Pathway	TF	NR	NT	Uniprot
					[Beta vulgaris subsp. vulgaris]		
DN122230_c4_g1_i15	DOWN	-	-	-	XP_021742053.1:protein RICE SALT SENSITIVE 3like isoform X1 [Chenopodium quinoa]	-	A0A0K9REW3|A0A0K9REW3_SPIOL:Uncharacterized protein;OS=Spinacia oleracea;
DN93805_c0_g1_i1	DOWN	-	-	-	-	-	-
DN125449_c0_g2_i6	DOWN	[GO:0005886:plasma membrane][GO:0005509:calcium ion binding]	-	XP_009149673.1:Brassica rapa|C3H|C3H family protein	XP_010686960.1:PREDICTED: epidermal growth factor receptor substrate 15like 1 [Beta vulgaris subsp. vulgaris] >KMT19263.1 hypothetical protein	gi|359491846|ref|XM_002272757.2|:PREDICTED: Vitis vinifera uncharacterized LOC100240982 (LOC100240982), mRNA	A0A0J8D520|A0A0J8D520_BETVU:Uncharacterized protein;OS=Beta vulgaris subsp. vulgaris.;|GO:0005886,|GO:0005509,

geneid	trend	GO	Pathway	TF	NR	NT	Uniprot
					BVRB_1g014450 [Beta vulgaris subsp. vulgaris]		
DN128036_c10_g1_i16	DOWN	[GO:0030246:carbohydrate binding][GO:0003824:catalytic activity][GO:0005975:carbohydrate metabolic process]	-	-	XP_021853123.1:probable rhamnogalacturonate lyase B isoform X2 [Spinacia oleracea]	gi|502131651|ref|XM_004500998.1|:PREDICTED: Cicer arietinum rhamnogalacturonate lyase B-like (LOC101488431), transcript variant X1, mRNA	A0A067K103|A0A067K103_JATCU:Uncharacterized protein;OS=Jatropha curcas;|GO:0030246,GO:0003824,|GO:0005975,
DN117050_c0_g1_i5	DOWN	[GO:0016021:integral component of membrane][GO:0005215:transporter activity]	-	GSVIVT01016458001:Vitis vinifera|TALE|TALE family protein	XP_010691634.1:PREDICTED: protein NRT1/ PTR FAMILY 5.6 [Beta vulgaris subsp. vulgaris] >KMT18416.1	-	A0A0J8FRN7|A0A0J8FRN7_BETVU:Uncharacterized protein;OS=Beta vulgaris subsp. vulgaris.;|GO:0016021,|GO:0005215,

geneid	trend	GO	Pathway	TF	NR	NT	Uniprot
					hypothetical protein BVRB_2g025620 [Beta vulgaris subsp. vulgaris]		
DN55301_c1_g1_i2	DOWN	-	-	-	-	-	-
DN127211_c3_g2_i1	DOWN	[GO:0016021:integral component of membrane]	-	-	KNA11609.1:hypothetical protein SOVF_133440 [Spinacia oleracea]	-	A0A0K9QWH7|A0A0K9QWH7_SPIOL:Uncharacterized protein;OS=Spinacia oleracea;|GO:0016021,
DN110383_c0_g1_i1	DOWN	[GO:0046872:metal ion binding][GO:0016491:oxidoreductase activity]	-	676709864:Sisymbrium irio|STAT|STAT family protein	XP_021734080.1:hyoscyamine 6dioxygenaselike [Chenopodium quinoa]	-	A0A0K9QUS5|A0A0K9QUS5_SPIOL: Uncharacterized protein;OS=Spinacia oleracea;|GO:0046872,GO:0016491,

geneid	trend	GO	Pathway	TF	NR	NT	Uniprot
DN125516_c0_g1_i4	DOWN	[GO:0003676:nucleic acid binding][GO:0000166:nucleotide binding]	[ko03040:Spliceosome]	Csa08g011330.1:Camelina sativa|ZF-HD|ZF-HD family protein	XP_002267230.2:PREDICTED: serine/argininerich SC35like splicing factor SCL33 isoform X2 [Vitis vinifera]	-	A0A061GSC2|A0A061GSC2_THECC: SC35-like splicing factor 33;OS=Theobroma cacao;|GO:0003676, GO:0000166,
DN51936_c0_g2_i1	DOWN	-	-	-	-	-	-
DN121774_c3_g1_i1	DOWN	[GO:0010181:FMN binding][GO:0003955:NAD(P)H dehydrogenase (quinone) activity]	[ko00130:Ubiquinone and other terpenoid-quinone biosynthesis]	-	XP_002864337.1:NAD(P)H dehydrogenase (quinone) FQR1 [Arabidopsis lyrata subsp. lyrata] >EFH40596.1 hypothetical protein ARALYDRAFT_495531 [Arabidopsis lyrata subsp. lyrata]	-	D7MUA0|D7MUA0_ARALL:Putative uncharacterized protein;OS=Arabidopsis lyrata subsp. lyrata;|GO:0010181, GO:0003955,
DN103187_c0_g4_i1	DOWN	-	-	-	-	-	-

geneid	trend	GO	Pathway	TF	NR	NT	Uniprot
DN125382_c0_g3_i1	DOWN	[GO:0016021:integral component of membrane][GO:0005215:transporter activity][GO:0006857:oligopeptide transport]	-	Sopen09g001330.1: Solanum pennellii|G2-like|G2-like family protein	XP_010680056.1:PREDICTED: protein NRT1/ PTR FAMILY 8.3 [Beta vulgaris subsp. vulgaris] >KMT09724.1 hypothetical protein BVRB_6g131730 [Beta vulgaris subsp. vulgaris]	-	A0A0J8CBX0|A0A0J8CBX0_BETVU: Uncharacterized protein;OS=Beta vulgaris subsp. vulgaris.;|GO:0016021,|GO:0005215,|GO:0006857,
DN103693_c0_g1_i1	DOWN	-	-	-	XP_021855872.1:uncharacterized protein LOC110795196 [Spinacia oleracea]	-	A0A0J8CGA9|A0A0J8CGA9_BETVU: Uncharacterized protein;OS=Beta vulgaris subsp. vulgaris.;
DN122230_c4_g1_i4	DOWN	-	-	-	XP_021742058.1:protein RICE SALT SENSITIVE 3like isoform X2 [Chenopodium quinoa]	gi|521664610|gb|GAIA01035088.1|:TSA: Salicornia europaea Unigene38844_Se200S transcribed RNA sequence	A0A0K9REW3|A0A0K9REW3_SPIOL:Uncharacterized protein;OS=Spinacia oleracea;

geneid	trend	GO	Pathway	TF	NR	NT	Uniprot
DN118652_c0_g1_i3	DOWN	[GO:0048046:apoplast][GO:0005507:copper ion binding][GO:0052716:hydroquinone:oxygen oxidoreductase activity][GO:0003676:nucleic acid binding][GO:0046274:lignin catabolic process]	-	Rsa1.0_03034.1_g00001.1:Raphanus sativus|ERF|ERF family protein	XP_021743328.1:laccase17like [Chenopodium quinoa]	gi|224057691|ref|XM_002299260.1|:Populus trichocarpa predicted protein, mRNA	A0A0J8BXR7|A0A0J8BXR7_BETVU: Laccase;OS=Beta vulgaris subsp. vulgaris.;|GO:0048046,|GO:0005507,GO:0052716,GO:0003676,|GO:0046274,
DN123335_c1_g1_i4	DOWN	[GO:0009626:plant-type hypersensitive response][GO:0010337:regulation of salicylic acid metabolic process]	-	-	XP_021772531.1:MACPF domaincontaining protein CAD1like [Chenopodium quinoa]	gi|359483793|ref|XM_002264002.2|:PREDICTED: Vitis vinifera uncharacterized LOC100244557 (LOC100244557), mRNA	A0A0J8E0K3|A0A0J8E0K3_BETVU:Uncharacterized protein;OS=Beta vulgaris subsp. vulgaris.;|GO:0009626,GO:0010337,

geneid	trend	GO	Pathway	TF	NR	NT	Uniprot
DN112884_c0_g1_i1	DOWN	[GO:0005576:extracellular region][GO:0020037:heme binding][GO:0046872:metal ion binding][GO:0004601:peroxidase activity][GO:0042744:hydrogen peroxide catabolic process][GO:0006979:response to oxidative stress]	[ko00940:Phenylpropanoid biosynthesis]	EPS67213.1:Genlisea aurea|NAC|NAC family protein	XP_021718077.1:peroxidase 72like [Chenopodium quinoa]	gi|7453850|gb|AF244922.1|AF244922:Spinacia oleracea peroxidase prx13 precursor, mRNA, complete cds	A0A0J8C6W9|A0A0J8C6W9_BETVU: Peroxidase;OS=Beta vulgaris subsp. vulgaris.;|GO:0005576,|GO:0020037,GO:0046872,GO:0004601,|GO:0042744,GO:0006979,
DN103642_c2_g7_i1	DOWN	-	-	-	-	-	-
DN126228_c4_g1_i1	DOWN	-	-	-	XP_006446874.1:hypothetical protein CICLE_v10016745mg [Citrus clementina] >ESR60114.1 hypothetical protein CICLE_v10016745mg [Citrus clementina]	-	V4W2F5|V4W2F5_9ROSI:Uncharacterized protein;OS=Citrus clementina.;

geneid	trend	GO	Pathway	TF	NR	NT	Uniprot
DN121170_c1_g2_i2	DOWN	[GO:0050660:flavin adenine dinucleotide binding][GO:0004499:N,N-dimethylaniline monooxygenase activity][GO:0050661:NADP binding]	-	-	XP_012080330.1:flavincontaining monooxygenase FMO GSOXlike 9 [Jatropha curcas] >KDP31304.1 hypothetical protein JCGZ_11680 [Jatropha curcas]	-	A0A067KHJ9|A0A067KHJ9_JATCU:Flavin-containing monooxygenase;OS=Jatropha curcas;|GO:0050660,GO:0004499,GO:0050661,
DN115628_c0_g6_i1	DOWN	-	-	-	-	-	-
DN121644_c2_g8_i6	DOWN	[GO:0016021:integral component of membrane][GO:0015079:potassium ion transmembrane transporter activity]	-	mrna09346.1-v1.0-hybrid:Fragaria vesca|NF-X1|NF-X1 family protein	KNA14108.1:hypothetical protein SOVF_110650 [Spinacia oleracea]	gi|60101761|gb|AY919346.1|:Phytolacca acinosa putative high-affinity potassium transporter protein mRNA, complete cds	A0A0K9R3N6|A0A0K9R3N6_SPIOL:Potassium transporter;OS=Spinacia oleracea;|GO:0016021,|GO:0015079,
DN118411_c3_g5_i1	DOWN	-	-	-	-	-	-

geneid	trend	GO	Pathway	TF	NR	NT	Uniprot
DN120667_c1_g6_i3	DOWN	[GO:0005634:nucleus][GO:2000032:regulation of secondary shoot formation][GO:0010223:secondary shoot formation]	-	Bv5_126100_ryfn.t1:Beta vulgaris|TCP|TCP family protein	XP_010695622.1:PREDICTED: transcription factor TCP18 [Beta vulgaris subsp. vulgaris] >KMS97548.1 hypothetical protein BVRB_5g126100 [Beta vulgaris subsp. vulgaris]	-	A0A0J8BBZ7|A0A0J8BBZ7_BETVU:Uncharacterized protein;OS=Beta vulgaris subsp. vulgaris.;|GO:0005634,|GO:2000032,GO:0010223,
DN114696_c1_g4_i3	DOWN	-	-	-	-	-	-
DN125458_c6_g1_i1	DOWN	[GO:0005737:cytoplasm][GO:0016021:integral component of membrane]	-	GSBRNA2T00056321001:Brassica napus|MIKC_MADS|MIKC_MADS family protein	XP_010261204.1:PREDICTED: uncharacterized protein At1g04910 isoform X1 [Nelumbo nucifera]	gi|255569961|ref|XM_002525898.1|:Ricinus communis conserved hypothetical protein, mRNA	U5FPC6|U5FPC6_POPTR:Uncharacterized protein;OS=Populus trichocarpa;|GO:0005737,GO:0016021,
DN104283_c6_g4_i3	DOWN	-	-	-	-	-	-

geneid	trend	GO	Pathway	TF	NR	NT	Uniprot
DN121406_c3_g3_i2	DOWN	-	-	Csa09g092790.1:Camelina sativa|BES1|BES1 family protein	XP_010692278.1:PREDICTED: protein trichome birefringencelike 19 [Beta vulgaris subsp. vulgaris]	-	A0A0K9QYV1|A0A0K9QYV1_SPIOL: Uncharacterized protein;OS=Spinacia oleracea;
DN128011_c3_g2_i4	DOWN	[GO:0010181:FMN binding][GO:0003955:NAD(P)H dehydrogenase (quinone) activity]	[ko00130:Ubiquinone and other terpenoid-quinone biosynthesis]	-	XP_006452110.1:hypothetical protein CICLE_v10009497mg [Citrus clementina] >XP_006475251.1 PREDICTED: probable NAD(P)H dehydrogenase (quinone) FQR1like 1 [Citrus sinensis] >ESR65350.1 hypothetical protein CICLE_v10009497mg [Citrus clementina] >KDO74382.1 hypothetical protein CISIN_1g028841mg	gi|359493917|ref|XM_002283250.2|:PREDICTED: Vitis vinifera quinone reductase-like, transcript variant 1 (QOR), mRNA	A0A067GG13|A0A067GG13_CITSI:Uncharacterized protein;OS=Citrus sinensis;|GO:0010181,GO:0003955,

geneid	trend	GO	Pathway	TF	NR	NT	Uniprot
					[Citrus sinensis]		
DN128542_c0_g1_i12	DOWN	-	-	-	XP_010682959.1:PREDICTED: uncharacterized protein LOC104897720 isoform X1 [Beta vulgaris subsp. vulgaris]	-	A0A0J8BZA1|A0A0J8BZA1_BETVU:Uncharacterized protein;OS=Beta vulgaris subsp. vulgaris.;

geneid	trend	GO	Pathway	TF	NR	NT	Uniprot
DN109781_c0_g1_i1	DOWN	[GO:0046872:metal ion binding]	-	XP_004508237.1:Cicer arietinum|C2H2|C2H2 family protein	XP_004508237.1:PREDICTED: zinc finger protein 2 [Cicer arietinum]	-	V4KXC1|V4KXC1_EUTSA:Uncharacterized protein;OS=Eutrema salsugineum;|GO:0046872,
DN120691_c2_g3_i13	DOWN	[GO:0005524:ATP binding][GO:0003723:RNA binding][GO:0003724:RNA helicase activity][GO:0006401:RNA catabolic process]	[ko03018:RNA degradation]	Pbr029461.1:Pyrus bretschneideri|C3H|C3H family protein	XP_021844057.1:DExHbox ATPdependent RNA helicase DExH10 [Spinacia oleracea] >KNA18394.1 hypothetical protein SOVF_071150 [Spinacia oleracea]	gi|521631404|gb|GAIA01050718.1|:TSA: Salicornia europaea Unigene59271_Se200S transcribed RNA sequence	A0A0K9RHG7|A0A0K9RHG7_SPIOL: Uncharacterized protein;OS=Spinacia oleracea;|GO:0005524,GO:0003723,GO:0003724,|GO:0006401,
DN117800_c4_g4_i4	DOWN	[GO:0016021:integral component of membrane]	[ko00520:Amino sugar and nucleotide sugar metabolism]	MDP0000247737:Malus domestica|FAR1|FAR1 family protein	XP_021775409.1:UDPglucuronic acid decarboxylase 2like [Chenopodium quinoa]	gi|470124261|ref|XM_004298087.1|:PREDICTED: Fragaria vesca subsp. vesca UDP-glucuronic acid decarboxylase 1-like (LOC101294461), mRNA	G7IF53|G7IF53_MEDTR:UDP-glucuronic acid decarboxylase-like protein;OS=Medicago truncatula;GN=MTR_1g088480 {ECO:0000313|EMBL:AES61772.1};|G

geneid	trend	GO	Pathway	TF	NR	NT	Uniprot
							O:0016021,
DN123001_c0_g2_i3	DOWN	[GO:0005840:ribosome][GO:0003735:structural constituent of ribosome][GO:0006412:translation]	[ko03010:Ribosome]	evm_27.model.AmTr_v1.0_scaffold00155.59:Amborella trichopoda|GATA|GATA family protein	XP_010691599.1:PREDICTED: 40S ribosomal protein S12 [Beta vulgaris subsp. vulgaris] >KMT01055.1 hypothetical protein BVRB_9g223120 [Beta vulgaris subsp. vulgaris]	gi|521726623|gb|GAIA01011047.1|:TSA: Salicornia europaea Unigene11660_Se200S transcribed RNA sequence	A0A0J8BI12|A0A0J8BI12_BETVU:40S ribosomal protein S12;OS=Beta vulgaris subsp. vulgaris.;|GO:0005840,|GO:0003735,|GO:0006412,
DN127167_c2_g1_i5	DOWN	[GO:0016021:integral component of membrane][GO:0005524:ATP binding][GO:0016887:ATPase activity]	-	-	XP_010690527.2:PREDICTED: ABC transporter G family member 11like [Beta vulgaris subsp. vulgaris]	gi|502181024|ref|XM_004516685.1|:PREDICTED: Cicer arietinum ABC transporter G family member 11-like (LOC101508979), mRNA	A0A0K9RSD8|A0A0K9RSD8_SPIOL:Uncharacterized protein;OS=Spinacia oleracea;|GO:0016021,|GO:0005524,GO:0016887,

geneid	trend	GO	Pathway	TF	NR	NT	Uniprot
DN114523_c1_g1_i7	DOWN	[GO:0016021:integral component of membrane][GO:0005215:transporter activity][GO:0006857:oligopeptide transport]	-	-	XP_010105518.1:Peptide transporter [Morus notabilis] >EXC05013.1 Peptide transporter [Morus notabilis]	-	W9SG60|W9SG60_9ROSA:Peptide transporter;OS=Morus notabilis.;|GO:0016021,|GO:0005215,|GO:0006857,
DN122397_c4_g3_i2	DOWN	-	-	-	-	-	-
DN113427_c0_g6_i5	DOWN	-	-	-	-	-	-
DN115525_c0_g4_i3	DOWN	-	-	-	-	-	-
DN116525_c1_g1_i10	DOWN	[GO:0046872:metal ion binding][GO:0016491:oxidoreductase activity]	[ko00904:Diterpenoid biosynthesis]	Zpz_sc00083.1.g00290.1.am.mk:Zoysia pacifica|Dof|Dof family protein	XP_021854850.1:gibberellin 2betadioxygenase 8like isoform X1 [Spinacia oleracea] >XP_021854851.1 gibberellin 2betadioxygenase 8like isoform X1 [Spinacia oleracea] >XP_021854852.1	gi|460405863|ref|XM_004248346.1|:PREDICTED: Solanum lycopersicum gibberellin 2-beta-dioxygenase 8-like (LOC101255340), mRNA	Q5D0G3|Q5D0G3_SPIOL:Gibberellin 2-oxidase 3;OS=Spinacia oleracea;|GO:0046872,GO:0016491,

geneid	trend	GO	Pathway	TF	NR	NT	Uniprot
					gibberellin 2betadioxygenase 8like isoform X1 [Spinacia oleracea]		
DN128011_c3_g2_i1	DOWN	[GO:0010181:FMN binding][GO:0003955:NAD(P)H dehydrogenase (quinone) activity]	[ko00130:Ubiquinone and other terpenoid-quinone biosynthesis]	-	XP_021652968.1:probable NAD(P)H dehydrogenase (quinone) FQR1like 1 [Hevea brasiliensis]	gi|359493917|ref|XM_002283250.2|:PREDICTED: Vitis vinifera quinone reductase-like, transcript variant 1 (QOR), mRNA	B9T876|B9T876_RICCO:Minor allergen Alt a, putative;OS=Ricinus communis;|GO:0010181,GO:0003955,

geneid	trend	GO	Pathway	TF	NR	NT	Uniprot
DN117097_c0_g1_i2	DOWN	[GO:0005634:nucleus][GO:0000977:RNA polymerase II regulatory region sequence-specific DNA binding][GO:0003700:DNA-binding transcription factor activity][GO:0000165:MAPK cascade][GO:0045944:positive regulation of transcription by RNA polymerase II][GO:0006351:transcription, DNA-templated]	-	Bv1_006280_gdiw.t1:Beta vulgaris|MIKC_MADS|MIKC_MADS family protein	CAA56657.1:SLM3 [Silene latifolia subsp. alba]	-	Q41354|Q41354_SILLA:SLM3 protein;OS=Silene latifolia;|GO:0005634,|GO:0000977,GO:0003700,|GO:0000165,GO:0045944,GO:0006351,
DN125811_c7_g5_i3	DOWN	-	-	-	KVH91876.1:Thaumatin [Cynara cardunculus var. scolymus]	gi|46949211|gb|AY589711.1|:Mirabilis jalapa clone FS-82 thaumatin-like protein mRNA, partial cds	A0A103XJD6|A0A103XJD6_CYNCS:Thaumatin;OS=Cynara cardunculus var. scolymus;

geneid	trend	GO	Pathway	TF	NR	NT	Uniprot
DN127497_c4_g1_i8	DOWN	[GO:0003824:catalytic activity][GO:0008152:metabolic process]	[ko00061:Fatty acid biosynthesis][ko00071:Fatty acid degradation][ko02024:Quorum sensing][ko03320:PPAR signaling pathway][ko04146:Peroxisome][ko04216:Ferroptosis][ko04920:Adipocytokine signaling pathway]	TRIUR3_12546-P1: Triticum urartu|WRKY|WRKY family protein	XP_021770012.1:long chain acylCoA synthetase 1like isoform X1 [Chenopodium quinoa]	gi|521696965|gb|GAIA01019096.1|:TSA: Salicornia europaea Unigene20056_Se200S transcribed RNA sequence	A0A0K9RJE7|A0A0K9RJE7_SPIOL:Uncharacterized protein;OS=Spinacia oleracea;|GO:0003824,|GO:0008152,
DN124854_c0_g1_i3	DOWN	[GO:0016021:integral component of membrane][GO:0015743:malate transport]	-	-	XP_010685434.1:PREDICTED: aluminumactivated malate transporter 2 [Beta vulgaris subsp. vulgaris] >KMT04941.1 hypothetical protein BVRB_7g171070 [Beta vulgaris subsp. vulgaris]	-	A0A0J8EPN0|A0A0J8EPN0_BETVU:Uncharacterized protein;OS=Beta vulgaris subsp. vulgaris.;|GO:0016021,|GO:0015743,
DN104283_c6_g1_i1	DOWN	-	-	-	-	-	-

geneid	trend	GO	Pathway	TF	NR	NT	Uniprot
DN124920_c9_g2_i7	DOWN	-	-	-	-	-	-
DN111918_c0_g1_i2	DOWN	[GO:0071944:cell periphery][GO:0005618:cell wall][GO:0005576:extracellular region][GO:0045330:aspartyl esterase activity][GO:0030599:pectinesterase activity][GO:0046910:pectinesterase inhibitor activity][GO:0042545:cell wall modification][GO:0045490:pectin catabolic process]	[ko00040:Pentose and glucuronate interconversions]	MDP0000320566:Malus domestica|NAC|NAC family protein	XP_021752630.1:pectinesteraselike [Chenopodium quinoa]	gi|449530262|ref|XM_004172067.1|:PREDICTED: Cucumis sativus pectinesterase 3-like (LOC101212388), partial mRNA	F6HAP0|F6HAP0_VITVI:Pectinesterase;OS=Vitis vinifera;GN=VIT_16s0022g00700{ECO:0000313|EMBL:CCB49323.1};|GO:0071944,GO:0005618,GO:0005576,|GO:0045330,GO:0030599,GO:0046910,|GO:0042545,GO:0045490,
DN122230_c4_g1_i16	DOWN	-	-	XP_008235702.1:Prunus mume|bHLH|bHLH family protein	XP_021742053.1:protein RICE SALT SENSITIVE 3like isoform X1 [Chenopodium quinoa]	gi|521664610|gb|GAIA01035088.1|:TSA: Salicornia europaea Unigene38844_Se200S transcribed RNA sequence	A0A0K9REW3|A0A0K9REW3_SPIOL:Uncharacterized protein;OS=Spinacia oleracea;

geneid	trend	GO	Pathway	TF	NR	NT	Uniprot
DN113697_c0_g1_i7	DOWN	[GO:0016757:transferase activity, transferring glycosyl groups]	[ko00052:Galactose metabolism]	-	XP_010674554.1:PREDICTED: galactinol synthase 2 [Beta vulgaris subsp. vulgaris] >KMT14183.1 hypothetical protein BVRB_4g080000 [Beta vulgaris subsp. vulgaris]	gi|512394503|gb|GAJZ01006667.1|:TSA: Prunus avium Pa_22453 transcribed RNA sequence	A0A0J8CQ88|A0A0J8CQ88_BETVU:Hexosyltransferase;OS=Beta vulgaris subsp. vulgaris.;|GO:0016757,
DN120115_c1_g1_i6	DOWN	[GO:0016021:integral component of membrane][GO:0006810:transport]	-	-	XP_021642145.1:mitochondrial phosphate carrier protein 3, mitochondriallike isoform X1 [Hevea brasiliensis] >XP_021642146.1 mitochondrial phosphate carrier protein 3, mitochondriallike isoform X2 [Hevea brasiliensis]	gi|440356729|gb|JQ343214.1|:Mesembryanthemum crystallinum phosphate transporter mRNA, partial cds	A0A0A1E867|A0A0A1E867_MANES:Phosphate transporter;OS=Manihot esculenta;|GO:0016021,|GO:0006810,

geneid	trend	GO	Pathway	TF	NR	NT	Uniprot
DN123252_c1_g1_i2	DOWN	[GO:0005634:nucleus][GO:0000977:RNA polymerase II regulatory region sequence-specific DNA binding][GO:0003700:DNA-binding transcription factor activity][GO:0000165:MAPK cascade][GO:0045944:positive regulation of transcription by RNA polymerase II][GO:0006351:transcription, DNA-templated]	-	Bv6_139720_hfgn.t1:Beta vulgaris|MIKC_MADS|MIKC_MADS family protein	AAP83384.1:euAP1 APETALA1like MADSbox, partial [Phytolacca americana]	-	Q7XBL6|Q7XBL6_PHYAM:EuAP1 APETALA1-like MADS-box;OS=Phytolacca americana;|GO:0005634,|GO:0000977,GO:0003700,|GO:0000165,GO:0045944,GO:0006351,
DN104283_c6_g8_i1	DOWN	-	-	-	-	-	-
DN116303_c3_g4_i1	DOWN	-	-	-	-	-	-
DN115628_c0_g2_i1	DOWN	-	-	-	-	-	-

geneid	trend	GO	Pathway	TF	NR	NT	Uniprot
DN123436_c3_g5_i3	DOWN	[GO:0016021:integral component of membrane][GO:0008324:cation transmembrane transporter activity]	-	-	ASM90218.1:sodium transporter HKT1 [Sesuvium portulacastrum]	gi|29424040|gb|AY231175.1|:Mesembryanthemum crystallinum high affinity potassium transporter 2 (HKT2) mRNA, complete cds	Q84UZ6|Q84UZ6_MESCR:High affinity potassium transporter 2;OS=Mesembryanthemum crystallinum;|GO:0016021,|GO:0008324,
DN126929_c1_g1_i2	DOWN	[GO:0016702:oxidoreductase activity, acting on single donors with incorporation of molecular oxygen, incorporation of two atoms of oxygen]	[ko00906:Carotenoid biosynthesis]	-	XP_021765560.1:probable carotenoid cleavage dioxygenase 4, chloroplastic [Chenopodium quinoa]	-	A0A0K9RVT5|A0A0K9RVT5_SPIOL: Uncharacterized protein;OS=Spinacia oleracea;|GO:0016702,
DN103642_c2_g4_i2	DOWN	-	-	-	-	-	-
DN124920_c9_g2_i1	DOWN	-	-	-	-	-	-

geneid	trend	GO	Pathway	TF	NR	NT	Uniprot
DN112958_c0_g1_i1	DOWN	[GO:0016788:hydrolase activity, acting on ester bonds]	[ko00562:Inositol phosphate metabolism][ko00564:Glycerophospholipid metabolism][ko00565:Ether lipid metabolism][ko02024:Quorum sensing][ko04919:Thyroid hormone signaling pathway]	Csa09g050690.1:Camelina sativa|NF-YC|NF-YC family protein	XP_010691938.1:PREDICTED: nonspecific phospholipase C4 [Beta vulgaris subsp. vulgaris] >KMS99829.1 hypothetical protein BVRB_1g016810 [Beta vulgaris subsp. vulgaris]	-	A0A0J8BEP0|A0A0J8BEP0_BETVU:Uncharacterized protein;OS=Beta vulgaris subsp. vulgaris.;|GO:0016788,

geneid	trend	GO	Pathway	TF	NR	NT	Uniprot
DN114407_c1_g3_i4	DOWN	[GO:0005634:nucleus][GO:0000977:RNA polymerase II regulatory region sequence-specific DNA binding][GO:0003700:DNA-binding transcription factor activity][GO:0010154:fruit development][GO:0010077:maintenance of inflorescence meristem identity][GO:0000165:MAPK cascade][GO:0045944:positive regulation of transcription by RNA polymerase II][GO:0006351:transcription, DNA-templated]	-	Bv5_113480_aoik.t1:Beta vulgaris|MIKC_MADS|MIKC_MADS family protein	XP_010678364.1:PREDICTED: truncated transcription factor CAULIFLOWER A isoform X1 [Beta vulgaris subsp. vulgaris] >KMT10844.1 hypothetical protein BVRB_5g113480 isoform A [Beta vulgaris subsp. vulgaris]	gi|23304681|emb|AJ505846.1|:Brassica oleracea var. botrytis mRNA for MADS-box protein AP1-c	A0A0J8CFD5|A0A0J8CFD5_BETVU:Uncharacterized protein;OS=Beta vulgaris subsp. vulgaris.;|GO:0005634,|GO:0000977,GO:0003700,|GO:0010154,GO:0010077,GO:0000165,GO:0045944,GO:0006351,

geneid	trend	GO	Pathway	TF	NR	NT	Uniprot
DN114407_c1_g3_i1	DOWN	[GO:0005634:nucleus][GO:0000977:RNA polymerase II regulatory region sequence-specific DNA binding][GO:0003700:DNA-binding transcription factor activity][GO:0010154:fruit development][GO:0010077:maintenance of inflorescence meristem identity][GO:0000165:MAPK cascade][GO:0045944:positive regulation of transcription by RNA polymerase II][GO:0006351:transcription, DNA-templated]	-	Bv5_113480_aoik.t1:Beta vulgaris|MIKC_MADS|MIKC_MADS family protein	XP_010678364.1:PREDICTED: truncated transcription factor CAULIFLOWER A isoform X1 [Beta vulgaris subsp. vulgaris] >KMT10844.1 hypothetical protein BVRB_5g113480 isoform A [Beta vulgaris subsp. vulgaris]	gi|10946428|gb|AF305076.1|AF305076:Eucalyptus globulus MADS-box protein EAP1 mRNA, complete cds	A0A0J8CFD5|A0A0J8CFD5_BETVU:Uncharacterized protein;OS=Beta vulgaris subsp. vulgaris.;|GO:0005634,|GO:0000977,GO:0003700,|GO:0010154,GO:0010077,GO:0000165,GO:0045944,GO:0006351,
DN122169_c0_g1_i2	DOWN	-	-	-	KNA09736.1:hypothetical protein SOVF_150850 [Spinacia oleracea]	-	A0A0K9QT91|A0A0K9QT91_SPIOL:Uncharacterized protein;OS=Spinacia oleracea;

geneid	trend	GO	Pathway	TF	NR	NT	Uniprot
DN114407_c1_g3_i7	DOWN	[GO:0005634:nucleus][GO:0000977:RNA polymerase II regulatory region sequence-specific DNA binding][GO:0003700:DNA-binding transcription factor activity][GO:0010154:fruit development][GO:0010077:maintenance of inflorescence meristem identity][GO:0000165:MAPK cascade][GO:0045944:positive regulation of transcription by RNA polymerase II][GO:0006351:transcription, DNA-templated]	-	Bv5_113480_aoik.t1:Beta vulgaris|MIKC_MADS|MIKC_MADS family protein	XP_010678364.1:PREDICTED: truncated transcription factor CAULIFLOWER A isoform X1 [Beta vulgaris subsp. vulgaris] >KMT10844.1 hypothetical protein BVRB_5g113480 isoform A [Beta vulgaris subsp. vulgaris]	gi|10946428|gb|AF305076.1|AF305076:Eucalyptus globulus MADS-box protein EAP1 mRNA, complete cds	A0A0J8CFD5|A0A0J8CFD5_BETVU:Uncharacterized protein;OS=Beta vulgaris subsp. vulgaris.;|GO:0005634,|GO:0000977,GO:0003700,|GO:0010154,GO:0010077,GO:0000165,GO:0045944,GO:0006351,
DN124262_c0_g1_i4	DOWN	-	-	Achn250761:Actinidia chinensis|TCP|TCP family protein	XP_012441714.1:PREDICTED: uncharacterized protein LOC105766694 [Gossypium raimondii]	-	A0A0D2QW30|A0A0D2QW30_GOSRA:Uncharacterized protein;OS=Gossypium raimondii;

geneid	trend	GO	Pathway	TF	NR	NT	Uniprot
					>KJB62177.1 hypothetical protein B456_009G405000 [Gossypium raimondii]		
DN127807_c0_g2_i4	DOWN	[GO:0008289:lipid binding][GO:0006869:lipid transport]	-	-	XP_010679071.1:PREDICTED: nonspecific lipid transfer protein GPIanchored 2 [Beta vulgaris subsp. vulgaris] >KMT10188.1 hypothetical protein BVRB_5g119490 [Beta vulgaris subsp. vulgaris]	-	A0A0J8CDW0|A0A0J8CDW0_BETVU: Uncharacterized protein;OS=Beta vulgaris subsp. vulgaris.;|GO:0008289,|GO:0006869,
